# Supplementary material for: TopEC: prediction of Enzyme Commission classes by 3D graph neural networks and localized 3D protein descriptor
Source: Nat Commun. 2025 Mar 20;16:2737. doi: 10.1038/s41467-025-57324-5 (PMC11923149; doi:10.1038/s41467-025-57324-5)
Supplement: Supplementary file 1 — Supplementary Information [file 41467_2025_57324_MOESM1_ESM.pdf]

Supporting Information

TopEC: Prediction of Enzyme Commission classes by 3D Graph Neural Networks and localized 3D protein descriptor

Karel van der Weg[1], Erinc Merdivan[2], Marie Piraud[2], Holger Gohlke[1,3,*]

[1]Institute of Bio- and Geosciences (IBG-4: Bioinformatics), Forschungszentrum Jülich GmbH, 52425 Jülich, Germany

[2]Helmholtz AI Central Unit, Ingolstädter Landstraße 1, 85764 Oberschleißheim, Germany

[3]Institute for Pharmaceutical and Medicinal Chemistry, Heinrich Heine University Düsseldorf, 40225 Düsseldorf, Germany

*Corresponding Author:
Prof. Dr. Holger Gohlke
Address: Wilhelm-Johnen-Str., 52425 Jülich, Germany.
Phone: (+49) 2461 61 85550
E-mail: h.gohlke@fz-juelich.de

Contents

Supplementary Tables 4

Supplementary Table 1. Details of the PrOSPECCTs datasets. 4

Supplementary Table 2. The pair-wise sequence identity for structures in Fig. 4g. 4

Supplementary Table 3. Atom annotation per residue type. 5

Supplementary Table 4. Training and inference speeds. 8

Supplementary Figures 9

Supplementary Figure 1. Our implementation of the SchNet and DimeNet++ architecture... 9

Supplementary Figure 2. Model performance when training with and without oversampling. 10

Supplementary Figure 3. EnzyNet results for all modes and datasets tested. 11

Supplementary Figure 4. F1 score as a function of graph node count for hierarchical EC classification. 12

Supplementary Figure 5. Model performance when training on *ab initio* predicted structures (AlphaFold2) or homology modeled structures (TopModel) for various descriptors and networks tested. 13

Supplementary Figure 6. The area under the precision-recall curve against different properties of the data. 14

Supplementary Figure 7. 10 different folds for type II site-specific deoxyribonucleases.......15

Supplementary Figure 8. F1 score for all Price and ProSPECCTs datasets broken down by tested network type. 16

Supplementary Figure 9. Importance gain per binding site and catalytic residue compared to regular residues. 17

Supplementary Figure 10. Investigation into the importance of the catalytic sites for PDB 1QF6 18

Supplementary Figure 11. Investigation into the importance of the catalytic sites for PDB 3UH0 19

Supplementary Figure 12. Investigation into the importance of the catalytic sites for PDB 3UGQ 20

Supplementary Figure 13. Investigation into the importance of the catalytic sites for PDB 6VU9 21

Supplementary Figure 14. Investigation into the importance of the catalytic sites for PDB 5ZY9 22

Supplementary Figure 15. Explained PDB structure 1GLA compared to stability predictors. 22

Supplementary Figure 16. Explained PDB structure 1WQ1 compared to stability predictors 24

Supplementary Figure 17. Explained PDB structure 3BLM compared to stability predictors. 25

Supplementary Figure 18. Explained PDB structure 2MAT compared to stability. 26
Supplementary Figure 19. Explained PDB structure 1A9U compared to stability predictors. 27
Supplementary Figure 20. Explained PDB structure 3DRC compared to stability predictors. 28
Supplementary Figure 21. Explained PDB structure 1BIW compared to stability predictors. 29
Supplementary Figure 22. Importance for all catalytic and binding atoms. 34
Supplementary Figure 23. Importance for all non-catalytic and non-binding atoms. 39
Supplementary Figure 24. Importance for all catalytic and binding atoms in wrongly predicted enzymes. 44

Supplementary Tables

Supplementary Table 1. Details of the PrOSPECCTs datasets.

**Name**	**Description**	**Goal**
DS1	Structures with identical sequences	Sensitivity with respect to the binding site definition.
DS1.2	Structures with identical sequences and similar ligands	Impact of ligand diversity on binding site comparison
DS2	NMR structures	Sensitivity with respect to the binding site flexibility
DS3	Decoy set 1	Differentiation between binding sites with different physicochemical properties
DS4	Decoy set 2	Differentiation between binding sites with different physicochemical and shape properties
DS5	Kahraman data set without phosphate binding sites	Classification of proteins binding to identical ligands and cofactors
DS5.2	Kahraman data set	Original data set
DS6	Barelier data set	Identification of distant relationships between protein binding sites with identical ligands which "observe" a similar environment
DS6.2	Barelier data set including cofactors	
DS7	Data set of successful applications	Recovery of known binding site similarities within a set of diverse proteins.

Adapted from: Ehrt C, Brinkjost T, Koch O (2018) A benchmark driven guide to binding site comparison: An exhaustive evaluation using tailor-made data sets (ProSPECCTs). PLoS Comput Biol 14(11): e1006483. https://doi.org/10.1371/journal.pcbi.1006483

Supplementary Table 2. The pair-wise sequence identity for structures in Figure 3g.

**PDB**	1QF6	3UH0	3UGQ	5ZY9	6VU9
1QF6	100%				
3UH0	38%	100%			
3UGQ	38%	100%	100%		
5ZY9	40%	43%	43%	100%	
6VU9	59%	37%	36%	40%	100%

Supplementary Table 3. Atom annotation per residue type.

**Backbone Atoms**		
*13 in GLY/PRO<br>**Not present in GLY/PRO	N	1
	C	2
	CA*	3 (13*)
	O / OXT	4
	H**	32
	HA**	33

**Neutral-Nonpolar R Groups**		
Glycine **GLY**	H	32
	1HA / 2HA	39
Alanine **ALA**	CB	8
	1HB / 2HB / 3HB	36
Valine **VAL**	CB	7
	CG1 / CG2	8
	HB	34
	1HG1 / 2HG1 / 3HG1 / 1HG2 / 2HG2 / 3HG2	36
Isoleucine **ILE**	CB	6
	CG1	7
	CG2 / CD1	8
	HB	34
	1HD1 / 2HD1 / 3HD1 / 1HG2 / 2HG2 / 3HG2	36
	1HG1 / 2HG1	35
Leucine **LEU**	CB	7
	CG	6
	CD1 / CD2	8
	HG	34
	1HB / 2HB	35
	1HD1 / 2HD1 / 3HD1 / 1HD2 / 2HD2 / 3HD2	36
Proline **PRO**	CB / CG / CD	14
	1HB / 2HB / 1HD / 2HD / 1HG / 2HG	35
Methionine **MET**	CB / CG	7
	CE	8
	SD	22
	1HB / 2HB / 1HG / 2HG	35

	1H / 2H / 3H / 1HE / 2HE / 3HE	36

**Neutral-Polar R Groups**		
Serine **SER**	CB	7
	OG	10
	1HB / 2 HB	35
	HG	38
Threonine **THR**	CB	6
	CG2	8
	OG1	10
	HB	34
	1HG2 / 2HG2 / 3HG2	36
	HG	38
Asparagine **ASN**	CB	7
	CG	5
	ND2	17
	OD1	12
	1HB / 2HB	35
	1HD2 / 2HD2	43
Glutamine **GLN**	CB / CG	7
	CD	5
	NE2	17
	OE1	12
	1HB / 2HB / 1HG / 2HG	35
	1HE2 / 2HE2	43
Cysteine **CYS**	CB	7
	SG	20
	1HB / 2HB	35
	HG	45

**Acidic R Groups**		
Aspartic Acid **ASP**	CB	7
	CG	5
	OD1 / OD2	11
	1HB / 2HB	35
Glutamic Acid **GLU**	CB / CG	7
	CD	5
	OE1 / OE2	11

**Basic R Groups**		
Lysine **LYS**	CB / CG / CD / CE	7
	NZ	26
	1 HB / 2HB / 1HD / 2 HD / 1 HE / 2HE / 1HG / 2HG	35
	1HZ / 2HZ / 3HZ	47
Arginine **ARG**	CB / CG / CD	7
	CZ	5
	NE	15
	NH1 / NH2	16
	HE	41
	1HB / 2HB / 1HD / 2HD / 1HG / 2HG	35
	1HH1 / 2HH1 / 1HH2 / 2HH2	42

**Aromatic R Groups**		
Histidine **HIS**	CB	7
	CG	28
	CD	27
	CE1	25
	ND1	15
	NE2	15
	1HB / 2HB	35
	HD1 / HE2	41
	HE1	46
	HD2	48
Phenylalanine **PHE**	CB	7
	CG	21
	CD1 / CD2 / CE1 / CE2 / CZ	9
	HD1 / HD2 / HE1 / HE2 / HZ	37
	1HB / 2HB	35
Tryptophan **TRP**	CB	7
	CG	29
	CD1 / CE3 / CZ3	9
	CD2 / CE2	24
	CH2 / CZ2	18
	NE1	15
	1HB / 2HB	35
	HD1 / HE3 / HZ3	37
	HE1	41

	HH2 / HZ2	44
Tyrosine **TYR**	CB	7
	CG	23
	CD1 / CD2 / CE1 / CE2	9
	CZ	19
	OH	10
	HD1 / HD2 / HE1 / HE2	37
	HH	38
	1HB / 2HB	35

Supplementary Table 4. Training and inference speeds.

**Training performance on 4x A100 using DDP using 100 nodes per graph.**		
Network:	Batches per second:	Proteins per second:
SchNett	2.52 ± 0.209 (batch = 256)	645
DimeNett	4.30 ± 0.238 (batch = 32)	137
**Predictive performance on an Intel Core i7-10700 @ 2.90GHz over 7 runs.**		
Number of nodes:	Residue resolution (seconds per 100 samples)	Atom resolution (seconds per 100 samples)
25	1.04 ± 0.017	1.43 ± 0.004
50	1.34 ± 0.009	2.05 ± 0.095
75	1.59 ± 0.011	2.88 ± 0.205
100	1.85 ± 0.033	3.86 ± 0.193
150	2.63 ± 0.114	5.85 ± 0.237
200	3.61 ± 0.391	8.19 ± 0.126

Supplementary Figures

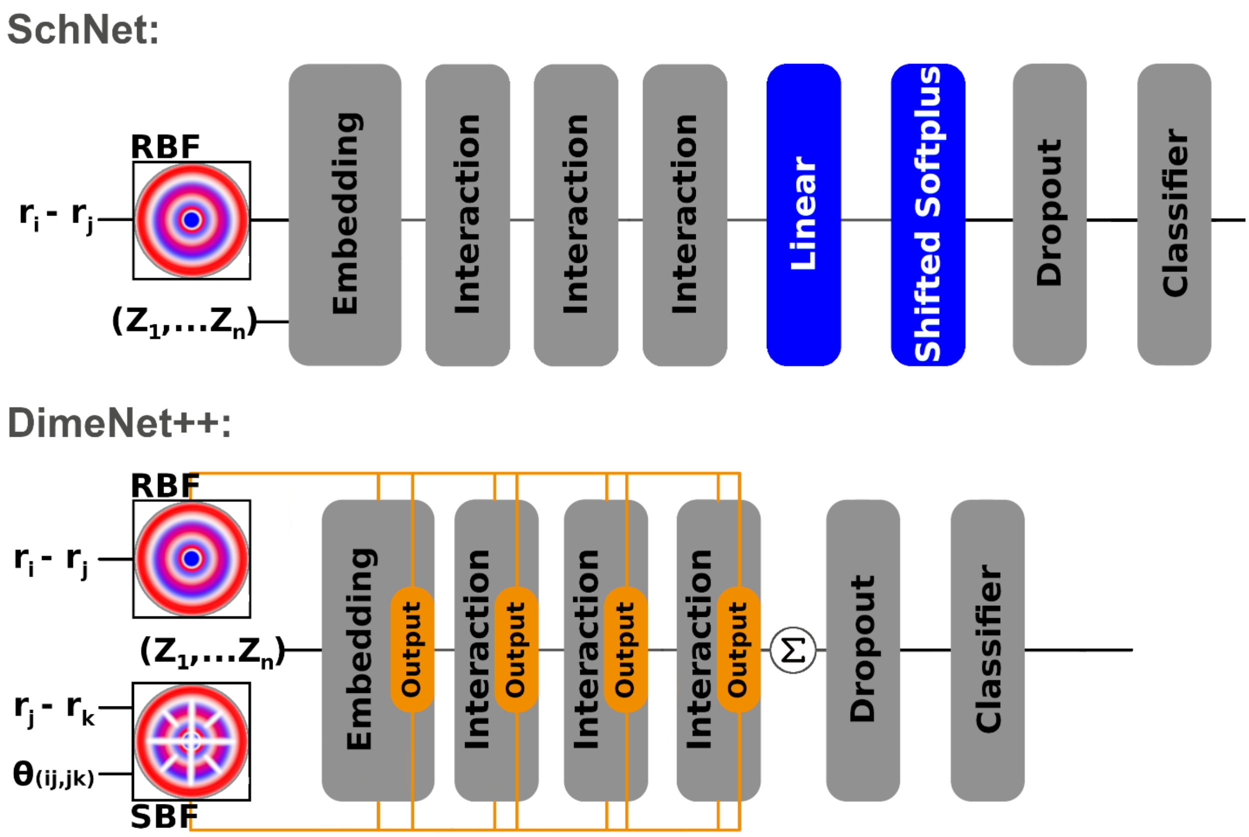

Supplementary Figure 1. Our implementation of the SchNet and DimeNet++ architecture.

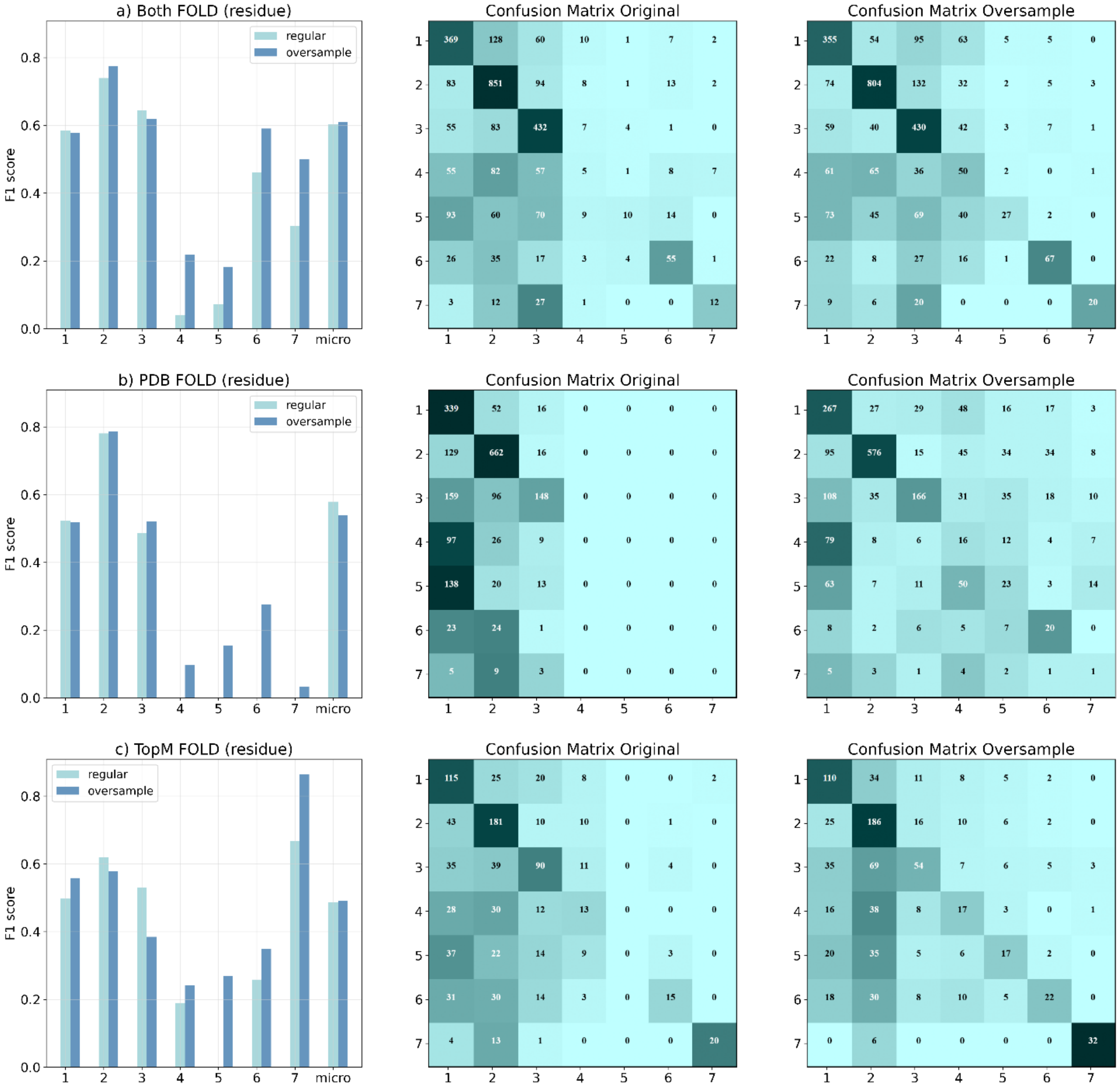

Supplementary Figure 2. Model performance when training with and without oversampling.

Left: The F-score for each mainclass of the fold networks tested in Table 1A. Middle: The confusion matrix for the networks tested without oversampling. Right: The confusion matrix for the networks tested with oversampling.

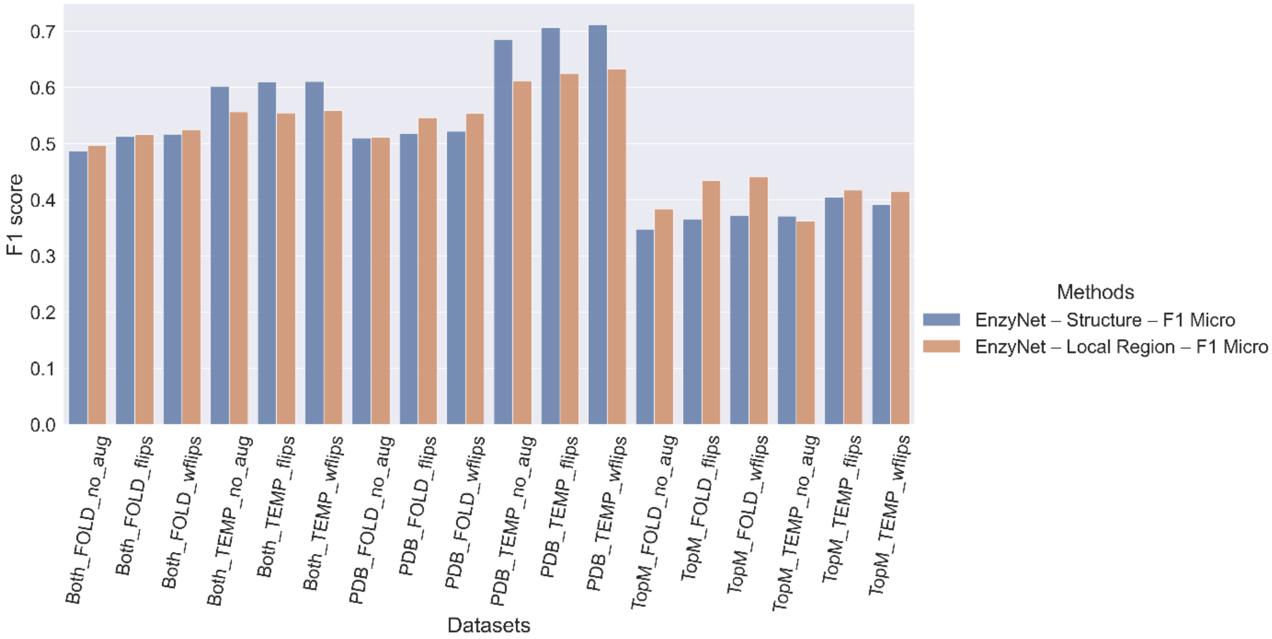

Supplementary Figure 3. EnzyNet results for all modes and datasets tested.

Each dataset and split for networks trained on the full structure and the local region (16 Å around the binding site). For each dataset and split combination, we tested three methods: no augmentation (*_no_aug),* flips (*_flips),* and weighted flips *(_wflips)*.

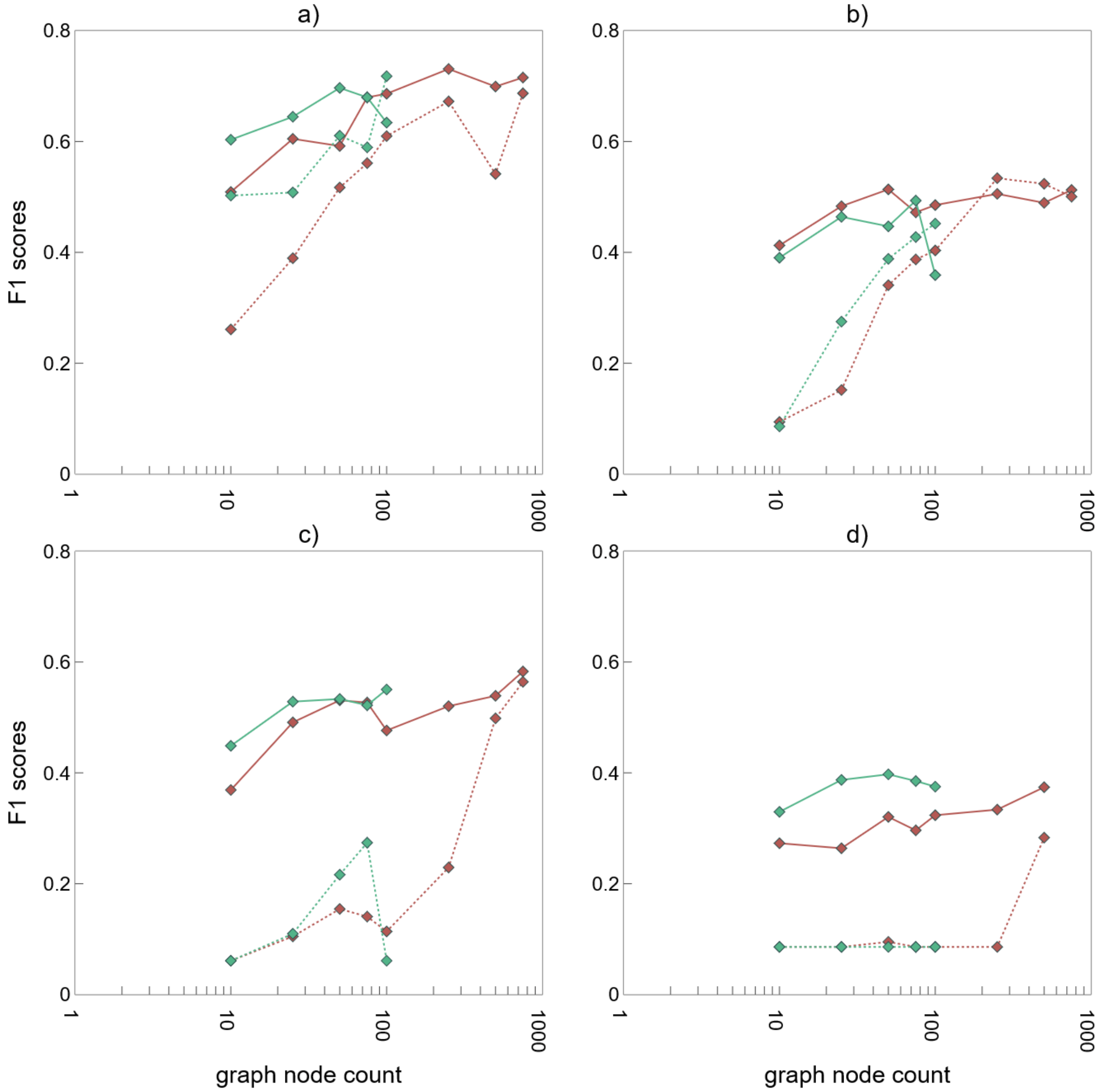

Supplementary Figure 4. F1 score as a function of graph node count for hierarchical EC classification.

Red: Networks trained with a SchNet model. Green: Networks trained with a DimeNet++ model. For the continuous lines, we created the localized 3D descriptors using binding site information. For the dotted lines, we created the localized 3D descriptor from a random point on the protein. We show the result for the combined dataset using a) residue resolution with a temporal split, b) residue resolution with a fold split, c) atom resolution with a temporal split, d) atom resolution with a fold split.

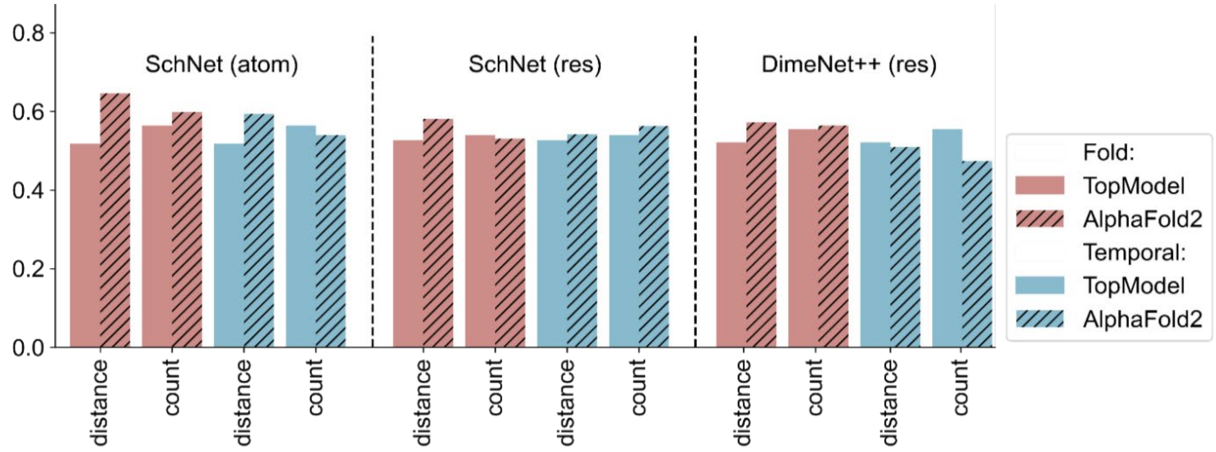

Supplementary Figure 5. Model performance when training on ab initio predicted structures (AlphaFold2) or homology modeled structures (TopModel) for various descriptors and networks tested.

F-score for the networks trained with models obtained from TopModel (no stripes) and AlphaFold2 (stripes) for a fold split (red) and a temporal split (blue).

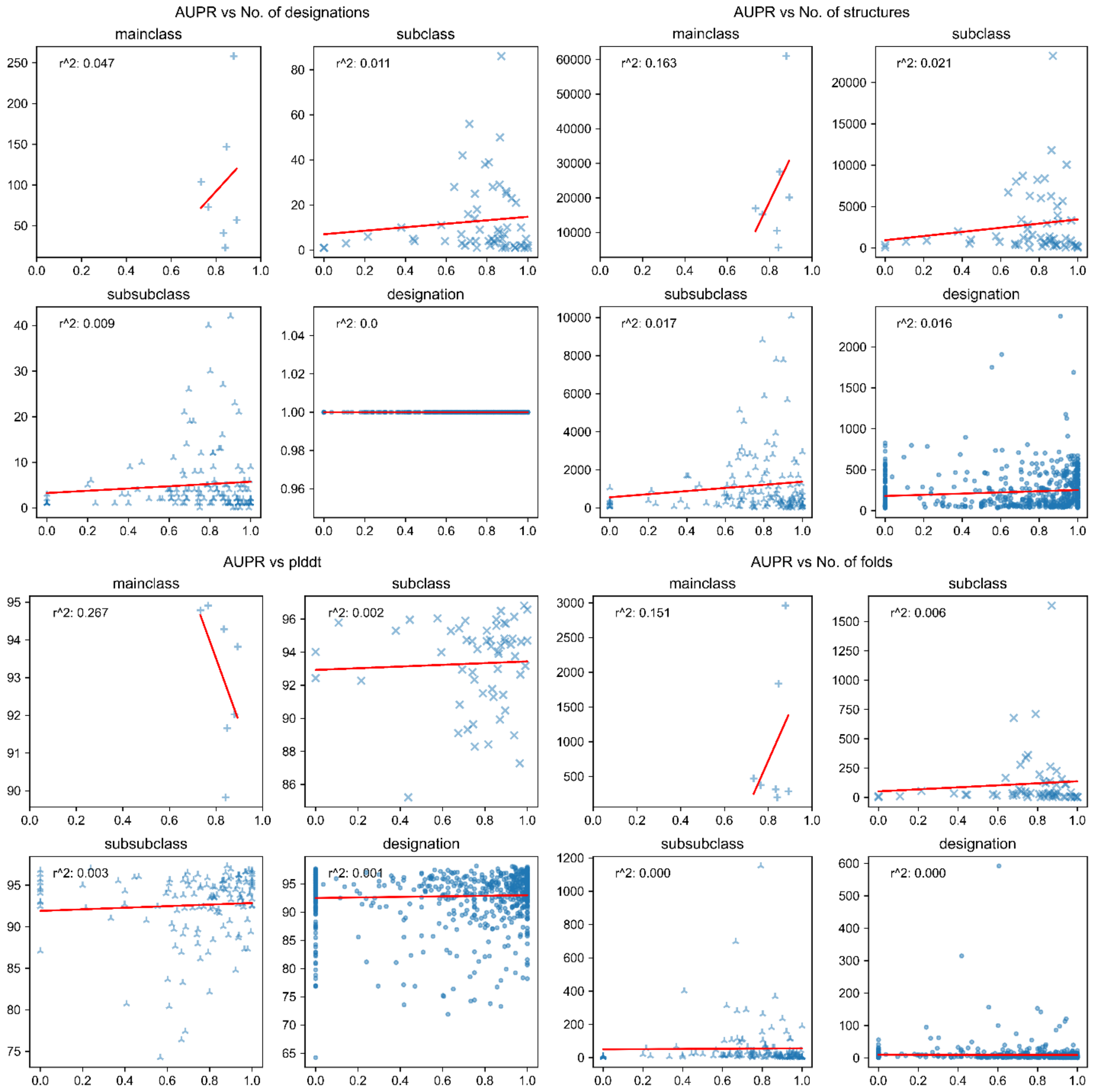

Supplementary Figure 6. The area under the precision-recall curve against different properties of the data.

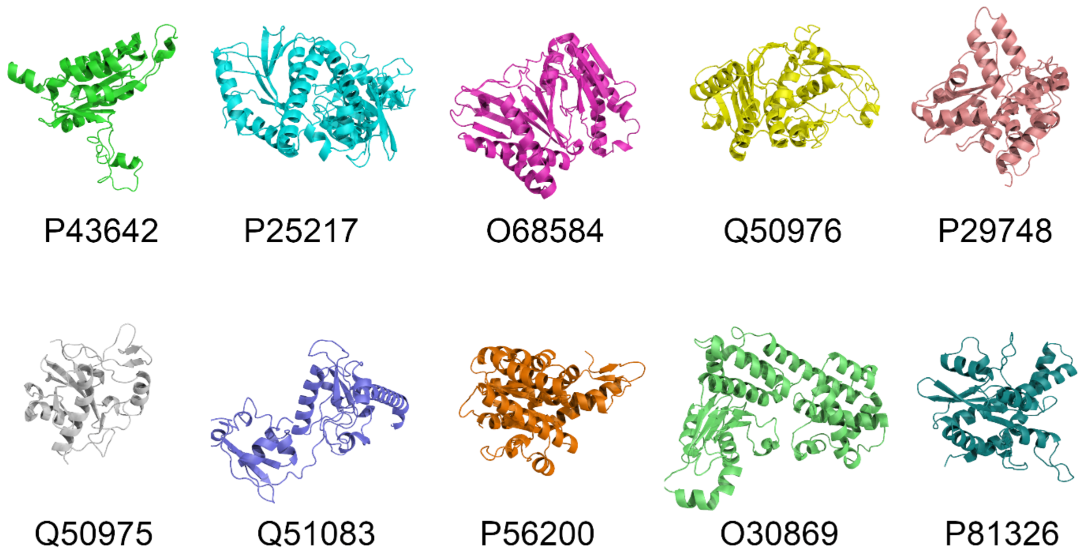

Supplementary Figure 7. 10 different folds for type II site-specific deoxyribonucleases. None of the structures are correctly predicted by a network trained on AF703 using a random split.

	Price	DS1	DS1.2	DS2	DS3	DS4	DS5	DS5.2	DS6	DS6.2	DS7
fold_SchNet_atom_distance	0.24	0.12	0.44	0.48	0.37	0.36	0.48	0.49	0.69	0.69	0.55
fold_SchNet_atom_count	0.10	0.04	0.11	0.20	0.33	0.34	0.40	0.46	0.38	0.38	0.35
fold_SchNet_residue_distance	0.14	0.37	0.56	0.44	0.56	0.56	0.68	0.65	0.69	0.69	0.66
fold_SchNet_residue_count	0.19	0.36	0.56	0.44	0.56	0.56	0.76	0.68	0.72	0.72	0.69
fold_DimeNet++_atom_distance	0.19	0.22	0.22	0.43	0.36	0.36	0.44	0.43	0.59	0.59	0.53
fold_DimeNet++_atom_count	0.10	0.09	0.11	0.19	0.35	0.33	0.20	0.24	0.34	0.34	0.35
fold_DimeNet++_residue_distance	0.10	0.38	0.56	0.53	0.22	0.30	0.48	0.51	0.55	0.55	0.64
fold_DimeNet++_residue_count	0.19	0.36	0.56	0.78	0.50	0.50	0.72	0.59	0.62	0.62	0.64

Supplementary Figure 8. F1 score for all Price and ProSPECCTs datasets broken down by tested network type.

In the left column, the split, network, resolution, and localized 3D descriptor type for each subset of the Price and ProSPECCTs dataset are given.

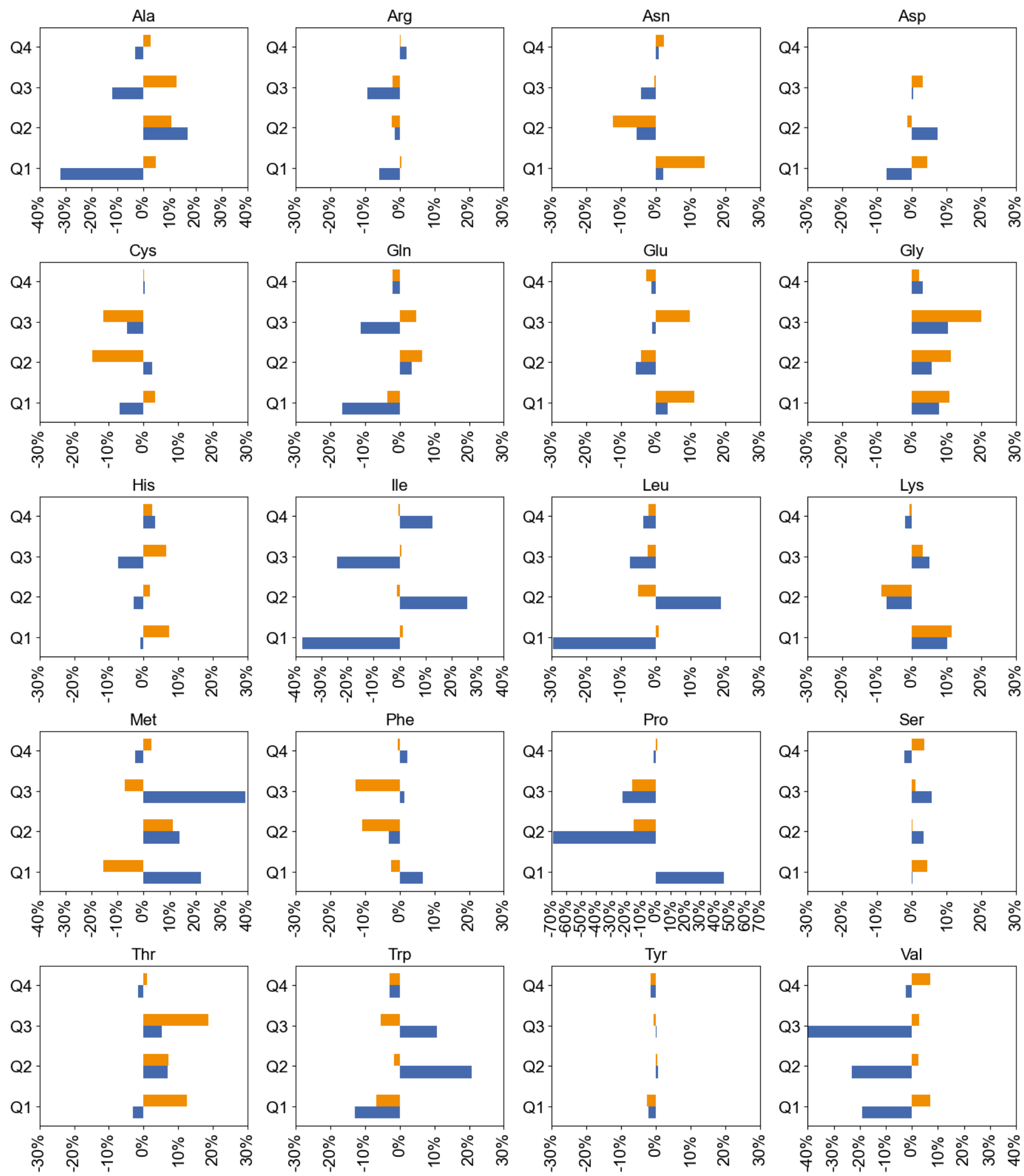

Supplementary Figure 9. Importance gain per binding site and catalytic residue compared to regular residues.

Each panel depicts a separate amino acid. We calculated the importance gain for catalytic (orange) and binding (blue) residues compared to all other residues of that type in the correctly predicted subset of the CSA. We have binned the importance values in four quartiles. A gain in Q1 and Q2 is characterized as positive if we find fewer binding or catalytic residues, while a gain in Q3 and Q4 is characterized as positive if we find more binding or catalytic residues.

PDB: 1QF6
Confidence: 0.99

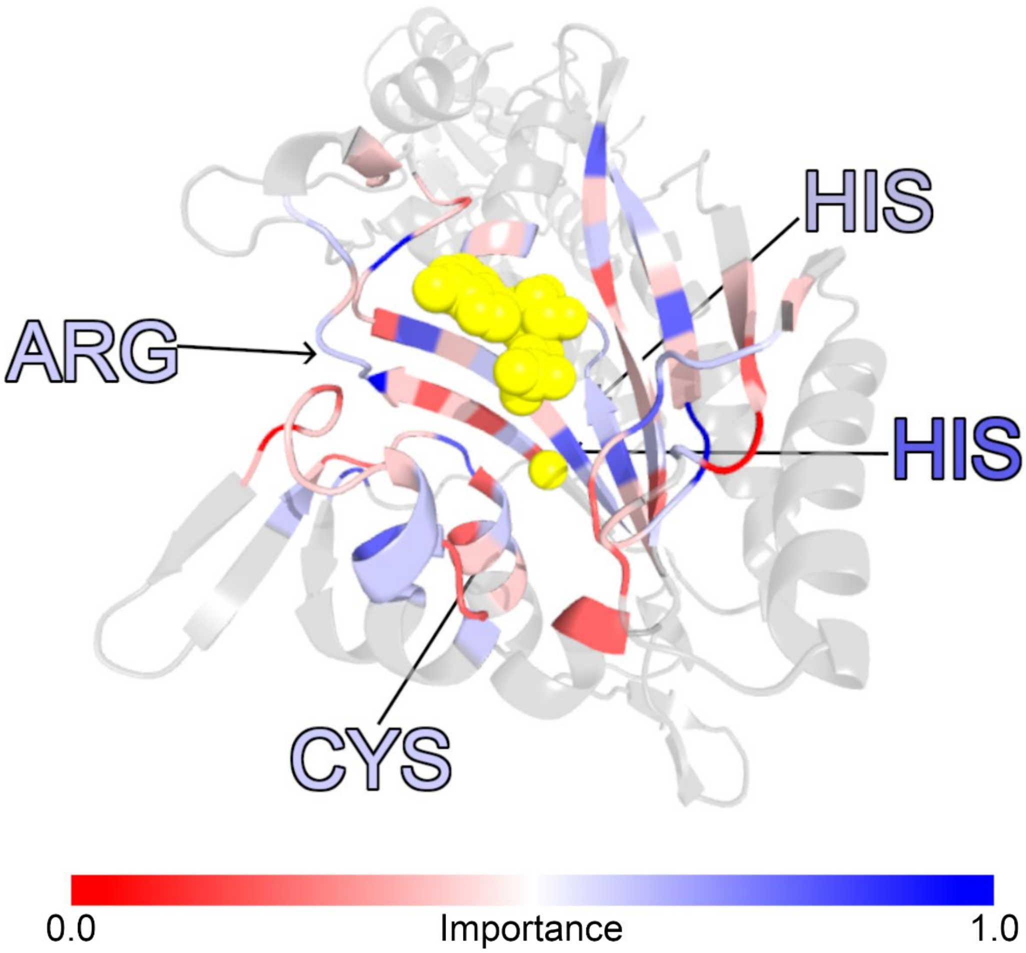

Supplementary Figure 10. Investigation into the importance of the catalytic sites for PDB 1QF6.

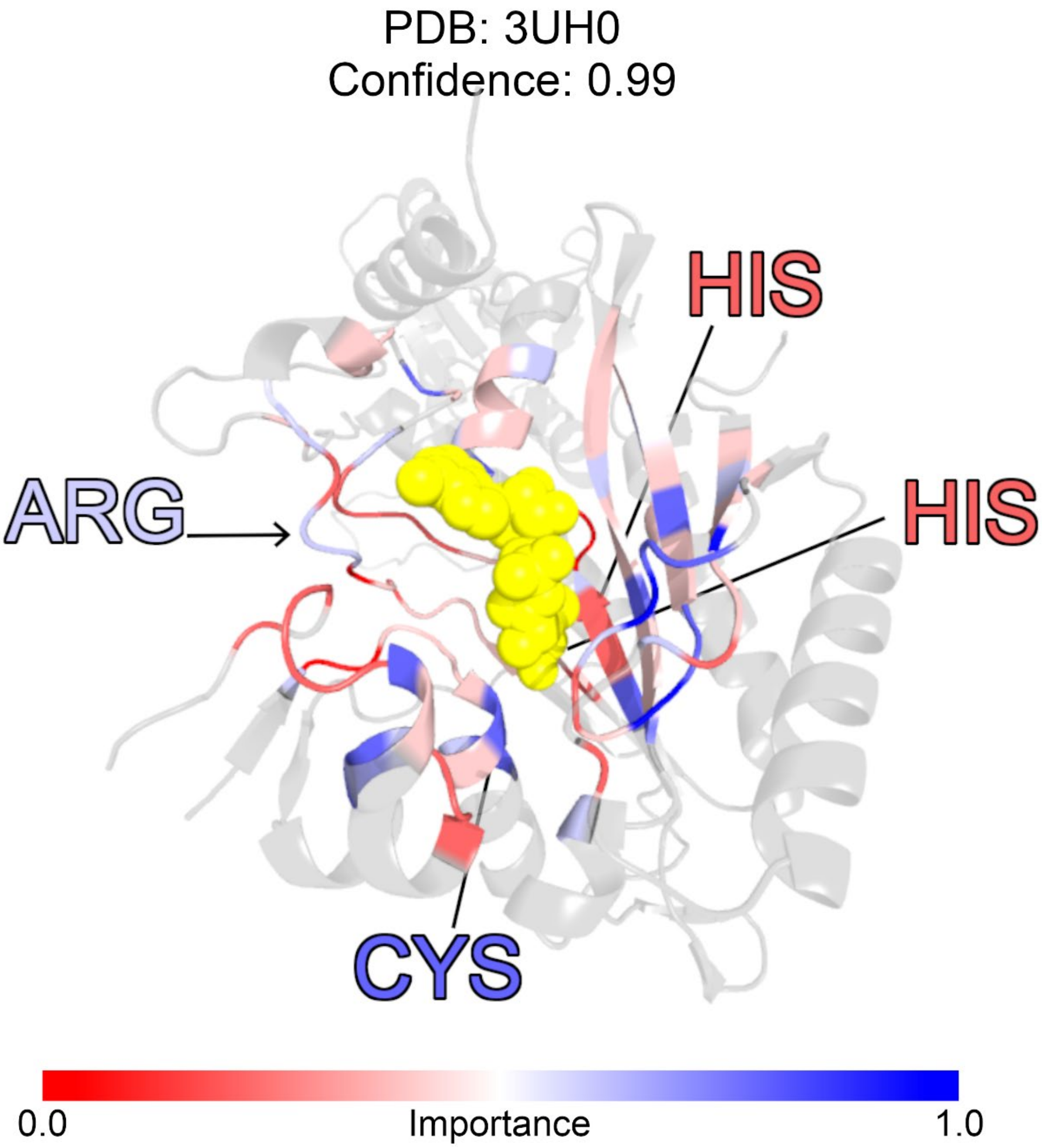

Supplementary Figure 11. Investigation into the importance of the catalytic sites for PDB 3UH0.

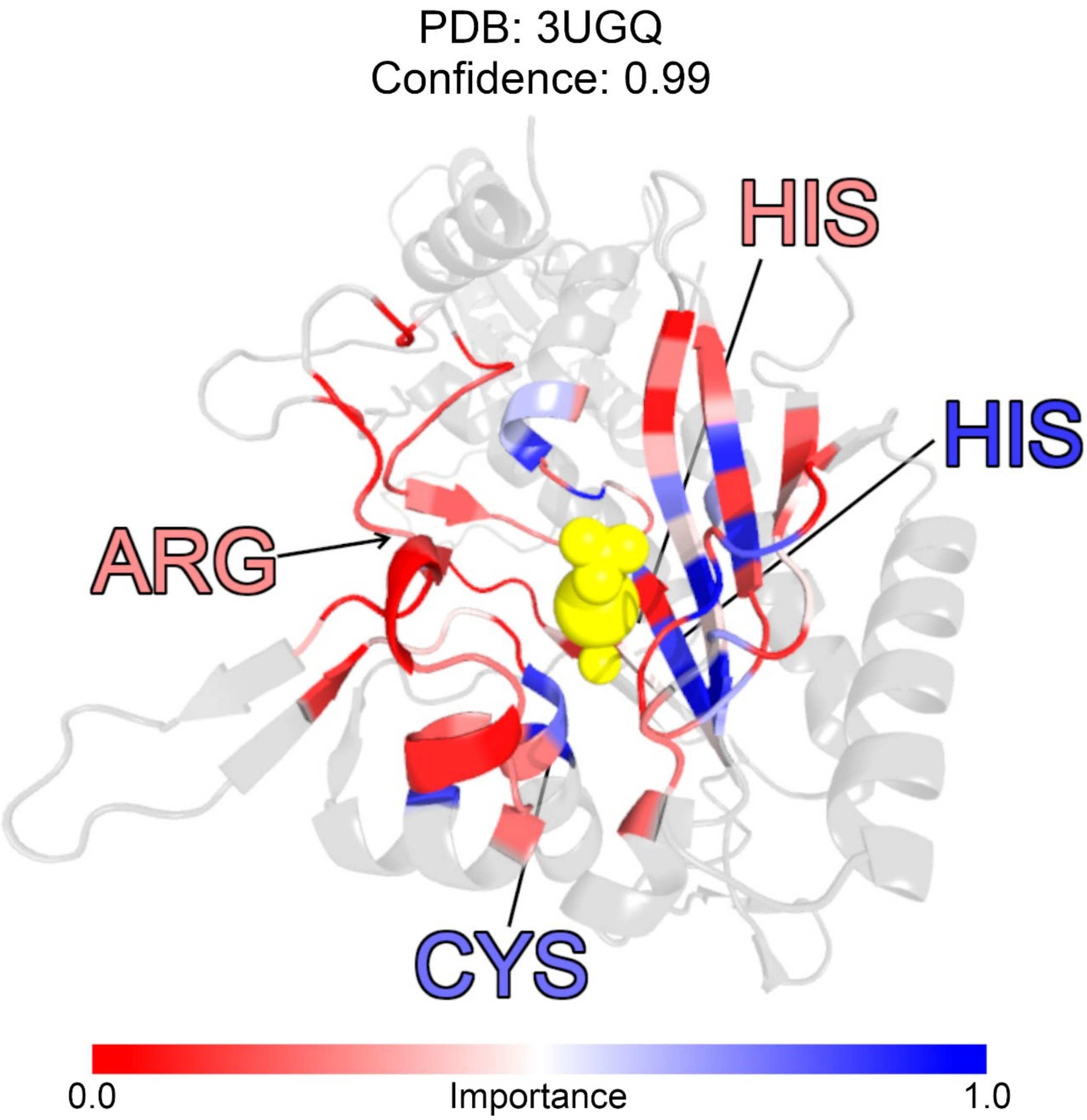

Supplementary Figure 12. Investigation into the importance of the catalytic sites for PDB 3UGQ.

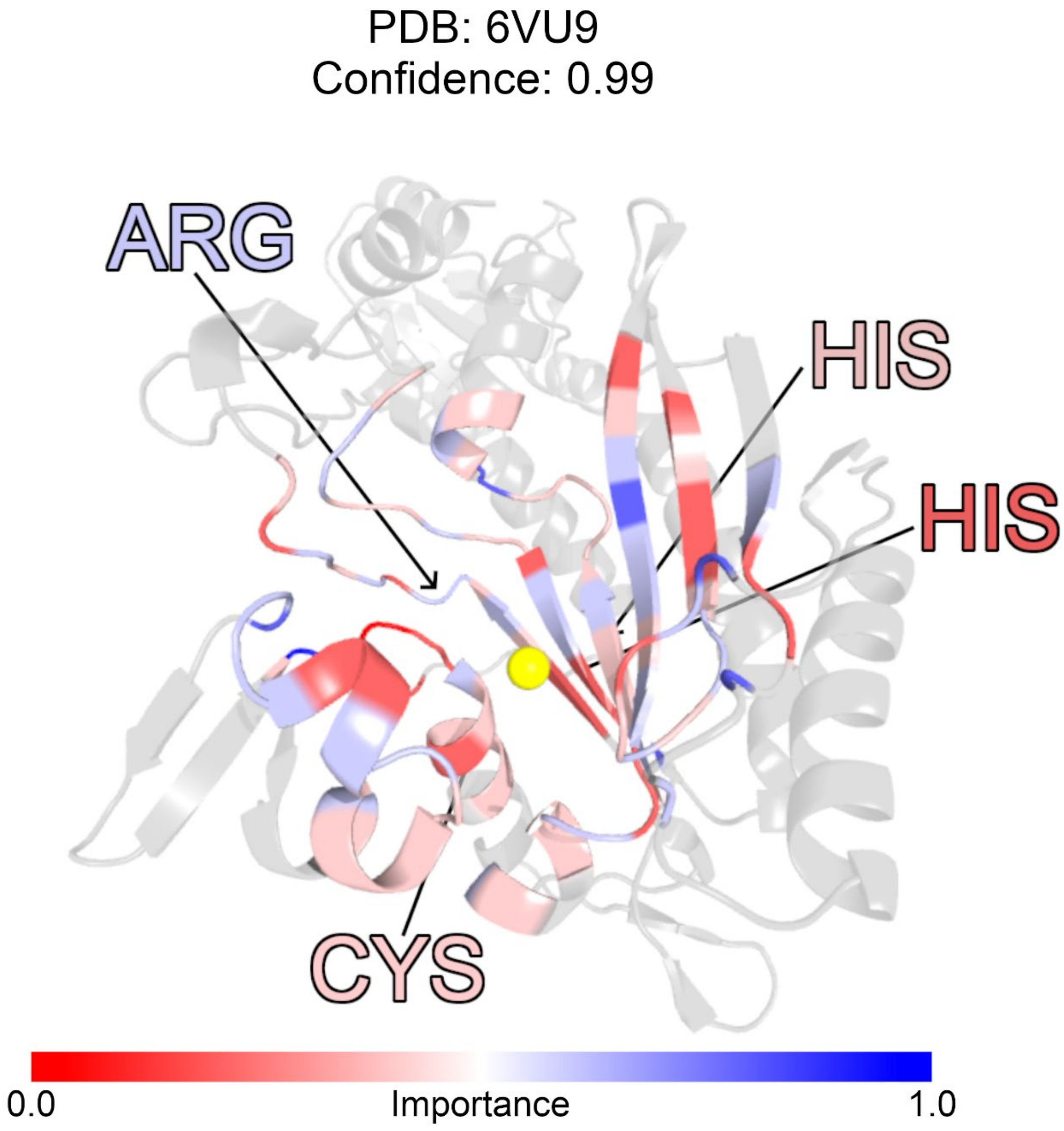

Supplementary Figure 13. Investigation into the importance of the catalytic sites for PDB 6VU9.

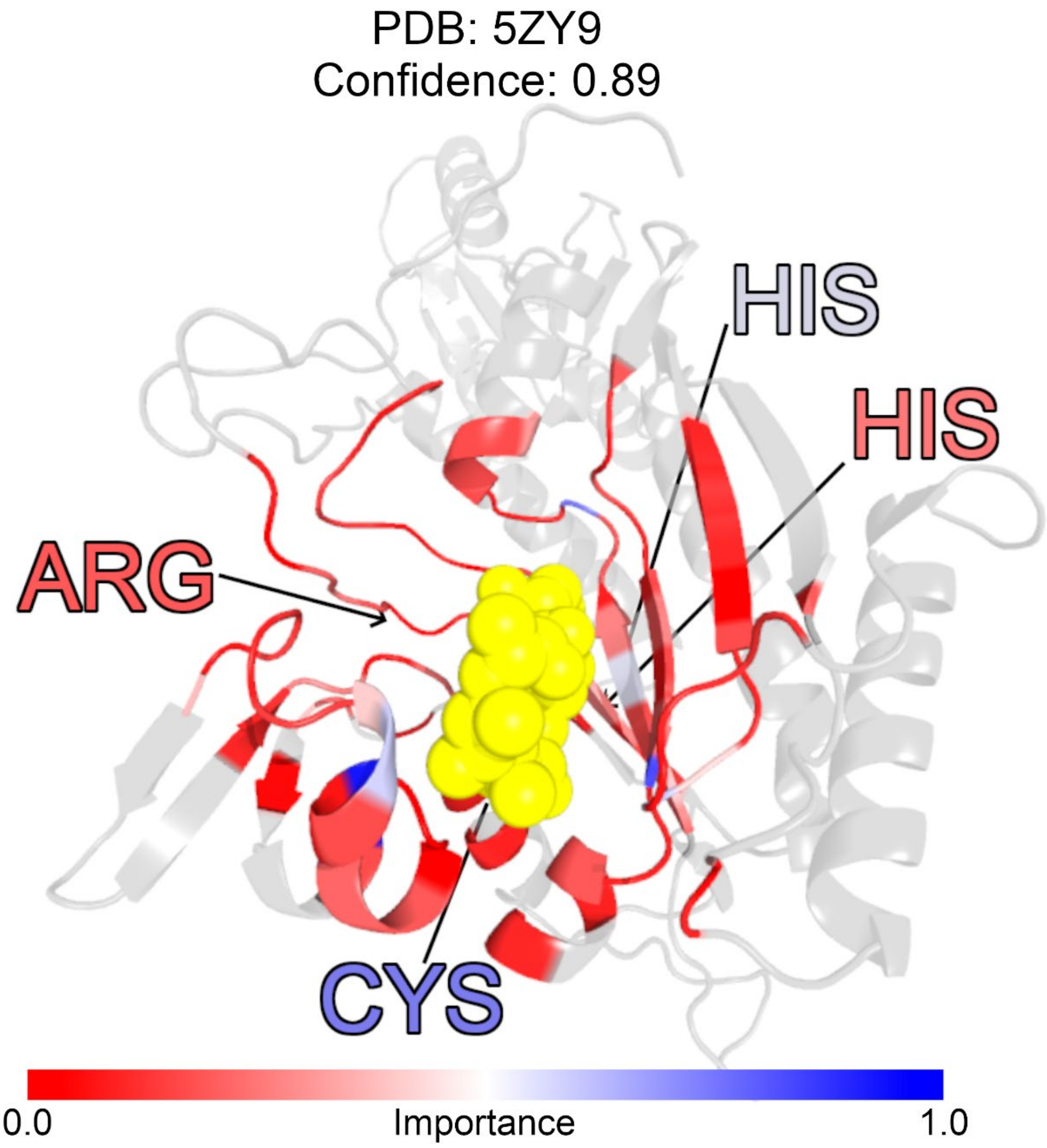

Supplementary Figure 14. Investigation into the importance of the catalytic sites for PDB 5ZY9.

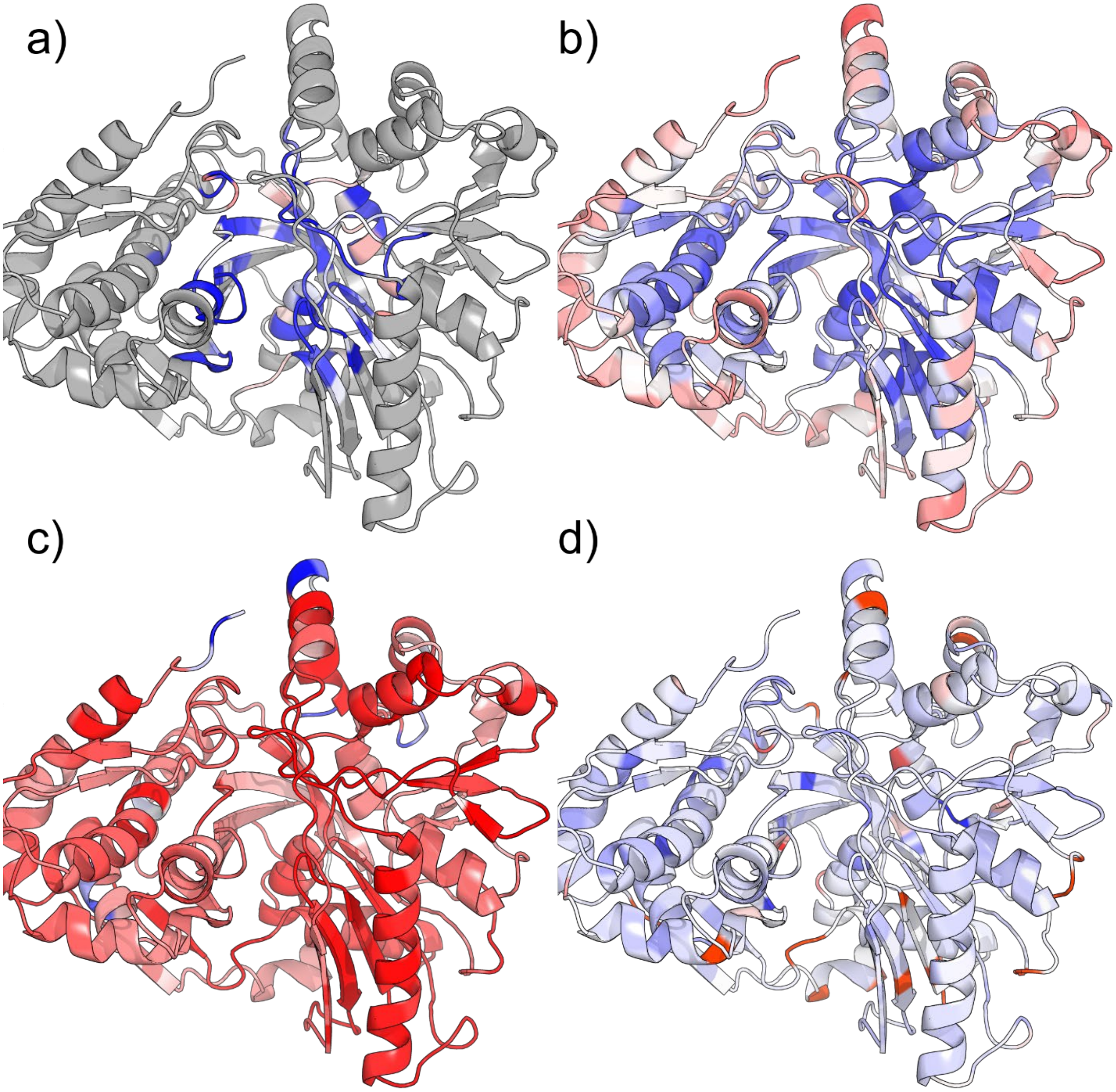

Supplementary Figure 15. Explained PDB structure 1GLA compared to stability predictors.

a) GNNExplainer, b) Amino Acids Interaction Webserver, c) Constraint Network Analysis, d) K-Fold. For this structure, we were not able to obtain Thermometer results. Blue indicates stable, red indicates unstable residues.

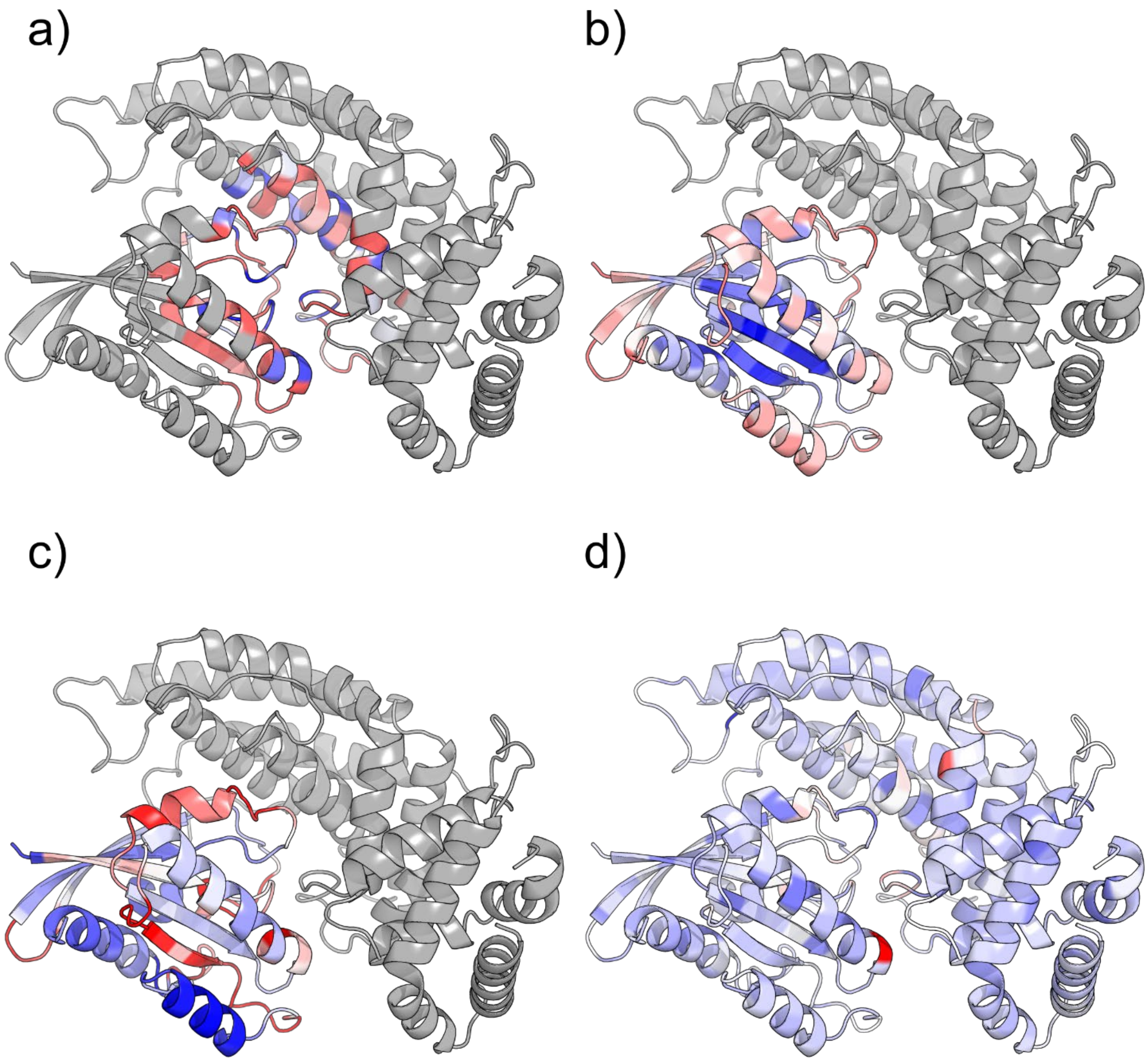

Supplementary Figure 16. Explained PDB structure 1WQ1 compared to stability predictors.

a) GNNExplainer, b) Amino Acids Interaction Webserver, c) Constraint Network Analysis, d) K-Fold. For this structure, we were not able to obtain Thermometer results. Blue indicates stable, red indicates unstable residues.

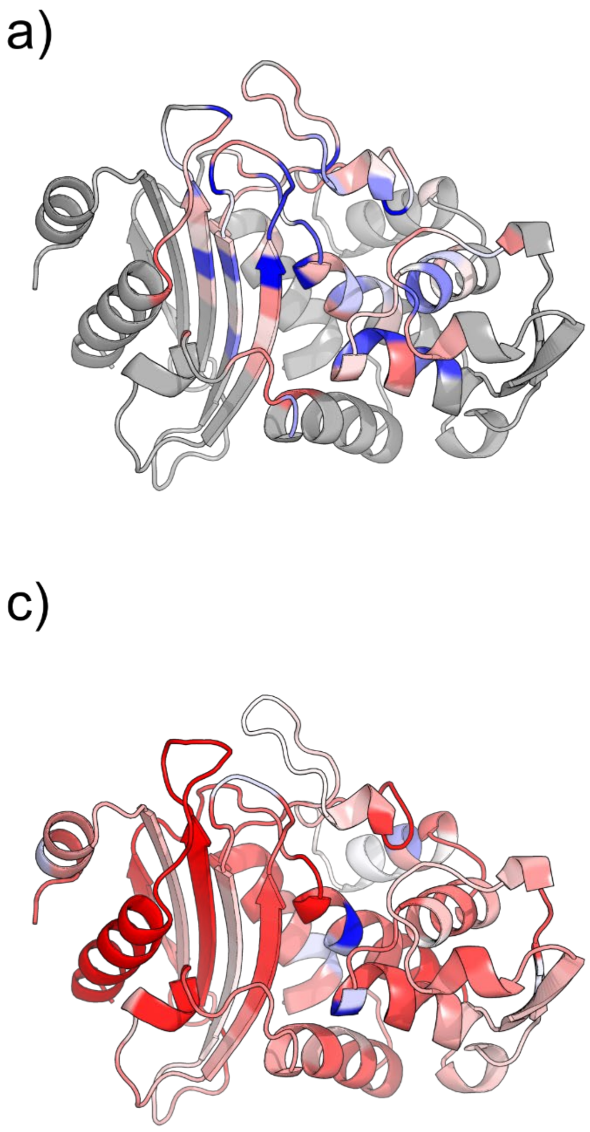

Supplementary Figure 17. Explained PDB structure 3BLM compared to stability predictors.

a) GNNExplainer, b) Amino Acids Interaction Webserver, c) Constraint Network Analysis, d) K-Fold. For this structure, we were not able to obtain Thermometer results. Blue indicates stable, red indicates unstable residues.

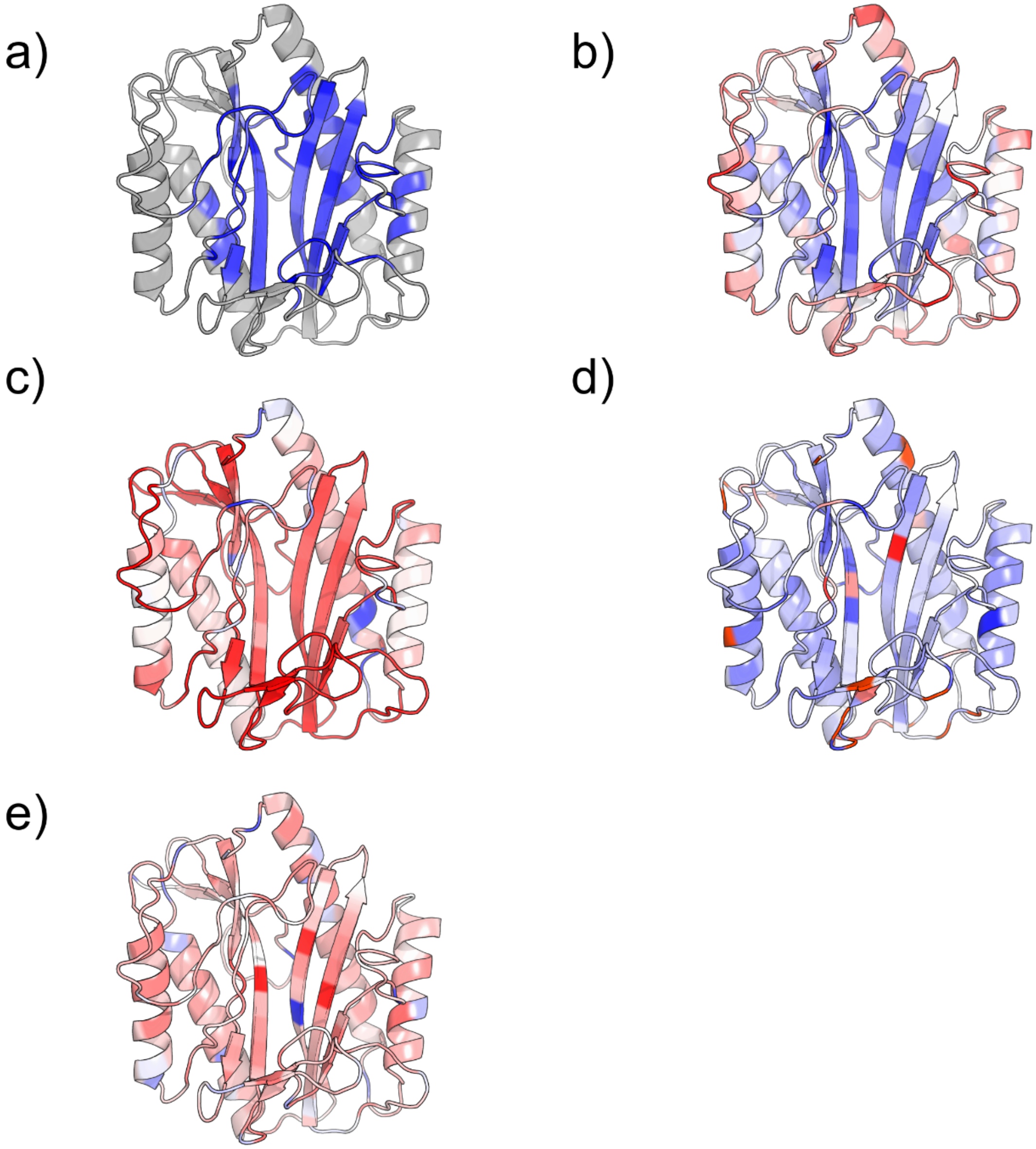

Supplementary Figure 18. Explained PDB structure 2MAT compared to stability.
a) GNNExplainer, b) Amino Acids Interaction Webserver, c) Constraint Network Analysis, d) K-Fold, and e) Thermometer. Blue indicates stable, red indicates unstable residues.

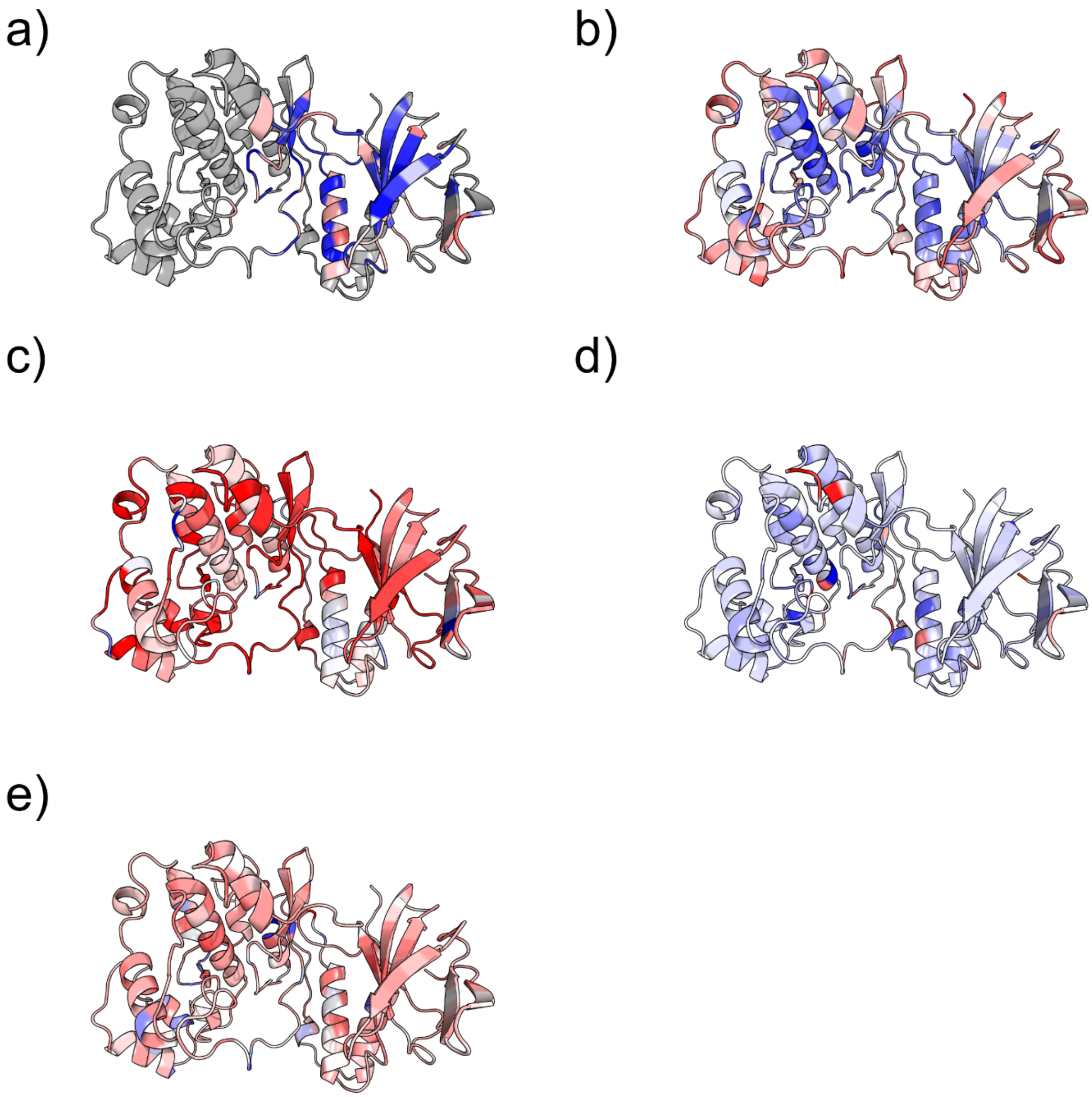

Supplementary Figure 19. Explained PDB structure 1A9U compared to stability predictors.

a) GNNExplainer, b) Amino Acids Interaction Webserver, c) Constraint Network Analysis,

d) K-Fold, and e) Thermometer. Blue indicates stable, red indicates unstable residues.

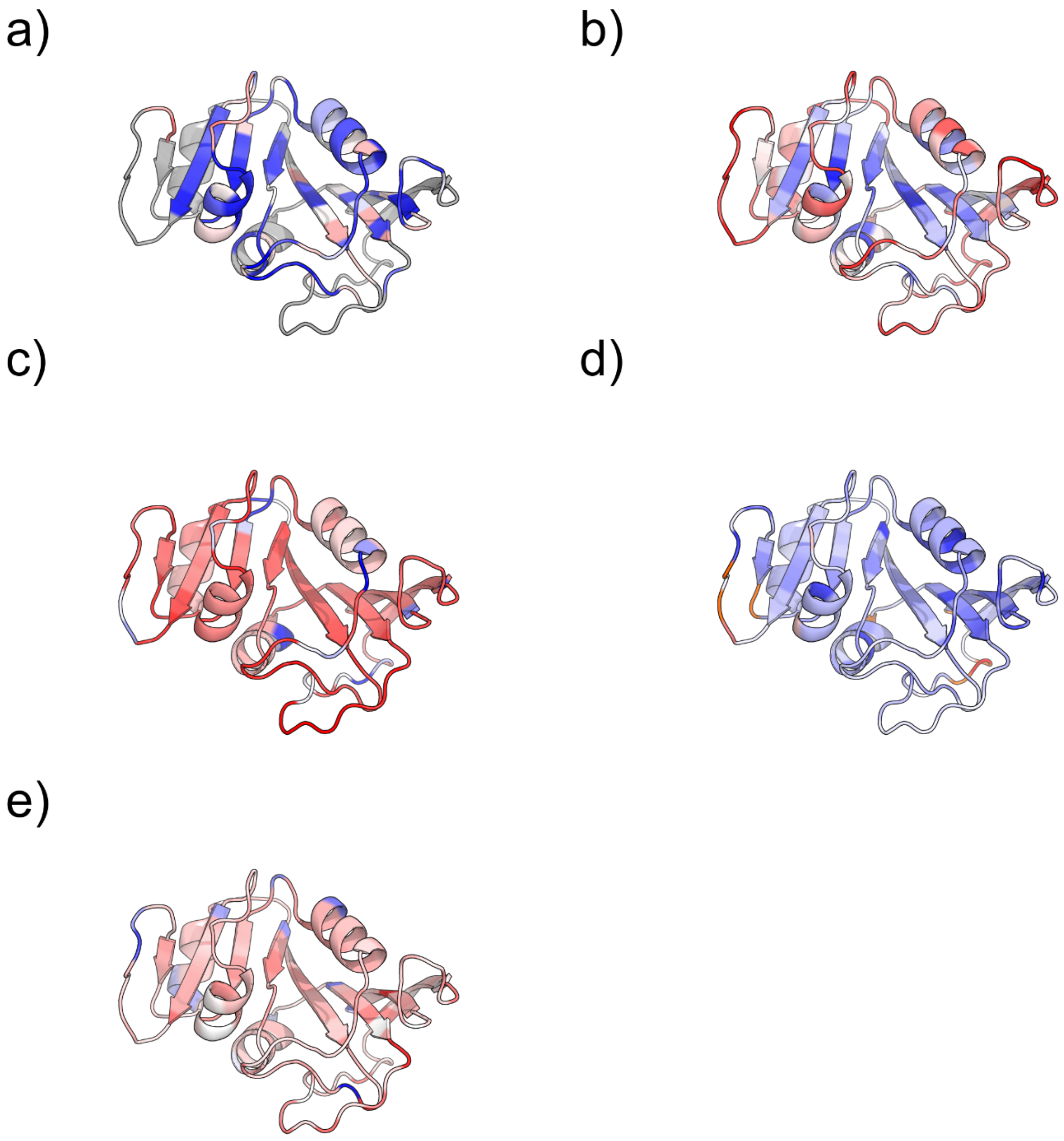

Supplementary Figure 20. Explained PDB structure 3DRC compared to stability predictors.

a) GNNExplainer, b) Amino Acids Interaction Webserver, c) Constraint Network Analysis, d) K-Fold, and e) Thermometer. Blue indicates stable, red indicates unstable residues.

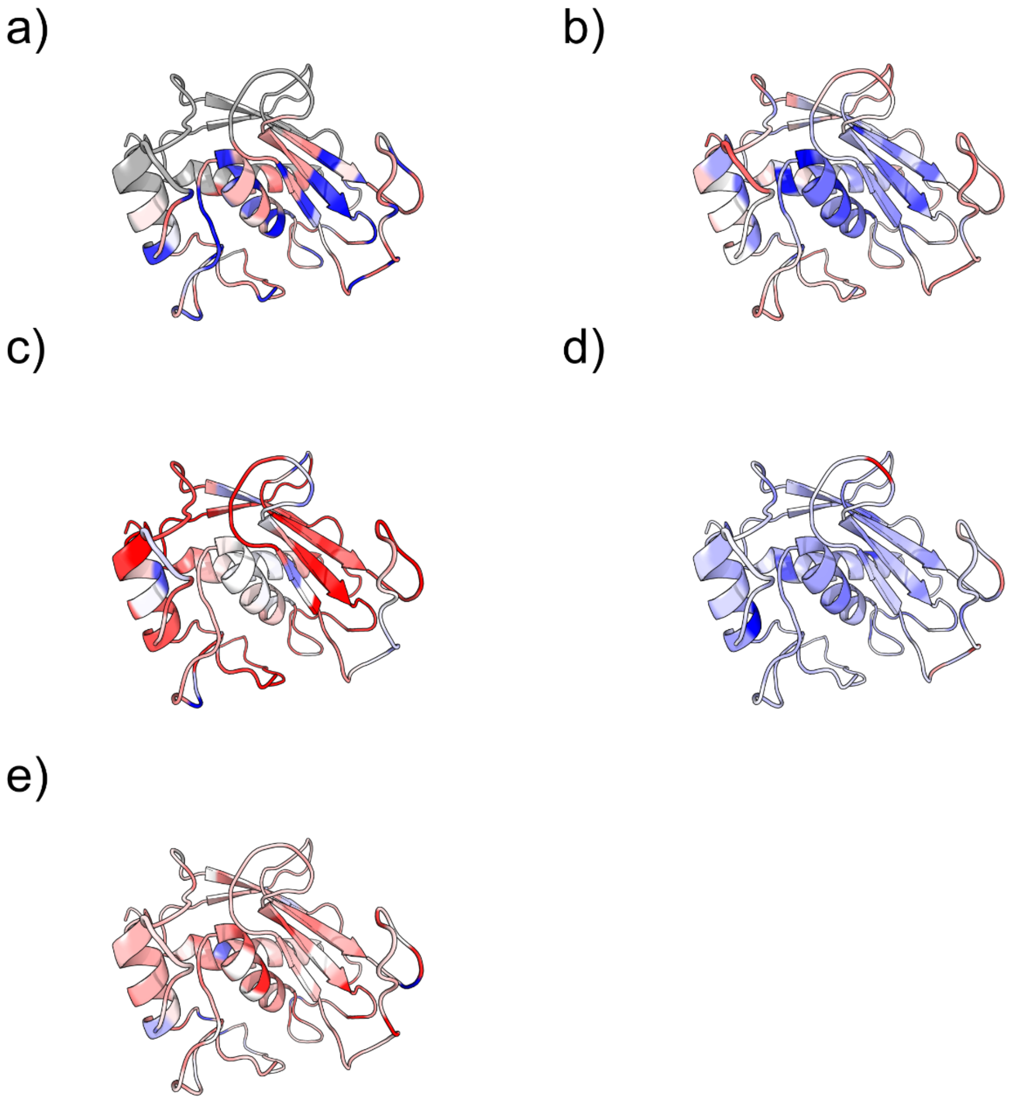

Supplementary Figure 21. Explained PDB structure 1BIW compared to stability predictors.

a) GNNExplainer, b) Amino Acids Interaction Webserver, c) Constraint Network Analysis, d) K-Fold, and e) Thermometer. Blue indicates stable, red indicates unstable residues.

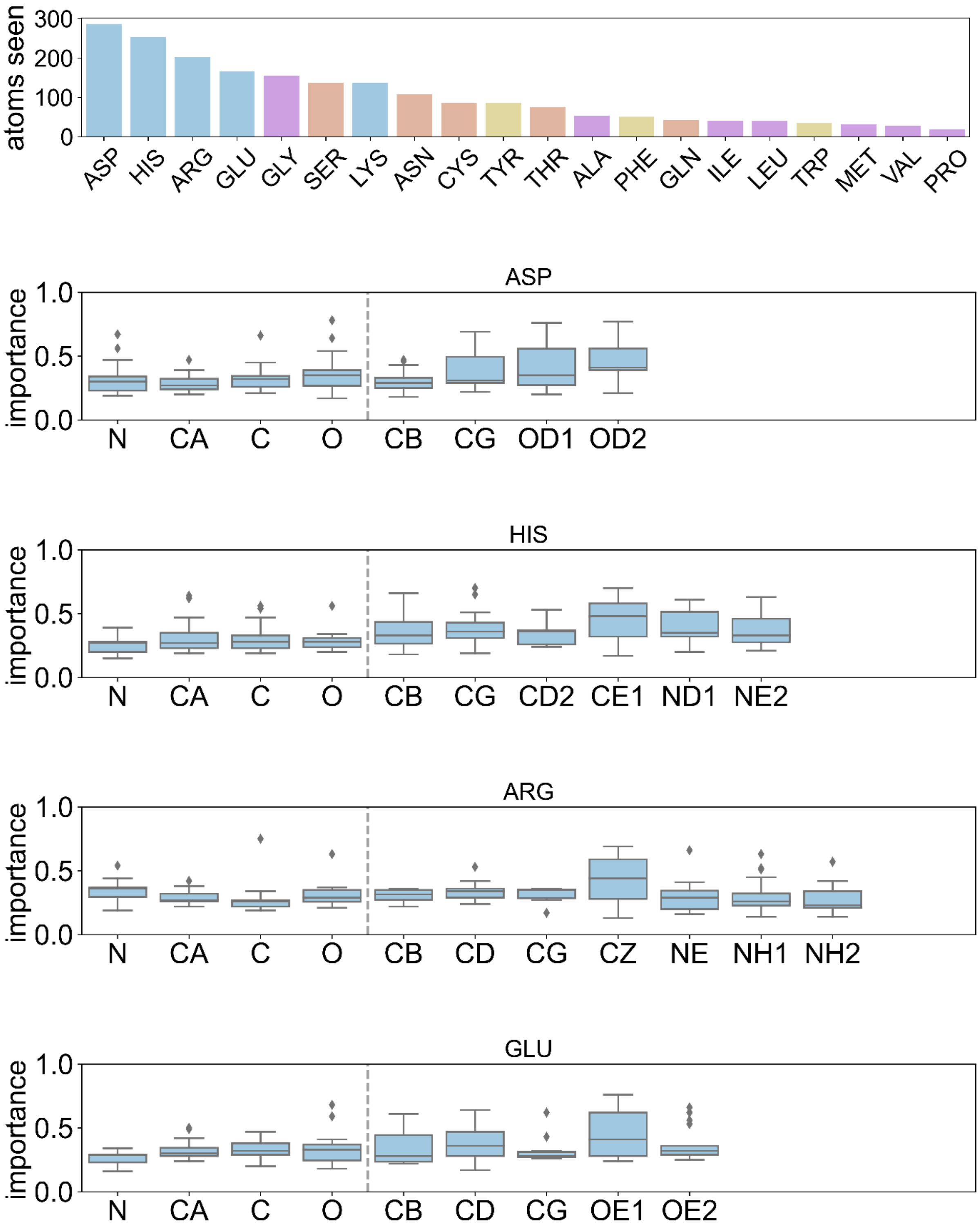
atoms seen
300
200
100
0
ASP HIS ARG GLU GLY SER LYS ASN CYS TYR THR ALA PHE GLN ILE LEU TRP MET VAL PRO
ASP
importance
1.0
0.5
0.0
N CA C O CB CG OD1 OD2
HIS
importance
1.0
0.5
0.0
N CA C O CB CG CD2 CE1 ND1 NE2
ARG
importance
1.0
0.5
0.0
N CA C O CB CD CG CZ NE NH1 NH2
GLU
importance
1.0
0.5
0.0
N CA C O CB CD CG OE1 OE2

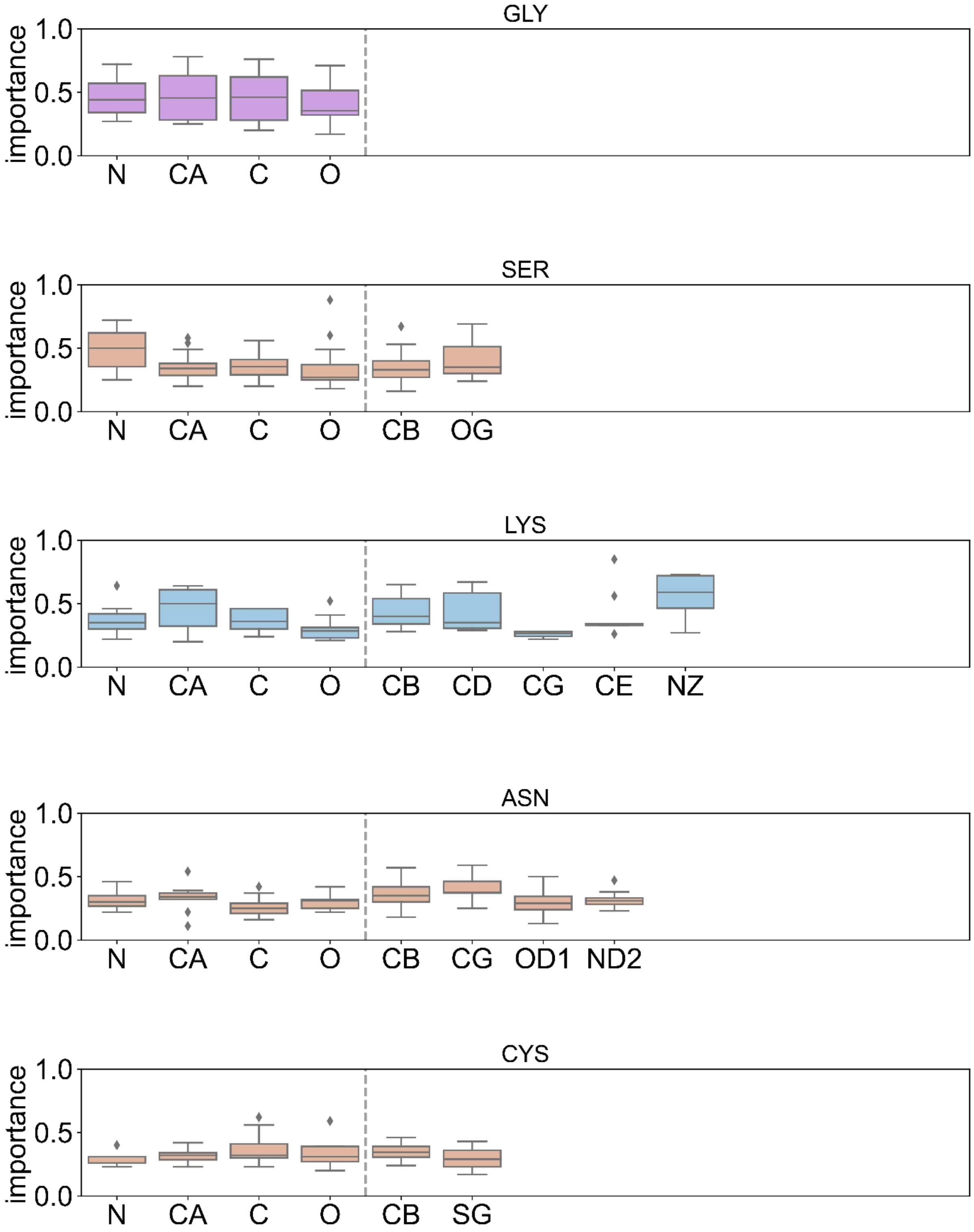
GLY
importance
1.0
0.5
0.0
N CA C O
SER
importance
1.0
0.5
0.0
N CA C O CB OG
LYS
importance
1.0
0.5
0.0
N CA C O CB CD CG CE NZ
ASN
importance
1.0
0.5
0.0
N CA C O CB CG OD1 ND2
CYS
importance
1.0
0.5
0.0
N CA C O CB SG

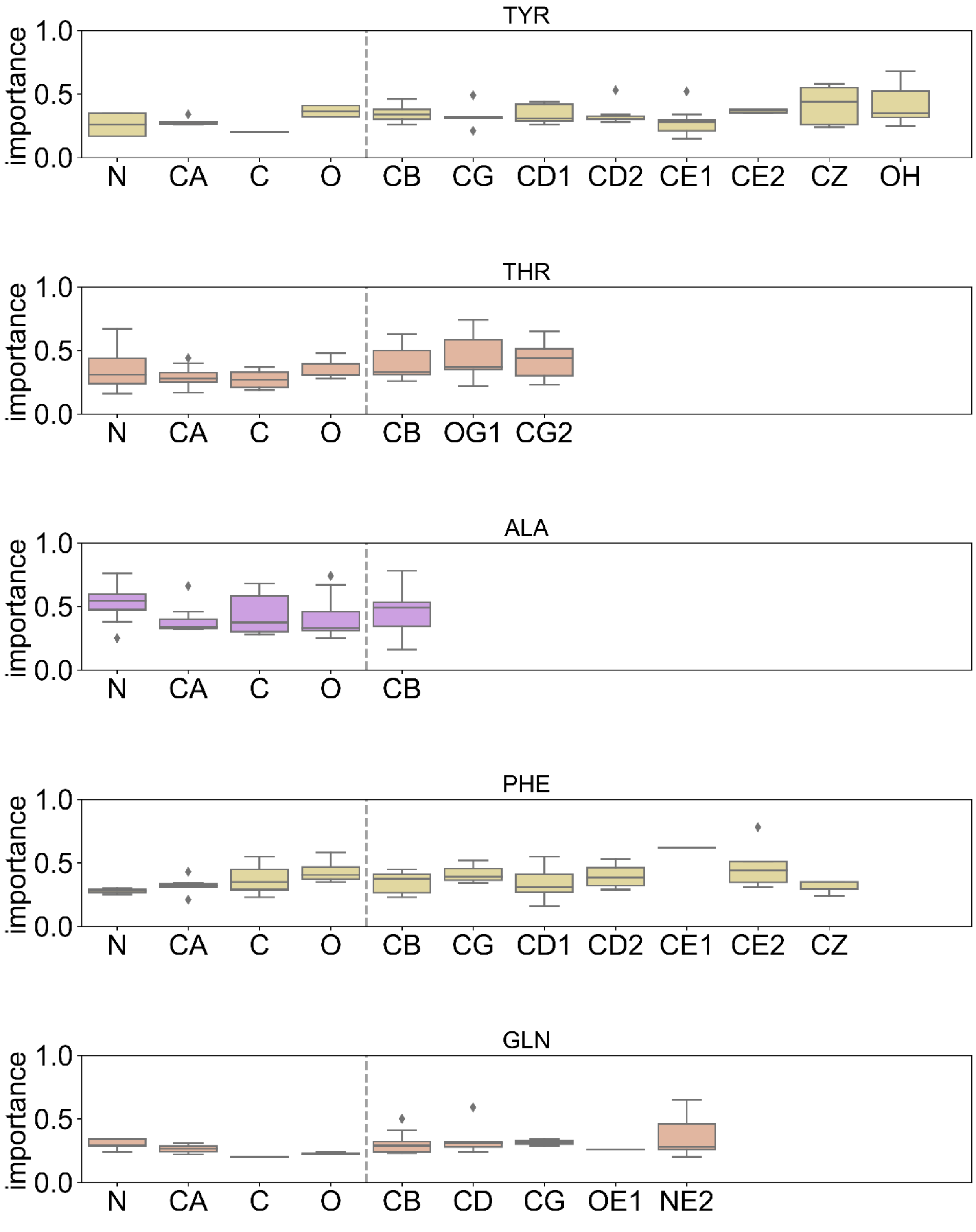
TYR
importance
1.0
0.5
0.0
N
CA
C
O
CB
CG
CD1
CD2
CE1
CE2
CZ
OH
THR
importance
1.0
0.5
0.0
N
CA
C
O
CB
OG1
CG2
ALA
importance
1.0
0.5
0.0
N
CA
C
O
CB
PHE
importance
1.0
0.5
0.0
N
CA
C
O
CB
CG
CD1
CD2
CE1
CE2
CZ
GLN
importance
1.0
0.5
0.0
N
CA
C
O
CB
CD
CG
OE1
NE2

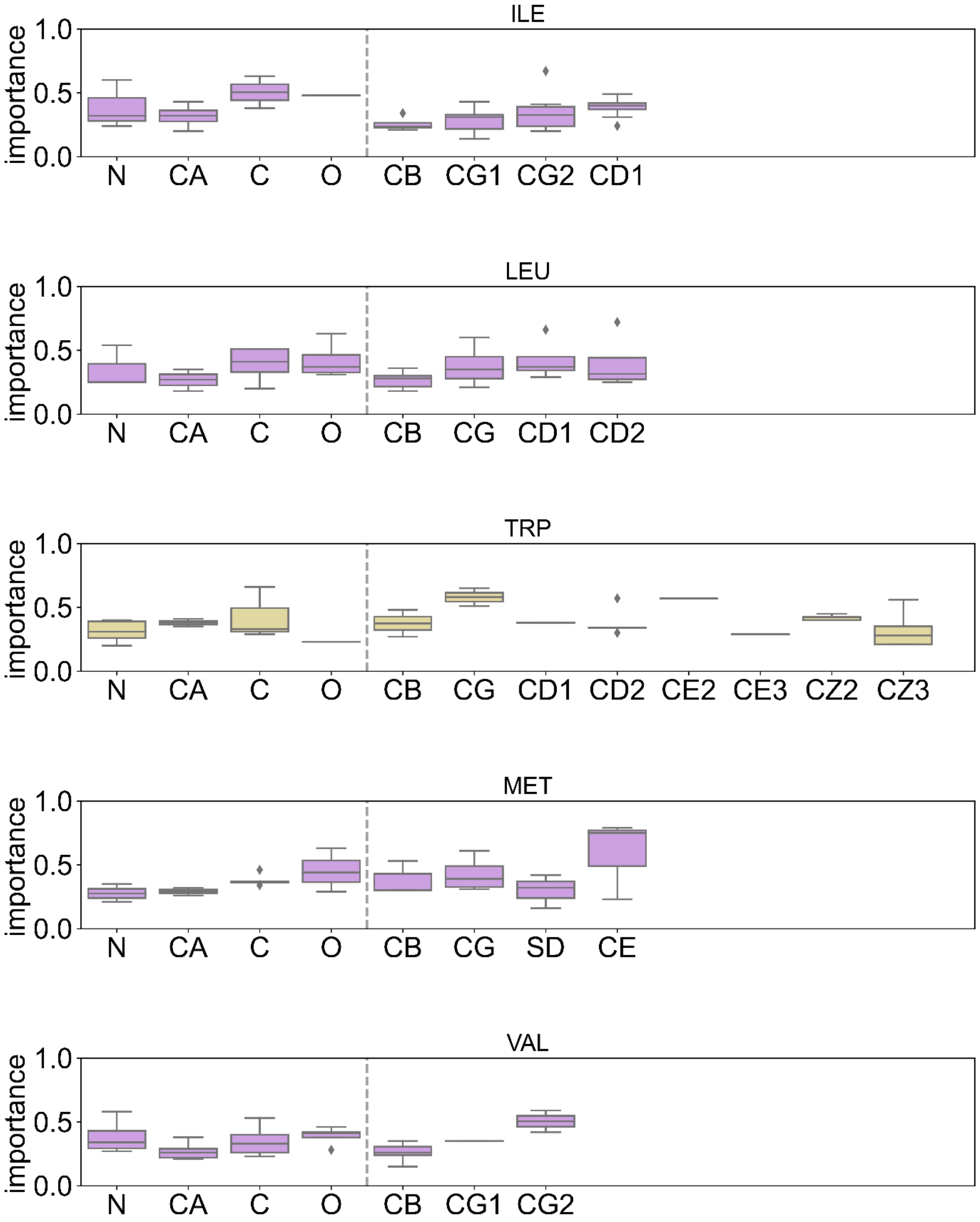
ILE
LEU
TRP
MET
VAL
importance
1.0
0.5
0.0
N
CA
C
O
CB
CG1
CG2
CD1
CG
CD2
CE2
CE3
CZ2
CZ3
SD
CE

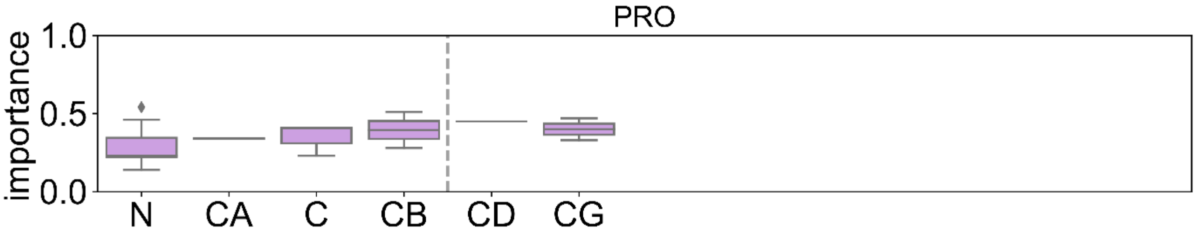

Supplementary Figure 22. Importance for all catalytic and binding atoms.

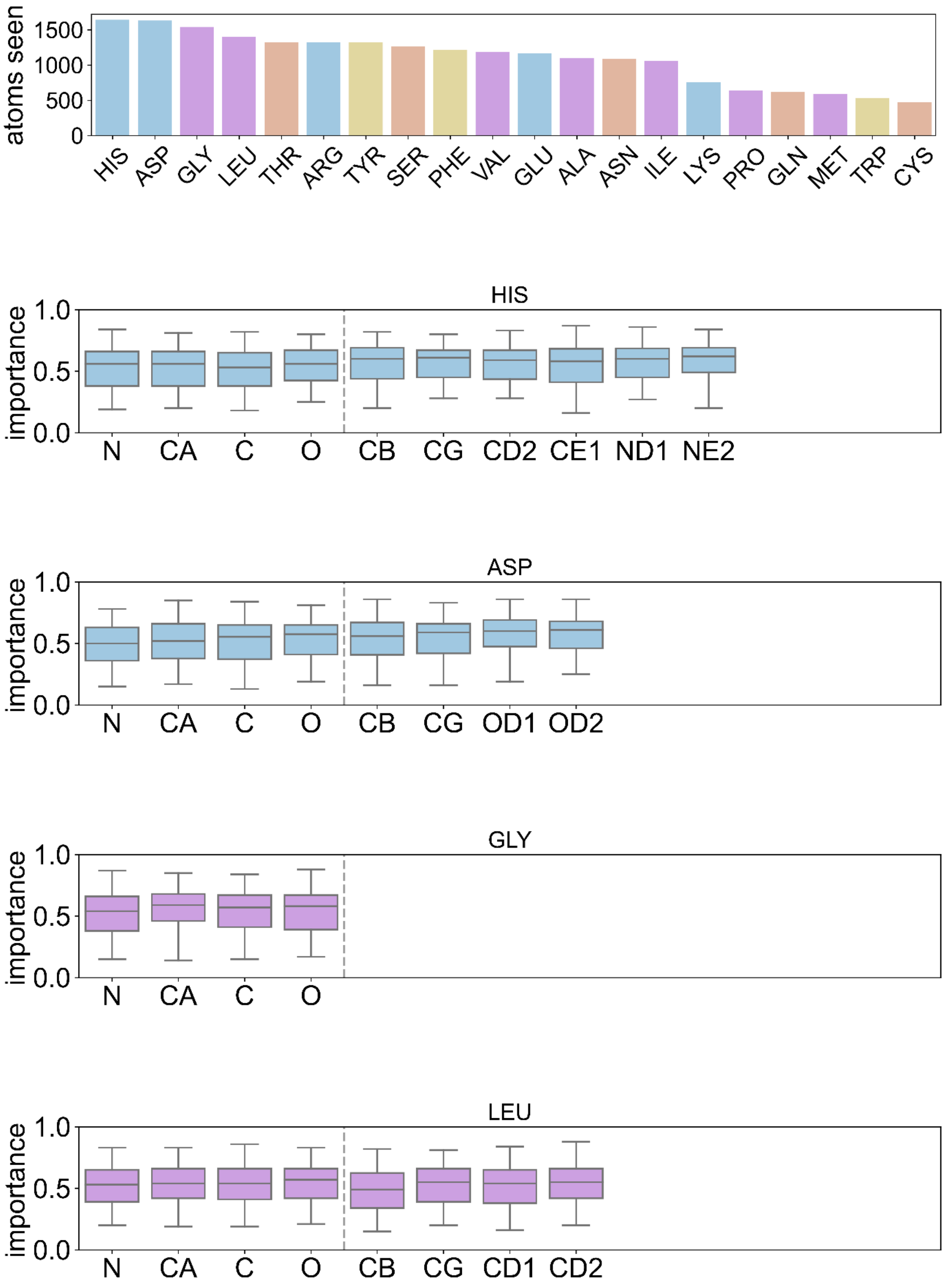
atoms seen
0
500
1000
1500
HIS ASP GLY LEU THR ARG TYR SER PHE VAL GLU ALA ASN ILE LYS PRO GLN MET TRP CYS
HIS
importance
0.0
0.5
1.0
N CA C O CB CG CD2 CE1 ND1 NE2
ASP
importance
0.0
0.5
1.0
N CA C O CB CG OD1 OD2
GLY
importance
0.0
0.5
1.0
N CA C O
LEU
importance
0.0
0.5
1.0
N CA C O CB CG CD1 CD2

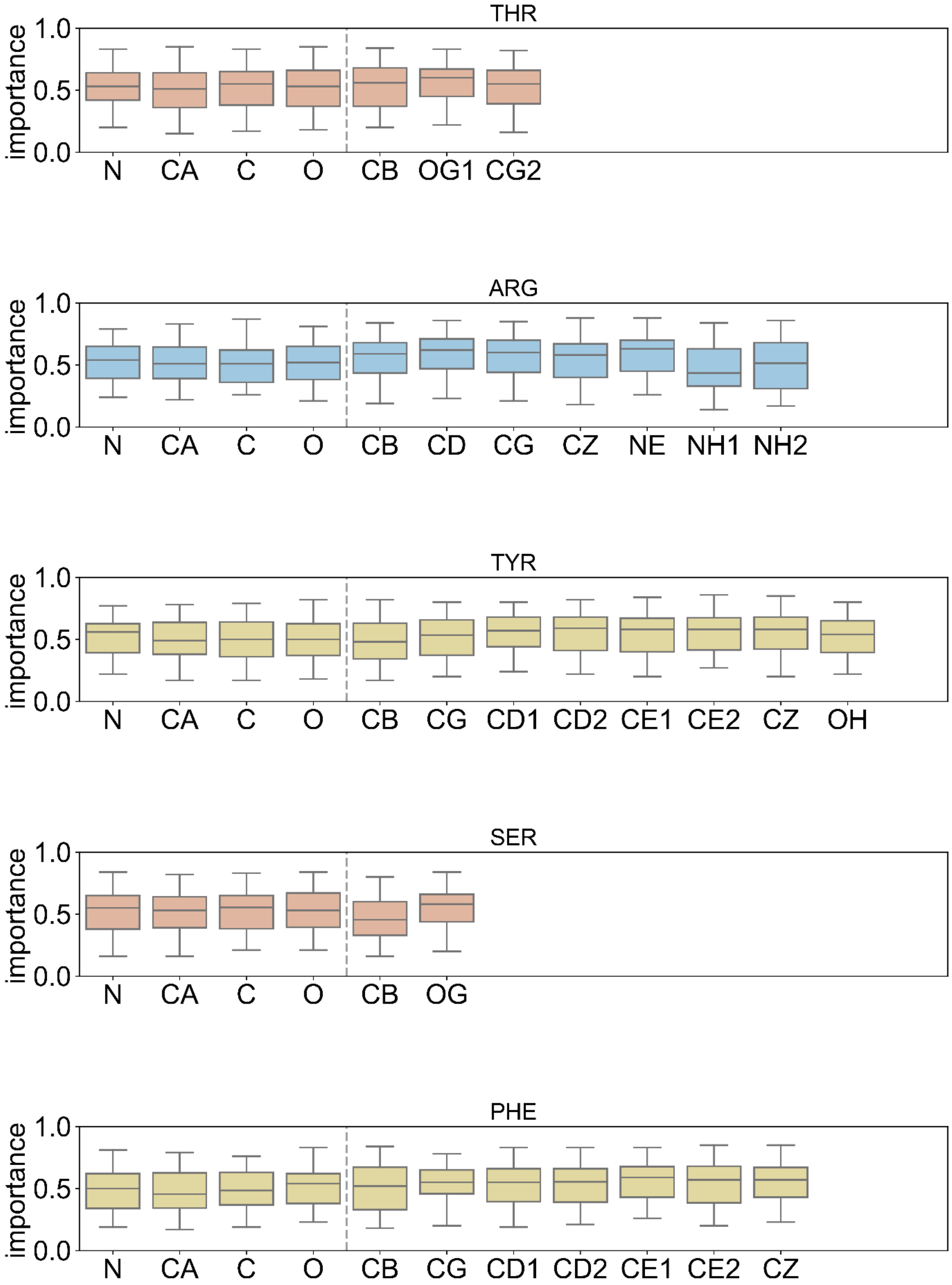
THR
importance
1.0
0.5
0.0
N CA C O CB OG1 CG2
ARG
importance
1.0
0.5
0.0
N CA C O CB CD CG CZ NE NH1 NH2
TYR
importance
1.0
0.5
0.0
N CA C O CB CG CD1 CD2 CE1 CE2 CZ OH
SER
importance
1.0
0.5
0.0
N CA C O CB OG
PHE
importance
1.0
0.5
0.0
N CA C O CB CG CD1 CD2 CE1 CE2 CZ

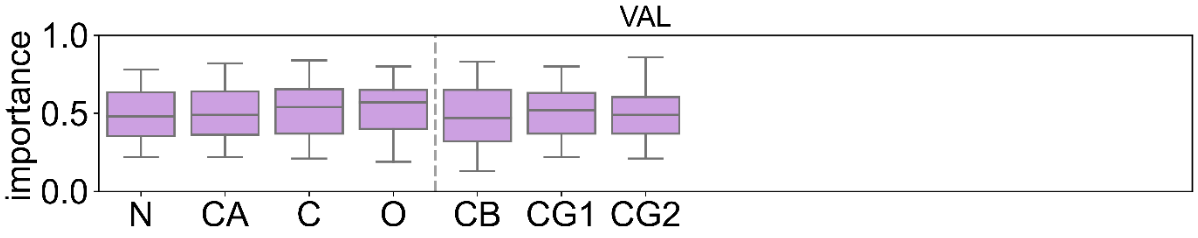
VAL
importance
1.0
0.5
0.0
N
CA
C
O
CB
CG1
CG2

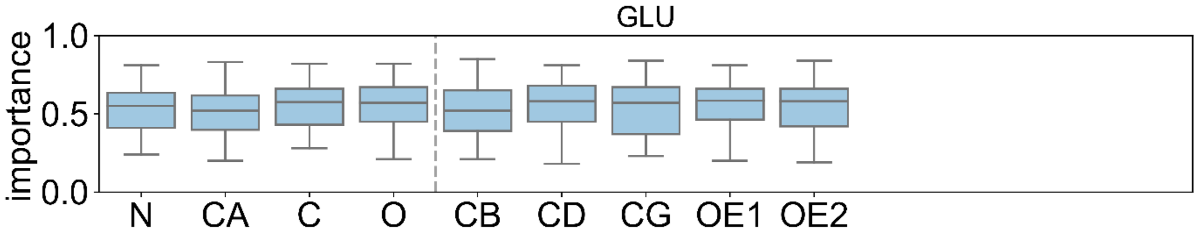
GLU
importance
1.0
0.5
0.0
N
CA
C
O
CB
CD
CG
OE1
OE2

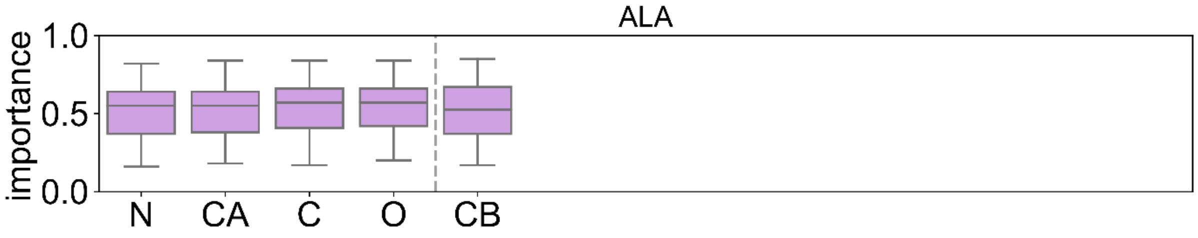
ALA
importance
1.0
0.5
0.0
N
CA
C
O
CB

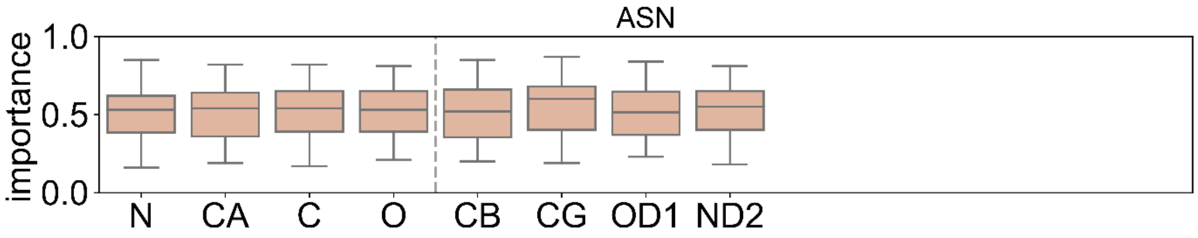
ASN
importance
1.0
0.5
0.0
N
CA
C
O
CB
CG
OD1
ND2

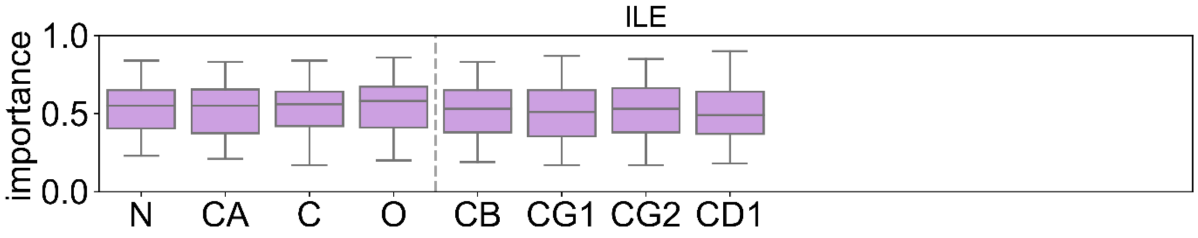
ILE
importance
1.0
0.5
0.0
N
CA
C
O
CB
CG1
CG2
CD1

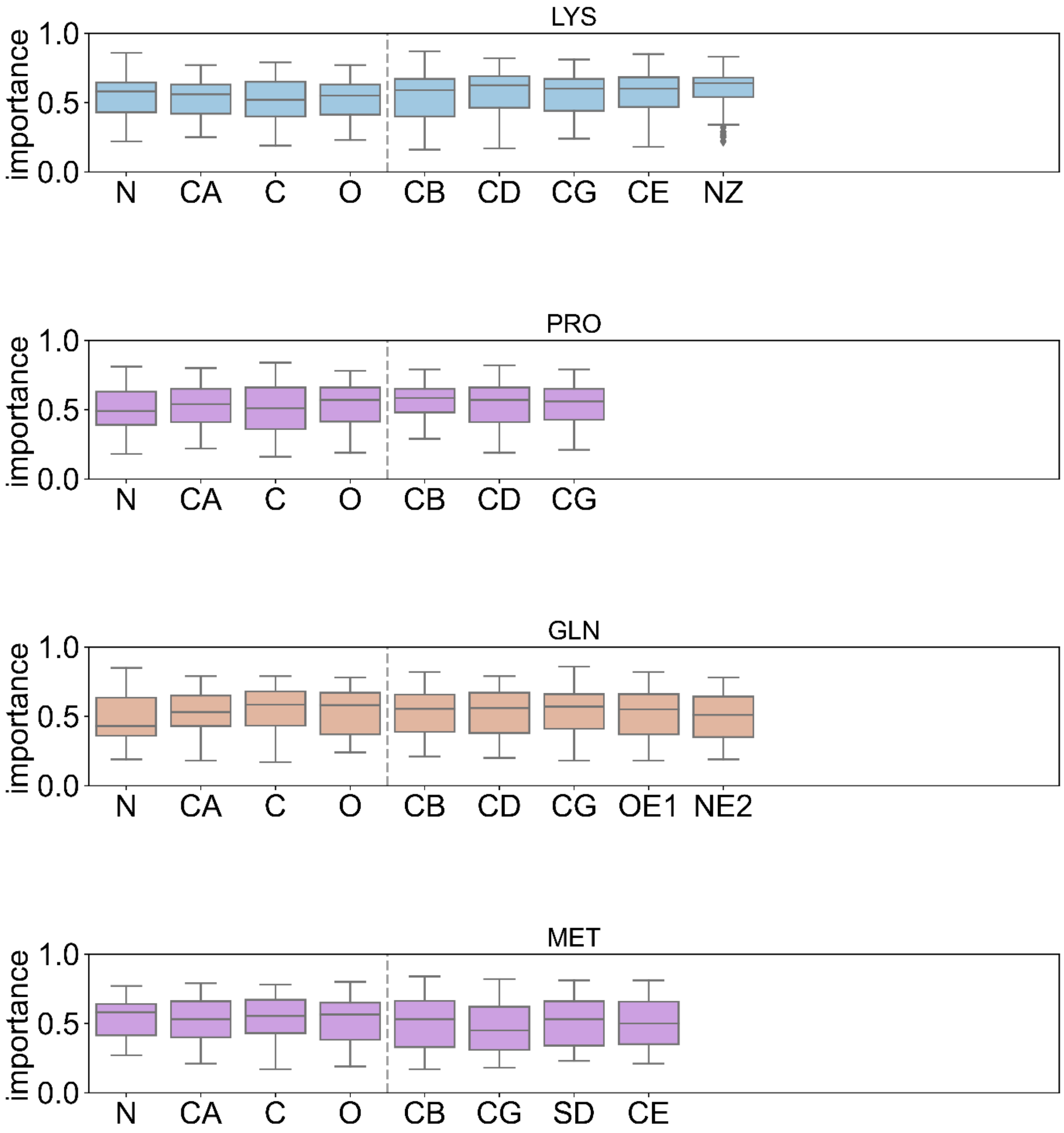
LYS
importance
1.0
0.5
0.0
N
CA
C
O
CB
CD
CG
CE
NZ
PRO
importance
1.0
0.5
0.0
N
CA
C
O
CB
CD
CG
GLN
importance
1.0
0.5
0.0
N
CA
C
O
CB
CD
CG
OE1
NE2
MET
importance
1.0
0.5
0.0
N
CA
C
O
CB
CG
SD
CE

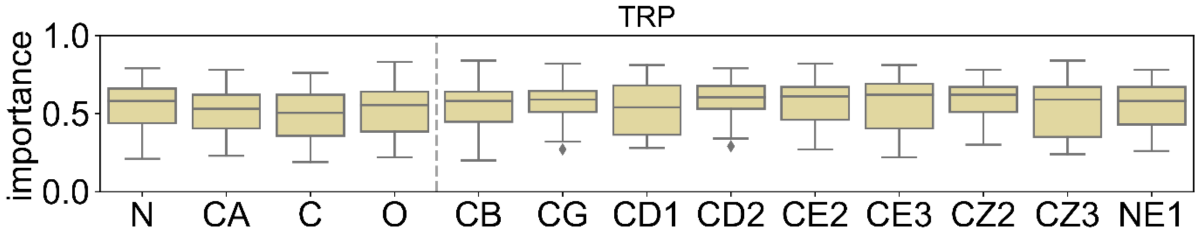

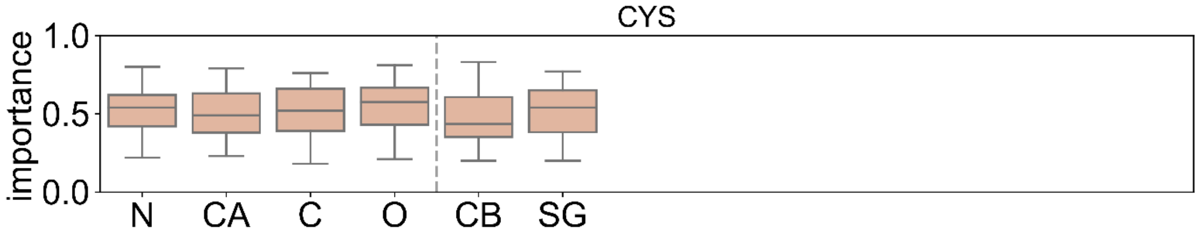

Supplementary Figure 23. Importance for all non-catalytic and non-binding atoms.

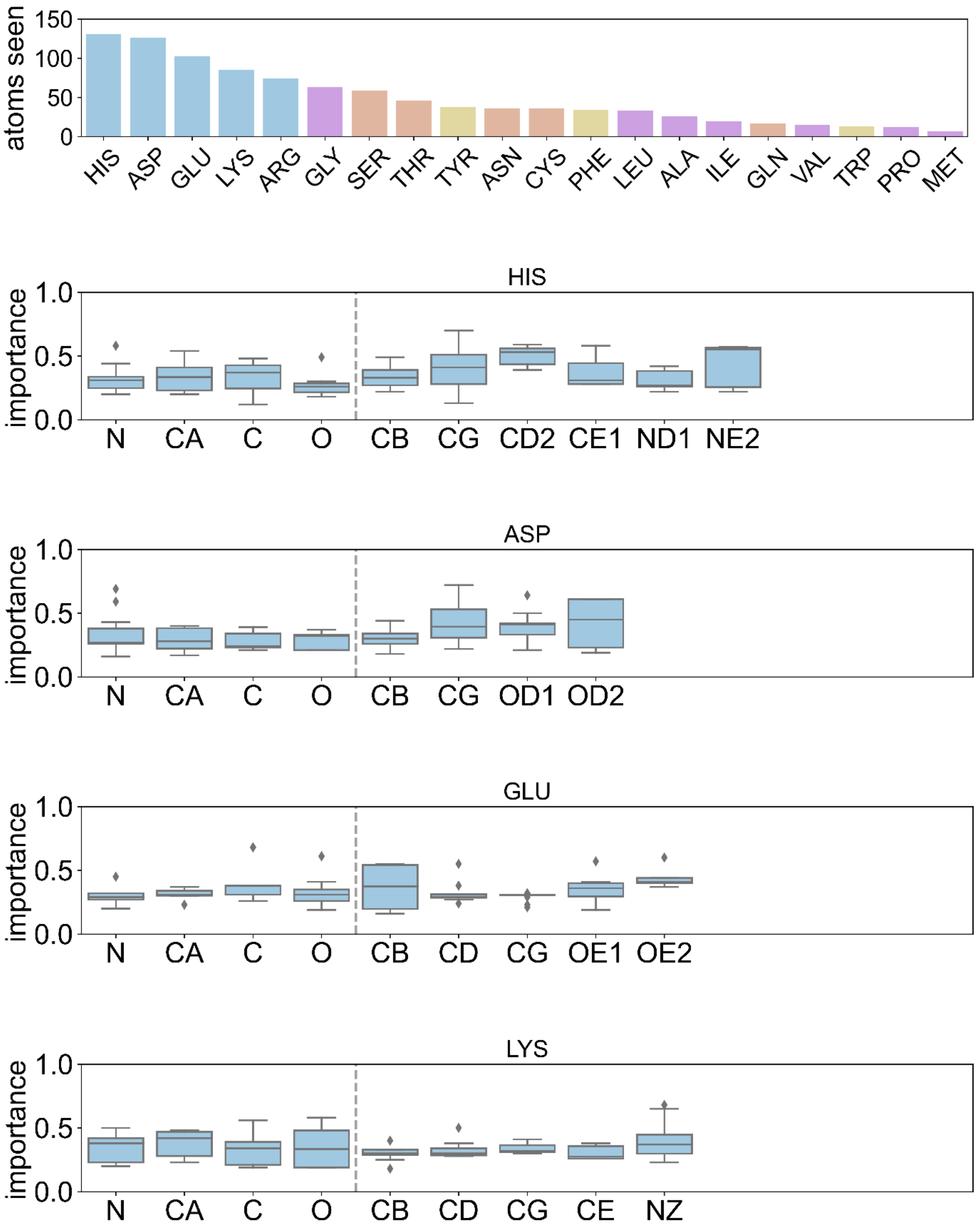
atoms seen
150
100
50
0
HIS ASP GLU LYS ARG GLY SER THR TYR ASN CYS PHE LEU ALA ILE GLN VAL TRP PRO MET
HIS
importance
1.0
0.5
0.0
N CA C O CB CG CD2 CE1 ND1 NE2
ASP
importance
1.0
0.5
0.0
N CA C O CB CG OD1 OD2
GLU
importance
1.0
0.5
0.0
N CA C O CB CD CG OE1 OE2
LYS
importance
1.0
0.5
0.0
N CA C O CB CD CG CE NZ

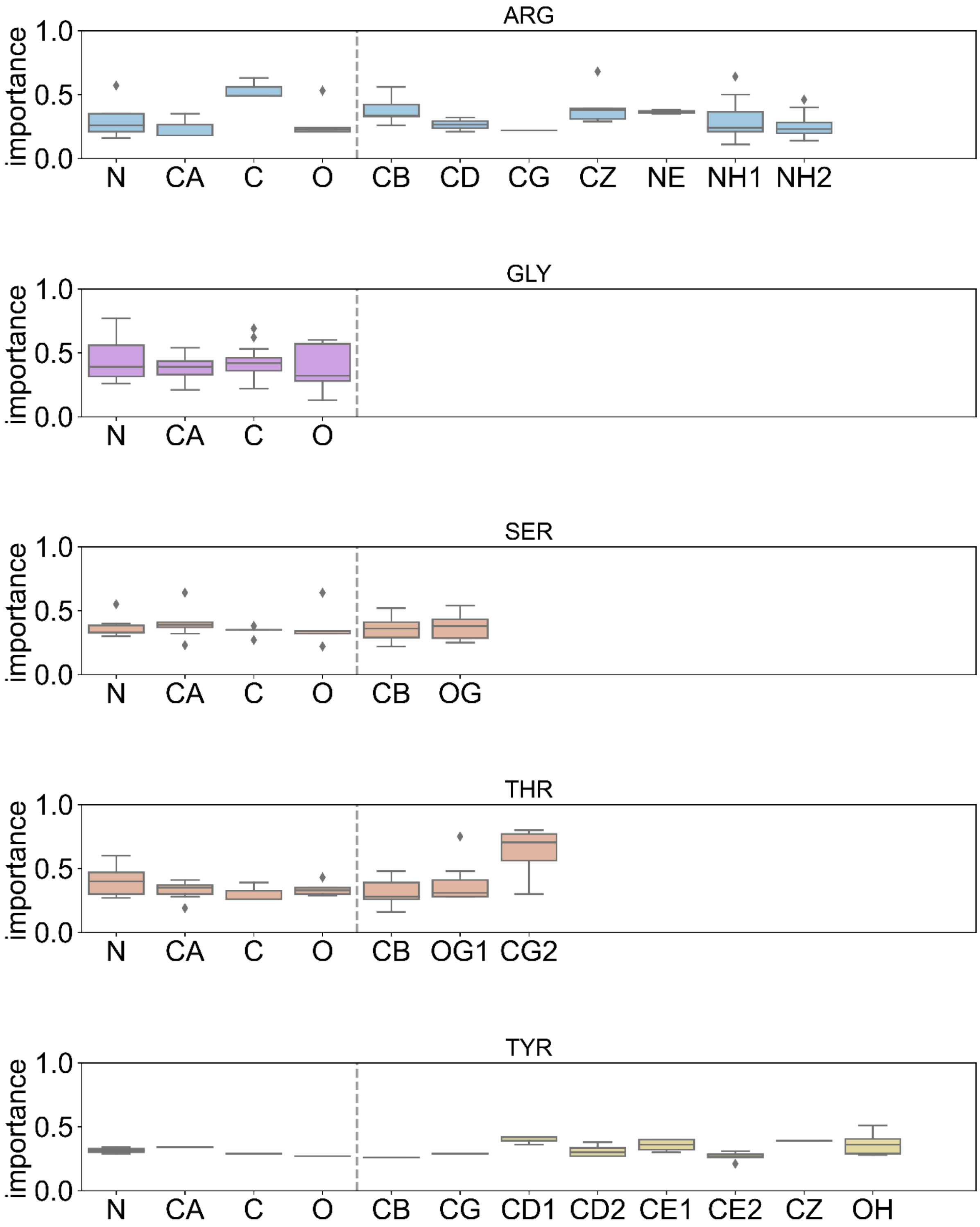

ARG
GLY
SER
THR
TYR
importance
1.0
0.5
0.0
N
CA
C
O
CB
CD
CG
CZ
NE
NH1
NH2
OG
OG1
CG2
CD1
CD2
CE1
CE2
OH

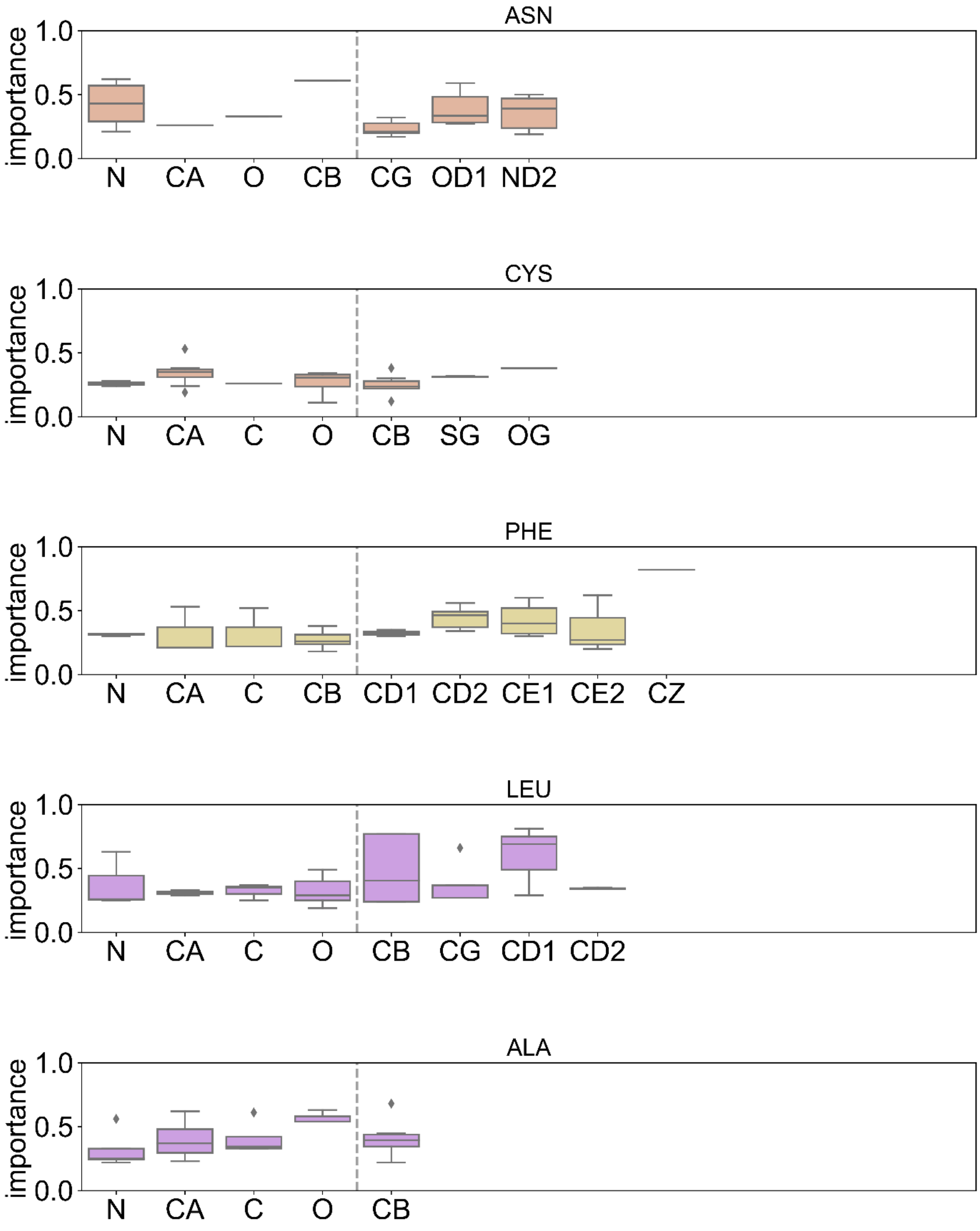
ASN
importance
1.0
0.5
0.0
N CA O CB CG OD1 ND2
CYS
importance
1.0
0.5
0.0
N CA C O CB SG OG
PHE
importance
1.0
0.5
0.0
N CA C CB CD1 CD2 CE1 CE2 CZ
LEU
importance
1.0
0.5
0.0
N CA C O CB CG CD1 CD2
ALA
importance
1.0
0.5
0.0
N CA C O CB

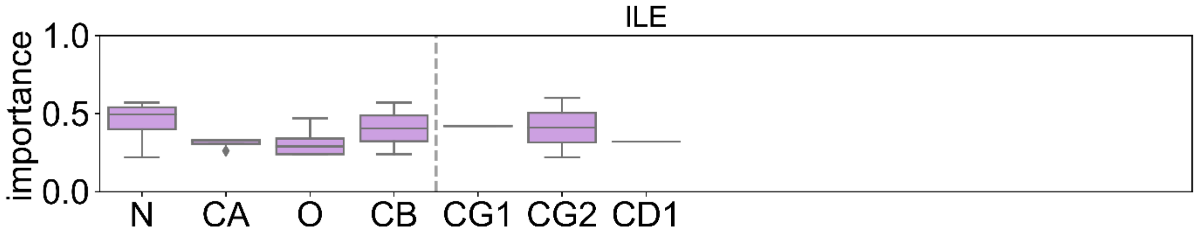
ILE
importance
1.0
0.5
0.0
N
CA
O
CB
CG1
CG2
CD1

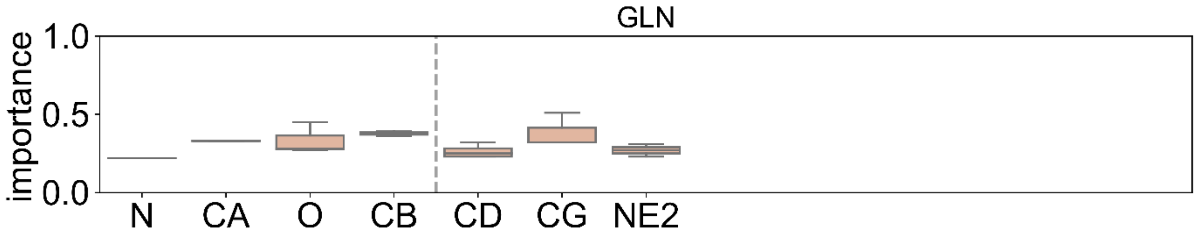
GLN
importance
1.0
0.5
0.0
N
CA
O
CB
CD
CG
NE2

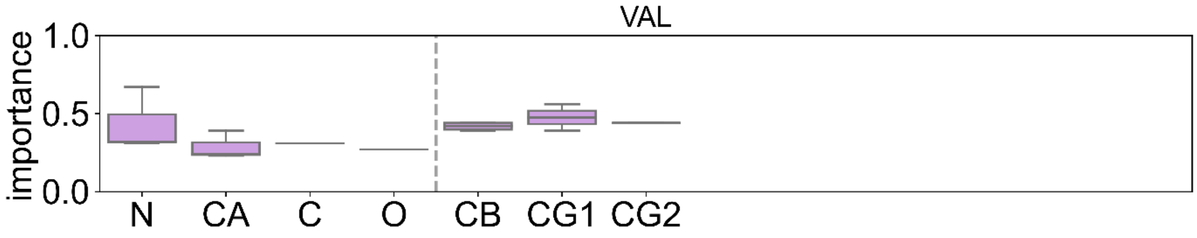
VAL
importance
1.0
0.5
0.0
N
CA
C
O
CB
CG1
CG2

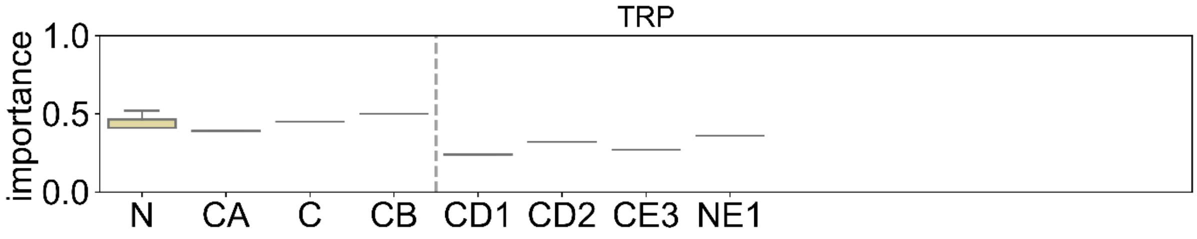
TRP
importance
1.0
0.5
0.0
N
CA
C
CB
CD1
CD2
CE3
NE1

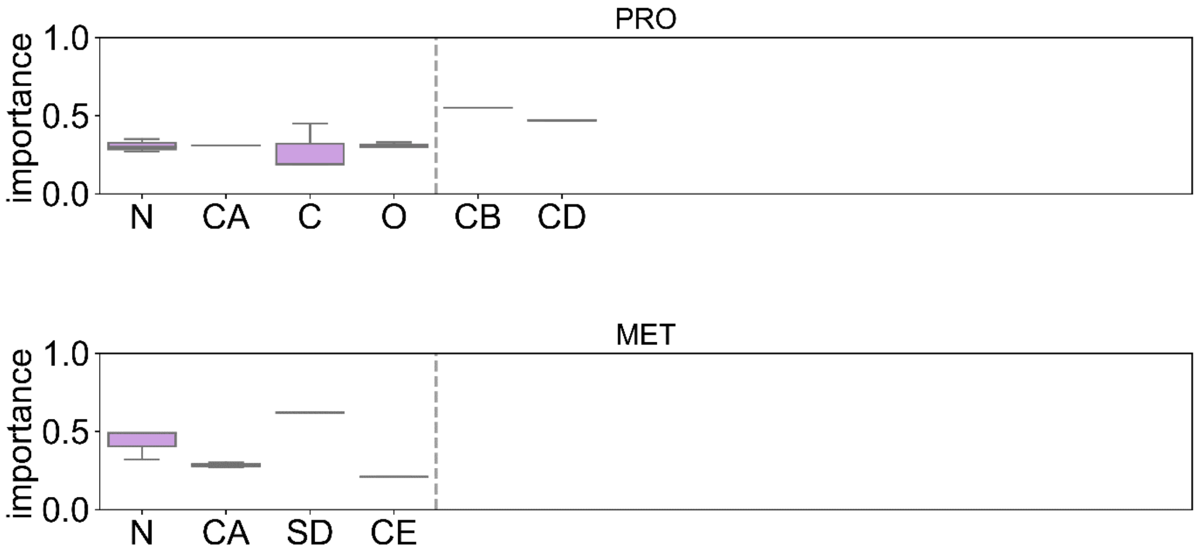

Supplementary Figure 24. Importance for all catalytic and binding atoms in wrongly predicted enzymes.
