# Supplementary material for: TopEC: prediction of Enzyme Commission classes by 3D graph neural networks and localized 3D protein descriptor
Source: Nat Commun. 2025 Mar 20;16:2737. doi: 10.1038/s41467-025-57324-5 (PMC11923149; doi:10.1038/s41467-025-57324-5)
Supplement: Supplementary file 2 — Description of Additional Supplementary Files [file 41467_2025_57324_MOESM2_ESM.docx]

**Description of Additional Supplementary Files**

**Supplementary Data 1:** PyCM reports for all networks trained for the manuscript.

**Supplementary Data 2:** Modified Price and PrOSPECCTs dataset. Contains a list of PDBs for each sub dataset.
