# Supplementary material for: TopEC: prediction of Enzyme Commission classes by 3D graph neural networks and localized 3D protein descriptor
Source: Nat Commun. 2025 Mar 20;16:2737. doi: 10.1038/s41467-025-57324-5 (PMC11923149; doi:10.1038/s41467-025-57324-5)
Supplement: Supplementary file 3 — Supplementary Data 1 [file 41467_2025_57324_MOESM3_ESM.zip › Data_S1/figure2/PDB300_sub.html]

PyCM Report


# PyCM Report

## Dataset Type :

- Multi-Class Classification
- Imbalanced

Note 1 : Recommended statistics for this type of classification highlighted in aqua

Note 2 : The recommender system assumes that the input is the result of classification over the whole data rather than just a part of it.
If the confusion matrix is the result of test data classification, the recommendation is not valid.

## Confusion Matrix :

|  |  |  |  |  |  |  |  |  |  |  |  |  |  |  |  |  |  |  |  |  |  |  |  |  |  |  |  |  |  |  |  |  |  |  |  |  |  |  |  |  |  |  |  |  |  |  |  |  |  |  |  |  |  |  |  |  |  |  |  |  |  |  |  |  |  |  |  |  |  |  |  |  |  |  |  |  |  |  |  |  |  |  |  |  |  |  |  |  |  |  |  |  |  |  |  |  |  |  |  |  |  |  |  |  |  |  |  |  |  |  |  |  |  |  |  |  |  |  |  |  |  |  |  |  |  |  |  |  |  |  |  |  |  |  |  |  |  |  |  |  |  |  |  |  |  |  |  |  |  |  |  |  |  |  |  |  |  |  |  |  |  |  |  |  |  |  |  |  |  |  |  |  |  |  |  |  |  |  |  |  |  |  |  |  |  |  |  |  |  |  |  |  |  |  |  |  |  |  |  |  |  |  |  |  |  |  |  |  |  |  |  |  |  |  |  |  |  |  |  |  |  |  |  |  |  |  |  |  |  |  |  |  |  |  |  |  |  |  |  |  |  |  |  |  |  |  |  |  |  |  |  |  |  |  |  |  |  |  |  |  |  |  |  |  |  |  |  |  |  |  |  |  |  |  |  |  |  |  |  |  |  |  |  |  |  |  |  |  |  |  |  |  |  |  |  |  |  |  |  |  |  |  |  |  |  |  |  |  |  |  |  |  |  |  |  |  |  |  |  |  |  |  |  |  |  |  |  |  |  |  |  |  |  |  |  |  |  |  |  |  |  |  |  |  |  |  |  |  |  |  |  |  |  |  |  |  |  |  |  |  |  |  |  |  |  |  |  |  |  |  |  |  |  |  |  |  |  |  |  |  |  |  |  |  |  |  |  |  |  |  |  |  |  |  |  |  |  |  |  |  |  |  |  |  |  |  |  |  |  |  |  |  |  |  |  |  |  |  |  |  |  |  |  |  |  |  |  |  |  |  |  |  |  |  |  |  |  |  |  |  |  |  |  |  |  |  |  |  |  |  |  |  |  |  |  |  |  |  |  |  |  |  |  |  |  |  |  |  |  |  |  |  |  |  |  |  |  |  |  |  |  |  |  |  |  |  |  |  |  |  |  |  |  |  |  |  |  |  |  |  |  |  |  |  |  |  |  |  |  |  |  |  |  |  |  |  |  |  |  |  |  |  |  |  |  |  |  |  |  |  |  |  |  |  |  |  |  |  |  |  |  |  |  |  |  |  |  |  |  |  |  |  |  |  |  |  |  |  |  |  |  |  |  |  |  |  |  |  |  |  |  |  |  |  |  |  |  |  |  |  |  |  |  |  |  |  |  |  |  |  |  |  |  |  |  |  |  |  |  |  |  |  |  |  |  |  |  |  |  |  |  |  |  |  |  |  |  |  |  |  |  |  |  |  |  |  |  |  |  |  |  |  |  |  |  |  |  |  |  |  |  |  |  |  |  |  |  |  |  |  |  |  |  |  |  |  |  |  |  |  |  |  |  |  |  |  |  |  |  |  |  |  |  |  |  |  |  |  |  |  |  |  |  |  |  |  |  |  |  |  |  |  |  |  |  |  |  |  |  |  |  |  |  |  |  |  |  |  |  |  |  |  |  |  |  |  |  |  |  |  |  |  |  |  |  |  |  |  |  |  |  |  |  |  |  |  |  |  |  |  |  |  |  |  |  |  |  |  |  |  |  |  |  |  |  |  |  |  |  |  |  |  |  |  |  |  |  |  |  |  |  |  |  |  |  |  |  |  |  |  |  |  |  |  |  |  |  |  |  |  |  |  |  |  |  |  |  |  |  |  |  |  |  |  |  |  |  |  |  |  |  |  |  |  |  |  |  |  |  |  |  |  |  |  |  |  |  |  |  |  |  |  |  |  |  |  |  |  |  |  |  |  |  |  |  |  |  |  |  |  |  |  |  |  |  |  |  |  |  |  |  |  |  |  |  |  |  |  |  |  |  |  |  |  |  |  |  |  |  |  |  |  |  |  |  |  |  |  |  |  |  |  |  |  |  |  |  |  |  |  |  |  |  |  |  |  |  |  |  |  |  |  |  |  |  |  |  |  |  |  |  |  |  |  |  |  |  |  |  |  |  |  |  |  |  |  |  |  |  |  |  |  |  |  |  |  |  |  |  |  |  |  |  |  |  |  |  |  |  |  |  |  |  |  |  |  |  |  |  |  |  |  |  |  |  |  |  |  |  |  |  |  |  |  |  |  |  |  |  |  |  |  |  |  |  |  |  |  |  |  |  |  |  |  |  |  |  |  |  |  |  |  |  |  |  |  |  |  |  |  |  |  |  |  |  |  |  |  |  |  |  |  |  |  |  |  |  |  |  |  |  |  |  |  |  |  |  |  |  |  |  |  |  |  |  |  |  |  |  |  |  |  |  |  |  |  |  |  |  |  |  |  |  |  |  |  |  |  |  |  |  |  |  |  |  |  |  |  |  |  |  |  |  |  |  |  |  |  |  |  |  |  |  |  |  |  |  |  |  |  |  |  |  |  |  |  |  |  |  |  |  |  |  |  |  |  |  |  |  |  |  |  |  |  |  |  |  |  |  |  |  |  |  |  |  |  |  |  |  |  |  |  |  |  |  |  |  |  |  |  |  |  |  |  |  |  |  |  |  |  |  |  |  |  |  |  |  |  |  |  |  |  |  |  |  |  |  |  |  |  |  |  |  |  |  |  |  |  |  |  |  |  |  |  |  |  |  |  |  |  |  |  |  |  |  |  |  |  |  |  |  |  |  |  |  |  |  |  |  |  |  |  |  |  |  |  |  |  |  |  |  |  |  |  |  |  |  |  |  |  |  |  |  |  |  |  |  |  |  |  |  |  |  |  |  |  |  |  |  |  |  |  |  |  |  |  |  |  |  |  |  |  |  |  |  |  |  |  |  |  |  |  |  |  |  |  |  |  |  |  |  |  |  |  |  |  |  |  |  |  |  |  |  |  |  |  |  |  |  |  |  |  |  |  |  |  |  |  |  |  |  |  |  |  |  |  |  |  |  |  |  |  |  |  |  |  |  |  |  |  |  |  |  |  |  |  |  |  |  |  |  |  |  |  |  |  |  |  |  |  |  |  |  |  |  |  |  |  |  |  |  |  |  |  |  |  |  |  |  |  |  |  |  |  |  |  |  |  |  |  |  |  |  |  |  |  |  |  |  |  |  |  |  |  |  |  |  |  |  |  |  |  |  |  |  |  |  |  |  |  |  |  |  |  |  |  |  |  |  |  |  |  |  |  |  |  |  |  |  |  |  |  |  |  |  |  |  |  |  |  |  |  |  |  |  |  |  |  |  |  |  |  |  |  |  |  |  |  |  |  |  |  |  |  |  |  |  |  |  |  |  |  |  |  |  |  |  |  |  |  |  |  |  |  |  |  |  |  |  |  |  |  |  |  |  |  |  |  |  |  |  |  |  |  |  |  |  |  |  |  |  |  |  |  |  |  |  |  |  |  |  |  |  |  |  |  |  |  |  |  |  |  |  |  |  |  |  |  |  |  |  |  |  |  |  |  |  |  |  |  |  |  |  |  |  |  |  |  |  |  |  |  |  |  |  |  |  |  |  |  |  |  |  |  |  |  |  |  |  |  |  |  |  |  |  |  |  |  |  |  |  |  |  |  |  |  |  |  |  |  |  |  |  |  |  |  |  |  |  |  |  |  |  |  |  |  |  |  |  |  |  |  |  |  |  |  |  |  |  |  |  |  |  |  |  |  |  |  |  |  |  |  |  |  |  |  |  |  |  |  |  |  |  |  |  |  |  |  |  |  |  |  |  |  |  |  |  |  |  |  |  |  |  |  |  |  |  |  |  |  |  |  |  |  |  |  |  |  |  |  |  |  |  |  |  |  |  |  |  |  |  |  |  |  |  |  |  |  |  |  |  |  |  |  |  |  |  |  |  |  |  |  |  |  |  |  |  |  |  |  |  |  |  |  |  |  |  |  |  |  |  |  |  |  |  |  |  |  |  |  |  |  |  |  |  |  |  |  |  |  |  |  |  |  |  |  |  |  |  |  |  |  |  |  |  |  |  |  |  |  |  |  |  |  |  |  |  |  |  |  |  |  |  |  |  |  |  |  |  |  |  |  |  |  |  |  |  |  |  |  |  |  |  |  |  |  |  |  |  |  |  |  |  |  |  |  |  |  |  |  |  |  |  |  |  |  |  |  |  |  |  |  |  |  |  |  |  |  |  |  |  |  |  |  |  |  |  |  |  |  |  |  |  |  |  |  |  |  |  |  |  |  |  |  |  |  |  |  |  |  |  |  |  |  |  |  |  |  |  |  |  |  |  |  |  |  |  |  |  |  |  |  |  |  |  |  |  |  |  |  |  |  |  |  |  |  |  |  |  |  |  |  |  |  |  |  |  |  |  |  |  |  |  |  |  |  |  |  |  |  |  |  |  |  |  |  |  |  |  |  |  |  |  |  |  |  |  |  |  |  |  |  |  |  |  |  |  |  |  |  |  |  |  |  |  |  |  |  |  |  |  |  |  |  |  |  |  |  |  |  |  |  |  |  |  |  |  |  |  |  |  |  |  |  |  |  |  |  |  |  |  |  |  |  |  |  |  |  |  |  |  |  |  |  |  |  |  |  |  |  |  |  |  |  |  |  |  |  |  |  |  |  |  |  |  |  |  |  |  |  |  |  |  |  |  |  |  |  |  |  |  |  |  |  |  |  |  |  |  |  |  |  |  |  |  |  |  |  |  |  |  |  |  |  |  |  |  |  |  |  |  |  |  |  |  |  |  |  |  |  |  |  |  |  |  |  |  |  |  |  |  |  |  |  |  |  |  |  |  |  |  |  |  |  |  |  |  |  |  |  |  |  |  |  |  |  |  |  |  |  |  |  |  |  |  |  |  |  |  |  |  |  |  |  |  |  |  |  |  |  |  |  |  |  |  |  |  |  |  |  |  |  |  |  |  |  |  |  |  |  |  |  |  |  |  |  |  |  |  |  |  |  |  |  |  |  |  |  |  |  |  |  |  |  |  |  |  |  |  |  |  |  |  |  |  |  |  |  |  |  |  |  |  |  |  |  |  |  |  |  |  |  |  |  |  |  |  |  |  |  |  |  |  |  |  |  |  |  |  |  |  |  |  |  |  |  |  |  |  |  |  |  |  |  |  |  |  |  |  |  |  |  |  |  |  |  |  |  |  |  |  |  |  |  |  |  |  |  |  |  |  |  |  |  |  |  |  |  |  |  |  |  |  |  |  |  |  |  |  |  |  |  |  |  |  |  |  |  |  |  |  |  |  |  |  |  |  |  |  |  |  |  |  |  |  |  |  |  |  |  |  |  |  |  |  |  |  |  |  |  |  |  |  |  |  |  |  |  |  |  |  |  |  |  |  |  |  |  |  |  |  |  |  |  |  |  |  |  |  |  |  |  |  |  |  |  |  |  |  |  |  |  |  |  |  |  |  |  |  |  |  |  |  |  |  |  |  |  |  |  |  |  |  |  |  |  |  |  |  |  |  |  |  |  |  |  |  |  |  |  |  |  |  |  |  |  |  |  |  |  |  |  |  |  |  |  |  |  |  |  |  |  |  |  |  |  |  |  |  |  |  |  |  |  |  |  |  |  |  |  |  |  |  |  |  |  |  |  |  |  |  |  |  |  |  |  |  |  |  |  |  |  |  |  |  |  |  |  |  |  |  |  |  |  |  |  |  |  |  |  |  |  |  |  |  |  |  |  |  |  |  |  |  |  |  |  |  |  |  |  |  |  |  |  |  |  |  |  |  |  |  |  |  |  |  |  |  |  |  |  |  |  |  |  |  |  |  |  |  |  |  |  |  |  |  |  |  |  |  |  |  |  |  |  |  |  |  |  |  |  |  |  |
| --- | --- | --- | --- | --- | --- | --- | --- | --- | --- | --- | --- | --- | --- | --- | --- | --- | --- | --- | --- | --- | --- | --- | --- | --- | --- | --- | --- | --- | --- | --- | --- | --- | --- | --- | --- | --- | --- | --- | --- | --- | --- | --- | --- | --- | --- | --- | --- | --- | --- | --- | --- | --- | --- | --- | --- | --- | --- | --- | --- | --- | --- | --- | --- | --- | --- | --- | --- | --- | --- | --- | --- | --- | --- | --- | --- | --- | --- | --- | --- | --- | --- | --- | --- | --- | --- | --- | --- | --- | --- | --- | --- | --- | --- | --- | --- | --- | --- | --- | --- | --- | --- | --- | --- | --- | --- | --- | --- | --- | --- | --- | --- | --- | --- | --- | --- | --- | --- | --- | --- | --- | --- | --- | --- | --- | --- | --- | --- | --- | --- | --- | --- | --- | --- | --- | --- | --- | --- | --- | --- | --- | --- | --- | --- | --- | --- | --- | --- | --- | --- | --- | --- | --- | --- | --- | --- | --- | --- | --- | --- | --- | --- | --- | --- | --- | --- | --- | --- | --- | --- | --- | --- | --- | --- | --- | --- | --- | --- | --- | --- | --- | --- | --- | --- | --- | --- | --- | --- | --- | --- | --- | --- | --- | --- | --- | --- | --- | --- | --- | --- | --- | --- | --- | --- | --- | --- | --- | --- | --- | --- | --- | --- | --- | --- | --- | --- | --- | --- | --- | --- | --- | --- | --- | --- | --- | --- | --- | --- | --- | --- | --- | --- | --- | --- | --- | --- | --- | --- | --- | --- | --- | --- | --- | --- | --- | --- | --- | --- | --- | --- | --- | --- | --- | --- | --- | --- | --- | --- | --- | --- | --- | --- | --- | --- | --- | --- | --- | --- | --- | --- | --- | --- | --- | --- | --- | --- | --- | --- | --- | --- | --- | --- | --- | --- | --- | --- | --- | --- | --- | --- | --- | --- | --- | --- | --- | --- | --- | --- | --- | --- | --- | --- | --- | --- | --- | --- | --- | --- | --- | --- | --- | --- | --- | --- | --- | --- | --- | --- | --- | --- | --- | --- | --- | --- | --- | --- | --- | --- | --- | --- | --- | --- | --- | --- | --- | --- | --- | --- | --- | --- | --- | --- | --- | --- | --- | --- | --- | --- | --- | --- | --- | --- | --- | --- | --- | --- | --- | --- | --- | --- | --- | --- | --- | --- | --- | --- | --- | --- | --- | --- | --- | --- | --- | --- | --- | --- | --- | --- | --- | --- | --- | --- | --- | --- | --- | --- | --- | --- | --- | --- | --- | --- | --- | --- | --- | --- | --- | --- | --- | --- | --- | --- | --- | --- | --- | --- | --- | --- | --- | --- | --- | --- | --- | --- | --- | --- | --- | --- | --- | --- | --- | --- | --- | --- | --- | --- | --- | --- | --- | --- | --- | --- | --- | --- | --- | --- | --- | --- | --- | --- | --- | --- | --- | --- | --- | --- | --- | --- | --- | --- | --- | --- | --- | --- | --- | --- | --- | --- | --- | --- | --- | --- | --- | --- | --- | --- | --- | --- | --- | --- | --- | --- | --- | --- | --- | --- | --- | --- | --- | --- | --- | --- | --- | --- | --- | --- | --- | --- | --- | --- | --- | --- | --- | --- | --- | --- | --- | --- | --- | --- | --- | --- | --- | --- | --- | --- | --- | --- | --- | --- | --- | --- | --- | --- | --- | --- | --- | --- | --- | --- | --- | --- | --- | --- | --- | --- | --- | --- | --- | --- | --- | --- | --- | --- | --- | --- | --- | --- | --- | --- | --- | --- | --- | --- | --- | --- | --- | --- | --- | --- | --- | --- | --- | --- | --- | --- | --- | --- | --- | --- | --- | --- | --- | --- | --- | --- | --- | --- | --- | --- | --- | --- | --- | --- | --- | --- | --- | --- | --- | --- | --- | --- | --- | --- | --- | --- | --- | --- | --- | --- | --- | --- | --- | --- | --- | --- | --- | --- | --- | --- | --- | --- | --- | --- | --- | --- | --- | --- | --- | --- | --- | --- | --- | --- | --- | --- | --- | --- | --- | --- | --- | --- | --- | --- | --- | --- | --- | --- | --- | --- | --- | --- | --- | --- | --- | --- | --- | --- | --- | --- | --- | --- | --- | --- | --- | --- | --- | --- | --- | --- | --- | --- | --- | --- | --- | --- | --- | --- | --- | --- | --- | --- | --- | --- | --- | --- | --- | --- | --- | --- | --- | --- | --- | --- | --- | --- | --- | --- | --- | --- | --- | --- | --- | --- | --- | --- | --- | --- | --- | --- | --- | --- | --- | --- | --- | --- | --- | --- | --- | --- | --- | --- | --- | --- | --- | --- | --- | --- | --- | --- | --- | --- | --- | --- | --- | --- | --- | --- | --- | --- | --- | --- | --- | --- | --- | --- | --- | --- | --- | --- | --- | --- | --- | --- | --- | --- | --- | --- | --- | --- | --- | --- | --- | --- | --- | --- | --- | --- | --- | --- | --- | --- | --- | --- | --- | --- | --- | --- | --- | --- | --- | --- | --- | --- | --- | --- | --- | --- | --- | --- | --- | --- | --- | --- | --- | --- | --- | --- | --- | --- | --- | --- | --- | --- | --- | --- | --- | --- | --- | --- | --- | --- | --- | --- | --- | --- | --- | --- | --- | --- | --- | --- | --- | --- | --- | --- | --- | --- | --- | --- | --- | --- | --- | --- | --- | --- | --- | --- | --- | --- | --- | --- | --- | --- | --- | --- | --- | --- | --- | --- | --- | --- | --- | --- | --- | --- | --- | --- | --- | --- | --- | --- | --- | --- | --- | --- | --- | --- | --- | --- | --- | --- | --- | --- | --- | --- | --- | --- | --- | --- | --- | --- | --- | --- | --- | --- | --- | --- | --- | --- | --- | --- | --- | --- | --- | --- | --- | --- | --- | --- | --- | --- | --- | --- | --- | --- | --- | --- | --- | --- | --- | --- | --- | --- | --- | --- | --- | --- | --- | --- | --- | --- | --- | --- | --- | --- | --- | --- | --- | --- | --- | --- | --- | --- | --- | --- | --- | --- | --- | --- | --- | --- | --- | --- | --- | --- | --- | --- | --- | --- | --- | --- | --- | --- | --- | --- | --- | --- | --- | --- | --- | --- | --- | --- | --- | --- | --- | --- | --- | --- | --- | --- | --- | --- | --- | --- | --- | --- | --- | --- | --- | --- | --- | --- | --- | --- | --- | --- | --- | --- | --- | --- | --- | --- | --- | --- | --- | --- | --- | --- | --- | --- | --- | --- | --- | --- | --- | --- | --- | --- | --- | --- | --- | --- | --- | --- | --- | --- | --- | --- | --- | --- | --- | --- | --- | --- | --- | --- | --- | --- | --- | --- | --- | --- | --- | --- | --- | --- | --- | --- | --- | --- | --- | --- | --- | --- | --- | --- | --- | --- | --- | --- | --- | --- | --- | --- | --- | --- | --- | --- | --- | --- | --- | --- | --- | --- | --- | --- | --- | --- | --- | --- | --- | --- | --- | --- | --- | --- | --- | --- | --- | --- | --- | --- | --- | --- | --- | --- | --- | --- | --- | --- | --- | --- | --- | --- | --- | --- | --- | --- | --- | --- | --- | --- | --- | --- | --- | --- | --- | --- | --- | --- | --- | --- | --- | --- | --- | --- | --- | --- | --- | --- | --- | --- | --- | --- | --- | --- | --- | --- | --- | --- | --- | --- | --- | --- | --- | --- | --- | --- | --- | --- | --- | --- | --- | --- | --- | --- | --- | --- | --- | --- | --- | --- | --- | --- | --- | --- | --- | --- | --- | --- | --- | --- | --- | --- | --- | --- | --- | --- | --- | --- | --- | --- | --- | --- | --- | --- | --- | --- | --- | --- | --- | --- | --- | --- | --- | --- | --- | --- | --- | --- | --- | --- | --- | --- | --- | --- | --- | --- | --- | --- | --- | --- | --- | --- | --- | --- | --- | --- | --- | --- | --- | --- | --- | --- | --- | --- | --- | --- | --- | --- | --- | --- | --- | --- | --- | --- | --- | --- | --- | --- | --- | --- | --- | --- | --- | --- | --- | --- | --- | --- | --- | --- | --- | --- | --- | --- | --- | --- | --- | --- | --- | --- | --- | --- | --- | --- | --- | --- | --- | --- | --- | --- | --- | --- | --- | --- | --- | --- | --- | --- | --- | --- | --- | --- | --- | --- | --- | --- | --- | --- | --- | --- | --- | --- | --- | --- | --- | --- | --- | --- | --- | --- | --- | --- | --- | --- | --- | --- | --- | --- | --- | --- | --- | --- | --- | --- | --- | --- | --- | --- | --- | --- | --- | --- | --- | --- | --- | --- | --- | --- | --- | --- | --- | --- | --- | --- | --- | --- | --- | --- | --- | --- | --- | --- | --- | --- | --- | --- | --- | --- | --- | --- | --- | --- | --- | --- | --- | --- | --- | --- | --- | --- | --- | --- | --- | --- | --- | --- | --- | --- | --- | --- | --- | --- | --- | --- | --- | --- | --- | --- | --- | --- | --- | --- | --- | --- | --- | --- | --- | --- | --- | --- | --- | --- | --- | --- | --- | --- | --- | --- | --- | --- | --- | --- | --- | --- | --- | --- | --- | --- | --- | --- | --- | --- | --- | --- | --- | --- | --- | --- | --- | --- | --- | --- | --- | --- | --- | --- | --- | --- | --- | --- | --- | --- | --- | --- | --- | --- | --- | --- | --- | --- | --- | --- | --- | --- | --- | --- | --- | --- | --- | --- | --- | --- | --- | --- | --- | --- | --- | --- | --- | --- | --- | --- | --- | --- | --- | --- | --- | --- | --- | --- | --- | --- | --- | --- | --- | --- | --- | --- | --- | --- | --- | --- | --- | --- | --- | --- | --- | --- | --- | --- | --- | --- | --- | --- | --- | --- | --- | --- | --- | --- | --- | --- | --- | --- | --- | --- | --- | --- | --- | --- | --- | --- | --- | --- | --- | --- | --- | --- | --- | --- | --- | --- | --- | --- | --- | --- | --- | --- | --- | --- | --- | --- | --- | --- | --- | --- | --- | --- | --- | --- | --- | --- | --- | --- | --- | --- | --- | --- | --- | --- | --- | --- | --- | --- | --- | --- | --- | --- | --- | --- | --- | --- | --- | --- | --- | --- | --- | --- | --- | --- | --- | --- | --- | --- | --- | --- | --- | --- | --- | --- | --- | --- | --- | --- | --- | --- | --- | --- | --- | --- | --- | --- | --- | --- | --- | --- | --- | --- | --- | --- | --- | --- | --- | --- | --- | --- | --- | --- | --- | --- | --- | --- | --- | --- | --- | --- | --- | --- | --- | --- | --- | --- | --- | --- | --- | --- | --- | --- | --- | --- | --- | --- | --- | --- | --- | --- | --- | --- | --- | --- | --- | --- | --- | --- | --- | --- | --- | --- | --- | --- | --- | --- | --- | --- | --- | --- | --- | --- | --- | --- | --- | --- | --- | --- | --- | --- | --- | --- | --- | --- | --- | --- | --- | --- | --- | --- | --- | --- | --- | --- | --- | --- | --- | --- | --- | --- | --- | --- | --- | --- | --- | --- | --- | --- | --- | --- | --- | --- | --- | --- | --- | --- | --- | --- | --- | --- | --- | --- | --- | --- | --- | --- | --- | --- | --- | --- | --- | --- | --- | --- | --- | --- | --- | --- | --- | --- | --- | --- | --- | --- | --- | --- | --- | --- | --- | --- | --- | --- | --- | --- | --- | --- | --- | --- | --- | --- | --- | --- | --- | --- | --- | --- | --- | --- | --- | --- | --- | --- | --- | --- | --- | --- | --- | --- | --- | --- | --- | --- | --- | --- | --- | --- | --- | --- | --- | --- | --- | --- | --- | --- | --- | --- | --- | --- | --- | --- | --- | --- | --- | --- | --- | --- | --- | --- | --- | --- | --- | --- | --- | --- | --- | --- | --- | --- | --- | --- | --- | --- | --- | --- | --- | --- | --- | --- | --- | --- | --- | --- | --- | --- | --- | --- | --- | --- | --- | --- | --- | --- | --- | --- | --- | --- | --- | --- | --- | --- | --- | --- | --- | --- | --- | --- | --- | --- | --- | --- | --- | --- | --- | --- | --- | --- | --- | --- | --- | --- | --- | --- | --- | --- | --- | --- | --- | --- | --- | --- | --- | --- | --- | --- | --- | --- | --- | --- | --- | --- | --- | --- | --- | --- | --- | --- | --- | --- | --- | --- | --- | --- | --- | --- | --- | --- | --- | --- | --- | --- | --- | --- | --- | --- | --- | --- | --- | --- | --- | --- | --- | --- | --- | --- | --- | --- | --- | --- | --- | --- | --- | --- | --- | --- | --- | --- | --- | --- | --- | --- | --- | --- | --- | --- | --- | --- | --- | --- | --- | --- | --- | --- | --- | --- | --- | --- | --- | --- | --- | --- | --- | --- | --- | --- | --- | --- | --- | --- | --- | --- | --- | --- | --- | --- | --- | --- | --- | --- | --- | --- | --- | --- | --- | --- | --- | --- | --- | --- | --- | --- | --- | --- | --- | --- | --- | --- | --- | --- | --- | --- | --- | --- | --- | --- | --- | --- | --- | --- | --- | --- | --- | --- | --- | --- | --- | --- | --- | --- | --- | --- | --- | --- | --- | --- | --- | --- | --- | --- | --- | --- | --- | --- | --- | --- | --- | --- | --- | --- | --- | --- | --- | --- | --- | --- | --- | --- | --- | --- | --- | --- | --- | --- | --- | --- | --- | --- | --- | --- | --- | --- | --- | --- | --- | --- | --- | --- | --- | --- | --- | --- | --- | --- | --- | --- | --- | --- | --- | --- | --- | --- | --- | --- | --- | --- | --- | --- | --- | --- | --- | --- | --- | --- | --- | --- | --- | --- | --- | --- | --- | --- | --- | --- | --- | --- | --- | --- | --- | --- | --- | --- | --- | --- | --- | --- | --- | --- | --- | --- | --- | --- | --- | --- | --- | --- | --- | --- | --- | --- | --- | --- | --- | --- | --- | --- | --- | --- | --- | --- | --- | --- | --- | --- | --- | --- | --- | --- | --- | --- | --- | --- | --- | --- | --- | --- | --- | --- | --- | --- | --- | --- | --- | --- | --- | --- | --- | --- | --- | --- | --- | --- | --- | --- | --- | --- | --- | --- | --- | --- | --- | --- | --- | --- | --- | --- | --- | --- | --- | --- | --- | --- | --- | --- | --- | --- | --- | --- | --- | --- | --- | --- | --- | --- | --- | --- | --- | --- | --- | --- | --- | --- | --- | --- | --- | --- | --- | --- | --- | --- | --- | --- | --- | --- | --- | --- | --- | --- | --- | --- | --- | --- | --- | --- | --- | --- | --- | --- | --- | --- | --- | --- | --- | --- | --- | --- | --- | --- | --- | --- | --- | --- | --- | --- | --- | --- | --- | --- | --- | --- | --- | --- | --- | --- | --- | --- | --- | --- | --- | --- | --- | --- | --- | --- | --- | --- | --- | --- | --- | --- | --- | --- | --- | --- | --- | --- | --- | --- | --- | --- | --- | --- | --- | --- | --- | --- | --- | --- | --- | --- | --- | --- | --- | --- | --- | --- | --- | --- | --- | --- | --- | --- | --- | --- | --- | --- | --- | --- | --- | --- | --- | --- | --- | --- | --- | --- | --- | --- | --- | --- | --- | --- | --- | --- | --- | --- | --- | --- | --- | --- | --- | --- | --- | --- | --- | --- | --- | --- | --- | --- | --- | --- | --- | --- | --- | --- | --- | --- | --- | --- | --- | --- | --- | --- | --- | --- | --- | --- | --- | --- | --- | --- | --- | --- | --- | --- | --- | --- | --- | --- | --- | --- | --- | --- | --- | --- | --- | --- | --- | --- | --- | --- | --- | --- | --- | --- | --- | --- | --- | --- | --- | --- | --- | --- | --- | --- | --- | --- | --- | --- | --- | --- | --- | --- | --- | --- | --- | --- | --- | --- | --- | --- | --- | --- | --- | --- | --- | --- | --- | --- | --- | --- | --- | --- | --- | --- | --- | --- | --- | --- | --- | --- | --- | --- | --- | --- | --- | --- | --- | --- | --- | --- | --- | --- | --- | --- | --- | --- | --- | --- | --- | --- | --- | --- | --- | --- | --- | --- | --- | --- | --- | --- | --- | --- | --- | --- | --- | --- | --- | --- | --- | --- | --- | --- | --- | --- | --- | --- | --- | --- | --- | --- | --- | --- | --- | --- | --- | --- | --- | --- | --- | --- | --- | --- | --- | --- | --- | --- | --- | --- | --- | --- | --- | --- | --- | --- | --- | --- | --- | --- | --- | --- | --- | --- | --- | --- | --- | --- | --- | --- | --- | --- | --- | --- | --- | --- | --- | --- | --- | --- | --- | --- | --- | --- | --- | --- | --- | --- | --- | --- | --- | --- | --- | --- | --- | --- | --- | --- | --- | --- | --- | --- | --- | --- | --- | --- | --- | --- | --- | --- | --- | --- | --- | --- | --- | --- | --- | --- | --- | --- | --- | --- | --- | --- | --- | --- | --- | --- | --- | --- | --- | --- | --- | --- | --- | --- | --- | --- | --- | --- | --- | --- | --- | --- | --- | --- | --- | --- | --- | --- | --- | --- | --- | --- | --- | --- | --- | --- | --- | --- | --- | --- | --- | --- | --- | --- | --- | --- | --- | --- | --- | --- | --- | --- | --- | --- | --- | --- | --- | --- | --- | --- | --- | --- | --- |
| Actual | Predict  |  |  |  |  |  |  |  |  |  |  |  |  |  |  |  |  |  |  |  |  |  |  |  |  |  |  |  |  |  |  |  |  |  |  |  |  |  |  |  |  |  |  |  |  |  |  |  |  |  |  |  | | --- | --- | --- | --- | --- | --- | --- | --- | --- | --- | --- | --- | --- | --- | --- | --- | --- | --- | --- | --- | --- | --- | --- | --- | --- | --- | --- | --- | --- | --- | --- | --- | --- | --- | --- | --- | --- | --- | --- | --- | --- | --- | --- | --- | --- | --- | --- | --- | --- | --- | --- | |  | 1.1 | 1.10 | 1.11 | 1.13 | 1.14 | 1.15 | 1.16 | 1.17 | 1.18 | 1.2 | 1.21 | 1.3 | 1.4 | 1.5 | 1.6 | 1.7 | 1.8 | 1.9 | 1.97 | 2.1 | 2.2 | 2.3 | 2.4 | 2.5 | 2.6 | 2.7 | 2.8 | 3.1 | 3.2 | 3.3 | 3.4 | 3.5 | 3.6 | 3.8 | 4.1 | 4.2 | 4.3 | 4.6 | 5.1 | 5.2 | 5.3 | 5.4 | 5.6 | 5.99 | 6.1 | 6.3 | 6.4 | 6.5 | 7.1 | 7.6 | | 1.1 | 47 | 0 | 1 | 0 | 0 | 0 | 0 | 0 | 0 | 0 | 0 | 1 | 1 | 0 | 0 | 0 | 1 | 0 | 0 | 0 | 0 | 1 | 0 | 0 | 0 | 2 | 0 | 0 | 0 | 0 | 1 | 1 | 3 | 0 | 3 | 3 | 0 | 0 | 2 | 1 | 1 | 0 | 0 | 0 | 0 | 2 | 0 | 0 | 0 | 0 | | 1.10 | 0 | 24 | 0 | 0 | 0 | 1 | 0 | 0 | 0 | 0 | 0 | 0 | 0 | 0 | 0 | 1 | 0 | 0 | 0 | 0 | 0 | 0 | 0 | 0 | 0 | 1 | 0 | 0 | 0 | 0 | 2 | 1 | 2 | 0 | 0 | 0 | 0 | 0 | 0 | 0 | 0 | 0 | 0 | 0 | 0 | 0 | 0 | 0 | 0 | 0 | | 1.11 | 1 | 0 | 12 | 0 | 0 | 0 | 0 | 0 | 0 | 2 | 0 | 0 | 0 | 1 | 1 | 0 | 0 | 0 | 0 | 0 | 0 | 1 | 0 | 2 | 1 | 13 | 0 | 3 | 15 | 0 | 0 | 2 | 10 | 0 | 7 | 2 | 0 | 0 | 0 | 0 | 0 | 0 | 0 | 0 | 0 | 0 | 0 | 0 | 1 | 0 | | 1.13 | 0 | 0 | 0 | 13 | 0 | 0 | 0 | 0 | 0 | 0 | 0 | 0 | 0 | 0 | 0 | 0 | 0 | 0 | 0 | 0 | 0 | 0 | 1 | 0 | 0 | 0 | 0 | 0 | 0 | 0 | 0 | 0 | 0 | 0 | 0 | 5 | 0 | 0 | 0 | 0 | 0 | 0 | 0 | 0 | 0 | 0 | 0 | 0 | 0 | 0 | | 1.14 | 0 | 2 | 0 | 0 | 56 | 0 | 0 | 0 | 0 | 2 | 0 | 0 | 0 | 0 | 0 | 0 | 0 | 0 | 0 | 0 | 0 | 6 | 0 | 0 | 0 | 14 | 0 | 6 | 0 | 0 | 1 | 1 | 0 | 0 | 0 | 2 | 0 | 0 | 0 | 5 | 1 | 0 | 0 | 0 | 0 | 0 | 0 | 0 | 0 | 0 | | 1.15 | 0 | 0 | 0 | 0 | 0 | 0 | 0 | 0 | 0 | 0 | 0 | 0 | 0 | 0 | 0 | 0 | 0 | 0 | 0 | 0 | 0 | 5 | 0 | 0 | 0 | 0 | 0 | 0 | 0 | 0 | 0 | 6 | 1 | 0 | 0 | 0 | 0 | 0 | 0 | 0 | 0 | 0 | 0 | 0 | 0 | 0 | 0 | 0 | 0 | 0 | | 1.16 | 0 | 0 | 0 | 0 | 0 | 0 | 16 | 0 | 0 | 0 | 0 | 0 | 0 | 0 | 0 | 0 | 0 | 0 | 0 | 0 | 0 | 0 | 0 | 0 | 0 | 0 | 0 | 0 | 0 | 0 | 0 | 0 | 0 | 0 | 0 | 0 | 0 | 0 | 0 | 0 | 0 | 0 | 0 | 0 | 0 | 0 | 0 | 0 | 0 | 0 | | 1.17 | 0 | 0 | 0 | 0 | 0 | 0 | 0 | 12 | 0 | 0 | 0 | 0 | 0 | 0 | 0 | 0 | 0 | 0 | 0 | 0 | 0 | 0 | 0 | 0 | 0 | 8 | 0 | 1 | 0 | 0 | 4 | 1 | 0 | 0 | 0 | 0 | 0 | 0 | 0 | 0 | 0 | 0 | 0 | 0 | 0 | 0 | 0 | 0 | 0 | 1 | | 1.18 | 9 | 0 | 0 | 0 | 0 | 0 | 0 | 0 | 4 | 0 | 0 | 0 | 0 | 0 | 0 | 0 | 1 | 0 | 0 | 0 | 0 | 0 | 8 | 0 | 0 | 4 | 0 | 0 | 0 | 0 | 0 | 2 | 1 | 0 | 0 | 0 | 0 | 0 | 0 | 0 | 0 | 0 | 1 | 0 | 0 | 0 | 0 | 0 | 0 | 0 | | 1.2 | 4 | 0 | 0 | 0 | 0 | 0 | 0 | 0 | 0 | 3 | 0 | 2 | 0 | 0 | 0 | 0 | 0 | 0 | 0 | 0 | 0 | 0 | 0 | 0 | 0 | 3 | 1 | 0 | 0 | 0 | 3 | 0 | 0 | 0 | 0 | 1 | 0 | 0 | 0 | 0 | 1 | 0 | 0 | 0 | 0 | 0 | 0 | 0 | 0 | 0 | | 1.21 | 0 | 0 | 0 | 0 | 0 | 0 | 0 | 0 | 0 | 0 | 6 | 0 | 0 | 0 | 0 | 0 | 0 | 0 | 0 | 0 | 0 | 0 | 0 | 0 | 0 | 1 | 0 | 0 | 0 | 0 | 0 | 0 | 0 | 0 | 0 | 0 | 0 | 0 | 0 | 0 | 0 | 0 | 0 | 0 | 0 | 0 | 0 | 0 | 0 | 0 | | 1.3 | 4 | 0 | 0 | 0 | 0 | 0 | 0 | 0 | 0 | 0 | 0 | 39 | 0 | 0 | 0 | 0 | 0 | 0 | 0 | 0 | 0 | 0 | 0 | 0 | 0 | 0 | 1 | 1 | 0 | 0 | 0 | 0 | 0 | 0 | 0 | 0 | 0 | 0 | 0 | 0 | 1 | 0 | 0 | 0 | 0 | 0 | 0 | 0 | 0 | 0 | | 1.4 | 0 | 0 | 0 | 0 | 0 | 0 | 0 | 0 | 0 | 0 | 0 | 0 | 3 | 0 | 0 | 0 | 0 | 0 | 0 | 0 | 0 | 0 | 0 | 7 | 0 | 2 | 0 | 0 | 0 | 0 | 0 | 0 | 0 | 0 | 0 | 1 | 0 | 0 | 0 | 5 | 0 | 0 | 0 | 0 | 0 | 0 | 0 | 0 | 0 | 0 | | 1.5 | 0 | 0 | 0 | 0 | 0 | 0 | 0 | 0 | 0 | 0 | 0 | 0 | 0 | 25 | 0 | 0 | 0 | 0 | 0 | 14 | 0 | 0 | 0 | 0 | 0 | 0 | 0 | 0 | 0 | 0 | 0 | 0 | 0 | 0 | 0 | 0 | 0 | 0 | 0 | 0 | 0 | 0 | 0 | 0 | 0 | 0 | 0 | 0 | 0 | 0 | | 1.6 | 0 | 0 | 0 | 0 | 0 | 0 | 0 | 0 | 0 | 0 | 0 | 0 | 0 | 0 | 3 | 0 | 0 | 0 | 0 | 0 | 0 | 0 | 0 | 0 | 0 | 0 | 0 | 0 | 0 | 0 | 0 | 0 | 0 | 0 | 0 | 0 | 0 | 0 | 0 | 0 | 0 | 0 | 0 | 0 | 0 | 0 | 0 | 0 | 0 | 0 | | 1.7 | 1 | 0 | 0 | 0 | 0 | 0 | 0 | 0 | 0 | 0 | 0 | 0 | 0 | 0 | 0 | 7 | 0 | 0 | 0 | 0 | 0 | 0 | 0 | 0 | 0 | 11 | 0 | 0 | 0 | 0 | 1 | 0 | 0 | 0 | 0 | 0 | 0 | 0 | 0 | 13 | 0 | 0 | 0 | 0 | 0 | 0 | 0 | 0 | 0 | 0 | | 1.8 | 0 | 0 | 0 | 0 | 0 | 0 | 0 | 0 | 0 | 0 | 0 | 0 | 0 | 0 | 0 | 0 | 0 | 0 | 0 | 0 | 0 | 0 | 0 | 0 | 0 | 2 | 0 | 0 | 0 | 0 | 0 | 0 | 0 | 0 | 0 | 0 | 0 | 0 | 0 | 0 | 0 | 0 | 0 | 0 | 0 | 0 | 0 | 0 | 0 | 0 | | 1.9 | 0 | 0 | 0 | 0 | 0 | 0 | 0 | 0 | 0 | 0 | 0 | 0 | 0 | 0 | 0 | 1 | 0 | 0 | 0 | 0 | 0 | 0 | 0 | 0 | 0 | 0 | 0 | 0 | 0 | 0 | 1 | 0 | 1 | 0 | 0 | 0 | 0 | 0 | 0 | 0 | 0 | 0 | 0 | 0 | 0 | 0 | 0 | 0 | 0 | 0 | | 1.97 | 0 | 0 | 0 | 0 | 0 | 0 | 0 | 0 | 0 | 0 | 0 | 0 | 0 | 0 | 0 | 0 | 0 | 0 | 2 | 0 | 0 | 0 | 0 | 0 | 0 | 0 | 0 | 0 | 0 | 0 | 0 | 0 | 0 | 0 | 0 | 0 | 0 | 0 | 0 | 0 | 0 | 0 | 0 | 0 | 0 | 0 | 0 | 0 | 0 | 0 | | 2.1 | 0 | 0 | 0 | 0 | 0 | 0 | 0 | 0 | 0 | 0 | 0 | 0 | 0 | 1 | 0 | 0 | 0 | 0 | 0 | 42 | 0 | 0 | 0 | 0 | 0 | 20 | 0 | 2 | 1 | 0 | 7 | 0 | 2 | 0 | 1 | 0 | 0 | 0 | 0 | 0 | 0 | 0 | 0 | 0 | 0 | 0 | 0 | 0 | 0 | 0 | | 2.2 | 0 | 0 | 0 | 0 | 0 | 0 | 0 | 0 | 0 | 0 | 0 | 0 | 0 | 0 | 0 | 0 | 0 | 0 | 0 | 0 | 2 | 0 | 0 | 0 | 0 | 0 | 0 | 0 | 0 | 0 | 1 | 1 | 3 | 0 | 0 | 4 | 0 | 0 | 0 | 0 | 0 | 0 | 0 | 0 | 0 | 0 | 0 | 0 | 0 | 0 | | 2.3 | 0 | 0 | 0 | 0 | 0 | 0 | 0 | 1 | 0 | 0 | 0 | 0 | 0 | 0 | 0 | 0 | 0 | 0 | 0 | 1 | 0 | 44 | 2 | 2 | 0 | 73 | 0 | 2 | 4 | 0 | 4 | 0 | 2 | 0 | 0 | 1 | 0 | 0 | 1 | 0 | 0 | 0 | 0 | 0 | 0 | 0 | 0 | 0 | 0 | 3 | | 2.4 | 0 | 2 | 0 | 0 | 0 | 0 | 0 | 0 | 0 | 0 | 0 | 0 | 0 | 0 | 0 | 0 | 0 | 0 | 0 | 0 | 0 | 0 | 28 | 0 | 2 | 31 | 0 | 0 | 5 | 0 | 1 | 2 | 1 | 0 | 0 | 0 | 0 | 0 | 3 | 2 | 0 | 0 | 0 | 0 | 5 | 0 | 0 | 0 | 0 | 0 | | 2.5 | 0 | 0 | 0 | 0 | 0 | 0 | 0 | 0 | 0 | 0 | 0 | 0 | 0 | 0 | 1 | 0 | 0 | 0 | 0 | 2 | 0 | 0 | 1 | 56 | 0 | 11 | 0 | 2 | 0 | 1 | 4 | 2 | 2 | 0 | 0 | 0 | 0 | 0 | 0 | 1 | 0 | 0 | 0 | 0 | 0 | 0 | 0 | 0 | 0 | 0 | | 2.6 | 0 | 0 | 0 | 0 | 0 | 0 | 0 | 0 | 0 | 1 | 0 | 0 | 0 | 0 | 0 | 0 | 0 | 0 | 0 | 1 | 0 | 0 | 0 | 2 | 5 | 0 | 0 | 4 | 5 | 0 | 0 | 3 | 0 | 0 | 0 | 2 | 0 | 0 | 0 | 0 | 0 | 0 | 0 | 0 | 0 | 0 | 0 | 0 | 0 | 0 | | 2.7 | 12 | 0 | 0 | 0 | 3 | 2 | 2 | 1 | 0 | 0 | 0 | 10 | 2 | 2 | 0 | 1 | 0 | 0 | 0 | 5 | 0 | 11 | 7 | 9 | 0 | 820 | 0 | 41 | 12 | 0 | 53 | 17 | 35 | 0 | 0 | 4 | 0 | 0 | 0 | 4 | 10 | 0 | 1 | 0 | 8 | 2 | 0 | 1 | 1 | 0 | | 2.8 | 3 | 0 | 0 | 0 | 0 | 0 | 0 | 0 | 0 | 0 | 0 | 0 | 0 | 0 | 0 | 0 | 0 | 0 | 0 | 3 | 0 | 0 | 0 | 0 | 0 | 9 | 7 | 4 | 1 | 0 | 4 | 0 | 0 | 0 | 1 | 0 | 0 | 0 | 0 | 0 | 0 | 0 | 0 | 0 | 0 | 0 | 0 | 0 | 0 | 0 | | 3.1 | 3 | 0 | 1 | 0 | 0 | 1 | 0 | 0 | 9 | 0 | 0 | 0 | 0 | 0 | 0 | 0 | 0 | 0 | 0 | 4 | 0 | 16 | 0 | 0 | 0 | 55 | 0 | 149 | 8 | 0 | 17 | 2 | 8 | 0 | 1 | 2 | 0 | 0 | 0 | 3 | 1 | 0 | 0 | 8 | 1 | 0 | 0 | 0 | 0 | 0 | | 3.2 | 1 | 0 | 0 | 1 | 1 | 0 | 0 | 1 | 0 | 0 | 0 | 0 | 0 | 0 | 2 | 0 | 0 | 0 | 0 | 1 | 0 | 2 | 2 | 2 | 0 | 21 | 0 | 21 | 280 | 0 | 5 | 10 | 7 | 0 | 1 | 4 | 0 | 2 | 3 | 1 | 2 | 0 | 0 | 0 | 1 | 0 | 2 | 0 | 0 | 0 | | 3.3 | 1 | 0 | 0 | 0 | 0 | 0 | 0 | 0 | 0 | 0 | 0 | 0 | 0 | 0 | 0 | 1 | 0 | 0 | 0 | 0 | 0 | 0 | 0 | 0 | 0 | 0 | 0 | 2 | 0 | 8 | 0 | 0 | 0 | 0 | 0 | 0 | 0 | 0 | 0 | 0 | 0 | 0 | 0 | 0 | 0 | 0 | 0 | 0 | 0 | 0 | | 3.4 | 12 | 0 | 5 | 0 | 2 | 5 | 1 | 0 | 0 | 1 | 0 | 0 | 0 | 0 | 0 | 3 | 0 | 0 | 0 | 6 | 0 | 8 | 1 | 1 | 0 | 120 | 0 | 9 | 8 | 0 | 306 | 5 | 17 | 0 | 0 | 14 | 0 | 0 | 0 | 5 | 0 | 0 | 0 | 2 | 0 | 0 | 4 | 0 | 0 | 0 | | 3.5 | 2 | 7 | 5 | 0 | 0 | 0 | 0 | 0 | 0 | 2 | 0 | 0 | 0 | 0 | 1 | 4 | 0 | 0 | 0 | 3 | 23 | 13 | 8 | 4 | 1 | 30 | 0 | 6 | 91 | 0 | 18 | 41 | 1 | 0 | 0 | 5 | 0 | 0 | 0 | 3 | 0 | 0 | 0 | 0 | 0 | 0 | 0 | 0 | 1 | 0 | | 3.6 | 3 | 3 | 0 | 0 | 0 | 0 | 4 | 0 | 0 | 0 | 0 | 0 | 0 | 4 | 0 | 0 | 2 | 0 | 0 | 0 | 0 | 4 | 3 | 0 | 0 | 89 | 0 | 5 | 20 | 2 | 15 | 3 | 43 | 0 | 0 | 8 | 0 | 0 | 0 | 0 | 1 | 0 | 2 | 7 | 0 | 1 | 0 | 0 | 7 | 0 | | 3.8 | 0 | 0 | 0 | 0 | 0 | 0 | 0 | 0 | 0 | 0 | 0 | 0 | 0 | 0 | 0 | 0 | 0 | 0 | 0 | 1 | 0 | 0 | 1 | 4 | 0 | 0 | 0 | 7 | 0 | 0 | 0 | 6 | 0 | 6 | 0 | 1 | 0 | 0 | 0 | 0 | 2 | 0 | 0 | 0 | 0 | 0 | 0 | 0 | 0 | 0 | | 4.1 | 1 | 0 | 0 | 0 | 0 | 0 | 0 | 0 | 0 | 0 | 0 | 0 | 0 | 0 | 0 | 2 | 0 | 0 | 0 | 0 | 0 | 1 | 0 | 0 | 0 | 2 | 0 | 1 | 3 | 0 | 0 | 2 | 0 | 0 | 18 | 0 | 0 | 0 | 0 | 0 | 0 | 0 | 0 | 0 | 0 | 0 | 0 | 0 | 0 | 0 | | 4.2 | 4 | 0 | 0 | 0 | 0 | 0 | 0 | 0 | 0 | 0 | 0 | 0 | 0 | 0 | 0 | 0 | 0 | 0 | 0 | 1 | 1 | 2 | 2 | 3 | 0 | 29 | 0 | 19 | 1 | 0 | 8 | 2 | 4 | 0 | 3 | 26 | 0 | 0 | 0 | 0 | 2 | 0 | 0 | 0 | 0 | 0 | 0 | 0 | 0 | 0 | | 4.3 | 0 | 0 | 0 | 0 | 0 | 0 | 0 | 0 | 0 | 0 | 0 | 0 | 0 | 0 | 0 | 0 | 0 | 0 | 0 | 0 | 0 | 0 | 0 | 0 | 0 | 0 | 0 | 0 | 0 | 0 | 0 | 0 | 0 | 0 | 0 | 3 | 5 | 0 | 0 | 0 | 0 | 0 | 0 | 0 | 0 | 0 | 0 | 0 | 1 | 0 | | 4.6 | 0 | 0 | 0 | 0 | 0 | 0 | 0 | 0 | 0 | 0 | 0 | 0 | 0 | 0 | 0 | 1 | 0 | 0 | 0 | 0 | 0 | 0 | 0 | 0 | 0 | 4 | 0 | 0 | 1 | 0 | 0 | 0 | 0 | 0 | 0 | 0 | 0 | 7 | 0 | 0 | 0 | 0 | 0 | 0 | 0 | 0 | 0 | 0 | 0 | 0 | | 5.1 | 0 | 0 | 0 | 0 | 0 | 0 | 0 | 0 | 0 | 0 | 0 | 0 | 0 | 0 | 1 | 0 | 0 | 0 | 0 | 0 | 0 | 0 | 0 | 0 | 0 | 0 | 0 | 0 | 0 | 0 | 0 | 0 | 0 | 0 | 0 | 0 | 0 | 0 | 2 | 0 | 0 | 0 | 0 | 0 | 0 | 0 | 0 | 0 | 0 | 0 | | 5.2 | 0 | 0 | 0 | 0 | 0 | 0 | 0 | 0 | 0 | 0 | 0 | 0 | 0 | 0 | 0 | 0 | 0 | 0 | 0 | 0 | 0 | 0 | 0 | 0 | 0 | 1 | 0 | 0 | 0 | 0 | 1 | 0 | 0 | 0 | 0 | 0 | 0 | 0 | 0 | 49 | 0 | 0 | 0 | 0 | 0 | 0 | 0 | 0 | 0 | 0 | | 5.3 | 11 | 0 | 0 | 0 | 0 | 1 | 0 | 0 | 2 | 0 | 0 | 0 | 0 | 3 | 0 | 1 | 0 | 0 | 0 | 0 | 0 | 3 | 0 | 2 | 0 | 20 | 0 | 12 | 4 | 0 | 5 | 17 | 3 | 0 | 0 | 3 | 0 | 0 | 0 | 1 | 11 | 0 | 0 | 0 | 0 | 0 | 0 | 0 | 0 | 0 | | 5.4 | 0 | 0 | 0 | 0 | 2 | 0 | 0 | 0 | 0 | 0 | 0 | 0 | 0 | 0 | 0 | 0 | 0 | 0 | 0 | 0 | 0 | 1 | 0 | 0 | 0 | 3 | 0 | 0 | 0 | 0 | 24 | 0 | 1 | 0 | 0 | 0 | 0 | 0 | 0 | 0 | 0 | 8 | 0 | 0 | 0 | 0 | 0 | 0 | 0 | 0 | | 5.6 | 0 | 0 | 0 | 0 | 0 | 0 | 0 | 0 | 0 | 0 | 0 | 0 | 0 | 0 | 0 | 0 | 0 | 0 | 0 | 0 | 0 | 0 | 0 | 0 | 0 | 0 | 0 | 1 | 1 | 0 | 0 | 0 | 1 | 0 | 0 | 0 | 0 | 0 | 0 | 0 | 0 | 0 | 4 | 0 | 0 | 0 | 0 | 0 | 0 | 0 | | 5.99 | 0 | 0 | 0 | 0 | 0 | 0 | 0 | 0 | 0 | 0 | 0 | 0 | 0 | 0 | 0 | 0 | 0 | 0 | 0 | 0 | 0 | 0 | 0 | 2 | 0 | 4 | 0 | 1 | 0 | 0 | 4 | 0 | 6 | 0 | 0 | 0 | 0 | 0 | 0 | 0 | 0 | 0 | 0 | 7 | 0 | 0 | 0 | 0 | 0 | 0 | | 6.1 | 0 | 0 | 0 | 0 | 0 | 0 | 0 | 0 | 0 | 0 | 0 | 0 | 0 | 0 | 0 | 0 | 0 | 0 | 0 | 0 | 0 | 1 | 0 | 2 | 0 | 9 | 0 | 0 | 2 | 0 | 2 | 0 | 0 | 0 | 0 | 0 | 0 | 0 | 0 | 2 | 0 | 0 | 0 | 0 | 15 | 0 | 0 | 0 | 0 | 0 | | 6.3 | 0 | 0 | 0 | 0 | 0 | 0 | 0 | 0 | 0 | 1 | 0 | 0 | 0 | 0 | 0 | 0 | 0 | 0 | 0 | 3 | 0 | 1 | 0 | 0 | 0 | 2 | 0 | 0 | 0 | 0 | 0 | 0 | 0 | 0 | 0 | 0 | 0 | 0 | 0 | 1 | 0 | 0 | 0 | 0 | 0 | 9 | 0 | 0 | 0 | 0 | | 6.4 | 0 | 0 | 0 | 0 | 0 | 0 | 0 | 0 | 0 | 0 | 0 | 0 | 0 | 0 | 0 | 0 | 0 | 0 | 0 | 0 | 0 | 1 | 0 | 0 | 0 | 3 | 0 | 0 | 0 | 0 | 0 | 0 | 0 | 0 | 0 | 0 | 0 | 0 | 0 | 0 | 0 | 0 | 0 | 0 | 0 | 0 | 0 | 0 | 0 | 0 | | 6.5 | 0 | 0 | 0 | 0 | 0 | 0 | 0 | 0 | 0 | 0 | 0 | 0 | 0 | 0 | 0 | 0 | 0 | 0 | 0 | 0 | 0 | 1 | 0 | 0 | 0 | 4 | 0 | 0 | 1 | 0 | 0 | 0 | 0 | 0 | 0 | 0 | 0 | 0 | 0 | 0 | 0 | 0 | 0 | 0 | 0 | 0 | 0 | 0 | 0 | 0 | | 7.1 | 0 | 0 | 1 | 0 | 0 | 0 | 0 | 0 | 0 | 0 | 0 | 0 | 0 | 0 | 0 | 0 | 0 | 0 | 0 | 0 | 0 | 0 | 0 | 1 | 0 | 2 | 0 | 0 | 2 | 0 | 1 | 0 | 11 | 0 | 0 | 0 | 0 | 0 | 0 | 0 | 1 | 0 | 0 | 0 | 0 | 0 | 0 | 0 | 0 | 0 | | 7.6 | 0 | 0 | 0 | 0 | 0 | 0 | 0 | 0 | 0 | 0 | 0 | 0 | 0 | 0 | 0 | 0 | 0 | 0 | 0 | 0 | 0 | 0 | 0 | 0 | 0 | 0 | 0 | 1 | 0 | 0 | 0 | 0 | 3 | 0 | 0 | 0 | 0 | 0 | 0 | 0 | 0 | 0 | 0 | 0 | 0 | 0 | 0 | 0 | 0 | 0 | |

## Overall Statistics :

|  |  |
| --- | --- |
| 95% CI | (0.51689,0.54684) |
| ACC Macro | 0.98127 |
| ARI | 0.33107 |
| AUNP | 0.73287 |
| AUNU | 0.70829 |
| Bangdiwala B | 0.40289 |
| Bennett S | 0.52231 |
| CBA | 0.3636 |
| CSI | None |
| Chi-Squared | None |
| Chi-Squared DF | 2401 |
| Conditional Entropy | 2.1192 |
| Cramer V | None |
| Cross Entropy | 4.27353 |
| F1 Macro | 0.4218 |
| F1 Micro | 0.53187 |
| FNR Macro | 0.57273 |
| FNR Micro | 0.46813 |
| FPR Macro | 0.01069 |
| FPR Micro | 0.00955 |
| Gwet AC1 | 0.52334 |
| Hamming Loss | 0.46813 |
| Joint Entropy | 6.27843 |
| KL Divergence | None |
| Kappa | 0.46718 |
| Kappa 95% CI | (0.45015,0.48422) |
| Kappa No Prevalence | 0.06373 |
| Kappa Standard Error | 0.00869 |
| Kappa Unbiased | 0.46576 |
| Krippendorff Alpha | 0.46582 |
| Lambda A | 0.38972 |
| Lambda B | 0.37153 |
| Mutual Information | 1.66705 |
| NIR | 0.25211 |
| Overall ACC | 0.53187 |
| Overall CEN | 0.35013 |
| Overall J | (15.31273,0.30625) |
| Overall MCC | 0.46985 |
| Overall MCEN | 0.43924 |
| Overall RACC | 0.12139 |
| Overall RACCU | 0.12374 |
| P-Value | None |
| PPV Macro | None |
| PPV Micro | 0.53187 |
| Pearson C | None |
| Phi-Squared | None |
| RCI | 0.40081 |
| RR | 85.36 |
| Reference Entropy | 4.15923 |
| Response Entropy | 3.78626 |
| SOA1(Landis & Koch) | Moderate |
| SOA2(Fleiss) | Intermediate to Good |
| SOA3(Altman) | Moderate |
| SOA4(Cicchetti) | Fair |
| SOA5(Cramer) | None |
| SOA6(Matthews) | Weak |
| Scott PI | 0.46576 |
| Standard Error | 0.00764 |
| TNR Macro | 0.98931 |
| TNR Micro | 0.99045 |
| TPR Macro | 0.42727 |
| TPR Micro | 0.53187 |
| Zero-one Loss | 1998 |

## Class Statistics :

|  |  |  |  |  |  |  |  |  |  |  |  |  |  |  |  |  |  |  |  |  |  |  |  |  |  |  |  |  |  |  |  |  |  |  |  |  |  |  |  |  |  |  |  |  |  |  |  |  |  |  |  |
| --- | --- | --- | --- | --- | --- | --- | --- | --- | --- | --- | --- | --- | --- | --- | --- | --- | --- | --- | --- | --- | --- | --- | --- | --- | --- | --- | --- | --- | --- | --- | --- | --- | --- | --- | --- | --- | --- | --- | --- | --- | --- | --- | --- | --- | --- | --- | --- | --- | --- | --- | --- |
| Class | 1.1 | 1.10 | 1.11 | 1.13 | 1.14 | 1.15 | 1.16 | 1.17 | 1.18 | 1.2 | 1.21 | 1.3 | 1.4 | 1.5 | 1.6 | 1.7 | 1.8 | 1.9 | 1.97 | 2.1 | 2.2 | 2.3 | 2.4 | 2.5 | 2.6 | 2.7 | 2.8 | 3.1 | 3.2 | 3.3 | 3.4 | 3.5 | 3.6 | 3.8 | 4.1 | 4.2 | 4.3 | 4.6 | 5.1 | 5.2 | 5.3 | 5.4 | 5.6 | 5.99 | 6.1 | 6.3 | 6.4 | 6.5 | 7.1 | 7.6 | Description |
| ACC | 0.97751 | 0.99485 | 0.98243 | 0.99836 | 0.98875 | 0.99485 | 0.99836 | 0.99578 | 0.99133 | 0.99438 | 0.99977 | 0.99531 | 0.99578 | 0.99414 | 0.99859 | 0.99039 | 0.99859 | 0.9993 | 1.0 | 0.98149 | 0.99227 | 0.95923 | 0.97891 | 0.9836 | 0.99485 | 0.79873 | 0.99367 | 0.93182 | 0.93486 | 0.99836 | 0.90253 | 0.92643 | 0.92784 | 0.99485 | 0.99321 | 0.96579 | 0.99906 | 0.99813 | 0.99766 | 0.98852 | 0.97399 | 0.99274 | 0.99836 | 0.99203 | 0.99227 | 0.99695 | 0.99766 | 0.99836 | 0.99297 | 0.99813 | Accuracy |
| AGF | 0.76054 | 0.84931 | 0.42966 | 0.84934 | 0.7874 | 0.0 | 0.95877 | 0.69739 | 0.38386 | 0.42189 | 0.93925 | 0.90811 | 0.43788 | 0.80559 | 0.84504 | 0.47539 | 0.0 | 0.0 | 1.0 | 0.72968 | 0.37743 | 0.56159 | 0.59402 | 0.80309 | 0.49663 | 0.79748 | 0.50423 | 0.70239 | 0.83338 | 0.82302 | 0.73963 | 0.40266 | 0.43835 | 0.50318 | 0.76083 | 0.4958 | 0.78058 | 0.75701 | 0.65918 | 0.90251 | 0.35445 | 0.49242 | 0.74508 | 0.53898 | 0.67902 | 0.74015 | 0.0 | 0.0 | 0.0 | 0.0 | Adjusted F-score |
| AGM | 0.89399 | 0.9304 | 0.69688 | 0.91323 | 0.87922 | 0 | 0.99877 | 0.83233 | 0.67992 | 0.70223 | 0.96288 | 0.95793 | 0.70307 | 0.89804 | 0.99894 | 0.72706 | 0 | 0 | 1.0 | 0.86321 | 0.70941 | 0.76465 | 0.78463 | 0.9026 | 0.73183 | 0.79685 | 0.73256 | 0.82865 | 0.8966 | 0.90762 | 0.83639 | 0.6727 | 0.6919 | 0.73057 | 0.88412 | 0.73358 | 0.87254 | 0.86638 | 0.90673 | 0.98174 | 0.65956 | 0.7252 | 0.87722 | 0.76684 | 0.8341 | 0.86273 | 0 | 0 | 0 | 0 | Adjusted geometric mean |
| AM | 48 | 6 | -49 | -5 | -32 | -2 | 7 | -12 | -15 | -6 | -1 | 6 | -12 | -3 | 6 | -11 | 2 | -3 | 0 | 11 | 15 | -18 | -18 | 16 | -14 | 347 | -23 | 11 | 92 | -1 | -42 | -142 | -58 | -22 | 5 | -16 | -4 | -4 | 8 | 45 | -65 | -31 | 1 | 0 | -3 | -3 | 2 | -5 | -8 | 0 | Difference between automatic and manual classification |
| AUC | 0.82241 | 0.87335 | 0.57953 | 0.84199 | 0.79071 | 0.49883 | 0.99918 | 0.72187 | 0.56537 | 0.58227 | 0.92857 | 0.92237 | 0.58298 | 0.81921 | 0.9993 | 0.60429 | 0.49953 | 0.5 | 1.0 | 0.77095 | 0.58809 | 0.6477 | 0.66643 | 0.83221 | 0.60822 | 0.78659 | 0.60914 | 0.73881 | 0.85159 | 0.83298 | 0.76093 | 0.56546 | 0.57967 | 0.60714 | 0.79799 | 0.61368 | 0.77778 | 0.769 | 0.83228 | 0.97482 | 0.5528 | 0.60256 | 0.78524 | 0.64383 | 0.7255 | 0.76412 | 0.4993 | 0.49988 | 0.49871 | 0.49953 | Area under the ROC curve |
| AUCI | Very Good | Very Good | Poor | Very Good | Good | Poor | Excellent | Good | Poor | Poor | Excellent | Excellent | Poor | Very Good | Excellent | Fair | Poor | Poor | Excellent | Good | Poor | Fair | Fair | Very Good | Fair | Good | Fair | Good | Very Good | Very Good | Good | Poor | Poor | Fair | Good | Fair | Good | Good | Very Good | Excellent | Poor | Fair | Good | Fair | Good | Good | Poor | Poor | Poor | Poor | AUC value interpretation |
| AUPR | 0.52846 | 0.69079 | 0.32108 | 0.80639 | 0.72917 | 0.0 | 0.84783 | 0.62222 | 0.2 | 0.20833 | 0.92857 | 0.79891 | 0.33333 | 0.66774 | 0.66667 | 0.26515 | 0.0 | None | 1.0 | 0.5177 | 0.12937 | 0.33747 | 0.38948 | 0.62018 | 0.38647 | 0.66916 | 0.49826 | 0.50612 | 0.67641 | 0.69697 | 0.59633 | 0.23763 | 0.22311 | 0.60714 | 0.55714 | 0.26435 | 0.77778 | 0.65812 | 0.42424 | 0.7356 | 0.21732 | 0.60256 | 0.53571 | 0.29167 | 0.47727 | 0.58613 | 0.0 | 0.0 | 0.0 | 0.0 | Area under the PR curve |
| BB | 0.39496 | 0.63158 | 0.16216 | 0.68421 | 0.58333 | 0.0 | 0.69565 | 0.44444 | 0.13333 | 0.16667 | 0.85714 | 0.75 | 0.16667 | 0.64103 | 0.33333 | 0.21212 | 0.0 | 0.0 | 1.0 | 0.48276 | 0.07692 | 0.31429 | 0.34146 | 0.56566 | 0.21739 | 0.57625 | 0.21875 | 0.49667 | 0.60215 | 0.66667 | 0.57196 | 0.15242 | 0.19027 | 0.21429 | 0.51429 | 0.24299 | 0.55556 | 0.53846 | 0.18182 | 0.51042 | 0.11111 | 0.20513 | 0.5 | 0.29167 | 0.45455 | 0.52941 | 0.0 | 0.0 | 0.0 | 0.0 | Braun-Blanquet similarity |
| BCD | 0.00562 | 0.0007 | 0.00574 | 0.00059 | 0.00375 | 0.00023 | 0.00082 | 0.00141 | 0.00176 | 0.0007 | 0.00012 | 0.0007 | 0.00141 | 0.00035 | 0.0007 | 0.00129 | 0.00023 | 0.00035 | 0.0 | 0.00129 | 0.00176 | 0.00211 | 0.00211 | 0.00187 | 0.00164 | 0.04065 | 0.00269 | 0.00129 | 0.01078 | 0.00012 | 0.00492 | 0.01664 | 0.00679 | 0.00258 | 0.00059 | 0.00187 | 0.00047 | 0.00047 | 0.00094 | 0.00527 | 0.00761 | 0.00363 | 0.00012 | 0.0 | 0.00035 | 0.00035 | 0.00023 | 0.00059 | 0.00094 | 0.0 | Bray-Curtis dissimilarity |
| BM | 0.64482 | 0.74669 | 0.15906 | 0.68398 | 0.58142 | -0.00235 | 0.99835 | 0.44374 | 0.13074 | 0.16455 | 0.85714 | 0.84475 | 0.16596 | 0.63842 | 0.99859 | 0.20858 | -0.00094 | 0.0 | 1.0 | 0.5419 | 0.17618 | 0.29539 | 0.33286 | 0.66442 | 0.21645 | 0.57317 | 0.21828 | 0.47762 | 0.70317 | 0.66596 | 0.52187 | 0.13091 | 0.15934 | 0.21429 | 0.59599 | 0.22737 | 0.55556 | 0.53799 | 0.66456 | 0.94964 | 0.10559 | 0.20513 | 0.57049 | 0.28766 | 0.451 | 0.52824 | -0.00141 | -0.00023 | -0.00259 | -0.00094 | Informedness or bookmaker informedness |
| CEN | 0.40862 | 0.22123 | 0.46063 | 0.10858 | 0.22749 | 0.39429 | 0.10465 | 0.23419 | 0.37002 | 0.4649 | 0.04303 | 0.13741 | 0.29775 | 0.1767 | 0.24579 | 0.37416 | 0.29 | 0.23961 | 0 | 0.33924 | 0.22519 | 0.40343 | 0.39257 | 0.32275 | 0.37622 | 0.32787 | 0.33412 | 0.39761 | 0.2792 | 0.18184 | 0.33531 | 0.53171 | 0.53557 | 0.29686 | 0.31336 | 0.5253 | 0.11311 | 0.17643 | 0.28685 | 0.2554 | 0.52775 | 0.17819 | 0.25547 | 0.34874 | 0.30022 | 0.26163 | 0.27914 | 0.25164 | 0.43087 | 0.27383 | Confusion entropy |
| DOR | 112.19618 | 904.71429 | 62.24814 | 9204.0 | 728.7 | 0.0 | None | 1130.13333 | 59.11888 | 94.24444 | None | 1803.85714 | 283.13333 | 684.74026 | None | 75.74359 | 0.0 | None | None | 113.83922 | 39.19444 | 23.79808 | 59.77366 | 199.78639 | 294.51389 | 13.75272 | 592.76 | 26.9807 | 60.3778 | 2835.33333 | 25.33863 | 8.18202 | 7.3631 | None | 372.44118 | 20.22716 | None | 2480.91667 | 945.77778 | 2173.7234 | 22.53261 | None | 1419.0 | 102.38408 | 234.44444 | 955.35 | 0.0 | 0.0 | 0.0 | 0.0 | Diagnostic odds ratio |
| DP | 1.13021 | 1.63001 | 0.98915 | 2.18545 | 1.57821 | None | None | 1.68328 | 0.9768 | 1.08846 | None | 1.79524 | 1.35185 | 1.56331 | None | 1.03614 | None | None | None | 1.13369 | 0.87839 | 0.75893 | 0.97944 | 1.26837 | 1.36129 | 0.62763 | 1.52877 | 0.78898 | 0.98185 | 1.90352 | 0.77395 | 0.50329 | 0.47804 | None | 1.4175 | 0.72 | None | 1.87155 | 1.64064 | 1.8399 | 0.74584 | None | 1.73778 | 1.1083 | 1.30667 | 1.64305 | None | None | None | None | Discriminant power |
| DPI | Limited | Limited | Poor | Fair | Limited | None | None | Limited | Poor | Limited | None | Limited | Limited | Limited | None | Limited | None | None | None | Limited | Poor | Poor | Poor | Limited | Limited | Poor | Limited | Poor | Poor | Limited | Poor | Poor | Poor | None | Limited | Poor | None | Limited | Limited | Limited | Poor | None | Limited | Limited | Limited | Limited | None | None | None | None | Discriminant power interpretation |
| ERR | 0.02249 | 0.00515 | 0.01757 | 0.00164 | 0.01125 | 0.00515 | 0.00164 | 0.00422 | 0.00867 | 0.00562 | 0.00023 | 0.00469 | 0.00422 | 0.00586 | 0.00141 | 0.00961 | 0.00141 | 0.0007 | 0.0 | 0.01851 | 0.00773 | 0.04077 | 0.02109 | 0.0164 | 0.00515 | 0.20127 | 0.00633 | 0.06818 | 0.06514 | 0.00164 | 0.09747 | 0.07357 | 0.07216 | 0.00515 | 0.00679 | 0.03421 | 0.00094 | 0.00187 | 0.00234 | 0.01148 | 0.02601 | 0.00726 | 0.00164 | 0.00797 | 0.00773 | 0.00305 | 0.00234 | 0.00164 | 0.00703 | 0.00187 | Error rate |
| F0.5 | 0.42962 | 0.65217 | 0.34483 | 0.86667 | 0.79545 | 0.0 | 0.74074 | 0.68966 | 0.22222 | 0.22727 | 0.96774 | 0.76772 | 0.35714 | 0.68306 | 0.38462 | 0.28926 | 0.0 | 0.0 | 1.0 | 0.49528 | 0.08696 | 0.35032 | 0.4142 | 0.58455 | 0.42373 | 0.60579 | 0.51471 | 0.50034 | 0.62696 | 0.71429 | 0.61029 | 0.26384 | 0.23942 | 0.57692 | 0.52941 | 0.27601 | 0.86207 | 0.71429 | 0.21277 | 0.56322 | 0.23404 | 0.56338 | 0.51282 | 0.29167 | 0.4902 | 0.61644 | 0.0 | 0.0 | 0.0 | 0.0 | F0.5 score |
| F1 | 0.49474 | 0.68571 | 0.24242 | 0.78788 | 0.7 | 0.0 | 0.82051 | 0.57143 | 0.17778 | 0.2 | 0.92308 | 0.79592 | 0.25 | 0.66667 | 0.5 | 0.25455 | 0.0 | 0.0 | 1.0 | 0.51534 | 0.10811 | 0.33588 | 0.38356 | 0.61538 | 0.3125 | 0.65626 | 0.34146 | 0.50594 | 0.66826 | 0.69565 | 0.59533 | 0.20707 | 0.21827 | 0.35294 | 0.55385 | 0.26263 | 0.71429 | 0.63636 | 0.28571 | 0.66667 | 0.16541 | 0.34043 | 0.53333 | 0.29167 | 0.47619 | 0.58065 | 0.0 | 0.0 | 0.0 | 0.0 | F1 score - harmonic mean of precision and sensitivity |
| F2 | 0.58313 | 0.72289 | 0.18692 | 0.72222 | 0.625 | 0.0 | 0.91954 | 0.4878 | 0.14815 | 0.17857 | 0.88235 | 0.82627 | 0.19231 | 0.65104 | 0.71429 | 0.22727 | 0.0 | 0.0 | 1.0 | 0.53708 | 0.14286 | 0.32258 | 0.35714 | 0.64965 | 0.24752 | 0.71591 | 0.25547 | 0.51168 | 0.71538 | 0.67797 | 0.58109 | 0.17041 | 0.20056 | 0.25424 | 0.58065 | 0.25048 | 0.60976 | 0.57377 | 0.43478 | 0.81667 | 0.12791 | 0.2439 | 0.55556 | 0.29167 | 0.46296 | 0.54878 | 0.0 | 0.0 | 0.0 | 0.0 | F2 score |
| FDR | 0.60504 | 0.36842 | 0.52 | 0.07143 | 0.125 | 1.0 | 0.30435 | 0.2 | 0.73333 | 0.75 | 0.0 | 0.25 | 0.5 | 0.30556 | 0.66667 | 0.68182 | 1.0 | None | 0.0 | 0.51724 | 0.92308 | 0.63934 | 0.5625 | 0.43434 | 0.44444 | 0.42375 | 0.22222 | 0.50333 | 0.39785 | 0.27273 | 0.37931 | 0.67717 | 0.74405 | 0.0 | 0.48571 | 0.71429 | 0.0 | 0.22222 | 0.81818 | 0.48958 | 0.67647 | 0.0 | 0.5 | 0.70833 | 0.5 | 0.35714 | 1.0 | 1.0 | 1.0 | 1.0 | False discovery rate |
| FN | 24 | 8 | 62 | 6 | 40 | 12 | 0 | 15 | 26 | 15 | 1 | 7 | 15 | 14 | 0 | 26 | 2 | 3 | 0 | 34 | 9 | 96 | 54 | 27 | 18 | 256 | 25 | 140 | 93 | 4 | 229 | 228 | 183 | 22 | 12 | 81 | 4 | 6 | 1 | 2 | 88 | 31 | 3 | 17 | 18 | 8 | 4 | 6 | 19 | 4 | False negative/miss/type 2 error |
| FNR | 0.33803 | 0.25 | 0.83784 | 0.31579 | 0.41667 | 1.0 | 0.0 | 0.55556 | 0.86667 | 0.83333 | 0.14286 | 0.15217 | 0.83333 | 0.35897 | 0.0 | 0.78788 | 1.0 | 1.0 | 0.0 | 0.44737 | 0.81818 | 0.68571 | 0.65854 | 0.3253 | 0.78261 | 0.23792 | 0.78125 | 0.48443 | 0.24933 | 0.33333 | 0.42804 | 0.84758 | 0.80973 | 0.78571 | 0.4 | 0.75701 | 0.44444 | 0.46154 | 0.33333 | 0.03922 | 0.88889 | 0.79487 | 0.42857 | 0.70833 | 0.54545 | 0.47059 | 1.0 | 1.0 | 1.0 | 1.0 | Miss rate or false negative rate |
| FOR | 0.00578 | 0.00189 | 0.01461 | 0.00141 | 0.00951 | 0.00282 | 0.0 | 0.00353 | 0.00611 | 0.00352 | 0.00023 | 0.00166 | 0.00352 | 0.00331 | 0.0 | 0.00612 | 0.00047 | 0.0007 | 0.0 | 0.00813 | 0.00212 | 0.02315 | 0.01284 | 0.00648 | 0.00423 | 0.08998 | 0.00587 | 0.03528 | 0.02445 | 0.00094 | 0.06066 | 0.05506 | 0.04463 | 0.00516 | 0.00283 | 0.01939 | 0.00094 | 0.00141 | 0.00023 | 0.00048 | 0.02078 | 0.00728 | 0.0007 | 0.00401 | 0.00425 | 0.00188 | 0.00094 | 0.00141 | 0.00446 | 0.00094 | False omission rate |
| FP | 72 | 14 | 13 | 1 | 8 | 10 | 7 | 3 | 11 | 9 | 0 | 13 | 3 | 11 | 6 | 15 | 4 | 0 | 0 | 45 | 24 | 78 | 36 | 43 | 4 | 603 | 2 | 151 | 185 | 3 | 187 | 86 | 125 | 0 | 17 | 65 | 0 | 2 | 9 | 47 | 23 | 0 | 4 | 17 | 15 | 5 | 6 | 1 | 11 | 4 | False positive/type 1 error/false alarm |
| FPR | 0.01716 | 0.00331 | 0.0031 | 0.00024 | 0.00192 | 0.00235 | 0.00165 | 0.00071 | 0.0026 | 0.00212 | 0.0 | 0.00308 | 0.00071 | 0.0026 | 0.00141 | 0.00354 | 0.00094 | 0.0 | 0.0 | 0.01073 | 0.00564 | 0.0189 | 0.0086 | 0.01027 | 0.00094 | 0.18891 | 0.00047 | 0.03795 | 0.0475 | 0.0007 | 0.05009 | 0.02151 | 0.03093 | 0.0 | 0.00401 | 0.01562 | 0.0 | 0.00047 | 0.00211 | 0.01115 | 0.00552 | 0.0 | 0.00094 | 0.00401 | 0.00354 | 0.00118 | 0.00141 | 0.00023 | 0.00259 | 0.00094 | Fall-out or false positive rate |
| G | 0.51132 | 0.68825 | 0.27899 | 0.79708 | 0.71443 | 0.0 | 0.83406 | 0.59628 | 0.18856 | 0.20412 | 0.92582 | 0.79741 | 0.28868 | 0.6672 | 0.57735 | 0.25979 | 0.0 | None | 1.0 | 0.51651 | 0.11826 | 0.33667 | 0.38651 | 0.61778 | 0.34752 | 0.66268 | 0.41248 | 0.50603 | 0.67232 | 0.69631 | 0.59583 | 0.22182 | 0.22068 | 0.46291 | 0.55549 | 0.26349 | 0.74536 | 0.64715 | 0.34816 | 0.70029 | 0.1896 | 0.45291 | 0.53452 | 0.29167 | 0.47673 | 0.58338 | 0.0 | 0.0 | 0.0 | 0.0 | G-measure geometric mean of precision and sensitivity |
| GI | 0.64482 | 0.74669 | 0.15906 | 0.68398 | 0.58142 | -0.00235 | 0.99835 | 0.44374 | 0.13074 | 0.16455 | 0.85714 | 0.84475 | 0.16596 | 0.63842 | 0.99859 | 0.20858 | -0.00094 | 0.0 | 1.0 | 0.5419 | 0.17618 | 0.29539 | 0.33286 | 0.66442 | 0.21645 | 0.57317 | 0.21828 | 0.47762 | 0.70317 | 0.66596 | 0.52187 | 0.13091 | 0.15934 | 0.21429 | 0.59599 | 0.22737 | 0.55556 | 0.53799 | 0.66456 | 0.94964 | 0.10559 | 0.20513 | 0.57049 | 0.28766 | 0.451 | 0.52824 | -0.00141 | -0.00023 | -0.00259 | -0.00094 | Gini index |
| GM | 0.80661 | 0.86459 | 0.40207 | 0.82707 | 0.76303 | 0.0 | 0.99918 | 0.66643 | 0.36467 | 0.40782 | 0.92582 | 0.91936 | 0.4081 | 0.7996 | 0.9993 | 0.45975 | 0.0 | 0.0 | 1.0 | 0.73939 | 0.4252 | 0.55529 | 0.58183 | 0.81717 | 0.46603 | 0.7862 | 0.4676 | 0.70428 | 0.84559 | 0.81621 | 0.7371 | 0.38618 | 0.4294 | 0.46291 | 0.77304 | 0.48908 | 0.74536 | 0.73363 | 0.81563 | 0.97472 | 0.33241 | 0.45291 | 0.75557 | 0.53898 | 0.673 | 0.72718 | 0.0 | 0.0 | 0.0 | 0.0 | G-mean geometric mean of specificity and sensitivity |
| HD | 96 | 22 | 75 | 7 | 48 | 22 | 7 | 18 | 37 | 24 | 1 | 20 | 18 | 25 | 6 | 41 | 6 | 3 | 0 | 79 | 33 | 174 | 90 | 70 | 22 | 859 | 27 | 291 | 278 | 7 | 416 | 314 | 308 | 22 | 29 | 146 | 4 | 8 | 10 | 49 | 111 | 31 | 7 | 34 | 33 | 13 | 10 | 7 | 30 | 8 | Hamming distance |
| IBA | 0.44185 | 0.56311 | 0.02672 | 0.46819 | 0.34074 | 0.0 | 1.0 | 0.19771 | 0.01808 | 0.02807 | 0.73469 | 0.7192 | 0.02788 | 0.41151 | 1.0 | 0.04558 | 0.0 | 0.0 | 1.0 | 0.30799 | 0.03389 | 0.10274 | 0.11851 | 0.4574 | 0.04742 | 0.58782 | 0.04793 | 0.27455 | 0.5707 | 0.4446 | 0.33797 | 0.02594 | 0.04078 | 0.04592 | 0.36095 | 0.06186 | 0.30864 | 0.29006 | 0.44491 | 0.92341 | 0.01289 | 0.04208 | 0.32676 | 0.08589 | 0.20748 | 0.28057 | 0.0 | 0.0 | 0.0 | 0.0 | Index of balanced accuracy |
| ICSI | 0.05693 | 0.38158 | -0.35784 | 0.61278 | 0.45833 | -1.0 | 0.69565 | 0.24444 | -0.6 | -0.58333 | 0.85714 | 0.59783 | -0.33333 | 0.33547 | 0.33333 | -0.4697 | -1.0 | None | 1.0 | 0.03539 | -0.74126 | -0.32506 | -0.22104 | 0.24036 | -0.22705 | 0.33833 | -0.00347 | 0.01224 | 0.35282 | 0.39394 | 0.19265 | -0.52475 | -0.55378 | 0.21429 | 0.11429 | -0.4713 | 0.55556 | 0.31624 | -0.15152 | 0.4712 | -0.56536 | 0.20513 | 0.07143 | -0.41667 | -0.04545 | 0.17227 | -1.0 | -1.0 | -1.0 | -1.0 | Individual classification success index |
| IS | 4.56937 | 6.39638 | 4.791 | 7.7045 | 5.28174 | None | 7.53578 | 6.98253 | 5.24556 | 5.88942 | 9.25199 | 6.12075 | 6.88942 | 6.24787 | 8.88942 | 5.36287 | None | None | 11.05934 | 4.76079 | 4.89947 | 3.45876 | 4.50915 | 4.8623 | 6.68779 | 1.19264 | 6.69677 | 2.87477 | 2.78451 | 8.01495 | 2.30789 | 2.35675 | 2.27311 | 7.25199 | 6.1931 | 3.51052 | 8.88942 | 7.99633 | 8.01495 | 5.41667 | 3.80196 | 6.77394 | 8.25199 | 5.69677 | 6.01495 | 7.33445 | None | None | None | None | Information score |
| J | 0.32867 | 0.52174 | 0.13793 | 0.65 | 0.53846 | 0.0 | 0.69565 | 0.4 | 0.09756 | 0.11111 | 0.85714 | 0.66102 | 0.14286 | 0.5 | 0.33333 | 0.14583 | 0.0 | 0.0 | 1.0 | 0.34711 | 0.05714 | 0.20183 | 0.23729 | 0.44444 | 0.18519 | 0.48839 | 0.20588 | 0.33864 | 0.50179 | 0.53333 | 0.42382 | 0.11549 | 0.12251 | 0.21429 | 0.38298 | 0.15116 | 0.55556 | 0.46667 | 0.16667 | 0.5 | 0.09016 | 0.20513 | 0.36364 | 0.17073 | 0.3125 | 0.40909 | 0.0 | 0.0 | 0.0 | 0.0 | Jaccard index |
| LS | 23.74198 | 84.23684 | 27.68432 | 208.58647 | 38.90104 | 0.0 | 185.56522 | 126.45926 | 37.93778 | 59.27778 | 609.71429 | 69.58696 | 118.55556 | 75.99715 | 474.22222 | 41.15152 | 0.0 | None | 2134.0 | 27.11071 | 29.84615 | 10.99485 | 22.77134 | 29.08701 | 103.09179 | 2.28571 | 103.73611 | 7.33486 | 6.89002 | 258.66667 | 4.9516 | 5.12215 | 4.83365 | 152.42857 | 73.16571 | 11.39653 | 474.22222 | 255.35043 | 258.66667 | 42.71487 | 13.94771 | 109.4359 | 304.85714 | 51.86806 | 64.66667 | 161.39496 | 0.0 | 0.0 | 0.0 | 0.0 | Lift score |
| MCC | 0.50094 | 0.6857 | 0.27208 | 0.79634 | 0.70937 | -0.00257 | 0.83337 | 0.5945 | 0.18456 | 0.20139 | 0.92571 | 0.79508 | 0.28705 | 0.66426 | 0.57694 | 0.25513 | -0.00066 | None | 1.0 | 0.50715 | 0.1148 | 0.31574 | 0.37597 | 0.60953 | 0.34545 | 0.52793 | 0.41048 | 0.46943 | 0.63735 | 0.69549 | 0.54061 | 0.18723 | 0.1835 | 0.46171 | 0.5521 | 0.24608 | 0.74501 | 0.64628 | 0.34738 | 0.69589 | 0.1788 | 0.45126 | 0.53371 | 0.28766 | 0.47285 | 0.58188 | -0.00115 | -0.00057 | -0.0034 | -0.00094 | Matthews correlation coefficient |
| MCCI | Moderate | Moderate | Negligible | Strong | Strong | Negligible | Strong | Moderate | Negligible | Negligible | Very Strong | Strong | Negligible | Moderate | Moderate | Negligible | Negligible | None | Very Strong | Moderate | Negligible | Weak | Weak | Moderate | Weak | Moderate | Weak | Weak | Moderate | Moderate | Moderate | Negligible | Negligible | Weak | Moderate | Negligible | Strong | Moderate | Weak | Moderate | Negligible | Weak | Moderate | Negligible | Weak | Moderate | Negligible | Negligible | Negligible | Negligible | Matthews correlation coefficient interpretation |
| MCEN | 0.50132 | 0.29287 | 0.49987 | 0.14093 | 0.30662 | 0.39429 | 0.1424 | 0.28383 | 0.38779 | 0.49613 | 0.06063 | 0.19072 | 0.31532 | 0.22084 | 0.28589 | 0.40336 | 0.29 | 0.23961 | 0 | 0.41457 | 0.22664 | 0.45285 | 0.4503 | 0.42164 | 0.4157 | 0.44362 | 0.37049 | 0.49019 | 0.37511 | 0.23532 | 0.43302 | 0.57203 | 0.57907 | 0.3272 | 0.38974 | 0.57865 | 0.13312 | 0.21422 | 0.30664 | 0.33888 | 0.5582 | 0.1824 | 0.30533 | 0.37977 | 0.35327 | 0.32447 | 0.27914 | 0.25164 | 0.43087 | 0.27383 | Modified confusion entropy |
| MK | 0.38917 | 0.62969 | 0.46539 | 0.92716 | 0.86549 | -0.00282 | 0.69565 | 0.79647 | 0.26055 | 0.24648 | 0.99977 | 0.74834 | 0.49648 | 0.69114 | 0.33333 | 0.31206 | -0.00047 | None | 1.0 | 0.47463 | 0.0748 | 0.3375 | 0.42466 | 0.55918 | 0.55133 | 0.48626 | 0.77191 | 0.46138 | 0.5777 | 0.72633 | 0.56003 | 0.26778 | 0.21132 | 0.99484 | 0.51145 | 0.26632 | 0.99906 | 0.77637 | 0.18158 | 0.50994 | 0.30275 | 0.99272 | 0.4993 | 0.28766 | 0.49575 | 0.64098 | -0.00094 | -0.00141 | -0.00446 | -0.00094 | Markedness |
| N | 4197 | 4236 | 4194 | 4249 | 4172 | 4256 | 4252 | 4241 | 4238 | 4250 | 4261 | 4222 | 4250 | 4229 | 4265 | 4235 | 4266 | 4265 | 4266 | 4192 | 4257 | 4128 | 4186 | 4185 | 4245 | 3192 | 4236 | 3979 | 3895 | 4256 | 3733 | 3999 | 4042 | 4240 | 4238 | 4161 | 4259 | 4255 | 4265 | 4217 | 4169 | 4229 | 4261 | 4244 | 4235 | 4251 | 4264 | 4262 | 4249 | 4264 | Condition negative |
| NLR | 0.34393 | 0.25083 | 0.84044 | 0.31586 | 0.41747 | 1.00236 | 0.0 | 0.55595 | 0.86892 | 0.8351 | 0.14286 | 0.15264 | 0.83392 | 0.35991 | 0.0 | 0.79068 | 1.00094 | 1.0 | 0.0 | 0.45222 | 0.82282 | 0.69892 | 0.66425 | 0.32868 | 0.78335 | 0.29333 | 0.78162 | 0.50354 | 0.26176 | 0.33357 | 0.45061 | 0.86621 | 0.83557 | 0.78571 | 0.40161 | 0.76902 | 0.44444 | 0.46176 | 0.33404 | 0.03966 | 0.89382 | 0.79487 | 0.42897 | 0.71118 | 0.54739 | 0.47114 | 1.00141 | 1.00023 | 1.0026 | 1.00094 | Negative likelihood ratio |
| NLRI | Poor | Poor | Negligible | Poor | Poor | Negligible | Good | Negligible | Negligible | Negligible | Fair | Fair | Negligible | Poor | Good | Negligible | Negligible | Negligible | Good | Poor | Negligible | Negligible | Negligible | Poor | Negligible | Poor | Negligible | Negligible | Poor | Poor | Poor | Negligible | Negligible | Negligible | Poor | Negligible | Poor | Poor | Poor | Good | Negligible | Negligible | Poor | Negligible | Negligible | Poor | Negligible | Negligible | Negligible | Negligible | Negative likelihood ratio interpretation |
| NPV | 0.99422 | 0.99811 | 0.98539 | 0.99859 | 0.99049 | 0.99718 | 1.0 | 0.99647 | 0.99389 | 0.99648 | 0.99977 | 0.99834 | 0.99648 | 0.99669 | 1.0 | 0.99388 | 0.99953 | 0.9993 | 1.0 | 0.99187 | 0.99788 | 0.97685 | 0.98716 | 0.99352 | 0.99577 | 0.91002 | 0.99413 | 0.96472 | 0.97555 | 0.99906 | 0.93934 | 0.94494 | 0.95537 | 0.99484 | 0.99717 | 0.98061 | 0.99906 | 0.99859 | 0.99977 | 0.99952 | 0.97922 | 0.99272 | 0.9993 | 0.99599 | 0.99575 | 0.99812 | 0.99906 | 0.99859 | 0.99554 | 0.99906 | Negative predictive value |
| OC | 0.66197 | 0.75 | 0.48 | 0.92857 | 0.875 | 0.0 | 1.0 | 0.8 | 0.26667 | 0.25 | 1.0 | 0.84783 | 0.5 | 0.69444 | 1.0 | 0.31818 | 0.0 | None | 1.0 | 0.55263 | 0.18182 | 0.36066 | 0.4375 | 0.6747 | 0.55556 | 0.76208 | 0.77778 | 0.51557 | 0.75067 | 0.72727 | 0.62069 | 0.32283 | 0.25595 | 1.0 | 0.6 | 0.28571 | 1.0 | 0.77778 | 0.66667 | 0.96078 | 0.32353 | 1.0 | 0.57143 | 0.29167 | 0.5 | 0.64286 | 0.0 | 0.0 | 0.0 | 0.0 | Overlap coefficient |
| OOC | 0.51132 | 0.68825 | 0.27899 | 0.79708 | 0.71443 | 0.0 | 0.83406 | 0.59628 | 0.18856 | 0.20412 | 0.92582 | 0.79741 | 0.28868 | 0.6672 | 0.57735 | 0.25979 | 0.0 | None | 1.0 | 0.51651 | 0.11826 | 0.33667 | 0.38651 | 0.61778 | 0.34752 | 0.66268 | 0.41248 | 0.50603 | 0.67232 | 0.69631 | 0.59583 | 0.22182 | 0.22068 | 0.46291 | 0.55549 | 0.26349 | 0.74536 | 0.64715 | 0.34816 | 0.70029 | 0.1896 | 0.45291 | 0.53452 | 0.29167 | 0.47673 | 0.58338 | 0.0 | 0.0 | 0.0 | 0.0 | Otsuka-Ochiai coefficient |
| OP | 0.78243 | 0.85361 | 0.26224 | 0.81097 | 0.72649 | -0.00515 | 0.99754 | 0.61147 | 0.22717 | 0.28061 | 0.92284 | 0.91449 | 0.28167 | 0.77663 | 0.99789 | 0.34142 | -0.00141 | -0.0007 | 1.0 | 0.69831 | 0.30144 | 0.44447 | 0.49129 | 0.79433 | 0.35226 | 0.76758 | 0.35279 | 0.62966 | 0.81636 | 0.7987 | 0.65419 | 0.19598 | 0.25607 | 0.34779 | 0.74509 | 0.36175 | 0.71335 | 0.69834 | 0.79867 | 0.97412 | 0.17499 | 0.33316 | 0.72607 | 0.44505 | 0.61879 | 0.68979 | -0.00234 | -0.00164 | -0.00703 | -0.00187 | Optimized precision |
| P | 71 | 32 | 74 | 19 | 96 | 12 | 16 | 27 | 30 | 18 | 7 | 46 | 18 | 39 | 3 | 33 | 2 | 3 | 2 | 76 | 11 | 140 | 82 | 83 | 23 | 1076 | 32 | 289 | 373 | 12 | 535 | 269 | 226 | 28 | 30 | 107 | 9 | 13 | 3 | 51 | 99 | 39 | 7 | 24 | 33 | 17 | 4 | 6 | 19 | 4 | Condition positive or support |
| PLR | 38.58744 | 226.92857 | 52.31601 | 2907.21053 | 304.20833 | 0.0 | 607.42857 | 628.2963 | 51.3697 | 78.7037 | None | 275.34783 | 236.11111 | 246.44522 | 710.83333 | 59.88889 | 0.0 | None | None | 51.4807 | 32.25 | 16.63297 | 39.70461 | 65.66545 | 230.70652 | 4.0341 | 463.3125 | 13.58581 | 15.80465 | 945.77778 | 11.41784 | 7.08736 | 6.15242 | None | 149.57647 | 15.55514 | None | 1145.57692 | 315.92593 | 86.20484 | 20.1401 | None | 608.71429 | 72.81373 | 128.33333 | 450.10588 | 0.0 | 0.0 | 0.0 | 0.0 | Positive likelihood ratio |
| PLRI | Good | Good | Good | Good | Good | Negligible | Good | Good | Good | Good | None | Good | Good | Good | Good | Good | Negligible | None | None | Good | Good | Good | Good | Good | Good | Poor | Good | Good | Good | Good | Good | Fair | Fair | None | Good | Good | None | Good | Good | Good | Good | None | Good | Good | Good | Good | Negligible | Negligible | Negligible | Negligible | Positive likelihood ratio interpretation |
| POP | 4268 | 4268 | 4268 | 4268 | 4268 | 4268 | 4268 | 4268 | 4268 | 4268 | 4268 | 4268 | 4268 | 4268 | 4268 | 4268 | 4268 | 4268 | 4268 | 4268 | 4268 | 4268 | 4268 | 4268 | 4268 | 4268 | 4268 | 4268 | 4268 | 4268 | 4268 | 4268 | 4268 | 4268 | 4268 | 4268 | 4268 | 4268 | 4268 | 4268 | 4268 | 4268 | 4268 | 4268 | 4268 | 4268 | 4268 | 4268 | 4268 | 4268 | Population |
| PPV | 0.39496 | 0.63158 | 0.48 | 0.92857 | 0.875 | 0.0 | 0.69565 | 0.8 | 0.26667 | 0.25 | 1.0 | 0.75 | 0.5 | 0.69444 | 0.33333 | 0.31818 | 0.0 | None | 1.0 | 0.48276 | 0.07692 | 0.36066 | 0.4375 | 0.56566 | 0.55556 | 0.57625 | 0.77778 | 0.49667 | 0.60215 | 0.72727 | 0.62069 | 0.32283 | 0.25595 | 1.0 | 0.51429 | 0.28571 | 1.0 | 0.77778 | 0.18182 | 0.51042 | 0.32353 | 1.0 | 0.5 | 0.29167 | 0.5 | 0.64286 | 0.0 | 0.0 | 0.0 | 0.0 | Precision or positive predictive value |
| PRE | 0.01664 | 0.0075 | 0.01734 | 0.00445 | 0.02249 | 0.00281 | 0.00375 | 0.00633 | 0.00703 | 0.00422 | 0.00164 | 0.01078 | 0.00422 | 0.00914 | 0.0007 | 0.00773 | 0.00047 | 0.0007 | 0.00047 | 0.01781 | 0.00258 | 0.0328 | 0.01921 | 0.01945 | 0.00539 | 0.25211 | 0.0075 | 0.06771 | 0.08739 | 0.00281 | 0.12535 | 0.06303 | 0.05295 | 0.00656 | 0.00703 | 0.02507 | 0.00211 | 0.00305 | 0.0007 | 0.01195 | 0.0232 | 0.00914 | 0.00164 | 0.00562 | 0.00773 | 0.00398 | 0.00094 | 0.00141 | 0.00445 | 0.00094 | Prevalence |
| Q | 0.98233 | 0.99779 | 0.96838 | 0.99978 | 0.99726 | -1.0 | None | 0.99823 | 0.96673 | 0.979 | None | 0.99889 | 0.99296 | 0.99708 | None | 0.97394 | -1.0 | None | None | 0.98258 | 0.95024 | 0.91935 | 0.96709 | 0.99004 | 0.99323 | 0.86443 | 0.99663 | 0.92852 | 0.96741 | 0.99929 | 0.92407 | 0.78218 | 0.76085 | None | 0.99464 | 0.90578 | None | 0.99919 | 0.99789 | 0.99908 | 0.91501 | None | 0.99859 | 0.98065 | 0.99151 | 0.99791 | -1.0 | -1.0 | -1.0 | -1.0 | Yule Q - coefficient of colligation |
| QI | Strong | Strong | Strong | Strong | Strong | Negligible | None | Strong | Strong | Strong | None | Strong | Strong | Strong | None | Strong | Negligible | None | None | Strong | Strong | Strong | Strong | Strong | Strong | Strong | Strong | Strong | Strong | Strong | Strong | Strong | Strong | None | Strong | Strong | None | Strong | Strong | Strong | Strong | None | Strong | Strong | Strong | Strong | Negligible | Negligible | Negligible | Negligible | Yule Q interpretation |
| RACC | 0.00046 | 7e-05 | 0.0001 | 1e-05 | 0.00034 | 1e-05 | 2e-05 | 2e-05 | 2e-05 | 1e-05 | 0.0 | 0.00013 | 1e-05 | 8e-05 | 0.0 | 4e-05 | 0.0 | 0.0 | 0.0 | 0.00036 | 2e-05 | 0.00094 | 0.00029 | 0.00045 | 1e-05 | 0.08406 | 2e-05 | 0.00476 | 0.00952 | 1e-05 | 0.01448 | 0.00188 | 0.00208 | 1e-05 | 6e-05 | 0.00053 | 0.0 | 1e-05 | 0.0 | 0.00027 | 0.00018 | 2e-05 | 0.0 | 3e-05 | 5e-05 | 1e-05 | 0.0 | 0.0 | 1e-05 | 0.0 | Random accuracy |
| RACCU | 0.0005 | 7e-05 | 0.00013 | 1e-05 | 0.00035 | 1e-05 | 2e-05 | 2e-05 | 3e-05 | 1e-05 | 0.0 | 0.00013 | 1e-05 | 8e-05 | 0.0 | 4e-05 | 0.0 | 0.0 | 0.0 | 0.00036 | 2e-05 | 0.00094 | 0.00029 | 0.00045 | 1e-05 | 0.08571 | 2e-05 | 0.00476 | 0.00964 | 1e-05 | 0.0145 | 0.00215 | 0.00213 | 2e-05 | 6e-05 | 0.00054 | 0.0 | 1e-05 | 0.0 | 0.0003 | 0.00024 | 3e-05 | 0.0 | 3e-05 | 5e-05 | 1e-05 | 0.0 | 0.0 | 1e-05 | 0.0 | Random accuracy unbiased |
| TN | 4125 | 4222 | 4181 | 4248 | 4164 | 4246 | 4245 | 4238 | 4227 | 4241 | 4261 | 4209 | 4247 | 4218 | 4259 | 4220 | 4262 | 4265 | 4266 | 4147 | 4233 | 4050 | 4150 | 4142 | 4241 | 2589 | 4234 | 3828 | 3710 | 4253 | 3546 | 3913 | 3917 | 4240 | 4221 | 4096 | 4259 | 4253 | 4256 | 4170 | 4146 | 4229 | 4257 | 4227 | 4220 | 4246 | 4258 | 4261 | 4238 | 4260 | True negative/correct rejection |
| TNR | 0.98284 | 0.99669 | 0.9969 | 0.99976 | 0.99808 | 0.99765 | 0.99835 | 0.99929 | 0.9974 | 0.99788 | 1.0 | 0.99692 | 0.99929 | 0.9974 | 0.99859 | 0.99646 | 0.99906 | 1.0 | 1.0 | 0.98927 | 0.99436 | 0.9811 | 0.9914 | 0.98973 | 0.99906 | 0.81109 | 0.99953 | 0.96205 | 0.9525 | 0.9993 | 0.94991 | 0.97849 | 0.96907 | 1.0 | 0.99599 | 0.98438 | 1.0 | 0.99953 | 0.99789 | 0.98885 | 0.99448 | 1.0 | 0.99906 | 0.99599 | 0.99646 | 0.99882 | 0.99859 | 0.99977 | 0.99741 | 0.99906 | Specificity or true negative rate |
| TON | 4149 | 4230 | 4243 | 4254 | 4204 | 4258 | 4245 | 4253 | 4253 | 4256 | 4262 | 4216 | 4262 | 4232 | 4259 | 4246 | 4264 | 4268 | 4266 | 4181 | 4242 | 4146 | 4204 | 4169 | 4259 | 2845 | 4259 | 3968 | 3803 | 4257 | 3775 | 4141 | 4100 | 4262 | 4233 | 4177 | 4263 | 4259 | 4257 | 4172 | 4234 | 4260 | 4260 | 4244 | 4238 | 4254 | 4262 | 4267 | 4257 | 4264 | Test outcome negative |
| TOP | 119 | 38 | 25 | 14 | 64 | 10 | 23 | 15 | 15 | 12 | 6 | 52 | 6 | 36 | 9 | 22 | 4 | 0 | 2 | 87 | 26 | 122 | 64 | 99 | 9 | 1423 | 9 | 300 | 465 | 11 | 493 | 127 | 168 | 6 | 35 | 91 | 5 | 9 | 11 | 96 | 34 | 8 | 8 | 24 | 30 | 14 | 6 | 1 | 11 | 4 | Test outcome positive |
| TP | 47 | 24 | 12 | 13 | 56 | 0 | 16 | 12 | 4 | 3 | 6 | 39 | 3 | 25 | 3 | 7 | 0 | 0 | 2 | 42 | 2 | 44 | 28 | 56 | 5 | 820 | 7 | 149 | 280 | 8 | 306 | 41 | 43 | 6 | 18 | 26 | 5 | 7 | 2 | 49 | 11 | 8 | 4 | 7 | 15 | 9 | 0 | 0 | 0 | 0 | True positive/hit |
| TPR | 0.66197 | 0.75 | 0.16216 | 0.68421 | 0.58333 | 0.0 | 1.0 | 0.44444 | 0.13333 | 0.16667 | 0.85714 | 0.84783 | 0.16667 | 0.64103 | 1.0 | 0.21212 | 0.0 | 0.0 | 1.0 | 0.55263 | 0.18182 | 0.31429 | 0.34146 | 0.6747 | 0.21739 | 0.76208 | 0.21875 | 0.51557 | 0.75067 | 0.66667 | 0.57196 | 0.15242 | 0.19027 | 0.21429 | 0.6 | 0.24299 | 0.55556 | 0.53846 | 0.66667 | 0.96078 | 0.11111 | 0.20513 | 0.57143 | 0.29167 | 0.45455 | 0.52941 | 0.0 | 0.0 | 0.0 | 0.0 | Sensitivity, recall, hit rate, or true positive rate |
| Y | 0.64482 | 0.74669 | 0.15906 | 0.68398 | 0.58142 | -0.00235 | 0.99835 | 0.44374 | 0.13074 | 0.16455 | 0.85714 | 0.84475 | 0.16596 | 0.63842 | 0.99859 | 0.20858 | -0.00094 | 0.0 | 1.0 | 0.5419 | 0.17618 | 0.29539 | 0.33286 | 0.66442 | 0.21645 | 0.57317 | 0.21828 | 0.47762 | 0.70317 | 0.66596 | 0.52187 | 0.13091 | 0.15934 | 0.21429 | 0.59599 | 0.22737 | 0.55556 | 0.53799 | 0.66456 | 0.94964 | 0.10559 | 0.20513 | 0.57049 | 0.28766 | 0.451 | 0.52824 | -0.00141 | -0.00023 | -0.00259 | -0.00094 | Youden index |
| dInd | 0.33846 | 0.25002 | 0.83784 | 0.31579 | 0.41667 | 1.0 | 0.00165 | 0.55556 | 0.86667 | 0.83334 | 0.14286 | 0.15221 | 0.83333 | 0.35898 | 0.00141 | 0.78789 | 1.0 | 1.0 | 0.0 | 0.4475 | 0.8182 | 0.68597 | 0.65859 | 0.32546 | 0.78261 | 0.3038 | 0.78125 | 0.48591 | 0.25381 | 0.33333 | 0.43096 | 0.84786 | 0.81032 | 0.78571 | 0.40002 | 0.75717 | 0.44444 | 0.46154 | 0.33334 | 0.04077 | 0.88891 | 0.79487 | 0.42857 | 0.70834 | 0.54547 | 0.47059 | 1.0 | 1.0 | 1.0 | 1.0 | Distance index |
| sInd | 0.76067 | 0.82321 | 0.40756 | 0.7767 | 0.70537 | 0.29289 | 0.99884 | 0.60716 | 0.38717 | 0.41074 | 0.89898 | 0.89237 | 0.41074 | 0.74616 | 0.99901 | 0.44288 | 0.29289 | 0.29289 | 1.0 | 0.68357 | 0.42144 | 0.51494 | 0.5343 | 0.76986 | 0.44661 | 0.78518 | 0.44757 | 0.65641 | 0.82053 | 0.7643 | 0.69527 | 0.40047 | 0.42701 | 0.44442 | 0.71714 | 0.4646 | 0.68573 | 0.67364 | 0.76429 | 0.97117 | 0.37145 | 0.43794 | 0.69695 | 0.49912 | 0.6143 | 0.66724 | 0.29289 | 0.29289 | 0.29289 | 0.29289 | Similarity index |

Generated By PyCM Version 3.6
