# Supplementary material for: TopEC: prediction of Enzyme Commission classes by 3D graph neural networks and localized 3D protein descriptor
Source: Nat Commun. 2025 Mar 20;16:2737. doi: 10.1038/s41467-025-57324-5 (PMC11923149; doi:10.1038/s41467-025-57324-5)
Supplement: Supplementary file 3 — Supplementary Data 1 [file 41467_2025_57324_MOESM3_ESM.zip › Data_S1/figure2/PDB300_main.html]

PyCM Report


# PyCM Report

## Dataset Type :

- Multi-Class Classification
- Imbalanced

Note 1 : Recommended statistics for this type of classification highlighted in aqua

Note 2 : The recommender system assumes that the input is the result of classification over the whole data rather than just a part of it.
If the confusion matrix is the result of test data classification, the recommendation is not valid.

## Confusion Matrix :

|  |  |  |  |  |  |  |  |  |  |  |  |  |  |  |  |  |  |  |  |  |  |  |  |  |  |  |  |  |  |  |  |  |  |  |  |  |  |  |  |  |  |  |  |  |  |  |  |  |  |  |  |  |  |  |  |  |  |  |  |  |  |  |  |  |  |
| --- | --- | --- | --- | --- | --- | --- | --- | --- | --- | --- | --- | --- | --- | --- | --- | --- | --- | --- | --- | --- | --- | --- | --- | --- | --- | --- | --- | --- | --- | --- | --- | --- | --- | --- | --- | --- | --- | --- | --- | --- | --- | --- | --- | --- | --- | --- | --- | --- | --- | --- | --- | --- | --- | --- | --- | --- | --- | --- | --- | --- | --- | --- | --- | --- | --- |
| Actual | Predict  |  |  |  |  |  |  |  |  | | --- | --- | --- | --- | --- | --- | --- | --- | |  | 1 | 2 | 3 | 4 | 5 | 6 | 7 | | 1 | 309 | 109 | 71 | 24 | 31 | 2 | 2 | | 2 | 44 | 1196 | 228 | 13 | 22 | 16 | 4 | | 3 | 88 | 423 | 1126 | 38 | 40 | 9 | 8 | | 4 | 8 | 45 | 41 | 62 | 2 | 0 | 1 | | 5 | 21 | 36 | 81 | 3 | 82 | 0 | 0 | | 6 | 1 | 27 | 5 | 0 | 3 | 24 | 0 | | 7 | 1 | 3 | 18 | 0 | 1 | 0 | 0 | |

## Overall Statistics :

|  |  |
| --- | --- |
| 95% CI | (0.64156,0.67006) |
| ACC Macro | 0.90166 |
| ARI | 0.29126 |
| AUNP | 0.74673 |
| AUNU | 0.68931 |
| Bangdiwala B | 0.47991 |
| Bennett S | 0.59845 |
| CBA | 0.43171 |
| CSI | -0.06491 |
| Chi-Squared | 4604.63917 |
| Chi-Squared DF | 36 |
| Conditional Entropy | 1.3963 |
| Cramer V | 0.42404 |
| Cross Entropy | 1.98243 |
| F1 Macro | 0.46464 |
| F1 Micro | 0.65581 |
| FNR Macro | 0.54901 |
| FNR Micro | 0.34419 |
| FPR Macro | 0.07236 |
| FPR Micro | 0.05736 |
| Gwet AC1 | 0.61201 |
| Hamming Loss | 0.34419 |
| Joint Entropy | 3.36146 |
| KL Divergence | 0.01727 |
| Kappa | 0.4932 |
| Kappa 95% CI | (0.47221,0.51419) |
| Kappa No Prevalence | 0.31162 |
| Kappa Standard Error | 0.01071 |
| Kappa Unbiased | 0.49182 |
| Krippendorff Alpha | 0.49188 |
| Lambda A | 0.4239 |
| Lambda B | 0.40387 |
| Mutual Information | 0.46917 |
| NIR | 0.40581 |
| Overall ACC | 0.65581 |
| Overall CEN | 0.39056 |
| Overall J | (2.29636,0.32805) |
| Overall MCC | 0.49596 |
| Overall MCEN | 0.50255 |
| Overall RACC | 0.32086 |
| Overall RACCU | 0.3227 |
| P-Value | None |
| PPV Macro | 0.4841 |
| PPV Micro | 0.65581 |
| Pearson C | 0.7204 |
| Phi-Squared | 1.07888 |
| RCI | 0.23874 |
| RR | 609.71429 |
| Reference Entropy | 1.96516 |
| Response Entropy | 1.86547 |
| SOA1(Landis & Koch) | Moderate |
| SOA2(Fleiss) | Intermediate to Good |
| SOA3(Altman) | Moderate |
| SOA4(Cicchetti) | Fair |
| SOA5(Cramer) | Relatively Strong |
| SOA6(Matthews) | Weak |
| Scott PI | 0.49182 |
| Standard Error | 0.00727 |
| TNR Macro | 0.92764 |
| TNR Micro | 0.94264 |
| TPR Macro | 0.45099 |
| TPR Micro | 0.65581 |
| Zero-one Loss | 1469 |

## Class Statistics :

|  |  |  |  |  |  |  |  |  |
| --- | --- | --- | --- | --- | --- | --- | --- | --- |
| Class | 1 | 2 | 3 | 4 | 5 | 6 | 7 | Description |
| ACC | 0.90581 | 0.77273 | 0.75398 | 0.959 | 0.94377 | 0.98524 | 0.9911 | Accuracy |
| AGF | 0.73866 | 0.79746 | 0.72107 | 0.62487 | 0.60802 | 0.63955 | 0.0 | Adjusted F-score |
| AGM | 0.83762 | 0.77166 | 0.76684 | 0.79632 | 0.78217 | 0.81072 | 0 | Adjusted geometric mean |
| AM | -76 | 316 | -162 | -19 | -42 | -9 | -8 | Difference between automatic and manual classification |
| AUC | 0.76003 | 0.77552 | 0.73752 | 0.68548 | 0.67162 | 0.69679 | 0.49823 | Area under the ROC curve |
| AUCI | Good | Good | Good | Fair | Fair | Fair | Poor | AUC value interpretation |
| AUPR | 0.60926 | 0.71782 | 0.68366 | 0.4164 | 0.41038 | 0.43529 | 0.0 | Area under the PR curve |
| BB | 0.56387 | 0.65035 | 0.65012 | 0.38994 | 0.36771 | 0.4 | 0.0 | Braun-Blanquet similarity |
| BCD | 0.0089 | 0.03702 | 0.01898 | 0.00223 | 0.00492 | 0.00105 | 0.00094 | Bray-Curtis dissimilarity |
| BM | 0.52005 | 0.55105 | 0.47504 | 0.37095 | 0.34324 | 0.39358 | -0.00353 | Informedness or bookmaker informedness |
| CEN | 0.45147 | 0.34223 | 0.38078 | 0.54491 | 0.55928 | 0.47438 | 0.62841 | Confusion entropy |
| DOR | 28.21349 | 11.95653 | 8.75476 | 33.03225 | 23.18017 | 103.23457 | 0.0 | Diagnostic odds ratio |
| DP | 0.79968 | 0.59411 | 0.51949 | 0.83743 | 0.75263 | 1.11028 | None | Discriminant power |
| DPI | Poor | Poor | Poor | Poor | Poor | Limited | None | Discriminant power interpretation |
| ERR | 0.09419 | 0.22727 | 0.24602 | 0.041 | 0.05623 | 0.01476 | 0.0089 | Error rate |
| F0.5 | 0.63424 | 0.6735 | 0.7027 | 0.43115 | 0.43295 | 0.45455 | 0.0 | F0.5 score |
| F1 | 0.60588 | 0.71148 | 0.68201 | 0.41472 | 0.40594 | 0.43243 | 0.0 | F1 score - harmonic mean of precision and sensitivity |
| F2 | 0.57995 | 0.754 | 0.66251 | 0.39948 | 0.38211 | 0.41237 | 0.0 | F2 score |
| FDR | 0.34534 | 0.34965 | 0.2828 | 0.55714 | 0.54696 | 0.52941 | 1.0 | False discovery rate |
| FN | 239 | 327 | 606 | 97 | 141 | 36 | 23 | False negative/miss/type 2 error |
| FNR | 0.43613 | 0.21471 | 0.34988 | 0.61006 | 0.63229 | 0.6 | 1.0 | Miss rate or false negative rate |
| FOR | 0.06296 | 0.13462 | 0.22461 | 0.0235 | 0.0345 | 0.00854 | 0.00541 | False omission rate |
| FP | 163 | 643 | 444 | 78 | 99 | 27 | 15 | False positive/type 1 error/false alarm |
| FPR | 0.04382 | 0.23424 | 0.17508 | 0.01898 | 0.02447 | 0.00642 | 0.00353 | Fall-out or false positive rate |
| G | 0.60757 | 0.71465 | 0.68283 | 0.41556 | 0.40815 | 0.43386 | 0.0 | G-measure geometric mean of precision and sensitivity |
| GI | 0.52005 | 0.55105 | 0.47504 | 0.37095 | 0.34324 | 0.39358 | -0.00353 | Gini index |
| GM | 0.73428 | 0.77546 | 0.73232 | 0.61849 | 0.59893 | 0.63042 | 0.0 | G-mean geometric mean of specificity and sensitivity |
| HD | 402 | 970 | 1050 | 175 | 240 | 63 | 38 | Hamming distance |
| IBA | 0.32764 | 0.61309 | 0.44255 | 0.15643 | 0.14068 | 0.16152 | 0.0 | Index of balanced accuracy |
| ICSI | 0.21853 | 0.43565 | 0.36731 | -0.16721 | -0.17925 | -0.12941 | -1.0 | Individual classification success index |
| IS | 2.35013 | 0.86594 | 0.82156 | 3.57137 | 3.11615 | 5.06499 | None | Information score |
| J | 0.4346 | 0.55217 | 0.51746 | 0.2616 | 0.25466 | 0.27586 | 0.0 | Jaccard index |
| LS | 5.09871 | 1.82253 | 1.76732 | 11.88751 | 8.67071 | 33.47451 | 0.0 | Lift score |
| MCC | 0.55472 | 0.5331 | 0.48373 | 0.39441 | 0.37902 | 0.42645 | -0.00437 | Matthews correlation coefficient |
| MCCI | Moderate | Moderate | Weak | Weak | Weak | Weak | Negligible | Matthews correlation coefficient interpretation |
| MCEN | 0.56556 | 0.45196 | 0.49683 | 0.6184 | 0.63365 | 0.53425 | 0.62841 | Modified confusion entropy |
| MK | 0.5917 | 0.51573 | 0.49259 | 0.41936 | 0.41854 | 0.46205 | -0.00541 | Markedness |
| N | 3720 | 2745 | 2536 | 4109 | 4045 | 4208 | 4245 | Condition negative |
| NLR | 0.45612 | 0.28039 | 0.42414 | 0.62187 | 0.64815 | 0.60387 | 1.00355 | Negative likelihood ratio |
| NLRI | Poor | Poor | Poor | Negligible | Negligible | Negligible | Negligible | Negative likelihood ratio interpretation |
| NPV | 0.93704 | 0.86538 | 0.77539 | 0.9765 | 0.9655 | 0.99146 | 0.99459 | Negative predictive value |
| OC | 0.65466 | 0.78529 | 0.7172 | 0.44286 | 0.45304 | 0.47059 | 0.0 | Overlap coefficient |
| OOC | 0.60757 | 0.71465 | 0.68283 | 0.41556 | 0.40815 | 0.43386 | 0.0 | Otsuka-Ochiai coefficient |
| OP | 0.64772 | 0.76013 | 0.63547 | 0.52785 | 0.49127 | 0.5593 | -0.0089 | Optimized precision |
| P | 548 | 1523 | 1732 | 159 | 223 | 60 | 23 | Condition positive or support |
| PLR | 12.86866 | 3.35245 | 3.71327 | 20.54169 | 15.02423 | 62.34074 | 0.0 | Positive likelihood ratio |
| PLRI | Good | Poor | Poor | Good | Good | Good | Negligible | Positive likelihood ratio interpretation |
| POP | 4268 | 4268 | 4268 | 4268 | 4268 | 4268 | 4268 | Population |
| PPV | 0.65466 | 0.65035 | 0.7172 | 0.44286 | 0.45304 | 0.47059 | 0.0 | Precision or positive predictive value |
| PRE | 0.1284 | 0.35684 | 0.40581 | 0.03725 | 0.05225 | 0.01406 | 0.00539 | Prevalence |
| Q | 0.93154 | 0.84564 | 0.79497 | 0.94123 | 0.91729 | 0.98081 | -1.0 | Yule Q - coefficient of colligation |
| QI | Strong | Strong | Strong | Strong | Strong | Strong | Negligible | Yule Q interpretation |
| RACC | 0.0142 | 0.15376 | 0.14928 | 0.00122 | 0.00222 | 0.00017 | 2e-05 | Random accuracy |
| RACCU | 0.01428 | 0.15513 | 0.14964 | 0.00123 | 0.00224 | 0.00017 | 2e-05 | Random accuracy unbiased |
| TN | 3557 | 2102 | 2092 | 4031 | 3946 | 4181 | 4230 | True negative/correct rejection |
| TNR | 0.95618 | 0.76576 | 0.82492 | 0.98102 | 0.97553 | 0.99358 | 0.99647 | Specificity or true negative rate |
| TON | 3796 | 2429 | 2698 | 4128 | 4087 | 4217 | 4253 | Test outcome negative |
| TOP | 472 | 1839 | 1570 | 140 | 181 | 51 | 15 | Test outcome positive |
| TP | 309 | 1196 | 1126 | 62 | 82 | 24 | 0 | True positive/hit |
| TPR | 0.56387 | 0.78529 | 0.65012 | 0.38994 | 0.36771 | 0.4 | 0.0 | Sensitivity, recall, hit rate, or true positive rate |
| Y | 0.52005 | 0.55105 | 0.47504 | 0.37095 | 0.34324 | 0.39358 | -0.00353 | Youden index |
| dInd | 0.43833 | 0.31776 | 0.39124 | 0.61036 | 0.63276 | 0.60003 | 1.00001 | Distance index |
| sInd | 0.69006 | 0.77531 | 0.72335 | 0.56841 | 0.55257 | 0.57571 | 0.29289 | Similarity index |

Generated By PyCM Version 3.6
