# Supplementary material for: TopEC: prediction of Enzyme Commission classes by 3D graph neural networks and localized 3D protein descriptor
Source: Nat Commun. 2025 Mar 20;16:2737. doi: 10.1038/s41467-025-57324-5 (PMC11923149; doi:10.1038/s41467-025-57324-5)
Supplement: Supplementary file 3 — Supplementary Data 1 [file 41467_2025_57324_MOESM3_ESM.zip › Data_S1/figure2/AF703_sub.html]

PyCM Report


# PyCM Report

## Dataset Type :

- Multi-Class Classification
- Imbalanced

Note 1 : Recommended statistics for this type of classification highlighted in aqua

Note 2 : The recommender system assumes that the input is the result of classification over the whole data rather than just a part of it.
If the confusion matrix is the result of test data classification, the recommendation is not valid.

## Confusion Matrix :

|  |  |  |  |  |  |  |  |  |  |  |  |  |  |  |  |  |  |  |  |  |  |  |  |  |  |  |  |  |  |  |  |  |  |  |  |  |  |  |  |  |  |  |  |  |  |  |  |  |  |  |  |  |  |  |  |  |  |  |  |  |  |  |  |  |  |  |  |  |  |  |  |  |  |  |  |  |  |  |  |  |  |  |  |  |  |  |  |  |  |  |  |  |  |  |  |  |  |  |  |  |  |  |  |  |  |  |  |  |  |  |  |  |  |  |  |  |  |  |  |  |  |  |  |  |  |  |  |  |  |  |  |  |  |  |  |  |  |  |  |  |  |  |  |  |  |  |  |  |  |  |  |  |  |  |  |  |  |  |  |  |  |  |  |  |  |  |  |  |  |  |  |  |  |  |  |  |  |  |  |  |  |  |  |  |  |  |  |  |  |  |  |  |  |  |  |  |  |  |  |  |  |  |  |  |  |  |  |  |  |  |  |  |  |  |  |  |  |  |  |  |  |  |  |  |  |  |  |  |  |  |  |  |  |  |  |  |  |  |  |  |  |  |  |  |  |  |  |  |  |  |  |  |  |  |  |  |  |  |  |  |  |  |  |  |  |  |  |  |  |  |  |  |  |  |  |  |  |  |  |  |  |  |  |  |  |  |  |  |  |  |  |  |  |  |  |  |  |  |  |  |  |  |  |  |  |  |  |  |  |  |  |  |  |  |  |  |  |  |  |  |  |  |  |  |  |  |  |  |  |  |  |  |  |  |  |  |  |  |  |  |  |  |  |  |  |  |  |  |  |  |  |  |  |  |  |  |  |  |  |  |  |  |  |  |  |  |  |  |  |  |  |  |  |  |  |  |  |  |  |  |  |  |  |  |  |  |  |  |  |  |  |  |  |  |  |  |  |  |  |  |  |  |  |  |  |  |  |  |  |  |  |  |  |  |  |  |  |  |  |  |  |  |  |  |  |  |  |  |  |  |  |  |  |  |  |  |  |  |  |  |  |  |  |  |  |  |  |  |  |  |  |  |  |  |  |  |  |  |  |  |  |  |  |  |  |  |  |  |  |  |  |  |  |  |  |  |  |  |  |  |  |  |  |  |  |  |  |  |  |  |  |  |  |  |  |  |  |  |  |  |  |  |  |  |  |  |  |  |  |  |  |  |  |  |  |  |  |  |  |  |  |  |  |  |  |  |  |  |  |  |  |  |  |  |  |  |  |  |  |  |  |  |  |  |  |  |  |  |  |  |  |  |  |  |  |  |  |  |  |  |  |  |  |  |  |  |  |  |  |  |  |  |  |  |  |  |  |  |  |  |  |  |  |  |  |  |  |  |  |  |  |  |  |  |  |  |  |  |  |  |  |  |  |  |  |  |  |  |  |  |  |  |  |  |  |  |  |  |  |  |  |  |  |  |  |  |  |  |  |  |  |  |  |  |  |  |  |  |  |  |  |  |  |  |  |  |  |  |  |  |  |  |  |  |  |  |  |  |  |  |  |  |  |  |  |  |  |  |  |  |  |  |  |  |  |  |  |  |  |  |  |  |  |  |  |  |  |  |  |  |  |  |  |  |  |  |  |  |  |  |  |  |  |  |  |  |  |  |  |  |  |  |  |  |  |  |  |  |  |  |  |  |  |  |  |  |  |  |  |  |  |  |  |  |  |  |  |  |  |  |  |  |  |  |  |  |  |  |  |  |  |  |  |  |  |  |  |  |  |  |  |  |  |  |  |  |  |  |  |  |  |  |  |  |  |  |  |  |  |  |  |  |  |  |  |  |  |  |  |  |  |  |  |  |  |  |  |  |  |  |  |  |  |  |  |  |  |  |  |  |  |  |  |  |  |  |  |  |  |  |  |  |  |  |  |  |  |  |  |  |  |  |  |  |  |  |  |  |  |  |  |  |  |  |  |  |  |  |  |  |  |  |  |  |  |  |  |  |  |  |  |  |  |  |  |  |  |  |  |  |  |  |  |  |  |  |  |  |  |  |  |  |  |  |  |  |  |  |  |  |  |  |  |  |  |  |  |  |  |  |  |  |  |  |  |  |  |  |  |  |  |  |  |  |  |  |  |  |  |  |  |  |  |  |  |  |  |  |  |  |  |  |  |  |  |  |  |  |  |  |  |  |  |  |  |  |  |  |  |  |  |  |  |  |  |  |  |  |  |  |  |  |  |  |  |  |  |  |  |  |  |  |  |  |  |  |  |  |  |  |  |  |  |  |  |  |  |  |  |  |  |  |  |  |  |  |  |  |  |  |  |  |  |  |  |  |  |  |  |  |  |  |  |  |  |  |  |  |  |  |  |  |  |  |  |  |  |  |  |  |  |  |  |  |  |  |  |  |  |  |  |  |  |  |  |  |  |  |  |  |  |  |  |  |  |  |  |  |  |  |  |  |  |  |  |  |  |  |  |  |  |  |  |  |  |  |  |  |  |  |  |  |  |  |  |  |  |  |  |  |  |  |  |  |  |  |  |  |  |  |  |  |  |  |  |  |  |  |  |  |  |  |  |  |  |  |  |  |  |  |  |  |  |  |  |  |  |  |  |  |  |  |  |  |  |  |  |  |  |  |  |  |  |  |  |  |  |  |  |  |  |  |  |  |  |  |  |  |  |  |  |  |  |  |  |  |  |  |  |  |  |  |  |  |  |  |  |  |  |  |  |  |  |  |  |  |  |  |  |  |  |  |  |  |  |  |  |  |  |  |  |  |  |  |  |  |  |  |  |  |  |  |  |  |  |  |  |  |  |  |  |  |  |  |  |  |  |  |  |  |  |  |  |  |  |  |  |  |  |  |  |  |  |  |  |  |  |  |  |  |  |  |  |  |  |  |  |  |  |  |  |  |  |  |  |  |  |  |  |  |  |  |  |  |  |  |  |  |  |  |  |  |  |  |  |  |  |  |  |  |  |  |  |  |  |  |  |  |  |  |  |  |  |  |  |  |  |  |  |  |  |  |  |  |  |  |  |  |  |  |  |  |  |  |  |  |  |  |  |  |  |  |  |  |  |  |  |  |  |  |  |  |  |  |  |  |  |  |  |  |  |  |  |  |  |  |  |  |  |  |  |  |  |  |  |  |  |  |  |  |  |  |  |  |  |  |  |  |  |  |  |  |  |  |  |  |  |  |  |  |  |  |  |  |  |  |  |  |  |  |  |  |  |  |  |  |  |  |  |  |  |  |  |  |  |  |  |  |  |  |  |  |  |  |  |  |  |  |  |  |  |  |  |  |  |  |  |  |  |  |  |  |  |  |  |  |  |  |  |  |  |  |  |  |  |  |  |  |  |  |  |  |  |  |  |  |  |  |  |  |  |  |  |  |  |  |  |  |  |  |  |  |  |  |  |  |  |  |  |  |  |  |  |  |  |  |  |  |  |  |  |  |  |  |  |  |  |  |  |  |  |  |  |  |  |  |  |  |  |  |  |  |  |  |  |  |  |  |  |  |  |  |  |  |  |  |  |  |  |  |  |  |  |  |  |  |  |  |  |  |  |  |  |  |  |  |  |  |  |  |  |  |  |  |  |  |  |  |  |  |  |  |  |  |  |  |  |  |  |  |  |  |  |  |  |  |  |  |  |  |  |  |  |  |  |  |  |  |  |  |  |  |  |  |  |  |  |  |  |  |  |  |  |  |  |  |  |  |  |  |  |  |  |  |  |  |  |  |  |  |  |  |  |  |  |  |  |  |  |  |  |  |  |  |  |  |  |  |  |  |  |  |  |  |  |  |  |  |  |  |  |  |  |  |  |  |  |  |  |  |  |  |  |  |  |  |  |  |  |  |  |  |  |  |  |  |  |  |  |  |  |  |  |  |  |  |  |  |  |  |  |  |  |  |  |  |  |  |  |  |  |  |  |  |  |  |  |  |  |  |  |  |  |  |  |  |  |  |  |  |  |  |  |  |  |  |  |  |  |  |  |  |  |  |  |  |  |  |  |  |  |  |  |  |  |  |  |  |  |  |  |  |  |  |  |  |  |  |  |  |  |  |  |  |  |  |  |  |  |  |  |  |  |  |  |  |  |  |  |  |  |  |  |  |  |  |  |  |  |  |  |  |  |  |  |  |  |  |  |  |  |  |  |  |  |  |  |  |  |  |  |  |  |  |  |  |  |  |  |  |  |  |  |  |  |  |  |  |  |  |  |  |  |  |  |  |  |  |  |  |  |  |  |  |  |  |  |  |  |  |  |  |  |  |  |  |  |  |  |  |  |  |  |  |  |  |  |  |  |  |  |  |  |  |  |  |  |  |  |  |  |  |  |  |  |  |  |  |  |  |  |  |  |  |  |  |  |  |  |  |  |  |  |  |  |  |  |  |  |  |  |  |  |  |  |  |  |  |  |  |  |  |  |  |  |  |  |  |  |  |  |  |  |  |  |  |  |  |  |  |  |  |  |  |  |  |  |  |  |  |  |  |  |  |  |  |  |  |  |  |  |  |  |  |  |  |  |  |  |  |  |  |  |  |  |  |  |  |  |  |  |  |  |  |  |  |  |  |  |  |  |  |  |  |  |  |  |  |  |  |  |  |  |  |  |  |  |  |  |  |  |  |  |  |  |  |  |  |  |  |  |  |  |  |  |  |  |  |  |  |  |  |  |  |  |  |  |  |  |  |  |  |  |  |  |  |  |  |  |  |  |  |  |  |  |  |  |  |  |  |  |  |  |  |  |  |  |  |  |  |  |  |  |  |  |  |  |  |  |  |  |  |  |  |  |  |  |  |  |  |  |  |  |  |  |  |  |  |  |  |  |  |  |  |  |  |  |  |  |  |  |  |  |  |  |  |  |  |  |  |  |  |  |  |  |  |  |  |  |  |  |  |  |  |  |  |  |  |  |  |  |  |  |  |  |  |  |  |  |  |  |  |  |  |  |  |  |  |  |  |  |  |  |  |  |  |  |  |  |  |  |  |  |  |  |  |  |  |  |  |  |  |  |  |  |  |  |  |  |  |  |  |  |  |  |  |  |  |  |  |  |  |  |  |  |  |  |  |  |  |  |  |  |  |  |  |  |  |  |  |  |  |  |  |  |  |  |  |  |  |  |  |  |  |  |  |  |  |  |  |  |  |  |  |  |  |  |  |  |  |  |  |  |  |  |  |  |  |  |  |  |  |  |  |  |  |  |  |  |  |  |  |  |  |  |  |  |  |  |  |  |  |  |  |  |  |  |  |  |  |  |  |  |  |  |  |  |  |  |  |  |  |  |  |  |  |  |  |  |  |  |  |  |  |  |  |  |  |  |  |  |  |  |  |  |  |  |  |  |  |  |  |  |  |  |  |  |  |  |  |  |  |  |  |  |  |  |  |  |  |  |  |  |  |  |  |  |  |  |  |  |  |  |  |  |  |  |  |  |  |  |  |  |  |  |  |  |  |  |  |  |  |  |  |  |  |  |  |  |  |  |  |  |  |  |  |  |  |  |  |  |  |  |  |  |  |  |  |  |  |  |  |  |  |  |  |  |  |  |  |  |  |  |  |  |  |  |  |  |  |  |  |  |  |  |  |  |  |  |  |  |  |  |  |  |  |  |  |  |  |  |  |  |  |  |  |  |  |  |  |  |  |  |  |  |  |  |  |  |  |  |  |  |  |  |  |  |  |  |  |  |  |  |  |  |  |  |  |  |  |  |  |  |  |  |  |  |  |  |  |  |  |  |  |  |  |  |  |  |  |  |  |  |  |  |  |  |  |  |  |  |  |  |  |  |  |  |  |  |  |  |  |  |  |  |  |  |  |  |  |  |  |  |  |  |  |  |  |  |  |  |  |  |  |  |  |  |  |  |  |  |  |  |  |  |  |  |  |  |  |  |  |  |  |  |  |  |  |  |  |  |  |  |  |  |  |  |  |  |  |  |  |  |  |  |  |  |  |  |  |  |  |  |  |  |  |  |  |  |  |  |  |  |  |  |  |  |  |  |  |  |  |  |  |  |  |  |  |  |  |  |  |  |  |  |  |  |  |  |  |  |  |  |  |  |  |  |  |  |  |  |  |  |  |  |  |  |  |  |  |  |  |  |  |  |  |  |  |  |  |  |  |  |  |  |  |  |  |  |  |  |  |  |  |  |  |  |  |  |  |  |  |  |  |  |  |  |  |  |  |  |  |  |  |  |  |  |  |  |  |  |  |  |  |  |  |  |  |  |  |  |  |  |  |  |  |  |  |  |  |  |  |  |  |  |  |  |  |  |  |  |  |  |  |  |  |  |  |  |  |  |  |  |  |  |  |  |  |  |  |  |  |  |  |  |  |  |  |  |  |  |  |  |  |  |  |  |  |  |  |  |  |  |  |  |  |  |  |  |  |  |  |  |  |  |  |  |  |  |  |  |  |  |  |  |  |  |  |  |  |  |  |  |  |  |  |  |  |  |  |  |  |  |  |  |  |  |  |  |  |  |  |  |  |  |  |  |  |  |  |  |  |  |  |  |  |  |  |  |  |  |  |  |  |  |  |  |  |  |  |  |  |  |  |  |  |  |  |  |  |  |  |  |  |  |  |  |  |  |  |  |  |  |  |  |  |  |  |  |  |  |  |  |  |  |  |  |  |  |  |  |  |  |  |  |  |  |  |  |  |  |  |  |  |  |  |  |  |  |  |  |  |  |  |  |  |  |  |  |  |  |  |  |  |  |  |  |  |  |  |  |  |  |  |  |  |  |  |  |  |  |  |  |  |  |  |  |  |  |  |  |  |  |  |  |  |  |  |  |  |  |  |  |  |  |  |  |  |  |  |  |  |  |  |  |  |  |  |  |  |  |  |  |  |  |  |  |  |  |  |  |  |  |  |  |  |  |  |  |  |  |  |  |  |  |  |  |  |  |  |  |  |  |  |  |  |  |  |  |  |  |  |  |  |  |  |  |  |  |  |  |  |  |  |  |  |  |  |  |  |  |  |  |  |  |  |  |  |  |  |  |  |  |  |  |  |  |  |  |  |  |  |  |  |  |  |  |  |  |  |  |  |  |  |  |  |  |  |  |  |  |  |  |  |  |  |  |  |  |  |  |  |  |  |  |  |  |  |  |  |  |  |  |  |  |  |  |  |  |  |  |  |  |  |  |  |  |  |  |  |  |  |  |  |  |  |  |  |  |  |  |  |  |  |  |  |  |  |  |  |  |  |  |  |  |  |  |  |  |  |  |  |  |  |  |  |  |  |  |  |  |  |  |  |  |  |  |  |  |  |  |  |  |  |  |  |  |  |  |  |  |  |  |  |  |  |  |  |  |  |  |  |  |  |  |  |  |  |  |  |  |  |  |  |  |  |  |  |  |  |  |  |  |  |  |  |  |  |  |  |  |  |  |  |  |  |  |  |  |  |  |  |  |  |  |  |  |  |  |  |  |  |  |  |  |  |  |  |  |  |  |  |  |  |  |  |  |  |  |  |  |  |  |  |  |  |  |  |  |  |  |  |  |  |  |  |  |  |  |  |  |  |  |  |  |  |  |  |  |  |  |  |  |  |  |  |  |  |  |  |  |  |  |  |  |  |  |  |  |  |  |  |  |  |  |  |  |  |  |  |  |  |  |  |  |  |  |  |  |  |  |  |  |  |  |  |  |  |  |  |  |  |  |  |  |
| --- | --- | --- | --- | --- | --- | --- | --- | --- | --- | --- | --- | --- | --- | --- | --- | --- | --- | --- | --- | --- | --- | --- | --- | --- | --- | --- | --- | --- | --- | --- | --- | --- | --- | --- | --- | --- | --- | --- | --- | --- | --- | --- | --- | --- | --- | --- | --- | --- | --- | --- | --- | --- | --- | --- | --- | --- | --- | --- | --- | --- | --- | --- | --- | --- | --- | --- | --- | --- | --- | --- | --- | --- | --- | --- | --- | --- | --- | --- | --- | --- | --- | --- | --- | --- | --- | --- | --- | --- | --- | --- | --- | --- | --- | --- | --- | --- | --- | --- | --- | --- | --- | --- | --- | --- | --- | --- | --- | --- | --- | --- | --- | --- | --- | --- | --- | --- | --- | --- | --- | --- | --- | --- | --- | --- | --- | --- | --- | --- | --- | --- | --- | --- | --- | --- | --- | --- | --- | --- | --- | --- | --- | --- | --- | --- | --- | --- | --- | --- | --- | --- | --- | --- | --- | --- | --- | --- | --- | --- | --- | --- | --- | --- | --- | --- | --- | --- | --- | --- | --- | --- | --- | --- | --- | --- | --- | --- | --- | --- | --- | --- | --- | --- | --- | --- | --- | --- | --- | --- | --- | --- | --- | --- | --- | --- | --- | --- | --- | --- | --- | --- | --- | --- | --- | --- | --- | --- | --- | --- | --- | --- | --- | --- | --- | --- | --- | --- | --- | --- | --- | --- | --- | --- | --- | --- | --- | --- | --- | --- | --- | --- | --- | --- | --- | --- | --- | --- | --- | --- | --- | --- | --- | --- | --- | --- | --- | --- | --- | --- | --- | --- | --- | --- | --- | --- | --- | --- | --- | --- | --- | --- | --- | --- | --- | --- | --- | --- | --- | --- | --- | --- | --- | --- | --- | --- | --- | --- | --- | --- | --- | --- | --- | --- | --- | --- | --- | --- | --- | --- | --- | --- | --- | --- | --- | --- | --- | --- | --- | --- | --- | --- | --- | --- | --- | --- | --- | --- | --- | --- | --- | --- | --- | --- | --- | --- | --- | --- | --- | --- | --- | --- | --- | --- | --- | --- | --- | --- | --- | --- | --- | --- | --- | --- | --- | --- | --- | --- | --- | --- | --- | --- | --- | --- | --- | --- | --- | --- | --- | --- | --- | --- | --- | --- | --- | --- | --- | --- | --- | --- | --- | --- | --- | --- | --- | --- | --- | --- | --- | --- | --- | --- | --- | --- | --- | --- | --- | --- | --- | --- | --- | --- | --- | --- | --- | --- | --- | --- | --- | --- | --- | --- | --- | --- | --- | --- | --- | --- | --- | --- | --- | --- | --- | --- | --- | --- | --- | --- | --- | --- | --- | --- | --- | --- | --- | --- | --- | --- | --- | --- | --- | --- | --- | --- | --- | --- | --- | --- | --- | --- | --- | --- | --- | --- | --- | --- | --- | --- | --- | --- | --- | --- | --- | --- | --- | --- | --- | --- | --- | --- | --- | --- | --- | --- | --- | --- | --- | --- | --- | --- | --- | --- | --- | --- | --- | --- | --- | --- | --- | --- | --- | --- | --- | --- | --- | --- | --- | --- | --- | --- | --- | --- | --- | --- | --- | --- | --- | --- | --- | --- | --- | --- | --- | --- | --- | --- | --- | --- | --- | --- | --- | --- | --- | --- | --- | --- | --- | --- | --- | --- | --- | --- | --- | --- | --- | --- | --- | --- | --- | --- | --- | --- | --- | --- | --- | --- | --- | --- | --- | --- | --- | --- | --- | --- | --- | --- | --- | --- | --- | --- | --- | --- | --- | --- | --- | --- | --- | --- | --- | --- | --- | --- | --- | --- | --- | --- | --- | --- | --- | --- | --- | --- | --- | --- | --- | --- | --- | --- | --- | --- | --- | --- | --- | --- | --- | --- | --- | --- | --- | --- | --- | --- | --- | --- | --- | --- | --- | --- | --- | --- | --- | --- | --- | --- | --- | --- | --- | --- | --- | --- | --- | --- | --- | --- | --- | --- | --- | --- | --- | --- | --- | --- | --- | --- | --- | --- | --- | --- | --- | --- | --- | --- | --- | --- | --- | --- | --- | --- | --- | --- | --- | --- | --- | --- | --- | --- | --- | --- | --- | --- | --- | --- | --- | --- | --- | --- | --- | --- | --- | --- | --- | --- | --- | --- | --- | --- | --- | --- | --- | --- | --- | --- | --- | --- | --- | --- | --- | --- | --- | --- | --- | --- | --- | --- | --- | --- | --- | --- | --- | --- | --- | --- | --- | --- | --- | --- | --- | --- | --- | --- | --- | --- | --- | --- | --- | --- | --- | --- | --- | --- | --- | --- | --- | --- | --- | --- | --- | --- | --- | --- | --- | --- | --- | --- | --- | --- | --- | --- | --- | --- | --- | --- | --- | --- | --- | --- | --- | --- | --- | --- | --- | --- | --- | --- | --- | --- | --- | --- | --- | --- | --- | --- | --- | --- | --- | --- | --- | --- | --- | --- | --- | --- | --- | --- | --- | --- | --- | --- | --- | --- | --- | --- | --- | --- | --- | --- | --- | --- | --- | --- | --- | --- | --- | --- | --- | --- | --- | --- | --- | --- | --- | --- | --- | --- | --- | --- | --- | --- | --- | --- | --- | --- | --- | --- | --- | --- | --- | --- | --- | --- | --- | --- | --- | --- | --- | --- | --- | --- | --- | --- | --- | --- | --- | --- | --- | --- | --- | --- | --- | --- | --- | --- | --- | --- | --- | --- | --- | --- | --- | --- | --- | --- | --- | --- | --- | --- | --- | --- | --- | --- | --- | --- | --- | --- | --- | --- | --- | --- | --- | --- | --- | --- | --- | --- | --- | --- | --- | --- | --- | --- | --- | --- | --- | --- | --- | --- | --- | --- | --- | --- | --- | --- | --- | --- | --- | --- | --- | --- | --- | --- | --- | --- | --- | --- | --- | --- | --- | --- | --- | --- | --- | --- | --- | --- | --- | --- | --- | --- | --- | --- | --- | --- | --- | --- | --- | --- | --- | --- | --- | --- | --- | --- | --- | --- | --- | --- | --- | --- | --- | --- | --- | --- | --- | --- | --- | --- | --- | --- | --- | --- | --- | --- | --- | --- | --- | --- | --- | --- | --- | --- | --- | --- | --- | --- | --- | --- | --- | --- | --- | --- | --- | --- | --- | --- | --- | --- | --- | --- | --- | --- | --- | --- | --- | --- | --- | --- | --- | --- | --- | --- | --- | --- | --- | --- | --- | --- | --- | --- | --- | --- | --- | --- | --- | --- | --- | --- | --- | --- | --- | --- | --- | --- | --- | --- | --- | --- | --- | --- | --- | --- | --- | --- | --- | --- | --- | --- | --- | --- | --- | --- | --- | --- | --- | --- | --- | --- | --- | --- | --- | --- | --- | --- | --- | --- | --- | --- | --- | --- | --- | --- | --- | --- | --- | --- | --- | --- | --- | --- | --- | --- | --- | --- | --- | --- | --- | --- | --- | --- | --- | --- | --- | --- | --- | --- | --- | --- | --- | --- | --- | --- | --- | --- | --- | --- | --- | --- | --- | --- | --- | --- | --- | --- | --- | --- | --- | --- | --- | --- | --- | --- | --- | --- | --- | --- | --- | --- | --- | --- | --- | --- | --- | --- | --- | --- | --- | --- | --- | --- | --- | --- | --- | --- | --- | --- | --- | --- | --- | --- | --- | --- | --- | --- | --- | --- | --- | --- | --- | --- | --- | --- | --- | --- | --- | --- | --- | --- | --- | --- | --- | --- | --- | --- | --- | --- | --- | --- | --- | --- | --- | --- | --- | --- | --- | --- | --- | --- | --- | --- | --- | --- | --- | --- | --- | --- | --- | --- | --- | --- | --- | --- | --- | --- | --- | --- | --- | --- | --- | --- | --- | --- | --- | --- | --- | --- | --- | --- | --- | --- | --- | --- | --- | --- | --- | --- | --- | --- | --- | --- | --- | --- | --- | --- | --- | --- | --- | --- | --- | --- | --- | --- | --- | --- | --- | --- | --- | --- | --- | --- | --- | --- | --- | --- | --- | --- | --- | --- | --- | --- | --- | --- | --- | --- | --- | --- | --- | --- | --- | --- | --- | --- | --- | --- | --- | --- | --- | --- | --- | --- | --- | --- | --- | --- | --- | --- | --- | --- | --- | --- | --- | --- | --- | --- | --- | --- | --- | --- | --- | --- | --- | --- | --- | --- | --- | --- | --- | --- | --- | --- | --- | --- | --- | --- | --- | --- | --- | --- | --- | --- | --- | --- | --- | --- | --- | --- | --- | --- | --- | --- | --- | --- | --- | --- | --- | --- | --- | --- | --- | --- | --- | --- | --- | --- | --- | --- | --- | --- | --- | --- | --- | --- | --- | --- | --- | --- | --- | --- | --- | --- | --- | --- | --- | --- | --- | --- | --- | --- | --- | --- | --- | --- | --- | --- | --- | --- | --- | --- | --- | --- | --- | --- | --- | --- | --- | --- | --- | --- | --- | --- | --- | --- | --- | --- | --- | --- | --- | --- | --- | --- | --- | --- | --- | --- | --- | --- | --- | --- | --- | --- | --- | --- | --- | --- | --- | --- | --- | --- | --- | --- | --- | --- | --- | --- | --- | --- | --- | --- | --- | --- | --- | --- | --- | --- | --- | --- | --- | --- | --- | --- | --- | --- | --- | --- | --- | --- | --- | --- | --- | --- | --- | --- | --- | --- | --- | --- | --- | --- | --- | --- | --- | --- | --- | --- | --- | --- | --- | --- | --- | --- | --- | --- | --- | --- | --- | --- | --- | --- | --- | --- | --- | --- | --- | --- | --- | --- | --- | --- | --- | --- | --- | --- | --- | --- | --- | --- | --- | --- | --- | --- | --- | --- | --- | --- | --- | --- | --- | --- | --- | --- | --- | --- | --- | --- | --- | --- | --- | --- | --- | --- | --- | --- | --- | --- | --- | --- | --- | --- | --- | --- | --- | --- | --- | --- | --- | --- | --- | --- | --- | --- | --- | --- | --- | --- | --- | --- | --- | --- | --- | --- | --- | --- | --- | --- | --- | --- | --- | --- | --- | --- | --- | --- | --- | --- | --- | --- | --- | --- | --- | --- | --- | --- | --- | --- | --- | --- | --- | --- | --- | --- | --- | --- | --- | --- | --- | --- | --- | --- | --- | --- | --- | --- | --- | --- | --- | --- | --- | --- | --- | --- | --- | --- | --- | --- | --- | --- | --- | --- | --- | --- | --- | --- | --- | --- | --- | --- | --- | --- | --- | --- | --- | --- | --- | --- | --- | --- | --- | --- | --- | --- | --- | --- | --- | --- | --- | --- | --- | --- | --- | --- | --- | --- | --- | --- | --- | --- | --- | --- | --- | --- | --- | --- | --- | --- | --- | --- | --- | --- | --- | --- | --- | --- | --- | --- | --- | --- | --- | --- | --- | --- | --- | --- | --- | --- | --- | --- | --- | --- | --- | --- | --- | --- | --- | --- | --- | --- | --- | --- | --- | --- | --- | --- | --- | --- | --- | --- | --- | --- | --- | --- | --- | --- | --- | --- | --- | --- | --- | --- | --- | --- | --- | --- | --- | --- | --- | --- | --- | --- | --- | --- | --- | --- | --- | --- | --- | --- | --- | --- | --- | --- | --- | --- | --- | --- | --- | --- | --- | --- | --- | --- | --- | --- | --- | --- | --- | --- | --- | --- | --- | --- | --- | --- | --- | --- | --- | --- | --- | --- | --- | --- | --- | --- | --- | --- | --- | --- | --- | --- | --- | --- | --- | --- | --- | --- | --- | --- | --- | --- | --- | --- | --- | --- | --- | --- | --- | --- | --- | --- | --- | --- | --- | --- | --- | --- | --- | --- | --- | --- | --- | --- | --- | --- | --- | --- | --- | --- | --- | --- | --- | --- | --- | --- | --- | --- | --- | --- | --- | --- | --- | --- | --- | --- | --- | --- | --- | --- | --- | --- | --- | --- | --- | --- | --- | --- | --- | --- | --- | --- | --- | --- | --- | --- | --- | --- | --- | --- | --- | --- | --- | --- | --- | --- | --- | --- | --- | --- | --- | --- | --- | --- | --- | --- | --- | --- | --- | --- | --- | --- | --- | --- | --- | --- | --- | --- | --- | --- | --- | --- | --- | --- | --- | --- | --- | --- | --- | --- | --- | --- | --- | --- | --- | --- | --- | --- | --- | --- | --- | --- | --- | --- | --- | --- | --- | --- | --- | --- | --- | --- | --- | --- | --- | --- | --- | --- | --- | --- | --- | --- | --- | --- | --- | --- | --- | --- | --- | --- | --- | --- | --- | --- | --- | --- | --- | --- | --- | --- | --- | --- | --- | --- | --- | --- | --- | --- | --- | --- | --- | --- | --- | --- | --- | --- | --- | --- | --- | --- | --- | --- | --- | --- | --- | --- | --- | --- | --- | --- | --- | --- | --- | --- | --- | --- | --- | --- | --- | --- | --- | --- | --- | --- | --- | --- | --- | --- | --- | --- | --- | --- | --- | --- | --- | --- | --- | --- | --- | --- | --- | --- | --- | --- | --- | --- | --- | --- | --- | --- | --- | --- | --- | --- | --- | --- | --- | --- | --- | --- | --- | --- | --- | --- | --- | --- | --- | --- | --- | --- | --- | --- | --- | --- | --- | --- | --- | --- | --- | --- | --- | --- | --- | --- | --- | --- | --- | --- | --- | --- | --- | --- | --- | --- | --- | --- | --- | --- | --- | --- | --- | --- | --- | --- | --- | --- | --- | --- | --- | --- | --- | --- | --- | --- | --- | --- | --- | --- | --- | --- | --- | --- | --- | --- | --- | --- | --- | --- | --- | --- | --- | --- | --- | --- | --- | --- | --- | --- | --- | --- | --- | --- | --- | --- | --- | --- | --- | --- | --- | --- | --- | --- | --- | --- | --- | --- | --- | --- | --- | --- | --- | --- | --- | --- | --- | --- | --- | --- | --- | --- | --- | --- | --- | --- | --- | --- | --- | --- | --- | --- | --- | --- | --- | --- | --- | --- | --- | --- | --- | --- | --- | --- | --- | --- | --- | --- | --- | --- | --- | --- | --- | --- | --- | --- | --- | --- | --- | --- | --- | --- | --- | --- | --- | --- | --- | --- | --- | --- | --- | --- | --- | --- | --- | --- | --- | --- | --- | --- | --- | --- | --- | --- | --- | --- | --- | --- | --- | --- | --- | --- | --- | --- | --- | --- | --- | --- | --- | --- | --- | --- | --- | --- | --- | --- | --- | --- | --- | --- | --- | --- | --- | --- | --- | --- | --- | --- | --- | --- | --- | --- | --- | --- | --- | --- | --- | --- | --- | --- | --- | --- | --- | --- | --- | --- | --- | --- | --- | --- | --- | --- | --- | --- | --- | --- | --- | --- | --- | --- | --- | --- | --- | --- | --- | --- | --- | --- | --- | --- | --- | --- | --- | --- | --- | --- | --- | --- | --- | --- | --- | --- | --- | --- | --- | --- | --- | --- | --- | --- | --- | --- | --- | --- | --- | --- | --- | --- | --- | --- | --- | --- | --- | --- | --- | --- | --- | --- | --- | --- | --- | --- | --- | --- | --- | --- | --- | --- | --- | --- | --- | --- | --- | --- | --- | --- | --- | --- | --- | --- | --- | --- | --- | --- | --- | --- | --- | --- | --- | --- | --- | --- | --- | --- | --- | --- | --- | --- | --- | --- | --- | --- | --- | --- | --- | --- | --- | --- | --- | --- | --- | --- | --- | --- | --- | --- | --- | --- | --- | --- | --- | --- | --- | --- | --- | --- | --- | --- | --- | --- | --- | --- | --- | --- | --- | --- | --- | --- | --- | --- | --- | --- | --- | --- | --- | --- | --- | --- | --- | --- | --- | --- | --- | --- | --- | --- | --- | --- | --- | --- | --- | --- | --- | --- | --- | --- | --- | --- | --- | --- | --- | --- | --- | --- | --- | --- | --- | --- | --- | --- | --- | --- | --- | --- | --- | --- | --- | --- | --- | --- | --- | --- | --- | --- | --- | --- | --- | --- | --- | --- | --- | --- | --- | --- | --- | --- | --- | --- | --- | --- | --- | --- | --- | --- | --- | --- | --- | --- | --- | --- | --- | --- | --- | --- | --- | --- | --- | --- | --- | --- | --- | --- | --- | --- | --- | --- | --- | --- | --- | --- | --- | --- | --- | --- | --- | --- | --- | --- | --- | --- | --- | --- | --- | --- | --- | --- | --- | --- | --- | --- | --- | --- | --- | --- | --- | --- | --- | --- | --- | --- | --- | --- | --- | --- | --- | --- | --- | --- | --- | --- | --- | --- | --- | --- | --- | --- | --- | --- | --- | --- | --- | --- | --- | --- | --- | --- | --- | --- | --- | --- | --- | --- | --- | --- | --- | --- | --- | --- | --- | --- | --- | --- | --- | --- | --- | --- | --- | --- | --- | --- | --- | --- | --- | --- | --- | --- | --- | --- | --- | --- | --- | --- | --- | --- | --- | --- | --- | --- | --- | --- | --- | --- | --- | --- | --- | --- | --- | --- | --- | --- | --- | --- | --- | --- | --- | --- | --- | --- | --- | --- | --- | --- | --- | --- | --- | --- | --- | --- | --- | --- | --- | --- | --- | --- | --- | --- | --- | --- | --- | --- | --- | --- | --- | --- | --- | --- | --- | --- | --- | --- | --- | --- | --- | --- | --- | --- | --- | --- | --- | --- | --- | --- | --- | --- | --- | --- | --- | --- | --- | --- | --- | --- | --- | --- | --- | --- | --- | --- | --- | --- | --- | --- | --- | --- | --- | --- | --- | --- | --- | --- | --- | --- | --- | --- | --- | --- | --- | --- | --- | --- | --- | --- | --- | --- | --- | --- | --- | --- | --- | --- | --- | --- | --- | --- | --- | --- | --- | --- | --- | --- | --- | --- | --- | --- | --- | --- | --- | --- | --- | --- | --- | --- | --- | --- | --- | --- | --- | --- | --- | --- | --- | --- | --- | --- | --- | --- | --- | --- | --- | --- | --- | --- | --- | --- | --- | --- | --- | --- | --- | --- | --- | --- | --- | --- | --- | --- | --- | --- | --- | --- | --- | --- | --- | --- | --- | --- | --- | --- | --- | --- | --- | --- | --- | --- | --- | --- | --- | --- | --- | --- | --- | --- | --- | --- | --- | --- | --- | --- | --- | --- | --- | --- | --- | --- | --- | --- | --- | --- | --- | --- | --- | --- | --- | --- | --- | --- | --- | --- | --- | --- | --- | --- | --- | --- | --- | --- | --- | --- | --- | --- | --- | --- | --- | --- | --- | --- | --- | --- | --- | --- | --- | --- | --- | --- | --- | --- | --- | --- | --- | --- | --- | --- | --- | --- | --- | --- | --- | --- | --- | --- | --- | --- | --- | --- | --- | --- | --- | --- | --- | --- | --- | --- | --- | --- | --- | --- | --- | --- | --- | --- | --- | --- | --- | --- | --- | --- | --- | --- | --- | --- | --- | --- | --- | --- | --- | --- | --- | --- | --- | --- | --- | --- | --- | --- | --- | --- | --- | --- | --- | --- | --- | --- | --- | --- | --- | --- | --- | --- | --- | --- | --- | --- | --- | --- | --- | --- | --- | --- | --- | --- | --- | --- | --- | --- | --- | --- | --- | --- | --- | --- | --- | --- | --- | --- | --- | --- | --- | --- | --- | --- | --- | --- | --- | --- | --- | --- | --- | --- | --- | --- | --- | --- | --- | --- | --- | --- | --- | --- | --- | --- | --- | --- | --- | --- | --- | --- | --- | --- | --- | --- | --- | --- | --- | --- | --- | --- | --- | --- | --- | --- | --- | --- | --- | --- | --- | --- | --- | --- | --- | --- | --- | --- | --- | --- | --- | --- | --- | --- | --- | --- | --- | --- | --- | --- | --- | --- | --- | --- | --- | --- | --- | --- | --- | --- | --- | --- | --- | --- | --- | --- | --- | --- | --- | --- | --- | --- | --- | --- | --- | --- | --- | --- | --- | --- | --- | --- | --- | --- | --- | --- | --- | --- | --- | --- | --- | --- | --- | --- | --- | --- | --- | --- | --- | --- | --- | --- | --- | --- | --- | --- | --- | --- | --- | --- | --- | --- | --- | --- | --- | --- | --- | --- | --- | --- | --- | --- | --- | --- | --- | --- | --- | --- | --- | --- | --- | --- | --- | --- | --- | --- | --- | --- | --- | --- | --- | --- | --- | --- | --- | --- | --- | --- | --- | --- | --- | --- | --- | --- | --- | --- | --- | --- | --- | --- | --- | --- | --- | --- | --- | --- | --- | --- | --- | --- | --- | --- | --- | --- | --- | --- | --- | --- | --- | --- | --- | --- | --- | --- | --- | --- | --- | --- | --- | --- | --- | --- | --- | --- | --- | --- | --- | --- | --- | --- | --- | --- | --- | --- | --- | --- | --- | --- | --- | --- | --- | --- | --- | --- | --- | --- | --- | --- | --- | --- | --- | --- | --- | --- | --- | --- | --- | --- | --- | --- | --- | --- | --- | --- | --- | --- | --- | --- | --- | --- | --- | --- | --- | --- | --- | --- | --- | --- | --- | --- | --- | --- | --- | --- | --- | --- | --- | --- | --- | --- | --- | --- | --- | --- | --- | --- | --- | --- | --- | --- | --- | --- | --- | --- | --- | --- | --- | --- | --- | --- | --- | --- | --- | --- | --- | --- | --- | --- | --- | --- | --- | --- | --- | --- | --- | --- | --- | --- | --- | --- | --- | --- | --- | --- | --- | --- | --- | --- | --- | --- | --- | --- | --- | --- | --- | --- | --- | --- | --- | --- | --- | --- | --- | --- | --- | --- | --- | --- | --- | --- | --- | --- | --- | --- | --- | --- | --- | --- | --- | --- | --- | --- | --- | --- | --- | --- | --- | --- | --- | --- | --- | --- | --- | --- | --- | --- | --- | --- | --- | --- | --- | --- | --- | --- | --- | --- | --- | --- | --- | --- | --- | --- | --- | --- | --- | --- | --- | --- | --- | --- | --- | --- | --- | --- | --- | --- | --- | --- | --- | --- | --- | --- | --- | --- | --- | --- | --- | --- | --- | --- | --- | --- | --- | --- | --- | --- | --- | --- | --- | --- | --- | --- | --- | --- | --- | --- | --- | --- | --- | --- | --- | --- | --- | --- | --- | --- | --- | --- | --- | --- | --- | --- | --- | --- | --- | --- | --- | --- | --- | --- | --- | --- | --- | --- | --- | --- | --- | --- | --- | --- | --- | --- | --- | --- | --- | --- | --- | --- | --- | --- | --- |
| Actual | Predict  |  |  |  |  |  |  |  |  |  |  |  |  |  |  |  |  |  |  |  |  |  |  |  |  |  |  |  |  |  |  |  |  |  |  |  |  |  |  |  |  |  |  |  |  |  |  |  |  |  |  |  |  |  |  |  |  |  |  | | --- | --- | --- | --- | --- | --- | --- | --- | --- | --- | --- | --- | --- | --- | --- | --- | --- | --- | --- | --- | --- | --- | --- | --- | --- | --- | --- | --- | --- | --- | --- | --- | --- | --- | --- | --- | --- | --- | --- | --- | --- | --- | --- | --- | --- | --- | --- | --- | --- | --- | --- | --- | --- | --- | --- | --- | --- | --- | |  | 1.1 | 1.10 | 1.11 | 1.13 | 1.14 | 1.15 | 1.16 | 1.17 | 1.18 | 1.2 | 1.3 | 1.4 | 1.5 | 1.6 | 1.7 | 1.8 | 1.9 | 1.97 | 2.1 | 2.2 | 2.3 | 2.4 | 2.5 | 2.6 | 2.7 | 2.8 | 2.9 | 3.1 | 3.11 | 3.2 | 3.3 | 3.4 | 3.5 | 3.6 | 3.7 | 4.1 | 4.2 | 4.3 | 4.4 | 4.6 | 4.99 | 5.1 | 5.2 | 5.3 | 5.4 | 5.5 | 5.6 | 6.1 | 6.2 | 6.3 | 6.5 | 7.1 | 7.2 | 7.3 | 7.4 | 7.5 | 7.6 | | 1.1 | 214 | 0 | 0 | 0 | 0 | 0 | 0 | 0 | 0 | 1 | 1 | 0 | 0 | 0 | 0 | 0 | 0 | 0 | 5 | 0 | 4 | 0 | 0 | 0 | 6 | 0 | 0 | 0 | 0 | 73 | 0 | 0 | 0 | 1 | 0 | 0 | 1 | 0 | 0 | 0 | 0 | 0 | 0 | 0 | 0 | 0 | 0 | 0 | 0 | 1 | 0 | 0 | 1 | 0 | 0 | 0 | 0 | | 1.10 | 0 | 43 | 1 | 0 | 0 | 0 | 0 | 0 | 0 | 0 | 0 | 0 | 0 | 0 | 0 | 0 | 0 | 0 | 0 | 0 | 0 | 2 | 0 | 0 | 1 | 0 | 0 | 0 | 0 | 2 | 0 | 0 | 0 | 0 | 0 | 0 | 0 | 0 | 0 | 0 | 0 | 0 | 0 | 1 | 0 | 0 | 0 | 0 | 0 | 0 | 0 | 0 | 0 | 0 | 0 | 0 | 0 | | 1.11 | 0 | 0 | 49 | 0 | 0 | 0 | 0 | 0 | 0 | 0 | 0 | 0 | 0 | 0 | 0 | 0 | 0 | 0 | 0 | 0 | 3 | 0 | 0 | 0 | 7 | 0 | 0 | 0 | 0 | 0 | 0 | 0 | 0 | 0 | 0 | 0 | 2 | 0 | 0 | 0 | 0 | 0 | 0 | 0 | 0 | 0 | 0 | 0 | 0 | 0 | 0 | 0 | 0 | 0 | 0 | 0 | 0 | | 1.13 | 0 | 0 | 0 | 55 | 0 | 0 | 0 | 0 | 0 | 0 | 0 | 0 | 0 | 0 | 0 | 0 | 0 | 0 | 0 | 0 | 1 | 0 | 0 | 0 | 0 | 0 | 0 | 0 | 0 | 0 | 0 | 0 | 0 | 0 | 0 | 0 | 0 | 0 | 0 | 0 | 0 | 0 | 0 | 0 | 0 | 0 | 0 | 0 | 0 | 0 | 0 | 0 | 0 | 0 | 0 | 0 | 0 | | 1.14 | 0 | 0 | 0 | 0 | 58 | 0 | 0 | 0 | 0 | 0 | 0 | 1 | 0 | 0 | 0 | 0 | 0 | 0 | 0 | 0 | 4 | 0 | 0 | 0 | 0 | 0 | 0 | 0 | 0 | 0 | 0 | 0 | 0 | 0 | 0 | 0 | 1 | 0 | 0 | 0 | 0 | 0 | 0 | 0 | 0 | 0 | 0 | 0 | 0 | 0 | 0 | 0 | 0 | 0 | 0 | 0 | 0 | | 1.15 | 0 | 0 | 0 | 0 | 0 | 24 | 0 | 0 | 0 | 0 | 0 | 0 | 0 | 0 | 0 | 0 | 0 | 0 | 0 | 0 | 0 | 0 | 0 | 0 | 0 | 0 | 0 | 0 | 0 | 0 | 0 | 2 | 0 | 0 | 0 | 0 | 0 | 0 | 0 | 0 | 0 | 0 | 0 | 0 | 0 | 0 | 0 | 0 | 0 | 0 | 0 | 0 | 0 | 0 | 0 | 0 | 0 | | 1.16 | 0 | 0 | 0 | 0 | 0 | 0 | 0 | 0 | 0 | 0 | 0 | 0 | 0 | 0 | 0 | 0 | 0 | 0 | 1 | 0 | 0 | 0 | 0 | 0 | 0 | 0 | 0 | 0 | 0 | 5 | 0 | 0 | 0 | 0 | 0 | 0 | 0 | 0 | 0 | 0 | 0 | 0 | 0 | 0 | 0 | 0 | 0 | 0 | 0 | 0 | 0 | 0 | 0 | 0 | 0 | 0 | 0 | | 1.17 | 0 | 0 | 0 | 0 | 0 | 0 | 0 | 59 | 0 | 0 | 0 | 0 | 0 | 0 | 0 | 0 | 0 | 0 | 3 | 0 | 0 | 0 | 0 | 0 | 0 | 0 | 0 | 0 | 0 | 0 | 0 | 0 | 0 | 0 | 0 | 3 | 0 | 0 | 0 | 0 | 0 | 2 | 0 | 0 | 0 | 0 | 0 | 0 | 0 | 6 | 0 | 0 | 0 | 0 | 0 | 0 | 0 | | 1.18 | 5 | 0 | 0 | 0 | 0 | 0 | 0 | 0 | 20 | 1 | 2 | 0 | 0 | 0 | 0 | 0 | 0 | 0 | 0 | 1 | 2 | 0 | 0 | 0 | 0 | 0 | 0 | 11 | 0 | 0 | 0 | 0 | 0 | 0 | 0 | 0 | 0 | 0 | 0 | 0 | 0 | 0 | 0 | 0 | 0 | 0 | 0 | 0 | 0 | 0 | 0 | 0 | 0 | 0 | 0 | 0 | 0 | | 1.2 | 1 | 0 | 0 | 0 | 0 | 0 | 0 | 0 | 0 | 116 | 0 | 0 | 1 | 0 | 0 | 0 | 0 | 0 | 0 | 6 | 3 | 1 | 0 | 1 | 15 | 0 | 0 | 1 | 0 | 0 | 0 | 2 | 0 | 3 | 0 | 0 | 3 | 2 | 0 | 0 | 0 | 5 | 0 | 0 | 0 | 0 | 0 | 0 | 0 | 0 | 0 | 0 | 0 | 0 | 0 | 0 | 0 | | 1.3 | 25 | 0 | 0 | 0 | 0 | 0 | 0 | 0 | 0 | 0 | 81 | 0 | 1 | 0 | 0 | 0 | 0 | 0 | 0 | 0 | 0 | 1 | 5 | 0 | 1 | 0 | 0 | 0 | 0 | 0 | 0 | 0 | 0 | 1 | 0 | 0 | 0 | 0 | 0 | 7 | 0 | 2 | 0 | 0 | 0 | 0 | 0 | 1 | 0 | 0 | 0 | 0 | 0 | 0 | 0 | 0 | 0 | | 1.4 | 0 | 0 | 0 | 0 | 0 | 0 | 0 | 0 | 0 | 0 | 0 | 2 | 0 | 0 | 0 | 0 | 0 | 0 | 0 | 0 | 8 | 0 | 0 | 99 | 1 | 0 | 0 | 0 | 0 | 3 | 0 | 1 | 0 | 0 | 0 | 0 | 4 | 0 | 0 | 0 | 0 | 2 | 0 | 0 | 0 | 0 | 0 | 0 | 0 | 3 | 0 | 0 | 0 | 0 | 0 | 0 | 0 | | 1.5 | 0 | 0 | 0 | 0 | 0 | 0 | 0 | 0 | 0 | 0 | 0 | 0 | 13 | 2 | 0 | 0 | 0 | 0 | 0 | 0 | 1 | 0 | 0 | 0 | 0 | 0 | 0 | 0 | 0 | 0 | 0 | 0 | 0 | 0 | 0 | 1 | 1 | 0 | 0 | 0 | 0 | 0 | 0 | 0 | 0 | 0 | 0 | 0 | 0 | 30 | 0 | 0 | 0 | 0 | 0 | 0 | 0 | | 1.6 | 0 | 0 | 0 | 0 | 0 | 0 | 0 | 0 | 0 | 0 | 0 | 0 | 0 | 13 | 1 | 0 | 0 | 0 | 0 | 0 | 3 | 0 | 0 | 0 | 1 | 0 | 0 | 3 | 0 | 0 | 0 | 0 | 1 | 0 | 0 | 0 | 2 | 0 | 0 | 0 | 0 | 0 | 0 | 0 | 0 | 0 | 0 | 0 | 0 | 0 | 0 | 0 | 0 | 0 | 0 | 0 | 0 | | 1.7 | 1 | 0 | 0 | 0 | 0 | 0 | 0 | 0 | 0 | 0 | 0 | 0 | 0 | 0 | 49 | 0 | 0 | 0 | 0 | 0 | 0 | 0 | 0 | 0 | 0 | 0 | 0 | 4 | 0 | 0 | 0 | 0 | 0 | 0 | 0 | 0 | 0 | 0 | 0 | 0 | 0 | 0 | 0 | 0 | 0 | 0 | 0 | 0 | 0 | 0 | 0 | 1 | 0 | 0 | 0 | 0 | 0 | | 1.8 | 0 | 0 | 0 | 0 | 0 | 0 | 0 | 0 | 0 | 0 | 0 | 0 | 0 | 0 | 0 | 30 | 0 | 0 | 0 | 0 | 0 | 0 | 1 | 0 | 1 | 0 | 0 | 3 | 0 | 0 | 0 | 0 | 0 | 1 | 0 | 0 | 0 | 0 | 1 | 0 | 0 | 0 | 0 | 0 | 0 | 0 | 0 | 0 | 0 | 0 | 0 | 0 | 0 | 0 | 0 | 0 | 0 | | 1.9 | 0 | 0 | 0 | 0 | 0 | 0 | 0 | 0 | 0 | 0 | 0 | 0 | 0 | 0 | 0 | 0 | 16 | 0 | 0 | 0 | 0 | 0 | 0 | 0 | 0 | 0 | 0 | 0 | 0 | 0 | 0 | 0 | 0 | 0 | 0 | 0 | 0 | 0 | 0 | 0 | 0 | 0 | 0 | 0 | 0 | 0 | 0 | 0 | 0 | 0 | 0 | 0 | 0 | 0 | 0 | 0 | 0 | | 1.97 | 0 | 0 | 0 | 0 | 0 | 0 | 0 | 0 | 0 | 0 | 0 | 0 | 0 | 0 | 0 | 0 | 0 | 0 | 0 | 0 | 161 | 0 | 0 | 0 | 2 | 0 | 0 | 0 | 0 | 0 | 0 | 0 | 0 | 0 | 0 | 0 | 0 | 0 | 0 | 0 | 0 | 0 | 0 | 0 | 0 | 0 | 0 | 0 | 0 | 0 | 0 | 0 | 0 | 0 | 0 | 0 | 0 | | 2.1 | 0 | 0 | 0 | 0 | 0 | 0 | 0 | 0 | 0 | 0 | 0 | 0 | 0 | 1 | 0 | 0 | 0 | 0 | 691 | 0 | 59 | 0 | 1 | 0 | 17 | 0 | 0 | 4 | 0 | 17 | 0 | 1 | 0 | 3 | 0 | 0 | 1 | 0 | 0 | 2 | 0 | 3 | 0 | 0 | 0 | 0 | 1 | 0 | 0 | 27 | 0 | 5 | 0 | 0 | 0 | 0 | 0 | | 2.2 | 0 | 0 | 0 | 0 | 0 | 0 | 0 | 0 | 0 | 1 | 0 | 0 | 0 | 0 | 0 | 0 | 0 | 0 | 0 | 122 | 1 | 0 | 0 | 0 | 1 | 0 | 0 | 4 | 0 | 1 | 0 | 0 | 0 | 0 | 0 | 0 | 0 | 0 | 0 | 0 | 0 | 1 | 0 | 0 | 0 | 0 | 0 | 0 | 0 | 0 | 0 | 0 | 0 | 0 | 0 | 0 | 0 | | 2.3 | 0 | 0 | 0 | 0 | 0 | 1 | 0 | 0 | 0 | 1 | 0 | 4 | 0 | 0 | 2 | 0 | 0 | 0 | 5 | 9 | 575 | 4 | 7 | 0 | 56 | 2 | 0 | 8 | 0 | 7 | 0 | 9 | 9 | 18 | 0 | 1 | 11 | 0 | 0 | 8 | 0 | 4 | 0 | 6 | 2 | 0 | 0 | 3 | 0 | 2 | 0 | 12 | 0 | 0 | 0 | 0 | 4 | | 2.4 | 0 | 0 | 0 | 0 | 0 | 0 | 0 | 0 | 0 | 0 | 0 | 0 | 0 | 0 | 0 | 0 | 0 | 0 | 0 | 0 | 5 | 422 | 4 | 0 | 3 | 0 | 0 | 2 | 0 | 1 | 0 | 0 | 1 | 1 | 0 | 0 | 1 | 0 | 0 | 3 | 0 | 0 | 2 | 0 | 0 | 0 | 0 | 0 | 0 | 6 | 0 | 1 | 0 | 0 | 0 | 0 | 0 | | 2.5 | 0 | 0 | 0 | 0 | 0 | 0 | 0 | 0 | 0 | 0 | 0 | 0 | 0 | 0 | 0 | 0 | 0 | 0 | 0 | 0 | 2 | 0 | 473 | 0 | 2 | 0 | 0 | 3 | 0 | 0 | 0 | 1 | 2 | 3 | 0 | 0 | 3 | 0 | 0 | 2 | 0 | 0 | 0 | 0 | 0 | 0 | 1 | 0 | 0 | 0 | 0 | 0 | 0 | 0 | 0 | 0 | 0 | | 2.6 | 0 | 0 | 0 | 0 | 0 | 0 | 0 | 0 | 0 | 0 | 7 | 0 | 0 | 0 | 0 | 0 | 0 | 0 | 0 | 0 | 0 | 0 | 0 | 69 | 0 | 0 | 0 | 0 | 0 | 4 | 0 | 2 | 0 | 0 | 1 | 0 | 5 | 0 | 0 | 0 | 0 | 1 | 0 | 0 | 1 | 0 | 0 | 0 | 0 | 0 | 0 | 0 | 0 | 0 | 0 | 0 | 0 | | 2.7 | 16 | 0 | 1 | 0 | 1 | 0 | 0 | 0 | 0 | 0 | 0 | 0 | 4 | 0 | 0 | 2 | 0 | 0 | 8 | 0 | 9 | 13 | 8 | 0 | 1622 | 0 | 0 | 15 | 0 | 18 | 0 | 3 | 2 | 21 | 0 | 0 | 15 | 10 | 0 | 4 | 2 | 4 | 0 | 1 | 0 | 0 | 0 | 2 | 1 | 53 | 1 | 2 | 3 | 0 | 1 | 0 | 1 | | 2.8 | 0 | 0 | 0 | 0 | 0 | 0 | 0 | 0 | 0 | 0 | 0 | 0 | 0 | 0 | 0 | 0 | 0 | 0 | 0 | 0 | 1 | 0 | 0 | 0 | 0 | 203 | 0 | 7 | 0 | 0 | 0 | 1 | 0 | 1 | 0 | 0 | 0 | 0 | 0 | 0 | 0 | 0 | 0 | 0 | 0 | 0 | 0 | 0 | 0 | 0 | 0 | 0 | 0 | 0 | 0 | 0 | 0 | | 2.9 | 0 | 0 | 0 | 0 | 0 | 0 | 0 | 0 | 0 | 0 | 0 | 0 | 0 | 0 | 0 | 0 | 0 | 0 | 0 | 0 | 0 | 0 | 0 | 0 | 0 | 0 | 27 | 0 | 0 | 0 | 0 | 0 | 0 | 0 | 0 | 0 | 0 | 0 | 0 | 0 | 0 | 0 | 0 | 0 | 0 | 0 | 0 | 0 | 0 | 0 | 0 | 0 | 0 | 0 | 0 | 0 | 0 | | 3.1 | 0 | 0 | 0 | 0 | 0 | 0 | 0 | 1 | 0 | 0 | 0 | 0 | 0 | 0 | 1 | 0 | 0 | 0 | 9 | 2 | 9 | 3 | 1 | 0 | 25 | 1 | 0 | 616 | 0 | 3 | 0 | 12 | 0 | 16 | 0 | 0 | 13 | 2 | 0 | 4 | 0 | 0 | 1 | 2 | 0 | 0 | 0 | 0 | 0 | 0 | 0 | 0 | 27 | 0 | 0 | 0 | 0 | | 3.11 | 0 | 0 | 0 | 0 | 0 | 0 | 0 | 0 | 0 | 0 | 0 | 0 | 0 | 0 | 0 | 0 | 0 | 0 | 0 | 0 | 0 | 0 | 0 | 0 | 0 | 0 | 0 | 0 | 8 | 0 | 0 | 0 | 0 | 0 | 0 | 0 | 0 | 0 | 0 | 0 | 0 | 0 | 0 | 0 | 0 | 0 | 0 | 0 | 0 | 0 | 0 | 0 | 0 | 0 | 0 | 0 | 0 | | 3.2 | 0 | 0 | 0 | 0 | 0 | 0 | 0 | 0 | 0 | 0 | 0 | 0 | 0 | 0 | 0 | 0 | 0 | 0 | 1 | 0 | 8 | 0 | 1 | 0 | 1 | 0 | 0 | 9 | 0 | 243 | 0 | 3 | 3 | 0 | 0 | 0 | 2 | 0 | 0 | 1 | 0 | 0 | 0 | 0 | 0 | 0 | 0 | 0 | 0 | 0 | 0 | 0 | 0 | 0 | 0 | 0 | 0 | | 3.3 | 0 | 0 | 0 | 0 | 0 | 0 | 0 | 0 | 0 | 0 | 0 | 0 | 0 | 0 | 0 | 0 | 0 | 0 | 0 | 0 | 0 | 0 | 0 | 0 | 0 | 0 | 0 | 0 | 0 | 1 | 3 | 0 | 0 | 0 | 0 | 0 | 0 | 0 | 0 | 0 | 0 | 0 | 0 | 0 | 0 | 0 | 0 | 0 | 0 | 0 | 0 | 0 | 0 | 0 | 0 | 0 | 0 | | 3.4 | 0 | 0 | 0 | 0 | 0 | 0 | 0 | 0 | 0 | 0 | 0 | 0 | 0 | 0 | 0 | 0 | 0 | 0 | 0 | 0 | 0 | 0 | 0 | 1 | 1 | 0 | 0 | 8 | 0 | 2 | 0 | 391 | 3 | 0 | 0 | 0 | 0 | 0 | 0 | 0 | 0 | 0 | 1 | 0 | 0 | 0 | 1 | 1 | 0 | 1 | 0 | 0 | 0 | 0 | 0 | 0 | 0 | | 3.5 | 0 | 0 | 0 | 0 | 0 | 0 | 0 | 3 | 2 | 0 | 0 | 0 | 0 | 0 | 0 | 0 | 1 | 0 | 2 | 0 | 4 | 75 | 0 | 1 | 22 | 0 | 0 | 29 | 0 | 8 | 0 | 7 | 491 | 0 | 0 | 2 | 7 | 7 | 0 | 42 | 0 | 2 | 10 | 0 | 0 | 0 | 0 | 0 | 0 | 1 | 2 | 3 | 0 | 0 | 0 | 0 | 0 | | 3.6 | 0 | 0 | 1 | 0 | 0 | 0 | 0 | 0 | 0 | 0 | 0 | 0 | 0 | 0 | 0 | 0 | 0 | 0 | 2 | 0 | 10 | 0 | 0 | 0 | 22 | 0 | 0 | 42 | 0 | 0 | 0 | 0 | 0 | 327 | 0 | 0 | 7 | 0 | 0 | 0 | 0 | 0 | 0 | 0 | 0 | 0 | 13 | 0 | 0 | 1 | 0 | 0 | 0 | 0 | 0 | 0 | 0 | | 3.7 | 0 | 0 | 0 | 0 | 0 | 0 | 0 | 0 | 0 | 0 | 0 | 0 | 0 | 0 | 0 | 0 | 0 | 0 | 0 | 0 | 0 | 0 | 0 | 0 | 1 | 0 | 0 | 0 | 0 | 0 | 0 | 1 | 0 | 0 | 37 | 0 | 2 | 0 | 0 | 0 | 0 | 0 | 0 | 0 | 0 | 0 | 0 | 0 | 0 | 0 | 0 | 0 | 0 | 0 | 0 | 0 | 0 | | 4.1 | 0 | 0 | 0 | 0 | 0 | 0 | 0 | 1 | 0 | 0 | 0 | 0 | 0 | 0 | 1 | 0 | 0 | 0 | 0 | 2 | 4 | 0 | 3 | 2 | 14 | 0 | 0 | 3 | 0 | 0 | 0 | 10 | 0 | 23 | 0 | 499 | 1 | 0 | 0 | 0 | 0 | 3 | 0 | 25 | 0 | 0 | 0 | 3 | 0 | 0 | 0 | 15 | 0 | 0 | 0 | 0 | 0 | | 4.2 | 5 | 0 | 0 | 0 | 1 | 0 | 0 | 2 | 0 | 0 | 1 | 0 | 0 | 0 | 3 | 0 | 0 | 0 | 20 | 1 | 1 | 0 | 4 | 2 | 2 | 0 | 0 | 2 | 0 | 3 | 0 | 1 | 1 | 1 | 0 | 4 | 202 | 15 | 0 | 0 | 0 | 0 | 4 | 51 | 0 | 0 | 1 | 0 | 0 | 0 | 0 | 0 | 0 | 0 | 0 | 0 | 0 | | 4.3 | 0 | 0 | 0 | 0 | 0 | 0 | 0 | 7 | 0 | 1 | 0 | 0 | 0 | 0 | 0 | 0 | 0 | 0 | 0 | 1 | 4 | 1 | 0 | 2 | 0 | 1 | 0 | 0 | 0 | 0 | 0 | 2 | 1 | 1 | 0 | 1 | 0 | 19 | 0 | 0 | 0 | 0 | 1 | 1 | 0 | 0 | 0 | 0 | 0 | 0 | 0 | 0 | 0 | 0 | 0 | 0 | 0 | | 4.4 | 0 | 0 | 0 | 0 | 0 | 0 | 0 | 0 | 0 | 0 | 0 | 0 | 0 | 0 | 0 | 0 | 0 | 0 | 0 | 0 | 0 | 0 | 0 | 0 | 0 | 0 | 0 | 0 | 0 | 0 | 0 | 0 | 0 | 0 | 0 | 0 | 0 | 0 | 32 | 0 | 0 | 0 | 0 | 0 | 0 | 0 | 0 | 0 | 0 | 0 | 0 | 0 | 0 | 0 | 0 | 0 | 0 | | 4.6 | 0 | 0 | 0 | 0 | 0 | 0 | 0 | 0 | 0 | 2 | 0 | 0 | 1 | 0 | 1 | 1 | 0 | 0 | 1 | 0 | 4 | 0 | 1 | 0 | 12 | 0 | 0 | 0 | 0 | 5 | 0 | 1 | 0 | 1 | 0 | 0 | 1 | 0 | 0 | 14 | 1 | 0 | 0 | 0 | 0 | 0 | 0 | 0 | 0 | 0 | 0 | 0 | 0 | 0 | 0 | 0 | 0 | | 4.99 | 0 | 0 | 0 | 0 | 0 | 0 | 0 | 0 | 0 | 0 | 0 | 0 | 0 | 0 | 0 | 0 | 0 | 0 | 1 | 0 | 0 | 2 | 0 | 0 | 0 | 0 | 0 | 0 | 0 | 1 | 0 | 0 | 0 | 0 | 0 | 0 | 1 | 0 | 0 | 0 | 45 | 0 | 0 | 0 | 0 | 0 | 0 | 0 | 0 | 0 | 0 | 0 | 0 | 0 | 0 | 0 | 0 | | 5.1 | 2 | 0 | 0 | 0 | 0 | 0 | 0 | 0 | 0 | 0 | 0 | 3 | 1 | 0 | 1 | 0 | 0 | 0 | 0 | 0 | 0 | 2 | 0 | 0 | 0 | 0 | 0 | 0 | 0 | 0 | 0 | 0 | 0 | 0 | 0 | 0 | 5 | 0 | 0 | 0 | 0 | 131 | 0 | 0 | 0 | 0 | 0 | 0 | 0 | 0 | 0 | 0 | 0 | 0 | 0 | 0 | 0 | | 5.2 | 0 | 0 | 0 | 0 | 0 | 0 | 0 | 0 | 0 | 0 | 0 | 0 | 0 | 0 | 0 | 0 | 0 | 0 | 0 | 0 | 0 | 0 | 0 | 0 | 0 | 0 | 0 | 0 | 0 | 0 | 0 | 0 | 0 | 0 | 0 | 0 | 0 | 0 | 0 | 0 | 0 | 0 | 155 | 0 | 0 | 0 | 0 | 0 | 0 | 0 | 0 | 0 | 0 | 0 | 0 | 0 | 0 | | 5.3 | 0 | 0 | 0 | 0 | 6 | 3 | 0 | 0 | 0 | 0 | 0 | 0 | 0 | 0 | 0 | 0 | 0 | 0 | 3 | 0 | 0 | 0 | 5 | 0 | 0 | 0 | 0 | 6 | 0 | 1 | 0 | 1 | 3 | 0 | 0 | 0 | 0 | 0 | 0 | 2 | 0 | 0 | 1 | 157 | 0 | 0 | 0 | 1 | 0 | 10 | 0 | 0 | 0 | 0 | 0 | 0 | 0 | | 5.4 | 6 | 0 | 0 | 0 | 0 | 0 | 0 | 0 | 0 | 0 | 0 | 0 | 0 | 0 | 0 | 0 | 0 | 0 | 0 | 5 | 3 | 0 | 0 | 1 | 5 | 0 | 0 | 13 | 0 | 4 | 0 | 7 | 4 | 0 | 0 | 3 | 5 | 0 | 0 | 0 | 0 | 0 | 0 | 0 | 270 | 0 | 0 | 0 | 0 | 1 | 7 | 0 | 0 | 0 | 0 | 0 | 0 | | 5.5 | 0 | 0 | 0 | 0 | 0 | 0 | 0 | 0 | 0 | 0 | 0 | 0 | 0 | 0 | 0 | 0 | 0 | 0 | 0 | 0 | 0 | 0 | 0 | 0 | 1 | 0 | 0 | 0 | 0 | 0 | 0 | 0 | 0 | 0 | 0 | 0 | 0 | 0 | 0 | 0 | 0 | 0 | 0 | 0 | 0 | 1 | 0 | 0 | 0 | 0 | 0 | 0 | 0 | 0 | 0 | 0 | 0 | | 5.6 | 0 | 0 | 0 | 0 | 0 | 0 | 0 | 0 | 0 | 0 | 0 | 0 | 0 | 0 | 0 | 0 | 0 | 0 | 1 | 0 | 1 | 0 | 0 | 0 | 5 | 0 | 0 | 1 | 0 | 0 | 0 | 0 | 0 | 0 | 0 | 1 | 0 | 3 | 0 | 0 | 0 | 0 | 0 | 0 | 0 | 0 | 38 | 0 | 0 | 0 | 0 | 0 | 0 | 1 | 0 | 0 | 0 | | 6.1 | 0 | 0 | 5 | 0 | 0 | 0 | 0 | 0 | 0 | 9 | 0 | 0 | 0 | 0 | 0 | 0 | 0 | 0 | 7 | 0 | 5 | 5 | 9 | 1 | 2 | 0 | 0 | 3 | 0 | 8 | 0 | 3 | 1 | 4 | 0 | 0 | 3 | 0 | 0 | 7 | 3 | 0 | 0 | 5 | 0 | 0 | 0 | 927 | 0 | 27 | 0 | 0 | 0 | 0 | 0 | 0 | 0 | | 6.2 | 0 | 0 | 0 | 0 | 0 | 0 | 0 | 0 | 0 | 22 | 0 | 0 | 0 | 0 | 0 | 0 | 0 | 0 | 5 | 0 | 2 | 0 | 0 | 0 | 0 | 0 | 0 | 4 | 0 | 0 | 0 | 5 | 1 | 0 | 0 | 0 | 0 | 0 | 0 | 0 | 0 | 0 | 0 | 2 | 0 | 0 | 0 | 0 | 14 | 0 | 0 | 0 | 0 | 0 | 0 | 0 | 0 | | 6.3 | 16 | 0 | 0 | 0 | 0 | 0 | 0 | 0 | 0 | 0 | 0 | 0 | 0 | 0 | 1 | 0 | 0 | 0 | 1 | 0 | 2 | 1 | 0 | 0 | 11 | 0 | 0 | 1 | 0 | 0 | 0 | 0 | 0 | 2 | 0 | 0 | 2 | 2 | 0 | 1 | 0 | 1 | 0 | 0 | 0 | 0 | 0 | 0 | 0 | 482 | 0 | 1 | 2 | 0 | 0 | 0 | 0 | | 6.5 | 1 | 0 | 1 | 0 | 0 | 0 | 0 | 0 | 0 | 0 | 0 | 0 | 0 | 0 | 0 | 0 | 0 | 0 | 0 | 0 | 7 | 0 | 1 | 0 | 10 | 0 | 0 | 9 | 0 | 1 | 0 | 0 | 1 | 0 | 0 | 0 | 0 | 0 | 0 | 1 | 0 | 1 | 1 | 0 | 0 | 0 | 0 | 0 | 0 | 0 | 59 | 0 | 0 | 0 | 0 | 0 | 0 | | 7.1 | 0 | 0 | 0 | 0 | 0 | 0 | 0 | 0 | 0 | 0 | 0 | 0 | 0 | 0 | 0 | 0 | 0 | 1 | 0 | 0 | 19 | 0 | 11 | 0 | 5 | 0 | 0 | 2 | 0 | 2 | 0 | 1 | 0 | 0 | 0 | 0 | 1 | 0 | 0 | 0 | 0 | 0 | 0 | 0 | 0 | 0 | 0 | 0 | 0 | 0 | 0 | 229 | 1 | 1 | 0 | 0 | 1 | | 7.2 | 0 | 0 | 0 | 0 | 0 | 0 | 0 | 0 | 0 | 0 | 0 | 0 | 0 | 0 | 0 | 0 | 0 | 0 | 0 | 0 | 10 | 1 | 0 | 0 | 4 | 0 | 0 | 1 | 0 | 0 | 0 | 0 | 0 | 15 | 0 | 0 | 4 | 0 | 0 | 12 | 0 | 0 | 0 | 0 | 0 | 0 | 0 | 0 | 0 | 0 | 0 | 2 | 32 | 0 | 0 | 0 | 1 | | 7.3 | 0 | 0 | 0 | 0 | 0 | 0 | 0 | 0 | 0 | 0 | 0 | 0 | 0 | 0 | 0 | 0 | 0 | 0 | 0 | 0 | 1 | 0 | 0 | 0 | 1 | 0 | 0 | 0 | 0 | 0 | 0 | 0 | 0 | 0 | 0 | 0 | 0 | 0 | 0 | 0 | 0 | 0 | 0 | 0 | 0 | 0 | 0 | 0 | 0 | 0 | 0 | 0 | 0 | 30 | 3 | 0 | 0 | | 7.4 | 0 | 0 | 0 | 0 | 0 | 0 | 0 | 0 | 0 | 0 | 0 | 0 | 0 | 0 | 0 | 0 | 0 | 0 | 2 | 0 | 0 | 0 | 0 | 0 | 2 | 0 | 0 | 0 | 0 | 0 | 0 | 0 | 0 | 2 | 0 | 0 | 0 | 0 | 0 | 0 | 0 | 0 | 1 | 0 | 0 | 0 | 0 | 0 | 0 | 0 | 0 | 0 | 0 | 0 | 147 | 0 | 0 | | 7.5 | 0 | 0 | 0 | 0 | 0 | 0 | 0 | 0 | 0 | 0 | 0 | 0 | 0 | 0 | 0 | 0 | 0 | 0 | 0 | 0 | 0 | 0 | 0 | 0 | 0 | 0 | 0 | 0 | 0 | 0 | 0 | 0 | 0 | 0 | 0 | 0 | 0 | 0 | 0 | 0 | 0 | 0 | 0 | 0 | 0 | 0 | 0 | 0 | 0 | 0 | 0 | 0 | 0 | 0 | 0 | 11 | 2 | | 7.6 | 0 | 0 | 0 | 0 | 1 | 0 | 0 | 0 | 0 | 0 | 0 | 0 | 0 | 0 | 0 | 0 | 0 | 0 | 1 | 0 | 2 | 1 | 1 | 0 | 1 | 0 | 0 | 0 | 0 | 0 | 0 | 0 | 0 | 1 | 0 | 0 | 0 | 0 | 0 | 0 | 0 | 1 | 0 | 0 | 0 | 0 | 0 | 0 | 0 | 0 | 0 | 0 | 0 | 5 | 0 | 3 | 26 | |

## Overall Statistics :

|  |  |
| --- | --- |
| 95% CI | (0.80137,0.81479) |
| ACC Macro | 0.99327 |
| ARI | 0.70277 |
| AUNP | 0.89953 |
| AUNU | 0.868 |
| Bangdiwala B | 0.71803 |
| Bennett S | 0.80465 |
| CBA | 0.69203 |
| CSI | None |
| Chi-Squared | None |
| Chi-Squared DF | 3136 |
| Conditional Entropy | 1.10403 |
| Cramer V | None |
| Cross Entropy | 4.95435 |
| F1 Macro | 0.74182 |
| F1 Micro | 0.80808 |
| FNR Macro | 0.26048 |
| FNR Micro | 0.19192 |
| FPR Macro | 0.00353 |
| FPR Micro | 0.00343 |
| Gwet AC1 | 0.80478 |
| Hamming Loss | 0.19192 |
| Joint Entropy | 5.94158 |
| KL Divergence | None |
| Kappa | 0.79754 |
| Kappa 95% CI | (0.79045,0.80462) |
| Kappa No Prevalence | 0.61616 |
| Kappa Standard Error | 0.00361 |
| Kappa Unbiased | 0.79747 |
| Krippendorff Alpha | 0.79748 |
| Lambda A | 0.78235 |
| Lambda B | 0.80161 |
| Mutual Information | 3.65854 |
| NIR | 0.13942 |
| Overall ACC | 0.80808 |
| Overall CEN | 0.16084 |
| Overall J | (36.35939,0.63788) |
| Overall MCC | 0.79804 |
| Overall MCEN | 0.23957 |
| Overall RACC | 0.05207 |
| Overall RACCU | 0.05237 |
| P-Value | None |
| PPV Macro | None |
| PPV Micro | 0.80808 |
| Pearson C | None |
| Phi-Squared | None |
| RCI | 0.75628 |
| RR | 231.91228 |
| Reference Entropy | 4.83755 |
| Response Entropy | 4.76257 |
| SOA1(Landis & Koch) | Substantial |
| SOA2(Fleiss) | Excellent |
| SOA3(Altman) | Good |
| SOA4(Cicchetti) | Excellent |
| SOA5(Cramer) | None |
| SOA6(Matthews) | Strong |
| Scott PI | 0.79747 |
| Standard Error | 0.00343 |
| TNR Macro | 0.99647 |
| TNR Micro | 0.99657 |
| TPR Macro | 0.73952 |
| TPR Micro | 0.80808 |
| Zero-one Loss | 2537 |

## Class Statistics :

|  |  |  |  |  |  |  |  |  |  |  |  |  |  |  |  |  |  |  |  |  |  |  |  |  |  |  |  |  |  |  |  |  |  |  |  |  |  |  |  |  |  |  |  |  |  |  |  |  |  |  |  |  |  |  |  |  |  |  |
| --- | --- | --- | --- | --- | --- | --- | --- | --- | --- | --- | --- | --- | --- | --- | --- | --- | --- | --- | --- | --- | --- | --- | --- | --- | --- | --- | --- | --- | --- | --- | --- | --- | --- | --- | --- | --- | --- | --- | --- | --- | --- | --- | --- | --- | --- | --- | --- | --- | --- | --- | --- | --- | --- | --- | --- | --- | --- | --- |
| Class | 1.1 | 1.10 | 1.11 | 1.13 | 1.14 | 1.15 | 1.16 | 1.17 | 1.18 | 1.2 | 1.3 | 1.4 | 1.5 | 1.6 | 1.7 | 1.8 | 1.9 | 1.97 | 2.1 | 2.2 | 2.3 | 2.4 | 2.5 | 2.6 | 2.7 | 2.8 | 2.9 | 3.1 | 3.11 | 3.2 | 3.3 | 3.4 | 3.5 | 3.6 | 3.7 | 4.1 | 4.2 | 4.3 | 4.4 | 4.6 | 4.99 | 5.1 | 5.2 | 5.3 | 5.4 | 5.5 | 5.6 | 6.1 | 6.2 | 6.3 | 6.5 | 7.1 | 7.2 | 7.3 | 7.4 | 7.5 | 7.6 | Description |
| ACC | 0.98699 | 0.99947 | 0.99841 | 0.99992 | 0.99887 | 0.99955 | 0.99955 | 0.99788 | 0.99818 | 0.9938 | 0.99584 | 0.99024 | 0.99675 | 0.99894 | 0.99871 | 0.99924 | 0.99992 | 0.98759 | 0.98336 | 0.99728 | 0.95779 | 0.98926 | 0.9938 | 0.99009 | 0.96354 | 0.99894 | 1.0 | 0.97504 | 1.0 | 0.98495 | 0.99992 | 0.99281 | 0.9801 | 0.98358 | 0.99962 | 0.99047 | 0.98268 | 0.99508 | 0.99992 | 0.99032 | 0.99917 | 0.99652 | 0.99834 | 0.98971 | 0.99493 | 0.99992 | 0.99773 | 0.99107 | 0.99682 | 0.98389 | 0.99667 | 0.99342 | 0.99365 | 0.99909 | 0.99917 | 0.99962 | 0.99803 | Accuracy |
| AGF | 0.83497 | 0.94042 | 0.90031 | 0.99278 | 0.9473 | 0.95338 | 0.0 | 0.89853 | 0.72498 | 0.85328 | 0.82594 | 0.14061 | 0.5518 | 0.76154 | 0.93517 | 0.91013 | 0.9938 | 0.0 | 0.91309 | 0.95159 | 0.838 | 0.94744 | 0.97078 | 0.79886 | 0.9266 | 0.97867 | 1.0 | 0.89431 | 1.0 | 0.89769 | 0.8885 | 0.96211 | 0.84233 | 0.86936 | 0.95687 | 0.91619 | 0.78735 | 0.63924 | 0.99688 | 0.48712 | 0.9466 | 0.93828 | 0.98594 | 0.86414 | 0.91415 | 0.74533 | 0.85605 | 0.95233 | 0.54509 | 0.93273 | 0.81698 | 0.91363 | 0.63612 | 0.92039 | 0.97868 | 0.91279 | 0.79201 | Adjusted F-score |
| AGM | 0.91153 | 0.96361 | 0.94751 | 0.99551 | 0.97542 | 0.98014 | 0 | 0.94859 | 0.84468 | 0.92321 | 0.90144 | 0.5614 | 0.75939 | 0.86771 | 0.97127 | 0.94999 | 0.99994 | 0 | 0.94941 | 0.9809 | 0.90936 | 0.97636 | 0.9864 | 0.93157 | 0.95032 | 0.9878 | 1.0 | 0.94097 | 1.0 | 0.96267 | 0.933 | 0.9837 | 0.90832 | 0.93092 | 0.97489 | 0.95057 | 0.88605 | 0.83002 | 0.99994 | 0.7708 | 0.97396 | 0.97331 | 0.99874 | 0.93849 | 0.94874 | 0.85354 | 0.93054 | 0.97169 | 0.7517 | 0.96841 | 0.89736 | 0.9543 | 0.81007 | 0.96247 | 0.98821 | 0.95975 | 0.88814 | Adjusted geometric mean |
| AM | -16 | -7 | -3 | -1 | 3 | 2 | -6 | 0 | -20 | -6 | -33 | -113 | -27 | -8 | 5 | -4 | 1 | -162 | -64 | 18 | 168 | 82 | 44 | 89 | 40 | -6 | 0 | 66 | 0 | 141 | -1 | 57 | -197 | 21 | -3 | -94 | -21 | 17 | 1 | 64 | 1 | 18 | 22 | 52 | -61 | -1 | 4 | -96 | -40 | 125 | -24 | -3 | -16 | 2 | -3 | 1 | -8 | Difference between automatic and manual classification |
| AUC | 0.84438 | 0.93 | 0.9013 | 0.99107 | 0.95278 | 0.96139 | 0.5 | 0.90358 | 0.73802 | 0.86105 | 0.82358 | 0.50782 | 0.63511 | 0.77072 | 0.94504 | 0.90529 | 0.99996 | 0.49996 | 0.91162 | 0.96462 | 0.8588 | 0.96243 | 0.97822 | 0.87914 | 0.92857 | 0.97637 | 1.0 | 0.90383 | 1.0 | 0.94013 | 0.875 | 0.97386 | 0.83918 | 0.88006 | 0.95118 | 0.90905 | 0.80483 | 0.71937 | 0.99996 | 0.64853 | 0.94977 | 0.9505 | 0.99916 | 0.89086 | 0.90408 | 0.75 | 0.8719 | 0.94781 | 0.62723 | 0.95152 | 0.81682 | 0.91626 | 0.69383 | 0.92831 | 0.97712 | 0.92296 | 0.80198 | Area under the ROC curve |
| AUCI | Very Good | Excellent | Excellent | Excellent | Excellent | Excellent | Poor | Excellent | Good | Very Good | Very Good | Poor | Fair | Good | Excellent | Excellent | Excellent | Poor | Excellent | Excellent | Very Good | Excellent | Excellent | Very Good | Excellent | Excellent | Excellent | Excellent | Excellent | Excellent | Very Good | Excellent | Very Good | Very Good | Excellent | Excellent | Very Good | Good | Excellent | Fair | Excellent | Excellent | Excellent | Very Good | Excellent | Good | Very Good | Excellent | Fair | Excellent | Very Good | Excellent | Fair | Excellent | Excellent | Excellent | Very Good | AUC value interpretation |
| AUPR | 0.71384 | 0.93 | 0.82405 | 0.99107 | 0.88596 | 0.89011 | None | 0.80822 | 0.69264 | 0.73912 | 0.76422 | 0.10813 | 0.44494 | 0.67708 | 0.85379 | 0.85995 | 0.97059 | 0.0 | 0.86405 | 0.87504 | 0.67988 | 0.86195 | 0.92192 | 0.57607 | 0.87074 | 0.96686 | 1.0 | 0.79014 | 1.0 | 0.74088 | 0.875 | 0.89546 | 0.80901 | 0.7513 | 0.93806 | 0.89415 | 0.63893 | 0.37926 | 0.98485 | 0.21581 | 0.89118 | 0.85356 | 0.93785 | 0.70722 | 0.8987 | 0.75 | 0.718 | 0.9424 | 0.59394 | 0.82837 | 0.74474 | 0.84039 | 0.43755 | 0.83398 | 0.96403 | 0.81593 | 0.67375 | Area under the PR curve |
| BB | 0.69481 | 0.86 | 0.80328 | 0.98214 | 0.86567 | 0.85714 | 0.0 | 0.80822 | 0.47619 | 0.725 | 0.648 | 0.01626 | 0.27083 | 0.54167 | 0.81667 | 0.81081 | 0.94118 | 0.0 | 0.82953 | 0.81879 | 0.61301 | 0.79026 | 0.88246 | 0.38547 | 0.86139 | 0.95305 | 1.0 | 0.75676 | 1.0 | 0.58838 | 0.75 | 0.83726 | 0.681 | 0.73318 | 0.90244 | 0.81938 | 0.61774 | 0.31667 | 0.9697 | 0.12727 | 0.88235 | 0.80368 | 0.87571 | 0.6255 | 0.80838 | 0.5 | 0.69091 | 0.89652 | 0.25455 | 0.7404 | 0.63441 | 0.83577 | 0.39024 | 0.81081 | 0.95455 | 0.78571 | 0.60465 | Braun-Blanquet similarity |
| BCD | 0.00061 | 0.00026 | 0.00011 | 4e-05 | 0.00011 | 8e-05 | 0.00023 | 0.0 | 0.00076 | 0.00023 | 0.00125 | 0.00427 | 0.00102 | 0.0003 | 0.00019 | 0.00015 | 4e-05 | 0.00613 | 0.00242 | 0.00068 | 0.00635 | 0.0031 | 0.00166 | 0.00337 | 0.00151 | 0.00023 | 0.0 | 0.0025 | 0.0 | 0.00533 | 4e-05 | 0.00216 | 0.00745 | 0.00079 | 0.00011 | 0.00356 | 0.00079 | 0.00064 | 4e-05 | 0.00242 | 4e-05 | 0.00068 | 0.00083 | 0.00197 | 0.00231 | 4e-05 | 0.00015 | 0.00363 | 0.00151 | 0.00473 | 0.00091 | 0.00011 | 0.00061 | 8e-05 | 0.00011 | 4e-05 | 0.0003 | Bray-Curtis dissimilarity |
| BM | 0.68876 | 0.86 | 0.80259 | 0.98214 | 0.90557 | 0.92277 | 0.0 | 0.80715 | 0.47604 | 0.72209 | 0.64716 | 0.01565 | 0.27023 | 0.54144 | 0.89007 | 0.81058 | 0.99992 | -8e-05 | 0.82323 | 0.92923 | 0.71759 | 0.92486 | 0.95643 | 0.75829 | 0.85714 | 0.95274 | 1.0 | 0.80765 | 1.0 | 0.88025 | 0.75 | 0.94773 | 0.67836 | 0.76011 | 0.90236 | 0.81811 | 0.60967 | 0.43875 | 0.99992 | 0.29706 | 0.89954 | 0.901 | 0.99832 | 0.78173 | 0.80815 | 0.5 | 0.74381 | 0.89562 | 0.25447 | 0.90304 | 0.63365 | 0.83252 | 0.38766 | 0.85661 | 0.95424 | 0.84593 | 0.60397 | Informedness or bookmaker informedness |
| CEN | 0.19967 | 0.06599 | 0.13181 | 0.00899 | 0.09194 | 0.07556 | 0.09549 | 0.1488 | 0.20276 | 0.20937 | 0.17322 | 0.21821 | 0.22452 | 0.22401 | 0.12981 | 0.11445 | 0.02246 | 0.02182 | 0.1364 | 0.11805 | 0.28952 | 0.12187 | 0.09203 | 0.19321 | 0.15135 | 0.0351 | 0 | 0.21694 | 0 | 0.23311 | 0.05891 | 0.12061 | 0.18561 | 0.21446 | 0.05489 | 0.11255 | 0.32129 | 0.4004 | 0.01361 | 0.47869 | 0.09379 | 0.15014 | 0.0624 | 0.21873 | 0.10992 | 0.07761 | 0.19037 | 0.0712 | 0.26044 | 0.16003 | 0.1994 | 0.14406 | 0.31525 | 0.11767 | 0.03854 | 0.0926 | 0.25436 | Confusion entropy |
| DOR | 374.55837 | None | 5965.75 | None | 14119.77778 | 39567.0 | None | 3953.0 | 5988.63636 | 903.37081 | 2189.51033 | 27.04132 | 611.13929 | 5196.84848 | 9765.10606 | 18827.14286 | None | 0.0 | 767.86096 | 6557.37449 | 98.17687 | 1589.40774 | 5004.23726 | 388.87922 | 312.55578 | 65985.15 | None | 289.26263 | None | 629.77911 | None | 3447.78601 | 806.36561 | 355.4043 | 121887.25 | 3570.68523 | 198.70585 | 253.62297 | None | 59.5957 | 19744.5 | 3813.62054 | None | 514.02786 | 18115.3125 | None | 2261.25792 | 9588.18862 | 4494.68293 | 811.80312 | 2276.01176 | 1563.37937 | 246.64471 | 11294.57143 | 68570.25 | 24205.5 | 2237.52941 | Diagnostic odds ratio |
| DP | 1.41886 | None | 2.08163 | None | 2.28792 | 2.53464 | None | 1.98309 | 2.08255 | 1.62965 | 1.84163 | 0.78952 | 1.53608 | 2.04859 | 2.19962 | 2.35681 | None | None | 1.59074 | 2.10427 | 1.09825 | 1.76493 | 2.03955 | 1.42784 | 1.37553 | 2.6571 | None | 1.35698 | None | 1.54327 | None | 1.95035 | 1.60246 | 1.40629 | 2.80404 | 1.95873 | 1.26707 | 1.3255 | None | 0.97873 | 2.3682 | 1.97449 | None | 1.49465 | 2.34758 | None | 1.84935 | 2.19525 | 2.01384 | 1.60406 | 1.85091 | 1.76098 | 1.31882 | 2.23446 | 2.6663 | 2.41698 | 1.84682 | Discriminant power |
| DPI | Limited | None | Fair | None | Fair | Fair | None | Limited | Fair | Limited | Limited | Poor | Limited | Fair | Fair | Fair | None | None | Limited | Fair | Limited | Limited | Fair | Limited | Limited | Fair | None | Limited | None | Limited | None | Limited | Limited | Limited | Fair | Limited | Limited | Limited | None | Poor | Fair | Limited | None | Limited | Fair | None | Limited | Fair | Fair | Limited | Limited | Limited | Limited | Fair | Fair | Fair | Limited | Discriminant power interpretation |
| ERR | 0.01301 | 0.00053 | 0.00159 | 8e-05 | 0.00113 | 0.00045 | 0.00045 | 0.00212 | 0.00182 | 0.0062 | 0.00416 | 0.00976 | 0.00325 | 0.00106 | 0.00129 | 0.00076 | 8e-05 | 0.01241 | 0.01664 | 0.00272 | 0.04221 | 0.01074 | 0.0062 | 0.00991 | 0.03646 | 0.00106 | 0.0 | 0.02496 | 0.0 | 0.01505 | 8e-05 | 0.00719 | 0.0199 | 0.01642 | 0.00038 | 0.00953 | 0.01732 | 0.00492 | 8e-05 | 0.00968 | 0.00083 | 0.00348 | 0.00166 | 0.01029 | 0.00507 | 8e-05 | 0.00227 | 0.00893 | 0.00318 | 0.01611 | 0.00333 | 0.00658 | 0.00635 | 0.00091 | 0.00083 | 0.00038 | 0.00197 | Error rate |
| F0.5 | 0.72493 | 0.96847 | 0.83618 | 0.99638 | 0.87349 | 0.86957 | 0.0 | 0.80822 | 0.76923 | 0.74742 | 0.8215 | 0.06135 | 0.49242 | 0.73864 | 0.83051 | 0.88757 | 0.95238 | 0.0 | 0.88386 | 0.83906 | 0.63578 | 0.8153 | 0.89719 | 0.42804 | 0.86507 | 0.97502 | 1.0 | 0.76923 | 1.0 | 0.6315 | 0.9375 | 0.85821 | 0.87149 | 0.74015 | 0.95855 | 0.93481 | 0.65119 | 0.33569 | 0.97561 | 0.14403 | 0.88583 | 0.82183 | 0.89803 | 0.65254 | 0.9467 | 0.83333 | 0.70111 | 0.96845 | 0.6087 | 0.76997 | 0.79946 | 0.84315 | 0.46243 | 0.81967 | 0.96966 | 0.7971 | 0.71038 | F0.5 score |
| F1 | 0.71333 | 0.92473 | 0.82353 | 0.99099 | 0.8855 | 0.88889 | 0.0 | 0.80822 | 0.625 | 0.73885 | 0.74654 | 0.03008 | 0.37681 | 0.65 | 0.85217 | 0.85714 | 0.9697 | 0.0 | 0.86267 | 0.87143 | 0.6733 | 0.85598 | 0.92023 | 0.51301 | 0.87064 | 0.96667 | 1.0 | 0.78873 | 1.0 | 0.70949 | 0.85714 | 0.89168 | 0.78876 | 0.75086 | 0.93671 | 0.8879 | 0.63823 | 0.36893 | 0.98462 | 0.17949 | 0.89109 | 0.85065 | 0.93373 | 0.69778 | 0.88962 | 0.66667 | 0.71698 | 0.94016 | 0.4 | 0.81903 | 0.7284 | 0.84037 | 0.43243 | 0.83333 | 0.96393 | 0.81481 | 0.66667 | F1 score - harmonic mean of precision and sensitivity |
| F2 | 0.7021 | 0.88477 | 0.81126 | 0.98566 | 0.89783 | 0.90909 | 0.0 | 0.80822 | 0.52632 | 0.73048 | 0.68412 | 0.01992 | 0.30516 | 0.58036 | 0.875 | 0.82873 | 0.98765 | 0.0 | 0.84248 | 0.90639 | 0.71553 | 0.90094 | 0.94449 | 0.64007 | 0.87628 | 0.95845 | 1.0 | 0.80925 | 1.0 | 0.80946 | 0.78947 | 0.92786 | 0.72036 | 0.76188 | 0.91584 | 0.84548 | 0.62577 | 0.40948 | 0.99379 | 0.2381 | 0.89641 | 0.88156 | 0.9724 | 0.74976 | 0.83903 | 0.55556 | 0.73359 | 0.91348 | 0.29787 | 0.87477 | 0.66893 | 0.8376 | 0.40609 | 0.84746 | 0.95828 | 0.83333 | 0.62802 | F2 score |
| FDR | 0.26712 | 0.0 | 0.15517 | 0.0 | 0.13433 | 0.14286 | None | 0.19178 | 0.09091 | 0.24675 | 0.11957 | 0.8 | 0.38095 | 0.1875 | 0.18333 | 0.09091 | 0.05882 | 1.0 | 0.10143 | 0.18121 | 0.38699 | 0.20974 | 0.11754 | 0.61453 | 0.13861 | 0.01932 | 0.0 | 0.24324 | 0.0 | 0.41162 | 0.0 | 0.16274 | 0.06298 | 0.26682 | 0.02632 | 0.03107 | 0.33987 | 0.68333 | 0.0303 | 0.87273 | 0.11765 | 0.19632 | 0.12429 | 0.3745 | 0.01099 | 0.0 | 0.30909 | 0.01173 | 0.06667 | 0.2596 | 0.14493 | 0.15498 | 0.51515 | 0.18919 | 0.02649 | 0.21429 | 0.25714 | False discovery rate |
| FN | 94 | 7 | 12 | 1 | 6 | 2 | 6 | 14 | 22 | 44 | 44 | 121 | 35 | 11 | 6 | 7 | 0 | 163 | 142 | 9 | 195 | 30 | 19 | 21 | 221 | 10 | 0 | 132 | 0 | 29 | 1 | 19 | 230 | 98 | 4 | 110 | 125 | 24 | 0 | 32 | 5 | 14 | 0 | 42 | 64 | 1 | 13 | 107 | 41 | 44 | 34 | 45 | 50 | 5 | 7 | 2 | 17 | False negative/miss/type 2 error |
| FNR | 0.30519 | 0.14 | 0.19672 | 0.01786 | 0.09375 | 0.07692 | 1.0 | 0.19178 | 0.52381 | 0.275 | 0.352 | 0.98374 | 0.72917 | 0.45833 | 0.10909 | 0.18919 | 0.0 | 1.0 | 0.17047 | 0.0687 | 0.25325 | 0.06637 | 0.03862 | 0.23333 | 0.11991 | 0.04695 | 0.0 | 0.17647 | 0.0 | 0.10662 | 0.25 | 0.04634 | 0.319 | 0.23059 | 0.09756 | 0.18062 | 0.38226 | 0.55814 | 0.0 | 0.69565 | 0.1 | 0.09655 | 0.0 | 0.21106 | 0.19162 | 0.5 | 0.2549 | 0.10348 | 0.74545 | 0.08365 | 0.36559 | 0.16423 | 0.60976 | 0.14286 | 0.04545 | 0.15385 | 0.39535 | Miss rate or false negative rate |
| FOR | 0.00727 | 0.00053 | 0.00091 | 8e-05 | 0.00046 | 0.00015 | 0.00045 | 0.00106 | 0.00167 | 0.00337 | 0.00335 | 0.00916 | 0.00265 | 0.00083 | 0.00046 | 0.00053 | 0.0 | 0.01233 | 0.01141 | 0.00069 | 0.01588 | 0.00236 | 0.0015 | 0.00161 | 0.0195 | 0.00077 | 0.0 | 0.01064 | 0.0 | 0.00226 | 8e-05 | 0.00149 | 0.01812 | 0.00767 | 0.0003 | 0.00866 | 0.00968 | 0.00182 | 0.0 | 0.00244 | 0.00038 | 0.00107 | 0.0 | 0.00324 | 0.00494 | 8e-05 | 0.00099 | 0.00871 | 0.00311 | 0.0035 | 0.00259 | 0.00348 | 0.0038 | 0.00038 | 0.00054 | 0.00015 | 0.00129 | False omission rate |
| FP | 78 | 0 | 9 | 0 | 9 | 4 | 0 | 14 | 2 | 38 | 11 | 8 | 8 | 3 | 11 | 3 | 1 | 1 | 78 | 27 | 363 | 112 | 63 | 110 | 261 | 4 | 0 | 198 | 0 | 170 | 0 | 76 | 33 | 119 | 1 | 16 | 104 | 41 | 1 | 96 | 6 | 32 | 22 | 94 | 3 | 0 | 17 | 11 | 1 | 169 | 10 | 42 | 34 | 7 | 4 | 3 | 9 | False positive/type 1 error/false alarm |
| FPR | 0.00604 | 0.0 | 0.00068 | 0.0 | 0.00068 | 0.0003 | 0.0 | 0.00106 | 0.00015 | 0.00291 | 0.00084 | 0.00061 | 0.00061 | 0.00023 | 0.00084 | 0.00023 | 8e-05 | 8e-05 | 0.0063 | 0.00206 | 0.02916 | 0.00877 | 0.00495 | 0.00838 | 0.02294 | 0.00031 | 0.0 | 0.01588 | 0.0 | 0.01313 | 0.0 | 0.00593 | 0.00264 | 0.0093 | 8e-05 | 0.00127 | 0.00807 | 0.00311 | 8e-05 | 0.00729 | 0.00046 | 0.00245 | 0.00168 | 0.00722 | 0.00023 | 0.0 | 0.00129 | 0.0009 | 8e-05 | 0.01331 | 0.00076 | 0.00324 | 0.00259 | 0.00053 | 0.00031 | 0.00023 | 0.00068 | Fall-out or false positive rate |
| G | 0.71359 | 0.92736 | 0.82379 | 0.99103 | 0.88573 | 0.8895 | None | 0.80822 | 0.65795 | 0.73899 | 0.75533 | 0.05703 | 0.40946 | 0.6634 | 0.85298 | 0.85855 | 0.97014 | 0.0 | 0.86336 | 0.87323 | 0.67658 | 0.85896 | 0.92108 | 0.54363 | 0.87069 | 0.96677 | 1.0 | 0.78944 | 1.0 | 0.72501 | 0.86603 | 0.89357 | 0.79882 | 0.75108 | 0.93738 | 0.89102 | 0.63858 | 0.37406 | 0.98473 | 0.19681 | 0.89113 | 0.85211 | 0.93579 | 0.70248 | 0.89415 | 0.70711 | 0.71749 | 0.94128 | 0.48742 | 0.82369 | 0.73652 | 0.84038 | 0.43498 | 0.83366 | 0.96398 | 0.81537 | 0.6702 | G-measure geometric mean of precision and sensitivity |
| GI | 0.68876 | 0.86 | 0.80259 | 0.98214 | 0.90557 | 0.92277 | 0.0 | 0.80715 | 0.47604 | 0.72209 | 0.64716 | 0.01565 | 0.27023 | 0.54144 | 0.89007 | 0.81058 | 0.99992 | -8e-05 | 0.82323 | 0.92923 | 0.71759 | 0.92486 | 0.95643 | 0.75829 | 0.85714 | 0.95274 | 1.0 | 0.80765 | 1.0 | 0.88025 | 0.75 | 0.94773 | 0.67836 | 0.76011 | 0.90236 | 0.81811 | 0.60967 | 0.43875 | 0.99992 | 0.29706 | 0.89954 | 0.901 | 0.99832 | 0.78173 | 0.80815 | 0.5 | 0.74381 | 0.89562 | 0.25447 | 0.90304 | 0.63365 | 0.83252 | 0.38766 | 0.85661 | 0.95424 | 0.84593 | 0.60397 | Gini index |
| GM | 0.83103 | 0.92736 | 0.89595 | 0.99103 | 0.95165 | 0.96062 | 0.0 | 0.89853 | 0.69001 | 0.85023 | 0.80465 | 0.12748 | 0.52026 | 0.7359 | 0.94349 | 0.90035 | 0.99996 | 0.0 | 0.90791 | 0.96404 | 0.85146 | 0.962 | 0.97807 | 0.87192 | 0.92731 | 0.97609 | 1.0 | 0.90025 | 1.0 | 0.93896 | 0.86603 | 0.97365 | 0.82414 | 0.87307 | 0.94993 | 0.90462 | 0.78279 | 0.66369 | 0.99996 | 0.54966 | 0.94847 | 0.94934 | 0.99916 | 0.88501 | 0.899 | 0.70711 | 0.86263 | 0.94642 | 0.50451 | 0.95087 | 0.79619 | 0.91272 | 0.62389 | 0.92557 | 0.97686 | 0.91976 | 0.77733 | G-mean geometric mean of specificity and sensitivity |
| HD | 172 | 7 | 21 | 1 | 15 | 6 | 6 | 28 | 24 | 82 | 55 | 129 | 43 | 14 | 17 | 10 | 1 | 164 | 220 | 36 | 558 | 142 | 82 | 131 | 482 | 14 | 0 | 330 | 0 | 199 | 1 | 95 | 263 | 217 | 5 | 126 | 229 | 65 | 1 | 128 | 11 | 46 | 22 | 136 | 67 | 1 | 30 | 118 | 42 | 213 | 44 | 87 | 84 | 12 | 11 | 5 | 26 | Hamming distance |
| IBA | 0.48401 | 0.7396 | 0.64536 | 0.9646 | 0.82135 | 0.85209 | 0.0 | 0.65338 | 0.2268 | 0.5262 | 0.4201 | 0.00027 | 0.07347 | 0.29346 | 0.7938 | 0.65745 | 1.0 | 0.0 | 0.68898 | 0.86744 | 0.56252 | 0.87213 | 0.92442 | 0.58922 | 0.77651 | 0.90832 | 1.0 | 0.6803 | 1.0 | 0.79923 | 0.5625 | 0.90969 | 0.46433 | 0.59358 | 0.8144 | 0.67156 | 0.38346 | 0.196 | 1.0 | 0.09415 | 0.81004 | 0.81643 | 1.0 | 0.62359 | 0.65352 | 0.25 | 0.55542 | 0.80383 | 0.06481 | 0.84056 | 0.40265 | 0.69894 | 0.1529 | 0.73476 | 0.91117 | 0.71601 | 0.36577 | Index of balanced accuracy |
| ICSI | 0.42768 | 0.86 | 0.64811 | 0.98214 | 0.77192 | 0.78022 | None | 0.61644 | 0.38528 | 0.47825 | 0.52843 | -0.78374 | -0.11012 | 0.35417 | 0.70758 | 0.7199 | 0.94118 | -1.0 | 0.7281 | 0.75009 | 0.35976 | 0.72389 | 0.84384 | 0.15214 | 0.74148 | 0.93373 | 1.0 | 0.58029 | 1.0 | 0.48176 | 0.75 | 0.79092 | 0.61802 | 0.5026 | 0.87612 | 0.78831 | 0.27787 | -0.24147 | 0.9697 | -0.56838 | 0.78235 | 0.70713 | 0.87571 | 0.41444 | 0.79739 | 0.5 | 0.43601 | 0.88479 | 0.18788 | 0.65675 | 0.48948 | 0.68078 | -0.12491 | 0.66795 | 0.92806 | 0.63187 | 0.34751 | Individual classification success index |
| IS | 4.97518 | 8.04647 | 7.51632 | 7.88297 | 7.48222 | 8.76749 | None | 7.19332 | 8.1605 | 5.95959 | 6.54083 | 4.42588 | 7.41349 | 8.8058 | 7.61678 | 8.34337 | 9.60286 | None | 3.83385 | 6.36847 | 3.39558 | 4.53055 | 4.56742 | 5.82318 | 2.62723 | 5.92746 | 8.93544 | 3.74133 | 10.69033 | 4.83768 | 11.69033 | 4.75459 | 4.10263 | 4.51125 | 8.2943 | 4.39449 | 4.738 | 6.6051 | 8.64593 | 5.19276 | 7.8659 | 6.19511 | 6.22272 | 5.37678 | 5.29068 | 12.69033 | 7.48447 | 3.65929 | 7.80943 | 4.21778 | 6.92529 | 5.34935 | 6.28838 | 8.25848 | 6.38481 | 9.64196 | 7.83522 | Information score |
| J | 0.5544 | 0.86 | 0.7 | 0.98214 | 0.79452 | 0.8 | 0.0 | 0.67816 | 0.45455 | 0.58586 | 0.59559 | 0.01527 | 0.23214 | 0.48148 | 0.74242 | 0.75 | 0.94118 | 0.0 | 0.75851 | 0.77215 | 0.5075 | 0.74823 | 0.85225 | 0.345 | 0.77091 | 0.93548 | 1.0 | 0.65116 | 1.0 | 0.54977 | 0.75 | 0.80453 | 0.65119 | 0.6011 | 0.88095 | 0.7984 | 0.46868 | 0.22619 | 0.9697 | 0.09859 | 0.80357 | 0.74011 | 0.87571 | 0.53584 | 0.80119 | 0.5 | 0.55882 | 0.88708 | 0.25 | 0.69353 | 0.57282 | 0.72468 | 0.27586 | 0.71429 | 0.93038 | 0.6875 | 0.5 | Jaccard index |
| LS | 31.45421 | 264.38 | 183.07829 | 236.05357 | 178.80177 | 435.79121 | None | 146.3541 | 286.12554 | 62.23231 | 93.10774 | 21.49431 | 170.48313 | 447.51823 | 196.28212 | 324.79115 | 777.58824 | 0.0 | 14.25953 | 82.62298 | 10.52381 | 23.11167 | 23.70991 | 56.61769 | 6.17837 | 60.86179 | 489.59259 | 13.37375 | 1652.375 | 28.59472 | 3304.75 | 26.99446 | 17.17962 | 22.80461 | 313.93004 | 21.03171 | 26.68583 | 97.34922 | 400.57576 | 36.57431 | 233.27647 | 73.26799 | 74.68362 | 41.55004 | 39.14292 | 6609.5 | 179.08093 | 12.63441 | 224.32242 | 18.60711 | 121.53982 | 40.76751 | 78.16112 | 306.23166 | 83.56382 | 798.95055 | 228.36811 | Lift score |
| MCC | 0.70694 | 0.92712 | 0.823 | 0.99099 | 0.88516 | 0.88927 | None | 0.80715 | 0.65724 | 0.73585 | 0.7534 | 0.05465 | 0.40813 | 0.66292 | 0.85234 | 0.85817 | 0.97011 | -0.00097 | 0.8546 | 0.8719 | 0.6546 | 0.85363 | 0.91792 | 0.53952 | 0.84949 | 0.96623 | 1.0 | 0.77627 | 1.0 | 0.71828 | 0.86599 | 0.88999 | 0.78952 | 0.74261 | 0.9372 | 0.88634 | 0.62973 | 0.37167 | 0.98469 | 0.19257 | 0.89072 | 0.85038 | 0.935 | 0.69745 | 0.89178 | 0.70708 | 0.71636 | 0.93665 | 0.48653 | 0.81575 | 0.73497 | 0.83702 | 0.43183 | 0.8332 | 0.96356 | 0.81519 | 0.66924 | Matthews correlation coefficient |
| MCCI | Strong | Very Strong | Strong | Very Strong | Strong | Strong | None | Strong | Moderate | Strong | Strong | Negligible | Weak | Moderate | Strong | Strong | Very Strong | Negligible | Strong | Strong | Moderate | Strong | Very Strong | Moderate | Strong | Very Strong | Very Strong | Strong | Very Strong | Strong | Strong | Strong | Strong | Strong | Very Strong | Strong | Moderate | Weak | Very Strong | Negligible | Strong | Strong | Very Strong | Moderate | Strong | Strong | Strong | Very Strong | Weak | Strong | Strong | Strong | Weak | Strong | Very Strong | Strong | Moderate | Matthews correlation coefficient interpretation |
| MCEN | 0.26872 | 0.10432 | 0.19034 | 0.01523 | 0.13953 | 0.11109 | 0.09549 | 0.2144 | 0.25161 | 0.29156 | 0.23634 | 0.21838 | 0.24267 | 0.28868 | 0.19587 | 0.17064 | 0.03532 | 0.02182 | 0.21098 | 0.18157 | 0.39362 | 0.18325 | 0.15115 | 0.21872 | 0.24027 | 0.0589 | 0 | 0.32113 | 0 | 0.31946 | 0.07345 | 0.19319 | 0.2694 | 0.30358 | 0.08731 | 0.17733 | 0.4286 | 0.45752 | 0.02246 | 0.50793 | 0.14461 | 0.23075 | 0.10048 | 0.29373 | 0.17319 | 0.07345 | 0.25525 | 0.11917 | 0.29008 | 0.2368 | 0.27263 | 0.21666 | 0.36483 | 0.16909 | 0.0647 | 0.12161 | 0.33857 | Modified confusion entropy |
| MK | 0.72561 | 0.99947 | 0.84392 | 0.99992 | 0.86522 | 0.85699 | None | 0.80715 | 0.90742 | 0.74988 | 0.87708 | 0.19084 | 0.6164 | 0.81167 | 0.81621 | 0.90856 | 0.94118 | -0.01233 | 0.88716 | 0.8181 | 0.59713 | 0.7879 | 0.88096 | 0.38386 | 0.8419 | 0.97991 | 1.0 | 0.74612 | 1.0 | 0.58611 | 0.99992 | 0.83577 | 0.91891 | 0.72551 | 0.97338 | 0.96027 | 0.65045 | 0.31484 | 0.9697 | 0.12483 | 0.88197 | 0.80261 | 0.87571 | 0.62226 | 0.98407 | 0.99992 | 0.68992 | 0.97956 | 0.93023 | 0.7369 | 0.85249 | 0.84154 | 0.48105 | 0.81043 | 0.97297 | 0.78556 | 0.74157 | Markedness |
| N | 12911 | 13169 | 13158 | 13163 | 13155 | 13193 | 13213 | 13146 | 13177 | 13059 | 13094 | 13096 | 13171 | 13195 | 13164 | 13182 | 13203 | 13056 | 12386 | 13088 | 12449 | 12767 | 12727 | 13129 | 11376 | 13006 | 13192 | 12471 | 13211 | 12947 | 13215 | 12809 | 12498 | 12794 | 13178 | 12610 | 12892 | 13176 | 13187 | 13173 | 13169 | 13074 | 13064 | 13020 | 12885 | 13217 | 13168 | 12185 | 13164 | 12693 | 13126 | 12945 | 13137 | 13184 | 13065 | 13206 | 13176 | Condition negative |
| NLR | 0.30705 | 0.14 | 0.19686 | 0.01786 | 0.09381 | 0.07695 | 1.0 | 0.19199 | 0.52389 | 0.2758 | 0.3523 | 0.98434 | 0.72961 | 0.45844 | 0.10918 | 0.18923 | 0.0 | 1.00008 | 0.17155 | 0.06884 | 0.26085 | 0.06696 | 0.03881 | 0.2353 | 0.12273 | 0.04696 | 0.0 | 0.17932 | 0.0 | 0.10804 | 0.25 | 0.04662 | 0.31985 | 0.23275 | 0.09757 | 0.18085 | 0.38537 | 0.55988 | 0.0 | 0.70076 | 0.10005 | 0.09679 | 0.0 | 0.21259 | 0.19166 | 0.5 | 0.25523 | 0.10358 | 0.74551 | 0.08478 | 0.36587 | 0.16477 | 0.61134 | 0.14293 | 0.04547 | 0.15388 | 0.39562 | Negative likelihood ratio |
| NLRI | Poor | Fair | Fair | Good | Good | Good | Negligible | Fair | Negligible | Poor | Poor | Negligible | Negligible | Poor | Fair | Fair | Good | Negligible | Fair | Good | Poor | Good | Good | Poor | Fair | Good | Good | Fair | Good | Fair | Poor | Good | Poor | Poor | Good | Fair | Poor | Negligible | Good | Negligible | Fair | Good | Good | Poor | Fair | Negligible | Poor | Fair | Negligible | Good | Poor | Fair | Negligible | Fair | Good | Fair | Poor | Negative likelihood ratio interpretation |
| NPV | 0.99273 | 0.99947 | 0.99909 | 0.99992 | 0.99954 | 0.99985 | 0.99955 | 0.99894 | 0.99833 | 0.99663 | 0.99665 | 0.99084 | 0.99735 | 0.99917 | 0.99954 | 0.99947 | 1.0 | 0.98767 | 0.98859 | 0.99931 | 0.98412 | 0.99764 | 0.9985 | 0.99839 | 0.9805 | 0.99923 | 1.0 | 0.98936 | 1.0 | 0.99774 | 0.99992 | 0.99851 | 0.98188 | 0.99233 | 0.9997 | 0.99134 | 0.99032 | 0.99818 | 1.0 | 0.99756 | 0.99962 | 0.99893 | 1.0 | 0.99676 | 0.99506 | 0.99992 | 0.99901 | 0.99129 | 0.99689 | 0.9965 | 0.99741 | 0.99652 | 0.9962 | 0.99962 | 0.99946 | 0.99985 | 0.99871 | Negative predictive value |
| OC | 0.73288 | 1.0 | 0.84483 | 1.0 | 0.90625 | 0.92308 | None | 0.80822 | 0.90909 | 0.75325 | 0.88043 | 0.2 | 0.61905 | 0.8125 | 0.89091 | 0.90909 | 1.0 | 0.0 | 0.89857 | 0.9313 | 0.74675 | 0.93363 | 0.96138 | 0.76667 | 0.88009 | 0.98068 | 1.0 | 0.82353 | 1.0 | 0.89338 | 1.0 | 0.95366 | 0.93702 | 0.76941 | 0.97368 | 0.96893 | 0.66013 | 0.44186 | 1.0 | 0.30435 | 0.9 | 0.90345 | 1.0 | 0.78894 | 0.98901 | 1.0 | 0.7451 | 0.98827 | 0.93333 | 0.91635 | 0.85507 | 0.84502 | 0.48485 | 0.85714 | 0.97351 | 0.84615 | 0.74286 | Overlap coefficient |
| OOC | 0.71359 | 0.92736 | 0.82379 | 0.99103 | 0.88573 | 0.8895 | None | 0.80822 | 0.65795 | 0.73899 | 0.75533 | 0.05703 | 0.40946 | 0.6634 | 0.85298 | 0.85855 | 0.97014 | 0.0 | 0.86336 | 0.87323 | 0.67658 | 0.85896 | 0.92108 | 0.54363 | 0.87069 | 0.96677 | 1.0 | 0.78944 | 1.0 | 0.72501 | 0.86603 | 0.89357 | 0.79882 | 0.75108 | 0.93738 | 0.89102 | 0.63858 | 0.37406 | 0.98473 | 0.19681 | 0.89113 | 0.85211 | 0.93579 | 0.70248 | 0.89415 | 0.70711 | 0.71749 | 0.94128 | 0.48742 | 0.82369 | 0.73652 | 0.84038 | 0.43498 | 0.83366 | 0.96398 | 0.81537 | 0.6702 | Otsuka-Ochiai coefficient |
| OP | 0.80984 | 0.9242 | 0.88966 | 0.99092 | 0.95003 | 0.9597 | -0.00045 | 0.89235 | 0.64341 | 0.8358 | 0.78265 | 0.02226 | 0.42318 | 0.70175 | 0.94144 | 0.89488 | 0.99989 | -0.01241 | 0.89331 | 0.96273 | 0.82732 | 0.95933 | 0.97659 | 0.86215 | 0.91132 | 0.97506 | 1.0 | 0.88619 | 1.0 | 0.93523 | 0.85707 | 0.97207 | 0.79161 | 0.85786 | 0.94838 | 0.89182 | 0.75021 | 0.60931 | 0.99989 | 0.45961 | 0.94676 | 0.94702 | 0.99749 | 0.87531 | 0.88909 | 0.66659 | 0.8523 | 0.93696 | 0.40264 | 0.94693 | 0.77335 | 0.90557 | 0.5561 | 0.92243 | 0.97607 | 0.9164 | 0.75198 | Optimized precision |
| P | 308 | 50 | 61 | 56 | 64 | 26 | 6 | 73 | 42 | 160 | 125 | 123 | 48 | 24 | 55 | 37 | 16 | 163 | 833 | 131 | 770 | 452 | 492 | 90 | 1843 | 213 | 27 | 748 | 8 | 272 | 4 | 410 | 721 | 425 | 41 | 609 | 327 | 43 | 32 | 46 | 50 | 145 | 155 | 199 | 334 | 2 | 51 | 1034 | 55 | 526 | 93 | 274 | 82 | 35 | 154 | 13 | 43 | Condition positive or support |
| PLR | 115.00808 | None | 1174.39344 | None | 1324.63542 | 3044.53846 | None | 758.91781 | 3137.38095 | 249.15197 | 771.35564 | 26.61789 | 445.89323 | 2382.43056 | 1066.17521 | 3562.7027 | 13203.0 | 0.0 | 131.7254 | 451.43794 | 25.60973 | 106.42529 | 194.21445 | 91.50515 | 38.35965 | 3098.84742 | None | 51.86988 | None | 68.03895 | None | 160.72911 | 257.91275 | 82.72146 | 11892.34146 | 645.77073 | 76.57563 | 141.99887 | 13187.0 | 41.76223 | 1975.35 | 369.11509 | 593.81818 | 109.27724 | 3472.00599 | None | 577.14418 | 993.09785 | 3350.83636 | 68.82384 | 832.72473 | 257.59515 | 150.78336 | 1614.36735 | 3117.78409 | 3724.76923 | 885.2093 | Positive likelihood ratio |
| PLRI | Good | None | Good | None | Good | Good | None | Good | Good | Good | Good | Good | Good | Good | Good | Good | Good | Negligible | Good | Good | Good | Good | Good | Good | Good | Good | None | Good | None | Good | None | Good | Good | Good | Good | Good | Good | Good | Good | Good | Good | Good | Good | Good | Good | None | Good | Good | Good | Good | Good | Good | Good | Good | Good | Good | Good | Positive likelihood ratio interpretation |
| POP | 13219 | 13219 | 13219 | 13219 | 13219 | 13219 | 13219 | 13219 | 13219 | 13219 | 13219 | 13219 | 13219 | 13219 | 13219 | 13219 | 13219 | 13219 | 13219 | 13219 | 13219 | 13219 | 13219 | 13219 | 13219 | 13219 | 13219 | 13219 | 13219 | 13219 | 13219 | 13219 | 13219 | 13219 | 13219 | 13219 | 13219 | 13219 | 13219 | 13219 | 13219 | 13219 | 13219 | 13219 | 13219 | 13219 | 13219 | 13219 | 13219 | 13219 | 13219 | 13219 | 13219 | 13219 | 13219 | 13219 | 13219 | Population |
| PPV | 0.73288 | 1.0 | 0.84483 | 1.0 | 0.86567 | 0.85714 | None | 0.80822 | 0.90909 | 0.75325 | 0.88043 | 0.2 | 0.61905 | 0.8125 | 0.81667 | 0.90909 | 0.94118 | 0.0 | 0.89857 | 0.81879 | 0.61301 | 0.79026 | 0.88246 | 0.38547 | 0.86139 | 0.98068 | 1.0 | 0.75676 | 1.0 | 0.58838 | 1.0 | 0.83726 | 0.93702 | 0.73318 | 0.97368 | 0.96893 | 0.66013 | 0.31667 | 0.9697 | 0.12727 | 0.88235 | 0.80368 | 0.87571 | 0.6255 | 0.98901 | 1.0 | 0.69091 | 0.98827 | 0.93333 | 0.7404 | 0.85507 | 0.84502 | 0.48485 | 0.81081 | 0.97351 | 0.78571 | 0.74286 | Precision or positive predictive value |
| PRE | 0.0233 | 0.00378 | 0.00461 | 0.00424 | 0.00484 | 0.00197 | 0.00045 | 0.00552 | 0.00318 | 0.0121 | 0.00946 | 0.0093 | 0.00363 | 0.00182 | 0.00416 | 0.0028 | 0.00121 | 0.01233 | 0.06302 | 0.00991 | 0.05825 | 0.03419 | 0.03722 | 0.00681 | 0.13942 | 0.01611 | 0.00204 | 0.05659 | 0.00061 | 0.02058 | 0.0003 | 0.03102 | 0.05454 | 0.03215 | 0.0031 | 0.04607 | 0.02474 | 0.00325 | 0.00242 | 0.00348 | 0.00378 | 0.01097 | 0.01173 | 0.01505 | 0.02527 | 0.00015 | 0.00386 | 0.07822 | 0.00416 | 0.03979 | 0.00704 | 0.02073 | 0.0062 | 0.00265 | 0.01165 | 0.00098 | 0.00325 | Prevalence |
| Q | 0.99467 | None | 0.99966 | None | 0.99986 | 0.99995 | None | 0.99949 | 0.99967 | 0.99779 | 0.99909 | 0.92868 | 0.99673 | 0.99962 | 0.9998 | 0.99989 | None | -1.0 | 0.9974 | 0.9997 | 0.97983 | 0.99874 | 0.9996 | 0.99487 | 0.99362 | 0.99997 | None | 0.99311 | None | 0.99683 | None | 0.99942 | 0.99752 | 0.99439 | 0.99998 | 0.99944 | 0.98999 | 0.99215 | None | 0.96699 | 0.9999 | 0.99948 | None | 0.99612 | 0.99989 | None | 0.99912 | 0.99979 | 0.99956 | 0.99754 | 0.99912 | 0.99872 | 0.99192 | 0.99982 | 0.99997 | 0.99992 | 0.99911 | Yule Q - coefficient of colligation |
| QI | Strong | None | Strong | None | Strong | Strong | None | Strong | Strong | Strong | Strong | Strong | Strong | Strong | Strong | Strong | None | Negligible | Strong | Strong | Strong | Strong | Strong | Strong | Strong | Strong | None | Strong | None | Strong | None | Strong | Strong | Strong | Strong | Strong | Strong | Strong | None | Strong | Strong | Strong | None | Strong | Strong | None | Strong | Strong | Strong | Strong | Strong | Strong | Strong | Strong | Strong | Strong | Strong | Yule Q interpretation |
| RACC | 0.00051 | 1e-05 | 2e-05 | 2e-05 | 2e-05 | 0.0 | 0.0 | 3e-05 | 1e-05 | 0.00014 | 7e-05 | 1e-05 | 1e-05 | 0.0 | 2e-05 | 1e-05 | 0.0 | 0.0 | 0.00367 | 0.00011 | 0.00413 | 0.00138 | 0.00151 | 9e-05 | 0.01986 | 0.00025 | 0.0 | 0.00348 | 0.0 | 0.00064 | 0.0 | 0.0011 | 0.00216 | 0.00108 | 1e-05 | 0.00179 | 0.00057 | 1e-05 | 1e-05 | 3e-05 | 1e-05 | 0.00014 | 0.00016 | 0.00029 | 0.00052 | 0.0 | 2e-05 | 0.00555 | 0.0 | 0.00196 | 4e-05 | 0.00042 | 3e-05 | 1e-05 | 0.00013 | 0.0 | 1e-05 | Random accuracy |
| RACCU | 0.00052 | 1e-05 | 2e-05 | 2e-05 | 2e-05 | 0.0 | 0.0 | 3e-05 | 1e-05 | 0.00014 | 7e-05 | 3e-05 | 1e-05 | 0.0 | 2e-05 | 1e-05 | 0.0 | 4e-05 | 0.00367 | 0.00011 | 0.00417 | 0.00139 | 0.00151 | 0.0001 | 0.01986 | 0.00025 | 0.0 | 0.00349 | 0.0 | 0.00067 | 0.0 | 0.0011 | 0.00222 | 0.00109 | 1e-05 | 0.00181 | 0.00057 | 2e-05 | 1e-05 | 3e-05 | 1e-05 | 0.00014 | 0.00016 | 0.00029 | 0.00053 | 0.0 | 2e-05 | 0.00556 | 1e-05 | 0.00198 | 4e-05 | 0.00042 | 3e-05 | 1e-05 | 0.00013 | 0.0 | 1e-05 | Random accuracy unbiased |
| TN | 12833 | 13169 | 13149 | 13163 | 13146 | 13189 | 13213 | 13132 | 13175 | 13021 | 13083 | 13088 | 13163 | 13192 | 13153 | 13179 | 13202 | 13055 | 12308 | 13061 | 12086 | 12655 | 12664 | 13019 | 11115 | 13002 | 13192 | 12273 | 13211 | 12777 | 13215 | 12733 | 12465 | 12675 | 13177 | 12594 | 12788 | 13135 | 13186 | 13077 | 13163 | 13042 | 13042 | 12926 | 12882 | 13217 | 13151 | 12174 | 13163 | 12524 | 13116 | 12903 | 13103 | 13177 | 13061 | 13203 | 13167 | True negative/correct rejection |
| TNR | 0.99396 | 1.0 | 0.99932 | 1.0 | 0.99932 | 0.9997 | 1.0 | 0.99894 | 0.99985 | 0.99709 | 0.99916 | 0.99939 | 0.99939 | 0.99977 | 0.99916 | 0.99977 | 0.99992 | 0.99992 | 0.9937 | 0.99794 | 0.97084 | 0.99123 | 0.99505 | 0.99162 | 0.97706 | 0.99969 | 1.0 | 0.98412 | 1.0 | 0.98687 | 1.0 | 0.99407 | 0.99736 | 0.9907 | 0.99992 | 0.99873 | 0.99193 | 0.99689 | 0.99992 | 0.99271 | 0.99954 | 0.99755 | 0.99832 | 0.99278 | 0.99977 | 1.0 | 0.99871 | 0.9991 | 0.99992 | 0.98669 | 0.99924 | 0.99676 | 0.99741 | 0.99947 | 0.99969 | 0.99977 | 0.99932 | Specificity or true negative rate |
| TON | 12927 | 13176 | 13161 | 13164 | 13152 | 13191 | 13219 | 13146 | 13197 | 13065 | 13127 | 13209 | 13198 | 13203 | 13159 | 13186 | 13202 | 13218 | 12450 | 13070 | 12281 | 12685 | 12683 | 13040 | 11336 | 13012 | 13192 | 12405 | 13211 | 12806 | 13216 | 12752 | 12695 | 12773 | 13181 | 12704 | 12913 | 13159 | 13186 | 13109 | 13168 | 13056 | 13042 | 12968 | 12946 | 13218 | 13164 | 12281 | 13204 | 12568 | 13150 | 12948 | 13153 | 13182 | 13068 | 13205 | 13184 | Test outcome negative |
| TOP | 292 | 43 | 58 | 55 | 67 | 28 | 0 | 73 | 22 | 154 | 92 | 10 | 21 | 16 | 60 | 33 | 17 | 1 | 769 | 149 | 938 | 534 | 536 | 179 | 1883 | 207 | 27 | 814 | 8 | 413 | 3 | 467 | 524 | 446 | 38 | 515 | 306 | 60 | 33 | 110 | 51 | 163 | 177 | 251 | 273 | 1 | 55 | 938 | 15 | 651 | 69 | 271 | 66 | 37 | 151 | 14 | 35 | Test outcome positive |
| TP | 214 | 43 | 49 | 55 | 58 | 24 | 0 | 59 | 20 | 116 | 81 | 2 | 13 | 13 | 49 | 30 | 16 | 0 | 691 | 122 | 575 | 422 | 473 | 69 | 1622 | 203 | 27 | 616 | 8 | 243 | 3 | 391 | 491 | 327 | 37 | 499 | 202 | 19 | 32 | 14 | 45 | 131 | 155 | 157 | 270 | 1 | 38 | 927 | 14 | 482 | 59 | 229 | 32 | 30 | 147 | 11 | 26 | True positive/hit |
| TPR | 0.69481 | 0.86 | 0.80328 | 0.98214 | 0.90625 | 0.92308 | 0.0 | 0.80822 | 0.47619 | 0.725 | 0.648 | 0.01626 | 0.27083 | 0.54167 | 0.89091 | 0.81081 | 1.0 | 0.0 | 0.82953 | 0.9313 | 0.74675 | 0.93363 | 0.96138 | 0.76667 | 0.88009 | 0.95305 | 1.0 | 0.82353 | 1.0 | 0.89338 | 0.75 | 0.95366 | 0.681 | 0.76941 | 0.90244 | 0.81938 | 0.61774 | 0.44186 | 1.0 | 0.30435 | 0.9 | 0.90345 | 1.0 | 0.78894 | 0.80838 | 0.5 | 0.7451 | 0.89652 | 0.25455 | 0.91635 | 0.63441 | 0.83577 | 0.39024 | 0.85714 | 0.95455 | 0.84615 | 0.60465 | Sensitivity, recall, hit rate, or true positive rate |
| Y | 0.68876 | 0.86 | 0.80259 | 0.98214 | 0.90557 | 0.92277 | 0.0 | 0.80715 | 0.47604 | 0.72209 | 0.64716 | 0.01565 | 0.27023 | 0.54144 | 0.89007 | 0.81058 | 0.99992 | -8e-05 | 0.82323 | 0.92923 | 0.71759 | 0.92486 | 0.95643 | 0.75829 | 0.85714 | 0.95274 | 1.0 | 0.80765 | 1.0 | 0.88025 | 0.75 | 0.94773 | 0.67836 | 0.76011 | 0.90236 | 0.81811 | 0.60967 | 0.43875 | 0.99992 | 0.29706 | 0.89954 | 0.901 | 0.99832 | 0.78173 | 0.80815 | 0.5 | 0.74381 | 0.89562 | 0.25447 | 0.90304 | 0.63365 | 0.83252 | 0.38766 | 0.85661 | 0.95424 | 0.84593 | 0.60397 | Youden index |
| dInd | 0.30525 | 0.14 | 0.19672 | 0.01786 | 0.09375 | 0.07692 | 1.0 | 0.19178 | 0.52381 | 0.27502 | 0.352 | 0.98374 | 0.72917 | 0.45833 | 0.10909 | 0.18919 | 8e-05 | 1.0 | 0.17058 | 0.06873 | 0.25492 | 0.06695 | 0.03893 | 0.23348 | 0.12209 | 0.04695 | 0.0 | 0.17718 | 0.0 | 0.10742 | 0.25 | 0.04672 | 0.31901 | 0.23078 | 0.09756 | 0.18063 | 0.38235 | 0.55815 | 8e-05 | 0.69569 | 0.1 | 0.09658 | 0.00168 | 0.21118 | 0.19162 | 0.5 | 0.25491 | 0.10349 | 0.74545 | 0.0847 | 0.36559 | 0.16427 | 0.60976 | 0.14286 | 0.04546 | 0.15385 | 0.39535 | Distance index |
| sInd | 0.78415 | 0.90101 | 0.8609 | 0.98737 | 0.93371 | 0.94561 | 0.29289 | 0.86439 | 0.62961 | 0.80553 | 0.7511 | 0.30439 | 0.4844 | 0.67591 | 0.92286 | 0.86622 | 0.99995 | 0.29289 | 0.87938 | 0.9514 | 0.81974 | 0.95266 | 0.97247 | 0.8349 | 0.91367 | 0.9668 | 1.0 | 0.87471 | 1.0 | 0.92404 | 0.82322 | 0.96696 | 0.77442 | 0.83682 | 0.93101 | 0.87228 | 0.72964 | 0.60533 | 0.99995 | 0.50807 | 0.92929 | 0.93171 | 0.99881 | 0.85067 | 0.86451 | 0.64645 | 0.81975 | 0.92682 | 0.47288 | 0.94011 | 0.74149 | 0.88385 | 0.56883 | 0.89898 | 0.96786 | 0.89121 | 0.72045 | Similarity index |

Generated By PyCM Version 3.6
