# Supplementary material for: TopEC: prediction of Enzyme Commission classes by 3D graph neural networks and localized 3D protein descriptor
Source: Nat Commun. 2025 Mar 20;16:2737. doi: 10.1038/s41467-025-57324-5 (PMC11923149; doi:10.1038/s41467-025-57324-5)
Supplement: Supplementary file 3 — Supplementary Data 1 [file 41467_2025_57324_MOESM3_ESM.zip › Data_S1/figure2/AF703_main.html]

PyCM Report


# PyCM Report

## Dataset Type :

- Multi-Class Classification
- Imbalanced

Note 1 : Recommended statistics for this type of classification highlighted in aqua

Note 2 : The recommender system assumes that the input is the result of classification over the whole data rather than just a part of it.
If the confusion matrix is the result of test data classification, the recommendation is not valid.

## Confusion Matrix :

|  |  |  |  |  |  |  |  |  |  |  |  |  |  |  |  |  |  |  |  |  |  |  |  |  |  |  |  |  |  |  |  |  |  |  |  |  |  |  |  |  |  |  |  |  |  |  |  |  |  |  |  |  |  |  |  |  |  |  |  |  |  |  |  |  |  |
| --- | --- | --- | --- | --- | --- | --- | --- | --- | --- | --- | --- | --- | --- | --- | --- | --- | --- | --- | --- | --- | --- | --- | --- | --- | --- | --- | --- | --- | --- | --- | --- | --- | --- | --- | --- | --- | --- | --- | --- | --- | --- | --- | --- | --- | --- | --- | --- | --- | --- | --- | --- | --- | --- | --- | --- | --- | --- | --- | --- | --- | --- | --- | --- | --- | --- |
| Actual | Predict  |  |  |  |  |  |  |  |  | | --- | --- | --- | --- | --- | --- | --- | --- | |  | 1 | 2 | 3 | 4 | 5 | 6 | 7 | | 1 | 886 | 351 | 117 | 28 | 12 | 41 | 2 | | 2 | 41 | 4421 | 170 | 68 | 27 | 95 | 29 | | 3 | 9 | 202 | 2263 | 89 | 30 | 6 | 30 | | 4 | 27 | 85 | 56 | 835 | 86 | 3 | 15 | | 5 | 22 | 32 | 40 | 19 | 753 | 19 | 1 | | 6 | 55 | 69 | 43 | 19 | 10 | 1509 | 3 | | 7 | 2 | 62 | 24 | 17 | 2 | 0 | 494 | |

## Overall Statistics :

|  |  |
| --- | --- |
| 95% CI | (0.83813,0.8505) |
| ACC Macro | 0.95552 |
| ARI | 0.67808 |
| AUNP | 0.8973 |
| AUNU | 0.89235 |
| Bangdiwala B | 0.74642 |
| Bennett S | 0.81837 |
| CBA | 0.7965 |
| CSI | 0.65531 |
| Chi-Squared | 50895.37037 |
| Chi-Squared DF | 36 |
| Conditional Entropy | 0.88469 |
| Cramer V | 0.80106 |
| Cross Entropy | 2.49645 |
| F1 Macro | 0.82465 |
| F1 Micro | 0.84432 |
| FNR Macro | 0.18595 |
| FNR Micro | 0.15568 |
| FPR Macro | 0.02934 |
| FPR Micro | 0.02595 |
| Gwet AC1 | 0.82121 |
| Hamming Loss | 0.15568 |
| Joint Entropy | 3.37199 |
| KL Divergence | 0.00914 |
| Kappa | 0.79933 |
| Kappa 95% CI | (0.79137,0.8073) |
| Kappa No Prevalence | 0.68863 |
| Kappa Standard Error | 0.00406 |
| Kappa Unbiased | 0.79922 |
| Krippendorff Alpha | 0.79923 |
| Lambda A | 0.75406 |
| Lambda B | 0.74265 |
| Mutual Information | 1.53835 |
| NIR | 0.36697 |
| Overall ACC | 0.84432 |
| Overall CEN | 0.24175 |
| Overall J | (4.93985,0.70569) |
| Overall MCC | 0.80029 |
| Overall MCEN | 0.36044 |
| Overall RACC | 0.22416 |
| Overall RACCU | 0.22459 |
| P-Value | None |
| PPV Macro | 0.84126 |
| PPV Micro | 0.84432 |
| Pearson C | 0.89097 |
| Phi-Squared | 3.85017 |
| RCI | 0.61848 |
| RR | 1888.42857 |
| Reference Entropy | 2.48731 |
| Response Entropy | 2.42304 |
| SOA1(Landis & Koch) | Substantial |
| SOA2(Fleiss) | Excellent |
| SOA3(Altman) | Good |
| SOA4(Cicchetti) | Excellent |
| SOA5(Cramer) | Very Strong |
| SOA6(Matthews) | Strong |
| Scott PI | 0.79922 |
| Standard Error | 0.00315 |
| TNR Macro | 0.97066 |
| TNR Micro | 0.97405 |
| TPR Macro | 0.81405 |
| TPR Micro | 0.84432 |
| Zero-one Loss | 2058 |

## Class Statistics :

|  |  |  |  |  |  |  |  |  |
| --- | --- | --- | --- | --- | --- | --- | --- | --- |
| Class | 1 | 2 | 3 | 4 | 5 | 6 | 7 | Description |
| ACC | 0.94652 | 0.90688 | 0.93827 | 0.96127 | 0.97731 | 0.97254 | 0.98585 | Accuracy |
| AGF | 0.79182 | 0.91736 | 0.90786 | 0.86144 | 0.91314 | 0.934 | 0.90705 | Adjusted F-score |
| AGM | 0.87744 | 0.90644 | 0.92994 | 0.91739 | 0.94982 | 0.95767 | 0.94766 | Adjusted geometric mean |
| AM | -395 | 371 | 84 | -32 | 34 | -35 | -27 | Difference between automatic and manual classification |
| AUC | 0.80166 | 0.90782 | 0.90915 | 0.86724 | 0.91817 | 0.93462 | 0.90781 | Area under the ROC curve |
| AUCI | Very Good | Excellent | Excellent | Very Good | Excellent | Excellent | Excellent | AUC value interpretation |
| AUPR | 0.73343 | 0.87898 | 0.84746 | 0.76552 | 0.83418 | 0.89273 | 0.8413 | Area under the PR curve |
| BB | 0.61656 | 0.84661 | 0.83413 | 0.75429 | 0.81848 | 0.88349 | 0.82196 | Braun-Blanquet similarity |
| BCD | 0.01494 | 0.01403 | 0.00318 | 0.00121 | 0.00129 | 0.00132 | 0.00102 | Bray-Curtis dissimilarity |
| BM | 0.60332 | 0.81564 | 0.81829 | 0.73448 | 0.83635 | 0.86924 | 0.81562 | Informedness or bookmaker informedness |
| CEN | 0.33827 | 0.20972 | 0.24532 | 0.34626 | 0.2636 | 0.1831 | 0.23763 | Confusion entropy |
| DOR | 119.83615 | 97.12774 | 139.32495 | 151.85539 | 412.4532 | 524.65446 | 723.5715 | Diagnostic odds ratio |
| DP | 1.14599 | 1.09568 | 1.18207 | 1.20269 | 1.44193 | 1.49954 | 1.57651 | Discriminant power |
| DPI | Limited | Limited | Limited | Limited | Limited | Limited | Limited | Discriminant power interpretation |
| ERR | 0.05348 | 0.09312 | 0.06173 | 0.03873 | 0.02269 | 0.02746 | 0.01415 | Error rate |
| F0.5 | 0.79037 | 0.85881 | 0.83933 | 0.77215 | 0.82457 | 0.89821 | 0.85261 | F0.5 score |
| F1 | 0.7148 | 0.87779 | 0.84725 | 0.76535 | 0.83389 | 0.89264 | 0.84085 | F1 score - harmonic mean of precision and sensitivity |
| F2 | 0.65243 | 0.89763 | 0.85532 | 0.75868 | 0.84341 | 0.88713 | 0.82942 | F2 score |
| FDR | 0.14971 | 0.15339 | 0.16587 | 0.22326 | 0.18152 | 0.09803 | 0.13937 | False discovery rate |
| FN | 551 | 430 | 366 | 272 | 133 | 199 | 107 | False negative/miss/type 2 error |
| FNR | 0.38344 | 0.08864 | 0.13922 | 0.24571 | 0.15011 | 0.11651 | 0.17804 | Miss rate or false negative rate |
| FOR | 0.04525 | 0.05377 | 0.03484 | 0.0224 | 0.01081 | 0.01724 | 0.00846 | False omission rate |
| FP | 156 | 801 | 450 | 240 | 167 | 164 | 80 | False positive/type 1 error/false alarm |
| FPR | 0.01324 | 0.09572 | 0.04249 | 0.01982 | 0.01354 | 0.01425 | 0.00634 | Fall-out or false positive rate |
| G | 0.72405 | 0.87839 | 0.84735 | 0.76544 | 0.83403 | 0.89268 | 0.84107 | G-measure geometric mean of precision and sensitivity |
| GI | 0.60332 | 0.81564 | 0.81829 | 0.73448 | 0.83635 | 0.86924 | 0.81562 | Gini index |
| GM | 0.78 | 0.90781 | 0.90786 | 0.85985 | 0.91563 | 0.93322 | 0.90374 | G-mean geometric mean of specificity and sensitivity |
| HD | 707 | 1231 | 816 | 512 | 300 | 363 | 187 | Hamming distance |
| IBA | 0.38317 | 0.82996 | 0.74449 | 0.57233 | 0.72388 | 0.78184 | 0.67652 | Index of balanced accuracy |
| ICSI | 0.46685 | 0.75797 | 0.69492 | 0.53104 | 0.66837 | 0.78546 | 0.68259 | Individual classification success index |
| IS | 2.9675 | 1.20603 | 2.06837 | 3.2134 | 3.61018 | 2.80339 | 4.24256 | Information score |
| J | 0.55618 | 0.7822 | 0.73498 | 0.6199 | 0.7151 | 0.80609 | 0.7254 | Jaccard index |
| LS | 7.82182 | 2.30702 | 4.19414 | 9.27532 | 12.21158 | 6.98078 | 18.9295 | Lift score |
| MCC | 0.69692 | 0.80416 | 0.80874 | 0.74434 | 0.82188 | 0.87696 | 0.83369 | Matthews correlation coefficient |
| MCCI | Moderate | Strong | Strong | Strong | Strong | Strong | Strong | Matthews correlation coefficient interpretation |
| MCEN | 0.44742 | 0.32312 | 0.36685 | 0.48713 | 0.39025 | 0.28456 | 0.34973 | Modified confusion entropy |
| MK | 0.80504 | 0.79284 | 0.79929 | 0.75435 | 0.80766 | 0.88474 | 0.85217 | Markedness |
| N | 11782 | 8368 | 10590 | 12112 | 12333 | 11511 | 12618 | Condition negative |
| NLR | 0.38858 | 0.09802 | 0.14539 | 0.25068 | 0.15217 | 0.11819 | 0.17917 | Negative likelihood ratio |
| NLRI | Poor | Good | Fair | Poor | Fair | Fair | Fair | Negative likelihood ratio interpretation |
| NPV | 0.95475 | 0.94623 | 0.96516 | 0.9776 | 0.98919 | 0.98276 | 0.99154 | Negative predictive value |
| OC | 0.85029 | 0.91136 | 0.86078 | 0.77674 | 0.84989 | 0.90197 | 0.86063 | Overlap coefficient |
| OOC | 0.72405 | 0.87839 | 0.84735 | 0.76544 | 0.83403 | 0.89268 | 0.84107 | Otsuka-Ochiai coefficient |
| OP | 0.71562 | 0.90298 | 0.88508 | 0.83103 | 0.90293 | 0.91783 | 0.89129 | Optimized precision |
| P | 1437 | 4851 | 2629 | 1107 | 886 | 1708 | 601 | Condition positive or support |
| PLR | 46.56626 | 9.52091 | 20.25711 | 38.06655 | 62.76442 | 62.01126 | 129.64418 | Positive likelihood ratio |
| PLRI | Good | Fair | Good | Good | Good | Good | Good | Positive likelihood ratio interpretation |
| POP | 13219 | 13219 | 13219 | 13219 | 13219 | 13219 | 13219 | Population |
| PPV | 0.85029 | 0.84661 | 0.83413 | 0.77674 | 0.81848 | 0.90197 | 0.86063 | Precision or positive predictive value |
| PRE | 0.10871 | 0.36697 | 0.19888 | 0.08374 | 0.06702 | 0.12921 | 0.04546 | Prevalence |
| Q | 0.98345 | 0.97962 | 0.98575 | 0.98692 | 0.99516 | 0.9962 | 0.99724 | Yule Q - coefficient of colligation |
| QI | Strong | Strong | Strong | Strong | Strong | Strong | Strong | Yule Q interpretation |
| RACC | 0.00857 | 0.14497 | 0.04082 | 0.00681 | 0.00466 | 0.01635 | 0.00197 | Random accuracy |
| RACCU | 0.00879 | 0.14516 | 0.04083 | 0.00681 | 0.00467 | 0.01635 | 0.00198 | Random accuracy unbiased |
| TN | 11626 | 7567 | 10140 | 11872 | 12166 | 11347 | 12538 | True negative/correct rejection |
| TNR | 0.98676 | 0.90428 | 0.95751 | 0.98018 | 0.98646 | 0.98575 | 0.99366 | Specificity or true negative rate |
| TON | 12177 | 7997 | 10506 | 12144 | 12299 | 11546 | 12645 | Test outcome negative |
| TOP | 1042 | 5222 | 2713 | 1075 | 920 | 1673 | 574 | Test outcome positive |
| TP | 886 | 4421 | 2263 | 835 | 753 | 1509 | 494 | True positive/hit |
| TPR | 0.61656 | 0.91136 | 0.86078 | 0.75429 | 0.84989 | 0.88349 | 0.82196 | Sensitivity, recall, hit rate, or true positive rate |
| Y | 0.60332 | 0.81564 | 0.81829 | 0.73448 | 0.83635 | 0.86924 | 0.81562 | Youden index |
| dInd | 0.38367 | 0.13046 | 0.14556 | 0.24651 | 0.15072 | 0.11738 | 0.17815 | Distance index |
| sInd | 0.72871 | 0.90775 | 0.89708 | 0.82569 | 0.89342 | 0.917 | 0.87403 | Similarity index |

Generated By PyCM Version 3.6
