# Supplementary material for: TopEC: prediction of Enzyme Commission classes by 3D graph neural networks and localized 3D protein descriptor
Source: Nat Commun. 2025 Mar 20;16:2737. doi: 10.1038/s41467-025-57324-5 (PMC11923149; doi:10.1038/s41467-025-57324-5)
Supplement: Supplementary file 3 — Supplementary Data 1 [file 41467_2025_57324_MOESM3_ESM.zip › Data_S1/figure2/AF703PDB300_sub.html]

PyCM Report


# PyCM Report

## Dataset Type :

- Multi-Class Classification
- Imbalanced

Note 1 : Recommended statistics for this type of classification highlighted in aqua

Note 2 : The recommender system assumes that the input is the result of classification over the whole data rather than just a part of it.
If the confusion matrix is the result of test data classification, the recommendation is not valid.

## Confusion Matrix :

|  |  |  |  |  |  |  |  |  |  |  |  |  |  |  |  |  |  |  |  |  |  |  |  |  |  |  |  |  |  |  |  |  |  |  |  |  |  |  |  |  |  |  |  |  |  |  |  |  |  |  |  |  |  |  |  |  |  |  |  |  |  |  |  |  |  |  |  |  |  |  |  |  |  |  |  |  |  |  |  |  |  |  |  |  |  |  |  |  |  |  |  |  |  |  |  |  |  |  |  |  |  |  |  |  |  |  |  |  |  |  |  |  |  |  |  |  |  |  |  |  |  |  |  |  |  |  |  |  |  |  |  |  |  |  |  |  |  |  |  |  |  |  |  |  |  |  |  |  |  |  |  |  |  |  |  |  |  |  |  |  |  |  |  |  |  |  |  |  |  |  |  |  |  |  |  |  |  |  |  |  |  |  |  |  |  |  |  |  |  |  |  |  |  |  |  |  |  |  |  |  |  |  |  |  |  |  |  |  |  |  |  |  |  |  |  |  |  |  |  |  |  |  |  |  |  |  |  |  |  |  |  |  |  |  |  |  |  |  |  |  |  |  |  |  |  |  |  |  |  |  |  |  |  |  |  |  |  |  |  |  |  |  |  |  |  |  |  |  |  |  |  |  |  |  |  |  |  |  |  |  |  |  |  |  |  |  |  |  |  |  |  |  |  |  |  |  |  |  |  |  |  |  |  |  |  |  |  |  |  |  |  |  |  |  |  |  |  |  |  |  |  |  |  |  |  |  |  |  |  |  |  |  |  |  |  |  |  |  |  |  |  |  |  |  |  |  |  |  |  |  |  |  |  |  |  |  |  |  |  |  |  |  |  |  |  |  |  |  |  |  |  |  |  |  |  |  |  |  |  |  |  |  |  |  |  |  |  |  |  |  |  |  |  |  |  |  |  |  |  |  |  |  |  |  |  |  |  |  |  |  |  |  |  |  |  |  |  |  |  |  |  |  |  |  |  |  |  |  |  |  |  |  |  |  |  |  |  |  |  |  |  |  |  |  |  |  |  |  |  |  |  |  |  |  |  |  |  |  |  |  |  |  |  |  |  |  |  |  |  |  |  |  |  |  |  |  |  |  |  |  |  |  |  |  |  |  |  |  |  |  |  |  |  |  |  |  |  |  |  |  |  |  |  |  |  |  |  |  |  |  |  |  |  |  |  |  |  |  |  |  |  |  |  |  |  |  |  |  |  |  |  |  |  |  |  |  |  |  |  |  |  |  |  |  |  |  |  |  |  |  |  |  |  |  |  |  |  |  |  |  |  |  |  |  |  |  |  |  |  |  |  |  |  |  |  |  |  |  |  |  |  |  |  |  |  |  |  |  |  |  |  |  |  |  |  |  |  |  |  |  |  |  |  |  |  |  |  |  |  |  |  |  |  |  |  |  |  |  |  |  |  |  |  |  |  |  |  |  |  |  |  |  |  |  |  |  |  |  |  |  |  |  |  |  |  |  |  |  |  |  |  |  |  |  |  |  |  |  |  |  |  |  |  |  |  |  |  |  |  |  |  |  |  |  |  |  |  |  |  |  |  |  |  |  |  |  |  |  |  |  |  |  |  |  |  |  |  |  |  |  |  |  |  |  |  |  |  |  |  |  |  |  |  |  |  |  |  |  |  |  |  |  |  |  |  |  |  |  |  |  |  |  |  |  |  |  |  |  |  |  |  |  |  |  |  |  |  |  |  |  |  |  |  |  |  |  |  |  |  |  |  |  |  |  |  |  |  |  |  |  |  |  |  |  |  |  |  |  |  |  |  |  |  |  |  |  |  |  |  |  |  |  |  |  |  |  |  |  |  |  |  |  |  |  |  |  |  |  |  |  |  |  |  |  |  |  |  |  |  |  |  |  |  |  |  |  |  |  |  |  |  |  |  |  |  |  |  |  |  |  |  |  |  |  |  |  |  |  |  |  |  |  |  |  |  |  |  |  |  |  |  |  |  |  |  |  |  |  |  |  |  |  |  |  |  |  |  |  |  |  |  |  |  |  |  |  |  |  |  |  |  |  |  |  |  |  |  |  |  |  |  |  |  |  |  |  |  |  |  |  |  |  |  |  |  |  |  |  |  |  |  |  |  |  |  |  |  |  |  |  |  |  |  |  |  |  |  |  |  |  |  |  |  |  |  |  |  |  |  |  |  |  |  |  |  |  |  |  |  |  |  |  |  |  |  |  |  |  |  |  |  |  |  |  |  |  |  |  |  |  |  |  |  |  |  |  |  |  |  |  |  |  |  |  |  |  |  |  |  |  |  |  |  |  |  |  |  |  |  |  |  |  |  |  |  |  |  |  |  |  |  |  |  |  |  |  |  |  |  |  |  |  |  |  |  |  |  |  |  |  |  |  |  |  |  |  |  |  |  |  |  |  |  |  |  |  |  |  |  |  |  |  |  |  |  |  |  |  |  |  |  |  |  |  |  |  |  |  |  |  |  |  |  |  |  |  |  |  |  |  |  |  |  |  |  |  |  |  |  |  |  |  |  |  |  |  |  |  |  |  |  |  |  |  |  |  |  |  |  |  |  |  |  |  |  |  |  |  |  |  |  |  |  |  |  |  |  |  |  |  |  |  |  |  |  |  |  |  |  |  |  |  |  |  |  |  |  |  |  |  |  |  |  |  |  |  |  |  |  |  |  |  |  |  |  |  |  |  |  |  |  |  |  |  |  |  |  |  |  |  |  |  |  |  |  |  |  |  |  |  |  |  |  |  |  |  |  |  |  |  |  |  |  |  |  |  |  |  |  |  |  |  |  |  |  |  |  |  |  |  |  |  |  |  |  |  |  |  |  |  |  |  |  |  |  |  |  |  |  |  |  |  |  |  |  |  |  |  |  |  |  |  |  |  |  |  |  |  |  |  |  |  |  |  |  |  |  |  |  |  |  |  |  |  |  |  |  |  |  |  |  |  |  |  |  |  |  |  |  |  |  |  |  |  |  |  |  |  |  |  |  |  |  |  |  |  |  |  |  |  |  |  |  |  |  |  |  |  |  |  |  |  |  |  |  |  |  |  |  |  |  |  |  |  |  |  |  |  |  |  |  |  |  |  |  |  |  |  |  |  |  |  |  |  |  |  |  |  |  |  |  |  |  |  |  |  |  |  |  |  |  |  |  |  |  |  |  |  |  |  |  |  |  |  |  |  |  |  |  |  |  |  |  |  |  |  |  |  |  |  |  |  |  |  |  |  |  |  |  |  |  |  |  |  |  |  |  |  |  |  |  |  |  |  |  |  |  |  |  |  |  |  |  |  |  |  |  |  |  |  |  |  |  |  |  |  |  |  |  |  |  |  |  |  |  |  |  |  |  |  |  |  |  |  |  |  |  |  |  |  |  |  |  |  |  |  |  |  |  |  |  |  |  |  |  |  |  |  |  |  |  |  |  |  |  |  |  |  |  |  |  |  |  |  |  |  |  |  |  |  |  |  |  |  |  |  |  |  |  |  |  |  |  |  |  |  |  |  |  |  |  |  |  |  |  |  |  |  |  |  |  |  |  |  |  |  |  |  |  |  |  |  |  |  |  |  |  |  |  |  |  |  |  |  |  |  |  |  |  |  |  |  |  |  |  |  |  |  |  |  |  |  |  |  |  |  |  |  |  |  |  |  |  |  |  |  |  |  |  |  |  |  |  |  |  |  |  |  |  |  |  |  |  |  |  |  |  |  |  |  |  |  |  |  |  |  |  |  |  |  |  |  |  |  |  |  |  |  |  |  |  |  |  |  |  |  |  |  |  |  |  |  |  |  |  |  |  |  |  |  |  |  |  |  |  |  |  |  |  |  |  |  |  |  |  |  |  |  |  |  |  |  |  |  |  |  |  |  |  |  |  |  |  |  |  |  |  |  |  |  |  |  |  |  |  |  |  |  |  |  |  |  |  |  |  |  |  |  |  |  |  |  |  |  |  |  |  |  |  |  |  |  |  |  |  |  |  |  |  |  |  |  |  |  |  |  |  |  |  |  |  |  |  |  |  |  |  |  |  |  |  |  |  |  |  |  |  |  |  |  |  |  |  |  |  |  |  |  |  |  |  |  |  |  |  |  |  |  |  |  |  |  |  |  |  |  |  |  |  |  |  |  |  |  |  |  |  |  |  |  |  |  |  |  |  |  |  |  |  |  |  |  |  |  |  |  |  |  |  |  |  |  |  |  |  |  |  |  |  |  |  |  |  |  |  |  |  |  |  |  |  |  |  |  |  |  |  |  |  |  |  |  |  |  |  |  |  |  |  |  |  |  |  |  |  |  |  |  |  |  |  |  |  |  |  |  |  |  |  |  |  |  |  |  |  |  |  |  |  |  |  |  |  |  |  |  |  |  |  |  |  |  |  |  |  |  |  |  |  |  |  |  |  |  |  |  |  |  |  |  |  |  |  |  |  |  |  |  |  |  |  |  |  |  |  |  |  |  |  |  |  |  |  |  |  |  |  |  |  |  |  |  |  |  |  |  |  |  |  |  |  |  |  |  |  |  |  |  |  |  |  |  |  |  |  |  |  |  |  |  |  |  |  |  |  |  |  |  |  |  |  |  |  |  |  |  |  |  |  |  |  |  |  |  |  |  |  |  |  |  |  |  |  |  |  |  |  |  |  |  |  |  |  |  |  |  |  |  |  |  |  |  |  |  |  |  |  |  |  |  |  |  |  |  |  |  |  |  |  |  |  |  |  |  |  |  |  |  |  |  |  |  |  |  |  |  |  |  |  |  |  |  |  |  |  |  |  |  |  |  |  |  |  |  |  |  |  |  |  |  |  |  |  |  |  |  |  |  |  |  |  |  |  |  |  |  |  |  |  |  |  |  |  |  |  |  |  |  |  |  |  |  |  |  |  |  |  |  |  |  |  |  |  |  |  |  |  |  |  |  |  |  |  |  |  |  |  |  |  |  |  |  |  |  |  |  |  |  |  |  |  |  |  |  |  |  |  |  |  |  |  |  |  |  |  |  |  |  |  |  |  |  |  |  |  |  |  |  |  |  |
| --- | --- | --- | --- | --- | --- | --- | --- | --- | --- | --- | --- | --- | --- | --- | --- | --- | --- | --- | --- | --- | --- | --- | --- | --- | --- | --- | --- | --- | --- | --- | --- | --- | --- | --- | --- | --- | --- | --- | --- | --- | --- | --- | --- | --- | --- | --- | --- | --- | --- | --- | --- | --- | --- | --- | --- | --- | --- | --- | --- | --- | --- | --- | --- | --- | --- | --- | --- | --- | --- | --- | --- | --- | --- | --- | --- | --- | --- | --- | --- | --- | --- | --- | --- | --- | --- | --- | --- | --- | --- | --- | --- | --- | --- | --- | --- | --- | --- | --- | --- | --- | --- | --- | --- | --- | --- | --- | --- | --- | --- | --- | --- | --- | --- | --- | --- | --- | --- | --- | --- | --- | --- | --- | --- | --- | --- | --- | --- | --- | --- | --- | --- | --- | --- | --- | --- | --- | --- | --- | --- | --- | --- | --- | --- | --- | --- | --- | --- | --- | --- | --- | --- | --- | --- | --- | --- | --- | --- | --- | --- | --- | --- | --- | --- | --- | --- | --- | --- | --- | --- | --- | --- | --- | --- | --- | --- | --- | --- | --- | --- | --- | --- | --- | --- | --- | --- | --- | --- | --- | --- | --- | --- | --- | --- | --- | --- | --- | --- | --- | --- | --- | --- | --- | --- | --- | --- | --- | --- | --- | --- | --- | --- | --- | --- | --- | --- | --- | --- | --- | --- | --- | --- | --- | --- | --- | --- | --- | --- | --- | --- | --- | --- | --- | --- | --- | --- | --- | --- | --- | --- | --- | --- | --- | --- | --- | --- | --- | --- | --- | --- | --- | --- | --- | --- | --- | --- | --- | --- | --- | --- | --- | --- | --- | --- | --- | --- | --- | --- | --- | --- | --- | --- | --- | --- | --- | --- | --- | --- | --- | --- | --- | --- | --- | --- | --- | --- | --- | --- | --- | --- | --- | --- | --- | --- | --- | --- | --- | --- | --- | --- | --- | --- | --- | --- | --- | --- | --- | --- | --- | --- | --- | --- | --- | --- | --- | --- | --- | --- | --- | --- | --- | --- | --- | --- | --- | --- | --- | --- | --- | --- | --- | --- | --- | --- | --- | --- | --- | --- | --- | --- | --- | --- | --- | --- | --- | --- | --- | --- | --- | --- | --- | --- | --- | --- | --- | --- | --- | --- | --- | --- | --- | --- | --- | --- | --- | --- | --- | --- | --- | --- | --- | --- | --- | --- | --- | --- | --- | --- | --- | --- | --- | --- | --- | --- | --- | --- | --- | --- | --- | --- | --- | --- | --- | --- | --- | --- | --- | --- | --- | --- | --- | --- | --- | --- | --- | --- | --- | --- | --- | --- | --- | --- | --- | --- | --- | --- | --- | --- | --- | --- | --- | --- | --- | --- | --- | --- | --- | --- | --- | --- | --- | --- | --- | --- | --- | --- | --- | --- | --- | --- | --- | --- | --- | --- | --- | --- | --- | --- | --- | --- | --- | --- | --- | --- | --- | --- | --- | --- | --- | --- | --- | --- | --- | --- | --- | --- | --- | --- | --- | --- | --- | --- | --- | --- | --- | --- | --- | --- | --- | --- | --- | --- | --- | --- | --- | --- | --- | --- | --- | --- | --- | --- | --- | --- | --- | --- | --- | --- | --- | --- | --- | --- | --- | --- | --- | --- | --- | --- | --- | --- | --- | --- | --- | --- | --- | --- | --- | --- | --- | --- | --- | --- | --- | --- | --- | --- | --- | --- | --- | --- | --- | --- | --- | --- | --- | --- | --- | --- | --- | --- | --- | --- | --- | --- | --- | --- | --- | --- | --- | --- | --- | --- | --- | --- | --- | --- | --- | --- | --- | --- | --- | --- | --- | --- | --- | --- | --- | --- | --- | --- | --- | --- | --- | --- | --- | --- | --- | --- | --- | --- | --- | --- | --- | --- | --- | --- | --- | --- | --- | --- | --- | --- | --- | --- | --- | --- | --- | --- | --- | --- | --- | --- | --- | --- | --- | --- | --- | --- | --- | --- | --- | --- | --- | --- | --- | --- | --- | --- | --- | --- | --- | --- | --- | --- | --- | --- | --- | --- | --- | --- | --- | --- | --- | --- | --- | --- | --- | --- | --- | --- | --- | --- | --- | --- | --- | --- | --- | --- | --- | --- | --- | --- | --- | --- | --- | --- | --- | --- | --- | --- | --- | --- | --- | --- | --- | --- | --- | --- | --- | --- | --- | --- | --- | --- | --- | --- | --- | --- | --- | --- | --- | --- | --- | --- | --- | --- | --- | --- | --- | --- | --- | --- | --- | --- | --- | --- | --- | --- | --- | --- | --- | --- | --- | --- | --- | --- | --- | --- | --- | --- | --- | --- | --- | --- | --- | --- | --- | --- | --- | --- | --- | --- | --- | --- | --- | --- | --- | --- | --- | --- | --- | --- | --- | --- | --- | --- | --- | --- | --- | --- | --- | --- | --- | --- | --- | --- | --- | --- | --- | --- | --- | --- | --- | --- | --- | --- | --- | --- | --- | --- | --- | --- | --- | --- | --- | --- | --- | --- | --- | --- | --- | --- | --- | --- | --- | --- | --- | --- | --- | --- | --- | --- | --- | --- | --- | --- | --- | --- | --- | --- | --- | --- | --- | --- | --- | --- | --- | --- | --- | --- | --- | --- | --- | --- | --- | --- | --- | --- | --- | --- | --- | --- | --- | --- | --- | --- | --- | --- | --- | --- | --- | --- | --- | --- | --- | --- | --- | --- | --- | --- | --- | --- | --- | --- | --- | --- | --- | --- | --- | --- | --- | --- | --- | --- | --- | --- | --- | --- | --- | --- | --- | --- | --- | --- | --- | --- | --- | --- | --- | --- | --- | --- | --- | --- | --- | --- | --- | --- | --- | --- | --- | --- | --- | --- | --- | --- | --- | --- | --- | --- | --- | --- | --- | --- | --- | --- | --- | --- | --- | --- | --- | --- | --- | --- | --- | --- | --- | --- | --- | --- | --- | --- | --- | --- | --- | --- | --- | --- | --- | --- | --- | --- | --- | --- | --- | --- | --- | --- | --- | --- | --- | --- | --- | --- | --- | --- | --- | --- | --- | --- | --- | --- | --- | --- | --- | --- | --- | --- | --- | --- | --- | --- | --- | --- | --- | --- | --- | --- | --- | --- | --- | --- | --- | --- | --- | --- | --- | --- | --- | --- | --- | --- | --- | --- | --- | --- | --- | --- | --- | --- | --- | --- | --- | --- | --- | --- | --- | --- | --- | --- | --- | --- | --- | --- | --- | --- | --- | --- | --- | --- | --- | --- | --- | --- | --- | --- | --- | --- | --- | --- | --- | --- | --- | --- | --- | --- | --- | --- | --- | --- | --- | --- | --- | --- | --- | --- | --- | --- | --- | --- | --- | --- | --- | --- | --- | --- | --- | --- | --- | --- | --- | --- | --- | --- | --- | --- | --- | --- | --- | --- | --- | --- | --- | --- | --- | --- | --- | --- | --- | --- | --- | --- | --- | --- | --- | --- | --- | --- | --- | --- | --- | --- | --- | --- | --- | --- | --- | --- | --- | --- | --- | --- | --- | --- | --- | --- | --- | --- | --- | --- | --- | --- | --- | --- | --- | --- | --- | --- | --- | --- | --- | --- | --- | --- | --- | --- | --- | --- | --- | --- | --- | --- | --- | --- | --- | --- | --- | --- | --- | --- | --- | --- | --- | --- | --- | --- | --- | --- | --- | --- | --- | --- | --- | --- | --- | --- | --- | --- | --- | --- | --- | --- | --- | --- | --- | --- | --- | --- | --- | --- | --- | --- | --- | --- | --- | --- | --- | --- | --- | --- | --- | --- | --- | --- | --- | --- | --- | --- | --- | --- | --- | --- | --- | --- | --- | --- | --- | --- | --- | --- | --- | --- | --- | --- | --- | --- | --- | --- | --- | --- | --- | --- | --- | --- | --- | --- | --- | --- | --- | --- | --- | --- | --- | --- | --- | --- | --- | --- | --- | --- | --- | --- | --- | --- | --- | --- | --- | --- | --- | --- | --- | --- | --- | --- | --- | --- | --- | --- | --- | --- | --- | --- | --- | --- | --- | --- | --- | --- | --- | --- | --- | --- | --- | --- | --- | --- | --- | --- | --- | --- | --- | --- | --- | --- | --- | --- | --- | --- | --- | --- | --- | --- | --- | --- | --- | --- | --- | --- | --- | --- | --- | --- | --- | --- | --- | --- | --- | --- | --- | --- | --- | --- | --- | --- | --- | --- | --- | --- | --- | --- | --- | --- | --- | --- | --- | --- | --- | --- | --- | --- | --- | --- | --- | --- | --- | --- | --- | --- | --- | --- | --- | --- | --- | --- | --- | --- | --- | --- | --- | --- | --- | --- | --- | --- | --- | --- | --- | --- | --- | --- | --- | --- | --- | --- | --- | --- | --- | --- | --- | --- | --- | --- | --- | --- | --- | --- | --- | --- | --- | --- | --- | --- | --- | --- | --- | --- | --- | --- | --- | --- | --- | --- | --- | --- | --- | --- | --- | --- | --- | --- | --- | --- | --- | --- | --- | --- | --- | --- | --- | --- | --- | --- | --- | --- | --- | --- | --- | --- | --- | --- | --- | --- | --- | --- | --- | --- | --- | --- | --- | --- | --- | --- | --- | --- | --- | --- | --- | --- | --- | --- | --- | --- | --- | --- | --- | --- | --- | --- | --- | --- | --- | --- | --- | --- | --- | --- | --- | --- | --- | --- | --- | --- | --- | --- | --- | --- | --- | --- | --- | --- | --- | --- | --- | --- | --- | --- | --- | --- | --- | --- | --- | --- | --- | --- | --- | --- | --- | --- | --- | --- | --- | --- | --- | --- | --- | --- | --- | --- | --- | --- | --- | --- | --- | --- | --- | --- | --- | --- | --- | --- | --- | --- | --- | --- | --- | --- | --- | --- | --- | --- | --- | --- | --- | --- | --- | --- | --- | --- | --- | --- | --- | --- | --- | --- | --- | --- | --- | --- | --- | --- | --- | --- | --- | --- | --- | --- | --- | --- | --- | --- | --- | --- | --- | --- | --- | --- | --- | --- | --- | --- | --- | --- | --- | --- | --- | --- | --- | --- | --- | --- | --- | --- | --- | --- | --- | --- | --- | --- | --- | --- | --- | --- | --- | --- | --- | --- | --- | --- | --- | --- | --- | --- | --- | --- | --- | --- | --- | --- | --- | --- | --- | --- | --- | --- | --- | --- | --- | --- | --- | --- | --- | --- | --- | --- | --- | --- | --- | --- | --- | --- | --- | --- | --- | --- | --- | --- | --- | --- | --- | --- | --- | --- | --- | --- | --- | --- | --- | --- | --- | --- | --- | --- | --- | --- | --- | --- | --- | --- | --- | --- | --- | --- | --- | --- | --- | --- | --- | --- | --- | --- | --- | --- | --- | --- | --- | --- | --- | --- | --- | --- | --- | --- | --- | --- | --- | --- | --- | --- | --- | --- | --- | --- | --- | --- | --- | --- | --- | --- | --- | --- | --- | --- | --- | --- | --- | --- | --- | --- | --- | --- | --- | --- | --- | --- | --- | --- | --- | --- | --- | --- | --- | --- | --- | --- | --- | --- | --- | --- | --- | --- | --- | --- | --- | --- | --- | --- | --- | --- | --- | --- | --- | --- | --- | --- | --- | --- | --- | --- | --- | --- | --- | --- | --- | --- | --- | --- | --- | --- | --- | --- | --- | --- | --- | --- | --- | --- | --- | --- | --- | --- | --- | --- | --- | --- | --- | --- | --- | --- | --- | --- | --- | --- | --- | --- | --- | --- | --- | --- | --- | --- | --- | --- | --- | --- | --- | --- | --- | --- | --- | --- | --- | --- | --- | --- | --- | --- | --- | --- | --- | --- | --- | --- | --- | --- | --- | --- | --- | --- | --- | --- | --- | --- | --- | --- | --- | --- | --- | --- | --- | --- | --- | --- | --- | --- | --- | --- | --- | --- | --- | --- | --- | --- | --- | --- | --- | --- | --- | --- | --- | --- | --- | --- | --- | --- | --- | --- | --- | --- | --- | --- | --- | --- | --- | --- | --- | --- | --- | --- | --- | --- | --- | --- | --- | --- | --- | --- | --- | --- | --- | --- | --- | --- | --- | --- | --- | --- | --- | --- | --- | --- | --- | --- | --- | --- | --- | --- | --- | --- | --- | --- | --- | --- | --- | --- | --- | --- | --- | --- | --- | --- | --- | --- | --- | --- | --- | --- | --- | --- | --- | --- | --- | --- | --- | --- | --- | --- | --- | --- | --- | --- | --- | --- | --- | --- | --- | --- | --- | --- | --- | --- | --- | --- | --- | --- | --- | --- | --- | --- | --- | --- | --- | --- | --- | --- | --- | --- | --- | --- | --- | --- | --- | --- | --- | --- | --- | --- | --- | --- | --- | --- | --- | --- | --- | --- | --- | --- | --- | --- | --- | --- | --- | --- | --- | --- | --- | --- | --- | --- | --- | --- | --- | --- | --- | --- | --- | --- | --- | --- | --- | --- | --- | --- | --- | --- | --- | --- | --- | --- | --- | --- | --- | --- | --- | --- | --- | --- | --- | --- | --- | --- | --- | --- | --- | --- | --- | --- | --- | --- | --- | --- | --- | --- | --- | --- | --- | --- | --- | --- | --- | --- | --- | --- | --- | --- | --- | --- | --- | --- | --- | --- | --- | --- | --- | --- | --- | --- | --- | --- | --- | --- | --- | --- | --- | --- | --- | --- | --- | --- | --- | --- | --- | --- | --- | --- | --- | --- | --- | --- | --- | --- | --- | --- | --- | --- | --- | --- | --- | --- | --- | --- | --- | --- | --- | --- | --- | --- | --- | --- | --- | --- | --- | --- | --- | --- | --- | --- | --- | --- | --- | --- | --- | --- | --- | --- | --- | --- | --- | --- | --- | --- | --- | --- | --- | --- | --- | --- | --- | --- | --- | --- | --- | --- | --- | --- | --- | --- | --- | --- | --- | --- | --- | --- | --- | --- | --- | --- | --- | --- | --- | --- | --- | --- | --- | --- | --- | --- | --- | --- | --- | --- | --- | --- | --- | --- | --- | --- | --- | --- | --- | --- | --- | --- | --- | --- | --- | --- | --- | --- | --- | --- | --- | --- | --- | --- | --- | --- | --- | --- | --- | --- | --- | --- | --- | --- | --- | --- | --- | --- | --- | --- | --- | --- | --- | --- | --- | --- | --- | --- | --- | --- | --- | --- | --- | --- | --- | --- | --- | --- | --- | --- | --- | --- | --- | --- | --- | --- | --- | --- | --- | --- | --- | --- | --- | --- | --- | --- | --- | --- | --- | --- | --- | --- | --- | --- | --- | --- | --- | --- | --- | --- | --- | --- | --- | --- | --- | --- | --- | --- | --- | --- | --- |
| Actual | Predict  |  |  |  |  |  |  |  |  |  |  |  |  |  |  |  |  |  |  |  |  |  |  |  |  |  |  |  |  |  |  |  |  |  |  |  |  |  |  |  |  |  |  |  |  |  |  |  | | --- | --- | --- | --- | --- | --- | --- | --- | --- | --- | --- | --- | --- | --- | --- | --- | --- | --- | --- | --- | --- | --- | --- | --- | --- | --- | --- | --- | --- | --- | --- | --- | --- | --- | --- | --- | --- | --- | --- | --- | --- | --- | --- | --- | --- | --- | --- | |  | 1.1 | 1.11 | 1.14 | 1.15 | 1.17 | 1.18 | 1.2 | 1.3 | 1.4 | 1.5 | 1.6 | 1.7 | 1.8 | 1.9 | 2.1 | 2.2 | 2.3 | 2.4 | 2.5 | 2.6 | 2.7 | 2.8 | 3.1 | 3.2 | 3.4 | 3.5 | 3.6 | 3.7 | 4.1 | 4.2 | 4.3 | 4.6 | 5.1 | 5.2 | 5.3 | 5.4 | 5.5 | 5.6 | 5.99 | 6.1 | 6.2 | 6.3 | 6.5 | 7.1 | 7.2 | 7.4 | | 1.1 | 18 | 0 | 0 | 0 | 0 | 0 | 0 | 0 | 0 | 1 | 0 | 0 | 0 | 0 | 0 | 0 | 0 | 0 | 0 | 0 | 0 | 0 | 0 | 1 | 0 | 0 | 0 | 0 | 0 | 0 | 1 | 0 | 1 | 0 | 0 | 0 | 0 | 0 | 0 | 0 | 0 | 0 | 0 | 0 | 0 | 0 | | 1.11 | 0 | 4 | 0 | 0 | 0 | 0 | 0 | 0 | 0 | 0 | 0 | 0 | 0 | 0 | 0 | 0 | 0 | 0 | 0 | 0 | 0 | 0 | 0 | 0 | 0 | 0 | 0 | 0 | 0 | 1 | 0 | 0 | 0 | 0 | 0 | 0 | 0 | 0 | 0 | 0 | 0 | 0 | 0 | 0 | 0 | 0 | | 1.14 | 0 | 0 | 2 | 0 | 0 | 0 | 0 | 0 | 0 | 0 | 0 | 0 | 0 | 0 | 1 | 0 | 0 | 0 | 0 | 0 | 0 | 0 | 0 | 0 | 0 | 0 | 0 | 0 | 0 | 0 | 0 | 0 | 0 | 0 | 0 | 0 | 0 | 0 | 0 | 0 | 0 | 0 | 0 | 0 | 0 | 0 | | 1.15 | 0 | 0 | 0 | 0 | 0 | 0 | 0 | 0 | 0 | 0 | 0 | 0 | 0 | 0 | 0 | 0 | 0 | 0 | 0 | 0 | 0 | 0 | 0 | 0 | 0 | 0 | 0 | 0 | 0 | 0 | 0 | 0 | 0 | 0 | 0 | 0 | 0 | 0 | 0 | 0 | 0 | 0 | 0 | 0 | 0 | 0 | | 1.17 | 0 | 0 | 0 | 0 | 2 | 0 | 0 | 0 | 0 | 0 | 0 | 0 | 0 | 0 | 0 | 0 | 0 | 0 | 1 | 0 | 0 | 0 | 0 | 0 | 0 | 0 | 0 | 0 | 0 | 0 | 0 | 0 | 0 | 0 | 0 | 0 | 0 | 0 | 0 | 0 | 0 | 0 | 0 | 0 | 0 | 0 | | 1.18 | 0 | 0 | 0 | 0 | 0 | 3 | 0 | 0 | 0 | 0 | 0 | 0 | 0 | 0 | 0 | 0 | 0 | 0 | 0 | 0 | 0 | 0 | 0 | 0 | 0 | 0 | 0 | 0 | 0 | 0 | 0 | 0 | 0 | 0 | 0 | 0 | 0 | 0 | 0 | 0 | 0 | 0 | 0 | 1 | 0 | 0 | | 1.2 | 0 | 0 | 0 | 0 | 0 | 0 | 7 | 0 | 0 | 0 | 0 | 0 | 0 | 0 | 0 | 0 | 0 | 0 | 1 | 0 | 0 | 0 | 0 | 0 | 0 | 0 | 0 | 0 | 0 | 0 | 0 | 0 | 1 | 0 | 1 | 0 | 0 | 0 | 0 | 0 | 0 | 1 | 0 | 0 | 0 | 0 | | 1.3 | 0 | 0 | 0 | 0 | 0 | 0 | 0 | 5 | 0 | 0 | 0 | 0 | 0 | 0 | 0 | 0 | 0 | 0 | 0 | 0 | 0 | 0 | 1 | 0 | 0 | 0 | 0 | 0 | 0 | 0 | 0 | 0 | 0 | 0 | 0 | 0 | 0 | 0 | 0 | 0 | 0 | 0 | 0 | 0 | 0 | 0 | | 1.4 | 0 | 0 | 0 | 0 | 0 | 0 | 0 | 0 | 3 | 0 | 0 | 0 | 0 | 0 | 0 | 0 | 0 | 0 | 0 | 0 | 0 | 0 | 0 | 0 | 0 | 0 | 0 | 0 | 0 | 0 | 0 | 0 | 0 | 0 | 0 | 0 | 0 | 0 | 0 | 0 | 0 | 0 | 0 | 0 | 0 | 0 | | 1.5 | 1 | 0 | 0 | 0 | 0 | 0 | 0 | 0 | 0 | 310 | 0 | 0 | 0 | 0 | 0 | 0 | 0 | 0 | 0 | 0 | 0 | 0 | 0 | 0 | 1 | 0 | 1 | 0 | 0 | 0 | 0 | 0 | 0 | 0 | 0 | 0 | 0 | 0 | 0 | 0 | 0 | 0 | 0 | 0 | 0 | 0 | | 1.6 | 0 | 0 | 0 | 0 | 0 | 0 | 0 | 0 | 0 | 0 | 6 | 0 | 0 | 0 | 0 | 0 | 0 | 0 | 0 | 0 | 0 | 0 | 0 | 0 | 0 | 0 | 0 | 0 | 0 | 0 | 0 | 0 | 0 | 0 | 0 | 0 | 0 | 0 | 0 | 0 | 0 | 0 | 0 | 0 | 0 | 0 | | 1.7 | 0 | 0 | 0 | 0 | 0 | 0 | 0 | 0 | 0 | 0 | 0 | 0 | 0 | 0 | 0 | 0 | 0 | 0 | 0 | 0 | 0 | 0 | 0 | 0 | 0 | 0 | 0 | 0 | 0 | 0 | 0 | 0 | 0 | 0 | 0 | 0 | 0 | 0 | 0 | 0 | 0 | 0 | 0 | 0 | 0 | 0 | | 1.8 | 0 | 0 | 0 | 0 | 0 | 0 | 0 | 0 | 0 | 0 | 0 | 0 | 2 | 0 | 0 | 0 | 0 | 0 | 0 | 0 | 0 | 0 | 0 | 0 | 0 | 0 | 0 | 0 | 0 | 0 | 0 | 0 | 0 | 0 | 0 | 0 | 0 | 0 | 0 | 0 | 0 | 0 | 0 | 0 | 0 | 0 | | 1.9 | 0 | 0 | 0 | 0 | 0 | 0 | 0 | 0 | 0 | 0 | 0 | 0 | 0 | 1 | 0 | 0 | 0 | 0 | 0 | 0 | 0 | 0 | 0 | 0 | 0 | 0 | 0 | 0 | 0 | 0 | 0 | 0 | 0 | 0 | 0 | 0 | 0 | 0 | 0 | 0 | 0 | 0 | 0 | 0 | 0 | 0 | | 2.1 | 0 | 0 | 0 | 0 | 0 | 0 | 0 | 0 | 0 | 0 | 0 | 0 | 0 | 0 | 26 | 0 | 0 | 0 | 0 | 0 | 0 | 0 | 0 | 0 | 0 | 0 | 0 | 0 | 0 | 0 | 0 | 0 | 0 | 0 | 0 | 0 | 0 | 0 | 0 | 0 | 0 | 0 | 0 | 0 | 0 | 0 | | 2.2 | 0 | 0 | 0 | 0 | 0 | 0 | 0 | 0 | 0 | 0 | 0 | 0 | 0 | 0 | 0 | 1 | 1 | 0 | 0 | 0 | 2 | 0 | 0 | 0 | 0 | 0 | 0 | 0 | 0 | 0 | 0 | 0 | 0 | 0 | 0 | 0 | 0 | 0 | 0 | 0 | 0 | 0 | 0 | 0 | 0 | 0 | | 2.3 | 0 | 0 | 0 | 0 | 0 | 0 | 0 | 0 | 0 | 0 | 0 | 0 | 0 | 0 | 0 | 0 | 13 | 0 | 0 | 0 | 1 | 0 | 0 | 0 | 1 | 1 | 0 | 0 | 0 | 0 | 0 | 0 | 0 | 0 | 0 | 0 | 0 | 0 | 0 | 0 | 0 | 0 | 0 | 0 | 0 | 0 | | 2.4 | 0 | 0 | 0 | 0 | 0 | 0 | 0 | 0 | 0 | 0 | 0 | 0 | 0 | 0 | 0 | 0 | 0 | 13 | 0 | 0 | 2 | 0 | 0 | 0 | 0 | 1 | 0 | 0 | 1 | 0 | 0 | 0 | 0 | 0 | 0 | 0 | 0 | 0 | 0 | 0 | 0 | 0 | 0 | 0 | 0 | 0 | | 2.5 | 0 | 0 | 0 | 0 | 0 | 0 | 0 | 0 | 0 | 0 | 0 | 0 | 0 | 0 | 0 | 0 | 0 | 0 | 13 | 0 | 5 | 0 | 0 | 0 | 0 | 0 | 0 | 0 | 0 | 0 | 0 | 0 | 0 | 0 | 0 | 0 | 0 | 0 | 0 | 0 | 0 | 0 | 0 | 0 | 0 | 0 | | 2.6 | 0 | 0 | 0 | 0 | 0 | 0 | 0 | 0 | 0 | 0 | 0 | 0 | 0 | 0 | 0 | 0 | 0 | 0 | 0 | 1 | 0 | 0 | 0 | 0 | 0 | 0 | 0 | 0 | 0 | 0 | 0 | 0 | 0 | 0 | 0 | 0 | 0 | 0 | 0 | 0 | 0 | 0 | 0 | 0 | 0 | 0 | | 2.7 | 0 | 0 | 0 | 0 | 0 | 1 | 0 | 0 | 1 | 0 | 0 | 1 | 0 | 0 | 0 | 1 | 4 | 0 | 1 | 0 | 183 | 1 | 4 | 0 | 3 | 0 | 7 | 0 | 1 | 1 | 1 | 0 | 0 | 0 | 1 | 0 | 0 | 0 | 0 | 1 | 0 | 0 | 0 | 1 | 0 | 0 | | 2.8 | 0 | 0 | 0 | 0 | 0 | 0 | 0 | 0 | 0 | 0 | 0 | 0 | 0 | 0 | 0 | 0 | 0 | 0 | 0 | 0 | 0 | 2 | 0 | 1 | 0 | 0 | 0 | 0 | 0 | 0 | 0 | 0 | 0 | 0 | 0 | 0 | 0 | 0 | 0 | 0 | 0 | 0 | 0 | 0 | 0 | 0 | | 3.1 | 0 | 0 | 0 | 0 | 0 | 0 | 0 | 0 | 0 | 0 | 0 | 0 | 0 | 0 | 0 | 0 | 1 | 0 | 0 | 0 | 0 | 0 | 20 | 1 | 19 | 0 | 0 | 0 | 1 | 0 | 0 | 0 | 0 | 0 | 0 | 0 | 0 | 0 | 0 | 0 | 0 | 0 | 0 | 0 | 0 | 0 | | 3.2 | 0 | 0 | 0 | 0 | 0 | 0 | 0 | 0 | 0 | 0 | 0 | 0 | 0 | 0 | 0 | 0 | 0 | 1 | 0 | 0 | 0 | 0 | 0 | 17 | 0 | 0 | 0 | 0 | 0 | 0 | 0 | 0 | 0 | 0 | 0 | 0 | 0 | 0 | 0 | 0 | 0 | 0 | 0 | 0 | 0 | 0 | | 3.4 | 0 | 4 | 69 | 0 | 0 | 0 | 0 | 0 | 0 | 0 | 0 | 66 | 0 | 0 | 0 | 0 | 2 | 0 | 3 | 0 | 0 | 0 | 0 | 15 | 9 | 5 | 1 | 0 | 0 | 0 | 0 | 0 | 0 | 0 | 0 | 0 | 0 | 0 | 0 | 0 | 0 | 0 | 0 | 0 | 0 | 0 | | 3.5 | 0 | 2 | 0 | 1 | 0 | 0 | 0 | 0 | 0 | 0 | 0 | 0 | 0 | 0 | 0 | 0 | 0 | 0 | 0 | 0 | 1 | 0 | 0 | 1 | 13 | 10 | 0 | 1 | 0 | 12 | 0 | 0 | 0 | 0 | 0 | 0 | 0 | 0 | 0 | 0 | 0 | 0 | 0 | 0 | 0 | 0 | | 3.6 | 0 | 0 | 0 | 0 | 1 | 0 | 0 | 0 | 0 | 0 | 0 | 0 | 0 | 0 | 0 | 0 | 0 | 0 | 0 | 0 | 1 | 0 | 0 | 0 | 0 | 0 | 634 | 0 | 0 | 0 | 0 | 0 | 0 | 0 | 0 | 0 | 0 | 0 | 0 | 0 | 0 | 0 | 0 | 0 | 0 | 0 | | 3.7 | 0 | 0 | 0 | 0 | 0 | 0 | 0 | 0 | 0 | 0 | 0 | 0 | 0 | 0 | 0 | 0 | 0 | 0 | 0 | 0 | 0 | 0 | 0 | 0 | 0 | 0 | 0 | 0 | 0 | 0 | 0 | 0 | 0 | 0 | 0 | 0 | 0 | 0 | 0 | 0 | 0 | 0 | 0 | 0 | 0 | 0 | | 4.1 | 0 | 0 | 0 | 0 | 0 | 0 | 1 | 0 | 0 | 0 | 0 | 0 | 0 | 0 | 0 | 0 | 0 | 0 | 0 | 0 | 0 | 0 | 0 | 0 | 0 | 0 | 0 | 0 | 6 | 0 | 0 | 0 | 0 | 0 | 0 | 0 | 0 | 0 | 0 | 0 | 0 | 0 | 0 | 0 | 0 | 0 | | 4.2 | 0 | 0 | 0 | 0 | 0 | 0 | 0 | 0 | 0 | 0 | 0 | 2 | 0 | 0 | 0 | 0 | 0 | 0 | 0 | 0 | 3 | 0 | 0 | 0 | 0 | 0 | 1 | 0 | 0 | 616 | 0 | 0 | 0 | 0 | 0 | 0 | 0 | 0 | 0 | 0 | 0 | 0 | 0 | 0 | 0 | 0 | | 4.3 | 0 | 0 | 0 | 0 | 0 | 0 | 0 | 0 | 0 | 0 | 0 | 0 | 0 | 0 | 0 | 0 | 0 | 0 | 0 | 0 | 0 | 0 | 0 | 0 | 0 | 0 | 0 | 0 | 0 | 0 | 1 | 0 | 0 | 0 | 0 | 0 | 0 | 0 | 0 | 0 | 0 | 0 | 0 | 0 | 0 | 0 | | 4.6 | 0 | 0 | 0 | 0 | 0 | 0 | 0 | 0 | 0 | 0 | 0 | 0 | 0 | 0 | 0 | 0 | 0 | 0 | 0 | 0 | 2 | 1 | 0 | 0 | 0 | 0 | 0 | 0 | 0 | 0 | 0 | 0 | 0 | 0 | 0 | 0 | 0 | 0 | 0 | 0 | 0 | 0 | 0 | 0 | 0 | 0 | | 5.1 | 0 | 0 | 0 | 0 | 0 | 0 | 0 | 0 | 0 | 0 | 0 | 0 | 0 | 0 | 0 | 0 | 1 | 0 | 0 | 0 | 1 | 0 | 0 | 0 | 0 | 0 | 0 | 0 | 0 | 0 | 0 | 0 | 4 | 0 | 0 | 0 | 0 | 0 | 0 | 0 | 0 | 0 | 0 | 0 | 0 | 0 | | 5.2 | 0 | 0 | 0 | 0 | 0 | 0 | 0 | 0 | 0 | 0 | 0 | 0 | 0 | 0 | 0 | 0 | 0 | 0 | 0 | 0 | 0 | 0 | 1 | 0 | 0 | 0 | 0 | 0 | 0 | 0 | 0 | 0 | 0 | 22 | 0 | 0 | 0 | 0 | 0 | 0 | 0 | 0 | 0 | 0 | 0 | 0 | | 5.3 | 0 | 0 | 0 | 0 | 0 | 0 | 0 | 0 | 0 | 0 | 0 | 0 | 0 | 0 | 0 | 0 | 0 | 0 | 0 | 0 | 0 | 0 | 1 | 0 | 0 | 0 | 0 | 0 | 0 | 0 | 0 | 0 | 0 | 0 | 1 | 0 | 0 | 0 | 0 | 0 | 0 | 0 | 0 | 0 | 0 | 0 | | 5.4 | 0 | 0 | 0 | 0 | 0 | 0 | 0 | 0 | 0 | 0 | 0 | 0 | 0 | 0 | 0 | 0 | 0 | 0 | 0 | 0 | 0 | 0 | 0 | 0 | 0 | 0 | 0 | 0 | 0 | 0 | 0 | 0 | 0 | 0 | 0 | 1 | 0 | 0 | 0 | 0 | 0 | 0 | 0 | 0 | 0 | 0 | | 5.5 | 0 | 0 | 0 | 0 | 0 | 0 | 0 | 0 | 0 | 0 | 0 | 0 | 0 | 0 | 0 | 0 | 0 | 0 | 0 | 0 | 0 | 0 | 0 | 0 | 0 | 0 | 0 | 0 | 0 | 0 | 0 | 0 | 0 | 0 | 0 | 0 | 3 | 0 | 0 | 0 | 0 | 0 | 0 | 0 | 0 | 0 | | 5.6 | 0 | 0 | 0 | 0 | 0 | 0 | 0 | 0 | 0 | 0 | 0 | 0 | 0 | 0 | 0 | 0 | 1 | 0 | 0 | 0 | 0 | 0 | 0 | 0 | 0 | 0 | 0 | 0 | 0 | 0 | 0 | 0 | 0 | 0 | 0 | 0 | 0 | 3 | 1 | 0 | 0 | 0 | 0 | 0 | 0 | 0 | | 5.99 | 0 | 0 | 0 | 0 | 0 | 0 | 0 | 0 | 0 | 0 | 0 | 0 | 0 | 0 | 0 | 0 | 0 | 0 | 0 | 0 | 0 | 0 | 0 | 0 | 0 | 0 | 0 | 0 | 0 | 0 | 0 | 0 | 0 | 0 | 0 | 0 | 0 | 0 | 0 | 0 | 0 | 0 | 0 | 0 | 0 | 0 | | 6.1 | 0 | 0 | 0 | 0 | 0 | 0 | 0 | 0 | 0 | 0 | 0 | 2 | 0 | 0 | 0 | 0 | 0 | 0 | 0 | 0 | 1 | 0 | 0 | 0 | 0 | 0 | 0 | 0 | 0 | 0 | 0 | 0 | 0 | 0 | 0 | 0 | 0 | 2 | 0 | 15 | 0 | 0 | 0 | 0 | 0 | 0 | | 6.2 | 0 | 0 | 0 | 0 | 0 | 0 | 0 | 0 | 0 | 0 | 0 | 0 | 0 | 0 | 0 | 0 | 0 | 0 | 0 | 0 | 0 | 0 | 0 | 0 | 0 | 0 | 0 | 0 | 0 | 0 | 0 | 0 | 0 | 0 | 0 | 0 | 0 | 0 | 0 | 0 | 3 | 0 | 0 | 0 | 0 | 0 | | 6.3 | 1 | 0 | 0 | 0 | 0 | 0 | 0 | 0 | 0 | 0 | 0 | 0 | 0 | 0 | 0 | 0 | 0 | 0 | 1 | 0 | 3 | 0 | 0 | 1 | 0 | 0 | 1 | 0 | 0 | 0 | 0 | 0 | 0 | 0 | 0 | 0 | 0 | 0 | 0 | 0 | 0 | 17 | 0 | 0 | 0 | 0 | | 6.5 | 0 | 0 | 0 | 0 | 0 | 0 | 0 | 0 | 0 | 0 | 0 | 0 | 0 | 0 | 0 | 0 | 0 | 0 | 0 | 0 | 2 | 0 | 0 | 0 | 0 | 0 | 0 | 0 | 0 | 0 | 0 | 0 | 0 | 0 | 0 | 0 | 0 | 0 | 0 | 0 | 0 | 0 | 2 | 0 | 0 | 0 | | 7.1 | 0 | 0 | 0 | 0 | 0 | 0 | 0 | 0 | 0 | 0 | 0 | 0 | 0 | 0 | 0 | 0 | 0 | 0 | 0 | 0 | 0 | 0 | 0 | 0 | 0 | 0 | 0 | 0 | 0 | 0 | 0 | 0 | 0 | 0 | 0 | 0 | 0 | 0 | 0 | 0 | 0 | 0 | 0 | 1 | 0 | 0 | | 7.2 | 0 | 0 | 0 | 0 | 0 | 0 | 0 | 0 | 0 | 0 | 0 | 0 | 0 | 0 | 0 | 0 | 0 | 1 | 0 | 0 | 0 | 0 | 0 | 0 | 1 | 0 | 1 | 0 | 0 | 0 | 0 | 0 | 0 | 0 | 0 | 0 | 0 | 0 | 0 | 0 | 0 | 0 | 0 | 0 | 0 | 0 | | 7.4 | 0 | 0 | 0 | 0 | 0 | 0 | 0 | 0 | 0 | 0 | 0 | 0 | 0 | 0 | 0 | 0 | 0 | 0 | 0 | 0 | 0 | 0 | 0 | 0 | 0 | 0 | 1 | 0 | 0 | 0 | 0 | 0 | 0 | 0 | 0 | 0 | 0 | 0 | 0 | 0 | 0 | 0 | 0 | 0 | 0 | 0 | |

## Overall Statistics :

|  |  |
| --- | --- |
| 95% CI | (0.84919,0.87718) |
| ACC Macro | 0.99405 |
| ARI | 0.93932 |
| AUNP | None |
| AUNU | None |
| Bangdiwala B | 0.95324 |
| Bennett S | 0.86014 |
| CBA | 0.57527 |
| CSI | None |
| Chi-Squared | None |
| Chi-Squared DF | 2025 |
| Conditional Entropy | 0.46352 |
| Cramer V | None |
| Cross Entropy | 3.2477 |
| F1 Macro | 0.62128 |
| F1 Micro | 0.86319 |
| FNR Macro | None |
| FNR Micro | 0.13681 |
| FPR Macro | 0.00313 |
| FPR Micro | 0.00304 |
| Gwet AC1 | 0.86065 |
| Hamming Loss | 0.13681 |
| Joint Entropy | 3.58069 |
| KL Divergence | None |
| Kappa | 0.83341 |
| Kappa 95% CI | (0.81637,0.85045) |
| Kappa No Prevalence | 0.72637 |
| Kappa Standard Error | 0.00869 |
| Kappa Unbiased | 0.83315 |
| Krippendorff Alpha | 0.83319 |
| Lambda A | 0.89827 |
| Lambda B | 0.8509 |
| Mutual Information | 2.73336 |
| NIR | 0.27449 |
| Overall ACC | 0.86319 |
| Overall CEN | 0.06548 |
| Overall J | (24.78247,0.53875) |
| Overall MCC | 0.83601 |
| Overall MCEN | 0.10382 |
| Overall RACC | 0.17873 |
| Overall RACCU | 0.18 |
| P-Value | None |
| PPV Macro | None |
| PPV Micro | 0.86319 |
| Pearson C | None |
| Phi-Squared | None |
| RCI | 0.87687 |
| RR | 50.36957 |
| Reference Entropy | 3.11717 |
| Response Entropy | 3.19688 |
| SOA1(Landis & Koch) | Almost Perfect |
| SOA2(Fleiss) | Excellent |
| SOA3(Altman) | Very Good |
| SOA4(Cicchetti) | Excellent |
| SOA5(Cramer) | None |
| SOA6(Matthews) | Strong |
| Scott PI | 0.83315 |
| Standard Error | 0.00714 |
| TNR Macro | 0.99687 |
| TNR Micro | 0.99696 |
| TPR Macro | None |
| TPR Micro | 0.86319 |
| Zero-one Loss | 317 |

## Class Statistics :

|  |  |  |  |  |  |  |  |  |  |  |  |  |  |  |  |  |  |  |  |  |  |  |  |  |  |  |  |  |  |  |  |  |  |  |  |  |  |  |  |  |  |  |  |  |  |  |  |
| --- | --- | --- | --- | --- | --- | --- | --- | --- | --- | --- | --- | --- | --- | --- | --- | --- | --- | --- | --- | --- | --- | --- | --- | --- | --- | --- | --- | --- | --- | --- | --- | --- | --- | --- | --- | --- | --- | --- | --- | --- | --- | --- | --- | --- | --- | --- | --- |
| Class | 1.1 | 1.11 | 1.14 | 1.15 | 1.17 | 1.18 | 1.2 | 1.3 | 1.4 | 1.5 | 1.6 | 1.7 | 1.8 | 1.9 | 2.1 | 2.2 | 2.3 | 2.4 | 2.5 | 2.6 | 2.7 | 2.8 | 3.1 | 3.2 | 3.4 | 3.5 | 3.6 | 3.7 | 4.1 | 4.2 | 4.3 | 4.6 | 5.1 | 5.2 | 5.3 | 5.4 | 5.5 | 5.6 | 5.99 | 6.1 | 6.2 | 6.3 | 6.5 | 7.1 | 7.2 | 7.4 | Description |
| ACC | 0.99741 | 0.99698 | 0.96979 | 0.99957 | 0.99914 | 0.99914 | 0.99784 | 0.99957 | 0.99957 | 0.99827 | 1.0 | 0.96936 | 1.0 | 1.0 | 0.99957 | 0.99827 | 0.99439 | 0.99741 | 0.99482 | 1.0 | 0.97669 | 0.99871 | 0.98748 | 0.99094 | 0.91239 | 0.9836 | 0.99353 | 0.99957 | 0.99827 | 0.99137 | 0.99914 | 0.99871 | 0.99827 | 0.99957 | 0.99871 | 1.0 | 1.0 | 0.99827 | 0.99957 | 0.99741 | 1.0 | 0.99655 | 0.99914 | 0.99914 | 0.99871 | 0.99957 | Accuracy |
| AGF | 0.91216 | 0.81614 | 0.34598 | 0.0 | 0.81632 | 0.86584 | 0.81981 | 0.92832 | 0.9682 | 0.99519 | 1.0 | 0.0 | 1.0 | 1.0 | 0.99613 | 0.52675 | 0.86354 | 0.88426 | 0.83956 | 1.0 | 0.92316 | 0.79036 | 0.71313 | 0.88214 | 0.23832 | 0.52258 | 0.99545 | 0.0 | 0.90018 | 0.99165 | 0.84508 | 0.0 | 0.81614 | 0.98213 | 0.67403 | 1.0 | 1.0 | 0.77426 | 0.0 | 0.88308 | 1.0 | 0.8624 | 0.7451 | 0.84508 | 0.0 | 0.0 | Adjusted F-score |
| AGM | 0.95141 | 0.94528 | 0.88715 | None | 0.90788 | 0.93265 | 0.89832 | 0.95638 | 0.99968 | 0.99706 | 1.0 | None | 1.0 | 1.0 | 0.99967 | 0.74951 | 0.94737 | 0.93638 | 0.92246 | 1.0 | 0.95349 | 0.90758 | 0.84155 | 0.9794 | 0.58907 | 0.74277 | 0.99359 | None | 0.9619 | 0.99134 | 0.99935 | 0 | 0.90752 | 0.98895 | 0.85291 | 1.0 | 1.0 | 0.88658 | None | 0.93241 | 1.0 | 0.92009 | 0.85343 | 0.99935 | 0 | 0 | Adjusted geometric mean |
| AM | -2 | 5 | 68 | 1 | 0 | 0 | -3 | -1 | 1 | -2 | 0 | 71 | 0 | 0 | 1 | -2 | 7 | -2 | 2 | 0 | -6 | 1 | -15 | 19 | -127 | -24 | 11 | 1 | 2 | 8 | 2 | -3 | 0 | -1 | 1 | 0 | 0 | 0 | 1 | -4 | 0 | -6 | -2 | 2 | -3 | -1 | Difference between automatic and manual classification |
| AUC | 0.90866 | 0.8987 | 0.81842 | None | 0.83312 | 0.87478 | 0.81796 | 0.91667 | 0.99978 | 0.99496 | 1.0 | None | 1.0 | 1.0 | 0.99978 | 0.62478 | 0.90408 | 0.88192 | 0.85959 | 1.0 | 0.92387 | 0.8329 | 0.73656 | 0.96787 | 0.517 | 0.62041 | 0.99456 | None | 0.92792 | 0.99105 | 0.99957 | 0.5 | 0.8329 | 0.97826 | 0.74957 | 1.0 | 1.0 | 0.79957 | None | 0.87478 | 1.0 | 0.85395 | 0.75 | 0.99957 | 0.5 | 0.5 | Area under the ROC curve |
| AUCI | Excellent | Very Good | Very Good | None | Very Good | Very Good | Very Good | Excellent | Excellent | Excellent | Excellent | None | Excellent | Excellent | Excellent | Fair | Excellent | Very Good | Very Good | Excellent | Excellent | Very Good | Good | Excellent | Poor | Fair | Excellent | None | Excellent | Excellent | Excellent | Poor | Very Good | Excellent | Good | Excellent | Excellent | Good | None | Very Good | Excellent | Very Good | Good | Excellent | Poor | Poor | AUC value interpretation |
| AUPR | 0.85909 | 0.6 | 0.34742 | None | 0.66667 | 0.75 | 0.75568 | 0.91667 | 0.875 | 0.9936 | 1.0 | None | 1.0 | 1.0 | 0.98148 | 0.375 | 0.68886 | 0.81569 | 0.68611 | 1.0 | 0.87161 | 0.58333 | 0.60847 | 0.70195 | 0.12161 | 0.41607 | 0.98838 | None | 0.7619 | 0.98407 | 0.66667 | None | 0.66667 | 0.97826 | 0.41667 | 1.0 | 1.0 | 0.6 | None | 0.84375 | 1.0 | 0.82639 | 0.75 | 0.66667 | None | None | Area under the PR curve |
| BB | 0.81818 | 0.4 | 0.02817 | 0.0 | 0.66667 | 0.75 | 0.63636 | 0.83333 | 0.75 | 0.99042 | 1.0 | 0.0 | 1.0 | 1.0 | 0.96296 | 0.25 | 0.56522 | 0.76471 | 0.65 | 1.0 | 0.85915 | 0.5 | 0.47619 | 0.45946 | 0.05172 | 0.2439 | 0.97991 | 0.0 | 0.66667 | 0.97778 | 0.33333 | 0.0 | 0.66667 | 0.95652 | 0.33333 | 1.0 | 1.0 | 0.6 | 0.0 | 0.75 | 1.0 | 0.70833 | 0.5 | 0.33333 | 0.0 | 0.0 | Braun-Blanquet similarity |
| BCD | 0.00043 | 0.00108 | 0.01467 | 0.00022 | 0.0 | 0.0 | 0.00065 | 0.00022 | 0.00022 | 0.00043 | 0.0 | 0.01532 | 0.0 | 0.0 | 0.00022 | 0.00043 | 0.00151 | 0.00043 | 0.00043 | 0.0 | 0.00129 | 0.00022 | 0.00324 | 0.0041 | 0.02741 | 0.00518 | 0.00237 | 0.00022 | 0.00043 | 0.00173 | 0.00043 | 0.00065 | 0.0 | 0.00022 | 0.00022 | 0.0 | 0.0 | 0.0 | 0.00022 | 0.00086 | 0.0 | 0.00129 | 0.00043 | 0.00043 | 0.00065 | 0.00022 | Bray-Curtis dissimilarity |
| BM | 0.81731 | 0.7974 | 0.63685 | None | 0.66623 | 0.74957 | 0.63593 | 0.83333 | 0.99957 | 0.98992 | 1.0 | None | 1.0 | 1.0 | 0.99956 | 0.24957 | 0.80815 | 0.76384 | 0.71918 | 1.0 | 0.84775 | 0.6658 | 0.47311 | 0.93575 | 0.03399 | 0.24083 | 0.98912 | None | 0.85584 | 0.98209 | 0.99914 | 0.0 | 0.6658 | 0.95652 | 0.49914 | 1.0 | 1.0 | 0.59913 | None | 0.74956 | 1.0 | 0.7079 | 0.5 | 0.99914 | 0.0 | 0.0 | Informedness or bookmaker informedness |
| CEN | 0.11866 | 0.17815 | 0.02742 | 0.0 | 0.13273 | 0.11553 | 0.1722 | 0.04844 | 0.06178 | 0.00917 | 0 | 0.07312 | 0 | 0 | 0.01665 | 0.21411 | 0.23189 | 0.13478 | 0.18894 | 0 | 0.14709 | 0.18533 | 0.19743 | 0.1759 | 0.37777 | 0.31304 | 0.01624 | 0.0 | 0.15404 | 0.0192 | 0.15404 | 0.14145 | 0.18407 | 0.0188 | 0.2146 | 0 | 0 | 0.17388 | 0.0 | 0.11561 | 0 | 0.14078 | 0.08138 | 0.15404 | 0.24415 | 0.0 | Confusion entropy |
| DOR | 5159.25 | 1537.33333 | 65.07246 | None | 4626.0 | 6936.0 | 4033.75 | None | None | 206976.66667 | None | None | None | None | None | 770.66667 | 992.76667 | 3734.25 | 851.31429 | None | 528.66667 | 2312.0 | 294.54545 | 1937.15 | 3.02153 | 104.56221 | 40673.53846 | None | 4614.0 | 12327.33333 | None | None | 2309.0 | None | 1156.5 | None | None | 1732.5 | None | 6888.0 | None | 5566.28571 | None | None | None | None | Diagnostic odds ratio |
| DP | 2.04686 | 1.75696 | 0.99978 | None | 2.02073 | 2.11771 | 1.98793 | None | None | 2.93082 | None | None | None | None | None | 1.59161 | 1.65225 | 1.96946 | 1.61544 | None | 1.50137 | 1.85466 | 1.36132 | 1.81231 | 0.26476 | 1.11334 | 2.54125 | None | 2.02011 | 2.25541 | None | None | 1.85435 | None | 1.6888 | None | None | 1.78557 | None | 2.11605 | None | 2.06504 | None | None | None | None | Discriminant power |
| DPI | Fair | Limited | Poor | None | Fair | Fair | Limited | None | None | Fair | None | None | None | None | None | Limited | Limited | Limited | Limited | None | Limited | Limited | Limited | Limited | Poor | Limited | Fair | None | Fair | Fair | None | None | Limited | None | Limited | None | None | Limited | None | Fair | None | Fair | None | None | None | None | Discriminant power interpretation |
| ERR | 0.00259 | 0.00302 | 0.03021 | 0.00043 | 0.00086 | 0.00086 | 0.00216 | 0.00043 | 0.00043 | 0.00173 | 0.0 | 0.03064 | 0.0 | 0.0 | 0.00043 | 0.00173 | 0.00561 | 0.00259 | 0.00518 | 0.0 | 0.02331 | 0.00129 | 0.01252 | 0.00906 | 0.08761 | 0.0164 | 0.00647 | 0.00043 | 0.00173 | 0.00863 | 0.00086 | 0.00129 | 0.00173 | 0.00043 | 0.00129 | 0.0 | 0.0 | 0.00173 | 0.00043 | 0.00259 | 0.0 | 0.00345 | 0.00086 | 0.00086 | 0.00129 | 0.00043 | Error rate |
| F0.5 | 0.88235 | 0.44444 | 0.03484 | 0.0 | 0.66667 | 0.75 | 0.81395 | 0.96154 | 0.78947 | 0.9955 | 1.0 | 0.0 | 1.0 | 1.0 | 0.97015 | 0.41667 | 0.60185 | 0.84416 | 0.66327 | 1.0 | 0.87896 | 0.52632 | 0.66667 | 0.51205 | 0.12431 | 0.45872 | 0.98325 | 0.0 | 0.69767 | 0.98027 | 0.38462 | 0.0 | 0.66667 | 0.99099 | 0.35714 | 1.0 | 1.0 | 0.6 | 0.0 | 0.89286 | 1.0 | 0.88542 | 0.83333 | 0.38462 | 0.0 | 0.0 | F0.5 score |
| F1 | 0.85714 | 0.53333 | 0.05405 | 0.0 | 0.66667 | 0.75 | 0.73684 | 0.90909 | 0.85714 | 0.99359 | 1.0 | 0.0 | 1.0 | 1.0 | 0.98113 | 0.33333 | 0.66667 | 0.8125 | 0.68421 | 1.0 | 0.87143 | 0.57143 | 0.57971 | 0.61818 | 0.08145 | 0.34483 | 0.98831 | 0.0 | 0.75 | 0.98403 | 0.5 | 0.0 | 0.66667 | 0.97778 | 0.4 | 1.0 | 1.0 | 0.6 | 0.0 | 0.83333 | 1.0 | 0.80952 | 0.66667 | 0.5 | 0.0 | 0.0 | F1 score - harmonic mean of precision and sensitivity |
| F2 | 0.83333 | 0.66667 | 0.12048 | 0.0 | 0.66667 | 0.75 | 0.67308 | 0.86207 | 0.9375 | 0.99168 | 1.0 | 0.0 | 1.0 | 1.0 | 0.99237 | 0.27778 | 0.74713 | 0.78313 | 0.70652 | 1.0 | 0.86402 | 0.625 | 0.51282 | 0.77982 | 0.06057 | 0.27624 | 0.99342 | 0.0 | 0.81081 | 0.98781 | 0.71429 | 0.0 | 0.66667 | 0.96491 | 0.45455 | 1.0 | 1.0 | 0.6 | 0.0 | 0.78125 | 1.0 | 0.74561 | 0.55556 | 0.71429 | 0.0 | 0.0 | F2 score |
| FDR | 0.1 | 0.6 | 0.97183 | 1.0 | 0.33333 | 0.25 | 0.125 | 0.0 | 0.25 | 0.00322 | 0.0 | 1.0 | 0.0 | 0.0 | 0.03704 | 0.5 | 0.43478 | 0.13333 | 0.35 | 0.0 | 0.11594 | 0.5 | 0.25926 | 0.54054 | 0.80851 | 0.41176 | 0.02009 | 1.0 | 0.33333 | 0.02222 | 0.66667 | None | 0.33333 | 0.0 | 0.66667 | 0.0 | 0.0 | 0.4 | 1.0 | 0.0625 | 0.0 | 0.05556 | 0.0 | 0.66667 | None | None | False discovery rate |
| FN | 4 | 1 | 1 | 0 | 1 | 1 | 4 | 1 | 0 | 3 | 0 | 0 | 0 | 0 | 0 | 3 | 3 | 4 | 5 | 0 | 30 | 1 | 22 | 1 | 165 | 31 | 2 | 0 | 1 | 6 | 0 | 3 | 2 | 1 | 1 | 0 | 0 | 2 | 0 | 5 | 0 | 7 | 2 | 0 | 3 | 1 | False negative/miss/type 2 error |
| FNR | 0.18182 | 0.2 | 0.33333 | None | 0.33333 | 0.25 | 0.36364 | 0.16667 | 0.0 | 0.00958 | 0.0 | None | 0.0 | 0.0 | 0.0 | 0.75 | 0.1875 | 0.23529 | 0.27778 | 0.0 | 0.14085 | 0.33333 | 0.52381 | 0.05556 | 0.94828 | 0.7561 | 0.00314 | None | 0.14286 | 0.00965 | 0.0 | 1.0 | 0.33333 | 0.04348 | 0.5 | 0.0 | 0.0 | 0.4 | None | 0.25 | 0.0 | 0.29167 | 0.5 | 0.0 | 1.0 | 1.0 | Miss rate or false negative rate |
| FOR | 0.00174 | 0.00043 | 0.00045 | 0.0 | 0.00043 | 0.00043 | 0.00173 | 0.00043 | 0.0 | 0.0015 | 0.0 | 0.0 | 0.0 | 0.0 | 0.0 | 0.0013 | 0.00131 | 0.00174 | 0.00218 | 0.0 | 0.01422 | 0.00043 | 0.00961 | 0.00044 | 0.07269 | 0.01348 | 0.0012 | 0.0 | 0.00043 | 0.00356 | 0.0 | 0.00129 | 0.00087 | 0.00044 | 0.00043 | 0.0 | 0.0 | 0.00087 | 0.0 | 0.00217 | 0.0 | 0.00304 | 0.00086 | 0.0 | 0.00129 | 0.00043 | False omission rate |
| FP | 2 | 6 | 69 | 1 | 1 | 1 | 1 | 0 | 1 | 1 | 0 | 71 | 0 | 0 | 1 | 1 | 10 | 2 | 7 | 0 | 24 | 2 | 7 | 20 | 38 | 7 | 13 | 1 | 3 | 14 | 2 | 0 | 2 | 0 | 2 | 0 | 0 | 2 | 1 | 1 | 0 | 1 | 0 | 2 | 0 | 0 | False positive/type 1 error/false alarm |
| FPR | 0.00087 | 0.0026 | 0.02982 | 0.00043 | 0.00043 | 0.00043 | 0.00043 | 0.0 | 0.00043 | 0.0005 | 0.0 | 0.03064 | 0.0 | 0.0 | 0.00044 | 0.00043 | 0.00435 | 0.00087 | 0.00304 | 0.0 | 0.01141 | 0.00086 | 0.00308 | 0.0087 | 0.01773 | 0.00308 | 0.00773 | 0.00043 | 0.0013 | 0.00826 | 0.00086 | 0.0 | 0.00087 | 0.0 | 0.00086 | 0.0 | 0.0 | 0.00087 | 0.00043 | 0.00044 | 0.0 | 0.00044 | 0.0 | 0.00086 | 0.0 | 0.0 | Fall-out or false positive rate |
| G | 0.85812 | 0.56569 | 0.13704 | None | 0.66667 | 0.75 | 0.7462 | 0.91287 | 0.86603 | 0.99359 | 1.0 | None | 1.0 | 1.0 | 0.98131 | 0.35355 | 0.67767 | 0.81409 | 0.68516 | 1.0 | 0.87152 | 0.57735 | 0.59391 | 0.65874 | 0.09952 | 0.37878 | 0.98834 | None | 0.75593 | 0.98405 | 0.57735 | None | 0.66667 | 0.97802 | 0.40825 | 1.0 | 1.0 | 0.6 | None | 0.83853 | 1.0 | 0.81791 | 0.70711 | 0.57735 | None | None | G-measure geometric mean of precision and sensitivity |
| GI | 0.81731 | 0.7974 | 0.63685 | None | 0.66623 | 0.74957 | 0.63593 | 0.83333 | 0.99957 | 0.98992 | 1.0 | None | 1.0 | 1.0 | 0.99956 | 0.24957 | 0.80815 | 0.76384 | 0.71918 | 1.0 | 0.84775 | 0.6658 | 0.47311 | 0.93575 | 0.03399 | 0.24083 | 0.98912 | None | 0.85584 | 0.98209 | 0.99914 | 0.0 | 0.6658 | 0.95652 | 0.49914 | 1.0 | 1.0 | 0.59913 | None | 0.74956 | 1.0 | 0.7079 | 0.5 | 0.99914 | 0.0 | 0.0 | Gini index |
| GM | 0.90414 | 0.89327 | 0.80423 | None | 0.81632 | 0.86584 | 0.79755 | 0.91287 | 0.99978 | 0.99495 | 1.0 | None | 1.0 | 1.0 | 0.99978 | 0.49989 | 0.89943 | 0.87409 | 0.84854 | 1.0 | 0.9216 | 0.81614 | 0.689 | 0.96759 | 0.2254 | 0.4931 | 0.99456 | None | 0.92522 | 0.99105 | 0.99957 | 0.0 | 0.81614 | 0.97802 | 0.7068 | 1.0 | 1.0 | 0.77426 | None | 0.86584 | 1.0 | 0.84144 | 0.70711 | 0.99957 | 0.0 | 0.0 | G-mean geometric mean of specificity and sensitivity |
| HD | 6 | 7 | 70 | 1 | 2 | 2 | 5 | 1 | 1 | 4 | 0 | 71 | 0 | 0 | 1 | 4 | 13 | 6 | 12 | 0 | 54 | 3 | 29 | 21 | 203 | 38 | 15 | 1 | 4 | 20 | 2 | 3 | 4 | 1 | 3 | 0 | 0 | 4 | 1 | 6 | 0 | 8 | 2 | 2 | 3 | 1 | Hamming distance |
| IBA | 0.66955 | 0.64041 | 0.45048 | None | 0.44454 | 0.56258 | 0.40506 | 0.69444 | 1.0 | 0.98093 | 1.0 | None | 1.0 | 1.0 | 1.0 | 0.06258 | 0.6608 | 0.58493 | 0.52221 | 1.0 | 0.73942 | 0.44464 | 0.22752 | 0.89236 | 0.00353 | 0.06005 | 0.99369 | None | 0.73485 | 0.98081 | 1.0 | 0.0 | 0.44464 | 0.91493 | 0.25022 | 1.0 | 1.0 | 0.36021 | None | 0.56258 | 1.0 | 0.50183 | 0.25 | 1.0 | 0.0 | 0.0 | Index of balanced accuracy |
| ICSI | 0.71818 | 0.2 | -0.30516 | None | 0.33333 | 0.5 | 0.51136 | 0.83333 | 0.75 | 0.9872 | 1.0 | None | 1.0 | 1.0 | 0.96296 | -0.25 | 0.37772 | 0.63137 | 0.37222 | 1.0 | 0.74321 | 0.16667 | 0.21693 | 0.4039 | -0.75679 | -0.16786 | 0.97676 | None | 0.52381 | 0.96813 | 0.33333 | None | 0.33333 | 0.95652 | -0.16667 | 1.0 | 1.0 | 0.2 | None | 0.6875 | 1.0 | 0.65278 | 0.5 | 0.33333 | None | None | Individual classification success index |
| IS | 6.56661 | 7.53419 | 4.44333 | None | 9.00812 | 8.763 | 7.52597 | 8.59308 | 9.17804 | 2.88338 | 8.59308 | None | 10.17804 | 11.17804 | 6.42315 | 8.17804 | 6.35492 | 6.88413 | 6.38663 | 11.17804 | 3.26555 | 8.59308 | 5.35277 | 5.88613 | 1.35043 | 5.05496 | 1.83588 | None | 7.78572 | 1.86485 | 9.59308 | None | 8.00812 | 6.65448 | 8.59308 | 11.17804 | 9.59308 | 8.11915 | None | 6.763 | 9.59308 | 6.51062 | 9.17804 | 9.59308 | None | None | Information score |
| J | 0.75 | 0.36364 | 0.02778 | 0.0 | 0.5 | 0.6 | 0.58333 | 0.83333 | 0.75 | 0.98726 | 1.0 | 0.0 | 1.0 | 1.0 | 0.96296 | 0.2 | 0.5 | 0.68421 | 0.52 | 1.0 | 0.77215 | 0.4 | 0.40816 | 0.44737 | 0.04245 | 0.20833 | 0.97689 | 0.0 | 0.6 | 0.96855 | 0.33333 | 0.0 | 0.5 | 0.95652 | 0.25 | 1.0 | 1.0 | 0.42857 | 0.0 | 0.71429 | 1.0 | 0.68 | 0.5 | 0.33333 | 0.0 | 0.0 | Jaccard index |
| LS | 94.78636 | 185.36 | 21.75587 | None | 514.88889 | 434.4375 | 184.30682 | 386.16667 | 579.25 | 7.37875 | 386.16667 | None | 1158.5 | 2317.0 | 85.81481 | 289.625 | 81.85054 | 118.12157 | 83.66944 | 2317.0 | 9.61672 | 386.16667 | 40.8642 | 59.14264 | 2.54989 | 33.24247 | 3.56988 | None | 220.66667 | 3.6423 | 772.33333 | None | 257.44444 | 100.73913 | 386.16667 | 2317.0 | 772.33333 | 278.04 | None | 108.60938 | 772.33333 | 91.17824 | 579.25 | 772.33333 | None | None | Lift score |
| MCC | 0.85683 | 0.56446 | 0.13288 | None | 0.66623 | 0.74957 | 0.74521 | 0.91267 | 0.86584 | 0.9926 | 1.0 | None | 1.0 | 1.0 | 0.98109 | 0.35279 | 0.67507 | 0.81281 | 0.68257 | 1.0 | 0.85872 | 0.57673 | 0.58814 | 0.65538 | 0.06355 | 0.37204 | 0.9839 | None | 0.75511 | 0.97815 | 0.5771 | None | 0.6658 | 0.97781 | 0.40763 | 1.0 | 1.0 | 0.59913 | None | 0.83731 | 1.0 | 0.81634 | 0.7068 | 0.5771 | None | None | Matthews correlation coefficient |
| MCCI | Strong | Moderate | Negligible | None | Moderate | Strong | Strong | Very Strong | Strong | Very Strong | Very Strong | None | Very Strong | Very Strong | Very Strong | Weak | Moderate | Strong | Moderate | Very Strong | Strong | Moderate | Moderate | Moderate | Negligible | Weak | Very Strong | None | Strong | Very Strong | Moderate | None | Moderate | Very Strong | Weak | Very Strong | Very Strong | Moderate | None | Strong | Very Strong | Strong | Strong | Moderate | None | None | Matthews correlation coefficient interpretation |
| MCEN | 0.17657 | 0.19907 | 0.02226 | 0.0 | 0.15404 | 0.14307 | 0.23009 | 0.06636 | 0.07702 | 0.01628 | 0 | 0.07312 | 0 | 0 | 0.02713 | 0.22452 | 0.30278 | 0.19042 | 0.24253 | 0 | 0.23169 | 0.2146 | 0.233 | 0.20918 | 0.38496 | 0.34496 | 0.0286 | 0.0 | 0.20468 | 0.03306 | 0.16276 | 0.14145 | 0.23106 | 0.0303 | 0.23106 | 0 | 0 | 0.2031 | 0.0 | 0.16397 | 0 | 0.19961 | 0.07702 | 0.16276 | 0.24415 | 0.0 | Modified confusion entropy |
| MK | 0.89826 | 0.39957 | 0.02772 | 0.0 | 0.66623 | 0.74957 | 0.87327 | 0.99957 | 0.75 | 0.99529 | 1.0 | 0.0 | 1.0 | 1.0 | 0.96296 | 0.4987 | 0.56391 | 0.86493 | 0.64782 | 1.0 | 0.86984 | 0.49957 | 0.73113 | 0.45902 | 0.1188 | 0.57476 | 0.97871 | 0.0 | 0.66623 | 0.97422 | 0.33333 | None | 0.6658 | 0.99956 | 0.3329 | 1.0 | 1.0 | 0.59913 | 0.0 | 0.93533 | 1.0 | 0.9414 | 0.99914 | 0.33333 | None | None | Markedness |
| N | 2295 | 2312 | 2314 | 2317 | 2314 | 2313 | 2306 | 2311 | 2314 | 2004 | 2311 | 2317 | 2315 | 2316 | 2291 | 2313 | 2301 | 2300 | 2299 | 2316 | 2104 | 2314 | 2275 | 2299 | 2143 | 2276 | 1681 | 2317 | 2310 | 1695 | 2316 | 2314 | 2311 | 2294 | 2315 | 2316 | 2314 | 2312 | 2317 | 2297 | 2314 | 2293 | 2313 | 2316 | 2314 | 2316 | Condition negative |
| NLR | 0.18198 | 0.20052 | 0.34358 | None | 0.33348 | 0.25011 | 0.36379 | 0.16667 | 0.0 | 0.00959 | 0.0 | None | 0.0 | 0.0 | 0.0 | 0.75032 | 0.18832 | 0.2355 | 0.27863 | 0.0 | 0.14247 | 0.33362 | 0.52543 | 0.05604 | 0.96539 | 0.75843 | 0.00317 | None | 0.14304 | 0.00973 | 0.0 | 1.0 | 0.33362 | 0.04348 | 0.50043 | 0.0 | 0.0 | 0.40035 | None | 0.25011 | 0.0 | 0.29179 | 0.5 | 0.0 | 1.0 | 1.0 | Negative likelihood ratio |
| NLRI | Fair | Poor | Poor | None | Poor | Poor | Poor | Fair | Good | Good | Good | None | Good | Good | Good | Negligible | Fair | Poor | Poor | Good | Fair | Poor | Negligible | Good | Negligible | Negligible | Good | None | Fair | Good | Good | Negligible | Poor | Good | Negligible | Good | Good | Poor | None | Poor | Good | Poor | Negligible | Good | Negligible | Negligible | Negative likelihood ratio interpretation |
| NPV | 0.99826 | 0.99957 | 0.99955 | 1.0 | 0.99957 | 0.99957 | 0.99827 | 0.99957 | 1.0 | 0.9985 | 1.0 | 1.0 | 1.0 | 1.0 | 1.0 | 0.9987 | 0.99869 | 0.99826 | 0.99782 | 1.0 | 0.98578 | 0.99957 | 0.99039 | 0.99956 | 0.92731 | 0.98652 | 0.9988 | 1.0 | 0.99957 | 0.99644 | 1.0 | 0.99871 | 0.99913 | 0.99956 | 0.99957 | 1.0 | 1.0 | 0.99913 | 1.0 | 0.99783 | 1.0 | 0.99696 | 0.99914 | 1.0 | 0.99871 | 0.99957 | Negative predictive value |
| OC | 0.9 | 0.8 | 0.66667 | None | 0.66667 | 0.75 | 0.875 | 1.0 | 1.0 | 0.99678 | 1.0 | None | 1.0 | 1.0 | 1.0 | 0.5 | 0.8125 | 0.86667 | 0.72222 | 1.0 | 0.88406 | 0.66667 | 0.74074 | 0.94444 | 0.19149 | 0.58824 | 0.99686 | None | 0.85714 | 0.99035 | 1.0 | None | 0.66667 | 1.0 | 0.5 | 1.0 | 1.0 | 0.6 | None | 0.9375 | 1.0 | 0.94444 | 1.0 | 1.0 | None | None | Overlap coefficient |
| OOC | 0.85812 | 0.56569 | 0.13704 | None | 0.66667 | 0.75 | 0.7462 | 0.91287 | 0.86603 | 0.99359 | 1.0 | None | 1.0 | 1.0 | 0.98131 | 0.35355 | 0.67767 | 0.81409 | 0.68516 | 1.0 | 0.87152 | 0.57735 | 0.59391 | 0.65874 | 0.09952 | 0.37878 | 0.98834 | None | 0.75593 | 0.98405 | 0.57735 | None | 0.66667 | 0.97802 | 0.40825 | 1.0 | 1.0 | 0.6 | None | 0.83853 | 1.0 | 0.81791 | 0.70711 | 0.57735 | None | None | Otsuka-Ochiai coefficient |
| OP | 0.89784 | 0.88715 | 0.78436 | None | 0.79934 | 0.85649 | 0.77583 | 0.90866 | 0.99935 | 0.99371 | 1.0 | None | 1.0 | 1.0 | 0.99935 | 0.39841 | 0.8931 | 0.8645 | 0.83502 | 1.0 | 0.90664 | 0.79912 | 0.63399 | 0.96673 | 0.01243 | 0.37673 | 0.99122 | None | 0.922 | 0.99067 | 0.9987 | -0.00129 | 0.79869 | 0.97735 | 0.66576 | 1.0 | 1.0 | 0.74868 | None | 0.85477 | 1.0 | 0.82603 | 0.6658 | 0.9987 | -0.00129 | -0.00043 | Optimized precision |
| P | 22 | 5 | 3 | 0 | 3 | 4 | 11 | 6 | 3 | 313 | 6 | 0 | 2 | 1 | 26 | 4 | 16 | 17 | 18 | 1 | 213 | 3 | 42 | 18 | 174 | 41 | 636 | 0 | 7 | 622 | 1 | 3 | 6 | 23 | 2 | 1 | 3 | 5 | 0 | 20 | 3 | 24 | 4 | 1 | 3 | 1 | Condition positive or support |
| PLR | 938.86364 | 308.26667 | 22.35749 | None | 1542.66667 | 1734.75 | 1467.45455 | None | 2314.0 | 1984.79233 | None | None | None | None | 2291.0 | 578.25 | 186.95625 | 879.41176 | 237.19841 | None | 75.31925 | 771.33333 | 154.7619 | 108.56389 | 2.91697 | 79.30314 | 128.90106 | None | 660.0 | 119.90354 | 1158.0 | None | 770.33333 | None | 578.75 | None | None | 693.6 | None | 1722.75 | None | 1624.20833 | None | 1158.0 | None | None | Positive likelihood ratio |
| PLRI | Good | Good | Good | None | Good | Good | Good | None | Good | Good | None | None | None | None | Good | Good | Good | Good | Good | None | Good | Good | Good | Good | Poor | Good | Good | None | Good | Good | Good | None | Good | None | Good | None | None | Good | None | Good | None | Good | None | Good | None | None | Positive likelihood ratio interpretation |
| POP | 2317 | 2317 | 2317 | 2317 | 2317 | 2317 | 2317 | 2317 | 2317 | 2317 | 2317 | 2317 | 2317 | 2317 | 2317 | 2317 | 2317 | 2317 | 2317 | 2317 | 2317 | 2317 | 2317 | 2317 | 2317 | 2317 | 2317 | 2317 | 2317 | 2317 | 2317 | 2317 | 2317 | 2317 | 2317 | 2317 | 2317 | 2317 | 2317 | 2317 | 2317 | 2317 | 2317 | 2317 | 2317 | 2317 | Population |
| PPV | 0.9 | 0.4 | 0.02817 | 0.0 | 0.66667 | 0.75 | 0.875 | 1.0 | 0.75 | 0.99678 | 1.0 | 0.0 | 1.0 | 1.0 | 0.96296 | 0.5 | 0.56522 | 0.86667 | 0.65 | 1.0 | 0.88406 | 0.5 | 0.74074 | 0.45946 | 0.19149 | 0.58824 | 0.97991 | 0.0 | 0.66667 | 0.97778 | 0.33333 | None | 0.66667 | 1.0 | 0.33333 | 1.0 | 1.0 | 0.6 | 0.0 | 0.9375 | 1.0 | 0.94444 | 1.0 | 0.33333 | None | None | Precision or positive predictive value |
| PRE | 0.0095 | 0.00216 | 0.00129 | 0.0 | 0.00129 | 0.00173 | 0.00475 | 0.00259 | 0.00129 | 0.13509 | 0.00259 | 0.0 | 0.00086 | 0.00043 | 0.01122 | 0.00173 | 0.00691 | 0.00734 | 0.00777 | 0.00043 | 0.09193 | 0.00129 | 0.01813 | 0.00777 | 0.0751 | 0.0177 | 0.27449 | 0.0 | 0.00302 | 0.26845 | 0.00043 | 0.00129 | 0.00259 | 0.00993 | 0.00086 | 0.00043 | 0.00129 | 0.00216 | 0.0 | 0.00863 | 0.00129 | 0.01036 | 0.00173 | 0.00043 | 0.00129 | 0.00043 | Prevalence |
| Q | 0.99961 | 0.9987 | 0.96973 | None | 0.99957 | 0.99971 | 0.9995 | None | None | 0.99999 | None | None | None | None | None | 0.99741 | 0.99799 | 0.99946 | 0.99765 | None | 0.99622 | 0.99914 | 0.99323 | 0.99897 | 0.50268 | 0.98105 | 0.99995 | None | 0.99957 | 0.99984 | None | None | 0.99913 | None | 0.99827 | None | None | 0.99885 | None | 0.99971 | None | 0.99964 | None | None | None | None | Yule Q - coefficient of colligation |
| QI | Strong | Strong | Strong | None | Strong | Strong | Strong | None | None | Strong | None | None | None | None | None | Strong | Strong | Strong | Strong | None | Strong | Strong | Strong | Strong | Moderate | Strong | Strong | None | Strong | Strong | None | None | Strong | None | Strong | None | None | Strong | None | Strong | None | Strong | None | None | None | None | Yule Q interpretation |
| RACC | 8e-05 | 1e-05 | 4e-05 | 0.0 | 0.0 | 0.0 | 2e-05 | 1e-05 | 0.0 | 0.01813 | 1e-05 | 0.0 | 0.0 | 0.0 | 0.00013 | 0.0 | 7e-05 | 5e-05 | 7e-05 | 0.0 | 0.00821 | 0.0 | 0.00021 | 0.00012 | 0.00152 | 0.00013 | 0.07665 | 0.0 | 1e-05 | 0.07299 | 0.0 | 0.0 | 1e-05 | 9e-05 | 0.0 | 0.0 | 0.0 | 0.0 | 0.0 | 6e-05 | 0.0 | 8e-05 | 0.0 | 0.0 | 0.0 | 0.0 | Random accuracy |
| RACCU | 8e-05 | 1e-05 | 0.00026 | 0.0 | 0.0 | 0.0 | 2e-05 | 1e-05 | 0.0 | 0.01813 | 1e-05 | 0.00023 | 0.0 | 0.0 | 0.00013 | 0.0 | 7e-05 | 5e-05 | 7e-05 | 0.0 | 0.00821 | 0.0 | 0.00022 | 0.00014 | 0.00227 | 0.00016 | 0.07666 | 0.0 | 1e-05 | 0.073 | 0.0 | 0.0 | 1e-05 | 9e-05 | 0.0 | 0.0 | 0.0 | 0.0 | 0.0 | 6e-05 | 0.0 | 8e-05 | 0.0 | 0.0 | 0.0 | 0.0 | Random accuracy unbiased |
| TN | 2293 | 2306 | 2245 | 2316 | 2313 | 2312 | 2305 | 2311 | 2313 | 2003 | 2311 | 2246 | 2315 | 2316 | 2290 | 2312 | 2291 | 2298 | 2292 | 2316 | 2080 | 2312 | 2268 | 2279 | 2105 | 2269 | 1668 | 2316 | 2307 | 1681 | 2314 | 2314 | 2309 | 2294 | 2313 | 2316 | 2314 | 2310 | 2316 | 2296 | 2314 | 2292 | 2313 | 2314 | 2314 | 2316 | True negative/correct rejection |
| TNR | 0.99913 | 0.9974 | 0.97018 | 0.99957 | 0.99957 | 0.99957 | 0.99957 | 1.0 | 0.99957 | 0.9995 | 1.0 | 0.96936 | 1.0 | 1.0 | 0.99956 | 0.99957 | 0.99565 | 0.99913 | 0.99696 | 1.0 | 0.98859 | 0.99914 | 0.99692 | 0.9913 | 0.98227 | 0.99692 | 0.99227 | 0.99957 | 0.9987 | 0.99174 | 0.99914 | 1.0 | 0.99913 | 1.0 | 0.99914 | 1.0 | 1.0 | 0.99913 | 0.99957 | 0.99956 | 1.0 | 0.99956 | 1.0 | 0.99914 | 1.0 | 1.0 | Specificity or true negative rate |
| TON | 2297 | 2307 | 2246 | 2316 | 2314 | 2313 | 2309 | 2312 | 2313 | 2006 | 2311 | 2246 | 2315 | 2316 | 2290 | 2315 | 2294 | 2302 | 2297 | 2316 | 2110 | 2313 | 2290 | 2280 | 2270 | 2300 | 1670 | 2316 | 2308 | 1687 | 2314 | 2317 | 2311 | 2295 | 2314 | 2316 | 2314 | 2312 | 2316 | 2301 | 2314 | 2299 | 2315 | 2314 | 2317 | 2317 | Test outcome negative |
| TOP | 20 | 10 | 71 | 1 | 3 | 4 | 8 | 5 | 4 | 311 | 6 | 71 | 2 | 1 | 27 | 2 | 23 | 15 | 20 | 1 | 207 | 4 | 27 | 37 | 47 | 17 | 647 | 1 | 9 | 630 | 3 | 0 | 6 | 22 | 3 | 1 | 3 | 5 | 1 | 16 | 3 | 18 | 2 | 3 | 0 | 0 | Test outcome positive |
| TP | 18 | 4 | 2 | 0 | 2 | 3 | 7 | 5 | 3 | 310 | 6 | 0 | 2 | 1 | 26 | 1 | 13 | 13 | 13 | 1 | 183 | 2 | 20 | 17 | 9 | 10 | 634 | 0 | 6 | 616 | 1 | 0 | 4 | 22 | 1 | 1 | 3 | 3 | 0 | 15 | 3 | 17 | 2 | 1 | 0 | 0 | True positive/hit |
| TPR | 0.81818 | 0.8 | 0.66667 | None | 0.66667 | 0.75 | 0.63636 | 0.83333 | 1.0 | 0.99042 | 1.0 | None | 1.0 | 1.0 | 1.0 | 0.25 | 0.8125 | 0.76471 | 0.72222 | 1.0 | 0.85915 | 0.66667 | 0.47619 | 0.94444 | 0.05172 | 0.2439 | 0.99686 | None | 0.85714 | 0.99035 | 1.0 | 0.0 | 0.66667 | 0.95652 | 0.5 | 1.0 | 1.0 | 0.6 | None | 0.75 | 1.0 | 0.70833 | 0.5 | 1.0 | 0.0 | 0.0 | Sensitivity, recall, hit rate, or true positive rate |
| Y | 0.81731 | 0.7974 | 0.63685 | None | 0.66623 | 0.74957 | 0.63593 | 0.83333 | 0.99957 | 0.98992 | 1.0 | None | 1.0 | 1.0 | 0.99956 | 0.24957 | 0.80815 | 0.76384 | 0.71918 | 1.0 | 0.84775 | 0.6658 | 0.47311 | 0.93575 | 0.03399 | 0.24083 | 0.98912 | None | 0.85584 | 0.98209 | 0.99914 | 0.0 | 0.6658 | 0.95652 | 0.49914 | 1.0 | 1.0 | 0.59913 | None | 0.74956 | 1.0 | 0.7079 | 0.5 | 0.99914 | 0.0 | 0.0 | Youden index |
| dInd | 0.18182 | 0.20002 | 0.33466 | None | 0.33333 | 0.25 | 0.36364 | 0.16667 | 0.00043 | 0.0096 | 0.0 | None | 0.0 | 0.0 | 0.00044 | 0.75 | 0.18755 | 0.2353 | 0.27779 | 0.0 | 0.14131 | 0.33333 | 0.52382 | 0.05623 | 0.94844 | 0.7561 | 0.00835 | None | 0.14286 | 0.0127 | 0.00086 | 1.0 | 0.33333 | 0.04348 | 0.5 | 0.0 | 0.0 | 0.4 | None | 0.25 | 0.0 | 0.29167 | 0.5 | 0.00086 | 1.0 | 1.0 | Distance index |
| sInd | 0.87143 | 0.85857 | 0.76336 | None | 0.7643 | 0.82322 | 0.74287 | 0.88215 | 0.99969 | 0.99321 | 1.0 | None | 1.0 | 1.0 | 0.99969 | 0.46967 | 0.86738 | 0.83362 | 0.80357 | 1.0 | 0.90008 | 0.7643 | 0.6296 | 0.96024 | 0.32935 | 0.46535 | 0.9941 | None | 0.89898 | 0.99102 | 0.99939 | 0.29289 | 0.7643 | 0.96926 | 0.64645 | 1.0 | 1.0 | 0.71716 | None | 0.82322 | 1.0 | 0.79376 | 0.64645 | 0.99939 | 0.29289 | 0.29289 | Similarity index |

Generated By PyCM Version 3.6
