# Supplementary material for: TopEC: prediction of Enzyme Commission classes by 3D graph neural networks and localized 3D protein descriptor
Source: Nat Commun. 2025 Mar 20;16:2737. doi: 10.1038/s41467-025-57324-5 (PMC11923149; doi:10.1038/s41467-025-57324-5)
Supplement: Supplementary file 3 — Supplementary Data 1 [file 41467_2025_57324_MOESM3_ESM.zip › Data_S1/figure2/AF703PDB300_main.html]

PyCM Report


# PyCM Report

## Dataset Type :

- Multi-Class Classification
- Imbalanced

Note 1 : Recommended statistics for this type of classification highlighted in aqua

Note 2 : The recommender system assumes that the input is the result of classification over the whole data rather than just a part of it.
If the confusion matrix is the result of test data classification, the recommendation is not valid.

## Confusion Matrix :

|  |  |  |  |  |  |  |  |  |  |  |  |  |  |  |  |  |  |  |  |  |  |  |  |  |  |  |  |  |  |  |  |  |  |  |  |  |  |  |  |  |  |  |  |  |  |  |  |  |  |  |  |  |  |  |  |  |  |  |  |  |  |  |  |  |  |
| --- | --- | --- | --- | --- | --- | --- | --- | --- | --- | --- | --- | --- | --- | --- | --- | --- | --- | --- | --- | --- | --- | --- | --- | --- | --- | --- | --- | --- | --- | --- | --- | --- | --- | --- | --- | --- | --- | --- | --- | --- | --- | --- | --- | --- | --- | --- | --- | --- | --- | --- | --- | --- | --- | --- | --- | --- | --- | --- | --- | --- | --- | --- | --- | --- | --- |
| Actual | Predict  |  |  |  |  |  |  |  |  | | --- | --- | --- | --- | --- | --- | --- | --- | |  | 1 | 2 | 3 | 4 | 5 | 6 | 7 | | 1 | 365 | 3 | 4 | 2 | 3 | 1 | 1 | | 2 | 3 | 270 | 18 | 4 | 1 | 1 | 1 | | 3 | 143 | 9 | 746 | 13 | 0 | 0 | 0 | | 4 | 3 | 6 | 1 | 623 | 0 | 0 | 0 | | 5 | 0 | 3 | 2 | 0 | 35 | 0 | 0 | | 6 | 3 | 7 | 2 | 0 | 2 | 37 | 0 | | 7 | 0 | 1 | 3 | 0 | 0 | 0 | 1 | |

## Overall Statistics :

|  |  |
| --- | --- |
| 95% CI | (0.88401,0.90883) |
| ACC Macro | 0.97041 |
| ARI | 0.76418 |
| AUNP | 0.9351 |
| AUNU | 0.88163 |
| Bangdiwala B | 0.82265 |
| Bennett S | 0.87915 |
| CBA | 0.73963 |
| CSI | 0.59273 |
| Chi-Squared | 8805.64157 |
| Chi-Squared DF | 36 |
| Conditional Entropy | 0.52931 |
| Cramer V | 0.79587 |
| Cross Entropy | 2.11115 |
| F1 Macro | 0.78819 |
| F1 Micro | 0.89642 |
| FNR Macro | 0.21819 |
| FNR Micro | 0.10358 |
| FPR Macro | 0.01854 |
| FPR Micro | 0.01726 |
| Gwet AC1 | 0.88191 |
| Hamming Loss | 0.10358 |
| Joint Entropy | 2.61944 |
| KL Divergence | 0.02101 |
| Kappa | 0.85981 |
| Kappa 95% CI | (0.84301,0.8766) |
| Kappa No Prevalence | 0.79284 |
| Kappa Standard Error | 0.00857 |
| Kappa Unbiased | 0.85947 |
| Krippendorff Alpha | 0.8595 |
| Lambda A | 0.8293 |
| Lambda B | 0.84555 |
| Mutual Information | 1.59095 |
| NIR | 0.39318 |
| Overall ACC | 0.89642 |
| Overall CEN | 0.13236 |
| Overall J | (4.86321,0.69474) |
| Overall MCC | 0.86395 |
| Overall MCEN | 0.19739 |
| Overall RACC | 0.26116 |
| Overall RACCU | 0.2629 |
| P-Value | None |
| PPV Macro | 0.81092 |
| PPV Micro | 0.89642 |
| Pearson C | 0.88977 |
| Phi-Squared | 3.80045 |
| RCI | 0.76117 |
| RR | 331.0 |
| Reference Entropy | 2.09013 |
| Response Entropy | 2.12026 |
| SOA1(Landis & Koch) | Almost Perfect |
| SOA2(Fleiss) | Excellent |
| SOA3(Altman) | Very Good |
| SOA4(Cicchetti) | Excellent |
| SOA5(Cramer) | Strong |
| SOA6(Matthews) | Strong |
| Scott PI | 0.85947 |
| Standard Error | 0.00633 |
| TNR Macro | 0.98146 |
| TNR Micro | 0.98274 |
| TPR Macro | 0.78181 |
| TPR Micro | 0.89642 |
| Zero-one Loss | 240 |

## Class Statistics :

|  |  |  |  |  |  |  |  |  |
| --- | --- | --- | --- | --- | --- | --- | --- | --- |
| Class | 1 | 2 | 3 | 4 | 5 | 6 | 7 | Description |
| ACC | 0.92836 | 0.9754 | 0.91584 | 0.98748 | 0.99525 | 0.99309 | 0.99741 | Accuracy |
| AGF | 0.93662 | 0.94487 | 0.87577 | 0.98717 | 0.93202 | 0.87031 | 0.46589 | Adjusted F-score |
| AGM | 0.93274 | 0.96392 | 0.92673 | 0.98741 | 0.9655 | 0.92443 | 0.72278 | Adjusted geometric mean |
| AM | 138 | 1 | -135 | 9 | 1 | -12 | -2 | Difference between automatic and manual classification |
| AUC | 0.94231 | 0.94584 | 0.89877 | 0.98646 | 0.93618 | 0.8623 | 0.59957 | Area under the ROC curve |
| AUCI | Excellent | Excellent | Very Good | Excellent | Excellent | Very Good | Poor | AUC value interpretation |
| AUPR | 0.83453 | 0.90453 | 0.89011 | 0.9773 | 0.86433 | 0.8371 | 0.26667 | Area under the PR curve |
| BB | 0.706 | 0.90301 | 0.81888 | 0.9704 | 0.85366 | 0.72549 | 0.2 | Braun-Blanquet similarity |
| BCD | 0.02978 | 0.00022 | 0.02913 | 0.00194 | 0.00022 | 0.00259 | 0.00043 | Bray-Curtis dissimilarity |
| BM | 0.88463 | 0.89168 | 0.79754 | 0.97292 | 0.87236 | 0.72461 | 0.19913 | Informedness or bookmaker informedness |
| CEN | 0.17757 | 0.17036 | 0.14851 | 0.04831 | 0.19364 | 0.23389 | 0.46183 | Confusion entropy |
| DOR | 306.33929 | 661.69951 | 207.37293 | 5459.44737 | 2649.5 | 2991.71429 | 288.75 | Diagnostic odds ratio |
| DP | 1.37072 | 1.55511 | 1.27729 | 2.0604 | 1.88729 | 1.91638 | 1.35656 | Discriminant power |
| DPI | Limited | Limited | Limited | Fair | Limited | Limited | Limited | Discriminant power interpretation |
| ERR | 0.07164 | 0.0246 | 0.08416 | 0.01252 | 0.00475 | 0.00691 | 0.00259 | Error rate |
| F0.5 | 0.74581 | 0.90361 | 0.92902 | 0.97313 | 0.85784 | 0.89372 | 0.29412 | F0.5 score |
| F1 | 0.81473 | 0.90452 | 0.88441 | 0.97725 | 0.8642 | 0.82222 | 0.25 | F1 score - harmonic mean of precision and sensitivity |
| F2 | 0.89769 | 0.90543 | 0.84389 | 0.98141 | 0.87065 | 0.76132 | 0.21739 | F2 score |
| FDR | 0.294 | 0.09699 | 0.03866 | 0.0296 | 0.14634 | 0.05128 | 0.66667 | False discovery rate |
| FN | 14 | 28 | 165 | 10 | 5 | 14 | 4 | False negative/miss/type 2 error |
| FNR | 0.03694 | 0.09396 | 0.18112 | 0.0158 | 0.125 | 0.27451 | 0.8 | Miss rate or false negative rate |
| FOR | 0.00778 | 0.01388 | 0.10707 | 0.00597 | 0.0022 | 0.00615 | 0.00173 | False omission rate |
| FP | 152 | 29 | 30 | 19 | 6 | 2 | 2 | False positive/type 1 error/false alarm |
| FPR | 0.07843 | 0.01436 | 0.02134 | 0.01128 | 0.00264 | 0.00088 | 0.00087 | Fall-out or false positive rate |
| G | 0.82457 | 0.90452 | 0.88726 | 0.97728 | 0.86426 | 0.82963 | 0.2582 | G-measure geometric mean of precision and sensitivity |
| GI | 0.88463 | 0.89168 | 0.79754 | 0.97292 | 0.87236 | 0.72461 | 0.19913 | Gini index |
| GM | 0.94209 | 0.945 | 0.89521 | 0.98646 | 0.93418 | 0.85138 | 0.44702 | G-mean geometric mean of specificity and sensitivity |
| HD | 166 | 57 | 195 | 29 | 11 | 16 | 6 | Hamming distance |
| IBA | 0.92435 | 0.82194 | 0.67336 | 0.9687 | 0.76591 | 0.52651 | 0.04014 | Index of balanced accuracy |
| ICSI | 0.66906 | 0.80905 | 0.78022 | 0.95461 | 0.72866 | 0.67421 | -0.46667 | Individual classification success index |
| IS | 2.10972 | 2.81169 | 1.28985 | 1.82864 | 5.62785 | 5.42967 | 7.27115 | Information score |
| J | 0.68738 | 0.82569 | 0.79277 | 0.95552 | 0.76087 | 0.69811 | 0.14286 | Jaccard index |
| LS | 4.31608 | 7.02105 | 2.44503 | 3.55202 | 49.44817 | 43.10156 | 154.46667 | Lift score |
| MCC | 0.78592 | 0.8904 | 0.82542 | 0.96867 | 0.86185 | 0.82644 | 0.25697 | Matthews correlation coefficient |
| MCCI | Strong | Strong | Strong | Very Strong | Strong | Strong | Negligible | Matthews correlation coefficient interpretation |
| MCEN | 0.23381 | 0.26881 | 0.21755 | 0.08246 | 0.28652 | 0.33284 | 0.48174 | Modified confusion entropy |
| MK | 0.69822 | 0.88913 | 0.85427 | 0.96443 | 0.85146 | 0.94257 | 0.3316 | Markedness |
| N | 1938 | 2019 | 1406 | 1684 | 2277 | 2266 | 2312 | Condition negative |
| NLR | 0.04008 | 0.09533 | 0.18507 | 0.01598 | 0.12533 | 0.27475 | 0.80069 | Negative likelihood ratio |
| NLRI | Good | Good | Fair | Good | Fair | Poor | Negligible | Negative likelihood ratio interpretation |
| NPV | 0.99222 | 0.98612 | 0.89293 | 0.99403 | 0.9978 | 0.99385 | 0.99827 | Negative predictive value |
| OC | 0.96306 | 0.90604 | 0.96134 | 0.9842 | 0.875 | 0.94872 | 0.33333 | Overlap coefficient |
| OOC | 0.82457 | 0.90452 | 0.88726 | 0.97728 | 0.86426 | 0.82963 | 0.2582 | Otsuka-Ochiai coefficient |
| OP | 0.90634 | 0.93332 | 0.82695 | 0.9852 | 0.9299 | 0.83443 | 0.33098 | Optimized precision |
| P | 379 | 298 | 911 | 633 | 40 | 51 | 5 | Condition positive or support |
| PLR | 12.27902 | 63.07915 | 38.37819 | 87.2314 | 332.0625 | 821.98039 | 231.2 | Positive likelihood ratio |
| PLRI | Good | Good | Good | Good | Good | Good | Good | Positive likelihood ratio interpretation |
| POP | 2317 | 2317 | 2317 | 2317 | 2317 | 2317 | 2317 | Population |
| PPV | 0.706 | 0.90301 | 0.96134 | 0.9704 | 0.85366 | 0.94872 | 0.33333 | Precision or positive predictive value |
| PRE | 0.16357 | 0.12861 | 0.39318 | 0.2732 | 0.01726 | 0.02201 | 0.00216 | Prevalence |
| Q | 0.99349 | 0.99698 | 0.9904 | 0.99963 | 0.99925 | 0.99933 | 0.9931 | Yule Q - coefficient of colligation |
| QI | Strong | Strong | Strong | Strong | Strong | Strong | Strong | Yule Q interpretation |
| RACC | 0.0365 | 0.0166 | 0.13168 | 0.0757 | 0.00031 | 0.00037 | 0.0 | Random accuracy |
| RACCU | 0.03739 | 0.0166 | 0.13253 | 0.0757 | 0.00031 | 0.00038 | 0.0 | Random accuracy unbiased |
| TN | 1786 | 1990 | 1376 | 1665 | 2271 | 2264 | 2310 | True negative/correct rejection |
| TNR | 0.92157 | 0.98564 | 0.97866 | 0.98872 | 0.99736 | 0.99912 | 0.99913 | Specificity or true negative rate |
| TON | 1800 | 2018 | 1541 | 1675 | 2276 | 2278 | 2314 | Test outcome negative |
| TOP | 517 | 299 | 776 | 642 | 41 | 39 | 3 | Test outcome positive |
| TP | 365 | 270 | 746 | 623 | 35 | 37 | 1 | True positive/hit |
| TPR | 0.96306 | 0.90604 | 0.81888 | 0.9842 | 0.875 | 0.72549 | 0.2 | Sensitivity, recall, hit rate, or true positive rate |
| Y | 0.88463 | 0.89168 | 0.79754 | 0.97292 | 0.87236 | 0.72461 | 0.19913 | Youden index |
| dInd | 0.08669 | 0.09505 | 0.18237 | 0.01941 | 0.12503 | 0.27451 | 0.8 | Distance index |
| sInd | 0.9387 | 0.93279 | 0.87104 | 0.98627 | 0.91159 | 0.80589 | 0.43431 | Similarity index |

Generated By PyCM Version 3.6
