# Supplementary material for: TopEC: prediction of Enzyme Commission classes by 3D graph neural networks and localized 3D protein descriptor
Source: Nat Commun. 2025 Mar 20;16:2737. doi: 10.1038/s41467-025-57324-5 (PMC11923149; doi:10.1038/s41467-025-57324-5)
Supplement: Supplementary file 3 — Supplementary Data 1 [file 41467_2025_57324_MOESM3_ESM.zip › Data_S1/table1/hierarchical/CLEAN_Comb826.html]

 
 
 
 PyCM Report 
 
 
 
 
 
 
 
 
 
 
 
 
 PyCM Report 
 Dataset Type :  
 
 Multi-Class Classification 
 Imbalanced 
 
  Note 1  : Recommended statistics for this type of classification highlighted in  aqua  
  Note 2  : The recommender system assumes that the input is the result of classification over the whole data rather than just a part of it.
If the confusion matrix is the result of test data classification, the recommendation is not valid. 
 Confusion Matrix :  
 
 
 Actual 
 Predict
 
 
  
 0 
 1 
 2 
 3 
 4 
 5 
 6 
 7 
 8 
 9 
 10 
 11 
 12 
 13 
 14 
 15 
 16 
 17 
 18 
 19 
 20 
 21 
 22 
 23 
 24 
 25 
 26 
 27 
 28 
 29 
 30 
 31 
 32 
 33 
 34 
 35 
 36 
 37 
 38 
 39 
 40 
 41 
 42 
 43 
 44 
 45 
 46 
 47 
 48 
 49 
 50 
 51 
 52 
 53 
 54 
 55 
 56 
 57 
 58 
 59 
 60 
 61 
 62 
 63 
 64 
 65 
 66 
 67 
 68 
 69 
 70 
 71 
 72 
 73 
 74 
 75 
 76 
 77 
 78 
 79 
 80 
 81 
 82 
 83 
 84 
 85 
 86 
 87 
 88 
 89 
 90 
 91 
 92 
 93 
 94 
 95 
 96 
 97 
 98 
 99 
 100 
 101 
 102 
 103 
 104 
 105 
 106 
 107 
 108 
 109 
 110 
 111 
 112 
 113 
 114 
 115 
 116 
 117 
 118 
 119 
 120 
 121 
 122 
 123 
 124 
 125 
 126 
 127 
 128 
 129 
 130 
 131 
 132 
 133 
 134 
 135 
 136 
 137 
 138 
 139 
 140 
 141 
 142 
 143 
 144 
 145 
 146 
 147 
 148 
 149 
 150 
 151 
 152 
 153 
 154 
 155 
 156 
 157 
 158 
 159 
 160 
 161 
 162 
 163 
 164 
 165 
 166 
 167 
 168 
 169 
 170 
 171 
 172 
 173 
 174 
 175 
 176 
 177 
 178 
 179 
 180 
 181 
 182 
 183 
 184 
 185 
 186 
 187 
 188 
 189 
 190 
 191 
 192 
 193 
 194 
 195 
 196 
 197 
 198 
 199 
 200 
 201 
 202 
 203 
 204 
 205 
 206 
 207 
 208 
 209 
 210 
 211 
 212 
 213 
 214 
 215 
 216 
 217 
 218 
 219 
 220 
 221 
 222 
 223 
 224 
 225 
 226 
 227 
 228 
 229 
 230 
 231 
 232 
 233 
 234 
 235 
 236 
 237 
 238 
 239 
 240 
 241 
 242 
 243 
 244 
 245 
 246 
 247 
 248 
 249 
 250 
 251 
 252 
 253 
 254 
 255 
 256 
 257 
 258 
 259 
 260 
 261 
 262 
 263 
 264 
 265 
 266 
 267 
 268 
 269 
 270 
 271 
 272 
 273 
 274 
 275 
 276 
 277 
 278 
 279 
 280 
 281 
 282 
 283 
 284 
 285 
 286 
 287 
 288 
 289 
 290 
 291 
 292 
 293 
 294 
 295 
 296 
 297 
 298 
 299 
 300 
 301 
 302 
 303 
 304 
 305 
 306 
 307 
 308 
 309 
 310 
 311 
 312 
 313 
 314 
 315 
 316 
 317 
 318 
 319 
 320 
 321 
 322 
 323 
 324 
 325 
 326 
 327 
 328 
 329 
 330 
 331 
 332 
 333 
 334 
 335 
 336 
 337 
 338 
 339 
 340 
 341 
 342 
 343 
 344 
 345 
 346 
 347 
 348 
 349 
 350 
 351 
 352 
 353 
 354 
 355 
 356 
 357 
 358 
 359 
 360 
 361 
 362 
 363 
 364 
 365 
 366 
 367 
 368 
 369 
 370 
 371 
 372 
 373 
 374 
 375 
 376 
 377 
 378 
 379 
 380 
 381 
 382 
 383 
 384 
 385 
 386 
 387 
 388 
 389 
 390 
 391 
 392 
 393 
 394 
 395 
 396 
 397 
 398 
 399 
 400 
 401 
 402 
 403 
 404 
 405 
 406 
 407 
 408 
 409 
 410 
 411 
 412 
 413 
 414 
 415 
 416 
 417 
 418 
 419 
 420 
 421 
 422 
 423 
 424 
 425 
 426 
 427 
 428 
 429 
 430 
 431 
 432 
 433 
 434 
 435 
 436 
 437 
 438 
 439 
 440 
 441 
 442 
 443 
 444 
 445 
 446 
 447 
 448 
 449 
 450 
 451 
 452 
 453 
 454 
 455 
 456 
 457 
 458 
 459 
 460 
 461 
 462 
 463 
 464 
 465 
 466 
 467 
 468 
 469 
 470 
 471 
 472 
 473 
 474 
 475 
 476 
 477 
 478 
 479 
 480 
 481 
 482 
 483 
 484 
 485 
 486 
 487 
 488 
 489 
 490 
 491 
 492 
 493 
 494 
 495 
 496 
 497 
 498 
 499 
 500 
 501 
 502 
 503 
 504 
 505 
 506 
 507 
 508 
 509 
 510 
 511 
 512 
 513 
 514 
 515 
 516 
 517 
 518 
 519 
 520 
 521 
 522 
 523 
 524 
 525 
 526 
 527 
 528 
 529 
 530 
 531 
 532 
 533 
 534 
 535 
 536 
 537 
 538 
 539 
 540 
 541 
 542 
 543 
 544 
 545 
 546 
 547 
 548 
 549 
 550 
 551 
 552 
 553 
 554 
 555 
 556 
 557 
 558 
 559 
 560 
 561 
 562 
 563 
 564 
 565 
 566 
 567 
 568 
 569 
 570 
 571 
 572 
 573 
 574 
 575 
 576 
 577 
 578 
 579 
 580 
 581 
 582 
 583 
 584 
 585 
 586 
 587 
 588 
 589 
 590 
 591 
 592 
 593 
 594 
 595 
 596 
 597 
 598 
 599 
 600 
 601 
 602 
 603 
 604 
 605 
 606 
 607 
 608 
 609 
 610 
 611 
 612 
 613 
 614 
 615 
 616 
 617 
 618 
 619 
 620 
 621 
 622 
 623 
 624 
 625 
 626 
 627 
 628 
 629 
 630 
 631 
 632 
 633 
 634 
 635 
 636 
 637 
 638 
 639 
 640 
 641 
 642 
 643 
 644 
 645 
 646 
 647 
 648 
 649 
 650 
 651 
 652 
 653 
 654 
 655 
 656 
 657 
 658 
 659 
 660 
 661 
 662 
 663 
 664 
 665 
 666 
 667 
 668 
 669 
 670 
 671 
 672 
 673 
 674 
 675 
 676 
 677 
 678 
 679 
 680 
 681 
 682 
 683 
 684 
 685 
 686 
 687 
 688 
 689 
 690 
 691 
 692 
 693 
 694 
 695 
 696 
 697 
 698 
 699 
 700 
 701 
 702 
 703 
 704 
 705 
 706 
 707 
 708 
 709 
 710 
 711 
 712 
 713 
 714 
 715 
 716 
 717 
 718 
 719 
 720 
 721 
 722 
 723 
 724 
 725 
 726 
 727 
 728 
 729 
 730 
 731 
 732 
 733 
 734 
 735 
 736 
 737 
 738 
 739 
 740 
 741 
 742 
 743 
 744 
 745 
 746 
 747 
 748 
 749 
 750 
 751 
 752 
 753 
 754 
 755 
 756 
 757 
 758 
 759 
 760 
 761 
 762 
 763 
 764 
 765 
 766 
 767 
 768 
 769 
 770 
 771 
 772 
 773 
 774 
 775 
 776 
 777 
 778 
 779 
 780 
 781 
 782 
 783 
 784 
 785 
 786 
 787 
 788 
 789 
 790 
 791 
 792 
 793 
 794 
 795 
 796 
 797 
 798 
 799 
 800 
 801 
 802 
 803 
 804 
 805 
 806 
 807 
 808 
 809 
 810 
 811 
 812 
 813 
 814 
 815 
 816 
 817 
 818 
 819 
 820 
 821 
 822 
 823 
 824 
 825 
 
 
 0 
 29 
 9 
 2 
 1 
 0 
 0 
 0 
 0 
 0 
 0 
 0 
 0 
 0 
 0 
 0 
 0 
 0 
 0 
 0 
 0 
 0 
 0 
 0 
 0 
 0 
 0 
 0 
 0 
 0 
 0 
 0 
 0 
 0 
 0 
 0 
 0 
 0 
 0 
 0 
 0 
 0 
 0 
 0 
 0 
 0 
 0 
 0 
 0 
 0 
 0 
 0 
 0 
 0 
 0 
 0 
 0 
 0 
 0 
 0 
 0 
 0 
 0 
 0 
 0 
 0 
 0 
 0 
 0 
 0 
 0 
 0 
 0 
 0 
 0 
 0 
 0 
 0 
 0 
 0 
 0 
 0 
 0 
 0 
 0 
 0 
 0 
 0 
 0 
 0 
 0 
 0 
 0 
 0 
 0 
 0 
 0 
 0 
 0 
 0 
 0 
 0 
 0 
 0 
 0 
 0 
 0 
 0 
 0 
 0 
 0 
 0 
 0 
 0 
 0 
 0 
 0 
 0 
 0 
 0 
 0 
 0 
 0 
 0 
 0 
 0 
 0 
 0 
 0 
 0 
 0 
 0 
 0 
 0 
 0 
 0 
 0 
 0 
 0 
 0 
 0 
 0 
 0 
 0 
 0 
 0 
 0 
 0 
 0 
 0 
 0 
 0 
 0 
 0 
 0 
 0 
 0 
 0 
 0 
 0 
 0 
 0 
 0 
 0 
 0 
 0 
 0 
 0 
 0 
 0 
 0 
 0 
 0 
 0 
 0 
 0 
 0 
 0 
 0 
 0 
 0 
 0 
 0 
 0 
 0 
 0 
 0 
 0 
 0 
 0 
 0 
 0 
 0 
 0 
 0 
 0 
 0 
 0 
 0 
 0 
 0 
 0 
 0 
 0 
 0 
 0 
 0 
 0 
 0 
 0 
 0 
 0 
 0 
 0 
 0 
 0 
 0 
 0 
 0 
 0 
 0 
 0 
 0 
 0 
 0 
 0 
 0 
 0 
 0 
 0 
 0 
 0 
 0 
 0 
 0 
 0 
 0 
 0 
 0 
 0 
 0 
 0 
 0 
 0 
 0 
 0 
 0 
 0 
 0 
 0 
 0 
 0 
 0 
 0 
 0 
 0 
 0 
 0 
 0 
 0 
 0 
 0 
 0 
 0 
 0 
 0 
 0 
 0 
 0 
 0 
 0 
 0 
 0 
 0 
 0 
 0 
 0 
 0 
 0 
 0 
 0 
 0 
 0 
 0 
 0 
 0 
 0 
 0 
 0 
 0 
 0 
 0 
 0 
 0 
 0 
 0 
 0 
 0 
 0 
 0 
 0 
 0 
 0 
 0 
 0 
 0 
 0 
 0 
 0 
 0 
 0 
 0 
 0 
 0 
 0 
 0 
 0 
 0 
 0 
 0 
 0 
 0 
 0 
 0 
 0 
 0 
 0 
 0 
 0 
 0 
 0 
 0 
 0 
 0 
 0 
 0 
 0 
 0 
 0 
 0 
 0 
 0 
 0 
 0 
 0 
 0 
 0 
 0 
 0 
 0 
 0 
 0 
 0 
 0 
 0 
 0 
 0 
 0 
 0 
 0 
 0 
 0 
 0 
 0 
 0 
 0 
 0 
 0 
 0 
 0 
 0 
 0 
 0 
 0 
 0 
 0 
 0 
 0 
 0 
 0 
 0 
 0 
 0 
 0 
 0 
 0 
 0 
 0 
 0 
 0 
 0 
 0 
 0 
 0 
 0 
 0 
 0 
 0 
 0 
 0 
 0 
 0 
 0 
 0 
 0 
 0 
 0 
 0 
 0 
 0 
 0 
 0 
 0 
 0 
 0 
 0 
 0 
 0 
 0 
 0 
 0 
 0 
 0 
 0 
 0 
 0 
 0 
 0 
 0 
 0 
 0 
 0 
 0 
 0 
 0 
 0 
 0 
 0 
 0 
 0 
 0 
 0 
 0 
 0 
 0 
 0 
 0 
 0 
 0 
 0 
 0 
 0 
 0 
 0 
 0 
 0 
 0 
 0 
 0 
 0 
 0 
 0 
 0 
 0 
 0 
 0 
 0 
 0 
 0 
 0 
 0 
 0 
 0 
 0 
 0 
 0 
 0 
 0 
 0 
 0 
 0 
 0 
 0 
 0 
 0 
 0 
 0 
 0 
 0 
 0 
 0 
 0 
 0 
 0 
 0 
 0 
 0 
 0 
 0 
 0 
 0 
 0 
 0 
 0 
 0 
 0 
 0 
 0 
 0 
 0 
 0 
 0 
 0 
 0 
 0 
 0 
 0 
 0 
 0 
 0 
 0 
 0 
 0 
 0 
 0 
 0 
 0 
 0 
 0 
 0 
 0 
 0 
 0 
 0 
 0 
 0 
 0 
 0 
 0 
 0 
 0 
 0 
 0 
 0 
 0 
 0 
 0 
 0 
 0 
 0 
 0 
 0 
 0 
 0 
 0 
 0 
 0 
 0 
 0 
 0 
 0 
 0 
 0 
 0 
 0 
 0 
 0 
 0 
 0 
 0 
 0 
 0 
 0 
 0 
 0 
 0 
 0 
 0 
 0 
 0 
 0 
 0 
 0 
 0 
 0 
 0 
 0 
 0 
 0 
 0 
 0 
 0 
 0 
 0 
 0 
 0 
 0 
 0 
 0 
 0 
 0 
 0 
 0 
 0 
 0 
 0 
 0 
 0 
 0 
 0 
 0 
 0 
 0 
 0 
 0 
 0 
 0 
 0 
 0 
 0 
 0 
 0 
 0 
 0 
 0 
 0 
 0 
 0 
 0 
 0 
 0 
 0 
 0 
 0 
 0 
 0 
 0 
 0 
 0 
 0 
 0 
 0 
 0 
 0 
 0 
 0 
 0 
 0 
 0 
 0 
 0 
 0 
 0 
 0 
 0 
 0 
 0 
 0 
 0 
 0 
 0 
 0 
 0 
 0 
 0 
 0 
 0 
 0 
 0 
 0 
 0 
 0 
 0 
 0 
 0 
 0 
 0 
 0 
 0 
 0 
 0 
 0 
 0 
 0 
 0 
 0 
 0 
 0 
 0 
 0 
 0 
 0 
 0 
 0 
 0 
 0 
 0 
 0 
 0 
 0 
 0 
 0 
 0 
 0 
 0 
 0 
 0 
 0 
 0 
 0 
 0 
 0 
 0 
 0 
 0 
 0 
 0 
 0 
 0 
 0 
 0 
 0 
 0 
 0 
 0 
 0 
 0 
 0 
 0 
 0 
 0 
 0 
 0 
 0 
 0 
 0 
 0 
 0 
 0 
 0 
 0 
 0 
 0 
 0 
 0 
 0 
 0 
 0 
 0 
 0 
 0 
 0 
 0 
 0 
 0 
 0 
 0 
 0 
 0 
 0 
 0 
 0 
 0 
 0 
 0 
 0 
 0 
 0 
 0 
 0 
 0 
 0 
 0 
 0 
 0 
 0 
 0 
 0 
 0 
 0 
 0 
 0 
 0 
 0 
 0 
 0 
 0 
 0 
 0 
 0 
 0 
 0 
 0 
 0 
 0 
 0 
 0 
 0 
 0 
 0 
 0 
 0 
 0 
 0 
 0 
 0 
 0 
 0 
 0 
 0 
 0 
 0 
 0 
 0 
 0 
 0 
 0 
 0 
 0 
 0 
 0 
 0 
 0 
 0 
 0 
 0 
 0 
 
 
 1 
 0 
 15 
 0 
 0 
 0 
 0 
 0 
 0 
 0 
 0 
 0 
 0 
 0 
 0 
 0 
 0 
 0 
 0 
 0 
 0 
 0 
 0 
 0 
 0 
 0 
 0 
 0 
 0 
 0 
 0 
 0 
 0 
 0 
 0 
 0 
 0 
 0 
 0 
 0 
 0 
 0 
 0 
 0 
 0 
 0 
 0 
 0 
 0 
 0 
 0 
 0 
 0 
 0 
 0 
 0 
 0 
 0 
 0 
 0 
 0 
 0 
 0 
 0 
 0 
 0 
 0 
 0 
 0 
 0 
 0 
 0 
 0 
 0 
 0 
 0 
 0 
 0 
 0 
 0 
 0 
 0 
 0 
 0 
 0 
 0 
 0 
 0 
 0 
 0 
 1 
 0 
 0 
 0 
 0 
 0 
 0 
 0 
 0 
 0 
 0 
 0 
 0 
 0 
 0 
 0 
 0 
 0 
 0 
 1 
 0 
 0 
 0 
 0 
 0 
 0 
 0 
 0 
 0 
 0 
 0 
 0 
 0 
 0 
 0 
 0 
 0 
 0 
 0 
 0 
 0 
 0 
 0 
 0 
 0 
 0 
 0 
 0 
 0 
 0 
 0 
 0 
 0 
 0 
 0 
 0 
 0 
 0 
 0 
 0 
 0 
 0 
 0 
 0 
 0 
 0 
 0 
 0 
 0 
 0 
 0 
 0 
 0 
 0 
 0 
 0 
 0 
 0 
 0 
 0 
 0 
 0 
 0 
 0 
 0 
 0 
 0 
 0 
 0 
 0 
 0 
 0 
 0 
 0 
 0 
 0 
 0 
 0 
 0 
 0 
 0 
 0 
 0 
 0 
 0 
 0 
 0 
 0 
 0 
 0 
 0 
 0 
 0 
 0 
 0 
 0 
 0 
 0 
 0 
 0 
 0 
 0 
 0 
 0 
 0 
 0 
 0 
 0 
 0 
 0 
 0 
 0 
 0 
 0 
 0 
 0 
 0 
 0 
 0 
 0 
 0 
 0 
 0 
 0 
 0 
 0 
 0 
 0 
 0 
 0 
 0 
 0 
 0 
 0 
 0 
 0 
 0 
 0 
 0 
 0 
 0 
 0 
 0 
 0 
 0 
 0 
 0 
 0 
 0 
 0 
 0 
 0 
 0 
 0 
 0 
 0 
 0 
 0 
 0 
 0 
 0 
 0 
 0 
 0 
 0 
 0 
 0 
 0 
 0 
 0 
 0 
 0 
 0 
 0 
 0 
 0 
 0 
 0 
 0 
 0 
 0 
 0 
 0 
 0 
 0 
 0 
 0 
 0 
 0 
 0 
 0 
 0 
 0 
 0 
 0 
 0 
 0 
 0 
 0 
 0 
 0 
 0 
 0 
 0 
 0 
 0 
 0 
 0 
 0 
 0 
 0 
 0 
 0 
 0 
 0 
 0 
 0 
 0 
 0 
 0 
 0 
 0 
 0 
 0 
 0 
 0 
 0 
 0 
 0 
 0 
 0 
 0 
 0 
 0 
 0 
 0 
 0 
 0 
 0 
 0 
 0 
 0 
 0 
 0 
 0 
 0 
 0 
 0 
 0 
 0 
 0 
 0 
 0 
 0 
 0 
 0 
 0 
 0 
 0 
 0 
 0 
 0 
 0 
 0 
 0 
 0 
 0 
 0 
 0 
 0 
 0 
 0 
 0 
 0 
 0 
 0 
 0 
 0 
 0 
 0 
 0 
 0 
 0 
 0 
 0 
 0 
 0 
 0 
 0 
 0 
 0 
 0 
 0 
 0 
 0 
 0 
 0 
 0 
 0 
 0 
 0 
 0 
 0 
 0 
 0 
 0 
 0 
 0 
 0 
 0 
 0 
 0 
 0 
 0 
 0 
 0 
 0 
 0 
 0 
 0 
 0 
 0 
 0 
 0 
 0 
 0 
 0 
 0 
 0 
 0 
 0 
 0 
 0 
 0 
 0 
 0 
 0 
 0 
 0 
 0 
 0 
 0 
 0 
 0 
 0 
 0 
 0 
 0 
 0 
 0 
 0 
 0 
 0 
 0 
 0 
 0 
 0 
 0 
 0 
 0 
 0 
 0 
 0 
 0 
 0 
 0 
 0 
 0 
 0 
 0 
 0 
 0 
 0 
 0 
 0 
 0 
 0 
 0 
 0 
 0 
 0 
 0 
 0 
 0 
 0 
 0 
 0 
 0 
 0 
 0 
 0 
 0 
 0 
 0 
 0 
 0 
 0 
 0 
 0 
 0 
 0 
 0 
 0 
 0 
 0 
 0 
 0 
 0 
 0 
 0 
 0 
 0 
 0 
 0 
 0 
 0 
 0 
 0 
 0 
 0 
 0 
 0 
 0 
 0 
 0 
 0 
 0 
 0 
 0 
 0 
 0 
 0 
 0 
 0 
 0 
 0 
 0 
 0 
 0 
 0 
 0 
 0 
 0 
 0 
 0 
 0 
 0 
 0 
 0 
 0 
 0 
 0 
 0 
 0 
 0 
 0 
 0 
 0 
 0 
 0 
 0 
 0 
 0 
 0 
 0 
 0 
 0 
 0 
 0 
 0 
 0 
 0 
 0 
 0 
 0 
 0 
 0 
 0 
 0 
 0 
 0 
 0 
 0 
 0 
 0 
 0 
 0 
 0 
 0 
 0 
 0 
 0 
 0 
 0 
 0 
 0 
 0 
 0 
 0 
 0 
 0 
 0 
 0 
 0 
 0 
 0 
 0 
 0 
 0 
 0 
 0 
 0 
 0 
 0 
 0 
 0 
 0 
 0 
 0 
 0 
 0 
 0 
 0 
 0 
 0 
 0 
 0 
 0 
 0 
 0 
 0 
 0 
 0 
 0 
 0 
 0 
 0 
 0 
 0 
 0 
 0 
 0 
 0 
 0 
 0 
 0 
 0 
 0 
 0 
 0 
 0 
 0 
 0 
 0 
 0 
 0 
 0 
 0 
 0 
 0 
 0 
 0 
 0 
 0 
 0 
 0 
 0 
 0 
 0 
 0 
 0 
 0 
 0 
 0 
 0 
 0 
 0 
 0 
 0 
 0 
 0 
 0 
 0 
 0 
 0 
 0 
 0 
 0 
 0 
 0 
 0 
 0 
 0 
 0 
 0 
 0 
 0 
 0 
 0 
 0 
 0 
 0 
 0 
 0 
 0 
 0 
 0 
 0 
 0 
 0 
 0 
 0 
 0 
 0 
 0 
 0 
 0 
 0 
 0 
 0 
 0 
 0 
 0 
 0 
 0 
 0 
 0 
 0 
 0 
 0 
 0 
 0 
 0 
 0 
 0 
 0 
 0 
 0 
 0 
 0 
 0 
 0 
 0 
 0 
 0 
 0 
 0 
 0 
 0 
 0 
 0 
 0 
 0 
 0 
 0 
 0 
 0 
 0 
 0 
 0 
 0 
 0 
 0 
 0 
 0 
 0 
 0 
 0 
 0 
 0 
 0 
 0 
 0 
 0 
 0 
 0 
 0 
 0 
 0 
 0 
 0 
 0 
 0 
 0 
 0 
 0 
 0 
 0 
 0 
 0 
 0 
 0 
 0 
 0 
 0 
 0 
 0 
 0 
 0 
 0 
 0 
 0 
 0 
 0 
 0 
 0 
 0 
 0 
 0 
 0 
 0 
 0 
 0 
 0 
 0 
 0 
 0 
 
 
 2 
 0 
 0 
 0 
 0 
 0 
 0 
 0 
 0 
 0 
 0 
 0 
 0 
 0 
 0 
 0 
 0 
 0 
 0 
 0 
 0 
 0 
 0 
 0 
 0 
 0 
 0 
 0 
 0 
 0 
 0 
 0 
 0 
 0 
 0 
 0 
 0 
 0 
 0 
 0 
 0 
 0 
 0 
 0 
 0 
 0 
 0 
 0 
 0 
 0 
 0 
 0 
 0 
 0 
 0 
 0 
 0 
 0 
 0 
 0 
 0 
 0 
 0 
 0 
 0 
 0 
 0 
 0 
 0 
 0 
 0 
 0 
 0 
 0 
 0 
 0 
 0 
 0 
 0 
 0 
 0 
 0 
 0 
 0 
 0 
 0 
 0 
 0 
 0 
 0 
 0 
 0 
 0 
 0 
 0 
 0 
 0 
 0 
 0 
 0 
 0 
 0 
 0 
 0 
 0 
 0 
 0 
 0 
 0 
 0 
 0 
 0 
 0 
 0 
 0 
 0 
 0 
 0 
 0 
 0 
 0 
 0 
 0 
 0 
 0 
 0 
 0 
 0 
 0 
 0 
 0 
 0 
 0 
 0 
 0 
 0 
 0 
 0 
 0 
 0 
 0 
 0 
 0 
 0 
 0 
 0 
 0 
 0 
 0 
 0 
 0 
 0 
 0 
 0 
 0 
 0 
 0 
 0 
 0 
 0 
 0 
 0 
 0 
 0 
 0 
 0 
 0 
 0 
 0 
 0 
 0 
 0 
 0 
 0 
 0 
 0 
 0 
 0 
 0 
 0 
 0 
 0 
 0 
 0 
 0 
 0 
 0 
 0 
 0 
 0 
 0 
 0 
 0 
 0 
 0 
 0 
 0 
 0 
 0 
 0 
 0 
 0 
 0 
 0 
 0 
 0 
 0 
 0 
 0 
 0 
 0 
 0 
 0 
 0 
 0 
 0 
 0 
 0 
 0 
 0 
 0 
 0 
 0 
 0 
 0 
 0 
 0 
 0 
 0 
 0 
 0 
 0 
 0 
 0 
 0 
 0 
 0 
 0 
 0 
 0 
 0 
 0 
 0 
 0 
 0 
 0 
 0 
 0 
 0 
 0 
 0 
 0 
 0 
 0 
 0 
 0 
 0 
 0 
 0 
 0 
 0 
 0 
 0 
 0 
 0 
 0 
 0 
 0 
 0 
 0 
 0 
 0 
 0 
 0 
 0 
 0 
 0 
 0 
 0 
 0 
 0 
 0 
 0 
 0 
 0 
 0 
 0 
 0 
 0 
 0 
 0 
 0 
 0 
 0 
 0 
 0 
 0 
 0 
 0 
 0 
 0 
 0 
 0 
 0 
 0 
 0 
 0 
 0 
 0 
 0 
 0 
 0 
 0 
 0 
 0 
 0 
 0 
 0 
 0 
 0 
 0 
 0 
 0 
 0 
 0 
 0 
 0 
 0 
 0 
 0 
 0 
 0 
 0 
 0 
 0 
 0 
 0 
 0 
 0 
 0 
 0 
 0 
 0 
 0 
 0 
 0 
 0 
 0 
 0 
 0 
 0 
 0 
 0 
 0 
 0 
 0 
 0 
 0 
 0 
 0 
 0 
 0 
 0 
 0 
 0 
 0 
 0 
 0 
 0 
 0 
 0 
 0 
 0 
 0 
 0 
 0 
 0 
 0 
 0 
 0 
 0 
 0 
 0 
 0 
 0 
 0 
 0 
 0 
 0 
 0 
 0 
 0 
 0 
 0 
 0 
 0 
 0 
 0 
 0 
 0 
 0 
 0 
 0 
 0 
 0 
 0 
 0 
 0 
 0 
 0 
 0 
 0 
 0 
 0 
 0 
 0 
 0 
 0 
 0 
 0 
 0 
 0 
 0 
 0 
 0 
 0 
 0 
 0 
 0 
 0 
 0 
 0 
 0 
 0 
 0 
 0 
 0 
 0 
 0 
 0 
 0 
 0 
 0 
 0 
 0 
 0 
 0 
 0 
 0 
 0 
 0 
 0 
 0 
 0 
 0 
 0 
 0 
 0 
 0 
 0 
 0 
 0 
 0 
 0 
 0 
 0 
 0 
 0 
 0 
 0 
 0 
 0 
 0 
 0 
 0 
 0 
 0 
 0 
 0 
 0 
 0 
 0 
 0 
 0 
 0 
 0 
 0 
 0 
 0 
 0 
 0 
 0 
 0 
 0 
 0 
 0 
 0 
 0 
 0 
 0 
 0 
 0 
 0 
 0 
 0 
 0 
 0 
 0 
 0 
 0 
 0 
 0 
 0 
 0 
 0 
 0 
 0 
 0 
 0 
 0 
 0 
 0 
 0 
 0 
 0 
 0 
 0 
 0 
 0 
 0 
 0 
 0 
 0 
 0 
 0 
 0 
 0 
 0 
 0 
 0 
 0 
 0 
 0 
 0 
 0 
 0 
 0 
 0 
 0 
 0 
 0 
 0 
 0 
 0 
 0 
 0 
 0 
 0 
 0 
 0 
 0 
 0 
 0 
 0 
 0 
 0 
 0 
 0 
 0 
 0 
 0 
 0 
 0 
 0 
 0 
 0 
 0 
 0 
 0 
 0 
 0 
 0 
 0 
 0 
 0 
 0 
 0 
 0 
 0 
 0 
 0 
 0 
 0 
 0 
 0 
 0 
 0 
 0 
 0 
 0 
 0 
 0 
 0 
 0 
 0 
 0 
 0 
 0 
 0 
 0 
 0 
 0 
 0 
 0 
 0 
 0 
 0 
 0 
 0 
 0 
 0 
 0 
 0 
 0 
 0 
 0 
 0 
 0 
 0 
 0 
 0 
 0 
 0 
 0 
 0 
 0 
 0 
 0 
 0 
 0 
 0 
 0 
 0 
 0 
 0 
 0 
 0 
 0 
 0 
 0 
 0 
 0 
 0 
 0 
 0 
 0 
 0 
 0 
 0 
 0 
 0 
 0 
 0 
 0 
 0 
 0 
 0 
 0 
 0 
 0 
 0 
 0 
 0 
 0 
 0 
 0 
 0 
 0 
 0 
 0 
 0 
 0 
 0 
 0 
 0 
 0 
 0 
 0 
 0 
 0 
 0 
 0 
 0 
 0 
 0 
 0 
 0 
 0 
 3 
 0 
 0 
 0 
 0 
 0 
 0 
 0 
 0 
 0 
 0 
 0 
 0 
 0 
 0 
 0 
 0 
 0 
 0 
 0 
 0 
 0 
 0 
 0 
 0 
 0 
 0 
 0 
 0 
 0 
 0 
 0 
 0 
 0 
 0 
 0 
 0 
 0 
 0 
 0 
 0 
 0 
 0 
 0 
 0 
 0 
 0 
 0 
 0 
 0 
 0 
 0 
 0 
 0 
 0 
 0 
 0 
 0 
 0 
 0 
 0 
 0 
 0 
 0 
 0 
 0 
 0 
 0 
 0 
 0 
 0 
 0 
 0 
 0 
 0 
 0 
 0 
 0 
 0 
 0 
 0 
 0 
 0 
 0 
 0 
 0 
 0 
 0 
 0 
 0 
 0 
 0 
 0 
 0 
 0 
 0 
 0 
 0 
 0 
 0 
 0 
 0 
 0 
 0 
 0 
 0 
 0 
 0 
 0 
 0 
 0 
 0 
 0 
 0 
 0 
 0 
 0 
 0 
 0 
 0 
 0 
 0 
 0 
 0 
 0 
 0 
 0 
 0 
 0 
 
 
 3 
 0 
 0 
 0 
 6 
 0 
 0 
 0 
 0 
 0 
 0 
 0 
 0 
 0 
 0 
 0 
 0 
 0 
 0 
 0 
 0 
 0 
 0 
 0 
 0 
 0 
 0 
 0 
 0 
 0 
 0 
 0 
 0 
 0 
 0 
 0 
 0 
 0 
 0 
 0 
 0 
 0 
 0 
 0 
 0 
 0 
 0 
 0 
 0 
 0 
 0 
 0 
 0 
 0 
 0 
 0 
 0 
 0 
 0 
 0 
 0 
 0 
 0 
 0 
 0 
 0 
 0 
 0 
 0 
 0 
 0 
 0 
 0 
 0 
 0 
 0 
 0 
 0 
 0 
 0 
 0 
 0 
 0 
 0 
 0 
 0 
 0 
 0 
 0 
 0 
 0 
 0 
 0 
 0 
 0 
 0 
 0 
 0 
 0 
 0 
 0 
 0 
 0 
 0 
 0 
 0 
 0 
 0 
 0 
 0 
 0 
 0 
 0 
 0 
 0 
 0 
 0 
 0 
 0 
 0 
 0 
 0 
 0 
 0 
 0 
 0 
 0 
 0 
 0 
 0 
 0 
 0 
 0 
 0 
 0 
 0 
 0 
 0 
 0 
 0 
 0 
 0 
 0 
 0 
 0 
 0 
 0 
 0 
 0 
 0 
 0 
 0 
 0 
 0 
 0 
 0 
 0 
 0 
 0 
 0 
 0 
 0 
 0 
 0 
 0 
 0 
 0 
 0 
 0 
 0 
 0 
 0 
 0 
 0 
 0 
 0 
 0 
 0 
 0 
 0 
 0 
 0 
 0 
 0 
 0 
 0 
 0 
 0 
 0 
 0 
 0 
 0 
 0 
 0 
 0 
 0 
 0 
 0 
 0 
 0 
 0 
 0 
 0 
 0 
 0 
 0 
 0 
 0 
 0 
 0 
 0 
 0 
 0 
 0 
 0 
 0 
 0 
 0 
 0 
 0 
 0 
 0 
 0 
 0 
 0 
 0 
 0 
 0 
 0 
 0 
 0 
 0 
 0 
 0 
 0 
 0 
 0 
 0 
 0 
 0 
 0 
 0 
 0 
 0 
 0 
 0 
 0 
 0 
 0 
 0 
 0 
 0 
 0 
 0 
 0 
 0 
 0 
 0 
 0 
 0 
 0 
 0 
 0 
 0 
 0 
 0 
 0 
 0 
 0 
 0 
 0 
 0 
 0 
 0 
 0 
 0 
 0 
 0 
 0 
 0 
 0 
 0 
 0 
 0 
 0 
 0 
 0 
 0 
 0 
 0 
 0 
 0 
 0 
 0 
 0 
 0 
 0 
 0 
 0 
 0 
 0 
 0 
 0 
 0 
 0 
 0 
 0 
 0 
 0 
 0 
 0 
 0 
 0 
 0 
 0 
 0 
 0 
 0 
 0 
 0 
 0 
 0 
 0 
 0 
 0 
 0 
 0 
 0 
 0 
 0 
 0 
 0 
 0 
 0 
 0 
 0 
 0 
 0 
 0 
 0 
 0 
 0 
 0 
 0 
 0 
 0 
 0 
 0 
 0 
 0 
 0 
 0 
 0 
 0 
 0 
 0 
 0 
 0 
 0 
 0 
 0 
 0 
 0 
 0 
 0 
 0 
 0 
 0 
 0 
 0 
 0 
 0 
 0 
 0 
 0 
 0 
 0 
 0 
 0 
 0 
 0 
 0 
 0 
 0 
 0 
 0 
 0 
 0 
 0 
 0 
 0 
 0 
 0 
 0 
 0 
 0 
 0 
 0 
 0 
 0 
 0 
 0 
 0 
 0 
 0 
 0 
 0 
 0 
 0 
 0 
 0 
 0 
 0 
 0 
 0 
 0 
 0 
 0 
 0 
 0 
 0 
 0 
 0 
 0 
 0 
 0 
 0 
 0 
 0 
 0 
 0 
 0 
 0 
 0 
 0 
 0 
 0 
 0 
 0 
 0 
 0 
 0 
 0 
 0 
 0 
 0 
 0 
 0 
 0 
 0 
 0 
 0 
 0 
 0 
 0 
 0 
 0 
 0 
 0 
 0 
 0 
 0 
 0 
 0 
 0 
 0 
 0 
 0 
 0 
 0 
 0 
 0 
 0 
 0 
 0 
 0 
 0 
 0 
 0 
 0 
 0 
 0 
 0 
 0 
 0 
 0 
 0 
 0 
 0 
 0 
 0 
 0 
 0 
 0 
 0 
 0 
 0 
 0 
 0 
 0 
 0 
 0 
 0 
 0 
 0 
 0 
 0 
 0 
 0 
 0 
 0 
 0 
 0 
 0 
 0 
 0 
 0 
 0 
 0 
 0 
 0 
 0 
 0 
 0 
 0 
 0 
 0 
 0 
 0 
 0 
 0 
 0 
 0 
 0 
 0 
 0 
 0 
 0 
 0 
 0 
 0 
 0 
 0 
 0 
 0 
 0 
 0 
 0 
 0 
 0 
 0 
 0 
 0 
 0 
 0 
 0 
 0 
 0 
 0 
 0 
 0 
 0 
 0 
 0 
 0 
 0 
 0 
 0 
 0 
 0 
 0 
 0 
 0 
 0 
 0 
 0 
 0 
 0 
 0 
 0 
 0 
 0 
 0 
 0 
 0 
 0 
 0 
 0 
 0 
 0 
 0 
 0 
 0 
 0 
 0 
 0 
 0 
 0 
 0 
 0 
 0 
 0 
 0 
 0 
 0 
 0 
 0 
 0 
 0 
 0 
 0 
 0 
 0 
 0 
 0 
 0 
 0 
 0 
 0 
 0 
 0 
 0 
 0 
 0 
 0 
 0 
 0 
 0 
 0 
 0 
 0 
 0 
 0 
 0 
 0 
 0 
 0 
 0 
 0 
 0 
 0 
 0 
 0 
 0 
 0 
 0 
 0 
 0 
 0 
 0 
 0 
 0 
 0 
 0 
 0 
 0 
 0 
 0 
 0 
 0 
 0 
 0 
 0 
 0 
 0 
 0 
 0 
 0 
 0 
 0 
 0 
 0 
 0 
 0 
 0 
 0 
 0 
 0 
 0 
 0 
 0 
 0 
 0 
 0 
 0 
 0 
 0 
 0 
 0 
 0 
 0 
 0 
 0 
 0 
 0 
 0 
 0 
 0 
 0 
 0 
 0 
 0 
 0 
 0 
 0 
 0 
 0 
 0 
 0 
 0 
 0 
 0 
 0 
 0 
 0 
 0 
 0 
 0 
 0 
 0 
 0 
 0 
 0 
 0 
 0 
 0 
 0 
 0 
 0 
 0 
 0 
 0 
 0 
 0 
 0 
 0 
 0 
 0 
 0 
 0 
 0 
 0 
 0 
 0 
 0 
 0 
 0 
 0 
 0 
 0 
 0 
 0 
 0 
 0 
 0 
 0 
 0 
 0 
 0 
 0 
 0 
 0 
 0 
 0 
 0 
 0 
 0 
 0 
 0 
 0 
 0 
 0 
 0 
 0 
 0 
 0 
 0 
 0 
 0 
 0 
 0 
 0 
 0 
 0 
 0 
 0 
 0 
 0 
 0 
 0 
 0 
 0 
 0 
 0 
 0 
 0 
 0 
 0 
 0 
 0 
 0 
 0 
 0 
 0 
 0 
 0 
 0 
 0 
 0 
 0 
 0 
 0 
 0 
 0 
 0 
 0 
 0 
 0 
 0 
 0 
 0 
 0 
 0 
 0 
 0 
 0 
 0 
 
 
 4 
 0 
 0 
 0 
 0 
 2 
 0 
 0 
 0 
 0 
 0 
 0 
 0 
 0 
 0 
 0 
 0 
 0 
 0 
 0 
 0 
 0 
 0 
 0 
 0 
 0 
 0 
 0 
 0 
 0 
 0 
 0 
 0 
 0 
 0 
 0 
 0 
 0 
 0 
 0 
 0 
 0 
 0 
 0 
 0 
 0 
 0 
 0 
 0 
 0 
 0 
 0 
 0 
 0 
 0 
 0 
 0 
 0 
 0 
 0 
 0 
 0 
 0 
 0 
 0 
 0 
 0 
 0 
 0 
 0 
 0 
 0 
 0 
 0 
 0 
 0 
 0 
 0 
 0 
 0 
 0 
 0 
 0 
 0 
 0 
 0 
 0 
 0 
 0 
 0 
 0 
 0 
 0 
 0 
 0 
 0 
 0 
 0 
 0 
 0 
 0 
 0 
 0 
 0 
 0 
 0 
 0 
 0 
 0 
 0 
 0 
 0 
 0 
 0 
 0 
 0 
 0 
 0 
 0 
 0 
 0 
 0 
 0 
 0 
 0 
 0 
 0 
 0 
 0 
 0 
 0 
 0 
 0 
 0 
 0 
 0 
 0 
 0 
 0 
 0 
 0 
 0 
 0 
 0 
 0 
 0 
 0 
 0 
 0 
 0 
 0 
 0 
 0 
 0 
 0 
 0 
 0 
 0 
 0 
 0 
 0 
 0 
 0 
 0 
 0 
 0 
 0 
 0 
 0 
 0 
 0 
 0 
 0 
 0 
 0 
 0 
 0 
 0 
 0 
 0 
 0 
 0 
 0 
 0 
 0 
 0 
 0 
 0 
 0 
 0 
 0 
 0 
 0 
 0 
 0 
 0 
 0 
 0 
 0 
 0 
 0 
 0 
 0 
 0 
 0 
 0 
 0 
 0 
 0 
 0 
 0 
 0 
 0 
 0 
 0 
 0 
 0 
 0 
 0 
 0 
 0 
 0 
 0 
 0 
 0 
 0 
 0 
 0 
 0 
 0 
 0 
 0 
 0 
 0 
 0 
 0 
 0 
 0 
 0 
 0 
 0 
 0 
 0 
 0 
 0 
 0 
 0 
 0 
 0 
 0 
 0 
 0 
 0 
 0 
 0 
 0 
 0 
 0 
 0 
 0 
 0 
 0 
 0 
 0 
 0 
 0 
 0 
 0 
 0 
 0 
 0 
 0 
 0 
 0 
 0 
 0 
 0 
 0 
 0 
 0 
 0 
 0 
 0 
 0 
 0 
 0 
 0 
 0 
 0 
 0 
 0 
 0 
 0 
 0 
 0 
 0 
 0 
 0 
 0 
 0 
 0 
 0 
 0 
 0 
 0 
 0 
 0 
 0 
 0 
 0 
 0 
 0 
 0 
 0 
 0 
 0 
 0 
 0 
 0 
 0 
 0 
 0 
 0 
 0 
 0 
 0 
 0 
 0 
 0 
 0 
 0 
 0 
 0 
 0 
 0 
 0 
 0 
 0 
 0 
 0 
 0 
 0 
 0 
 0 
 0 
 0 
 0 
 0 
 0 
 0 
 0 
 0 
 0 
 0 
 0 
 0 
 0 
 0 
 0 
 0 
 0 
 0 
 0 
 0 
 0 
 0 
 0 
 0 
 0 
 0 
 0 
 0 
 0 
 0 
 0 
 0 
 0 
 0 
 0 
 0 
 0 
 0 
 0 
 0 
 0 
 0 
 0 
 0 
 0 
 0 
 0 
 0 
 0 
 0 
 0 
 0 
 0 
 0 
 0 
 0 
 0 
 0 
 0 
 0 
 0 
 0 
 0 
 0 
 0 
 0 
 0 
 0 
 0 
 0 
 0 
 0 
 0 
 0 
 0 
 0 
 0 
 0 
 0 
 0 
 0 
 0 
 0 
 0 
 0 
 0 
 0 
 0 
 0 
 0 
 0 
 0 
 0 
 0 
 0 
 0 
 0 
 0 
 0 
 0 
 0 
 0 
 0 
 0 
 0 
 0 
 0 
 0 
 0 
 0 
 0 
 0 
 0 
 0 
 0 
 0 
 0 
 0 
 0 
 0 
 0 
 0 
 0 
 0 
 0 
 0 
 0 
 0 
 0 
 0 
 0 
 0 
 0 
 0 
 0 
 0 
 0 
 0 
 0 
 0 
 0 
 0 
 0 
 0 
 0 
 0 
 0 
 0 
 0 
 0 
 0 
 0 
 0 
 0 
 0 
 0 
 0 
 0 
 0 
 0 
 0 
 0 
 0 
 0 
 0 
 0 
 0 
 0 
 0 
 0 
 0 
 0 
 0 
 0 
 0 
 0 
 0 
 0 
 0 
 0 
 0 
 0 
 0 
 0 
 0 
 0 
 0 
 0 
 0 
 0 
 0 
 0 
 0 
 0 
 0 
 0 
 0 
 0 
 0 
 0 
 0 
 0 
 0 
 0 
 0 
 0 
 0 
 0 
 0 
 0 
 0 
 0 
 0 
 0 
 0 
 0 
 0 
 0 
 0 
 0 
 0 
 0 
 0 
 0 
 0 
 0 
 0 
 0 
 0 
 0 
 0 
 0 
 0 
 0 
 0 
 0 
 0 
 0 
 0 
 0 
 0 
 0 
 0 
 0 
 0 
 0 
 0 
 0 
 0 
 0 
 0 
 0 
 0 
 0 
 0 
 0 
 0 
 0 
 0 
 0 
 0 
 0 
 0 
 0 
 0 
 0 
 0 
 0 
 0 
 0 
 0 
 0 
 0 
 0 
 0 
 0 
 0 
 0 
 0 
 0 
 0 
 0 
 0 
 0 
 0 
 0 
 0 
 0 
 0 
 0 
 0 
 0 
 0 
 0 
 0 
 0 
 0 
 0 
 0 
 0 
 0 
 0 
 0 
 0 
 0 
 0 
 0 
 0 
 0 
 0 
 0 
 0 
 0 
 0 
 0 
 0 
 0 
 0 
 0 
 0 
 0 
 0 
 0 
 0 
 0 
 0 
 0 
 0 
 0 
 0 
 0 
 0 
 0 
 0 
 0 
 0 
 0 
 0 
 0 
 0 
 0 
 0 
 0 
 0 
 0 
 0 
 0 
 0 
 0 
 0 
 0 
 0 
 0 
 0 
 0 
 0 
 0 
 0 
 0 
 0 
 0 
 0 
 0 
 0 
 0 
 0 
 0 
 0 
 0 
 0 
 0 
 0 
 0 
 0 
 0 
 0 
 0 
 0 
 0 
 0 
 0 
 0 
 0 
 0 
 0 
 0 
 0 
 0 
 0 
 0 
 0 
 0 
 0 
 0 
 0 
 0 
 0 
 0 
 0 
 0 
 0 
 0 
 0 
 0 
 0 
 0 
 0 
 0 
 0 
 0 
 0 
 0 
 0 
 0 
 0 
 0 
 0 
 0 
 0 
 0 
 0 
 0 
 0 
 0 
 0 
 0 
 0 
 0 
 0 
 0 
 0 
 0 
 0 
 0 
 0 
 0 
 0 
 0 
 0 
 0 
 0 
 0 
 0 
 0 
 0 
 0 
 0 
 0 
 0 
 0 
 0 
 0 
 0 
 0 
 0 
 0 
 0 
 0 
 0 
 0 
 0 
 0 
 0 
 0 
 0 
 0 
 0 
 0 
 0 
 0 
 0 
 0 
 0 
 0 
 0 
 0 
 0 
 0 
 0 
 0 
 0 
 0 
 0 
 
 
 5 
 0 
 0 
 0 
 0 
 0 
 8 
 0 
 0 
 0 
 0 
 0 
 0 
 0 
 0 
 0 
 0 
 0 
 0 
 0 
 0 
 0 
 0 
 0 
 0 
 0 
 0 
 0 
 0 
 0 
 0 
 0 
 0 
 0 
 0 
 0 
 0 
 0 
 0 
 0 
 0 
 0 
 0 
 0 
 0 
 0 
 0 
 0 
 0 
 0 
 0 
 0 
 0 
 0 
 0 
 0 
 0 
 0 
 0 
 0 
 0 
 0 
 0 
 0 
 0 
 0 
 0 
 0 
 0 
 0 
 0 
 0 
 0 
 0 
 0 
 0 
 0 
 0 
 0 
 0 
 0 
 0 
 0 
 0 
 0 
 0 
 0 
 0 
 0 
 0 
 0 
 0 
 0 
 0 
 0 
 0 
 0 
 0 
 0 
 0 
 0 
 0 
 0 
 0 
 0 
 0 
 0 
 0 
 0 
 0 
 0 
 0 
 0 
 0 
 0 
 0 
 0 
 0 
 0 
 0 
 0 
 0 
 0 
 0 
 0 
 0 
 0 
 0 
 0 
 0 
 0 
 0 
 0 
 0 
 0 
 0 
 0 
 0 
 0 
 0 
 0 
 0 
 0 
 0 
 0 
 0 
 0 
 0 
 0 
 0 
 0 
 0 
 0 
 0 
 0 
 0 
 0 
 0 
 0 
 0 
 0 
 0 
 0 
 0 
 0 
 0 
 0 
 0 
 0 
 0 
 0 
 0 
 0 
 0 
 0 
 0 
 0 
 0 
 0 
 0 
 0 
 0 
 0 
 0 
 0 
 0 
 0 
 0 
 0 
 0 
 0 
 0 
 0 
 0 
 0 
 0 
 0 
 0 
 0 
 0 
 0 
 0 
 0 
 0 
 0 
 0 
 0 
 0 
 0 
 0 
 0 
 0 
 0 
 0 
 0 
 0 
 0 
 0 
 0 
 0 
 0 
 0 
 0 
 0 
 0 
 0 
 0 
 0 
 0 
 0 
 0 
 0 
 0 
 0 
 0 
 0 
 0 
 0 
 0 
 0 
 0 
 0 
 0 
 0 
 0 
 0 
 0 
 0 
 0 
 0 
 0 
 0 
 0 
 0 
 0 
 0 
 0 
 0 
 0 
 0 
 0 
 0 
 0 
 0 
 0 
 0 
 0 
 0 
 0 
 0 
 0 
 0 
 0 
 0 
 0 
 0 
 0 
 0 
 0 
 0 
 0 
 0 
 0 
 0 
 0 
 0 
 0 
 0 
 0 
 0 
 0 
 0 
 0 
 0 
 0 
 0 
 0 
 0 
 0 
 0 
 0 
 0 
 0 
 0 
 0 
 0 
 0 
 0 
 0 
 0 
 0 
 0 
 0 
 0 
 0 
 0 
 0 
 0 
 0 
 0 
 0 
 0 
 0 
 0 
 0 
 0 
 0 
 0 
 0 
 0 
 0 
 0 
 0 
 0 
 0 
 0 
 0 
 0 
 0 
 0 
 0 
 0 
 0 
 0 
 0 
 0 
 0 
 0 
 0 
 0 
 0 
 0 
 0 
 0 
 0 
 0 
 0 
 0 
 0 
 0 
 0 
 0 
 0 
 0 
 0 
 0 
 0 
 0 
 0 
 0 
 0 
 0 
 0 
 0 
 0 
 0 
 0 
 0 
 0 
 0 
 0 
 0 
 0 
 0 
 0 
 0 
 0 
 0 
 0 
 0 
 0 
 0 
 0 
 0 
 0 
 0 
 0 
 0 
 0 
 0 
 0 
 0 
 0 
 0 
 0 
 0 
 0 
 0 
 0 
 0 
 0 
 0 
 0 
 0 
 0 
 0 
 0 
 0 
 0 
 0 
 0 
 0 
 0 
 0 
 0 
 0 
 0 
 0 
 0 
 0 
 0 
 0 
 0 
 0 
 0 
 0 
 0 
 0 
 0 
 0 
 0 
 0 
 0 
 0 
 0 
 0 
 0 
 0 
 0 
 0 
 0 
 0 
 0 
 0 
 0 
 0 
 0 
 0 
 0 
 0 
 0 
 0 
 0 
 0 
 0 
 0 
 0 
 0 
 0 
 0 
 0 
 0 
 0 
 0 
 0 
 0 
 0 
 0 
 0 
 0 
 0 
 0 
 0 
 0 
 0 
 0 
 0 
 0 
 0 
 0 
 0 
 0 
 0 
 0 
 0 
 0 
 0 
 0 
 0 
 0 
 0 
 0 
 0 
 0 
 0 
 0 
 0 
 0 
 0 
 0 
 0 
 0 
 0 
 0 
 0 
 0 
 0 
 0 
 0 
 0 
 0 
 0 
 0 
 0 
 0 
 0 
 0 
 0 
 0 
 0 
 0 
 0 
 0 
 0 
 0 
 0 
 0 
 0 
 0 
 0 
 0 
 0 
 0 
 0 
 0 
 0 
 0 
 0 
 0 
 0 
 0 
 0 
 0 
 0 
 0 
 0 
 0 
 0 
 0 
 0 
 0 
 0 
 0 
 0 
 0 
 0 
 0 
 0 
 0 
 0 
 0 
 0 
 0 
 0 
 0 
 0 
 0 
 0 
 0 
 0 
 0 
 0 
 0 
 0 
 0 
 0 
 0 
 0 
 0 
 0 
 0 
 0 
 0 
 0 
 0 
 0 
 0 
 0 
 0 
 0 
 0 
 0 
 0 
 0 
 0 
 0 
 0 
 0 
 0 
 0 
 0 
 0 
 0 
 0 
 0 
 0 
 0 
 0 
 0 
 0 
 0 
 0 
 0 
 0 
 0 
 0 
 0 
 0 
 0 
 0 
 0 
 0 
 0 
 0 
 0 
 0 
 0 
 0 
 0 
 0 
 0 
 0 
 0 
 0 
 0 
 0 
 0 
 0 
 0 
 0 
 0 
 0 
 0 
 0 
 0 
 0 
 0 
 0 
 0 
 0 
 0 
 0 
 0 
 0 
 0 
 0 
 0 
 0 
 0 
 0 
 0 
 0 
 0 
 0 
 0 
 0 
 0 
 0 
 0 
 0 
 0 
 0 
 0 
 0 
 0 
 0 
 0 
 0 
 0 
 0 
 0 
 0 
 0 
 0 
 0 
 0 
 0 
 0 
 0 
 0 
 0 
 0 
 0 
 0 
 0 
 0 
 0 
 0 
 0 
 0 
 0 
 0 
 0 
 0 
 0 
 0 
 0 
 0 
 0 
 0 
 0 
 0 
 0 
 0 
 0 
 0 
 0 
 0 
 0 
 0 
 0 
 0 
 0 
 0 
 0 
 0 
 0 
 0 
 0 
 0 
 0 
 0 
 0 
 0 
 0 
 0 
 0 
 0 
 0 
 0 
 0 
 0 
 0 
 0 
 0 
 0 
 0 
 0 
 0 
 0 
 0 
 0 
 0 
 0 
 0 
 0 
 0 
 0 
 0 
 0 
 0 
 0 
 0 
 0 
 0 
 0 
 0 
 0 
 0 
 0 
 0 
 0 
 0 
 0 
 0 
 0 
 0 
 0 
 0 
 0 
 0 
 0 
 0 
 0 
 0 
 0 
 0 
 0 
 0 
 0 
 0 
 0 
 0 
 0 
 0 
 0 
 0 
 0 
 0 
 0 
 0 
 0 
 0 
 0 
 0 
 0 
 0 
 0 
 0 
 0 
 0 
 0 
 0 
 0 
 0 
 0 
 0 
 
 
 6 
 0 
 0 
 0 
 0 
 0 
 0 
 1 
 0 
 0 
 0 
 0 
 0 
 0 
 0 
 0 
 0 
 0 
 0 
 0 
 0 
 0 
 0 
 0 
 0 
 0 
 0 
 0 
 0 
 0 
 0 
 0 
 0 
 0 
 0 
 0 
 0 
 0 
 0 
 0 
 0 
 0 
 0 
 0 
 0 
 0 
 0 
 0 
 0 
 0 
 0 
 0 
 0 
 0 
 0 
 0 
 0 
 0 
 0 
 0 
 0 
 0 
 0 
 0 
 0 
 0 
 0 
 0 
 0 
 0 
 0 
 0 
 0 
 0 
 0 
 0 
 0 
 0 
 0 
 0 
 0 
 0 
 0 
 0 
 0 
 0 
 0 
 0 
 0 
 0 
 0 
 0 
 0 
 0 
 0 
 0 
 1 
 0 
 0 
 0 
 0 
 0 
 0 
 0 
 0 
 0 
 0 
 0 
 0 
 0 
 0 
 0 
 0 
 0 
 0 
 7 
 0 
 0 
 0 
 0 
 0 
 0 
 0 
 0 
 0 
 0 
 0 
 0 
 0 
 0 
 0 
 0 
 0 
 0 
 0 
 0 
 0 
 0 
 0 
 0 
 0 
 0 
 0 
 0 
 0 
 0 
 0 
 0 
 0 
 0 
 0 
 0 
 0 
 0 
 0 
 0 
 0 
 0 
 0 
 0 
 0 
 0 
 0 
 0 
 0 
 0 
 0 
 0 
 0 
 0 
 0 
 0 
 0 
 0 
 0 
 0 
 0 
 0 
 0 
 0 
 0 
 0 
 0 
 0 
 0 
 0 
 0 
 0 
 0 
 0 
 0 
 0 
 0 
 0 
 0 
 0 
 0 
 0 
 0 
 0 
 0 
 0 
 0 
 0 
 0 
 0 
 0 
 0 
 0 
 0 
 0 
 0 
 0 
 0 
 0 
 0 
 0 
 0 
 0 
 0 
 0 
 0 
 0 
 0 
 0 
 0 
 0 
 0 
 0 
 0 
 0 
 0 
 0 
 0 
 0 
 0 
 0 
 0 
 0 
 0 
 0 
 0 
 0 
 0 
 0 
 0 
 0 
 0 
 0 
 0 
 0 
 0 
 0 
 0 
 0 
 0 
 0 
 0 
 0 
 0 
 0 
 0 
 0 
 0 
 0 
 0 
 0 
 0 
 0 
 0 
 0 
 0 
 0 
 0 
 0 
 0 
 0 
 0 
 0 
 0 
 0 
 0 
 0 
 0 
 0 
 1 
 0 
 0 
 0 
 0 
 0 
 0 
 0 
 0 
 0 
 0 
 0 
 0 
 0 
 0 
 0 
 0 
 0 
 0 
 0 
 0 
 0 
 0 
 0 
 0 
 0 
 0 
 0 
 0 
 0 
 0 
 0 
 0 
 0 
 0 
 0 
 0 
 0 
 0 
 0 
 0 
 0 
 0 
 0 
 0 
 0 
 0 
 0 
 0 
 0 
 0 
 0 
 0 
 0 
 0 
 0 
 0 
 0 
 0 
 0 
 0 
 0 
 0 
 0 
 0 
 0 
 0 
 0 
 0 
 0 
 0 
 0 
 0 
 0 
 0 
 0 
 0 
 0 
 0 
 0 
 0 
 0 
 0 
 0 
 0 
 0 
 0 
 0 
 0 
 0 
 0 
 0 
 0 
 0 
 0 
 0 
 0 
 0 
 0 
 0 
 0 
 0 
 0 
 0 
 0 
 0 
 0 
 0 
 0 
 0 
 0 
 0 
 0 
 0 
 0 
 0 
 0 
 0 
 0 
 0 
 0 
 0 
 0 
 0 
 0 
 0 
 0 
 0 
 0 
 0 
 0 
 0 
 0 
 0 
 0 
 0 
 0 
 0 
 0 
 0 
 0 
 0 
 0 
 0 
 0 
 0 
 0 
 0 
 0 
 0 
 0 
 0 
 0 
 0 
 0 
 0 
 0 
 0 
 0 
 0 
 0 
 0 
 0 
 0 
 0 
 0 
 0 
 0 
 0 
 0 
 0 
 0 
 0 
 0 
 0 
 0 
 0 
 0 
 0 
 0 
 0 
 0 
 0 
 0 
 0 
 0 
 0 
 0 
 0 
 0 
 0 
 0 
 0 
 0 
 0 
 0 
 0 
 0 
 0 
 0 
 0 
 0 
 0 
 0 
 0 
 0 
 0 
 0 
 0 
 0 
 0 
 0 
 0 
 0 
 0 
 0 
 0 
 0 
 0 
 0 
 0 
 0 
 0 
 0 
 0 
 0 
 0 
 0 
 0 
 0 
 0 
 0 
 0 
 0 
 0 
 0 
 0 
 0 
 0 
 0 
 0 
 0 
 0 
 0 
 0 
 0 
 0 
 0 
 0 
 0 
 0 
 0 
 0 
 0 
 0 
 0 
 0 
 0 
 0 
 0 
 0 
 0 
 0 
 0 
 0 
 0 
 0 
 0 
 0 
 0 
 0 
 0 
 0 
 0 
 0 
 0 
 0 
 0 
 0 
 0 
 0 
 0 
 0 
 0 
 0 
 0 
 0 
 0 
 0 
 0 
 0 
 0 
 0 
 0 
 0 
 0 
 0 
 0 
 0 
 0 
 0 
 0 
 0 
 0 
 0 
 0 
 0 
 0 
 0 
 0 
 0 
 0 
 0 
 0 
 0 
 0 
 0 
 0 
 0 
 0 
 0 
 0 
 0 
 0 
 0 
 0 
 0 
 0 
 0 
 0 
 0 
 0 
 0 
 0 
 0 
 0 
 0 
 0 
 0 
 0 
 0 
 0 
 0 
 0 
 0 
 0 
 0 
 0 
 0 
 0 
 0 
 0 
 0 
 0 
 0 
 0 
 0 
 0 
 0 
 0 
 0 
 0 
 0 
 0 
 0 
 0 
 0 
 0 
 0 
 0 
 0 
 0 
 0 
 0 
 0 
 0 
 0 
 0 
 0 
 0 
 0 
 0 
 0 
 0 
 0 
 0 
 0 
 0 
 0 
 0 
 0 
 0 
 0 
 0 
 0 
 0 
 0 
 0 
 0 
 0 
 0 
 0 
 0 
 0 
 0 
 0 
 0 
 0 
 0 
 0 
 0 
 0 
 0 
 0 
 0 
 0 
 0 
 0 
 0 
 0 
 0 
 0 
 0 
 0 
 0 
 0 
 0 
 0 
 0 
 0 
 0 
 0 
 0 
 0 
 0 
 0 
 0 
 0 
 0 
 0 
 0 
 0 
 0 
 0 
 0 
 0 
 0 
 0 
 0 
 0 
 0 
 0 
 0 
 0 
 0 
 0 
 0 
 0 
 0 
 0 
 0 
 0 
 0 
 0 
 0 
 0 
 0 
 0 
 0 
 0 
 0 
 0 
 0 
 0 
 0 
 0 
 0 
 0 
 0 
 0 
 0 
 0 
 0 
 0 
 0 
 0 
 0 
 0 
 0 
 0 
 0 
 0 
 0 
 0 
 0 
 0 
 0 
 0 
 0 
 0 
 0 
 0 
 0 
 0 
 0 
 0 
 0 
 0 
 0 
 0 
 0 
 0 
 0 
 0 
 0 
 0 
 0 
 0 
 0 
 0 
 0 
 0 
 0 
 0 
 0 
 0 
 0 
 0 
 0 
 0 
 0 
 0 
 0 
 0 
 0 
 0 
 0 
 0 
 0 
 0 
 0 
 0 
 
 
 7 
 0 
 0 
 0 
 0 
 0 
 0 
 0 
 1 
 0 
 0 
 0 
 0 
 0 
 0 
 0 
 0 
 0 
 0 
 0 
 0 
 0 
 0 
 0 
 0 
 0 
 0 
 0 
 0 
 0 
 0 
 0 
 0 
 0 
 0 
 0 
 0 
 0 
 0 
 0 
 0 
 0 
 0 
 0 
 0 
 0 
 0 
 0 
 0 
 0 
 0 
 0 
 0 
 0 
 0 
 0 
 0 
 0 
 0 
 0 
 0 
 0 
 0 
 0 
 0 
 0 
 0 
 0 
 0 
 0 
 0 
 0 
 0 
 0 
 0 
 0 
 0 
 0 
 0 
 0 
 0 
 0 
 0 
 0 
 0 
 0 
 0 
 0 
 0 
 0 
 0 
 0 
 0 
 0 
 0 
 0 
 0 
 0 
 0 
 0 
 0 
 0 
 0 
 0 
 0 
 0 
 0 
 0 
 0 
 0 
 0 
 0 
 0 
 0 
 0 
 0 
 0 
 0 
 0 
 0 
 0 
 0 
 0 
 0 
 0 
 0 
 0 
 0 
 0 
 0 
 0 
 0 
 0 
 0 
 0 
 0 
 0 
 0 
 0 
 0 
 0 
 0 
 0 
 0 
 0 
 0 
 0 
 0 
 0 
 0 
 0 
 0 
 0 
 0 
 0 
 0 
 0 
 0 
 0 
 0 
 0 
 0 
 0 
 0 
 0 
 0 
 0 
 0 
 0 
 0 
 0 
 0 
 0 
 0 
 0 
 0 
 0 
 0 
 0 
 0 
 0 
 0 
 0 
 0 
 0 
 0 
 0 
 0 
 0 
 0 
 0 
 0 
 0 
 0 
 0 
 0 
 0 
 0 
 0 
 0 
 0 
 0 
 0 
 0 
 0 
 0 
 0 
 0 
 0 
 0 
 0 
 0 
 0 
 0 
 0 
 0 
 0 
 0 
 0 
 0 
 0 
 0 
 0 
 0 
 0 
 0 
 0 
 0 
 0 
 0 
 0 
 0 
 0 
 0 
 0 
 0 
 0 
 0 
 0 
 0 
 0 
 0 
 0 
 0 
 0 
 0 
 0 
 0 
 0 
 0 
 0 
 0 
 0 
 0 
 0 
 0 
 0 
 0 
 0 
 0 
 0 
 0 
 0 
 0 
 0 
 0 
 0 
 0 
 0 
 0 
 0 
 0 
 0 
 0 
 0 
 0 
 0 
 0 
 0 
 0 
 0 
 0 
 0 
 0 
 0 
 0 
 0 
 0 
 0 
 0 
 0 
 0 
 0 
 0 
 0 
 0 
 0 
 0 
 0 
 0 
 0 
 0 
 0 
 0 
 0 
 0 
 0 
 0 
 0 
 0 
 0 
 0 
 0 
 0 
 0 
 0 
 0 
 0 
 0 
 0 
 0 
 0 
 0 
 0 
 0 
 0 
 0 
 0 
 0 
 0 
 0 
 0 
 0 
 0 
 0 
 0 
 0 
 0 
 0 
 0 
 0 
 0 
 0 
 0 
 0 
 0 
 0 
 0 
 0 
 0 
 0 
 0 
 0 
 0 
 0 
 0 
 0 
 0 
 0 
 0 
 0 
 0 
 0 
 0 
 0 
 0 
 0 
 0 
 0 
 0 
 0 
 0 
 0 
 0 
 0 
 0 
 0 
 0 
 0 
 0 
 0 
 0 
 0 
 0 
 0 
 0 
 0 
 0 
 0 
 0 
 0 
 0 
 0 
 0 
 0 
 0 
 0 
 0 
 0 
 0 
 0 
 0 
 0 
 0 
 0 
 0 
 0 
 0 
 0 
 0 
 0 
 0 
 0 
 0 
 0 
 0 
 0 
 0 
 0 
 0 
 0 
 0 
 0 
 0 
 0 
 0 
 0 
 0 
 0 
 0 
 0 
 0 
 0 
 0 
 0 
 0 
 0 
 0 
 0 
 0 
 0 
 0 
 0 
 0 
 0 
 0 
 0 
 0 
 0 
 0 
 0 
 0 
 0 
 0 
 0 
 0 
 0 
 0 
 0 
 0 
 0 
 0 
 0 
 0 
 0 
 0 
 0 
 0 
 0 
 0 
 0 
 0 
 0 
 0 
 0 
 0 
 0 
 0 
 0 
 0 
 0 
 0 
 0 
 0 
 0 
 0 
 0 
 0 
 0 
 0 
 0 
 0 
 0 
 0 
 0 
 0 
 0 
 0 
 0 
 0 
 0 
 0 
 0 
 0 
 0 
 0 
 0 
 0 
 0 
 0 
 0 
 0 
 0 
 0 
 0 
 0 
 0 
 0 
 0 
 0 
 0 
 0 
 0 
 0 
 0 
 0 
 0 
 0 
 0 
 0 
 0 
 0 
 0 
 0 
 0 
 0 
 0 
 0 
 0 
 0 
 0 
 0 
 0 
 0 
 0 
 0 
 0 
 0 
 0 
 0 
 0 
 0 
 0 
 0 
 0 
 0 
 0 
 0 
 0 
 0 
 0 
 0 
 0 
 0 
 0 
 0 
 0 
 0 
 0 
 0 
 0 
 0 
 0 
 0 
 0 
 0 
 0 
 0 
 0 
 0 
 0 
 0 
 0 
 0 
 0 
 0 
 0 
 0 
 0 
 0 
 0 
 0 
 0 
 0 
 0 
 0 
 0 
 0 
 0 
 0 
 0 
 0 
 0 
 0 
 0 
 0 
 0 
 0 
 0 
 0 
 0 
 0 
 0 
 0 
 0 
 0 
 0 
 0 
 0 
 0 
 0 
 0 
 0 
 0 
 0 
 0 
 0 
 0 
 0 
 0 
 0 
 0 
 0 
 0 
 0 
 0 
 0 
 0 
 0 
 0 
 0 
 0 
 0 
 0 
 0 
 0 
 0 
 0 
 0 
 0 
 0 
 0 
 0 
 0 
 0 
 0 
 0 
 0 
 0 
 0 
 0 
 0 
 0 
 0 
 0 
 0 
 0 
 0 
 0 
 0 
 0 
 0 
 0 
 0 
 0 
 0 
 0 
 0 
 0 
 0 
 0 
 0 
 0 
 0 
 0 
 0 
 0 
 0 
 0 
 0 
 0 
 0 
 0 
 0 
 0 
 0 
 0 
 0 
 0 
 0 
 0 
 0 
 0 
 0 
 0 
 0 
 0 
 0 
 0 
 0 
 0 
 0 
 0 
 0 
 0 
 0 
 0 
 0 
 0 
 0 
 0 
 0 
 0 
 0 
 0 
 0 
 0 
 0 
 0 
 0 
 0 
 0 
 0 
 0 
 0 
 0 
 0 
 0 
 0 
 0 
 0 
 0 
 0 
 0 
 0 
 0 
 0 
 0 
 0 
 0 
 0 
 0 
 0 
 0 
 0 
 0 
 0 
 0 
 0 
 0 
 0 
 0 
 0 
 0 
 0 
 0 
 0 
 0 
 0 
 0 
 0 
 0 
 0 
 0 
 0 
 0 
 0 
 0 
 0 
 0 
 0 
 0 
 0 
 0 
 0 
 0 
 0 
 0 
 0 
 0 
 0 
 0 
 0 
 0 
 0 
 0 
 0 
 0 
 0 
 0 
 0 
 0 
 0 
 0 
 0 
 0 
 0 
 0 
 0 
 0 
 0 
 0 
 0 
 0 
 0 
 0 
 0 
 0 
 0 
 0 
 0 
 0 
 0 
 0 
 0 
 0 
 0 
 
 
 8 
 0 
 0 
 0 
 0 
 0 
 0 
 0 
 0 
 6 
 0 
 0 
 0 
 0 
 0 
 0 
 0 
 0 
 0 
 0 
 0 
 0 
 0 
 0 
 0 
 0 
 0 
 0 
 0 
 0 
 0 
 0 
 0 
 0 
 0 
 0 
 0 
 0 
 0 
 0 
 0 
 0 
 0 
 0 
 0 
 0 
 0 
 0 
 0 
 0 
 0 
 0 
 0 
 0 
 0 
 0 
 0 
 0 
 0 
 0 
 0 
 0 
 0 
 0 
 0 
 0 
 0 
 0 
 0 
 0 
 0 
 0 
 0 
 0 
 0 
 0 
 0 
 0 
 0 
 0 
 0 
 0 
 0 
 0 
 0 
 0 
 0 
 0 
 0 
 0 
 0 
 0 
 0 
 0 
 0 
 0 
 0 
 0 
 0 
 0 
 0 
 0 
 0 
 0 
 0 
 0 
 0 
 0 
 0 
 0 
 0 
 0 
 0 
 0 
 0 
 0 
 0 
 0 
 0 
 0 
 0 
 0 
 0 
 0 
 0 
 0 
 0 
 0 
 0 
 0 
 0 
 0 
 0 
 0 
 0 
 0 
 0 
 0 
 0 
 0 
 0 
 0 
 0 
 0 
 0 
 0 
 0 
 0 
 0 
 0 
 0 
 0 
 0 
 0 
 0 
 0 
 0 
 0 
 0 
 0 
 0 
 0 
 0 
 0 
 0 
 0 
 0 
 0 
 0 
 0 
 0 
 0 
 0 
 0 
 0 
 0 
 0 
 0 
 0 
 0 
 0 
 0 
 0 
 0 
 0 
 0 
 0 
 0 
 0 
 0 
 0 
 0 
 0 
 0 
 0 
 0 
 0 
 0 
 0 
 0 
 0 
 0 
 0 
 0 
 0 
 0 
 0 
 0 
 0 
 0 
 0 
 0 
 0 
 0 
 0 
 0 
 0 
 0 
 0 
 0 
 0 
 0 
 0 
 0 
 0 
 0 
 0 
 0 
 0 
 0 
 0 
 0 
 0 
 0 
 0 
 0 
 0 
 0 
 0 
 0 
 0 
 0 
 0 
 0 
 0 
 0 
 0 
 0 
 0 
 0 
 0 
 0 
 0 
 0 
 0 
 0 
 0 
 0 
 0 
 0 
 0 
 0 
 0 
 0 
 0 
 0 
 0 
 0 
 0 
 0 
 0 
 0 
 0 
 0 
 0 
 0 
 0 
 0 
 0 
 0 
 0 
 0 
 0 
 0 
 0 
 0 
 0 
 0 
 0 
 0 
 0 
 0 
 0 
 0 
 0 
 0 
 0 
 0 
 0 
 0 
 0 
 0 
 0 
 0 
 0 
 0 
 0 
 0 
 0 
 0 
 0 
 0 
 0 
 0 
 0 
 0 
 0 
 0 
 0 
 0 
 0 
 0 
 0 
 0 
 0 
 0 
 0 
 0 
 0 
 0 
 0 
 0 
 0 
 0 
 0 
 0 
 0 
 0 
 0 
 0 
 0 
 0 
 0 
 0 
 0 
 0 
 0 
 0 
 0 
 0 
 0 
 0 
 0 
 0 
 0 
 0 
 0 
 0 
 0 
 0 
 0 
 0 
 0 
 0 
 0 
 0 
 0 
 0 
 0 
 0 
 0 
 0 
 0 
 0 
 0 
 0 
 0 
 0 
 0 
 0 
 0 
 0 
 0 
 0 
 0 
 0 
 0 
 0 
 0 
 0 
 0 
 0 
 0 
 0 
 0 
 0 
 0 
 0 
 0 
 0 
 0 
 0 
 0 
 0 
 0 
 0 
 0 
 0 
 0 
 0 
 0 
 0 
 0 
 0 
 0 
 0 
 0 
 0 
 0 
 0 
 0 
 0 
 0 
 0 
 0 
 0 
 0 
 0 
 0 
 0 
 0 
 0 
 0 
 0 
 0 
 0 
 0 
 0 
 0 
 0 
 0 
 0 
 0 
 0 
 0 
 0 
 0 
 0 
 0 
 0 
 0 
 0 
 0 
 0 
 0 
 0 
 0 
 0 
 0 
 0 
 0 
 0 
 0 
 0 
 0 
 0 
 0 
 0 
 0 
 0 
 0 
 0 
 0 
 0 
 0 
 0 
 0 
 0 
 0 
 0 
 0 
 0 
 0 
 0 
 0 
 0 
 0 
 0 
 0 
 0 
 0 
 0 
 0 
 0 
 0 
 0 
 0 
 0 
 0 
 0 
 0 
 0 
 0 
 0 
 0 
 0 
 0 
 0 
 0 
 0 
 0 
 0 
 0 
 0 
 0 
 0 
 0 
 0 
 0 
 0 
 0 
 0 
 0 
 0 
 0 
 0 
 0 
 0 
 0 
 0 
 0 
 0 
 0 
 0 
 0 
 0 
 0 
 0 
 0 
 0 
 0 
 0 
 0 
 0 
 0 
 0 
 0 
 0 
 0 
 0 
 0 
 0 
 0 
 0 
 0 
 0 
 0 
 0 
 0 
 0 
 0 
 0 
 0 
 0 
 0 
 0 
 0 
 0 
 0 
 0 
 0 
 0 
 0 
 0 
 0 
 0 
 0 
 0 
 0 
 0 
 0 
 0 
 0 
 0 
 0 
 0 
 0 
 0 
 0 
 0 
 0 
 0 
 0 
 0 
 0 
 0 
 0 
 0 
 0 
 0 
 0 
 0 
 0 
 0 
 0 
 0 
 0 
 0 
 0 
 0 
 0 
 0 
 0 
 0 
 0 
 0 
 0 
 0 
 0 
 0 
 0 
 0 
 0 
 0 
 0 
 0 
 0 
 0 
 0 
 0 
 0 
 0 
 0 
 0 
 0 
 0 
 0 
 0 
 0 
 0 
 0 
 0 
 0 
 0 
 0 
 0 
 0 
 0 
 0 
 0 
 0 
 0 
 0 
 0 
 0 
 0 
 0 
 0 
 0 
 0 
 0 
 0 
 0 
 0 
 0 
 0 
 0 
 0 
 0 
 0 
 0 
 0 
 0 
 0 
 0 
 0 
 0 
 0 
 0 
 0 
 0 
 0 
 0 
 0 
 0 
 0 
 0 
 0 
 0 
 0 
 0 
 0 
 0 
 0 
 0 
 0 
 0 
 0 
 0 
 0 
 0 
 0 
 0 
 0 
 0 
 0 
 0 
 0 
 0 
 0 
 0 
 0 
 0 
 0 
 0 
 0 
 0 
 0 
 0 
 0 
 0 
 0 
 0 
 0 
 0 
 0 
 0 
 0 
 0 
 0 
 0 
 0 
 0 
 0 
 0 
 0 
 0 
 0 
 0 
 0 
 0 
 0 
 0 
 0 
 0 
 0 
 0 
 0 
 0 
 0 
 0 
 0 
 0 
 0 
 0 
 0 
 0 
 0 
 0 
 0 
 0 
 0 
 0 
 0 
 0 
 0 
 0 
 0 
 0 
 0 
 0 
 0 
 0 
 0 
 0 
 0 
 0 
 0 
 0 
 0 
 0 
 0 
 0 
 0 
 0 
 0 
 0 
 0 
 0 
 0 
 0 
 0 
 0 
 0 
 0 
 0 
 0 
 0 
 0 
 0 
 0 
 0 
 0 
 0 
 0 
 0 
 0 
 0 
 0 
 0 
 0 
 0 
 0 
 0 
 0 
 0 
 0 
 0 
 0 
 0 
 0 
 0 
 0 
 0 
 0 
 0 
 0 
 
 
 9 
 0 
 0 
 0 
 0 
 0 
 0 
 0 
 0 
 0 
 3 
 0 
 0 
 0 
 0 
 0 
 0 
 0 
 0 
 0 
 0 
 0 
 0 
 0 
 0 
 0 
 0 
 0 
 0 
 0 
 0 
 0 
 0 
 0 
 0 
 0 
 0 
 0 
 0 
 0 
 0 
 0 
 0 
 0 
 0 
 0 
 0 
 0 
 0 
 0 
 0 
 0 
 0 
 0 
 0 
 0 
 0 
 0 
 0 
 0 
 0 
 0 
 0 
 0 
 0 
 0 
 0 
 0 
 0 
 0 
 0 
 0 
 0 
 0 
 0 
 0 
 0 
 0 
 0 
 0 
 0 
 0 
 0 
 0 
 0 
 0 
 0 
 0 
 0 
 0 
 0 
 0 
 0 
 0 
 0 
 0 
 0 
 0 
 0 
 0 
 0 
 0 
 0 
 0 
 0 
 0 
 0 
 0 
 0 
 0 
 0 
 0 
 0 
 0 
 0 
 0 
 0 
 0 
 0 
 0 
 0 
 0 
 0 
 0 
 0 
 0 
 0 
 0 
 0 
 0 
 0 
 0 
 0 
 0 
 0 
 0 
 0 
 0 
 0 
 0 
 0 
 0 
 0 
 0 
 0 
 0 
 0 
 0 
 0 
 0 
 0 
 0 
 0 
 0 
 0 
 0 
 0 
 0 
 0 
 0 
 0 
 0 
 0 
 0 
 0 
 0 
 0 
 0 
 0 
 0 
 0 
 0 
 0 
 0 
 0 
 0 
 0 
 0 
 0 
 0 
 0 
 0 
 0 
 0 
 0 
 0 
 0 
 0 
 0 
 0 
 0 
 0 
 0 
 0 
 0 
 0 
 0 
 0 
 0 
 0 
 0 
 0 
 0 
 0 
 0 
 0 
 0 
 0 
 0 
 0 
 0 
 0 
 0 
 0 
 0 
 0 
 0 
 0 
 0 
 0 
 0 
 0 
 0 
 0 
 0 
 0 
 0 
 0 
 0 
 0 
 0 
 0 
 0 
 0 
 0 
 0 
 0 
 0 
 0 
 0 
 0 
 0 
 0 
 0 
 0 
 0 
 0 
 0 
 0 
 0 
 0 
 0 
 0 
 0 
 0 
 0 
 0 
 0 
 0 
 0 
 0 
 0 
 0 
 0 
 0 
 0 
 0 
 0 
 0 
 0 
 0 
 0 
 0 
 0 
 0 
 0 
 0 
 0 
 0 
 0 
 0 
 0 
 0 
 0 
 0 
 0 
 0 
 0 
 0 
 0 
 0 
 0 
 0 
 0 
 0 
 0 
 0 
 0 
 0 
 0 
 0 
 0 
 0 
 0 
 0 
 0 
 0 
 0 
 0 
 0 
 0 
 0 
 0 
 0 
 0 
 0 
 0 
 0 
 0 
 0 
 0 
 0 
 0 
 0 
 0 
 0 
 0 
 0 
 0 
 0 
 0 
 0 
 0 
 0 
 0 
 0 
 0 
 0 
 0 
 0 
 0 
 0 
 0 
 0 
 0 
 0 
 0 
 0 
 0 
 0 
 0 
 0 
 0 
 0 
 0 
 0 
 0 
 0 
 0 
 0 
 0 
 0 
 0 
 0 
 0 
 0 
 0 
 0 
 0 
 0 
 0 
 0 
 0 
 0 
 0 
 0 
 0 
 0 
 0 
 0 
 0 
 0 
 0 
 0 
 0 
 0 
 0 
 0 
 0 
 0 
 0 
 0 
 0 
 0 
 0 
 0 
 0 
 0 
 0 
 0 
 0 
 0 
 0 
 0 
 0 
 0 
 0 
 0 
 0 
 0 
 0 
 0 
 0 
 0 
 0 
 0 
 0 
 0 
 0 
 0 
 0 
 0 
 0 
 0 
 0 
 0 
 0 
 0 
 0 
 0 
 0 
 0 
 0 
 0 
 0 
 0 
 0 
 0 
 0 
 0 
 0 
 0 
 0 
 0 
 0 
 0 
 0 
 0 
 0 
 0 
 0 
 0 
 0 
 0 
 0 
 0 
 0 
 0 
 0 
 0 
 0 
 0 
 0 
 0 
 0 
 0 
 0 
 0 
 0 
 0 
 0 
 0 
 0 
 0 
 0 
 0 
 0 
 0 
 0 
 0 
 0 
 0 
 0 
 0 
 0 
 0 
 0 
 0 
 0 
 0 
 0 
 0 
 0 
 0 
 0 
 0 
 0 
 0 
 0 
 0 
 0 
 0 
 0 
 0 
 0 
 0 
 0 
 0 
 0 
 0 
 0 
 0 
 0 
 0 
 0 
 0 
 0 
 0 
 0 
 0 
 0 
 0 
 0 
 0 
 0 
 0 
 0 
 0 
 0 
 0 
 0 
 0 
 0 
 0 
 0 
 0 
 0 
 0 
 0 
 0 
 0 
 0 
 0 
 0 
 0 
 0 
 0 
 0 
 0 
 0 
 0 
 0 
 0 
 0 
 0 
 0 
 0 
 0 
 0 
 0 
 0 
 0 
 0 
 0 
 0 
 0 
 0 
 0 
 0 
 0 
 0 
 0 
 0 
 0 
 0 
 0 
 0 
 0 
 0 
 0 
 0 
 0 
 0 
 0 
 0 
 0 
 0 
 0 
 0 
 0 
 0 
 0 
 0 
 0 
 0 
 0 
 0 
 0 
 0 
 0 
 0 
 0 
 0 
 0 
 0 
 0 
 0 
 0 
 0 
 0 
 0 
 0 
 0 
 0 
 0 
 0 
 0 
 0 
 0 
 0 
 0 
 0 
 0 
 0 
 0 
 0 
 0 
 0 
 0 
 0 
 0 
 0 
 0 
 0 
 0 
 0 
 0 
 0 
 0 
 0 
 0 
 0 
 0 
 0 
 0 
 0 
 0 
 0 
 0 
 0 
 0 
 0 
 0 
 0 
 0 
 0 
 0 
 0 
 0 
 0 
 0 
 0 
 0 
 0 
 0 
 0 
 0 
 0 
 0 
 0 
 0 
 0 
 0 
 0 
 0 
 0 
 0 
 0 
 0 
 0 
 0 
 0 
 0 
 0 
 0 
 0 
 0 
 0 
 0 
 0 
 0 
 0 
 0 
 0 
 0 
 0 
 0 
 0 
 0 
 0 
 0 
 0 
 0 
 0 
 0 
 0 
 0 
 0 
 0 
 0 
 0 
 0 
 0 
 0 
 0 
 0 
 0 
 0 
 0 
 0 
 0 
 0 
 0 
 0 
 0 
 0 
 0 
 0 
 0 
 0 
 0 
 0 
 0 
 0 
 0 
 0 
 0 
 0 
 0 
 0 
 0 
 0 
 0 
 0 
 0 
 0 
 0 
 0 
 0 
 0 
 0 
 0 
 0 
 0 
 0 
 0 
 0 
 0 
 0 
 0 
 0 
 0 
 0 
 0 
 0 
 0 
 0 
 0 
 0 
 0 
 0 
 0 
 0 
 0 
 0 
 0 
 0 
 0 
 0 
 0 
 0 
 0 
 0 
 0 
 0 
 0 
 0 
 0 
 0 
 0 
 0 
 0 
 0 
 0 
 0 
 0 
 0 
 0 
 0 
 0 
 0 
 0 
 0 
 0 
 0 
 0 
 0 
 0 
 0 
 0 
 0 
 0 
 0 
 0 
 0 
 0 
 0 
 0 
 0 
 0 
 0 
 0 
 0 
 0 
 0 
 0 
 0 
 
 
 10 
 0 
 0 
 0 
 0 
 0 
 0 
 0 
 0 
 0 
 0 
 7 
 0 
 0 
 0 
 0 
 0 
 0 
 0 
 0 
 0 
 0 
 0 
 0 
 0 
 0 
 0 
 0 
 0 
 0 
 0 
 0 
 0 
 0 
 0 
 0 
 0 
 0 
 0 
 0 
 0 
 0 
 0 
 0 
 0 
 0 
 0 
 0 
 0 
 0 
 0 
 0 
 0 
 0 
 0 
 0 
 0 
 0 
 0 
 0 
 0 
 0 
 0 
 0 
 0 
 0 
 0 
 0 
 0 
 0 
 0 
 0 
 0 
 0 
 0 
 0 
 0 
 0 
 0 
 0 
 0 
 0 
 0 
 0 
 0 
 0 
 0 
 0 
 0 
 0 
 0 
 0 
 0 
 0 
 0 
 0 
 0 
 0 
 0 
 0 
 0 
 0 
 0 
 0 
 0 
 0 
 0 
 0 
 0 
 0 
 0 
 0 
 0 
 0 
 0 
 0 
 0 
 0 
 0 
 0 
 0 
 0 
 0 
 0 
 0 
 0 
 0 
 0 
 0 
 0 
 0 
 0 
 0 
 0 
 0 
 0 
 0 
 0 
 0 
 0 
 0 
 0 
 0 
 0 
 0 
 0 
 0 
 0 
 0 
 0 
 0 
 0 
 0 
 0 
 0 
 0 
 0 
 0 
 0 
 0 
 0 
 0 
 0 
 0 
 0 
 0 
 0 
 0 
 0 
 0 
 0 
 0 
 0 
 0 
 0 
 0 
 0 
 0 
 0 
 0 
 0 
 0 
 0 
 0 
 0 
 0 
 0 
 0 
 0 
 0 
 0 
 0 
 0 
 0 
 0 
 0 
 0 
 0 
 0 
 0 
 0 
 0 
 0 
 0 
 0 
 0 
 0 
 0 
 0 
 0 
 0 
 0 
 0 
 0 
 0 
 0 
 0 
 0 
 0 
 0 
 0 
 0 
 0 
 0 
 0 
 0 
 0 
 0 
 0 
 0 
 0 
 0 
 0 
 0 
 0 
 0 
 0 
 0 
 0 
 0 
 0 
 0 
 0 
 0 
 0 
 0 
 0 
 0 
 0 
 0 
 0 
 0 
 0 
 0 
 0 
 0 
 0 
 0 
 0 
 0 
 0 
 0 
 0 
 0 
 0 
 0 
 0 
 0 
 0 
 0 
 0 
 0 
 0 
 0 
 0 
 0 
 0 
 0 
 0 
 0 
 0 
 0 
 0 
 0 
 0 
 0 
 0 
 0 
 0 
 0 
 0 
 0 
 0 
 0 
 0 
 0 
 0 
 0 
 0 
 0 
 0 
 0 
 0 
 0 
 0 
 0 
 0 
 0 
 0 
 0 
 0 
 0 
 0 
 0 
 0 
 0 
 0 
 0 
 0 
 0 
 0 
 0 
 0 
 0 
 0 
 0 
 0 
 0 
 0 
 0 
 0 
 0 
 0 
 0 
 0 
 0 
 0 
 0 
 0 
 0 
 0 
 0 
 0 
 0 
 0 
 0 
 0 
 0 
 0 
 0 
 0 
 0 
 0 
 0 
 0 
 0 
 0 
 0 
 0 
 0 
 0 
 0 
 0 
 0 
 0 
 0 
 0 
 0 
 0 
 0 
 0 
 0 
 0 
 0 
 0 
 0 
 0 
 0 
 0 
 0 
 0 
 0 
 0 
 0 
 0 
 0 
 0 
 0 
 0 
 0 
 0 
 0 
 0 
 0 
 0 
 0 
 0 
 0 
 0 
 0 
 0 
 0 
 0 
 0 
 0 
 0 
 0 
 0 
 0 
 0 
 0 
 0 
 0 
 0 
 0 
 0 
 0 
 0 
 0 
 0 
 0 
 0 
 0 
 0 
 0 
 0 
 0 
 0 
 0 
 0 
 0 
 0 
 0 
 0 
 0 
 0 
 0 
 0 
 0 
 0 
 0 
 0 
 0 
 0 
 0 
 0 
 0 
 0 
 0 
 0 
 0 
 0 
 0 
 0 
 0 
 0 
 0 
 0 
 0 
 0 
 0 
 0 
 0 
 0 
 0 
 0 
 0 
 0 
 0 
 0 
 0 
 0 
 0 
 0 
 0 
 0 
 0 
 0 
 0 
 0 
 0 
 0 
 0 
 0 
 0 
 0 
 0 
 0 
 0 
 0 
 0 
 0 
 0 
 0 
 0 
 0 
 0 
 0 
 0 
 0 
 0 
 0 
 0 
 0 
 0 
 0 
 0 
 0 
 0 
 0 
 0 
 0 
 0 
 0 
 0 
 0 
 0 
 0 
 0 
 0 
 0 
 0 
 0 
 0 
 0 
 0 
 0 
 0 
 0 
 0 
 0 
 0 
 0 
 0 
 0 
 0 
 0 
 0 
 0 
 0 
 0 
 0 
 0 
 0 
 0 
 0 
 0 
 0 
 0 
 0 
 0 
 0 
 0 
 0 
 0 
 0 
 0 
 0 
 0 
 0 
 0 
 0 
 0 
 0 
 0 
 0 
 0 
 0 
 0 
 0 
 0 
 0 
 0 
 0 
 0 
 0 
 0 
 0 
 0 
 0 
 0 
 0 
 0 
 0 
 0 
 0 
 0 
 0 
 0 
 0 
 0 
 0 
 0 
 0 
 0 
 0 
 0 
 0 
 0 
 0 
 0 
 0 
 0 
 0 
 0 
 0 
 0 
 0 
 0 
 0 
 0 
 0 
 0 
 0 
 0 
 0 
 0 
 0 
 0 
 0 
 0 
 0 
 0 
 0 
 0 
 0 
 0 
 0 
 0 
 0 
 0 
 0 
 0 
 0 
 0 
 0 
 0 
 0 
 0 
 0 
 0 
 0 
 0 
 0 
 0 
 0 
 0 
 0 
 0 
 0 
 0 
 0 
 0 
 0 
 0 
 0 
 0 
 0 
 0 
 0 
 0 
 0 
 0 
 0 
 0 
 0 
 0 
 0 
 0 
 0 
 0 
 0 
 0 
 0 
 0 
 0 
 0 
 0 
 0 
 0 
 0 
 0 
 0 
 0 
 0 
 0 
 0 
 0 
 0 
 0 
 0 
 0 
 0 
 0 
 0 
 0 
 0 
 0 
 0 
 0 
 0 
 0 
 0 
 0 
 0 
 0 
 0 
 0 
 0 
 0 
 0 
 0 
 0 
 0 
 0 
 0 
 0 
 0 
 0 
 0 
 0 
 0 
 0 
 0 
 0 
 0 
 0 
 0 
 0 
 0 
 0 
 0 
 0 
 0 
 0 
 0 
 0 
 0 
 0 
 0 
 0 
 0 
 0 
 0 
 0 
 0 
 0 
 0 
 0 
 0 
 0 
 0 
 0 
 0 
 0 
 0 
 0 
 0 
 0 
 0 
 0 
 0 
 0 
 0 
 0 
 0 
 0 
 0 
 0 
 0 
 0 
 0 
 0 
 0 
 0 
 0 
 0 
 0 
 0 
 0 
 0 
 0 
 0 
 0 
 0 
 0 
 0 
 0 
 0 
 0 
 0 
 0 
 0 
 0 
 0 
 0 
 0 
 0 
 0 
 0 
 0 
 0 
 0 
 0 
 0 
 0 
 0 
 0 
 0 
 0 
 0 
 0 
 0 
 0 
 0 
 0 
 0 
 0 
 0 
 0 
 0 
 0 
 0 
 0 
 0 
 0 
 0 
 
 
 11 
 0 
 0 
 0 
 0 
 0 
 0 
 0 
 0 
 0 
 0 
 0 
 11 
 0 
 0 
 0 
 0 
 0 
 0 
 0 
 0 
 0 
 0 
 0 
 0 
 0 
 0 
 0 
 0 
 0 
 0 
 0 
 0 
 0 
 0 
 0 
 0 
 0 
 0 
 0 
 0 
 0 
 0 
 0 
 0 
 0 
 0 
 0 
 0 
 0 
 0 
 0 
 0 
 0 
 0 
 0 
 0 
 0 
 0 
 0 
 0 
 0 
 0 
 0 
 0 
 0 
 0 
 0 
 0 
 0 
 0 
 0 
 0 
 0 
 0 
 0 
 0 
 0 
 0 
 0 
 0 
 0 
 0 
 0 
 0 
 0 
 0 
 0 
 0 
 0 
 0 
 0 
 0 
 0 
 0 
 0 
 0 
 0 
 0 
 0 
 0 
 0 
 0 
 0 
 0 
 0 
 0 
 0 
 0 
 0 
 0 
 0 
 0 
 0 
 0 
 0 
 0 
 0 
 0 
 0 
 0 
 0 
 0 
 0 
 0 
 0 
 0 
 0 
 0 
 0 
 0 
 0 
 0 
 0 
 0 
 0 
 0 
 0 
 0 
 0 
 0 
 0 
 0 
 0 
 0 
 0 
 0 
 0 
 0 
 0 
 0 
 0 
 0 
 0 
 0 
 0 
 0 
 0 
 0 
 0 
 0 
 0 
 0 
 0 
 0 
 0 
 0 
 0 
 0 
 0 
 0 
 0 
 0 
 0 
 0 
 0 
 0 
 0 
 0 
 0 
 0 
 0 
 0 
 0 
 0 
 0 
 0 
 0 
 0 
 0 
 0 
 0 
 0 
 0 
 0 
 0 
 0 
 0 
 0 
 0 
 0 
 0 
 0 
 0 
 0 
 0 
 0 
 0 
 0 
 0 
 0 
 0 
 0 
 0 
 0 
 0 
 0 
 0 
 0 
 0 
 0 
 0 
 0 
 0 
 0 
 0 
 0 
 0 
 0 
 0 
 0 
 0 
 0 
 0 
 0 
 0 
 0 
 0 
 0 
 0 
 0 
 0 
 0 
 0 
 0 
 0 
 0 
 0 
 0 
 0 
 0 
 0 
 0 
 0 
 0 
 0 
 0 
 0 
 0 
 0 
 0 
 0 
 0 
 0 
 0 
 0 
 0 
 0 
 0 
 0 
 0 
 0 
 0 
 0 
 0 
 0 
 0 
 0 
 0 
 0 
 0 
 0 
 0 
 0 
 0 
 0 
 0 
 0 
 0 
 0 
 0 
 0 
 0 
 0 
 0 
 0 
 0 
 0 
 0 
 0 
 0 
 0 
 0 
 0 
 0 
 0 
 0 
 0 
 0 
 0 
 0 
 0 
 0 
 0 
 0 
 0 
 0 
 0 
 0 
 0 
 0 
 0 
 0 
 0 
 0 
 0 
 0 
 0 
 0 
 0 
 0 
 0 
 0 
 0 
 0 
 0 
 0 
 0 
 0 
 0 
 0 
 0 
 0 
 0 
 0 
 0 
 0 
 0 
 0 
 0 
 0 
 0 
 0 
 0 
 0 
 0 
 0 
 0 
 0 
 0 
 0 
 0 
 0 
 0 
 0 
 0 
 0 
 0 
 0 
 0 
 0 
 0 
 0 
 0 
 0 
 0 
 0 
 0 
 0 
 0 
 0 
 0 
 0 
 0 
 0 
 0 
 0 
 0 
 0 
 0 
 0 
 0 
 0 
 0 
 0 
 0 
 0 
 0 
 0 
 0 
 0 
 0 
 0 
 0 
 0 
 0 
 0 
 0 
 0 
 0 
 0 
 0 
 0 
 0 
 0 
 0 
 0 
 0 
 0 
 0 
 0 
 0 
 0 
 0 
 0 
 0 
 0 
 0 
 0 
 0 
 0 
 0 
 0 
 0 
 0 
 0 
 0 
 0 
 0 
 0 
 0 
 0 
 0 
 0 
 0 
 0 
 0 
 0 
 0 
 0 
 0 
 0 
 0 
 0 
 0 
 0 
 0 
 0 
 0 
 0 
 0 
 0 
 0 
 0 
 0 
 0 
 0 
 0 
 0 
 0 
 0 
 0 
 0 
 0 
 0 
 0 
 0 
 0 
 0 
 0 
 0 
 0 
 0 
 0 
 0 
 0 
 0 
 0 
 0 
 0 
 0 
 0 
 0 
 0 
 0 
 0 
 0 
 0 
 0 
 0 
 0 
 0 
 0 
 0 
 0 
 0 
 0 
 0 
 0 
 0 
 0 
 0 
 0 
 0 
 0 
 0 
 0 
 0 
 0 
 0 
 0 
 0 
 0 
 0 
 0 
 0 
 0 
 0 
 0 
 0 
 0 
 0 
 0 
 0 
 0 
 0 
 0 
 0 
 0 
 0 
 0 
 0 
 0 
 0 
 0 
 0 
 0 
 0 
 0 
 0 
 0 
 0 
 0 
 0 
 0 
 0 
 0 
 0 
 0 
 0 
 0 
 0 
 0 
 0 
 0 
 0 
 0 
 0 
 0 
 0 
 0 
 0 
 0 
 0 
 0 
 0 
 0 
 0 
 0 
 0 
 0 
 0 
 0 
 0 
 0 
 0 
 0 
 0 
 0 
 0 
 0 
 0 
 0 
 0 
 0 
 0 
 0 
 0 
 0 
 0 
 0 
 0 
 0 
 0 
 0 
 0 
 0 
 0 
 0 
 0 
 0 
 0 
 0 
 0 
 0 
 0 
 0 
 0 
 0 
 0 
 0 
 0 
 0 
 0 
 0 
 0 
 0 
 0 
 0 
 0 
 0 
 0 
 0 
 0 
 0 
 0 
 0 
 0 
 0 
 0 
 0 
 0 
 0 
 0 
 0 
 0 
 0 
 0 
 0 
 0 
 0 
 0 
 0 
 0 
 0 
 0 
 0 
 0 
 0 
 0 
 0 
 0 
 0 
 0 
 0 
 0 
 0 
 0 
 0 
 0 
 0 
 0 
 0 
 0 
 0 
 0 
 0 
 0 
 0 
 0 
 0 
 0 
 0 
 0 
 0 
 0 
 0 
 0 
 0 
 0 
 0 
 0 
 0 
 0 
 0 
 0 
 0 
 0 
 0 
 0 
 0 
 0 
 0 
 0 
 0 
 0 
 0 
 0 
 0 
 0 
 0 
 0 
 0 
 0 
 0 
 0 
 0 
 0 
 0 
 0 
 0 
 0 
 0 
 0 
 0 
 0 
 0 
 0 
 0 
 0 
 0 
 0 
 0 
 0 
 0 
 0 
 0 
 0 
 0 
 0 
 0 
 0 
 0 
 0 
 0 
 0 
 0 
 0 
 0 
 0 
 0 
 0 
 0 
 0 
 0 
 0 
 0 
 0 
 0 
 0 
 0 
 0 
 0 
 0 
 0 
 0 
 0 
 0 
 0 
 0 
 0 
 0 
 0 
 0 
 0 
 0 
 0 
 0 
 0 
 0 
 0 
 0 
 0 
 0 
 0 
 0 
 0 
 0 
 0 
 0 
 0 
 0 
 0 
 0 
 0 
 0 
 0 
 0 
 0 
 0 
 0 
 0 
 0 
 0 
 0 
 0 
 0 
 0 
 0 
 0 
 0 
 0 
 0 
 0 
 0 
 0 
 0 
 0 
 0 
 0 
 0 
 0 
 0 
 0 
 0 
 0 
 0 
 
 
 12 
 0 
 0 
 0 
 0 
 0 
 0 
 0 
 0 
 0 
 0 
 0 
 0 
 11 
 0 
 0 
 0 
 0 
 0 
 0 
 0 
 0 
 0 
 0 
 0 
 0 
 0 
 0 
 0 
 0 
 0 
 0 
 0 
 0 
 0 
 0 
 0 
 0 
 0 
 0 
 0 
 0 
 0 
 0 
 0 
 0 
 0 
 0 
 0 
 0 
 0 
 0 
 0 
 0 
 0 
 0 
 0 
 0 
 0 
 0 
 0 
 0 
 0 
 0 
 0 
 0 
 0 
 0 
 0 
 0 
 0 
 0 
 0 
 0 
 0 
 0 
 0 
 0 
 0 
 0 
 0 
 0 
 0 
 0 
 0 
 0 
 0 
 0 
 0 
 0 
 0 
 0 
 0 
 0 
 0 
 0 
 0 
 0 
 0 
 0 
 0 
 0 
 0 
 0 
 0 
 0 
 0 
 0 
 0 
 0 
 0 
 0 
 0 
 0 
 0 
 0 
 0 
 0 
 0 
 0 
 0 
 0 
 0 
 0 
 0 
 0 
 0 
 0 
 0 
 0 
 0 
 0 
 0 
 0 
 0 
 0 
 0 
 0 
 0 
 0 
 0 
 0 
 0 
 0 
 0 
 0 
 0 
 0 
 0 
 0 
 0 
 0 
 0 
 0 
 0 
 0 
 0 
 0 
 0 
 0 
 0 
 0 
 0 
 0 
 0 
 0 
 0 
 0 
 0 
 0 
 0 
 0 
 0 
 0 
 0 
 0 
 0 
 0 
 0 
 0 
 0 
 0 
 0 
 0 
 0 
 0 
 0 
 0 
 0 
 0 
 0 
 0 
 0 
 0 
 0 
 0 
 0 
 0 
 0 
 0 
 0 
 0 
 0 
 0 
 0 
 0 
 0 
 0 
 0 
 0 
 0 
 0 
 0 
 0 
 0 
 0 
 0 
 0 
 0 
 0 
 0 
 0 
 0 
 0 
 0 
 0 
 0 
 0 
 0 
 0 
 0 
 0 
 0 
 0 
 0 
 0 
 0 
 0 
 0 
 0 
 0 
 0 
 0 
 0 
 0 
 0 
 0 
 0 
 0 
 0 
 0 
 0 
 0 
 0 
 0 
 0 
 0 
 0 
 0 
 0 
 0 
 0 
 0 
 0 
 0 
 0 
 0 
 0 
 0 
 0 
 0 
 0 
 0 
 0 
 0 
 0 
 0 
 0 
 0 
 0 
 0 
 0 
 0 
 0 
 0 
 0 
 0 
 0 
 0 
 0 
 0 
 0 
 0 
 0 
 0 
 0 
 0 
 0 
 0 
 0 
 0 
 0 
 0 
 0 
 0 
 0 
 0 
 0 
 0 
 0 
 0 
 0 
 0 
 0 
 0 
 0 
 0 
 0 
 0 
 0 
 0 
 0 
 0 
 0 
 0 
 0 
 0 
 0 
 0 
 0 
 0 
 0 
 0 
 0 
 0 
 0 
 0 
 0 
 0 
 0 
 0 
 0 
 0 
 0 
 0 
 0 
 0 
 0 
 0 
 0 
 0 
 0 
 0 
 0 
 0 
 0 
 0 
 0 
 0 
 0 
 0 
 0 
 0 
 0 
 0 
 0 
 0 
 0 
 0 
 0 
 0 
 0 
 0 
 0 
 0 
 0 
 0 
 0 
 0 
 0 
 0 
 0 
 0 
 0 
 0 
 0 
 0 
 0 
 0 
 0 
 0 
 0 
 0 
 0 
 0 
 0 
 0 
 0 
 0 
 0 
 0 
 0 
 0 
 0 
 0 
 0 
 0 
 0 
 0 
 0 
 0 
 0 
 0 
 0 
 0 
 0 
 0 
 0 
 0 
 0 
 0 
 0 
 0 
 0 
 0 
 0 
 0 
 0 
 0 
 0 
 0 
 0 
 0 
 0 
 0 
 0 
 0 
 0 
 0 
 0 
 0 
 0 
 0 
 0 
 0 
 0 
 0 
 0 
 0 
 0 
 0 
 0 
 0 
 0 
 0 
 0 
 0 
 0 
 0 
 0 
 0 
 0 
 0 
 0 
 0 
 0 
 0 
 0 
 0 
 0 
 0 
 0 
 0 
 0 
 0 
 0 
 0 
 0 
 0 
 0 
 0 
 0 
 0 
 0 
 0 
 0 
 0 
 0 
 0 
 0 
 0 
 0 
 0 
 0 
 0 
 0 
 0 
 0 
 0 
 0 
 0 
 0 
 0 
 0 
 0 
 0 
 0 
 0 
 0 
 0 
 0 
 0 
 0 
 0 
 0 
 0 
 0 
 0 
 0 
 0 
 0 
 0 
 0 
 0 
 0 
 0 
 0 
 0 
 0 
 0 
 0 
 0 
 0 
 0 
 0 
 0 
 0 
 0 
 0 
 0 
 0 
 0 
 0 
 0 
 0 
 0 
 0 
 0 
 0 
 0 
 0 
 0 
 0 
 0 
 0 
 0 
 0 
 0 
 0 
 0 
 0 
 0 
 0 
 0 
 0 
 0 
 0 
 0 
 0 
 0 
 0 
 0 
 0 
 0 
 0 
 0 
 0 
 0 
 0 
 0 
 0 
 0 
 0 
 0 
 0 
 0 
 0 
 0 
 0 
 0 
 0 
 0 
 0 
 0 
 0 
 0 
 0 
 0 
 0 
 0 
 0 
 0 
 0 
 0 
 0 
 0 
 0 
 0 
 0 
 0 
 0 
 0 
 0 
 0 
 0 
 0 
 0 
 0 
 0 
 0 
 0 
 0 
 0 
 0 
 0 
 0 
 0 
 0 
 0 
 0 
 0 
 0 
 0 
 0 
 0 
 0 
 0 
 0 
 0 
 0 
 0 
 0 
 0 
 0 
 0 
 0 
 0 
 0 
 0 
 0 
 0 
 0 
 0 
 0 
 0 
 0 
 0 
 0 
 0 
 0 
 0 
 0 
 0 
 0 
 0 
 0 
 0 
 0 
 0 
 0 
 0 
 0 
 0 
 0 
 0 
 0 
 0 
 0 
 0 
 0 
 0 
 0 
 0 
 0 
 0 
 0 
 0 
 0 
 0 
 0 
 0 
 0 
 0 
 0 
 0 
 0 
 0 
 0 
 0 
 0 
 0 
 0 
 0 
 0 
 0 
 0 
 0 
 0 
 0 
 0 
 0 
 0 
 0 
 0 
 0 
 0 
 0 
 0 
 0 
 0 
 0 
 0 
 0 
 0 
 0 
 0 
 0 
 0 
 0 
 0 
 0 
 0 
 0 
 0 
 0 
 0 
 0 
 0 
 0 
 0 
 0 
 0 
 0 
 0 
 0 
 0 
 0 
 0 
 0 
 0 
 0 
 0 
 0 
 0 
 0 
 0 
 0 
 0 
 0 
 0 
 0 
 0 
 0 
 0 
 0 
 0 
 0 
 0 
 0 
 0 
 0 
 0 
 0 
 0 
 0 
 0 
 0 
 0 
 0 
 0 
 0 
 0 
 0 
 0 
 0 
 0 
 0 
 0 
 0 
 0 
 0 
 0 
 0 
 0 
 0 
 0 
 0 
 0 
 0 
 0 
 0 
 0 
 0 
 0 
 0 
 0 
 0 
 0 
 0 
 0 
 0 
 0 
 0 
 0 
 0 
 0 
 0 
 0 
 0 
 0 
 0 
 0 
 0 
 0 
 0 
 0 
 0 
 
 
 13 
 0 
 0 
 0 
 0 
 0 
 0 
 0 
 0 
 0 
 0 
 0 
 0 
 0 
 0 
 0 
 0 
 0 
 0 
 4 
 0 
 0 
 0 
 0 
 0 
 0 
 0 
 0 
 0 
 0 
 0 
 0 
 0 
 0 
 0 
 0 
 0 
 0 
 0 
 0 
 0 
 0 
 0 
 0 
 0 
 0 
 0 
 0 
 0 
 0 
 0 
 0 
 0 
 0 
 0 
 0 
 0 
 0 
 0 
 0 
 0 
 0 
 0 
 0 
 0 
 0 
 0 
 0 
 0 
 0 
 0 
 0 
 0 
 0 
 0 
 0 
 0 
 0 
 0 
 0 
 0 
 0 
 0 
 0 
 0 
 0 
 0 
 0 
 0 
 0 
 0 
 0 
 0 
 0 
 0 
 0 
 0 
 0 
 0 
 0 
 0 
 0 
 0 
 0 
 0 
 0 
 0 
 0 
 0 
 0 
 0 
 0 
 0 
 0 
 0 
 0 
 0 
 0 
 0 
 0 
 0 
 0 
 0 
 0 
 0 
 0 
 0 
 0 
 0 
 0 
 0 
 0 
 0 
 0 
 0 
 0 
 0 
 0 
 0 
 0 
 0 
 0 
 0 
 0 
 0 
 0 
 0 
 0 
 0 
 0 
 0 
 0 
 0 
 0 
 0 
 0 
 0 
 0 
 0 
 0 
 0 
 0 
 0 
 0 
 0 
 0 
 0 
 0 
 0 
 0 
 0 
 0 
 0 
 0 
 0 
 0 
 0 
 0 
 0 
 0 
 0 
 0 
 0 
 0 
 0 
 0 
 0 
 0 
 0 
 0 
 0 
 0 
 0 
 0 
 0 
 0 
 1 
 0 
 0 
 0 
 0 
 0 
 0 
 0 
 0 
 0 
 0 
 0 
 0 
 0 
 0 
 0 
 0 
 0 
 0 
 0 
 0 
 0 
 0 
 0 
 0 
 0 
 0 
 0 
 0 
 0 
 0 
 0 
 0 
 0 
 0 
 0 
 0 
 0 
 0 
 0 
 0 
 0 
 0 
 0 
 0 
 0 
 0 
 0 
 0 
 0 
 0 
 0 
 0 
 0 
 0 
 0 
 0 
 0 
 0 
 0 
 0 
 0 
 0 
 0 
 0 
 0 
 0 
 0 
 0 
 0 
 0 
 0 
 0 
 0 
 0 
 0 
 0 
 0 
 0 
 0 
 0 
 0 
 0 
 0 
 0 
 0 
 0 
 0 
 0 
 0 
 0 
 0 
 0 
 0 
 0 
 0 
 0 
 0 
 0 
 0 
 0 
 0 
 0 
 0 
 0 
 0 
 0 
 0 
 0 
 0 
 0 
 0 
 0 
 0 
 0 
 0 
 0 
 0 
 0 
 0 
 0 
 0 
 0 
 0 
 0 
 0 
 0 
 0 
 0 
 0 
 0 
 0 
 0 
 0 
 0 
 0 
 0 
 0 
 0 
 0 
 0 
 0 
 0 
 0 
 0 
 0 
 0 
 0 
 0 
 0 
 0 
 0 
 0 
 0 
 0 
 0 
 0 
 0 
 0 
 0 
 0 
 0 
 0 
 0 
 0 
 0 
 0 
 0 
 0 
 0 
 0 
 0 
 0 
 0 
 0 
 0 
 0 
 0 
 0 
 0 
 0 
 0 
 0 
 0 
 0 
 0 
 0 
 0 
 0 
 0 
 0 
 0 
 0 
 0 
 0 
 0 
 0 
 0 
 0 
 0 
 0 
 0 
 0 
 0 
 0 
 0 
 0 
 0 
 0 
 0 
 0 
 0 
 0 
 0 
 0 
 0 
 0 
 0 
 0 
 0 
 0 
 0 
 0 
 0 
 0 
 0 
 0 
 0 
 0 
 0 
 0 
 0 
 0 
 0 
 0 
 0 
 0 
 0 
 0 
 0 
 0 
 0 
 0 
 0 
 0 
 0 
 0 
 0 
 0 
 0 
 0 
 0 
 0 
 0 
 0 
 0 
 0 
 0 
 0 
 0 
 0 
 0 
 0 
 0 
 0 
 0 
 0 
 0 
 0 
 0 
 0 
 0 
 0 
 0 
 0 
 0 
 0 
 0 
 0 
 0 
 0 
 0 
 0 
 0 
 0 
 0 
 0 
 0 
 0 
 0 
 0 
 0 
 0 
 0 
 0 
 0 
 0 
 0 
 0 
 0 
 0 
 0 
 0 
 0 
 0 
 0 
 0 
 0 
 0 
 0 
 0 
 0 
 0 
 0 
 0 
 0 
 0 
 0 
 0 
 0 
 0 
 0 
 0 
 0 
 0 
 0 
 0 
 0 
 0 
 0 
 0 
 0 
 0 
 0 
 0 
 0 
 0 
 0 
 0 
 0 
 0 
 0 
 0 
 0 
 0 
 0 
 0 
 0 
 0 
 0 
 0 
 0 
 0 
 0 
 0 
 0 
 0 
 0 
 0 
 0 
 0 
 0 
 0 
 0 
 0 
 0 
 0 
 0 
 0 
 0 
 0 
 0 
 0 
 0 
 0 
 0 
 0 
 0 
 0 
 0 
 0 
 0 
 0 
 0 
 0 
 0 
 0 
 0 
 0 
 0 
 0 
 0 
 0 
 0 
 0 
 0 
 0 
 0 
 0 
 0 
 0 
 0 
 0 
 0 
 0 
 0 
 0 
 0 
 0 
 0 
 0 
 0 
 0 
 0 
 0 
 0 
 0 
 0 
 0 
 0 
 0 
 0 
 0 
 0 
 0 
 0 
 0 
 0 
 0 
 0 
 0 
 0 
 0 
 0 
 0 
 0 
 0 
 0 
 0 
 0 
 0 
 0 
 0 
 0 
 0 
 0 
 0 
 0 
 0 
 0 
 0 
 0 
 0 
 0 
 0 
 0 
 0 
 0 
 0 
 0 
 0 
 0 
 0 
 0 
 0 
 0 
 0 
 0 
 0 
 0 
 0 
 0 
 0 
 0 
 0 
 0 
 0 
 0 
 0 
 0 
 0 
 0 
 0 
 0 
 0 
 0 
 0 
 0 
 0 
 0 
 0 
 0 
 0 
 0 
 0 
 0 
 0 
 0 
 0 
 0 
 0 
 0 
 0 
 0 
 0 
 0 
 0 
 0 
 0 
 0 
 0 
 0 
 0 
 0 
 0 
 0 
 0 
 0 
 0 
 0 
 0 
 0 
 0 
 0 
 0 
 0 
 0 
 0 
 0 
 0 
 0 
 0 
 0 
 0 
 0 
 0 
 0 
 0 
 0 
 0 
 0 
 0 
 0 
 0 
 0 
 0 
 0 
 0 
 0 
 0 
 0 
 0 
 0 
 0 
 0 
 0 
 0 
 0 
 0 
 0 
 0 
 0 
 0 
 0 
 0 
 0 
 0 
 0 
 0 
 0 
 0 
 0 
 0 
 0 
 0 
 0 
 0 
 0 
 0 
 0 
 0 
 0 
 0 
 0 
 0 
 0 
 0 
 0 
 0 
 0 
 0 
 0 
 0 
 0 
 0 
 0 
 0 
 0 
 0 
 0 
 0 
 0 
 0 
 0 
 0 
 0 
 0 
 0 
 0 
 0 
 0 
 0 
 0 
 0 
 0 
 0 
 0 
 0 
 0 
 0 
 0 
 0 
 0 
 0 
 0 
 0 
 0 
 0 
 0 
 0 
 0 
 
 
 14 
 0 
 0 
 0 
 0 
 0 
 0 
 0 
 0 
 0 
 0 
 0 
 0 
 0 
 0 
 3 
 0 
 0 
 0 
 0 
 0 
 0 
 0 
 0 
 0 
 0 
 0 
 0 
 0 
 0 
 0 
 0 
 0 
 0 
 0 
 0 
 0 
 0 
 0 
 0 
 0 
 0 
 0 
 0 
 0 
 0 
 0 
 0 
 0 
 0 
 0 
 0 
 0 
 0 
 0 
 0 
 0 
 0 
 0 
 0 
 0 
 0 
 0 
 0 
 0 
 0 
 0 
 0 
 0 
 0 
 0 
 0 
 0 
 0 
 0 
 0 
 0 
 0 
 0 
 0 
 0 
 0 
 0 
 0 
 0 
 0 
 0 
 0 
 0 
 0 
 0 
 0 
 0 
 0 
 0 
 0 
 0 
 0 
 0 
 0 
 0 
 0 
 0 
 0 
 0 
 0 
 0 
 0 
 0 
 0 
 0 
 0 
 0 
 0 
 0 
 0 
 0 
 0 
 0 
 0 
 0 
 0 
 0 
 0 
 0 
 0 
 0 
 0 
 0 
 0 
 0 
 0 
 0 
 0 
 0 
 0 
 0 
 0 
 0 
 0 
 0 
 0 
 0 
 0 
 0 
 0 
 0 
 0 
 0 
 0 
 0 
 0 
 0 
 0 
 0 
 0 
 0 
 0 
 0 
 0 
 0 
 0 
 0 
 0 
 0 
 0 
 0 
 0 
 0 
 0 
 0 
 0 
 0 
 0 
 0 
 0 
 0 
 0 
 0 
 0 
 0 
 0 
 0 
 0 
 0 
 0 
 0 
 0 
 0 
 0 
 0 
 0 
 0 
 0 
 0 
 0 
 0 
 0 
 0 
 0 
 0 
 0 
 0 
 0 
 0 
 0 
 0 
 0 
 0 
 0 
 0 
 0 
 0 
 0 
 0 
 0 
 0 
 0 
 0 
 0 
 0 
 0 
 0 
 0 
 0 
 0 
 0 
 0 
 0 
 0 
 0 
 0 
 0 
 0 
 0 
 0 
 0 
 0 
 0 
 0 
 0 
 0 
 0 
 0 
 0 
 0 
 0 
 0 
 0 
 0 
 0 
 0 
 0 
 0 
 0 
 0 
 0 
 0 
 0 
 0 
 0 
 0 
 0 
 0 
 0 
 0 
 0 
 0 
 0 
 0 
 0 
 0 
 0 
 0 
 0 
 0 
 0 
 0 
 0 
 0 
 0 
 0 
 0 
 0 
 0 
 0 
 0 
 0 
 0 
 0 
 0 
 0 
 0 
 0 
 0 
 0 
 0 
 0 
 0 
 0 
 0 
 0 
 0 
 0 
 0 
 0 
 0 
 0 
 0 
 0 
 0 
 0 
 0 
 0 
 0 
 0 
 0 
 0 
 0 
 0 
 0 
 0 
 0 
 0 
 0 
 0 
 0 
 0 
 0 
 0 
 0 
 0 
 0 
 0 
 0 
 0 
 0 
 0 
 0 
 0 
 0 
 0 
 0 
 0 
 0 
 0 
 0 
 0 
 0 
 0 
 0 
 0 
 0 
 0 
 0 
 0 
 0 
 0 
 0 
 0 
 0 
 0 
 0 
 0 
 0 
 0 
 0 
 0 
 0 
 0 
 0 
 0 
 0 
 0 
 0 
 0 
 0 
 0 
 0 
 0 
 0 
 0 
 0 
 0 
 0 
 0 
 0 
 0 
 0 
 0 
 0 
 0 
 0 
 0 
 0 
 0 
 0 
 0 
 0 
 0 
 0 
 0 
 0 
 0 
 0 
 0 
 0 
 0 
 0 
 0 
 0 
 0 
 0 
 0 
 0 
 0 
 0 
 0 
 0 
 0 
 0 
 0 
 0 
 0 
 0 
 0 
 0 
 0 
 0 
 0 
 0 
 0 
 0 
 0 
 0 
 0 
 0 
 0 
 0 
 0 
 0 
 0 
 0 
 0 
 0 
 0 
 0 
 0 
 0 
 0 
 0 
 0 
 0 
 0 
 0 
 0 
 0 
 0 
 0 
 0 
 0 
 0 
 0 
 0 
 0 
 0 
 0 
 0 
 0 
 0 
 0 
 0 
 0 
 0 
 0 
 0 
 0 
 0 
 0 
 0 
 0 
 0 
 0 
 0 
 0 
 0 
 0 
 0 
 0 
 0 
 0 
 0 
 0 
 0 
 0 
 0 
 0 
 0 
 0 
 0 
 0 
 0 
 0 
 0 
 0 
 0 
 0 
 0 
 0 
 0 
 0 
 0 
 0 
 0 
 0 
 0 
 0 
 0 
 0 
 0 
 0 
 0 
 0 
 0 
 0 
 0 
 0 
 0 
 0 
 0 
 0 
 0 
 0 
 0 
 0 
 0 
 0 
 0 
 0 
 0 
 0 
 0 
 0 
 0 
 0 
 0 
 0 
 0 
 0 
 0 
 0 
 0 
 0 
 0 
 0 
 0 
 0 
 0 
 0 
 0 
 0 
 0 
 0 
 0 
 0 
 0 
 0 
 0 
 0 
 0 
 0 
 0 
 0 
 0 
 0 
 0 
 0 
 0 
 0 
 0 
 0 
 0 
 0 
 0 
 0 
 0 
 0 
 0 
 0 
 0 
 0 
 0 
 0 
 0 
 0 
 0 
 0 
 0 
 0 
 0 
 0 
 0 
 0 
 0 
 0 
 0 
 0 
 0 
 0 
 0 
 0 
 0 
 0 
 0 
 0 
 0 
 0 
 0 
 0 
 0 
 0 
 0 
 0 
 0 
 0 
 0 
 0 
 0 
 0 
 0 
 0 
 0 
 0 
 0 
 0 
 0 
 0 
 0 
 0 
 0 
 0 
 0 
 0 
 0 
 0 
 0 
 0 
 0 
 0 
 0 
 0 
 0 
 0 
 0 
 0 
 0 
 0 
 0 
 0 
 0 
 0 
 0 
 0 
 0 
 0 
 0 
 0 
 0 
 0 
 0 
 0 
 0 
 0 
 0 
 0 
 0 
 0 
 0 
 0 
 0 
 0 
 0 
 0 
 0 
 0 
 0 
 0 
 0 
 0 
 0 
 0 
 0 
 0 
 0 
 0 
 0 
 0 
 0 
 0 
 0 
 0 
 0 
 0 
 0 
 0 
 0 
 0 
 0 
 0 
 0 
 0 
 0 
 0 
 0 
 0 
 0 
 0 
 0 
 0 
 0 
 0 
 0 
 0 
 0 
 0 
 0 
 0 
 0 
 0 
 0 
 0 
 0 
 0 
 0 
 0 
 0 
 0 
 0 
 0 
 0 
 0 
 0 
 0 
 0 
 0 
 0 
 0 
 0 
 0 
 0 
 0 
 0 
 0 
 0 
 0 
 0 
 0 
 0 
 0 
 0 
 0 
 0 
 0 
 0 
 0 
 0 
 0 
 0 
 0 
 0 
 0 
 0 
 0 
 0 
 0 
 0 
 0 
 0 
 0 
 0 
 0 
 0 
 0 
 0 
 0 
 0 
 0 
 0 
 0 
 0 
 0 
 0 
 0 
 0 
 0 
 0 
 0 
 0 
 0 
 0 
 0 
 0 
 0 
 0 
 0 
 0 
 0 
 0 
 0 
 0 
 0 
 0 
 0 
 0 
 0 
 0 
 0 
 0 
 0 
 0 
 0 
 0 
 0 
 0 
 0 
 0 
 0 
 
 
 15 
 0 
 0 
 0 
 0 
 0 
 0 
 0 
 0 
 0 
 0 
 0 
 0 
 0 
 0 
 0 
 1 
 0 
 0 
 0 
 0 
 0 
 0 
 0 
 0 
 0 
 0 
 0 
 0 
 0 
 0 
 0 
 0 
 0 
 0 
 0 
 0 
 0 
 0 
 0 
 0 
 0 
 0 
 0 
 0 
 0 
 0 
 0 
 0 
 0 
 0 
 0 
 0 
 0 
 0 
 0 
 0 
 0 
 0 
 0 
 0 
 0 
 0 
 0 
 0 
 0 
 0 
 0 
 0 
 0 
 0 
 0 
 0 
 0 
 0 
 0 
 0 
 0 
 0 
 0 
 0 
 0 
 0 
 0 
 0 
 0 
 0 
 0 
 0 
 0 
 0 
 0 
 0 
 0 
 0 
 0 
 0 
 0 
 0 
 0 
 0 
 0 
 0 
 0 
 0 
 0 
 0 
 0 
 0 
 0 
 0 
 0 
 0 
 0 
 0 
 0 
 0 
 0 
 0 
 0 
 0 
 0 
 0 
 0 
 0 
 0 
 0 
 0 
 0 
 0 
 0 
 0 
 0 
 0 
 0 
 0 
 0 
 0 
 0 
 0 
 0 
 0 
 0 
 0 
 0 
 0 
 0 
 0 
 0 
 0 
 0 
 0 
 0 
 0 
 0 
 0 
 0 
 0 
 0 
 0 
 0 
 0 
 0 
 0 
 0 
 0 
 0 
 0 
 0 
 0 
 0 
 0 
 0 
 0 
 0 
 0 
 0 
 0 
 0 
 0 
 0 
 0 
 0 
 0 
 0 
 0 
 0 
 0 
 0 
 0 
 0 
 0 
 0 
 0 
 0 
 0 
 0 
 0 
 0 
 0 
 0 
 0 
 0 
 0 
 0 
 0 
 0 
 0 
 0 
 0 
 0 
 0 
 0 
 0 
 0 
 0 
 0 
 0 
 0 
 0 
 0 
 0 
 0 
 0 
 0 
 0 
 0 
 0 
 0 
 0 
 0 
 0 
 0 
 0 
 0 
 0 
 0 
 0 
 0 
 0 
 0 
 0 
 0 
 0 
 0 
 0 
 0 
 0 
 0 
 0 
 0 
 0 
 0 
 0 
 0 
 0 
 0 
 0 
 0 
 0 
 0 
 0 
 0 
 0 
 0 
 0 
 0 
 0 
 0 
 0 
 0 
 0 
 0 
 0 
 0 
 0 
 0 
 0 
 0 
 0 
 0 
 0 
 0 
 0 
 0 
 0 
 0 
 0 
 0 
 0 
 0 
 0 
 0 
 0 
 0 
 0 
 0 
 0 
 0 
 0 
 0 
 0 
 0 
 0 
 0 
 0 
 0 
 0 
 0 
 0 
 0 
 0 
 0 
 0 
 0 
 0 
 0 
 0 
 0 
 0 
 0 
 0 
 0 
 0 
 0 
 0 
 0 
 0 
 0 
 0 
 0 
 0 
 0 
 0 
 0 
 0 
 0 
 0 
 0 
 0 
 0 
 0 
 0 
 0 
 0 
 0 
 0 
 0 
 0 
 0 
 0 
 0 
 0 
 0 
 0 
 0 
 0 
 0 
 0 
 0 
 0 
 0 
 0 
 0 
 0 
 0 
 0 
 0 
 0 
 0 
 0 
 0 
 0 
 0 
 0 
 0 
 0 
 0 
 0 
 0 
 0 
 0 
 0 
 0 
 0 
 0 
 0 
 0 
 0 
 0 
 0 
 0 
 0 
 0 
 0 
 0 
 0 
 0 
 0 
 0 
 0 
 0 
 0 
 0 
 0 
 0 
 0 
 0 
 0 
 0 
 0 
 0 
 0 
 0 
 0 
 0 
 0 
 0 
 0 
 0 
 0 
 0 
 0 
 0 
 0 
 0 
 0 
 0 
 0 
 0 
 0 
 0 
 0 
 0 
 0 
 0 
 0 
 0 
 0 
 0 
 0 
 0 
 0 
 0 
 0 
 0 
 0 
 0 
 0 
 0 
 0 
 0 
 0 
 0 
 0 
 0 
 0 
 0 
 0 
 0 
 0 
 0 
 0 
 0 
 0 
 0 
 0 
 0 
 0 
 0 
 0 
 0 
 0 
 0 
 0 
 0 
 0 
 0 
 0 
 0 
 0 
 0 
 0 
 0 
 0 
 0 
 0 
 0 
 0 
 0 
 0 
 0 
 0 
 0 
 0 
 0 
 0 
 0 
 0 
 0 
 0 
 0 
 0 
 0 
 0 
 0 
 0 
 0 
 0 
 0 
 0 
 0 
 0 
 0 
 0 
 0 
 0 
 0 
 0 
 0 
 0 
 0 
 0 
 0 
 0 
 0 
 0 
 0 
 0 
 0 
 0 
 0 
 0 
 0 
 0 
 0 
 0 
 0 
 0 
 0 
 0 
 0 
 0 
 0 
 0 
 0 
 0 
 0 
 0 
 0 
 0 
 0 
 0 
 0 
 0 
 0 
 0 
 0 
 0 
 0 
 0 
 0 
 0 
 0 
 0 
 0 
 0 
 0 
 0 
 0 
 0 
 0 
 0 
 0 
 0 
 0 
 0 
 0 
 0 
 0 
 0 
 0 
 0 
 0 
 0 
 0 
 0 
 0 
 0 
 0 
 0 
 0 
 0 
 0 
 0 
 0 
 0 
 0 
 0 
 0 
 0 
 0 
 0 
 0 
 0 
 0 
 0 
 0 
 0 
 0 
 0 
 0 
 0 
 0 
 0 
 0 
 0 
 0 
 0 
 0 
 0 
 0 
 0 
 0 
 0 
 0 
 0 
 0 
 0 
 0 
 0 
 0 
 0 
 0 
 0 
 0 
 0 
 0 
 0 
 0 
 0 
 0 
 0 
 0 
 0 
 0 
 0 
 0 
 0 
 0 
 0 
 0 
 0 
 0 
 0 
 0 
 0 
 0 
 0 
 0 
 0 
 0 
 0 
 0 
 0 
 0 
 0 
 0 
 0 
 0 
 0 
 0 
 0 
 0 
 0 
 0 
 0 
 0 
 0 
 0 
 0 
 0 
 0 
 0 
 0 
 0 
 0 
 0 
 0 
 0 
 0 
 0 
 0 
 0 
 0 
 0 
 0 
 0 
 0 
 0 
 0 
 0 
 0 
 0 
 0 
 0 
 0 
 0 
 0 
 0 
 0 
 0 
 0 
 0 
 0 
 0 
 0 
 0 
 0 
 0 
 0 
 0 
 0 
 0 
 0 
 0 
 0 
 0 
 0 
 0 
 0 
 0 
 0 
 0 
 0 
 0 
 0 
 0 
 0 
 0 
 0 
 0 
 0 
 0 
 0 
 0 
 0 
 0 
 0 
 0 
 0 
 0 
 0 
 0 
 0 
 0 
 0 
 0 
 0 
 0 
 0 
 0 
 0 
 0 
 0 
 0 
 0 
 0 
 0 
 0 
 0 
 0 
 0 
 0 
 0 
 0 
 0 
 0 
 0 
 0 
 0 
 0 
 0 
 0 
 0 
 0 
 0 
 0 
 0 
 0 
 0 
 0 
 0 
 0 
 0 
 0 
 0 
 0 
 0 
 0 
 0 
 0 
 0 
 0 
 0 
 0 
 0 
 0 
 0 
 0 
 0 
 0 
 0 
 0 
 0 
 0 
 0 
 0 
 0 
 0 
 0 
 0 
 0 
 0 
 0 
 0 
 0 
 
 
 16 
 0 
 0 
 0 
 0 
 0 
 0 
 0 
 0 
 0 
 0 
 0 
 0 
 0 
 0 
 0 
 0 
 9 
 0 
 0 
 0 
 0 
 0 
 0 
 0 
 0 
 0 
 0 
 0 
 0 
 0 
 0 
 0 
 0 
 0 
 0 
 0 
 0 
 0 
 0 
 0 
 0 
 0 
 0 
 0 
 0 
 0 
 0 
 0 
 0 
 0 
 0 
 0 
 0 
 0 
 0 
 0 
 0 
 0 
 0 
 0 
 0 
 0 
 0 
 0 
 0 
 0 
 0 
 0 
 0 
 0 
 0 
 0 
 0 
 0 
 0 
 0 
 0 
 0 
 0 
 0 
 0 
 0 
 0 
 0 
 0 
 0 
 0 
 0 
 0 
 0 
 0 
 0 
 0 
 0 
 0 
 0 
 0 
 0 
 0 
 0 
 0 
 0 
 0 
 0 
 0 
 0 
 0 
 0 
 0 
 0 
 0 
 0 
 0 
 0 
 0 
 0 
 0 
 0 
 0 
 0 
 0 
 0 
 0 
 0 
 0 
 0 
 0 
 0 
 0 
 0 
 0 
 0 
 0 
 0 
 0 
 0 
 0 
 0 
 0 
 0 
 0 
 0 
 0 
 0 
 0 
 0 
 0 
 0 
 0 
 0 
 0 
 0 
 0 
 0 
 0 
 0 
 0 
 0 
 0 
 0 
 0 
 0 
 0 
 0 
 0 
 0 
 0 
 0 
 0 
 0 
 0 
 0 
 0 
 0 
 0 
 0 
 0 
 0 
 0 
 0 
 0 
 0 
 0 
 0 
 0 
 0 
 0 
 0 
 0 
 0 
 0 
 0 
 0 
 0 
 0 
 0 
 0 
 0 
 0 
 0 
 0 
 0 
 0 
 0 
 0 
 0 
 0 
 0 
 0 
 0 
 0 
 0 
 0 
 0 
 0 
 0 
 0 
 0 
 0 
 0 
 0 
 0 
 0 
 0 
 0 
 0 
 0 
 0 
 0 
 0 
 0 
 0 
 0 
 0 
 0 
 0 
 0 
 0 
 0 
 0 
 0 
 0 
 0 
 0 
 0 
 0 
 0 
 0 
 0 
 0 
 0 
 0 
 0 
 0 
 0 
 0 
 0 
 0 
 0 
 0 
 0 
 0 
 0 
 0 
 0 
 0 
 0 
 0 
 0 
 0 
 0 
 0 
 0 
 0 
 0 
 0 
 0 
 0 
 0 
 0 
 0 
 0 
 0 
 0 
 0 
 0 
 0 
 0 
 0 
 0 
 0 
 0 
 0 
 0 
 0 
 0 
 0 
 0 
 0 
 0 
 0 
 0 
 0 
 0 
 0 
 0 
 0 
 0 
 0 
 0 
 0 
 0 
 0 
 0 
 0 
 0 
 0 
 0 
 0 
 0 
 0 
 0 
 0 
 0 
 0 
 0 
 0 
 0 
 0 
 0 
 0 
 0 
 0 
 0 
 0 
 0 
 0 
 0 
 0 
 0 
 0 
 0 
 0 
 0 
 0 
 0 
 0 
 0 
 0 
 0 
 0 
 0 
 0 
 0 
 0 
 0 
 0 
 0 
 0 
 0 
 0 
 0 
 0 
 0 
 0 
 0 
 0 
 0 
 0 
 0 
 0 
 0 
 0 
 0 
 0 
 0 
 0 
 0 
 0 
 0 
 0 
 0 
 0 
 0 
 0 
 0 
 0 
 0 
 0 
 0 
 0 
 0 
 0 
 0 
 0 
 0 
 0 
 0 
 0 
 0 
 0 
 0 
 0 
 0 
 0 
 0 
 0 
 0 
 0 
 0 
 0 
 0 
 0 
 0 
 0 
 0 
 0 
 0 
 0 
 0 
 0 
 0 
 0 
 0 
 0 
 0 
 0 
 0 
 0 
 0 
 0 
 0 
 0 
 0 
 0 
 0 
 0 
 0 
 0 
 0 
 0 
 0 
 0 
 0 
 0 
 0 
 0 
 0 
 0 
 0 
 0 
 0 
 0 
 0 
 0 
 0 
 0 
 0 
 0 
 0 
 0 
 0 
 0 
 0 
 0 
 0 
 0 
 0 
 0 
 0 
 0 
 0 
 0 
 0 
 0 
 0 
 0 
 0 
 0 
 0 
 0 
 0 
 0 
 0 
 0 
 0 
 0 
 0 
 0 
 0 
 0 
 0 
 0 
 0 
 0 
 0 
 0 
 0 
 0 
 0 
 0 
 0 
 0 
 0 
 0 
 0 
 0 
 0 
 0 
 0 
 0 
 0 
 0 
 0 
 0 
 0 
 0 
 0 
 0 
 0 
 0 
 0 
 0 
 0 
 0 
 0 
 0 
 0 
 0 
 0 
 0 
 0 
 0 
 0 
 0 
 0 
 0 
 0 
 0 
 0 
 0 
 0 
 0 
 0 
 0 
 0 
 0 
 0 
 0 
 0 
 0 
 0 
 0 
 0 
 0 
 0 
 0 
 0 
 0 
 0 
 0 
 0 
 0 
 0 
 0 
 0 
 0 
 0 
 0 
 0 
 0 
 0 
 0 
 0 
 0 
 0 
 0 
 0 
 0 
 0 
 0 
 0 
 0 
 0 
 0 
 0 
 0 
 0 
 0 
 0 
 0 
 0 
 0 
 0 
 0 
 0 
 0 
 0 
 0 
 0 
 0 
 0 
 0 
 0 
 0 
 0 
 0 
 0 
 0 
 0 
 0 
 0 
 0 
 0 
 0 
 0 
 0 
 0 
 0 
 0 
 0 
 0 
 0 
 0 
 0 
 0 
 0 
 0 
 0 
 0 
 0 
 0 
 0 
 0 
 0 
 0 
 0 
 0 
 0 
 0 
 0 
 0 
 0 
 0 
 0 
 0 
 0 
 0 
 0 
 0 
 0 
 0 
 0 
 0 
 0 
 0 
 0 
 0 
 0 
 0 
 0 
 0 
 0 
 0 
 0 
 0 
 0 
 0 
 0 
 0 
 0 
 0 
 0 
 0 
 0 
 0 
 0 
 0 
 0 
 0 
 0 
 0 
 0 
 0 
 0 
 0 
 0 
 0 
 0 
 0 
 0 
 0 
 0 
 0 
 0 
 0 
 0 
 0 
 0 
 0 
 0 
 0 
 0 
 0 
 0 
 0 
 0 
 0 
 0 
 0 
 0 
 0 
 0 
 0 
 0 
 0 
 0 
 0 
 0 
 0 
 0 
 0 
 0 
 0 
 0 
 0 
 0 
 0 
 0 
 0 
 0 
 0 
 0 
 0 
 0 
 0 
 0 
 0 
 0 
 0 
 0 
 0 
 0 
 0 
 0 
 0 
 0 
 0 
 0 
 0 
 0 
 0 
 0 
 0 
 0 
 0 
 0 
 0 
 0 
 0 
 0 
 0 
 0 
 0 
 0 
 0 
 0 
 0 
 0 
 0 
 0 
 0 
 0 
 0 
 0 
 0 
 0 
 0 
 0 
 0 
 0 
 0 
 0 
 0 
 0 
 0 
 0 
 0 
 0 
 0 
 0 
 0 
 0 
 0 
 0 
 0 
 0 
 0 
 0 
 0 
 0 
 0 
 0 
 0 
 0 
 0 
 0 
 0 
 0 
 0 
 0 
 0 
 0 
 0 
 0 
 0 
 0 
 0 
 0 
 0 
 0 
 0 
 0 
 0 
 0 
 0 
 
 
 17 
 0 
 0 
 0 
 0 
 0 
 0 
 0 
 0 
 0 
 0 
 0 
 0 
 0 
 0 
 0 
 0 
 0 
 3 
 0 
 0 
 0 
 0 
 0 
 0 
 0 
 0 
 0 
 0 
 0 
 0 
 0 
 0 
 0 
 0 
 0 
 0 
 0 
 0 
 0 
 0 
 0 
 0 
 0 
 0 
 0 
 0 
 0 
 0 
 0 
 0 
 0 
 0 
 0 
 0 
 0 
 0 
 0 
 0 
 0 
 0 
 0 
 0 
 0 
 0 
 0 
 0 
 0 
 0 
 0 
 0 
 0 
 0 
 0 
 0 
 0 
 0 
 0 
 0 
 0 
 0 
 0 
 0 
 0 
 0 
 0 
 0 
 0 
 0 
 0 
 0 
 0 
 0 
 0 
 0 
 0 
 0 
 0 
 0 
 0 
 0 
 0 
 0 
 0 
 0 
 0 
 0 
 0 
 0 
 0 
 0 
 0 
 0 
 0 
 0 
 0 
 0 
 0 
 0 
 0 
 0 
 0 
 0 
 0 
 0 
 0 
 0 
 0 
 0 
 0 
 0 
 0 
 0 
 0 
 0 
 0 
 0 
 0 
 0 
 0 
 0 
 0 
 0 
 0 
 0 
 0 
 0 
 0 
 0 
 0 
 0 
 0 
 0 
 0 
 0 
 0 
 0 
 0 
 0 
 0 
 0 
 0 
 0 
 0 
 0 
 0 
 0 
 0 
 0 
 0 
 0 
 0 
 0 
 0 
 0 
 0 
 0 
 0 
 0 
 0 
 0 
 0 
 0 
 0 
 0 
 0 
 0 
 0 
 0 
 0 
 0 
 0 
 0 
 0 
 0 
 0 
 0 
 0 
 0 
 0 
 0 
 0 
 0 
 0 
 0 
 0 
 0 
 0 
 0 
 0 
 0 
 0 
 0 
 0 
 0 
 0 
 0 
 0 
 0 
 0 
 0 
 0 
 0 
 0 
 0 
 0 
 0 
 0 
 0 
 0 
 0 
 0 
 0 
 0 
 0 
 0 
 0 
 0 
 0 
 0 
 0 
 0 
 0 
 0 
 0 
 0 
 0 
 0 
 0 
 0 
 0 
 0 
 0 
 0 
 0 
 0 
 0 
 0 
 0 
 0 
 0 
 0 
 0 
 0 
 0 
 0 
 0 
 0 
 0 
 0 
 0 
 0 
 0 
 0 
 0 
 0 
 0 
 0 
 0 
 0 
 0 
 0 
 0 
 0 
 0 
 0 
 0 
 0 
 0 
 0 
 0 
 0 
 0 
 0 
 0 
 0 
 0 
 0 
 0 
 0 
 0 
 0 
 0 
 0 
 0 
 0 
 0 
 0 
 0 
 0 
 0 
 0 
 0 
 0 
 0 
 0 
 0 
 0 
 0 
 0 
 0 
 0 
 0 
 0 
 0 
 0 
 0 
 0 
 0 
 0 
 0 
 0 
 0 
 0 
 0 
 0 
 0 
 0 
 0 
 0 
 0 
 0 
 0 
 0 
 0 
 0 
 0 
 0 
 0 
 0 
 0 
 0 
 0 
 0 
 0 
 0 
 0 
 0 
 0 
 0 
 0 
 0 
 0 
 0 
 0 
 0 
 0 
 0 
 0 
 0 
 0 
 0 
 0 
 0 
 0 
 0 
 0 
 0 
 0 
 0 
 0 
 0 
 0 
 0 
 0 
 0 
 0 
 0 
 0 
 0 
 0 
 0 
 0 
 0 
 0 
 0 
 0 
 0 
 0 
 0 
 0 
 0 
 0 
 0 
 0 
 0 
 0 
 0 
 0 
 0 
 0 
 0 
 0 
 0 
 0 
 0 
 0 
 0 
 0 
 0 
 0 
 0 
 0 
 0 
 0 
 0 
 0 
 0 
 0 
 0 
 0 
 0 
 0 
 0 
 0 
 0 
 0 
 0 
 0 
 0 
 0 
 0 
 0 
 0 
 0 
 0 
 0 
 0 
 0 
 0 
 0 
 0 
 0 
 0 
 0 
 0 
 0 
 0 
 0 
 0 
 0 
 0 
 0 
 0 
 0 
 0 
 0 
 0 
 0 
 0 
 0 
 0 
 0 
 0 
 0 
 0 
 0 
 0 
 0 
 0 
 0 
 0 
 0 
 0 
 0 
 0 
 0 
 0 
 0 
 0 
 0 
 0 
 0 
 0 
 0 
 0 
 0 
 0 
 0 
 0 
 0 
 0 
 0 
 0 
 0 
 0 
 0 
 0 
 0 
 0 
 0 
 0 
 0 
 0 
 0 
 0 
 0 
 0 
 0 
 0 
 0 
 0 
 0 
 0 
 0 
 0 
 0 
 0 
 0 
 0 
 0 
 0 
 0 
 0 
 0 
 0 
 0 
 0 
 0 
 0 
 0 
 0 
 0 
 0 
 0 
 0 
 0 
 0 
 0 
 0 
 0 
 0 
 0 
 0 
 0 
 0 
 0 
 0 
 0 
 0 
 0 
 0 
 0 
 0 
 0 
 0 
 0 
 0 
 0 
 0 
 0 
 0 
 0 
 0 
 0 
 0 
 0 
 0 
 0 
 0 
 0 
 0 
 0 
 0 
 0 
 0 
 0 
 0 
 0 
 0 
 0 
 0 
 0 
 0 
 0 
 0 
 0 
 0 
 0 
 0 
 0 
 0 
 0 
 0 
 0 
 0 
 0 
 0 
 0 
 0 
 0 
 0 
 0 
 0 
 0 
 0 
 0 
 0 
 0 
 0 
 0 
 0 
 0 
 0 
 0 
 0 
 0 
 0 
 0 
 0 
 0 
 0 
 0 
 0 
 0 
 0 
 0 
 0 
 0 
 0 
 0 
 0 
 0 
 0 
 0 
 0 
 0 
 0 
 0 
 0 
 0 
 0 
 0 
 0 
 0 
 0 
 0 
 0 
 0 
 0 
 0 
 0 
 0 
 0 
 0 
 0 
 0 
 0 
 0 
 0 
 0 
 0 
 0 
 0 
 0 
 0 
 0 
 0 
 0 
 0 
 0 
 0 
 0 
 0 
 0 
 0 
 0 
 0 
 0 
 0 
 0 
 0 
 0 
 0 
 0 
 0 
 0 
 0 
 0 
 0 
 0 
 0 
 0 
 0 
 0 
 0 
 0 
 0 
 0 
 0 
 0 
 0 
 0 
 0 
 0 
 0 
 0 
 0 
 0 
 0 
 0 
 0 
 0 
 0 
 0 
 0 
 0 
 0 
 0 
 0 
 0 
 0 
 0 
 0 
 0 
 0 
 0 
 0 
 0 
 0 
 0 
 0 
 0 
 0 
 0 
 0 
 0 
 0 
 0 
 0 
 0 
 0 
 0 
 0 
 0 
 0 
 0 
 0 
 0 
 0 
 0 
 0 
 0 
 0 
 0 
 0 
 0 
 0 
 0 
 0 
 0 
 0 
 0 
 0 
 0 
 0 
 0 
 0 
 0 
 0 
 0 
 0 
 0 
 0 
 0 
 0 
 0 
 0 
 0 
 0 
 0 
 0 
 0 
 0 
 0 
 0 
 0 
 0 
 0 
 0 
 0 
 0 
 0 
 0 
 0 
 0 
 0 
 0 
 0 
 0 
 0 
 0 
 0 
 0 
 0 
 0 
 0 
 0 
 0 
 0 
 0 
 0 
 0 
 0 
 0 
 0 
 0 
 
 
 18 
 0 
 0 
 0 
 0 
 0 
 0 
 0 
 0 
 0 
 0 
 0 
 0 
 0 
 0 
 0 
 0 
 0 
 0 
 15 
 0 
 0 
 0 
 0 
 0 
 0 
 0 
 0 
 0 
 0 
 0 
 0 
 0 
 0 
 0 
 0 
 0 
 0 
 0 
 0 
 0 
 0 
 0 
 0 
 0 
 0 
 0 
 0 
 0 
 0 
 0 
 0 
 0 
 0 
 0 
 0 
 0 
 0 
 0 
 0 
 0 
 0 
 0 
 0 
 0 
 0 
 0 
 0 
 0 
 0 
 0 
 0 
 0 
 0 
 0 
 0 
 0 
 0 
 0 
 0 
 0 
 0 
 0 
 0 
 0 
 0 
 0 
 0 
 0 
 0 
 0 
 0 
 0 
 0 
 0 
 0 
 0 
 0 
 0 
 0 
 0 
 0 
 0 
 0 
 0 
 0 
 0 
 0 
 0 
 0 
 0 
 0 
 0 
 0 
 0 
 0 
 0 
 0 
 0 
 0 
 0 
 0 
 0 
 0 
 0 
 0 
 0 
 0 
 0 
 0 
 0 
 0 
 0 
 0 
 0 
 0 
 0 
 0 
 0 
 0 
 0 
 0 
 0 
 0 
 0 
 0 
 0 
 0 
 0 
 0 
 0 
 0 
 0 
 0 
 0 
 0 
 0 
 0 
 0 
 0 
 0 
 0 
 0 
 0 
 0 
 0 
 0 
 0 
 0 
 0 
 0 
 0 
 0 
 0 
 0 
 0 
 0 
 0 
 0 
 0 
 0 
 0 
 0 
 0 
 0 
 0 
 0 
 0 
 0 
 0 
 0 
 0 
 0 
 0 
 0 
 0 
 0 
 0 
 0 
 0 
 0 
 0 
 0 
 0 
 0 
 0 
 0 
 0 
 0 
 0 
 0 
 0 
 0 
 0 
 0 
 0 
 0 
 0 
 0 
 0 
 0 
 0 
 1 
 0 
 0 
 0 
 0 
 0 
 0 
 0 
 0 
 0 
 0 
 0 
 0 
 0 
 0 
 0 
 0 
 0 
 0 
 0 
 0 
 0 
 0 
 0 
 0 
 0 
 0 
 0 
 0 
 0 
 0 
 0 
 0 
 0 
 0 
 0 
 0 
 0 
 0 
 0 
 0 
 0 
 0 
 0 
 0 
 0 
 0 
 0 
 0 
 0 
 0 
 0 
 0 
 0 
 0 
 0 
 0 
 0 
 0 
 0 
 0 
 0 
 0 
 0 
 0 
 0 
 0 
 0 
 0 
 0 
 0 
 0 
 0 
 0 
 0 
 0 
 0 
 0 
 0 
 0 
 0 
 0 
 0 
 0 
 0 
 0 
 0 
 0 
 0 
 0 
 0 
 0 
 0 
 0 
 0 
 0 
 0 
 0 
 0 
 0 
 0 
 0 
 0 
 0 
 0 
 0 
 0 
 0 
 0 
 0 
 0 
 0 
 0 
 0 
 0 
 0 
 0 
 0 
 0 
 0 
 0 
 0 
 0 
 0 
 0 
 0 
 0 
 0 
 0 
 0 
 0 
 0 
 0 
 0 
 0 
 0 
 0 
 0 
 0 
 0 
 0 
 0 
 0 
 0 
 0 
 0 
 0 
 0 
 0 
 0 
 0 
 0 
 0 
 0 
 0 
 0 
 0 
 0 
 0 
 0 
 0 
 0 
 0 
 0 
 0 
 0 
 0 
 0 
 0 
 0 
 0 
 0 
 0 
 0 
 0 
 0 
 0 
 0 
 0 
 0 
 0 
 0 
 0 
 0 
 0 
 0 
 0 
 0 
 0 
 0 
 0 
 0 
 0 
 0 
 0 
 0 
 0 
 0 
 0 
 0 
 0 
 0 
 0 
 0 
 0 
 0 
 0 
 0 
 0 
 0 
 0 
 0 
 0 
 0 
 0 
 0 
 0 
 0 
 0 
 0 
 0 
 0 
 0 
 0 
 0 
 0 
 0 
 0 
 0 
 0 
 0 
 0 
 0 
 0 
 0 
 0 
 0 
 0 
 0 
 0 
 0 
 0 
 0 
 0 
 0 
 0 
 0 
 0 
 0 
 0 
 0 
 0 
 0 
 0 
 0 
 0 
 0 
 0 
 0 
 0 
 0 
 0 
 0 
 0 
 0 
 0 
 0 
 0 
 0 
 0 
 0 
 0 
 0 
 0 
 0 
 0 
 0 
 0 
 0 
 0 
 0 
 0 
 0 
 0 
 0 
 0 
 0 
 0 
 0 
 0 
 0 
 0 
 0 
 0 
 0 
 0 
 0 
 0 
 0 
 0 
 0 
 0 
 0 
 0 
 0 
 0 
 0 
 0 
 0 
 0 
 0 
 0 
 0 
 0 
 0 
 0 
 0 
 0 
 0 
 0 
 0 
 0 
 0 
 0 
 0 
 0 
 0 
 0 
 0 
 0 
 0 
 0 
 0 
 0 
 0 
 0 
 0 
 0 
 0 
 0 
 0 
 0 
 0 
 0 
 0 
 0 
 0 
 0 
 0 
 0 
 0 
 0 
 0 
 0 
 0 
 0 
 0 
 0 
 0 
 0 
 0 
 0 
 0 
 0 
 0 
 0 
 0 
 0 
 0 
 0 
 0 
 0 
 0 
 0 
 0 
 0 
 0 
 0 
 0 
 0 
 0 
 0 
 0 
 0 
 0 
 0 
 0 
 0 
 0 
 0 
 0 
 0 
 0 
 0 
 0 
 0 
 0 
 0 
 0 
 0 
 0 
 0 
 0 
 0 
 0 
 0 
 0 
 0 
 0 
 0 
 0 
 0 
 0 
 0 
 0 
 0 
 0 
 0 
 0 
 0 
 0 
 0 
 0 
 0 
 0 
 0 
 0 
 0 
 0 
 0 
 0 
 0 
 0 
 0 
 0 
 0 
 0 
 0 
 0 
 0 
 0 
 0 
 0 
 0 
 0 
 0 
 0 
 0 
 0 
 0 
 0 
 0 
 0 
 0 
 0 
 0 
 0 
 0 
 0 
 0 
 0 
 0 
 0 
 0 
 0 
 0 
 0 
 0 
 0 
 0 
 0 
 0 
 0 
 0 
 0 
 0 
 0 
 0 
 0 
 0 
 0 
 0 
 0 
 0 
 0 
 0 
 0 
 0 
 0 
 0 
 0 
 0 
 0 
 0 
 0 
 0 
 0 
 0 
 0 
 0 
 0 
 0 
 0 
 0 
 0 
 0 
 0 
 0 
 0 
 0 
 0 
 0 
 0 
 0 
 0 
 0 
 0 
 0 
 0 
 0 
 0 
 0 
 0 
 0 
 0 
 0 
 0 
 0 
 0 
 0 
 0 
 0 
 0 
 0 
 0 
 0 
 0 
 0 
 0 
 0 
 0 
 0 
 0 
 0 
 0 
 0 
 0 
 0 
 0 
 0 
 0 
 0 
 0 
 0 
 0 
 0 
 0 
 0 
 0 
 0 
 0 
 0 
 0 
 0 
 0 
 0 
 0 
 0 
 0 
 0 
 0 
 0 
 0 
 0 
 0 
 0 
 0 
 0 
 0 
 0 
 0 
 0 
 0 
 0 
 0 
 0 
 0 
 0 
 0 
 0 
 0 
 0 
 0 
 0 
 0 
 0 
 0 
 0 
 0 
 0 
 0 
 0 
 0 
 0 
 0 
 
 
 19 
 0 
 0 
 0 
 0 
 0 
 0 
 0 
 0 
 0 
 0 
 0 
 0 
 0 
 0 
 0 
 0 
 0 
 0 
 0 
 0 
 0 
 0 
 0 
 0 
 0 
 0 
 0 
 0 
 0 
 0 
 0 
 0 
 0 
 0 
 0 
 0 
 0 
 0 
 0 
 0 
 0 
 0 
 0 
 0 
 0 
 0 
 0 
 0 
 0 
 0 
 0 
 0 
 0 
 0 
 0 
 0 
 0 
 0 
 0 
 0 
 0 
 0 
 0 
 0 
 0 
 0 
 0 
 0 
 0 
 0 
 0 
 0 
 0 
 0 
 0 
 0 
 0 
 0 
 0 
 0 
 0 
 0 
 0 
 0 
 0 
 0 
 0 
 0 
 0 
 0 
 0 
 0 
 0 
 0 
 0 
 0 
 0 
 0 
 0 
 0 
 0 
 0 
 0 
 0 
 0 
 0 
 0 
 0 
 0 
 0 
 0 
 0 
 0 
 0 
 0 
 0 
 0 
 0 
 0 
 0 
 0 
 0 
 0 
 0 
 0 
 0 
 0 
 0 
 0 
 0 
 0 
 0 
 0 
 0 
 0 
 0 
 0 
 0 
 0 
 0 
 0 
 0 
 0 
 0 
 0 
 0 
 0 
 0 
 0 
 0 
 0 
 0 
 0 
 0 
 0 
 0 
 0 
 0 
 0 
 0 
 0 
 0 
 0 
 0 
 0 
 0 
 0 
 0 
 0 
 0 
 0 
 0 
 0 
 0 
 0 
 0 
 0 
 0 
 0 
 0 
 0 
 0 
 0 
 0 
 0 
 0 
 0 
 0 
 0 
 0 
 0 
 0 
 0 
 0 
 0 
 0 
 0 
 0 
 0 
 0 
 0 
 0 
 0 
 0 
 0 
 0 
 0 
 0 
 0 
 0 
 0 
 0 
 0 
 0 
 0 
 0 
 0 
 0 
 0 
 0 
 0 
 0 
 0 
 0 
 0 
 0 
 0 
 0 
 0 
 0 
 0 
 0 
 0 
 0 
 0 
 0 
 0 
 0 
 0 
 0 
 0 
 0 
 0 
 0 
 0 
 0 
 0 
 0 
 0 
 0 
 0 
 0 
 0 
 0 
 0 
 0 
 0 
 0 
 0 
 0 
 0 
 0 
 0 
 0 
 0 
 0 
 0 
 0 
 0 
 0 
 0 
 0 
 0 
 0 
 0 
 0 
 0 
 0 
 0 
 0 
 0 
 0 
 0 
 0 
 0 
 0 
 0 
 0 
 0 
 0 
 0 
 0 
 0 
 0 
 0 
 0 
 0 
 0 
 0 
 0 
 0 
 0 
 0 
 0 
 0 
 0 
 0 
 0 
 0 
 0 
 0 
 0 
 0 
 0 
 0 
 0 
 0 
 0 
 0 
 0 
 0 
 0 
 0 
 0 
 0 
 0 
 0 
 0 
 0 
 0 
 0 
 0 
 0 
 0 
 0 
 0 
 0 
 0 
 0 
 0 
 0 
 0 
 0 
 0 
 0 
 0 
 0 
 0 
 0 
 0 
 0 
 0 
 0 
 0 
 0 
 0 
 0 
 0 
 0 
 0 
 0 
 0 
 0 
 0 
 0 
 0 
 0 
 0 
 0 
 0 
 0 
 0 
 0 
 0 
 0 
 0 
 0 
 0 
 0 
 0 
 0 
 0 
 0 
 0 
 0 
 0 
 0 
 0 
 0 
 0 
 0 
 0 
 0 
 0 
 0 
 0 
 0 
 0 
 0 
 0 
 0 
 0 
 0 
 0 
 0 
 0 
 0 
 0 
 0 
 0 
 0 
 0 
 0 
 0 
 0 
 0 
 0 
 0 
 0 
 0 
 0 
 0 
 0 
 0 
 0 
 0 
 0 
 0 
 0 
 0 
 0 
 0 
 0 
 0 
 0 
 0 
 0 
 0 
 0 
 0 
 0 
 0 
 0 
 0 
 0 
 0 
 0 
 0 
 0 
 0 
 0 
 0 
 0 
 0 
 0 
 0 
 0 
 0 
 0 
 0 
 0 
 0 
 0 
 0 
 0 
 0 
 0 
 0 
 0 
 0 
 0 
 0 
 0 
 0 
 0 
 0 
 0 
 0 
 0 
 0 
 0 
 0 
 0 
 0 
 0 
 0 
 0 
 0 
 0 
 0 
 0 
 0 
 0 
 2 
 0 
 0 
 0 
 0 
 0 
 0 
 0 
 0 
 0 
 0 
 0 
 0 
 0 
 0 
 0 
 0 
 0 
 0 
 0 
 0 
 0 
 0 
 0 
 0 
 0 
 0 
 0 
 0 
 0 
 0 
 0 
 0 
 0 
 0 
 0 
 0 
 0 
 0 
 0 
 0 
 0 
 0 
 0 
 0 
 0 
 0 
 0 
 0 
 0 
 0 
 0 
 0 
 0 
 0 
 0 
 0 
 0 
 0 
 0 
 0 
 0 
 0 
 0 
 0 
 0 
 0 
 0 
 0 
 0 
 0 
 0 
 0 
 0 
 0 
 0 
 0 
 0 
 0 
 0 
 0 
 0 
 0 
 0 
 0 
 0 
 0 
 0 
 0 
 0 
 0 
 0 
 0 
 0 
 0 
 0 
 0 
 0 
 0 
 0 
 0 
 0 
 0 
 0 
 0 
 0 
 0 
 0 
 0 
 0 
 0 
 0 
 0 
 0 
 0 
 0 
 0 
 0 
 0 
 0 
 0 
 0 
 0 
 0 
 0 
 0 
 0 
 0 
 0 
 0 
 0 
 0 
 0 
 0 
 0 
 0 
 0 
 0 
 0 
 0 
 0 
 0 
 0 
 0 
 0 
 0 
 0 
 0 
 0 
 0 
 0 
 0 
 0 
 0 
 0 
 0 
 0 
 0 
 0 
 0 
 0 
 0 
 0 
 0 
 0 
 0 
 0 
 0 
 0 
 0 
 0 
 0 
 0 
 0 
 0 
 0 
 0 
 0 
 0 
 0 
 0 
 0 
 0 
 0 
 0 
 0 
 0 
 0 
 0 
 0 
 0 
 0 
 0 
 0 
 0 
 0 
 0 
 0 
 0 
 0 
 0 
 0 
 0 
 0 
 0 
 0 
 0 
 0 
 0 
 0 
 0 
 0 
 0 
 0 
 0 
 0 
 0 
 0 
 0 
 0 
 0 
 0 
 0 
 0 
 0 
 0 
 0 
 0 
 0 
 0 
 0 
 0 
 0 
 0 
 0 
 0 
 0 
 0 
 0 
 0 
 0 
 0 
 0 
 0 
 0 
 0 
 0 
 0 
 0 
 0 
 0 
 0 
 0 
 0 
 0 
 0 
 0 
 0 
 0 
 0 
 0 
 0 
 0 
 0 
 0 
 0 
 0 
 0 
 0 
 0 
 0 
 0 
 0 
 0 
 0 
 0 
 0 
 0 
 0 
 0 
 0 
 0 
 0 
 0 
 0 
 0 
 0 
 0 
 0 
 0 
 0 
 0 
 0 
 0 
 0 
 0 
 0 
 0 
 0 
 0 
 0 
 0 
 0 
 0 
 0 
 0 
 0 
 0 
 0 
 0 
 0 
 0 
 0 
 0 
 0 
 0 
 0 
 0 
 0 
 0 
 0 
 0 
 0 
 0 
 0 
 0 
 0 
 0 
 0 
 0 
 0 
 0 
 0 
 
 
 20 
 0 
 0 
 0 
 0 
 0 
 0 
 0 
 0 
 0 
 0 
 0 
 0 
 0 
 0 
 0 
 0 
 0 
 0 
 0 
 0 
 2 
 0 
 0 
 0 
 0 
 4 
 0 
 0 
 0 
 0 
 0 
 0 
 0 
 0 
 0 
 0 
 0 
 0 
 0 
 0 
 0 
 0 
 0 
 0 
 0 
 0 
 0 
 0 
 0 
 0 
 0 
 0 
 0 
 0 
 0 
 0 
 0 
 0 
 0 
 0 
 0 
 0 
 0 
 0 
 0 
 0 
 0 
 0 
 0 
 0 
 0 
 0 
 0 
 0 
 0 
 0 
 0 
 0 
 0 
 0 
 0 
 0 
 0 
 0 
 0 
 0 
 0 
 0 
 0 
 0 
 0 
 0 
 0 
 0 
 0 
 0 
 0 
 0 
 0 
 0 
 0 
 0 
 0 
 0 
 0 
 0 
 0 
 0 
 0 
 0 
 0 
 0 
 0 
 0 
 0 
 0 
 0 
 0 
 0 
 0 
 0 
 0 
 0 
 0 
 0 
 0 
 0 
 0 
 0 
 0 
 0 
 0 
 0 
 0 
 0 
 0 
 0 
 0 
 0 
 0 
 0 
 0 
 0 
 0 
 0 
 0 
 0 
 0 
 0 
 0 
 0 
 0 
 0 
 0 
 0 
 0 
 0 
 0 
 0 
 0 
 0 
 0 
 0 
 0 
 0 
 0 
 0 
 0 
 0 
 0 
 0 
 0 
 0 
 0 
 0 
 0 
 0 
 0 
 0 
 0 
 0 
 0 
 0 
 0 
 0 
 0 
 0 
 0 
 0 
 0 
 0 
 0 
 0 
 0 
 0 
 0 
 0 
 0 
 0 
 0 
 0 
 0 
 0 
 0 
 0 
 0 
 0 
 0 
 0 
 0 
 0 
 0 
 0 
 0 
 0 
 0 
 0 
 0 
 0 
 0 
 0 
 0 
 0 
 0 
 0 
 0 
 0 
 0 
 0 
 0 
 0 
 0 
 6 
 0 
 0 
 0 
 0 
 0 
 0 
 0 
 0 
 0 
 0 
 0 
 0 
 0 
 0 
 0 
 0 
 0 
 0 
 0 
 0 
 0 
 0 
 0 
 0 
 0 
 0 
 0 
 0 
 0 
 0 
 0 
 0 
 0 
 0 
 0 
 0 
 0 
 0 
 0 
 0 
 0 
 0 
 0 
 0 
 0 
 0 
 0 
 0 
 0 
 0 
 0 
 0 
 0 
 0 
 0 
 0 
 0 
 0 
 0 
 0 
 0 
 0 
 0 
 0 
 0 
 0 
 0 
 0 
 0 
 0 
 0 
 0 
 0 
 0 
 0 
 0 
 0 
 0 
 0 
 0 
 0 
 0 
 0 
 0 
 0 
 0 
 0 
 0 
 0 
 0 
 0 
 0 
 0 
 0 
 0 
 0 
 0 
 0 
 0 
 0 
 0 
 0 
 0 
 0 
 0 
 0 
 0 
 0 
 0 
 0 
 0 
 0 
 0 
 0 
 0 
 0 
 0 
 0 
 0 
 0 
 0 
 0 
 0 
 0 
 0 
 0 
 0 
 0 
 0 
 0 
 0 
 0 
 0 
 0 
 0 
 0 
 0 
 0 
 0 
 0 
 0 
 0 
 0 
 0 
 0 
 0 
 0 
 0 
 0 
 0 
 0 
 0 
 0 
 0 
 0 
 0 
 0 
 0 
 0 
 0 
 0 
 0 
 0 
 0 
 0 
 0 
 0 
 0 
 0 
 0 
 0 
 0 
 0 
 0 
 0 
 0 
 0 
 0 
 0 
 0 
 0 
 0 
 0 
 0 
 0 
 0 
 0 
 0 
 0 
 0 
 0 
 0 
 0 
 0 
 0 
 0 
 0 
 0 
 0 
 0 
 0 
 0 
 0 
 0 
 0 
 0 
 0 
 0 
 0 
 0 
 0 
 0 
 0 
 0 
 0 
 0 
 0 
 0 
 0 
 0 
 0 
 0 
 0 
 0 
 0 
 0 
 0 
 0 
 0 
 0 
 0 
 0 
 0 
 0 
 0 
 0 
 0 
 0 
 0 
 0 
 0 
 0 
 0 
 0 
 0 
 0 
 0 
 0 
 0 
 0 
 0 
 0 
 0 
 0 
 0 
 0 
 0 
 0 
 0 
 0 
 0 
 0 
 0 
 0 
 0 
 0 
 0 
 0 
 0 
 0 
 0 
 0 
 0 
 0 
 0 
 0 
 0 
 0 
 0 
 0 
 0 
 0 
 0 
 0 
 0 
 0 
 0 
 0 
 0 
 0 
 0 
 0 
 0 
 0 
 0 
 0 
 0 
 0 
 0 
 0 
 0 
 0 
 0 
 0 
 0 
 0 
 0 
 0 
 0 
 0 
 0 
 0 
 0 
 0 
 0 
 0 
 0 
 0 
 0 
 0 
 0 
 0 
 0 
 0 
 0 
 0 
 0 
 0 
 0 
 0 
 0 
 0 
 0 
 0 
 0 
 0 
 0 
 0 
 0 
 0 
 0 
 0 
 0 
 0 
 0 
 0 
 0 
 0 
 0 
 0 
 0 
 0 
 0 
 0 
 0 
 0 
 0 
 0 
 0 
 0 
 0 
 0 
 0 
 0 
 0 
 0 
 0 
 0 
 0 
 0 
 0 
 0 
 0 
 0 
 0 
 0 
 0 
 0 
 0 
 0 
 0 
 0 
 0 
 0 
 0 
 0 
 0 
 0 
 0 
 0 
 0 
 0 
 0 
 0 
 0 
 0 
 0 
 0 
 0 
 0 
 0 
 0 
 0 
 0 
 0 
 0 
 0 
 0 
 0 
 0 
 0 
 0 
 0 
 0 
 0 
 0 
 0 
 0 
 0 
 0 
 0 
 0 
 0 
 0 
 0 
 0 
 0 
 0 
 0 
 0 
 0 
 0 
 0 
 0 
 0 
 0 
 0 
 0 
 0 
 0 
 0 
 0 
 0 
 0 
 0 
 0 
 0 
 0 
 0 
 0 
 0 
 0 
 0 
 0 
 0 
 0 
 0 
 0 
 0 
 0 
 0 
 0 
 0 
 0 
 0 
 0 
 0 
 0 
 0 
 0 
 0 
 0 
 0 
 0 
 0 
 0 
 0 
 0 
 0 
 0 
 0 
 0 
 0 
 0 
 0 
 0 
 0 
 0 
 0 
 0 
 0 
 0 
 0 
 0 
 0 
 0 
 0 
 0 
 0 
 0 
 2 
 0 
 0 
 0 
 0 
 0 
 0 
 0 
 0 
 0 
 0 
 0 
 0 
 0 
 0 
 0 
 0 
 0 
 0 
 0 
 0 
 0 
 0 
 0 
 0 
 0 
 0 
 0 
 0 
 0 
 0 
 0 
 0 
 0 
 0 
 0 
 0 
 0 
 0 
 0 
 0 
 0 
 0 
 0 
 0 
 0 
 0 
 0 
 0 
 0 
 0 
 0 
 0 
 0 
 0 
 0 
 0 
 0 
 0 
 0 
 0 
 0 
 0 
 0 
 0 
 0 
 0 
 0 
 0 
 0 
 0 
 0 
 0 
 0 
 0 
 0 
 0 
 0 
 0 
 0 
 0 
 0 
 0 
 0 
 0 
 0 
 0 
 0 
 0 
 0 
 0 
 0 
 0 
 
 
 21 
 0 
 0 
 0 
 0 
 0 
 0 
 0 
 0 
 0 
 0 
 0 
 0 
 0 
 0 
 0 
 0 
 0 
 0 
 0 
 0 
 0 
 1 
 0 
 0 
 0 
 0 
 0 
 0 
 0 
 0 
 0 
 0 
 0 
 0 
 0 
 0 
 0 
 0 
 0 
 0 
 0 
 0 
 0 
 0 
 0 
 0 
 0 
 0 
 0 
 0 
 0 
 0 
 0 
 0 
 0 
 0 
 0 
 0 
 0 
 0 
 0 
 0 
 0 
 0 
 0 
 0 
 0 
 0 
 0 
 0 
 0 
 0 
 0 
 0 
 0 
 0 
 0 
 0 
 0 
 0 
 0 
 0 
 0 
 0 
 0 
 0 
 0 
 0 
 0 
 0 
 0 
 0 
 0 
 0 
 0 
 0 
 0 
 0 
 0 
 0 
 0 
 0 
 0 
 0 
 0 
 0 
 0 
 0 
 0 
 0 
 0 
 0 
 0 
 0 
 0 
 0 
 0 
 0 
 0 
 0 
 0 
 0 
 0 
 0 
 0 
 0 
 0 
 0 
 0 
 0 
 0 
 0 
 0 
 0 
 0 
 0 
 0 
 0 
 0 
 0 
 0 
 0 
 0 
 0 
 0 
 0 
 0 
 0 
 0 
 0 
 0 
 0 
 0 
 0 
 0 
 0 
 0 
 0 
 0 
 0 
 0 
 0 
 0 
 0 
 0 
 0 
 0 
 0 
 0 
 0 
 0 
 0 
 0 
 0 
 0 
 0 
 0 
 0 
 0 
 0 
 0 
 0 
 0 
 0 
 0 
 0 
 0 
 0 
 0 
 0 
 0 
 0 
 0 
 0 
 0 
 0 
 0 
 0 
 0 
 0 
 0 
 0 
 0 
 0 
 0 
 0 
 0 
 0 
 0 
 0 
 0 
 0 
 0 
 0 
 0 
 0 
 0 
 0 
 0 
 0 
 0 
 0 
 0 
 0 
 0 
 0 
 0 
 0 
 0 
 0 
 0 
 0 
 0 
 0 
 0 
 0 
 0 
 0 
 0 
 0 
 0 
 0 
 0 
 0 
 0 
 0 
 0 
 0 
 0 
 0 
 0 
 0 
 0 
 0 
 0 
 0 
 0 
 0 
 0 
 0 
 0 
 0 
 0 
 0 
 0 
 0 
 0 
 0 
 0 
 0 
 0 
 0 
 0 
 0 
 0 
 0 
 0 
 0 
 0 
 0 
 0 
 0 
 0 
 0 
 0 
 0 
 0 
 0 
 0 
 0 
 0 
 0 
 0 
 0 
 0 
 0 
 0 
 0 
 0 
 0 
 0 
 0 
 0 
 0 
 0 
 0 
 0 
 0 
 0 
 0 
 0 
 0 
 0 
 0 
 0 
 0 
 0 
 0 
 0 
 0 
 0 
 0 
 0 
 0 
 0 
 0 
 0 
 0 
 0 
 0 
 0 
 0 
 0 
 0 
 0 
 0 
 0 
 0 
 0 
 0 
 0 
 0 
 0 
 0 
 0 
 0 
 0 
 0 
 0 
 0 
 0 
 0 
 0 
 0 
 0 
 0 
 0 
 0 
 0 
 0 
 0 
 0 
 0 
 0 
 0 
 0 
 0 
 0 
 0 
 0 
 0 
 0 
 0 
 0 
 0 
 0 
 0 
 0 
 0 
 0 
 0 
 0 
 0 
 0 
 0 
 0 
 0 
 0 
 0 
 0 
 0 
 0 
 0 
 0 
 0 
 0 
 0 
 0 
 0 
 0 
 0 
 0 
 0 
 0 
 0 
 0 
 0 
 0 
 0 
 0 
 0 
 0 
 0 
 0 
 0 
 0 
 0 
 0 
 0 
 0 
 0 
 0 
 0 
 0 
 0 
 0 
 0 
 0 
 0 
 0 
 0 
 0 
 0 
 0 
 0 
 0 
 0 
 0 
 0 
 0 
 0 
 0 
 0 
 0 
 0 
 0 
 0 
 0 
 0 
 0 
 0 
 0 
 0 
 0 
 0 
 0 
 0 
 0 
 0 
 0 
 0 
 0 
 0 
 0 
 0 
 0 
 0 
 0 
 0 
 0 
 0 
 0 
 0 
 0 
 0 
 0 
 0 
 0 
 0 
 0 
 0 
 0 
 0 
 0 
 0 
 0 
 0 
 0 
 0 
 0 
 0 
 0 
 0 
 0 
 0 
 0 
 0 
 0 
 0 
 0 
 0 
 0 
 0 
 0 
 0 
 0 
 0 
 0 
 0 
 0 
 0 
 0 
 0 
 0 
 0 
 0 
 0 
 0 
 0 
 0 
 0 
 0 
 0 
 0 
 0 
 0 
 0 
 0 
 0 
 0 
 0 
 0 
 0 
 0 
 0 
 0 
 0 
 0 
 0 
 0 
 0 
 0 
 0 
 0 
 0 
 0 
 0 
 0 
 0 
 0 
 0 
 0 
 0 
 0 
 0 
 0 
 0 
 0 
 0 
 0 
 0 
 0 
 0 
 0 
 0 
 0 
 0 
 0 
 0 
 0 
 0 
 0 
 0 
 0 
 0 
 0 
 0 
 0 
 0 
 0 
 0 
 0 
 0 
 0 
 0 
 0 
 0 
 0 
 0 
 0 
 0 
 0 
 0 
 0 
 0 
 0 
 0 
 0 
 0 
 0 
 0 
 0 
 0 
 0 
 0 
 0 
 0 
 0 
 0 
 0 
 0 
 0 
 0 
 0 
 0 
 0 
 0 
 0 
 0 
 0 
 0 
 0 
 0 
 0 
 0 
 0 
 0 
 0 
 0 
 0 
 0 
 0 
 0 
 0 
 0 
 0 
 0 
 0 
 0 
 0 
 0 
 0 
 0 
 0 
 0 
 0 
 0 
 0 
 0 
 0 
 0 
 0 
 0 
 0 
 0 
 0 
 0 
 0 
 0 
 0 
 0 
 0 
 0 
 0 
 0 
 0 
 0 
 0 
 0 
 0 
 0 
 0 
 0 
 0 
 0 
 0 
 0 
 0 
 0 
 0 
 0 
 0 
 0 
 0 
 0 
 0 
 0 
 0 
 0 
 0 
 0 
 0 
 0 
 0 
 0 
 0 
 0 
 0 
 0 
 0 
 0 
 0 
 0 
 0 
 0 
 0 
 0 
 0 
 0 
 0 
 0 
 0 
 0 
 0 
 0 
 0 
 0 
 0 
 0 
 0 
 0 
 0 
 0 
 0 
 0 
 0 
 0 
 0 
 0 
 0 
 0 
 0 
 0 
 0 
 0 
 0 
 0 
 0 
 0 
 0 
 0 
 0 
 0 
 0 
 0 
 0 
 0 
 0 
 0 
 0 
 0 
 0 
 0 
 0 
 0 
 0 
 0 
 0 
 0 
 0 
 0 
 0 
 0 
 0 
 0 
 0 
 0 
 0 
 0 
 0 
 0 
 0 
 0 
 0 
 0 
 0 
 0 
 0 
 0 
 0 
 0 
 0 
 0 
 0 
 0 
 0 
 0 
 0 
 0 
 0 
 0 
 0 
 0 
 0 
 0 
 0 
 0 
 0 
 0 
 0 
 0 
 0 
 0 
 0 
 0 
 0 
 0 
 0 
 0 
 0 
 0 
 0 
 0 
 0 
 0 
 0 
 0 
 0 
 0 
 0 
 0 
 0 
 0 
 0 
 0 
 0 
 
 
 22 
 0 
 0 
 0 
 0 
 0 
 0 
 0 
 0 
 0 
 0 
 0 
 0 
 0 
 0 
 0 
 0 
 0 
 0 
 0 
 0 
 0 
 0 
 9 
 0 
 0 
 0 
 0 
 0 
 0 
 0 
 0 
 0 
 0 
 0 
 0 
 0 
 0 
 0 
 0 
 0 
 0 
 0 
 0 
 0 
 0 
 0 
 0 
 0 
 0 
 0 
 0 
 0 
 0 
 0 
 0 
 0 
 0 
 0 
 0 
 0 
 0 
 0 
 0 
 0 
 0 
 0 
 0 
 0 
 0 
 0 
 0 
 0 
 0 
 0 
 0 
 0 
 0 
 0 
 0 
 0 
 0 
 0 
 0 
 0 
 0 
 0 
 0 
 0 
 0 
 0 
 0 
 0 
 0 
 0 
 0 
 0 
 0 
 0 
 0 
 0 
 0 
 0 
 0 
 0 
 0 
 0 
 0 
 0 
 0 
 0 
 0 
 0 
 0 
 0 
 0 
 0 
 0 
 0 
 0 
 0 
 0 
 0 
 0 
 0 
 0 
 0 
 0 
 0 
 0 
 0 
 0 
 0 
 0 
 0 
 0 
 0 
 0 
 0 
 0 
 0 
 0 
 0 
 0 
 0 
 0 
 0 
 0 
 0 
 0 
 0 
 0 
 0 
 0 
 0 
 0 
 0 
 0 
 0 
 0 
 0 
 0 
 0 
 0 
 0 
 0 
 0 
 0 
 0 
 0 
 0 
 0 
 0 
 0 
 0 
 0 
 0 
 0 
 0 
 0 
 0 
 0 
 0 
 0 
 0 
 0 
 0 
 0 
 0 
 0 
 0 
 0 
 0 
 0 
 0 
 0 
 0 
 0 
 0 
 0 
 0 
 0 
 0 
 0 
 0 
 0 
 0 
 0 
 0 
 0 
 0 
 0 
 0 
 0 
 0 
 0 
 0 
 0 
 0 
 0 
 0 
 0 
 0 
 0 
 0 
 0 
 0 
 0 
 0 
 0 
 0 
 0 
 0 
 0 
 0 
 0 
 0 
 0 
 0 
 0 
 0 
 0 
 0 
 0 
 0 
 0 
 0 
 0 
 0 
 0 
 0 
 0 
 0 
 0 
 0 
 0 
 0 
 0 
 0 
 0 
 0 
 0 
 0 
 0 
 0 
 0 
 0 
 0 
 0 
 0 
 0 
 0 
 0 
 0 
 0 
 0 
 0 
 0 
 0 
 0 
 0 
 0 
 0 
 0 
 0 
 0 
 0 
 0 
 0 
 0 
 0 
 0 
 0 
 0 
 0 
 0 
 0 
 0 
 0 
 0 
 0 
 0 
 0 
 0 
 0 
 0 
 0 
 0 
 0 
 0 
 0 
 0 
 0 
 0 
 0 
 0 
 0 
 0 
 0 
 0 
 0 
 0 
 0 
 0 
 0 
 0 
 0 
 0 
 0 
 0 
 0 
 0 
 0 
 0 
 0 
 0 
 0 
 0 
 0 
 0 
 0 
 0 
 0 
 0 
 0 
 0 
 0 
 0 
 0 
 0 
 0 
 0 
 0 
 0 
 0 
 0 
 0 
 0 
 0 
 0 
 0 
 0 
 0 
 0 
 0 
 0 
 0 
 0 
 0 
 0 
 0 
 0 
 0 
 0 
 0 
 0 
 0 
 0 
 0 
 0 
 0 
 0 
 0 
 0 
 0 
 0 
 0 
 0 
 0 
 0 
 0 
 0 
 0 
 0 
 0 
 0 
 0 
 0 
 0 
 0 
 0 
 0 
 0 
 0 
 0 
 0 
 0 
 0 
 0 
 0 
 0 
 0 
 0 
 0 
 0 
 0 
 0 
 0 
 0 
 0 
 0 
 0 
 0 
 0 
 0 
 0 
 0 
 0 
 0 
 0 
 0 
 0 
 0 
 0 
 0 
 0 
 0 
 0 
 0 
 0 
 0 
 0 
 0 
 0 
 0 
 0 
 0 
 0 
 0 
 0 
 0 
 0 
 0 
 0 
 0 
 0 
 0 
 0 
 0 
 0 
 0 
 0 
 0 
 0 
 0 
 0 
 0 
 0 
 0 
 0 
 0 
 0 
 0 
 0 
 0 
 0 
 0 
 0 
 0 
 0 
 0 
 0 
 0 
 0 
 0 
 0 
 0 
 0 
 0 
 0 
 0 
 0 
 0 
 0 
 0 
 0 
 0 
 0 
 0 
 0 
 0 
 0 
 0 
 0 
 0 
 0 
 0 
 0 
 0 
 0 
 0 
 0 
 0 
 0 
 0 
 0 
 0 
 0 
 0 
 0 
 0 
 0 
 0 
 0 
 0 
 0 
 0 
 0 
 0 
 0 
 0 
 0 
 0 
 0 
 0 
 0 
 0 
 0 
 0 
 0 
 0 
 0 
 0 
 0 
 0 
 0 
 0 
 0 
 0 
 0 
 0 
 0 
 0 
 0 
 0 
 0 
 0 
 0 
 0 
 0 
 0 
 0 
 0 
 0 
 0 
 0 
 0 
 0 
 0 
 0 
 0 
 0 
 0 
 0 
 0 
 0 
 0 
 0 
 0 
 0 
 0 
 0 
 0 
 0 
 0 
 0 
 0 
 0 
 0 
 0 
 0 
 0 
 0 
 0 
 0 
 0 
 0 
 0 
 0 
 0 
 0 
 0 
 0 
 0 
 0 
 0 
 0 
 0 
 0 
 0 
 0 
 0 
 0 
 0 
 0 
 0 
 0 
 0 
 0 
 0 
 0 
 0 
 0 
 0 
 0 
 0 
 0 
 0 
 0 
 0 
 0 
 0 
 0 
 0 
 0 
 0 
 0 
 0 
 0 
 0 
 0 
 0 
 0 
 0 
 0 
 0 
 0 
 0 
 0 
 0 
 0 
 0 
 0 
 0 
 0 
 0 
 0 
 0 
 0 
 0 
 0 
 0 
 0 
 0 
 0 
 0 
 0 
 0 
 0 
 0 
 0 
 0 
 0 
 0 
 0 
 0 
 0 
 0 
 0 
 0 
 0 
 0 
 0 
 0 
 0 
 0 
 0 
 0 
 0 
 0 
 0 
 0 
 0 
 0 
 0 
 0 
 0 
 0 
 0 
 0 
 0 
 0 
 0 
 0 
 0 
 0 
 0 
 0 
 0 
 0 
 0 
 0 
 0 
 0 
 0 
 0 
 0 
 0 
 0 
 0 
 0 
 0 
 0 
 0 
 0 
 0 
 0 
 0 
 0 
 0 
 0 
 0 
 0 
 0 
 0 
 0 
 0 
 0 
 0 
 0 
 0 
 0 
 0 
 0 
 0 
 0 
 0 
 0 
 0 
 0 
 0 
 0 
 0 
 0 
 0 
 0 
 0 
 0 
 0 
 0 
 0 
 0 
 0 
 0 
 0 
 0 
 0 
 0 
 0 
 0 
 0 
 0 
 0 
 0 
 0 
 0 
 0 
 0 
 0 
 0 
 0 
 0 
 0 
 0 
 0 
 0 
 0 
 0 
 0 
 0 
 0 
 0 
 0 
 0 
 0 
 0 
 0 
 0 
 0 
 0 
 0 
 0 
 0 
 0 
 0 
 0 
 0 
 0 
 0 
 0 
 0 
 0 
 0 
 0 
 0 
 0 
 0 
 0 
 0 
 0 
 0 
 0 
 0 
 0 
 0 
 0 
 0 
 
 
 23 
 0 
 0 
 0 
 0 
 0 
 0 
 0 
 0 
 0 
 0 
 0 
 0 
 0 
 0 
 0 
 0 
 0 
 0 
 0 
 0 
 0 
 0 
 0 
 0 
 2 
 0 
 0 
 0 
 0 
 0 
 0 
 0 
 0 
 0 
 0 
 0 
 0 
 0 
 0 
 0 
 0 
 0 
 0 
 0 
 0 
 0 
 0 
 0 
 0 
 0 
 0 
 0 
 0 
 0 
 0 
 0 
 0 
 0 
 0 
 0 
 0 
 0 
 0 
 0 
 0 
 0 
 0 
 0 
 0 
 0 
 0 
 0 
 0 
 0 
 0 
 0 
 0 
 0 
 0 
 0 
 0 
 0 
 0 
 0 
 0 
 0 
 0 
 0 
 0 
 0 
 0 
 0 
 0 
 0 
 0 
 0 
 0 
 0 
 0 
 0 
 0 
 0 
 0 
 0 
 0 
 0 
 0 
 0 
 0 
 0 
 0 
 0 
 0 
 0 
 0 
 0 
 0 
 0 
 0 
 0 
 0 
 0 
 0 
 0 
 0 
 0 
 0 
 0 
 0 
 0 
 0 
 0 
 0 
 0 
 0 
 0 
 0 
 0 
 0 
 0 
 0 
 0 
 0 
 0 
 0 
 0 
 0 
 0 
 0 
 0 
 0 
 0 
 0 
 0 
 0 
 0 
 0 
 0 
 0 
 0 
 0 
 0 
 0 
 0 
 0 
 0 
 0 
 0 
 0 
 0 
 0 
 0 
 0 
 0 
 0 
 0 
 0 
 0 
 0 
 0 
 0 
 0 
 0 
 0 
 0 
 0 
 0 
 0 
 0 
 0 
 0 
 0 
 0 
 0 
 0 
 0 
 0 
 0 
 0 
 0 
 0 
 0 
 0 
 0 
 0 
 0 
 0 
 0 
 0 
 0 
 0 
 0 
 0 
 0 
 0 
 0 
 0 
 0 
 0 
 0 
 0 
 0 
 0 
 0 
 0 
 0 
 0 
 0 
 0 
 0 
 0 
 0 
 0 
 0 
 0 
 0 
 0 
 0 
 0 
 0 
 0 
 0 
 0 
 0 
 0 
 0 
 0 
 0 
 0 
 0 
 0 
 0 
 0 
 0 
 0 
 0 
 0 
 0 
 0 
 0 
 0 
 0 
 0 
 0 
 0 
 0 
 0 
 0 
 0 
 0 
 0 
 0 
 0 
 0 
 0 
 0 
 0 
 0 
 0 
 0 
 0 
 0 
 0 
 0 
 0 
 0 
 0 
 0 
 0 
 0 
 0 
 0 
 0 
 0 
 0 
 0 
 0 
 0 
 0 
 0 
 0 
 0 
 0 
 0 
 0 
 0 
 0 
 0 
 0 
 0 
 0 
 0 
 0 
 0 
 0 
 0 
 0 
 0 
 0 
 0 
 0 
 0 
 0 
 0 
 0 
 0 
 0 
 0 
 0 
 0 
 0 
 0 
 0 
 0 
 0 
 0 
 0 
 0 
 0 
 0 
 0 
 0 
 0 
 0 
 0 
 0 
 0 
 0 
 0 
 0 
 0 
 0 
 0 
 0 
 0 
 0 
 0 
 0 
 0 
 0 
 0 
 0 
 0 
 0 
 0 
 0 
 0 
 0 
 0 
 0 
 0 
 0 
 0 
 0 
 0 
 0 
 0 
 0 
 0 
 0 
 0 
 0 
 0 
 0 
 0 
 0 
 0 
 0 
 0 
 0 
 0 
 0 
 0 
 0 
 0 
 0 
 0 
 0 
 0 
 0 
 0 
 0 
 0 
 0 
 0 
 0 
 0 
 0 
 0 
 0 
 0 
 0 
 0 
 0 
 0 
 0 
 0 
 0 
 0 
 0 
 0 
 0 
 0 
 0 
 0 
 0 
 0 
 0 
 0 
 0 
 0 
 0 
 0 
 0 
 0 
 0 
 0 
 0 
 0 
 0 
 0 
 0 
 0 
 0 
 0 
 0 
 0 
 0 
 0 
 0 
 0 
 0 
 0 
 0 
 0 
 0 
 0 
 0 
 0 
 0 
 0 
 0 
 0 
 0 
 0 
 0 
 0 
 0 
 0 
 0 
 0 
 0 
 0 
 0 
 0 
 0 
 0 
 0 
 0 
 0 
 0 
 0 
 0 
 0 
 0 
 0 
 0 
 0 
 0 
 0 
 0 
 0 
 0 
 0 
 0 
 0 
 0 
 0 
 0 
 0 
 0 
 0 
 0 
 0 
 0 
 0 
 0 
 0 
 0 
 0 
 0 
 0 
 0 
 0 
 0 
 0 
 0 
 0 
 0 
 0 
 0 
 0 
 0 
 0 
 0 
 0 
 0 
 0 
 0 
 0 
 0 
 0 
 0 
 0 
 0 
 0 
 0 
 0 
 0 
 0 
 0 
 0 
 0 
 0 
 0 
 0 
 0 
 0 
 0 
 0 
 0 
 0 
 0 
 0 
 0 
 0 
 0 
 0 
 0 
 0 
 0 
 0 
 0 
 0 
 0 
 0 
 0 
 0 
 0 
 0 
 0 
 0 
 0 
 0 
 0 
 0 
 0 
 0 
 0 
 0 
 0 
 0 
 0 
 0 
 0 
 0 
 0 
 0 
 0 
 0 
 0 
 0 
 0 
 0 
 0 
 0 
 0 
 0 
 0 
 0 
 0 
 0 
 0 
 0 
 0 
 0 
 0 
 0 
 0 
 0 
 0 
 0 
 0 
 0 
 0 
 0 
 0 
 0 
 0 
 0 
 0 
 0 
 0 
 0 
 0 
 0 
 0 
 0 
 0 
 0 
 0 
 0 
 0 
 0 
 0 
 0 
 0 
 0 
 0 
 0 
 0 
 0 
 0 
 0 
 0 
 0 
 0 
 0 
 0 
 0 
 0 
 0 
 0 
 0 
 0 
 0 
 0 
 0 
 0 
 0 
 0 
 0 
 0 
 0 
 0 
 0 
 0 
 0 
 0 
 0 
 0 
 0 
 0 
 0 
 0 
 0 
 0 
 0 
 0 
 0 
 0 
 0 
 0 
 0 
 0 
 0 
 0 
 0 
 0 
 0 
 0 
 0 
 0 
 0 
 0 
 0 
 0 
 0 
 0 
 0 
 0 
 0 
 0 
 0 
 0 
 0 
 0 
 0 
 0 
 0 
 0 
 0 
 0 
 0 
 0 
 0 
 0 
 0 
 0 
 0 
 0 
 0 
 0 
 0 
 0 
 0 
 0 
 0 
 0 
 0 
 0 
 0 
 0 
 0 
 0 
 0 
 0 
 0 
 0 
 0 
 0 
 0 
 0 
 0 
 0 
 0 
 0 
 0 
 0 
 0 
 0 
 0 
 0 
 0 
 0 
 0 
 0 
 0 
 0 
 0 
 0 
 0 
 0 
 0 
 0 
 0 
 0 
 0 
 0 
 0 
 0 
 0 
 0 
 0 
 0 
 0 
 0 
 0 
 0 
 0 
 0 
 0 
 0 
 0 
 0 
 0 
 0 
 0 
 0 
 0 
 0 
 0 
 0 
 0 
 0 
 0 
 0 
 0 
 0 
 0 
 0 
 0 
 0 
 0 
 0 
 0 
 0 
 0 
 0 
 0 
 0 
 0 
 0 
 0 
 0 
 0 
 0 
 0 
 0 
 0 
 0 
 0 
 0 
 0 
 0 
 0 
 
 
 24 
 0 
 0 
 0 
 0 
 0 
 0 
 0 
 0 
 0 
 0 
 0 
 0 
 0 
 0 
 0 
 0 
 0 
 0 
 0 
 0 
 0 
 0 
 0 
 0 
 5 
 0 
 0 
 0 
 0 
 0 
 0 
 0 
 0 
 0 
 0 
 0 
 0 
 0 
 0 
 0 
 0 
 0 
 0 
 0 
 0 
 0 
 0 
 0 
 0 
 0 
 0 
 0 
 0 
 0 
 0 
 0 
 0 
 0 
 0 
 0 
 0 
 0 
 0 
 0 
 0 
 0 
 0 
 0 
 0 
 0 
 0 
 0 
 0 
 0 
 0 
 0 
 0 
 0 
 0 
 0 
 0 
 0 
 0 
 0 
 0 
 0 
 0 
 0 
 0 
 0 
 0 
 0 
 0 
 0 
 0 
 0 
 0 
 0 
 0 
 0 
 0 
 0 
 0 
 0 
 0 
 0 
 0 
 0 
 0 
 0 
 0 
 0 
 0 
 0 
 0 
 0 
 0 
 0 
 0 
 0 
 0 
 0 
 0 
 0 
 0 
 0 
 0 
 0 
 0 
 0 
 0 
 0 
 0 
 0 
 0 
 0 
 0 
 0 
 0 
 0 
 0 
 0 
 0 
 0 
 0 
 0 
 0 
 0 
 0 
 0 
 0 
 0 
 0 
 0 
 0 
 0 
 0 
 0 
 0 
 0 
 0 
 0 
 0 
 0 
 0 
 0 
 0 
 0 
 0 
 0 
 0 
 0 
 0 
 0 
 0 
 0 
 0 
 0 
 0 
 0 
 0 
 0 
 0 
 0 
 0 
 0 
 0 
 0 
 0 
 0 
 0 
 0 
 0 
 0 
 0 
 0 
 0 
 0 
 0 
 0 
 0 
 0 
 0 
 0 
 0 
 0 
 0 
 0 
 0 
 0 
 0 
 0 
 0 
 0 
 0 
 0 
 0 
 0 
 0 
 0 
 0 
 0 
 0 
 0 
 0 
 0 
 0 
 0 
 0 
 0 
 0 
 0 
 0 
 0 
 0 
 0 
 0 
 0 
 0 
 0 
 0 
 0 
 0 
 0 
 0 
 0 
 0 
 0 
 0 
 0 
 0 
 0 
 0 
 0 
 0 
 0 
 0 
 0 
 0 
 0 
 0 
 0 
 0 
 0 
 0 
 0 
 0 
 0 
 0 
 0 
 0 
 0 
 0 
 0 
 0 
 0 
 0 
 0 
 0 
 0 
 0 
 0 
 0 
 0 
 0 
 0 
 0 
 0 
 0 
 0 
 0 
 0 
 0 
 0 
 0 
 0 
 0 
 0 
 0 
 0 
 0 
 0 
 0 
 0 
 0 
 0 
 0 
 0 
 0 
 0 
 0 
 0 
 0 
 0 
 0 
 0 
 0 
 0 
 0 
 0 
 0 
 0 
 0 
 0 
 0 
 0 
 0 
 0 
 0 
 0 
 0 
 0 
 0 
 0 
 0 
 0 
 0 
 0 
 0 
 0 
 0 
 0 
 0 
 0 
 0 
 0 
 0 
 0 
 0 
 0 
 0 
 0 
 0 
 0 
 0 
 0 
 0 
 0 
 0 
 0 
 0 
 0 
 0 
 0 
 0 
 0 
 0 
 0 
 0 
 0 
 0 
 0 
 0 
 0 
 0 
 0 
 0 
 0 
 0 
 0 
 0 
 0 
 0 
 0 
 0 
 0 
 0 
 0 
 0 
 0 
 0 
 0 
 0 
 0 
 0 
 0 
 0 
 0 
 0 
 0 
 0 
 0 
 0 
 0 
 0 
 0 
 0 
 0 
 0 
 0 
 0 
 0 
 0 
 0 
 0 
 0 
 0 
 0 
 0 
 0 
 0 
 0 
 0 
 0 
 0 
 0 
 0 
 0 
 0 
 0 
 0 
 0 
 0 
 0 
 0 
 0 
 0 
 0 
 0 
 0 
 0 
 0 
 0 
 0 
 0 
 0 
 0 
 0 
 0 
 0 
 0 
 0 
 0 
 0 
 0 
 0 
 0 
 0 
 0 
 0 
 0 
 0 
 0 
 0 
 0 
 0 
 0 
 0 
 0 
 0 
 0 
 0 
 0 
 0 
 0 
 0 
 0 
 0 
 0 
 0 
 0 
 0 
 0 
 0 
 0 
 0 
 0 
 0 
 0 
 0 
 0 
 0 
 0 
 0 
 0 
 0 
 0 
 0 
 0 
 0 
 0 
 0 
 0 
 0 
 0 
 0 
 0 
 0 
 0 
 0 
 0 
 0 
 0 
 0 
 0 
 0 
 0 
 0 
 0 
 0 
 0 
 0 
 0 
 0 
 0 
 0 
 0 
 0 
 0 
 0 
 0 
 0 
 0 
 0 
 0 
 0 
 0 
 0 
 0 
 0 
 0 
 0 
 0 
 0 
 0 
 0 
 0 
 0 
 0 
 0 
 0 
 0 
 0 
 0 
 0 
 0 
 0 
 0 
 0 
 0 
 0 
 0 
 0 
 0 
 0 
 0 
 0 
 0 
 0 
 0 
 0 
 0 
 0 
 0 
 0 
 0 
 0 
 0 
 0 
 0 
 0 
 0 
 0 
 0 
 0 
 0 
 0 
 0 
 0 
 0 
 0 
 0 
 0 
 0 
 0 
 0 
 0 
 0 
 0 
 0 
 0 
 0 
 0 
 0 
 0 
 0 
 0 
 0 
 0 
 0 
 0 
 0 
 0 
 0 
 0 
 0 
 0 
 0 
 0 
 0 
 0 
 0 
 0 
 0 
 0 
 0 
 0 
 0 
 0 
 0 
 0 
 0 
 0 
 0 
 0 
 0 
 0 
 0 
 0 
 0 
 0 
 0 
 0 
 0 
 0 
 0 
 0 
 0 
 0 
 0 
 0 
 0 
 0 
 0 
 0 
 0 
 0 
 0 
 0 
 0 
 0 
 0 
 0 
 0 
 0 
 0 
 0 
 0 
 0 
 0 
 0 
 0 
 0 
 0 
 0 
 0 
 0 
 0 
 0 
 0 
 0 
 0 
 0 
 0 
 0 
 0 
 0 
 0 
 0 
 0 
 0 
 0 
 0 
 0 
 0 
 0 
 0 
 0 
 0 
 0 
 0 
 0 
 0 
 0 
 0 
 0 
 0 
 0 
 0 
 0 
 0 
 0 
 0 
 0 
 0 
 0 
 0 
 0 
 0 
 0 
 0 
 0 
 0 
 0 
 0 
 0 
 0 
 0 
 0 
 0 
 0 
 0 
 0 
 0 
 0 
 0 
 0 
 0 
 0 
 0 
 0 
 0 
 0 
 0 
 0 
 0 
 0 
 0 
 0 
 0 
 0 
 0 
 0 
 0 
 0 
 0 
 0 
 0 
 0 
 0 
 0 
 0 
 0 
 0 
 0 
 0 
 0 
 0 
 0 
 0 
 0 
 0 
 0 
 0 
 0 
 0 
 0 
 0 
 0 
 0 
 0 
 0 
 0 
 0 
 0 
 0 
 0 
 0 
 0 
 0 
 0 
 0 
 0 
 0 
 0 
 0 
 0 
 0 
 0 
 0 
 0 
 0 
 0 
 0 
 0 
 0 
 0 
 0 
 0 
 0 
 0 
 0 
 0 
 0 
 0 
 0 
 0 
 0 
 0 
 0 
 0 
 0 
 0 
 0 
 0 
 0 
 
 
 25 
 0 
 0 
 0 
 0 
 0 
 0 
 0 
 0 
 0 
 0 
 0 
 0 
 0 
 0 
 0 
 0 
 0 
 0 
 0 
 0 
 0 
 0 
 0 
 0 
 0 
 12 
 0 
 0 
 0 
 0 
 0 
 0 
 0 
 0 
 0 
 0 
 0 
 0 
 0 
 0 
 0 
 0 
 0 
 0 
 0 
 0 
 0 
 0 
 0 
 0 
 0 
 0 
 0 
 0 
 0 
 0 
 0 
 0 
 0 
 0 
 0 
 0 
 0 
 0 
 0 
 0 
 0 
 0 
 0 
 0 
 0 
 0 
 0 
 0 
 0 
 0 
 0 
 0 
 0 
 0 
 0 
 0 
 0 
 0 
 0 
 0 
 0 
 0 
 0 
 0 
 0 
 0 
 0 
 0 
 0 
 0 
 0 
 0 
 0 
 0 
 0 
 0 
 0 
 0 
 0 
 0 
 0 
 0 
 0 
 0 
 0 
 0 
 0 
 0 
 0 
 0 
 0 
 0 
 0 
 0 
 0 
 0 
 0 
 0 
 0 
 0 
 0 
 0 
 0 
 0 
 0 
 0 
 0 
 0 
 0 
 0 
 0 
 0 
 0 
 0 
 0 
 0 
 0 
 0 
 0 
 0 
 0 
 0 
 0 
 0 
 0 
 0 
 0 
 0 
 0 
 0 
 0 
 0 
 0 
 0 
 0 
 0 
 0 
 0 
 0 
 0 
 0 
 0 
 0 
 0 
 0 
 0 
 0 
 0 
 0 
 0 
 0 
 0 
 0 
 0 
 0 
 0 
 0 
 0 
 0 
 0 
 0 
 0 
 0 
 0 
 0 
 0 
 0 
 0 
 0 
 0 
 0 
 0 
 0 
 0 
 0 
 0 
 0 
 0 
 0 
 0 
 0 
 0 
 0 
 0 
 0 
 0 
 0 
 0 
 0 
 0 
 0 
 0 
 0 
 0 
 0 
 0 
 0 
 0 
 0 
 0 
 0 
 0 
 0 
 0 
 0 
 0 
 0 
 0 
 0 
 0 
 0 
 0 
 0 
 0 
 0 
 0 
 0 
 0 
 0 
 0 
 0 
 0 
 0 
 0 
 0 
 0 
 0 
 0 
 0 
 0 
 0 
 0 
 0 
 0 
 0 
 0 
 0 
 0 
 0 
 0 
 0 
 0 
 0 
 0 
 0 
 0 
 0 
 0 
 0 
 0 
 0 
 0 
 0 
 0 
 0 
 0 
 0 
 0 
 0 
 0 
 0 
 0 
 0 
 0 
 0 
 0 
 0 
 0 
 0 
 0 
 0 
 0 
 0 
 0 
 0 
 0 
 0 
 0 
 0 
 0 
 0 
 0 
 0 
 0 
 0 
 0 
 0 
 0 
 0 
 0 
 0 
 0 
 0 
 0 
 0 
 0 
 0 
 0 
 0 
 0 
 0 
 0 
 0 
 0 
 0 
 0 
 0 
 0 
 0 
 0 
 0 
 0 
 0 
 0 
 0 
 0 
 0 
 0 
 0 
 0 
 0 
 0 
 0 
 0 
 0 
 0 
 0 
 0 
 0 
 0 
 0 
 0 
 0 
 0 
 0 
 0 
 0 
 0 
 0 
 0 
 0 
 0 
 0 
 0 
 0 
 0 
 0 
 0 
 0 
 0 
 0 
 0 
 0 
 0 
 0 
 0 
 0 
 0 
 0 
 0 
 0 
 0 
 0 
 0 
 0 
 0 
 0 
 0 
 0 
 0 
 0 
 0 
 0 
 0 
 0 
 0 
 0 
 0 
 0 
 0 
 0 
 0 
 0 
 0 
 0 
 0 
 0 
 0 
 0 
 0 
 0 
 0 
 0 
 0 
 0 
 0 
 0 
 0 
 0 
 0 
 0 
 0 
 0 
 0 
 0 
 0 
 0 
 0 
 0 
 0 
 0 
 0 
 0 
 0 
 0 
 0 
 0 
 0 
 0 
 0 
 0 
 0 
 0 
 0 
 0 
 0 
 0 
 0 
 0 
 0 
 0 
 0 
 0 
 0 
 0 
 0 
 0 
 0 
 0 
 0 
 0 
 0 
 0 
 0 
 0 
 0 
 0 
 0 
 0 
 0 
 0 
 0 
 0 
 0 
 0 
 0 
 0 
 0 
 0 
 0 
 0 
 0 
 0 
 0 
 0 
 0 
 0 
 0 
 0 
 0 
 0 
 0 
 0 
 0 
 0 
 0 
 0 
 0 
 0 
 0 
 0 
 0 
 0 
 0 
 0 
 0 
 0 
 0 
 0 
 0 
 0 
 0 
 0 
 0 
 0 
 0 
 0 
 0 
 0 
 0 
 0 
 0 
 0 
 0 
 0 
 0 
 0 
 0 
 0 
 0 
 0 
 0 
 0 
 0 
 0 
 0 
 0 
 0 
 0 
 0 
 0 
 0 
 0 
 0 
 0 
 0 
 0 
 0 
 0 
 0 
 0 
 0 
 0 
 0 
 0 
 0 
 0 
 0 
 0 
 0 
 0 
 0 
 0 
 0 
 0 
 0 
 0 
 0 
 0 
 0 
 0 
 0 
 0 
 0 
 0 
 0 
 0 
 0 
 0 
 0 
 0 
 0 
 0 
 0 
 0 
 0 
 0 
 0 
 0 
 0 
 0 
 0 
 0 
 0 
 0 
 0 
 0 
 0 
 0 
 0 
 0 
 0 
 0 
 0 
 0 
 0 
 0 
 0 
 0 
 0 
 0 
 0 
 0 
 0 
 0 
 0 
 0 
 0 
 0 
 0 
 0 
 0 
 0 
 0 
 0 
 0 
 0 
 0 
 0 
 0 
 0 
 0 
 0 
 0 
 0 
 0 
 0 
 0 
 0 
 0 
 0 
 0 
 0 
 0 
 0 
 0 
 0 
 0 
 0 
 0 
 0 
 0 
 0 
 0 
 0 
 0 
 0 
 0 
 0 
 0 
 0 
 0 
 0 
 0 
 0 
 0 
 0 
 0 
 0 
 0 
 0 
 0 
 0 
 0 
 0 
 0 
 0 
 0 
 0 
 0 
 0 
 0 
 0 
 0 
 0 
 0 
 0 
 0 
 0 
 0 
 0 
 0 
 0 
 0 
 0 
 0 
 0 
 0 
 0 
 0 
 0 
 0 
 0 
 0 
 0 
 0 
 0 
 0 
 0 
 0 
 0 
 0 
 0 
 0 
 0 
 0 
 0 
 0 
 0 
 0 
 0 
 0 
 0 
 0 
 0 
 0 
 0 
 0 
 0 
 0 
 0 
 0 
 0 
 0 
 0 
 0 
 0 
 0 
 0 
 0 
 0 
 0 
 0 
 0 
 0 
 0 
 0 
 0 
 0 
 0 
 0 
 0 
 0 
 0 
 0 
 0 
 0 
 0 
 0 
 0 
 0 
 0 
 0 
 0 
 0 
 0 
 0 
 0 
 0 
 0 
 0 
 0 
 0 
 0 
 0 
 0 
 0 
 0 
 0 
 0 
 0 
 0 
 0 
 0 
 0 
 0 
 0 
 0 
 0 
 0 
 0 
 0 
 0 
 0 
 0 
 0 
 0 
 0 
 0 
 0 
 0 
 0 
 0 
 0 
 0 
 0 
 0 
 0 
 0 
 0 
 0 
 0 
 0 
 0 
 0 
 0 
 0 
 0 
 0 
 0 
 
 
 26 
 0 
 0 
 0 
 0 
 0 
 0 
 0 
 0 
 0 
 0 
 0 
 0 
 0 
 0 
 0 
 0 
 0 
 0 
 0 
 0 
 0 
 2 
 0 
 0 
 0 
 0 
 113 
 0 
 0 
 0 
 0 
 0 
 0 
 0 
 0 
 0 
 0 
 0 
 0 
 0 
 0 
 0 
 0 
 0 
 0 
 0 
 0 
 0 
 0 
 0 
 0 
 0 
 0 
 0 
 0 
 0 
 0 
 0 
 0 
 0 
 0 
 0 
 0 
 0 
 0 
 0 
 0 
 0 
 0 
 0 
 0 
 0 
 0 
 0 
 0 
 0 
 0 
 0 
 0 
 0 
 0 
 0 
 0 
 0 
 0 
 0 
 0 
 0 
 0 
 0 
 0 
 0 
 0 
 0 
 0 
 0 
 0 
 0 
 0 
 0 
 0 
 0 
 0 
 0 
 0 
 0 
 0 
 0 
 0 
 0 
 0 
 0 
 0 
 0 
 0 
 0 
 0 
 0 
 0 
 0 
 0 
 0 
 0 
 0 
 0 
 0 
 0 
 0 
 0 
 0 
 0 
 0 
 0 
 0 
 0 
 0 
 0 
 0 
 0 
 0 
 0 
 0 
 0 
 0 
 0 
 0 
 0 
 0 
 0 
 0 
 0 
 0 
 0 
 0 
 0 
 0 
 0 
 0 
 0 
 0 
 0 
 0 
 0 
 0 
 0 
 0 
 0 
 0 
 0 
 0 
 0 
 0 
 0 
 0 
 0 
 0 
 0 
 0 
 0 
 0 
 0 
 0 
 0 
 0 
 0 
 0 
 0 
 0 
 0 
 0 
 0 
 0 
 0 
 0 
 0 
 0 
 0 
 0 
 0 
 0 
 0 
 0 
 0 
 0 
 0 
 0 
 0 
 0 
 0 
 0 
 0 
 0 
 0 
 0 
 0 
 0 
 0 
 0 
 0 
 0 
 0 
 0 
 0 
 0 
 0 
 0 
 0 
 0 
 0 
 0 
 0 
 0 
 0 
 0 
 0 
 0 
 0 
 0 
 0 
 0 
 0 
 0 
 0 
 0 
 0 
 0 
 0 
 0 
 0 
 0 
 0 
 0 
 0 
 0 
 0 
 0 
 0 
 0 
 0 
 0 
 0 
 0 
 0 
 0 
 0 
 0 
 0 
 0 
 0 
 0 
 0 
 0 
 0 
 0 
 0 
 0 
 0 
 0 
 0 
 0 
 0 
 0 
 0 
 0 
 0 
 0 
 0 
 0 
 0 
 0 
 0 
 0 
 0 
 0 
 0 
 0 
 0 
 0 
 0 
 0 
 0 
 0 
 0 
 0 
 0 
 0 
 0 
 0 
 0 
 0 
 0 
 0 
 0 
 0 
 0 
 0 
 0 
 0 
 0 
 0 
 0 
 0 
 0 
 0 
 0 
 0 
 0 
 0 
 0 
 0 
 0 
 0 
 0 
 0 
 0 
 0 
 0 
 0 
 0 
 0 
 0 
 0 
 0 
 0 
 0 
 0 
 0 
 0 
 0 
 0 
 0 
 0 
 0 
 0 
 0 
 0 
 0 
 0 
 0 
 0 
 0 
 0 
 0 
 0 
 0 
 0 
 0 
 0 
 0 
 0 
 0 
 0 
 0 
 0 
 0 
 0 
 0 
 0 
 0 
 0 
 0 
 0 
 0 
 0 
 0 
 0 
 0 
 0 
 0 
 0 
 0 
 0 
 0 
 0 
 0 
 0 
 0 
 0 
 0 
 0 
 0 
 0 
 0 
 0 
 0 
 0 
 0 
 0 
 0 
 0 
 0 
 0 
 0 
 0 
 0 
 0 
 0 
 0 
 0 
 0 
 0 
 0 
 0 
 0 
 0 
 0 
 0 
 0 
 0 
 0 
 0 
 0 
 0 
 0 
 0 
 0 
 0 
 0 
 0 
 0 
 0 
 0 
 0 
 0 
 0 
 0 
 0 
 0 
 0 
 0 
 0 
 0 
 0 
 0 
 0 
 0 
 0 
 0 
 0 
 0 
 0 
 0 
 0 
 0 
 0 
 0 
 0 
 0 
 0 
 0 
 0 
 0 
 0 
 0 
 0 
 0 
 0 
 0 
 0 
 0 
 0 
 0 
 0 
 0 
 0 
 0 
 0 
 0 
 0 
 0 
 0 
 0 
 0 
 0 
 0 
 0 
 0 
 0 
 0 
 0 
 0 
 0 
 0 
 0 
 0 
 0 
 0 
 0 
 0 
 0 
 0 
 0 
 0 
 0 
 0 
 0 
 0 
 0 
 0 
 0 
 0 
 0 
 0 
 0 
 0 
 0 
 0 
 0 
 0 
 0 
 0 
 0 
 0 
 0 
 0 
 0 
 0 
 0 
 0 
 0 
 0 
 0 
 0 
 0 
 0 
 0 
 0 
 0 
 0 
 0 
 0 
 0 
 0 
 0 
 0 
 0 
 0 
 0 
 0 
 0 
 0 
 0 
 0 
 0 
 0 
 0 
 0 
 0 
 0 
 0 
 0 
 0 
 0 
 0 
 0 
 0 
 0 
 0 
 0 
 0 
 0 
 0 
 0 
 0 
 0 
 0 
 0 
 0 
 0 
 0 
 0 
 0 
 0 
 0 
 0 
 0 
 0 
 0 
 0 
 0 
 0 
 0 
 0 
 0 
 0 
 0 
 0 
 0 
 0 
 0 
 0 
 0 
 0 
 0 
 0 
 0 
 0 
 0 
 0 
 0 
 0 
 0 
 0 
 0 
 0 
 0 
 0 
 0 
 0 
 0 
 0 
 0 
 0 
 0 
 0 
 0 
 0 
 0 
 0 
 0 
 0 
 0 
 0 
 0 
 0 
 0 
 0 
 0 
 0 
 0 
 0 
 0 
 0 
 0 
 0 
 0 
 0 
 0 
 0 
 0 
 0 
 0 
 0 
 0 
 0 
 0 
 0 
 0 
 0 
 0 
 0 
 0 
 0 
 0 
 0 
 0 
 0 
 0 
 0 
 0 
 0 
 0 
 0 
 0 
 0 
 0 
 0 
 0 
 0 
 0 
 0 
 0 
 0 
 0 
 0 
 0 
 0 
 0 
 0 
 0 
 0 
 0 
 0 
 0 
 0 
 0 
 0 
 0 
 0 
 0 
 0 
 0 
 0 
 0 
 0 
 0 
 0 
 0 
 0 
 0 
 0 
 0 
 0 
 0 
 0 
 0 
 0 
 0 
 0 
 0 
 0 
 0 
 0 
 0 
 0 
 0 
 0 
 0 
 0 
 0 
 0 
 0 
 0 
 0 
 0 
 0 
 0 
 0 
 0 
 0 
 0 
 0 
 0 
 0 
 0 
 0 
 0 
 0 
 0 
 0 
 0 
 0 
 0 
 0 
 0 
 0 
 0 
 0 
 0 
 0 
 0 
 0 
 0 
 0 
 0 
 0 
 0 
 0 
 0 
 0 
 0 
 0 
 0 
 0 
 0 
 0 
 0 
 0 
 0 
 0 
 0 
 0 
 0 
 0 
 0 
 0 
 0 
 0 
 0 
 0 
 0 
 0 
 0 
 0 
 0 
 0 
 0 
 0 
 0 
 0 
 0 
 0 
 0 
 0 
 0 
 0 
 0 
 0 
 0 
 0 
 0 
 0 
 0 
 0 
 0 
 0 
 
 
 27 
 0 
 0 
 0 
 0 
 0 
 0 
 0 
 0 
 0 
 0 
 0 
 0 
 0 
 0 
 0 
 0 
 0 
 0 
 0 
 0 
 0 
 0 
 0 
 0 
 0 
 0 
 0 
 12 
 0 
 0 
 0 
 0 
 0 
 0 
 0 
 0 
 0 
 0 
 0 
 0 
 0 
 0 
 0 
 0 
 0 
 0 
 0 
 0 
 0 
 0 
 0 
 0 
 0 
 0 
 0 
 0 
 0 
 0 
 0 
 0 
 0 
 0 
 0 
 0 
 0 
 0 
 0 
 0 
 0 
 0 
 0 
 0 
 0 
 0 
 0 
 0 
 0 
 0 
 0 
 0 
 0 
 0 
 0 
 0 
 0 
 0 
 0 
 0 
 0 
 0 
 0 
 0 
 0 
 0 
 0 
 0 
 0 
 0 
 0 
 0 
 0 
 0 
 0 
 0 
 0 
 0 
 0 
 0 
 0 
 0 
 0 
 0 
 0 
 0 
 0 
 0 
 0 
 0 
 0 
 0 
 0 
 0 
 0 
 0 
 0 
 0 
 0 
 0 
 0 
 0 
 0 
 0 
 0 
 0 
 0 
 0 
 0 
 0 
 0 
 0 
 0 
 0 
 0 
 0 
 0 
 0 
 0 
 0 
 0 
 0 
 0 
 0 
 0 
 0 
 0 
 0 
 0 
 0 
 0 
 0 
 0 
 0 
 0 
 0 
 0 
 0 
 0 
 0 
 0 
 0 
 0 
 0 
 0 
 0 
 0 
 0 
 0 
 0 
 0 
 0 
 0 
 0 
 0 
 0 
 0 
 0 
 0 
 0 
 0 
 0 
 0 
 0 
 0 
 0 
 0 
 0 
 0 
 0 
 0 
 0 
 0 
 0 
 0 
 0 
 0 
 0 
 0 
 0 
 0 
 0 
 0 
 0 
 0 
 0 
 0 
 0 
 0 
 0 
 0 
 0 
 0 
 0 
 0 
 0 
 0 
 0 
 0 
 0 
 0 
 0 
 0 
 0 
 0 
 0 
 0 
 0 
 0 
 0 
 0 
 0 
 0 
 0 
 0 
 0 
 0 
 0 
 0 
 0 
 0 
 0 
 0 
 0 
 0 
 0 
 0 
 0 
 0 
 0 
 0 
 0 
 0 
 0 
 0 
 0 
 0 
 0 
 0 
 0 
 0 
 0 
 0 
 0 
 0 
 0 
 0 
 0 
 0 
 0 
 0 
 0 
 0 
 0 
 0 
 0 
 0 
 0 
 0 
 0 
 0 
 0 
 0 
 0 
 0 
 0 
 0 
 0 
 0 
 0 
 0 
 0 
 0 
 0 
 0 
 0 
 0 
 0 
 0 
 0 
 0 
 0 
 0 
 0 
 0 
 0 
 0 
 0 
 0 
 0 
 0 
 0 
 0 
 0 
 0 
 0 
 0 
 0 
 0 
 0 
 0 
 0 
 0 
 0 
 0 
 0 
 0 
 0 
 0 
 0 
 0 
 0 
 0 
 0 
 0 
 0 
 0 
 0 
 0 
 0 
 0 
 0 
 0 
 0 
 0 
 0 
 0 
 0 
 0 
 0 
 0 
 0 
 0 
 0 
 0 
 0 
 0 
 0 
 0 
 0 
 0 
 0 
 0 
 0 
 0 
 0 
 0 
 0 
 0 
 0 
 0 
 0 
 0 
 0 
 0 
 0 
 0 
 0 
 0 
 0 
 0 
 0 
 0 
 0 
 0 
 0 
 0 
 0 
 0 
 0 
 0 
 0 
 0 
 0 
 0 
 0 
 0 
 0 
 0 
 0 
 0 
 0 
 0 
 0 
 0 
 0 
 0 
 0 
 0 
 0 
 0 
 0 
 0 
 0 
 0 
 0 
 0 
 0 
 0 
 0 
 0 
 0 
 0 
 0 
 0 
 0 
 0 
 0 
 0 
 0 
 0 
 0 
 0 
 0 
 0 
 0 
 0 
 0 
 0 
 0 
 0 
 0 
 0 
 0 
 0 
 0 
 0 
 0 
 0 
 0 
 0 
 0 
 0 
 0 
 0 
 0 
 0 
 0 
 0 
 0 
 0 
 0 
 0 
 0 
 0 
 0 
 0 
 0 
 0 
 0 
 0 
 0 
 0 
 0 
 0 
 0 
 0 
 0 
 0 
 0 
 0 
 0 
 0 
 0 
 0 
 0 
 0 
 0 
 0 
 0 
 0 
 0 
 0 
 0 
 0 
 0 
 0 
 0 
 0 
 0 
 0 
 0 
 0 
 0 
 0 
 0 
 0 
 0 
 0 
 0 
 0 
 0 
 0 
 0 
 0 
 0 
 0 
 0 
 0 
 0 
 0 
 0 
 0 
 0 
 0 
 0 
 0 
 0 
 0 
 0 
 0 
 0 
 0 
 0 
 0 
 0 
 0 
 0 
 0 
 0 
 0 
 0 
 0 
 0 
 0 
 0 
 0 
 0 
 0 
 0 
 0 
 0 
 0 
 0 
 0 
 0 
 0 
 0 
 0 
 0 
 0 
 0 
 0 
 0 
 0 
 0 
 0 
 0 
 0 
 0 
 0 
 0 
 0 
 0 
 0 
 0 
 0 
 0 
 0 
 0 
 0 
 0 
 0 
 0 
 0 
 0 
 0 
 0 
 0 
 0 
 0 
 0 
 0 
 0 
 0 
 0 
 0 
 0 
 0 
 0 
 0 
 0 
 0 
 0 
 0 
 0 
 0 
 0 
 0 
 0 
 0 
 0 
 0 
 0 
 0 
 0 
 0 
 0 
 0 
 0 
 0 
 0 
 0 
 0 
 0 
 0 
 0 
 0 
 0 
 0 
 0 
 0 
 0 
 0 
 0 
 0 
 0 
 0 
 0 
 0 
 0 
 0 
 0 
 0 
 0 
 0 
 0 
 0 
 0 
 0 
 0 
 0 
 0 
 0 
 0 
 0 
 0 
 0 
 0 
 0 
 0 
 0 
 0 
 0 
 0 
 0 
 0 
 0 
 0 
 0 
 0 
 0 
 0 
 0 
 0 
 0 
 0 
 0 
 0 
 0 
 0 
 0 
 0 
 0 
 0 
 0 
 0 
 0 
 0 
 0 
 0 
 0 
 0 
 0 
 0 
 0 
 0 
 0 
 0 
 0 
 0 
 0 
 0 
 0 
 0 
 0 
 0 
 0 
 0 
 0 
 0 
 0 
 0 
 0 
 0 
 0 
 0 
 0 
 0 
 0 
 0 
 0 
 0 
 0 
 0 
 0 
 0 
 0 
 0 
 0 
 0 
 0 
 0 
 0 
 0 
 0 
 0 
 0 
 0 
 0 
 0 
 0 
 0 
 0 
 0 
 0 
 0 
 0 
 0 
 0 
 0 
 0 
 0 
 0 
 0 
 0 
 0 
 0 
 0 
 0 
 0 
 0 
 0 
 0 
 0 
 0 
 0 
 0 
 0 
 0 
 0 
 0 
 0 
 0 
 0 
 0 
 0 
 0 
 0 
 0 
 0 
 0 
 0 
 0 
 0 
 0 
 0 
 0 
 0 
 0 
 0 
 0 
 0 
 0 
 0 
 0 
 0 
 0 
 0 
 0 
 0 
 0 
 0 
 0 
 0 
 0 
 0 
 0 
 0 
 0 
 0 
 0 
 0 
 0 
 0 
 0 
 0 
 0 
 
 
 28 
 0 
 0 
 0 
 0 
 0 
 0 
 0 
 0 
 0 
 0 
 0 
 0 
 0 
 0 
 0 
 0 
 0 
 0 
 0 
 0 
 0 
 0 
 0 
 0 
 0 
 0 
 0 
 0 
 8 
 0 
 0 
 0 
 0 
 0 
 0 
 0 
 0 
 0 
 0 
 0 
 0 
 0 
 0 
 0 
 0 
 0 
 0 
 0 
 0 
 0 
 0 
 0 
 0 
 0 
 0 
 0 
 0 
 0 
 0 
 0 
 0 
 0 
 0 
 0 
 0 
 0 
 0 
 0 
 0 
 0 
 0 
 0 
 0 
 0 
 0 
 0 
 0 
 0 
 0 
 0 
 0 
 0 
 0 
 0 
 0 
 0 
 0 
 0 
 0 
 0 
 0 
 0 
 0 
 0 
 0 
 0 
 0 
 0 
 0 
 0 
 0 
 0 
 0 
 0 
 0 
 0 
 0 
 0 
 0 
 0 
 0 
 0 
 0 
 0 
 0 
 0 
 0 
 0 
 0 
 0 
 0 
 0 
 0 
 0 
 0 
 0 
 0 
 0 
 0 
 0 
 0 
 0 
 0 
 0 
 0 
 0 
 0 
 0 
 0 
 0 
 0 
 0 
 0 
 0 
 0 
 0 
 0 
 0 
 0 
 0 
 0 
 0 
 0 
 0 
 0 
 0 
 0 
 0 
 0 
 0 
 0 
 0 
 0 
 0 
 0 
 0 
 0 
 0 
 0 
 0 
 0 
 0 
 0 
 0 
 0 
 0 
 0 
 0 
 0 
 0 
 0 
 0 
 0 
 0 
 0 
 0 
 0 
 0 
 0 
 0 
 0 
 0 
 0 
 0 
 0 
 0 
 0 
 0 
 0 
 0 
 0 
 0 
 0 
 0 
 0 
 0 
 0 
 0 
 0 
 0 
 0 
 0 
 0 
 0 
 0 
 0 
 0 
 0 
 0 
 0 
 0 
 0 
 0 
 0 
 0 
 0 
 0 
 0 
 0 
 0 
 0 
 0 
 0 
 0 
 0 
 0 
 0 
 0 
 0 
 0 
 0 
 0 
 0 
 0 
 0 
 0 
 0 
 0 
 0 
 0 
 0 
 0 
 0 
 0 
 0 
 0 
 0 
 0 
 0 
 0 
 0 
 0 
 0 
 0 
 0 
 0 
 0 
 0 
 0 
 0 
 0 
 0 
 0 
 0 
 0 
 0 
 0 
 0 
 0 
 0 
 0 
 0 
 0 
 0 
 0 
 0 
 0 
 0 
 0 
 0 
 0 
 0 
 0 
 0 
 0 
 0 
 0 
 0 
 0 
 0 
 0 
 0 
 0 
 0 
 0 
 0 
 0 
 0 
 0 
 0 
 0 
 0 
 0 
 0 
 0 
 0 
 0 
 0 
 0 
 0 
 0 
 0 
 0 
 0 
 0 
 0 
 0 
 0 
 0 
 0 
 0 
 0 
 0 
 0 
 0 
 0 
 0 
 0 
 0 
 0 
 0 
 0 
 0 
 0 
 0 
 0 
 0 
 0 
 0 
 0 
 0 
 0 
 0 
 0 
 0 
 0 
 0 
 0 
 0 
 0 
 0 
 0 
 0 
 0 
 0 
 0 
 0 
 0 
 0 
 0 
 0 
 0 
 0 
 0 
 0 
 0 
 0 
 0 
 0 
 0 
 0 
 0 
 0 
 0 
 0 
 0 
 0 
 0 
 0 
 0 
 0 
 0 
 0 
 0 
 0 
 0 
 0 
 0 
 0 
 0 
 0 
 0 
 0 
 0 
 0 
 0 
 0 
 0 
 0 
 0 
 0 
 0 
 0 
 0 
 0 
 0 
 0 
 0 
 0 
 0 
 0 
 0 
 0 
 0 
 0 
 0 
 0 
 0 
 0 
 0 
 0 
 0 
 0 
 0 
 0 
 0 
 0 
 0 
 0 
 0 
 0 
 0 
 0 
 0 
 0 
 0 
 0 
 0 
 0 
 0 
 0 
 0 
 0 
 0 
 0 
 0 
 0 
 0 
 0 
 0 
 0 
 0 
 0 
 0 
 0 
 0 
 0 
 0 
 0 
 0 
 0 
 0 
 0 
 0 
 0 
 0 
 0 
 0 
 0 
 0 
 0 
 0 
 0 
 0 
 0 
 0 
 0 
 0 
 0 
 0 
 0 
 0 
 0 
 0 
 0 
 0 
 0 
 0 
 0 
 0 
 0 
 0 
 0 
 0 
 0 
 0 
 0 
 0 
 0 
 0 
 0 
 0 
 0 
 0 
 0 
 0 
 0 
 0 
 0 
 0 
 0 
 0 
 0 
 0 
 0 
 0 
 0 
 0 
 0 
 0 
 0 
 0 
 0 
 0 
 0 
 0 
 0 
 0 
 0 
 0 
 0 
 0 
 0 
 0 
 0 
 0 
 0 
 0 
 0 
 0 
 0 
 0 
 0 
 0 
 0 
 0 
 0 
 0 
 0 
 0 
 0 
 0 
 0 
 0 
 0 
 0 
 0 
 0 
 0 
 0 
 0 
 0 
 0 
 0 
 0 
 0 
 0 
 0 
 0 
 0 
 0 
 0 
 0 
 0 
 0 
 0 
 0 
 0 
 0 
 0 
 0 
 0 
 0 
 0 
 0 
 0 
 0 
 0 
 0 
 0 
 0 
 0 
 0 
 0 
 0 
 0 
 0 
 0 
 0 
 0 
 0 
 0 
 0 
 0 
 0 
 0 
 0 
 0 
 0 
 0 
 0 
 0 
 0 
 0 
 0 
 0 
 0 
 0 
 0 
 0 
 0 
 0 
 0 
 0 
 0 
 0 
 0 
 0 
 0 
 0 
 0 
 0 
 0 
 0 
 0 
 0 
 0 
 0 
 0 
 0 
 0 
 0 
 0 
 0 
 0 
 0 
 0 
 0 
 0 
 0 
 0 
 0 
 0 
 0 
 0 
 0 
 0 
 0 
 0 
 0 
 0 
 0 
 0 
 0 
 0 
 0 
 0 
 0 
 0 
 0 
 0 
 0 
 0 
 0 
 0 
 0 
 0 
 0 
 0 
 0 
 0 
 0 
 0 
 0 
 0 
 0 
 0 
 0 
 0 
 0 
 0 
 0 
 0 
 0 
 0 
 0 
 0 
 0 
 0 
 0 
 0 
 0 
 0 
 0 
 0 
 0 
 0 
 0 
 0 
 0 
 0 
 0 
 0 
 0 
 0 
 0 
 0 
 0 
 0 
 0 
 0 
 0 
 0 
 0 
 0 
 0 
 0 
 0 
 0 
 0 
 0 
 0 
 0 
 0 
 0 
 0 
 0 
 0 
 0 
 0 
 0 
 0 
 0 
 0 
 0 
 0 
 0 
 0 
 0 
 0 
 0 
 0 
 0 
 0 
 0 
 0 
 0 
 0 
 0 
 0 
 0 
 0 
 0 
 0 
 0 
 0 
 0 
 0 
 0 
 0 
 0 
 0 
 0 
 0 
 0 
 0 
 0 
 0 
 0 
 0 
 0 
 0 
 0 
 0 
 0 
 0 
 0 
 0 
 0 
 0 
 0 
 0 
 0 
 0 
 0 
 0 
 0 
 0 
 0 
 0 
 0 
 0 
 0 
 0 
 0 
 0 
 0 
 0 
 0 
 0 
 0 
 0 
 0 
 0 
 0 
 0 
 
 
 29 
 0 
 0 
 0 
 0 
 0 
 0 
 0 
 0 
 0 
 0 
 0 
 0 
 0 
 0 
 0 
 0 
 0 
 0 
 0 
 0 
 0 
 0 
 0 
 0 
 0 
 0 
 0 
 0 
 1 
 5 
 0 
 0 
 0 
 0 
 0 
 0 
 0 
 0 
 0 
 0 
 0 
 0 
 0 
 0 
 0 
 0 
 0 
 0 
 0 
 0 
 0 
 0 
 0 
 0 
 0 
 0 
 0 
 0 
 0 
 0 
 0 
 0 
 0 
 0 
 0 
 0 
 0 
 0 
 0 
 0 
 0 
 0 
 0 
 0 
 0 
 0 
 0 
 0 
 0 
 0 
 0 
 0 
 0 
 0 
 0 
 0 
 0 
 0 
 0 
 0 
 0 
 0 
 0 
 0 
 0 
 0 
 0 
 0 
 0 
 0 
 0 
 0 
 0 
 0 
 0 
 0 
 0 
 0 
 0 
 0 
 0 
 0 
 0 
 0 
 0 
 0 
 0 
 0 
 0 
 0 
 0 
 0 
 0 
 0 
 0 
 0 
 0 
 0 
 0 
 0 
 0 
 0 
 0 
 0 
 0 
 0 
 0 
 0 
 0 
 0 
 0 
 0 
 0 
 0 
 0 
 0 
 0 
 0 
 0 
 0 
 0 
 0 
 0 
 0 
 0 
 0 
 0 
 0 
 0 
 0 
 0 
 0 
 0 
 0 
 0 
 0 
 0 
 0 
 0 
 0 
 0 
 0 
 0 
 0 
 0 
 0 
 0 
 0 
 0 
 0 
 0 
 0 
 0 
 0 
 0 
 0 
 0 
 0 
 0 
 0 
 0 
 0 
 0 
 0 
 0 
 0 
 0 
 0 
 0 
 0 
 0 
 0 
 0 
 0 
 0 
 0 
 0 
 0 
 0 
 0 
 0 
 0 
 0 
 0 
 0 
 0 
 0 
 0 
 0 
 0 
 0 
 0 
 0 
 0 
 0 
 0 
 0 
 0 
 0 
 0 
 0 
 0 
 0 
 0 
 0 
 0 
 0 
 0 
 0 
 0 
 0 
 0 
 0 
 0 
 0 
 0 
 0 
 0 
 0 
 0 
 0 
 0 
 0 
 0 
 0 
 0 
 0 
 0 
 0 
 0 
 0 
 0 
 0 
 0 
 0 
 0 
 0 
 0 
 0 
 0 
 0 
 0 
 0 
 0 
 0 
 0 
 0 
 0 
 0 
 0 
 0 
 0 
 0 
 0 
 0 
 0 
 0 
 0 
 0 
 0 
 0 
 0 
 0 
 0 
 0 
 0 
 0 
 0 
 0 
 0 
 0 
 0 
 0 
 0 
 0 
 0 
 0 
 0 
 0 
 0 
 0 
 0 
 0 
 0 
 0 
 0 
 0 
 0 
 0 
 0 
 0 
 0 
 0 
 0 
 0 
 0 
 0 
 0 
 0 
 0 
 0 
 0 
 0 
 0 
 0 
 0 
 0 
 0 
 0 
 0 
 0 
 0 
 0 
 0 
 0 
 0 
 0 
 0 
 0 
 0 
 0 
 0 
 0 
 0 
 0 
 0 
 0 
 0 
 0 
 0 
 0 
 0 
 0 
 0 
 0 
 0 
 0 
 0 
 0 
 0 
 0 
 0 
 0 
 0 
 0 
 0 
 0 
 0 
 0 
 0 
 0 
 0 
 0 
 0 
 0 
 0 
 0 
 0 
 0 
 0 
 0 
 0 
 0 
 0 
 0 
 0 
 0 
 0 
 0 
 0 
 0 
 0 
 0 
 0 
 0 
 0 
 0 
 0 
 0 
 0 
 0 
 0 
 0 
 0 
 0 
 0 
 0 
 0 
 0 
 0 
 0 
 0 
 0 
 0 
 0 
 0 
 0 
 0 
 0 
 0 
 0 
 0 
 0 
 0 
 0 
 0 
 0 
 0 
 0 
 0 
 0 
 0 
 0 
 0 
 0 
 0 
 0 
 0 
 0 
 0 
 0 
 0 
 0 
 0 
 0 
 0 
 0 
 0 
 0 
 0 
 0 
 0 
 0 
 0 
 0 
 0 
 0 
 0 
 0 
 0 
 0 
 0 
 0 
 0 
 0 
 0 
 0 
 0 
 0 
 0 
 0 
 0 
 0 
 0 
 0 
 0 
 0 
 0 
 0 
 0 
 0 
 0 
 0 
 0 
 0 
 0 
 0 
 0 
 0 
 0 
 0 
 0 
 0 
 0 
 0 
 0 
 0 
 0 
 0 
 0 
 0 
 0 
 0 
 0 
 0 
 0 
 0 
 0 
 0 
 0 
 0 
 0 
 0 
 0 
 0 
 0 
 0 
 0 
 0 
 0 
 0 
 0 
 0 
 0 
 0 
 0 
 0 
 0 
 0 
 0 
 0 
 0 
 0 
 0 
 0 
 0 
 0 
 0 
 0 
 0 
 0 
 0 
 0 
 0 
 0 
 0 
 0 
 0 
 0 
 0 
 0 
 0 
 0 
 0 
 0 
 0 
 0 
 0 
 0 
 0 
 0 
 0 
 0 
 0 
 0 
 0 
 0 
 0 
 0 
 0 
 0 
 0 
 0 
 0 
 0 
 0 
 0 
 0 
 0 
 0 
 0 
 0 
 0 
 0 
 0 
 0 
 0 
 0 
 0 
 0 
 0 
 0 
 0 
 0 
 0 
 0 
 0 
 0 
 0 
 0 
 0 
 0 
 0 
 0 
 0 
 0 
 0 
 0 
 0 
 0 
 0 
 0 
 0 
 0 
 0 
 0 
 0 
 0 
 0 
 0 
 0 
 0 
 0 
 0 
 0 
 0 
 0 
 0 
 0 
 0 
 0 
 0 
 0 
 0 
 0 
 0 
 0 
 0 
 0 
 0 
 0 
 0 
 0 
 0 
 0 
 0 
 0 
 0 
 0 
 0 
 0 
 0 
 0 
 0 
 0 
 0 
 0 
 0 
 0 
 0 
 0 
 0 
 0 
 0 
 0 
 0 
 0 
 0 
 0 
 0 
 0 
 0 
 0 
 0 
 0 
 0 
 0 
 0 
 0 
 0 
 0 
 0 
 0 
 0 
 0 
 0 
 0 
 0 
 0 
 0 
 0 
 0 
 0 
 0 
 0 
 0 
 0 
 0 
 0 
 0 
 0 
 0 
 0 
 0 
 0 
 0 
 0 
 0 
 0 
 0 
 0 
 0 
 0 
 0 
 0 
 0 
 0 
 0 
 0 
 0 
 0 
 0 
 0 
 0 
 0 
 0 
 0 
 0 
 0 
 0 
 0 
 0 
 0 
 0 
 0 
 0 
 0 
 0 
 0 
 0 
 0 
 0 
 0 
 0 
 0 
 0 
 0 
 0 
 0 
 0 
 0 
 0 
 0 
 0 
 0 
 0 
 0 
 0 
 0 
 0 
 0 
 0 
 0 
 0 
 0 
 0 
 0 
 0 
 0 
 0 
 0 
 0 
 0 
 0 
 0 
 0 
 0 
 0 
 0 
 0 
 0 
 0 
 0 
 0 
 0 
 0 
 0 
 0 
 0 
 0 
 0 
 0 
 0 
 0 
 0 
 0 
 0 
 0 
 0 
 0 
 0 
 0 
 0 
 0 
 0 
 0 
 0 
 0 
 0 
 0 
 0 
 0 
 0 
 0 
 0 
 0 
 
 
 30 
 0 
 0 
 0 
 0 
 0 
 0 
 0 
 0 
 0 
 0 
 0 
 0 
 0 
 0 
 0 
 0 
 0 
 0 
 0 
 0 
 0 
 0 
 0 
 0 
 0 
 0 
 0 
 0 
 0 
 0 
 14 
 0 
 0 
 0 
 0 
 0 
 0 
 0 
 0 
 0 
 0 
 0 
 0 
 0 
 0 
 0 
 0 
 0 
 0 
 0 
 0 
 0 
 0 
 0 
 0 
 0 
 0 
 0 
 0 
 0 
 0 
 0 
 0 
 0 
 0 
 0 
 0 
 0 
 0 
 0 
 0 
 0 
 0 
 0 
 0 
 0 
 0 
 0 
 0 
 0 
 0 
 0 
 0 
 0 
 0 
 0 
 0 
 0 
 0 
 0 
 0 
 0 
 0 
 0 
 0 
 0 
 0 
 0 
 0 
 0 
 0 
 0 
 0 
 0 
 0 
 0 
 0 
 0 
 0 
 0 
 0 
 0 
 0 
 0 
 0 
 0 
 0 
 0 
 0 
 0 
 0 
 0 
 0 
 0 
 0 
 0 
 0 
 0 
 0 
 0 
 0 
 0 
 0 
 0 
 0 
 0 
 0 
 0 
 0 
 0 
 0 
 0 
 0 
 0 
 0 
 0 
 0 
 0 
 0 
 0 
 0 
 0 
 0 
 0 
 0 
 0 
 0 
 0 
 0 
 0 
 0 
 0 
 0 
 0 
 0 
 0 
 0 
 0 
 0 
 0 
 0 
 0 
 0 
 0 
 0 
 0 
 0 
 0 
 0 
 0 
 0 
 0 
 0 
 0 
 0 
 0 
 0 
 0 
 0 
 0 
 0 
 0 
 0 
 0 
 0 
 0 
 0 
 0 
 0 
 0 
 0 
 0 
 0 
 0 
 0 
 0 
 0 
 0 
 0 
 0 
 0 
 0 
 0 
 0 
 0 
 0 
 0 
 0 
 0 
 0 
 0 
 0 
 0 
 0 
 0 
 0 
 0 
 0 
 0 
 0 
 0 
 0 
 0 
 0 
 0 
 0 
 0 
 0 
 0 
 0 
 0 
 0 
 0 
 0 
 0 
 0 
 0 
 0 
 0 
 0 
 0 
 0 
 0 
 0 
 0 
 0 
 0 
 0 
 0 
 0 
 0 
 0 
 0 
 0 
 0 
 0 
 0 
 0 
 0 
 0 
 0 
 0 
 0 
 0 
 0 
 0 
 0 
 0 
 0 
 0 
 0 
 0 
 0 
 0 
 0 
 0 
 0 
 0 
 0 
 0 
 0 
 0 
 0 
 0 
 0 
 0 
 0 
 0 
 0 
 0 
 0 
 0 
 0 
 0 
 0 
 0 
 0 
 0 
 0 
 0 
 0 
 0 
 0 
 0 
 0 
 0 
 0 
 0 
 0 
 0 
 0 
 0 
 0 
 0 
 0 
 0 
 0 
 0 
 0 
 0 
 0 
 0 
 0 
 0 
 0 
 0 
 0 
 0 
 0 
 0 
 0 
 0 
 0 
 0 
 0 
 0 
 0 
 0 
 0 
 0 
 0 
 0 
 0 
 0 
 0 
 0 
 0 
 0 
 0 
 0 
 0 
 0 
 0 
 0 
 0 
 0 
 0 
 0 
 0 
 0 
 0 
 0 
 0 
 0 
 0 
 0 
 0 
 0 
 0 
 0 
 0 
 0 
 0 
 0 
 0 
 0 
 0 
 0 
 0 
 0 
 0 
 0 
 0 
 0 
 0 
 0 
 0 
 0 
 0 
 0 
 0 
 0 
 0 
 0 
 0 
 0 
 0 
 0 
 0 
 0 
 0 
 0 
 0 
 0 
 0 
 0 
 0 
 0 
 0 
 0 
 0 
 0 
 0 
 0 
 0 
 0 
 0 
 0 
 0 
 0 
 0 
 0 
 0 
 0 
 0 
 0 
 0 
 0 
 0 
 0 
 0 
 0 
 0 
 0 
 0 
 0 
 0 
 0 
 0 
 0 
 0 
 0 
 0 
 0 
 0 
 0 
 0 
 0 
 0 
 0 
 0 
 0 
 0 
 0 
 0 
 0 
 0 
 0 
 0 
 0 
 0 
 0 
 0 
 0 
 0 
 0 
 0 
 0 
 0 
 0 
 0 
 0 
 0 
 0 
 0 
 0 
 0 
 0 
 0 
 0 
 0 
 0 
 0 
 0 
 0 
 0 
 0 
 0 
 0 
 0 
 0 
 0 
 0 
 0 
 0 
 0 
 0 
 0 
 0 
 0 
 0 
 0 
 0 
 0 
 0 
 0 
 0 
 0 
 0 
 0 
 0 
 0 
 0 
 0 
 0 
 0 
 0 
 0 
 0 
 0 
 0 
 0 
 0 
 0 
 0 
 0 
 0 
 0 
 0 
 0 
 0 
 0 
 0 
 0 
 0 
 0 
 0 
 0 
 0 
 0 
 0 
 0 
 0 
 0 
 0 
 0 
 0 
 0 
 0 
 0 
 0 
 0 
 0 
 0 
 0 
 0 
 0 
 0 
 0 
 0 
 0 
 0 
 0 
 0 
 0 
 0 
 0 
 0 
 0 
 0 
 0 
 0 
 0 
 0 
 0 
 0 
 0 
 0 
 0 
 0 
 0 
 0 
 0 
 0 
 0 
 0 
 0 
 0 
 0 
 0 
 0 
 0 
 0 
 0 
 0 
 0 
 0 
 0 
 0 
 0 
 0 
 0 
 0 
 0 
 0 
 0 
 0 
 0 
 0 
 0 
 0 
 0 
 0 
 0 
 0 
 0 
 0 
 0 
 0 
 0 
 0 
 0 
 0 
 0 
 0 
 0 
 0 
 0 
 0 
 0 
 0 
 0 
 0 
 0 
 0 
 0 
 0 
 0 
 0 
 0 
 0 
 0 
 0 
 0 
 0 
 0 
 0 
 0 
 0 
 0 
 0 
 0 
 0 
 0 
 0 
 0 
 0 
 0 
 0 
 0 
 0 
 0 
 0 
 0 
 0 
 0 
 0 
 0 
 0 
 0 
 0 
 0 
 0 
 0 
 0 
 0 
 0 
 0 
 0 
 0 
 0 
 0 
 0 
 0 
 0 
 0 
 0 
 0 
 0 
 0 
 0 
 0 
 0 
 0 
 0 
 0 
 0 
 0 
 0 
 0 
 0 
 0 
 0 
 0 
 0 
 0 
 0 
 0 
 0 
 0 
 0 
 0 
 0 
 0 
 0 
 0 
 0 
 0 
 0 
 0 
 0 
 0 
 0 
 0 
 0 
 0 
 0 
 0 
 0 
 0 
 0 
 0 
 0 
 0 
 0 
 0 
 0 
 0 
 0 
 0 
 0 
 0 
 0 
 0 
 0 
 0 
 0 
 0 
 0 
 0 
 0 
 0 
 0 
 0 
 0 
 0 
 0 
 0 
 0 
 0 
 0 
 0 
 0 
 0 
 0 
 0 
 0 
 0 
 0 
 0 
 0 
 0 
 0 
 0 
 0 
 0 
 0 
 0 
 0 
 0 
 0 
 0 
 0 
 0 
 0 
 0 
 0 
 0 
 0 
 0 
 0 
 0 
 0 
 0 
 0 
 0 
 0 
 0 
 0 
 0 
 0 
 0 
 0 
 0 
 0 
 0 
 0 
 0 
 0 
 0 
 0 
 0 
 0 
 0 
 0 
 0 
 
 
 31 
 0 
 0 
 0 
 0 
 0 
 0 
 0 
 0 
 0 
 0 
 0 
 0 
 0 
 0 
 0 
 0 
 0 
 0 
 0 
 0 
 0 
 0 
 0 
 0 
 0 
 0 
 0 
 0 
 0 
 0 
 0 
 31 
 0 
 0 
 0 
 0 
 0 
 0 
 0 
 0 
 0 
 0 
 0 
 0 
 0 
 0 
 0 
 0 
 0 
 0 
 0 
 0 
 0 
 0 
 0 
 0 
 0 
 0 
 0 
 0 
 0 
 0 
 0 
 0 
 0 
 0 
 0 
 0 
 0 
 0 
 0 
 0 
 0 
 0 
 0 
 0 
 0 
 0 
 0 
 0 
 0 
 0 
 0 
 0 
 0 
 0 
 0 
 0 
 0 
 0 
 0 
 0 
 0 
 0 
 0 
 0 
 0 
 0 
 0 
 0 
 0 
 0 
 0 
 0 
 0 
 0 
 0 
 0 
 0 
 0 
 0 
 0 
 0 
 0 
 0 
 0 
 0 
 0 
 0 
 0 
 0 
 0 
 0 
 0 
 0 
 0 
 0 
 0 
 0 
 0 
 0 
 0 
 0 
 0 
 0 
 0 
 0 
 0 
 0 
 0 
 0 
 0 
 0 
 0 
 0 
 0 
 0 
 0 
 0 
 0 
 0 
 0 
 0 
 0 
 0 
 0 
 0 
 0 
 0 
 0 
 0 
 0 
 0 
 0 
 0 
 0 
 0 
 0 
 0 
 0 
 0 
 0 
 0 
 0 
 0 
 0 
 0 
 0 
 0 
 0 
 0 
 0 
 0 
 0 
 0 
 0 
 0 
 0 
 0 
 0 
 0 
 0 
 0 
 0 
 0 
 0 
 0 
 0 
 0 
 0 
 0 
 0 
 0 
 0 
 0 
 0 
 0 
 0 
 0 
 0 
 0 
 0 
 0 
 0 
 0 
 0 
 0 
 0 
 0 
 0 
 0 
 0 
 0 
 0 
 0 
 0 
 0 
 0 
 0 
 0 
 0 
 0 
 0 
 0 
 0 
 0 
 0 
 0 
 0 
 0 
 0 
 0 
 0 
 0 
 0 
 0 
 0 
 0 
 0 
 0 
 0 
 0 
 0 
 0 
 0 
 0 
 0 
 0 
 0 
 0 
 0 
 0 
 0 
 0 
 0 
 0 
 0 
 0 
 0 
 0 
 0 
 0 
 0 
 0 
 0 
 0 
 0 
 0 
 0 
 0 
 0 
 0 
 0 
 0 
 0 
 0 
 0 
 0 
 0 
 0 
 0 
 0 
 0 
 0 
 0 
 0 
 0 
 0 
 0 
 0 
 0 
 0 
 0 
 0 
 0 
 0 
 0 
 0 
 0 
 0 
 0 
 0 
 0 
 0 
 0 
 0 
 0 
 0 
 0 
 0 
 0 
 0 
 0 
 0 
 0 
 0 
 0 
 0 
 0 
 0 
 0 
 0 
 0 
 0 
 0 
 0 
 0 
 0 
 0 
 0 
 0 
 0 
 0 
 0 
 0 
 0 
 0 
 0 
 0 
 0 
 0 
 0 
 0 
 0 
 0 
 0 
 0 
 0 
 0 
 0 
 0 
 0 
 0 
 0 
 0 
 0 
 0 
 0 
 0 
 0 
 0 
 0 
 0 
 0 
 0 
 0 
 0 
 0 
 0 
 0 
 0 
 0 
 0 
 0 
 0 
 0 
 0 
 0 
 0 
 0 
 0 
 0 
 0 
 0 
 0 
 0 
 0 
 0 
 0 
 0 
 0 
 0 
 0 
 0 
 0 
 0 
 0 
 0 
 0 
 0 
 0 
 0 
 0 
 0 
 0 
 0 
 0 
 0 
 0 
 0 
 0 
 0 
 0 
 0 
 0 
 0 
 0 
 0 
 0 
 0 
 0 
 0 
 0 
 0 
 0 
 0 
 0 
 0 
 0 
 0 
 0 
 0 
 0 
 0 
 0 
 0 
 0 
 0 
 0 
 0 
 0 
 0 
 0 
 0 
 0 
 0 
 0 
 0 
 0 
 0 
 0 
 0 
 0 
 0 
 0 
 0 
 0 
 0 
 0 
 0 
 0 
 0 
 0 
 0 
 0 
 0 
 0 
 0 
 0 
 0 
 0 
 0 
 0 
 0 
 0 
 0 
 0 
 0 
 0 
 0 
 0 
 0 
 0 
 0 
 0 
 0 
 0 
 0 
 0 
 0 
 0 
 0 
 0 
 0 
 0 
 0 
 0 
 0 
 0 
 0 
 0 
 0 
 0 
 0 
 0 
 0 
 0 
 0 
 0 
 0 
 0 
 0 
 0 
 0 
 0 
 0 
 0 
 0 
 0 
 0 
 0 
 0 
 0 
 0 
 0 
 0 
 0 
 0 
 0 
 0 
 0 
 0 
 0 
 0 
 0 
 0 
 0 
 0 
 0 
 0 
 0 
 0 
 0 
 0 
 0 
 0 
 0 
 0 
 0 
 0 
 0 
 0 
 0 
 0 
 0 
 0 
 0 
 0 
 0 
 0 
 0 
 0 
 0 
 0 
 0 
 0 
 0 
 0 
 0 
 0 
 0 
 0 
 0 
 0 
 0 
 0 
 0 
 0 
 0 
 0 
 0 
 0 
 0 
 0 
 0 
 0 
 0 
 0 
 0 
 0 
 0 
 0 
 0 
 0 
 0 
 0 
 0 
 0 
 0 
 0 
 0 
 0 
 0 
 0 
 0 
 0 
 0 
 0 
 0 
 0 
 0 
 0 
 0 
 0 
 0 
 0 
 0 
 0 
 0 
 0 
 0 
 0 
 0 
 0 
 0 
 0 
 0 
 0 
 0 
 0 
 0 
 0 
 0 
 0 
 0 
 0 
 0 
 0 
 0 
 0 
 0 
 0 
 0 
 0 
 0 
 0 
 0 
 0 
 0 
 0 
 0 
 0 
 0 
 0 
 0 
 0 
 0 
 0 
 0 
 0 
 0 
 0 
 0 
 0 
 0 
 0 
 0 
 0 
 0 
 0 
 0 
 0 
 0 
 0 
 0 
 0 
 0 
 0 
 0 
 0 
 0 
 0 
 0 
 0 
 0 
 0 
 0 
 0 
 0 
 0 
 0 
 0 
 0 
 0 
 0 
 0 
 0 
 0 
 0 
 0 
 0 
 0 
 0 
 0 
 0 
 0 
 0 
 0 
 0 
 0 
 0 
 0 
 0 
 0 
 0 
 0 
 0 
 0 
 0 
 0 
 0 
 0 
 0 
 0 
 0 
 0 
 0 
 0 
 0 
 0 
 0 
 0 
 0 
 0 
 0 
 0 
 0 
 0 
 0 
 0 
 0 
 0 
 0 
 0 
 0 
 0 
 0 
 0 
 0 
 0 
 0 
 0 
 0 
 0 
 0 
 0 
 0 
 0 
 0 
 0 
 0 
 0 
 0 
 0 
 0 
 0 
 0 
 0 
 0 
 0 
 0 
 0 
 0 
 0 
 0 
 0 
 0 
 0 
 0 
 0 
 0 
 0 
 0 
 0 
 0 
 0 
 0 
 0 
 0 
 0 
 0 
 0 
 0 
 0 
 0 
 0 
 0 
 0 
 0 
 0 
 0 
 0 
 0 
 0 
 0 
 0 
 0 
 0 
 0 
 0 
 0 
 0 
 0 
 0 
 0 
 0 
 
 
 32 
 0 
 0 
 0 
 0 
 0 
 0 
 0 
 0 
 0 
 0 
 0 
 0 
 0 
 0 
 0 
 0 
 0 
 0 
 0 
 0 
 0 
 0 
 0 
 0 
 0 
 0 
 0 
 0 
 0 
 0 
 0 
 0 
 44 
 0 
 0 
 0 
 0 
 0 
 0 
 0 
 0 
 0 
 0 
 0 
 0 
 0 
 0 
 0 
 0 
 0 
 0 
 0 
 0 
 0 
 0 
 0 
 0 
 0 
 0 
 0 
 0 
 0 
 0 
 0 
 0 
 0 
 0 
 0 
 0 
 0 
 0 
 0 
 0 
 0 
 0 
 0 
 0 
 0 
 0 
 0 
 0 
 0 
 0 
 0 
 0 
 0 
 0 
 0 
 0 
 0 
 0 
 0 
 0 
 0 
 0 
 0 
 0 
 0 
 0 
 0 
 0 
 0 
 0 
 0 
 0 
 0 
 0 
 0 
 0 
 0 
 0 
 0 
 0 
 0 
 0 
 0 
 0 
 0 
 0 
 0 
 0 
 0 
 0 
 0 
 0 
 0 
 0 
 0 
 0 
 0 
 0 
 0 
 0 
 0 
 0 
 0 
 0 
 0 
 0 
 0 
 0 
 0 
 0 
 0 
 0 
 0 
 0 
 0 
 0 
 0 
 0 
 0 
 0 
 0 
 0 
 0 
 0 
 0 
 0 
 0 
 0 
 0 
 0 
 0 
 0 
 0 
 0 
 0 
 0 
 0 
 0 
 0 
 0 
 0 
 0 
 0 
 0 
 0 
 0 
 0 
 0 
 0 
 0 
 0 
 0 
 0 
 0 
 0 
 0 
 0 
 0 
 0 
 0 
 0 
 0 
 0 
 0 
 0 
 0 
 0 
 0 
 0 
 0 
 0 
 0 
 0 
 0 
 0 
 0 
 0 
 0 
 0 
 0 
 0 
 0 
 0 
 0 
 0 
 0 
 0 
 0 
 0 
 0 
 0 
 0 
 0 
 0 
 0 
 0 
 0 
 0 
 0 
 0 
 0 
 0 
 0 
 0 
 0 
 0 
 0 
 0 
 0 
 0 
 0 
 0 
 0 
 0 
 0 
 0 
 0 
 0 
 0 
 0 
 0 
 0 
 0 
 0 
 0 
 0 
 0 
 0 
 0 
 0 
 0 
 0 
 0 
 0 
 0 
 0 
 0 
 0 
 0 
 0 
 0 
 0 
 0 
 0 
 0 
 0 
 0 
 0 
 0 
 0 
 0 
 0 
 0 
 0 
 0 
 0 
 0 
 0 
 0 
 0 
 0 
 0 
 0 
 0 
 0 
 0 
 0 
 0 
 0 
 0 
 0 
 0 
 0 
 0 
 0 
 0 
 0 
 0 
 0 
 0 
 0 
 0 
 0 
 0 
 0 
 0 
 0 
 0 
 0 
 0 
 0 
 0 
 0 
 0 
 0 
 0 
 0 
 0 
 0 
 0 
 0 
 0 
 0 
 0 
 0 
 0 
 0 
 0 
 0 
 0 
 0 
 0 
 0 
 0 
 0 
 0 
 0 
 0 
 0 
 0 
 0 
 0 
 0 
 0 
 0 
 0 
 0 
 0 
 0 
 0 
 0 
 0 
 0 
 0 
 0 
 0 
 0 
 0 
 0 
 0 
 0 
 0 
 0 
 0 
 0 
 0 
 0 
 0 
 0 
 0 
 0 
 0 
 0 
 0 
 0 
 0 
 0 
 0 
 0 
 0 
 0 
 0 
 0 
 0 
 0 
 0 
 0 
 0 
 0 
 0 
 0 
 0 
 0 
 0 
 0 
 0 
 0 
 0 
 0 
 0 
 0 
 0 
 0 
 0 
 0 
 0 
 0 
 0 
 0 
 0 
 0 
 0 
 0 
 0 
 0 
 0 
 0 
 0 
 0 
 0 
 0 
 0 
 0 
 0 
 0 
 0 
 0 
 0 
 0 
 0 
 0 
 0 
 0 
 0 
 0 
 0 
 0 
 0 
 0 
 0 
 0 
 0 
 0 
 0 
 0 
 0 
 0 
 0 
 0 
 0 
 0 
 0 
 0 
 0 
 0 
 0 
 0 
 0 
 0 
 0 
 0 
 0 
 0 
 0 
 0 
 0 
 0 
 0 
 0 
 0 
 0 
 0 
 0 
 0 
 0 
 0 
 0 
 0 
 0 
 0 
 0 
 0 
 0 
 0 
 0 
 0 
 0 
 0 
 0 
 0 
 0 
 0 
 0 
 0 
 0 
 0 
 0 
 0 
 0 
 0 
 0 
 0 
 0 
 0 
 0 
 0 
 0 
 0 
 0 
 0 
 0 
 0 
 0 
 0 
 0 
 0 
 0 
 0 
 0 
 0 
 0 
 0 
 0 
 0 
 0 
 0 
 0 
 0 
 0 
 0 
 0 
 0 
 0 
 0 
 0 
 0 
 0 
 0 
 0 
 0 
 0 
 0 
 0 
 0 
 0 
 0 
 0 
 0 
 0 
 0 
 0 
 0 
 0 
 0 
 0 
 0 
 0 
 0 
 0 
 0 
 0 
 0 
 0 
 0 
 0 
 0 
 0 
 0 
 0 
 0 
 0 
 0 
 0 
 0 
 0 
 0 
 0 
 0 
 0 
 0 
 0 
 0 
 0 
 0 
 0 
 0 
 0 
 0 
 0 
 0 
 0 
 0 
 0 
 0 
 0 
 0 
 0 
 0 
 0 
 0 
 0 
 0 
 0 
 0 
 0 
 0 
 0 
 0 
 0 
 0 
 0 
 0 
 0 
 0 
 0 
 0 
 0 
 0 
 0 
 0 
 0 
 0 
 0 
 0 
 0 
 0 
 0 
 0 
 0 
 0 
 0 
 0 
 0 
 0 
 0 
 0 
 0 
 0 
 0 
 0 
 0 
 0 
 0 
 0 
 0 
 0 
 0 
 0 
 0 
 0 
 0 
 0 
 0 
 0 
 0 
 0 
 0 
 0 
 0 
 0 
 0 
 0 
 0 
 0 
 0 
 0 
 0 
 0 
 0 
 0 
 0 
 0 
 0 
 0 
 0 
 0 
 0 
 0 
 0 
 0 
 0 
 0 
 0 
 0 
 0 
 0 
 0 
 0 
 0 
 0 
 0 
 0 
 0 
 0 
 0 
 0 
 0 
 0 
 0 
 0 
 0 
 0 
 0 
 0 
 0 
 0 
 0 
 0 
 0 
 0 
 0 
 0 
 0 
 0 
 0 
 0 
 0 
 0 
 0 
 0 
 0 
 0 
 0 
 0 
 0 
 0 
 0 
 0 
 0 
 0 
 0 
 0 
 0 
 0 
 0 
 0 
 0 
 0 
 0 
 0 
 0 
 0 
 0 
 0 
 0 
 0 
 0 
 0 
 0 
 0 
 0 
 0 
 0 
 0 
 0 
 0 
 0 
 0 
 0 
 0 
 0 
 0 
 0 
 0 
 0 
 0 
 0 
 0 
 0 
 0 
 0 
 0 
 0 
 0 
 0 
 0 
 0 
 0 
 0 
 0 
 0 
 0 
 0 
 0 
 0 
 0 
 0 
 0 
 0 
 0 
 0 
 0 
 0 
 0 
 0 
 0 
 0 
 0 
 0 
 0 
 0 
 0 
 0 
 0 
 0 
 0 
 0 
 0 
 0 
 0 
 0 
 0 
 0 
 
 
 33 
 0 
 0 
 0 
 0 
 0 
 0 
 0 
 0 
 0 
 0 
 0 
 0 
 0 
 0 
 0 
 0 
 0 
 0 
 0 
 0 
 0 
 0 
 0 
 0 
 0 
 0 
 0 
 0 
 0 
 0 
 0 
 0 
 0 
 7 
 0 
 0 
 0 
 0 
 0 
 0 
 0 
 0 
 0 
 0 
 0 
 0 
 0 
 0 
 0 
 0 
 0 
 0 
 0 
 0 
 0 
 0 
 0 
 0 
 0 
 0 
 0 
 0 
 0 
 0 
 0 
 0 
 0 
 0 
 0 
 0 
 0 
 0 
 0 
 0 
 0 
 0 
 0 
 0 
 0 
 0 
 0 
 0 
 0 
 0 
 0 
 0 
 0 
 0 
 0 
 0 
 0 
 0 
 0 
 0 
 0 
 0 
 0 
 0 
 0 
 0 
 0 
 0 
 0 
 0 
 0 
 0 
 0 
 0 
 0 
 0 
 0 
 0 
 0 
 0 
 0 
 0 
 0 
 0 
 0 
 0 
 0 
 0 
 0 
 0 
 0 
 0 
 0 
 0 
 0 
 0 
 0 
 0 
 0 
 0 
 0 
 0 
 0 
 0 
 0 
 0 
 0 
 0 
 0 
 0 
 0 
 0 
 0 
 0 
 0 
 0 
 0 
 0 
 0 
 0 
 0 
 0 
 0 
 0 
 0 
 0 
 0 
 0 
 0 
 0 
 0 
 0 
 0 
 0 
 0 
 0 
 0 
 0 
 0 
 0 
 0 
 0 
 0 
 0 
 0 
 0 
 0 
 0 
 0 
 0 
 0 
 0 
 0 
 0 
 0 
 0 
 0 
 0 
 0 
 0 
 0 
 0 
 0 
 0 
 0 
 0 
 0 
 0 
 0 
 0 
 0 
 0 
 0 
 0 
 0 
 0 
 0 
 0 
 0 
 0 
 0 
 0 
 0 
 0 
 0 
 0 
 0 
 0 
 0 
 0 
 0 
 0 
 0 
 0 
 0 
 0 
 0 
 0 
 0 
 0 
 0 
 0 
 0 
 0 
 0 
 0 
 0 
 0 
 0 
 0 
 0 
 0 
 0 
 0 
 0 
 0 
 0 
 0 
 0 
 0 
 0 
 0 
 0 
 0 
 0 
 0 
 0 
 0 
 0 
 0 
 0 
 0 
 0 
 0 
 0 
 0 
 0 
 0 
 0 
 0 
 0 
 0 
 0 
 0 
 0 
 0 
 0 
 0 
 0 
 0 
 0 
 0 
 0 
 0 
 0 
 0 
 0 
 0 
 0 
 0 
 0 
 0 
 0 
 0 
 0 
 0 
 0 
 0 
 0 
 0 
 0 
 0 
 0 
 0 
 0 
 0 
 0 
 0 
 0 
 0 
 0 
 0 
 0 
 0 
 0 
 0 
 0 
 0 
 0 
 0 
 0 
 0 
 0 
 0 
 0 
 0 
 0 
 0 
 0 
 0 
 0 
 0 
 0 
 0 
 0 
 0 
 0 
 0 
 0 
 0 
 0 
 0 
 0 
 0 
 0 
 0 
 0 
 0 
 0 
 0 
 0 
 0 
 0 
 0 
 0 
 0 
 0 
 0 
 0 
 0 
 0 
 0 
 0 
 0 
 0 
 0 
 0 
 0 
 0 
 0 
 0 
 0 
 0 
 0 
 0 
 0 
 0 
 0 
 0 
 0 
 0 
 0 
 0 
 0 
 0 
 0 
 0 
 0 
 0 
 0 
 0 
 0 
 0 
 0 
 0 
 0 
 0 
 0 
 0 
 0 
 0 
 0 
 0 
 0 
 0 
 0 
 0 
 0 
 0 
 0 
 0 
 0 
 0 
 0 
 0 
 0 
 0 
 0 
 0 
 0 
 0 
 0 
 0 
 0 
 0 
 0 
 0 
 0 
 0 
 0 
 0 
 0 
 0 
 0 
 0 
 0 
 0 
 0 
 0 
 0 
 0 
 0 
 0 
 0 
 0 
 0 
 0 
 0 
 0 
 0 
 0 
 0 
 0 
 0 
 0 
 0 
 0 
 0 
 0 
 0 
 0 
 0 
 0 
 0 
 0 
 0 
 0 
 0 
 0 
 0 
 0 
 0 
 0 
 0 
 0 
 0 
 0 
 0 
 0 
 0 
 0 
 0 
 0 
 0 
 0 
 0 
 0 
 0 
 0 
 0 
 0 
 0 
 0 
 0 
 0 
 0 
 0 
 0 
 0 
 0 
 0 
 0 
 0 
 0 
 0 
 0 
 0 
 0 
 0 
 0 
 0 
 0 
 0 
 0 
 0 
 0 
 0 
 0 
 0 
 0 
 0 
 0 
 0 
 0 
 0 
 0 
 0 
 0 
 0 
 0 
 0 
 0 
 0 
 0 
 0 
 0 
 0 
 0 
 0 
 0 
 0 
 0 
 0 
 0 
 0 
 0 
 0 
 0 
 0 
 0 
 0 
 0 
 0 
 0 
 0 
 0 
 0 
 0 
 0 
 0 
 0 
 0 
 0 
 0 
 0 
 0 
 0 
 0 
 0 
 0 
 0 
 0 
 0 
 0 
 0 
 0 
 0 
 0 
 0 
 0 
 0 
 0 
 0 
 0 
 0 
 0 
 0 
 0 
 0 
 0 
 0 
 0 
 0 
 0 
 0 
 0 
 0 
 0 
 0 
 0 
 0 
 0 
 0 
 0 
 0 
 0 
 0 
 0 
 0 
 0 
 0 
 0 
 0 
 0 
 0 
 0 
 0 
 0 
 0 
 0 
 0 
 0 
 0 
 0 
 0 
 0 
 0 
 0 
 0 
 0 
 0 
 0 
 0 
 0 
 0 
 0 
 0 
 0 
 0 
 0 
 0 
 0 
 0 
 0 
 0 
 0 
 0 
 0 
 0 
 0 
 0 
 0 
 0 
 0 
 0 
 0 
 0 
 0 
 0 
 0 
 0 
 0 
 0 
 0 
 0 
 0 
 0 
 0 
 0 
 0 
 0 
 0 
 0 
 0 
 0 
 0 
 0 
 0 
 0 
 0 
 0 
 0 
 0 
 0 
 0 
 0 
 0 
 0 
 0 
 0 
 0 
 0 
 0 
 0 
 0 
 0 
 0 
 0 
 0 
 0 
 0 
 0 
 0 
 0 
 0 
 0 
 0 
 0 
 0 
 0 
 0 
 0 
 0 
 0 
 0 
 0 
 0 
 0 
 0 
 0 
 0 
 0 
 0 
 0 
 0 
 0 
 0 
 0 
 0 
 0 
 0 
 0 
 0 
 0 
 0 
 0 
 0 
 0 
 0 
 0 
 0 
 0 
 0 
 0 
 0 
 0 
 0 
 0 
 0 
 0 
 0 
 0 
 0 
 0 
 0 
 0 
 0 
 0 
 0 
 0 
 0 
 0 
 0 
 0 
 0 
 0 
 0 
 0 
 0 
 0 
 0 
 0 
 0 
 0 
 0 
 0 
 0 
 0 
 0 
 0 
 0 
 0 
 0 
 0 
 0 
 0 
 0 
 0 
 0 
 0 
 0 
 0 
 0 
 0 
 0 
 0 
 0 
 0 
 0 
 0 
 0 
 0 
 0 
 0 
 0 
 0 
 0 
 0 
 0 
 0 
 0 
 0 
 0 
 0 
 0 
 0 
 0 
 0 
 0 
 0 
 0 
 0 
 
 
 34 
 0 
 0 
 0 
 0 
 0 
 0 
 0 
 0 
 0 
 0 
 0 
 0 
 0 
 0 
 0 
 0 
 0 
 0 
 0 
 0 
 0 
 0 
 0 
 0 
 0 
 0 
 0 
 0 
 0 
 0 
 0 
 0 
 0 
 0 
 10 
 0 
 10 
 0 
 0 
 0 
 0 
 0 
 0 
 0 
 0 
 0 
 0 
 0 
 0 
 0 
 0 
 0 
 0 
 0 
 0 
 0 
 0 
 0 
 0 
 0 
 0 
 0 
 0 
 0 
 0 
 0 
 0 
 0 
 0 
 0 
 0 
 0 
 0 
 0 
 0 
 0 
 0 
 0 
 0 
 0 
 0 
 0 
 0 
 0 
 0 
 0 
 0 
 0 
 0 
 0 
 0 
 0 
 0 
 0 
 0 
 0 
 0 
 0 
 0 
 0 
 0 
 0 
 0 
 0 
 0 
 0 
 0 
 0 
 0 
 0 
 0 
 0 
 0 
 0 
 0 
 0 
 0 
 0 
 0 
 0 
 0 
 0 
 0 
 0 
 0 
 0 
 0 
 0 
 0 
 0 
 0 
 0 
 0 
 0 
 0 
 0 
 0 
 0 
 0 
 0 
 0 
 0 
 0 
 0 
 0 
 0 
 0 
 0 
 0 
 0 
 0 
 0 
 0 
 0 
 0 
 0 
 0 
 0 
 0 
 0 
 0 
 0 
 0 
 0 
 0 
 0 
 0 
 0 
 0 
 0 
 0 
 0 
 0 
 0 
 0 
 0 
 0 
 0 
 0 
 0 
 0 
 0 
 0 
 0 
 0 
 0 
 0 
 0 
 0 
 0 
 0 
 0 
 0 
 0 
 0 
 0 
 0 
 0 
 0 
 0 
 0 
 0 
 0 
 0 
 0 
 0 
 0 
 0 
 0 
 0 
 0 
 0 
 0 
 0 
 0 
 0 
 0 
 0 
 0 
 0 
 0 
 0 
 0 
 0 
 0 
 0 
 0 
 0 
 0 
 0 
 0 
 0 
 0 
 0 
 0 
 0 
 0 
 0 
 0 
 0 
 0 
 0 
 0 
 0 
 0 
 0 
 0 
 0 
 0 
 0 
 0 
 0 
 0 
 0 
 0 
 0 
 0 
 0 
 0 
 0 
 0 
 0 
 0 
 0 
 0 
 0 
 0 
 0 
 0 
 0 
 0 
 0 
 0 
 0 
 0 
 0 
 0 
 0 
 0 
 0 
 0 
 0 
 0 
 0 
 0 
 0 
 0 
 0 
 0 
 0 
 0 
 0 
 0 
 0 
 0 
 0 
 0 
 0 
 0 
 0 
 0 
 0 
 0 
 0 
 0 
 0 
 0 
 0 
 0 
 0 
 0 
 0 
 0 
 0 
 0 
 0 
 0 
 0 
 0 
 0 
 0 
 0 
 0 
 0 
 0 
 0 
 0 
 0 
 0 
 0 
 0 
 0 
 0 
 0 
 0 
 0 
 0 
 0 
 0 
 0 
 0 
 0 
 0 
 0 
 0 
 0 
 0 
 0 
 0 
 0 
 0 
 0 
 0 
 0 
 0 
 0 
 0 
 0 
 0 
 0 
 0 
 0 
 0 
 0 
 0 
 0 
 0 
 0 
 0 
 0 
 0 
 0 
 0 
 0 
 0 
 0 
 0 
 0 
 0 
 0 
 0 
 0 
 0 
 0 
 0 
 0 
 0 
 0 
 0 
 0 
 0 
 0 
 0 
 0 
 0 
 0 
 0 
 0 
 0 
 0 
 0 
 0 
 0 
 0 
 0 
 0 
 0 
 0 
 0 
 0 
 0 
 0 
 0 
 0 
 0 
 0 
 0 
 0 
 0 
 0 
 0 
 0 
 0 
 0 
 0 
 0 
 0 
 0 
 0 
 0 
 0 
 0 
 0 
 0 
 0 
 0 
 0 
 0 
 0 
 0 
 0 
 0 
 0 
 0 
 0 
 0 
 0 
 0 
 0 
 0 
 0 
 0 
 0 
 0 
 0 
 0 
 0 
 0 
 0 
 0 
 0 
 0 
 0 
 0 
 0 
 0 
 0 
 0 
 0 
 0 
 0 
 0 
 0 
 0 
 0 
 0 
 0 
 0 
 0 
 0 
 0 
 0 
 0 
 0 
 0 
 0 
 0 
 0 
 0 
 0 
 0 
 0 
 0 
 0 
 0 
 0 
 0 
 0 
 0 
 0 
 0 
 0 
 0 
 0 
 0 
 0 
 0 
 0 
 0 
 0 
 0 
 0 
 0 
 0 
 0 
 0 
 0 
 0 
 0 
 0 
 0 
 0 
 0 
 0 
 0 
 0 
 0 
 0 
 0 
 0 
 0 
 0 
 0 
 0 
 0 
 0 
 0 
 0 
 0 
 0 
 0 
 0 
 0 
 0 
 0 
 0 
 0 
 0 
 0 
 0 
 0 
 0 
 0 
 0 
 0 
 0 
 0 
 0 
 0 
 0 
 0 
 0 
 0 
 0 
 0 
 0 
 0 
 0 
 0 
 0 
 0 
 0 
 0 
 0 
 0 
 0 
 0 
 0 
 0 
 0 
 0 
 0 
 0 
 0 
 0 
 0 
 0 
 0 
 0 
 0 
 0 
 0 
 0 
 0 
 0 
 0 
 0 
 0 
 0 
 0 
 0 
 0 
 0 
 0 
 0 
 0 
 0 
 0 
 0 
 0 
 0 
 0 
 0 
 0 
 0 
 0 
 0 
 0 
 0 
 0 
 0 
 0 
 0 
 0 
 0 
 0 
 0 
 0 
 0 
 0 
 0 
 0 
 0 
 0 
 0 
 0 
 0 
 0 
 0 
 0 
 0 
 0 
 0 
 0 
 0 
 0 
 0 
 0 
 0 
 0 
 0 
 0 
 0 
 0 
 0 
 0 
 0 
 0 
 0 
 0 
 0 
 0 
 0 
 0 
 0 
 0 
 0 
 0 
 0 
 0 
 0 
 0 
 0 
 0 
 0 
 0 
 0 
 0 
 0 
 0 
 0 
 0 
 0 
 0 
 0 
 0 
 0 
 0 
 0 
 0 
 0 
 0 
 0 
 0 
 0 
 0 
 0 
 0 
 0 
 0 
 0 
 0 
 0 
 0 
 0 
 0 
 0 
 0 
 0 
 0 
 0 
 0 
 0 
 0 
 0 
 0 
 0 
 0 
 0 
 0 
 0 
 0 
 0 
 0 
 0 
 0 
 0 
 0 
 0 
 0 
 0 
 0 
 0 
 0 
 0 
 0 
 0 
 0 
 0 
 0 
 0 
 0 
 0 
 0 
 0 
 0 
 0 
 0 
 0 
 0 
 0 
 0 
 0 
 0 
 0 
 0 
 0 
 0 
 0 
 0 
 0 
 0 
 0 
 0 
 0 
 0 
 0 
 0 
 0 
 0 
 0 
 0 
 0 
 0 
 0 
 0 
 0 
 0 
 0 
 0 
 0 
 0 
 0 
 0 
 0 
 0 
 0 
 0 
 0 
 0 
 0 
 0 
 0 
 0 
 0 
 0 
 0 
 0 
 0 
 0 
 0 
 0 
 0 
 0 
 0 
 0 
 0 
 0 
 0 
 0 
 0 
 0 
 0 
 0 
 0 
 0 
 0 
 0 
 0 
 0 
 0 
 0 
 0 
 0 
 0 
 0 
 
 
 35 
 0 
 0 
 0 
 0 
 0 
 0 
 0 
 0 
 0 
 0 
 0 
 0 
 0 
 0 
 0 
 0 
 0 
 0 
 0 
 0 
 0 
 0 
 0 
 0 
 0 
 0 
 0 
 0 
 0 
 0 
 0 
 0 
 0 
 0 
 0 
 8 
 0 
 0 
 3 
 0 
 0 
 0 
 0 
 0 
 0 
 0 
 0 
 0 
 0 
 0 
 0 
 0 
 0 
 0 
 0 
 0 
 0 
 0 
 0 
 0 
 0 
 0 
 0 
 0 
 0 
 0 
 0 
 0 
 0 
 0 
 0 
 0 
 0 
 0 
 0 
 0 
 0 
 0 
 0 
 0 
 0 
 0 
 0 
 0 
 0 
 0 
 0 
 0 
 0 
 0 
 0 
 0 
 0 
 0 
 0 
 0 
 0 
 0 
 0 
 0 
 0 
 0 
 0 
 0 
 0 
 0 
 0 
 0 
 0 
 0 
 0 
 0 
 0 
 0 
 0 
 0 
 0 
 0 
 0 
 0 
 0 
 0 
 0 
 0 
 0 
 0 
 0 
 0 
 0 
 0 
 0 
 0 
 0 
 0 
 0 
 0 
 0 
 0 
 0 
 0 
 0 
 0 
 0 
 0 
 0 
 0 
 0 
 0 
 0 
 0 
 0 
 0 
 0 
 0 
 0 
 0 
 0 
 0 
 0 
 0 
 0 
 0 
 0 
 0 
 0 
 0 
 0 
 0 
 0 
 0 
 0 
 0 
 0 
 0 
 0 
 0 
 0 
 0 
 0 
 0 
 0 
 0 
 0 
 0 
 0 
 0 
 0 
 0 
 0 
 0 
 0 
 0 
 0 
 0 
 0 
 0 
 0 
 0 
 0 
 0 
 0 
 0 
 0 
 0 
 0 
 0 
 0 
 0 
 0 
 0 
 0 
 0 
 0 
 0 
 0 
 0 
 0 
 0 
 0 
 0 
 0 
 0 
 0 
 0 
 0 
 0 
 0 
 0 
 0 
 0 
 0 
 0 
 0 
 0 
 0 
 0 
 0 
 0 
 0 
 0 
 0 
 0 
 0 
 0 
 0 
 0 
 0 
 0 
 0 
 0 
 0 
 0 
 0 
 0 
 0 
 0 
 0 
 0 
 0 
 0 
 0 
 0 
 0 
 0 
 0 
 0 
 0 
 0 
 0 
 0 
 0 
 0 
 0 
 0 
 0 
 0 
 0 
 0 
 0 
 0 
 0 
 0 
 0 
 0 
 0 
 0 
 0 
 0 
 0 
 0 
 0 
 0 
 0 
 0 
 0 
 0 
 0 
 0 
 0 
 0 
 0 
 0 
 0 
 0 
 0 
 0 
 0 
 0 
 0 
 0 
 0 
 0 
 0 
 0 
 0 
 0 
 0 
 0 
 0 
 0 
 0 
 0 
 0 
 0 
 0 
 0 
 0 
 0 
 0 
 0 
 0 
 0 
 0 
 0 
 0 
 0 
 0 
 0 
 0 
 0 
 0 
 0 
 0 
 0 
 0 
 0 
 0 
 0 
 0 
 0 
 0 
 0 
 0 
 0 
 0 
 0 
 0 
 0 
 0 
 0 
 0 
 0 
 0 
 0 
 0 
 0 
 0 
 0 
 0 
 0 
 0 
 0 
 0 
 0 
 0 
 0 
 0 
 0 
 0 
 0 
 0 
 0 
 0 
 0 
 0 
 0 
 0 
 0 
 0 
 0 
 0 
 0 
 0 
 0 
 0 
 0 
 0 
 0 
 0 
 0 
 0 
 0 
 0 
 0 
 0 
 0 
 0 
 0 
 0 
 0 
 0 
 0 
 0 
 0 
 0 
 0 
 0 
 0 
 0 
 0 
 0 
 0 
 0 
 0 
 0 
 0 
 0 
 0 
 0 
 0 
 0 
 0 
 0 
 0 
 0 
 0 
 0 
 0 
 0 
 0 
 0 
 0 
 0 
 0 
 0 
 0 
 0 
 0 
 0 
 0 
 0 
 0 
 0 
 0 
 0 
 0 
 0 
 0 
 0 
 0 
 0 
 0 
 0 
 0 
 0 
 0 
 0 
 0 
 0 
 0 
 0 
 0 
 0 
 0 
 0 
 0 
 0 
 0 
 0 
 0 
 0 
 0 
 0 
 0 
 0 
 0 
 0 
 0 
 0 
 0 
 0 
 0 
 0 
 0 
 0 
 0 
 0 
 0 
 0 
 0 
 0 
 0 
 0 
 0 
 0 
 0 
 0 
 0 
 0 
 0 
 0 
 0 
 0 
 0 
 0 
 0 
 0 
 0 
 0 
 0 
 0 
 0 
 0 
 0 
 0 
 0 
 0 
 0 
 0 
 0 
 0 
 0 
 0 
 0 
 0 
 0 
 0 
 0 
 0 
 0 
 0 
 0 
 0 
 0 
 0 
 0 
 0 
 0 
 0 
 0 
 0 
 0 
 0 
 0 
 0 
 0 
 0 
 0 
 0 
 0 
 0 
 0 
 0 
 0 
 0 
 0 
 0 
 0 
 0 
 0 
 0 
 0 
 0 
 0 
 0 
 0 
 0 
 0 
 0 
 0 
 0 
 0 
 0 
 0 
 0 
 0 
 0 
 0 
 0 
 0 
 0 
 0 
 0 
 0 
 0 
 0 
 0 
 0 
 0 
 0 
 0 
 0 
 0 
 0 
 0 
 0 
 0 
 0 
 0 
 0 
 0 
 0 
 0 
 0 
 0 
 0 
 0 
 0 
 0 
 0 
 0 
 0 
 0 
 0 
 0 
 0 
 0 
 0 
 0 
 0 
 0 
 0 
 0 
 0 
 0 
 0 
 0 
 0 
 0 
 0 
 0 
 0 
 0 
 0 
 0 
 0 
 0 
 0 
 0 
 0 
 0 
 0 
 0 
 0 
 0 
 0 
 0 
 0 
 0 
 0 
 0 
 0 
 0 
 0 
 0 
 0 
 0 
 0 
 0 
 0 
 0 
 0 
 0 
 0 
 0 
 0 
 0 
 0 
 0 
 0 
 0 
 0 
 0 
 0 
 0 
 0 
 0 
 0 
 0 
 0 
 0 
 0 
 0 
 0 
 0 
 0 
 0 
 0 
 0 
 0 
 0 
 0 
 0 
 0 
 0 
 0 
 0 
 0 
 0 
 0 
 0 
 0 
 0 
 0 
 0 
 0 
 0 
 0 
 0 
 0 
 0 
 0 
 0 
 0 
 0 
 0 
 0 
 0 
 0 
 0 
 0 
 0 
 0 
 0 
 0 
 0 
 0 
 0 
 0 
 0 
 0 
 0 
 0 
 0 
 0 
 0 
 0 
 0 
 0 
 0 
 0 
 0 
 0 
 0 
 0 
 0 
 0 
 0 
 0 
 0 
 0 
 0 
 0 
 0 
 0 
 0 
 0 
 0 
 0 
 0 
 0 
 0 
 0 
 0 
 0 
 0 
 0 
 0 
 0 
 0 
 0 
 0 
 0 
 0 
 0 
 0 
 0 
 0 
 0 
 0 
 0 
 0 
 0 
 0 
 0 
 0 
 0 
 0 
 0 
 0 
 0 
 0 
 0 
 0 
 0 
 0 
 0 
 0 
 0 
 0 
 0 
 0 
 0 
 0 
 0 
 0 
 0 
 0 
 0 
 0 
 0 
 0 
 0 
 0 
 0 
 0 
 
 
 36 
 0 
 0 
 0 
 0 
 0 
 0 
 0 
 0 
 0 
 0 
 0 
 0 
 0 
 0 
 0 
 0 
 0 
 0 
 0 
 0 
 0 
 0 
 0 
 0 
 0 
 0 
 0 
 0 
 0 
 0 
 0 
 0 
 0 
 0 
 0 
 0 
 7 
 0 
 0 
 0 
 0 
 0 
 0 
 0 
 0 
 0 
 0 
 0 
 0 
 0 
 0 
 0 
 0 
 0 
 0 
 0 
 0 
 0 
 0 
 0 
 0 
 0 
 0 
 0 
 0 
 0 
 0 
 0 
 0 
 0 
 0 
 0 
 0 
 0 
 0 
 0 
 0 
 0 
 0 
 0 
 0 
 0 
 0 
 0 
 0 
 0 
 0 
 0 
 0 
 0 
 0 
 0 
 0 
 0 
 0 
 0 
 0 
 0 
 0 
 0 
 0 
 0 
 0 
 0 
 0 
 0 
 0 
 0 
 0 
 0 
 0 
 0 
 0 
 0 
 0 
 0 
 0 
 0 
 0 
 0 
 0 
 0 
 0 
 0 
 0 
 0 
 0 
 0 
 0 
 0 
 0 
 0 
 0 
 0 
 0 
 0 
 0 
 0 
 0 
 0 
 0 
 0 
 0 
 0 
 0 
 0 
 0 
 0 
 0 
 0 
 0 
 0 
 0 
 0 
 0 
 0 
 0 
 0 
 0 
 0 
 0 
 0 
 0 
 0 
 0 
 0 
 0 
 0 
 0 
 0 
 0 
 0 
 0 
 0 
 0 
 0 
 0 
 0 
 0 
 0 
 0 
 0 
 0 
 0 
 0 
 0 
 0 
 0 
 0 
 0 
 0 
 0 
 0 
 0 
 0 
 0 
 0 
 0 
 0 
 0 
 0 
 0 
 0 
 0 
 0 
 0 
 0 
 0 
 0 
 0 
 0 
 0 
 0 
 0 
 0 
 0 
 0 
 0 
 0 
 0 
 0 
 0 
 0 
 0 
 0 
 0 
 0 
 0 
 0 
 0 
 0 
 0 
 0 
 0 
 0 
 0 
 0 
 0 
 0 
 0 
 0 
 0 
 0 
 0 
 0 
 0 
 0 
 0 
 0 
 0 
 0 
 0 
 0 
 0 
 0 
 0 
 0 
 0 
 0 
 0 
 0 
 0 
 0 
 0 
 0 
 0 
 0 
 0 
 0 
 0 
 0 
 0 
 0 
 0 
 0 
 0 
 0 
 0 
 0 
 0 
 0 
 0 
 0 
 0 
 0 
 0 
 0 
 0 
 0 
 0 
 0 
 0 
 0 
 0 
 0 
 0 
 0 
 0 
 0 
 0 
 0 
 0 
 0 
 0 
 0 
 0 
 0 
 0 
 0 
 0 
 0 
 0 
 0 
 0 
 0 
 0 
 0 
 0 
 0 
 0 
 0 
 0 
 0 
 0 
 0 
 0 
 0 
 0 
 0 
 0 
 0 
 0 
 0 
 0 
 0 
 0 
 0 
 0 
 0 
 0 
 0 
 0 
 0 
 0 
 0 
 0 
 0 
 0 
 0 
 0 
 0 
 0 
 0 
 0 
 0 
 0 
 0 
 0 
 0 
 0 
 0 
 0 
 0 
 0 
 0 
 0 
 0 
 0 
 0 
 0 
 0 
 0 
 0 
 0 
 0 
 0 
 0 
 0 
 0 
 0 
 0 
 0 
 0 
 0 
 0 
 0 
 0 
 0 
 0 
 0 
 0 
 0 
 0 
 0 
 0 
 0 
 0 
 0 
 0 
 0 
 0 
 0 
 0 
 0 
 0 
 0 
 0 
 0 
 0 
 0 
 0 
 0 
 0 
 0 
 0 
 0 
 0 
 0 
 0 
 0 
 0 
 0 
 0 
 0 
 0 
 0 
 0 
 0 
 0 
 0 
 0 
 0 
 0 
 0 
 0 
 0 
 0 
 0 
 0 
 0 
 0 
 0 
 0 
 0 
 0 
 0 
 0 
 0 
 0 
 0 
 0 
 0 
 0 
 0 
 0 
 0 
 0 
 0 
 0 
 0 
 0 
 0 
 0 
 0 
 0 
 0 
 0 
 0 
 0 
 0 
 0 
 0 
 0 
 0 
 0 
 0 
 0 
 0 
 0 
 0 
 0 
 0 
 0 
 0 
 0 
 0 
 0 
 0 
 0 
 0 
 0 
 0 
 0 
 0 
 0 
 0 
 0 
 0 
 0 
 0 
 0 
 0 
 0 
 0 
 0 
 0 
 0 
 0 
 0 
 0 
 0 
 0 
 0 
 0 
 0 
 0 
 0 
 0 
 0 
 0 
 0 
 0 
 0 
 0 
 0 
 0 
 0 
 0 
 0 
 0 
 0 
 0 
 0 
 0 
 0 
 0 
 0 
 0 
 0 
 0 
 0 
 0 
 0 
 0 
 0 
 0 
 0 
 0 
 0 
 0 
 0 
 0 
 0 
 0 
 0 
 0 
 0 
 0 
 0 
 0 
 0 
 0 
 0 
 0 
 0 
 0 
 0 
 0 
 0 
 0 
 0 
 0 
 0 
 0 
 0 
 0 
 0 
 0 
 0 
 0 
 0 
 0 
 0 
 0 
 0 
 0 
 0 
 0 
 0 
 0 
 0 
 0 
 0 
 0 
 0 
 0 
 0 
 0 
 0 
 0 
 0 
 0 
 0 
 0 
 0 
 0 
 0 
 0 
 0 
 0 
 0 
 0 
 0 
 0 
 0 
 0 
 0 
 0 
 0 
 0 
 0 
 0 
 0 
 0 
 0 
 0 
 0 
 0 
 0 
 0 
 0 
 0 
 0 
 0 
 0 
 0 
 0 
 0 
 0 
 0 
 0 
 0 
 0 
 0 
 0 
 0 
 0 
 0 
 0 
 0 
 0 
 0 
 0 
 0 
 0 
 0 
 0 
 0 
 0 
 0 
 0 
 0 
 0 
 0 
 0 
 0 
 0 
 0 
 0 
 0 
 0 
 0 
 0 
 0 
 0 
 0 
 0 
 0 
 0 
 0 
 0 
 0 
 0 
 0 
 0 
 0 
 0 
 0 
 0 
 0 
 0 
 0 
 0 
 0 
 0 
 0 
 0 
 0 
 0 
 0 
 0 
 0 
 0 
 0 
 0 
 0 
 0 
 0 
 0 
 0 
 0 
 0 
 0 
 0 
 0 
 0 
 0 
 0 
 0 
 0 
 0 
 0 
 0 
 0 
 0 
 0 
 0 
 0 
 0 
 0 
 0 
 0 
 0 
 0 
 0 
 0 
 0 
 0 
 0 
 0 
 0 
 0 
 0 
 0 
 0 
 0 
 0 
 0 
 0 
 0 
 0 
 0 
 0 
 0 
 0 
 0 
 0 
 0 
 0 
 0 
 0 
 0 
 0 
 0 
 0 
 0 
 0 
 0 
 0 
 0 
 0 
 0 
 0 
 0 
 0 
 0 
 0 
 0 
 0 
 0 
 0 
 0 
 0 
 0 
 0 
 0 
 0 
 0 
 0 
 0 
 0 
 0 
 0 
 0 
 0 
 0 
 0 
 0 
 0 
 0 
 0 
 0 
 0 
 0 
 0 
 0 
 0 
 0 
 0 
 0 
 0 
 0 
 0 
 0 
 0 
 0 
 0 
 0 
 0 
 0 
 0 
 0 
 0 
 0 
 0 
 0 
 
 
 37 
 0 
 0 
 0 
 0 
 0 
 0 
 0 
 0 
 0 
 0 
 0 
 0 
 0 
 0 
 0 
 0 
 0 
 0 
 0 
 0 
 0 
 0 
 0 
 0 
 0 
 0 
 0 
 0 
 0 
 0 
 0 
 0 
 0 
 0 
 0 
 0 
 0 
 27 
 0 
 0 
 0 
 0 
 0 
 0 
 0 
 0 
 0 
 0 
 0 
 0 
 0 
 0 
 0 
 0 
 0 
 0 
 0 
 0 
 0 
 0 
 0 
 0 
 0 
 0 
 0 
 0 
 0 
 0 
 0 
 0 
 0 
 0 
 0 
 0 
 0 
 0 
 0 
 0 
 0 
 0 
 0 
 0 
 0 
 0 
 0 
 0 
 0 
 0 
 0 
 0 
 0 
 0 
 0 
 0 
 0 
 0 
 0 
 0 
 0 
 0 
 0 
 0 
 0 
 0 
 0 
 0 
 0 
 0 
 0 
 0 
 0 
 0 
 0 
 0 
 0 
 0 
 0 
 0 
 0 
 0 
 0 
 0 
 0 
 0 
 0 
 0 
 0 
 0 
 0 
 0 
 0 
 0 
 0 
 0 
 0 
 0 
 0 
 0 
 0 
 0 
 0 
 0 
 0 
 0 
 0 
 0 
 0 
 0 
 0 
 0 
 0 
 0 
 0 
 0 
 0 
 0 
 0 
 0 
 0 
 0 
 0 
 0 
 0 
 0 
 0 
 0 
 0 
 0 
 0 
 0 
 0 
 0 
 0 
 0 
 0 
 0 
 0 
 0 
 0 
 0 
 0 
 0 
 0 
 0 
 0 
 0 
 0 
 0 
 0 
 0 
 0 
 0 
 0 
 0 
 0 
 0 
 0 
 0 
 0 
 0 
 0 
 0 
 0 
 0 
 0 
 0 
 0 
 0 
 0 
 0 
 0 
 0 
 0 
 0 
 0 
 0 
 0 
 0 
 0 
 0 
 0 
 0 
 0 
 0 
 0 
 0 
 0 
 0 
 0 
 0 
 0 
 0 
 0 
 0 
 0 
 0 
 0 
 0 
 0 
 0 
 0 
 0 
 0 
 0 
 0 
 0 
 0 
 0 
 0 
 0 
 0 
 0 
 0 
 0 
 0 
 0 
 0 
 0 
 0 
 0 
 0 
 0 
 0 
 0 
 0 
 0 
 0 
 0 
 0 
 0 
 0 
 0 
 0 
 0 
 0 
 0 
 0 
 0 
 0 
 0 
 0 
 0 
 0 
 0 
 0 
 0 
 0 
 0 
 0 
 0 
 0 
 0 
 0 
 0 
 0 
 0 
 0 
 0 
 0 
 0 
 0 
 0 
 0 
 0 
 0 
 0 
 0 
 0 
 0 
 0 
 0 
 0 
 0 
 0 
 0 
 0 
 0 
 0 
 0 
 0 
 0 
 0 
 0 
 0 
 0 
 0 
 0 
 0 
 0 
 0 
 0 
 0 
 0 
 0 
 0 
 0 
 0 
 0 
 0 
 0 
 0 
 0 
 0 
 0 
 0 
 0 
 0 
 0 
 0 
 0 
 0 
 0 
 0 
 0 
 0 
 0 
 0 
 0 
 0 
 0 
 0 
 0 
 0 
 0 
 0 
 0 
 0 
 0 
 0 
 0 
 0 
 0 
 0 
 0 
 0 
 0 
 0 
 0 
 0 
 0 
 0 
 0 
 0 
 0 
 0 
 0 
 0 
 0 
 0 
 0 
 0 
 0 
 0 
 0 
 0 
 0 
 0 
 0 
 0 
 0 
 0 
 0 
 0 
 0 
 0 
 0 
 0 
 0 
 0 
 0 
 0 
 0 
 0 
 0 
 0 
 0 
 0 
 0 
 0 
 0 
 0 
 0 
 0 
 0 
 0 
 0 
 0 
 0 
 0 
 0 
 0 
 0 
 0 
 0 
 0 
 0 
 0 
 0 
 0 
 0 
 0 
 0 
 0 
 0 
 0 
 0 
 0 
 0 
 0 
 0 
 0 
 0 
 0 
 0 
 0 
 0 
 0 
 0 
 0 
 0 
 0 
 0 
 0 
 0 
 0 
 0 
 0 
 0 
 0 
 0 
 0 
 0 
 0 
 0 
 0 
 0 
 0 
 0 
 0 
 0 
 0 
 0 
 0 
 0 
 0 
 0 
 0 
 0 
 0 
 0 
 0 
 0 
 0 
 0 
 0 
 0 
 0 
 0 
 0 
 0 
 0 
 0 
 0 
 0 
 0 
 0 
 0 
 0 
 0 
 0 
 0 
 0 
 0 
 0 
 0 
 0 
 0 
 0 
 0 
 0 
 0 
 0 
 0 
 0 
 0 
 0 
 0 
 0 
 0 
 0 
 0 
 0 
 0 
 0 
 0 
 0 
 0 
 0 
 0 
 0 
 0 
 0 
 0 
 0 
 0 
 0 
 0 
 0 
 0 
 0 
 0 
 0 
 0 
 0 
 0 
 0 
 0 
 0 
 0 
 0 
 0 
 0 
 0 
 0 
 0 
 0 
 0 
 0 
 0 
 0 
 0 
 0 
 0 
 0 
 0 
 0 
 0 
 0 
 0 
 0 
 0 
 0 
 0 
 0 
 0 
 0 
 0 
 0 
 0 
 0 
 0 
 0 
 0 
 0 
 0 
 0 
 0 
 0 
 0 
 0 
 0 
 0 
 0 
 0 
 0 
 0 
 0 
 0 
 0 
 0 
 0 
 0 
 0 
 0 
 0 
 0 
 0 
 0 
 0 
 0 
 0 
 0 
 0 
 0 
 0 
 0 
 0 
 0 
 0 
 0 
 0 
 0 
 0 
 0 
 0 
 0 
 0 
 0 
 0 
 0 
 0 
 0 
 0 
 0 
 0 
 0 
 0 
 0 
 0 
 0 
 0 
 0 
 0 
 0 
 0 
 0 
 0 
 0 
 0 
 0 
 0 
 0 
 0 
 0 
 0 
 0 
 0 
 0 
 0 
 0 
 0 
 0 
 0 
 0 
 0 
 0 
 0 
 0 
 0 
 0 
 0 
 0 
 0 
 0 
 0 
 0 
 0 
 0 
 0 
 0 
 0 
 0 
 0 
 0 
 0 
 0 
 0 
 0 
 0 
 0 
 0 
 0 
 0 
 0 
 0 
 0 
 0 
 0 
 0 
 0 
 0 
 0 
 0 
 0 
 0 
 0 
 0 
 0 
 0 
 0 
 0 
 0 
 0 
 0 
 0 
 0 
 0 
 0 
 0 
 0 
 0 
 0 
 0 
 0 
 0 
 0 
 0 
 0 
 0 
 0 
 0 
 0 
 0 
 0 
 0 
 0 
 0 
 0 
 0 
 0 
 0 
 0 
 0 
 0 
 0 
 0 
 0 
 0 
 0 
 0 
 0 
 0 
 0 
 0 
 0 
 0 
 0 
 0 
 0 
 0 
 0 
 0 
 0 
 0 
 0 
 0 
 0 
 0 
 0 
 0 
 0 
 0 
 0 
 0 
 0 
 0 
 0 
 0 
 0 
 0 
 0 
 0 
 0 
 0 
 0 
 0 
 0 
 0 
 0 
 0 
 0 
 0 
 0 
 0 
 0 
 0 
 0 
 0 
 0 
 0 
 0 
 0 
 0 
 0 
 0 
 0 
 0 
 0 
 0 
 0 
 0 
 0 
 0 
 0 
 0 
 0 
 
 
 38 
 0 
 0 
 0 
 0 
 0 
 0 
 0 
 0 
 0 
 0 
 0 
 0 
 0 
 0 
 0 
 0 
 0 
 0 
 0 
 0 
 0 
 0 
 0 
 0 
 0 
 0 
 0 
 0 
 0 
 0 
 0 
 0 
 0 
 0 
 0 
 0 
 0 
 0 
 0 
 0 
 0 
 0 
 0 
 0 
 0 
 0 
 0 
 0 
 0 
 0 
 0 
 0 
 0 
 0 
 0 
 0 
 0 
 0 
 0 
 0 
 0 
 0 
 0 
 0 
 0 
 0 
 0 
 0 
 0 
 0 
 0 
 0 
 0 
 0 
 0 
 0 
 0 
 0 
 0 
 0 
 0 
 0 
 0 
 0 
 0 
 0 
 0 
 0 
 0 
 0 
 0 
 0 
 0 
 0 
 0 
 0 
 0 
 0 
 0 
 0 
 0 
 0 
 0 
 0 
 0 
 0 
 0 
 0 
 0 
 0 
 0 
 0 
 0 
 0 
 0 
 3 
 1 
 0 
 0 
 0 
 0 
 0 
 0 
 0 
 0 
 0 
 0 
 0 
 0 
 0 
 3 
 0 
 0 
 0 
 0 
 0 
 0 
 0 
 0 
 0 
 0 
 0 
 0 
 0 
 0 
 0 
 0 
 0 
 0 
 0 
 0 
 0 
 0 
 0 
 0 
 0 
 0 
 0 
 0 
 0 
 0 
 0 
 0 
 0 
 0 
 0 
 0 
 0 
 0 
 0 
 0 
 0 
 0 
 0 
 0 
 0 
 0 
 0 
 0 
 0 
 0 
 0 
 0 
 0 
 0 
 0 
 0 
 0 
 0 
 0 
 0 
 0 
 0 
 0 
 0 
 0 
 0 
 0 
 0 
 0 
 0 
 0 
 0 
 0 
 0 
 0 
 0 
 0 
 0 
 0 
 0 
 0 
 0 
 0 
 0 
 0 
 0 
 0 
 0 
 0 
 0 
 0 
 0 
 0 
 0 
 0 
 0 
 0 
 0 
 0 
 0 
 0 
 0 
 0 
 0 
 0 
 0 
 0 
 0 
 0 
 0 
 0 
 0 
 0 
 0 
 0 
 0 
 0 
 0 
 0 
 0 
 0 
 0 
 0 
 0 
 0 
 0 
 0 
 0 
 0 
 0 
 0 
 0 
 0 
 0 
 0 
 0 
 0 
 0 
 0 
 0 
 0 
 0 
 0 
 0 
 0 
 0 
 0 
 0 
 0 
 0 
 0 
 0 
 0 
 0 
 0 
 0 
 0 
 0 
 0 
 0 
 0 
 0 
 0 
 0 
 0 
 0 
 0 
 0 
 0 
 0 
 0 
 0 
 0 
 0 
 0 
 0 
 0 
 0 
 0 
 0 
 0 
 0 
 0 
 0 
 0 
 0 
 0 
 0 
 0 
 0 
 0 
 0 
 0 
 0 
 0 
 0 
 0 
 0 
 0 
 0 
 0 
 0 
 0 
 0 
 0 
 0 
 0 
 0 
 0 
 0 
 0 
 0 
 0 
 0 
 0 
 0 
 0 
 0 
 0 
 0 
 0 
 0 
 0 
 0 
 0 
 0 
 0 
 0 
 0 
 0 
 0 
 0 
 0 
 0 
 0 
 0 
 0 
 0 
 0 
 0 
 0 
 0 
 0 
 0 
 0 
 0 
 0 
 0 
 0 
 0 
 0 
 0 
 0 
 0 
 0 
 0 
 0 
 0 
 0 
 0 
 0 
 0 
 0 
 0 
 0 
 0 
 0 
 0 
 0 
 0 
 0 
 0 
 0 
 0 
 0 
 0 
 0 
 0 
 0 
 0 
 0 
 0 
 0 
 0 
 0 
 0 
 0 
 0 
 0 
 0 
 0 
 0 
 0 
 0 
 0 
 0 
 0 
 0 
 0 
 0 
 0 
 0 
 0 
 0 
 0 
 0 
 0 
 0 
 0 
 0 
 0 
 0 
 0 
 0 
 0 
 0 
 0 
 0 
 0 
 0 
 0 
 0 
 0 
 0 
 0 
 0 
 0 
 0 
 0 
 0 
 0 
 0 
 0 
 0 
 0 
 0 
 0 
 0 
 0 
 0 
 0 
 0 
 0 
 0 
 0 
 0 
 0 
 0 
 0 
 0 
 0 
 0 
 0 
 0 
 0 
 0 
 0 
 0 
 0 
 0 
 0 
 0 
 0 
 0 
 0 
 0 
 0 
 0 
 0 
 0 
 0 
 0 
 0 
 0 
 0 
 0 
 0 
 0 
 0 
 0 
 0 
 0 
 0 
 0 
 0 
 0 
 0 
 0 
 0 
 0 
 0 
 0 
 0 
 0 
 0 
 0 
 0 
 0 
 0 
 0 
 0 
 0 
 0 
 0 
 0 
 0 
 0 
 0 
 0 
 0 
 0 
 0 
 0 
 0 
 0 
 0 
 0 
 0 
 0 
 0 
 0 
 0 
 0 
 0 
 0 
 0 
 0 
 0 
 0 
 0 
 0 
 0 
 0 
 0 
 0 
 0 
 0 
 0 
 0 
 0 
 0 
 0 
 0 
 0 
 0 
 0 
 0 
 0 
 0 
 0 
 0 
 0 
 0 
 0 
 0 
 0 
 0 
 0 
 0 
 0 
 0 
 0 
 0 
 0 
 0 
 0 
 0 
 0 
 0 
 0 
 0 
 0 
 0 
 0 
 0 
 0 
 0 
 0 
 0 
 0 
 0 
 0 
 0 
 0 
 0 
 0 
 0 
 0 
 0 
 0 
 0 
 0 
 0 
 0 
 0 
 0 
 0 
 0 
 0 
 0 
 0 
 0 
 0 
 0 
 0 
 0 
 0 
 0 
 0 
 0 
 0 
 0 
 0 
 0 
 0 
 0 
 0 
 0 
 0 
 0 
 0 
 0 
 0 
 0 
 0 
 0 
 0 
 0 
 0 
 0 
 0 
 0 
 0 
 0 
 0 
 0 
 0 
 0 
 0 
 0 
 0 
 0 
 0 
 0 
 0 
 0 
 0 
 0 
 0 
 0 
 0 
 0 
 0 
 0 
 0 
 0 
 0 
 0 
 0 
 0 
 0 
 0 
 0 
 0 
 0 
 0 
 0 
 0 
 0 
 0 
 0 
 0 
 0 
 0 
 0 
 0 
 0 
 0 
 0 
 0 
 0 
 0 
 0 
 0 
 0 
 0 
 0 
 0 
 0 
 0 
 10 
 0 
 0 
 0 
 0 
 0 
 0 
 0 
 0 
 0 
 0 
 0 
 0 
 0 
 0 
 0 
 0 
 0 
 0 
 0 
 0 
 0 
 0 
 0 
 0 
 0 
 0 
 0 
 0 
 0 
 0 
 0 
 0 
 0 
 0 
 0 
 0 
 0 
 0 
 0 
 0 
 0 
 0 
 0 
 0 
 0 
 0 
 0 
 0 
 0 
 0 
 0 
 0 
 0 
 0 
 0 
 0 
 0 
 0 
 0 
 0 
 0 
 0 
 0 
 0 
 0 
 0 
 0 
 0 
 0 
 0 
 0 
 0 
 0 
 0 
 0 
 0 
 0 
 0 
 0 
 0 
 0 
 0 
 3 
 0 
 0 
 0 
 0 
 0 
 0 
 0 
 0 
 0 
 0 
 0 
 0 
 0 
 0 
 0 
 0 
 0 
 0 
 0 
 0 
 0 
 
 
 39 
 0 
 0 
 0 
 0 
 0 
 0 
 0 
 0 
 0 
 0 
 0 
 0 
 0 
 0 
 0 
 0 
 0 
 0 
 0 
 0 
 0 
 0 
 0 
 0 
 0 
 0 
 0 
 0 
 0 
 0 
 0 
 0 
 0 
 0 
 0 
 3 
 0 
 0 
 1 
 18 
 0 
 0 
 0 
 0 
 0 
 0 
 0 
 0 
 0 
 0 
 0 
 0 
 0 
 0 
 0 
 0 
 0 
 0 
 0 
 0 
 0 
 0 
 0 
 0 
 0 
 0 
 0 
 0 
 0 
 0 
 0 
 0 
 0 
 0 
 0 
 0 
 0 
 0 
 0 
 0 
 0 
 0 
 0 
 0 
 0 
 0 
 0 
 0 
 0 
 0 
 0 
 0 
 0 
 0 
 0 
 0 
 0 
 0 
 0 
 0 
 0 
 0 
 0 
 0 
 0 
 0 
 0 
 0 
 0 
 0 
 0 
 0 
 0 
 0 
 0 
 0 
 0 
 0 
 0 
 0 
 0 
 0 
 0 
 0 
 0 
 0 
 0 
 0 
 0 
 0 
 0 
 0 
 0 
 0 
 0 
 0 
 0 
 0 
 0 
 0 
 0 
 0 
 0 
 0 
 0 
 0 
 0 
 0 
 0 
 0 
 0 
 0 
 0 
 0 
 0 
 0 
 0 
 0 
 0 
 0 
 0 
 0 
 0 
 0 
 0 
 0 
 0 
 0 
 0 
 0 
 0 
 0 
 0 
 0 
 0 
 0 
 0 
 0 
 0 
 0 
 0 
 0 
 0 
 0 
 0 
 0 
 0 
 0 
 0 
 0 
 0 
 0 
 0 
 0 
 0 
 0 
 0 
 0 
 0 
 0 
 0 
 0 
 0 
 0 
 0 
 0 
 0 
 0 
 0 
 0 
 0 
 0 
 0 
 0 
 0 
 0 
 0 
 0 
 0 
 0 
 0 
 0 
 0 
 0 
 0 
 0 
 0 
 0 
 0 
 0 
 0 
 0 
 0 
 0 
 0 
 0 
 0 
 0 
 0 
 0 
 0 
 0 
 0 
 0 
 0 
 0 
 0 
 0 
 0 
 0 
 0 
 0 
 0 
 0 
 0 
 0 
 0 
 0 
 0 
 0 
 0 
 0 
 0 
 0 
 0 
 0 
 0 
 0 
 0 
 0 
 0 
 0 
 0 
 0 
 0 
 0 
 0 
 0 
 0 
 0 
 0 
 0 
 0 
 0 
 0 
 0 
 0 
 0 
 0 
 0 
 0 
 0 
 0 
 0 
 0 
 0 
 0 
 0 
 0 
 0 
 0 
 0 
 0 
 0 
 0 
 0 
 0 
 0 
 0 
 0 
 0 
 0 
 0 
 0 
 0 
 0 
 0 
 0 
 0 
 0 
 0 
 0 
 0 
 0 
 0 
 0 
 0 
 0 
 0 
 0 
 0 
 0 
 0 
 0 
 0 
 0 
 0 
 0 
 0 
 0 
 0 
 0 
 0 
 0 
 0 
 0 
 0 
 0 
 0 
 0 
 0 
 0 
 0 
 0 
 0 
 0 
 0 
 0 
 0 
 0 
 0 
 0 
 0 
 0 
 0 
 0 
 0 
 0 
 0 
 0 
 0 
 0 
 0 
 0 
 0 
 0 
 0 
 0 
 0 
 0 
 0 
 0 
 0 
 0 
 0 
 0 
 0 
 0 
 0 
 0 
 0 
 0 
 0 
 0 
 0 
 0 
 0 
 0 
 0 
 0 
 0 
 0 
 0 
 0 
 0 
 0 
 0 
 0 
 0 
 0 
 0 
 0 
 0 
 0 
 0 
 0 
 0 
 0 
 0 
 0 
 0 
 0 
 0 
 0 
 0 
 0 
 0 
 0 
 0 
 0 
 0 
 0 
 0 
 0 
 0 
 0 
 0 
 0 
 0 
 0 
 0 
 0 
 0 
 0 
 0 
 0 
 0 
 0 
 0 
 0 
 0 
 0 
 0 
 0 
 0 
 0 
 0 
 0 
 0 
 0 
 0 
 0 
 0 
 0 
 0 
 0 
 0 
 0 
 0 
 0 
 0 
 0 
 0 
 0 
 0 
 0 
 0 
 0 
 0 
 0 
 0 
 0 
 0 
 0 
 0 
 0 
 0 
 0 
 0 
 0 
 0 
 0 
 0 
 0 
 0 
 0 
 0 
 0 
 0 
 0 
 0 
 0 
 0 
 0 
 0 
 0 
 0 
 0 
 0 
 0 
 0 
 0 
 0 
 0 
 0 
 0 
 0 
 0 
 0 
 0 
 0 
 0 
 0 
 0 
 0 
 0 
 0 
 0 
 0 
 0 
 0 
 0 
 0 
 0 
 0 
 0 
 0 
 0 
 0 
 0 
 0 
 0 
 0 
 0 
 0 
 0 
 0 
 0 
 0 
 0 
 0 
 0 
 0 
 0 
 0 
 0 
 0 
 0 
 0 
 0 
 0 
 0 
 0 
 0 
 0 
 0 
 0 
 0 
 0 
 0 
 0 
 0 
 0 
 0 
 0 
 0 
 0 
 0 
 0 
 0 
 0 
 0 
 0 
 0 
 0 
 0 
 0 
 0 
 0 
 0 
 0 
 0 
 0 
 0 
 0 
 0 
 0 
 0 
 0 
 0 
 0 
 0 
 0 
 0 
 0 
 0 
 0 
 0 
 0 
 0 
 0 
 0 
 0 
 0 
 0 
 0 
 0 
 0 
 0 
 0 
 0 
 0 
 0 
 0 
 0 
 0 
 0 
 0 
 0 
 0 
 0 
 0 
 0 
 0 
 0 
 0 
 0 
 0 
 0 
 0 
 0 
 0 
 0 
 0 
 0 
 0 
 0 
 0 
 0 
 0 
 0 
 0 
 0 
 0 
 0 
 0 
 0 
 0 
 0 
 0 
 0 
 0 
 0 
 0 
 0 
 0 
 0 
 0 
 0 
 0 
 0 
 0 
 0 
 0 
 0 
 0 
 0 
 0 
 0 
 0 
 0 
 0 
 0 
 0 
 0 
 0 
 0 
 0 
 0 
 0 
 0 
 0 
 0 
 0 
 0 
 0 
 0 
 0 
 0 
 0 
 0 
 0 
 0 
 0 
 0 
 0 
 0 
 0 
 0 
 0 
 0 
 0 
 0 
 0 
 0 
 0 
 0 
 0 
 0 
 0 
 0 
 0 
 0 
 0 
 0 
 0 
 0 
 0 
 0 
 0 
 0 
 0 
 0 
 0 
 0 
 0 
 0 
 0 
 0 
 0 
 0 
 0 
 0 
 0 
 0 
 0 
 0 
 0 
 0 
 0 
 0 
 0 
 0 
 0 
 0 
 0 
 0 
 0 
 0 
 0 
 0 
 0 
 0 
 0 
 0 
 0 
 0 
 0 
 0 
 0 
 0 
 0 
 0 
 0 
 0 
 0 
 0 
 0 
 0 
 0 
 0 
 0 
 0 
 0 
 0 
 0 
 0 
 0 
 0 
 0 
 0 
 0 
 0 
 0 
 0 
 0 
 0 
 0 
 1 
 0 
 0 
 0 
 0 
 0 
 0 
 0 
 0 
 0 
 0 
 0 
 0 
 0 
 0 
 0 
 0 
 0 
 0 
 0 
 0 
 0 
 0 
 0 
 0 
 0 
 0 
 0 
 
 
 40 
 0 
 0 
 0 
 0 
 0 
 0 
 0 
 0 
 0 
 0 
 0 
 0 
 0 
 0 
 0 
 0 
 0 
 0 
 0 
 0 
 0 
 0 
 0 
 0 
 0 
 0 
 0 
 0 
 0 
 0 
 0 
 0 
 0 
 0 
 0 
 0 
 0 
 0 
 5 
 0 
 22 
 0 
 0 
 0 
 0 
 0 
 0 
 0 
 0 
 0 
 0 
 0 
 0 
 0 
 0 
 0 
 0 
 0 
 0 
 0 
 0 
 0 
 0 
 0 
 0 
 0 
 0 
 0 
 0 
 0 
 0 
 0 
 0 
 0 
 0 
 0 
 0 
 0 
 0 
 0 
 0 
 0 
 0 
 0 
 0 
 0 
 0 
 0 
 0 
 0 
 0 
 0 
 0 
 0 
 0 
 0 
 0 
 0 
 0 
 0 
 0 
 0 
 0 
 0 
 0 
 0 
 0 
 0 
 0 
 0 
 0 
 0 
 0 
 0 
 0 
 0 
 0 
 0 
 0 
 0 
 0 
 0 
 0 
 0 
 0 
 0 
 0 
 0 
 0 
 0 
 0 
 0 
 0 
 0 
 0 
 0 
 0 
 0 
 0 
 0 
 0 
 0 
 0 
 0 
 0 
 0 
 0 
 0 
 0 
 0 
 0 
 0 
 0 
 0 
 0 
 0 
 0 
 0 
 0 
 0 
 0 
 0 
 0 
 0 
 0 
 0 
 0 
 0 
 0 
 0 
 0 
 0 
 0 
 0 
 0 
 0 
 0 
 0 
 0 
 0 
 0 
 0 
 0 
 0 
 0 
 0 
 0 
 0 
 0 
 0 
 0 
 0 
 0 
 0 
 0 
 0 
 0 
 0 
 0 
 0 
 0 
 0 
 0 
 0 
 0 
 0 
 0 
 0 
 0 
 0 
 0 
 0 
 0 
 0 
 0 
 0 
 0 
 0 
 0 
 0 
 0 
 0 
 0 
 0 
 0 
 0 
 0 
 0 
 0 
 0 
 0 
 0 
 0 
 0 
 0 
 0 
 0 
 0 
 0 
 0 
 0 
 0 
 0 
 0 
 0 
 0 
 0 
 0 
 0 
 0 
 0 
 0 
 0 
 0 
 0 
 0 
 0 
 0 
 0 
 0 
 0 
 0 
 0 
 0 
 0 
 0 
 0 
 0 
 0 
 0 
 0 
 0 
 0 
 0 
 0 
 0 
 0 
 0 
 0 
 0 
 0 
 0 
 0 
 0 
 0 
 0 
 0 
 0 
 0 
 0 
 0 
 0 
 0 
 0 
 0 
 0 
 0 
 0 
 0 
 0 
 0 
 0 
 0 
 0 
 0 
 0 
 0 
 0 
 0 
 0 
 0 
 0 
 0 
 0 
 0 
 0 
 0 
 0 
 0 
 0 
 0 
 0 
 0 
 0 
 0 
 0 
 0 
 0 
 0 
 0 
 0 
 0 
 0 
 0 
 0 
 0 
 0 
 0 
 0 
 0 
 0 
 0 
 0 
 0 
 0 
 0 
 0 
 0 
 0 
 0 
 0 
 0 
 0 
 0 
 0 
 0 
 0 
 0 
 0 
 0 
 0 
 0 
 0 
 0 
 0 
 0 
 0 
 0 
 0 
 0 
 0 
 0 
 0 
 0 
 0 
 0 
 0 
 0 
 0 
 0 
 0 
 0 
 0 
 0 
 0 
 0 
 0 
 0 
 0 
 0 
 0 
 0 
 0 
 0 
 0 
 0 
 0 
 0 
 0 
 0 
 0 
 0 
 0 
 0 
 0 
 0 
 0 
 0 
 0 
 0 
 0 
 0 
 0 
 0 
 0 
 0 
 0 
 0 
 0 
 0 
 0 
 0 
 0 
 0 
 0 
 0 
 0 
 0 
 0 
 0 
 0 
 0 
 0 
 0 
 0 
 0 
 0 
 0 
 0 
 0 
 0 
 0 
 0 
 0 
 0 
 0 
 0 
 0 
 0 
 0 
 0 
 0 
 0 
 0 
 0 
 0 
 0 
 0 
 0 
 0 
 0 
 0 
 0 
 0 
 0 
 0 
 0 
 0 
 0 
 0 
 0 
 0 
 0 
 0 
 0 
 0 
 0 
 0 
 0 
 0 
 0 
 0 
 0 
 0 
 0 
 0 
 0 
 0 
 0 
 0 
 0 
 0 
 0 
 0 
 0 
 0 
 0 
 0 
 0 
 0 
 0 
 0 
 0 
 0 
 0 
 0 
 0 
 0 
 0 
 0 
 0 
 0 
 0 
 0 
 0 
 0 
 0 
 0 
 0 
 0 
 0 
 0 
 0 
 0 
 0 
 0 
 0 
 0 
 0 
 0 
 0 
 0 
 0 
 0 
 0 
 0 
 0 
 0 
 0 
 0 
 0 
 0 
 0 
 0 
 0 
 0 
 0 
 0 
 0 
 0 
 0 
 0 
 0 
 0 
 0 
 0 
 0 
 0 
 0 
 0 
 0 
 0 
 0 
 0 
 0 
 0 
 0 
 0 
 0 
 0 
 0 
 0 
 0 
 0 
 0 
 0 
 0 
 0 
 0 
 0 
 0 
 0 
 0 
 0 
 0 
 0 
 0 
 0 
 0 
 0 
 0 
 0 
 0 
 0 
 0 
 0 
 0 
 0 
 0 
 0 
 0 
 0 
 0 
 0 
 0 
 0 
 0 
 0 
 0 
 0 
 0 
 0 
 0 
 0 
 0 
 0 
 0 
 0 
 0 
 0 
 0 
 0 
 0 
 0 
 0 
 0 
 0 
 0 
 0 
 0 
 0 
 0 
 0 
 0 
 0 
 0 
 0 
 0 
 0 
 0 
 0 
 0 
 0 
 0 
 0 
 0 
 0 
 0 
 0 
 0 
 0 
 0 
 0 
 0 
 0 
 0 
 0 
 0 
 0 
 0 
 0 
 0 
 0 
 0 
 0 
 0 
 0 
 0 
 0 
 0 
 0 
 0 
 0 
 0 
 0 
 0 
 0 
 0 
 0 
 0 
 0 
 0 
 0 
 0 
 0 
 0 
 0 
 0 
 0 
 0 
 0 
 0 
 0 
 0 
 0 
 0 
 0 
 0 
 0 
 0 
 0 
 0 
 0 
 0 
 0 
 0 
 0 
 0 
 0 
 0 
 0 
 0 
 0 
 0 
 0 
 0 
 0 
 0 
 0 
 0 
 0 
 0 
 0 
 0 
 0 
 0 
 0 
 0 
 0 
 0 
 0 
 0 
 0 
 0 
 0 
 0 
 0 
 0 
 0 
 0 
 0 
 0 
 0 
 0 
 0 
 0 
 0 
 0 
 0 
 0 
 0 
 0 
 0 
 0 
 0 
 0 
 0 
 0 
 0 
 0 
 0 
 0 
 0 
 0 
 0 
 0 
 0 
 0 
 0 
 0 
 0 
 0 
 0 
 0 
 0 
 0 
 0 
 0 
 0 
 0 
 0 
 0 
 0 
 0 
 0 
 0 
 0 
 0 
 0 
 0 
 0 
 0 
 0 
 0 
 0 
 0 
 0 
 0 
 0 
 0 
 0 
 0 
 0 
 0 
 0 
 0 
 0 
 0 
 0 
 0 
 0 
 0 
 0 
 0 
 0 
 0 
 0 
 0 
 0 
 0 
 0 
 0 
 0 
 0 
 0 
 0 
 
 
 41 
 0 
 0 
 0 
 0 
 0 
 0 
 0 
 0 
 0 
 0 
 0 
 0 
 0 
 0 
 0 
 0 
 0 
 0 
 0 
 0 
 0 
 0 
 0 
 0 
 0 
 0 
 0 
 0 
 0 
 0 
 0 
 0 
 0 
 0 
 0 
 0 
 0 
 0 
 0 
 0 
 0 
 31 
 0 
 0 
 0 
 0 
 0 
 0 
 0 
 0 
 0 
 0 
 0 
 0 
 0 
 0 
 0 
 0 
 0 
 0 
 0 
 0 
 0 
 0 
 0 
 0 
 0 
 0 
 0 
 0 
 0 
 0 
 0 
 0 
 0 
 0 
 0 
 0 
 0 
 0 
 0 
 0 
 0 
 0 
 0 
 0 
 0 
 0 
 0 
 0 
 0 
 0 
 0 
 0 
 0 
 0 
 0 
 0 
 0 
 0 
 0 
 0 
 0 
 0 
 0 
 0 
 0 
 0 
 0 
 0 
 0 
 0 
 0 
 0 
 0 
 0 
 0 
 0 
 0 
 0 
 0 
 0 
 0 
 0 
 0 
 0 
 0 
 0 
 0 
 0 
 0 
 0 
 0 
 0 
 0 
 0 
 0 
 0 
 0 
 0 
 0 
 0 
 0 
 0 
 0 
 0 
 0 
 0 
 0 
 0 
 0 
 0 
 0 
 0 
 0 
 0 
 0 
 0 
 0 
 0 
 0 
 0 
 0 
 0 
 0 
 0 
 0 
 0 
 0 
 0 
 0 
 0 
 0 
 0 
 0 
 0 
 0 
 0 
 0 
 0 
 0 
 0 
 0 
 0 
 0 
 0 
 0 
 0 
 0 
 0 
 0 
 0 
 0 
 0 
 0 
 0 
 0 
 0 
 0 
 0 
 0 
 0 
 0 
 0 
 0 
 0 
 0 
 0 
 0 
 0 
 0 
 0 
 0 
 0 
 0 
 0 
 0 
 0 
 0 
 0 
 0 
 0 
 0 
 0 
 0 
 0 
 0 
 0 
 0 
 0 
 0 
 0 
 0 
 0 
 0 
 0 
 0 
 0 
 0 
 0 
 0 
 0 
 0 
 0 
 0 
 0 
 0 
 0 
 0 
 0 
 0 
 0 
 0 
 0 
 0 
 0 
 0 
 0 
 0 
 0 
 0 
 0 
 0 
 0 
 0 
 0 
 0 
 0 
 0 
 0 
 0 
 0 
 0 
 0 
 0 
 0 
 0 
 0 
 0 
 0 
 0 
 0 
 0 
 0 
 0 
 0 
 0 
 0 
 0 
 0 
 0 
 0 
 0 
 0 
 0 
 0 
 0 
 0 
 0 
 0 
 0 
 0 
 0 
 0 
 0 
 0 
 0 
 0 
 0 
 0 
 0 
 0 
 0 
 0 
 0 
 0 
 0 
 0 
 0 
 0 
 0 
 0 
 0 
 0 
 0 
 0 
 0 
 0 
 0 
 0 
 0 
 0 
 0 
 0 
 0 
 0 
 0 
 0 
 0 
 0 
 0 
 0 
 0 
 0 
 0 
 0 
 0 
 0 
 0 
 0 
 0 
 0 
 0 
 0 
 0 
 0 
 0 
 0 
 0 
 0 
 0 
 0 
 0 
 0 
 0 
 0 
 0 
 0 
 0 
 0 
 0 
 0 
 0 
 0 
 0 
 0 
 0 
 0 
 0 
 0 
 0 
 0 
 0 
 0 
 0 
 0 
 0 
 0 
 0 
 0 
 0 
 0 
 0 
 0 
 0 
 0 
 0 
 0 
 0 
 0 
 0 
 0 
 0 
 0 
 0 
 0 
 0 
 0 
 0 
 0 
 0 
 0 
 0 
 0 
 0 
 0 
 0 
 0 
 0 
 0 
 0 
 0 
 0 
 0 
 0 
 0 
 0 
 0 
 0 
 0 
 0 
 0 
 0 
 0 
 0 
 0 
 0 
 0 
 0 
 0 
 0 
 0 
 0 
 0 
 0 
 0 
 0 
 0 
 0 
 0 
 0 
 0 
 0 
 0 
 0 
 0 
 0 
 0 
 0 
 0 
 0 
 0 
 0 
 0 
 0 
 0 
 0 
 0 
 0 
 0 
 0 
 0 
 0 
 0 
 0 
 0 
 0 
 0 
 0 
 0 
 0 
 0 
 0 
 0 
 0 
 0 
 0 
 0 
 0 
 0 
 0 
 0 
 0 
 0 
 0 
 0 
 0 
 0 
 0 
 0 
 0 
 0 
 0 
 0 
 0 
 0 
 0 
 0 
 0 
 0 
 0 
 0 
 0 
 0 
 0 
 0 
 0 
 0 
 0 
 0 
 0 
 0 
 0 
 0 
 0 
 0 
 0 
 0 
 0 
 0 
 0 
 0 
 0 
 0 
 0 
 0 
 0 
 0 
 0 
 0 
 0 
 0 
 0 
 0 
 0 
 0 
 0 
 0 
 0 
 0 
 0 
 0 
 0 
 0 
 0 
 0 
 0 
 0 
 0 
 0 
 0 
 0 
 0 
 0 
 0 
 0 
 0 
 0 
 0 
 0 
 0 
 0 
 0 
 0 
 0 
 0 
 0 
 0 
 0 
 0 
 0 
 0 
 0 
 0 
 0 
 0 
 0 
 0 
 0 
 0 
 0 
 0 
 0 
 0 
 0 
 0 
 0 
 0 
 0 
 0 
 0 
 0 
 0 
 0 
 0 
 0 
 0 
 0 
 0 
 0 
 0 
 0 
 0 
 0 
 0 
 0 
 0 
 0 
 0 
 0 
 0 
 0 
 0 
 0 
 0 
 0 
 0 
 0 
 0 
 0 
 0 
 0 
 0 
 0 
 0 
 0 
 0 
 0 
 0 
 0 
 0 
 0 
 0 
 0 
 0 
 0 
 0 
 0 
 0 
 0 
 0 
 0 
 0 
 0 
 0 
 0 
 0 
 0 
 0 
 0 
 0 
 0 
 0 
 0 
 0 
 0 
 0 
 0 
 0 
 0 
 0 
 0 
 0 
 0 
 0 
 0 
 0 
 0 
 0 
 0 
 0 
 0 
 0 
 0 
 0 
 0 
 0 
 0 
 0 
 0 
 0 
 0 
 0 
 0 
 0 
 0 
 0 
 0 
 0 
 0 
 0 
 0 
 0 
 0 
 0 
 0 
 0 
 0 
 0 
 0 
 0 
 0 
 0 
 0 
 0 
 0 
 0 
 0 
 0 
 0 
 0 
 0 
 0 
 0 
 0 
 0 
 0 
 0 
 0 
 0 
 0 
 0 
 0 
 0 
 0 
 0 
 0 
 0 
 0 
 0 
 0 
 0 
 0 
 0 
 0 
 0 
 0 
 0 
 0 
 0 
 0 
 0 
 0 
 0 
 0 
 0 
 0 
 0 
 0 
 0 
 0 
 0 
 0 
 0 
 0 
 0 
 0 
 0 
 0 
 0 
 0 
 0 
 0 
 0 
 0 
 0 
 0 
 0 
 0 
 0 
 0 
 0 
 0 
 0 
 0 
 0 
 0 
 0 
 0 
 0 
 0 
 0 
 0 
 0 
 0 
 0 
 0 
 0 
 0 
 0 
 0 
 0 
 0 
 0 
 0 
 0 
 0 
 0 
 0 
 0 
 0 
 0 
 0 
 0 
 0 
 0 
 0 
 0 
 0 
 0 
 0 
 0 
 0 
 0 
 0 
 0 
 
 
 42 
 0 
 0 
 0 
 0 
 0 
 0 
 0 
 0 
 0 
 0 
 0 
 0 
 0 
 0 
 0 
 0 
 0 
 0 
 0 
 0 
 0 
 0 
 0 
 0 
 0 
 0 
 0 
 0 
 0 
 0 
 0 
 0 
 0 
 0 
 0 
 0 
 0 
 0 
 0 
 0 
 0 
 0 
 6 
 0 
 0 
 0 
 0 
 0 
 0 
 0 
 0 
 0 
 0 
 0 
 0 
 0 
 0 
 0 
 0 
 0 
 0 
 0 
 0 
 0 
 0 
 0 
 0 
 0 
 0 
 0 
 0 
 0 
 0 
 0 
 0 
 0 
 0 
 0 
 0 
 0 
 0 
 0 
 0 
 0 
 0 
 0 
 0 
 0 
 0 
 0 
 0 
 0 
 0 
 0 
 0 
 0 
 0 
 0 
 0 
 0 
 0 
 0 
 0 
 0 
 0 
 0 
 0 
 0 
 0 
 0 
 0 
 0 
 0 
 0 
 0 
 0 
 0 
 0 
 0 
 0 
 0 
 0 
 0 
 0 
 0 
 0 
 0 
 0 
 0 
 0 
 0 
 0 
 0 
 0 
 0 
 0 
 0 
 0 
 0 
 0 
 0 
 0 
 0 
 0 
 0 
 0 
 0 
 0 
 0 
 0 
 0 
 0 
 0 
 0 
 0 
 0 
 0 
 0 
 0 
 0 
 0 
 0 
 0 
 0 
 0 
 0 
 0 
 0 
 0 
 0 
 0 
 0 
 0 
 0 
 0 
 0 
 0 
 0 
 0 
 0 
 0 
 0 
 0 
 0 
 0 
 0 
 0 
 0 
 0 
 0 
 0 
 0 
 0 
 0 
 0 
 0 
 0 
 0 
 0 
 0 
 0 
 0 
 0 
 0 
 0 
 0 
 0 
 0 
 0 
 0 
 0 
 0 
 0 
 0 
 0 
 0 
 0 
 0 
 0 
 0 
 0 
 0 
 0 
 0 
 0 
 0 
 0 
 0 
 0 
 0 
 0 
 0 
 0 
 0 
 0 
 0 
 0 
 0 
 0 
 0 
 0 
 0 
 0 
 0 
 0 
 0 
 0 
 0 
 0 
 0 
 0 
 0 
 0 
 0 
 0 
 0 
 0 
 0 
 0 
 0 
 0 
 0 
 0 
 0 
 0 
 0 
 0 
 0 
 0 
 0 
 0 
 0 
 0 
 0 
 0 
 0 
 0 
 0 
 0 
 0 
 0 
 0 
 0 
 0 
 0 
 0 
 0 
 0 
 0 
 0 
 0 
 0 
 0 
 0 
 0 
 0 
 0 
 0 
 0 
 0 
 0 
 0 
 0 
 0 
 0 
 0 
 0 
 0 
 0 
 0 
 0 
 0 
 0 
 0 
 0 
 0 
 0 
 0 
 0 
 0 
 0 
 0 
 0 
 0 
 0 
 0 
 0 
 0 
 0 
 0 
 0 
 0 
 0 
 0 
 0 
 0 
 0 
 0 
 0 
 0 
 0 
 0 
 0 
 0 
 0 
 0 
 0 
 0 
 0 
 0 
 0 
 0 
 0 
 0 
 0 
 0 
 0 
 0 
 0 
 0 
 0 
 0 
 0 
 0 
 0 
 0 
 0 
 0 
 0 
 0 
 0 
 0 
 0 
 0 
 0 
 0 
 0 
 0 
 0 
 0 
 0 
 0 
 0 
 0 
 0 
 0 
 0 
 0 
 0 
 0 
 0 
 0 
 0 
 0 
 0 
 0 
 0 
 0 
 0 
 0 
 0 
 0 
 0 
 0 
 0 
 0 
 0 
 0 
 0 
 0 
 0 
 0 
 0 
 0 
 0 
 0 
 0 
 0 
 0 
 0 
 0 
 0 
 0 
 0 
 0 
 0 
 0 
 0 
 0 
 0 
 0 
 0 
 0 
 0 
 0 
 0 
 0 
 0 
 0 
 0 
 0 
 0 
 0 
 0 
 0 
 0 
 0 
 0 
 0 
 0 
 0 
 0 
 0 
 0 
 0 
 0 
 0 
 0 
 0 
 0 
 0 
 0 
 0 
 0 
 0 
 0 
 0 
 0 
 0 
 0 
 0 
 0 
 0 
 0 
 0 
 0 
 0 
 0 
 0 
 0 
 0 
 0 
 0 
 0 
 0 
 0 
 0 
 0 
 0 
 0 
 0 
 0 
 0 
 0 
 0 
 0 
 0 
 0 
 0 
 0 
 0 
 0 
 0 
 0 
 0 
 0 
 0 
 0 
 0 
 0 
 0 
 0 
 0 
 0 
 0 
 0 
 0 
 0 
 0 
 0 
 0 
 0 
 0 
 0 
 0 
 0 
 0 
 0 
 0 
 0 
 0 
 0 
 0 
 0 
 0 
 0 
 0 
 0 
 0 
 0 
 0 
 0 
 0 
 0 
 0 
 0 
 0 
 0 
 0 
 0 
 0 
 0 
 0 
 0 
 0 
 0 
 0 
 0 
 0 
 0 
 0 
 0 
 0 
 0 
 0 
 0 
 0 
 0 
 0 
 0 
 0 
 0 
 0 
 0 
 0 
 0 
 0 
 0 
 0 
 0 
 0 
 0 
 0 
 0 
 0 
 0 
 0 
 0 
 0 
 0 
 0 
 0 
 0 
 0 
 0 
 0 
 0 
 0 
 0 
 0 
 0 
 0 
 0 
 0 
 0 
 0 
 0 
 0 
 0 
 0 
 0 
 0 
 0 
 0 
 0 
 0 
 0 
 0 
 0 
 0 
 0 
 0 
 0 
 0 
 0 
 0 
 0 
 0 
 0 
 0 
 0 
 0 
 0 
 0 
 0 
 0 
 0 
 0 
 0 
 0 
 0 
 0 
 0 
 0 
 0 
 0 
 0 
 0 
 0 
 0 
 0 
 0 
 0 
 0 
 0 
 0 
 0 
 0 
 0 
 0 
 0 
 0 
 0 
 0 
 0 
 0 
 0 
 0 
 0 
 0 
 0 
 0 
 0 
 0 
 0 
 0 
 0 
 0 
 0 
 0 
 0 
 0 
 0 
 0 
 0 
 0 
 0 
 0 
 0 
 0 
 0 
 0 
 0 
 0 
 0 
 0 
 0 
 0 
 0 
 0 
 0 
 0 
 0 
 0 
 0 
 0 
 0 
 0 
 0 
 0 
 0 
 0 
 0 
 0 
 0 
 0 
 0 
 0 
 0 
 0 
 0 
 0 
 0 
 0 
 0 
 0 
 0 
 0 
 0 
 0 
 0 
 0 
 0 
 0 
 0 
 0 
 0 
 0 
 0 
 0 
 0 
 0 
 0 
 0 
 0 
 0 
 0 
 0 
 0 
 0 
 0 
 0 
 0 
 0 
 0 
 0 
 0 
 0 
 0 
 0 
 0 
 0 
 0 
 0 
 0 
 0 
 0 
 0 
 0 
 0 
 0 
 0 
 0 
 0 
 0 
 0 
 0 
 0 
 0 
 0 
 0 
 0 
 0 
 0 
 0 
 0 
 0 
 0 
 0 
 0 
 0 
 0 
 0 
 0 
 0 
 0 
 0 
 0 
 0 
 0 
 0 
 0 
 0 
 0 
 0 
 0 
 0 
 0 
 0 
 0 
 0 
 0 
 0 
 0 
 0 
 0 
 0 
 0 
 0 
 0 
 0 
 0 
 0 
 0 
 0 
 0 
 
 
 43 
 0 
 0 
 0 
 0 
 0 
 0 
 0 
 0 
 0 
 0 
 0 
 0 
 0 
 0 
 0 
 0 
 0 
 0 
 0 
 0 
 0 
 0 
 0 
 0 
 0 
 0 
 0 
 0 
 0 
 0 
 0 
 0 
 0 
 0 
 0 
 0 
 0 
 0 
 0 
 0 
 0 
 0 
 0 
 6 
 0 
 0 
 0 
 0 
 0 
 0 
 0 
 0 
 0 
 0 
 0 
 0 
 0 
 0 
 0 
 0 
 0 
 0 
 0 
 0 
 0 
 0 
 0 
 0 
 0 
 0 
 0 
 0 
 0 
 0 
 0 
 0 
 0 
 0 
 0 
 0 
 0 
 0 
 0 
 0 
 0 
 0 
 0 
 0 
 0 
 0 
 0 
 0 
 0 
 0 
 0 
 0 
 0 
 0 
 0 
 0 
 0 
 0 
 0 
 0 
 0 
 0 
 0 
 0 
 0 
 0 
 0 
 0 
 0 
 0 
 0 
 0 
 0 
 0 
 0 
 0 
 0 
 0 
 0 
 0 
 0 
 0 
 0 
 0 
 0 
 0 
 0 
 0 
 0 
 0 
 0 
 0 
 0 
 0 
 0 
 0 
 0 
 0 
 0 
 0 
 0 
 0 
 0 
 0 
 0 
 0 
 0 
 0 
 0 
 0 
 0 
 0 
 0 
 0 
 0 
 0 
 0 
 0 
 0 
 0 
 0 
 0 
 0 
 0 
 0 
 0 
 0 
 0 
 0 
 0 
 0 
 0 
 0 
 0 
 0 
 0 
 0 
 0 
 0 
 0 
 0 
 0 
 0 
 0 
 0 
 0 
 0 
 0 
 0 
 0 
 0 
 0 
 0 
 0 
 0 
 0 
 0 
 0 
 0 
 0 
 0 
 0 
 0 
 0 
 0 
 0 
 0 
 0 
 0 
 0 
 0 
 0 
 0 
 0 
 0 
 0 
 0 
 0 
 0 
 0 
 0 
 0 
 0 
 0 
 0 
 0 
 0 
 0 
 0 
 0 
 0 
 0 
 0 
 0 
 0 
 0 
 0 
 0 
 0 
 0 
 0 
 0 
 0 
 0 
 0 
 0 
 0 
 0 
 0 
 0 
 0 
 0 
 0 
 0 
 0 
 0 
 0 
 0 
 0 
 0 
 0 
 0 
 0 
 0 
 0 
 0 
 0 
 0 
 0 
 0 
 0 
 0 
 0 
 0 
 0 
 0 
 0 
 0 
 0 
 0 
 0 
 0 
 0 
 0 
 0 
 0 
 0 
 0 
 0 
 0 
 0 
 0 
 0 
 0 
 0 
 0 
 0 
 0 
 0 
 0 
 0 
 0 
 0 
 0 
 0 
 0 
 0 
 0 
 0 
 0 
 0 
 0 
 0 
 0 
 0 
 0 
 0 
 0 
 0 
 0 
 0 
 0 
 0 
 0 
 0 
 0 
 0 
 0 
 0 
 0 
 0 
 0 
 0 
 0 
 0 
 0 
 0 
 0 
 0 
 0 
 0 
 0 
 0 
 0 
 0 
 0 
 0 
 0 
 0 
 0 
 0 
 0 
 0 
 0 
 0 
 0 
 0 
 0 
 0 
 0 
 0 
 0 
 0 
 0 
 0 
 0 
 0 
 0 
 0 
 0 
 0 
 0 
 0 
 0 
 0 
 0 
 0 
 0 
 0 
 0 
 0 
 0 
 0 
 0 
 0 
 0 
 0 
 0 
 0 
 0 
 0 
 0 
 0 
 0 
 0 
 0 
 0 
 0 
 0 
 0 
 0 
 0 
 0 
 0 
 0 
 0 
 0 
 0 
 0 
 0 
 0 
 0 
 0 
 0 
 0 
 0 
 0 
 0 
 0 
 0 
 0 
 0 
 0 
 0 
 0 
 0 
 0 
 0 
 0 
 0 
 0 
 0 
 0 
 0 
 0 
 0 
 0 
 0 
 0 
 0 
 0 
 0 
 0 
 0 
 0 
 0 
 0 
 0 
 0 
 0 
 0 
 0 
 0 
 0 
 0 
 0 
 0 
 0 
 0 
 0 
 0 
 0 
 0 
 0 
 0 
 0 
 0 
 0 
 0 
 0 
 0 
 0 
 0 
 0 
 0 
 0 
 0 
 0 
 0 
 0 
 0 
 0 
 0 
 0 
 0 
 0 
 0 
 0 
 0 
 0 
 0 
 0 
 0 
 0 
 0 
 0 
 0 
 0 
 0 
 0 
 0 
 0 
 0 
 0 
 0 
 0 
 0 
 0 
 0 
 0 
 0 
 0 
 0 
 0 
 0 
 0 
 0 
 0 
 0 
 0 
 0 
 0 
 0 
 0 
 0 
 0 
 0 
 0 
 0 
 0 
 0 
 0 
 0 
 0 
 0 
 0 
 0 
 0 
 0 
 0 
 0 
 0 
 0 
 0 
 0 
 0 
 0 
 0 
 0 
 0 
 0 
 0 
 0 
 0 
 0 
 0 
 0 
 0 
 0 
 0 
 0 
 0 
 0 
 0 
 0 
 0 
 0 
 0 
 0 
 0 
 0 
 0 
 0 
 0 
 0 
 0 
 0 
 0 
 0 
 0 
 0 
 0 
 0 
 0 
 0 
 0 
 0 
 0 
 0 
 0 
 0 
 0 
 0 
 0 
 0 
 0 
 0 
 0 
 0 
 0 
 0 
 0 
 0 
 0 
 0 
 0 
 0 
 0 
 0 
 0 
 0 
 0 
 0 
 0 
 0 
 0 
 0 
 0 
 0 
 0 
 0 
 0 
 0 
 0 
 0 
 0 
 0 
 0 
 0 
 0 
 0 
 0 
 0 
 0 
 0 
 0 
 0 
 0 
 0 
 0 
 0 
 0 
 0 
 0 
 0 
 0 
 0 
 0 
 0 
 0 
 0 
 0 
 0 
 0 
 0 
 0 
 0 
 0 
 0 
 0 
 0 
 0 
 0 
 0 
 0 
 0 
 0 
 0 
 0 
 0 
 0 
 0 
 0 
 0 
 0 
 0 
 0 
 0 
 0 
 0 
 0 
 0 
 0 
 0 
 0 
 0 
 0 
 0 
 0 
 0 
 0 
 0 
 0 
 0 
 0 
 0 
 0 
 0 
 0 
 0 
 0 
 0 
 0 
 0 
 0 
 0 
 0 
 0 
 0 
 0 
 0 
 0 
 0 
 0 
 0 
 0 
 0 
 0 
 0 
 0 
 0 
 0 
 0 
 0 
 0 
 0 
 0 
 0 
 0 
 0 
 0 
 0 
 0 
 0 
 0 
 0 
 0 
 0 
 0 
 0 
 0 
 0 
 0 
 0 
 0 
 0 
 0 
 0 
 0 
 0 
 0 
 0 
 0 
 0 
 0 
 0 
 0 
 0 
 0 
 0 
 0 
 0 
 0 
 0 
 0 
 0 
 0 
 0 
 0 
 0 
 0 
 0 
 0 
 0 
 0 
 0 
 0 
 0 
 0 
 0 
 0 
 0 
 0 
 0 
 0 
 0 
 0 
 0 
 0 
 0 
 0 
 0 
 0 
 0 
 0 
 0 
 0 
 0 
 0 
 0 
 0 
 0 
 0 
 0 
 0 
 0 
 0 
 0 
 0 
 0 
 0 
 0 
 0 
 0 
 0 
 0 
 0 
 0 
 0 
 0 
 0 
 0 
 
 
 44 
 0 
 0 
 0 
 0 
 0 
 0 
 0 
 0 
 0 
 0 
 0 
 0 
 0 
 0 
 0 
 0 
 0 
 0 
 0 
 0 
 0 
 0 
 0 
 0 
 0 
 0 
 0 
 0 
 0 
 0 
 0 
 0 
 0 
 0 
 0 
 0 
 0 
 0 
 0 
 0 
 0 
 0 
 0 
 0 
 10 
 0 
 0 
 0 
 0 
 0 
 0 
 0 
 0 
 0 
 0 
 0 
 0 
 0 
 0 
 0 
 0 
 0 
 0 
 0 
 0 
 0 
 0 
 0 
 0 
 0 
 0 
 0 
 0 
 0 
 0 
 0 
 0 
 0 
 0 
 0 
 0 
 0 
 0 
 0 
 0 
 0 
 0 
 0 
 0 
 0 
 0 
 0 
 0 
 0 
 0 
 0 
 0 
 0 
 0 
 0 
 0 
 0 
 0 
 0 
 0 
 0 
 0 
 0 
 0 
 0 
 0 
 0 
 0 
 0 
 0 
 0 
 0 
 0 
 0 
 0 
 0 
 0 
 0 
 0 
 0 
 0 
 0 
 0 
 0 
 0 
 0 
 0 
 0 
 0 
 0 
 0 
 0 
 0 
 0 
 0 
 0 
 0 
 0 
 0 
 0 
 0 
 0 
 0 
 0 
 0 
 0 
 0 
 0 
 0 
 0 
 0 
 0 
 0 
 0 
 0 
 0 
 0 
 0 
 0 
 0 
 0 
 0 
 0 
 0 
 0 
 0 
 0 
 0 
 0 
 0 
 0 
 0 
 0 
 0 
 0 
 0 
 0 
 0 
 0 
 0 
 0 
 0 
 0 
 0 
 0 
 0 
 0 
 0 
 0 
 0 
 0 
 0 
 0 
 0 
 0 
 0 
 0 
 0 
 0 
 0 
 0 
 0 
 0 
 0 
 0 
 0 
 0 
 0 
 0 
 0 
 0 
 0 
 0 
 0 
 0 
 0 
 0 
 0 
 0 
 0 
 0 
 0 
 0 
 0 
 0 
 0 
 0 
 0 
 0 
 0 
 0 
 0 
 0 
 0 
 0 
 0 
 0 
 0 
 0 
 0 
 0 
 0 
 0 
 0 
 0 
 0 
 0 
 0 
 0 
 0 
 0 
 0 
 0 
 0 
 0 
 0 
 0 
 0 
 0 
 0 
 0 
 0 
 0 
 0 
 0 
 0 
 0 
 0 
 0 
 0 
 0 
 0 
 0 
 0 
 0 
 0 
 0 
 0 
 0 
 0 
 0 
 0 
 0 
 0 
 0 
 0 
 0 
 0 
 0 
 0 
 0 
 0 
 0 
 0 
 0 
 0 
 0 
 0 
 0 
 0 
 0 
 0 
 0 
 0 
 0 
 0 
 0 
 0 
 0 
 0 
 0 
 0 
 0 
 0 
 0 
 0 
 0 
 0 
 0 
 0 
 0 
 0 
 0 
 0 
 0 
 0 
 0 
 0 
 0 
 0 
 0 
 0 
 0 
 0 
 0 
 0 
 0 
 0 
 0 
 0 
 0 
 0 
 0 
 0 
 0 
 0 
 0 
 0 
 0 
 0 
 0 
 0 
 0 
 0 
 0 
 0 
 0 
 0 
 0 
 0 
 0 
 0 
 0 
 0 
 0 
 0 
 0 
 0 
 0 
 0 
 0 
 0 
 0 
 0 
 0 
 0 
 0 
 0 
 0 
 0 
 0 
 0 
 0 
 0 
 0 
 0 
 0 
 0 
 0 
 0 
 0 
 0 
 0 
 0 
 0 
 0 
 0 
 0 
 0 
 0 
 0 
 0 
 0 
 0 
 0 
 0 
 0 
 0 
 0 
 0 
 0 
 0 
 0 
 0 
 0 
 0 
 0 
 0 
 0 
 0 
 0 
 0 
 0 
 0 
 0 
 0 
 0 
 0 
 0 
 0 
 0 
 0 
 0 
 0 
 0 
 0 
 0 
 0 
 0 
 0 
 0 
 0 
 0 
 0 
 0 
 0 
 0 
 0 
 0 
 0 
 0 
 0 
 0 
 0 
 0 
 0 
 0 
 0 
 0 
 0 
 0 
 0 
 0 
 0 
 0 
 0 
 0 
 0 
 0 
 0 
 0 
 0 
 0 
 0 
 0 
 0 
 0 
 0 
 0 
 0 
 0 
 0 
 0 
 0 
 0 
 0 
 0 
 0 
 0 
 0 
 0 
 0 
 0 
 0 
 0 
 0 
 0 
 0 
 0 
 0 
 0 
 0 
 0 
 0 
 0 
 0 
 0 
 0 
 0 
 0 
 0 
 0 
 0 
 0 
 0 
 0 
 0 
 0 
 0 
 0 
 0 
 0 
 0 
 0 
 0 
 0 
 0 
 0 
 0 
 0 
 0 
 0 
 0 
 0 
 0 
 0 
 0 
 0 
 0 
 0 
 0 
 0 
 0 
 0 
 0 
 0 
 0 
 0 
 0 
 0 
 0 
 0 
 0 
 0 
 0 
 0 
 0 
 0 
 0 
 0 
 0 
 0 
 0 
 0 
 0 
 0 
 0 
 0 
 0 
 0 
 0 
 0 
 0 
 0 
 0 
 0 
 0 
 0 
 0 
 0 
 0 
 0 
 0 
 0 
 0 
 0 
 0 
 0 
 0 
 0 
 0 
 0 
 0 
 0 
 0 
 0 
 0 
 0 
 0 
 0 
 0 
 0 
 0 
 0 
 0 
 0 
 0 
 0 
 0 
 0 
 0 
 0 
 0 
 0 
 0 
 0 
 0 
 0 
 0 
 0 
 0 
 0 
 0 
 0 
 0 
 0 
 0 
 0 
 0 
 0 
 0 
 0 
 0 
 0 
 0 
 0 
 0 
 0 
 0 
 0 
 0 
 0 
 0 
 0 
 0 
 0 
 0 
 0 
 0 
 0 
 0 
 0 
 0 
 0 
 0 
 0 
 0 
 0 
 0 
 0 
 0 
 0 
 0 
 0 
 0 
 0 
 0 
 0 
 0 
 0 
 0 
 0 
 0 
 0 
 0 
 0 
 0 
 0 
 0 
 0 
 0 
 0 
 0 
 0 
 0 
 0 
 0 
 0 
 0 
 0 
 0 
 0 
 0 
 0 
 0 
 0 
 0 
 0 
 0 
 0 
 0 
 0 
 0 
 0 
 0 
 0 
 0 
 0 
 0 
 0 
 0 
 0 
 0 
 0 
 0 
 0 
 0 
 0 
 0 
 0 
 0 
 0 
 0 
 0 
 0 
 0 
 0 
 0 
 0 
 0 
 0 
 0 
 0 
 0 
 0 
 0 
 0 
 0 
 0 
 0 
 0 
 0 
 0 
 0 
 0 
 0 
 0 
 0 
 0 
 0 
 0 
 0 
 0 
 0 
 0 
 0 
 0 
 0 
 0 
 0 
 0 
 0 
 0 
 0 
 0 
 0 
 0 
 0 
 0 
 0 
 0 
 0 
 0 
 0 
 0 
 0 
 0 
 0 
 0 
 0 
 0 
 0 
 0 
 0 
 0 
 0 
 0 
 0 
 0 
 0 
 0 
 0 
 0 
 0 
 0 
 0 
 0 
 0 
 0 
 0 
 0 
 0 
 0 
 0 
 0 
 0 
 0 
 0 
 0 
 0 
 0 
 0 
 0 
 0 
 0 
 0 
 0 
 0 
 0 
 0 
 0 
 
 
 45 
 0 
 0 
 0 
 0 
 0 
 0 
 0 
 0 
 0 
 0 
 0 
 0 
 0 
 0 
 0 
 0 
 0 
 0 
 0 
 0 
 0 
 0 
 0 
 0 
 0 
 0 
 0 
 0 
 0 
 0 
 0 
 0 
 0 
 0 
 0 
 0 
 0 
 0 
 0 
 0 
 0 
 0 
 0 
 0 
 0 
 7 
 0 
 0 
 0 
 0 
 0 
 0 
 0 
 0 
 0 
 0 
 0 
 0 
 0 
 0 
 0 
 0 
 0 
 0 
 0 
 0 
 0 
 0 
 0 
 0 
 0 
 0 
 0 
 0 
 0 
 0 
 0 
 0 
 0 
 0 
 0 
 0 
 0 
 0 
 0 
 0 
 0 
 0 
 0 
 0 
 0 
 0 
 0 
 0 
 0 
 0 
 0 
 0 
 0 
 0 
 0 
 0 
 0 
 0 
 0 
 0 
 0 
 0 
 0 
 0 
 0 
 0 
 0 
 0 
 0 
 0 
 0 
 0 
 0 
 0 
 0 
 0 
 0 
 0 
 0 
 0 
 0 
 0 
 0 
 0 
 0 
 0 
 0 
 0 
 0 
 0 
 0 
 0 
 0 
 0 
 0 
 0 
 0 
 0 
 0 
 0 
 0 
 0 
 0 
 0 
 0 
 0 
 0 
 0 
 0 
 0 
 0 
 0 
 0 
 0 
 0 
 0 
 0 
 0 
 0 
 0 
 0 
 0 
 0 
 0 
 0 
 0 
 0 
 0 
 0 
 0 
 0 
 0 
 0 
 0 
 0 
 0 
 0 
 0 
 0 
 0 
 0 
 0 
 0 
 0 
 0 
 0 
 0 
 0 
 0 
 0 
 0 
 0 
 0 
 0 
 0 
 0 
 0 
 0 
 0 
 0 
 0 
 0 
 0 
 0 
 0 
 0 
 0 
 0 
 0 
 0 
 0 
 0 
 0 
 0 
 0 
 0 
 0 
 0 
 0 
 0 
 0 
 0 
 0 
 0 
 0 
 0 
 0 
 0 
 0 
 0 
 0 
 0 
 0 
 0 
 0 
 0 
 0 
 0 
 0 
 0 
 0 
 0 
 0 
 0 
 0 
 0 
 0 
 0 
 0 
 0 
 0 
 0 
 0 
 0 
 0 
 0 
 0 
 0 
 0 
 0 
 0 
 0 
 0 
 0 
 0 
 0 
 0 
 0 
 0 
 0 
 0 
 0 
 0 
 0 
 0 
 0 
 0 
 0 
 0 
 0 
 0 
 0 
 0 
 0 
 0 
 0 
 0 
 0 
 0 
 0 
 0 
 0 
 0 
 0 
 0 
 0 
 0 
 0 
 0 
 0 
 0 
 0 
 0 
 0 
 0 
 0 
 0 
 0 
 0 
 0 
 0 
 0 
 0 
 0 
 0 
 0 
 0 
 0 
 0 
 0 
 0 
 0 
 0 
 0 
 0 
 0 
 0 
 0 
 0 
 0 
 0 
 0 
 0 
 0 
 0 
 0 
 0 
 0 
 0 
 0 
 0 
 0 
 0 
 0 
 0 
 0 
 0 
 0 
 0 
 0 
 0 
 0 
 0 
 0 
 0 
 0 
 0 
 0 
 0 
 0 
 0 
 0 
 0 
 0 
 0 
 0 
 0 
 0 
 0 
 0 
 0 
 0 
 0 
 0 
 0 
 0 
 0 
 0 
 0 
 0 
 0 
 0 
 0 
 0 
 0 
 0 
 0 
 0 
 0 
 0 
 0 
 0 
 0 
 0 
 0 
 0 
 0 
 0 
 0 
 0 
 0 
 0 
 0 
 0 
 0 
 0 
 0 
 0 
 0 
 0 
 0 
 0 
 0 
 0 
 0 
 0 
 0 
 0 
 0 
 0 
 0 
 0 
 0 
 0 
 0 
 0 
 0 
 0 
 0 
 0 
 0 
 0 
 0 
 0 
 0 
 0 
 0 
 0 
 0 
 0 
 0 
 0 
 0 
 0 
 0 
 0 
 0 
 0 
 0 
 0 
 0 
 0 
 0 
 0 
 0 
 0 
 0 
 0 
 0 
 0 
 0 
 0 
 0 
 0 
 0 
 0 
 0 
 0 
 0 
 0 
 0 
 0 
 0 
 0 
 0 
 0 
 0 
 0 
 0 
 0 
 0 
 0 
 0 
 0 
 0 
 0 
 0 
 0 
 0 
 0 
 0 
 0 
 0 
 0 
 0 
 0 
 0 
 0 
 0 
 0 
 0 
 0 
 0 
 0 
 0 
 0 
 0 
 0 
 0 
 0 
 0 
 0 
 0 
 0 
 0 
 0 
 0 
 0 
 0 
 0 
 0 
 0 
 0 
 0 
 0 
 0 
 0 
 0 
 0 
 0 
 0 
 0 
 0 
 0 
 0 
 0 
 0 
 0 
 0 
 0 
 0 
 0 
 0 
 0 
 0 
 0 
 0 
 0 
 0 
 0 
 0 
 0 
 0 
 0 
 0 
 0 
 0 
 0 
 0 
 0 
 0 
 0 
 0 
 0 
 0 
 0 
 0 
 0 
 0 
 0 
 0 
 0 
 0 
 0 
 0 
 0 
 0 
 0 
 0 
 0 
 0 
 0 
 0 
 0 
 0 
 0 
 0 
 0 
 0 
 0 
 0 
 0 
 0 
 0 
 0 
 0 
 0 
 0 
 0 
 0 
 0 
 0 
 0 
 0 
 0 
 0 
 0 
 0 
 0 
 0 
 0 
 0 
 0 
 0 
 0 
 0 
 0 
 0 
 0 
 0 
 0 
 0 
 0 
 0 
 0 
 0 
 0 
 0 
 0 
 0 
 0 
 0 
 0 
 0 
 0 
 0 
 0 
 0 
 0 
 0 
 0 
 0 
 0 
 0 
 0 
 0 
 0 
 0 
 0 
 0 
 0 
 0 
 0 
 0 
 0 
 0 
 0 
 0 
 0 
 0 
 0 
 0 
 0 
 0 
 0 
 0 
 0 
 0 
 0 
 0 
 0 
 0 
 0 
 0 
 0 
 0 
 0 
 0 
 0 
 0 
 0 
 0 
 0 
 0 
 0 
 0 
 0 
 0 
 0 
 0 
 0 
 0 
 0 
 0 
 0 
 0 
 0 
 0 
 0 
 0 
 0 
 0 
 0 
 0 
 0 
 0 
 0 
 0 
 0 
 0 
 0 
 0 
 0 
 0 
 0 
 0 
 0 
 0 
 0 
 0 
 0 
 0 
 0 
 0 
 0 
 0 
 0 
 0 
 0 
 0 
 0 
 0 
 0 
 0 
 0 
 0 
 0 
 0 
 0 
 0 
 0 
 0 
 0 
 0 
 0 
 0 
 0 
 0 
 0 
 0 
 0 
 0 
 0 
 0 
 0 
 0 
 0 
 0 
 0 
 0 
 0 
 0 
 0 
 0 
 0 
 0 
 0 
 0 
 0 
 0 
 0 
 0 
 0 
 0 
 0 
 0 
 0 
 0 
 0 
 0 
 0 
 0 
 0 
 0 
 0 
 0 
 0 
 0 
 0 
 0 
 0 
 0 
 0 
 0 
 0 
 0 
 0 
 0 
 0 
 0 
 0 
 0 
 0 
 0 
 0 
 0 
 0 
 0 
 0 
 0 
 0 
 0 
 0 
 0 
 0 
 0 
 0 
 0 
 0 
 0 
 
 
 46 
 0 
 0 
 0 
 0 
 0 
 0 
 0 
 0 
 0 
 0 
 0 
 0 
 0 
 0 
 0 
 0 
 0 
 0 
 0 
 0 
 0 
 0 
 0 
 0 
 0 
 0 
 0 
 0 
 0 
 0 
 0 
 0 
 0 
 0 
 0 
 0 
 0 
 0 
 0 
 0 
 0 
 0 
 0 
 0 
 0 
 0 
 2 
 0 
 0 
 0 
 0 
 0 
 0 
 0 
 0 
 0 
 0 
 0 
 0 
 0 
 0 
 0 
 0 
 0 
 0 
 0 
 0 
 0 
 0 
 0 
 0 
 0 
 0 
 0 
 0 
 0 
 0 
 0 
 0 
 0 
 0 
 0 
 0 
 0 
 0 
 0 
 0 
 0 
 0 
 0 
 0 
 0 
 0 
 0 
 0 
 0 
 0 
 0 
 0 
 0 
 0 
 0 
 0 
 0 
 0 
 0 
 0 
 0 
 0 
 0 
 0 
 0 
 0 
 0 
 0 
 0 
 0 
 0 
 0 
 0 
 0 
 0 
 0 
 0 
 0 
 0 
 0 
 0 
 0 
 0 
 0 
 0 
 0 
 0 
 0 
 0 
 0 
 0 
 0 
 0 
 0 
 0 
 0 
 0 
 0 
 0 
 0 
 0 
 0 
 0 
 0 
 0 
 0 
 0 
 0 
 0 
 0 
 0 
 0 
 0 
 0 
 0 
 0 
 0 
 0 
 0 
 0 
 0 
 0 
 0 
 0 
 0 
 0 
 0 
 0 
 0 
 0 
 0 
 0 
 0 
 0 
 0 
 0 
 0 
 0 
 0 
 0 
 0 
 0 
 0 
 0 
 0 
 0 
 0 
 0 
 0 
 0 
 0 
 0 
 0 
 0 
 0 
 0 
 0 
 0 
 0 
 0 
 0 
 0 
 0 
 0 
 0 
 0 
 0 
 0 
 0 
 0 
 0 
 0 
 0 
 0 
 0 
 0 
 0 
 0 
 0 
 0 
 0 
 0 
 0 
 0 
 0 
 0 
 0 
 0 
 0 
 0 
 0 
 0 
 0 
 0 
 0 
 0 
 0 
 0 
 0 
 0 
 0 
 0 
 0 
 0 
 0 
 0 
 0 
 0 
 0 
 0 
 0 
 0 
 0 
 0 
 0 
 0 
 0 
 0 
 0 
 0 
 0 
 0 
 0 
 0 
 0 
 0 
 0 
 0 
 0 
 0 
 0 
 0 
 0 
 0 
 0 
 0 
 0 
 0 
 0 
 0 
 0 
 0 
 0 
 0 
 0 
 0 
 0 
 0 
 0 
 0 
 0 
 0 
 0 
 0 
 0 
 0 
 0 
 0 
 0 
 0 
 0 
 0 
 0 
 0 
 0 
 0 
 0 
 0 
 0 
 0 
 0 
 0 
 0 
 0 
 0 
 0 
 0 
 0 
 0 
 0 
 0 
 0 
 0 
 0 
 0 
 0 
 0 
 0 
 0 
 0 
 0 
 0 
 0 
 0 
 0 
 0 
 0 
 0 
 0 
 0 
 0 
 0 
 0 
 0 
 0 
 0 
 0 
 0 
 0 
 0 
 0 
 0 
 0 
 0 
 0 
 0 
 0 
 0 
 0 
 0 
 0 
 0 
 0 
 0 
 0 
 0 
 0 
 0 
 0 
 0 
 0 
 0 
 0 
 0 
 0 
 0 
 0 
 0 
 0 
 0 
 0 
 0 
 0 
 0 
 0 
 0 
 0 
 0 
 0 
 0 
 0 
 0 
 0 
 0 
 0 
 0 
 0 
 0 
 0 
 0 
 0 
 0 
 0 
 0 
 0 
 0 
 0 
 0 
 0 
 0 
 0 
 0 
 0 
 0 
 0 
 0 
 0 
 0 
 0 
 0 
 0 
 0 
 0 
 0 
 0 
 0 
 0 
 0 
 0 
 0 
 0 
 0 
 0 
 0 
 0 
 0 
 0 
 0 
 0 
 0 
 0 
 0 
 0 
 0 
 0 
 0 
 0 
 0 
 0 
 0 
 0 
 0 
 0 
 0 
 0 
 0 
 0 
 0 
 0 
 0 
 0 
 0 
 0 
 0 
 0 
 0 
 0 
 0 
 0 
 0 
 0 
 0 
 0 
 0 
 0 
 0 
 0 
 0 
 0 
 0 
 0 
 0 
 0 
 0 
 0 
 0 
 0 
 0 
 0 
 0 
 0 
 0 
 0 
 0 
 0 
 0 
 0 
 0 
 0 
 0 
 0 
 0 
 0 
 0 
 0 
 0 
 0 
 0 
 0 
 0 
 0 
 0 
 0 
 0 
 0 
 0 
 0 
 0 
 0 
 0 
 0 
 0 
 0 
 0 
 0 
 0 
 0 
 0 
 0 
 0 
 0 
 0 
 0 
 0 
 0 
 0 
 0 
 0 
 0 
 0 
 0 
 0 
 0 
 0 
 0 
 0 
 0 
 0 
 0 
 0 
 0 
 0 
 0 
 0 
 0 
 0 
 0 
 0 
 0 
 0 
 0 
 0 
 0 
 0 
 0 
 0 
 0 
 0 
 0 
 0 
 0 
 0 
 0 
 0 
 0 
 0 
 0 
 0 
 0 
 0 
 0 
 0 
 0 
 0 
 0 
 0 
 0 
 0 
 0 
 0 
 0 
 0 
 0 
 0 
 0 
 0 
 0 
 0 
 0 
 0 
 0 
 0 
 0 
 0 
 0 
 0 
 0 
 0 
 0 
 0 
 0 
 0 
 0 
 0 
 0 
 0 
 0 
 0 
 0 
 0 
 0 
 0 
 0 
 0 
 0 
 0 
 0 
 0 
 0 
 0 
 0 
 0 
 0 
 0 
 0 
 0 
 0 
 0 
 0 
 0 
 0 
 0 
 0 
 0 
 0 
 0 
 0 
 0 
 0 
 0 
 0 
 0 
 0 
 0 
 0 
 0 
 0 
 0 
 0 
 0 
 0 
 0 
 0 
 0 
 0 
 0 
 0 
 0 
 0 
 0 
 0 
 0 
 0 
 0 
 0 
 0 
 0 
 0 
 0 
 0 
 0 
 0 
 0 
 0 
 0 
 0 
 0 
 0 
 0 
 0 
 0 
 0 
 0 
 0 
 0 
 0 
 0 
 0 
 0 
 0 
 0 
 0 
 0 
 0 
 0 
 0 
 0 
 0 
 0 
 0 
 0 
 0 
 0 
 0 
 0 
 0 
 0 
 0 
 0 
 0 
 0 
 0 
 0 
 0 
 0 
 0 
 0 
 0 
 0 
 0 
 0 
 0 
 0 
 0 
 0 
 0 
 0 
 0 
 0 
 0 
 0 
 0 
 0 
 0 
 0 
 0 
 0 
 0 
 0 
 0 
 0 
 0 
 0 
 0 
 0 
 0 
 0 
 0 
 0 
 0 
 0 
 0 
 0 
 0 
 0 
 0 
 0 
 0 
 0 
 0 
 0 
 0 
 0 
 0 
 0 
 0 
 0 
 0 
 0 
 0 
 0 
 0 
 0 
 0 
 0 
 0 
 0 
 0 
 0 
 0 
 0 
 0 
 0 
 0 
 0 
 0 
 0 
 0 
 0 
 0 
 0 
 0 
 0 
 0 
 0 
 0 
 0 
 0 
 0 
 0 
 0 
 0 
 0 
 0 
 0 
 0 
 0 
 0 
 0 
 
 
 47 
 0 
 0 
 0 
 0 
 0 
 0 
 0 
 0 
 0 
 0 
 0 
 0 
 0 
 0 
 0 
 0 
 0 
 0 
 0 
 0 
 0 
 0 
 0 
 0 
 0 
 0 
 0 
 0 
 0 
 0 
 0 
 0 
 0 
 0 
 0 
 0 
 0 
 0 
 0 
 0 
 0 
 0 
 0 
 0 
 0 
 0 
 0 
 3 
 0 
 0 
 0 
 0 
 0 
 0 
 0 
 0 
 0 
 0 
 0 
 0 
 0 
 0 
 0 
 0 
 0 
 0 
 0 
 0 
 0 
 0 
 0 
 0 
 0 
 0 
 0 
 0 
 0 
 0 
 0 
 0 
 0 
 0 
 0 
 0 
 0 
 0 
 0 
 0 
 0 
 0 
 0 
 0 
 0 
 0 
 0 
 0 
 0 
 0 
 0 
 0 
 0 
 0 
 0 
 0 
 0 
 0 
 0 
 0 
 0 
 0 
 0 
 0 
 0 
 0 
 0 
 0 
 0 
 0 
 0 
 0 
 0 
 0 
 0 
 0 
 0 
 0 
 0 
 0 
 0 
 0 
 0 
 0 
 0 
 0 
 0 
 0 
 0 
 0 
 0 
 0 
 0 
 0 
 0 
 0 
 0 
 0 
 0 
 0 
 0 
 0 
 0 
 0 
 0 
 0 
 0 
 0 
 0 
 0 
 0 
 0 
 0 
 0 
 0 
 0 
 0 
 0 
 0 
 0 
 0 
 0 
 0 
 0 
 0 
 0 
 0 
 0 
 0 
 0 
 0 
 0 
 0 
 0 
 0 
 0 
 0 
 0 
 0 
 0 
 0 
 0 
 0 
 0 
 0 
 0 
 0 
 0 
 0 
 0 
 0 
 0 
 0 
 0 
 0 
 0 
 0 
 0 
 0 
 0 
 0 
 0 
 0 
 0 
 0 
 0 
 0 
 0 
 0 
 0 
 0 
 0 
 0 
 0 
 0 
 0 
 0 
 0 
 0 
 0 
 0 
 0 
 0 
 0 
 0 
 0 
 0 
 0 
 0 
 0 
 0 
 0 
 0 
 0 
 0 
 0 
 0 
 0 
 0 
 0 
 0 
 0 
 0 
 0 
 0 
 0 
 0 
 0 
 0 
 0 
 0 
 0 
 0 
 0 
 0 
 0 
 0 
 0 
 0 
 0 
 0 
 0 
 0 
 0 
 0 
 0 
 0 
 0 
 0 
 0 
 0 
 0 
 0 
 0 
 0 
 0 
 0 
 0 
 0 
 0 
 0 
 0 
 0 
 0 
 0 
 0 
 0 
 0 
 0 
 0 
 0 
 0 
 0 
 0 
 0 
 0 
 0 
 0 
 0 
 0 
 0 
 0 
 0 
 0 
 0 
 0 
 0 
 0 
 0 
 0 
 0 
 0 
 0 
 0 
 0 
 0 
 0 
 0 
 0 
 0 
 0 
 0 
 0 
 0 
 0 
 0 
 0 
 0 
 0 
 0 
 0 
 0 
 0 
 0 
 0 
 0 
 0 
 0 
 0 
 0 
 0 
 0 
 0 
 0 
 0 
 0 
 0 
 0 
 0 
 0 
 0 
 0 
 0 
 0 
 0 
 0 
 0 
 0 
 0 
 0 
 0 
 0 
 0 
 0 
 0 
 0 
 0 
 0 
 0 
 0 
 0 
 0 
 0 
 0 
 0 
 0 
 0 
 0 
 0 
 0 
 0 
 0 
 0 
 0 
 0 
 0 
 0 
 0 
 0 
 0 
 0 
 0 
 0 
 0 
 0 
 0 
 0 
 0 
 0 
 0 
 0 
 0 
 0 
 0 
 0 
 0 
 0 
 0 
 0 
 0 
 0 
 0 
 0 
 0 
 0 
 0 
 0 
 0 
 0 
 0 
 0 
 0 
 0 
 0 
 0 
 0 
 0 
 0 
 0 
 0 
 0 
 0 
 0 
 0 
 0 
 0 
 0 
 0 
 0 
 0 
 0 
 0 
 0 
 0 
 0 
 0 
 0 
 0 
 0 
 0 
 0 
 0 
 0 
 0 
 0 
 0 
 0 
 0 
 0 
 0 
 0 
 0 
 0 
 0 
 0 
 0 
 0 
 0 
 0 
 0 
 0 
 0 
 0 
 0 
 0 
 0 
 0 
 0 
 0 
 0 
 0 
 0 
 0 
 0 
 0 
 0 
 0 
 0 
 0 
 0 
 0 
 0 
 0 
 0 
 0 
 0 
 0 
 0 
 0 
 0 
 0 
 0 
 0 
 0 
 0 
 0 
 0 
 0 
 0 
 0 
 0 
 0 
 0 
 0 
 0 
 0 
 0 
 0 
 0 
 0 
 0 
 0 
 0 
 0 
 0 
 0 
 0 
 0 
 0 
 0 
 0 
 0 
 0 
 0 
 0 
 0 
 0 
 0 
 0 
 0 
 0 
 0 
 0 
 0 
 0 
 0 
 0 
 0 
 0 
 0 
 0 
 0 
 0 
 0 
 0 
 0 
 0 
 0 
 0 
 0 
 0 
 0 
 0 
 0 
 0 
 0 
 0 
 0 
 0 
 0 
 0 
 0 
 0 
 0 
 0 
 0 
 0 
 0 
 0 
 0 
 0 
 0 
 0 
 0 
 0 
 0 
 0 
 0 
 0 
 0 
 0 
 0 
 0 
 0 
 0 
 0 
 0 
 0 
 0 
 0 
 0 
 0 
 0 
 0 
 0 
 0 
 0 
 0 
 0 
 0 
 0 
 0 
 0 
 0 
 0 
 0 
 0 
 0 
 0 
 0 
 0 
 0 
 0 
 0 
 0 
 0 
 0 
 0 
 0 
 0 
 0 
 0 
 0 
 0 
 0 
 0 
 0 
 0 
 0 
 0 
 0 
 0 
 0 
 0 
 0 
 0 
 0 
 0 
 0 
 0 
 0 
 0 
 0 
 0 
 0 
 0 
 0 
 0 
 0 
 0 
 0 
 0 
 0 
 0 
 0 
 0 
 0 
 0 
 0 
 0 
 0 
 0 
 0 
 0 
 0 
 0 
 0 
 0 
 0 
 0 
 0 
 0 
 0 
 0 
 0 
 0 
 0 
 0 
 0 
 0 
 0 
 0 
 0 
 0 
 0 
 0 
 0 
 0 
 0 
 0 
 0 
 0 
 0 
 0 
 0 
 0 
 0 
 0 
 0 
 0 
 0 
 0 
 0 
 0 
 0 
 0 
 0 
 0 
 0 
 0 
 0 
 0 
 0 
 0 
 0 
 0 
 0 
 0 
 0 
 0 
 0 
 0 
 0 
 0 
 0 
 0 
 0 
 0 
 0 
 0 
 0 
 0 
 0 
 0 
 0 
 0 
 0 
 0 
 0 
 0 
 0 
 0 
 0 
 0 
 0 
 0 
 0 
 0 
 0 
 0 
 0 
 0 
 0 
 0 
 0 
 0 
 0 
 0 
 0 
 0 
 0 
 0 
 0 
 0 
 0 
 0 
 0 
 0 
 0 
 0 
 0 
 0 
 0 
 0 
 0 
 0 
 0 
 0 
 0 
 0 
 0 
 0 
 0 
 0 
 0 
 0 
 0 
 0 
 0 
 0 
 0 
 0 
 0 
 0 
 0 
 0 
 0 
 0 
 0 
 0 
 0 
 0 
 0 
 0 
 0 
 0 
 0 
 0 
 
 
 48 
 0 
 0 
 0 
 0 
 0 
 0 
 0 
 0 
 0 
 0 
 0 
 0 
 0 
 0 
 0 
 0 
 0 
 0 
 0 
 0 
 0 
 0 
 0 
 0 
 0 
 0 
 0 
 0 
 0 
 0 
 0 
 0 
 0 
 0 
 0 
 0 
 0 
 0 
 0 
 0 
 0 
 0 
 0 
 0 
 0 
 0 
 0 
 0 
 10 
 0 
 0 
 0 
 0 
 0 
 0 
 0 
 0 
 0 
 0 
 0 
 0 
 0 
 0 
 0 
 0 
 0 
 0 
 0 
 0 
 0 
 0 
 0 
 0 
 0 
 0 
 0 
 0 
 0 
 0 
 0 
 0 
 0 
 0 
 0 
 0 
 0 
 0 
 0 
 0 
 0 
 0 
 0 
 0 
 0 
 0 
 0 
 0 
 0 
 0 
 0 
 0 
 0 
 0 
 0 
 0 
 0 
 0 
 0 
 0 
 0 
 0 
 0 
 0 
 0 
 0 
 0 
 0 
 0 
 0 
 0 
 0 
 0 
 0 
 0 
 0 
 0 
 0 
 0 
 0 
 0 
 0 
 0 
 0 
 0 
 0 
 0 
 0 
 0 
 0 
 0 
 0 
 0 
 0 
 0 
 0 
 0 
 0 
 0 
 0 
 0 
 0 
 0 
 0 
 0 
 0 
 0 
 0 
 0 
 0 
 0 
 0 
 0 
 0 
 0 
 0 
 0 
 0 
 0 
 0 
 0 
 0 
 0 
 0 
 0 
 0 
 0 
 0 
 0 
 0 
 0 
 0 
 0 
 0 
 0 
 0 
 0 
 0 
 0 
 0 
 0 
 0 
 0 
 0 
 0 
 0 
 0 
 0 
 0 
 0 
 0 
 0 
 0 
 0 
 0 
 0 
 0 
 0 
 0 
 0 
 0 
 0 
 0 
 0 
 0 
 0 
 0 
 0 
 0 
 0 
 0 
 0 
 0 
 0 
 0 
 0 
 0 
 0 
 0 
 0 
 0 
 0 
 0 
 0 
 0 
 0 
 0 
 0 
 0 
 0 
 0 
 0 
 0 
 0 
 0 
 0 
 0 
 0 
 0 
 0 
 0 
 0 
 0 
 0 
 0 
 0 
 0 
 0 
 0 
 0 
 0 
 0 
 0 
 0 
 0 
 0 
 0 
 0 
 0 
 0 
 0 
 0 
 0 
 0 
 0 
 0 
 0 
 0 
 0 
 0 
 0 
 0 
 0 
 0 
 0 
 0 
 0 
 0 
 0 
 0 
 0 
 0 
 0 
 0 
 0 
 0 
 0 
 0 
 0 
 0 
 0 
 0 
 0 
 0 
 0 
 0 
 0 
 0 
 0 
 0 
 0 
 0 
 0 
 0 
 0 
 0 
 0 
 0 
 0 
 0 
 0 
 0 
 0 
 0 
 0 
 0 
 0 
 0 
 0 
 0 
 0 
 0 
 0 
 0 
 0 
 0 
 0 
 0 
 0 
 0 
 0 
 0 
 0 
 0 
 0 
 0 
 0 
 0 
 0 
 0 
 0 
 0 
 0 
 0 
 0 
 0 
 0 
 0 
 0 
 0 
 0 
 0 
 0 
 0 
 0 
 0 
 0 
 0 
 0 
 0 
 0 
 0 
 0 
 0 
 0 
 0 
 0 
 0 
 0 
 0 
 0 
 0 
 0 
 0 
 0 
 0 
 0 
 0 
 0 
 0 
 0 
 0 
 0 
 0 
 0 
 0 
 0 
 0 
 0 
 0 
 0 
 0 
 0 
 0 
 0 
 0 
 0 
 0 
 0 
 0 
 0 
 0 
 0 
 0 
 0 
 0 
 0 
 0 
 0 
 0 
 0 
 0 
 0 
 0 
 0 
 0 
 0 
 0 
 0 
 0 
 0 
 0 
 0 
 0 
 0 
 0 
 0 
 0 
 0 
 0 
 0 
 0 
 0 
 0 
 0 
 0 
 0 
 0 
 0 
 0 
 0 
 0 
 0 
 0 
 0 
 0 
 0 
 0 
 0 
 0 
 0 
 0 
 0 
 0 
 0 
 0 
 0 
 0 
 0 
 0 
 0 
 0 
 0 
 0 
 0 
 0 
 0 
 0 
 0 
 0 
 0 
 0 
 0 
 0 
 0 
 0 
 0 
 0 
 0 
 0 
 0 
 0 
 0 
 0 
 0 
 0 
 0 
 0 
 0 
 0 
 0 
 0 
 0 
 0 
 0 
 0 
 0 
 0 
 0 
 0 
 0 
 0 
 0 
 0 
 0 
 0 
 0 
 0 
 0 
 0 
 0 
 0 
 0 
 0 
 0 
 0 
 0 
 0 
 0 
 0 
 0 
 0 
 0 
 0 
 0 
 0 
 0 
 0 
 0 
 0 
 0 
 0 
 0 
 0 
 0 
 0 
 0 
 0 
 0 
 0 
 0 
 0 
 0 
 0 
 0 
 0 
 0 
 0 
 0 
 0 
 0 
 0 
 0 
 0 
 0 
 0 
 0 
 0 
 0 
 0 
 0 
 0 
 0 
 0 
 0 
 0 
 0 
 0 
 0 
 0 
 0 
 0 
 0 
 0 
 0 
 0 
 0 
 0 
 0 
 0 
 0 
 0 
 0 
 0 
 0 
 0 
 0 
 0 
 0 
 0 
 0 
 0 
 0 
 0 
 0 
 0 
 0 
 0 
 0 
 0 
 0 
 0 
 0 
 0 
 0 
 0 
 0 
 0 
 0 
 0 
 0 
 0 
 0 
 0 
 0 
 0 
 0 
 0 
 0 
 0 
 0 
 0 
 0 
 0 
 0 
 0 
 0 
 0 
 0 
 0 
 0 
 0 
 0 
 0 
 0 
 0 
 0 
 0 
 0 
 0 
 0 
 0 
 0 
 0 
 0 
 0 
 0 
 0 
 0 
 0 
 0 
 0 
 0 
 0 
 0 
 0 
 0 
 0 
 0 
 0 
 0 
 0 
 0 
 0 
 0 
 0 
 0 
 0 
 0 
 0 
 0 
 0 
 0 
 0 
 0 
 0 
 0 
 0 
 0 
 0 
 0 
 0 
 0 
 0 
 0 
 0 
 0 
 0 
 0 
 0 
 0 
 0 
 0 
 0 
 0 
 0 
 0 
 0 
 0 
 0 
 0 
 0 
 0 
 0 
 0 
 0 
 0 
 0 
 0 
 0 
 0 
 0 
 0 
 0 
 0 
 0 
 0 
 0 
 0 
 0 
 0 
 0 
 0 
 0 
 0 
 0 
 0 
 0 
 0 
 0 
 0 
 0 
 0 
 0 
 0 
 0 
 0 
 0 
 0 
 0 
 0 
 0 
 0 
 0 
 0 
 0 
 0 
 0 
 0 
 0 
 0 
 0 
 0 
 0 
 0 
 0 
 0 
 0 
 0 
 0 
 0 
 0 
 0 
 0 
 0 
 0 
 0 
 0 
 0 
 0 
 0 
 0 
 0 
 0 
 0 
 0 
 0 
 0 
 0 
 0 
 0 
 0 
 0 
 0 
 0 
 0 
 0 
 0 
 0 
 0 
 0 
 0 
 0 
 0 
 0 
 0 
 0 
 0 
 0 
 0 
 0 
 0 
 0 
 0 
 0 
 0 
 0 
 0 
 0 
 0 
 0 
 0 
 0 
 0 
 0 
 0 
 0 
 
 
 49 
 0 
 0 
 0 
 0 
 0 
 0 
 0 
 0 
 0 
 0 
 0 
 0 
 0 
 0 
 0 
 0 
 0 
 0 
 0 
 0 
 0 
 0 
 0 
 0 
 0 
 0 
 0 
 0 
 0 
 0 
 0 
 0 
 0 
 0 
 0 
 0 
 0 
 0 
 0 
 0 
 0 
 0 
 0 
 0 
 0 
 0 
 0 
 0 
 0 
 6 
 0 
 0 
 0 
 0 
 0 
 0 
 0 
 0 
 0 
 0 
 0 
 0 
 0 
 0 
 0 
 0 
 0 
 0 
 0 
 0 
 0 
 0 
 0 
 0 
 0 
 0 
 0 
 0 
 0 
 0 
 0 
 0 
 0 
 0 
 0 
 0 
 0 
 0 
 0 
 0 
 0 
 0 
 0 
 0 
 0 
 0 
 0 
 0 
 0 
 0 
 0 
 0 
 0 
 0 
 0 
 0 
 0 
 0 
 0 
 0 
 0 
 0 
 0 
 0 
 0 
 0 
 0 
 0 
 0 
 0 
 0 
 0 
 0 
 0 
 0 
 0 
 0 
 0 
 0 
 0 
 0 
 0 
 0 
 0 
 0 
 0 
 0 
 0 
 0 
 0 
 0 
 0 
 0 
 0 
 0 
 0 
 0 
 0 
 0 
 0 
 0 
 0 
 0 
 0 
 0 
 0 
 0 
 0 
 0 
 0 
 0 
 0 
 0 
 0 
 0 
 0 
 0 
 0 
 0 
 0 
 0 
 0 
 0 
 0 
 0 
 0 
 0 
 0 
 0 
 0 
 0 
 0 
 0 
 0 
 0 
 0 
 0 
 0 
 0 
 0 
 0 
 0 
 0 
 0 
 0 
 0 
 0 
 0 
 0 
 0 
 0 
 0 
 0 
 0 
 0 
 0 
 0 
 0 
 0 
 0 
 0 
 0 
 0 
 0 
 0 
 0 
 0 
 0 
 0 
 0 
 0 
 0 
 0 
 0 
 0 
 0 
 0 
 0 
 0 
 0 
 0 
 0 
 0 
 0 
 0 
 0 
 0 
 0 
 0 
 0 
 0 
 0 
 0 
 0 
 0 
 0 
 0 
 0 
 0 
 0 
 0 
 0 
 0 
 0 
 0 
 0 
 0 
 0 
 0 
 0 
 0 
 0 
 0 
 0 
 0 
 0 
 0 
 0 
 0 
 0 
 0 
 0 
 0 
 0 
 0 
 0 
 0 
 0 
 0 
 0 
 0 
 0 
 0 
 0 
 0 
 0 
 0 
 0 
 0 
 0 
 0 
 0 
 0 
 0 
 0 
 0 
 0 
 0 
 0 
 0 
 0 
 0 
 0 
 0 
 0 
 0 
 0 
 0 
 0 
 0 
 0 
 0 
 0 
 0 
 0 
 0 
 0 
 0 
 0 
 0 
 0 
 0 
 0 
 0 
 0 
 0 
 0 
 0 
 0 
 0 
 0 
 0 
 0 
 0 
 0 
 0 
 0 
 0 
 0 
 0 
 0 
 0 
 0 
 0 
 0 
 0 
 0 
 0 
 0 
 0 
 0 
 0 
 0 
 0 
 0 
 0 
 0 
 0 
 0 
 0 
 0 
 0 
 0 
 0 
 0 
 0 
 0 
 0 
 0 
 0 
 0 
 0 
 0 
 0 
 0 
 0 
 0 
 0 
 0 
 0 
 0 
 0 
 0 
 0 
 0 
 0 
 0 
 0 
 0 
 0 
 0 
 0 
 0 
 0 
 0 
 0 
 0 
 0 
 0 
 0 
 0 
 0 
 0 
 0 
 0 
 0 
 0 
 0 
 0 
 0 
 0 
 0 
 0 
 0 
 0 
 0 
 0 
 0 
 0 
 0 
 0 
 0 
 0 
 0 
 0 
 0 
 0 
 0 
 0 
 0 
 0 
 0 
 0 
 0 
 0 
 0 
 0 
 0 
 0 
 0 
 0 
 0 
 0 
 0 
 0 
 0 
 0 
 0 
 0 
 0 
 0 
 0 
 0 
 0 
 0 
 0 
 0 
 0 
 0 
 0 
 0 
 0 
 0 
 0 
 0 
 0 
 0 
 0 
 0 
 0 
 0 
 0 
 0 
 0 
 0 
 0 
 0 
 0 
 0 
 0 
 0 
 0 
 0 
 0 
 0 
 0 
 0 
 0 
 0 
 0 
 0 
 0 
 0 
 0 
 0 
 0 
 0 
 0 
 0 
 0 
 0 
 0 
 0 
 0 
 0 
 0 
 0 
 0 
 0 
 0 
 0 
 0 
 0 
 0 
 0 
 0 
 0 
 0 
 0 
 0 
 0 
 0 
 0 
 0 
 0 
 0 
 0 
 0 
 0 
 0 
 0 
 0 
 0 
 0 
 0 
 0 
 0 
 0 
 0 
 0 
 0 
 0 
 0 
 0 
 0 
 0 
 0 
 0 
 0 
 0 
 0 
 0 
 0 
 0 
 0 
 0 
 0 
 0 
 0 
 0 
 0 
 0 
 0 
 0 
 0 
 0 
 0 
 0 
 0 
 0 
 0 
 0 
 0 
 0 
 0 
 0 
 0 
 0 
 0 
 0 
 0 
 0 
 0 
 0 
 0 
 0 
 0 
 0 
 0 
 0 
 0 
 0 
 0 
 0 
 0 
 0 
 0 
 0 
 0 
 0 
 0 
 0 
 0 
 0 
 0 
 0 
 0 
 0 
 0 
 0 
 0 
 0 
 0 
 0 
 0 
 0 
 0 
 0 
 0 
 0 
 0 
 0 
 0 
 0 
 0 
 0 
 0 
 0 
 0 
 0 
 0 
 0 
 0 
 0 
 0 
 0 
 0 
 0 
 0 
 0 
 0 
 0 
 0 
 0 
 0 
 0 
 0 
 0 
 0 
 0 
 0 
 0 
 0 
 0 
 0 
 0 
 0 
 0 
 0 
 0 
 0 
 0 
 0 
 0 
 0 
 0 
 0 
 0 
 0 
 0 
 0 
 0 
 0 
 0 
 0 
 0 
 0 
 0 
 0 
 0 
 0 
 0 
 0 
 0 
 0 
 0 
 0 
 0 
 0 
 0 
 0 
 0 
 0 
 0 
 0 
 0 
 0 
 0 
 0 
 0 
 0 
 0 
 0 
 0 
 0 
 0 
 0 
 0 
 0 
 0 
 0 
 0 
 0 
 0 
 0 
 0 
 0 
 0 
 0 
 0 
 0 
 0 
 0 
 0 
 0 
 0 
 0 
 0 
 0 
 0 
 0 
 0 
 0 
 0 
 0 
 0 
 0 
 0 
 0 
 0 
 0 
 0 
 0 
 0 
 0 
 0 
 0 
 0 
 0 
 0 
 0 
 0 
 0 
 0 
 0 
 0 
 0 
 0 
 0 
 0 
 0 
 0 
 0 
 0 
 0 
 0 
 0 
 0 
 0 
 0 
 0 
 0 
 0 
 0 
 0 
 0 
 0 
 0 
 0 
 0 
 0 
 0 
 0 
 0 
 0 
 0 
 0 
 0 
 0 
 0 
 0 
 0 
 0 
 0 
 0 
 0 
 0 
 0 
 0 
 0 
 0 
 0 
 0 
 0 
 0 
 0 
 0 
 0 
 0 
 0 
 0 
 0 
 0 
 0 
 0 
 0 
 0 
 0 
 0 
 0 
 0 
 0 
 0 
 0 
 0 
 0 
 
 
 50 
 0 
 0 
 0 
 0 
 0 
 0 
 0 
 0 
 0 
 0 
 0 
 0 
 0 
 0 
 0 
 0 
 0 
 0 
 0 
 0 
 0 
 0 
 0 
 0 
 0 
 0 
 0 
 0 
 0 
 0 
 0 
 0 
 0 
 0 
 0 
 0 
 0 
 0 
 0 
 0 
 0 
 0 
 0 
 0 
 0 
 0 
 0 
 0 
 0 
 0 
 0 
 0 
 0 
 0 
 0 
 0 
 0 
 0 
 0 
 0 
 0 
 0 
 0 
 0 
 0 
 0 
 0 
 0 
 0 
 0 
 0 
 0 
 0 
 0 
 0 
 0 
 0 
 0 
 0 
 0 
 0 
 0 
 0 
 0 
 0 
 0 
 0 
 0 
 0 
 0 
 0 
 0 
 0 
 0 
 0 
 0 
 0 
 0 
 0 
 0 
 0 
 0 
 0 
 0 
 0 
 0 
 0 
 0 
 0 
 0 
 0 
 0 
 0 
 0 
 0 
 0 
 0 
 0 
 0 
 0 
 0 
 0 
 0 
 0 
 0 
 0 
 0 
 0 
 0 
 0 
 0 
 0 
 0 
 0 
 0 
 0 
 0 
 0 
 0 
 0 
 0 
 0 
 0 
 0 
 0 
 0 
 0 
 0 
 0 
 0 
 0 
 0 
 0 
 0 
 0 
 0 
 0 
 0 
 0 
 0 
 0 
 0 
 0 
 3 
 0 
 0 
 0 
 0 
 0 
 0 
 0 
 0 
 0 
 0 
 0 
 0 
 0 
 0 
 0 
 0 
 0 
 0 
 0 
 0 
 0 
 0 
 0 
 0 
 0 
 0 
 0 
 0 
 0 
 0 
 0 
 0 
 0 
 0 
 0 
 0 
 0 
 0 
 0 
 0 
 0 
 0 
 0 
 0 
 0 
 0 
 0 
 0 
 0 
 0 
 0 
 0 
 0 
 0 
 0 
 0 
 0 
 0 
 0 
 0 
 0 
 0 
 0 
 0 
 0 
 0 
 0 
 0 
 0 
 0 
 0 
 0 
 0 
 0 
 0 
 0 
 0 
 0 
 0 
 0 
 0 
 0 
 0 
 0 
 0 
 0 
 0 
 0 
 0 
 0 
 0 
 0 
 0 
 0 
 0 
 0 
 0 
 0 
 0 
 0 
 0 
 0 
 0 
 0 
 0 
 0 
 0 
 0 
 0 
 0 
 0 
 0 
 0 
 0 
 0 
 0 
 0 
 0 
 0 
 0 
 0 
 0 
 0 
 0 
 0 
 0 
 0 
 0 
 0 
 0 
 0 
 0 
 0 
 0 
 0 
 0 
 0 
 0 
 0 
 0 
 0 
 0 
 0 
 0 
 0 
 0 
 0 
 0 
 0 
 0 
 0 
 0 
 0 
 0 
 0 
 0 
 0 
 0 
 0 
 0 
 0 
 0 
 0 
 0 
 0 
 0 
 0 
 0 
 0 
 0 
 0 
 0 
 0 
 0 
 0 
 0 
 0 
 0 
 0 
 0 
 0 
 0 
 0 
 0 
 0 
 0 
 0 
 0 
 0 
 0 
 0 
 0 
 0 
 0 
 0 
 0 
 0 
 0 
 0 
 0 
 0 
 0 
 0 
 0 
 0 
 0 
 0 
 0 
 0 
 0 
 0 
 0 
 0 
 0 
 0 
 0 
 0 
 0 
 0 
 0 
 0 
 0 
 0 
 0 
 0 
 0 
 0 
 0 
 0 
 0 
 0 
 0 
 0 
 0 
 0 
 0 
 0 
 0 
 0 
 0 
 0 
 0 
 0 
 0 
 0 
 0 
 0 
 0 
 0 
 0 
 0 
 0 
 0 
 0 
 0 
 0 
 0 
 0 
 0 
 0 
 0 
 0 
 0 
 0 
 0 
 0 
 0 
 0 
 0 
 0 
 0 
 0 
 0 
 0 
 0 
 0 
 0 
 0 
 0 
 0 
 0 
 0 
 0 
 0 
 0 
 0 
 0 
 0 
 0 
 0 
 0 
 0 
 0 
 0 
 0 
 0 
 0 
 0 
 0 
 0 
 0 
 0 
 0 
 0 
 0 
 0 
 0 
 0 
 0 
 0 
 0 
 0 
 0 
 0 
 0 
 0 
 0 
 0 
 0 
 0 
 0 
 0 
 0 
 0 
 0 
 0 
 0 
 0 
 0 
 0 
 0 
 0 
 0 
 0 
 0 
 0 
 0 
 0 
 0 
 0 
 0 
 0 
 0 
 0 
 0 
 0 
 0 
 0 
 0 
 0 
 0 
 0 
 0 
 0 
 0 
 0 
 0 
 0 
 0 
 0 
 0 
 0 
 0 
 0 
 0 
 0 
 0 
 0 
 0 
 0 
 0 
 0 
 0 
 0 
 0 
 0 
 0 
 0 
 0 
 0 
 0 
 0 
 0 
 0 
 0 
 0 
 0 
 0 
 0 
 0 
 0 
 0 
 0 
 0 
 0 
 0 
 0 
 0 
 0 
 0 
 0 
 0 
 0 
 0 
 0 
 0 
 0 
 0 
 0 
 0 
 0 
 0 
 0 
 0 
 0 
 0 
 0 
 0 
 0 
 0 
 0 
 0 
 0 
 0 
 0 
 0 
 0 
 0 
 0 
 0 
 0 
 0 
 0 
 0 
 0 
 0 
 0 
 0 
 0 
 0 
 0 
 0 
 0 
 0 
 0 
 0 
 0 
 0 
 0 
 0 
 0 
 0 
 0 
 0 
 0 
 0 
 0 
 0 
 0 
 0 
 0 
 0 
 0 
 0 
 0 
 0 
 0 
 0 
 0 
 0 
 0 
 0 
 0 
 0 
 0 
 0 
 0 
 0 
 0 
 0 
 0 
 0 
 0 
 0 
 0 
 0 
 0 
 0 
 0 
 0 
 0 
 0 
 0 
 0 
 0 
 0 
 0 
 0 
 0 
 0 
 0 
 0 
 0 
 0 
 0 
 0 
 0 
 0 
 0 
 0 
 0 
 0 
 0 
 0 
 0 
 0 
 0 
 0 
 0 
 0 
 0 
 0 
 0 
 0 
 0 
 0 
 0 
 0 
 0 
 0 
 0 
 0 
 0 
 0 
 0 
 0 
 0 
 0 
 0 
 0 
 0 
 0 
 0 
 0 
 0 
 0 
 0 
 0 
 0 
 0 
 0 
 0 
 0 
 0 
 0 
 0 
 0 
 0 
 0 
 0 
 0 
 0 
 0 
 0 
 0 
 0 
 0 
 0 
 0 
 0 
 0 
 0 
 0 
 0 
 0 
 0 
 0 
 0 
 0 
 0 
 0 
 0 
 0 
 0 
 0 
 0 
 0 
 0 
 0 
 0 
 0 
 0 
 0 
 0 
 0 
 0 
 0 
 0 
 0 
 0 
 0 
 0 
 0 
 0 
 0 
 0 
 0 
 0 
 0 
 0 
 0 
 0 
 0 
 0 
 0 
 0 
 0 
 0 
 0 
 0 
 0 
 0 
 0 
 0 
 0 
 0 
 0 
 0 
 0 
 0 
 0 
 0 
 0 
 0 
 0 
 0 
 0 
 0 
 0 
 0 
 0 
 0 
 0 
 0 
 0 
 0 
 0 
 0 
 0 
 0 
 0 
 0 
 0 
 0 
 0 
 0 
 0 
 0 
 0 
 0 
 0 
 0 
 
 
 51 
 0 
 0 
 0 
 0 
 0 
 0 
 0 
 0 
 0 
 0 
 0 
 0 
 0 
 0 
 0 
 0 
 0 
 0 
 0 
 0 
 0 
 0 
 0 
 0 
 0 
 0 
 0 
 0 
 0 
 0 
 0 
 0 
 0 
 0 
 0 
 0 
 0 
 0 
 0 
 0 
 0 
 0 
 0 
 0 
 0 
 0 
 0 
 0 
 0 
 0 
 0 
 8 
 0 
 0 
 0 
 0 
 0 
 0 
 0 
 0 
 0 
 0 
 0 
 0 
 0 
 0 
 0 
 0 
 0 
 0 
 0 
 0 
 0 
 0 
 0 
 0 
 0 
 0 
 0 
 0 
 0 
 0 
 0 
 0 
 0 
 0 
 0 
 0 
 0 
 0 
 0 
 0 
 0 
 0 
 0 
 0 
 0 
 0 
 0 
 0 
 0 
 0 
 0 
 0 
 0 
 0 
 0 
 0 
 0 
 0 
 0 
 0 
 0 
 0 
 0 
 0 
 0 
 0 
 0 
 0 
 0 
 0 
 0 
 0 
 0 
 0 
 0 
 0 
 0 
 0 
 0 
 0 
 0 
 0 
 0 
 0 
 0 
 0 
 0 
 0 
 0 
 0 
 0 
 0 
 0 
 0 
 0 
 0 
 0 
 0 
 0 
 0 
 0 
 0 
 0 
 0 
 0 
 0 
 0 
 0 
 0 
 0 
 0 
 0 
 0 
 0 
 0 
 0 
 0 
 0 
 0 
 0 
 0 
 0 
 0 
 0 
 0 
 0 
 0 
 0 
 0 
 0 
 0 
 0 
 0 
 0 
 0 
 0 
 0 
 0 
 0 
 0 
 0 
 0 
 0 
 0 
 0 
 0 
 0 
 0 
 0 
 0 
 0 
 0 
 0 
 0 
 0 
 0 
 0 
 0 
 0 
 0 
 0 
 0 
 0 
 0 
 0 
 0 
 0 
 0 
 0 
 0 
 0 
 0 
 0 
 0 
 0 
 0 
 0 
 0 
 0 
 0 
 0 
 0 
 0 
 0 
 0 
 0 
 0 
 0 
 0 
 0 
 0 
 0 
 0 
 0 
 0 
 0 
 0 
 0 
 0 
 0 
 0 
 0 
 0 
 0 
 0 
 0 
 0 
 0 
 0 
 0 
 0 
 0 
 0 
 0 
 0 
 0 
 0 
 0 
 0 
 0 
 0 
 0 
 0 
 0 
 0 
 0 
 0 
 0 
 0 
 0 
 0 
 0 
 0 
 0 
 0 
 0 
 0 
 0 
 0 
 0 
 0 
 0 
 0 
 0 
 0 
 0 
 0 
 0 
 0 
 0 
 0 
 0 
 0 
 0 
 0 
 0 
 0 
 0 
 0 
 0 
 0 
 0 
 0 
 0 
 0 
 0 
 0 
 0 
 0 
 0 
 0 
 0 
 0 
 0 
 0 
 0 
 0 
 0 
 0 
 0 
 0 
 0 
 0 
 0 
 0 
 0 
 0 
 0 
 0 
 0 
 0 
 0 
 0 
 0 
 0 
 0 
 0 
 0 
 0 
 0 
 0 
 0 
 0 
 0 
 0 
 0 
 0 
 0 
 0 
 0 
 0 
 0 
 0 
 0 
 0 
 0 
 0 
 0 
 0 
 0 
 0 
 0 
 0 
 0 
 0 
 0 
 0 
 0 
 0 
 0 
 0 
 0 
 0 
 0 
 0 
 0 
 0 
 0 
 0 
 0 
 0 
 0 
 0 
 0 
 0 
 0 
 0 
 0 
 0 
 0 
 0 
 0 
 0 
 0 
 0 
 0 
 0 
 0 
 0 
 0 
 0 
 0 
 0 
 0 
 0 
 0 
 0 
 0 
 0 
 0 
 0 
 0 
 0 
 0 
 0 
 0 
 0 
 0 
 0 
 0 
 0 
 0 
 0 
 0 
 0 
 0 
 0 
 0 
 0 
 0 
 0 
 0 
 0 
 0 
 0 
 0 
 0 
 0 
 0 
 0 
 0 
 0 
 0 
 0 
 0 
 0 
 0 
 0 
 0 
 0 
 0 
 0 
 0 
 0 
 0 
 0 
 0 
 0 
 0 
 0 
 0 
 0 
 0 
 0 
 0 
 0 
 0 
 0 
 0 
 0 
 0 
 0 
 0 
 0 
 0 
 0 
 0 
 0 
 0 
 0 
 0 
 0 
 0 
 0 
 0 
 0 
 0 
 0 
 0 
 0 
 0 
 0 
 0 
 0 
 0 
 0 
 0 
 0 
 0 
 0 
 0 
 0 
 0 
 0 
 0 
 0 
 0 
 0 
 0 
 0 
 0 
 0 
 0 
 0 
 0 
 0 
 0 
 0 
 0 
 0 
 0 
 0 
 0 
 0 
 0 
 0 
 0 
 0 
 0 
 0 
 0 
 0 
 0 
 0 
 0 
 0 
 0 
 0 
 0 
 0 
 0 
 0 
 0 
 0 
 0 
 0 
 0 
 0 
 0 
 0 
 0 
 0 
 0 
 0 
 0 
 0 
 0 
 0 
 0 
 0 
 0 
 0 
 0 
 0 
 0 
 0 
 0 
 0 
 0 
 0 
 0 
 0 
 0 
 0 
 0 
 0 
 0 
 0 
 0 
 0 
 0 
 0 
 0 
 0 
 0 
 0 
 0 
 0 
 0 
 0 
 0 
 0 
 0 
 0 
 0 
 0 
 0 
 0 
 0 
 0 
 0 
 0 
 0 
 0 
 0 
 0 
 0 
 0 
 0 
 0 
 0 
 0 
 0 
 0 
 0 
 0 
 0 
 0 
 0 
 0 
 0 
 0 
 0 
 0 
 0 
 0 
 0 
 0 
 0 
 0 
 0 
 0 
 0 
 0 
 0 
 0 
 0 
 0 
 0 
 0 
 0 
 0 
 0 
 0 
 0 
 0 
 0 
 0 
 0 
 0 
 0 
 0 
 0 
 0 
 0 
 0 
 0 
 0 
 0 
 0 
 0 
 0 
 0 
 0 
 0 
 0 
 0 
 0 
 0 
 0 
 0 
 0 
 0 
 0 
 0 
 0 
 0 
 0 
 0 
 0 
 0 
 0 
 0 
 0 
 0 
 0 
 0 
 0 
 0 
 0 
 0 
 0 
 0 
 0 
 0 
 0 
 0 
 0 
 0 
 0 
 0 
 0 
 0 
 0 
 0 
 0 
 0 
 0 
 0 
 0 
 0 
 0 
 0 
 0 
 0 
 0 
 0 
 0 
 0 
 0 
 0 
 0 
 0 
 0 
 0 
 0 
 0 
 0 
 0 
 0 
 0 
 0 
 0 
 0 
 0 
 0 
 0 
 0 
 0 
 0 
 0 
 0 
 0 
 0 
 0 
 0 
 0 
 0 
 0 
 0 
 0 
 0 
 0 
 0 
 0 
 0 
 0 
 0 
 0 
 0 
 0 
 0 
 0 
 0 
 0 
 0 
 0 
 0 
 0 
 0 
 0 
 0 
 0 
 0 
 0 
 0 
 0 
 0 
 0 
 0 
 0 
 0 
 0 
 0 
 0 
 0 
 0 
 0 
 0 
 0 
 0 
 0 
 0 
 0 
 0 
 0 
 0 
 0 
 0 
 0 
 0 
 0 
 0 
 0 
 0 
 0 
 0 
 0 
 0 
 0 
 0 
 0 
 0 
 0 
 
 
 52 
 0 
 0 
 0 
 0 
 0 
 0 
 0 
 0 
 0 
 0 
 0 
 0 
 0 
 0 
 0 
 0 
 0 
 0 
 0 
 0 
 0 
 0 
 0 
 0 
 0 
 0 
 0 
 0 
 0 
 0 
 0 
 0 
 0 
 0 
 0 
 0 
 0 
 0 
 0 
 0 
 0 
 0 
 0 
 0 
 0 
 0 
 0 
 0 
 0 
 0 
 0 
 0 
 0 
 0 
 0 
 0 
 0 
 0 
 0 
 0 
 0 
 0 
 0 
 0 
 0 
 0 
 0 
 0 
 0 
 0 
 0 
 0 
 0 
 0 
 0 
 0 
 0 
 0 
 0 
 0 
 0 
 0 
 0 
 0 
 0 
 0 
 0 
 0 
 0 
 0 
 0 
 0 
 0 
 0 
 0 
 0 
 0 
 0 
 0 
 0 
 0 
 0 
 0 
 0 
 0 
 0 
 0 
 0 
 0 
 0 
 0 
 0 
 0 
 0 
 0 
 0 
 0 
 0 
 0 
 0 
 0 
 0 
 0 
 0 
 0 
 0 
 0 
 0 
 0 
 0 
 0 
 0 
 0 
 0 
 0 
 0 
 0 
 0 
 0 
 0 
 0 
 0 
 0 
 0 
 0 
 0 
 0 
 0 
 0 
 0 
 0 
 0 
 0 
 0 
 0 
 0 
 0 
 0 
 0 
 0 
 0 
 0 
 0 
 0 
 0 
 0 
 0 
 0 
 0 
 0 
 0 
 0 
 0 
 0 
 0 
 0 
 0 
 0 
 0 
 0 
 0 
 0 
 0 
 0 
 0 
 0 
 0 
 0 
 0 
 0 
 0 
 0 
 0 
 0 
 0 
 0 
 0 
 0 
 0 
 0 
 0 
 0 
 0 
 0 
 0 
 0 
 0 
 0 
 0 
 0 
 0 
 0 
 0 
 0 
 0 
 0 
 0 
 0 
 0 
 0 
 0 
 0 
 0 
 0 
 0 
 0 
 0 
 0 
 0 
 0 
 0 
 0 
 0 
 0 
 0 
 0 
 0 
 0 
 0 
 0 
 0 
 0 
 0 
 0 
 0 
 0 
 0 
 0 
 0 
 0 
 0 
 0 
 0 
 0 
 0 
 0 
 0 
 0 
 0 
 0 
 0 
 0 
 0 
 0 
 0 
 0 
 0 
 0 
 0 
 0 
 0 
 0 
 0 
 0 
 0 
 0 
 0 
 0 
 0 
 0 
 0 
 0 
 0 
 0 
 0 
 0 
 0 
 0 
 0 
 0 
 0 
 0 
 0 
 0 
 0 
 0 
 0 
 0 
 0 
 0 
 0 
 0 
 0 
 0 
 0 
 0 
 0 
 0 
 0 
 0 
 0 
 0 
 0 
 0 
 0 
 0 
 0 
 0 
 0 
 0 
 0 
 0 
 0 
 0 
 0 
 0 
 0 
 0 
 0 
 0 
 0 
 0 
 0 
 0 
 0 
 3 
 1 
 0 
 0 
 0 
 0 
 0 
 0 
 0 
 0 
 0 
 0 
 0 
 0 
 0 
 0 
 0 
 0 
 0 
 0 
 0 
 0 
 0 
 0 
 0 
 0 
 0 
 0 
 0 
 0 
 0 
 0 
 0 
 0 
 0 
 0 
 0 
 0 
 0 
 0 
 0 
 0 
 0 
 0 
 0 
 0 
 0 
 0 
 0 
 0 
 0 
 0 
 0 
 0 
 0 
 0 
 0 
 0 
 0 
 0 
 0 
 0 
 0 
 0 
 0 
 0 
 0 
 0 
 0 
 0 
 0 
 0 
 0 
 0 
 0 
 0 
 0 
 0 
 0 
 0 
 0 
 0 
 0 
 0 
 0 
 0 
 0 
 0 
 0 
 0 
 0 
 0 
 0 
 0 
 0 
 0 
 0 
 0 
 0 
 0 
 0 
 0 
 0 
 0 
 0 
 0 
 0 
 0 
 0 
 0 
 0 
 0 
 0 
 0 
 0 
 0 
 0 
 0 
 0 
 0 
 0 
 0 
 0 
 0 
 0 
 0 
 0 
 0 
 0 
 0 
 0 
 0 
 0 
 0 
 0 
 0 
 0 
 0 
 0 
 0 
 0 
 0 
 0 
 0 
 0 
 0 
 0 
 0 
 0 
 0 
 0 
 0 
 0 
 0 
 0 
 0 
 0 
 0 
 0 
 0 
 0 
 0 
 0 
 0 
 0 
 0 
 0 
 0 
 0 
 0 
 0 
 0 
 0 
 0 
 0 
 0 
 0 
 0 
 0 
 0 
 0 
 0 
 0 
 0 
 0 
 0 
 0 
 0 
 0 
 0 
 0 
 0 
 0 
 0 
 0 
 0 
 0 
 0 
 0 
 0 
 0 
 0 
 0 
 0 
 0 
 0 
 0 
 0 
 0 
 0 
 0 
 0 
 0 
 0 
 0 
 0 
 0 
 0 
 0 
 0 
 0 
 0 
 0 
 0 
 0 
 0 
 0 
 0 
 0 
 0 
 0 
 0 
 0 
 0 
 0 
 0 
 0 
 0 
 0 
 0 
 0 
 0 
 0 
 0 
 0 
 0 
 0 
 0 
 0 
 0 
 0 
 0 
 0 
 0 
 0 
 0 
 0 
 0 
 0 
 0 
 0 
 0 
 0 
 0 
 0 
 0 
 0 
 0 
 0 
 0 
 0 
 0 
 0 
 0 
 0 
 0 
 0 
 0 
 0 
 0 
 0 
 0 
 0 
 0 
 0 
 0 
 0 
 0 
 0 
 0 
 0 
 0 
 0 
 0 
 0 
 0 
 0 
 0 
 0 
 0 
 0 
 0 
 0 
 0 
 0 
 0 
 0 
 0 
 0 
 0 
 0 
 0 
 0 
 0 
 0 
 0 
 0 
 0 
 0 
 0 
 0 
 0 
 0 
 0 
 0 
 0 
 0 
 0 
 0 
 0 
 0 
 0 
 0 
 0 
 0 
 0 
 0 
 0 
 0 
 0 
 0 
 0 
 0 
 0 
 0 
 0 
 0 
 0 
 0 
 0 
 0 
 0 
 0 
 0 
 0 
 0 
 0 
 0 
 0 
 0 
 0 
 0 
 0 
 0 
 0 
 0 
 0 
 0 
 0 
 0 
 0 
 0 
 0 
 0 
 0 
 0 
 0 
 0 
 0 
 0 
 0 
 0 
 0 
 0 
 0 
 0 
 0 
 0 
 0 
 0 
 0 
 0 
 0 
 0 
 0 
 0 
 0 
 0 
 0 
 0 
 0 
 0 
 0 
 0 
 0 
 0 
 0 
 0 
 0 
 0 
 0 
 0 
 0 
 0 
 0 
 0 
 0 
 0 
 0 
 0 
 0 
 0 
 0 
 0 
 0 
 0 
 0 
 0 
 0 
 0 
 0 
 0 
 0 
 0 
 0 
 0 
 0 
 0 
 0 
 0 
 0 
 0 
 0 
 0 
 0 
 0 
 0 
 0 
 0 
 0 
 0 
 0 
 0 
 0 
 0 
 0 
 0 
 0 
 0 
 0 
 0 
 0 
 0 
 0 
 0 
 0 
 0 
 0 
 0 
 0 
 0 
 0 
 0 
 0 
 0 
 0 
 0 
 0 
 0 
 0 
 0 
 0 
 0 
 0 
 0 
 0 
 0 
 0 
 0 
 0 
 0 
 
 
 53 
 0 
 0 
 0 
 0 
 0 
 0 
 0 
 0 
 0 
 0 
 0 
 0 
 0 
 0 
 0 
 0 
 0 
 0 
 0 
 0 
 0 
 0 
 0 
 0 
 0 
 0 
 0 
 0 
 0 
 0 
 0 
 0 
 0 
 0 
 0 
 0 
 0 
 0 
 0 
 0 
 0 
 0 
 0 
 0 
 0 
 0 
 0 
 0 
 0 
 0 
 0 
 0 
 0 
 22 
 0 
 0 
 0 
 0 
 0 
 0 
 0 
 0 
 0 
 0 
 0 
 0 
 0 
 0 
 0 
 0 
 0 
 0 
 0 
 0 
 0 
 0 
 0 
 0 
 0 
 0 
 0 
 0 
 0 
 0 
 0 
 0 
 0 
 0 
 0 
 0 
 0 
 0 
 0 
 0 
 0 
 0 
 0 
 0 
 0 
 0 
 0 
 0 
 0 
 0 
 0 
 0 
 0 
 0 
 0 
 0 
 0 
 0 
 0 
 0 
 0 
 0 
 0 
 0 
 0 
 0 
 0 
 0 
 0 
 0 
 0 
 0 
 0 
 0 
 0 
 0 
 0 
 0 
 0 
 0 
 0 
 0 
 0 
 0 
 0 
 0 
 0 
 0 
 0 
 0 
 0 
 0 
 0 
 0 
 0 
 0 
 0 
 0 
 0 
 0 
 0 
 0 
 0 
 0 
 0 
 0 
 0 
 0 
 0 
 0 
 0 
 0 
 0 
 0 
 0 
 0 
 0 
 0 
 0 
 0 
 0 
 0 
 0 
 0 
 0 
 0 
 0 
 0 
 0 
 0 
 0 
 0 
 0 
 0 
 0 
 0 
 0 
 0 
 0 
 0 
 0 
 0 
 0 
 0 
 0 
 0 
 0 
 0 
 0 
 0 
 0 
 0 
 0 
 0 
 0 
 0 
 0 
 0 
 0 
 0 
 0 
 0 
 0 
 0 
 0 
 0 
 0 
 0 
 0 
 0 
 0 
 0 
 0 
 0 
 0 
 0 
 0 
 0 
 0 
 0 
 0 
 0 
 0 
 0 
 0 
 0 
 0 
 0 
 0 
 0 
 0 
 0 
 0 
 0 
 0 
 0 
 0 
 0 
 0 
 0 
 0 
 0 
 0 
 0 
 0 
 0 
 0 
 0 
 0 
 0 
 0 
 0 
 0 
 0 
 0 
 0 
 0 
 0 
 0 
 0 
 0 
 0 
 0 
 0 
 0 
 0 
 0 
 0 
 0 
 0 
 0 
 0 
 0 
 0 
 0 
 0 
 0 
 0 
 0 
 0 
 0 
 0 
 0 
 0 
 0 
 0 
 0 
 0 
 0 
 0 
 0 
 0 
 0 
 0 
 0 
 0 
 0 
 0 
 0 
 0 
 0 
 0 
 0 
 0 
 0 
 0 
 0 
 0 
 0 
 0 
 0 
 0 
 0 
 0 
 0 
 0 
 0 
 0 
 0 
 0 
 0 
 0 
 0 
 0 
 0 
 0 
 0 
 0 
 0 
 0 
 0 
 0 
 0 
 0 
 0 
 0 
 0 
 0 
 0 
 0 
 0 
 0 
 0 
 0 
 0 
 0 
 0 
 0 
 0 
 0 
 0 
 0 
 0 
 0 
 0 
 0 
 0 
 0 
 0 
 0 
 0 
 0 
 0 
 0 
 0 
 0 
 0 
 0 
 0 
 0 
 0 
 0 
 0 
 0 
 0 
 0 
 0 
 0 
 0 
 0 
 0 
 0 
 0 
 0 
 0 
 0 
 0 
 0 
 0 
 0 
 0 
 0 
 0 
 0 
 0 
 0 
 0 
 0 
 0 
 0 
 0 
 0 
 0 
 0 
 0 
 0 
 0 
 0 
 0 
 0 
 0 
 0 
 0 
 0 
 0 
 0 
 0 
 0 
 0 
 0 
 0 
 0 
 0 
 0 
 0 
 0 
 0 
 0 
 0 
 0 
 0 
 0 
 0 
 0 
 0 
 0 
 0 
 0 
 0 
 0 
 0 
 0 
 0 
 0 
 0 
 0 
 0 
 0 
 0 
 0 
 0 
 0 
 0 
 0 
 0 
 0 
 0 
 0 
 0 
 0 
 0 
 0 
 0 
 0 
 0 
 0 
 0 
 0 
 0 
 0 
 0 
 0 
 0 
 0 
 0 
 0 
 0 
 0 
 0 
 0 
 0 
 0 
 0 
 0 
 0 
 0 
 0 
 0 
 0 
 0 
 0 
 0 
 0 
 0 
 0 
 0 
 0 
 0 
 0 
 0 
 0 
 0 
 0 
 0 
 0 
 0 
 0 
 0 
 0 
 0 
 0 
 0 
 0 
 0 
 0 
 0 
 0 
 0 
 0 
 0 
 0 
 0 
 0 
 0 
 0 
 0 
 0 
 0 
 0 
 0 
 0 
 0 
 0 
 0 
 0 
 0 
 0 
 0 
 0 
 0 
 0 
 0 
 0 
 0 
 0 
 0 
 0 
 0 
 0 
 0 
 0 
 0 
 0 
 0 
 0 
 0 
 0 
 0 
 0 
 0 
 0 
 0 
 0 
 0 
 0 
 0 
 0 
 0 
 0 
 0 
 0 
 0 
 0 
 0 
 0 
 0 
 0 
 0 
 0 
 0 
 0 
 0 
 0 
 0 
 0 
 0 
 0 
 0 
 0 
 0 
 0 
 0 
 0 
 0 
 0 
 0 
 0 
 0 
 0 
 0 
 0 
 0 
 0 
 0 
 0 
 0 
 0 
 0 
 0 
 0 
 0 
 0 
 0 
 0 
 0 
 0 
 0 
 0 
 0 
 0 
 0 
 0 
 0 
 0 
 0 
 0 
 0 
 0 
 0 
 0 
 0 
 0 
 0 
 0 
 0 
 0 
 0 
 0 
 0 
 0 
 0 
 0 
 0 
 0 
 0 
 0 
 0 
 0 
 0 
 0 
 0 
 0 
 0 
 0 
 0 
 0 
 0 
 0 
 0 
 0 
 0 
 0 
 0 
 0 
 0 
 0 
 0 
 0 
 0 
 0 
 0 
 0 
 0 
 0 
 0 
 0 
 0 
 0 
 0 
 0 
 0 
 0 
 0 
 0 
 0 
 0 
 0 
 0 
 0 
 0 
 0 
 0 
 0 
 0 
 0 
 0 
 0 
 0 
 0 
 0 
 0 
 0 
 0 
 0 
 0 
 0 
 0 
 0 
 0 
 0 
 0 
 0 
 0 
 0 
 0 
 0 
 0 
 0 
 0 
 0 
 0 
 0 
 0 
 0 
 0 
 0 
 0 
 0 
 0 
 0 
 0 
 0 
 0 
 0 
 0 
 0 
 0 
 0 
 0 
 0 
 0 
 0 
 0 
 0 
 0 
 0 
 0 
 0 
 0 
 0 
 0 
 0 
 0 
 0 
 0 
 0 
 0 
 0 
 0 
 0 
 0 
 0 
 0 
 0 
 0 
 0 
 0 
 0 
 0 
 0 
 0 
 0 
 0 
 0 
 0 
 0 
 0 
 0 
 0 
 0 
 0 
 0 
 0 
 0 
 0 
 0 
 0 
 0 
 0 
 0 
 0 
 0 
 0 
 0 
 0 
 0 
 0 
 0 
 0 
 0 
 0 
 0 
 0 
 0 
 0 
 0 
 0 
 0 
 0 
 0 
 0 
 0 
 
 
 54 
 0 
 0 
 0 
 0 
 0 
 0 
 0 
 0 
 0 
 0 
 0 
 0 
 0 
 0 
 0 
 0 
 0 
 0 
 0 
 0 
 0 
 0 
 0 
 0 
 0 
 0 
 0 
 0 
 0 
 0 
 0 
 0 
 0 
 0 
 0 
 0 
 0 
 0 
 0 
 0 
 0 
 0 
 0 
 0 
 0 
 0 
 0 
 0 
 0 
 0 
 0 
 0 
 0 
 0 
 6 
 0 
 0 
 0 
 0 
 0 
 0 
 0 
 0 
 0 
 0 
 0 
 0 
 0 
 0 
 0 
 0 
 0 
 0 
 0 
 0 
 0 
 0 
 0 
 0 
 0 
 0 
 0 
 0 
 0 
 0 
 0 
 0 
 0 
 0 
 0 
 0 
 0 
 0 
 0 
 0 
 0 
 0 
 0 
 0 
 0 
 0 
 0 
 0 
 0 
 0 
 0 
 0 
 0 
 0 
 0 
 0 
 0 
 0 
 0 
 0 
 0 
 0 
 0 
 0 
 0 
 0 
 0 
 0 
 0 
 0 
 0 
 0 
 0 
 0 
 0 
 0 
 0 
 0 
 0 
 0 
 0 
 0 
 0 
 0 
 0 
 0 
 0 
 0 
 0 
 0 
 0 
 0 
 0 
 0 
 0 
 0 
 0 
 0 
 0 
 0 
 0 
 0 
 0 
 0 
 0 
 0 
 0 
 0 
 0 
 0 
 0 
 0 
 0 
 0 
 0 
 0 
 0 
 0 
 0 
 0 
 0 
 0 
 0 
 0 
 0 
 0 
 0 
 0 
 0 
 0 
 0 
 0 
 0 
 0 
 0 
 0 
 0 
 0 
 0 
 0 
 0 
 0 
 0 
 0 
 0 
 0 
 0 
 0 
 0 
 0 
 0 
 0 
 0 
 0 
 0 
 0 
 0 
 0 
 0 
 0 
 0 
 0 
 0 
 0 
 0 
 0 
 0 
 0 
 0 
 0 
 0 
 0 
 0 
 0 
 0 
 0 
 0 
 0 
 0 
 0 
 0 
 0 
 0 
 0 
 0 
 0 
 0 
 0 
 0 
 0 
 0 
 0 
 0 
 0 
 0 
 0 
 0 
 0 
 0 
 0 
 0 
 0 
 0 
 0 
 0 
 0 
 0 
 0 
 0 
 0 
 0 
 0 
 0 
 0 
 0 
 0 
 0 
 0 
 0 
 0 
 0 
 0 
 0 
 0 
 0 
 0 
 0 
 0 
 0 
 0 
 0 
 0 
 0 
 0 
 0 
 0 
 0 
 0 
 0 
 0 
 0 
 0 
 0 
 0 
 0 
 0 
 0 
 0 
 0 
 0 
 0 
 0 
 0 
 0 
 0 
 0 
 0 
 0 
 0 
 0 
 0 
 0 
 0 
 0 
 0 
 0 
 0 
 0 
 0 
 0 
 0 
 0 
 0 
 0 
 0 
 0 
 0 
 0 
 0 
 0 
 0 
 0 
 0 
 0 
 0 
 0 
 0 
 0 
 0 
 0 
 0 
 0 
 0 
 0 
 0 
 0 
 0 
 0 
 0 
 0 
 0 
 0 
 0 
 0 
 0 
 0 
 0 
 0 
 0 
 0 
 0 
 0 
 0 
 0 
 0 
 0 
 0 
 0 
 0 
 0 
 0 
 0 
 0 
 0 
 0 
 0 
 0 
 0 
 0 
 0 
 0 
 0 
 0 
 0 
 0 
 0 
 0 
 0 
 0 
 0 
 0 
 0 
 0 
 0 
 0 
 0 
 0 
 0 
 0 
 0 
 0 
 0 
 0 
 0 
 0 
 0 
 0 
 0 
 0 
 0 
 0 
 0 
 0 
 0 
 0 
 0 
 0 
 0 
 0 
 0 
 0 
 0 
 0 
 0 
 0 
 0 
 0 
 0 
 0 
 0 
 0 
 0 
 0 
 0 
 0 
 0 
 0 
 0 
 0 
 0 
 0 
 0 
 0 
 0 
 0 
 0 
 0 
 0 
 0 
 0 
 0 
 0 
 0 
 0 
 0 
 0 
 0 
 0 
 0 
 0 
 0 
 0 
 0 
 0 
 0 
 0 
 0 
 0 
 0 
 0 
 0 
 0 
 0 
 0 
 0 
 0 
 0 
 0 
 0 
 0 
 0 
 0 
 0 
 0 
 0 
 0 
 0 
 0 
 0 
 0 
 0 
 0 
 0 
 0 
 0 
 0 
 0 
 0 
 0 
 0 
 0 
 0 
 0 
 0 
 0 
 0 
 0 
 0 
 0 
 0 
 0 
 0 
 0 
 0 
 0 
 0 
 0 
 0 
 0 
 0 
 0 
 0 
 0 
 0 
 0 
 0 
 0 
 0 
 0 
 0 
 0 
 0 
 0 
 0 
 0 
 0 
 0 
 0 
 0 
 0 
 0 
 0 
 0 
 0 
 0 
 0 
 0 
 0 
 0 
 0 
 0 
 0 
 0 
 0 
 0 
 0 
 0 
 0 
 0 
 0 
 0 
 0 
 0 
 0 
 0 
 0 
 0 
 0 
 0 
 0 
 0 
 0 
 0 
 0 
 0 
 0 
 0 
 0 
 0 
 0 
 0 
 0 
 0 
 0 
 0 
 0 
 0 
 0 
 0 
 0 
 0 
 0 
 0 
 0 
 0 
 0 
 0 
 0 
 0 
 0 
 0 
 0 
 0 
 0 
 0 
 0 
 0 
 0 
 0 
 0 
 0 
 0 
 0 
 0 
 0 
 0 
 0 
 0 
 0 
 0 
 0 
 0 
 0 
 0 
 0 
 0 
 0 
 0 
 0 
 0 
 0 
 0 
 0 
 0 
 0 
 0 
 0 
 0 
 0 
 0 
 0 
 0 
 0 
 0 
 0 
 0 
 0 
 0 
 0 
 0 
 0 
 0 
 0 
 0 
 0 
 0 
 0 
 0 
 0 
 0 
 0 
 0 
 0 
 0 
 0 
 0 
 0 
 0 
 0 
 0 
 0 
 0 
 0 
 0 
 0 
 0 
 0 
 0 
 0 
 0 
 0 
 0 
 0 
 0 
 0 
 0 
 0 
 0 
 0 
 0 
 0 
 0 
 0 
 0 
 0 
 0 
 0 
 0 
 0 
 0 
 0 
 0 
 0 
 0 
 0 
 0 
 0 
 0 
 0 
 0 
 0 
 0 
 0 
 0 
 0 
 0 
 0 
 0 
 0 
 0 
 0 
 0 
 0 
 0 
 0 
 0 
 0 
 0 
 0 
 0 
 0 
 0 
 0 
 0 
 0 
 0 
 0 
 0 
 0 
 0 
 0 
 0 
 0 
 0 
 0 
 0 
 0 
 0 
 0 
 0 
 0 
 0 
 0 
 0 
 0 
 0 
 0 
 0 
 0 
 0 
 0 
 0 
 0 
 0 
 0 
 0 
 0 
 0 
 0 
 0 
 0 
 0 
 0 
 0 
 0 
 0 
 0 
 0 
 0 
 0 
 0 
 0 
 0 
 0 
 0 
 0 
 0 
 0 
 0 
 0 
 0 
 0 
 0 
 0 
 0 
 0 
 0 
 0 
 0 
 0 
 0 
 0 
 0 
 0 
 0 
 0 
 0 
 0 
 0 
 0 
 0 
 0 
 0 
 0 
 0 
 0 
 0 
 0 
 0 
 0 
 0 
 
 
 55 
 0 
 0 
 0 
 0 
 0 
 0 
 0 
 0 
 0 
 0 
 0 
 0 
 0 
 0 
 0 
 0 
 0 
 0 
 0 
 0 
 0 
 0 
 0 
 0 
 0 
 0 
 0 
 0 
 0 
 0 
 0 
 0 
 0 
 0 
 0 
 0 
 0 
 0 
 0 
 0 
 0 
 0 
 0 
 0 
 0 
 0 
 0 
 0 
 0 
 0 
 0 
 0 
 0 
 11 
 0 
 36 
 0 
 0 
 0 
 0 
 0 
 0 
 0 
 0 
 0 
 0 
 0 
 0 
 0 
 0 
 0 
 0 
 0 
 0 
 0 
 0 
 0 
 0 
 0 
 0 
 0 
 0 
 0 
 0 
 0 
 0 
 0 
 0 
 0 
 0 
 0 
 0 
 0 
 0 
 0 
 0 
 0 
 0 
 0 
 0 
 0 
 0 
 0 
 0 
 0 
 0 
 0 
 0 
 0 
 0 
 0 
 0 
 0 
 0 
 0 
 0 
 0 
 0 
 0 
 0 
 0 
 0 
 0 
 0 
 0 
 0 
 0 
 0 
 0 
 0 
 0 
 0 
 0 
 0 
 0 
 0 
 0 
 0 
 0 
 0 
 0 
 0 
 0 
 0 
 0 
 0 
 0 
 0 
 0 
 0 
 0 
 0 
 0 
 0 
 0 
 0 
 0 
 0 
 0 
 0 
 0 
 0 
 0 
 0 
 0 
 0 
 0 
 0 
 0 
 0 
 0 
 0 
 0 
 0 
 0 
 0 
 0 
 0 
 0 
 0 
 0 
 0 
 0 
 0 
 0 
 0 
 0 
 0 
 0 
 0 
 0 
 0 
 0 
 0 
 0 
 0 
 0 
 0 
 0 
 0 
 0 
 0 
 0 
 0 
 0 
 0 
 0 
 0 
 0 
 0 
 0 
 0 
 0 
 0 
 0 
 0 
 0 
 0 
 0 
 0 
 0 
 0 
 0 
 0 
 0 
 0 
 0 
 0 
 0 
 0 
 0 
 0 
 0 
 0 
 0 
 0 
 0 
 0 
 0 
 0 
 0 
 0 
 0 
 0 
 0 
 0 
 0 
 0 
 0 
 0 
 0 
 0 
 0 
 0 
 0 
 0 
 0 
 0 
 0 
 0 
 0 
 0 
 0 
 0 
 0 
 0 
 0 
 0 
 0 
 0 
 0 
 0 
 0 
 0 
 0 
 0 
 0 
 0 
 0 
 0 
 0 
 0 
 0 
 0 
 0 
 0 
 0 
 0 
 0 
 0 
 0 
 0 
 0 
 0 
 0 
 0 
 0 
 0 
 0 
 0 
 0 
 0 
 0 
 0 
 0 
 0 
 0 
 0 
 0 
 0 
 0 
 0 
 0 
 0 
 0 
 0 
 0 
 0 
 0 
 0 
 0 
 0 
 0 
 0 
 0 
 0 
 0 
 0 
 0 
 0 
 0 
 0 
 0 
 0 
 0 
 0 
 0 
 0 
 0 
 0 
 0 
 0 
 0 
 0 
 0 
 0 
 0 
 0 
 0 
 0 
 0 
 0 
 0 
 0 
 0 
 0 
 0 
 0 
 0 
 0 
 0 
 0 
 0 
 0 
 0 
 0 
 0 
 0 
 0 
 0 
 0 
 0 
 0 
 0 
 0 
 0 
 0 
 0 
 0 
 0 
 0 
 0 
 0 
 0 
 0 
 0 
 0 
 0 
 0 
 0 
 0 
 0 
 0 
 0 
 0 
 0 
 0 
 0 
 0 
 0 
 0 
 0 
 0 
 0 
 0 
 0 
 0 
 0 
 0 
 0 
 0 
 0 
 0 
 0 
 0 
 0 
 0 
 0 
 0 
 0 
 0 
 0 
 0 
 0 
 0 
 0 
 0 
 0 
 0 
 0 
 0 
 0 
 0 
 0 
 0 
 0 
 0 
 0 
 0 
 0 
 0 
 0 
 0 
 0 
 0 
 0 
 0 
 0 
 0 
 0 
 0 
 0 
 0 
 0 
 0 
 0 
 0 
 0 
 0 
 0 
 0 
 0 
 0 
 0 
 0 
 0 
 0 
 0 
 0 
 0 
 0 
 0 
 0 
 0 
 0 
 0 
 0 
 0 
 0 
 0 
 0 
 0 
 0 
 0 
 0 
 0 
 0 
 0 
 0 
 0 
 0 
 0 
 0 
 0 
 0 
 0 
 0 
 0 
 0 
 0 
 0 
 0 
 0 
 0 
 0 
 0 
 0 
 0 
 0 
 0 
 0 
 0 
 0 
 0 
 0 
 0 
 0 
 0 
 0 
 0 
 0 
 0 
 0 
 0 
 0 
 0 
 0 
 0 
 0 
 0 
 0 
 0 
 0 
 0 
 0 
 0 
 0 
 0 
 0 
 0 
 0 
 0 
 0 
 0 
 0 
 0 
 0 
 0 
 0 
 0 
 0 
 0 
 0 
 0 
 0 
 0 
 0 
 0 
 0 
 0 
 0 
 0 
 0 
 0 
 0 
 0 
 0 
 0 
 0 
 0 
 0 
 0 
 0 
 0 
 0 
 0 
 0 
 0 
 0 
 0 
 0 
 0 
 0 
 0 
 0 
 0 
 0 
 0 
 0 
 0 
 0 
 0 
 0 
 0 
 0 
 0 
 0 
 0 
 0 
 0 
 0 
 0 
 0 
 0 
 0 
 0 
 0 
 0 
 0 
 0 
 0 
 0 
 0 
 0 
 0 
 0 
 0 
 0 
 0 
 0 
 0 
 0 
 0 
 0 
 0 
 0 
 0 
 0 
 0 
 0 
 0 
 0 
 0 
 0 
 0 
 0 
 0 
 0 
 0 
 0 
 0 
 0 
 0 
 0 
 0 
 0 
 0 
 0 
 0 
 0 
 0 
 0 
 0 
 0 
 0 
 0 
 0 
 0 
 0 
 0 
 0 
 0 
 0 
 0 
 0 
 0 
 0 
 0 
 0 
 0 
 0 
 0 
 0 
 0 
 0 
 0 
 0 
 0 
 0 
 0 
 0 
 0 
 0 
 0 
 0 
 0 
 0 
 0 
 0 
 0 
 0 
 0 
 0 
 0 
 0 
 0 
 0 
 0 
 0 
 0 
 0 
 0 
 0 
 0 
 0 
 0 
 0 
 0 
 0 
 0 
 0 
 0 
 0 
 0 
 0 
 0 
 0 
 0 
 0 
 0 
 0 
 0 
 0 
 0 
 0 
 0 
 0 
 0 
 0 
 0 
 0 
 0 
 0 
 0 
 0 
 0 
 0 
 0 
 0 
 0 
 0 
 0 
 0 
 0 
 0 
 0 
 0 
 0 
 0 
 0 
 0 
 0 
 0 
 0 
 0 
 0 
 0 
 0 
 0 
 0 
 0 
 0 
 0 
 0 
 0 
 0 
 0 
 0 
 0 
 0 
 0 
 0 
 0 
 0 
 0 
 0 
 0 
 0 
 0 
 0 
 0 
 0 
 0 
 0 
 0 
 0 
 0 
 0 
 0 
 0 
 0 
 0 
 0 
 0 
 0 
 0 
 0 
 0 
 0 
 0 
 0 
 0 
 0 
 0 
 0 
 0 
 0 
 0 
 0 
 0 
 0 
 0 
 0 
 0 
 0 
 0 
 0 
 0 
 0 
 0 
 0 
 0 
 0 
 0 
 0 
 0 
 
 
 56 
 0 
 0 
 0 
 0 
 0 
 0 
 0 
 0 
 0 
 0 
 0 
 0 
 0 
 0 
 0 
 0 
 0 
 0 
 0 
 0 
 0 
 0 
 0 
 0 
 0 
 0 
 0 
 0 
 0 
 0 
 0 
 0 
 0 
 0 
 0 
 0 
 0 
 0 
 0 
 0 
 0 
 0 
 0 
 0 
 0 
 0 
 0 
 0 
 0 
 0 
 0 
 0 
 0 
 0 
 0 
 0 
 6 
 0 
 0 
 0 
 0 
 0 
 0 
 0 
 0 
 0 
 0 
 0 
 0 
 0 
 0 
 0 
 0 
 0 
 0 
 0 
 0 
 0 
 0 
 0 
 0 
 0 
 0 
 0 
 0 
 0 
 0 
 0 
 0 
 0 
 0 
 0 
 0 
 0 
 0 
 0 
 0 
 0 
 0 
 0 
 0 
 0 
 0 
 0 
 0 
 0 
 0 
 0 
 0 
 0 
 0 
 0 
 0 
 0 
 0 
 0 
 0 
 0 
 0 
 0 
 0 
 0 
 0 
 0 
 0 
 0 
 0 
 0 
 0 
 0 
 0 
 0 
 0 
 0 
 0 
 0 
 0 
 0 
 0 
 0 
 0 
 0 
 0 
 0 
 0 
 0 
 0 
 0 
 0 
 0 
 0 
 0 
 0 
 0 
 0 
 0 
 0 
 0 
 0 
 0 
 0 
 0 
 0 
 0 
 0 
 0 
 0 
 0 
 0 
 0 
 0 
 0 
 0 
 0 
 0 
 0 
 0 
 0 
 0 
 0 
 0 
 0 
 0 
 0 
 0 
 0 
 0 
 0 
 0 
 0 
 0 
 0 
 0 
 0 
 0 
 0 
 0 
 0 
 0 
 0 
 0 
 0 
 0 
 0 
 0 
 0 
 0 
 0 
 0 
 0 
 0 
 0 
 0 
 0 
 0 
 0 
 0 
 0 
 0 
 0 
 0 
 0 
 0 
 0 
 0 
 0 
 0 
 0 
 0 
 0 
 0 
 0 
 0 
 0 
 0 
 0 
 0 
 0 
 0 
 0 
 0 
 0 
 0 
 0 
 0 
 0 
 0 
 0 
 0 
 0 
 0 
 0 
 0 
 0 
 0 
 0 
 0 
 0 
 0 
 0 
 0 
 0 
 0 
 0 
 0 
 0 
 0 
 0 
 0 
 0 
 0 
 0 
 0 
 0 
 0 
 0 
 0 
 0 
 0 
 0 
 0 
 0 
 0 
 0 
 0 
 0 
 0 
 0 
 0 
 0 
 0 
 0 
 0 
 0 
 0 
 0 
 0 
 0 
 0 
 0 
 0 
 0 
 0 
 0 
 0 
 0 
 0 
 0 
 0 
 0 
 0 
 0 
 0 
 0 
 0 
 0 
 0 
 0 
 0 
 0 
 0 
 0 
 0 
 0 
 0 
 0 
 0 
 0 
 0 
 0 
 0 
 0 
 0 
 0 
 0 
 0 
 0 
 0 
 0 
 0 
 0 
 0 
 0 
 0 
 0 
 0 
 0 
 0 
 0 
 0 
 0 
 0 
 0 
 0 
 0 
 0 
 0 
 0 
 0 
 0 
 0 
 0 
 0 
 0 
 0 
 0 
 0 
 0 
 0 
 0 
 0 
 0 
 0 
 0 
 0 
 0 
 0 
 0 
 0 
 0 
 0 
 0 
 0 
 0 
 0 
 0 
 0 
 0 
 0 
 0 
 0 
 0 
 0 
 0 
 0 
 0 
 0 
 0 
 0 
 0 
 0 
 0 
 0 
 0 
 0 
 0 
 0 
 0 
 0 
 0 
 0 
 0 
 0 
 0 
 0 
 0 
 0 
 0 
 0 
 0 
 0 
 0 
 0 
 0 
 0 
 0 
 0 
 0 
 0 
 0 
 0 
 0 
 0 
 0 
 0 
 0 
 0 
 0 
 0 
 0 
 0 
 0 
 0 
 0 
 0 
 0 
 0 
 0 
 0 
 0 
 0 
 0 
 0 
 0 
 0 
 0 
 0 
 0 
 0 
 0 
 0 
 0 
 0 
 0 
 0 
 0 
 0 
 0 
 0 
 0 
 0 
 0 
 0 
 0 
 0 
 0 
 0 
 0 
 0 
 0 
 0 
 0 
 0 
 0 
 0 
 0 
 0 
 0 
 0 
 0 
 0 
 0 
 0 
 0 
 0 
 0 
 0 
 0 
 0 
 0 
 0 
 0 
 0 
 0 
 0 
 0 
 0 
 0 
 0 
 0 
 0 
 0 
 0 
 0 
 0 
 0 
 0 
 0 
 0 
 0 
 0 
 0 
 0 
 0 
 0 
 0 
 0 
 0 
 0 
 0 
 0 
 0 
 0 
 0 
 0 
 0 
 0 
 0 
 0 
 0 
 0 
 0 
 0 
 0 
 0 
 0 
 0 
 0 
 0 
 0 
 0 
 0 
 0 
 0 
 0 
 0 
 0 
 0 
 0 
 0 
 0 
 0 
 0 
 0 
 0 
 0 
 0 
 0 
 0 
 0 
 0 
 0 
 0 
 0 
 0 
 0 
 0 
 0 
 0 
 0 
 0 
 0 
 0 
 0 
 0 
 0 
 0 
 0 
 0 
 0 
 0 
 0 
 0 
 0 
 0 
 0 
 0 
 0 
 0 
 0 
 0 
 0 
 0 
 0 
 0 
 0 
 0 
 0 
 0 
 0 
 0 
 0 
 0 
 0 
 0 
 0 
 0 
 0 
 0 
 0 
 0 
 0 
 0 
 0 
 0 
 0 
 0 
 0 
 0 
 0 
 0 
 0 
 0 
 0 
 0 
 0 
 0 
 0 
 0 
 0 
 0 
 0 
 0 
 0 
 0 
 0 
 0 
 0 
 0 
 0 
 0 
 0 
 0 
 0 
 0 
 0 
 0 
 0 
 0 
 0 
 0 
 0 
 0 
 0 
 0 
 0 
 0 
 0 
 0 
 0 
 0 
 0 
 0 
 0 
 0 
 0 
 0 
 0 
 0 
 0 
 0 
 0 
 0 
 0 
 0 
 0 
 0 
 0 
 0 
 0 
 0 
 0 
 0 
 0 
 0 
 0 
 0 
 0 
 0 
 0 
 0 
 0 
 0 
 0 
 0 
 0 
 0 
 0 
 0 
 0 
 0 
 0 
 0 
 0 
 0 
 0 
 0 
 0 
 0 
 0 
 0 
 0 
 0 
 0 
 0 
 0 
 0 
 0 
 0 
 0 
 0 
 0 
 0 
 0 
 0 
 0 
 0 
 0 
 0 
 0 
 0 
 0 
 0 
 0 
 0 
 0 
 0 
 0 
 0 
 0 
 0 
 0 
 0 
 0 
 0 
 0 
 0 
 0 
 0 
 0 
 0 
 0 
 0 
 0 
 0 
 0 
 0 
 0 
 0 
 0 
 0 
 0 
 0 
 0 
 0 
 0 
 0 
 0 
 0 
 0 
 0 
 0 
 0 
 0 
 0 
 0 
 0 
 0 
 0 
 0 
 0 
 0 
 0 
 0 
 0 
 0 
 0 
 0 
 0 
 0 
 0 
 0 
 0 
 0 
 0 
 0 
 0 
 0 
 0 
 0 
 0 
 0 
 0 
 0 
 0 
 0 
 0 
 0 
 0 
 0 
 0 
 0 
 0 
 0 
 0 
 0 
 
 
 57 
 0 
 0 
 0 
 0 
 0 
 0 
 0 
 0 
 0 
 0 
 0 
 0 
 0 
 0 
 0 
 0 
 0 
 0 
 0 
 0 
 0 
 0 
 0 
 0 
 0 
 0 
 0 
 0 
 0 
 0 
 0 
 0 
 0 
 0 
 0 
 0 
 0 
 0 
 0 
 0 
 0 
 0 
 0 
 0 
 0 
 0 
 0 
 0 
 0 
 0 
 0 
 0 
 0 
 0 
 0 
 0 
 0 
 10 
 0 
 0 
 0 
 0 
 0 
 0 
 0 
 0 
 0 
 0 
 0 
 0 
 0 
 0 
 0 
 0 
 0 
 0 
 0 
 0 
 0 
 0 
 0 
 0 
 0 
 0 
 0 
 0 
 0 
 0 
 0 
 0 
 0 
 0 
 0 
 0 
 0 
 0 
 0 
 0 
 0 
 0 
 0 
 0 
 0 
 0 
 0 
 0 
 0 
 0 
 0 
 0 
 0 
 0 
 0 
 0 
 0 
 0 
 0 
 0 
 0 
 0 
 0 
 0 
 0 
 0 
 0 
 0 
 0 
 0 
 0 
 0 
 0 
 0 
 0 
 0 
 0 
 0 
 0 
 0 
 0 
 0 
 0 
 0 
 0 
 0 
 0 
 0 
 0 
 0 
 0 
 0 
 0 
 0 
 0 
 0 
 0 
 0 
 0 
 0 
 0 
 0 
 0 
 0 
 0 
 0 
 0 
 0 
 0 
 0 
 0 
 0 
 0 
 0 
 0 
 0 
 0 
 0 
 0 
 0 
 0 
 0 
 0 
 0 
 0 
 0 
 0 
 0 
 0 
 0 
 0 
 0 
 0 
 0 
 0 
 0 
 0 
 0 
 0 
 0 
 0 
 0 
 0 
 0 
 0 
 0 
 0 
 0 
 0 
 0 
 0 
 0 
 0 
 0 
 0 
 0 
 0 
 0 
 0 
 0 
 0 
 0 
 0 
 0 
 0 
 0 
 0 
 0 
 0 
 0 
 0 
 0 
 0 
 0 
 0 
 0 
 0 
 0 
 0 
 0 
 0 
 0 
 0 
 0 
 0 
 0 
 0 
 0 
 0 
 0 
 0 
 0 
 0 
 0 
 0 
 0 
 0 
 0 
 0 
 0 
 0 
 0 
 0 
 0 
 0 
 0 
 0 
 0 
 0 
 0 
 0 
 0 
 0 
 0 
 0 
 0 
 0 
 0 
 0 
 0 
 0 
 0 
 0 
 0 
 0 
 0 
 0 
 0 
 0 
 0 
 0 
 0 
 0 
 0 
 0 
 0 
 0 
 0 
 0 
 0 
 0 
 0 
 0 
 0 
 0 
 0 
 0 
 0 
 0 
 0 
 0 
 0 
 0 
 0 
 0 
 0 
 0 
 0 
 0 
 0 
 0 
 0 
 0 
 0 
 0 
 0 
 0 
 0 
 0 
 0 
 0 
 0 
 0 
 0 
 0 
 0 
 0 
 0 
 0 
 0 
 0 
 0 
 0 
 0 
 0 
 0 
 0 
 0 
 0 
 0 
 0 
 0 
 0 
 0 
 0 
 0 
 0 
 0 
 0 
 0 
 0 
 0 
 0 
 0 
 0 
 0 
 0 
 0 
 0 
 0 
 0 
 0 
 0 
 0 
 0 
 0 
 0 
 0 
 0 
 0 
 0 
 0 
 0 
 0 
 0 
 0 
 0 
 0 
 0 
 0 
 0 
 0 
 0 
 0 
 0 
 0 
 0 
 0 
 0 
 0 
 0 
 0 
 0 
 0 
 0 
 0 
 0 
 0 
 0 
 0 
 0 
 0 
 0 
 0 
 0 
 0 
 0 
 0 
 0 
 0 
 0 
 0 
 0 
 0 
 0 
 0 
 0 
 0 
 0 
 0 
 0 
 0 
 0 
 0 
 0 
 0 
 0 
 0 
 0 
 0 
 0 
 0 
 0 
 0 
 0 
 0 
 0 
 0 
 0 
 0 
 0 
 0 
 0 
 0 
 0 
 0 
 0 
 0 
 0 
 0 
 0 
 0 
 0 
 0 
 0 
 0 
 0 
 0 
 0 
 0 
 0 
 0 
 0 
 0 
 0 
 0 
 0 
 0 
 0 
 0 
 0 
 0 
 0 
 0 
 0 
 0 
 0 
 0 
 0 
 0 
 0 
 0 
 0 
 0 
 0 
 0 
 0 
 0 
 0 
 0 
 0 
 0 
 0 
 0 
 0 
 0 
 0 
 0 
 0 
 0 
 0 
 0 
 0 
 0 
 0 
 0 
 0 
 0 
 0 
 0 
 0 
 0 
 0 
 0 
 0 
 0 
 0 
 0 
 0 
 0 
 0 
 0 
 0 
 0 
 0 
 0 
 0 
 0 
 0 
 0 
 0 
 0 
 0 
 0 
 0 
 0 
 0 
 0 
 0 
 0 
 0 
 0 
 0 
 0 
 0 
 0 
 0 
 0 
 0 
 0 
 0 
 0 
 0 
 0 
 0 
 0 
 0 
 0 
 0 
 0 
 0 
 0 
 0 
 0 
 0 
 0 
 0 
 0 
 0 
 0 
 0 
 0 
 0 
 0 
 0 
 0 
 0 
 0 
 0 
 0 
 0 
 0 
 0 
 0 
 0 
 0 
 0 
 0 
 0 
 0 
 0 
 0 
 0 
 0 
 0 
 0 
 0 
 0 
 0 
 0 
 0 
 0 
 0 
 0 
 0 
 0 
 0 
 0 
 0 
 0 
 0 
 0 
 0 
 0 
 0 
 0 
 0 
 0 
 0 
 0 
 0 
 0 
 0 
 0 
 0 
 0 
 0 
 0 
 0 
 0 
 0 
 0 
 0 
 0 
 0 
 0 
 0 
 0 
 0 
 0 
 0 
 0 
 0 
 0 
 0 
 0 
 0 
 0 
 0 
 0 
 0 
 0 
 0 
 0 
 0 
 0 
 0 
 0 
 0 
 0 
 0 
 0 
 0 
 0 
 0 
 0 
 0 
 0 
 0 
 0 
 0 
 0 
 0 
 0 
 0 
 0 
 0 
 0 
 0 
 0 
 0 
 0 
 0 
 0 
 0 
 0 
 0 
 0 
 0 
 0 
 0 
 0 
 0 
 0 
 0 
 0 
 0 
 0 
 0 
 0 
 0 
 0 
 0 
 0 
 0 
 0 
 0 
 0 
 0 
 0 
 0 
 0 
 0 
 0 
 0 
 0 
 0 
 0 
 0 
 0 
 0 
 0 
 0 
 0 
 0 
 0 
 0 
 0 
 0 
 0 
 0 
 0 
 0 
 0 
 0 
 0 
 0 
 0 
 0 
 0 
 0 
 0 
 0 
 0 
 0 
 0 
 0 
 0 
 0 
 0 
 0 
 0 
 0 
 0 
 0 
 0 
 0 
 0 
 0 
 0 
 0 
 0 
 0 
 0 
 0 
 0 
 0 
 0 
 0 
 0 
 0 
 0 
 0 
 0 
 0 
 0 
 0 
 0 
 0 
 0 
 0 
 0 
 0 
 0 
 0 
 0 
 0 
 0 
 0 
 0 
 0 
 0 
 0 
 0 
 0 
 0 
 0 
 0 
 0 
 0 
 0 
 0 
 0 
 0 
 0 
 0 
 0 
 0 
 0 
 0 
 0 
 0 
 0 
 0 
 0 
 0 
 0 
 0 
 
 
 58 
 0 
 0 
 0 
 0 
 0 
 0 
 0 
 0 
 0 
 0 
 0 
 0 
 0 
 0 
 0 
 0 
 0 
 0 
 0 
 0 
 0 
 0 
 0 
 0 
 0 
 0 
 0 
 0 
 0 
 0 
 0 
 0 
 0 
 0 
 0 
 0 
 0 
 0 
 0 
 0 
 0 
 0 
 0 
 0 
 0 
 0 
 0 
 0 
 0 
 0 
 0 
 0 
 0 
 0 
 0 
 0 
 0 
 0 
 15 
 0 
 0 
 0 
 0 
 0 
 0 
 0 
 0 
 0 
 0 
 0 
 0 
 0 
 0 
 0 
 0 
 0 
 0 
 0 
 0 
 0 
 0 
 0 
 0 
 0 
 0 
 0 
 0 
 0 
 0 
 0 
 0 
 0 
 0 
 0 
 0 
 0 
 0 
 0 
 0 
 0 
 0 
 0 
 0 
 0 
 0 
 0 
 0 
 0 
 0 
 0 
 0 
 0 
 0 
 0 
 0 
 0 
 0 
 0 
 0 
 0 
 0 
 0 
 0 
 0 
 0 
 0 
 0 
 0 
 0 
 0 
 0 
 0 
 0 
 0 
 0 
 0 
 0 
 0 
 0 
 0 
 0 
 0 
 0 
 0 
 0 
 0 
 0 
 0 
 0 
 0 
 0 
 0 
 0 
 0 
 0 
 0 
 0 
 0 
 0 
 0 
 0 
 0 
 0 
 0 
 0 
 0 
 0 
 0 
 0 
 0 
 0 
 0 
 0 
 0 
 0 
 0 
 0 
 0 
 0 
 0 
 0 
 0 
 0 
 0 
 0 
 0 
 0 
 0 
 0 
 0 
 0 
 0 
 0 
 0 
 0 
 0 
 0 
 0 
 0 
 0 
 0 
 0 
 0 
 0 
 0 
 0 
 0 
 0 
 0 
 0 
 0 
 0 
 0 
 0 
 0 
 0 
 0 
 0 
 0 
 0 
 0 
 0 
 0 
 0 
 0 
 0 
 0 
 0 
 0 
 0 
 0 
 0 
 0 
 0 
 0 
 0 
 0 
 0 
 0 
 0 
 0 
 0 
 0 
 0 
 0 
 0 
 0 
 0 
 0 
 0 
 0 
 0 
 0 
 0 
 0 
 0 
 0 
 0 
 0 
 0 
 0 
 0 
 0 
 0 
 0 
 0 
 0 
 0 
 0 
 0 
 0 
 0 
 0 
 0 
 0 
 0 
 0 
 0 
 0 
 0 
 0 
 0 
 0 
 0 
 0 
 0 
 0 
 0 
 0 
 0 
 0 
 0 
 0 
 0 
 0 
 0 
 0 
 0 
 0 
 0 
 0 
 0 
 0 
 0 
 0 
 0 
 0 
 0 
 0 
 0 
 0 
 0 
 0 
 0 
 0 
 0 
 0 
 0 
 0 
 0 
 0 
 0 
 0 
 0 
 0 
 0 
 0 
 0 
 0 
 0 
 0 
 0 
 0 
 0 
 0 
 0 
 0 
 0 
 0 
 0 
 0 
 0 
 0 
 0 
 0 
 0 
 0 
 0 
 0 
 0 
 0 
 0 
 0 
 0 
 0 
 0 
 0 
 0 
 0 
 0 
 0 
 0 
 0 
 0 
 0 
 0 
 0 
 0 
 0 
 0 
 0 
 0 
 0 
 0 
 0 
 0 
 0 
 0 
 0 
 0 
 0 
 0 
 0 
 0 
 0 
 0 
 0 
 0 
 0 
 0 
 0 
 0 
 0 
 0 
 0 
 0 
 0 
 0 
 0 
 0 
 0 
 0 
 0 
 0 
 0 
 0 
 0 
 0 
 0 
 0 
 0 
 0 
 0 
 0 
 0 
 0 
 0 
 0 
 0 
 0 
 0 
 0 
 0 
 0 
 0 
 0 
 0 
 0 
 0 
 0 
 0 
 0 
 0 
 0 
 0 
 0 
 0 
 0 
 0 
 0 
 0 
 0 
 0 
 0 
 0 
 0 
 0 
 0 
 0 
 0 
 0 
 0 
 0 
 0 
 0 
 0 
 0 
 0 
 0 
 0 
 0 
 0 
 0 
 0 
 0 
 0 
 0 
 0 
 0 
 0 
 0 
 0 
 0 
 0 
 0 
 0 
 0 
 0 
 0 
 0 
 0 
 0 
 0 
 0 
 0 
 0 
 0 
 0 
 0 
 0 
 0 
 0 
 0 
 0 
 0 
 0 
 0 
 0 
 0 
 0 
 0 
 0 
 0 
 0 
 0 
 0 
 0 
 0 
 0 
 0 
 0 
 0 
 0 
 0 
 0 
 0 
 0 
 0 
 0 
 0 
 0 
 0 
 0 
 0 
 0 
 0 
 0 
 0 
 0 
 0 
 0 
 0 
 0 
 0 
 0 
 0 
 0 
 0 
 0 
 0 
 0 
 0 
 0 
 0 
 0 
 0 
 0 
 0 
 0 
 0 
 0 
 0 
 0 
 0 
 0 
 0 
 0 
 0 
 0 
 0 
 0 
 0 
 0 
 0 
 0 
 0 
 0 
 0 
 0 
 0 
 0 
 0 
 0 
 0 
 0 
 0 
 0 
 0 
 0 
 0 
 0 
 0 
 0 
 0 
 0 
 0 
 0 
 0 
 0 
 0 
 0 
 0 
 0 
 0 
 0 
 0 
 0 
 0 
 0 
 0 
 0 
 0 
 0 
 0 
 0 
 0 
 0 
 0 
 0 
 0 
 0 
 0 
 0 
 0 
 0 
 0 
 0 
 0 
 0 
 0 
 0 
 0 
 0 
 0 
 0 
 0 
 0 
 0 
 0 
 0 
 0 
 0 
 0 
 0 
 0 
 0 
 0 
 0 
 0 
 0 
 0 
 0 
 0 
 0 
 0 
 0 
 0 
 0 
 0 
 0 
 0 
 0 
 0 
 0 
 0 
 0 
 0 
 0 
 0 
 0 
 0 
 0 
 0 
 0 
 0 
 0 
 0 
 0 
 0 
 0 
 0 
 0 
 0 
 0 
 0 
 0 
 0 
 0 
 0 
 0 
 0 
 0 
 0 
 0 
 0 
 0 
 0 
 0 
 0 
 0 
 0 
 0 
 0 
 0 
 0 
 0 
 0 
 0 
 0 
 0 
 0 
 0 
 0 
 0 
 0 
 0 
 0 
 0 
 0 
 0 
 0 
 0 
 0 
 0 
 0 
 0 
 0 
 0 
 0 
 0 
 0 
 0 
 0 
 0 
 0 
 0 
 0 
 0 
 0 
 0 
 0 
 0 
 0 
 0 
 0 
 0 
 0 
 0 
 0 
 0 
 0 
 0 
 0 
 0 
 0 
 0 
 0 
 0 
 0 
 0 
 0 
 0 
 0 
 0 
 0 
 0 
 0 
 0 
 0 
 0 
 0 
 0 
 0 
 0 
 0 
 0 
 0 
 0 
 0 
 0 
 0 
 0 
 0 
 0 
 0 
 0 
 0 
 0 
 0 
 0 
 0 
 0 
 0 
 0 
 0 
 0 
 0 
 0 
 0 
 0 
 0 
 0 
 0 
 0 
 0 
 0 
 0 
 0 
 0 
 0 
 0 
 0 
 0 
 0 
 0 
 0 
 0 
 0 
 0 
 0 
 0 
 0 
 0 
 0 
 0 
 0 
 0 
 0 
 0 
 0 
 0 
 0 
 0 
 0 
 0 
 0 
 
 
 59 
 0 
 0 
 0 
 0 
 0 
 0 
 0 
 0 
 0 
 0 
 0 
 0 
 0 
 0 
 0 
 0 
 0 
 0 
 0 
 0 
 0 
 0 
 0 
 0 
 0 
 0 
 0 
 0 
 0 
 0 
 0 
 0 
 0 
 0 
 0 
 0 
 0 
 0 
 0 
 0 
 0 
 0 
 0 
 0 
 0 
 0 
 0 
 0 
 0 
 0 
 0 
 0 
 0 
 0 
 0 
 0 
 0 
 0 
 0 
 8 
 0 
 0 
 0 
 0 
 0 
 0 
 0 
 0 
 0 
 0 
 0 
 0 
 0 
 0 
 0 
 0 
 0 
 0 
 0 
 0 
 0 
 0 
 0 
 0 
 0 
 0 
 0 
 0 
 0 
 0 
 0 
 0 
 0 
 0 
 0 
 0 
 0 
 0 
 0 
 0 
 0 
 0 
 0 
 0 
 0 
 0 
 0 
 0 
 0 
 0 
 0 
 0 
 0 
 0 
 0 
 0 
 0 
 0 
 0 
 0 
 0 
 0 
 0 
 0 
 0 
 0 
 0 
 0 
 0 
 0 
 0 
 0 
 0 
 0 
 0 
 0 
 0 
 0 
 0 
 0 
 0 
 0 
 0 
 0 
 0 
 0 
 0 
 0 
 0 
 0 
 0 
 0 
 0 
 0 
 0 
 0 
 0 
 0 
 0 
 0 
 0 
 0 
 0 
 0 
 0 
 0 
 0 
 0 
 0 
 0 
 0 
 0 
 0 
 0 
 0 
 0 
 0 
 0 
 0 
 0 
 0 
 0 
 0 
 0 
 0 
 0 
 0 
 0 
 0 
 0 
 0 
 0 
 0 
 0 
 0 
 0 
 0 
 0 
 0 
 0 
 0 
 0 
 0 
 0 
 0 
 0 
 0 
 0 
 0 
 0 
 0 
 0 
 0 
 0 
 0 
 0 
 0 
 0 
 0 
 0 
 0 
 0 
 0 
 0 
 0 
 0 
 0 
 0 
 0 
 0 
 0 
 0 
 0 
 0 
 0 
 0 
 0 
 0 
 0 
 0 
 0 
 0 
 0 
 0 
 0 
 0 
 0 
 0 
 0 
 0 
 0 
 0 
 0 
 0 
 0 
 0 
 0 
 0 
 0 
 0 
 0 
 0 
 0 
 0 
 0 
 0 
 0 
 0 
 0 
 0 
 0 
 0 
 0 
 0 
 0 
 0 
 0 
 0 
 0 
 0 
 0 
 0 
 0 
 0 
 0 
 0 
 0 
 0 
 0 
 0 
 0 
 0 
 0 
 0 
 0 
 0 
 0 
 0 
 0 
 0 
 0 
 0 
 0 
 0 
 0 
 0 
 0 
 0 
 0 
 0 
 0 
 0 
 0 
 0 
 0 
 0 
 0 
 0 
 0 
 0 
 0 
 0 
 0 
 0 
 0 
 0 
 0 
 0 
 0 
 0 
 0 
 0 
 0 
 0 
 0 
 0 
 0 
 0 
 0 
 0 
 0 
 0 
 0 
 0 
 0 
 0 
 0 
 0 
 0 
 0 
 0 
 0 
 0 
 0 
 0 
 0 
 0 
 0 
 0 
 0 
 0 
 0 
 0 
 0 
 0 
 0 
 0 
 0 
 0 
 0 
 0 
 0 
 0 
 0 
 0 
 0 
 0 
 0 
 0 
 0 
 0 
 0 
 0 
 0 
 0 
 0 
 0 
 0 
 0 
 0 
 0 
 0 
 0 
 0 
 0 
 0 
 0 
 0 
 0 
 0 
 0 
 0 
 0 
 0 
 0 
 0 
 0 
 0 
 0 
 0 
 0 
 0 
 0 
 0 
 0 
 0 
 0 
 0 
 0 
 0 
 0 
 0 
 0 
 0 
 0 
 0 
 0 
 0 
 0 
 0 
 0 
 0 
 0 
 0 
 0 
 0 
 0 
 0 
 0 
 0 
 0 
 0 
 0 
 0 
 0 
 0 
 0 
 0 
 0 
 0 
 0 
 0 
 0 
 0 
 0 
 0 
 0 
 0 
 0 
 0 
 0 
 0 
 0 
 0 
 0 
 0 
 0 
 0 
 0 
 0 
 0 
 0 
 0 
 0 
 0 
 0 
 0 
 0 
 0 
 0 
 0 
 0 
 0 
 0 
 0 
 0 
 0 
 0 
 0 
 0 
 0 
 0 
 0 
 0 
 0 
 0 
 0 
 0 
 0 
 0 
 0 
 0 
 0 
 0 
 0 
 0 
 0 
 0 
 0 
 0 
 0 
 0 
 0 
 0 
 0 
 0 
 0 
 0 
 0 
 0 
 0 
 0 
 0 
 0 
 0 
 0 
 0 
 0 
 0 
 0 
 0 
 0 
 0 
 0 
 0 
 0 
 0 
 0 
 0 
 0 
 0 
 0 
 0 
 0 
 0 
 0 
 0 
 0 
 0 
 0 
 0 
 0 
 0 
 0 
 0 
 0 
 0 
 0 
 0 
 0 
 0 
 0 
 0 
 0 
 0 
 0 
 0 
 0 
 0 
 0 
 0 
 0 
 0 
 0 
 0 
 0 
 0 
 0 
 0 
 0 
 0 
 0 
 0 
 0 
 0 
 0 
 0 
 0 
 0 
 0 
 0 
 0 
 0 
 0 
 0 
 0 
 0 
 0 
 0 
 0 
 0 
 0 
 0 
 0 
 0 
 0 
 0 
 0 
 0 
 0 
 0 
 0 
 0 
 0 
 0 
 0 
 0 
 0 
 0 
 0 
 0 
 0 
 0 
 0 
 0 
 0 
 0 
 0 
 0 
 0 
 0 
 0 
 0 
 0 
 0 
 0 
 0 
 0 
 0 
 0 
 0 
 0 
 0 
 0 
 0 
 0 
 0 
 0 
 0 
 0 
 0 
 0 
 0 
 0 
 0 
 0 
 0 
 0 
 0 
 0 
 0 
 0 
 0 
 0 
 0 
 0 
 0 
 0 
 0 
 0 
 0 
 0 
 0 
 0 
 0 
 0 
 0 
 0 
 0 
 0 
 0 
 0 
 0 
 0 
 0 
 0 
 0 
 0 
 0 
 0 
 0 
 0 
 0 
 0 
 0 
 0 
 0 
 0 
 0 
 0 
 0 
 0 
 0 
 0 
 0 
 0 
 0 
 0 
 0 
 0 
 0 
 0 
 0 
 0 
 0 
 0 
 0 
 0 
 0 
 0 
 0 
 0 
 0 
 0 
 0 
 0 
 0 
 0 
 0 
 0 
 0 
 0 
 0 
 0 
 0 
 0 
 0 
 0 
 0 
 0 
 0 
 0 
 0 
 0 
 0 
 0 
 0 
 0 
 0 
 0 
 0 
 0 
 0 
 0 
 0 
 0 
 0 
 0 
 0 
 0 
 0 
 0 
 0 
 0 
 0 
 0 
 0 
 0 
 0 
 0 
 0 
 0 
 0 
 0 
 0 
 0 
 0 
 0 
 0 
 0 
 0 
 0 
 0 
 0 
 0 
 0 
 0 
 0 
 0 
 0 
 0 
 0 
 0 
 0 
 0 
 0 
 0 
 0 
 0 
 0 
 0 
 0 
 0 
 0 
 0 
 0 
 0 
 0 
 0 
 0 
 0 
 0 
 0 
 0 
 0 
 0 
 0 
 0 
 0 
 0 
 0 
 0 
 0 
 0 
 0 
 0 
 
 
 60 
 0 
 0 
 0 
 0 
 0 
 0 
 0 
 0 
 0 
 0 
 0 
 0 
 0 
 0 
 0 
 0 
 0 
 0 
 0 
 0 
 0 
 0 
 0 
 0 
 0 
 0 
 0 
 0 
 0 
 0 
 0 
 0 
 0 
 0 
 0 
 0 
 0 
 0 
 0 
 0 
 0 
 0 
 0 
 0 
 0 
 0 
 0 
 0 
 0 
 0 
 0 
 0 
 0 
 0 
 0 
 0 
 0 
 0 
 0 
 0 
 1 
 0 
 0 
 0 
 0 
 0 
 0 
 0 
 0 
 0 
 0 
 0 
 0 
 0 
 0 
 0 
 0 
 0 
 0 
 0 
 0 
 0 
 0 
 0 
 0 
 0 
 0 
 0 
 0 
 0 
 0 
 0 
 0 
 0 
 0 
 0 
 0 
 0 
 0 
 0 
 0 
 0 
 0 
 0 
 0 
 0 
 0 
 0 
 0 
 0 
 0 
 0 
 0 
 0 
 0 
 0 
 0 
 0 
 0 
 0 
 0 
 0 
 0 
 0 
 0 
 0 
 0 
 0 
 0 
 0 
 0 
 0 
 0 
 0 
 0 
 0 
 0 
 0 
 0 
 0 
 0 
 0 
 0 
 0 
 0 
 0 
 0 
 0 
 0 
 0 
 0 
 0 
 0 
 0 
 0 
 0 
 0 
 0 
 0 
 0 
 0 
 0 
 0 
 0 
 0 
 0 
 0 
 0 
 0 
 0 
 0 
 0 
 0 
 0 
 0 
 0 
 0 
 0 
 0 
 0 
 0 
 0 
 0 
 0 
 0 
 0 
 0 
 0 
 0 
 0 
 0 
 0 
 0 
 0 
 0 
 0 
 0 
 0 
 0 
 0 
 0 
 0 
 0 
 0 
 0 
 0 
 0 
 0 
 0 
 0 
 0 
 0 
 0 
 0 
 0 
 0 
 0 
 0 
 0 
 0 
 0 
 0 
 0 
 0 
 0 
 0 
 0 
 0 
 0 
 0 
 0 
 0 
 0 
 0 
 0 
 0 
 0 
 0 
 0 
 0 
 0 
 0 
 0 
 0 
 0 
 0 
 0 
 0 
 0 
 0 
 0 
 0 
 0 
 0 
 0 
 0 
 0 
 0 
 0 
 0 
 0 
 0 
 0 
 0 
 0 
 0 
 0 
 0 
 0 
 0 
 0 
 0 
 0 
 0 
 0 
 0 
 0 
 0 
 0 
 0 
 0 
 0 
 0 
 0 
 0 
 0 
 0 
 0 
 0 
 0 
 0 
 0 
 0 
 0 
 0 
 0 
 0 
 0 
 0 
 0 
 0 
 0 
 0 
 0 
 0 
 0 
 0 
 0 
 0 
 0 
 0 
 0 
 0 
 0 
 0 
 0 
 0 
 0 
 0 
 0 
 0 
 0 
 0 
 0 
 0 
 0 
 0 
 0 
 0 
 0 
 0 
 0 
 0 
 0 
 0 
 0 
 0 
 0 
 0 
 0 
 0 
 0 
 0 
 0 
 0 
 0 
 0 
 0 
 0 
 0 
 0 
 0 
 0 
 0 
 0 
 0 
 0 
 0 
 0 
 0 
 0 
 0 
 0 
 0 
 0 
 0 
 0 
 0 
 0 
 0 
 0 
 0 
 0 
 0 
 0 
 0 
 0 
 0 
 0 
 0 
 0 
 0 
 0 
 0 
 0 
 0 
 0 
 0 
 0 
 0 
 0 
 0 
 0 
 0 
 0 
 0 
 0 
 0 
 0 
 0 
 0 
 0 
 0 
 0 
 0 
 0 
 0 
 0 
 0 
 0 
 0 
 0 
 0 
 0 
 0 
 0 
 0 
 0 
 0 
 0 
 0 
 0 
 0 
 0 
 0 
 0 
 0 
 0 
 0 
 0 
 0 
 0 
 0 
 0 
 0 
 0 
 0 
 0 
 0 
 0 
 0 
 0 
 0 
 0 
 0 
 0 
 0 
 0 
 0 
 0 
 0 
 0 
 0 
 0 
 0 
 0 
 0 
 0 
 0 
 0 
 0 
 0 
 0 
 0 
 0 
 0 
 0 
 0 
 0 
 0 
 0 
 0 
 0 
 0 
 0 
 0 
 0 
 0 
 0 
 0 
 0 
 0 
 0 
 0 
 0 
 0 
 0 
 0 
 0 
 0 
 0 
 0 
 0 
 0 
 0 
 0 
 0 
 0 
 0 
 0 
 0 
 0 
 0 
 0 
 0 
 0 
 0 
 0 
 0 
 0 
 0 
 0 
 0 
 0 
 0 
 0 
 0 
 0 
 0 
 0 
 0 
 0 
 0 
 0 
 0 
 0 
 0 
 0 
 0 
 0 
 0 
 0 
 0 
 0 
 0 
 0 
 0 
 0 
 0 
 0 
 0 
 0 
 0 
 0 
 0 
 0 
 0 
 0 
 0 
 0 
 0 
 0 
 0 
 0 
 0 
 0 
 0 
 0 
 0 
 0 
 0 
 0 
 0 
 0 
 0 
 0 
 0 
 0 
 0 
 0 
 0 
 0 
 0 
 0 
 0 
 0 
 0 
 0 
 0 
 0 
 0 
 0 
 0 
 0 
 0 
 0 
 0 
 0 
 0 
 0 
 0 
 0 
 0 
 0 
 0 
 0 
 0 
 0 
 0 
 0 
 0 
 0 
 0 
 0 
 0 
 0 
 0 
 0 
 0 
 0 
 0 
 0 
 0 
 0 
 0 
 0 
 0 
 0 
 0 
 0 
 0 
 0 
 0 
 0 
 0 
 0 
 0 
 0 
 0 
 0 
 0 
 0 
 0 
 0 
 0 
 0 
 0 
 0 
 0 
 0 
 0 
 0 
 0 
 0 
 0 
 0 
 0 
 0 
 0 
 0 
 0 
 0 
 0 
 0 
 0 
 0 
 0 
 0 
 0 
 0 
 0 
 0 
 0 
 0 
 0 
 0 
 0 
 0 
 0 
 0 
 0 
 0 
 0 
 0 
 0 
 0 
 0 
 0 
 0 
 0 
 0 
 0 
 0 
 0 
 0 
 0 
 0 
 0 
 0 
 0 
 0 
 0 
 0 
 0 
 0 
 0 
 0 
 0 
 0 
 0 
 0 
 0 
 0 
 0 
 0 
 0 
 0 
 0 
 0 
 0 
 0 
 0 
 0 
 0 
 0 
 0 
 0 
 0 
 0 
 0 
 0 
 0 
 0 
 0 
 0 
 0 
 0 
 0 
 0 
 0 
 0 
 0 
 0 
 0 
 0 
 0 
 0 
 0 
 0 
 0 
 0 
 0 
 0 
 0 
 0 
 0 
 0 
 0 
 0 
 0 
 0 
 0 
 0 
 0 
 0 
 0 
 0 
 0 
 0 
 0 
 0 
 0 
 0 
 0 
 0 
 0 
 0 
 0 
 0 
 0 
 0 
 0 
 0 
 0 
 0 
 0 
 0 
 0 
 0 
 0 
 0 
 0 
 0 
 0 
 0 
 0 
 0 
 0 
 0 
 0 
 0 
 0 
 0 
 0 
 0 
 0 
 0 
 0 
 0 
 0 
 0 
 0 
 0 
 0 
 0 
 0 
 0 
 0 
 0 
 0 
 0 
 0 
 0 
 0 
 0 
 0 
 0 
 0 
 0 
 0 
 0 
 0 
 0 
 0 
 0 
 0 
 
 
 61 
 0 
 0 
 0 
 0 
 0 
 0 
 0 
 0 
 0 
 0 
 0 
 0 
 0 
 0 
 0 
 0 
 0 
 0 
 0 
 0 
 0 
 0 
 0 
 0 
 0 
 0 
 0 
 0 
 0 
 0 
 0 
 0 
 0 
 0 
 0 
 0 
 0 
 0 
 0 
 0 
 0 
 0 
 0 
 0 
 0 
 0 
 0 
 0 
 0 
 0 
 0 
 0 
 0 
 0 
 0 
 0 
 4 
 0 
 0 
 0 
 0 
 3 
 0 
 0 
 0 
 0 
 0 
 0 
 0 
 0 
 0 
 0 
 0 
 0 
 0 
 0 
 0 
 0 
 0 
 0 
 0 
 0 
 0 
 0 
 0 
 0 
 0 
 0 
 0 
 0 
 0 
 0 
 0 
 0 
 0 
 0 
 0 
 0 
 0 
 0 
 0 
 0 
 0 
 0 
 0 
 0 
 0 
 0 
 0 
 0 
 0 
 0 
 0 
 0 
 0 
 0 
 0 
 0 
 0 
 0 
 0 
 0 
 0 
 0 
 0 
 0 
 0 
 0 
 0 
 0 
 0 
 0 
 0 
 0 
 0 
 0 
 0 
 0 
 0 
 0 
 0 
 0 
 0 
 0 
 0 
 0 
 0 
 0 
 0 
 0 
 0 
 0 
 0 
 0 
 0 
 0 
 0 
 0 
 0 
 0 
 0 
 0 
 0 
 0 
 0 
 0 
 0 
 0 
 0 
 0 
 0 
 0 
 0 
 0 
 0 
 0 
 0 
 0 
 0 
 0 
 0 
 0 
 0 
 0 
 0 
 0 
 0 
 0 
 0 
 0 
 0 
 0 
 0 
 0 
 0 
 0 
 0 
 0 
 0 
 0 
 0 
 0 
 0 
 0 
 0 
 0 
 0 
 0 
 0 
 0 
 0 
 0 
 0 
 0 
 0 
 0 
 0 
 0 
 0 
 0 
 0 
 0 
 0 
 0 
 0 
 0 
 0 
 0 
 0 
 0 
 0 
 0 
 0 
 0 
 0 
 0 
 0 
 0 
 0 
 0 
 0 
 0 
 0 
 0 
 0 
 0 
 0 
 0 
 0 
 0 
 0 
 0 
 0 
 0 
 0 
 0 
 0 
 0 
 0 
 0 
 0 
 0 
 0 
 0 
 0 
 0 
 0 
 0 
 0 
 0 
 0 
 0 
 0 
 0 
 0 
 0 
 0 
 0 
 0 
 0 
 0 
 0 
 0 
 0 
 0 
 0 
 0 
 0 
 0 
 0 
 0 
 0 
 0 
 0 
 0 
 0 
 0 
 0 
 0 
 0 
 0 
 0 
 0 
 0 
 0 
 0 
 0 
 0 
 0 
 0 
 0 
 0 
 0 
 0 
 0 
 0 
 0 
 0 
 0 
 0 
 0 
 0 
 0 
 0 
 0 
 0 
 0 
 0 
 0 
 0 
 0 
 0 
 0 
 0 
 0 
 0 
 0 
 0 
 0 
 0 
 0 
 0 
 0 
 0 
 0 
 0 
 0 
 0 
 0 
 0 
 0 
 0 
 0 
 0 
 0 
 0 
 0 
 0 
 0 
 0 
 0 
 0 
 0 
 0 
 0 
 0 
 0 
 0 
 0 
 0 
 0 
 0 
 0 
 0 
 0 
 0 
 0 
 0 
 0 
 0 
 0 
 0 
 0 
 0 
 0 
 0 
 0 
 0 
 0 
 0 
 0 
 0 
 0 
 0 
 0 
 0 
 0 
 0 
 0 
 0 
 0 
 0 
 0 
 0 
 0 
 0 
 0 
 0 
 0 
 0 
 0 
 0 
 0 
 0 
 0 
 0 
 0 
 0 
 0 
 0 
 0 
 0 
 0 
 0 
 0 
 0 
 0 
 0 
 0 
 0 
 0 
 0 
 0 
 0 
 0 
 0 
 0 
 0 
 0 
 0 
 0 
 0 
 0 
 0 
 0 
 0 
 0 
 0 
 0 
 0 
 0 
 0 
 0 
 0 
 0 
 0 
 0 
 0 
 0 
 0 
 0 
 0 
 0 
 0 
 0 
 0 
 0 
 0 
 0 
 0 
 0 
 0 
 0 
 0 
 0 
 0 
 0 
 0 
 0 
 0 
 0 
 0 
 0 
 0 
 0 
 0 
 0 
 0 
 0 
 0 
 0 
 0 
 0 
 0 
 0 
 0 
 0 
 0 
 0 
 0 
 0 
 0 
 0 
 0 
 0 
 0 
 0 
 0 
 0 
 0 
 0 
 0 
 0 
 0 
 0 
 0 
 0 
 0 
 0 
 0 
 0 
 0 
 0 
 0 
 0 
 0 
 0 
 0 
 0 
 0 
 0 
 0 
 0 
 0 
 0 
 0 
 0 
 0 
 0 
 0 
 0 
 0 
 0 
 0 
 0 
 0 
 0 
 0 
 0 
 0 
 0 
 0 
 0 
 0 
 0 
 0 
 0 
 0 
 0 
 0 
 0 
 0 
 0 
 0 
 0 
 0 
 0 
 0 
 0 
 0 
 0 
 0 
 0 
 0 
 0 
 0 
 0 
 0 
 0 
 0 
 0 
 0 
 0 
 0 
 0 
 0 
 0 
 0 
 0 
 0 
 0 
 0 
 0 
 0 
 0 
 0 
 0 
 0 
 0 
 0 
 0 
 0 
 0 
 0 
 0 
 0 
 0 
 0 
 0 
 0 
 0 
 0 
 0 
 0 
 0 
 0 
 0 
 0 
 0 
 0 
 0 
 0 
 0 
 0 
 0 
 0 
 0 
 0 
 0 
 0 
 0 
 0 
 0 
 0 
 0 
 0 
 0 
 0 
 0 
 0 
 0 
 0 
 0 
 0 
 0 
 0 
 0 
 0 
 0 
 0 
 0 
 0 
 0 
 0 
 0 
 0 
 0 
 0 
 0 
 0 
 0 
 0 
 0 
 0 
 0 
 0 
 0 
 0 
 0 
 0 
 0 
 0 
 0 
 0 
 0 
 0 
 0 
 0 
 0 
 0 
 0 
 0 
 0 
 0 
 0 
 0 
 0 
 0 
 0 
 0 
 0 
 0 
 0 
 0 
 0 
 0 
 0 
 0 
 0 
 0 
 0 
 0 
 0 
 0 
 0 
 0 
 0 
 0 
 0 
 0 
 0 
 0 
 0 
 0 
 0 
 0 
 0 
 0 
 0 
 0 
 0 
 0 
 0 
 0 
 0 
 0 
 0 
 0 
 0 
 0 
 0 
 0 
 0 
 0 
 0 
 0 
 0 
 0 
 0 
 0 
 0 
 0 
 0 
 0 
 0 
 0 
 0 
 0 
 0 
 0 
 0 
 0 
 0 
 0 
 0 
 0 
 0 
 0 
 0 
 0 
 0 
 0 
 0 
 0 
 0 
 0 
 0 
 0 
 0 
 0 
 0 
 0 
 0 
 0 
 0 
 0 
 0 
 0 
 0 
 0 
 0 
 0 
 0 
 0 
 0 
 0 
 0 
 0 
 0 
 0 
 0 
 0 
 0 
 0 
 0 
 0 
 0 
 0 
 0 
 0 
 0 
 0 
 0 
 0 
 0 
 0 
 0 
 0 
 0 
 0 
 0 
 0 
 0 
 0 
 0 
 0 
 0 
 0 
 0 
 0 
 0 
 0 
 0 
 0 
 0 
 0 
 
 
 62 
 0 
 0 
 0 
 0 
 0 
 0 
 0 
 0 
 0 
 0 
 0 
 0 
 0 
 0 
 0 
 0 
 0 
 0 
 0 
 0 
 0 
 0 
 0 
 0 
 0 
 0 
 0 
 0 
 0 
 0 
 0 
 0 
 0 
 0 
 0 
 0 
 0 
 0 
 0 
 0 
 0 
 0 
 0 
 0 
 0 
 0 
 0 
 0 
 0 
 0 
 0 
 0 
 0 
 0 
 0 
 0 
 0 
 0 
 0 
 0 
 0 
 0 
 8 
 0 
 0 
 0 
 0 
 0 
 0 
 0 
 0 
 0 
 0 
 0 
 0 
 0 
 0 
 0 
 0 
 0 
 0 
 0 
 0 
 0 
 0 
 0 
 0 
 0 
 0 
 0 
 0 
 0 
 0 
 0 
 0 
 0 
 0 
 0 
 0 
 0 
 0 
 0 
 0 
 0 
 0 
 0 
 0 
 0 
 0 
 0 
 0 
 0 
 0 
 0 
 0 
 0 
 0 
 0 
 0 
 0 
 0 
 0 
 0 
 0 
 0 
 0 
 0 
 0 
 0 
 0 
 0 
 0 
 0 
 0 
 0 
 0 
 0 
 0 
 0 
 0 
 0 
 0 
 0 
 0 
 0 
 0 
 0 
 0 
 0 
 0 
 0 
 0 
 0 
 0 
 0 
 0 
 0 
 0 
 0 
 0 
 0 
 0 
 0 
 0 
 0 
 0 
 0 
 0 
 0 
 0 
 0 
 0 
 0 
 0 
 0 
 0 
 0 
 0 
 0 
 0 
 0 
 0 
 0 
 0 
 0 
 0 
 0 
 0 
 0 
 0 
 0 
 0 
 0 
 0 
 0 
 0 
 0 
 0 
 0 
 0 
 0 
 0 
 0 
 0 
 0 
 0 
 0 
 0 
 0 
 0 
 0 
 0 
 0 
 0 
 0 
 0 
 0 
 0 
 0 
 0 
 0 
 0 
 0 
 0 
 0 
 0 
 0 
 0 
 0 
 0 
 0 
 0 
 0 
 0 
 0 
 0 
 0 
 0 
 0 
 0 
 0 
 0 
 0 
 0 
 0 
 0 
 0 
 0 
 0 
 0 
 0 
 0 
 0 
 0 
 0 
 0 
 0 
 0 
 0 
 0 
 0 
 0 
 0 
 0 
 0 
 0 
 0 
 0 
 0 
 0 
 0 
 0 
 0 
 0 
 0 
 0 
 0 
 0 
 0 
 0 
 0 
 0 
 0 
 0 
 0 
 0 
 0 
 0 
 0 
 0 
 0 
 0 
 0 
 0 
 0 
 0 
 0 
 0 
 0 
 0 
 0 
 0 
 0 
 0 
 0 
 0 
 0 
 0 
 0 
 0 
 0 
 0 
 0 
 0 
 0 
 0 
 0 
 0 
 0 
 0 
 0 
 0 
 0 
 0 
 0 
 0 
 0 
 0 
 0 
 0 
 0 
 0 
 0 
 0 
 0 
 0 
 0 
 0 
 0 
 0 
 0 
 0 
 0 
 0 
 0 
 0 
 0 
 0 
 0 
 0 
 0 
 0 
 0 
 0 
 0 
 0 
 0 
 0 
 0 
 0 
 0 
 0 
 0 
 0 
 0 
 0 
 0 
 0 
 0 
 0 
 0 
 0 
 0 
 0 
 0 
 0 
 0 
 0 
 0 
 0 
 0 
 0 
 0 
 0 
 0 
 0 
 0 
 0 
 0 
 0 
 0 
 0 
 0 
 0 
 0 
 0 
 0 
 0 
 0 
 0 
 0 
 0 
 0 
 0 
 0 
 0 
 0 
 0 
 0 
 0 
 0 
 0 
 0 
 0 
 0 
 0 
 0 
 0 
 0 
 0 
 0 
 0 
 0 
 0 
 0 
 0 
 0 
 0 
 0 
 0 
 0 
 0 
 0 
 0 
 0 
 0 
 0 
 0 
 0 
 0 
 0 
 0 
 0 
 0 
 0 
 0 
 0 
 0 
 0 
 0 
 0 
 0 
 0 
 0 
 0 
 0 
 0 
 0 
 0 
 0 
 0 
 0 
 0 
 0 
 0 
 0 
 0 
 0 
 0 
 0 
 0 
 0 
 0 
 0 
 0 
 0 
 0 
 0 
 0 
 0 
 0 
 0 
 0 
 0 
 0 
 0 
 0 
 0 
 0 
 0 
 0 
 0 
 0 
 0 
 0 
 0 
 0 
 0 
 0 
 0 
 0 
 0 
 0 
 0 
 0 
 0 
 0 
 0 
 0 
 0 
 0 
 0 
 0 
 0 
 0 
 0 
 0 
 0 
 0 
 0 
 0 
 0 
 0 
 0 
 0 
 0 
 0 
 0 
 0 
 0 
 0 
 0 
 0 
 0 
 0 
 0 
 0 
 0 
 0 
 0 
 0 
 0 
 0 
 0 
 0 
 0 
 0 
 0 
 0 
 0 
 0 
 0 
 0 
 0 
 0 
 0 
 0 
 0 
 0 
 0 
 0 
 0 
 0 
 0 
 0 
 0 
 0 
 0 
 0 
 0 
 0 
 0 
 0 
 0 
 0 
 0 
 0 
 0 
 0 
 0 
 0 
 0 
 0 
 0 
 0 
 0 
 0 
 0 
 0 
 0 
 0 
 0 
 0 
 0 
 0 
 0 
 0 
 0 
 0 
 0 
 0 
 0 
 0 
 0 
 0 
 0 
 0 
 0 
 0 
 0 
 0 
 0 
 0 
 0 
 0 
 0 
 0 
 0 
 0 
 0 
 0 
 0 
 0 
 0 
 0 
 0 
 0 
 0 
 0 
 0 
 0 
 0 
 0 
 0 
 0 
 0 
 0 
 0 
 0 
 0 
 0 
 0 
 0 
 0 
 0 
 0 
 0 
 0 
 0 
 0 
 0 
 0 
 0 
 0 
 0 
 0 
 0 
 0 
 0 
 0 
 0 
 0 
 0 
 0 
 0 
 0 
 0 
 0 
 0 
 0 
 0 
 0 
 0 
 0 
 0 
 0 
 0 
 0 
 0 
 0 
 0 
 0 
 0 
 0 
 0 
 0 
 0 
 0 
 0 
 0 
 0 
 0 
 0 
 0 
 0 
 0 
 0 
 0 
 0 
 0 
 0 
 0 
 0 
 0 
 0 
 0 
 0 
 0 
 0 
 0 
 0 
 0 
 0 
 0 
 0 
 0 
 0 
 0 
 0 
 0 
 0 
 0 
 0 
 0 
 0 
 0 
 0 
 0 
 0 
 0 
 0 
 0 
 0 
 0 
 0 
 0 
 0 
 0 
 0 
 0 
 0 
 0 
 0 
 0 
 0 
 0 
 0 
 0 
 0 
 0 
 0 
 0 
 0 
 0 
 0 
 0 
 0 
 0 
 0 
 0 
 0 
 0 
 0 
 0 
 0 
 0 
 0 
 0 
 0 
 0 
 0 
 0 
 0 
 0 
 0 
 0 
 0 
 0 
 0 
 0 
 0 
 0 
 0 
 0 
 0 
 0 
 0 
 0 
 0 
 0 
 0 
 0 
 0 
 0 
 0 
 0 
 0 
 0 
 0 
 0 
 0 
 0 
 0 
 0 
 0 
 0 
 0 
 0 
 0 
 0 
 0 
 0 
 0 
 0 
 0 
 0 
 0 
 0 
 0 
 0 
 0 
 0 
 0 
 0 
 0 
 0 
 
 
 63 
 0 
 0 
 0 
 0 
 0 
 0 
 0 
 0 
 0 
 0 
 0 
 0 
 0 
 0 
 0 
 0 
 0 
 0 
 0 
 0 
 0 
 0 
 0 
 0 
 0 
 0 
 0 
 0 
 0 
 0 
 0 
 0 
 0 
 0 
 0 
 0 
 0 
 0 
 0 
 0 
 0 
 0 
 0 
 0 
 0 
 0 
 0 
 0 
 0 
 0 
 0 
 0 
 0 
 0 
 0 
 0 
 0 
 0 
 0 
 0 
 0 
 0 
 0 
 9 
 0 
 0 
 0 
 0 
 0 
 0 
 0 
 0 
 0 
 0 
 0 
 0 
 0 
 0 
 0 
 0 
 0 
 0 
 0 
 0 
 0 
 0 
 0 
 0 
 0 
 0 
 0 
 0 
 0 
 0 
 0 
 0 
 0 
 0 
 0 
 0 
 0 
 0 
 0 
 0 
 0 
 0 
 0 
 0 
 0 
 0 
 0 
 0 
 0 
 0 
 0 
 0 
 0 
 0 
 0 
 0 
 0 
 0 
 0 
 0 
 0 
 0 
 0 
 0 
 0 
 0 
 0 
 0 
 0 
 0 
 0 
 0 
 0 
 0 
 0 
 0 
 0 
 0 
 0 
 0 
 0 
 0 
 0 
 0 
 0 
 0 
 0 
 0 
 0 
 0 
 0 
 0 
 0 
 0 
 0 
 0 
 0 
 0 
 0 
 0 
 0 
 0 
 0 
 0 
 0 
 0 
 0 
 0 
 0 
 0 
 0 
 0 
 0 
 0 
 0 
 0 
 0 
 0 
 0 
 0 
 0 
 0 
 0 
 0 
 0 
 0 
 0 
 0 
 0 
 0 
 0 
 0 
 0 
 0 
 0 
 0 
 0 
 0 
 0 
 0 
 0 
 0 
 0 
 0 
 0 
 0 
 0 
 0 
 0 
 0 
 0 
 0 
 0 
 0 
 0 
 0 
 0 
 0 
 0 
 0 
 0 
 0 
 0 
 0 
 0 
 0 
 0 
 0 
 0 
 0 
 0 
 0 
 0 
 0 
 0 
 0 
 0 
 0 
 0 
 0 
 0 
 0 
 0 
 0 
 0 
 0 
 0 
 0 
 0 
 0 
 0 
 0 
 0 
 0 
 0 
 0 
 0 
 0 
 0 
 0 
 0 
 0 
 0 
 0 
 0 
 0 
 0 
 0 
 0 
 0 
 0 
 0 
 0 
 0 
 0 
 0 
 0 
 0 
 0 
 0 
 0 
 0 
 0 
 0 
 0 
 0 
 0 
 0 
 0 
 0 
 0 
 0 
 0 
 0 
 0 
 0 
 0 
 0 
 0 
 0 
 0 
 0 
 0 
 0 
 0 
 0 
 0 
 0 
 0 
 0 
 0 
 0 
 0 
 0 
 0 
 0 
 0 
 0 
 0 
 0 
 0 
 0 
 0 
 0 
 0 
 0 
 0 
 0 
 0 
 0 
 0 
 0 
 0 
 0 
 0 
 0 
 0 
 0 
 0 
 0 
 0 
 0 
 0 
 0 
 0 
 0 
 0 
 0 
 0 
 0 
 0 
 0 
 0 
 0 
 0 
 0 
 0 
 0 
 0 
 0 
 0 
 0 
 0 
 0 
 0 
 0 
 0 
 0 
 0 
 0 
 0 
 0 
 0 
 0 
 0 
 0 
 0 
 0 
 0 
 0 
 0 
 0 
 0 
 0 
 0 
 0 
 0 
 0 
 0 
 0 
 0 
 0 
 0 
 0 
 0 
 0 
 0 
 0 
 0 
 0 
 0 
 0 
 0 
 0 
 0 
 0 
 0 
 0 
 0 
 0 
 0 
 0 
 0 
 0 
 0 
 0 
 0 
 0 
 0 
 0 
 0 
 0 
 0 
 0 
 0 
 0 
 0 
 0 
 0 
 0 
 0 
 0 
 0 
 0 
 0 
 0 
 0 
 0 
 0 
 0 
 0 
 0 
 0 
 0 
 0 
 0 
 0 
 0 
 0 
 0 
 0 
 0 
 0 
 0 
 0 
 0 
 0 
 0 
 0 
 0 
 0 
 0 
 0 
 0 
 0 
 0 
 0 
 0 
 0 
 0 
 0 
 0 
 0 
 0 
 0 
 0 
 0 
 0 
 0 
 0 
 0 
 0 
 0 
 0 
 0 
 0 
 0 
 0 
 0 
 0 
 0 
 0 
 0 
 0 
 0 
 0 
 0 
 0 
 0 
 0 
 0 
 0 
 0 
 0 
 0 
 0 
 0 
 0 
 0 
 0 
 0 
 0 
 0 
 0 
 0 
 0 
 0 
 0 
 0 
 0 
 0 
 0 
 0 
 0 
 0 
 0 
 0 
 0 
 0 
 0 
 0 
 0 
 0 
 0 
 0 
 0 
 0 
 0 
 0 
 0 
 0 
 0 
 0 
 0 
 0 
 0 
 0 
 0 
 0 
 0 
 0 
 0 
 0 
 0 
 0 
 0 
 0 
 0 
 0 
 0 
 0 
 0 
 0 
 0 
 0 
 0 
 0 
 0 
 0 
 0 
 0 
 0 
 0 
 0 
 0 
 0 
 0 
 0 
 0 
 0 
 0 
 0 
 0 
 0 
 0 
 0 
 0 
 0 
 0 
 0 
 0 
 0 
 0 
 0 
 0 
 0 
 0 
 0 
 0 
 0 
 0 
 0 
 0 
 0 
 0 
 0 
 0 
 0 
 0 
 0 
 0 
 0 
 0 
 0 
 0 
 0 
 0 
 0 
 0 
 0 
 0 
 0 
 0 
 0 
 0 
 0 
 0 
 0 
 0 
 0 
 0 
 0 
 0 
 0 
 0 
 0 
 0 
 0 
 0 
 0 
 0 
 0 
 0 
 0 
 0 
 0 
 0 
 0 
 0 
 0 
 0 
 0 
 0 
 0 
 0 
 0 
 0 
 0 
 0 
 0 
 0 
 0 
 0 
 0 
 0 
 0 
 0 
 0 
 0 
 0 
 0 
 0 
 0 
 0 
 0 
 0 
 0 
 0 
 0 
 0 
 0 
 0 
 0 
 0 
 0 
 0 
 0 
 0 
 0 
 0 
 0 
 0 
 0 
 0 
 0 
 0 
 0 
 0 
 0 
 0 
 0 
 0 
 0 
 0 
 0 
 0 
 0 
 0 
 0 
 0 
 0 
 0 
 0 
 0 
 0 
 0 
 0 
 0 
 0 
 0 
 0 
 0 
 0 
 0 
 0 
 0 
 0 
 0 
 0 
 0 
 0 
 0 
 0 
 0 
 0 
 0 
 0 
 0 
 0 
 0 
 0 
 0 
 0 
 0 
 0 
 0 
 0 
 0 
 0 
 0 
 0 
 0 
 0 
 0 
 0 
 0 
 0 
 0 
 0 
 0 
 0 
 0 
 0 
 0 
 0 
 0 
 0 
 0 
 0 
 0 
 0 
 0 
 0 
 0 
 0 
 0 
 0 
 0 
 0 
 0 
 0 
 0 
 0 
 0 
 0 
 0 
 0 
 0 
 0 
 0 
 0 
 0 
 0 
 0 
 0 
 0 
 0 
 0 
 0 
 0 
 0 
 0 
 0 
 0 
 0 
 0 
 0 
 0 
 0 
 0 
 0 
 0 
 0 
 0 
 0 
 0 
 0 
 0 
 0 
 0 
 0 
 0 
 
 
 64 
 0 
 0 
 0 
 0 
 0 
 0 
 0 
 0 
 0 
 0 
 0 
 0 
 0 
 0 
 0 
 0 
 0 
 0 
 0 
 0 
 0 
 0 
 0 
 0 
 0 
 0 
 0 
 0 
 0 
 0 
 0 
 0 
 0 
 0 
 0 
 0 
 0 
 0 
 0 
 0 
 0 
 0 
 0 
 0 
 0 
 0 
 0 
 0 
 0 
 0 
 0 
 0 
 0 
 0 
 0 
 0 
 0 
 0 
 0 
 0 
 0 
 0 
 0 
 0 
 26 
 0 
 0 
 0 
 0 
 0 
 0 
 0 
 0 
 0 
 0 
 0 
 0 
 0 
 0 
 0 
 0 
 0 
 0 
 0 
 0 
 0 
 0 
 0 
 0 
 0 
 0 
 0 
 0 
 0 
 0 
 0 
 0 
 0 
 0 
 0 
 0 
 0 
 0 
 0 
 0 
 0 
 0 
 0 
 0 
 0 
 0 
 0 
 0 
 0 
 0 
 0 
 0 
 0 
 0 
 0 
 0 
 0 
 0 
 0 
 0 
 0 
 0 
 0 
 0 
 0 
 0 
 0 
 0 
 0 
 0 
 0 
 0 
 0 
 0 
 0 
 0 
 0 
 0 
 0 
 0 
 0 
 0 
 0 
 0 
 0 
 0 
 0 
 0 
 0 
 0 
 0 
 0 
 0 
 0 
 0 
 0 
 0 
 0 
 0 
 0 
 0 
 0 
 0 
 0 
 0 
 0 
 0 
 0 
 0 
 0 
 0 
 0 
 0 
 0 
 0 
 0 
 0 
 0 
 0 
 0 
 0 
 0 
 0 
 0 
 0 
 0 
 0 
 0 
 0 
 0 
 0 
 0 
 0 
 0 
 0 
 0 
 0 
 0 
 0 
 0 
 0 
 0 
 0 
 0 
 0 
 0 
 0 
 0 
 0 
 0 
 0 
 0 
 0 
 0 
 0 
 0 
 0 
 0 
 0 
 0 
 0 
 0 
 0 
 0 
 0 
 0 
 0 
 0 
 0 
 0 
 0 
 0 
 0 
 0 
 0 
 0 
 0 
 0 
 0 
 0 
 0 
 0 
 0 
 0 
 0 
 0 
 0 
 0 
 0 
 0 
 0 
 0 
 0 
 0 
 0 
 0 
 0 
 0 
 0 
 0 
 0 
 0 
 0 
 0 
 0 
 0 
 0 
 0 
 0 
 0 
 0 
 0 
 0 
 0 
 0 
 0 
 0 
 0 
 0 
 0 
 0 
 0 
 0 
 0 
 0 
 0 
 0 
 0 
 0 
 0 
 0 
 0 
 0 
 0 
 0 
 0 
 0 
 0 
 0 
 0 
 0 
 0 
 0 
 0 
 0 
 0 
 0 
 0 
 0 
 0 
 0 
 0 
 0 
 0 
 0 
 0 
 0 
 0 
 0 
 0 
 0 
 0 
 0 
 0 
 0 
 0 
 0 
 0 
 0 
 0 
 0 
 0 
 0 
 0 
 0 
 0 
 0 
 0 
 0 
 0 
 0 
 0 
 0 
 0 
 0 
 0 
 0 
 0 
 0 
 0 
 0 
 0 
 0 
 0 
 0 
 0 
 0 
 0 
 0 
 0 
 0 
 0 
 0 
 0 
 0 
 0 
 0 
 0 
 0 
 0 
 0 
 0 
 0 
 0 
 0 
 0 
 0 
 0 
 0 
 0 
 0 
 0 
 0 
 0 
 0 
 0 
 0 
 0 
 0 
 0 
 0 
 0 
 0 
 0 
 0 
 0 
 0 
 0 
 0 
 0 
 0 
 0 
 0 
 0 
 0 
 0 
 0 
 0 
 0 
 0 
 0 
 0 
 0 
 0 
 0 
 0 
 0 
 0 
 0 
 0 
 0 
 0 
 0 
 0 
 0 
 0 
 0 
 0 
 0 
 0 
 0 
 0 
 0 
 0 
 0 
 0 
 0 
 0 
 0 
 0 
 0 
 0 
 0 
 0 
 0 
 0 
 0 
 0 
 0 
 0 
 0 
 0 
 0 
 0 
 0 
 0 
 0 
 0 
 0 
 0 
 0 
 0 
 0 
 0 
 0 
 0 
 0 
 0 
 0 
 0 
 0 
 0 
 0 
 0 
 0 
 0 
 0 
 0 
 0 
 0 
 0 
 0 
 0 
 0 
 0 
 0 
 0 
 0 
 0 
 0 
 0 
 0 
 0 
 0 
 0 
 0 
 0 
 0 
 0 
 0 
 0 
 0 
 0 
 0 
 0 
 0 
 0 
 0 
 0 
 0 
 0 
 0 
 0 
 0 
 0 
 0 
 0 
 0 
 0 
 0 
 0 
 0 
 0 
 0 
 0 
 0 
 0 
 0 
 0 
 0 
 0 
 0 
 0 
 0 
 0 
 0 
 0 
 0 
 0 
 0 
 0 
 0 
 0 
 0 
 0 
 0 
 0 
 0 
 0 
 0 
 0 
 0 
 0 
 0 
 0 
 0 
 0 
 0 
 0 
 0 
 0 
 0 
 0 
 0 
 0 
 0 
 0 
 0 
 0 
 0 
 0 
 0 
 0 
 0 
 0 
 0 
 0 
 0 
 0 
 0 
 0 
 0 
 0 
 0 
 0 
 0 
 0 
 0 
 0 
 0 
 0 
 0 
 0 
 0 
 0 
 0 
 0 
 0 
 0 
 0 
 0 
 0 
 0 
 0 
 0 
 0 
 0 
 0 
 0 
 0 
 0 
 0 
 0 
 0 
 0 
 0 
 0 
 0 
 0 
 0 
 0 
 0 
 0 
 0 
 0 
 0 
 0 
 0 
 0 
 0 
 0 
 0 
 0 
 0 
 0 
 0 
 0 
 0 
 0 
 0 
 0 
 7 
 0 
 0 
 0 
 0 
 0 
 0 
 0 
 0 
 0 
 0 
 0 
 0 
 0 
 0 
 0 
 0 
 0 
 0 
 0 
 0 
 0 
 0 
 0 
 0 
 0 
 0 
 0 
 0 
 0 
 0 
 0 
 0 
 0 
 0 
 0 
 0 
 0 
 0 
 0 
 0 
 0 
 0 
 0 
 0 
 0 
 0 
 0 
 0 
 0 
 0 
 0 
 0 
 0 
 0 
 0 
 0 
 0 
 0 
 0 
 0 
 0 
 0 
 0 
 0 
 0 
 0 
 0 
 0 
 2 
 0 
 0 
 0 
 0 
 0 
 0 
 0 
 0 
 0 
 0 
 0 
 0 
 0 
 0 
 0 
 0 
 0 
 3 
 0 
 0 
 0 
 0 
 0 
 0 
 0 
 0 
 0 
 0 
 0 
 0 
 0 
 0 
 0 
 0 
 0 
 0 
 0 
 0 
 0 
 0 
 0 
 0 
 0 
 0 
 0 
 0 
 0 
 0 
 0 
 0 
 0 
 0 
 0 
 0 
 0 
 0 
 0 
 0 
 0 
 0 
 0 
 0 
 0 
 0 
 0 
 0 
 0 
 0 
 0 
 0 
 0 
 0 
 0 
 0 
 0 
 0 
 0 
 0 
 0 
 0 
 0 
 0 
 0 
 0 
 0 
 0 
 0 
 0 
 0 
 0 
 0 
 0 
 0 
 0 
 0 
 0 
 0 
 0 
 0 
 0 
 0 
 0 
 0 
 0 
 0 
 0 
 0 
 0 
 0 
 0 
 
 
 65 
 0 
 0 
 0 
 0 
 0 
 0 
 0 
 0 
 0 
 0 
 0 
 0 
 0 
 0 
 0 
 0 
 0 
 0 
 0 
 0 
 0 
 0 
 0 
 0 
 0 
 0 
 0 
 0 
 0 
 0 
 0 
 4 
 0 
 0 
 0 
 0 
 0 
 0 
 0 
 0 
 0 
 0 
 0 
 0 
 0 
 0 
 0 
 0 
 0 
 0 
 0 
 0 
 0 
 0 
 0 
 0 
 0 
 0 
 0 
 0 
 0 
 0 
 0 
 0 
 0 
 14 
 0 
 0 
 0 
 0 
 0 
 0 
 0 
 0 
 0 
 0 
 0 
 0 
 0 
 0 
 0 
 0 
 0 
 0 
 0 
 0 
 0 
 0 
 0 
 0 
 0 
 0 
 0 
 0 
 0 
 0 
 0 
 0 
 0 
 0 
 0 
 0 
 0 
 0 
 0 
 0 
 0 
 0 
 0 
 0 
 0 
 0 
 0 
 0 
 0 
 0 
 0 
 0 
 0 
 0 
 0 
 0 
 0 
 0 
 0 
 0 
 0 
 0 
 0 
 0 
 0 
 0 
 0 
 0 
 0 
 0 
 0 
 0 
 0 
 0 
 0 
 0 
 0 
 0 
 0 
 0 
 0 
 0 
 0 
 0 
 0 
 0 
 0 
 0 
 0 
 0 
 0 
 0 
 0 
 0 
 0 
 0 
 0 
 0 
 0 
 0 
 0 
 0 
 0 
 0 
 0 
 0 
 0 
 0 
 0 
 0 
 0 
 0 
 0 
 0 
 0 
 0 
 0 
 0 
 0 
 0 
 0 
 0 
 0 
 0 
 0 
 0 
 0 
 0 
 0 
 0 
 0 
 0 
 0 
 0 
 0 
 0 
 0 
 0 
 0 
 0 
 0 
 0 
 0 
 0 
 0 
 0 
 0 
 0 
 0 
 0 
 0 
 0 
 0 
 0 
 0 
 0 
 0 
 0 
 0 
 0 
 0 
 0 
 0 
 0 
 0 
 0 
 0 
 0 
 0 
 0 
 0 
 0 
 0 
 0 
 0 
 0 
 0 
 0 
 0 
 0 
 0 
 0 
 0 
 0 
 0 
 0 
 0 
 0 
 0 
 0 
 0 
 0 
 0 
 0 
 0 
 0 
 0 
 0 
 0 
 0 
 0 
 0 
 0 
 0 
 0 
 0 
 0 
 0 
 0 
 0 
 0 
 0 
 0 
 0 
 0 
 0 
 0 
 0 
 0 
 0 
 0 
 0 
 0 
 0 
 0 
 0 
 0 
 0 
 0 
 0 
 0 
 0 
 0 
 0 
 0 
 0 
 0 
 0 
 0 
 0 
 0 
 0 
 0 
 0 
 0 
 0 
 0 
 0 
 0 
 0 
 0 
 0 
 0 
 0 
 0 
 0 
 0 
 0 
 0 
 0 
 0 
 0 
 0 
 0 
 0 
 0 
 0 
 0 
 0 
 0 
 0 
 0 
 0 
 0 
 0 
 0 
 0 
 0 
 0 
 0 
 0 
 0 
 0 
 0 
 0 
 0 
 0 
 0 
 0 
 0 
 0 
 0 
 0 
 0 
 0 
 0 
 0 
 0 
 0 
 0 
 0 
 0 
 0 
 0 
 0 
 0 
 0 
 0 
 0 
 0 
 0 
 0 
 0 
 0 
 0 
 0 
 0 
 0 
 0 
 0 
 0 
 0 
 0 
 0 
 0 
 0 
 0 
 0 
 0 
 0 
 0 
 0 
 0 
 0 
 0 
 0 
 0 
 0 
 0 
 0 
 0 
 0 
 0 
 0 
 0 
 0 
 0 
 0 
 0 
 0 
 0 
 0 
 0 
 0 
 0 
 0 
 0 
 0 
 0 
 0 
 0 
 0 
 0 
 0 
 0 
 0 
 0 
 0 
 0 
 0 
 0 
 0 
 0 
 0 
 0 
 0 
 0 
 0 
 0 
 0 
 0 
 0 
 0 
 0 
 0 
 0 
 0 
 0 
 0 
 0 
 0 
 0 
 0 
 0 
 0 
 0 
 0 
 0 
 0 
 0 
 0 
 0 
 0 
 0 
 0 
 0 
 0 
 0 
 0 
 0 
 0 
 0 
 0 
 0 
 0 
 0 
 0 
 0 
 0 
 0 
 0 
 0 
 0 
 0 
 0 
 0 
 0 
 0 
 0 
 0 
 0 
 0 
 0 
 0 
 0 
 0 
 0 
 0 
 0 
 0 
 0 
 0 
 0 
 0 
 0 
 0 
 0 
 0 
 0 
 0 
 0 
 0 
 0 
 0 
 0 
 0 
 0 
 0 
 0 
 0 
 0 
 0 
 0 
 0 
 0 
 0 
 0 
 0 
 0 
 0 
 0 
 0 
 0 
 0 
 0 
 0 
 0 
 0 
 0 
 0 
 0 
 0 
 0 
 0 
 0 
 0 
 0 
 0 
 0 
 0 
 0 
 0 
 0 
 0 
 0 
 0 
 0 
 0 
 0 
 0 
 0 
 0 
 0 
 0 
 0 
 0 
 0 
 0 
 0 
 0 
 0 
 0 
 0 
 0 
 0 
 0 
 0 
 0 
 0 
 0 
 0 
 0 
 0 
 0 
 0 
 0 
 0 
 0 
 0 
 0 
 0 
 0 
 0 
 0 
 0 
 0 
 0 
 0 
 0 
 0 
 0 
 0 
 0 
 0 
 0 
 0 
 0 
 0 
 0 
 0 
 0 
 0 
 0 
 0 
 0 
 0 
 0 
 0 
 0 
 0 
 0 
 0 
 0 
 0 
 0 
 0 
 0 
 0 
 0 
 0 
 0 
 0 
 0 
 0 
 0 
 0 
 0 
 0 
 0 
 0 
 0 
 0 
 0 
 0 
 0 
 0 
 0 
 0 
 0 
 0 
 0 
 0 
 0 
 0 
 0 
 0 
 0 
 0 
 0 
 0 
 0 
 0 
 0 
 0 
 0 
 0 
 0 
 0 
 0 
 0 
 0 
 0 
 0 
 0 
 0 
 0 
 0 
 0 
 0 
 0 
 0 
 0 
 0 
 0 
 0 
 0 
 0 
 0 
 0 
 0 
 0 
 0 
 0 
 0 
 0 
 0 
 0 
 0 
 0 
 4 
 0 
 0 
 0 
 0 
 0 
 0 
 0 
 0 
 0 
 0 
 0 
 0 
 0 
 0 
 0 
 0 
 0 
 0 
 0 
 0 
 0 
 0 
 0 
 0 
 0 
 0 
 0 
 0 
 0 
 0 
 0 
 0 
 0 
 0 
 0 
 0 
 0 
 0 
 0 
 0 
 0 
 0 
 0 
 0 
 0 
 0 
 0 
 0 
 0 
 0 
 0 
 0 
 0 
 0 
 0 
 0 
 0 
 0 
 0 
 0 
 0 
 0 
 0 
 0 
 0 
 0 
 0 
 0 
 0 
 0 
 0 
 0 
 0 
 0 
 0 
 0 
 0 
 0 
 0 
 0 
 0 
 0 
 0 
 0 
 0 
 0 
 0 
 0 
 0 
 0 
 0 
 0 
 0 
 0 
 0 
 0 
 0 
 0 
 0 
 0 
 0 
 0 
 0 
 0 
 0 
 0 
 0 
 0 
 0 
 0 
 0 
 0 
 0 
 0 
 0 
 0 
 0 
 0 
 0 
 0 
 
 
 66 
 0 
 0 
 0 
 0 
 0 
 0 
 0 
 0 
 0 
 0 
 0 
 0 
 0 
 0 
 0 
 0 
 0 
 0 
 0 
 0 
 0 
 0 
 0 
 0 
 0 
 0 
 0 
 0 
 0 
 0 
 0 
 0 
 0 
 0 
 0 
 0 
 0 
 0 
 0 
 0 
 0 
 0 
 0 
 0 
 0 
 0 
 0 
 0 
 0 
 0 
 0 
 0 
 0 
 0 
 0 
 0 
 0 
 0 
 0 
 0 
 0 
 0 
 0 
 0 
 0 
 0 
 4 
 0 
 0 
 0 
 0 
 0 
 0 
 0 
 0 
 0 
 0 
 0 
 0 
 0 
 0 
 0 
 0 
 0 
 0 
 0 
 0 
 0 
 0 
 0 
 0 
 0 
 0 
 0 
 0 
 0 
 0 
 0 
 0 
 0 
 0 
 0 
 0 
 0 
 0 
 0 
 0 
 0 
 0 
 0 
 0 
 0 
 0 
 0 
 0 
 0 
 0 
 0 
 0 
 0 
 0 
 0 
 0 
 0 
 0 
 0 
 0 
 0 
 0 
 0 
 0 
 0 
 0 
 0 
 0 
 0 
 0 
 0 
 0 
 0 
 0 
 0 
 0 
 0 
 0 
 0 
 0 
 0 
 0 
 0 
 0 
 0 
 0 
 0 
 0 
 0 
 0 
 0 
 0 
 0 
 0 
 0 
 0 
 0 
 0 
 0 
 0 
 0 
 0 
 0 
 0 
 0 
 0 
 0 
 0 
 0 
 0 
 0 
 0 
 0 
 0 
 0 
 0 
 0 
 0 
 0 
 0 
 0 
 0 
 0 
 0 
 0 
 0 
 0 
 0 
 0 
 0 
 0 
 0 
 0 
 0 
 0 
 0 
 0 
 0 
 0 
 0 
 0 
 0 
 0 
 0 
 0 
 0 
 0 
 0 
 0 
 0 
 0 
 0 
 0 
 0 
 0 
 0 
 0 
 0 
 0 
 0 
 0 
 0 
 0 
 0 
 0 
 0 
 0 
 0 
 0 
 0 
 0 
 0 
 0 
 0 
 0 
 0 
 0 
 0 
 0 
 0 
 0 
 0 
 0 
 0 
 0 
 0 
 0 
 0 
 0 
 0 
 0 
 0 
 0 
 0 
 0 
 0 
 0 
 0 
 0 
 0 
 0 
 0 
 0 
 0 
 0 
 0 
 0 
 0 
 0 
 0 
 0 
 0 
 0 
 0 
 0 
 0 
 0 
 0 
 0 
 0 
 0 
 0 
 0 
 0 
 0 
 0 
 0 
 0 
 0 
 0 
 0 
 0 
 0 
 0 
 0 
 0 
 0 
 0 
 0 
 0 
 0 
 0 
 0 
 0 
 0 
 0 
 0 
 0 
 0 
 0 
 0 
 0 
 0 
 0 
 0 
 0 
 0 
 0 
 0 
 0 
 0 
 0 
 0 
 0 
 0 
 0 
 0 
 0 
 0 
 0 
 0 
 0 
 0 
 0 
 0 
 0 
 0 
 0 
 0 
 0 
 0 
 0 
 0 
 0 
 0 
 0 
 0 
 0 
 0 
 0 
 0 
 0 
 0 
 0 
 0 
 0 
 0 
 0 
 0 
 0 
 0 
 0 
 0 
 0 
 0 
 0 
 0 
 0 
 0 
 0 
 0 
 0 
 0 
 0 
 0 
 0 
 0 
 0 
 0 
 0 
 0 
 0 
 0 
 0 
 0 
 0 
 0 
 0 
 0 
 0 
 0 
 0 
 0 
 0 
 0 
 0 
 0 
 0 
 0 
 0 
 0 
 0 
 0 
 0 
 0 
 0 
 0 
 0 
 0 
 0 
 0 
 0 
 0 
 0 
 0 
 0 
 0 
 0 
 0 
 0 
 0 
 0 
 0 
 0 
 0 
 0 
 0 
 0 
 0 
 0 
 0 
 0 
 0 
 0 
 0 
 0 
 0 
 0 
 0 
 0 
 0 
 0 
 0 
 0 
 0 
 0 
 0 
 0 
 0 
 0 
 0 
 0 
 0 
 0 
 0 
 0 
 0 
 0 
 0 
 0 
 0 
 0 
 0 
 0 
 0 
 0 
 0 
 0 
 0 
 0 
 0 
 0 
 0 
 0 
 0 
 0 
 0 
 0 
 0 
 0 
 0 
 0 
 0 
 0 
 0 
 0 
 0 
 0 
 0 
 0 
 0 
 0 
 0 
 0 
 0 
 0 
 0 
 0 
 0 
 0 
 0 
 0 
 0 
 0 
 0 
 0 
 0 
 0 
 0 
 0 
 0 
 0 
 0 
 0 
 0 
 0 
 0 
 0 
 0 
 0 
 0 
 0 
 0 
 0 
 0 
 0 
 0 
 0 
 0 
 0 
 0 
 0 
 0 
 0 
 0 
 0 
 0 
 0 
 0 
 0 
 0 
 0 
 0 
 0 
 0 
 0 
 0 
 0 
 0 
 0 
 0 
 0 
 0 
 0 
 0 
 0 
 0 
 0 
 0 
 0 
 0 
 0 
 0 
 0 
 0 
 0 
 0 
 0 
 0 
 0 
 0 
 0 
 0 
 0 
 0 
 0 
 0 
 0 
 0 
 0 
 0 
 0 
 0 
 0 
 0 
 0 
 0 
 0 
 0 
 0 
 0 
 0 
 0 
 0 
 0 
 0 
 0 
 0 
 0 
 0 
 0 
 0 
 0 
 0 
 0 
 0 
 0 
 0 
 0 
 0 
 0 
 0 
 0 
 0 
 0 
 0 
 0 
 0 
 0 
 0 
 0 
 0 
 0 
 0 
 0 
 0 
 0 
 0 
 0 
 0 
 0 
 0 
 0 
 0 
 0 
 0 
 0 
 0 
 0 
 0 
 0 
 0 
 0 
 0 
 0 
 0 
 0 
 0 
 0 
 0 
 0 
 0 
 0 
 0 
 0 
 0 
 0 
 0 
 0 
 0 
 0 
 0 
 0 
 0 
 0 
 0 
 0 
 0 
 0 
 0 
 0 
 0 
 0 
 0 
 0 
 0 
 0 
 0 
 0 
 0 
 0 
 0 
 0 
 0 
 0 
 0 
 0 
 0 
 0 
 0 
 0 
 0 
 0 
 0 
 0 
 0 
 0 
 0 
 0 
 0 
 0 
 0 
 0 
 0 
 0 
 0 
 0 
 0 
 0 
 0 
 0 
 0 
 0 
 0 
 0 
 0 
 0 
 0 
 0 
 0 
 0 
 0 
 0 
 0 
 0 
 0 
 0 
 0 
 0 
 0 
 0 
 0 
 0 
 0 
 0 
 0 
 0 
 0 
 0 
 0 
 0 
 0 
 0 
 0 
 0 
 0 
 0 
 0 
 0 
 0 
 0 
 0 
 0 
 0 
 0 
 0 
 0 
 0 
 0 
 0 
 0 
 0 
 0 
 0 
 0 
 0 
 0 
 0 
 0 
 0 
 0 
 0 
 0 
 0 
 0 
 0 
 0 
 0 
 0 
 0 
 0 
 0 
 0 
 0 
 0 
 0 
 0 
 0 
 0 
 0 
 0 
 0 
 0 
 0 
 0 
 0 
 0 
 0 
 0 
 0 
 0 
 0 
 0 
 0 
 0 
 0 
 0 
 0 
 0 
 0 
 0 
 0 
 0 
 0 
 0 
 0 
 0 
 0 
 0 
 
 
 67 
 0 
 0 
 0 
 0 
 0 
 0 
 0 
 0 
 0 
 0 
 0 
 0 
 0 
 0 
 0 
 0 
 0 
 0 
 0 
 0 
 0 
 0 
 0 
 0 
 0 
 0 
 0 
 0 
 0 
 0 
 0 
 0 
 0 
 0 
 0 
 0 
 0 
 0 
 0 
 0 
 0 
 0 
 0 
 0 
 0 
 0 
 0 
 0 
 0 
 0 
 0 
 0 
 0 
 0 
 0 
 0 
 0 
 0 
 0 
 0 
 0 
 0 
 0 
 0 
 0 
 0 
 0 
 38 
 0 
 0 
 0 
 0 
 0 
 0 
 0 
 0 
 0 
 0 
 0 
 0 
 0 
 0 
 0 
 0 
 0 
 0 
 0 
 0 
 0 
 0 
 0 
 0 
 0 
 0 
 0 
 0 
 0 
 0 
 0 
 0 
 0 
 0 
 0 
 0 
 0 
 0 
 0 
 0 
 0 
 0 
 0 
 0 
 0 
 0 
 0 
 0 
 0 
 0 
 0 
 0 
 0 
 0 
 0 
 0 
 0 
 0 
 0 
 0 
 0 
 0 
 0 
 0 
 0 
 0 
 0 
 0 
 0 
 0 
 0 
 0 
 0 
 0 
 0 
 0 
 0 
 0 
 0 
 0 
 0 
 0 
 0 
 0 
 0 
 0 
 0 
 0 
 0 
 0 
 0 
 0 
 0 
 0 
 0 
 0 
 0 
 0 
 0 
 0 
 0 
 0 
 0 
 0 
 0 
 0 
 0 
 0 
 0 
 0 
 0 
 0 
 0 
 0 
 0 
 0 
 0 
 0 
 0 
 0 
 0 
 0 
 0 
 0 
 0 
 0 
 0 
 0 
 0 
 0 
 0 
 0 
 0 
 0 
 0 
 0 
 0 
 0 
 0 
 0 
 0 
 0 
 0 
 0 
 0 
 0 
 0 
 0 
 0 
 0 
 0 
 0 
 0 
 0 
 0 
 0 
 0 
 0 
 0 
 0 
 0 
 0 
 0 
 0 
 0 
 0 
 0 
 0 
 0 
 0 
 0 
 0 
 0 
 0 
 0 
 0 
 0 
 0 
 0 
 0 
 0 
 0 
 0 
 0 
 0 
 0 
 0 
 0 
 0 
 0 
 0 
 0 
 0 
 0 
 0 
 0 
 0 
 0 
 0 
 0 
 0 
 0 
 0 
 0 
 0 
 0 
 0 
 0 
 0 
 0 
 0 
 0 
 0 
 0 
 0 
 0 
 0 
 0 
 0 
 0 
 0 
 0 
 0 
 0 
 0 
 0 
 0 
 0 
 0 
 0 
 0 
 0 
 0 
 0 
 0 
 0 
 0 
 0 
 0 
 0 
 0 
 0 
 0 
 0 
 0 
 0 
 0 
 0 
 0 
 0 
 0 
 0 
 0 
 0 
 0 
 0 
 0 
 0 
 0 
 0 
 0 
 0 
 0 
 0 
 0 
 0 
 0 
 0 
 0 
 0 
 0 
 0 
 0 
 0 
 0 
 0 
 0 
 0 
 0 
 0 
 0 
 0 
 0 
 0 
 0 
 0 
 0 
 0 
 0 
 0 
 0 
 0 
 0 
 0 
 0 
 0 
 0 
 0 
 0 
 0 
 0 
 0 
 0 
 0 
 0 
 0 
 0 
 0 
 0 
 0 
 0 
 0 
 0 
 0 
 0 
 0 
 0 
 0 
 0 
 0 
 0 
 0 
 0 
 0 
 0 
 0 
 0 
 0 
 0 
 0 
 0 
 0 
 0 
 0 
 0 
 0 
 0 
 0 
 0 
 0 
 0 
 0 
 0 
 0 
 0 
 0 
 0 
 0 
 0 
 0 
 0 
 0 
 0 
 0 
 0 
 0 
 0 
 0 
 0 
 0 
 0 
 0 
 0 
 0 
 0 
 0 
 0 
 0 
 0 
 0 
 0 
 0 
 0 
 0 
 0 
 0 
 0 
 0 
 0 
 0 
 0 
 0 
 0 
 0 
 0 
 0 
 0 
 0 
 0 
 0 
 0 
 0 
 0 
 0 
 0 
 0 
 0 
 0 
 0 
 0 
 0 
 0 
 0 
 0 
 0 
 0 
 0 
 0 
 0 
 0 
 0 
 0 
 0 
 0 
 0 
 0 
 0 
 0 
 0 
 0 
 0 
 0 
 0 
 0 
 0 
 0 
 0 
 0 
 0 
 0 
 0 
 0 
 0 
 0 
 0 
 0 
 0 
 0 
 0 
 0 
 0 
 0 
 0 
 0 
 0 
 0 
 0 
 0 
 0 
 0 
 0 
 0 
 0 
 0 
 0 
 0 
 0 
 0 
 0 
 0 
 0 
 0 
 0 
 0 
 0 
 0 
 0 
 0 
 0 
 0 
 0 
 0 
 0 
 0 
 0 
 0 
 0 
 0 
 0 
 0 
 0 
 0 
 0 
 0 
 0 
 0 
 0 
 0 
 0 
 0 
 0 
 0 
 0 
 0 
 0 
 0 
 0 
 0 
 0 
 0 
 0 
 0 
 0 
 0 
 0 
 0 
 0 
 0 
 0 
 0 
 0 
 0 
 0 
 0 
 0 
 0 
 0 
 0 
 0 
 0 
 0 
 0 
 0 
 0 
 0 
 0 
 0 
 0 
 0 
 0 
 0 
 0 
 0 
 0 
 0 
 0 
 0 
 0 
 0 
 0 
 0 
 0 
 0 
 0 
 0 
 0 
 0 
 0 
 0 
 0 
 0 
 0 
 0 
 0 
 0 
 0 
 0 
 0 
 0 
 0 
 0 
 0 
 0 
 0 
 0 
 0 
 0 
 0 
 0 
 0 
 0 
 0 
 0 
 0 
 0 
 0 
 0 
 0 
 0 
 0 
 0 
 0 
 0 
 0 
 0 
 0 
 0 
 0 
 0 
 0 
 0 
 0 
 0 
 0 
 0 
 0 
 0 
 0 
 0 
 0 
 0 
 0 
 0 
 0 
 0 
 0 
 0 
 0 
 0 
 0 
 0 
 0 
 0 
 0 
 0 
 0 
 0 
 0 
 0 
 0 
 0 
 0 
 0 
 0 
 0 
 0 
 0 
 0 
 0 
 0 
 0 
 0 
 0 
 0 
 0 
 0 
 0 
 0 
 0 
 0 
 0 
 0 
 0 
 0 
 0 
 0 
 0 
 0 
 0 
 0 
 0 
 0 
 0 
 0 
 0 
 0 
 0 
 0 
 0 
 0 
 0 
 0 
 0 
 0 
 0 
 0 
 0 
 0 
 0 
 0 
 0 
 0 
 0 
 0 
 0 
 0 
 0 
 0 
 0 
 0 
 0 
 0 
 0 
 0 
 0 
 0 
 0 
 0 
 0 
 0 
 0 
 0 
 0 
 0 
 0 
 0 
 0 
 0 
 0 
 0 
 0 
 0 
 0 
 0 
 0 
 0 
 0 
 0 
 0 
 0 
 0 
 0 
 0 
 0 
 0 
 0 
 0 
 0 
 0 
 0 
 0 
 0 
 0 
 0 
 0 
 0 
 0 
 0 
 0 
 0 
 0 
 0 
 0 
 0 
 0 
 0 
 0 
 0 
 0 
 0 
 0 
 0 
 0 
 0 
 0 
 0 
 0 
 0 
 0 
 0 
 0 
 0 
 0 
 0 
 0 
 0 
 0 
 0 
 
 
 68 
 0 
 0 
 0 
 0 
 0 
 0 
 0 
 0 
 0 
 0 
 0 
 0 
 0 
 0 
 0 
 0 
 0 
 0 
 0 
 0 
 0 
 0 
 0 
 0 
 0 
 0 
 0 
 0 
 0 
 0 
 0 
 0 
 0 
 0 
 0 
 0 
 0 
 0 
 0 
 0 
 0 
 0 
 0 
 0 
 0 
 0 
 0 
 0 
 0 
 0 
 0 
 0 
 0 
 0 
 0 
 0 
 0 
 0 
 0 
 0 
 0 
 0 
 0 
 0 
 0 
 0 
 0 
 0 
 22 
 0 
 0 
 0 
 0 
 0 
 0 
 0 
 0 
 0 
 0 
 0 
 0 
 0 
 0 
 0 
 0 
 0 
 0 
 0 
 0 
 0 
 0 
 0 
 0 
 0 
 0 
 0 
 0 
 0 
 0 
 0 
 0 
 0 
 0 
 0 
 0 
 0 
 0 
 0 
 0 
 0 
 0 
 0 
 0 
 0 
 0 
 0 
 0 
 0 
 0 
 0 
 0 
 0 
 0 
 0 
 0 
 0 
 0 
 0 
 0 
 0 
 0 
 0 
 0 
 0 
 0 
 0 
 0 
 0 
 0 
 0 
 0 
 0 
 0 
 0 
 0 
 0 
 0 
 0 
 0 
 0 
 0 
 0 
 0 
 0 
 0 
 0 
 0 
 0 
 0 
 0 
 0 
 0 
 0 
 0 
 0 
 0 
 0 
 0 
 0 
 0 
 0 
 0 
 0 
 0 
 0 
 0 
 0 
 0 
 0 
 0 
 0 
 0 
 0 
 0 
 0 
 0 
 0 
 0 
 0 
 0 
 0 
 0 
 0 
 0 
 0 
 0 
 0 
 0 
 0 
 0 
 0 
 0 
 0 
 0 
 0 
 0 
 0 
 0 
 0 
 0 
 0 
 0 
 0 
 0 
 0 
 0 
 0 
 0 
 0 
 0 
 0 
 0 
 0 
 0 
 0 
 0 
 0 
 0 
 0 
 0 
 0 
 0 
 0 
 0 
 0 
 0 
 0 
 0 
 0 
 0 
 0 
 0 
 0 
 0 
 0 
 0 
 0 
 0 
 0 
 0 
 0 
 0 
 0 
 0 
 0 
 0 
 0 
 0 
 0 
 0 
 0 
 0 
 0 
 0 
 0 
 0 
 0 
 0 
 0 
 0 
 0 
 0 
 0 
 0 
 0 
 0 
 0 
 0 
 0 
 0 
 0 
 0 
 0 
 0 
 0 
 0 
 0 
 0 
 0 
 0 
 0 
 0 
 0 
 0 
 0 
 0 
 0 
 0 
 0 
 0 
 0 
 0 
 0 
 0 
 0 
 0 
 0 
 0 
 0 
 0 
 0 
 0 
 0 
 0 
 0 
 0 
 0 
 0 
 0 
 0 
 0 
 0 
 0 
 0 
 0 
 0 
 0 
 0 
 0 
 0 
 0 
 0 
 0 
 0 
 0 
 0 
 0 
 0 
 0 
 0 
 0 
 0 
 0 
 0 
 0 
 0 
 0 
 0 
 0 
 0 
 0 
 0 
 0 
 0 
 0 
 0 
 0 
 0 
 0 
 0 
 0 
 0 
 0 
 0 
 0 
 0 
 0 
 0 
 0 
 0 
 0 
 0 
 0 
 0 
 0 
 0 
 0 
 0 
 0 
 0 
 0 
 0 
 0 
 0 
 0 
 0 
 0 
 0 
 0 
 0 
 0 
 0 
 0 
 0 
 0 
 0 
 0 
 0 
 0 
 0 
 0 
 0 
 0 
 0 
 0 
 0 
 0 
 0 
 0 
 0 
 0 
 0 
 0 
 0 
 0 
 0 
 0 
 0 
 0 
 0 
 0 
 0 
 0 
 0 
 0 
 0 
 0 
 0 
 0 
 0 
 0 
 0 
 0 
 0 
 0 
 0 
 0 
 0 
 0 
 0 
 0 
 0 
 0 
 0 
 0 
 0 
 0 
 0 
 0 
 0 
 0 
 0 
 0 
 0 
 0 
 0 
 0 
 0 
 0 
 0 
 0 
 0 
 0 
 0 
 0 
 0 
 0 
 0 
 0 
 0 
 0 
 0 
 0 
 0 
 0 
 0 
 0 
 0 
 0 
 0 
 0 
 0 
 0 
 0 
 0 
 0 
 0 
 0 
 0 
 0 
 0 
 0 
 0 
 0 
 0 
 0 
 0 
 0 
 0 
 0 
 0 
 0 
 0 
 0 
 0 
 0 
 0 
 0 
 0 
 0 
 0 
 0 
 0 
 0 
 0 
 0 
 0 
 0 
 0 
 0 
 0 
 0 
 0 
 0 
 0 
 0 
 0 
 0 
 0 
 0 
 0 
 0 
 0 
 0 
 0 
 0 
 0 
 0 
 0 
 0 
 0 
 0 
 0 
 0 
 0 
 0 
 0 
 0 
 0 
 0 
 0 
 0 
 0 
 0 
 0 
 0 
 0 
 0 
 0 
 0 
 0 
 0 
 0 
 0 
 0 
 0 
 0 
 0 
 0 
 0 
 0 
 0 
 0 
 0 
 0 
 0 
 0 
 0 
 0 
 0 
 0 
 0 
 0 
 0 
 0 
 0 
 0 
 0 
 0 
 0 
 0 
 0 
 0 
 0 
 0 
 0 
 0 
 0 
 0 
 0 
 0 
 0 
 0 
 0 
 0 
 0 
 0 
 0 
 0 
 0 
 0 
 0 
 0 
 0 
 0 
 0 
 0 
 0 
 0 
 0 
 0 
 0 
 0 
 0 
 0 
 0 
 0 
 0 
 0 
 0 
 0 
 0 
 0 
 0 
 0 
 0 
 0 
 0 
 0 
 0 
 0 
 0 
 0 
 0 
 0 
 0 
 0 
 0 
 0 
 0 
 0 
 0 
 0 
 0 
 0 
 0 
 0 
 0 
 0 
 0 
 0 
 0 
 0 
 0 
 0 
 0 
 0 
 0 
 0 
 0 
 0 
 0 
 0 
 0 
 0 
 0 
 0 
 0 
 0 
 0 
 0 
 0 
 0 
 0 
 0 
 0 
 0 
 0 
 0 
 0 
 0 
 0 
 0 
 0 
 0 
 0 
 0 
 0 
 0 
 0 
 0 
 0 
 0 
 0 
 0 
 0 
 0 
 0 
 0 
 0 
 0 
 0 
 0 
 0 
 0 
 0 
 0 
 0 
 0 
 0 
 0 
 0 
 0 
 0 
 0 
 0 
 0 
 0 
 0 
 0 
 0 
 0 
 0 
 0 
 0 
 0 
 0 
 0 
 0 
 0 
 0 
 0 
 0 
 0 
 0 
 0 
 0 
 0 
 0 
 0 
 0 
 0 
 0 
 0 
 0 
 0 
 0 
 0 
 0 
 0 
 0 
 0 
 0 
 0 
 0 
 0 
 0 
 0 
 0 
 0 
 0 
 0 
 0 
 0 
 0 
 0 
 0 
 0 
 0 
 0 
 0 
 0 
 0 
 0 
 0 
 0 
 0 
 0 
 0 
 0 
 0 
 0 
 0 
 0 
 0 
 0 
 0 
 0 
 0 
 0 
 0 
 0 
 0 
 0 
 0 
 0 
 0 
 0 
 0 
 0 
 0 
 0 
 0 
 0 
 0 
 0 
 0 
 0 
 0 
 0 
 0 
 0 
 0 
 0 
 0 
 0 
 
 
 69 
 0 
 0 
 0 
 0 
 0 
 0 
 0 
 0 
 0 
 0 
 0 
 0 
 0 
 0 
 0 
 0 
 0 
 0 
 0 
 0 
 0 
 0 
 0 
 0 
 0 
 0 
 0 
 0 
 0 
 0 
 0 
 0 
 0 
 0 
 0 
 0 
 0 
 0 
 0 
 0 
 0 
 0 
 0 
 0 
 0 
 0 
 0 
 0 
 0 
 0 
 0 
 0 
 0 
 0 
 0 
 0 
 0 
 0 
 0 
 0 
 0 
 0 
 0 
 0 
 0 
 0 
 0 
 0 
 0 
 36 
 0 
 0 
 0 
 0 
 0 
 0 
 0 
 0 
 0 
 0 
 0 
 0 
 0 
 0 
 0 
 0 
 0 
 0 
 0 
 0 
 0 
 0 
 0 
 0 
 0 
 0 
 0 
 0 
 0 
 0 
 0 
 0 
 0 
 0 
 0 
 0 
 0 
 0 
 0 
 0 
 0 
 0 
 0 
 0 
 0 
 0 
 0 
 0 
 0 
 0 
 0 
 0 
 0 
 0 
 0 
 0 
 0 
 0 
 0 
 0 
 0 
 0 
 0 
 0 
 0 
 0 
 0 
 0 
 0 
 0 
 0 
 0 
 0 
 0 
 0 
 0 
 0 
 0 
 0 
 0 
 0 
 0 
 0 
 0 
 0 
 0 
 0 
 0 
 0 
 0 
 0 
 0 
 0 
 0 
 0 
 0 
 0 
 0 
 0 
 0 
 0 
 0 
 0 
 0 
 0 
 0 
 0 
 0 
 0 
 0 
 0 
 0 
 0 
 0 
 0 
 0 
 0 
 0 
 0 
 0 
 0 
 0 
 0 
 0 
 0 
 0 
 0 
 0 
 0 
 0 
 0 
 0 
 0 
 0 
 0 
 0 
 0 
 0 
 0 
 0 
 0 
 0 
 0 
 0 
 0 
 0 
 0 
 0 
 0 
 0 
 0 
 0 
 0 
 0 
 0 
 0 
 0 
 0 
 0 
 0 
 0 
 0 
 0 
 0 
 0 
 0 
 0 
 0 
 0 
 0 
 0 
 0 
 0 
 0 
 0 
 0 
 0 
 0 
 0 
 0 
 0 
 0 
 0 
 0 
 0 
 0 
 0 
 0 
 0 
 0 
 0 
 0 
 0 
 0 
 0 
 0 
 0 
 0 
 0 
 0 
 0 
 0 
 0 
 0 
 0 
 0 
 0 
 0 
 0 
 0 
 0 
 0 
 0 
 0 
 0 
 0 
 0 
 0 
 0 
 0 
 0 
 0 
 0 
 0 
 0 
 0 
 0 
 0 
 0 
 0 
 0 
 0 
 0 
 0 
 0 
 0 
 0 
 0 
 0 
 0 
 0 
 0 
 0 
 0 
 0 
 0 
 0 
 0 
 0 
 0 
 0 
 0 
 0 
 0 
 0 
 0 
 0 
 0 
 0 
 0 
 0 
 0 
 0 
 0 
 0 
 0 
 0 
 0 
 0 
 0 
 0 
 0 
 0 
 0 
 0 
 0 
 0 
 0 
 0 
 0 
 0 
 0 
 0 
 0 
 0 
 0 
 0 
 0 
 0 
 0 
 0 
 0 
 0 
 0 
 0 
 0 
 0 
 0 
 0 
 0 
 0 
 0 
 0 
 0 
 0 
 0 
 0 
 0 
 0 
 0 
 0 
 0 
 0 
 0 
 0 
 0 
 0 
 0 
 0 
 0 
 0 
 0 
 0 
 0 
 0 
 0 
 0 
 0 
 0 
 0 
 0 
 0 
 0 
 0 
 0 
 0 
 0 
 0 
 0 
 0 
 0 
 0 
 0 
 0 
 0 
 0 
 0 
 0 
 0 
 0 
 0 
 0 
 0 
 0 
 0 
 0 
 0 
 0 
 0 
 0 
 0 
 0 
 0 
 0 
 0 
 0 
 0 
 0 
 0 
 0 
 0 
 0 
 0 
 0 
 0 
 0 
 0 
 0 
 0 
 0 
 0 
 0 
 0 
 0 
 0 
 0 
 0 
 0 
 0 
 0 
 0 
 0 
 0 
 0 
 0 
 0 
 0 
 0 
 0 
 0 
 0 
 0 
 0 
 0 
 0 
 0 
 0 
 0 
 0 
 0 
 0 
 0 
 0 
 0 
 0 
 0 
 0 
 0 
 0 
 0 
 0 
 0 
 0 
 0 
 0 
 0 
 0 
 0 
 0 
 0 
 0 
 0 
 0 
 0 
 0 
 0 
 0 
 0 
 0 
 0 
 0 
 0 
 0 
 0 
 0 
 0 
 0 
 0 
 0 
 0 
 0 
 0 
 0 
 0 
 0 
 0 
 0 
 0 
 0 
 0 
 0 
 0 
 0 
 0 
 0 
 0 
 0 
 0 
 0 
 0 
 0 
 0 
 0 
 0 
 0 
 0 
 0 
 0 
 0 
 0 
 0 
 0 
 0 
 0 
 0 
 0 
 0 
 0 
 0 
 0 
 0 
 0 
 0 
 0 
 0 
 0 
 0 
 0 
 0 
 0 
 0 
 0 
 0 
 0 
 0 
 0 
 0 
 0 
 0 
 0 
 0 
 0 
 0 
 0 
 0 
 0 
 0 
 0 
 0 
 0 
 0 
 0 
 0 
 0 
 0 
 0 
 0 
 0 
 0 
 0 
 0 
 0 
 0 
 0 
 0 
 0 
 0 
 0 
 0 
 0 
 0 
 0 
 0 
 0 
 0 
 0 
 0 
 0 
 0 
 0 
 0 
 0 
 0 
 0 
 0 
 0 
 0 
 0 
 0 
 0 
 0 
 0 
 0 
 0 
 0 
 0 
 0 
 0 
 0 
 0 
 0 
 0 
 0 
 0 
 0 
 0 
 0 
 0 
 0 
 0 
 0 
 0 
 0 
 0 
 0 
 0 
 0 
 0 
 0 
 0 
 0 
 0 
 0 
 0 
 0 
 0 
 0 
 0 
 0 
 0 
 0 
 0 
 0 
 0 
 0 
 0 
 0 
 0 
 0 
 0 
 0 
 0 
 0 
 0 
 0 
 0 
 0 
 0 
 0 
 0 
 0 
 0 
 0 
 0 
 0 
 0 
 0 
 0 
 0 
 0 
 0 
 0 
 0 
 0 
 0 
 0 
 0 
 0 
 0 
 0 
 0 
 0 
 0 
 0 
 0 
 0 
 0 
 0 
 0 
 0 
 0 
 0 
 0 
 0 
 0 
 0 
 0 
 0 
 0 
 0 
 0 
 0 
 0 
 0 
 0 
 0 
 0 
 0 
 0 
 0 
 0 
 0 
 0 
 0 
 0 
 0 
 0 
 0 
 0 
 0 
 0 
 0 
 0 
 0 
 0 
 0 
 0 
 0 
 0 
 0 
 0 
 0 
 0 
 0 
 0 
 0 
 0 
 0 
 0 
 0 
 0 
 0 
 0 
 0 
 0 
 0 
 0 
 0 
 0 
 0 
 0 
 0 
 0 
 0 
 0 
 0 
 0 
 0 
 0 
 0 
 0 
 0 
 0 
 0 
 0 
 0 
 0 
 0 
 0 
 0 
 0 
 0 
 0 
 0 
 0 
 0 
 0 
 0 
 0 
 0 
 0 
 0 
 0 
 0 
 0 
 0 
 0 
 0 
 0 
 0 
 0 
 0 
 0 
 0 
 0 
 0 
 
 
 70 
 0 
 0 
 0 
 0 
 0 
 0 
 0 
 0 
 0 
 0 
 0 
 0 
 0 
 0 
 0 
 0 
 0 
 0 
 0 
 0 
 0 
 0 
 0 
 0 
 0 
 0 
 0 
 0 
 0 
 0 
 0 
 0 
 0 
 0 
 0 
 0 
 0 
 0 
 0 
 0 
 0 
 0 
 0 
 0 
 0 
 0 
 0 
 0 
 0 
 0 
 0 
 0 
 0 
 0 
 0 
 0 
 0 
 0 
 0 
 0 
 0 
 0 
 0 
 0 
 0 
 0 
 0 
 0 
 0 
 0 
 30 
 0 
 0 
 0 
 0 
 0 
 0 
 0 
 0 
 0 
 0 
 0 
 0 
 0 
 0 
 0 
 0 
 0 
 0 
 0 
 0 
 0 
 0 
 0 
 0 
 0 
 0 
 0 
 0 
 0 
 0 
 0 
 0 
 0 
 0 
 0 
 0 
 0 
 0 
 0 
 0 
 0 
 0 
 0 
 0 
 0 
 0 
 0 
 0 
 0 
 0 
 0 
 0 
 0 
 0 
 0 
 0 
 0 
 0 
 0 
 0 
 0 
 0 
 0 
 0 
 0 
 0 
 0 
 0 
 0 
 0 
 0 
 0 
 0 
 0 
 0 
 0 
 0 
 0 
 0 
 0 
 0 
 0 
 0 
 0 
 0 
 0 
 0 
 0 
 0 
 0 
 0 
 0 
 0 
 0 
 0 
 0 
 0 
 0 
 0 
 0 
 0 
 0 
 0 
 0 
 0 
 0 
 0 
 0 
 0 
 0 
 0 
 0 
 0 
 0 
 0 
 0 
 0 
 0 
 0 
 0 
 0 
 0 
 0 
 0 
 0 
 0 
 0 
 0 
 0 
 0 
 0 
 0 
 0 
 0 
 0 
 0 
 0 
 0 
 0 
 0 
 0 
 0 
 0 
 0 
 0 
 0 
 0 
 0 
 0 
 0 
 0 
 0 
 0 
 0 
 0 
 0 
 0 
 0 
 0 
 0 
 0 
 0 
 0 
 0 
 0 
 0 
 0 
 0 
 0 
 0 
 0 
 0 
 0 
 0 
 0 
 0 
 0 
 0 
 0 
 0 
 0 
 0 
 0 
 0 
 0 
 0 
 0 
 0 
 0 
 0 
 0 
 0 
 0 
 0 
 0 
 0 
 0 
 0 
 0 
 0 
 0 
 0 
 0 
 0 
 0 
 0 
 0 
 0 
 0 
 0 
 0 
 0 
 0 
 0 
 0 
 0 
 0 
 0 
 0 
 0 
 0 
 0 
 0 
 0 
 0 
 0 
 0 
 0 
 0 
 0 
 0 
 0 
 0 
 0 
 0 
 0 
 0 
 0 
 0 
 0 
 0 
 0 
 0 
 0 
 0 
 0 
 0 
 0 
 0 
 0 
 0 
 0 
 0 
 0 
 0 
 0 
 0 
 0 
 0 
 0 
 0 
 0 
 0 
 0 
 0 
 0 
 0 
 0 
 0 
 0 
 0 
 0 
 0 
 0 
 0 
 0 
 0 
 0 
 0 
 0 
 0 
 0 
 0 
 0 
 0 
 0 
 0 
 0 
 0 
 0 
 0 
 0 
 0 
 0 
 0 
 0 
 0 
 0 
 0 
 0 
 0 
 0 
 0 
 0 
 0 
 0 
 0 
 0 
 0 
 0 
 0 
 0 
 0 
 0 
 0 
 0 
 0 
 0 
 0 
 0 
 0 
 0 
 0 
 0 
 0 
 0 
 0 
 0 
 0 
 0 
 0 
 0 
 0 
 0 
 0 
 0 
 0 
 0 
 0 
 0 
 0 
 0 
 0 
 0 
 0 
 0 
 0 
 0 
 0 
 0 
 0 
 0 
 0 
 0 
 0 
 0 
 0 
 0 
 0 
 0 
 0 
 0 
 0 
 0 
 0 
 0 
 0 
 0 
 0 
 0 
 0 
 0 
 0 
 0 
 0 
 0 
 0 
 0 
 0 
 0 
 0 
 0 
 0 
 0 
 0 
 0 
 0 
 0 
 0 
 0 
 0 
 0 
 0 
 0 
 0 
 0 
 0 
 0 
 0 
 0 
 0 
 0 
 0 
 0 
 0 
 0 
 0 
 0 
 0 
 0 
 0 
 0 
 0 
 0 
 0 
 0 
 0 
 0 
 0 
 0 
 0 
 0 
 0 
 0 
 0 
 0 
 0 
 0 
 0 
 0 
 0 
 0 
 0 
 0 
 0 
 0 
 0 
 0 
 0 
 0 
 0 
 0 
 0 
 0 
 0 
 0 
 0 
 0 
 0 
 0 
 0 
 0 
 0 
 0 
 0 
 0 
 0 
 0 
 0 
 0 
 0 
 0 
 0 
 0 
 0 
 0 
 0 
 0 
 0 
 0 
 0 
 0 
 0 
 0 
 0 
 0 
 0 
 0 
 0 
 0 
 0 
 0 
 0 
 0 
 0 
 0 
 0 
 0 
 0 
 0 
 0 
 0 
 0 
 0 
 0 
 0 
 0 
 0 
 0 
 0 
 0 
 0 
 0 
 0 
 0 
 0 
 0 
 0 
 0 
 0 
 0 
 0 
 0 
 0 
 0 
 0 
 0 
 0 
 0 
 0 
 0 
 0 
 0 
 0 
 0 
 0 
 0 
 0 
 0 
 0 
 0 
 0 
 0 
 0 
 0 
 0 
 0 
 0 
 0 
 0 
 0 
 0 
 0 
 0 
 0 
 0 
 0 
 0 
 0 
 0 
 0 
 0 
 0 
 0 
 0 
 0 
 0 
 0 
 0 
 0 
 0 
 0 
 0 
 0 
 0 
 0 
 0 
 0 
 0 
 0 
 0 
 0 
 0 
 0 
 0 
 0 
 0 
 0 
 0 
 0 
 0 
 0 
 0 
 0 
 0 
 0 
 0 
 0 
 0 
 0 
 0 
 0 
 0 
 0 
 0 
 0 
 0 
 0 
 0 
 0 
 0 
 0 
 0 
 0 
 0 
 0 
 0 
 0 
 0 
 0 
 0 
 0 
 0 
 0 
 0 
 0 
 0 
 0 
 0 
 0 
 0 
 0 
 0 
 0 
 0 
 0 
 0 
 0 
 0 
 0 
 0 
 0 
 0 
 0 
 0 
 0 
 0 
 0 
 0 
 0 
 0 
 0 
 0 
 0 
 0 
 0 
 0 
 0 
 0 
 0 
 0 
 0 
 0 
 0 
 0 
 0 
 0 
 0 
 0 
 0 
 0 
 0 
 0 
 0 
 0 
 0 
 0 
 0 
 0 
 0 
 0 
 0 
 0 
 0 
 0 
 0 
 0 
 0 
 0 
 0 
 0 
 0 
 0 
 0 
 0 
 0 
 0 
 0 
 0 
 0 
 0 
 0 
 0 
 0 
 0 
 0 
 0 
 0 
 0 
 0 
 0 
 0 
 0 
 0 
 0 
 0 
 0 
 0 
 0 
 0 
 0 
 0 
 0 
 0 
 0 
 0 
 0 
 0 
 0 
 0 
 0 
 0 
 0 
 0 
 0 
 0 
 0 
 0 
 0 
 0 
 0 
 0 
 0 
 0 
 0 
 0 
 0 
 0 
 0 
 0 
 0 
 0 
 0 
 0 
 0 
 0 
 0 
 0 
 0 
 0 
 0 
 0 
 0 
 0 
 0 
 
 
 71 
 0 
 0 
 0 
 0 
 0 
 0 
 0 
 0 
 0 
 0 
 0 
 0 
 0 
 0 
 0 
 0 
 0 
 0 
 0 
 0 
 0 
 0 
 0 
 0 
 0 
 0 
 0 
 0 
 0 
 0 
 0 
 0 
 0 
 0 
 0 
 0 
 0 
 0 
 0 
 0 
 0 
 0 
 0 
 0 
 0 
 0 
 0 
 0 
 0 
 0 
 0 
 0 
 0 
 0 
 0 
 0 
 0 
 0 
 0 
 0 
 0 
 0 
 0 
 0 
 0 
 0 
 0 
 0 
 0 
 0 
 0 
 31 
 0 
 0 
 0 
 0 
 0 
 0 
 0 
 0 
 0 
 0 
 0 
 0 
 0 
 0 
 0 
 0 
 0 
 0 
 0 
 0 
 0 
 0 
 11 
 0 
 0 
 0 
 0 
 0 
 0 
 0 
 0 
 0 
 0 
 0 
 0 
 0 
 0 
 0 
 0 
 0 
 0 
 0 
 0 
 0 
 0 
 0 
 0 
 0 
 0 
 0 
 0 
 0 
 0 
 0 
 0 
 0 
 0 
 0 
 0 
 0 
 0 
 0 
 0 
 0 
 0 
 0 
 0 
 0 
 0 
 0 
 0 
 0 
 0 
 0 
 0 
 0 
 0 
 0 
 0 
 0 
 0 
 0 
 0 
 0 
 0 
 0 
 0 
 0 
 0 
 0 
 0 
 0 
 0 
 0 
 0 
 0 
 0 
 0 
 0 
 0 
 0 
 0 
 0 
 0 
 0 
 0 
 0 
 0 
 0 
 0 
 0 
 0 
 0 
 0 
 0 
 0 
 0 
 0 
 0 
 0 
 0 
 0 
 0 
 0 
 0 
 0 
 0 
 0 
 0 
 0 
 0 
 0 
 0 
 0 
 0 
 0 
 0 
 0 
 0 
 0 
 0 
 0 
 0 
 0 
 0 
 0 
 0 
 0 
 0 
 0 
 0 
 0 
 0 
 0 
 0 
 0 
 0 
 0 
 0 
 0 
 0 
 0 
 0 
 0 
 0 
 0 
 0 
 0 
 0 
 0 
 0 
 0 
 0 
 0 
 0 
 0 
 0 
 0 
 0 
 0 
 0 
 0 
 0 
 0 
 0 
 0 
 0 
 0 
 0 
 0 
 0 
 0 
 0 
 0 
 0 
 0 
 0 
 0 
 0 
 0 
 0 
 0 
 0 
 0 
 0 
 0 
 0 
 0 
 0 
 0 
 0 
 0 
 0 
 0 
 0 
 0 
 0 
 0 
 0 
 0 
 0 
 0 
 0 
 0 
 0 
 0 
 0 
 0 
 0 
 0 
 0 
 0 
 0 
 0 
 0 
 0 
 0 
 0 
 0 
 0 
 0 
 0 
 0 
 0 
 0 
 0 
 0 
 0 
 0 
 0 
 0 
 0 
 0 
 0 
 0 
 0 
 0 
 0 
 0 
 0 
 0 
 0 
 0 
 0 
 0 
 0 
 0 
 0 
 0 
 0 
 0 
 0 
 0 
 0 
 0 
 0 
 0 
 0 
 0 
 0 
 0 
 0 
 0 
 0 
 0 
 0 
 0 
 0 
 0 
 0 
 0 
 0 
 0 
 0 
 0 
 0 
 0 
 0 
 0 
 0 
 0 
 0 
 0 
 0 
 0 
 0 
 0 
 0 
 0 
 0 
 0 
 0 
 0 
 0 
 0 
 0 
 0 
 0 
 0 
 0 
 0 
 0 
 0 
 0 
 0 
 0 
 0 
 0 
 0 
 0 
 0 
 0 
 0 
 0 
 0 
 0 
 0 
 0 
 0 
 0 
 0 
 0 
 0 
 0 
 0 
 0 
 0 
 0 
 0 
 0 
 0 
 0 
 0 
 0 
 0 
 0 
 0 
 0 
 0 
 0 
 0 
 0 
 0 
 0 
 0 
 0 
 0 
 0 
 0 
 0 
 0 
 0 
 0 
 0 
 0 
 0 
 0 
 0 
 0 
 0 
 0 
 0 
 0 
 0 
 0 
 0 
 0 
 0 
 0 
 0 
 0 
 0 
 0 
 0 
 0 
 0 
 0 
 0 
 0 
 0 
 0 
 0 
 0 
 0 
 0 
 0 
 0 
 0 
 0 
 0 
 0 
 0 
 0 
 0 
 0 
 0 
 0 
 0 
 0 
 0 
 0 
 0 
 0 
 0 
 0 
 0 
 0 
 0 
 0 
 0 
 0 
 0 
 0 
 0 
 0 
 0 
 0 
 0 
 0 
 0 
 0 
 0 
 0 
 0 
 0 
 0 
 0 
 0 
 0 
 0 
 0 
 0 
 0 
 0 
 0 
 0 
 0 
 0 
 0 
 0 
 0 
 0 
 0 
 0 
 0 
 0 
 0 
 0 
 0 
 0 
 0 
 0 
 0 
 0 
 0 
 0 
 0 
 0 
 0 
 0 
 0 
 0 
 0 
 0 
 0 
 0 
 0 
 0 
 0 
 0 
 0 
 0 
 0 
 0 
 0 
 0 
 0 
 0 
 0 
 0 
 0 
 0 
 0 
 0 
 0 
 0 
 0 
 0 
 0 
 0 
 0 
 0 
 0 
 0 
 0 
 0 
 0 
 0 
 0 
 0 
 0 
 0 
 0 
 0 
 0 
 0 
 0 
 0 
 0 
 0 
 0 
 0 
 0 
 0 
 0 
 0 
 0 
 0 
 0 
 0 
 0 
 0 
 0 
 0 
 0 
 0 
 0 
 0 
 0 
 0 
 0 
 0 
 0 
 0 
 0 
 0 
 0 
 0 
 0 
 0 
 0 
 0 
 0 
 0 
 0 
 0 
 0 
 0 
 0 
 0 
 0 
 0 
 0 
 0 
 0 
 0 
 0 
 0 
 0 
 0 
 0 
 0 
 0 
 0 
 0 
 0 
 0 
 0 
 0 
 0 
 0 
 0 
 0 
 0 
 0 
 0 
 0 
 0 
 0 
 0 
 0 
 0 
 0 
 0 
 0 
 0 
 0 
 0 
 0 
 0 
 0 
 0 
 0 
 0 
 0 
 0 
 0 
 0 
 0 
 0 
 0 
 0 
 0 
 0 
 0 
 0 
 0 
 0 
 0 
 0 
 0 
 0 
 0 
 0 
 0 
 0 
 0 
 0 
 0 
 0 
 0 
 0 
 0 
 0 
 0 
 0 
 0 
 0 
 0 
 0 
 0 
 0 
 0 
 0 
 0 
 0 
 0 
 0 
 0 
 0 
 0 
 0 
 0 
 0 
 0 
 0 
 0 
 0 
 0 
 0 
 0 
 0 
 0 
 0 
 0 
 0 
 0 
 0 
 0 
 0 
 0 
 0 
 0 
 0 
 0 
 0 
 0 
 0 
 0 
 0 
 0 
 0 
 0 
 0 
 0 
 0 
 0 
 0 
 0 
 0 
 0 
 0 
 0 
 0 
 0 
 0 
 0 
 0 
 0 
 0 
 0 
 0 
 0 
 0 
 0 
 0 
 0 
 0 
 0 
 0 
 0 
 0 
 0 
 0 
 0 
 0 
 0 
 0 
 0 
 0 
 0 
 0 
 0 
 0 
 0 
 0 
 0 
 0 
 0 
 0 
 0 
 0 
 0 
 0 
 0 
 0 
 0 
 0 
 0 
 0 
 0 
 0 
 0 
 0 
 
 
 72 
 0 
 0 
 0 
 0 
 0 
 0 
 0 
 0 
 0 
 0 
 0 
 0 
 0 
 0 
 0 
 0 
 0 
 0 
 0 
 0 
 0 
 0 
 0 
 0 
 0 
 0 
 0 
 0 
 0 
 0 
 0 
 0 
 0 
 0 
 0 
 0 
 0 
 0 
 0 
 0 
 0 
 0 
 0 
 0 
 0 
 0 
 0 
 0 
 0 
 0 
 0 
 0 
 0 
 0 
 0 
 0 
 0 
 0 
 0 
 0 
 0 
 0 
 0 
 0 
 0 
 0 
 0 
 0 
 0 
 0 
 0 
 0 
 0 
 0 
 0 
 0 
 0 
 2 
 0 
 0 
 0 
 0 
 0 
 1 
 0 
 0 
 0 
 0 
 0 
 0 
 0 
 0 
 0 
 0 
 0 
 0 
 0 
 0 
 0 
 0 
 0 
 0 
 0 
 0 
 0 
 0 
 0 
 0 
 0 
 0 
 0 
 0 
 0 
 0 
 0 
 0 
 0 
 0 
 0 
 0 
 0 
 0 
 0 
 0 
 0 
 0 
 0 
 0 
 0 
 0 
 0 
 0 
 0 
 0 
 0 
 0 
 0 
 0 
 0 
 0 
 0 
 0 
 0 
 0 
 0 
 0 
 0 
 0 
 0 
 0 
 0 
 0 
 0 
 0 
 0 
 0 
 0 
 0 
 0 
 0 
 0 
 0 
 0 
 0 
 0 
 0 
 0 
 0 
 0 
 0 
 0 
 0 
 0 
 0 
 0 
 0 
 0 
 0 
 0 
 0 
 0 
 0 
 0 
 0 
 0 
 0 
 0 
 0 
 0 
 0 
 0 
 0 
 0 
 0 
 0 
 0 
 0 
 0 
 0 
 0 
 0 
 0 
 0 
 0 
 0 
 0 
 0 
 0 
 0 
 0 
 0 
 0 
 0 
 0 
 0 
 0 
 0 
 0 
 0 
 0 
 0 
 0 
 0 
 0 
 0 
 0 
 0 
 0 
 0 
 0 
 0 
 0 
 0 
 0 
 0 
 0 
 0 
 0 
 0 
 0 
 0 
 0 
 0 
 0 
 0 
 0 
 0 
 0 
 0 
 0 
 0 
 0 
 0 
 0 
 0 
 0 
 0 
 0 
 0 
 0 
 0 
 0 
 0 
 0 
 0 
 0 
 0 
 0 
 0 
 0 
 0 
 0 
 0 
 0 
 0 
 0 
 0 
 0 
 0 
 0 
 0 
 0 
 0 
 0 
 0 
 0 
 0 
 0 
 0 
 0 
 0 
 0 
 0 
 0 
 0 
 0 
 0 
 0 
 0 
 0 
 0 
 0 
 0 
 0 
 0 
 0 
 0 
 0 
 0 
 0 
 0 
 0 
 0 
 0 
 0 
 0 
 0 
 0 
 0 
 0 
 0 
 0 
 0 
 0 
 0 
 0 
 0 
 0 
 0 
 0 
 0 
 0 
 0 
 0 
 0 
 0 
 0 
 0 
 0 
 0 
 0 
 0 
 0 
 0 
 0 
 0 
 0 
 0 
 0 
 0 
 0 
 0 
 0 
 0 
 0 
 0 
 0 
 0 
 0 
 0 
 0 
 0 
 0 
 0 
 0 
 0 
 0 
 0 
 0 
 0 
 0 
 0 
 0 
 0 
 0 
 0 
 0 
 0 
 0 
 0 
 0 
 0 
 0 
 0 
 0 
 0 
 0 
 0 
 0 
 0 
 0 
 0 
 0 
 0 
 0 
 0 
 0 
 0 
 0 
 0 
 0 
 0 
 0 
 0 
 0 
 0 
 0 
 0 
 0 
 0 
 0 
 0 
 0 
 0 
 0 
 0 
 0 
 0 
 0 
 0 
 0 
 0 
 0 
 0 
 0 
 0 
 0 
 0 
 0 
 0 
 0 
 0 
 0 
 0 
 0 
 0 
 0 
 0 
 0 
 0 
 0 
 0 
 0 
 0 
 0 
 0 
 0 
 0 
 0 
 0 
 0 
 0 
 0 
 0 
 0 
 0 
 0 
 0 
 0 
 0 
 0 
 0 
 0 
 0 
 0 
 0 
 0 
 0 
 0 
 0 
 0 
 0 
 0 
 0 
 0 
 0 
 0 
 0 
 0 
 0 
 0 
 0 
 0 
 0 
 0 
 0 
 0 
 0 
 0 
 0 
 0 
 0 
 0 
 0 
 0 
 0 
 0 
 0 
 0 
 0 
 0 
 0 
 0 
 0 
 0 
 0 
 0 
 0 
 0 
 0 
 0 
 0 
 0 
 0 
 0 
 0 
 0 
 0 
 0 
 0 
 0 
 0 
 0 
 0 
 0 
 0 
 0 
 0 
 0 
 0 
 0 
 0 
 0 
 0 
 0 
 0 
 0 
 0 
 0 
 0 
 0 
 0 
 0 
 0 
 0 
 0 
 0 
 0 
 0 
 0 
 0 
 0 
 0 
 0 
 0 
 0 
 0 
 0 
 0 
 0 
 0 
 0 
 0 
 0 
 0 
 0 
 0 
 0 
 0 
 0 
 0 
 0 
 0 
 0 
 0 
 0 
 0 
 0 
 0 
 0 
 0 
 0 
 0 
 0 
 0 
 0 
 0 
 0 
 0 
 0 
 0 
 0 
 0 
 0 
 0 
 0 
 0 
 0 
 0 
 0 
 0 
 0 
 0 
 0 
 0 
 0 
 0 
 0 
 0 
 0 
 0 
 0 
 0 
 0 
 0 
 0 
 0 
 0 
 0 
 0 
 0 
 0 
 0 
 0 
 0 
 0 
 0 
 0 
 0 
 0 
 0 
 0 
 0 
 0 
 0 
 0 
 0 
 0 
 0 
 0 
 0 
 0 
 0 
 0 
 0 
 0 
 0 
 0 
 0 
 0 
 0 
 0 
 0 
 0 
 0 
 0 
 0 
 0 
 0 
 0 
 0 
 0 
 0 
 0 
 0 
 0 
 0 
 0 
 0 
 0 
 0 
 0 
 0 
 0 
 0 
 0 
 0 
 0 
 0 
 0 
 0 
 0 
 0 
 0 
 0 
 0 
 0 
 0 
 0 
 0 
 0 
 0 
 0 
 0 
 0 
 0 
 0 
 0 
 0 
 0 
 0 
 0 
 0 
 0 
 0 
 0 
 0 
 0 
 0 
 0 
 0 
 0 
 0 
 0 
 0 
 0 
 0 
 0 
 0 
 0 
 0 
 0 
 0 
 0 
 0 
 0 
 0 
 0 
 0 
 0 
 0 
 0 
 0 
 0 
 0 
 0 
 0 
 0 
 0 
 0 
 0 
 0 
 0 
 0 
 0 
 0 
 0 
 0 
 0 
 0 
 0 
 0 
 0 
 0 
 0 
 0 
 0 
 0 
 0 
 0 
 0 
 0 
 0 
 0 
 0 
 0 
 0 
 0 
 0 
 0 
 0 
 0 
 0 
 0 
 0 
 0 
 0 
 0 
 0 
 0 
 0 
 0 
 0 
 0 
 0 
 0 
 0 
 0 
 0 
 0 
 0 
 0 
 0 
 0 
 0 
 0 
 0 
 0 
 0 
 0 
 0 
 0 
 0 
 0 
 0 
 0 
 0 
 0 
 0 
 0 
 0 
 0 
 0 
 0 
 0 
 0 
 0 
 0 
 0 
 0 
 0 
 0 
 0 
 0 
 0 
 0 
 
 
 73 
 0 
 0 
 0 
 0 
 0 
 0 
 0 
 0 
 0 
 0 
 0 
 0 
 0 
 0 
 0 
 0 
 0 
 0 
 0 
 0 
 0 
 0 
 0 
 0 
 0 
 0 
 0 
 0 
 0 
 0 
 0 
 0 
 0 
 0 
 0 
 0 
 0 
 0 
 0 
 0 
 0 
 0 
 0 
 0 
 0 
 0 
 0 
 0 
 0 
 0 
 0 
 0 
 0 
 0 
 0 
 0 
 0 
 0 
 0 
 0 
 0 
 0 
 0 
 0 
 0 
 0 
 0 
 0 
 0 
 0 
 0 
 0 
 0 
 16 
 0 
 0 
 0 
 0 
 0 
 0 
 0 
 0 
 0 
 0 
 0 
 0 
 0 
 0 
 0 
 0 
 0 
 0 
 0 
 0 
 0 
 0 
 0 
 0 
 0 
 0 
 0 
 0 
 0 
 0 
 0 
 0 
 0 
 0 
 0 
 0 
 0 
 0 
 0 
 0 
 0 
 0 
 0 
 0 
 0 
 0 
 0 
 0 
 0 
 0 
 0 
 0 
 0 
 0 
 0 
 0 
 0 
 0 
 0 
 0 
 0 
 0 
 0 
 0 
 0 
 0 
 0 
 0 
 0 
 0 
 0 
 0 
 0 
 0 
 0 
 0 
 0 
 0 
 0 
 0 
 0 
 0 
 0 
 0 
 0 
 0 
 0 
 0 
 0 
 0 
 0 
 0 
 0 
 0 
 0 
 0 
 0 
 0 
 0 
 0 
 0 
 0 
 0 
 0 
 0 
 0 
 0 
 0 
 0 
 0 
 0 
 0 
 0 
 0 
 0 
 0 
 0 
 0 
 0 
 0 
 0 
 0 
 0 
 0 
 0 
 0 
 0 
 0 
 0 
 0 
 0 
 0 
 0 
 0 
 0 
 0 
 0 
 0 
 0 
 0 
 0 
 0 
 0 
 0 
 0 
 0 
 0 
 0 
 0 
 0 
 0 
 0 
 0 
 0 
 0 
 0 
 0 
 0 
 0 
 0 
 0 
 0 
 0 
 0 
 0 
 0 
 0 
 0 
 0 
 0 
 0 
 0 
 0 
 0 
 0 
 0 
 0 
 0 
 0 
 0 
 0 
 0 
 0 
 0 
 0 
 0 
 0 
 0 
 0 
 0 
 0 
 0 
 0 
 0 
 0 
 0 
 0 
 0 
 0 
 0 
 0 
 0 
 0 
 0 
 0 
 0 
 0 
 0 
 0 
 0 
 0 
 0 
 0 
 0 
 0 
 0 
 0 
 0 
 0 
 0 
 0 
 0 
 0 
 0 
 0 
 0 
 0 
 0 
 0 
 0 
 0 
 0 
 0 
 0 
 0 
 0 
 0 
 0 
 0 
 0 
 0 
 0 
 0 
 0 
 0 
 0 
 0 
 0 
 0 
 0 
 0 
 0 
 0 
 0 
 0 
 0 
 0 
 0 
 0 
 0 
 0 
 0 
 0 
 0 
 0 
 0 
 0 
 0 
 0 
 0 
 0 
 0 
 0 
 0 
 0 
 0 
 0 
 0 
 0 
 0 
 0 
 0 
 0 
 0 
 0 
 0 
 0 
 0 
 0 
 0 
 0 
 0 
 0 
 0 
 0 
 0 
 0 
 0 
 0 
 0 
 0 
 0 
 0 
 0 
 0 
 0 
 0 
 0 
 0 
 0 
 0 
 0 
 0 
 0 
 0 
 0 
 0 
 0 
 0 
 0 
 0 
 0 
 0 
 0 
 0 
 0 
 0 
 0 
 0 
 0 
 0 
 0 
 0 
 0 
 0 
 0 
 0 
 0 
 0 
 0 
 0 
 0 
 0 
 0 
 0 
 0 
 0 
 0 
 0 
 0 
 0 
 0 
 0 
 0 
 0 
 0 
 0 
 0 
 0 
 0 
 0 
 0 
 0 
 0 
 0 
 0 
 0 
 0 
 0 
 0 
 0 
 0 
 0 
 0 
 0 
 0 
 0 
 0 
 0 
 0 
 0 
 0 
 0 
 0 
 0 
 0 
 0 
 0 
 0 
 0 
 0 
 0 
 0 
 0 
 0 
 0 
 0 
 0 
 0 
 0 
 0 
 0 
 0 
 0 
 0 
 0 
 0 
 0 
 0 
 0 
 0 
 0 
 0 
 0 
 0 
 0 
 0 
 0 
 0 
 0 
 0 
 0 
 0 
 0 
 0 
 0 
 0 
 0 
 0 
 0 
 0 
 0 
 0 
 0 
 0 
 0 
 0 
 0 
 0 
 0 
 0 
 0 
 0 
 0 
 0 
 0 
 0 
 0 
 0 
 0 
 0 
 0 
 0 
 0 
 0 
 0 
 0 
 0 
 0 
 0 
 0 
 0 
 0 
 0 
 0 
 0 
 0 
 0 
 0 
 0 
 0 
 0 
 0 
 0 
 0 
 0 
 0 
 0 
 0 
 0 
 0 
 0 
 0 
 0 
 0 
 0 
 0 
 0 
 0 
 0 
 0 
 0 
 0 
 0 
 0 
 0 
 0 
 0 
 0 
 0 
 0 
 0 
 0 
 0 
 0 
 0 
 0 
 0 
 0 
 0 
 0 
 0 
 0 
 0 
 0 
 0 
 0 
 0 
 0 
 0 
 0 
 0 
 0 
 0 
 0 
 0 
 0 
 0 
 0 
 0 
 0 
 0 
 0 
 0 
 0 
 0 
 0 
 0 
 0 
 0 
 0 
 0 
 0 
 0 
 0 
 0 
 0 
 0 
 0 
 0 
 0 
 0 
 0 
 0 
 0 
 0 
 0 
 0 
 0 
 0 
 0 
 0 
 0 
 0 
 0 
 0 
 0 
 0 
 0 
 0 
 0 
 0 
 0 
 0 
 0 
 0 
 0 
 0 
 0 
 0 
 0 
 0 
 0 
 0 
 0 
 0 
 0 
 0 
 0 
 0 
 0 
 0 
 0 
 0 
 0 
 0 
 0 
 0 
 0 
 0 
 0 
 0 
 0 
 0 
 0 
 0 
 0 
 0 
 0 
 0 
 0 
 0 
 0 
 0 
 0 
 0 
 0 
 0 
 0 
 0 
 0 
 0 
 0 
 0 
 0 
 0 
 0 
 0 
 0 
 0 
 0 
 0 
 0 
 0 
 0 
 0 
 0 
 0 
 0 
 0 
 0 
 0 
 0 
 0 
 0 
 0 
 0 
 0 
 0 
 0 
 0 
 0 
 0 
 0 
 0 
 0 
 0 
 0 
 0 
 0 
 0 
 0 
 0 
 0 
 0 
 0 
 0 
 0 
 0 
 0 
 0 
 0 
 0 
 0 
 0 
 0 
 0 
 0 
 0 
 0 
 0 
 0 
 0 
 0 
 0 
 0 
 0 
 0 
 0 
 0 
 0 
 0 
 0 
 0 
 0 
 0 
 0 
 0 
 0 
 0 
 0 
 0 
 0 
 0 
 0 
 0 
 0 
 0 
 0 
 0 
 0 
 0 
 0 
 0 
 0 
 0 
 0 
 0 
 0 
 0 
 0 
 0 
 0 
 0 
 0 
 0 
 0 
 0 
 0 
 0 
 0 
 0 
 0 
 0 
 0 
 0 
 0 
 0 
 0 
 0 
 0 
 0 
 0 
 0 
 0 
 0 
 0 
 0 
 0 
 0 
 0 
 0 
 
 
 74 
 0 
 0 
 0 
 0 
 0 
 0 
 0 
 0 
 0 
 0 
 0 
 0 
 0 
 0 
 0 
 0 
 0 
 0 
 0 
 0 
 0 
 0 
 0 
 0 
 0 
 0 
 0 
 0 
 0 
 0 
 0 
 0 
 0 
 0 
 0 
 0 
 0 
 0 
 0 
 0 
 0 
 0 
 0 
 0 
 0 
 0 
 0 
 0 
 0 
 0 
 0 
 0 
 0 
 0 
 0 
 0 
 0 
 0 
 0 
 0 
 0 
 0 
 0 
 0 
 0 
 0 
 0 
 0 
 0 
 0 
 0 
 0 
 0 
 1 
 1 
 0 
 0 
 0 
 0 
 0 
 0 
 0 
 0 
 0 
 0 
 0 
 0 
 0 
 0 
 0 
 0 
 0 
 0 
 0 
 0 
 0 
 0 
 0 
 0 
 0 
 0 
 0 
 0 
 0 
 0 
 0 
 0 
 0 
 0 
 0 
 0 
 0 
 0 
 0 
 0 
 0 
 0 
 0 
 0 
 0 
 0 
 0 
 0 
 0 
 0 
 0 
 0 
 0 
 0 
 0 
 0 
 0 
 0 
 0 
 0 
 0 
 0 
 0 
 0 
 0 
 0 
 0 
 0 
 0 
 0 
 0 
 0 
 0 
 0 
 0 
 0 
 0 
 0 
 0 
 0 
 0 
 0 
 0 
 0 
 0 
 0 
 0 
 0 
 0 
 0 
 0 
 0 
 0 
 0 
 0 
 0 
 0 
 0 
 0 
 0 
 0 
 0 
 0 
 0 
 0 
 0 
 0 
 0 
 0 
 0 
 0 
 0 
 0 
 0 
 0 
 0 
 0 
 0 
 0 
 0 
 0 
 0 
 0 
 0 
 0 
 0 
 0 
 0 
 0 
 0 
 0 
 0 
 0 
 0 
 0 
 0 
 0 
 0 
 0 
 0 
 0 
 0 
 0 
 0 
 0 
 0 
 0 
 0 
 0 
 0 
 0 
 0 
 0 
 0 
 0 
 0 
 0 
 0 
 0 
 0 
 0 
 0 
 0 
 0 
 0 
 0 
 0 
 0 
 0 
 0 
 0 
 0 
 0 
 0 
 0 
 0 
 0 
 0 
 0 
 0 
 0 
 0 
 0 
 0 
 0 
 0 
 0 
 0 
 0 
 0 
 0 
 0 
 0 
 0 
 0 
 0 
 0 
 0 
 0 
 0 
 0 
 0 
 0 
 0 
 0 
 0 
 0 
 0 
 0 
 0 
 0 
 0 
 0 
 0 
 0 
 0 
 0 
 0 
 0 
 0 
 0 
 0 
 0 
 0 
 0 
 0 
 0 
 0 
 0 
 0 
 0 
 0 
 0 
 0 
 0 
 0 
 0 
 0 
 0 
 0 
 0 
 0 
 0 
 0 
 0 
 0 
 0 
 0 
 0 
 0 
 0 
 0 
 0 
 0 
 0 
 0 
 0 
 0 
 0 
 0 
 0 
 0 
 0 
 0 
 0 
 0 
 0 
 0 
 0 
 0 
 0 
 0 
 0 
 0 
 0 
 0 
 0 
 0 
 0 
 0 
 0 
 0 
 0 
 0 
 0 
 0 
 0 
 0 
 0 
 0 
 0 
 0 
 0 
 0 
 0 
 0 
 0 
 0 
 0 
 0 
 0 
 0 
 0 
 0 
 0 
 0 
 0 
 0 
 0 
 0 
 0 
 0 
 0 
 0 
 0 
 0 
 0 
 0 
 0 
 0 
 0 
 0 
 0 
 0 
 0 
 0 
 0 
 0 
 0 
 0 
 0 
 0 
 0 
 0 
 0 
 0 
 0 
 0 
 0 
 0 
 0 
 0 
 0 
 0 
 0 
 0 
 0 
 0 
 0 
 0 
 0 
 0 
 0 
 0 
 0 
 0 
 0 
 0 
 0 
 0 
 0 
 0 
 0 
 0 
 0 
 0 
 0 
 0 
 0 
 0 
 0 
 0 
 0 
 0 
 0 
 0 
 0 
 0 
 0 
 0 
 0 
 0 
 0 
 0 
 0 
 0 
 0 
 0 
 0 
 0 
 0 
 0 
 0 
 0 
 0 
 0 
 0 
 0 
 0 
 0 
 0 
 0 
 0 
 0 
 0 
 0 
 0 
 0 
 0 
 0 
 0 
 0 
 0 
 0 
 0 
 0 
 0 
 0 
 0 
 0 
 0 
 0 
 0 
 0 
 0 
 0 
 0 
 0 
 0 
 0 
 0 
 0 
 0 
 0 
 0 
 0 
 0 
 0 
 0 
 0 
 0 
 0 
 0 
 0 
 0 
 0 
 0 
 0 
 0 
 0 
 0 
 0 
 0 
 0 
 0 
 0 
 0 
 0 
 0 
 0 
 0 
 0 
 0 
 0 
 0 
 0 
 0 
 0 
 0 
 0 
 0 
 0 
 0 
 0 
 0 
 0 
 0 
 0 
 0 
 0 
 0 
 0 
 0 
 0 
 0 
 0 
 0 
 0 
 1 
 0 
 0 
 0 
 0 
 0 
 0 
 0 
 0 
 0 
 0 
 0 
 0 
 0 
 0 
 0 
 0 
 0 
 0 
 0 
 0 
 0 
 0 
 0 
 0 
 0 
 0 
 0 
 0 
 0 
 0 
 0 
 0 
 0 
 0 
 0 
 0 
 0 
 0 
 0 
 0 
 0 
 0 
 0 
 0 
 0 
 0 
 0 
 0 
 0 
 0 
 0 
 0 
 0 
 0 
 0 
 0 
 0 
 0 
 0 
 0 
 0 
 0 
 0 
 0 
 0 
 0 
 0 
 0 
 0 
 0 
 0 
 0 
 0 
 0 
 0 
 0 
 0 
 0 
 0 
 0 
 0 
 0 
 0 
 0 
 0 
 0 
 0 
 0 
 0 
 0 
 0 
 0 
 0 
 0 
 0 
 0 
 0 
 0 
 0 
 0 
 0 
 0 
 0 
 0 
 0 
 0 
 0 
 0 
 0 
 0 
 0 
 0 
 0 
 0 
 0 
 0 
 0 
 0 
 0 
 0 
 0 
 0 
 0 
 0 
 0 
 0 
 0 
 0 
 0 
 0 
 0 
 0 
 0 
 0 
 0 
 0 
 0 
 0 
 0 
 0 
 0 
 0 
 0 
 0 
 0 
 0 
 0 
 0 
 0 
 0 
 0 
 0 
 0 
 0 
 0 
 0 
 0 
 0 
 0 
 0 
 0 
 0 
 0 
 0 
 0 
 0 
 0 
 0 
 0 
 0 
 0 
 0 
 0 
 0 
 0 
 0 
 0 
 0 
 0 
 0 
 0 
 0 
 0 
 0 
 0 
 0 
 0 
 0 
 0 
 0 
 0 
 0 
 0 
 0 
 0 
 0 
 0 
 0 
 0 
 0 
 0 
 0 
 0 
 0 
 0 
 0 
 0 
 0 
 0 
 0 
 0 
 0 
 0 
 0 
 0 
 0 
 0 
 0 
 0 
 0 
 0 
 0 
 0 
 0 
 0 
 0 
 0 
 0 
 0 
 0 
 0 
 0 
 0 
 0 
 0 
 0 
 0 
 0 
 0 
 0 
 0 
 0 
 0 
 0 
 0 
 0 
 0 
 0 
 0 
 0 
 0 
 0 
 0 
 0 
 0 
 0 
 0 
 0 
 0 
 0 
 0 
 0 
 
 
 75 
 0 
 0 
 0 
 0 
 0 
 0 
 0 
 0 
 0 
 0 
 0 
 0 
 0 
 0 
 0 
 0 
 0 
 0 
 0 
 0 
 0 
 0 
 0 
 0 
 0 
 0 
 0 
 0 
 0 
 0 
 0 
 0 
 0 
 0 
 0 
 0 
 0 
 0 
 0 
 0 
 0 
 0 
 0 
 0 
 0 
 0 
 0 
 0 
 0 
 0 
 0 
 0 
 0 
 0 
 0 
 0 
 0 
 0 
 0 
 0 
 0 
 0 
 0 
 0 
 0 
 0 
 0 
 0 
 0 
 0 
 0 
 0 
 0 
 0 
 0 
 5 
 0 
 0 
 0 
 0 
 2 
 0 
 10 
 0 
 0 
 0 
 0 
 0 
 0 
 0 
 0 
 0 
 0 
 0 
 0 
 0 
 0 
 0 
 0 
 0 
 0 
 0 
 0 
 0 
 0 
 0 
 0 
 0 
 0 
 0 
 0 
 0 
 0 
 0 
 0 
 0 
 0 
 0 
 0 
 0 
 0 
 0 
 0 
 0 
 0 
 0 
 0 
 0 
 0 
 0 
 0 
 0 
 0 
 0 
 0 
 0 
 0 
 0 
 0 
 0 
 0 
 0 
 0 
 0 
 0 
 0 
 0 
 0 
 0 
 0 
 0 
 0 
 0 
 0 
 0 
 0 
 0 
 0 
 0 
 0 
 0 
 0 
 0 
 0 
 0 
 0 
 0 
 0 
 0 
 0 
 0 
 0 
 0 
 0 
 0 
 0 
 0 
 0 
 0 
 0 
 0 
 0 
 0 
 0 
 0 
 0 
 0 
 0 
 0 
 0 
 0 
 0 
 0 
 0 
 0 
 0 
 0 
 0 
 0 
 0 
 0 
 0 
 0 
 0 
 0 
 0 
 0 
 0 
 0 
 0 
 0 
 0 
 0 
 0 
 0 
 0 
 0 
 0 
 0 
 0 
 0 
 0 
 0 
 0 
 0 
 0 
 0 
 0 
 0 
 0 
 0 
 0 
 0 
 0 
 0 
 0 
 0 
 0 
 0 
 0 
 0 
 0 
 0 
 0 
 0 
 0 
 0 
 0 
 0 
 0 
 0 
 0 
 0 
 0 
 0 
 0 
 0 
 0 
 0 
 0 
 0 
 0 
 0 
 0 
 0 
 0 
 0 
 0 
 0 
 0 
 0 
 0 
 0 
 0 
 0 
 0 
 0 
 0 
 0 
 0 
 0 
 0 
 0 
 0 
 0 
 0 
 0 
 0 
 0 
 0 
 0 
 0 
 0 
 0 
 0 
 0 
 0 
 0 
 0 
 0 
 0 
 0 
 0 
 0 
 0 
 0 
 0 
 0 
 0 
 0 
 0 
 0 
 0 
 0 
 0 
 0 
 0 
 0 
 0 
 0 
 0 
 0 
 0 
 0 
 0 
 0 
 0 
 0 
 0 
 0 
 0 
 0 
 0 
 0 
 0 
 0 
 0 
 0 
 0 
 0 
 0 
 0 
 0 
 0 
 0 
 0 
 0 
 0 
 0 
 0 
 0 
 0 
 0 
 0 
 0 
 0 
 0 
 0 
 0 
 0 
 0 
 0 
 0 
 0 
 0 
 0 
 0 
 0 
 0 
 0 
 0 
 0 
 0 
 0 
 0 
 0 
 0 
 0 
 0 
 0 
 0 
 0 
 0 
 0 
 0 
 0 
 0 
 0 
 0 
 0 
 0 
 0 
 0 
 0 
 0 
 0 
 0 
 0 
 0 
 0 
 0 
 0 
 0 
 0 
 0 
 0 
 0 
 0 
 0 
 0 
 0 
 0 
 0 
 0 
 0 
 0 
 0 
 0 
 0 
 0 
 0 
 0 
 0 
 0 
 0 
 0 
 0 
 0 
 0 
 0 
 0 
 0 
 0 
 0 
 0 
 0 
 0 
 0 
 0 
 0 
 0 
 0 
 0 
 0 
 0 
 0 
 0 
 0 
 0 
 0 
 0 
 0 
 0 
 0 
 0 
 0 
 0 
 0 
 0 
 0 
 0 
 0 
 0 
 0 
 0 
 0 
 0 
 0 
 0 
 0 
 0 
 0 
 0 
 0 
 0 
 0 
 0 
 0 
 0 
 0 
 0 
 0 
 0 
 0 
 0 
 0 
 0 
 0 
 0 
 0 
 0 
 0 
 0 
 0 
 0 
 0 
 0 
 0 
 0 
 0 
 0 
 0 
 0 
 0 
 0 
 0 
 0 
 0 
 0 
 0 
 0 
 0 
 0 
 0 
 0 
 0 
 0 
 0 
 0 
 0 
 0 
 0 
 0 
 0 
 0 
 0 
 0 
 0 
 0 
 0 
 0 
 0 
 0 
 0 
 0 
 0 
 0 
 0 
 0 
 0 
 0 
 0 
 0 
 0 
 0 
 0 
 0 
 0 
 0 
 0 
 0 
 0 
 0 
 0 
 0 
 0 
 0 
 0 
 0 
 0 
 0 
 0 
 0 
 0 
 0 
 0 
 0 
 0 
 0 
 0 
 0 
 0 
 0 
 0 
 0 
 0 
 0 
 0 
 0 
 0 
 0 
 0 
 0 
 0 
 0 
 0 
 0 
 0 
 0 
 0 
 0 
 0 
 0 
 0 
 0 
 0 
 0 
 0 
 0 
 0 
 0 
 0 
 0 
 0 
 0 
 0 
 0 
 0 
 0 
 0 
 0 
 0 
 0 
 0 
 0 
 0 
 0 
 0 
 0 
 0 
 0 
 0 
 0 
 0 
 0 
 0 
 0 
 0 
 0 
 0 
 0 
 0 
 0 
 0 
 0 
 0 
 0 
 0 
 0 
 0 
 0 
 0 
 0 
 0 
 0 
 0 
 0 
 0 
 0 
 0 
 0 
 0 
 0 
 0 
 0 
 0 
 0 
 0 
 0 
 0 
 0 
 0 
 0 
 0 
 0 
 0 
 0 
 0 
 0 
 0 
 0 
 0 
 0 
 0 
 0 
 0 
 0 
 0 
 0 
 0 
 0 
 0 
 0 
 0 
 0 
 0 
 0 
 0 
 0 
 0 
 0 
 0 
 0 
 0 
 0 
 0 
 0 
 0 
 0 
 0 
 0 
 0 
 0 
 0 
 0 
 0 
 0 
 0 
 0 
 0 
 0 
 0 
 0 
 0 
 0 
 0 
 0 
 0 
 0 
 0 
 0 
 0 
 0 
 0 
 0 
 0 
 0 
 0 
 0 
 0 
 0 
 0 
 0 
 0 
 0 
 0 
 0 
 0 
 0 
 0 
 0 
 0 
 0 
 0 
 0 
 0 
 0 
 0 
 0 
 0 
 0 
 0 
 0 
 0 
 0 
 0 
 0 
 0 
 0 
 0 
 0 
 0 
 0 
 0 
 0 
 0 
 0 
 0 
 0 
 0 
 0 
 0 
 0 
 0 
 0 
 0 
 0 
 0 
 0 
 0 
 0 
 0 
 0 
 0 
 0 
 0 
 0 
 0 
 0 
 0 
 0 
 0 
 0 
 0 
 0 
 0 
 0 
 0 
 0 
 0 
 0 
 0 
 0 
 0 
 0 
 0 
 0 
 0 
 0 
 0 
 0 
 0 
 0 
 0 
 0 
 0 
 0 
 0 
 0 
 0 
 0 
 0 
 0 
 0 
 0 
 0 
 
 
 76 
 0 
 0 
 0 
 0 
 0 
 0 
 0 
 0 
 0 
 0 
 0 
 0 
 0 
 0 
 0 
 0 
 0 
 0 
 0 
 0 
 0 
 0 
 0 
 0 
 0 
 0 
 0 
 0 
 0 
 0 
 0 
 0 
 0 
 0 
 0 
 0 
 0 
 0 
 0 
 0 
 0 
 0 
 0 
 0 
 0 
 0 
 0 
 0 
 0 
 0 
 0 
 0 
 0 
 0 
 0 
 0 
 0 
 0 
 0 
 0 
 0 
 0 
 0 
 0 
 0 
 0 
 0 
 0 
 0 
 0 
 0 
 0 
 0 
 30 
 0 
 0 
 8 
 0 
 0 
 0 
 0 
 0 
 0 
 0 
 0 
 0 
 0 
 0 
 0 
 0 
 0 
 0 
 0 
 0 
 0 
 0 
 0 
 0 
 0 
 0 
 0 
 0 
 0 
 0 
 0 
 0 
 0 
 0 
 0 
 0 
 0 
 0 
 0 
 0 
 0 
 0 
 0 
 0 
 0 
 0 
 0 
 0 
 0 
 0 
 0 
 0 
 0 
 0 
 0 
 0 
 0 
 0 
 0 
 0 
 0 
 0 
 0 
 0 
 0 
 0 
 0 
 0 
 0 
 0 
 0 
 0 
 0 
 0 
 0 
 0 
 0 
 0 
 0 
 0 
 0 
 0 
 0 
 0 
 0 
 0 
 0 
 0 
 0 
 0 
 0 
 0 
 0 
 0 
 0 
 0 
 0 
 0 
 0 
 0 
 0 
 0 
 0 
 0 
 0 
 0 
 0 
 0 
 0 
 0 
 0 
 0 
 0 
 0 
 0 
 0 
 0 
 0 
 0 
 0 
 0 
 0 
 0 
 0 
 0 
 0 
 0 
 0 
 0 
 0 
 0 
 0 
 0 
 0 
 0 
 0 
 0 
 0 
 0 
 0 
 0 
 0 
 0 
 0 
 0 
 0 
 0 
 0 
 0 
 0 
 0 
 0 
 0 
 0 
 0 
 0 
 0 
 0 
 0 
 0 
 0 
 0 
 0 
 0 
 0 
 0 
 0 
 0 
 0 
 0 
 0 
 0 
 0 
 0 
 0 
 0 
 0 
 0 
 0 
 0 
 0 
 0 
 0 
 0 
 0 
 0 
 0 
 0 
 0 
 0 
 0 
 0 
 0 
 0 
 0 
 0 
 0 
 0 
 0 
 0 
 0 
 0 
 0 
 0 
 0 
 0 
 0 
 0 
 0 
 0 
 0 
 0 
 0 
 0 
 0 
 0 
 0 
 0 
 0 
 0 
 0 
 0 
 0 
 0 
 0 
 0 
 0 
 0 
 0 
 0 
 0 
 0 
 0 
 0 
 0 
 0 
 0 
 0 
 0 
 0 
 0 
 0 
 0 
 0 
 0 
 0 
 0 
 0 
 0 
 0 
 0 
 0 
 0 
 0 
 0 
 0 
 0 
 0 
 0 
 0 
 0 
 0 
 0 
 0 
 0 
 0 
 0 
 0 
 0 
 0 
 0 
 0 
 0 
 0 
 0 
 0 
 0 
 0 
 0 
 0 
 0 
 0 
 0 
 0 
 0 
 0 
 0 
 0 
 0 
 0 
 0 
 0 
 0 
 0 
 0 
 0 
 0 
 0 
 0 
 0 
 0 
 0 
 0 
 0 
 0 
 0 
 0 
 0 
 0 
 0 
 0 
 0 
 0 
 0 
 0 
 0 
 0 
 0 
 0 
 0 
 0 
 0 
 0 
 0 
 0 
 0 
 0 
 0 
 0 
 0 
 0 
 0 
 0 
 0 
 0 
 0 
 0 
 0 
 0 
 0 
 0 
 0 
 0 
 0 
 0 
 0 
 0 
 0 
 0 
 0 
 0 
 0 
 0 
 0 
 0 
 0 
 0 
 0 
 0 
 0 
 0 
 0 
 0 
 0 
 0 
 0 
 0 
 0 
 0 
 0 
 0 
 0 
 0 
 0 
 0 
 0 
 0 
 0 
 0 
 0 
 0 
 0 
 0 
 0 
 0 
 0 
 0 
 0 
 0 
 0 
 0 
 0 
 0 
 0 
 0 
 0 
 0 
 0 
 0 
 0 
 0 
 0 
 0 
 0 
 0 
 0 
 0 
 0 
 0 
 0 
 0 
 0 
 0 
 0 
 0 
 0 
 0 
 0 
 0 
 0 
 0 
 0 
 0 
 0 
 0 
 0 
 0 
 0 
 0 
 0 
 0 
 0 
 0 
 0 
 0 
 0 
 0 
 0 
 0 
 0 
 0 
 0 
 0 
 0 
 0 
 0 
 0 
 0 
 0 
 0 
 0 
 0 
 0 
 0 
 0 
 0 
 0 
 0 
 0 
 0 
 0 
 0 
 0 
 0 
 0 
 0 
 0 
 0 
 0 
 0 
 0 
 0 
 0 
 0 
 0 
 0 
 0 
 0 
 0 
 0 
 0 
 0 
 0 
 0 
 0 
 0 
 0 
 0 
 0 
 0 
 0 
 0 
 0 
 0 
 0 
 0 
 0 
 0 
 0 
 0 
 0 
 0 
 0 
 0 
 0 
 0 
 0 
 0 
 0 
 0 
 0 
 0 
 0 
 0 
 0 
 0 
 0 
 0 
 0 
 0 
 0 
 0 
 0 
 0 
 0 
 0 
 0 
 0 
 0 
 0 
 0 
 0 
 0 
 0 
 0 
 0 
 0 
 0 
 0 
 0 
 0 
 0 
 0 
 0 
 0 
 0 
 0 
 0 
 0 
 0 
 0 
 0 
 0 
 0 
 0 
 0 
 0 
 0 
 0 
 0 
 0 
 0 
 0 
 0 
 0 
 0 
 0 
 0 
 0 
 0 
 0 
 0 
 0 
 0 
 0 
 0 
 0 
 0 
 0 
 0 
 0 
 0 
 0 
 0 
 0 
 0 
 0 
 0 
 0 
 0 
 0 
 0 
 0 
 0 
 0 
 0 
 0 
 0 
 0 
 0 
 0 
 0 
 0 
 0 
 0 
 0 
 0 
 0 
 0 
 0 
 0 
 0 
 0 
 0 
 0 
 0 
 0 
 0 
 0 
 0 
 0 
 0 
 0 
 0 
 0 
 0 
 0 
 0 
 0 
 0 
 0 
 0 
 0 
 0 
 0 
 0 
 0 
 0 
 0 
 0 
 0 
 0 
 0 
 0 
 0 
 0 
 0 
 0 
 0 
 0 
 0 
 0 
 0 
 0 
 0 
 0 
 0 
 0 
 0 
 0 
 0 
 0 
 0 
 0 
 0 
 0 
 0 
 0 
 0 
 0 
 0 
 0 
 0 
 0 
 0 
 0 
 0 
 0 
 0 
 0 
 0 
 0 
 0 
 0 
 0 
 0 
 0 
 0 
 0 
 0 
 0 
 0 
 0 
 0 
 0 
 0 
 0 
 0 
 0 
 0 
 0 
 0 
 0 
 0 
 0 
 0 
 0 
 0 
 0 
 0 
 0 
 0 
 0 
 0 
 0 
 0 
 0 
 0 
 0 
 0 
 0 
 0 
 0 
 0 
 0 
 0 
 0 
 0 
 0 
 0 
 0 
 0 
 0 
 0 
 0 
 0 
 0 
 0 
 0 
 0 
 0 
 0 
 0 
 0 
 0 
 0 
 0 
 0 
 0 
 0 
 0 
 0 
 
 
 77 
 0 
 0 
 0 
 0 
 0 
 0 
 0 
 0 
 3 
 0 
 0 
 0 
 0 
 0 
 0 
 0 
 0 
 0 
 0 
 0 
 0 
 0 
 0 
 0 
 0 
 0 
 0 
 0 
 0 
 0 
 0 
 0 
 0 
 0 
 0 
 0 
 0 
 0 
 0 
 0 
 0 
 0 
 0 
 0 
 0 
 0 
 0 
 0 
 0 
 0 
 0 
 0 
 0 
 0 
 0 
 0 
 0 
 0 
 0 
 0 
 0 
 0 
 0 
 0 
 0 
 0 
 0 
 0 
 0 
 0 
 0 
 0 
 0 
 0 
 0 
 0 
 0 
 5 
 0 
 0 
 0 
 0 
 0 
 0 
 0 
 0 
 0 
 0 
 0 
 0 
 0 
 0 
 0 
 0 
 0 
 0 
 0 
 0 
 0 
 0 
 0 
 0 
 0 
 0 
 0 
 0 
 0 
 0 
 0 
 0 
 0 
 0 
 0 
 0 
 0 
 0 
 0 
 0 
 0 
 0 
 0 
 0 
 0 
 0 
 0 
 0 
 0 
 0 
 0 
 0 
 0 
 0 
 0 
 0 
 0 
 0 
 0 
 0 
 0 
 0 
 0 
 0 
 0 
 0 
 0 
 0 
 0 
 0 
 0 
 0 
 0 
 0 
 0 
 0 
 0 
 0 
 0 
 0 
 0 
 0 
 0 
 0 
 0 
 0 
 0 
 0 
 0 
 0 
 0 
 0 
 0 
 0 
 0 
 0 
 0 
 0 
 0 
 0 
 0 
 0 
 0 
 0 
 0 
 0 
 0 
 0 
 0 
 0 
 0 
 0 
 0 
 0 
 0 
 0 
 0 
 0 
 0 
 0 
 0 
 0 
 0 
 0 
 0 
 0 
 0 
 0 
 0 
 0 
 0 
 0 
 0 
 0 
 0 
 0 
 0 
 0 
 0 
 0 
 0 
 0 
 0 
 0 
 0 
 0 
 0 
 0 
 0 
 0 
 0 
 0 
 0 
 0 
 0 
 0 
 0 
 0 
 0 
 0 
 0 
 0 
 0 
 0 
 0 
 0 
 0 
 0 
 0 
 0 
 0 
 0 
 0 
 0 
 0 
 0 
 0 
 0 
 0 
 0 
 0 
 0 
 0 
 0 
 0 
 0 
 0 
 0 
 0 
 0 
 0 
 0 
 0 
 0 
 0 
 0 
 0 
 0 
 0 
 0 
 0 
 0 
 0 
 0 
 0 
 0 
 0 
 0 
 0 
 0 
 0 
 0 
 0 
 0 
 0 
 0 
 0 
 0 
 0 
 0 
 0 
 0 
 0 
 0 
 0 
 0 
 0 
 0 
 0 
 0 
 0 
 0 
 0 
 0 
 0 
 0 
 0 
 0 
 0 
 0 
 0 
 0 
 0 
 0 
 0 
 0 
 0 
 0 
 0 
 0 
 0 
 0 
 0 
 0 
 0 
 0 
 0 
 0 
 0 
 0 
 0 
 0 
 0 
 0 
 0 
 0 
 0 
 0 
 0 
 0 
 0 
 0 
 0 
 0 
 0 
 0 
 0 
 0 
 0 
 0 
 0 
 0 
 0 
 0 
 0 
 0 
 0 
 0 
 0 
 0 
 0 
 0 
 0 
 0 
 0 
 0 
 0 
 0 
 0 
 0 
 0 
 0 
 0 
 0 
 0 
 0 
 0 
 0 
 0 
 0 
 0 
 0 
 0 
 0 
 0 
 0 
 0 
 0 
 0 
 0 
 0 
 0 
 0 
 0 
 0 
 0 
 0 
 0 
 0 
 0 
 0 
 0 
 0 
 0 
 0 
 0 
 0 
 0 
 0 
 0 
 0 
 0 
 0 
 0 
 0 
 0 
 0 
 0 
 0 
 0 
 0 
 0 
 0 
 0 
 0 
 0 
 0 
 0 
 0 
 0 
 0 
 0 
 0 
 0 
 0 
 0 
 0 
 0 
 0 
 0 
 0 
 0 
 0 
 0 
 0 
 0 
 0 
 0 
 0 
 0 
 0 
 0 
 0 
 0 
 0 
 0 
 0 
 0 
 0 
 0 
 0 
 0 
 0 
 0 
 0 
 0 
 0 
 0 
 0 
 0 
 0 
 0 
 0 
 0 
 0 
 0 
 0 
 0 
 0 
 0 
 0 
 0 
 0 
 0 
 0 
 0 
 0 
 0 
 4 
 0 
 0 
 0 
 0 
 0 
 0 
 0 
 0 
 0 
 0 
 0 
 0 
 0 
 0 
 0 
 0 
 0 
 0 
 0 
 0 
 0 
 0 
 0 
 0 
 0 
 0 
 0 
 0 
 0 
 0 
 0 
 0 
 0 
 0 
 0 
 0 
 0 
 0 
 0 
 0 
 0 
 0 
 0 
 0 
 0 
 0 
 0 
 0 
 0 
 0 
 0 
 0 
 0 
 0 
 0 
 0 
 0 
 0 
 0 
 0 
 0 
 0 
 0 
 0 
 0 
 0 
 0 
 0 
 0 
 0 
 0 
 0 
 0 
 0 
 0 
 0 
 0 
 0 
 0 
 0 
 0 
 0 
 0 
 0 
 0 
 0 
 0 
 0 
 0 
 0 
 0 
 0 
 0 
 0 
 0 
 0 
 0 
 0 
 0 
 0 
 0 
 0 
 0 
 0 
 0 
 0 
 0 
 0 
 0 
 0 
 0 
 0 
 0 
 0 
 0 
 0 
 0 
 0 
 0 
 0 
 0 
 0 
 0 
 0 
 0 
 0 
 0 
 0 
 0 
 0 
 0 
 0 
 0 
 0 
 0 
 0 
 0 
 0 
 0 
 0 
 0 
 0 
 0 
 0 
 0 
 0 
 0 
 0 
 0 
 0 
 0 
 0 
 0 
 0 
 0 
 0 
 0 
 0 
 0 
 0 
 0 
 0 
 0 
 0 
 0 
 0 
 0 
 0 
 0 
 0 
 0 
 0 
 0 
 0 
 0 
 0 
 0 
 0 
 0 
 0 
 0 
 0 
 0 
 0 
 0 
 0 
 0 
 0 
 0 
 0 
 0 
 0 
 0 
 0 
 0 
 0 
 0 
 0 
 0 
 0 
 0 
 0 
 0 
 0 
 0 
 0 
 0 
 0 
 0 
 0 
 0 
 0 
 0 
 0 
 0 
 0 
 0 
 0 
 0 
 0 
 0 
 0 
 0 
 0 
 0 
 0 
 0 
 0 
 0 
 0 
 0 
 0 
 0 
 0 
 0 
 0 
 0 
 0 
 0 
 0 
 0 
 0 
 0 
 0 
 0 
 0 
 0 
 0 
 0 
 0 
 0 
 0 
 0 
 0 
 0 
 0 
 0 
 0 
 0 
 0 
 0 
 0 
 0 
 0 
 0 
 0 
 0 
 0 
 0 
 0 
 0 
 0 
 0 
 0 
 0 
 0 
 0 
 0 
 0 
 0 
 0 
 0 
 0 
 0 
 0 
 0 
 0 
 0 
 0 
 0 
 0 
 0 
 0 
 0 
 0 
 0 
 0 
 0 
 0 
 0 
 0 
 0 
 0 
 0 
 0 
 0 
 0 
 0 
 0 
 0 
 0 
 0 
 0 
 0 
 0 
 0 
 0 
 0 
 0 
 0 
 0 
 0 
 0 
 0 
 0 
 0 
 0 
 0 
 0 
 
 
 78 
 0 
 0 
 0 
 0 
 0 
 0 
 0 
 0 
 0 
 0 
 0 
 0 
 0 
 0 
 0 
 0 
 0 
 0 
 0 
 0 
 0 
 0 
 0 
 0 
 0 
 0 
 0 
 0 
 0 
 0 
 0 
 0 
 0 
 0 
 0 
 0 
 0 
 0 
 0 
 0 
 0 
 0 
 0 
 0 
 0 
 0 
 0 
 0 
 0 
 0 
 0 
 0 
 0 
 0 
 0 
 0 
 0 
 0 
 0 
 0 
 0 
 0 
 0 
 0 
 0 
 0 
 0 
 0 
 0 
 0 
 0 
 0 
 0 
 0 
 6 
 0 
 0 
 0 
 0 
 0 
 0 
 0 
 0 
 0 
 0 
 0 
 0 
 0 
 0 
 0 
 0 
 0 
 0 
 0 
 0 
 0 
 0 
 0 
 0 
 0 
 0 
 0 
 0 
 0 
 0 
 0 
 0 
 0 
 0 
 0 
 0 
 0 
 0 
 0 
 0 
 0 
 0 
 0 
 0 
 0 
 0 
 0 
 0 
 0 
 0 
 0 
 0 
 0 
 0 
 0 
 0 
 0 
 0 
 0 
 0 
 0 
 0 
 0 
 0 
 0 
 0 
 0 
 0 
 0 
 0 
 0 
 0 
 0 
 0 
 0 
 0 
 0 
 0 
 0 
 0 
 0 
 0 
 0 
 0 
 0 
 0 
 0 
 0 
 0 
 0 
 0 
 0 
 0 
 0 
 0 
 0 
 0 
 0 
 0 
 0 
 0 
 0 
 0 
 0 
 0 
 0 
 0 
 0 
 0 
 0 
 0 
 0 
 0 
 0 
 0 
 0 
 0 
 0 
 0 
 0 
 0 
 0 
 0 
 0 
 0 
 0 
 0 
 0 
 0 
 0 
 0 
 0 
 0 
 0 
 0 
 0 
 0 
 0 
 0 
 0 
 0 
 0 
 0 
 0 
 0 
 0 
 0 
 0 
 0 
 0 
 0 
 0 
 0 
 0 
 0 
 0 
 0 
 0 
 0 
 0 
 0 
 0 
 0 
 0 
 0 
 0 
 0 
 0 
 0 
 0 
 0 
 0 
 0 
 0 
 0 
 0 
 0 
 0 
 0 
 0 
 0 
 0 
 0 
 0 
 0 
 0 
 0 
 0 
 0 
 0 
 0 
 0 
 0 
 0 
 0 
 0 
 0 
 0 
 0 
 0 
 0 
 0 
 0 
 0 
 0 
 0 
 0 
 0 
 0 
 0 
 0 
 0 
 0 
 0 
 0 
 0 
 0 
 0 
 0 
 0 
 0 
 0 
 0 
 0 
 0 
 0 
 0 
 0 
 0 
 0 
 0 
 0 
 0 
 0 
 0 
 0 
 0 
 0 
 0 
 0 
 0 
 0 
 0 
 0 
 0 
 0 
 0 
 0 
 0 
 0 
 0 
 0 
 0 
 0 
 0 
 0 
 0 
 0 
 0 
 0 
 0 
 0 
 0 
 0 
 0 
 0 
 0 
 0 
 0 
 0 
 0 
 0 
 0 
 0 
 0 
 0 
 0 
 0 
 0 
 0 
 0 
 0 
 0 
 0 
 0 
 0 
 0 
 0 
 0 
 0 
 0 
 0 
 0 
 0 
 0 
 0 
 0 
 0 
 0 
 0 
 0 
 0 
 0 
 0 
 0 
 0 
 0 
 0 
 0 
 0 
 0 
 0 
 0 
 0 
 0 
 0 
 0 
 0 
 0 
 0 
 0 
 0 
 0 
 0 
 0 
 0 
 0 
 0 
 0 
 0 
 0 
 0 
 0 
 0 
 0 
 0 
 0 
 0 
 0 
 0 
 0 
 0 
 0 
 0 
 0 
 0 
 0 
 0 
 0 
 0 
 0 
 0 
 0 
 0 
 0 
 0 
 0 
 0 
 0 
 0 
 0 
 0 
 0 
 0 
 0 
 0 
 0 
 0 
 0 
 0 
 0 
 0 
 0 
 0 
 0 
 0 
 0 
 0 
 0 
 0 
 0 
 0 
 0 
 0 
 0 
 0 
 0 
 0 
 0 
 0 
 0 
 0 
 0 
 0 
 0 
 0 
 0 
 0 
 0 
 0 
 0 
 0 
 0 
 0 
 0 
 0 
 0 
 0 
 0 
 0 
 0 
 0 
 0 
 0 
 0 
 0 
 0 
 0 
 0 
 0 
 0 
 0 
 0 
 0 
 0 
 0 
 0 
 0 
 0 
 0 
 0 
 0 
 0 
 0 
 0 
 0 
 0 
 0 
 0 
 0 
 0 
 0 
 0 
 0 
 0 
 0 
 0 
 0 
 0 
 0 
 0 
 0 
 0 
 0 
 0 
 0 
 0 
 0 
 0 
 0 
 0 
 0 
 0 
 0 
 0 
 0 
 0 
 0 
 0 
 0 
 0 
 0 
 0 
 0 
 0 
 0 
 0 
 0 
 0 
 0 
 0 
 0 
 0 
 0 
 0 
 0 
 0 
 0 
 0 
 0 
 0 
 0 
 0 
 0 
 0 
 0 
 0 
 0 
 0 
 0 
 0 
 0 
 0 
 0 
 0 
 0 
 0 
 0 
 0 
 0 
 0 
 0 
 0 
 0 
 0 
 0 
 0 
 0 
 0 
 0 
 0 
 0 
 0 
 0 
 0 
 0 
 0 
 0 
 0 
 0 
 0 
 0 
 0 
 0 
 0 
 0 
 0 
 0 
 0 
 0 
 0 
 0 
 0 
 0 
 0 
 0 
 0 
 0 
 0 
 0 
 0 
 0 
 0 
 0 
 0 
 0 
 0 
 0 
 0 
 0 
 0 
 0 
 0 
 0 
 0 
 0 
 0 
 0 
 0 
 0 
 0 
 0 
 0 
 0 
 0 
 0 
 0 
 0 
 0 
 0 
 0 
 0 
 0 
 0 
 0 
 0 
 0 
 0 
 0 
 0 
 0 
 0 
 0 
 0 
 0 
 0 
 0 
 0 
 0 
 0 
 0 
 0 
 0 
 0 
 0 
 0 
 0 
 0 
 0 
 0 
 0 
 0 
 0 
 0 
 0 
 0 
 0 
 0 
 0 
 0 
 0 
 0 
 0 
 0 
 0 
 0 
 0 
 0 
 0 
 0 
 0 
 0 
 0 
 0 
 0 
 0 
 0 
 0 
 0 
 0 
 0 
 0 
 0 
 0 
 0 
 0 
 0 
 0 
 0 
 0 
 0 
 0 
 0 
 0 
 0 
 0 
 0 
 0 
 0 
 0 
 0 
 0 
 0 
 0 
 0 
 0 
 0 
 0 
 0 
 0 
 0 
 0 
 0 
 0 
 0 
 0 
 0 
 0 
 0 
 0 
 0 
 0 
 0 
 0 
 0 
 0 
 0 
 0 
 0 
 0 
 0 
 0 
 0 
 0 
 0 
 0 
 0 
 0 
 0 
 0 
 0 
 0 
 0 
 0 
 0 
 0 
 0 
 0 
 0 
 0 
 0 
 0 
 0 
 0 
 0 
 0 
 0 
 0 
 0 
 0 
 0 
 0 
 0 
 0 
 0 
 0 
 0 
 0 
 0 
 0 
 0 
 0 
 0 
 0 
 0 
 0 
 0 
 0 
 0 
 0 
 0 
 0 
 0 
 0 
 0 
 0 
 0 
 0 
 0 
 0 
 0 
 
 
 79 
 0 
 0 
 0 
 0 
 0 
 0 
 0 
 0 
 0 
 0 
 0 
 0 
 0 
 0 
 0 
 0 
 0 
 0 
 0 
 0 
 0 
 0 
 0 
 0 
 0 
 0 
 0 
 0 
 0 
 0 
 0 
 0 
 0 
 0 
 0 
 0 
 0 
 0 
 0 
 0 
 0 
 0 
 0 
 0 
 0 
 0 
 0 
 0 
 0 
 0 
 0 
 0 
 0 
 0 
 0 
 0 
 0 
 0 
 0 
 0 
 0 
 0 
 0 
 0 
 0 
 0 
 0 
 0 
 0 
 0 
 0 
 0 
 0 
 0 
 0 
 0 
 0 
 0 
 0 
 14 
 0 
 0 
 0 
 0 
 0 
 0 
 0 
 0 
 0 
 0 
 0 
 0 
 0 
 0 
 0 
 0 
 0 
 0 
 0 
 0 
 0 
 0 
 0 
 0 
 0 
 0 
 0 
 0 
 0 
 0 
 0 
 0 
 0 
 0 
 0 
 0 
 0 
 0 
 0 
 0 
 0 
 0 
 0 
 0 
 0 
 0 
 0 
 0 
 0 
 0 
 0 
 0 
 0 
 0 
 0 
 0 
 0 
 0 
 0 
 0 
 0 
 0 
 0 
 0 
 0 
 0 
 0 
 0 
 0 
 0 
 0 
 0 
 0 
 0 
 0 
 0 
 0 
 0 
 0 
 0 
 0 
 0 
 0 
 0 
 0 
 0 
 0 
 0 
 0 
 0 
 0 
 0 
 0 
 0 
 0 
 0 
 0 
 0 
 0 
 0 
 0 
 0 
 0 
 0 
 0 
 0 
 0 
 0 
 0 
 0 
 0 
 0 
 0 
 0 
 0 
 0 
 0 
 0 
 0 
 0 
 0 
 0 
 0 
 0 
 0 
 0 
 0 
 0 
 0 
 0 
 0 
 0 
 0 
 0 
 0 
 0 
 0 
 0 
 0 
 0 
 0 
 0 
 0 
 0 
 0 
 0 
 0 
 0 
 0 
 0 
 0 
 0 
 0 
 0 
 0 
 0 
 0 
 0 
 0 
 0 
 0 
 0 
 0 
 0 
 0 
 0 
 0 
 0 
 0 
 0 
 0 
 0 
 0 
 0 
 0 
 0 
 0 
 0 
 0 
 0 
 0 
 0 
 0 
 0 
 0 
 0 
 0 
 0 
 0 
 0 
 0 
 0 
 0 
 0 
 0 
 0 
 0 
 0 
 0 
 0 
 0 
 0 
 0 
 0 
 0 
 0 
 0 
 0 
 0 
 0 
 0 
 0 
 0 
 0 
 0 
 0 
 0 
 0 
 0 
 0 
 0 
 0 
 0 
 0 
 0 
 0 
 0 
 0 
 0 
 0 
 0 
 0 
 0 
 0 
 0 
 0 
 0 
 0 
 0 
 0 
 0 
 0 
 0 
 0 
 0 
 0 
 0 
 0 
 0 
 0 
 0 
 0 
 0 
 0 
 0 
 0 
 0 
 0 
 0 
 0 
 0 
 0 
 0 
 0 
 0 
 0 
 0 
 0 
 0 
 0 
 0 
 0 
 0 
 0 
 0 
 0 
 0 
 0 
 0 
 0 
 0 
 0 
 0 
 0 
 0 
 0 
 0 
 0 
 0 
 0 
 0 
 0 
 0 
 0 
 0 
 0 
 0 
 0 
 0 
 0 
 0 
 0 
 0 
 0 
 0 
 0 
 0 
 0 
 0 
 0 
 0 
 0 
 0 
 0 
 0 
 0 
 0 
 0 
 0 
 0 
 0 
 0 
 0 
 0 
 0 
 0 
 0 
 0 
 0 
 0 
 0 
 0 
 0 
 0 
 0 
 0 
 0 
 0 
 0 
 0 
 0 
 0 
 0 
 0 
 0 
 0 
 0 
 0 
 0 
 0 
 0 
 0 
 0 
 0 
 0 
 0 
 0 
 0 
 0 
 0 
 0 
 0 
 0 
 0 
 0 
 0 
 0 
 0 
 0 
 0 
 0 
 0 
 0 
 0 
 0 
 0 
 0 
 0 
 0 
 0 
 0 
 0 
 0 
 0 
 0 
 0 
 0 
 0 
 0 
 0 
 0 
 0 
 0 
 0 
 0 
 0 
 0 
 0 
 0 
 0 
 0 
 0 
 0 
 0 
 0 
 0 
 0 
 0 
 0 
 0 
 0 
 0 
 0 
 0 
 0 
 0 
 0 
 0 
 0 
 0 
 0 
 0 
 0 
 0 
 0 
 0 
 0 
 0 
 0 
 0 
 0 
 0 
 0 
 0 
 0 
 0 
 0 
 0 
 0 
 0 
 0 
 0 
 0 
 0 
 0 
 0 
 0 
 0 
 0 
 0 
 0 
 0 
 0 
 0 
 0 
 0 
 0 
 0 
 0 
 0 
 0 
 0 
 0 
 0 
 0 
 0 
 0 
 0 
 0 
 0 
 0 
 0 
 0 
 0 
 0 
 0 
 0 
 0 
 0 
 0 
 0 
 0 
 0 
 0 
 0 
 0 
 0 
 0 
 0 
 0 
 0 
 0 
 0 
 0 
 0 
 0 
 0 
 0 
 0 
 0 
 0 
 0 
 0 
 0 
 0 
 0 
 0 
 0 
 0 
 0 
 0 
 0 
 0 
 0 
 0 
 0 
 0 
 0 
 0 
 0 
 0 
 0 
 0 
 0 
 0 
 0 
 0 
 0 
 0 
 0 
 0 
 0 
 0 
 0 
 0 
 0 
 0 
 0 
 0 
 0 
 0 
 0 
 0 
 0 
 0 
 0 
 0 
 0 
 0 
 0 
 0 
 0 
 0 
 0 
 0 
 0 
 0 
 0 
 0 
 0 
 0 
 0 
 0 
 0 
 0 
 0 
 0 
 0 
 0 
 0 
 0 
 0 
 0 
 0 
 0 
 0 
 0 
 0 
 0 
 0 
 0 
 0 
 0 
 0 
 0 
 0 
 0 
 0 
 0 
 0 
 0 
 0 
 0 
 0 
 0 
 0 
 0 
 0 
 0 
 0 
 0 
 0 
 0 
 0 
 0 
 0 
 0 
 0 
 0 
 0 
 0 
 0 
 0 
 0 
 0 
 0 
 0 
 0 
 0 
 0 
 0 
 0 
 0 
 0 
 0 
 0 
 0 
 0 
 0 
 0 
 0 
 0 
 0 
 0 
 0 
 0 
 0 
 0 
 0 
 0 
 0 
 0 
 0 
 0 
 0 
 0 
 0 
 0 
 0 
 0 
 0 
 0 
 0 
 0 
 0 
 0 
 0 
 0 
 0 
 0 
 0 
 0 
 0 
 0 
 0 
 0 
 0 
 0 
 0 
 0 
 0 
 0 
 0 
 0 
 0 
 0 
 0 
 0 
 0 
 0 
 0 
 0 
 0 
 0 
 0 
 0 
 0 
 0 
 0 
 0 
 0 
 0 
 0 
 0 
 0 
 0 
 0 
 0 
 0 
 0 
 0 
 0 
 0 
 0 
 0 
 0 
 0 
 0 
 0 
 0 
 0 
 0 
 0 
 0 
 0 
 0 
 0 
 0 
 0 
 0 
 0 
 0 
 0 
 0 
 0 
 0 
 0 
 0 
 0 
 0 
 0 
 0 
 0 
 0 
 0 
 0 
 0 
 0 
 0 
 0 
 0 
 0 
 0 
 0 
 0 
 0 
 
 
 80 
 0 
 0 
 0 
 0 
 0 
 0 
 0 
 0 
 0 
 0 
 0 
 0 
 0 
 0 
 0 
 0 
 0 
 0 
 0 
 0 
 0 
 0 
 0 
 0 
 0 
 0 
 0 
 0 
 0 
 0 
 0 
 0 
 0 
 0 
 0 
 0 
 0 
 0 
 0 
 0 
 0 
 0 
 0 
 0 
 0 
 0 
 0 
 0 
 0 
 0 
 0 
 0 
 0 
 0 
 0 
 0 
 0 
 0 
 0 
 0 
 0 
 0 
 0 
 0 
 0 
 0 
 0 
 0 
 0 
 0 
 0 
 0 
 0 
 0 
 0 
 0 
 0 
 0 
 0 
 0 
 15 
 0 
 0 
 0 
 0 
 0 
 0 
 0 
 0 
 0 
 0 
 0 
 0 
 0 
 0 
 0 
 0 
 0 
 0 
 0 
 0 
 0 
 0 
 0 
 0 
 0 
 0 
 0 
 0 
 0 
 0 
 0 
 0 
 0 
 0 
 0 
 0 
 0 
 0 
 0 
 0 
 0 
 0 
 0 
 0 
 0 
 0 
 0 
 0 
 0 
 0 
 0 
 0 
 0 
 0 
 0 
 0 
 0 
 0 
 0 
 0 
 0 
 0 
 0 
 0 
 0 
 0 
 0 
 0 
 0 
 0 
 0 
 0 
 0 
 0 
 0 
 0 
 0 
 0 
 0 
 0 
 0 
 0 
 0 
 0 
 0 
 0 
 0 
 0 
 0 
 0 
 0 
 0 
 0 
 0 
 0 
 0 
 0 
 0 
 0 
 0 
 0 
 0 
 0 
 0 
 0 
 0 
 0 
 0 
 0 
 0 
 0 
 0 
 0 
 0 
 0 
 0 
 0 
 0 
 0 
 0 
 0 
 0 
 0 
 0 
 0 
 0 
 0 
 0 
 0 
 0 
 0 
 0 
 0 
 0 
 0 
 0 
 0 
 0 
 0 
 0 
 0 
 0 
 0 
 0 
 0 
 0 
 0 
 0 
 0 
 0 
 0 
 0 
 0 
 0 
 0 
 0 
 0 
 0 
 0 
 0 
 0 
 0 
 0 
 0 
 0 
 0 
 0 
 0 
 0 
 0 
 0 
 0 
 0 
 0 
 0 
 0 
 0 
 0 
 0 
 0 
 0 
 0 
 0 
 0 
 0 
 0 
 0 
 0 
 0 
 0 
 0 
 0 
 0 
 0 
 0 
 0 
 0 
 0 
 0 
 0 
 0 
 0 
 0 
 0 
 0 
 0 
 0 
 0 
 0 
 0 
 0 
 0 
 0 
 0 
 0 
 0 
 0 
 0 
 0 
 0 
 0 
 0 
 0 
 0 
 0 
 0 
 0 
 0 
 0 
 0 
 0 
 0 
 0 
 0 
 0 
 0 
 0 
 0 
 0 
 0 
 0 
 0 
 0 
 0 
 0 
 0 
 0 
 0 
 0 
 0 
 0 
 0 
 0 
 0 
 0 
 0 
 0 
 0 
 0 
 0 
 0 
 0 
 0 
 0 
 0 
 0 
 0 
 0 
 0 
 0 
 0 
 0 
 0 
 0 
 0 
 0 
 0 
 0 
 0 
 0 
 0 
 0 
 0 
 0 
 0 
 0 
 0 
 0 
 0 
 0 
 0 
 0 
 0 
 0 
 0 
 0 
 0 
 0 
 0 
 0 
 0 
 0 
 0 
 0 
 0 
 0 
 0 
 0 
 0 
 0 
 0 
 0 
 0 
 0 
 0 
 0 
 0 
 0 
 0 
 0 
 0 
 0 
 0 
 0 
 0 
 0 
 0 
 0 
 0 
 0 
 0 
 0 
 0 
 0 
 0 
 0 
 0 
 0 
 0 
 0 
 0 
 0 
 0 
 0 
 0 
 0 
 0 
 0 
 0 
 0 
 0 
 0 
 0 
 0 
 0 
 0 
 0 
 0 
 0 
 0 
 0 
 0 
 0 
 0 
 0 
 0 
 0 
 0 
 0 
 0 
 0 
 0 
 0 
 0 
 0 
 0 
 0 
 0 
 0 
 0 
 0 
 0 
 0 
 0 
 0 
 0 
 0 
 0 
 0 
 0 
 0 
 0 
 0 
 0 
 0 
 0 
 0 
 0 
 0 
 0 
 0 
 0 
 0 
 0 
 0 
 0 
 0 
 0 
 0 
 0 
 0 
 0 
 0 
 0 
 0 
 0 
 0 
 0 
 0 
 0 
 0 
 0 
 0 
 0 
 0 
 0 
 0 
 0 
 0 
 0 
 0 
 0 
 0 
 0 
 0 
 0 
 0 
 0 
 0 
 0 
 0 
 0 
 0 
 0 
 0 
 0 
 0 
 0 
 0 
 0 
 0 
 0 
 0 
 0 
 0 
 0 
 0 
 0 
 0 
 0 
 0 
 0 
 0 
 0 
 0 
 0 
 0 
 0 
 0 
 0 
 0 
 0 
 0 
 0 
 0 
 0 
 0 
 0 
 0 
 0 
 0 
 0 
 0 
 0 
 0 
 0 
 0 
 0 
 0 
 0 
 0 
 0 
 0 
 0 
 0 
 0 
 0 
 0 
 0 
 0 
 0 
 0 
 0 
 0 
 0 
 0 
 0 
 0 
 0 
 0 
 0 
 0 
 0 
 0 
 0 
 0 
 0 
 0 
 0 
 0 
 0 
 0 
 0 
 0 
 0 
 0 
 0 
 0 
 0 
 0 
 0 
 0 
 0 
 0 
 0 
 0 
 0 
 0 
 0 
 0 
 0 
 0 
 0 
 0 
 0 
 0 
 0 
 0 
 0 
 0 
 0 
 0 
 0 
 0 
 0 
 0 
 0 
 0 
 0 
 0 
 0 
 0 
 0 
 0 
 0 
 0 
 0 
 0 
 0 
 0 
 0 
 0 
 0 
 0 
 0 
 0 
 0 
 0 
 0 
 0 
 0 
 0 
 0 
 0 
 0 
 0 
 0 
 0 
 0 
 0 
 0 
 0 
 0 
 0 
 0 
 0 
 0 
 0 
 0 
 0 
 0 
 0 
 0 
 0 
 0 
 0 
 0 
 0 
 0 
 0 
 0 
 0 
 0 
 0 
 0 
 0 
 0 
 0 
 0 
 0 
 0 
 0 
 0 
 0 
 0 
 0 
 0 
 0 
 0 
 0 
 0 
 0 
 0 
 0 
 0 
 0 
 0 
 0 
 0 
 0 
 0 
 0 
 0 
 0 
 0 
 0 
 0 
 0 
 0 
 0 
 0 
 0 
 0 
 0 
 0 
 0 
 0 
 0 
 0 
 0 
 0 
 0 
 0 
 0 
 0 
 0 
 0 
 0 
 0 
 0 
 0 
 0 
 0 
 0 
 0 
 0 
 0 
 0 
 0 
 0 
 0 
 0 
 0 
 0 
 0 
 0 
 0 
 0 
 0 
 0 
 0 
 0 
 0 
 0 
 0 
 0 
 0 
 0 
 0 
 0 
 0 
 0 
 0 
 0 
 0 
 0 
 0 
 0 
 0 
 0 
 0 
 0 
 0 
 0 
 0 
 0 
 0 
 0 
 0 
 0 
 0 
 0 
 0 
 0 
 0 
 0 
 0 
 0 
 0 
 0 
 0 
 0 
 0 
 0 
 0 
 0 
 0 
 0 
 0 
 0 
 0 
 0 
 0 
 0 
 0 
 
 
 81 
 0 
 0 
 0 
 0 
 0 
 0 
 0 
 0 
 0 
 0 
 0 
 0 
 0 
 0 
 0 
 0 
 0 
 0 
 0 
 0 
 0 
 0 
 0 
 0 
 0 
 0 
 0 
 0 
 0 
 0 
 0 
 0 
 0 
 0 
 0 
 0 
 0 
 0 
 0 
 0 
 0 
 0 
 0 
 0 
 0 
 0 
 0 
 0 
 0 
 0 
 0 
 0 
 0 
 0 
 0 
 0 
 0 
 0 
 0 
 0 
 0 
 0 
 0 
 0 
 0 
 0 
 0 
 0 
 0 
 0 
 0 
 0 
 0 
 0 
 0 
 0 
 0 
 0 
 0 
 0 
 0 
 11 
 0 
 0 
 0 
 0 
 0 
 0 
 0 
 0 
 0 
 0 
 0 
 0 
 0 
 0 
 0 
 0 
 0 
 0 
 0 
 0 
 0 
 0 
 0 
 0 
 0 
 0 
 0 
 0 
 0 
 0 
 0 
 0 
 0 
 0 
 0 
 0 
 0 
 0 
 0 
 0 
 0 
 0 
 0 
 0 
 0 
 0 
 0 
 0 
 0 
 0 
 0 
 0 
 0 
 0 
 0 
 0 
 0 
 0 
 0 
 0 
 0 
 0 
 0 
 0 
 0 
 0 
 0 
 0 
 0 
 0 
 0 
 0 
 0 
 0 
 0 
 0 
 0 
 0 
 0 
 0 
 0 
 0 
 0 
 0 
 0 
 0 
 0 
 0 
 0 
 0 
 0 
 0 
 0 
 0 
 0 
 0 
 0 
 0 
 0 
 0 
 0 
 0 
 0 
 0 
 0 
 0 
 0 
 0 
 0 
 0 
 0 
 0 
 0 
 0 
 0 
 0 
 0 
 0 
 0 
 0 
 0 
 0 
 0 
 0 
 0 
 0 
 0 
 0 
 0 
 0 
 0 
 0 
 0 
 0 
 0 
 0 
 0 
 0 
 0 
 0 
 0 
 0 
 0 
 0 
 0 
 0 
 0 
 0 
 0 
 0 
 0 
 0 
 0 
 0 
 0 
 0 
 0 
 0 
 0 
 0 
 0 
 0 
 0 
 0 
 0 
 0 
 0 
 0 
 0 
 0 
 0 
 0 
 0 
 0 
 0 
 0 
 0 
 0 
 0 
 0 
 0 
 0 
 0 
 0 
 0 
 0 
 0 
 0 
 0 
 0 
 0 
 0 
 0 
 0 
 0 
 0 
 0 
 0 
 0 
 0 
 0 
 0 
 0 
 0 
 0 
 0 
 0 
 0 
 0 
 0 
 0 
 0 
 0 
 0 
 0 
 0 
 0 
 0 
 0 
 0 
 0 
 0 
 0 
 0 
 0 
 0 
 0 
 0 
 0 
 0 
 0 
 0 
 0 
 0 
 0 
 0 
 0 
 0 
 0 
 0 
 0 
 0 
 0 
 0 
 0 
 0 
 0 
 0 
 0 
 0 
 0 
 0 
 0 
 0 
 0 
 0 
 0 
 0 
 0 
 0 
 0 
 0 
 0 
 0 
 0 
 0 
 0 
 0 
 0 
 0 
 0 
 0 
 0 
 0 
 0 
 0 
 0 
 0 
 0 
 0 
 0 
 0 
 0 
 0 
 0 
 0 
 0 
 0 
 0 
 0 
 0 
 0 
 0 
 0 
 0 
 0 
 0 
 0 
 0 
 0 
 0 
 0 
 0 
 0 
 0 
 0 
 0 
 0 
 0 
 0 
 0 
 0 
 0 
 0 
 0 
 0 
 0 
 0 
 0 
 0 
 0 
 0 
 0 
 0 
 0 
 0 
 0 
 0 
 0 
 0 
 0 
 0 
 0 
 0 
 0 
 0 
 0 
 0 
 0 
 0 
 0 
 0 
 0 
 0 
 0 
 0 
 0 
 0 
 0 
 0 
 0 
 0 
 0 
 0 
 0 
 0 
 0 
 0 
 0 
 0 
 0 
 0 
 0 
 0 
 0 
 0 
 0 
 0 
 0 
 0 
 0 
 0 
 0 
 0 
 0 
 0 
 0 
 0 
 0 
 0 
 0 
 0 
 0 
 0 
 0 
 0 
 0 
 0 
 0 
 0 
 0 
 0 
 0 
 0 
 0 
 0 
 0 
 0 
 0 
 0 
 0 
 0 
 0 
 0 
 0 
 0 
 0 
 0 
 0 
 0 
 0 
 0 
 0 
 0 
 0 
 0 
 0 
 0 
 0 
 0 
 0 
 0 
 0 
 0 
 0 
 0 
 0 
 0 
 0 
 0 
 0 
 0 
 0 
 0 
 0 
 0 
 0 
 0 
 0 
 0 
 0 
 0 
 0 
 0 
 0 
 0 
 0 
 0 
 0 
 0 
 0 
 0 
 0 
 0 
 0 
 0 
 0 
 0 
 0 
 0 
 0 
 0 
 0 
 0 
 0 
 0 
 0 
 0 
 0 
 0 
 0 
 0 
 0 
 0 
 0 
 0 
 0 
 0 
 0 
 0 
 0 
 0 
 0 
 0 
 0 
 0 
 0 
 0 
 0 
 0 
 0 
 0 
 0 
 0 
 0 
 0 
 0 
 0 
 0 
 0 
 0 
 0 
 0 
 0 
 0 
 0 
 0 
 0 
 0 
 0 
 0 
 0 
 0 
 0 
 0 
 0 
 0 
 0 
 0 
 0 
 0 
 0 
 0 
 0 
 0 
 0 
 0 
 0 
 0 
 0 
 0 
 0 
 0 
 0 
 0 
 0 
 0 
 0 
 0 
 0 
 0 
 0 
 0 
 0 
 0 
 0 
 0 
 0 
 0 
 0 
 0 
 0 
 0 
 0 
 0 
 0 
 0 
 0 
 0 
 0 
 0 
 0 
 0 
 0 
 0 
 0 
 0 
 0 
 0 
 0 
 0 
 0 
 0 
 0 
 0 
 0 
 0 
 0 
 0 
 0 
 0 
 0 
 0 
 0 
 0 
 0 
 0 
 0 
 0 
 0 
 0 
 0 
 0 
 0 
 0 
 0 
 0 
 0 
 0 
 0 
 0 
 0 
 0 
 0 
 0 
 0 
 0 
 0 
 0 
 0 
 0 
 0 
 0 
 0 
 0 
 0 
 0 
 0 
 0 
 0 
 0 
 0 
 0 
 0 
 0 
 0 
 0 
 0 
 0 
 0 
 0 
 0 
 0 
 0 
 0 
 0 
 0 
 0 
 0 
 0 
 0 
 0 
 0 
 0 
 0 
 0 
 0 
 0 
 0 
 0 
 0 
 0 
 0 
 0 
 0 
 0 
 0 
 0 
 0 
 0 
 0 
 0 
 0 
 0 
 0 
 0 
 0 
 0 
 0 
 0 
 0 
 0 
 0 
 0 
 0 
 0 
 0 
 0 
 0 
 0 
 0 
 0 
 0 
 0 
 0 
 0 
 0 
 0 
 0 
 0 
 0 
 0 
 0 
 0 
 0 
 0 
 0 
 0 
 0 
 0 
 0 
 0 
 0 
 0 
 0 
 0 
 0 
 0 
 0 
 0 
 0 
 0 
 0 
 0 
 0 
 0 
 0 
 0 
 0 
 0 
 0 
 0 
 0 
 0 
 0 
 0 
 0 
 0 
 0 
 0 
 0 
 0 
 0 
 0 
 0 
 0 
 0 
 0 
 0 
 0 
 0 
 0 
 0 
 0 
 
 
 82 
 0 
 0 
 0 
 0 
 0 
 0 
 0 
 0 
 0 
 0 
 0 
 0 
 0 
 0 
 0 
 0 
 0 
 0 
 0 
 0 
 0 
 0 
 0 
 0 
 0 
 0 
 0 
 0 
 0 
 0 
 0 
 0 
 0 
 0 
 0 
 0 
 0 
 0 
 0 
 0 
 0 
 0 
 0 
 0 
 0 
 0 
 0 
 0 
 0 
 0 
 0 
 0 
 0 
 0 
 0 
 0 
 0 
 0 
 0 
 0 
 0 
 0 
 0 
 0 
 0 
 0 
 0 
 0 
 0 
 0 
 0 
 0 
 0 
 0 
 0 
 2 
 0 
 0 
 0 
 0 
 0 
 0 
 12 
 0 
 0 
 0 
 0 
 0 
 0 
 0 
 0 
 0 
 0 
 0 
 0 
 0 
 0 
 0 
 0 
 0 
 0 
 0 
 0 
 0 
 0 
 0 
 0 
 0 
 0 
 0 
 0 
 0 
 0 
 0 
 0 
 0 
 0 
 0 
 0 
 0 
 0 
 0 
 0 
 0 
 0 
 0 
 0 
 0 
 0 
 0 
 0 
 0 
 0 
 0 
 0 
 0 
 0 
 0 
 0 
 0 
 0 
 0 
 0 
 0 
 0 
 0 
 0 
 0 
 0 
 0 
 0 
 0 
 0 
 0 
 0 
 0 
 0 
 0 
 0 
 0 
 0 
 0 
 0 
 0 
 0 
 0 
 0 
 0 
 0 
 0 
 0 
 0 
 0 
 0 
 0 
 0 
 0 
 0 
 0 
 0 
 0 
 0 
 0 
 0 
 0 
 0 
 0 
 0 
 0 
 0 
 0 
 0 
 0 
 0 
 0 
 0 
 0 
 0 
 0 
 0 
 0 
 0 
 0 
 0 
 0 
 0 
 0 
 0 
 0 
 0 
 0 
 0 
 0 
 0 
 0 
 0 
 0 
 0 
 0 
 0 
 0 
 0 
 0 
 0 
 0 
 0 
 0 
 0 
 0 
 0 
 0 
 0 
 0 
 0 
 0 
 0 
 0 
 0 
 0 
 0 
 0 
 0 
 0 
 0 
 0 
 0 
 0 
 0 
 0 
 0 
 0 
 0 
 0 
 0 
 0 
 0 
 0 
 0 
 0 
 0 
 0 
 0 
 0 
 0 
 0 
 0 
 0 
 0 
 0 
 0 
 0 
 0 
 0 
 0 
 0 
 0 
 0 
 0 
 0 
 0 
 0 
 0 
 0 
 0 
 0 
 0 
 0 
 0 
 0 
 0 
 0 
 0 
 0 
 0 
 0 
 0 
 0 
 0 
 0 
 0 
 0 
 0 
 0 
 0 
 0 
 0 
 0 
 0 
 0 
 0 
 0 
 0 
 0 
 0 
 0 
 0 
 0 
 0 
 0 
 0 
 0 
 0 
 0 
 0 
 0 
 0 
 0 
 0 
 0 
 0 
 0 
 0 
 0 
 0 
 0 
 0 
 0 
 0 
 0 
 0 
 0 
 0 
 0 
 0 
 0 
 0 
 0 
 0 
 0 
 0 
 0 
 0 
 0 
 0 
 0 
 0 
 0 
 0 
 0 
 0 
 0 
 0 
 0 
 0 
 0 
 0 
 0 
 0 
 0 
 0 
 0 
 0 
 0 
 0 
 0 
 0 
 0 
 0 
 0 
 0 
 0 
 0 
 0 
 0 
 0 
 0 
 0 
 0 
 0 
 0 
 0 
 0 
 0 
 0 
 0 
 0 
 0 
 0 
 0 
 0 
 0 
 0 
 0 
 0 
 0 
 0 
 0 
 0 
 0 
 0 
 0 
 0 
 0 
 0 
 0 
 0 
 0 
 0 
 0 
 0 
 0 
 0 
 0 
 0 
 0 
 0 
 0 
 0 
 0 
 0 
 0 
 0 
 0 
 0 
 0 
 0 
 0 
 0 
 0 
 0 
 0 
 0 
 0 
 0 
 0 
 0 
 0 
 0 
 0 
 0 
 0 
 0 
 0 
 0 
 0 
 0 
 0 
 0 
 0 
 0 
 0 
 0 
 0 
 0 
 0 
 0 
 0 
 0 
 0 
 0 
 0 
 0 
 0 
 0 
 0 
 0 
 0 
 0 
 0 
 0 
 0 
 0 
 0 
 0 
 0 
 0 
 0 
 0 
 0 
 0 
 0 
 0 
 0 
 0 
 0 
 0 
 0 
 0 
 0 
 0 
 0 
 0 
 0 
 0 
 0 
 0 
 0 
 0 
 0 
 0 
 0 
 0 
 0 
 0 
 0 
 0 
 0 
 0 
 0 
 0 
 0 
 0 
 0 
 0 
 0 
 0 
 0 
 0 
 0 
 0 
 0 
 0 
 0 
 0 
 0 
 0 
 0 
 0 
 0 
 0 
 0 
 0 
 0 
 0 
 0 
 0 
 0 
 0 
 0 
 0 
 0 
 0 
 0 
 0 
 0 
 0 
 0 
 0 
 0 
 0 
 0 
 0 
 0 
 0 
 0 
 0 
 0 
 0 
 0 
 0 
 0 
 0 
 0 
 0 
 0 
 0 
 0 
 0 
 0 
 0 
 0 
 0 
 0 
 0 
 0 
 0 
 0 
 0 
 0 
 0 
 0 
 0 
 0 
 0 
 0 
 0 
 0 
 0 
 0 
 0 
 0 
 0 
 0 
 0 
 0 
 0 
 0 
 0 
 0 
 0 
 0 
 0 
 0 
 0 
 0 
 0 
 0 
 0 
 0 
 0 
 0 
 0 
 0 
 0 
 0 
 0 
 0 
 0 
 0 
 0 
 0 
 0 
 0 
 0 
 0 
 0 
 0 
 0 
 0 
 0 
 0 
 0 
 0 
 0 
 0 
 0 
 0 
 0 
 0 
 0 
 0 
 0 
 0 
 0 
 0 
 0 
 0 
 0 
 0 
 0 
 0 
 0 
 0 
 0 
 0 
 0 
 0 
 0 
 0 
 0 
 0 
 0 
 0 
 0 
 0 
 0 
 0 
 0 
 0 
 0 
 0 
 0 
 0 
 0 
 0 
 0 
 0 
 0 
 0 
 0 
 0 
 0 
 0 
 0 
 0 
 0 
 0 
 0 
 0 
 0 
 0 
 0 
 0 
 0 
 0 
 0 
 0 
 0 
 0 
 0 
 0 
 0 
 0 
 0 
 0 
 0 
 0 
 0 
 0 
 0 
 0 
 0 
 0 
 0 
 0 
 0 
 0 
 0 
 0 
 0 
 0 
 0 
 0 
 0 
 0 
 0 
 0 
 0 
 0 
 0 
 0 
 0 
 0 
 0 
 0 
 0 
 0 
 0 
 0 
 0 
 0 
 0 
 0 
 0 
 0 
 0 
 0 
 0 
 0 
 0 
 0 
 0 
 0 
 0 
 0 
 0 
 0 
 0 
 0 
 0 
 0 
 0 
 0 
 0 
 0 
 0 
 0 
 0 
 0 
 0 
 0 
 0 
 0 
 0 
 0 
 0 
 0 
 0 
 0 
 0 
 0 
 0 
 0 
 0 
 0 
 0 
 0 
 0 
 0 
 0 
 0 
 0 
 0 
 0 
 0 
 0 
 0 
 0 
 0 
 0 
 0 
 0 
 0 
 0 
 0 
 0 
 0 
 0 
 0 
 0 
 0 
 0 
 0 
 0 
 0 
 0 
 
 
 83 
 0 
 0 
 0 
 0 
 0 
 0 
 0 
 0 
 0 
 0 
 0 
 0 
 0 
 0 
 0 
 0 
 0 
 0 
 0 
 0 
 0 
 0 
 0 
 0 
 0 
 0 
 0 
 0 
 0 
 0 
 0 
 0 
 0 
 0 
 0 
 0 
 0 
 0 
 0 
 0 
 0 
 0 
 0 
 0 
 0 
 0 
 0 
 0 
 0 
 0 
 0 
 0 
 0 
 0 
 0 
 0 
 0 
 0 
 0 
 0 
 0 
 0 
 0 
 0 
 0 
 0 
 0 
 0 
 0 
 0 
 0 
 0 
 0 
 0 
 0 
 0 
 0 
 4 
 0 
 0 
 0 
 0 
 0 
 0 
 0 
 0 
 0 
 0 
 0 
 0 
 0 
 0 
 0 
 0 
 0 
 0 
 0 
 0 
 0 
 0 
 0 
 0 
 0 
 0 
 0 
 0 
 0 
 0 
 0 
 0 
 0 
 0 
 0 
 0 
 0 
 0 
 0 
 0 
 0 
 0 
 0 
 0 
 0 
 0 
 0 
 0 
 0 
 0 
 0 
 0 
 0 
 0 
 0 
 0 
 0 
 0 
 0 
 0 
 0 
 0 
 0 
 0 
 0 
 0 
 0 
 0 
 0 
 0 
 0 
 0 
 0 
 0 
 0 
 0 
 0 
 0 
 0 
 0 
 0 
 0 
 0 
 0 
 0 
 0 
 0 
 0 
 0 
 0 
 0 
 0 
 0 
 0 
 0 
 0 
 0 
 0 
 0 
 0 
 0 
 0 
 0 
 0 
 0 
 0 
 0 
 0 
 0 
 0 
 0 
 0 
 0 
 0 
 0 
 0 
 0 
 0 
 0 
 0 
 0 
 0 
 0 
 0 
 0 
 0 
 0 
 0 
 0 
 0 
 0 
 0 
 0 
 0 
 0 
 0 
 0 
 0 
 0 
 0 
 0 
 0 
 0 
 0 
 0 
 0 
 0 
 0 
 0 
 0 
 0 
 0 
 0 
 0 
 0 
 0 
 0 
 0 
 0 
 0 
 0 
 0 
 0 
 0 
 0 
 0 
 0 
 0 
 0 
 0 
 0 
 0 
 0 
 0 
 0 
 0 
 0 
 0 
 0 
 0 
 0 
 0 
 0 
 0 
 0 
 0 
 0 
 0 
 0 
 0 
 0 
 0 
 0 
 0 
 0 
 0 
 0 
 0 
 0 
 0 
 0 
 0 
 0 
 0 
 0 
 0 
 0 
 0 
 0 
 0 
 0 
 0 
 0 
 0 
 0 
 0 
 0 
 0 
 0 
 0 
 0 
 0 
 0 
 0 
 0 
 0 
 0 
 0 
 0 
 0 
 0 
 0 
 0 
 0 
 0 
 0 
 0 
 0 
 0 
 0 
 0 
 0 
 0 
 0 
 0 
 0 
 0 
 0 
 0 
 0 
 0 
 0 
 0 
 0 
 0 
 0 
 0 
 0 
 0 
 0 
 0 
 0 
 0 
 0 
 0 
 0 
 0 
 0 
 0 
 0 
 0 
 0 
 0 
 0 
 0 
 0 
 0 
 0 
 0 
 0 
 0 
 0 
 0 
 0 
 0 
 0 
 0 
 0 
 0 
 0 
 0 
 0 
 0 
 0 
 0 
 0 
 0 
 0 
 0 
 0 
 0 
 0 
 0 
 0 
 0 
 0 
 0 
 0 
 0 
 0 
 0 
 0 
 0 
 0 
 0 
 0 
 0 
 0 
 0 
 0 
 0 
 0 
 0 
 0 
 0 
 0 
 0 
 0 
 0 
 0 
 0 
 0 
 0 
 0 
 0 
 0 
 0 
 0 
 0 
 0 
 0 
 0 
 0 
 0 
 0 
 0 
 0 
 0 
 0 
 0 
 0 
 0 
 0 
 0 
 0 
 0 
 0 
 0 
 0 
 0 
 0 
 0 
 0 
 0 
 0 
 0 
 0 
 0 
 0 
 0 
 0 
 0 
 0 
 0 
 0 
 0 
 0 
 0 
 0 
 0 
 0 
 0 
 0 
 0 
 0 
 0 
 0 
 0 
 0 
 0 
 0 
 0 
 0 
 0 
 0 
 0 
 0 
 0 
 0 
 0 
 0 
 0 
 0 
 0 
 0 
 0 
 0 
 0 
 0 
 0 
 0 
 0 
 0 
 0 
 0 
 0 
 0 
 0 
 0 
 0 
 0 
 0 
 0 
 0 
 0 
 0 
 0 
 0 
 0 
 0 
 0 
 0 
 0 
 0 
 0 
 0 
 0 
 0 
 0 
 0 
 0 
 0 
 0 
 0 
 0 
 0 
 0 
 0 
 0 
 0 
 0 
 0 
 0 
 0 
 0 
 0 
 0 
 0 
 0 
 0 
 0 
 0 
 0 
 0 
 0 
 0 
 0 
 0 
 0 
 0 
 0 
 0 
 0 
 0 
 0 
 0 
 0 
 0 
 0 
 0 
 0 
 0 
 0 
 0 
 0 
 0 
 0 
 0 
 0 
 0 
 0 
 0 
 0 
 0 
 0 
 0 
 0 
 0 
 0 
 0 
 0 
 0 
 0 
 0 
 0 
 0 
 0 
 0 
 0 
 0 
 0 
 0 
 0 
 0 
 0 
 0 
 0 
 0 
 0 
 0 
 0 
 0 
 0 
 0 
 0 
 0 
 0 
 0 
 0 
 0 
 0 
 0 
 0 
 0 
 0 
 0 
 0 
 0 
 0 
 0 
 0 
 0 
 0 
 0 
 0 
 0 
 0 
 0 
 0 
 0 
 0 
 0 
 0 
 0 
 0 
 0 
 0 
 0 
 0 
 0 
 0 
 0 
 0 
 0 
 0 
 0 
 0 
 0 
 0 
 0 
 0 
 0 
 0 
 0 
 0 
 0 
 0 
 0 
 0 
 0 
 0 
 0 
 0 
 0 
 0 
 0 
 0 
 0 
 0 
 0 
 0 
 0 
 0 
 0 
 0 
 0 
 0 
 0 
 0 
 0 
 0 
 0 
 0 
 0 
 0 
 0 
 0 
 0 
 0 
 0 
 0 
 0 
 0 
 0 
 0 
 0 
 0 
 0 
 0 
 0 
 0 
 0 
 0 
 0 
 0 
 0 
 0 
 0 
 0 
 0 
 0 
 0 
 0 
 0 
 0 
 0 
 0 
 0 
 0 
 0 
 0 
 0 
 0 
 0 
 0 
 0 
 0 
 0 
 0 
 0 
 0 
 0 
 0 
 0 
 0 
 0 
 0 
 0 
 0 
 0 
 0 
 0 
 0 
 0 
 0 
 0 
 0 
 0 
 0 
 0 
 0 
 0 
 0 
 0 
 0 
 0 
 0 
 0 
 0 
 0 
 0 
 0 
 0 
 0 
 0 
 0 
 0 
 0 
 0 
 0 
 0 
 0 
 0 
 0 
 0 
 0 
 0 
 0 
 0 
 0 
 0 
 0 
 0 
 0 
 0 
 0 
 0 
 0 
 0 
 0 
 0 
 0 
 0 
 0 
 0 
 0 
 0 
 0 
 0 
 0 
 0 
 0 
 0 
 0 
 0 
 0 
 0 
 0 
 0 
 0 
 0 
 0 
 0 
 0 
 0 
 0 
 0 
 0 
 0 
 0 
 0 
 0 
 0 
 0 
 0 
 0 
 0 
 0 
 
 
 84 
 0 
 0 
 0 
 0 
 0 
 0 
 0 
 0 
 0 
 0 
 0 
 0 
 0 
 0 
 0 
 0 
 0 
 0 
 0 
 0 
 0 
 0 
 0 
 0 
 0 
 0 
 0 
 0 
 0 
 0 
 0 
 0 
 0 
 0 
 0 
 0 
 0 
 0 
 0 
 0 
 0 
 0 
 0 
 0 
 0 
 0 
 0 
 0 
 0 
 0 
 0 
 0 
 0 
 0 
 0 
 0 
 0 
 0 
 0 
 0 
 0 
 0 
 0 
 0 
 0 
 0 
 0 
 0 
 0 
 0 
 0 
 0 
 0 
 0 
 0 
 0 
 0 
 0 
 0 
 0 
 0 
 0 
 0 
 0 
 0 
 0 
 0 
 0 
 0 
 0 
 0 
 0 
 0 
 0 
 0 
 0 
 0 
 0 
 0 
 0 
 0 
 0 
 0 
 0 
 0 
 0 
 0 
 0 
 0 
 0 
 0 
 0 
 0 
 0 
 0 
 0 
 0 
 0 
 0 
 0 
 0 
 0 
 0 
 0 
 0 
 0 
 0 
 0 
 0 
 0 
 0 
 0 
 0 
 0 
 0 
 0 
 0 
 0 
 0 
 0 
 0 
 0 
 0 
 0 
 0 
 0 
 0 
 0 
 0 
 0 
 0 
 0 
 0 
 0 
 0 
 0 
 0 
 0 
 0 
 0 
 0 
 0 
 0 
 0 
 0 
 0 
 0 
 0 
 0 
 0 
 0 
 0 
 0 
 0 
 0 
 0 
 0 
 0 
 0 
 0 
 0 
 0 
 0 
 12 
 0 
 0 
 0 
 0 
 0 
 0 
 0 
 0 
 0 
 0 
 0 
 0 
 0 
 0 
 0 
 0 
 0 
 0 
 0 
 0 
 0 
 0 
 0 
 0 
 0 
 0 
 0 
 0 
 0 
 0 
 0 
 0 
 0 
 0 
 0 
 0 
 0 
 0 
 0 
 0 
 0 
 0 
 0 
 0 
 0 
 0 
 0 
 0 
 0 
 0 
 0 
 0 
 0 
 0 
 0 
 0 
 0 
 0 
 0 
 0 
 0 
 0 
 0 
 0 
 0 
 0 
 0 
 0 
 0 
 0 
 0 
 0 
 0 
 0 
 0 
 0 
 0 
 0 
 0 
 0 
 0 
 0 
 0 
 0 
 0 
 0 
 0 
 0 
 0 
 0 
 0 
 0 
 0 
 0 
 0 
 0 
 0 
 0 
 0 
 0 
 0 
 0 
 0 
 0 
 0 
 0 
 0 
 0 
 0 
 0 
 0 
 0 
 0 
 0 
 0 
 0 
 0 
 0 
 0 
 0 
 0 
 0 
 0 
 0 
 0 
 0 
 0 
 0 
 0 
 0 
 0 
 0 
 0 
 0 
 0 
 0 
 0 
 0 
 0 
 0 
 0 
 0 
 0 
 0 
 0 
 0 
 0 
 0 
 0 
 0 
 0 
 0 
 0 
 0 
 0 
 0 
 0 
 0 
 0 
 0 
 0 
 0 
 0 
 0 
 0 
 0 
 0 
 0 
 0 
 0 
 0 
 0 
 0 
 0 
 0 
 0 
 0 
 0 
 0 
 0 
 0 
 0 
 0 
 0 
 0 
 0 
 0 
 0 
 0 
 0 
 0 
 0 
 0 
 0 
 0 
 0 
 0 
 0 
 0 
 0 
 0 
 0 
 0 
 0 
 0 
 0 
 0 
 0 
 0 
 0 
 0 
 0 
 0 
 0 
 0 
 0 
 0 
 0 
 0 
 0 
 0 
 0 
 0 
 0 
 0 
 0 
 0 
 0 
 0 
 0 
 0 
 0 
 0 
 0 
 0 
 0 
 0 
 0 
 0 
 0 
 0 
 0 
 0 
 0 
 0 
 0 
 0 
 0 
 0 
 0 
 0 
 0 
 0 
 0 
 0 
 0 
 0 
 0 
 0 
 0 
 0 
 0 
 0 
 0 
 0 
 0 
 0 
 0 
 0 
 0 
 0 
 0 
 0 
 0 
 0 
 0 
 0 
 0 
 0 
 0 
 0 
 0 
 0 
 0 
 0 
 0 
 0 
 0 
 0 
 0 
 0 
 0 
 0 
 0 
 0 
 0 
 0 
 0 
 0 
 0 
 0 
 0 
 0 
 0 
 0 
 0 
 0 
 0 
 0 
 0 
 0 
 0 
 0 
 0 
 0 
 0 
 0 
 0 
 0 
 0 
 0 
 0 
 0 
 0 
 0 
 0 
 0 
 0 
 0 
 0 
 0 
 0 
 0 
 0 
 0 
 0 
 0 
 0 
 0 
 0 
 0 
 0 
 0 
 0 
 0 
 0 
 0 
 0 
 0 
 0 
 0 
 0 
 0 
 0 
 0 
 0 
 0 
 0 
 0 
 0 
 0 
 0 
 0 
 0 
 0 
 0 
 0 
 0 
 0 
 0 
 0 
 0 
 0 
 0 
 0 
 0 
 0 
 0 
 0 
 0 
 0 
 0 
 0 
 0 
 0 
 0 
 0 
 0 
 0 
 0 
 0 
 0 
 0 
 0 
 0 
 0 
 0 
 0 
 0 
 0 
 0 
 0 
 0 
 0 
 0 
 0 
 0 
 0 
 0 
 0 
 0 
 0 
 0 
 0 
 0 
 0 
 0 
 0 
 0 
 0 
 0 
 0 
 0 
 0 
 0 
 0 
 0 
 0 
 0 
 0 
 0 
 0 
 0 
 0 
 0 
 0 
 0 
 0 
 0 
 0 
 0 
 0 
 0 
 0 
 0 
 0 
 0 
 0 
 0 
 0 
 0 
 0 
 0 
 0 
 0 
 0 
 0 
 0 
 0 
 0 
 0 
 0 
 0 
 0 
 0 
 0 
 0 
 0 
 0 
 0 
 0 
 0 
 0 
 0 
 0 
 0 
 0 
 0 
 0 
 0 
 0 
 0 
 0 
 0 
 0 
 0 
 0 
 0 
 0 
 0 
 0 
 0 
 0 
 0 
 0 
 0 
 0 
 0 
 0 
 0 
 0 
 0 
 0 
 0 
 0 
 0 
 0 
 0 
 0 
 0 
 0 
 0 
 0 
 0 
 0 
 0 
 0 
 0 
 0 
 0 
 0 
 0 
 0 
 0 
 0 
 0 
 0 
 0 
 0 
 0 
 0 
 0 
 0 
 0 
 0 
 0 
 0 
 0 
 0 
 0 
 0 
 0 
 0 
 0 
 0 
 0 
 0 
 0 
 0 
 0 
 0 
 0 
 0 
 0 
 0 
 0 
 0 
 0 
 0 
 0 
 0 
 0 
 0 
 0 
 0 
 0 
 0 
 0 
 0 
 0 
 0 
 0 
 0 
 0 
 0 
 0 
 0 
 0 
 0 
 0 
 0 
 0 
 0 
 0 
 0 
 0 
 0 
 0 
 0 
 0 
 0 
 0 
 0 
 0 
 0 
 0 
 0 
 0 
 0 
 0 
 0 
 0 
 0 
 0 
 0 
 0 
 0 
 0 
 0 
 0 
 0 
 0 
 0 
 0 
 0 
 0 
 0 
 0 
 0 
 0 
 0 
 0 
 0 
 0 
 0 
 0 
 0 
 0 
 0 
 0 
 0 
 0 
 0 
 0 
 0 
 0 
 0 
 0 
 0 
 0 
 0 
 0 
 
 
 85 
 0 
 0 
 0 
 0 
 0 
 0 
 0 
 0 
 0 
 0 
 0 
 0 
 0 
 0 
 0 
 0 
 0 
 0 
 0 
 0 
 0 
 0 
 0 
 0 
 0 
 0 
 0 
 0 
 0 
 0 
 0 
 0 
 0 
 0 
 0 
 0 
 0 
 0 
 0 
 0 
 0 
 0 
 0 
 0 
 0 
 0 
 0 
 0 
 0 
 0 
 0 
 0 
 0 
 0 
 0 
 0 
 0 
 0 
 0 
 0 
 0 
 0 
 0 
 0 
 0 
 0 
 0 
 0 
 0 
 0 
 0 
 0 
 0 
 0 
 0 
 0 
 0 
 0 
 0 
 0 
 0 
 0 
 0 
 0 
 0 
 0 
 0 
 0 
 0 
 0 
 0 
 0 
 0 
 0 
 0 
 0 
 0 
 0 
 0 
 0 
 0 
 0 
 0 
 0 
 0 
 0 
 0 
 0 
 0 
 0 
 0 
 0 
 0 
 0 
 0 
 0 
 0 
 0 
 0 
 0 
 0 
 0 
 0 
 0 
 0 
 0 
 0 
 0 
 0 
 0 
 0 
 0 
 0 
 0 
 0 
 0 
 0 
 0 
 0 
 0 
 0 
 0 
 0 
 0 
 0 
 0 
 0 
 0 
 0 
 0 
 0 
 0 
 0 
 0 
 0 
 0 
 0 
 0 
 0 
 0 
 0 
 0 
 0 
 0 
 0 
 0 
 0 
 0 
 0 
 0 
 0 
 0 
 0 
 0 
 0 
 0 
 0 
 0 
 0 
 0 
 0 
 0 
 0 
 0 
 0 
 0 
 0 
 0 
 0 
 0 
 0 
 0 
 0 
 0 
 0 
 0 
 0 
 0 
 0 
 0 
 0 
 0 
 0 
 0 
 0 
 0 
 0 
 0 
 0 
 0 
 0 
 0 
 0 
 0 
 0 
 0 
 0 
 0 
 0 
 0 
 0 
 0 
 0 
 0 
 0 
 0 
 0 
 0 
 0 
 0 
 0 
 0 
 0 
 0 
 0 
 0 
 0 
 0 
 0 
 0 
 0 
 0 
 0 
 0 
 0 
 0 
 0 
 0 
 0 
 0 
 0 
 0 
 0 
 0 
 0 
 0 
 0 
 0 
 0 
 0 
 0 
 0 
 0 
 0 
 0 
 0 
 0 
 0 
 0 
 0 
 0 
 0 
 0 
 0 
 0 
 0 
 0 
 0 
 0 
 0 
 0 
 0 
 0 
 0 
 0 
 0 
 0 
 0 
 0 
 0 
 0 
 0 
 0 
 0 
 0 
 0 
 0 
 0 
 0 
 0 
 0 
 0 
 0 
 0 
 0 
 0 
 0 
 0 
 0 
 0 
 0 
 0 
 0 
 0 
 0 
 0 
 0 
 0 
 0 
 0 
 0 
 0 
 0 
 0 
 0 
 0 
 0 
 0 
 0 
 0 
 0 
 0 
 0 
 0 
 0 
 0 
 0 
 0 
 0 
 0 
 0 
 0 
 0 
 0 
 0 
 0 
 0 
 0 
 0 
 0 
 0 
 0 
 0 
 0 
 0 
 0 
 0 
 0 
 0 
 0 
 0 
 0 
 0 
 0 
 0 
 0 
 0 
 0 
 0 
 0 
 0 
 0 
 0 
 0 
 0 
 0 
 0 
 0 
 0 
 0 
 0 
 0 
 0 
 0 
 0 
 0 
 0 
 0 
 0 
 0 
 0 
 0 
 0 
 0 
 0 
 0 
 0 
 0 
 0 
 0 
 0 
 0 
 0 
 0 
 0 
 0 
 0 
 0 
 0 
 0 
 0 
 0 
 0 
 0 
 0 
 0 
 0 
 0 
 0 
 0 
 0 
 0 
 0 
 0 
 0 
 0 
 0 
 0 
 0 
 0 
 0 
 0 
 0 
 0 
 0 
 0 
 0 
 0 
 0 
 0 
 0 
 0 
 0 
 0 
 0 
 0 
 0 
 0 
 0 
 0 
 0 
 0 
 0 
 0 
 0 
 0 
 0 
 0 
 0 
 0 
 0 
 0 
 0 
 0 
 0 
 0 
 0 
 0 
 0 
 0 
 0 
 0 
 0 
 0 
 0 
 0 
 0 
 0 
 0 
 0 
 0 
 0 
 0 
 0 
 0 
 0 
 0 
 0 
 0 
 0 
 0 
 0 
 0 
 0 
 0 
 0 
 0 
 0 
 0 
 0 
 0 
 0 
 0 
 0 
 0 
 0 
 0 
 0 
 0 
 0 
 0 
 0 
 0 
 0 
 0 
 0 
 0 
 0 
 0 
 0 
 0 
 0 
 0 
 0 
 0 
 0 
 0 
 0 
 0 
 0 
 0 
 0 
 0 
 0 
 0 
 0 
 0 
 0 
 0 
 0 
 0 
 0 
 0 
 0 
 0 
 0 
 0 
 0 
 0 
 0 
 0 
 0 
 0 
 0 
 0 
 0 
 0 
 0 
 0 
 0 
 0 
 0 
 0 
 0 
 0 
 0 
 0 
 0 
 0 
 0 
 0 
 0 
 0 
 0 
 0 
 0 
 0 
 0 
 0 
 0 
 0 
 0 
 0 
 0 
 0 
 0 
 0 
 0 
 0 
 0 
 0 
 0 
 0 
 0 
 0 
 0 
 0 
 0 
 0 
 0 
 0 
 0 
 0 
 0 
 0 
 0 
 0 
 0 
 0 
 0 
 0 
 0 
 0 
 0 
 0 
 0 
 0 
 0 
 0 
 0 
 0 
 0 
 0 
 0 
 0 
 0 
 0 
 0 
 0 
 0 
 0 
 0 
 0 
 0 
 0 
 0 
 0 
 0 
 0 
 0 
 0 
 0 
 0 
 0 
 0 
 0 
 0 
 0 
 0 
 0 
 0 
 0 
 0 
 0 
 0 
 0 
 0 
 0 
 0 
 0 
 0 
 0 
 0 
 0 
 0 
 0 
 0 
 0 
 0 
 0 
 0 
 0 
 0 
 0 
 0 
 0 
 0 
 0 
 0 
 0 
 0 
 0 
 0 
 0 
 0 
 0 
 0 
 0 
 0 
 0 
 0 
 0 
 0 
 0 
 0 
 0 
 0 
 0 
 0 
 0 
 0 
 0 
 0 
 0 
 0 
 0 
 0 
 0 
 0 
 0 
 0 
 0 
 0 
 0 
 0 
 0 
 0 
 0 
 0 
 0 
 0 
 0 
 0 
 0 
 0 
 0 
 0 
 0 
 0 
 0 
 0 
 0 
 0 
 1 
 0 
 0 
 0 
 0 
 0 
 0 
 0 
 0 
 0 
 0 
 0 
 0 
 0 
 0 
 0 
 0 
 0 
 0 
 0 
 0 
 0 
 0 
 0 
 0 
 0 
 0 
 0 
 0 
 0 
 0 
 0 
 0 
 0 
 0 
 0 
 0 
 0 
 0 
 0 
 0 
 0 
 0 
 0 
 0 
 0 
 0 
 0 
 0 
 0 
 0 
 0 
 0 
 0 
 0 
 0 
 0 
 0 
 0 
 0 
 0 
 0 
 0 
 0 
 0 
 0 
 0 
 0 
 0 
 0 
 0 
 0 
 0 
 0 
 0 
 0 
 0 
 0 
 0 
 0 
 0 
 0 
 0 
 0 
 0 
 0 
 0 
 0 
 0 
 0 
 0 
 0 
 0 
 
 
 86 
 0 
 0 
 0 
 0 
 0 
 0 
 0 
 0 
 0 
 0 
 0 
 0 
 0 
 0 
 0 
 0 
 0 
 0 
 0 
 0 
 0 
 0 
 0 
 0 
 0 
 0 
 0 
 0 
 0 
 0 
 0 
 0 
 0 
 0 
 0 
 0 
 0 
 0 
 0 
 0 
 0 
 0 
 0 
 0 
 0 
 0 
 0 
 0 
 0 
 0 
 0 
 0 
 0 
 0 
 0 
 0 
 0 
 0 
 0 
 0 
 0 
 0 
 0 
 0 
 0 
 0 
 0 
 0 
 0 
 0 
 0 
 0 
 0 
 0 
 0 
 0 
 0 
 0 
 0 
 0 
 0 
 0 
 0 
 0 
 0 
 0 
 7 
 0 
 0 
 0 
 0 
 0 
 0 
 0 
 0 
 0 
 0 
 0 
 0 
 0 
 0 
 0 
 0 
 0 
 0 
 0 
 0 
 0 
 0 
 0 
 0 
 0 
 0 
 0 
 0 
 0 
 0 
 0 
 0 
 0 
 0 
 0 
 0 
 0 
 0 
 0 
 0 
 0 
 0 
 0 
 0 
 0 
 0 
 0 
 0 
 0 
 0 
 0 
 0 
 0 
 0 
 0 
 0 
 0 
 0 
 0 
 0 
 0 
 0 
 0 
 0 
 0 
 0 
 0 
 0 
 0 
 0 
 0 
 0 
 0 
 0 
 0 
 0 
 0 
 0 
 0 
 0 
 0 
 0 
 0 
 0 
 0 
 0 
 0 
 0 
 0 
 0 
 0 
 0 
 0 
 0 
 0 
 0 
 0 
 0 
 0 
 0 
 0 
 0 
 0 
 0 
 0 
 0 
 0 
 0 
 0 
 0 
 0 
 0 
 0 
 0 
 0 
 0 
 0 
 0 
 0 
 0 
 0 
 0 
 0 
 0 
 0 
 0 
 0 
 0 
 0 
 0 
 0 
 0 
 0 
 0 
 0 
 0 
 0 
 0 
 0 
 0 
 0 
 0 
 0 
 0 
 0 
 0 
 0 
 0 
 0 
 0 
 0 
 0 
 0 
 0 
 0 
 0 
 0 
 0 
 0 
 0 
 0 
 0 
 0 
 0 
 0 
 0 
 0 
 0 
 0 
 0 
 0 
 0 
 0 
 0 
 0 
 0 
 0 
 0 
 0 
 0 
 0 
 0 
 0 
 0 
 0 
 0 
 0 
 0 
 0 
 0 
 0 
 0 
 0 
 0 
 0 
 0 
 0 
 0 
 0 
 0 
 0 
 0 
 0 
 0 
 0 
 0 
 0 
 0 
 0 
 0 
 0 
 0 
 0 
 0 
 0 
 0 
 0 
 0 
 0 
 0 
 0 
 0 
 0 
 0 
 0 
 0 
 0 
 0 
 0 
 0 
 0 
 0 
 0 
 0 
 0 
 0 
 0 
 0 
 0 
 0 
 0 
 0 
 0 
 0 
 0 
 0 
 0 
 0 
 0 
 0 
 0 
 0 
 0 
 0 
 0 
 0 
 0 
 0 
 0 
 0 
 0 
 0 
 0 
 0 
 0 
 0 
 0 
 0 
 0 
 0 
 0 
 0 
 0 
 0 
 0 
 0 
 0 
 0 
 0 
 0 
 0 
 0 
 0 
 0 
 0 
 0 
 0 
 0 
 0 
 0 
 0 
 0 
 0 
 0 
 0 
 0 
 0 
 0 
 0 
 0 
 0 
 0 
 0 
 0 
 0 
 0 
 0 
 0 
 0 
 0 
 0 
 0 
 0 
 0 
 0 
 0 
 0 
 0 
 0 
 0 
 0 
 0 
 0 
 0 
 0 
 0 
 0 
 0 
 0 
 0 
 0 
 0 
 0 
 0 
 0 
 0 
 0 
 0 
 0 
 0 
 0 
 0 
 0 
 0 
 0 
 0 
 0 
 0 
 0 
 0 
 0 
 0 
 0 
 0 
 0 
 0 
 0 
 0 
 0 
 0 
 0 
 0 
 0 
 0 
 0 
 0 
 0 
 0 
 0 
 0 
 0 
 0 
 0 
 0 
 0 
 0 
 0 
 0 
 0 
 0 
 0 
 0 
 0 
 0 
 0 
 0 
 0 
 0 
 0 
 0 
 0 
 0 
 0 
 0 
 0 
 0 
 0 
 0 
 0 
 0 
 0 
 0 
 0 
 0 
 0 
 0 
 0 
 0 
 0 
 0 
 0 
 0 
 0 
 0 
 0 
 0 
 0 
 0 
 0 
 0 
 0 
 0 
 0 
 0 
 0 
 0 
 0 
 0 
 0 
 0 
 0 
 0 
 0 
 0 
 0 
 0 
 0 
 0 
 0 
 0 
 0 
 0 
 0 
 0 
 0 
 0 
 0 
 0 
 0 
 0 
 0 
 0 
 0 
 0 
 0 
 0 
 0 
 0 
 0 
 0 
 0 
 0 
 0 
 0 
 0 
 0 
 0 
 0 
 0 
 0 
 0 
 0 
 0 
 0 
 0 
 0 
 0 
 0 
 0 
 0 
 0 
 0 
 0 
 0 
 0 
 0 
 0 
 0 
 0 
 0 
 0 
 0 
 0 
 0 
 0 
 0 
 0 
 0 
 0 
 0 
 0 
 0 
 0 
 0 
 0 
 0 
 0 
 0 
 0 
 0 
 0 
 0 
 0 
 0 
 0 
 0 
 0 
 0 
 0 
 0 
 0 
 0 
 0 
 0 
 0 
 0 
 0 
 0 
 0 
 0 
 0 
 0 
 0 
 0 
 0 
 0 
 0 
 0 
 0 
 0 
 0 
 0 
 0 
 0 
 0 
 0 
 0 
 0 
 0 
 0 
 0 
 0 
 0 
 0 
 0 
 0 
 0 
 0 
 0 
 0 
 0 
 0 
 0 
 0 
 0 
 0 
 0 
 0 
 0 
 0 
 0 
 0 
 0 
 0 
 0 
 0 
 0 
 0 
 0 
 0 
 0 
 0 
 0 
 0 
 0 
 0 
 0 
 0 
 0 
 0 
 0 
 0 
 0 
 0 
 0 
 0 
 0 
 0 
 0 
 0 
 0 
 0 
 0 
 0 
 0 
 0 
 0 
 0 
 0 
 0 
 0 
 0 
 0 
 0 
 0 
 0 
 0 
 0 
 0 
 0 
 0 
 0 
 0 
 0 
 0 
 0 
 0 
 0 
 0 
 0 
 0 
 0 
 0 
 0 
 0 
 0 
 0 
 0 
 0 
 0 
 0 
 0 
 0 
 0 
 0 
 0 
 0 
 0 
 0 
 0 
 0 
 0 
 0 
 0 
 0 
 0 
 0 
 0 
 0 
 0 
 0 
 0 
 0 
 0 
 0 
 0 
 0 
 0 
 0 
 0 
 0 
 0 
 0 
 0 
 0 
 0 
 0 
 0 
 0 
 0 
 0 
 0 
 0 
 0 
 0 
 0 
 0 
 0 
 0 
 0 
 0 
 0 
 0 
 0 
 0 
 0 
 0 
 0 
 0 
 0 
 0 
 0 
 0 
 0 
 0 
 0 
 0 
 0 
 0 
 0 
 0 
 0 
 0 
 0 
 0 
 0 
 0 
 0 
 0 
 0 
 0 
 0 
 0 
 0 
 0 
 0 
 0 
 0 
 0 
 0 
 0 
 0 
 0 
 0 
 0 
 0 
 0 
 0 
 0 
 
 
 87 
 0 
 0 
 0 
 0 
 0 
 0 
 0 
 0 
 0 
 0 
 0 
 0 
 0 
 0 
 0 
 0 
 0 
 0 
 0 
 0 
 0 
 0 
 0 
 0 
 0 
 0 
 0 
 0 
 0 
 0 
 0 
 0 
 0 
 0 
 0 
 0 
 0 
 0 
 0 
 0 
 0 
 0 
 0 
 0 
 0 
 0 
 0 
 0 
 0 
 0 
 0 
 0 
 0 
 0 
 0 
 0 
 0 
 0 
 0 
 0 
 0 
 0 
 0 
 0 
 0 
 0 
 0 
 0 
 0 
 0 
 0 
 0 
 0 
 0 
 0 
 0 
 0 
 0 
 0 
 0 
 0 
 0 
 0 
 0 
 0 
 0 
 0 
 11 
 0 
 0 
 0 
 0 
 0 
 0 
 0 
 0 
 0 
 0 
 0 
 0 
 0 
 0 
 0 
 0 
 0 
 0 
 0 
 0 
 0 
 0 
 0 
 0 
 0 
 0 
 0 
 0 
 0 
 0 
 0 
 0 
 0 
 0 
 0 
 0 
 0 
 0 
 0 
 0 
 0 
 0 
 0 
 0 
 0 
 0 
 0 
 0 
 0 
 0 
 0 
 0 
 0 
 0 
 0 
 0 
 0 
 0 
 0 
 0 
 0 
 0 
 0 
 0 
 0 
 0 
 0 
 0 
 0 
 0 
 0 
 0 
 0 
 0 
 0 
 0 
 0 
 0 
 0 
 0 
 0 
 0 
 0 
 0 
 0 
 0 
 0 
 0 
 0 
 0 
 0 
 0 
 0 
 0 
 0 
 0 
 0 
 0 
 0 
 0 
 0 
 0 
 0 
 0 
 0 
 0 
 0 
 0 
 0 
 0 
 0 
 0 
 0 
 0 
 0 
 0 
 0 
 0 
 0 
 0 
 0 
 0 
 0 
 0 
 0 
 0 
 0 
 0 
 0 
 0 
 0 
 0 
 0 
 0 
 0 
 0 
 0 
 0 
 0 
 0 
 0 
 0 
 0 
 0 
 0 
 0 
 0 
 0 
 0 
 0 
 0 
 0 
 0 
 0 
 0 
 0 
 0 
 0 
 0 
 0 
 0 
 0 
 0 
 0 
 0 
 0 
 0 
 0 
 0 
 0 
 0 
 0 
 0 
 0 
 0 
 0 
 0 
 0 
 0 
 0 
 0 
 0 
 0 
 0 
 0 
 0 
 0 
 0 
 0 
 0 
 0 
 0 
 0 
 0 
 0 
 0 
 0 
 0 
 0 
 0 
 0 
 0 
 0 
 0 
 0 
 0 
 0 
 0 
 0 
 0 
 0 
 0 
 0 
 0 
 0 
 0 
 0 
 0 
 0 
 0 
 0 
 0 
 0 
 0 
 0 
 0 
 0 
 0 
 0 
 0 
 0 
 0 
 0 
 0 
 0 
 0 
 0 
 0 
 0 
 0 
 0 
 0 
 0 
 0 
 0 
 0 
 0 
 0 
 0 
 0 
 0 
 0 
 0 
 0 
 0 
 0 
 0 
 0 
 0 
 0 
 0 
 0 
 0 
 0 
 0 
 0 
 0 
 0 
 0 
 0 
 0 
 0 
 0 
 0 
 0 
 0 
 0 
 0 
 0 
 0 
 0 
 0 
 0 
 0 
 0 
 0 
 0 
 0 
 0 
 0 
 0 
 0 
 0 
 0 
 0 
 0 
 0 
 0 
 0 
 0 
 0 
 0 
 0 
 0 
 0 
 0 
 0 
 0 
 0 
 0 
 0 
 0 
 0 
 0 
 0 
 0 
 0 
 0 
 0 
 0 
 0 
 0 
 0 
 0 
 0 
 0 
 0 
 0 
 0 
 0 
 0 
 0 
 0 
 0 
 0 
 0 
 0 
 0 
 0 
 0 
 0 
 0 
 0 
 0 
 0 
 0 
 0 
 0 
 0 
 0 
 0 
 0 
 0 
 0 
 0 
 0 
 0 
 0 
 0 
 0 
 0 
 0 
 0 
 0 
 0 
 0 
 0 
 0 
 0 
 0 
 0 
 0 
 0 
 0 
 0 
 0 
 0 
 0 
 0 
 0 
 0 
 0 
 0 
 0 
 0 
 0 
 0 
 0 
 0 
 0 
 0 
 0 
 0 
 0 
 0 
 0 
 0 
 0 
 0 
 0 
 0 
 0 
 0 
 0 
 0 
 0 
 0 
 0 
 0 
 0 
 0 
 0 
 0 
 0 
 0 
 0 
 0 
 0 
 0 
 0 
 0 
 0 
 0 
 0 
 0 
 0 
 0 
 0 
 0 
 0 
 0 
 0 
 0 
 0 
 0 
 0 
 0 
 0 
 0 
 0 
 0 
 0 
 0 
 0 
 0 
 0 
 0 
 0 
 0 
 0 
 0 
 0 
 0 
 0 
 0 
 0 
 0 
 0 
 0 
 0 
 0 
 0 
 0 
 0 
 0 
 0 
 0 
 0 
 0 
 0 
 0 
 0 
 0 
 0 
 0 
 0 
 0 
 0 
 0 
 0 
 0 
 0 
 0 
 0 
 0 
 0 
 0 
 0 
 0 
 0 
 0 
 0 
 0 
 0 
 0 
 0 
 0 
 0 
 0 
 0 
 0 
 0 
 0 
 0 
 0 
 0 
 0 
 0 
 0 
 0 
 0 
 0 
 0 
 0 
 0 
 0 
 0 
 0 
 0 
 0 
 0 
 0 
 0 
 0 
 0 
 0 
 0 
 0 
 0 
 0 
 0 
 0 
 0 
 0 
 0 
 0 
 0 
 0 
 0 
 0 
 0 
 0 
 0 
 0 
 0 
 0 
 0 
 0 
 0 
 0 
 0 
 0 
 0 
 0 
 0 
 0 
 0 
 0 
 0 
 0 
 0 
 0 
 0 
 0 
 0 
 0 
 0 
 0 
 0 
 0 
 0 
 0 
 0 
 0 
 0 
 0 
 0 
 0 
 0 
 0 
 0 
 0 
 0 
 0 
 0 
 0 
 0 
 0 
 0 
 0 
 0 
 0 
 0 
 0 
 0 
 0 
 0 
 0 
 0 
 0 
 0 
 0 
 0 
 0 
 0 
 0 
 0 
 0 
 0 
 0 
 0 
 0 
 0 
 0 
 0 
 0 
 0 
 0 
 0 
 0 
 0 
 0 
 0 
 0 
 0 
 0 
 0 
 0 
 0 
 0 
 0 
 0 
 0 
 0 
 0 
 0 
 0 
 0 
 0 
 0 
 0 
 0 
 0 
 0 
 0 
 0 
 0 
 0 
 0 
 0 
 0 
 0 
 0 
 0 
 0 
 0 
 0 
 0 
 0 
 0 
 0 
 0 
 0 
 0 
 0 
 0 
 0 
 0 
 0 
 0 
 0 
 0 
 0 
 0 
 0 
 0 
 0 
 0 
 0 
 0 
 0 
 0 
 0 
 0 
 0 
 0 
 0 
 0 
 0 
 0 
 0 
 0 
 0 
 0 
 0 
 0 
 0 
 0 
 0 
 0 
 0 
 0 
 0 
 0 
 0 
 0 
 0 
 0 
 0 
 0 
 0 
 0 
 0 
 0 
 0 
 0 
 0 
 0 
 0 
 0 
 0 
 0 
 0 
 0 
 0 
 0 
 0 
 0 
 0 
 0 
 0 
 0 
 0 
 0 
 0 
 0 
 0 
 0 
 
 
 88 
 0 
 0 
 0 
 0 
 0 
 0 
 0 
 0 
 0 
 0 
 0 
 0 
 0 
 0 
 0 
 0 
 0 
 0 
 0 
 0 
 0 
 0 
 0 
 0 
 0 
 0 
 0 
 0 
 0 
 0 
 0 
 0 
 0 
 0 
 0 
 0 
 0 
 0 
 0 
 0 
 0 
 0 
 0 
 0 
 0 
 0 
 0 
 0 
 0 
 0 
 0 
 0 
 0 
 0 
 0 
 0 
 0 
 0 
 0 
 0 
 0 
 0 
 0 
 0 
 0 
 0 
 0 
 0 
 0 
 0 
 0 
 0 
 0 
 0 
 0 
 0 
 0 
 0 
 0 
 2 
 0 
 0 
 0 
 0 
 0 
 0 
 0 
 0 
 0 
 0 
 0 
 0 
 0 
 0 
 0 
 0 
 0 
 0 
 0 
 0 
 0 
 0 
 0 
 0 
 0 
 0 
 0 
 0 
 0 
 0 
 0 
 0 
 0 
 0 
 0 
 0 
 0 
 0 
 0 
 0 
 0 
 0 
 0 
 0 
 0 
 0 
 0 
 0 
 0 
 0 
 0 
 0 
 0 
 0 
 0 
 0 
 0 
 0 
 0 
 0 
 0 
 0 
 0 
 0 
 0 
 0 
 0 
 0 
 0 
 0 
 0 
 0 
 0 
 0 
 0 
 0 
 0 
 0 
 0 
 0 
 0 
 0 
 0 
 0 
 0 
 0 
 0 
 0 
 0 
 0 
 0 
 0 
 0 
 0 
 0 
 0 
 0 
 0 
 0 
 0 
 0 
 0 
 0 
 0 
 0 
 0 
 0 
 0 
 0 
 0 
 0 
 0 
 0 
 0 
 0 
 0 
 0 
 0 
 0 
 0 
 0 
 0 
 0 
 0 
 0 
 0 
 0 
 0 
 0 
 0 
 0 
 0 
 0 
 0 
 0 
 0 
 0 
 0 
 0 
 0 
 0 
 0 
 0 
 0 
 0 
 0 
 0 
 0 
 0 
 0 
 0 
 0 
 0 
 0 
 0 
 0 
 0 
 0 
 0 
 0 
 0 
 0 
 0 
 0 
 0 
 0 
 0 
 0 
 0 
 0 
 0 
 0 
 0 
 0 
 0 
 0 
 0 
 0 
 0 
 0 
 0 
 0 
 0 
 0 
 0 
 0 
 0 
 0 
 0 
 0 
 0 
 0 
 0 
 0 
 0 
 0 
 0 
 0 
 0 
 0 
 0 
 0 
 0 
 0 
 0 
 0 
 0 
 0 
 0 
 0 
 0 
 0 
 0 
 0 
 0 
 0 
 0 
 0 
 0 
 0 
 0 
 0 
 0 
 0 
 0 
 0 
 0 
 0 
 0 
 0 
 0 
 0 
 0 
 0 
 0 
 0 
 0 
 0 
 0 
 0 
 0 
 0 
 0 
 0 
 0 
 0 
 0 
 0 
 0 
 0 
 0 
 0 
 0 
 0 
 0 
 0 
 0 
 0 
 0 
 0 
 0 
 0 
 0 
 0 
 0 
 0 
 0 
 0 
 0 
 0 
 0 
 0 
 0 
 0 
 0 
 0 
 0 
 0 
 0 
 0 
 0 
 0 
 0 
 0 
 0 
 0 
 0 
 0 
 0 
 0 
 0 
 0 
 0 
 0 
 0 
 0 
 0 
 0 
 0 
 0 
 0 
 0 
 0 
 0 
 0 
 0 
 0 
 0 
 0 
 0 
 0 
 0 
 0 
 0 
 0 
 0 
 0 
 0 
 0 
 0 
 0 
 0 
 0 
 0 
 0 
 0 
 0 
 0 
 0 
 0 
 0 
 0 
 0 
 0 
 0 
 0 
 0 
 0 
 0 
 0 
 0 
 0 
 0 
 0 
 0 
 0 
 0 
 0 
 0 
 0 
 0 
 0 
 0 
 0 
 0 
 0 
 0 
 0 
 0 
 0 
 0 
 0 
 0 
 0 
 0 
 0 
 0 
 0 
 0 
 0 
 0 
 0 
 0 
 0 
 0 
 0 
 0 
 0 
 0 
 0 
 0 
 0 
 0 
 0 
 0 
 0 
 0 
 0 
 0 
 0 
 0 
 0 
 0 
 0 
 0 
 0 
 0 
 0 
 0 
 0 
 0 
 0 
 0 
 0 
 0 
 0 
 0 
 0 
 0 
 0 
 0 
 0 
 0 
 0 
 0 
 0 
 0 
 0 
 0 
 0 
 0 
 0 
 0 
 0 
 0 
 0 
 0 
 0 
 0 
 0 
 0 
 0 
 0 
 0 
 0 
 0 
 0 
 0 
 0 
 0 
 0 
 0 
 0 
 0 
 0 
 0 
 0 
 0 
 0 
 0 
 0 
 0 
 0 
 0 
 0 
 0 
 0 
 0 
 0 
 0 
 0 
 0 
 0 
 0 
 0 
 0 
 0 
 0 
 0 
 0 
 0 
 0 
 0 
 0 
 0 
 0 
 0 
 0 
 0 
 0 
 0 
 0 
 0 
 0 
 0 
 0 
 0 
 0 
 0 
 0 
 0 
 0 
 0 
 0 
 0 
 0 
 0 
 0 
 0 
 0 
 0 
 0 
 0 
 0 
 0 
 0 
 0 
 0 
 0 
 0 
 0 
 0 
 0 
 0 
 0 
 0 
 0 
 0 
 0 
 0 
 0 
 0 
 0 
 0 
 0 
 0 
 0 
 0 
 0 
 0 
 0 
 0 
 0 
 0 
 0 
 0 
 0 
 0 
 0 
 0 
 0 
 0 
 0 
 0 
 0 
 0 
 0 
 0 
 0 
 0 
 0 
 0 
 0 
 0 
 0 
 0 
 0 
 0 
 0 
 0 
 0 
 0 
 0 
 0 
 0 
 0 
 0 
 0 
 0 
 0 
 0 
 0 
 0 
 0 
 0 
 0 
 0 
 0 
 0 
 0 
 0 
 0 
 0 
 0 
 0 
 0 
 0 
 0 
 0 
 0 
 0 
 0 
 0 
 0 
 0 
 0 
 0 
 0 
 0 
 0 
 0 
 0 
 0 
 0 
 0 
 0 
 0 
 0 
 0 
 0 
 0 
 0 
 0 
 0 
 0 
 0 
 0 
 0 
 0 
 0 
 0 
 0 
 0 
 0 
 0 
 0 
 0 
 0 
 0 
 0 
 0 
 0 
 0 
 0 
 0 
 0 
 0 
 0 
 0 
 0 
 0 
 0 
 0 
 0 
 0 
 0 
 0 
 0 
 0 
 0 
 0 
 0 
 0 
 0 
 0 
 0 
 0 
 0 
 0 
 0 
 0 
 0 
 0 
 0 
 0 
 0 
 0 
 0 
 0 
 0 
 0 
 0 
 0 
 0 
 0 
 0 
 0 
 0 
 0 
 0 
 0 
 0 
 0 
 0 
 0 
 0 
 0 
 0 
 0 
 0 
 0 
 0 
 0 
 0 
 0 
 0 
 0 
 0 
 0 
 0 
 0 
 0 
 0 
 0 
 0 
 0 
 0 
 0 
 0 
 0 
 0 
 0 
 0 
 0 
 0 
 0 
 0 
 0 
 0 
 0 
 0 
 0 
 0 
 0 
 0 
 0 
 0 
 0 
 0 
 0 
 0 
 0 
 0 
 0 
 0 
 0 
 0 
 0 
 0 
 0 
 0 
 0 
 0 
 0 
 0 
 0 
 
 
 89 
 0 
 1 
 0 
 0 
 0 
 0 
 0 
 0 
 0 
 0 
 0 
 0 
 0 
 0 
 0 
 0 
 0 
 0 
 0 
 0 
 0 
 0 
 0 
 0 
 0 
 0 
 0 
 0 
 0 
 0 
 0 
 0 
 0 
 0 
 0 
 0 
 0 
 0 
 0 
 0 
 0 
 0 
 0 
 0 
 0 
 0 
 0 
 0 
 0 
 0 
 0 
 0 
 0 
 0 
 0 
 0 
 0 
 0 
 0 
 0 
 0 
 0 
 0 
 0 
 0 
 0 
 0 
 0 
 0 
 0 
 0 
 0 
 0 
 0 
 0 
 0 
 0 
 0 
 0 
 0 
 0 
 0 
 0 
 0 
 0 
 0 
 0 
 0 
 0 
 58 
 0 
 0 
 0 
 0 
 0 
 0 
 0 
 0 
 0 
 0 
 0 
 0 
 0 
 0 
 0 
 4 
 0 
 0 
 0 
 0 
 0 
 0 
 0 
 0 
 0 
 0 
 0 
 0 
 0 
 0 
 0 
 0 
 0 
 0 
 0 
 0 
 0 
 0 
 0 
 0 
 0 
 0 
 0 
 0 
 0 
 0 
 0 
 0 
 0 
 0 
 0 
 0 
 0 
 0 
 0 
 0 
 0 
 0 
 0 
 0 
 0 
 0 
 0 
 0 
 0 
 0 
 0 
 0 
 0 
 0 
 0 
 0 
 0 
 0 
 0 
 0 
 0 
 0 
 0 
 0 
 0 
 0 
 0 
 0 
 0 
 0 
 0 
 0 
 0 
 0 
 0 
 0 
 0 
 0 
 0 
 0 
 0 
 0 
 0 
 0 
 0 
 0 
 0 
 0 
 0 
 0 
 0 
 0 
 0 
 0 
 0 
 0 
 0 
 0 
 0 
 0 
 0 
 0 
 0 
 0 
 0 
 0 
 0 
 0 
 0 
 0 
 0 
 0 
 0 
 0 
 0 
 0 
 0 
 0 
 0 
 0 
 0 
 0 
 0 
 0 
 0 
 0 
 0 
 0 
 0 
 0 
 0 
 0 
 0 
 0 
 0 
 0 
 0 
 0 
 0 
 0 
 0 
 0 
 0 
 0 
 0 
 0 
 0 
 0 
 0 
 0 
 0 
 0 
 0 
 0 
 0 
 0 
 0 
 0 
 0 
 0 
 0 
 0 
 0 
 0 
 0 
 0 
 0 
 0 
 0 
 0 
 0 
 0 
 0 
 0 
 0 
 0 
 0 
 0 
 0 
 0 
 0 
 0 
 0 
 0 
 0 
 0 
 0 
 0 
 0 
 0 
 0 
 0 
 0 
 0 
 0 
 0 
 0 
 0 
 0 
 0 
 0 
 0 
 0 
 0 
 0 
 0 
 0 
 0 
 0 
 0 
 0 
 0 
 0 
 0 
 0 
 0 
 0 
 0 
 0 
 0 
 0 
 0 
 0 
 0 
 0 
 0 
 0 
 0 
 0 
 0 
 0 
 0 
 0 
 0 
 0 
 0 
 0 
 0 
 0 
 0 
 0 
 0 
 0 
 0 
 0 
 0 
 0 
 0 
 0 
 0 
 0 
 0 
 0 
 0 
 0 
 0 
 0 
 0 
 0 
 0 
 0 
 0 
 0 
 0 
 0 
 0 
 0 
 0 
 0 
 0 
 0 
 0 
 0 
 0 
 0 
 0 
 0 
 0 
 0 
 0 
 0 
 0 
 0 
 0 
 0 
 0 
 0 
 0 
 0 
 0 
 0 
 0 
 0 
 0 
 0 
 0 
 0 
 0 
 0 
 0 
 0 
 0 
 0 
 0 
 0 
 0 
 0 
 0 
 0 
 0 
 0 
 0 
 0 
 0 
 0 
 0 
 0 
 0 
 0 
 0 
 0 
 0 
 0 
 0 
 0 
 0 
 0 
 0 
 0 
 0 
 0 
 0 
 0 
 0 
 0 
 0 
 0 
 0 
 0 
 0 
 0 
 0 
 0 
 0 
 0 
 0 
 0 
 0 
 0 
 0 
 0 
 0 
 0 
 0 
 0 
 0 
 0 
 0 
 0 
 0 
 0 
 0 
 0 
 0 
 0 
 0 
 0 
 0 
 0 
 0 
 0 
 0 
 0 
 0 
 0 
 0 
 0 
 0 
 0 
 0 
 0 
 0 
 0 
 0 
 0 
 0 
 0 
 0 
 0 
 0 
 0 
 0 
 0 
 0 
 0 
 0 
 0 
 0 
 0 
 0 
 0 
 0 
 0 
 0 
 0 
 0 
 0 
 0 
 0 
 0 
 0 
 0 
 0 
 0 
 0 
 0 
 0 
 0 
 0 
 0 
 0 
 0 
 0 
 0 
 0 
 0 
 0 
 0 
 0 
 0 
 0 
 0 
 0 
 0 
 0 
 0 
 0 
 0 
 0 
 0 
 0 
 0 
 0 
 0 
 0 
 0 
 0 
 0 
 0 
 0 
 0 
 0 
 0 
 0 
 0 
 0 
 0 
 0 
 0 
 0 
 0 
 0 
 0 
 0 
 0 
 0 
 0 
 0 
 0 
 0 
 0 
 0 
 0 
 0 
 0 
 0 
 0 
 0 
 0 
 0 
 0 
 0 
 0 
 0 
 0 
 0 
 0 
 0 
 0 
 0 
 0 
 0 
 0 
 0 
 0 
 0 
 0 
 0 
 0 
 0 
 0 
 0 
 0 
 0 
 0 
 0 
 0 
 0 
 0 
 0 
 0 
 0 
 0 
 0 
 0 
 0 
 0 
 0 
 0 
 0 
 0 
 0 
 0 
 0 
 0 
 0 
 0 
 0 
 0 
 0 
 0 
 0 
 0 
 0 
 0 
 0 
 0 
 0 
 0 
 0 
 0 
 0 
 0 
 0 
 0 
 0 
 0 
 0 
 0 
 0 
 0 
 0 
 0 
 0 
 0 
 0 
 0 
 0 
 0 
 0 
 0 
 0 
 0 
 0 
 0 
 0 
 0 
 0 
 0 
 0 
 0 
 0 
 0 
 0 
 0 
 0 
 0 
 0 
 0 
 0 
 0 
 0 
 0 
 0 
 0 
 0 
 0 
 0 
 0 
 0 
 0 
 0 
 0 
 0 
 0 
 0 
 0 
 0 
 0 
 0 
 0 
 0 
 0 
 0 
 0 
 0 
 0 
 0 
 0 
 0 
 0 
 0 
 0 
 0 
 0 
 0 
 0 
 0 
 0 
 0 
 0 
 0 
 0 
 0 
 0 
 0 
 0 
 0 
 0 
 0 
 0 
 0 
 0 
 0 
 0 
 0 
 0 
 0 
 0 
 0 
 0 
 0 
 0 
 0 
 0 
 0 
 0 
 0 
 0 
 0 
 0 
 0 
 0 
 0 
 0 
 0 
 0 
 0 
 0 
 0 
 0 
 0 
 0 
 0 
 0 
 0 
 0 
 0 
 0 
 0 
 0 
 0 
 0 
 0 
 0 
 0 
 0 
 0 
 0 
 0 
 0 
 0 
 0 
 0 
 0 
 0 
 0 
 0 
 0 
 0 
 0 
 0 
 0 
 0 
 0 
 0 
 0 
 0 
 0 
 0 
 0 
 0 
 0 
 0 
 0 
 0 
 0 
 0 
 0 
 0 
 0 
 0 
 0 
 0 
 0 
 0 
 0 
 0 
 0 
 0 
 
 
 90 
 0 
 0 
 0 
 0 
 0 
 0 
 0 
 0 
 0 
 0 
 0 
 0 
 0 
 0 
 0 
 0 
 0 
 0 
 0 
 0 
 0 
 0 
 0 
 0 
 0 
 0 
 0 
 0 
 0 
 0 
 0 
 0 
 0 
 0 
 0 
 0 
 0 
 0 
 0 
 0 
 0 
 0 
 0 
 0 
 0 
 0 
 0 
 0 
 0 
 0 
 0 
 0 
 0 
 0 
 0 
 0 
 0 
 0 
 0 
 0 
 0 
 0 
 0 
 0 
 0 
 0 
 0 
 0 
 0 
 0 
 0 
 0 
 0 
 0 
 0 
 0 
 0 
 0 
 0 
 0 
 0 
 0 
 0 
 0 
 0 
 0 
 0 
 0 
 0 
 0 
 3 
 0 
 0 
 0 
 0 
 0 
 0 
 0 
 0 
 0 
 0 
 0 
 0 
 0 
 0 
 0 
 0 
 0 
 0 
 0 
 0 
 0 
 0 
 0 
 0 
 0 
 0 
 0 
 0 
 0 
 0 
 0 
 0 
 0 
 0 
 0 
 0 
 0 
 0 
 0 
 0 
 0 
 0 
 0 
 0 
 0 
 0 
 0 
 0 
 0 
 0 
 0 
 0 
 0 
 0 
 0 
 0 
 0 
 0 
 0 
 0 
 0 
 0 
 0 
 0 
 0 
 0 
 0 
 0 
 0 
 0 
 0 
 0 
 0 
 0 
 0 
 0 
 0 
 0 
 0 
 0 
 0 
 0 
 0 
 0 
 0 
 0 
 0 
 0 
 0 
 0 
 0 
 0 
 0 
 0 
 0 
 0 
 0 
 0 
 0 
 0 
 0 
 0 
 0 
 0 
 0 
 0 
 0 
 0 
 0 
 0 
 0 
 0 
 0 
 0 
 0 
 0 
 0 
 0 
 0 
 0 
 0 
 0 
 0 
 0 
 0 
 0 
 0 
 0 
 0 
 0 
 0 
 0 
 0 
 0 
 0 
 0 
 0 
 0 
 0 
 0 
 0 
 0 
 0 
 0 
 0 
 0 
 0 
 0 
 0 
 0 
 0 
 0 
 0 
 0 
 0 
 0 
 0 
 0 
 0 
 0 
 0 
 0 
 0 
 0 
 0 
 0 
 0 
 0 
 0 
 0 
 0 
 0 
 0 
 0 
 0 
 0 
 0 
 0 
 0 
 0 
 0 
 0 
 0 
 0 
 0 
 0 
 0 
 0 
 0 
 0 
 0 
 0 
 0 
 0 
 0 
 0 
 0 
 0 
 0 
 0 
 0 
 0 
 0 
 0 
 0 
 0 
 0 
 0 
 0 
 0 
 0 
 0 
 0 
 0 
 0 
 0 
 0 
 0 
 0 
 0 
 0 
 0 
 0 
 0 
 0 
 0 
 0 
 0 
 0 
 0 
 0 
 0 
 0 
 0 
 0 
 0 
 0 
 0 
 0 
 0 
 0 
 0 
 0 
 0 
 0 
 0 
 0 
 0 
 0 
 0 
 0 
 0 
 0 
 0 
 0 
 0 
 0 
 0 
 0 
 0 
 0 
 0 
 0 
 0 
 0 
 0 
 0 
 0 
 0 
 0 
 0 
 0 
 0 
 0 
 0 
 0 
 0 
 0 
 0 
 0 
 0 
 0 
 0 
 0 
 0 
 0 
 0 
 0 
 0 
 0 
 0 
 0 
 0 
 0 
 0 
 0 
 0 
 0 
 0 
 0 
 0 
 0 
 0 
 0 
 0 
 0 
 0 
 0 
 0 
 0 
 0 
 0 
 0 
 0 
 0 
 0 
 0 
 0 
 0 
 0 
 0 
 0 
 0 
 0 
 0 
 0 
 0 
 0 
 0 
 0 
 0 
 0 
 0 
 0 
 0 
 0 
 0 
 0 
 0 
 0 
 0 
 0 
 0 
 0 
 0 
 0 
 0 
 0 
 0 
 0 
 0 
 0 
 0 
 0 
 0 
 0 
 0 
 0 
 0 
 0 
 0 
 0 
 0 
 0 
 0 
 0 
 0 
 0 
 0 
 0 
 0 
 0 
 0 
 0 
 0 
 0 
 0 
 0 
 0 
 0 
 0 
 0 
 0 
 0 
 0 
 0 
 0 
 0 
 0 
 0 
 0 
 0 
 0 
 0 
 0 
 0 
 0 
 0 
 0 
 0 
 0 
 0 
 0 
 0 
 0 
 0 
 0 
 0 
 0 
 0 
 0 
 0 
 0 
 0 
 0 
 0 
 0 
 0 
 0 
 0 
 0 
 0 
 0 
 0 
 0 
 0 
 0 
 0 
 0 
 0 
 0 
 0 
 0 
 0 
 0 
 0 
 0 
 0 
 0 
 0 
 0 
 0 
 0 
 0 
 0 
 0 
 0 
 0 
 0 
 0 
 0 
 0 
 0 
 0 
 0 
 0 
 0 
 0 
 0 
 0 
 0 
 0 
 0 
 0 
 0 
 0 
 0 
 0 
 0 
 0 
 0 
 0 
 0 
 0 
 0 
 0 
 0 
 0 
 0 
 0 
 0 
 0 
 0 
 0 
 0 
 0 
 0 
 0 
 0 
 0 
 0 
 0 
 0 
 0 
 0 
 0 
 0 
 0 
 0 
 0 
 0 
 0 
 0 
 0 
 0 
 0 
 0 
 0 
 0 
 0 
 0 
 0 
 0 
 0 
 0 
 0 
 0 
 0 
 0 
 0 
 0 
 0 
 0 
 0 
 0 
 0 
 0 
 0 
 0 
 0 
 0 
 0 
 0 
 0 
 0 
 0 
 0 
 0 
 0 
 0 
 0 
 0 
 0 
 0 
 0 
 0 
 0 
 0 
 0 
 0 
 0 
 0 
 0 
 0 
 0 
 0 
 0 
 0 
 0 
 0 
 0 
 0 
 0 
 0 
 0 
 0 
 0 
 0 
 0 
 0 
 0 
 0 
 0 
 0 
 0 
 0 
 0 
 0 
 0 
 0 
 0 
 0 
 0 
 0 
 0 
 0 
 0 
 0 
 0 
 0 
 0 
 0 
 0 
 0 
 0 
 0 
 0 
 0 
 0 
 0 
 0 
 0 
 0 
 0 
 0 
 0 
 0 
 0 
 0 
 0 
 0 
 0 
 0 
 0 
 0 
 0 
 0 
 0 
 0 
 0 
 0 
 0 
 0 
 0 
 0 
 0 
 0 
 0 
 0 
 0 
 0 
 0 
 0 
 0 
 0 
 0 
 0 
 0 
 0 
 0 
 0 
 0 
 0 
 0 
 0 
 0 
 0 
 0 
 0 
 0 
 0 
 0 
 0 
 0 
 0 
 0 
 0 
 0 
 0 
 0 
 0 
 0 
 0 
 0 
 0 
 0 
 0 
 0 
 0 
 0 
 0 
 0 
 0 
 0 
 0 
 0 
 0 
 0 
 0 
 0 
 0 
 0 
 0 
 0 
 0 
 0 
 0 
 0 
 0 
 0 
 0 
 0 
 0 
 0 
 0 
 0 
 0 
 0 
 0 
 0 
 0 
 0 
 0 
 0 
 0 
 0 
 0 
 0 
 0 
 0 
 0 
 0 
 0 
 0 
 0 
 0 
 0 
 0 
 0 
 0 
 0 
 0 
 0 
 0 
 0 
 0 
 0 
 0 
 0 
 0 
 0 
 0 
 0 
 0 
 0 
 
 
 91 
 0 
 0 
 0 
 0 
 0 
 0 
 0 
 0 
 0 
 0 
 0 
 0 
 0 
 0 
 0 
 0 
 0 
 0 
 0 
 0 
 0 
 0 
 0 
 0 
 0 
 0 
 0 
 0 
 0 
 0 
 0 
 0 
 0 
 0 
 0 
 0 
 0 
 0 
 0 
 0 
 0 
 0 
 0 
 0 
 0 
 0 
 0 
 0 
 0 
 0 
 0 
 0 
 0 
 0 
 0 
 0 
 0 
 0 
 0 
 0 
 0 
 0 
 0 
 0 
 0 
 0 
 0 
 0 
 0 
 0 
 0 
 0 
 0 
 0 
 0 
 0 
 0 
 0 
 0 
 0 
 0 
 0 
 0 
 0 
 0 
 0 
 0 
 0 
 0 
 0 
 0 
 9 
 0 
 0 
 0 
 0 
 0 
 0 
 0 
 0 
 0 
 0 
 0 
 0 
 0 
 0 
 0 
 0 
 0 
 0 
 0 
 0 
 0 
 0 
 0 
 0 
 0 
 0 
 0 
 0 
 0 
 0 
 0 
 0 
 0 
 0 
 0 
 0 
 0 
 0 
 0 
 0 
 0 
 0 
 0 
 0 
 0 
 0 
 0 
 0 
 0 
 0 
 0 
 0 
 0 
 0 
 0 
 0 
 0 
 0 
 0 
 0 
 0 
 0 
 0 
 0 
 0 
 0 
 0 
 0 
 0 
 0 
 0 
 0 
 0 
 0 
 0 
 0 
 0 
 0 
 0 
 0 
 0 
 0 
 0 
 0 
 0 
 0 
 0 
 0 
 0 
 0 
 0 
 0 
 0 
 0 
 0 
 0 
 0 
 0 
 0 
 0 
 0 
 0 
 0 
 0 
 0 
 0 
 0 
 0 
 0 
 0 
 0 
 0 
 0 
 0 
 0 
 0 
 0 
 0 
 0 
 0 
 0 
 0 
 0 
 0 
 0 
 0 
 0 
 0 
 0 
 0 
 0 
 0 
 0 
 0 
 0 
 0 
 0 
 0 
 0 
 0 
 0 
 0 
 0 
 0 
 0 
 0 
 0 
 0 
 0 
 0 
 0 
 0 
 0 
 0 
 0 
 0 
 0 
 0 
 0 
 0 
 0 
 0 
 0 
 0 
 0 
 0 
 0 
 0 
 0 
 0 
 0 
 0 
 0 
 0 
 0 
 0 
 0 
 0 
 0 
 0 
 0 
 0 
 0 
 0 
 0 
 0 
 0 
 0 
 0 
 0 
 0 
 0 
 0 
 0 
 0 
 0 
 0 
 0 
 0 
 0 
 0 
 0 
 0 
 0 
 0 
 0 
 0 
 0 
 0 
 0 
 0 
 0 
 0 
 0 
 0 
 0 
 0 
 0 
 0 
 0 
 0 
 0 
 0 
 0 
 0 
 0 
 0 
 0 
 0 
 0 
 0 
 0 
 0 
 0 
 0 
 0 
 0 
 0 
 0 
 0 
 0 
 0 
 0 
 0 
 0 
 0 
 0 
 0 
 0 
 0 
 0 
 0 
 0 
 0 
 0 
 0 
 0 
 0 
 0 
 0 
 0 
 0 
 0 
 0 
 0 
 0 
 0 
 0 
 0 
 0 
 0 
 0 
 0 
 0 
 0 
 0 
 0 
 0 
 0 
 0 
 0 
 0 
 0 
 0 
 0 
 0 
 0 
 0 
 0 
 0 
 0 
 0 
 0 
 0 
 0 
 0 
 0 
 0 
 0 
 0 
 0 
 0 
 0 
 0 
 0 
 0 
 0 
 0 
 0 
 0 
 0 
 0 
 0 
 0 
 0 
 0 
 0 
 0 
 0 
 0 
 0 
 0 
 0 
 0 
 0 
 0 
 0 
 0 
 0 
 0 
 0 
 0 
 0 
 0 
 0 
 0 
 0 
 0 
 0 
 0 
 0 
 0 
 0 
 0 
 0 
 0 
 0 
 0 
 0 
 0 
 0 
 0 
 0 
 0 
 0 
 0 
 0 
 0 
 0 
 0 
 0 
 0 
 0 
 0 
 0 
 0 
 0 
 0 
 0 
 0 
 0 
 0 
 0 
 0 
 0 
 0 
 0 
 0 
 0 
 0 
 0 
 0 
 0 
 0 
 0 
 0 
 0 
 0 
 0 
 0 
 0 
 0 
 0 
 0 
 0 
 0 
 0 
 0 
 0 
 0 
 0 
 0 
 0 
 0 
 0 
 0 
 0 
 0 
 0 
 0 
 0 
 0 
 0 
 0 
 0 
 0 
 0 
 0 
 0 
 0 
 0 
 0 
 0 
 0 
 0 
 0 
 0 
 0 
 0 
 0 
 0 
 0 
 0 
 0 
 0 
 0 
 0 
 0 
 0 
 0 
 0 
 0 
 0 
 0 
 0 
 0 
 0 
 0 
 0 
 0 
 0 
 0 
 0 
 0 
 0 
 0 
 0 
 0 
 0 
 0 
 0 
 0 
 0 
 0 
 0 
 0 
 0 
 0 
 0 
 0 
 0 
 0 
 0 
 0 
 0 
 0 
 0 
 0 
 0 
 0 
 0 
 0 
 0 
 0 
 0 
 0 
 0 
 0 
 0 
 0 
 0 
 0 
 0 
 0 
 0 
 0 
 0 
 0 
 0 
 0 
 0 
 0 
 0 
 0 
 0 
 0 
 0 
 0 
 0 
 0 
 0 
 0 
 0 
 0 
 0 
 0 
 0 
 0 
 0 
 0 
 0 
 0 
 0 
 0 
 0 
 0 
 0 
 0 
 0 
 0 
 0 
 0 
 0 
 0 
 0 
 0 
 0 
 0 
 0 
 0 
 0 
 0 
 0 
 0 
 0 
 0 
 0 
 0 
 0 
 0 
 0 
 0 
 0 
 0 
 0 
 0 
 0 
 0 
 0 
 0 
 0 
 0 
 0 
 0 
 0 
 0 
 0 
 0 
 0 
 0 
 0 
 0 
 0 
 0 
 0 
 0 
 0 
 0 
 0 
 0 
 0 
 0 
 0 
 0 
 0 
 0 
 0 
 0 
 0 
 0 
 0 
 0 
 0 
 0 
 0 
 0 
 0 
 0 
 0 
 0 
 0 
 0 
 0 
 0 
 0 
 0 
 0 
 0 
 0 
 0 
 0 
 0 
 0 
 0 
 0 
 0 
 0 
 0 
 0 
 0 
 0 
 0 
 0 
 0 
 0 
 0 
 0 
 0 
 0 
 0 
 0 
 0 
 0 
 0 
 0 
 0 
 0 
 0 
 0 
 0 
 0 
 0 
 0 
 0 
 0 
 0 
 0 
 0 
 0 
 0 
 0 
 0 
 0 
 0 
 0 
 0 
 0 
 0 
 0 
 0 
 0 
 0 
 0 
 0 
 0 
 0 
 0 
 0 
 0 
 0 
 0 
 0 
 0 
 0 
 0 
 0 
 0 
 0 
 0 
 0 
 0 
 0 
 0 
 0 
 0 
 0 
 0 
 0 
 0 
 0 
 0 
 0 
 0 
 0 
 0 
 0 
 0 
 0 
 0 
 0 
 0 
 0 
 0 
 0 
 0 
 0 
 0 
 0 
 0 
 0 
 0 
 0 
 0 
 0 
 0 
 0 
 0 
 0 
 0 
 0 
 0 
 0 
 0 
 0 
 0 
 0 
 0 
 0 
 0 
 0 
 0 
 0 
 0 
 0 
 
 
 92 
 0 
 0 
 0 
 0 
 0 
 0 
 0 
 0 
 0 
 0 
 0 
 0 
 0 
 0 
 0 
 0 
 0 
 0 
 0 
 0 
 0 
 0 
 0 
 0 
 0 
 0 
 0 
 0 
 0 
 0 
 0 
 0 
 0 
 0 
 0 
 0 
 0 
 0 
 0 
 0 
 0 
 0 
 0 
 0 
 0 
 0 
 0 
 0 
 0 
 0 
 0 
 0 
 0 
 0 
 0 
 0 
 0 
 0 
 0 
 0 
 0 
 0 
 0 
 0 
 0 
 0 
 0 
 0 
 0 
 0 
 0 
 0 
 0 
 0 
 0 
 0 
 0 
 0 
 0 
 0 
 0 
 0 
 0 
 0 
 0 
 0 
 0 
 0 
 0 
 0 
 0 
 0 
 7 
 0 
 0 
 0 
 0 
 0 
 0 
 0 
 0 
 0 
 0 
 0 
 0 
 0 
 0 
 0 
 0 
 0 
 0 
 0 
 0 
 0 
 0 
 0 
 0 
 0 
 0 
 0 
 0 
 0 
 0 
 0 
 0 
 0 
 0 
 0 
 0 
 0 
 0 
 0 
 0 
 0 
 0 
 0 
 0 
 0 
 0 
 0 
 0 
 0 
 0 
 0 
 0 
 0 
 0 
 0 
 0 
 0 
 0 
 0 
 0 
 0 
 0 
 0 
 0 
 0 
 0 
 0 
 0 
 0 
 0 
 0 
 0 
 0 
 0 
 0 
 0 
 0 
 0 
 0 
 0 
 0 
 0 
 0 
 0 
 0 
 0 
 0 
 0 
 0 
 0 
 0 
 0 
 0 
 0 
 0 
 0 
 0 
 0 
 0 
 0 
 0 
 0 
 0 
 0 
 0 
 0 
 0 
 0 
 0 
 0 
 0 
 0 
 0 
 0 
 0 
 0 
 0 
 0 
 0 
 0 
 0 
 0 
 0 
 0 
 0 
 0 
 0 
 0 
 0 
 0 
 0 
 0 
 0 
 0 
 0 
 0 
 0 
 0 
 0 
 0 
 0 
 0 
 0 
 0 
 0 
 0 
 0 
 0 
 0 
 0 
 0 
 0 
 0 
 0 
 0 
 0 
 0 
 0 
 0 
 0 
 0 
 0 
 0 
 0 
 0 
 0 
 0 
 0 
 0 
 0 
 0 
 0 
 0 
 0 
 0 
 0 
 0 
 0 
 0 
 0 
 0 
 0 
 0 
 0 
 0 
 0 
 0 
 0 
 0 
 0 
 0 
 0 
 0 
 0 
 0 
 0 
 0 
 0 
 0 
 0 
 0 
 0 
 0 
 0 
 0 
 0 
 0 
 0 
 0 
 0 
 0 
 0 
 0 
 0 
 0 
 0 
 0 
 0 
 0 
 0 
 0 
 0 
 0 
 0 
 0 
 0 
 0 
 0 
 0 
 0 
 0 
 0 
 0 
 0 
 0 
 0 
 0 
 0 
 0 
 0 
 0 
 0 
 0 
 0 
 0 
 0 
 0 
 0 
 0 
 0 
 0 
 0 
 0 
 0 
 0 
 0 
 0 
 0 
 0 
 0 
 0 
 0 
 0 
 0 
 0 
 0 
 0 
 0 
 0 
 0 
 0 
 0 
 0 
 0 
 0 
 0 
 0 
 0 
 0 
 0 
 0 
 0 
 0 
 0 
 0 
 0 
 0 
 0 
 0 
 0 
 0 
 0 
 0 
 0 
 0 
 0 
 0 
 0 
 0 
 0 
 0 
 0 
 0 
 0 
 0 
 0 
 0 
 0 
 0 
 0 
 0 
 0 
 0 
 0 
 0 
 0 
 0 
 0 
 0 
 0 
 0 
 0 
 0 
 0 
 0 
 0 
 0 
 0 
 0 
 0 
 0 
 0 
 0 
 0 
 0 
 0 
 0 
 0 
 0 
 0 
 0 
 0 
 0 
 0 
 0 
 0 
 0 
 0 
 0 
 0 
 0 
 0 
 0 
 0 
 0 
 0 
 0 
 0 
 0 
 0 
 0 
 0 
 0 
 0 
 0 
 0 
 0 
 0 
 0 
 0 
 0 
 0 
 0 
 0 
 0 
 0 
 0 
 0 
 0 
 0 
 0 
 0 
 0 
 0 
 0 
 0 
 0 
 0 
 0 
 0 
 0 
 0 
 0 
 0 
 0 
 0 
 0 
 0 
 0 
 0 
 0 
 0 
 0 
 0 
 0 
 0 
 0 
 0 
 0 
 0 
 0 
 0 
 0 
 0 
 0 
 0 
 0 
 0 
 0 
 0 
 0 
 0 
 0 
 0 
 0 
 0 
 0 
 0 
 0 
 0 
 0 
 0 
 0 
 0 
 0 
 0 
 0 
 0 
 0 
 0 
 0 
 0 
 0 
 0 
 0 
 0 
 0 
 0 
 0 
 0 
 0 
 0 
 0 
 0 
 0 
 0 
 0 
 0 
 0 
 0 
 0 
 0 
 0 
 0 
 0 
 0 
 0 
 0 
 0 
 0 
 0 
 0 
 0 
 0 
 0 
 0 
 0 
 0 
 0 
 0 
 0 
 0 
 0 
 0 
 0 
 0 
 0 
 0 
 0 
 0 
 0 
 0 
 0 
 0 
 0 
 0 
 0 
 0 
 0 
 0 
 0 
 0 
 0 
 0 
 0 
 0 
 0 
 0 
 0 
 0 
 0 
 0 
 0 
 0 
 0 
 0 
 0 
 0 
 0 
 0 
 0 
 0 
 0 
 0 
 0 
 0 
 0 
 0 
 0 
 0 
 0 
 0 
 0 
 0 
 0 
 0 
 0 
 0 
 0 
 0 
 0 
 0 
 0 
 0 
 0 
 0 
 0 
 0 
 0 
 0 
 0 
 0 
 0 
 0 
 0 
 0 
 0 
 0 
 0 
 0 
 0 
 0 
 0 
 0 
 0 
 0 
 0 
 0 
 0 
 0 
 0 
 0 
 0 
 0 
 0 
 0 
 0 
 0 
 0 
 0 
 0 
 0 
 0 
 0 
 0 
 0 
 0 
 0 
 0 
 0 
 0 
 0 
 0 
 0 
 0 
 0 
 0 
 0 
 0 
 0 
 0 
 0 
 0 
 0 
 0 
 0 
 0 
 0 
 0 
 0 
 0 
 0 
 0 
 0 
 0 
 0 
 0 
 0 
 0 
 0 
 0 
 0 
 0 
 0 
 0 
 0 
 0 
 0 
 0 
 0 
 0 
 0 
 0 
 0 
 0 
 0 
 0 
 0 
 0 
 0 
 0 
 0 
 0 
 0 
 0 
 0 
 0 
 0 
 0 
 0 
 0 
 0 
 0 
 0 
 0 
 0 
 0 
 0 
 0 
 0 
 0 
 0 
 0 
 0 
 0 
 0 
 0 
 0 
 0 
 0 
 0 
 0 
 0 
 0 
 0 
 0 
 0 
 0 
 0 
 0 
 0 
 0 
 0 
 0 
 0 
 0 
 0 
 0 
 0 
 0 
 0 
 0 
 0 
 0 
 0 
 0 
 0 
 0 
 0 
 0 
 0 
 0 
 0 
 0 
 0 
 0 
 0 
 0 
 0 
 0 
 0 
 0 
 0 
 0 
 0 
 0 
 0 
 0 
 0 
 0 
 0 
 0 
 0 
 0 
 0 
 0 
 0 
 0 
 0 
 0 
 0 
 0 
 0 
 
 
 93 
 0 
 0 
 0 
 0 
 0 
 0 
 0 
 0 
 0 
 0 
 0 
 0 
 0 
 0 
 0 
 0 
 0 
 0 
 0 
 0 
 0 
 0 
 0 
 0 
 0 
 0 
 0 
 0 
 0 
 0 
 0 
 0 
 0 
 0 
 0 
 0 
 0 
 0 
 0 
 0 
 0 
 0 
 0 
 0 
 0 
 0 
 0 
 0 
 0 
 0 
 0 
 0 
 0 
 0 
 0 
 0 
 0 
 0 
 0 
 0 
 0 
 0 
 0 
 0 
 0 
 0 
 0 
 0 
 0 
 0 
 0 
 0 
 0 
 0 
 0 
 0 
 0 
 0 
 0 
 0 
 0 
 0 
 0 
 0 
 0 
 0 
 0 
 0 
 0 
 0 
 0 
 0 
 0 
 31 
 0 
 6 
 0 
 0 
 0 
 0 
 0 
 0 
 0 
 0 
 0 
 0 
 0 
 0 
 0 
 0 
 0 
 0 
 0 
 0 
 0 
 0 
 0 
 0 
 0 
 0 
 0 
 0 
 0 
 0 
 0 
 0 
 0 
 0 
 0 
 0 
 0 
 0 
 0 
 0 
 0 
 0 
 0 
 0 
 0 
 0 
 0 
 0 
 0 
 0 
 0 
 0 
 0 
 0 
 0 
 0 
 0 
 0 
 0 
 0 
 0 
 0 
 0 
 0 
 0 
 0 
 0 
 0 
 0 
 0 
 0 
 0 
 0 
 0 
 0 
 0 
 0 
 0 
 0 
 0 
 0 
 0 
 0 
 0 
 0 
 0 
 0 
 0 
 0 
 0 
 0 
 0 
 0 
 0 
 0 
 0 
 0 
 0 
 0 
 0 
 0 
 0 
 0 
 0 
 0 
 0 
 0 
 0 
 0 
 0 
 0 
 0 
 0 
 0 
 0 
 0 
 0 
 0 
 0 
 0 
 0 
 0 
 0 
 0 
 0 
 0 
 0 
 0 
 0 
 0 
 0 
 0 
 0 
 0 
 0 
 0 
 0 
 0 
 0 
 0 
 0 
 0 
 0 
 0 
 0 
 0 
 0 
 0 
 0 
 0 
 0 
 0 
 0 
 0 
 0 
 0 
 0 
 0 
 0 
 0 
 0 
 0 
 0 
 0 
 0 
 0 
 0 
 0 
 0 
 0 
 0 
 0 
 0 
 0 
 0 
 0 
 0 
 0 
 0 
 0 
 0 
 0 
 0 
 0 
 0 
 0 
 0 
 0 
 0 
 0 
 0 
 0 
 0 
 0 
 0 
 0 
 0 
 0 
 0 
 0 
 0 
 0 
 0 
 0 
 0 
 0 
 0 
 0 
 0 
 0 
 0 
 0 
 0 
 0 
 0 
 0 
 0 
 0 
 0 
 0 
 0 
 0 
 0 
 0 
 0 
 0 
 0 
 0 
 0 
 0 
 0 
 0 
 0 
 0 
 0 
 0 
 0 
 0 
 0 
 0 
 0 
 0 
 0 
 0 
 0 
 0 
 0 
 0 
 0 
 0 
 0 
 0 
 0 
 0 
 0 
 0 
 0 
 0 
 0 
 0 
 0 
 0 
 0 
 0 
 0 
 0 
 0 
 0 
 0 
 0 
 0 
 0 
 0 
 0 
 0 
 0 
 0 
 0 
 0 
 0 
 0 
 0 
 0 
 0 
 0 
 0 
 0 
 0 
 0 
 0 
 0 
 0 
 0 
 0 
 0 
 0 
 0 
 0 
 0 
 0 
 0 
 0 
 0 
 0 
 0 
 0 
 0 
 0 
 0 
 0 
 0 
 0 
 0 
 0 
 0 
 0 
 0 
 0 
 0 
 0 
 0 
 0 
 0 
 0 
 0 
 0 
 0 
 0 
 0 
 0 
 0 
 0 
 0 
 0 
 0 
 0 
 0 
 0 
 0 
 0 
 0 
 0 
 0 
 0 
 0 
 0 
 0 
 0 
 0 
 0 
 0 
 0 
 0 
 0 
 0 
 0 
 0 
 0 
 0 
 0 
 0 
 0 
 0 
 0 
 0 
 0 
 0 
 0 
 0 
 0 
 0 
 0 
 0 
 0 
 0 
 0 
 0 
 0 
 0 
 0 
 0 
 0 
 0 
 0 
 0 
 0 
 0 
 0 
 0 
 0 
 0 
 0 
 0 
 0 
 0 
 0 
 0 
 0 
 0 
 0 
 0 
 0 
 0 
 0 
 0 
 0 
 0 
 0 
 0 
 0 
 0 
 0 
 0 
 0 
 0 
 0 
 0 
 0 
 0 
 0 
 0 
 0 
 0 
 0 
 0 
 0 
 0 
 0 
 0 
 0 
 0 
 0 
 0 
 0 
 0 
 0 
 0 
 0 
 0 
 0 
 0 
 0 
 0 
 0 
 0 
 0 
 0 
 0 
 0 
 0 
 0 
 0 
 0 
 0 
 0 
 0 
 0 
 0 
 0 
 0 
 0 
 0 
 0 
 0 
 0 
 0 
 0 
 0 
 0 
 0 
 0 
 0 
 0 
 0 
 0 
 0 
 0 
 0 
 0 
 0 
 0 
 0 
 0 
 0 
 0 
 0 
 0 
 0 
 0 
 0 
 0 
 0 
 0 
 0 
 0 
 0 
 0 
 0 
 0 
 0 
 0 
 0 
 0 
 0 
 0 
 0 
 0 
 0 
 0 
 0 
 0 
 0 
 0 
 0 
 0 
 0 
 0 
 0 
 0 
 0 
 0 
 0 
 0 
 0 
 0 
 0 
 0 
 0 
 0 
 0 
 0 
 0 
 0 
 0 
 0 
 0 
 0 
 0 
 0 
 0 
 0 
 0 
 0 
 0 
 0 
 0 
 0 
 0 
 0 
 0 
 0 
 0 
 0 
 0 
 0 
 0 
 0 
 0 
 0 
 0 
 0 
 0 
 0 
 0 
 0 
 0 
 0 
 0 
 0 
 0 
 0 
 0 
 0 
 0 
 0 
 0 
 0 
 0 
 0 
 0 
 0 
 0 
 0 
 0 
 0 
 0 
 0 
 0 
 0 
 0 
 0 
 0 
 0 
 0 
 0 
 0 
 0 
 0 
 0 
 0 
 0 
 0 
 0 
 0 
 0 
 0 
 0 
 0 
 0 
 0 
 0 
 0 
 0 
 0 
 0 
 0 
 0 
 0 
 0 
 0 
 0 
 0 
 0 
 0 
 0 
 0 
 0 
 0 
 0 
 0 
 0 
 0 
 0 
 0 
 0 
 0 
 0 
 0 
 0 
 0 
 0 
 0 
 0 
 0 
 0 
 0 
 0 
 0 
 0 
 0 
 0 
 0 
 0 
 0 
 0 
 0 
 0 
 0 
 0 
 0 
 0 
 0 
 0 
 0 
 0 
 0 
 0 
 0 
 0 
 0 
 0 
 0 
 0 
 0 
 0 
 0 
 0 
 0 
 0 
 0 
 0 
 0 
 0 
 0 
 0 
 0 
 0 
 0 
 0 
 0 
 0 
 0 
 0 
 0 
 0 
 0 
 0 
 0 
 0 
 0 
 0 
 0 
 0 
 0 
 0 
 0 
 0 
 0 
 0 
 0 
 0 
 0 
 0 
 0 
 0 
 0 
 0 
 0 
 0 
 0 
 0 
 0 
 0 
 0 
 0 
 0 
 0 
 0 
 0 
 0 
 0 
 0 
 
 
 94 
 0 
 0 
 0 
 0 
 0 
 0 
 0 
 0 
 0 
 0 
 0 
 0 
 0 
 0 
 0 
 0 
 0 
 0 
 0 
 0 
 0 
 0 
 0 
 0 
 0 
 0 
 0 
 0 
 0 
 0 
 0 
 0 
 0 
 0 
 0 
 0 
 0 
 0 
 0 
 0 
 0 
 0 
 0 
 0 
 0 
 0 
 0 
 0 
 0 
 0 
 0 
 0 
 0 
 0 
 0 
 0 
 0 
 0 
 0 
 0 
 0 
 0 
 0 
 0 
 0 
 0 
 0 
 0 
 0 
 0 
 0 
 0 
 0 
 0 
 0 
 0 
 0 
 0 
 0 
 0 
 0 
 0 
 0 
 0 
 0 
 0 
 0 
 0 
 0 
 0 
 0 
 0 
 0 
 0 
 34 
 0 
 0 
 0 
 0 
 0 
 0 
 0 
 0 
 0 
 0 
 0 
 0 
 0 
 0 
 0 
 0 
 0 
 0 
 0 
 0 
 0 
 0 
 0 
 0 
 0 
 0 
 0 
 0 
 0 
 0 
 0 
 0 
 0 
 0 
 0 
 0 
 0 
 0 
 0 
 0 
 0 
 0 
 0 
 0 
 0 
 0 
 0 
 0 
 0 
 0 
 0 
 0 
 0 
 0 
 0 
 0 
 0 
 0 
 0 
 0 
 0 
 0 
 0 
 0 
 0 
 0 
 0 
 0 
 0 
 0 
 0 
 0 
 0 
 0 
 0 
 0 
 0 
 0 
 0 
 0 
 0 
 0 
 0 
 0 
 0 
 0 
 0 
 0 
 0 
 0 
 0 
 0 
 0 
 0 
 0 
 0 
 0 
 0 
 0 
 0 
 0 
 0 
 0 
 0 
 0 
 0 
 0 
 0 
 0 
 0 
 0 
 0 
 0 
 0 
 0 
 0 
 0 
 0 
 0 
 0 
 0 
 0 
 0 
 0 
 0 
 0 
 0 
 0 
 0 
 0 
 0 
 0 
 0 
 0 
 0 
 0 
 0 
 0 
 0 
 0 
 0 
 0 
 0 
 0 
 0 
 0 
 0 
 0 
 0 
 0 
 0 
 0 
 0 
 0 
 0 
 0 
 0 
 0 
 0 
 0 
 0 
 0 
 0 
 0 
 0 
 0 
 0 
 0 
 0 
 0 
 0 
 0 
 0 
 0 
 0 
 0 
 0 
 0 
 0 
 0 
 0 
 0 
 0 
 0 
 0 
 0 
 0 
 0 
 0 
 0 
 0 
 0 
 0 
 0 
 0 
 0 
 0 
 0 
 0 
 0 
 0 
 0 
 0 
 0 
 0 
 0 
 0 
 0 
 0 
 0 
 0 
 0 
 0 
 0 
 0 
 0 
 0 
 0 
 0 
 0 
 0 
 0 
 0 
 0 
 0 
 0 
 0 
 0 
 0 
 0 
 0 
 0 
 0 
 0 
 0 
 0 
 0 
 0 
 0 
 0 
 0 
 0 
 0 
 0 
 0 
 0 
 0 
 0 
 0 
 0 
 0 
 0 
 0 
 0 
 0 
 0 
 0 
 0 
 0 
 0 
 0 
 0 
 0 
 0 
 0 
 0 
 0 
 0 
 0 
 0 
 0 
 0 
 0 
 0 
 0 
 0 
 0 
 0 
 0 
 0 
 0 
 0 
 0 
 0 
 0 
 0 
 0 
 0 
 0 
 0 
 0 
 0 
 0 
 0 
 0 
 0 
 0 
 0 
 0 
 0 
 0 
 0 
 0 
 0 
 0 
 0 
 0 
 0 
 0 
 0 
 0 
 0 
 0 
 0 
 0 
 0 
 0 
 0 
 0 
 0 
 0 
 0 
 0 
 0 
 0 
 0 
 0 
 0 
 0 
 0 
 0 
 0 
 0 
 0 
 0 
 0 
 0 
 0 
 0 
 0 
 0 
 0 
 0 
 0 
 0 
 0 
 0 
 0 
 0 
 0 
 0 
 0 
 0 
 0 
 0 
 0 
 0 
 0 
 0 
 0 
 0 
 0 
 0 
 0 
 0 
 0 
 0 
 0 
 0 
 0 
 0 
 0 
 0 
 0 
 0 
 0 
 0 
 0 
 0 
 0 
 0 
 0 
 0 
 0 
 0 
 0 
 0 
 0 
 0 
 0 
 0 
 0 
 0 
 0 
 0 
 0 
 0 
 0 
 0 
 0 
 0 
 0 
 0 
 0 
 0 
 0 
 0 
 0 
 0 
 0 
 0 
 0 
 0 
 0 
 0 
 0 
 0 
 0 
 0 
 0 
 0 
 0 
 0 
 0 
 0 
 0 
 0 
 0 
 0 
 0 
 0 
 0 
 0 
 0 
 0 
 0 
 0 
 0 
 0 
 0 
 0 
 0 
 0 
 0 
 0 
 0 
 0 
 0 
 0 
 0 
 0 
 0 
 0 
 0 
 0 
 0 
 0 
 0 
 0 
 0 
 0 
 0 
 0 
 0 
 0 
 0 
 0 
 0 
 0 
 0 
 0 
 0 
 0 
 0 
 0 
 0 
 0 
 0 
 0 
 0 
 0 
 0 
 0 
 0 
 0 
 0 
 0 
 0 
 0 
 0 
 0 
 0 
 0 
 0 
 0 
 0 
 0 
 0 
 0 
 0 
 0 
 0 
 0 
 0 
 0 
 0 
 0 
 0 
 0 
 0 
 0 
 0 
 0 
 0 
 0 
 0 
 0 
 0 
 0 
 0 
 0 
 0 
 0 
 0 
 0 
 0 
 0 
 0 
 0 
 0 
 0 
 0 
 0 
 0 
 0 
 0 
 0 
 0 
 0 
 0 
 0 
 0 
 0 
 0 
 0 
 0 
 0 
 0 
 0 
 0 
 0 
 0 
 0 
 0 
 0 
 0 
 0 
 0 
 0 
 0 
 0 
 0 
 0 
 0 
 0 
 0 
 0 
 0 
 0 
 0 
 0 
 0 
 0 
 0 
 0 
 0 
 0 
 0 
 0 
 0 
 0 
 0 
 0 
 0 
 0 
 0 
 0 
 0 
 0 
 0 
 0 
 0 
 0 
 0 
 0 
 0 
 0 
 0 
 0 
 0 
 0 
 0 
 0 
 0 
 0 
 0 
 0 
 0 
 0 
 0 
 0 
 0 
 0 
 0 
 0 
 0 
 0 
 0 
 0 
 0 
 0 
 0 
 0 
 0 
 0 
 0 
 0 
 0 
 0 
 0 
 0 
 0 
 0 
 0 
 0 
 0 
 0 
 0 
 0 
 0 
 0 
 0 
 0 
 0 
 0 
 0 
 0 
 0 
 0 
 0 
 0 
 0 
 0 
 0 
 0 
 0 
 0 
 0 
 0 
 0 
 0 
 0 
 0 
 0 
 0 
 0 
 0 
 0 
 0 
 0 
 0 
 0 
 0 
 0 
 0 
 0 
 0 
 0 
 0 
 0 
 0 
 0 
 0 
 0 
 0 
 0 
 0 
 0 
 0 
 0 
 0 
 0 
 0 
 0 
 0 
 0 
 0 
 0 
 0 
 0 
 0 
 0 
 0 
 0 
 0 
 0 
 0 
 0 
 0 
 0 
 0 
 0 
 0 
 0 
 0 
 0 
 0 
 0 
 0 
 0 
 0 
 0 
 0 
 0 
 0 
 0 
 0 
 0 
 0 
 0 
 0 
 
 
 95 
 0 
 0 
 0 
 0 
 0 
 0 
 0 
 0 
 0 
 0 
 0 
 0 
 0 
 0 
 0 
 0 
 0 
 0 
 0 
 0 
 0 
 0 
 0 
 0 
 0 
 0 
 0 
 0 
 0 
 0 
 0 
 0 
 0 
 0 
 0 
 0 
 0 
 0 
 0 
 0 
 0 
 0 
 0 
 0 
 0 
 0 
 0 
 0 
 0 
 0 
 0 
 0 
 0 
 0 
 0 
 0 
 0 
 0 
 0 
 0 
 0 
 0 
 0 
 0 
 0 
 0 
 0 
 0 
 0 
 0 
 0 
 0 
 0 
 0 
 0 
 0 
 0 
 0 
 0 
 0 
 0 
 0 
 0 
 0 
 0 
 0 
 0 
 0 
 0 
 0 
 0 
 0 
 0 
 1 
 0 
 0 
 0 
 0 
 0 
 0 
 0 
 0 
 0 
 0 
 0 
 0 
 0 
 0 
 0 
 0 
 0 
 0 
 0 
 0 
 0 
 0 
 0 
 0 
 0 
 0 
 0 
 0 
 0 
 0 
 0 
 0 
 0 
 0 
 0 
 0 
 0 
 0 
 0 
 0 
 0 
 0 
 0 
 0 
 0 
 0 
 0 
 0 
 0 
 0 
 0 
 0 
 0 
 0 
 0 
 0 
 0 
 0 
 0 
 0 
 0 
 0 
 0 
 0 
 0 
 0 
 0 
 0 
 0 
 0 
 0 
 0 
 0 
 0 
 0 
 0 
 0 
 0 
 0 
 0 
 0 
 0 
 0 
 0 
 0 
 0 
 0 
 0 
 0 
 0 
 0 
 0 
 0 
 0 
 0 
 0 
 0 
 0 
 0 
 0 
 0 
 0 
 0 
 0 
 0 
 0 
 0 
 0 
 0 
 0 
 0 
 0 
 0 
 0 
 0 
 0 
 0 
 0 
 0 
 0 
 0 
 0 
 0 
 0 
 0 
 0 
 0 
 0 
 0 
 0 
 0 
 0 
 0 
 0 
 0 
 0 
 0 
 0 
 0 
 0 
 0 
 0 
 0 
 0 
 0 
 0 
 0 
 0 
 0 
 0 
 0 
 0 
 0 
 0 
 0 
 0 
 0 
 0 
 0 
 0 
 0 
 0 
 0 
 0 
 0 
 0 
 0 
 0 
 0 
 0 
 0 
 0 
 0 
 0 
 0 
 0 
 0 
 0 
 0 
 0 
 0 
 0 
 0 
 0 
 0 
 0 
 0 
 0 
 0 
 0 
 0 
 0 
 0 
 0 
 0 
 0 
 0 
 0 
 0 
 0 
 0 
 0 
 0 
 0 
 0 
 0 
 0 
 0 
 0 
 0 
 0 
 0 
 0 
 0 
 0 
 0 
 0 
 0 
 0 
 0 
 0 
 0 
 0 
 0 
 0 
 0 
 0 
 0 
 0 
 0 
 0 
 0 
 0 
 0 
 0 
 0 
 0 
 0 
 0 
 0 
 0 
 0 
 0 
 0 
 0 
 0 
 0 
 0 
 0 
 0 
 0 
 0 
 0 
 0 
 0 
 0 
 0 
 0 
 0 
 0 
 0 
 0 
 0 
 0 
 0 
 0 
 0 
 0 
 0 
 0 
 0 
 0 
 0 
 0 
 0 
 0 
 0 
 0 
 0 
 0 
 0 
 0 
 0 
 0 
 0 
 0 
 0 
 0 
 0 
 0 
 0 
 0 
 0 
 0 
 0 
 0 
 0 
 0 
 0 
 0 
 0 
 0 
 0 
 0 
 0 
 0 
 0 
 0 
 0 
 0 
 0 
 0 
 0 
 0 
 0 
 0 
 0 
 0 
 0 
 0 
 0 
 0 
 0 
 0 
 0 
 0 
 0 
 0 
 0 
 0 
 0 
 0 
 0 
 0 
 0 
 0 
 0 
 0 
 0 
 0 
 0 
 0 
 0 
 0 
 0 
 0 
 0 
 0 
 0 
 0 
 0 
 0 
 0 
 0 
 0 
 0 
 0 
 0 
 0 
 0 
 0 
 0 
 0 
 0 
 0 
 0 
 0 
 0 
 0 
 0 
 0 
 0 
 0 
 0 
 0 
 0 
 0 
 0 
 0 
 0 
 0 
 0 
 0 
 0 
 0 
 0 
 0 
 0 
 0 
 0 
 0 
 0 
 0 
 0 
 0 
 0 
 0 
 0 
 0 
 0 
 0 
 0 
 0 
 0 
 0 
 0 
 0 
 0 
 0 
 0 
 0 
 0 
 0 
 0 
 0 
 0 
 0 
 0 
 0 
 0 
 0 
 0 
 0 
 0 
 0 
 0 
 0 
 0 
 0 
 0 
 0 
 0 
 0 
 0 
 0 
 0 
 0 
 0 
 0 
 0 
 0 
 0 
 0 
 0 
 0 
 0 
 0 
 0 
 0 
 0 
 0 
 0 
 0 
 0 
 0 
 0 
 0 
 0 
 0 
 0 
 0 
 0 
 0 
 0 
 0 
 0 
 0 
 0 
 0 
 0 
 0 
 0 
 0 
 0 
 0 
 0 
 0 
 0 
 0 
 0 
 0 
 0 
 0 
 0 
 0 
 0 
 0 
 0 
 0 
 0 
 0 
 0 
 0 
 0 
 0 
 0 
 0 
 0 
 0 
 0 
 0 
 0 
 0 
 0 
 0 
 0 
 0 
 0 
 0 
 0 
 0 
 0 
 0 
 0 
 0 
 0 
 0 
 0 
 0 
 0 
 0 
 0 
 0 
 0 
 0 
 0 
 0 
 0 
 0 
 0 
 0 
 0 
 0 
 0 
 0 
 0 
 0 
 0 
 0 
 0 
 0 
 0 
 0 
 0 
 0 
 0 
 0 
 0 
 0 
 0 
 0 
 0 
 0 
 0 
 0 
 0 
 0 
 0 
 0 
 0 
 0 
 0 
 0 
 0 
 0 
 0 
 0 
 0 
 0 
 0 
 0 
 0 
 0 
 0 
 0 
 0 
 0 
 0 
 0 
 0 
 0 
 0 
 0 
 0 
 0 
 0 
 0 
 0 
 0 
 0 
 0 
 0 
 0 
 0 
 0 
 0 
 0 
 0 
 0 
 0 
 0 
 0 
 0 
 0 
 0 
 0 
 0 
 0 
 0 
 0 
 0 
 0 
 0 
 0 
 0 
 0 
 0 
 0 
 0 
 0 
 0 
 0 
 0 
 0 
 0 
 0 
 0 
 0 
 0 
 0 
 0 
 0 
 0 
 0 
 0 
 0 
 0 
 0 
 0 
 0 
 0 
 0 
 0 
 0 
 0 
 0 
 0 
 0 
 0 
 0 
 0 
 0 
 0 
 0 
 0 
 0 
 0 
 0 
 0 
 0 
 0 
 0 
 0 
 0 
 0 
 0 
 0 
 0 
 0 
 0 
 0 
 0 
 0 
 0 
 0 
 0 
 0 
 0 
 0 
 0 
 0 
 0 
 0 
 0 
 0 
 0 
 0 
 0 
 0 
 0 
 0 
 0 
 0 
 0 
 0 
 0 
 0 
 0 
 0 
 0 
 0 
 0 
 0 
 0 
 0 
 0 
 0 
 0 
 0 
 0 
 0 
 0 
 0 
 0 
 0 
 0 
 0 
 0 
 0 
 0 
 0 
 0 
 0 
 0 
 0 
 0 
 0 
 0 
 0 
 0 
 0 
 0 
 
 
 96 
 0 
 0 
 0 
 0 
 0 
 0 
 0 
 0 
 0 
 0 
 0 
 0 
 0 
 0 
 0 
 0 
 0 
 0 
 0 
 0 
 0 
 0 
 0 
 0 
 0 
 0 
 0 
 0 
 0 
 0 
 0 
 0 
 0 
 0 
 0 
 0 
 0 
 0 
 0 
 0 
 0 
 0 
 0 
 0 
 0 
 0 
 0 
 0 
 0 
 0 
 0 
 0 
 0 
 0 
 0 
 0 
 0 
 0 
 0 
 0 
 0 
 0 
 0 
 0 
 0 
 0 
 0 
 0 
 0 
 0 
 0 
 0 
 0 
 0 
 0 
 0 
 0 
 0 
 0 
 0 
 0 
 0 
 0 
 0 
 0 
 0 
 0 
 0 
 0 
 0 
 0 
 0 
 0 
 0 
 0 
 0 
 4 
 0 
 0 
 0 
 0 
 0 
 0 
 0 
 0 
 0 
 0 
 0 
 0 
 0 
 0 
 0 
 0 
 0 
 0 
 0 
 0 
 0 
 0 
 0 
 0 
 0 
 0 
 0 
 0 
 0 
 0 
 0 
 0 
 0 
 0 
 0 
 0 
 0 
 0 
 0 
 0 
 0 
 0 
 0 
 0 
 0 
 0 
 0 
 0 
 0 
 0 
 0 
 0 
 0 
 0 
 0 
 0 
 0 
 0 
 0 
 0 
 0 
 0 
 0 
 0 
 0 
 0 
 0 
 0 
 0 
 0 
 0 
 0 
 0 
 0 
 0 
 0 
 0 
 0 
 0 
 0 
 0 
 0 
 0 
 0 
 0 
 0 
 0 
 0 
 0 
 0 
 0 
 0 
 0 
 0 
 0 
 0 
 0 
 0 
 0 
 0 
 0 
 0 
 0 
 0 
 0 
 0 
 0 
 0 
 0 
 0 
 0 
 0 
 0 
 0 
 0 
 0 
 0 
 0 
 0 
 0 
 0 
 0 
 0 
 0 
 0 
 0 
 0 
 0 
 0 
 0 
 0 
 0 
 0 
 0 
 0 
 0 
 0 
 0 
 0 
 0 
 0 
 0 
 0 
 0 
 0 
 0 
 0 
 0 
 0 
 0 
 0 
 0 
 0 
 0 
 0 
 0 
 0 
 0 
 0 
 0 
 0 
 0 
 0 
 0 
 0 
 0 
 0 
 0 
 0 
 0 
 0 
 0 
 0 
 0 
 0 
 0 
 0 
 0 
 0 
 0 
 0 
 0 
 0 
 0 
 0 
 0 
 0 
 0 
 0 
 0 
 0 
 0 
 0 
 0 
 0 
 0 
 0 
 0 
 0 
 0 
 0 
 0 
 0 
 0 
 0 
 0 
 0 
 0 
 0 
 0 
 0 
 0 
 0 
 0 
 0 
 0 
 0 
 0 
 0 
 0 
 0 
 0 
 0 
 0 
 0 
 0 
 0 
 0 
 0 
 0 
 0 
 0 
 0 
 0 
 0 
 0 
 0 
 0 
 0 
 0 
 0 
 0 
 0 
 0 
 0 
 0 
 0 
 0 
 0 
 0 
 0 
 0 
 0 
 0 
 0 
 0 
 0 
 0 
 0 
 0 
 0 
 0 
 0 
 0 
 0 
 0 
 0 
 0 
 0 
 0 
 0 
 0 
 0 
 0 
 0 
 0 
 0 
 0 
 0 
 0 
 0 
 0 
 0 
 0 
 0 
 0 
 0 
 0 
 0 
 0 
 0 
 0 
 0 
 0 
 0 
 0 
 0 
 0 
 0 
 0 
 0 
 0 
 0 
 0 
 0 
 0 
 0 
 0 
 0 
 0 
 0 
 0 
 0 
 0 
 0 
 0 
 0 
 0 
 0 
 0 
 0 
 0 
 0 
 0 
 0 
 0 
 0 
 0 
 0 
 0 
 0 
 0 
 0 
 0 
 0 
 0 
 0 
 0 
 0 
 0 
 0 
 0 
 0 
 0 
 0 
 0 
 0 
 0 
 0 
 0 
 0 
 0 
 0 
 0 
 0 
 0 
 0 
 0 
 0 
 0 
 0 
 0 
 0 
 0 
 0 
 0 
 0 
 0 
 0 
 0 
 0 
 0 
 0 
 0 
 0 
 0 
 0 
 0 
 0 
 0 
 0 
 0 
 0 
 0 
 0 
 0 
 0 
 0 
 0 
 0 
 0 
 0 
 0 
 0 
 0 
 0 
 0 
 0 
 0 
 0 
 0 
 0 
 0 
 0 
 0 
 0 
 0 
 0 
 0 
 0 
 0 
 0 
 0 
 0 
 0 
 0 
 0 
 0 
 0 
 0 
 0 
 0 
 0 
 0 
 0 
 0 
 0 
 0 
 0 
 0 
 0 
 0 
 0 
 0 
 0 
 0 
 0 
 0 
 0 
 0 
 0 
 0 
 0 
 0 
 0 
 0 
 0 
 0 
 0 
 0 
 0 
 0 
 0 
 0 
 0 
 0 
 0 
 0 
 0 
 0 
 0 
 0 
 0 
 0 
 0 
 0 
 0 
 0 
 0 
 0 
 0 
 0 
 0 
 0 
 0 
 0 
 0 
 0 
 0 
 0 
 0 
 0 
 0 
 0 
 0 
 0 
 0 
 0 
 0 
 0 
 0 
 0 
 0 
 0 
 0 
 0 
 0 
 0 
 0 
 0 
 0 
 0 
 0 
 0 
 0 
 0 
 0 
 0 
 0 
 0 
 0 
 0 
 0 
 0 
 0 
 0 
 0 
 0 
 0 
 0 
 0 
 0 
 0 
 0 
 0 
 0 
 0 
 0 
 0 
 0 
 0 
 0 
 0 
 0 
 0 
 0 
 0 
 0 
 0 
 0 
 0 
 0 
 0 
 0 
 0 
 0 
 0 
 0 
 0 
 0 
 0 
 0 
 0 
 0 
 0 
 0 
 0 
 0 
 0 
 0 
 0 
 0 
 0 
 0 
 0 
 0 
 0 
 0 
 0 
 0 
 0 
 0 
 0 
 0 
 0 
 0 
 0 
 0 
 0 
 0 
 0 
 0 
 0 
 0 
 0 
 0 
 0 
 0 
 0 
 0 
 0 
 0 
 0 
 0 
 0 
 0 
 0 
 0 
 0 
 0 
 0 
 0 
 0 
 0 
 0 
 0 
 0 
 0 
 0 
 0 
 0 
 0 
 0 
 0 
 0 
 0 
 0 
 0 
 0 
 0 
 0 
 0 
 0 
 0 
 0 
 0 
 0 
 0 
 0 
 0 
 0 
 0 
 0 
 0 
 0 
 0 
 0 
 0 
 0 
 0 
 0 
 0 
 0 
 0 
 0 
 0 
 0 
 0 
 0 
 0 
 0 
 0 
 0 
 0 
 0 
 0 
 0 
 0 
 0 
 0 
 0 
 0 
 0 
 0 
 0 
 0 
 0 
 0 
 0 
 0 
 0 
 0 
 0 
 0 
 0 
 0 
 0 
 0 
 0 
 0 
 0 
 0 
 0 
 0 
 0 
 0 
 0 
 0 
 0 
 0 
 0 
 0 
 0 
 0 
 0 
 0 
 0 
 0 
 0 
 0 
 0 
 0 
 0 
 0 
 0 
 0 
 0 
 0 
 0 
 0 
 0 
 0 
 0 
 0 
 0 
 0 
 0 
 0 
 0 
 0 
 0 
 0 
 0 
 0 
 0 
 0 
 0 
 0 
 0 
 
 
 97 
 0 
 0 
 0 
 0 
 0 
 0 
 0 
 0 
 0 
 0 
 0 
 0 
 0 
 0 
 0 
 0 
 0 
 0 
 0 
 0 
 0 
 0 
 0 
 0 
 0 
 0 
 0 
 0 
 0 
 0 
 0 
 0 
 0 
 0 
 0 
 0 
 0 
 0 
 0 
 0 
 0 
 0 
 0 
 0 
 0 
 0 
 0 
 0 
 0 
 0 
 0 
 0 
 0 
 0 
 0 
 0 
 0 
 0 
 0 
 0 
 0 
 0 
 0 
 0 
 0 
 0 
 1 
 0 
 0 
 0 
 0 
 0 
 0 
 0 
 0 
 0 
 0 
 0 
 0 
 0 
 0 
 0 
 0 
 0 
 0 
 0 
 0 
 0 
 0 
 0 
 0 
 0 
 0 
 0 
 0 
 0 
 0 
 4 
 0 
 0 
 0 
 0 
 0 
 0 
 0 
 0 
 0 
 0 
 0 
 0 
 0 
 0 
 0 
 0 
 0 
 0 
 0 
 0 
 0 
 0 
 0 
 0 
 0 
 0 
 0 
 0 
 0 
 0 
 0 
 0 
 0 
 0 
 0 
 0 
 0 
 0 
 0 
 0 
 0 
 0 
 0 
 0 
 0 
 0 
 0 
 0 
 0 
 0 
 0 
 0 
 0 
 0 
 0 
 0 
 0 
 0 
 0 
 0 
 0 
 0 
 0 
 0 
 0 
 0 
 0 
 0 
 0 
 0 
 0 
 0 
 0 
 0 
 0 
 0 
 0 
 0 
 0 
 0 
 0 
 0 
 0 
 0 
 0 
 0 
 0 
 0 
 0 
 0 
 0 
 0 
 0 
 0 
 0 
 0 
 0 
 0 
 0 
 0 
 0 
 0 
 0 
 0 
 0 
 0 
 0 
 0 
 0 
 0 
 0 
 0 
 0 
 0 
 0 
 0 
 0 
 0 
 0 
 0 
 0 
 0 
 0 
 0 
 0 
 0 
 0 
 0 
 0 
 0 
 0 
 0 
 0 
 0 
 0 
 0 
 0 
 0 
 0 
 0 
 0 
 0 
 0 
 0 
 0 
 0 
 0 
 0 
 0 
 0 
 0 
 0 
 0 
 0 
 0 
 0 
 0 
 0 
 0 
 0 
 0 
 0 
 0 
 0 
 0 
 0 
 0 
 0 
 0 
 0 
 0 
 0 
 0 
 0 
 0 
 0 
 0 
 0 
 0 
 0 
 0 
 0 
 0 
 0 
 0 
 0 
 0 
 0 
 0 
 0 
 0 
 0 
 0 
 0 
 0 
 0 
 0 
 0 
 0 
 0 
 0 
 0 
 0 
 0 
 0 
 0 
 0 
 0 
 0 
 0 
 0 
 0 
 0 
 0 
 0 
 0 
 0 
 0 
 0 
 0 
 0 
 0 
 0 
 0 
 0 
 0 
 0 
 0 
 0 
 0 
 0 
 0 
 0 
 0 
 0 
 0 
 0 
 0 
 0 
 0 
 0 
 0 
 0 
 0 
 0 
 0 
 0 
 0 
 0 
 0 
 0 
 0 
 0 
 0 
 0 
 0 
 0 
 0 
 0 
 0 
 0 
 0 
 0 
 0 
 0 
 0 
 0 
 0 
 0 
 0 
 0 
 0 
 0 
 0 
 0 
 0 
 0 
 0 
 0 
 0 
 0 
 0 
 0 
 0 
 0 
 0 
 0 
 0 
 0 
 0 
 0 
 0 
 0 
 0 
 0 
 0 
 0 
 0 
 0 
 0 
 0 
 0 
 0 
 0 
 0 
 0 
 0 
 0 
 0 
 0 
 0 
 0 
 0 
 0 
 0 
 0 
 0 
 0 
 0 
 0 
 0 
 0 
 0 
 0 
 0 
 0 
 0 
 0 
 0 
 0 
 0 
 0 
 0 
 0 
 0 
 0 
 0 
 0 
 0 
 0 
 0 
 0 
 0 
 0 
 0 
 0 
 0 
 0 
 0 
 0 
 0 
 0 
 0 
 0 
 0 
 0 
 0 
 0 
 0 
 0 
 0 
 0 
 0 
 0 
 0 
 0 
 0 
 0 
 0 
 0 
 0 
 0 
 0 
 0 
 0 
 0 
 0 
 0 
 0 
 0 
 0 
 0 
 0 
 0 
 0 
 0 
 0 
 0 
 0 
 0 
 0 
 0 
 0 
 0 
 0 
 0 
 0 
 0 
 0 
 0 
 0 
 0 
 0 
 0 
 0 
 0 
 0 
 0 
 0 
 0 
 0 
 0 
 0 
 0 
 0 
 0 
 0 
 0 
 0 
 0 
 0 
 0 
 0 
 0 
 0 
 0 
 0 
 0 
 0 
 0 
 0 
 0 
 0 
 0 
 0 
 0 
 0 
 0 
 0 
 0 
 0 
 0 
 0 
 0 
 0 
 0 
 0 
 0 
 0 
 0 
 0 
 0 
 0 
 0 
 0 
 0 
 0 
 0 
 0 
 0 
 0 
 0 
 0 
 0 
 0 
 0 
 0 
 0 
 0 
 0 
 0 
 0 
 0 
 0 
 0 
 0 
 0 
 0 
 0 
 0 
 0 
 0 
 0 
 0 
 0 
 0 
 0 
 0 
 0 
 0 
 0 
 0 
 0 
 0 
 0 
 0 
 0 
 0 
 0 
 0 
 0 
 0 
 0 
 0 
 0 
 0 
 0 
 0 
 0 
 0 
 0 
 0 
 0 
 0 
 0 
 0 
 0 
 0 
 0 
 0 
 0 
 0 
 0 
 0 
 0 
 0 
 0 
 0 
 0 
 0 
 0 
 0 
 0 
 0 
 0 
 0 
 0 
 0 
 0 
 0 
 0 
 0 
 0 
 0 
 0 
 0 
 0 
 0 
 0 
 0 
 0 
 0 
 0 
 0 
 0 
 0 
 0 
 0 
 0 
 0 
 0 
 0 
 0 
 0 
 0 
 0 
 0 
 0 
 0 
 0 
 0 
 0 
 0 
 0 
 0 
 0 
 0 
 0 
 0 
 0 
 0 
 0 
 0 
 0 
 0 
 0 
 0 
 0 
 0 
 0 
 0 
 0 
 0 
 0 
 0 
 0 
 0 
 0 
 0 
 0 
 0 
 0 
 0 
 0 
 0 
 0 
 0 
 0 
 0 
 0 
 0 
 0 
 0 
 0 
 0 
 0 
 0 
 0 
 0 
 0 
 0 
 0 
 0 
 0 
 0 
 0 
 0 
 0 
 0 
 0 
 0 
 0 
 0 
 0 
 0 
 0 
 0 
 0 
 0 
 0 
 0 
 0 
 0 
 0 
 0 
 0 
 0 
 0 
 0 
 0 
 0 
 0 
 0 
 0 
 0 
 0 
 0 
 0 
 0 
 0 
 0 
 0 
 0 
 0 
 0 
 0 
 0 
 0 
 0 
 0 
 0 
 0 
 0 
 0 
 0 
 0 
 0 
 0 
 0 
 0 
 0 
 0 
 0 
 0 
 0 
 0 
 0 
 0 
 0 
 0 
 0 
 0 
 0 
 0 
 0 
 0 
 0 
 0 
 0 
 0 
 0 
 0 
 0 
 0 
 0 
 0 
 0 
 0 
 0 
 0 
 0 
 0 
 0 
 0 
 0 
 0 
 0 
 0 
 0 
 0 
 0 
 0 
 0 
 0 
 0 
 0 
 0 
 0 
 
 
 98 
 0 
 0 
 0 
 0 
 0 
 0 
 0 
 0 
 0 
 0 
 0 
 0 
 0 
 0 
 0 
 0 
 0 
 0 
 0 
 0 
 0 
 0 
 0 
 0 
 0 
 0 
 0 
 0 
 0 
 0 
 0 
 0 
 0 
 0 
 0 
 0 
 0 
 0 
 0 
 0 
 0 
 0 
 0 
 0 
 0 
 0 
 0 
 0 
 0 
 0 
 0 
 0 
 0 
 0 
 0 
 0 
 0 
 0 
 0 
 0 
 0 
 0 
 0 
 0 
 0 
 0 
 0 
 0 
 0 
 0 
 0 
 0 
 0 
 0 
 0 
 0 
 0 
 0 
 0 
 0 
 0 
 0 
 0 
 0 
 0 
 0 
 0 
 0 
 0 
 0 
 0 
 0 
 0 
 0 
 0 
 0 
 0 
 0 
 0 
 0 
 0 
 5 
 0 
 0 
 0 
 0 
 0 
 0 
 0 
 0 
 0 
 0 
 0 
 0 
 0 
 0 
 0 
 0 
 0 
 0 
 0 
 0 
 0 
 0 
 0 
 0 
 0 
 0 
 0 
 0 
 0 
 0 
 0 
 0 
 0 
 0 
 0 
 0 
 0 
 0 
 0 
 0 
 0 
 0 
 0 
 0 
 0 
 0 
 0 
 0 
 0 
 0 
 0 
 0 
 0 
 0 
 0 
 0 
 0 
 0 
 0 
 0 
 0 
 0 
 0 
 0 
 0 
 0 
 0 
 0 
 0 
 0 
 0 
 0 
 0 
 0 
 0 
 0 
 0 
 0 
 0 
 0 
 0 
 0 
 0 
 0 
 0 
 0 
 0 
 0 
 0 
 0 
 0 
 0 
 0 
 0 
 0 
 0 
 0 
 0 
 0 
 0 
 0 
 0 
 0 
 0 
 0 
 0 
 0 
 0 
 0 
 0 
 0 
 0 
 0 
 0 
 0 
 0 
 0 
 0 
 0 
 0 
 0 
 0 
 0 
 0 
 0 
 0 
 0 
 0 
 0 
 0 
 0 
 0 
 0 
 0 
 0 
 0 
 0 
 0 
 0 
 0 
 0 
 0 
 0 
 0 
 0 
 0 
 0 
 0 
 0 
 0 
 0 
 0 
 0 
 0 
 0 
 0 
 0 
 0 
 0 
 0 
 0 
 0 
 0 
 0 
 0 
 0 
 0 
 0 
 0 
 0 
 0 
 0 
 0 
 0 
 0 
 0 
 0 
 0 
 0 
 0 
 0 
 0 
 0 
 0 
 0 
 0 
 0 
 0 
 0 
 0 
 0 
 0 
 0 
 0 
 0 
 0 
 0 
 0 
 0 
 0 
 0 
 0 
 0 
 0 
 0 
 0 
 0 
 0 
 0 
 0 
 0 
 0 
 0 
 0 
 0 
 0 
 0 
 0 
 0 
 0 
 0 
 0 
 0 
 0 
 0 
 0 
 0 
 0 
 0 
 0 
 0 
 0 
 0 
 0 
 0 
 0 
 0 
 0 
 0 
 0 
 0 
 0 
 0 
 0 
 0 
 0 
 0 
 0 
 0 
 0 
 0 
 0 
 0 
 0 
 0 
 0 
 0 
 0 
 0 
 0 
 0 
 0 
 0 
 0 
 0 
 0 
 0 
 0 
 0 
 0 
 0 
 0 
 0 
 0 
 0 
 0 
 0 
 0 
 0 
 0 
 0 
 0 
 0 
 0 
 0 
 0 
 0 
 0 
 0 
 0 
 0 
 0 
 0 
 0 
 0 
 0 
 0 
 0 
 0 
 0 
 0 
 0 
 0 
 0 
 0 
 0 
 0 
 0 
 0 
 0 
 0 
 0 
 0 
 0 
 0 
 0 
 0 
 0 
 0 
 0 
 0 
 0 
 0 
 0 
 0 
 0 
 0 
 0 
 0 
 0 
 0 
 0 
 0 
 0 
 0 
 0 
 0 
 0 
 0 
 0 
 0 
 0 
 0 
 0 
 0 
 0 
 0 
 0 
 0 
 0 
 0 
 0 
 0 
 0 
 0 
 0 
 0 
 0 
 0 
 0 
 0 
 0 
 0 
 0 
 0 
 0 
 0 
 0 
 0 
 0 
 0 
 0 
 0 
 0 
 0 
 0 
 0 
 0 
 0 
 0 
 0 
 0 
 0 
 0 
 0 
 0 
 0 
 0 
 0 
 0 
 0 
 0 
 0 
 0 
 0 
 0 
 0 
 0 
 0 
 0 
 0 
 0 
 0 
 0 
 0 
 0 
 0 
 0 
 0 
 0 
 0 
 0 
 0 
 0 
 0 
 0 
 0 
 0 
 0 
 0 
 0 
 0 
 0 
 0 
 0 
 0 
 0 
 0 
 0 
 0 
 0 
 0 
 0 
 0 
 0 
 0 
 0 
 0 
 0 
 0 
 0 
 0 
 0 
 0 
 0 
 0 
 0 
 0 
 0 
 0 
 0 
 0 
 0 
 0 
 0 
 0 
 0 
 0 
 0 
 0 
 0 
 0 
 0 
 0 
 0 
 0 
 0 
 0 
 0 
 0 
 0 
 0 
 0 
 0 
 0 
 0 
 0 
 0 
 0 
 0 
 0 
 0 
 0 
 0 
 0 
 0 
 0 
 0 
 0 
 0 
 0 
 0 
 0 
 0 
 0 
 0 
 0 
 0 
 0 
 0 
 0 
 0 
 0 
 0 
 0 
 0 
 0 
 0 
 0 
 0 
 0 
 0 
 0 
 0 
 0 
 0 
 0 
 0 
 0 
 0 
 0 
 0 
 0 
 0 
 0 
 0 
 0 
 0 
 0 
 0 
 0 
 0 
 0 
 0 
 0 
 0 
 0 
 0 
 0 
 0 
 0 
 0 
 0 
 0 
 0 
 0 
 0 
 0 
 0 
 0 
 0 
 0 
 0 
 0 
 0 
 0 
 0 
 0 
 0 
 0 
 0 
 0 
 0 
 0 
 0 
 0 
 0 
 0 
 0 
 0 
 0 
 0 
 0 
 0 
 0 
 0 
 0 
 0 
 0 
 0 
 0 
 0 
 0 
 0 
 0 
 0 
 0 
 0 
 0 
 0 
 0 
 0 
 0 
 0 
 0 
 0 
 0 
 0 
 0 
 0 
 0 
 0 
 0 
 0 
 0 
 0 
 0 
 0 
 0 
 0 
 0 
 0 
 0 
 0 
 0 
 0 
 0 
 0 
 0 
 0 
 0 
 0 
 0 
 0 
 0 
 0 
 0 
 0 
 0 
 0 
 0 
 0 
 0 
 0 
 0 
 0 
 0 
 0 
 0 
 0 
 0 
 0 
 0 
 0 
 0 
 0 
 0 
 0 
 0 
 0 
 0 
 0 
 0 
 0 
 0 
 0 
 0 
 0 
 0 
 0 
 0 
 0 
 0 
 0 
 0 
 0 
 0 
 0 
 0 
 0 
 0 
 0 
 0 
 0 
 0 
 0 
 0 
 0 
 0 
 0 
 0 
 0 
 0 
 0 
 0 
 0 
 0 
 0 
 0 
 0 
 0 
 0 
 0 
 0 
 0 
 0 
 0 
 0 
 0 
 0 
 0 
 0 
 0 
 0 
 0 
 0 
 0 
 0 
 0 
 0 
 0 
 0 
 0 
 0 
 0 
 0 
 0 
 0 
 0 
 0 
 0 
 0 
 0 
 0 
 
 
 99 
 0 
 0 
 0 
 0 
 0 
 0 
 0 
 0 
 0 
 0 
 0 
 0 
 0 
 0 
 0 
 0 
 0 
 0 
 0 
 0 
 0 
 0 
 0 
 0 
 0 
 0 
 0 
 0 
 0 
 0 
 0 
 0 
 0 
 0 
 0 
 0 
 0 
 0 
 0 
 0 
 0 
 0 
 0 
 0 
 0 
 0 
 0 
 0 
 0 
 0 
 0 
 0 
 0 
 0 
 0 
 0 
 0 
 0 
 0 
 0 
 0 
 0 
 0 
 0 
 0 
 0 
 0 
 0 
 0 
 0 
 0 
 0 
 0 
 0 
 0 
 0 
 0 
 0 
 0 
 0 
 0 
 0 
 0 
 0 
 0 
 0 
 0 
 0 
 0 
 0 
 0 
 0 
 0 
 0 
 0 
 0 
 0 
 0 
 6 
 0 
 0 
 0 
 0 
 0 
 0 
 0 
 0 
 0 
 0 
 0 
 0 
 0 
 0 
 0 
 0 
 0 
 0 
 0 
 0 
 0 
 0 
 0 
 0 
 0 
 0 
 0 
 0 
 0 
 0 
 0 
 0 
 0 
 0 
 0 
 0 
 0 
 0 
 0 
 0 
 0 
 0 
 0 
 0 
 0 
 0 
 0 
 0 
 0 
 0 
 0 
 0 
 0 
 0 
 0 
 0 
 0 
 0 
 0 
 0 
 0 
 0 
 0 
 0 
 0 
 0 
 0 
 0 
 0 
 0 
 0 
 0 
 0 
 0 
 0 
 0 
 0 
 0 
 0 
 0 
 0 
 0 
 0 
 0 
 0 
 0 
 0 
 0 
 0 
 0 
 0 
 0 
 0 
 0 
 0 
 0 
 0 
 0 
 0 
 0 
 0 
 0 
 0 
 0 
 0 
 0 
 0 
 0 
 0 
 0 
 0 
 0 
 0 
 0 
 0 
 0 
 0 
 0 
 0 
 0 
 0 
 0 
 0 
 0 
 0 
 0 
 0 
 0 
 0 
 0 
 0 
 0 
 0 
 0 
 0 
 0 
 0 
 0 
 0 
 0 
 0 
 0 
 0 
 0 
 0 
 0 
 0 
 0 
 0 
 0 
 0 
 0 
 0 
 0 
 0 
 0 
 0 
 0 
 0 
 0 
 0 
 0 
 0 
 0 
 0 
 0 
 0 
 0 
 0 
 0 
 0 
 0 
 0 
 0 
 0 
 0 
 0 
 0 
 0 
 0 
 0 
 0 
 0 
 0 
 0 
 0 
 0 
 0 
 0 
 0 
 0 
 0 
 0 
 0 
 0 
 0 
 0 
 0 
 0 
 0 
 0 
 0 
 0 
 0 
 0 
 0 
 0 
 0 
 0 
 0 
 0 
 0 
 0 
 0 
 0 
 0 
 0 
 0 
 0 
 0 
 0 
 0 
 0 
 0 
 0 
 0 
 0 
 0 
 0 
 0 
 0 
 0 
 0 
 0 
 0 
 0 
 0 
 0 
 0 
 0 
 0 
 0 
 0 
 0 
 0 
 0 
 0 
 0 
 0 
 0 
 0 
 0 
 0 
 0 
 0 
 0 
 0 
 0 
 0 
 0 
 0 
 0 
 0 
 0 
 0 
 0 
 0 
 0 
 0 
 0 
 0 
 0 
 0 
 0 
 0 
 0 
 0 
 0 
 0 
 0 
 0 
 0 
 0 
 0 
 0 
 0 
 0 
 0 
 0 
 0 
 0 
 0 
 0 
 0 
 0 
 0 
 0 
 0 
 0 
 0 
 0 
 0 
 0 
 0 
 0 
 0 
 0 
 0 
 0 
 0 
 0 
 0 
 0 
 0 
 0 
 0 
 0 
 0 
 0 
 0 
 0 
 0 
 0 
 0 
 0 
 0 
 0 
 0 
 0 
 0 
 0 
 0 
 0 
 0 
 0 
 0 
 0 
 0 
 0 
 0 
 0 
 0 
 0 
 0 
 0 
 0 
 0 
 0 
 0 
 0 
 0 
 0 
 0 
 0 
 0 
 0 
 0 
 0 
 0 
 0 
 0 
 0 
 0 
 0 
 0 
 0 
 0 
 0 
 0 
 0 
 0 
 0 
 0 
 0 
 0 
 0 
 0 
 0 
 0 
 0 
 0 
 0 
 0 
 0 
 0 
 0 
 0 
 0 
 0 
 0 
 0 
 0 
 0 
 0 
 0 
 0 
 0 
 0 
 0 
 0 
 0 
 0 
 0 
 0 
 0 
 0 
 0 
 0 
 0 
 0 
 0 
 0 
 0 
 0 
 0 
 0 
 0 
 0 
 0 
 0 
 0 
 0 
 0 
 0 
 0 
 0 
 0 
 0 
 0 
 0 
 0 
 0 
 0 
 0 
 0 
 0 
 0 
 0 
 0 
 0 
 0 
 0 
 0 
 0 
 0 
 0 
 0 
 0 
 0 
 0 
 0 
 0 
 0 
 0 
 0 
 0 
 0 
 0 
 0 
 0 
 0 
 0 
 0 
 0 
 0 
 0 
 0 
 0 
 0 
 0 
 0 
 0 
 0 
 0 
 0 
 0 
 0 
 0 
 0 
 0 
 0 
 0 
 0 
 0 
 0 
 0 
 0 
 0 
 0 
 0 
 0 
 0 
 0 
 0 
 0 
 0 
 0 
 0 
 0 
 0 
 0 
 0 
 0 
 0 
 0 
 0 
 0 
 0 
 0 
 0 
 0 
 0 
 0 
 0 
 0 
 0 
 0 
 0 
 0 
 0 
 0 
 0 
 0 
 0 
 0 
 0 
 0 
 0 
 0 
 0 
 0 
 0 
 0 
 0 
 0 
 0 
 0 
 0 
 0 
 0 
 0 
 0 
 0 
 0 
 0 
 0 
 0 
 0 
 0 
 0 
 0 
 0 
 0 
 0 
 0 
 0 
 0 
 0 
 0 
 0 
 0 
 0 
 0 
 0 
 0 
 0 
 0 
 0 
 0 
 0 
 0 
 0 
 0 
 0 
 0 
 0 
 0 
 0 
 0 
 0 
 0 
 0 
 0 
 0 
 0 
 0 
 0 
 0 
 0 
 0 
 0 
 0 
 0 
 0 
 0 
 0 
 0 
 0 
 0 
 0 
 0 
 0 
 0 
 0 
 0 
 0 
 0 
 0 
 0 
 0 
 0 
 0 
 0 
 0 
 0 
 0 
 0 
 0 
 0 
 0 
 0 
 0 
 0 
 0 
 0 
 0 
 0 
 0 
 0 
 0 
 0 
 0 
 0 
 0 
 0 
 0 
 0 
 0 
 0 
 0 
 0 
 0 
 0 
 0 
 0 
 0 
 0 
 0 
 0 
 0 
 0 
 0 
 0 
 0 
 0 
 0 
 0 
 0 
 0 
 0 
 0 
 0 
 0 
 0 
 0 
 0 
 0 
 0 
 0 
 0 
 0 
 0 
 0 
 0 
 0 
 0 
 0 
 0 
 0 
 0 
 0 
 0 
 0 
 0 
 0 
 0 
 0 
 0 
 0 
 0 
 0 
 0 
 0 
 0 
 0 
 0 
 0 
 0 
 0 
 0 
 0 
 0 
 0 
 0 
 0 
 0 
 0 
 0 
 0 
 0 
 0 
 0 
 0 
 0 
 0 
 0 
 0 
 0 
 0 
 0 
 0 
 0 
 0 
 0 
 0 
 0 
 0 
 
 
 100 
 0 
 0 
 0 
 0 
 0 
 0 
 0 
 0 
 0 
 0 
 0 
 0 
 0 
 0 
 0 
 0 
 0 
 0 
 0 
 0 
 0 
 0 
 0 
 0 
 0 
 0 
 0 
 0 
 0 
 0 
 0 
 0 
 0 
 0 
 0 
 0 
 0 
 0 
 0 
 0 
 0 
 0 
 0 
 0 
 0 
 0 
 0 
 0 
 0 
 0 
 0 
 0 
 0 
 0 
 0 
 0 
 0 
 0 
 0 
 0 
 0 
 0 
 0 
 0 
 0 
 0 
 0 
 0 
 0 
 0 
 0 
 0 
 0 
 0 
 0 
 0 
 0 
 0 
 0 
 0 
 0 
 0 
 0 
 0 
 0 
 0 
 0 
 0 
 0 
 0 
 0 
 0 
 0 
 0 
 0 
 0 
 0 
 0 
 0 
 0 
 0 
 0 
 0 
 0 
 0 
 0 
 0 
 0 
 0 
 0 
 0 
 0 
 0 
 0 
 0 
 0 
 0 
 0 
 0 
 0 
 0 
 0 
 0 
 0 
 0 
 0 
 0 
 0 
 0 
 0 
 0 
 0 
 0 
 0 
 0 
 0 
 0 
 0 
 0 
 0 
 0 
 0 
 0 
 0 
 0 
 0 
 0 
 0 
 0 
 0 
 0 
 0 
 0 
 0 
 0 
 1 
 0 
 0 
 0 
 0 
 0 
 0 
 0 
 0 
 0 
 0 
 0 
 0 
 0 
 0 
 0 
 0 
 0 
 0 
 0 
 0 
 0 
 0 
 0 
 0 
 0 
 0 
 0 
 0 
 0 
 0 
 0 
 0 
 0 
 0 
 0 
 0 
 0 
 0 
 0 
 0 
 0 
 0 
 0 
 0 
 0 
 0 
 0 
 0 
 0 
 0 
 0 
 0 
 0 
 0 
 0 
 0 
 0 
 0 
 0 
 0 
 0 
 0 
 0 
 0 
 0 
 0 
 0 
 0 
 0 
 0 
 0 
 0 
 0 
 0 
 0 
 0 
 0 
 0 
 0 
 0 
 0 
 0 
 0 
 0 
 0 
 0 
 0 
 0 
 0 
 0 
 0 
 0 
 0 
 0 
 0 
 0 
 0 
 0 
 0 
 0 
 0 
 0 
 0 
 0 
 0 
 0 
 0 
 0 
 0 
 0 
 0 
 0 
 0 
 0 
 0 
 0 
 0 
 0 
 0 
 0 
 0 
 0 
 0 
 0 
 0 
 0 
 0 
 0 
 0 
 0 
 0 
 0 
 0 
 0 
 0 
 0 
 0 
 0 
 0 
 0 
 0 
 0 
 0 
 0 
 0 
 0 
 0 
 0 
 0 
 0 
 0 
 0 
 0 
 0 
 0 
 0 
 0 
 0 
 0 
 0 
 0 
 0 
 0 
 0 
 0 
 0 
 0 
 0 
 0 
 0 
 0 
 0 
 0 
 0 
 0 
 0 
 0 
 0 
 0 
 0 
 0 
 0 
 0 
 0 
 0 
 0 
 0 
 0 
 0 
 0 
 0 
 0 
 0 
 0 
 0 
 0 
 0 
 0 
 0 
 0 
 0 
 0 
 0 
 0 
 0 
 0 
 0 
 0 
 0 
 0 
 0 
 0 
 0 
 0 
 0 
 0 
 0 
 0 
 0 
 0 
 0 
 0 
 0 
 0 
 0 
 0 
 0 
 0 
 0 
 0 
 0 
 0 
 0 
 0 
 0 
 0 
 0 
 0 
 0 
 0 
 0 
 0 
 0 
 0 
 0 
 0 
 0 
 0 
 0 
 0 
 0 
 0 
 0 
 0 
 0 
 0 
 0 
 0 
 0 
 0 
 0 
 0 
 0 
 0 
 0 
 0 
 0 
 0 
 0 
 0 
 0 
 0 
 0 
 0 
 0 
 0 
 0 
 0 
 0 
 0 
 0 
 0 
 0 
 0 
 0 
 0 
 0 
 0 
 0 
 0 
 0 
 0 
 0 
 0 
 0 
 0 
 0 
 0 
 0 
 0 
 0 
 0 
 0 
 0 
 0 
 0 
 0 
 0 
 0 
 0 
 0 
 0 
 0 
 0 
 0 
 0 
 0 
 0 
 0 
 0 
 0 
 0 
 0 
 0 
 0 
 0 
 0 
 0 
 0 
 0 
 0 
 0 
 0 
 0 
 0 
 0 
 0 
 0 
 0 
 0 
 0 
 0 
 0 
 0 
 0 
 0 
 0 
 0 
 0 
 0 
 0 
 0 
 0 
 0 
 0 
 0 
 0 
 0 
 0 
 0 
 0 
 0 
 0 
 0 
 0 
 0 
 0 
 0 
 0 
 0 
 0 
 0 
 0 
 0 
 0 
 0 
 0 
 0 
 0 
 0 
 0 
 0 
 0 
 0 
 0 
 0 
 0 
 0 
 0 
 0 
 0 
 0 
 0 
 0 
 0 
 0 
 0 
 0 
 0 
 0 
 0 
 0 
 0 
 0 
 0 
 0 
 0 
 0 
 0 
 0 
 0 
 0 
 0 
 0 
 0 
 0 
 0 
 0 
 0 
 0 
 0 
 0 
 0 
 0 
 0 
 0 
 0 
 0 
 0 
 0 
 0 
 0 
 0 
 0 
 0 
 0 
 0 
 0 
 0 
 0 
 0 
 0 
 0 
 0 
 0 
 0 
 0 
 0 
 0 
 0 
 0 
 0 
 0 
 0 
 0 
 0 
 0 
 0 
 0 
 0 
 0 
 0 
 0 
 0 
 0 
 0 
 0 
 0 
 0 
 0 
 0 
 0 
 0 
 0 
 0 
 0 
 0 
 0 
 0 
 0 
 0 
 0 
 0 
 0 
 0 
 0 
 0 
 0 
 0 
 0 
 0 
 0 
 0 
 0 
 0 
 0 
 0 
 0 
 0 
 0 
 0 
 0 
 0 
 0 
 0 
 0 
 0 
 0 
 0 
 0 
 0 
 0 
 0 
 0 
 0 
 0 
 0 
 0 
 0 
 0 
 0 
 0 
 0 
 0 
 0 
 0 
 0 
 0 
 0 
 0 
 0 
 0 
 0 
 0 
 0 
 0 
 0 
 0 
 0 
 0 
 0 
 0 
 0 
 0 
 0 
 0 
 0 
 0 
 0 
 0 
 0 
 0 
 0 
 0 
 0 
 0 
 0 
 0 
 0 
 0 
 0 
 0 
 0 
 0 
 0 
 0 
 0 
 0 
 0 
 0 
 0 
 0 
 0 
 0 
 0 
 0 
 0 
 0 
 0 
 0 
 0 
 0 
 0 
 0 
 0 
 0 
 0 
 0 
 0 
 0 
 0 
 0 
 0 
 0 
 0 
 0 
 0 
 0 
 0 
 0 
 0 
 0 
 0 
 0 
 0 
 0 
 0 
 0 
 0 
 0 
 0 
 0 
 0 
 0 
 0 
 0 
 0 
 0 
 0 
 0 
 0 
 0 
 0 
 0 
 0 
 0 
 0 
 0 
 0 
 0 
 0 
 0 
 0 
 0 
 0 
 0 
 0 
 0 
 0 
 0 
 0 
 0 
 0 
 0 
 0 
 0 
 0 
 0 
 0 
 0 
 0 
 0 
 0 
 0 
 0 
 0 
 0 
 0 
 0 
 0 
 0 
 0 
 0 
 0 
 0 
 0 
 0 
 0 
 0 
 0 
 
 
 101 
 0 
 0 
 0 
 0 
 0 
 0 
 0 
 0 
 0 
 0 
 0 
 0 
 0 
 0 
 0 
 0 
 0 
 0 
 0 
 0 
 0 
 0 
 0 
 0 
 0 
 0 
 0 
 0 
 0 
 0 
 0 
 0 
 0 
 0 
 0 
 0 
 0 
 0 
 0 
 0 
 0 
 0 
 0 
 0 
 0 
 0 
 0 
 0 
 0 
 0 
 0 
 0 
 0 
 0 
 0 
 0 
 0 
 0 
 0 
 0 
 0 
 0 
 0 
 0 
 0 
 0 
 0 
 0 
 0 
 0 
 0 
 0 
 0 
 0 
 0 
 0 
 0 
 0 
 0 
 0 
 0 
 0 
 0 
 0 
 0 
 0 
 0 
 0 
 0 
 0 
 0 
 0 
 0 
 0 
 0 
 0 
 0 
 0 
 0 
 0 
 0 
 7 
 0 
 0 
 0 
 0 
 0 
 0 
 0 
 0 
 0 
 0 
 0 
 0 
 0 
 0 
 0 
 0 
 0 
 0 
 0 
 0 
 0 
 0 
 0 
 0 
 0 
 0 
 0 
 0 
 0 
 0 
 0 
 0 
 0 
 0 
 0 
 0 
 0 
 0 
 0 
 0 
 0 
 0 
 0 
 0 
 0 
 0 
 0 
 0 
 0 
 0 
 0 
 0 
 0 
 0 
 0 
 0 
 0 
 0 
 0 
 0 
 0 
 0 
 0 
 0 
 0 
 0 
 0 
 0 
 0 
 0 
 0 
 0 
 0 
 0 
 0 
 0 
 0 
 0 
 0 
 0 
 0 
 0 
 0 
 0 
 0 
 0 
 0 
 0 
 0 
 0 
 0 
 0 
 0 
 0 
 0 
 0 
 0 
 0 
 0 
 0 
 0 
 0 
 0 
 0 
 0 
 0 
 0 
 0 
 0 
 0 
 0 
 0 
 0 
 0 
 0 
 0 
 0 
 0 
 0 
 0 
 0 
 0 
 0 
 0 
 0 
 0 
 0 
 0 
 0 
 0 
 0 
 0 
 0 
 0 
 0 
 0 
 0 
 0 
 0 
 0 
 0 
 0 
 0 
 0 
 0 
 0 
 0 
 0 
 0 
 0 
 0 
 0 
 0 
 0 
 0 
 0 
 0 
 0 
 0 
 0 
 0 
 0 
 0 
 0 
 0 
 0 
 0 
 0 
 0 
 0 
 0 
 0 
 0 
 0 
 0 
 0 
 0 
 0 
 0 
 0 
 0 
 0 
 0 
 0 
 0 
 0 
 0 
 0 
 0 
 0 
 0 
 0 
 0 
 0 
 0 
 0 
 0 
 0 
 0 
 0 
 0 
 0 
 0 
 0 
 0 
 0 
 0 
 0 
 0 
 0 
 0 
 0 
 0 
 0 
 0 
 0 
 0 
 0 
 0 
 0 
 0 
 0 
 0 
 0 
 0 
 0 
 0 
 0 
 0 
 0 
 0 
 0 
 0 
 0 
 0 
 0 
 0 
 0 
 0 
 0 
 0 
 0 
 0 
 0 
 0 
 0 
 0 
 0 
 0 
 0 
 0 
 0 
 0 
 0 
 0 
 0 
 0 
 0 
 0 
 0 
 0 
 0 
 0 
 0 
 0 
 0 
 0 
 0 
 0 
 0 
 0 
 0 
 0 
 0 
 0 
 0 
 0 
 0 
 0 
 0 
 0 
 0 
 0 
 0 
 0 
 0 
 0 
 0 
 0 
 0 
 0 
 0 
 0 
 0 
 0 
 0 
 0 
 0 
 0 
 0 
 0 
 0 
 0 
 0 
 0 
 0 
 0 
 0 
 0 
 0 
 0 
 0 
 0 
 0 
 0 
 0 
 0 
 0 
 0 
 0 
 0 
 0 
 0 
 0 
 0 
 0 
 0 
 0 
 0 
 0 
 0 
 0 
 0 
 0 
 0 
 0 
 0 
 0 
 0 
 0 
 0 
 0 
 0 
 0 
 0 
 0 
 0 
 0 
 0 
 0 
 0 
 0 
 0 
 0 
 0 
 0 
 0 
 0 
 0 
 0 
 0 
 0 
 0 
 0 
 0 
 0 
 0 
 0 
 0 
 0 
 0 
 0 
 0 
 0 
 0 
 0 
 0 
 0 
 0 
 0 
 0 
 0 
 0 
 0 
 0 
 0 
 0 
 0 
 0 
 0 
 0 
 0 
 0 
 0 
 0 
 0 
 0 
 0 
 0 
 0 
 0 
 0 
 0 
 0 
 0 
 0 
 0 
 0 
 0 
 0 
 0 
 0 
 0 
 0 
 0 
 0 
 0 
 0 
 0 
 0 
 0 
 0 
 0 
 0 
 0 
 0 
 0 
 0 
 0 
 0 
 0 
 0 
 0 
 0 
 0 
 0 
 0 
 0 
 0 
 0 
 0 
 0 
 0 
 0 
 0 
 0 
 0 
 0 
 0 
 0 
 0 
 0 
 0 
 0 
 0 
 0 
 0 
 0 
 0 
 0 
 0 
 0 
 0 
 0 
 0 
 0 
 0 
 0 
 0 
 0 
 0 
 0 
 0 
 0 
 0 
 0 
 0 
 0 
 0 
 0 
 0 
 0 
 0 
 0 
 0 
 0 
 0 
 0 
 0 
 0 
 0 
 0 
 0 
 0 
 0 
 0 
 0 
 0 
 0 
 0 
 0 
 0 
 0 
 0 
 0 
 0 
 0 
 0 
 0 
 0 
 0 
 0 
 0 
 0 
 0 
 0 
 0 
 0 
 0 
 0 
 0 
 0 
 0 
 0 
 0 
 0 
 0 
 0 
 0 
 0 
 0 
 0 
 0 
 0 
 0 
 0 
 0 
 0 
 0 
 0 
 0 
 0 
 0 
 0 
 0 
 0 
 0 
 0 
 0 
 0 
 0 
 0 
 0 
 0 
 0 
 0 
 0 
 0 
 0 
 0 
 0 
 0 
 0 
 0 
 0 
 0 
 0 
 0 
 0 
 0 
 0 
 0 
 0 
 0 
 0 
 0 
 0 
 0 
 0 
 0 
 0 
 0 
 0 
 0 
 0 
 0 
 0 
 0 
 0 
 0 
 0 
 0 
 0 
 0 
 0 
 0 
 0 
 0 
 0 
 0 
 0 
 0 
 0 
 0 
 0 
 0 
 0 
 0 
 0 
 0 
 0 
 0 
 0 
 0 
 0 
 0 
 0 
 0 
 0 
 0 
 0 
 0 
 0 
 0 
 0 
 0 
 0 
 0 
 0 
 0 
 0 
 0 
 0 
 0 
 0 
 0 
 0 
 0 
 0 
 0 
 0 
 0 
 0 
 0 
 0 
 0 
 0 
 0 
 0 
 0 
 0 
 0 
 0 
 0 
 0 
 0 
 0 
 0 
 0 
 0 
 0 
 0 
 0 
 0 
 0 
 0 
 0 
 0 
 0 
 0 
 0 
 0 
 0 
 0 
 0 
 0 
 0 
 0 
 0 
 0 
 0 
 0 
 0 
 0 
 0 
 0 
 0 
 0 
 0 
 0 
 0 
 0 
 0 
 0 
 0 
 0 
 0 
 0 
 0 
 0 
 0 
 0 
 0 
 0 
 0 
 0 
 0 
 0 
 0 
 0 
 0 
 0 
 0 
 0 
 0 
 0 
 0 
 0 
 0 
 0 
 0 
 0 
 0 
 0 
 
 
 102 
 0 
 0 
 0 
 0 
 0 
 0 
 0 
 0 
 0 
 0 
 0 
 0 
 0 
 0 
 0 
 0 
 0 
 0 
 0 
 0 
 0 
 0 
 0 
 0 
 0 
 0 
 0 
 0 
 0 
 0 
 0 
 0 
 0 
 0 
 0 
 0 
 0 
 0 
 0 
 0 
 0 
 0 
 0 
 0 
 0 
 0 
 0 
 0 
 0 
 0 
 0 
 0 
 0 
 0 
 0 
 0 
 0 
 0 
 0 
 0 
 0 
 0 
 0 
 0 
 0 
 0 
 0 
 0 
 0 
 0 
 0 
 0 
 0 
 0 
 0 
 0 
 0 
 0 
 0 
 0 
 0 
 0 
 0 
 0 
 0 
 0 
 0 
 0 
 0 
 0 
 0 
 0 
 0 
 0 
 0 
 0 
 0 
 0 
 0 
 0 
 0 
 0 
 92 
 0 
 0 
 0 
 0 
 0 
 0 
 0 
 0 
 0 
 0 
 0 
 0 
 0 
 0 
 0 
 0 
 0 
 0 
 0 
 0 
 0 
 0 
 0 
 0 
 0 
 0 
 0 
 0 
 0 
 0 
 0 
 0 
 0 
 0 
 0 
 0 
 0 
 0 
 0 
 0 
 0 
 0 
 0 
 0 
 0 
 0 
 0 
 0 
 0 
 0 
 0 
 0 
 0 
 0 
 0 
 0 
 0 
 0 
 0 
 0 
 0 
 0 
 0 
 0 
 0 
 0 
 0 
 0 
 0 
 0 
 0 
 0 
 0 
 0 
 0 
 0 
 0 
 0 
 0 
 0 
 0 
 0 
 0 
 0 
 0 
 0 
 0 
 0 
 0 
 0 
 0 
 0 
 0 
 0 
 0 
 0 
 0 
 0 
 0 
 0 
 0 
 0 
 0 
 0 
 0 
 0 
 0 
 0 
 0 
 0 
 0 
 0 
 0 
 0 
 0 
 0 
 0 
 0 
 0 
 0 
 0 
 0 
 0 
 0 
 0 
 0 
 0 
 0 
 0 
 0 
 0 
 0 
 0 
 0 
 0 
 0 
 0 
 0 
 0 
 0 
 0 
 0 
 0 
 0 
 0 
 0 
 0 
 0 
 0 
 0 
 0 
 0 
 0 
 0 
 0 
 0 
 0 
 0 
 0 
 0 
 0 
 0 
 0 
 0 
 0 
 0 
 0 
 0 
 0 
 0 
 0 
 0 
 0 
 0 
 0 
 0 
 0 
 0 
 0 
 0 
 0 
 0 
 0 
 0 
 0 
 0 
 0 
 0 
 0 
 0 
 0 
 0 
 0 
 0 
 0 
 0 
 0 
 0 
 0 
 0 
 0 
 0 
 0 
 0 
 0 
 0 
 0 
 0 
 0 
 0 
 0 
 0 
 0 
 0 
 0 
 0 
 0 
 0 
 0 
 0 
 0 
 0 
 0 
 0 
 0 
 0 
 0 
 0 
 0 
 0 
 0 
 0 
 0 
 0 
 0 
 0 
 0 
 0 
 0 
 0 
 0 
 0 
 0 
 0 
 0 
 0 
 0 
 0 
 0 
 0 
 0 
 0 
 0 
 0 
 0 
 0 
 0 
 0 
 0 
 0 
 0 
 0 
 0 
 0 
 0 
 0 
 0 
 0 
 0 
 0 
 0 
 0 
 0 
 0 
 0 
 0 
 0 
 0 
 0 
 0 
 0 
 0 
 0 
 0 
 0 
 0 
 0 
 0 
 0 
 0 
 0 
 0 
 0 
 0 
 0 
 0 
 0 
 0 
 0 
 0 
 0 
 0 
 0 
 0 
 0 
 0 
 0 
 0 
 0 
 0 
 0 
 0 
 0 
 0 
 0 
 0 
 0 
 0 
 0 
 0 
 0 
 0 
 0 
 0 
 0 
 0 
 0 
 0 
 0 
 0 
 0 
 0 
 0 
 0 
 0 
 0 
 0 
 0 
 0 
 0 
 0 
 0 
 0 
 0 
 0 
 0 
 0 
 0 
 0 
 0 
 0 
 0 
 0 
 0 
 0 
 0 
 0 
 0 
 0 
 0 
 0 
 0 
 0 
 0 
 0 
 0 
 0 
 0 
 0 
 0 
 0 
 0 
 0 
 0 
 0 
 0 
 0 
 0 
 0 
 0 
 0 
 0 
 0 
 0 
 0 
 0 
 0 
 0 
 0 
 0 
 0 
 0 
 0 
 0 
 0 
 0 
 0 
 0 
 0 
 0 
 0 
 0 
 0 
 0 
 0 
 0 
 0 
 0 
 0 
 0 
 0 
 0 
 0 
 0 
 0 
 0 
 0 
 0 
 0 
 0 
 0 
 0 
 0 
 0 
 0 
 0 
 0 
 0 
 0 
 0 
 0 
 0 
 0 
 0 
 0 
 0 
 0 
 0 
 0 
 0 
 0 
 0 
 0 
 0 
 0 
 0 
 0 
 0 
 0 
 0 
 0 
 0 
 0 
 0 
 0 
 0 
 0 
 0 
 0 
 0 
 0 
 0 
 0 
 0 
 0 
 0 
 0 
 0 
 0 
 0 
 0 
 0 
 0 
 0 
 0 
 0 
 0 
 0 
 0 
 0 
 0 
 0 
 0 
 0 
 0 
 0 
 0 
 0 
 0 
 0 
 0 
 0 
 0 
 0 
 0 
 0 
 0 
 0 
 0 
 0 
 0 
 0 
 0 
 0 
 0 
 0 
 0 
 0 
 0 
 0 
 0 
 0 
 0 
 0 
 0 
 0 
 0 
 0 
 0 
 0 
 0 
 0 
 0 
 0 
 0 
 0 
 0 
 0 
 0 
 0 
 0 
 0 
 0 
 0 
 0 
 0 
 0 
 0 
 24 
 0 
 0 
 0 
 0 
 0 
 0 
 0 
 0 
 0 
 0 
 0 
 0 
 0 
 0 
 0 
 0 
 0 
 0 
 0 
 0 
 0 
 0 
 0 
 0 
 0 
 0 
 0 
 0 
 0 
 0 
 0 
 0 
 0 
 0 
 0 
 0 
 0 
 0 
 0 
 0 
 0 
 0 
 0 
 0 
 0 
 0 
 0 
 0 
 0 
 0 
 0 
 0 
 0 
 0 
 0 
 0 
 0 
 0 
 0 
 0 
 0 
 0 
 0 
 0 
 0 
 0 
 0 
 0 
 0 
 0 
 0 
 0 
 0 
 0 
 0 
 0 
 0 
 0 
 0 
 0 
 0 
 0 
 0 
 0 
 0 
 0 
 0 
 0 
 0 
 0 
 0 
 0 
 0 
 0 
 0 
 0 
 0 
 0 
 0 
 0 
 0 
 0 
 0 
 0 
 0 
 0 
 0 
 0 
 0 
 0 
 0 
 0 
 0 
 0 
 0 
 0 
 0 
 0 
 0 
 0 
 0 
 0 
 0 
 0 
 0 
 0 
 0 
 0 
 0 
 0 
 0 
 0 
 0 
 0 
 0 
 0 
 0 
 0 
 0 
 0 
 0 
 0 
 0 
 0 
 0 
 0 
 0 
 0 
 0 
 0 
 0 
 0 
 0 
 0 
 0 
 0 
 0 
 0 
 0 
 0 
 0 
 0 
 0 
 0 
 0 
 0 
 0 
 0 
 0 
 0 
 0 
 0 
 0 
 0 
 0 
 0 
 0 
 0 
 0 
 0 
 0 
 0 
 0 
 0 
 
 
 103 
 0 
 0 
 0 
 0 
 0 
 0 
 0 
 0 
 0 
 0 
 0 
 0 
 0 
 0 
 0 
 0 
 0 
 0 
 0 
 0 
 0 
 0 
 0 
 0 
 0 
 0 
 0 
 0 
 0 
 0 
 0 
 0 
 0 
 0 
 0 
 0 
 0 
 0 
 0 
 0 
 0 
 0 
 0 
 0 
 0 
 0 
 0 
 0 
 0 
 0 
 0 
 0 
 0 
 0 
 0 
 0 
 0 
 0 
 0 
 0 
 0 
 0 
 0 
 0 
 0 
 0 
 0 
 0 
 0 
 0 
 0 
 0 
 0 
 0 
 0 
 0 
 0 
 0 
 0 
 0 
 0 
 0 
 0 
 0 
 0 
 0 
 0 
 0 
 0 
 0 
 0 
 0 
 0 
 0 
 0 
 0 
 0 
 0 
 0 
 0 
 0 
 0 
 0 
 2 
 0 
 0 
 0 
 0 
 0 
 0 
 0 
 0 
 0 
 0 
 0 
 0 
 0 
 0 
 0 
 0 
 0 
 0 
 0 
 0 
 0 
 0 
 0 
 0 
 0 
 0 
 0 
 0 
 0 
 0 
 0 
 0 
 0 
 0 
 0 
 0 
 0 
 0 
 0 
 0 
 0 
 0 
 0 
 0 
 0 
 0 
 0 
 0 
 0 
 0 
 0 
 0 
 0 
 0 
 0 
 0 
 0 
 0 
 0 
 0 
 0 
 0 
 0 
 0 
 0 
 0 
 0 
 0 
 0 
 0 
 0 
 0 
 0 
 0 
 0 
 0 
 0 
 0 
 0 
 0 
 0 
 0 
 0 
 0 
 0 
 0 
 0 
 0 
 0 
 0 
 0 
 0 
 0 
 0 
 0 
 0 
 0 
 0 
 0 
 0 
 0 
 0 
 0 
 0 
 0 
 0 
 0 
 0 
 0 
 0 
 0 
 0 
 0 
 0 
 0 
 0 
 0 
 0 
 0 
 0 
 0 
 0 
 0 
 0 
 0 
 0 
 0 
 0 
 0 
 0 
 0 
 0 
 0 
 0 
 0 
 0 
 0 
 0 
 0 
 0 
 0 
 0 
 0 
 0 
 0 
 0 
 0 
 0 
 0 
 0 
 0 
 0 
 0 
 0 
 0 
 0 
 0 
 0 
 0 
 0 
 0 
 0 
 0 
 0 
 0 
 0 
 0 
 0 
 0 
 0 
 0 
 0 
 0 
 0 
 0 
 0 
 0 
 0 
 0 
 0 
 0 
 0 
 0 
 0 
 0 
 0 
 0 
 0 
 0 
 0 
 0 
 0 
 0 
 0 
 0 
 0 
 0 
 0 
 0 
 0 
 0 
 0 
 0 
 0 
 0 
 0 
 0 
 0 
 0 
 0 
 0 
 0 
 0 
 0 
 0 
 0 
 0 
 0 
 0 
 0 
 0 
 0 
 0 
 0 
 0 
 0 
 0 
 0 
 0 
 0 
 0 
 0 
 0 
 0 
 0 
 0 
 0 
 0 
 0 
 0 
 0 
 0 
 0 
 0 
 0 
 0 
 0 
 0 
 0 
 0 
 0 
 0 
 0 
 0 
 0 
 0 
 0 
 0 
 0 
 0 
 0 
 0 
 0 
 0 
 0 
 0 
 0 
 0 
 0 
 0 
 0 
 0 
 0 
 0 
 0 
 0 
 0 
 0 
 0 
 0 
 0 
 0 
 0 
 0 
 0 
 0 
 0 
 0 
 0 
 0 
 0 
 0 
 0 
 0 
 0 
 0 
 0 
 0 
 0 
 0 
 0 
 0 
 0 
 0 
 0 
 0 
 0 
 0 
 0 
 0 
 0 
 0 
 0 
 0 
 0 
 0 
 0 
 0 
 0 
 0 
 0 
 0 
 0 
 0 
 0 
 0 
 0 
 0 
 0 
 0 
 0 
 0 
 0 
 0 
 0 
 0 
 0 
 0 
 0 
 0 
 0 
 0 
 0 
 0 
 0 
 0 
 0 
 0 
 0 
 0 
 0 
 0 
 0 
 0 
 0 
 0 
 0 
 0 
 0 
 0 
 0 
 0 
 0 
 0 
 0 
 0 
 0 
 0 
 0 
 0 
 0 
 0 
 0 
 0 
 0 
 0 
 0 
 0 
 0 
 0 
 0 
 0 
 0 
 0 
 0 
 0 
 0 
 0 
 0 
 0 
 0 
 0 
 0 
 0 
 0 
 0 
 0 
 0 
 0 
 0 
 0 
 0 
 0 
 0 
 0 
 0 
 0 
 0 
 0 
 0 
 0 
 0 
 0 
 0 
 0 
 0 
 0 
 0 
 0 
 0 
 0 
 0 
 0 
 0 
 0 
 0 
 0 
 0 
 0 
 0 
 0 
 0 
 0 
 0 
 0 
 0 
 0 
 0 
 0 
 0 
 0 
 0 
 0 
 0 
 0 
 0 
 0 
 0 
 0 
 0 
 0 
 0 
 0 
 0 
 0 
 0 
 0 
 0 
 0 
 0 
 0 
 0 
 0 
 0 
 0 
 0 
 0 
 0 
 0 
 0 
 0 
 0 
 0 
 0 
 0 
 0 
 0 
 0 
 0 
 0 
 0 
 0 
 0 
 0 
 0 
 0 
 0 
 0 
 0 
 0 
 0 
 0 
 0 
 0 
 0 
 0 
 0 
 0 
 0 
 0 
 0 
 0 
 0 
 0 
 0 
 0 
 0 
 0 
 0 
 0 
 0 
 0 
 0 
 0 
 0 
 0 
 0 
 0 
 0 
 0 
 0 
 0 
 0 
 0 
 0 
 0 
 0 
 0 
 0 
 0 
 0 
 0 
 0 
 0 
 0 
 0 
 0 
 0 
 0 
 0 
 0 
 0 
 0 
 0 
 0 
 0 
 0 
 0 
 0 
 0 
 0 
 0 
 0 
 0 
 0 
 0 
 0 
 0 
 0 
 0 
 0 
 0 
 0 
 0 
 0 
 0 
 0 
 0 
 0 
 0 
 0 
 0 
 0 
 0 
 0 
 0 
 0 
 0 
 0 
 0 
 0 
 0 
 0 
 0 
 0 
 0 
 0 
 0 
 0 
 0 
 0 
 0 
 0 
 0 
 0 
 0 
 0 
 0 
 0 
 0 
 0 
 0 
 0 
 0 
 0 
 0 
 0 
 0 
 0 
 0 
 0 
 0 
 0 
 0 
 0 
 0 
 0 
 0 
 0 
 0 
 0 
 0 
 0 
 0 
 0 
 0 
 0 
 0 
 0 
 0 
 0 
 0 
 0 
 0 
 0 
 0 
 0 
 0 
 0 
 0 
 0 
 0 
 0 
 0 
 0 
 0 
 0 
 0 
 0 
 0 
 0 
 0 
 0 
 0 
 0 
 0 
 0 
 0 
 0 
 0 
 0 
 0 
 0 
 0 
 0 
 0 
 0 
 0 
 0 
 0 
 0 
 0 
 0 
 0 
 0 
 0 
 0 
 0 
 0 
 0 
 0 
 0 
 0 
 0 
 0 
 0 
 0 
 0 
 0 
 0 
 0 
 0 
 0 
 0 
 0 
 0 
 0 
 0 
 0 
 0 
 0 
 0 
 0 
 0 
 0 
 0 
 0 
 0 
 0 
 0 
 0 
 0 
 0 
 0 
 0 
 0 
 0 
 0 
 0 
 0 
 0 
 0 
 
 
 104 
 0 
 0 
 0 
 0 
 0 
 0 
 0 
 0 
 0 
 0 
 0 
 0 
 0 
 0 
 0 
 0 
 0 
 0 
 0 
 0 
 0 
 0 
 0 
 0 
 0 
 0 
 0 
 0 
 0 
 0 
 0 
 0 
 0 
 0 
 0 
 0 
 0 
 0 
 0 
 0 
 0 
 0 
 0 
 0 
 0 
 0 
 0 
 0 
 0 
 0 
 0 
 0 
 0 
 0 
 0 
 0 
 0 
 0 
 0 
 0 
 0 
 0 
 0 
 0 
 0 
 0 
 0 
 0 
 0 
 0 
 0 
 0 
 0 
 0 
 0 
 0 
 0 
 0 
 0 
 0 
 0 
 0 
 0 
 0 
 0 
 0 
 0 
 0 
 0 
 0 
 0 
 0 
 0 
 0 
 0 
 0 
 0 
 0 
 0 
 0 
 0 
 0 
 0 
 0 
 14 
 0 
 0 
 0 
 0 
 0 
 0 
 0 
 0 
 0 
 0 
 0 
 0 
 0 
 0 
 0 
 0 
 0 
 0 
 0 
 0 
 0 
 0 
 0 
 0 
 0 
 0 
 0 
 0 
 0 
 0 
 0 
 0 
 0 
 0 
 0 
 0 
 0 
 0 
 0 
 0 
 0 
 0 
 0 
 0 
 0 
 0 
 0 
 0 
 0 
 0 
 0 
 0 
 0 
 0 
 0 
 0 
 0 
 0 
 0 
 30 
 0 
 0 
 0 
 0 
 0 
 0 
 0 
 0 
 0 
 0 
 0 
 0 
 0 
 0 
 0 
 0 
 0 
 0 
 0 
 0 
 0 
 0 
 0 
 0 
 0 
 0 
 0 
 0 
 0 
 0 
 0 
 0 
 0 
 0 
 0 
 0 
 0 
 0 
 0 
 0 
 0 
 0 
 0 
 0 
 0 
 0 
 0 
 0 
 0 
 0 
 0 
 0 
 0 
 0 
 1 
 0 
 0 
 0 
 0 
 0 
 0 
 0 
 0 
 0 
 0 
 0 
 0 
 0 
 0 
 0 
 0 
 0 
 0 
 0 
 0 
 0 
 0 
 0 
 0 
 0 
 0 
 0 
 0 
 0 
 0 
 0 
 0 
 0 
 0 
 0 
 0 
 0 
 0 
 0 
 0 
 0 
 0 
 0 
 0 
 0 
 0 
 0 
 0 
 0 
 0 
 0 
 0 
 0 
 0 
 0 
 0 
 0 
 0 
 0 
 0 
 0 
 0 
 0 
 0 
 0 
 0 
 0 
 0 
 0 
 0 
 0 
 0 
 0 
 0 
 0 
 0 
 0 
 0 
 0 
 0 
 0 
 0 
 0 
 0 
 0 
 0 
 0 
 0 
 0 
 0 
 0 
 0 
 0 
 0 
 0 
 0 
 0 
 0 
 0 
 0 
 0 
 0 
 0 
 0 
 0 
 0 
 0 
 0 
 0 
 0 
 0 
 0 
 0 
 0 
 0 
 0 
 0 
 0 
 0 
 0 
 0 
 0 
 0 
 0 
 0 
 0 
 0 
 0 
 0 
 0 
 0 
 0 
 0 
 0 
 0 
 0 
 0 
 0 
 0 
 0 
 0 
 0 
 0 
 0 
 0 
 0 
 0 
 0 
 0 
 0 
 0 
 0 
 0 
 0 
 0 
 0 
 0 
 0 
 0 
 0 
 0 
 0 
 0 
 0 
 0 
 0 
 0 
 0 
 0 
 0 
 0 
 0 
 0 
 0 
 0 
 0 
 0 
 0 
 0 
 0 
 0 
 0 
 0 
 0 
 0 
 0 
 0 
 0 
 0 
 0 
 0 
 0 
 0 
 0 
 0 
 0 
 0 
 0 
 0 
 0 
 0 
 0 
 0 
 0 
 0 
 0 
 0 
 0 
 0 
 0 
 0 
 0 
 0 
 0 
 0 
 0 
 0 
 0 
 0 
 0 
 0 
 0 
 0 
 0 
 0 
 0 
 0 
 0 
 0 
 0 
 0 
 0 
 0 
 0 
 0 
 0 
 0 
 0 
 0 
 0 
 0 
 0 
 0 
 0 
 0 
 0 
 0 
 0 
 0 
 0 
 0 
 0 
 0 
 0 
 0 
 0 
 0 
 0 
 0 
 0 
 0 
 0 
 0 
 0 
 0 
 0 
 0 
 0 
 0 
 0 
 0 
 0 
 0 
 0 
 0 
 0 
 0 
 0 
 0 
 0 
 0 
 0 
 0 
 0 
 0 
 0 
 0 
 0 
 0 
 0 
 0 
 0 
 0 
 0 
 0 
 0 
 0 
 0 
 0 
 0 
 0 
 0 
 0 
 0 
 0 
 0 
 0 
 0 
 0 
 0 
 0 
 0 
 0 
 0 
 0 
 0 
 0 
 0 
 0 
 0 
 0 
 0 
 0 
 0 
 0 
 0 
 0 
 0 
 0 
 0 
 0 
 0 
 0 
 0 
 0 
 0 
 0 
 0 
 0 
 0 
 0 
 0 
 0 
 0 
 0 
 0 
 0 
 0 
 0 
 0 
 0 
 0 
 0 
 0 
 0 
 0 
 0 
 0 
 0 
 0 
 0 
 0 
 0 
 0 
 0 
 0 
 0 
 0 
 0 
 0 
 0 
 0 
 0 
 0 
 0 
 0 
 0 
 0 
 0 
 0 
 0 
 0 
 0 
 0 
 0 
 0 
 0 
 0 
 0 
 0 
 0 
 0 
 0 
 0 
 0 
 0 
 0 
 0 
 0 
 0 
 0 
 0 
 0 
 0 
 0 
 0 
 0 
 0 
 0 
 0 
 0 
 0 
 0 
 0 
 0 
 0 
 0 
 0 
 0 
 0 
 0 
 0 
 0 
 0 
 0 
 0 
 0 
 0 
 0 
 0 
 0 
 0 
 0 
 0 
 0 
 0 
 0 
 0 
 0 
 0 
 0 
 0 
 0 
 0 
 0 
 0 
 0 
 0 
 0 
 0 
 0 
 0 
 0 
 0 
 0 
 0 
 0 
 0 
 0 
 0 
 0 
 0 
 0 
 0 
 0 
 0 
 0 
 0 
 0 
 0 
 0 
 0 
 0 
 0 
 0 
 0 
 0 
 0 
 0 
 0 
 0 
 0 
 0 
 0 
 0 
 0 
 0 
 0 
 0 
 0 
 0 
 0 
 0 
 0 
 0 
 0 
 0 
 0 
 0 
 0 
 0 
 0 
 0 
 0 
 0 
 0 
 0 
 0 
 0 
 0 
 0 
 0 
 0 
 0 
 0 
 0 
 0 
 0 
 0 
 0 
 0 
 0 
 0 
 0 
 0 
 0 
 0 
 0 
 0 
 0 
 0 
 0 
 0 
 0 
 0 
 0 
 0 
 0 
 0 
 0 
 0 
 0 
 0 
 0 
 0 
 0 
 0 
 0 
 0 
 0 
 0 
 0 
 0 
 0 
 0 
 0 
 0 
 0 
 0 
 0 
 0 
 0 
 0 
 0 
 0 
 0 
 0 
 0 
 0 
 0 
 0 
 0 
 0 
 0 
 0 
 0 
 0 
 0 
 0 
 0 
 0 
 0 
 0 
 0 
 0 
 0 
 0 
 0 
 0 
 0 
 0 
 0 
 0 
 0 
 0 
 0 
 0 
 0 
 0 
 0 
 0 
 0 
 0 
 0 
 0 
 0 
 
 
 105 
 0 
 0 
 0 
 0 
 0 
 0 
 0 
 0 
 0 
 0 
 0 
 0 
 0 
 0 
 0 
 0 
 0 
 0 
 0 
 0 
 0 
 0 
 0 
 0 
 0 
 0 
 0 
 0 
 0 
 0 
 0 
 0 
 0 
 0 
 0 
 0 
 0 
 0 
 0 
 0 
 0 
 0 
 0 
 0 
 0 
 0 
 0 
 0 
 0 
 0 
 0 
 0 
 0 
 0 
 0 
 0 
 0 
 0 
 0 
 0 
 0 
 0 
 0 
 0 
 0 
 0 
 0 
 0 
 0 
 0 
 0 
 0 
 0 
 0 
 0 
 0 
 0 
 0 
 0 
 0 
 0 
 0 
 0 
 0 
 0 
 0 
 0 
 0 
 0 
 0 
 0 
 0 
 0 
 0 
 0 
 0 
 0 
 0 
 0 
 0 
 0 
 0 
 0 
 0 
 0 
 7 
 0 
 0 
 0 
 0 
 0 
 0 
 0 
 0 
 0 
 0 
 0 
 0 
 0 
 0 
 0 
 0 
 0 
 0 
 0 
 0 
 0 
 0 
 0 
 0 
 0 
 0 
 0 
 0 
 0 
 0 
 0 
 0 
 0 
 0 
 0 
 0 
 0 
 0 
 0 
 0 
 0 
 0 
 0 
 0 
 0 
 0 
 0 
 0 
 0 
 0 
 0 
 0 
 0 
 0 
 0 
 0 
 0 
 0 
 0 
 0 
 0 
 0 
 0 
 0 
 0 
 0 
 0 
 0 
 0 
 0 
 0 
 0 
 0 
 0 
 0 
 0 
 0 
 0 
 0 
 0 
 0 
 0 
 0 
 0 
 0 
 0 
 0 
 0 
 0 
 0 
 0 
 0 
 0 
 0 
 0 
 0 
 0 
 0 
 0 
 0 
 0 
 0 
 0 
 0 
 0 
 0 
 0 
 0 
 0 
 0 
 0 
 0 
 0 
 0 
 0 
 0 
 0 
 0 
 0 
 0 
 0 
 0 
 0 
 0 
 0 
 0 
 0 
 0 
 0 
 0 
 0 
 0 
 0 
 0 
 0 
 0 
 0 
 0 
 0 
 0 
 0 
 0 
 0 
 0 
 0 
 0 
 0 
 0 
 0 
 0 
 0 
 0 
 0 
 0 
 0 
 0 
 0 
 0 
 0 
 0 
 0 
 0 
 0 
 0 
 0 
 0 
 0 
 0 
 0 
 0 
 0 
 0 
 0 
 0 
 0 
 0 
 0 
 0 
 0 
 0 
 0 
 0 
 0 
 0 
 0 
 0 
 0 
 0 
 0 
 0 
 0 
 0 
 0 
 0 
 0 
 0 
 0 
 0 
 0 
 0 
 0 
 0 
 0 
 0 
 0 
 0 
 0 
 0 
 0 
 0 
 0 
 0 
 0 
 0 
 0 
 0 
 0 
 0 
 0 
 0 
 0 
 0 
 0 
 0 
 0 
 0 
 0 
 0 
 0 
 0 
 0 
 0 
 0 
 0 
 0 
 0 
 0 
 0 
 0 
 0 
 0 
 0 
 0 
 0 
 0 
 0 
 0 
 0 
 0 
 0 
 0 
 0 
 0 
 0 
 0 
 0 
 0 
 0 
 0 
 0 
 0 
 0 
 0 
 0 
 0 
 0 
 0 
 0 
 0 
 0 
 0 
 0 
 0 
 0 
 0 
 0 
 0 
 0 
 0 
 0 
 0 
 0 
 0 
 0 
 0 
 0 
 0 
 0 
 0 
 0 
 0 
 0 
 0 
 0 
 0 
 0 
 0 
 0 
 0 
 0 
 0 
 0 
 0 
 0 
 0 
 0 
 0 
 0 
 0 
 0 
 0 
 0 
 0 
 0 
 0 
 0 
 0 
 0 
 0 
 0 
 0 
 0 
 0 
 0 
 0 
 0 
 0 
 0 
 0 
 0 
 0 
 0 
 0 
 0 
 0 
 0 
 0 
 0 
 0 
 0 
 0 
 0 
 0 
 0 
 0 
 0 
 0 
 0 
 0 
 0 
 0 
 0 
 0 
 0 
 0 
 0 
 0 
 0 
 0 
 0 
 0 
 0 
 0 
 0 
 0 
 0 
 0 
 0 
 0 
 0 
 0 
 0 
 0 
 0 
 0 
 0 
 0 
 0 
 0 
 0 
 0 
 0 
 0 
 0 
 0 
 0 
 0 
 0 
 0 
 0 
 0 
 0 
 0 
 0 
 0 
 0 
 0 
 0 
 0 
 0 
 0 
 0 
 0 
 0 
 0 
 0 
 0 
 0 
 0 
 0 
 0 
 0 
 0 
 0 
 0 
 0 
 0 
 0 
 0 
 0 
 0 
 0 
 0 
 0 
 0 
 0 
 0 
 0 
 0 
 0 
 0 
 0 
 0 
 0 
 0 
 0 
 0 
 0 
 0 
 0 
 0 
 0 
 0 
 0 
 0 
 0 
 0 
 0 
 0 
 0 
 0 
 0 
 0 
 0 
 0 
 0 
 0 
 0 
 0 
 0 
 0 
 0 
 0 
 0 
 0 
 0 
 0 
 0 
 0 
 0 
 0 
 0 
 0 
 0 
 0 
 0 
 0 
 0 
 0 
 0 
 0 
 0 
 0 
 0 
 0 
 0 
 0 
 0 
 0 
 0 
 0 
 0 
 0 
 0 
 0 
 0 
 0 
 0 
 0 
 0 
 0 
 0 
 0 
 0 
 0 
 0 
 0 
 0 
 0 
 0 
 0 
 0 
 0 
 0 
 0 
 0 
 0 
 0 
 0 
 0 
 0 
 0 
 0 
 0 
 0 
 0 
 0 
 0 
 0 
 0 
 0 
 0 
 0 
 0 
 0 
 0 
 0 
 0 
 0 
 0 
 0 
 0 
 0 
 0 
 0 
 0 
 0 
 0 
 0 
 0 
 0 
 0 
 0 
 0 
 0 
 0 
 0 
 0 
 0 
 0 
 0 
 0 
 0 
 0 
 0 
 0 
 0 
 0 
 0 
 0 
 0 
 0 
 0 
 0 
 0 
 0 
 0 
 0 
 0 
 0 
 0 
 0 
 0 
 0 
 0 
 0 
 0 
 0 
 0 
 0 
 0 
 0 
 0 
 0 
 0 
 0 
 0 
 0 
 0 
 0 
 0 
 0 
 0 
 0 
 0 
 0 
 0 
 0 
 0 
 0 
 0 
 0 
 0 
 0 
 0 
 0 
 0 
 0 
 0 
 0 
 0 
 0 
 0 
 0 
 0 
 0 
 0 
 0 
 0 
 0 
 0 
 0 
 0 
 0 
 0 
 0 
 0 
 0 
 0 
 0 
 0 
 0 
 0 
 0 
 0 
 0 
 0 
 0 
 0 
 0 
 0 
 0 
 0 
 0 
 0 
 0 
 0 
 0 
 0 
 0 
 0 
 0 
 0 
 0 
 0 
 0 
 0 
 0 
 0 
 0 
 0 
 0 
 0 
 0 
 0 
 0 
 0 
 0 
 0 
 0 
 0 
 0 
 0 
 0 
 0 
 0 
 0 
 0 
 0 
 0 
 0 
 0 
 0 
 0 
 0 
 0 
 0 
 0 
 0 
 0 
 0 
 0 
 0 
 0 
 0 
 0 
 0 
 0 
 0 
 0 
 0 
 0 
 0 
 0 
 0 
 0 
 0 
 0 
 0 
 0 
 
 
 106 
 0 
 0 
 0 
 0 
 0 
 0 
 0 
 0 
 0 
 0 
 0 
 0 
 0 
 0 
 0 
 0 
 0 
 0 
 0 
 0 
 0 
 0 
 0 
 0 
 0 
 0 
 0 
 0 
 0 
 0 
 0 
 0 
 0 
 0 
 0 
 0 
 0 
 0 
 0 
 0 
 0 
 0 
 0 
 0 
 0 
 0 
 0 
 0 
 0 
 0 
 0 
 0 
 0 
 0 
 0 
 0 
 0 
 0 
 0 
 0 
 0 
 0 
 0 
 0 
 0 
 0 
 0 
 0 
 0 
 0 
 0 
 0 
 0 
 0 
 0 
 0 
 0 
 0 
 0 
 0 
 0 
 0 
 0 
 0 
 0 
 0 
 0 
 0 
 0 
 0 
 0 
 0 
 0 
 0 
 0 
 0 
 0 
 0 
 0 
 0 
 0 
 0 
 0 
 0 
 0 
 0 
 0 
 0 
 0 
 0 
 0 
 0 
 0 
 3 
 0 
 0 
 0 
 0 
 0 
 0 
 0 
 0 
 0 
 0 
 0 
 0 
 0 
 0 
 0 
 0 
 0 
 0 
 0 
 0 
 0 
 0 
 0 
 0 
 0 
 0 
 0 
 0 
 0 
 0 
 0 
 0 
 0 
 0 
 0 
 0 
 0 
 0 
 0 
 0 
 0 
 0 
 0 
 0 
 0 
 0 
 0 
 0 
 0 
 0 
 0 
 0 
 0 
 0 
 0 
 0 
 0 
 0 
 0 
 0 
 0 
 0 
 0 
 0 
 0 
 0 
 0 
 0 
 0 
 0 
 0 
 0 
 0 
 0 
 0 
 0 
 0 
 0 
 0 
 0 
 0 
 0 
 0 
 0 
 0 
 0 
 0 
 0 
 0 
 0 
 0 
 0 
 0 
 0 
 0 
 0 
 0 
 0 
 0 
 0 
 0 
 0 
 0 
 0 
 0 
 0 
 0 
 0 
 0 
 0 
 0 
 0 
 0 
 0 
 0 
 0 
 0 
 0 
 0 
 0 
 0 
 0 
 0 
 0 
 0 
 0 
 0 
 0 
 0 
 0 
 0 
 0 
 0 
 0 
 0 
 0 
 0 
 0 
 0 
 0 
 0 
 0 
 0 
 0 
 0 
 0 
 0 
 0 
 0 
 0 
 0 
 0 
 0 
 0 
 0 
 0 
 0 
 0 
 0 
 0 
 0 
 0 
 0 
 0 
 0 
 0 
 0 
 0 
 0 
 0 
 0 
 0 
 0 
 0 
 0 
 0 
 0 
 0 
 0 
 0 
 0 
 0 
 0 
 0 
 0 
 0 
 0 
 0 
 0 
 0 
 0 
 0 
 0 
 0 
 0 
 0 
 0 
 0 
 0 
 0 
 0 
 0 
 0 
 0 
 0 
 0 
 0 
 0 
 0 
 0 
 0 
 0 
 0 
 0 
 0 
 0 
 0 
 0 
 0 
 0 
 0 
 0 
 0 
 0 
 0 
 0 
 0 
 0 
 0 
 0 
 0 
 0 
 0 
 0 
 0 
 0 
 0 
 0 
 0 
 0 
 0 
 0 
 0 
 0 
 0 
 0 
 0 
 0 
 0 
 0 
 0 
 0 
 0 
 0 
 0 
 0 
 0 
 0 
 0 
 0 
 0 
 0 
 0 
 0 
 0 
 0 
 0 
 0 
 0 
 0 
 0 
 0 
 0 
 0 
 0 
 0 
 0 
 0 
 0 
 0 
 0 
 0 
 0 
 0 
 0 
 0 
 0 
 0 
 0 
 0 
 0 
 0 
 0 
 0 
 0 
 0 
 0 
 0 
 0 
 0 
 0 
 0 
 0 
 0 
 0 
 0 
 0 
 0 
 0 
 0 
 0 
 0 
 0 
 0 
 0 
 0 
 0 
 0 
 0 
 0 
 0 
 0 
 0 
 0 
 0 
 0 
 0 
 0 
 0 
 0 
 0 
 0 
 0 
 0 
 0 
 0 
 0 
 0 
 0 
 0 
 0 
 0 
 0 
 0 
 0 
 0 
 0 
 0 
 0 
 0 
 0 
 0 
 0 
 0 
 0 
 0 
 0 
 0 
 0 
 0 
 0 
 0 
 0 
 0 
 0 
 0 
 0 
 0 
 0 
 0 
 0 
 0 
 0 
 0 
 0 
 0 
 0 
 0 
 0 
 0 
 0 
 0 
 0 
 0 
 0 
 0 
 0 
 0 
 0 
 0 
 0 
 0 
 0 
 0 
 0 
 0 
 0 
 0 
 0 
 0 
 0 
 0 
 0 
 0 
 0 
 0 
 0 
 0 
 0 
 0 
 0 
 0 
 0 
 0 
 0 
 0 
 0 
 0 
 0 
 0 
 0 
 0 
 0 
 0 
 0 
 0 
 0 
 0 
 0 
 0 
 0 
 0 
 0 
 0 
 0 
 0 
 0 
 0 
 0 
 0 
 0 
 0 
 0 
 0 
 0 
 0 
 0 
 0 
 0 
 0 
 0 
 0 
 0 
 0 
 0 
 0 
 0 
 0 
 0 
 0 
 0 
 0 
 0 
 0 
 0 
 0 
 0 
 0 
 0 
 0 
 0 
 0 
 0 
 0 
 0 
 0 
 0 
 0 
 0 
 0 
 0 
 0 
 0 
 0 
 0 
 0 
 0 
 0 
 0 
 0 
 0 
 0 
 0 
 0 
 0 
 0 
 0 
 0 
 0 
 0 
 0 
 0 
 0 
 0 
 0 
 0 
 0 
 0 
 0 
 0 
 0 
 0 
 0 
 0 
 0 
 0 
 0 
 0 
 0 
 0 
 0 
 0 
 0 
 0 
 0 
 0 
 0 
 0 
 0 
 0 
 0 
 0 
 0 
 0 
 0 
 0 
 0 
 0 
 0 
 0 
 0 
 0 
 0 
 0 
 0 
 0 
 0 
 0 
 0 
 0 
 0 
 0 
 0 
 0 
 0 
 0 
 0 
 0 
 0 
 0 
 0 
 0 
 0 
 0 
 0 
 0 
 0 
 0 
 0 
 0 
 0 
 0 
 0 
 0 
 0 
 0 
 0 
 0 
 0 
 0 
 0 
 0 
 0 
 0 
 0 
 0 
 0 
 0 
 0 
 0 
 0 
 0 
 0 
 0 
 0 
 0 
 0 
 0 
 0 
 0 
 0 
 0 
 0 
 0 
 0 
 0 
 0 
 0 
 0 
 0 
 0 
 0 
 0 
 0 
 0 
 0 
 0 
 0 
 0 
 0 
 0 
 0 
 0 
 0 
 0 
 0 
 0 
 0 
 0 
 0 
 0 
 0 
 0 
 0 
 0 
 0 
 0 
 0 
 0 
 0 
 0 
 0 
 0 
 0 
 0 
 0 
 0 
 0 
 0 
 0 
 0 
 0 
 0 
 0 
 0 
 0 
 0 
 0 
 0 
 0 
 0 
 0 
 0 
 0 
 0 
 0 
 0 
 0 
 0 
 0 
 0 
 0 
 0 
 0 
 0 
 0 
 0 
 0 
 0 
 0 
 0 
 0 
 0 
 0 
 0 
 0 
 0 
 0 
 0 
 0 
 0 
 0 
 0 
 0 
 0 
 0 
 0 
 0 
 0 
 0 
 0 
 0 
 0 
 0 
 0 
 0 
 0 
 0 
 0 
 0 
 0 
 0 
 
 
 107 
 0 
 0 
 0 
 0 
 0 
 0 
 0 
 0 
 0 
 0 
 0 
 0 
 0 
 0 
 0 
 0 
 0 
 0 
 0 
 4 
 0 
 0 
 0 
 0 
 0 
 0 
 0 
 0 
 0 
 0 
 0 
 0 
 0 
 0 
 0 
 0 
 0 
 0 
 0 
 0 
 0 
 0 
 0 
 0 
 0 
 0 
 0 
 0 
 0 
 0 
 0 
 0 
 0 
 0 
 0 
 0 
 0 
 0 
 0 
 0 
 0 
 0 
 0 
 0 
 0 
 0 
 0 
 0 
 0 
 0 
 0 
 0 
 0 
 0 
 0 
 0 
 0 
 0 
 0 
 0 
 0 
 0 
 0 
 0 
 0 
 0 
 0 
 0 
 0 
 0 
 0 
 0 
 0 
 0 
 0 
 0 
 0 
 0 
 0 
 0 
 0 
 0 
 0 
 0 
 0 
 0 
 0 
 0 
 0 
 0 
 0 
 0 
 0 
 0 
 0 
 0 
 0 
 0 
 0 
 0 
 0 
 0 
 0 
 0 
 0 
 0 
 0 
 0 
 0 
 0 
 0 
 0 
 0 
 0 
 0 
 0 
 0 
 0 
 0 
 0 
 0 
 0 
 0 
 0 
 0 
 0 
 0 
 0 
 0 
 0 
 0 
 0 
 0 
 0 
 0 
 0 
 0 
 0 
 0 
 0 
 0 
 0 
 0 
 0 
 0 
 0 
 0 
 0 
 0 
 0 
 0 
 0 
 0 
 0 
 0 
 0 
 0 
 0 
 0 
 0 
 0 
 0 
 0 
 0 
 0 
 0 
 0 
 0 
 0 
 0 
 0 
 0 
 0 
 0 
 0 
 0 
 0 
 0 
 0 
 0 
 0 
 0 
 0 
 0 
 0 
 0 
 0 
 0 
 0 
 0 
 0 
 0 
 0 
 0 
 0 
 0 
 0 
 0 
 0 
 0 
 0 
 0 
 0 
 0 
 0 
 0 
 0 
 0 
 0 
 0 
 0 
 0 
 0 
 0 
 0 
 0 
 0 
 0 
 0 
 0 
 0 
 0 
 0 
 0 
 0 
 0 
 0 
 0 
 0 
 0 
 0 
 0 
 0 
 0 
 0 
 0 
 0 
 0 
 0 
 0 
 0 
 0 
 0 
 0 
 0 
 0 
 0 
 0 
 0 
 0 
 0 
 0 
 0 
 0 
 0 
 0 
 0 
 0 
 0 
 0 
 0 
 0 
 0 
 0 
 0 
 0 
 0 
 0 
 0 
 0 
 0 
 0 
 0 
 0 
 0 
 0 
 0 
 0 
 0 
 0 
 0 
 0 
 0 
 0 
 0 
 0 
 0 
 0 
 0 
 0 
 0 
 0 
 0 
 0 
 0 
 0 
 0 
 0 
 0 
 0 
 0 
 0 
 0 
 0 
 0 
 0 
 0 
 0 
 0 
 0 
 0 
 0 
 0 
 0 
 0 
 0 
 0 
 0 
 0 
 0 
 0 
 0 
 0 
 0 
 0 
 0 
 0 
 0 
 0 
 0 
 0 
 0 
 0 
 0 
 0 
 0 
 0 
 0 
 0 
 0 
 0 
 0 
 0 
 0 
 0 
 0 
 0 
 0 
 0 
 0 
 0 
 0 
 0 
 0 
 0 
 0 
 0 
 0 
 0 
 0 
 0 
 0 
 0 
 0 
 0 
 0 
 0 
 0 
 0 
 0 
 0 
 0 
 0 
 0 
 0 
 0 
 0 
 0 
 0 
 0 
 0 
 0 
 0 
 0 
 0 
 0 
 0 
 0 
 0 
 0 
 0 
 0 
 0 
 0 
 0 
 0 
 0 
 0 
 0 
 0 
 0 
 0 
 0 
 0 
 0 
 0 
 0 
 0 
 0 
 0 
 0 
 0 
 0 
 0 
 0 
 0 
 0 
 0 
 0 
 0 
 0 
 0 
 0 
 0 
 0 
 0 
 0 
 0 
 0 
 0 
 0 
 0 
 0 
 0 
 0 
 0 
 0 
 0 
 0 
 0 
 0 
 0 
 0 
 0 
 0 
 0 
 0 
 0 
 0 
 0 
 0 
 0 
 0 
 0 
 0 
 0 
 0 
 0 
 0 
 0 
 0 
 0 
 0 
 0 
 0 
 0 
 0 
 0 
 0 
 0 
 0 
 0 
 0 
 0 
 0 
 0 
 0 
 0 
 0 
 0 
 0 
 0 
 0 
 0 
 0 
 0 
 0 
 0 
 0 
 0 
 0 
 0 
 0 
 0 
 0 
 0 
 0 
 0 
 0 
 0 
 0 
 0 
 0 
 0 
 0 
 0 
 0 
 0 
 0 
 0 
 0 
 0 
 0 
 0 
 0 
 0 
 0 
 0 
 0 
 0 
 0 
 0 
 0 
 0 
 0 
 0 
 0 
 0 
 0 
 0 
 0 
 0 
 0 
 0 
 0 
 0 
 0 
 0 
 0 
 0 
 0 
 0 
 0 
 0 
 0 
 0 
 0 
 0 
 0 
 0 
 0 
 0 
 0 
 0 
 0 
 0 
 0 
 0 
 0 
 0 
 0 
 0 
 0 
 0 
 0 
 0 
 0 
 0 
 0 
 0 
 0 
 0 
 0 
 0 
 0 
 0 
 0 
 0 
 0 
 0 
 0 
 0 
 0 
 0 
 0 
 0 
 0 
 0 
 0 
 0 
 0 
 0 
 0 
 0 
 0 
 0 
 0 
 0 
 0 
 0 
 0 
 0 
 0 
 0 
 0 
 0 
 0 
 0 
 0 
 0 
 0 
 0 
 0 
 0 
 0 
 0 
 0 
 0 
 0 
 0 
 0 
 0 
 0 
 0 
 0 
 0 
 0 
 0 
 0 
 0 
 0 
 0 
 0 
 0 
 0 
 0 
 0 
 0 
 0 
 0 
 0 
 0 
 0 
 0 
 0 
 0 
 0 
 0 
 0 
 0 
 0 
 0 
 0 
 0 
 0 
 0 
 0 
 0 
 0 
 0 
 0 
 0 
 0 
 0 
 0 
 0 
 0 
 0 
 0 
 0 
 0 
 0 
 0 
 0 
 0 
 0 
 0 
 0 
 0 
 0 
 0 
 0 
 0 
 0 
 0 
 0 
 0 
 0 
 0 
 0 
 0 
 0 
 0 
 0 
 0 
 0 
 0 
 0 
 0 
 0 
 0 
 0 
 0 
 0 
 0 
 0 
 0 
 0 
 0 
 0 
 0 
 0 
 0 
 0 
 0 
 0 
 0 
 0 
 0 
 0 
 0 
 0 
 0 
 0 
 0 
 0 
 0 
 0 
 0 
 0 
 0 
 0 
 0 
 0 
 0 
 0 
 0 
 0 
 0 
 0 
 0 
 0 
 0 
 0 
 0 
 0 
 0 
 0 
 0 
 0 
 0 
 0 
 0 
 0 
 0 
 0 
 0 
 0 
 0 
 0 
 0 
 0 
 0 
 0 
 0 
 0 
 0 
 0 
 0 
 0 
 0 
 6 
 0 
 0 
 0 
 0 
 0 
 0 
 0 
 0 
 0 
 0 
 0 
 0 
 0 
 0 
 0 
 0 
 0 
 0 
 0 
 0 
 0 
 0 
 0 
 0 
 0 
 0 
 0 
 0 
 0 
 0 
 0 
 0 
 0 
 0 
 
 
 108 
 0 
 0 
 0 
 0 
 0 
 0 
 0 
 0 
 0 
 0 
 0 
 0 
 0 
 0 
 0 
 0 
 0 
 0 
 0 
 0 
 0 
 0 
 0 
 0 
 0 
 0 
 0 
 0 
 0 
 0 
 0 
 0 
 0 
 0 
 0 
 0 
 0 
 0 
 0 
 0 
 0 
 0 
 0 
 0 
 0 
 0 
 0 
 0 
 0 
 0 
 0 
 0 
 0 
 0 
 0 
 0 
 0 
 0 
 0 
 0 
 0 
 0 
 0 
 0 
 0 
 0 
 0 
 0 
 0 
 0 
 0 
 0 
 0 
 0 
 0 
 0 
 0 
 0 
 0 
 0 
 0 
 0 
 0 
 0 
 0 
 0 
 0 
 0 
 0 
 0 
 0 
 0 
 0 
 0 
 0 
 0 
 0 
 0 
 0 
 0 
 0 
 0 
 0 
 0 
 0 
 0 
 0 
 0 
 11 
 0 
 0 
 0 
 0 
 0 
 0 
 0 
 0 
 0 
 0 
 0 
 0 
 0 
 0 
 0 
 0 
 0 
 0 
 0 
 0 
 0 
 0 
 0 
 0 
 0 
 0 
 0 
 0 
 0 
 0 
 0 
 0 
 0 
 0 
 0 
 0 
 0 
 0 
 0 
 0 
 0 
 0 
 0 
 0 
 0 
 0 
 0 
 0 
 0 
 0 
 0 
 0 
 0 
 0 
 0 
 0 
 0 
 0 
 0 
 0 
 0 
 0 
 0 
 0 
 0 
 0 
 0 
 0 
 0 
 0 
 0 
 0 
 0 
 0 
 0 
 0 
 0 
 0 
 0 
 0 
 0 
 0 
 0 
 0 
 0 
 0 
 0 
 0 
 0 
 0 
 0 
 0 
 0 
 0 
 0 
 0 
 0 
 0 
 0 
 0 
 0 
 0 
 0 
 0 
 0 
 0 
 0 
 0 
 0 
 0 
 0 
 0 
 0 
 0 
 0 
 0 
 0 
 0 
 0 
 0 
 0 
 0 
 0 
 0 
 0 
 0 
 0 
 0 
 0 
 0 
 0 
 0 
 0 
 0 
 0 
 0 
 0 
 0 
 0 
 0 
 0 
 0 
 0 
 0 
 0 
 0 
 0 
 0 
 0 
 0 
 0 
 0 
 0 
 0 
 0 
 0 
 0 
 0 
 0 
 0 
 0 
 0 
 0 
 0 
 0 
 0 
 0 
 0 
 0 
 0 
 0 
 0 
 0 
 0 
 0 
 0 
 0 
 0 
 0 
 0 
 0 
 0 
 0 
 0 
 0 
 0 
 0 
 0 
 0 
 0 
 0 
 0 
 0 
 0 
 0 
 0 
 0 
 0 
 0 
 0 
 0 
 0 
 0 
 0 
 0 
 0 
 0 
 0 
 0 
 0 
 0 
 0 
 0 
 0 
 0 
 0 
 0 
 0 
 0 
 0 
 0 
 0 
 0 
 0 
 0 
 0 
 0 
 0 
 0 
 0 
 0 
 0 
 0 
 0 
 0 
 0 
 0 
 0 
 0 
 0 
 0 
 0 
 0 
 0 
 0 
 0 
 0 
 0 
 0 
 0 
 0 
 0 
 0 
 0 
 0 
 0 
 0 
 0 
 0 
 0 
 0 
 0 
 0 
 0 
 0 
 0 
 0 
 0 
 0 
 0 
 0 
 0 
 0 
 0 
 0 
 0 
 0 
 0 
 0 
 0 
 0 
 0 
 0 
 0 
 0 
 0 
 0 
 0 
 0 
 0 
 0 
 0 
 0 
 0 
 0 
 0 
 0 
 0 
 0 
 0 
 0 
 0 
 0 
 0 
 0 
 0 
 0 
 0 
 0 
 0 
 0 
 0 
 0 
 0 
 0 
 0 
 0 
 0 
 0 
 0 
 0 
 0 
 0 
 0 
 0 
 0 
 0 
 0 
 0 
 0 
 0 
 0 
 0 
 0 
 0 
 0 
 0 
 0 
 0 
 0 
 0 
 0 
 0 
 0 
 0 
 0 
 0 
 0 
 0 
 0 
 0 
 0 
 0 
 0 
 0 
 0 
 0 
 0 
 0 
 0 
 0 
 0 
 0 
 0 
 0 
 0 
 0 
 0 
 0 
 0 
 0 
 0 
 0 
 0 
 0 
 0 
 0 
 0 
 0 
 0 
 0 
 0 
 0 
 0 
 0 
 0 
 0 
 0 
 0 
 0 
 0 
 0 
 0 
 0 
 0 
 0 
 0 
 0 
 0 
 0 
 0 
 0 
 0 
 0 
 0 
 0 
 0 
 0 
 0 
 0 
 0 
 0 
 0 
 0 
 0 
 0 
 0 
 0 
 0 
 0 
 0 
 0 
 0 
 0 
 0 
 0 
 0 
 0 
 0 
 0 
 0 
 0 
 0 
 0 
 0 
 0 
 0 
 0 
 0 
 0 
 0 
 0 
 0 
 0 
 0 
 0 
 0 
 0 
 0 
 0 
 0 
 0 
 0 
 0 
 0 
 0 
 0 
 0 
 0 
 0 
 0 
 0 
 0 
 0 
 0 
 0 
 0 
 0 
 0 
 0 
 0 
 0 
 0 
 0 
 0 
 0 
 0 
 0 
 0 
 0 
 0 
 0 
 0 
 0 
 0 
 0 
 0 
 0 
 0 
 0 
 0 
 0 
 0 
 0 
 0 
 0 
 0 
 0 
 0 
 0 
 0 
 0 
 0 
 0 
 0 
 0 
 0 
 0 
 0 
 0 
 0 
 0 
 0 
 0 
 0 
 0 
 0 
 0 
 0 
 0 
 0 
 0 
 0 
 0 
 0 
 0 
 0 
 0 
 0 
 0 
 0 
 0 
 0 
 0 
 0 
 0 
 0 
 0 
 0 
 0 
 0 
 0 
 0 
 0 
 0 
 0 
 0 
 0 
 0 
 0 
 0 
 0 
 0 
 0 
 0 
 0 
 0 
 0 
 0 
 0 
 0 
 0 
 0 
 0 
 0 
 0 
 0 
 0 
 0 
 0 
 0 
 0 
 0 
 0 
 0 
 0 
 0 
 0 
 0 
 0 
 0 
 0 
 0 
 0 
 0 
 0 
 0 
 0 
 0 
 0 
 0 
 0 
 0 
 0 
 0 
 0 
 0 
 0 
 0 
 0 
 0 
 0 
 0 
 0 
 0 
 0 
 0 
 0 
 0 
 0 
 0 
 0 
 0 
 0 
 0 
 0 
 0 
 0 
 0 
 0 
 0 
 0 
 0 
 0 
 0 
 0 
 0 
 0 
 0 
 0 
 0 
 0 
 0 
 0 
 0 
 0 
 0 
 0 
 0 
 0 
 0 
 0 
 0 
 0 
 0 
 0 
 0 
 0 
 0 
 0 
 0 
 0 
 0 
 0 
 0 
 0 
 0 
 0 
 0 
 0 
 0 
 0 
 0 
 0 
 0 
 0 
 0 
 0 
 0 
 0 
 0 
 0 
 0 
 0 
 0 
 0 
 0 
 0 
 0 
 0 
 0 
 0 
 0 
 0 
 0 
 0 
 0 
 0 
 0 
 0 
 0 
 0 
 0 
 0 
 0 
 0 
 0 
 0 
 0 
 0 
 0 
 0 
 0 
 0 
 0 
 0 
 0 
 0 
 0 
 0 
 0 
 0 
 0 
 0 
 0 
 0 
 0 
 
 
 109 
 0 
 0 
 0 
 0 
 0 
 0 
 0 
 0 
 0 
 0 
 0 
 0 
 0 
 0 
 0 
 0 
 0 
 0 
 0 
 0 
 0 
 0 
 0 
 0 
 0 
 0 
 0 
 0 
 0 
 0 
 0 
 0 
 0 
 0 
 0 
 0 
 0 
 0 
 0 
 0 
 0 
 0 
 0 
 0 
 0 
 0 
 0 
 0 
 0 
 0 
 0 
 0 
 0 
 0 
 0 
 0 
 0 
 0 
 0 
 0 
 0 
 0 
 0 
 0 
 0 
 0 
 0 
 0 
 0 
 0 
 0 
 0 
 0 
 0 
 0 
 0 
 0 
 0 
 0 
 0 
 0 
 0 
 0 
 0 
 0 
 0 
 0 
 0 
 0 
 0 
 0 
 0 
 0 
 0 
 0 
 0 
 0 
 0 
 0 
 0 
 0 
 0 
 0 
 0 
 0 
 0 
 0 
 0 
 0 
 0 
 0 
 0 
 0 
 0 
 0 
 0 
 0 
 0 
 0 
 0 
 0 
 0 
 0 
 0 
 0 
 0 
 0 
 0 
 0 
 0 
 0 
 0 
 0 
 0 
 0 
 0 
 0 
 0 
 0 
 0 
 0 
 0 
 0 
 0 
 0 
 0 
 0 
 0 
 0 
 0 
 0 
 0 
 0 
 0 
 0 
 0 
 0 
 0 
 0 
 0 
 0 
 0 
 0 
 0 
 0 
 0 
 0 
 0 
 0 
 0 
 0 
 0 
 0 
 0 
 0 
 0 
 0 
 0 
 0 
 0 
 0 
 0 
 0 
 0 
 0 
 0 
 0 
 0 
 0 
 0 
 0 
 0 
 0 
 0 
 0 
 0 
 0 
 0 
 0 
 0 
 0 
 0 
 0 
 0 
 0 
 0 
 0 
 0 
 0 
 0 
 0 
 0 
 0 
 0 
 5 
 0 
 0 
 0 
 0 
 0 
 0 
 0 
 0 
 0 
 0 
 0 
 0 
 0 
 0 
 0 
 0 
 0 
 0 
 0 
 0 
 0 
 0 
 0 
 0 
 0 
 0 
 0 
 0 
 0 
 0 
 0 
 0 
 0 
 0 
 0 
 0 
 0 
 0 
 0 
 0 
 0 
 0 
 0 
 0 
 0 
 0 
 0 
 0 
 0 
 0 
 0 
 0 
 0 
 0 
 0 
 0 
 0 
 0 
 0 
 0 
 0 
 0 
 0 
 0 
 0 
 0 
 0 
 0 
 0 
 0 
 0 
 0 
 0 
 0 
 0 
 0 
 0 
 0 
 0 
 0 
 0 
 0 
 0 
 0 
 0 
 0 
 0 
 0 
 0 
 0 
 0 
 0 
 0 
 0 
 0 
 0 
 0 
 0 
 0 
 0 
 0 
 0 
 0 
 0 
 0 
 0 
 0 
 0 
 0 
 0 
 0 
 0 
 0 
 0 
 0 
 0 
 0 
 0 
 0 
 0 
 0 
 0 
 0 
 0 
 0 
 0 
 0 
 0 
 0 
 0 
 0 
 0 
 0 
 0 
 0 
 0 
 0 
 0 
 0 
 0 
 0 
 0 
 0 
 0 
 0 
 0 
 0 
 0 
 0 
 0 
 0 
 0 
 0 
 0 
 0 
 0 
 0 
 0 
 0 
 0 
 0 
 0 
 0 
 0 
 0 
 0 
 0 
 0 
 0 
 0 
 0 
 0 
 0 
 0 
 0 
 0 
 0 
 0 
 0 
 0 
 0 
 0 
 0 
 0 
 0 
 0 
 0 
 0 
 0 
 0 
 0 
 0 
 0 
 0 
 0 
 0 
 0 
 0 
 0 
 0 
 0 
 0 
 0 
 0 
 0 
 0 
 0 
 0 
 0 
 0 
 0 
 0 
 0 
 0 
 0 
 0 
 0 
 0 
 0 
 0 
 0 
 0 
 0 
 0 
 0 
 0 
 0 
 0 
 0 
 0 
 0 
 0 
 0 
 0 
 0 
 0 
 0 
 0 
 0 
 0 
 0 
 0 
 0 
 0 
 0 
 0 
 0 
 0 
 0 
 0 
 0 
 0 
 0 
 0 
 0 
 0 
 0 
 0 
 0 
 0 
 0 
 0 
 0 
 0 
 0 
 0 
 0 
 0 
 0 
 0 
 0 
 0 
 0 
 0 
 0 
 0 
 0 
 0 
 0 
 0 
 0 
 0 
 0 
 0 
 0 
 0 
 0 
 0 
 0 
 0 
 0 
 0 
 0 
 0 
 0 
 0 
 0 
 0 
 0 
 0 
 0 
 0 
 0 
 0 
 0 
 0 
 0 
 0 
 0 
 0 
 0 
 0 
 0 
 0 
 0 
 0 
 0 
 0 
 0 
 0 
 0 
 0 
 0 
 0 
 0 
 0 
 0 
 0 
 0 
 0 
 0 
 0 
 0 
 0 
 0 
 0 
 0 
 0 
 0 
 0 
 0 
 0 
 0 
 0 
 0 
 0 
 0 
 0 
 0 
 0 
 0 
 0 
 0 
 0 
 0 
 0 
 0 
 0 
 0 
 0 
 0 
 0 
 0 
 0 
 0 
 0 
 0 
 0 
 0 
 0 
 0 
 0 
 0 
 0 
 0 
 0 
 0 
 0 
 0 
 0 
 0 
 0 
 0 
 0 
 0 
 0 
 0 
 0 
 0 
 0 
 0 
 0 
 0 
 0 
 0 
 0 
 0 
 0 
 0 
 0 
 0 
 0 
 0 
 0 
 0 
 0 
 0 
 0 
 0 
 0 
 0 
 0 
 0 
 0 
 0 
 0 
 0 
 0 
 0 
 0 
 0 
 0 
 0 
 0 
 0 
 0 
 0 
 0 
 0 
 0 
 0 
 0 
 0 
 0 
 0 
 0 
 0 
 0 
 0 
 0 
 0 
 0 
 0 
 0 
 0 
 0 
 0 
 0 
 0 
 0 
 0 
 0 
 0 
 0 
 0 
 0 
 0 
 0 
 0 
 0 
 0 
 0 
 0 
 0 
 0 
 0 
 0 
 0 
 0 
 0 
 0 
 0 
 0 
 0 
 0 
 0 
 0 
 0 
 0 
 0 
 0 
 0 
 0 
 0 
 0 
 0 
 0 
 0 
 0 
 0 
 0 
 0 
 0 
 0 
 0 
 0 
 0 
 0 
 0 
 0 
 0 
 0 
 0 
 0 
 0 
 0 
 0 
 0 
 0 
 0 
 0 
 0 
 0 
 0 
 0 
 0 
 0 
 0 
 0 
 0 
 0 
 0 
 0 
 0 
 0 
 0 
 0 
 0 
 0 
 0 
 0 
 0 
 0 
 0 
 0 
 0 
 0 
 0 
 0 
 0 
 0 
 0 
 0 
 0 
 0 
 0 
 0 
 0 
 0 
 0 
 0 
 0 
 0 
 0 
 0 
 0 
 0 
 0 
 0 
 0 
 0 
 0 
 0 
 0 
 0 
 0 
 0 
 0 
 0 
 0 
 0 
 0 
 0 
 0 
 0 
 0 
 0 
 0 
 0 
 0 
 0 
 0 
 0 
 0 
 1 
 0 
 0 
 0 
 0 
 0 
 0 
 0 
 0 
 0 
 0 
 0 
 0 
 0 
 0 
 0 
 0 
 0 
 0 
 0 
 0 
 0 
 0 
 0 
 0 
 0 
 0 
 
 
 110 
 0 
 0 
 0 
 0 
 0 
 0 
 0 
 0 
 0 
 0 
 0 
 0 
 0 
 0 
 0 
 0 
 0 
 0 
 0 
 0 
 0 
 0 
 0 
 0 
 0 
 0 
 0 
 0 
 0 
 0 
 0 
 0 
 0 
 0 
 0 
 0 
 0 
 0 
 0 
 0 
 0 
 0 
 0 
 0 
 0 
 0 
 0 
 0 
 0 
 0 
 0 
 0 
 0 
 0 
 0 
 0 
 0 
 0 
 0 
 0 
 0 
 0 
 0 
 0 
 0 
 0 
 0 
 0 
 0 
 0 
 0 
 0 
 0 
 0 
 0 
 0 
 0 
 0 
 0 
 0 
 0 
 0 
 0 
 0 
 0 
 0 
 0 
 0 
 0 
 0 
 0 
 0 
 0 
 0 
 0 
 0 
 0 
 0 
 0 
 0 
 0 
 0 
 0 
 0 
 0 
 0 
 0 
 0 
 0 
 0 
 2 
 0 
 0 
 5 
 0 
 0 
 0 
 0 
 0 
 0 
 0 
 0 
 0 
 0 
 0 
 0 
 0 
 0 
 0 
 0 
 0 
 0 
 0 
 0 
 0 
 0 
 0 
 0 
 0 
 0 
 0 
 0 
 0 
 0 
 0 
 0 
 0 
 0 
 0 
 0 
 0 
 0 
 0 
 0 
 0 
 0 
 0 
 0 
 0 
 0 
 0 
 0 
 0 
 0 
 0 
 0 
 0 
 0 
 0 
 0 
 0 
 0 
 0 
 0 
 0 
 0 
 0 
 0 
 0 
 0 
 0 
 0 
 0 
 0 
 0 
 0 
 0 
 0 
 0 
 0 
 0 
 0 
 0 
 0 
 0 
 0 
 0 
 0 
 0 
 0 
 0 
 0 
 0 
 0 
 0 
 0 
 0 
 0 
 0 
 0 
 0 
 0 
 0 
 0 
 0 
 0 
 0 
 0 
 0 
 0 
 0 
 0 
 0 
 0 
 0 
 0 
 0 
 0 
 0 
 0 
 0 
 0 
 0 
 0 
 0 
 0 
 0 
 0 
 0 
 0 
 0 
 0 
 0 
 0 
 0 
 0 
 0 
 0 
 0 
 0 
 0 
 0 
 0 
 0 
 0 
 0 
 0 
 0 
 0 
 0 
 0 
 0 
 0 
 0 
 0 
 0 
 0 
 0 
 0 
 0 
 0 
 0 
 0 
 0 
 0 
 0 
 0 
 0 
 0 
 0 
 0 
 0 
 0 
 0 
 0 
 0 
 0 
 0 
 0 
 0 
 0 
 0 
 0 
 0 
 0 
 0 
 0 
 0 
 0 
 0 
 0 
 0 
 0 
 0 
 0 
 0 
 0 
 0 
 0 
 0 
 0 
 0 
 0 
 0 
 0 
 0 
 0 
 0 
 0 
 0 
 0 
 0 
 0 
 0 
 0 
 0 
 0 
 0 
 0 
 0 
 0 
 0 
 0 
 0 
 0 
 0 
 0 
 0 
 0 
 0 
 0 
 0 
 0 
 0 
 0 
 0 
 0 
 0 
 0 
 0 
 0 
 0 
 0 
 0 
 0 
 0 
 0 
 0 
 0 
 0 
 0 
 0 
 0 
 0 
 0 
 0 
 0 
 0 
 0 
 0 
 0 
 0 
 0 
 0 
 0 
 0 
 0 
 0 
 0 
 0 
 0 
 0 
 0 
 0 
 0 
 0 
 0 
 0 
 0 
 0 
 0 
 0 
 0 
 0 
 0 
 0 
 0 
 0 
 0 
 0 
 0 
 0 
 0 
 0 
 0 
 0 
 0 
 0 
 0 
 0 
 0 
 0 
 0 
 0 
 0 
 0 
 0 
 0 
 0 
 0 
 0 
 0 
 0 
 0 
 0 
 0 
 0 
 0 
 0 
 0 
 0 
 0 
 0 
 0 
 0 
 0 
 0 
 0 
 0 
 0 
 0 
 0 
 0 
 0 
 0 
 0 
 0 
 0 
 0 
 0 
 0 
 0 
 0 
 0 
 0 
 0 
 0 
 0 
 0 
 0 
 0 
 0 
 0 
 0 
 0 
 0 
 0 
 0 
 0 
 0 
 0 
 0 
 0 
 0 
 0 
 0 
 0 
 0 
 0 
 0 
 0 
 0 
 0 
 0 
 0 
 0 
 0 
 0 
 0 
 0 
 0 
 0 
 0 
 0 
 0 
 0 
 0 
 0 
 0 
 0 
 0 
 0 
 0 
 0 
 0 
 0 
 0 
 0 
 0 
 0 
 0 
 0 
 0 
 0 
 0 
 0 
 0 
 0 
 0 
 0 
 0 
 0 
 0 
 0 
 0 
 0 
 0 
 0 
 0 
 0 
 0 
 0 
 0 
 0 
 0 
 0 
 0 
 0 
 0 
 0 
 0 
 0 
 0 
 0 
 0 
 0 
 0 
 0 
 0 
 0 
 0 
 0 
 0 
 0 
 0 
 0 
 0 
 0 
 0 
 0 
 0 
 0 
 0 
 0 
 0 
 0 
 0 
 0 
 0 
 0 
 0 
 0 
 0 
 0 
 0 
 0 
 0 
 0 
 0 
 0 
 0 
 0 
 0 
 0 
 0 
 0 
 0 
 0 
 0 
 0 
 0 
 0 
 0 
 0 
 0 
 0 
 0 
 0 
 0 
 0 
 0 
 0 
 0 
 0 
 0 
 0 
 0 
 0 
 0 
 0 
 0 
 0 
 0 
 0 
 0 
 0 
 0 
 0 
 0 
 0 
 0 
 0 
 0 
 0 
 0 
 0 
 0 
 0 
 0 
 0 
 0 
 0 
 0 
 0 
 0 
 0 
 0 
 0 
 0 
 0 
 0 
 0 
 0 
 0 
 0 
 0 
 0 
 0 
 0 
 0 
 0 
 0 
 0 
 0 
 0 
 0 
 0 
 0 
 0 
 0 
 0 
 0 
 0 
 0 
 0 
 0 
 0 
 0 
 0 
 0 
 0 
 0 
 0 
 0 
 0 
 0 
 0 
 0 
 0 
 0 
 0 
 0 
 0 
 0 
 0 
 0 
 0 
 0 
 0 
 0 
 0 
 0 
 0 
 0 
 0 
 0 
 0 
 0 
 0 
 0 
 0 
 0 
 0 
 0 
 0 
 0 
 0 
 0 
 0 
 0 
 0 
 0 
 0 
 0 
 0 
 0 
 0 
 0 
 0 
 0 
 0 
 0 
 0 
 0 
 0 
 0 
 0 
 0 
 0 
 0 
 0 
 0 
 0 
 0 
 0 
 0 
 0 
 0 
 0 
 0 
 0 
 0 
 0 
 0 
 0 
 0 
 0 
 0 
 0 
 0 
 0 
 0 
 0 
 0 
 0 
 0 
 0 
 0 
 0 
 0 
 0 
 0 
 0 
 0 
 0 
 0 
 0 
 0 
 0 
 0 
 0 
 0 
 0 
 0 
 0 
 0 
 0 
 0 
 0 
 0 
 0 
 0 
 0 
 0 
 0 
 0 
 0 
 0 
 0 
 0 
 0 
 0 
 0 
 0 
 0 
 0 
 0 
 0 
 0 
 0 
 0 
 0 
 0 
 0 
 0 
 0 
 0 
 0 
 0 
 0 
 0 
 0 
 0 
 0 
 0 
 0 
 0 
 0 
 0 
 0 
 0 
 0 
 0 
 0 
 0 
 0 
 
 
 111 
 0 
 0 
 0 
 0 
 0 
 0 
 0 
 0 
 0 
 0 
 0 
 0 
 0 
 0 
 0 
 0 
 0 
 0 
 0 
 0 
 0 
 0 
 0 
 0 
 0 
 0 
 0 
 0 
 0 
 0 
 0 
 0 
 0 
 0 
 0 
 0 
 0 
 0 
 0 
 0 
 0 
 0 
 0 
 0 
 0 
 0 
 0 
 0 
 0 
 0 
 0 
 0 
 0 
 0 
 0 
 0 
 0 
 0 
 0 
 0 
 0 
 0 
 0 
 0 
 0 
 0 
 0 
 0 
 0 
 0 
 0 
 0 
 0 
 0 
 0 
 0 
 0 
 0 
 0 
 0 
 0 
 0 
 0 
 0 
 0 
 0 
 0 
 0 
 0 
 0 
 0 
 0 
 0 
 0 
 0 
 0 
 0 
 0 
 0 
 0 
 0 
 0 
 0 
 0 
 0 
 0 
 0 
 0 
 0 
 0 
 0 
 3 
 0 
 0 
 0 
 0 
 0 
 0 
 0 
 0 
 0 
 0 
 0 
 0 
 0 
 0 
 0 
 0 
 0 
 0 
 0 
 0 
 0 
 0 
 0 
 0 
 0 
 0 
 0 
 0 
 0 
 0 
 0 
 0 
 0 
 0 
 0 
 0 
 0 
 0 
 0 
 0 
 0 
 0 
 0 
 0 
 0 
 0 
 0 
 0 
 0 
 0 
 0 
 0 
 0 
 0 
 0 
 0 
 0 
 0 
 0 
 0 
 0 
 0 
 0 
 0 
 0 
 0 
 0 
 0 
 0 
 0 
 0 
 0 
 0 
 0 
 0 
 0 
 0 
 0 
 0 
 0 
 0 
 0 
 0 
 0 
 0 
 0 
 0 
 0 
 0 
 0 
 0 
 0 
 0 
 0 
 0 
 0 
 0 
 0 
 0 
 0 
 0 
 0 
 0 
 0 
 0 
 0 
 0 
 0 
 0 
 0 
 0 
 0 
 0 
 0 
 0 
 0 
 0 
 0 
 0 
 0 
 0 
 0 
 0 
 0 
 0 
 0 
 0 
 0 
 0 
 0 
 0 
 0 
 0 
 0 
 0 
 0 
 0 
 0 
 0 
 0 
 0 
 0 
 0 
 0 
 0 
 0 
 0 
 0 
 0 
 0 
 0 
 0 
 0 
 0 
 0 
 0 
 0 
 0 
 0 
 0 
 0 
 0 
 0 
 0 
 0 
 0 
 0 
 0 
 0 
 0 
 0 
 0 
 0 
 0 
 0 
 0 
 0 
 0 
 0 
 0 
 0 
 0 
 0 
 0 
 0 
 0 
 0 
 0 
 0 
 0 
 0 
 0 
 0 
 0 
 0 
 0 
 0 
 0 
 0 
 0 
 0 
 0 
 0 
 0 
 0 
 0 
 0 
 0 
 0 
 0 
 0 
 0 
 0 
 0 
 0 
 0 
 0 
 0 
 0 
 0 
 0 
 0 
 0 
 0 
 0 
 0 
 0 
 0 
 0 
 0 
 0 
 0 
 0 
 0 
 0 
 0 
 0 
 0 
 0 
 0 
 0 
 0 
 0 
 0 
 0 
 0 
 0 
 0 
 0 
 0 
 0 
 0 
 0 
 0 
 0 
 0 
 0 
 0 
 0 
 0 
 0 
 0 
 0 
 0 
 0 
 0 
 0 
 0 
 0 
 0 
 0 
 0 
 0 
 0 
 0 
 0 
 0 
 0 
 0 
 0 
 0 
 0 
 0 
 0 
 0 
 0 
 0 
 0 
 0 
 0 
 0 
 0 
 0 
 0 
 0 
 0 
 0 
 0 
 0 
 0 
 0 
 0 
 0 
 0 
 0 
 0 
 0 
 0 
 0 
 0 
 0 
 0 
 0 
 0 
 0 
 0 
 0 
 0 
 0 
 0 
 0 
 0 
 0 
 0 
 0 
 0 
 0 
 0 
 0 
 0 
 0 
 0 
 0 
 0 
 0 
 0 
 0 
 0 
 0 
 0 
 0 
 0 
 0 
 0 
 0 
 0 
 0 
 0 
 0 
 0 
 0 
 0 
 0 
 0 
 0 
 0 
 0 
 0 
 0 
 0 
 0 
 0 
 0 
 0 
 0 
 0 
 0 
 0 
 0 
 0 
 0 
 0 
 0 
 0 
 0 
 0 
 0 
 0 
 0 
 0 
 0 
 0 
 0 
 0 
 0 
 0 
 0 
 0 
 0 
 0 
 0 
 0 
 0 
 0 
 0 
 0 
 0 
 0 
 0 
 0 
 0 
 0 
 0 
 0 
 0 
 0 
 0 
 0 
 0 
 0 
 0 
 0 
 0 
 0 
 0 
 0 
 0 
 0 
 0 
 0 
 0 
 0 
 0 
 0 
 0 
 0 
 0 
 0 
 0 
 0 
 0 
 0 
 0 
 0 
 0 
 0 
 0 
 0 
 0 
 0 
 0 
 0 
 0 
 0 
 0 
 0 
 0 
 0 
 0 
 0 
 0 
 0 
 0 
 0 
 0 
 0 
 0 
 0 
 0 
 0 
 0 
 0 
 0 
 0 
 0 
 0 
 0 
 0 
 0 
 0 
 0 
 0 
 0 
 0 
 0 
 0 
 0 
 0 
 0 
 0 
 0 
 0 
 0 
 0 
 0 
 0 
 0 
 0 
 0 
 0 
 0 
 0 
 0 
 0 
 0 
 0 
 0 
 0 
 0 
 0 
 0 
 0 
 0 
 0 
 0 
 0 
 0 
 0 
 0 
 0 
 0 
 0 
 0 
 0 
 0 
 0 
 0 
 0 
 0 
 0 
 0 
 0 
 0 
 0 
 0 
 0 
 0 
 0 
 0 
 0 
 0 
 0 
 0 
 0 
 0 
 0 
 0 
 0 
 0 
 0 
 0 
 0 
 0 
 0 
 0 
 0 
 0 
 0 
 0 
 0 
 0 
 0 
 0 
 0 
 0 
 0 
 0 
 0 
 0 
 0 
 0 
 0 
 0 
 0 
 0 
 0 
 0 
 0 
 0 
 0 
 0 
 0 
 0 
 0 
 0 
 0 
 0 
 0 
 0 
 0 
 0 
 0 
 0 
 0 
 0 
 0 
 0 
 0 
 0 
 0 
 0 
 0 
 0 
 0 
 0 
 0 
 0 
 0 
 0 
 0 
 0 
 0 
 0 
 0 
 0 
 0 
 0 
 0 
 0 
 0 
 0 
 0 
 0 
 0 
 0 
 0 
 0 
 0 
 0 
 0 
 0 
 0 
 0 
 0 
 0 
 0 
 0 
 0 
 0 
 0 
 0 
 0 
 0 
 0 
 0 
 0 
 0 
 0 
 0 
 0 
 0 
 0 
 0 
 0 
 0 
 0 
 0 
 0 
 0 
 0 
 0 
 0 
 0 
 0 
 0 
 0 
 0 
 0 
 0 
 0 
 0 
 0 
 0 
 0 
 0 
 0 
 0 
 0 
 0 
 0 
 0 
 0 
 0 
 0 
 0 
 0 
 0 
 0 
 0 
 0 
 0 
 0 
 0 
 0 
 0 
 0 
 0 
 0 
 0 
 0 
 0 
 0 
 0 
 0 
 0 
 0 
 0 
 0 
 0 
 0 
 0 
 0 
 0 
 0 
 0 
 0 
 0 
 0 
 0 
 0 
 0 
 0 
 0 
 0 
 
 
 112 
 0 
 0 
 0 
 0 
 0 
 0 
 0 
 0 
 0 
 0 
 0 
 0 
 0 
 0 
 0 
 0 
 0 
 0 
 0 
 0 
 0 
 0 
 0 
 0 
 0 
 0 
 0 
 0 
 0 
 0 
 0 
 0 
 0 
 0 
 0 
 0 
 0 
 0 
 0 
 0 
 0 
 0 
 0 
 0 
 0 
 0 
 0 
 0 
 0 
 0 
 0 
 0 
 0 
 0 
 0 
 0 
 0 
 0 
 0 
 0 
 0 
 0 
 0 
 0 
 0 
 0 
 0 
 0 
 0 
 0 
 0 
 0 
 0 
 0 
 0 
 0 
 0 
 0 
 0 
 0 
 0 
 0 
 0 
 0 
 0 
 0 
 0 
 0 
 0 
 0 
 0 
 0 
 0 
 0 
 0 
 0 
 0 
 0 
 0 
 0 
 0 
 0 
 0 
 0 
 0 
 0 
 0 
 0 
 0 
 0 
 0 
 0 
 33 
 0 
 0 
 0 
 0 
 0 
 0 
 0 
 0 
 0 
 0 
 0 
 0 
 0 
 0 
 0 
 0 
 0 
 0 
 0 
 0 
 0 
 0 
 0 
 0 
 0 
 0 
 0 
 0 
 0 
 0 
 0 
 0 
 0 
 0 
 0 
 0 
 0 
 0 
 0 
 0 
 0 
 0 
 0 
 0 
 0 
 0 
 0 
 0 
 0 
 0 
 0 
 0 
 0 
 0 
 0 
 0 
 0 
 0 
 0 
 0 
 0 
 0 
 0 
 0 
 0 
 0 
 0 
 0 
 0 
 0 
 0 
 0 
 0 
 0 
 0 
 0 
 0 
 0 
 0 
 0 
 0 
 0 
 0 
 0 
 0 
 0 
 0 
 0 
 0 
 0 
 0 
 0 
 0 
 0 
 0 
 0 
 0 
 0 
 0 
 0 
 0 
 0 
 0 
 0 
 0 
 0 
 0 
 0 
 0 
 0 
 0 
 0 
 0 
 0 
 0 
 0 
 0 
 0 
 0 
 0 
 0 
 0 
 0 
 0 
 0 
 0 
 0 
 0 
 0 
 0 
 0 
 0 
 0 
 0 
 0 
 0 
 0 
 0 
 0 
 0 
 0 
 0 
 0 
 0 
 0 
 0 
 0 
 0 
 0 
 0 
 0 
 0 
 0 
 0 
 0 
 0 
 0 
 0 
 0 
 0 
 0 
 0 
 0 
 0 
 0 
 0 
 0 
 0 
 0 
 0 
 0 
 0 
 0 
 0 
 0 
 0 
 0 
 0 
 0 
 0 
 0 
 0 
 0 
 0 
 0 
 0 
 0 
 0 
 0 
 0 
 0 
 0 
 0 
 0 
 0 
 0 
 0 
 0 
 0 
 0 
 0 
 0 
 0 
 0 
 0 
 0 
 0 
 0 
 0 
 0 
 0 
 0 
 0 
 0 
 0 
 0 
 0 
 0 
 0 
 0 
 0 
 0 
 0 
 0 
 0 
 0 
 0 
 0 
 0 
 0 
 0 
 0 
 0 
 0 
 0 
 0 
 0 
 0 
 0 
 0 
 0 
 0 
 0 
 0 
 0 
 0 
 0 
 0 
 0 
 0 
 0 
 0 
 0 
 0 
 0 
 0 
 0 
 0 
 0 
 0 
 0 
 0 
 0 
 0 
 0 
 0 
 0 
 0 
 0 
 0 
 0 
 0 
 0 
 0 
 0 
 0 
 0 
 0 
 0 
 0 
 0 
 0 
 0 
 0 
 0 
 0 
 0 
 0 
 0 
 0 
 0 
 0 
 0 
 0 
 0 
 0 
 0 
 0 
 0 
 0 
 0 
 0 
 0 
 0 
 0 
 0 
 0 
 0 
 0 
 0 
 0 
 0 
 0 
 0 
 0 
 0 
 0 
 0 
 0 
 0 
 0 
 0 
 0 
 0 
 0 
 0 
 0 
 0 
 0 
 0 
 0 
 0 
 0 
 0 
 0 
 0 
 0 
 0 
 0 
 0 
 0 
 0 
 0 
 0 
 0 
 0 
 0 
 0 
 0 
 0 
 0 
 0 
 0 
 0 
 0 
 0 
 0 
 0 
 0 
 0 
 0 
 0 
 0 
 0 
 0 
 0 
 0 
 0 
 0 
 0 
 0 
 0 
 0 
 0 
 0 
 0 
 0 
 0 
 0 
 0 
 0 
 0 
 0 
 0 
 0 
 0 
 0 
 0 
 0 
 0 
 0 
 0 
 0 
 0 
 0 
 0 
 0 
 0 
 0 
 0 
 0 
 0 
 0 
 0 
 0 
 0 
 0 
 0 
 0 
 0 
 0 
 0 
 0 
 0 
 0 
 0 
 0 
 0 
 0 
 0 
 0 
 0 
 0 
 0 
 0 
 0 
 0 
 0 
 0 
 0 
 0 
 0 
 0 
 0 
 0 
 0 
 0 
 0 
 0 
 0 
 0 
 0 
 0 
 0 
 0 
 0 
 0 
 0 
 0 
 0 
 0 
 0 
 0 
 0 
 0 
 0 
 0 
 0 
 0 
 0 
 0 
 0 
 0 
 0 
 0 
 0 
 0 
 0 
 0 
 0 
 0 
 0 
 0 
 0 
 0 
 0 
 0 
 0 
 0 
 0 
 0 
 0 
 0 
 0 
 0 
 0 
 0 
 0 
 0 
 0 
 0 
 0 
 0 
 0 
 0 
 0 
 0 
 0 
 0 
 0 
 0 
 0 
 0 
 0 
 0 
 0 
 0 
 0 
 0 
 0 
 0 
 0 
 0 
 0 
 0 
 0 
 0 
 0 
 0 
 0 
 0 
 0 
 0 
 0 
 0 
 0 
 0 
 0 
 0 
 0 
 0 
 0 
 0 
 0 
 0 
 0 
 0 
 0 
 0 
 0 
 0 
 0 
 0 
 0 
 0 
 0 
 0 
 0 
 0 
 0 
 0 
 0 
 0 
 0 
 0 
 0 
 0 
 0 
 0 
 0 
 0 
 0 
 0 
 0 
 0 
 0 
 0 
 0 
 0 
 0 
 0 
 0 
 0 
 0 
 0 
 0 
 0 
 0 
 0 
 0 
 0 
 0 
 0 
 0 
 0 
 0 
 0 
 0 
 0 
 0 
 0 
 0 
 0 
 0 
 0 
 0 
 0 
 0 
 0 
 0 
 0 
 0 
 0 
 0 
 0 
 0 
 0 
 0 
 0 
 0 
 0 
 0 
 0 
 0 
 0 
 0 
 0 
 0 
 0 
 0 
 0 
 0 
 0 
 0 
 0 
 0 
 0 
 0 
 0 
 0 
 0 
 0 
 0 
 0 
 0 
 0 
 0 
 0 
 0 
 0 
 0 
 0 
 0 
 0 
 0 
 0 
 0 
 0 
 0 
 0 
 0 
 0 
 0 
 0 
 0 
 0 
 0 
 0 
 0 
 0 
 0 
 0 
 0 
 0 
 0 
 0 
 0 
 0 
 0 
 0 
 0 
 0 
 0 
 0 
 0 
 0 
 0 
 0 
 0 
 0 
 0 
 0 
 0 
 0 
 0 
 0 
 0 
 0 
 0 
 0 
 0 
 0 
 0 
 0 
 0 
 0 
 0 
 0 
 0 
 0 
 0 
 0 
 0 
 0 
 0 
 0 
 0 
 0 
 0 
 0 
 0 
 0 
 0 
 
 
 113 
 0 
 0 
 0 
 0 
 0 
 0 
 0 
 0 
 0 
 0 
 0 
 0 
 0 
 0 
 0 
 0 
 0 
 0 
 0 
 0 
 0 
 0 
 0 
 0 
 0 
 0 
 0 
 0 
 0 
 0 
 0 
 0 
 0 
 0 
 0 
 0 
 0 
 0 
 0 
 0 
 0 
 0 
 0 
 0 
 0 
 0 
 0 
 0 
 0 
 0 
 0 
 0 
 0 
 0 
 0 
 0 
 0 
 0 
 0 
 0 
 0 
 0 
 0 
 0 
 0 
 0 
 0 
 0 
 0 
 0 
 0 
 0 
 0 
 0 
 0 
 0 
 0 
 0 
 0 
 0 
 0 
 0 
 0 
 0 
 0 
 0 
 0 
 0 
 0 
 0 
 0 
 0 
 0 
 0 
 0 
 0 
 0 
 0 
 0 
 0 
 0 
 0 
 0 
 0 
 0 
 0 
 0 
 0 
 0 
 0 
 0 
 0 
 0 
 15 
 0 
 0 
 0 
 0 
 0 
 0 
 0 
 0 
 0 
 0 
 0 
 0 
 0 
 0 
 0 
 0 
 0 
 0 
 0 
 0 
 0 
 0 
 0 
 0 
 0 
 0 
 0 
 0 
 0 
 0 
 0 
 0 
 0 
 0 
 0 
 0 
 0 
 0 
 0 
 0 
 0 
 0 
 0 
 0 
 0 
 0 
 0 
 0 
 0 
 0 
 0 
 0 
 0 
 0 
 0 
 0 
 0 
 0 
 0 
 0 
 0 
 0 
 0 
 0 
 0 
 0 
 0 
 0 
 0 
 0 
 0 
 0 
 0 
 0 
 0 
 0 
 0 
 0 
 0 
 0 
 0 
 0 
 0 
 0 
 0 
 0 
 0 
 0 
 0 
 0 
 0 
 0 
 0 
 0 
 0 
 0 
 0 
 0 
 0 
 0 
 0 
 0 
 0 
 0 
 0 
 0 
 0 
 0 
 0 
 0 
 0 
 0 
 0 
 0 
 0 
 0 
 0 
 0 
 0 
 0 
 0 
 0 
 0 
 0 
 0 
 0 
 0 
 0 
 0 
 0 
 0 
 0 
 0 
 0 
 0 
 0 
 0 
 0 
 0 
 0 
 0 
 0 
 0 
 0 
 0 
 0 
 0 
 0 
 0 
 0 
 0 
 0 
 0 
 0 
 0 
 0 
 0 
 0 
 0 
 0 
 0 
 0 
 0 
 0 
 0 
 0 
 0 
 0 
 0 
 0 
 0 
 0 
 0 
 0 
 0 
 0 
 0 
 0 
 0 
 0 
 0 
 0 
 0 
 0 
 0 
 0 
 0 
 0 
 0 
 0 
 0 
 0 
 0 
 0 
 0 
 0 
 0 
 0 
 0 
 0 
 0 
 0 
 0 
 0 
 0 
 0 
 0 
 0 
 0 
 0 
 0 
 0 
 0 
 0 
 0 
 0 
 0 
 0 
 0 
 0 
 0 
 0 
 0 
 0 
 0 
 0 
 0 
 0 
 0 
 0 
 0 
 0 
 0 
 0 
 0 
 0 
 0 
 0 
 0 
 0 
 0 
 0 
 0 
 0 
 0 
 0 
 0 
 0 
 0 
 0 
 0 
 0 
 0 
 0 
 0 
 0 
 0 
 0 
 0 
 0 
 0 
 0 
 0 
 0 
 0 
 0 
 0 
 0 
 0 
 0 
 0 
 0 
 0 
 0 
 0 
 0 
 0 
 0 
 0 
 0 
 0 
 0 
 0 
 0 
 0 
 0 
 0 
 0 
 0 
 0 
 0 
 0 
 0 
 0 
 0 
 0 
 0 
 0 
 0 
 0 
 0 
 0 
 0 
 0 
 0 
 0 
 0 
 0 
 0 
 0 
 0 
 0 
 0 
 0 
 0 
 0 
 0 
 0 
 0 
 0 
 0 
 0 
 0 
 0 
 0 
 0 
 0 
 0 
 0 
 0 
 0 
 0 
 0 
 0 
 0 
 0 
 0 
 0 
 0 
 0 
 0 
 0 
 0 
 0 
 0 
 0 
 0 
 0 
 0 
 0 
 0 
 0 
 0 
 0 
 0 
 0 
 0 
 0 
 0 
 0 
 0 
 0 
 0 
 0 
 0 
 0 
 0 
 0 
 0 
 0 
 0 
 0 
 0 
 0 
 0 
 0 
 0 
 0 
 0 
 0 
 0 
 0 
 0 
 0 
 0 
 0 
 0 
 0 
 0 
 0 
 0 
 0 
 0 
 0 
 0 
 0 
 0 
 0 
 0 
 0 
 0 
 0 
 0 
 0 
 0 
 0 
 0 
 0 
 0 
 0 
 0 
 0 
 0 
 0 
 0 
 0 
 0 
 0 
 0 
 0 
 0 
 0 
 0 
 0 
 0 
 0 
 0 
 0 
 0 
 0 
 0 
 0 
 0 
 0 
 0 
 0 
 0 
 0 
 0 
 0 
 0 
 0 
 0 
 0 
 0 
 0 
 0 
 0 
 0 
 0 
 0 
 0 
 0 
 0 
 0 
 0 
 0 
 0 
 0 
 0 
 0 
 0 
 0 
 0 
 0 
 0 
 0 
 0 
 0 
 0 
 0 
 0 
 0 
 0 
 0 
 0 
 0 
 0 
 0 
 0 
 0 
 0 
 0 
 0 
 0 
 0 
 0 
 0 
 0 
 0 
 0 
 0 
 0 
 0 
 0 
 0 
 0 
 0 
 0 
 0 
 0 
 0 
 0 
 0 
 0 
 0 
 0 
 0 
 0 
 0 
 0 
 0 
 0 
 0 
 0 
 0 
 0 
 0 
 0 
 0 
 0 
 0 
 0 
 0 
 0 
 0 
 0 
 0 
 0 
 0 
 0 
 0 
 0 
 0 
 0 
 0 
 0 
 0 
 0 
 0 
 0 
 0 
 0 
 0 
 0 
 0 
 0 
 0 
 0 
 0 
 0 
 0 
 0 
 0 
 0 
 0 
 0 
 0 
 0 
 0 
 0 
 0 
 0 
 0 
 0 
 0 
 0 
 0 
 0 
 0 
 0 
 0 
 0 
 0 
 0 
 0 
 0 
 0 
 0 
 0 
 0 
 0 
 0 
 0 
 0 
 0 
 0 
 0 
 0 
 0 
 0 
 0 
 0 
 0 
 0 
 0 
 0 
 0 
 0 
 0 
 0 
 0 
 0 
 0 
 0 
 0 
 0 
 0 
 0 
 0 
 0 
 0 
 0 
 0 
 0 
 0 
 0 
 0 
 0 
 0 
 0 
 0 
 0 
 0 
 0 
 0 
 0 
 0 
 0 
 0 
 0 
 0 
 0 
 0 
 0 
 0 
 0 
 0 
 0 
 0 
 0 
 0 
 0 
 0 
 0 
 0 
 0 
 0 
 0 
 0 
 0 
 0 
 0 
 0 
 0 
 0 
 0 
 0 
 0 
 0 
 0 
 0 
 0 
 0 
 0 
 0 
 0 
 0 
 0 
 0 
 0 
 0 
 0 
 0 
 0 
 0 
 0 
 0 
 0 
 0 
 0 
 0 
 0 
 0 
 0 
 0 
 0 
 0 
 0 
 0 
 0 
 0 
 0 
 0 
 0 
 0 
 0 
 0 
 0 
 0 
 0 
 0 
 0 
 0 
 0 
 0 
 0 
 0 
 0 
 0 
 0 
 0 
 
 
 114 
 0 
 0 
 0 
 0 
 0 
 0 
 2 
 0 
 0 
 0 
 0 
 0 
 0 
 0 
 0 
 0 
 0 
 0 
 0 
 0 
 0 
 0 
 0 
 0 
 0 
 0 
 0 
 0 
 0 
 0 
 0 
 0 
 0 
 0 
 0 
 0 
 0 
 0 
 0 
 0 
 0 
 0 
 0 
 0 
 0 
 0 
 0 
 0 
 0 
 0 
 0 
 0 
 0 
 0 
 0 
 0 
 0 
 0 
 0 
 0 
 0 
 0 
 0 
 0 
 0 
 0 
 0 
 0 
 0 
 0 
 0 
 0 
 0 
 0 
 0 
 0 
 0 
 0 
 0 
 0 
 0 
 0 
 0 
 0 
 0 
 0 
 0 
 0 
 0 
 0 
 0 
 0 
 0 
 0 
 0 
 0 
 0 
 0 
 0 
 0 
 0 
 0 
 0 
 0 
 0 
 0 
 0 
 0 
 0 
 0 
 0 
 0 
 0 
 0 
 0 
 0 
 0 
 0 
 0 
 0 
 0 
 0 
 0 
 0 
 0 
 0 
 0 
 0 
 0 
 0 
 0 
 0 
 0 
 0 
 0 
 0 
 0 
 0 
 0 
 0 
 0 
 0 
 0 
 0 
 0 
 0 
 0 
 0 
 0 
 0 
 0 
 0 
 0 
 0 
 0 
 0 
 0 
 0 
 0 
 0 
 0 
 0 
 0 
 0 
 0 
 0 
 0 
 0 
 0 
 0 
 0 
 0 
 0 
 0 
 0 
 0 
 0 
 0 
 0 
 0 
 0 
 0 
 0 
 0 
 0 
 0 
 0 
 0 
 0 
 0 
 0 
 0 
 0 
 0 
 0 
 0 
 0 
 0 
 0 
 0 
 0 
 0 
 0 
 0 
 0 
 0 
 0 
 0 
 0 
 0 
 0 
 0 
 0 
 0 
 0 
 0 
 0 
 0 
 0 
 0 
 0 
 0 
 0 
 0 
 0 
 0 
 0 
 0 
 0 
 0 
 0 
 0 
 0 
 0 
 0 
 0 
 0 
 0 
 0 
 0 
 0 
 0 
 0 
 0 
 0 
 0 
 0 
 0 
 0 
 0 
 0 
 0 
 0 
 0 
 0 
 0 
 0 
 0 
 0 
 0 
 0 
 0 
 0 
 0 
 0 
 0 
 0 
 0 
 0 
 0 
 0 
 0 
 0 
 0 
 0 
 0 
 0 
 0 
 0 
 0 
 0 
 0 
 0 
 0 
 0 
 0 
 0 
 0 
 0 
 0 
 0 
 0 
 0 
 0 
 0 
 0 
 0 
 0 
 0 
 0 
 0 
 0 
 0 
 0 
 0 
 0 
 0 
 0 
 0 
 0 
 0 
 0 
 0 
 0 
 0 
 0 
 0 
 0 
 0 
 0 
 0 
 0 
 0 
 0 
 0 
 0 
 0 
 0 
 0 
 0 
 0 
 0 
 0 
 0 
 0 
 0 
 0 
 0 
 0 
 0 
 0 
 0 
 0 
 0 
 0 
 0 
 0 
 0 
 0 
 0 
 0 
 0 
 0 
 0 
 0 
 0 
 0 
 0 
 0 
 0 
 0 
 0 
 0 
 0 
 0 
 0 
 0 
 0 
 0 
 0 
 0 
 0 
 0 
 0 
 0 
 0 
 0 
 0 
 0 
 0 
 0 
 0 
 0 
 0 
 0 
 0 
 0 
 0 
 0 
 0 
 0 
 0 
 0 
 0 
 0 
 0 
 0 
 0 
 0 
 0 
 0 
 0 
 0 
 0 
 0 
 0 
 0 
 0 
 0 
 0 
 0 
 0 
 0 
 0 
 0 
 0 
 0 
 0 
 0 
 0 
 0 
 0 
 0 
 0 
 0 
 0 
 0 
 0 
 0 
 0 
 0 
 0 
 0 
 0 
 0 
 0 
 0 
 0 
 0 
 0 
 0 
 0 
 0 
 0 
 0 
 0 
 0 
 0 
 0 
 0 
 0 
 0 
 0 
 0 
 0 
 0 
 0 
 0 
 0 
 0 
 0 
 0 
 0 
 0 
 0 
 0 
 0 
 0 
 0 
 0 
 0 
 0 
 0 
 0 
 0 
 0 
 0 
 0 
 0 
 0 
 0 
 0 
 0 
 0 
 0 
 0 
 0 
 0 
 0 
 0 
 0 
 0 
 0 
 0 
 0 
 0 
 0 
 0 
 0 
 0 
 0 
 0 
 0 
 0 
 0 
 0 
 0 
 0 
 0 
 0 
 0 
 0 
 0 
 0 
 0 
 0 
 0 
 0 
 0 
 0 
 0 
 0 
 0 
 0 
 0 
 0 
 0 
 0 
 0 
 0 
 0 
 0 
 0 
 0 
 0 
 0 
 0 
 0 
 0 
 0 
 0 
 0 
 0 
 0 
 0 
 0 
 0 
 0 
 0 
 0 
 0 
 0 
 0 
 0 
 0 
 0 
 0 
 0 
 0 
 0 
 0 
 0 
 0 
 0 
 0 
 0 
 0 
 0 
 0 
 0 
 0 
 0 
 0 
 0 
 0 
 0 
 0 
 0 
 0 
 0 
 0 
 0 
 0 
 0 
 0 
 0 
 0 
 0 
 0 
 0 
 0 
 0 
 0 
 0 
 0 
 0 
 0 
 0 
 0 
 0 
 0 
 0 
 0 
 0 
 0 
 0 
 0 
 0 
 0 
 0 
 0 
 0 
 0 
 0 
 0 
 0 
 0 
 0 
 0 
 0 
 0 
 0 
 0 
 0 
 0 
 0 
 0 
 0 
 0 
 0 
 0 
 0 
 0 
 0 
 0 
 0 
 0 
 0 
 0 
 0 
 0 
 0 
 0 
 0 
 0 
 0 
 0 
 0 
 0 
 0 
 0 
 0 
 0 
 0 
 0 
 0 
 0 
 0 
 0 
 0 
 0 
 0 
 0 
 0 
 0 
 0 
 0 
 0 
 0 
 0 
 0 
 0 
 0 
 0 
 0 
 0 
 0 
 0 
 0 
 0 
 0 
 0 
 0 
 0 
 0 
 0 
 0 
 0 
 0 
 0 
 0 
 0 
 0 
 0 
 0 
 0 
 0 
 0 
 0 
 0 
 0 
 0 
 0 
 0 
 0 
 0 
 0 
 0 
 0 
 0 
 0 
 0 
 0 
 0 
 0 
 0 
 0 
 0 
 0 
 0 
 0 
 0 
 0 
 0 
 0 
 0 
 0 
 0 
 0 
 0 
 0 
 0 
 0 
 0 
 0 
 0 
 0 
 0 
 0 
 0 
 0 
 0 
 0 
 0 
 0 
 0 
 0 
 0 
 0 
 0 
 0 
 0 
 0 
 0 
 0 
 0 
 0 
 0 
 0 
 0 
 0 
 0 
 0 
 0 
 0 
 0 
 0 
 0 
 0 
 0 
 0 
 0 
 0 
 0 
 0 
 0 
 0 
 0 
 0 
 0 
 0 
 0 
 0 
 0 
 0 
 0 
 0 
 0 
 0 
 0 
 0 
 0 
 0 
 0 
 0 
 0 
 0 
 0 
 0 
 0 
 0 
 0 
 0 
 0 
 0 
 0 
 0 
 0 
 0 
 0 
 0 
 0 
 0 
 0 
 0 
 0 
 0 
 0 
 0 
 0 
 0 
 0 
 0 
 0 
 0 
 0 
 
 
 115 
 0 
 0 
 0 
 0 
 0 
 0 
 0 
 0 
 0 
 0 
 0 
 0 
 0 
 0 
 0 
 0 
 0 
 0 
 0 
 0 
 0 
 0 
 0 
 0 
 0 
 0 
 0 
 0 
 0 
 0 
 0 
 0 
 0 
 0 
 0 
 0 
 0 
 0 
 0 
 0 
 0 
 0 
 0 
 0 
 0 
 0 
 0 
 0 
 0 
 0 
 0 
 0 
 0 
 0 
 0 
 0 
 0 
 0 
 0 
 0 
 0 
 0 
 0 
 0 
 0 
 0 
 0 
 0 
 0 
 0 
 0 
 0 
 0 
 0 
 0 
 0 
 0 
 0 
 0 
 0 
 0 
 0 
 0 
 0 
 0 
 0 
 0 
 0 
 0 
 0 
 0 
 0 
 0 
 0 
 0 
 0 
 0 
 0 
 0 
 0 
 0 
 0 
 0 
 0 
 0 
 0 
 0 
 0 
 0 
 0 
 0 
 0 
 0 
 0 
 0 
 15 
 0 
 0 
 0 
 0 
 0 
 0 
 0 
 0 
 0 
 0 
 0 
 0 
 0 
 0 
 0 
 0 
 0 
 0 
 0 
 0 
 0 
 0 
 0 
 0 
 0 
 0 
 0 
 0 
 0 
 0 
 0 
 0 
 0 
 0 
 0 
 0 
 0 
 0 
 0 
 0 
 0 
 0 
 0 
 0 
 0 
 0 
 0 
 0 
 0 
 0 
 0 
 0 
 0 
 0 
 0 
 0 
 0 
 0 
 0 
 0 
 0 
 0 
 0 
 0 
 0 
 0 
 0 
 0 
 0 
 0 
 0 
 0 
 0 
 0 
 0 
 0 
 0 
 0 
 0 
 0 
 0 
 0 
 0 
 0 
 0 
 0 
 0 
 0 
 0 
 0 
 0 
 0 
 0 
 0 
 0 
 0 
 0 
 0 
 0 
 0 
 0 
 0 
 0 
 0 
 0 
 0 
 0 
 0 
 0 
 0 
 0 
 0 
 0 
 0 
 0 
 0 
 0 
 0 
 0 
 0 
 0 
 0 
 0 
 0 
 0 
 0 
 0 
 0 
 0 
 0 
 0 
 0 
 0 
 0 
 0 
 0 
 0 
 0 
 0 
 0 
 0 
 0 
 0 
 0 
 0 
 0 
 0 
 0 
 0 
 0 
 0 
 0 
 0 
 0 
 0 
 0 
 0 
 0 
 0 
 0 
 0 
 0 
 0 
 0 
 0 
 0 
 0 
 0 
 0 
 0 
 0 
 0 
 0 
 0 
 0 
 0 
 0 
 0 
 0 
 0 
 0 
 0 
 0 
 0 
 0 
 0 
 0 
 0 
 0 
 0 
 0 
 0 
 0 
 0 
 0 
 0 
 0 
 0 
 0 
 0 
 0 
 0 
 0 
 0 
 0 
 0 
 0 
 0 
 0 
 0 
 0 
 0 
 0 
 0 
 0 
 0 
 0 
 0 
 0 
 0 
 0 
 0 
 0 
 0 
 0 
 0 
 0 
 0 
 0 
 0 
 0 
 0 
 0 
 0 
 0 
 0 
 0 
 0 
 0 
 0 
 0 
 0 
 0 
 0 
 0 
 0 
 0 
 0 
 0 
 0 
 0 
 0 
 0 
 0 
 0 
 0 
 0 
 0 
 0 
 0 
 0 
 0 
 0 
 0 
 0 
 0 
 0 
 0 
 0 
 0 
 0 
 0 
 0 
 0 
 0 
 0 
 0 
 0 
 0 
 0 
 0 
 0 
 0 
 0 
 0 
 0 
 0 
 0 
 0 
 0 
 0 
 0 
 0 
 0 
 0 
 0 
 0 
 0 
 0 
 0 
 0 
 0 
 0 
 0 
 0 
 0 
 0 
 0 
 0 
 0 
 0 
 0 
 0 
 0 
 0 
 0 
 0 
 0 
 0 
 0 
 0 
 0 
 0 
 0 
 0 
 0 
 0 
 0 
 0 
 0 
 0 
 0 
 0 
 0 
 0 
 0 
 0 
 0 
 0 
 0 
 0 
 0 
 0 
 0 
 0 
 0 
 0 
 0 
 0 
 0 
 0 
 0 
 0 
 0 
 0 
 0 
 0 
 0 
 0 
 0 
 0 
 0 
 0 
 0 
 0 
 0 
 0 
 0 
 0 
 0 
 0 
 0 
 0 
 0 
 0 
 0 
 0 
 0 
 0 
 0 
 0 
 0 
 0 
 0 
 0 
 0 
 0 
 0 
 0 
 0 
 0 
 0 
 0 
 0 
 0 
 0 
 0 
 0 
 0 
 0 
 0 
 0 
 0 
 0 
 0 
 0 
 0 
 0 
 0 
 0 
 0 
 0 
 0 
 0 
 0 
 0 
 0 
 0 
 0 
 0 
 0 
 0 
 0 
 0 
 0 
 0 
 0 
 0 
 0 
 0 
 0 
 0 
 0 
 0 
 0 
 0 
 0 
 0 
 0 
 0 
 0 
 0 
 0 
 0 
 0 
 0 
 0 
 0 
 0 
 0 
 0 
 0 
 0 
 0 
 0 
 0 
 0 
 0 
 0 
 0 
 0 
 0 
 0 
 0 
 0 
 0 
 0 
 0 
 0 
 0 
 0 
 0 
 0 
 0 
 0 
 0 
 0 
 0 
 0 
 0 
 0 
 0 
 0 
 0 
 0 
 0 
 0 
 0 
 0 
 0 
 0 
 0 
 0 
 0 
 0 
 0 
 0 
 0 
 0 
 0 
 0 
 0 
 0 
 0 
 0 
 0 
 0 
 0 
 0 
 0 
 0 
 0 
 0 
 0 
 0 
 0 
 0 
 0 
 0 
 0 
 0 
 0 
 0 
 0 
 0 
 0 
 0 
 0 
 0 
 0 
 0 
 0 
 0 
 0 
 0 
 0 
 0 
 0 
 0 
 0 
 0 
 0 
 0 
 0 
 0 
 0 
 0 
 0 
 0 
 0 
 0 
 0 
 0 
 0 
 0 
 0 
 0 
 0 
 0 
 0 
 0 
 0 
 0 
 0 
 0 
 0 
 0 
 0 
 0 
 0 
 0 
 0 
 0 
 0 
 0 
 0 
 0 
 0 
 0 
 0 
 0 
 0 
 0 
 0 
 0 
 0 
 0 
 0 
 0 
 0 
 0 
 0 
 0 
 0 
 0 
 0 
 0 
 0 
 0 
 0 
 0 
 0 
 0 
 0 
 0 
 0 
 0 
 0 
 0 
 0 
 0 
 0 
 0 
 0 
 0 
 0 
 0 
 0 
 0 
 0 
 0 
 0 
 0 
 0 
 0 
 0 
 0 
 0 
 0 
 0 
 0 
 0 
 0 
 0 
 0 
 0 
 0 
 0 
 0 
 0 
 0 
 0 
 0 
 0 
 0 
 0 
 0 
 0 
 0 
 0 
 0 
 0 
 0 
 0 
 0 
 0 
 0 
 0 
 0 
 0 
 0 
 0 
 0 
 0 
 0 
 0 
 0 
 0 
 0 
 0 
 0 
 0 
 0 
 0 
 0 
 0 
 0 
 0 
 0 
 0 
 0 
 0 
 0 
 0 
 0 
 0 
 0 
 0 
 0 
 0 
 0 
 0 
 0 
 0 
 0 
 0 
 0 
 0 
 0 
 0 
 0 
 0 
 0 
 0 
 0 
 0 
 0 
 0 
 0 
 0 
 
 
 116 
 0 
 0 
 0 
 0 
 0 
 0 
 0 
 0 
 0 
 0 
 0 
 0 
 0 
 0 
 0 
 0 
 0 
 0 
 0 
 0 
 0 
 0 
 0 
 0 
 0 
 0 
 0 
 0 
 0 
 0 
 0 
 0 
 0 
 0 
 0 
 0 
 0 
 0 
 0 
 0 
 0 
 0 
 0 
 0 
 0 
 0 
 0 
 0 
 0 
 0 
 0 
 0 
 0 
 0 
 0 
 0 
 0 
 0 
 0 
 0 
 0 
 0 
 0 
 0 
 0 
 0 
 0 
 0 
 0 
 0 
 0 
 0 
 0 
 0 
 0 
 0 
 0 
 0 
 0 
 0 
 0 
 0 
 0 
 0 
 0 
 0 
 0 
 0 
 0 
 0 
 0 
 0 
 0 
 0 
 0 
 0 
 0 
 0 
 0 
 0 
 0 
 0 
 0 
 0 
 0 
 0 
 0 
 0 
 0 
 0 
 0 
 0 
 0 
 0 
 0 
 0 
 1 
 0 
 0 
 0 
 0 
 0 
 0 
 0 
 0 
 0 
 0 
 0 
 0 
 0 
 0 
 0 
 0 
 0 
 0 
 0 
 0 
 0 
 0 
 0 
 0 
 0 
 0 
 0 
 0 
 0 
 0 
 0 
 0 
 0 
 0 
 0 
 0 
 0 
 0 
 0 
 0 
 0 
 0 
 0 
 0 
 0 
 0 
 0 
 0 
 0 
 0 
 0 
 0 
 0 
 0 
 0 
 0 
 0 
 0 
 0 
 0 
 0 
 0 
 0 
 0 
 0 
 0 
 0 
 0 
 0 
 0 
 0 
 0 
 0 
 0 
 0 
 0 
 0 
 0 
 0 
 0 
 0 
 0 
 0 
 0 
 0 
 0 
 0 
 0 
 0 
 0 
 0 
 0 
 0 
 0 
 0 
 0 
 0 
 0 
 0 
 0 
 0 
 0 
 0 
 0 
 0 
 0 
 0 
 0 
 0 
 0 
 0 
 0 
 0 
 0 
 0 
 0 
 0 
 0 
 0 
 0 
 0 
 0 
 0 
 0 
 0 
 0 
 0 
 0 
 0 
 0 
 0 
 0 
 0 
 0 
 0 
 0 
 0 
 0 
 0 
 0 
 0 
 0 
 0 
 0 
 0 
 0 
 0 
 0 
 0 
 0 
 0 
 0 
 0 
 0 
 0 
 0 
 0 
 0 
 0 
 0 
 0 
 0 
 0 
 0 
 0 
 0 
 0 
 0 
 0 
 0 
 0 
 0 
 0 
 0 
 0 
 0 
 0 
 0 
 0 
 0 
 0 
 0 
 0 
 0 
 0 
 0 
 0 
 0 
 0 
 0 
 0 
 0 
 0 
 0 
 0 
 0 
 0 
 0 
 0 
 0 
 0 
 0 
 0 
 0 
 0 
 0 
 0 
 0 
 0 
 0 
 0 
 0 
 0 
 0 
 0 
 0 
 0 
 0 
 0 
 0 
 0 
 0 
 0 
 0 
 0 
 0 
 0 
 0 
 0 
 0 
 0 
 0 
 0 
 0 
 0 
 0 
 0 
 0 
 0 
 0 
 0 
 0 
 0 
 0 
 0 
 0 
 0 
 0 
 0 
 0 
 0 
 0 
 0 
 0 
 0 
 0 
 0 
 0 
 0 
 0 
 0 
 0 
 0 
 0 
 0 
 0 
 0 
 0 
 0 
 0 
 0 
 0 
 0 
 0 
 0 
 0 
 0 
 0 
 0 
 0 
 0 
 0 
 0 
 0 
 0 
 0 
 0 
 0 
 0 
 0 
 0 
 0 
 0 
 0 
 0 
 0 
 0 
 0 
 0 
 0 
 0 
 0 
 0 
 0 
 0 
 0 
 0 
 0 
 0 
 0 
 0 
 0 
 0 
 0 
 0 
 0 
 0 
 0 
 0 
 0 
 0 
 0 
 0 
 0 
 0 
 0 
 0 
 0 
 0 
 0 
 0 
 0 
 0 
 0 
 0 
 0 
 0 
 0 
 0 
 0 
 0 
 0 
 0 
 0 
 0 
 0 
 0 
 0 
 0 
 0 
 0 
 0 
 0 
 0 
 0 
 0 
 0 
 0 
 0 
 0 
 0 
 0 
 0 
 0 
 0 
 0 
 0 
 0 
 0 
 0 
 0 
 0 
 0 
 0 
 0 
 0 
 0 
 0 
 0 
 0 
 0 
 0 
 0 
 0 
 0 
 0 
 0 
 0 
 0 
 0 
 0 
 0 
 0 
 0 
 0 
 0 
 0 
 0 
 0 
 0 
 0 
 0 
 0 
 0 
 0 
 0 
 0 
 0 
 0 
 0 
 0 
 0 
 0 
 0 
 0 
 0 
 0 
 0 
 0 
 0 
 0 
 0 
 0 
 0 
 0 
 0 
 0 
 0 
 0 
 0 
 0 
 0 
 0 
 0 
 0 
 0 
 0 
 0 
 0 
 0 
 0 
 0 
 0 
 0 
 0 
 0 
 0 
 0 
 0 
 0 
 0 
 0 
 0 
 0 
 0 
 0 
 0 
 0 
 0 
 0 
 0 
 0 
 0 
 0 
 0 
 0 
 0 
 0 
 0 
 0 
 0 
 0 
 0 
 0 
 0 
 0 
 0 
 0 
 0 
 0 
 0 
 0 
 0 
 0 
 0 
 0 
 0 
 0 
 0 
 0 
 0 
 0 
 0 
 0 
 0 
 0 
 0 
 0 
 0 
 0 
 0 
 0 
 0 
 0 
 0 
 0 
 0 
 0 
 0 
 0 
 0 
 0 
 0 
 0 
 0 
 0 
 0 
 0 
 0 
 0 
 0 
 0 
 0 
 0 
 0 
 0 
 0 
 0 
 0 
 0 
 0 
 0 
 0 
 0 
 0 
 0 
 0 
 0 
 0 
 0 
 0 
 0 
 0 
 0 
 0 
 0 
 0 
 0 
 0 
 0 
 0 
 0 
 0 
 0 
 0 
 0 
 0 
 0 
 0 
 0 
 0 
 0 
 0 
 0 
 0 
 0 
 0 
 0 
 0 
 0 
 0 
 0 
 0 
 0 
 0 
 0 
 0 
 0 
 0 
 0 
 0 
 0 
 0 
 0 
 0 
 0 
 0 
 0 
 0 
 0 
 0 
 0 
 0 
 0 
 0 
 0 
 0 
 0 
 0 
 0 
 0 
 0 
 0 
 0 
 0 
 0 
 0 
 0 
 0 
 0 
 0 
 0 
 0 
 0 
 0 
 0 
 0 
 0 
 0 
 0 
 0 
 0 
 0 
 0 
 0 
 0 
 0 
 0 
 0 
 0 
 0 
 0 
 0 
 0 
 0 
 0 
 0 
 0 
 0 
 0 
 0 
 0 
 0 
 0 
 0 
 0 
 0 
 0 
 0 
 0 
 0 
 0 
 0 
 0 
 0 
 0 
 0 
 0 
 0 
 0 
 0 
 0 
 0 
 0 
 0 
 0 
 0 
 0 
 0 
 0 
 0 
 0 
 0 
 0 
 0 
 0 
 0 
 0 
 0 
 0 
 0 
 0 
 0 
 0 
 0 
 0 
 0 
 0 
 0 
 0 
 0 
 0 
 0 
 0 
 0 
 0 
 0 
 0 
 0 
 0 
 0 
 0 
 0 
 0 
 0 
 0 
 0 
 0 
 0 
 
 
 117 
 0 
 0 
 0 
 0 
 0 
 0 
 0 
 0 
 0 
 0 
 0 
 0 
 0 
 0 
 0 
 0 
 0 
 0 
 0 
 0 
 0 
 0 
 0 
 0 
 0 
 0 
 0 
 0 
 0 
 0 
 0 
 0 
 0 
 0 
 0 
 0 
 0 
 0 
 0 
 0 
 0 
 0 
 0 
 0 
 0 
 0 
 0 
 0 
 0 
 0 
 0 
 0 
 0 
 0 
 0 
 0 
 0 
 0 
 0 
 0 
 0 
 0 
 0 
 0 
 0 
 0 
 0 
 0 
 0 
 0 
 0 
 0 
 0 
 0 
 0 
 0 
 0 
 0 
 0 
 0 
 0 
 0 
 0 
 0 
 0 
 0 
 0 
 0 
 0 
 0 
 0 
 0 
 0 
 0 
 0 
 0 
 0 
 0 
 0 
 0 
 0 
 0 
 0 
 0 
 0 
 0 
 0 
 0 
 0 
 0 
 0 
 0 
 0 
 0 
 0 
 0 
 0 
 10 
 0 
 0 
 0 
 0 
 0 
 0 
 0 
 0 
 0 
 0 
 0 
 0 
 0 
 0 
 0 
 0 
 0 
 0 
 0 
 0 
 0 
 0 
 0 
 0 
 0 
 0 
 0 
 0 
 0 
 0 
 0 
 0 
 0 
 0 
 0 
 0 
 0 
 0 
 0 
 0 
 0 
 0 
 0 
 0 
 0 
 0 
 0 
 0 
 0 
 0 
 0 
 0 
 0 
 0 
 0 
 0 
 0 
 0 
 0 
 0 
 0 
 0 
 0 
 0 
 0 
 0 
 0 
 0 
 0 
 0 
 0 
 0 
 0 
 0 
 0 
 0 
 0 
 0 
 0 
 0 
 0 
 0 
 0 
 0 
 0 
 0 
 0 
 0 
 0 
 0 
 0 
 0 
 0 
 0 
 0 
 0 
 0 
 0 
 0 
 0 
 0 
 0 
 0 
 0 
 0 
 0 
 0 
 0 
 0 
 0 
 0 
 0 
 0 
 0 
 0 
 0 
 0 
 0 
 0 
 0 
 0 
 0 
 0 
 0 
 0 
 0 
 0 
 0 
 0 
 0 
 0 
 0 
 0 
 0 
 0 
 0 
 0 
 0 
 0 
 0 
 0 
 0 
 0 
 0 
 0 
 0 
 0 
 0 
 0 
 0 
 0 
 0 
 0 
 0 
 0 
 0 
 0 
 0 
 0 
 0 
 0 
 0 
 0 
 0 
 0 
 0 
 0 
 0 
 0 
 0 
 0 
 0 
 0 
 0 
 0 
 0 
 0 
 0 
 0 
 0 
 0 
 0 
 0 
 0 
 0 
 0 
 0 
 0 
 0 
 0 
 0 
 0 
 0 
 0 
 0 
 0 
 0 
 0 
 0 
 0 
 0 
 0 
 0 
 0 
 0 
 0 
 0 
 0 
 0 
 0 
 0 
 0 
 0 
 0 
 0 
 0 
 0 
 0 
 0 
 0 
 0 
 0 
 0 
 0 
 0 
 0 
 0 
 0 
 0 
 0 
 0 
 0 
 0 
 0 
 0 
 0 
 0 
 0 
 0 
 0 
 0 
 0 
 0 
 0 
 0 
 0 
 0 
 0 
 0 
 0 
 0 
 0 
 0 
 0 
 0 
 0 
 0 
 0 
 0 
 0 
 0 
 0 
 0 
 0 
 0 
 0 
 0 
 0 
 0 
 0 
 0 
 0 
 0 
 0 
 0 
 0 
 0 
 0 
 0 
 0 
 0 
 0 
 0 
 0 
 0 
 0 
 0 
 0 
 0 
 0 
 0 
 0 
 0 
 0 
 0 
 0 
 0 
 0 
 0 
 0 
 0 
 0 
 0 
 0 
 0 
 0 
 0 
 0 
 0 
 0 
 0 
 0 
 0 
 0 
 0 
 0 
 0 
 0 
 0 
 0 
 0 
 0 
 0 
 0 
 0 
 0 
 0 
 0 
 0 
 0 
 0 
 0 
 0 
 0 
 0 
 0 
 0 
 0 
 0 
 0 
 0 
 0 
 0 
 0 
 0 
 0 
 0 
 0 
 0 
 0 
 0 
 0 
 0 
 0 
 0 
 0 
 0 
 0 
 0 
 0 
 0 
 0 
 0 
 0 
 0 
 0 
 0 
 0 
 0 
 0 
 0 
 0 
 1 
 0 
 0 
 0 
 0 
 0 
 0 
 0 
 0 
 0 
 0 
 0 
 0 
 0 
 0 
 0 
 0 
 0 
 0 
 0 
 0 
 0 
 0 
 0 
 0 
 0 
 0 
 0 
 0 
 0 
 0 
 0 
 0 
 0 
 0 
 0 
 0 
 0 
 0 
 0 
 0 
 0 
 0 
 0 
 0 
 0 
 0 
 0 
 0 
 0 
 0 
 0 
 0 
 0 
 0 
 0 
 0 
 0 
 0 
 0 
 0 
 0 
 0 
 0 
 0 
 0 
 0 
 0 
 0 
 0 
 0 
 0 
 0 
 0 
 0 
 0 
 0 
 0 
 0 
 0 
 0 
 0 
 0 
 0 
 0 
 0 
 0 
 0 
 0 
 0 
 0 
 0 
 0 
 0 
 0 
 0 
 0 
 0 
 0 
 0 
 0 
 0 
 0 
 0 
 0 
 0 
 0 
 0 
 0 
 0 
 0 
 0 
 0 
 0 
 0 
 0 
 0 
 0 
 0 
 0 
 0 
 0 
 0 
 0 
 0 
 0 
 0 
 0 
 0 
 0 
 0 
 0 
 0 
 0 
 0 
 0 
 0 
 0 
 0 
 0 
 0 
 0 
 0 
 0 
 0 
 0 
 0 
 0 
 0 
 0 
 0 
 0 
 0 
 0 
 0 
 0 
 0 
 0 
 0 
 0 
 0 
 0 
 0 
 0 
 0 
 0 
 0 
 0 
 0 
 0 
 0 
 0 
 0 
 0 
 0 
 0 
 0 
 0 
 0 
 0 
 0 
 0 
 0 
 0 
 0 
 0 
 0 
 0 
 0 
 0 
 0 
 0 
 0 
 0 
 0 
 0 
 0 
 0 
 0 
 0 
 0 
 0 
 0 
 0 
 0 
 0 
 0 
 0 
 0 
 0 
 0 
 0 
 0 
 0 
 0 
 0 
 0 
 0 
 0 
 0 
 0 
 0 
 0 
 0 
 0 
 0 
 0 
 0 
 0 
 0 
 0 
 0 
 0 
 0 
 0 
 0 
 0 
 0 
 0 
 0 
 0 
 0 
 0 
 0 
 0 
 0 
 0 
 0 
 0 
 0 
 0 
 0 
 0 
 0 
 0 
 0 
 0 
 0 
 0 
 0 
 0 
 0 
 0 
 0 
 0 
 0 
 0 
 0 
 0 
 0 
 0 
 0 
 0 
 0 
 0 
 0 
 0 
 0 
 0 
 0 
 0 
 0 
 0 
 0 
 0 
 0 
 0 
 0 
 0 
 0 
 0 
 0 
 0 
 0 
 0 
 0 
 0 
 0 
 0 
 0 
 0 
 0 
 0 
 0 
 0 
 0 
 0 
 0 
 0 
 0 
 0 
 0 
 0 
 0 
 0 
 0 
 0 
 0 
 0 
 0 
 0 
 0 
 0 
 0 
 0 
 0 
 0 
 0 
 0 
 0 
 0 
 0 
 0 
 0 
 0 
 0 
 
 
 118 
 0 
 0 
 0 
 0 
 0 
 0 
 0 
 0 
 0 
 0 
 0 
 0 
 0 
 0 
 0 
 0 
 0 
 0 
 0 
 0 
 0 
 0 
 0 
 0 
 0 
 0 
 0 
 0 
 0 
 0 
 0 
 0 
 0 
 0 
 0 
 0 
 0 
 0 
 0 
 0 
 0 
 0 
 0 
 0 
 0 
 0 
 0 
 0 
 0 
 0 
 0 
 0 
 0 
 0 
 0 
 0 
 0 
 0 
 0 
 0 
 0 
 0 
 0 
 0 
 0 
 0 
 0 
 0 
 0 
 0 
 0 
 0 
 0 
 0 
 0 
 0 
 0 
 0 
 0 
 0 
 0 
 0 
 0 
 0 
 0 
 0 
 0 
 0 
 0 
 0 
 0 
 0 
 0 
 0 
 0 
 0 
 0 
 0 
 0 
 0 
 0 
 0 
 0 
 0 
 0 
 0 
 0 
 0 
 0 
 0 
 0 
 0 
 0 
 0 
 0 
 0 
 0 
 0 
 7 
 0 
 0 
 0 
 0 
 0 
 0 
 0 
 0 
 0 
 0 
 0 
 0 
 0 
 0 
 0 
 0 
 0 
 0 
 0 
 0 
 0 
 0 
 0 
 0 
 0 
 0 
 0 
 0 
 0 
 0 
 0 
 0 
 0 
 0 
 0 
 0 
 0 
 0 
 0 
 0 
 0 
 0 
 0 
 0 
 0 
 0 
 0 
 0 
 0 
 0 
 0 
 0 
 0 
 0 
 0 
 0 
 0 
 0 
 0 
 0 
 0 
 0 
 0 
 0 
 0 
 0 
 0 
 0 
 0 
 0 
 0 
 0 
 0 
 0 
 0 
 0 
 0 
 0 
 0 
 0 
 0 
 0 
 0 
 0 
 0 
 0 
 0 
 0 
 0 
 0 
 0 
 0 
 0 
 0 
 0 
 0 
 0 
 0 
 0 
 0 
 0 
 0 
 0 
 0 
 0 
 0 
 0 
 0 
 0 
 0 
 0 
 0 
 0 
 0 
 0 
 0 
 0 
 0 
 0 
 0 
 0 
 0 
 0 
 0 
 0 
 0 
 0 
 0 
 0 
 0 
 0 
 0 
 0 
 0 
 0 
 0 
 0 
 0 
 0 
 0 
 0 
 0 
 0 
 0 
 0 
 0 
 0 
 0 
 0 
 0 
 0 
 0 
 0 
 0 
 0 
 0 
 0 
 0 
 0 
 0 
 0 
 0 
 0 
 0 
 0 
 0 
 0 
 0 
 0 
 0 
 0 
 0 
 0 
 0 
 0 
 0 
 0 
 0 
 0 
 0 
 0 
 0 
 0 
 0 
 0 
 0 
 0 
 0 
 0 
 0 
 0 
 0 
 0 
 0 
 0 
 0 
 0 
 0 
 0 
 0 
 0 
 0 
 0 
 0 
 0 
 0 
 0 
 0 
 0 
 0 
 0 
 0 
 0 
 0 
 0 
 0 
 0 
 0 
 0 
 0 
 0 
 0 
 0 
 0 
 0 
 0 
 0 
 0 
 0 
 0 
 0 
 0 
 0 
 0 
 0 
 0 
 0 
 0 
 0 
 0 
 0 
 0 
 0 
 0 
 0 
 0 
 0 
 0 
 0 
 0 
 0 
 0 
 0 
 0 
 0 
 0 
 0 
 0 
 0 
 0 
 0 
 0 
 0 
 0 
 0 
 0 
 0 
 0 
 0 
 0 
 0 
 0 
 0 
 0 
 0 
 0 
 0 
 0 
 0 
 0 
 0 
 0 
 0 
 0 
 0 
 0 
 0 
 0 
 0 
 0 
 0 
 0 
 0 
 0 
 0 
 0 
 0 
 0 
 0 
 0 
 0 
 0 
 0 
 0 
 0 
 0 
 0 
 0 
 0 
 0 
 0 
 0 
 0 
 0 
 0 
 0 
 0 
 0 
 0 
 0 
 0 
 0 
 0 
 0 
 0 
 0 
 0 
 0 
 0 
 0 
 0 
 0 
 0 
 0 
 0 
 0 
 0 
 0 
 0 
 0 
 0 
 0 
 0 
 0 
 0 
 0 
 0 
 0 
 0 
 0 
 0 
 0 
 0 
 0 
 0 
 0 
 0 
 0 
 0 
 0 
 0 
 0 
 0 
 0 
 0 
 0 
 0 
 0 
 0 
 0 
 0 
 0 
 0 
 0 
 0 
 0 
 0 
 0 
 0 
 0 
 0 
 0 
 0 
 0 
 0 
 0 
 0 
 0 
 0 
 0 
 0 
 0 
 0 
 0 
 0 
 0 
 0 
 0 
 0 
 0 
 0 
 0 
 0 
 0 
 0 
 0 
 0 
 0 
 0 
 0 
 0 
 0 
 0 
 0 
 0 
 0 
 0 
 0 
 0 
 0 
 0 
 0 
 0 
 0 
 0 
 0 
 0 
 0 
 0 
 0 
 0 
 0 
 0 
 0 
 0 
 0 
 0 
 0 
 0 
 0 
 0 
 0 
 0 
 0 
 0 
 0 
 0 
 0 
 0 
 0 
 0 
 0 
 0 
 0 
 0 
 0 
 0 
 0 
 0 
 0 
 0 
 0 
 0 
 0 
 0 
 0 
 0 
 0 
 0 
 0 
 0 
 0 
 0 
 0 
 0 
 0 
 0 
 0 
 0 
 0 
 0 
 0 
 0 
 0 
 0 
 0 
 0 
 0 
 0 
 0 
 0 
 0 
 0 
 0 
 0 
 0 
 0 
 0 
 0 
 0 
 0 
 0 
 0 
 0 
 0 
 0 
 0 
 0 
 0 
 0 
 0 
 0 
 0 
 0 
 0 
 0 
 0 
 0 
 0 
 0 
 0 
 0 
 0 
 0 
 0 
 0 
 0 
 0 
 0 
 0 
 0 
 0 
 0 
 0 
 0 
 0 
 0 
 0 
 0 
 0 
 0 
 0 
 0 
 0 
 0 
 0 
 0 
 0 
 0 
 0 
 0 
 0 
 0 
 0 
 0 
 0 
 0 
 0 
 0 
 0 
 0 
 0 
 0 
 0 
 0 
 0 
 0 
 0 
 0 
 0 
 0 
 0 
 0 
 0 
 0 
 0 
 0 
 0 
 0 
 0 
 0 
 0 
 0 
 0 
 0 
 0 
 0 
 0 
 0 
 0 
 0 
 0 
 0 
 0 
 0 
 0 
 0 
 0 
 0 
 0 
 0 
 0 
 0 
 0 
 0 
 0 
 0 
 0 
 0 
 0 
 0 
 0 
 0 
 0 
 0 
 0 
 0 
 0 
 0 
 0 
 0 
 0 
 0 
 0 
 0 
 0 
 0 
 0 
 0 
 0 
 0 
 0 
 0 
 0 
 0 
 0 
 0 
 0 
 0 
 0 
 0 
 0 
 0 
 0 
 0 
 0 
 0 
 0 
 0 
 0 
 0 
 0 
 0 
 0 
 0 
 0 
 0 
 0 
 0 
 0 
 0 
 0 
 0 
 0 
 0 
 0 
 0 
 0 
 0 
 0 
 0 
 0 
 0 
 0 
 0 
 0 
 0 
 0 
 0 
 0 
 0 
 0 
 0 
 0 
 0 
 0 
 0 
 0 
 0 
 0 
 0 
 0 
 0 
 0 
 0 
 0 
 0 
 0 
 0 
 0 
 0 
 0 
 0 
 0 
 0 
 0 
 0 
 
 
 119 
 0 
 0 
 0 
 0 
 0 
 0 
 0 
 0 
 0 
 0 
 0 
 0 
 0 
 0 
 0 
 0 
 0 
 0 
 0 
 0 
 0 
 0 
 0 
 0 
 0 
 0 
 0 
 0 
 0 
 0 
 0 
 0 
 0 
 0 
 0 
 0 
 0 
 0 
 0 
 0 
 0 
 0 
 0 
 0 
 0 
 0 
 0 
 0 
 0 
 0 
 0 
 0 
 0 
 0 
 0 
 0 
 0 
 0 
 0 
 0 
 0 
 0 
 0 
 0 
 0 
 0 
 0 
 0 
 0 
 0 
 0 
 0 
 0 
 0 
 0 
 0 
 0 
 0 
 0 
 0 
 0 
 0
[truncated: 2,740,643 more chars]
