# Supplementary material for: TopEC: prediction of Enzyme Commission classes by 3D graph neural networks and localized 3D protein descriptor
Source: Nat Commun. 2025 Mar 20;16:2737. doi: 10.1038/s41467-025-57324-5 (PMC11923149; doi:10.1038/s41467-025-57324-5)
Supplement: Supplementary file 3 — Supplementary Data 1 [file 41467_2025_57324_MOESM3_ESM.zip › Data_S1/table1/hierarchical/TopEC_distance_TEMP_1digs.html]

PyCM Report


# PyCM Report

## Dataset Type :

- Multi-Class Classification
- Imbalanced

Note 1 : Recommended statistics for this type of classification highlighted in aqua

Note 2 : The recommender system assumes that the input is the result of classification over the whole data rather than just a part of it.
If the confusion matrix is the result of test data classification, the recommendation is not valid.

## Confusion Matrix :

|  |  |  |  |  |  |  |  |  |  |  |  |  |  |  |  |  |  |  |  |  |  |  |  |  |  |  |  |  |  |  |  |  |  |  |  |  |  |  |  |  |  |  |  |  |  |  |  |  |  |  |  |  |  |  |  |  |  |  |  |  |  |  |  |  |  |
| --- | --- | --- | --- | --- | --- | --- | --- | --- | --- | --- | --- | --- | --- | --- | --- | --- | --- | --- | --- | --- | --- | --- | --- | --- | --- | --- | --- | --- | --- | --- | --- | --- | --- | --- | --- | --- | --- | --- | --- | --- | --- | --- | --- | --- | --- | --- | --- | --- | --- | --- | --- | --- | --- | --- | --- | --- | --- | --- | --- | --- | --- | --- | --- | --- | --- |
| Actual | Predict  |  |  |  |  |  |  |  |  | | --- | --- | --- | --- | --- | --- | --- | --- | |  | 1 | 2 | 3 | 4 | 5 | 6 | 7 | | 1 | 192 | 12 | 12 | 2 | 0 | 0 | 6 | | 2 | 30 | 391 | 27 | 7 | 8 | 1 | 7 | | 3 | 18 | 9 | 396 | 4 | 2 | 0 | 12 | | 4 | 0 | 1 | 0 | 60 | 5 | 0 | 0 | | 5 | 0 | 0 | 2 | 0 | 33 | 0 | 0 | | 6 | 0 | 0 | 1 | 0 | 0 | 7 | 0 | | 7 | 6 | 12 | 14 | 0 | 4 | 0 | 16 | |

## Overall Statistics :

|  |  |
| --- | --- |
| 95% CI | (0.82452,0.86399) |
| ACC Macro | 0.9555 |
| ARI | 0.67138 |
| AUNP | 0.89831 |
| AUNU | 0.88689 |
| Bangdiwala B | 0.75857 |
| Bennett S | 0.8183 |
| CBA | 0.73228 |
| CSI | 0.55975 |
| Chi-Squared | 4564.53965 |
| Chi-Squared DF | 36 |
| Conditional Entropy | 0.86859 |
| Cramer V | 0.76587 |
| Cross Entropy | 2.09739 |
| F1 Macro | 0.77396 |
| F1 Micro | 0.84426 |
| FNR Macro | 0.19716 |
| FNR Micro | 0.15574 |
| FPR Macro | 0.02905 |
| FPR Micro | 0.02596 |
| Gwet AC1 | 0.82288 |
| Hamming Loss | 0.15574 |
| Joint Entropy | 2.95664 |
| KL Divergence | 0.00935 |
| Kappa | 0.78503 |
| Kappa 95% CI | (0.75779,0.81227) |
| Kappa No Prevalence | 0.68851 |
| Kappa Standard Error | 0.0139 |
| Kappa Unbiased | 0.78489 |
| Krippendorff Alpha | 0.78498 |
| Lambda A | 0.75545 |
| Lambda B | 0.76095 |
| Mutual Information | 1.26626 |
| NIR | 0.36315 |
| Overall ACC | 0.84426 |
| Overall CEN | 0.22409 |
| Overall J | (4.61786,0.65969) |
| Overall MCC | 0.78608 |
| Overall MCEN | 0.33326 |
| Overall RACC | 0.2755 |
| Overall RACCU | 0.27597 |
| P-Value | None |
| PPV Macro | 0.75691 |
| PPV Micro | 0.84426 |
| Pearson C | 0.88246 |
| Phi-Squared | 3.51931 |
| RCI | 0.60643 |
| RR | 185.28571 |
| Reference Entropy | 2.08805 |
| Response Entropy | 2.13485 |
| SOA1(Landis & Koch) | Substantial |
| SOA2(Fleiss) | Excellent |
| SOA3(Altman) | Good |
| SOA4(Cicchetti) | Excellent |
| SOA5(Cramer) | Strong |
| SOA6(Matthews) | Strong |
| Scott PI | 0.78489 |
| Standard Error | 0.01007 |
| TNR Macro | 0.97095 |
| TNR Micro | 0.97404 |
| TPR Macro | 0.80284 |
| TPR Micro | 0.84426 |
| Zero-one Loss | 202 |

## Class Statistics :

|  |  |  |  |  |  |  |  |  |
| --- | --- | --- | --- | --- | --- | --- | --- | --- |
| Class | 1 | 2 | 3 | 4 | 5 | 6 | 7 | Description |
| ACC | 0.93369 | 0.9121 | 0.92213 | 0.98535 | 0.98381 | 0.99846 | 0.95297 | Accuracy |
| AGF | 0.90091 | 0.88159 | 0.91854 | 0.94066 | 0.92502 | 0.93505 | 0.55913 | Adjusted F-score |
| AGM | 0.92371 | 0.91811 | 0.92344 | 0.96839 | 0.97416 | 0.96704 | 0.7601 | Adjusted geometric mean |
| AM | 22 | -46 | 11 | 7 | 17 | 0 | -11 | Difference between automatic and manual classification |
| AUC | 0.90341 | 0.89449 | 0.91627 | 0.94927 | 0.9639 | 0.93711 | 0.64381 | Area under the ROC curve |
| AUCI | Excellent | Very Good | Excellent | Excellent | Excellent | Excellent | Fair | AUC value interpretation |
| AUPR | 0.81882 | 0.87507 | 0.88703 | 0.8655 | 0.78874 | 0.875 | 0.34897 | Area under the PR curve |
| BCD | 0.00848 | 0.01773 | 0.00424 | 0.0027 | 0.00655 | 0.0 | 0.00424 | Bray-Curtis dissimilarity |
| BM | 0.80682 | 0.78899 | 0.83254 | 0.89853 | 0.9278 | 0.87422 | 0.28761 | Informedness or bookmaker informedness |
| CEN | 0.25049 | 0.2069 | 0.18953 | 0.18864 | 0.28116 | 0.13947 | 0.6025 | Confusion entropy |
| DOR | 113.22222 | 113.85 | 125.71429 | 936.92308 | 1079.44737 | 9016.0 | 21.68889 | Diagnostic odds ratio |
| DP | 1.13239 | 1.13372 | 1.15745 | 1.63839 | 1.67229 | 2.18051 | 0.73671 | Discriminant power |
| DPI | Limited | Limited | Limited | Limited | Limited | Fair | Poor | Discriminant power interpretation |
| ERR | 0.06631 | 0.0879 | 0.07787 | 0.01465 | 0.01619 | 0.00154 | 0.04703 | Error rate |
| F0.5 | 0.7947 | 0.90051 | 0.88039 | 0.83799 | 0.67901 | 0.875 | 0.37037 | F0.5 score |
| F1 | 0.81702 | 0.87277 | 0.8869 | 0.86331 | 0.75862 | 0.875 | 0.34409 | F1 score - harmonic mean of precision and sensitivity |
| F2 | 0.84063 | 0.84669 | 0.8935 | 0.89021 | 0.85938 | 0.875 | 0.32129 | F2 score |
| FDR | 0.21951 | 0.08 | 0.12389 | 0.17808 | 0.36538 | 0.125 | 0.60976 | False discovery rate |
| FN | 32 | 80 | 45 | 6 | 2 | 1 | 36 | False negative/miss/type 2 error |
| FNR | 0.14286 | 0.16985 | 0.10204 | 0.09091 | 0.05714 | 0.125 | 0.69231 | Miss rate or false negative rate |
| FOR | 0.03045 | 0.09174 | 0.05325 | 0.0049 | 0.00161 | 0.00078 | 0.02866 | False omission rate |
| FP | 54 | 34 | 56 | 13 | 19 | 1 | 25 | False positive/type 1 error/false alarm |
| FPR | 0.05033 | 0.04116 | 0.06542 | 0.01056 | 0.01506 | 0.00078 | 0.02008 | Fall-out or false positive rate |
| G | 0.81792 | 0.87392 | 0.88697 | 0.86441 | 0.77353 | 0.875 | 0.34652 | G-measure geometric mean of precision and sensitivity |
| GI | 0.80682 | 0.78899 | 0.83254 | 0.89853 | 0.9278 | 0.87422 | 0.28761 | Gini index |
| GM | 0.90222 | 0.89218 | 0.91609 | 0.94841 | 0.96367 | 0.93505 | 0.5491 | G-mean geometric mean of specificity and sensitivity |
| IBA | 0.73869 | 0.69354 | 0.80848 | 0.82722 | 0.88958 | 0.76571 | 0.09883 | Index of balanced accuracy |
| ICSI | 0.63763 | 0.75015 | 0.77407 | 0.73101 | 0.57747 | 0.75 | -0.30206 | Individual classification success index |
| IS | 2.17606 | 1.34109 | 1.36551 | 4.01363 | 4.55563 | 7.14832 | 3.28297 | Information score |
| J | 0.69065 | 0.77426 | 0.79678 | 0.75949 | 0.61111 | 0.77778 | 0.20779 | Jaccard index |
| LS | 4.51916 | 2.53342 | 2.57667 | 16.15193 | 23.51703 | 141.85938 | 9.73358 | Lift score |
| MCC | 0.77791 | 0.80838 | 0.82768 | 0.8568 | 0.76636 | 0.87422 | 0.32248 | Matthews correlation coefficient |
| MCCI | Strong | Strong | Strong | Strong | Strong | Strong | Weak | Matthews correlation coefficient interpretation |
| MCEN | 0.35812 | 0.315 | 0.29261 | 0.27722 | 0.37834 | 0.1965 | 0.66751 | Modified confusion entropy |
| MK | 0.75004 | 0.82826 | 0.82285 | 0.81702 | 0.63301 | 0.87422 | 0.36158 | Markedness |
| N | 1073 | 826 | 856 | 1231 | 1262 | 1289 | 1245 | Condition negative |
| NLR | 0.15043 | 0.17714 | 0.10918 | 0.09188 | 0.05802 | 0.1251 | 0.70649 | Negative likelihood ratio |
| NLRI | Fair | Fair | Fair | Good | Good | Fair | Negligible | Negative likelihood ratio interpretation |
| NPV | 0.96955 | 0.90826 | 0.94675 | 0.9951 | 0.99839 | 0.99922 | 0.97134 | Negative predictive value |
| OC | 0.85714 | 0.92 | 0.89796 | 0.90909 | 0.94286 | 0.875 | 0.39024 | Overlap coefficient |
| OOC | 0.81792 | 0.87392 | 0.88697 | 0.86441 | 0.77353 | 0.875 | 0.34652 | Otsuka-Ochiai coefficient |
| OP | 0.88248 | 0.84017 | 0.90214 | 0.94303 | 0.96198 | 0.93218 | 0.4309 | Optimized precision |
| P | 224 | 471 | 441 | 66 | 35 | 8 | 52 | Condition positive or support |
| PLR | 17.03175 | 20.16773 | 13.72595 | 86.08392 | 62.62556 | 1127.875 | 15.32308 | Positive likelihood ratio |
| PLRI | Good | Good | Good | Good | Good | Good | Good | Positive likelihood ratio interpretation |
| POP | 1297 | 1297 | 1297 | 1297 | 1297 | 1297 | 1297 | Population |
| PPV | 0.78049 | 0.92 | 0.87611 | 0.82192 | 0.63462 | 0.875 | 0.39024 | Precision or positive predictive value |
| PRE | 0.17271 | 0.36315 | 0.34002 | 0.05089 | 0.02699 | 0.00617 | 0.04009 | Prevalence |
| Q | 0.98249 | 0.98259 | 0.98422 | 0.99787 | 0.99815 | 0.99978 | 0.91185 | Yule Q - coefficient of colligation |
| QI | Strong | Strong | Strong | Strong | Strong | Strong | Strong | Yule Q interpretation |
| RACC | 0.03276 | 0.119 | 0.11849 | 0.00286 | 0.00108 | 4e-05 | 0.00127 | Random accuracy |
| RACCU | 0.03283 | 0.11931 | 0.11851 | 0.00287 | 0.00112 | 4e-05 | 0.00129 | Random accuracy unbiased |
| TN | 1019 | 792 | 800 | 1218 | 1243 | 1288 | 1220 | True negative/correct rejection |
| TNR | 0.94967 | 0.95884 | 0.93458 | 0.98944 | 0.98494 | 0.99922 | 0.97992 | Specificity or true negative rate |
| TON | 1051 | 872 | 845 | 1224 | 1245 | 1289 | 1256 | Test outcome negative |
| TOP | 246 | 425 | 452 | 73 | 52 | 8 | 41 | Test outcome positive |
| TP | 192 | 391 | 396 | 60 | 33 | 7 | 16 | True positive/hit |
| TPR | 0.85714 | 0.83015 | 0.89796 | 0.90909 | 0.94286 | 0.875 | 0.30769 | Sensitivity, recall, hit rate, or true positive rate |
| Y | 0.80682 | 0.78899 | 0.83254 | 0.89853 | 0.9278 | 0.87422 | 0.28761 | Youden index |
| dInd | 0.15146 | 0.17477 | 0.12121 | 0.09152 | 0.05909 | 0.125 | 0.6926 | Distance index |
| sInd | 0.8929 | 0.87642 | 0.91429 | 0.93529 | 0.95821 | 0.91161 | 0.51026 | Similarity index |

Generated By PyCM Version 3.4
