# Supplementary material for: TopEC: prediction of Enzyme Commission classes by 3D graph neural networks and localized 3D protein descriptor
Source: Nat Commun. 2025 Mar 20;16:2737. doi: 10.1038/s41467-025-57324-5 (PMC11923149; doi:10.1038/s41467-025-57324-5)
Supplement: Supplementary file 3 — Supplementary Data 1 [file 41467_2025_57324_MOESM3_ESM.zip › Data_S1/table1/hierarchical/TopEC_distance_TEMP_3digs.html]

PyCM Report


# PyCM Report

## Dataset Type :

- Multi-Class Classification
- Imbalanced

Note 1 : Recommended statistics for this type of classification highlighted in aqua

Note 2 : The recommender system assumes that the input is the result of classification over the whole data rather than just a part of it.
If the confusion matrix is the result of test data classification, the recommendation is not valid.

## Confusion Matrix :

|  |  |  |  |  |  |  |  |  |  |  |  |  |  |  |  |  |  |  |  |  |  |  |  |  |  |  |  |  |  |  |  |  |  |  |  |  |  |  |  |  |  |  |  |  |  |  |  |  |  |  |  |  |  |  |  |  |  |  |  |  |  |  |  |  |  |  |  |  |  |  |  |  |  |  |  |  |  |  |  |  |  |  |  |  |  |  |  |  |  |  |  |  |  |  |  |  |  |  |  |  |  |  |  |  |  |  |  |  |  |  |  |  |  |  |  |  |  |  |  |  |  |  |  |  |  |  |  |  |  |  |  |  |  |  |  |  |  |  |  |  |  |  |  |  |  |  |  |  |  |  |  |  |  |  |  |  |  |  |  |  |  |  |  |  |  |  |  |  |  |  |  |  |  |  |  |  |  |  |  |  |  |  |  |  |  |  |  |  |  |  |  |  |  |  |  |  |  |  |  |  |  |  |  |  |  |  |  |  |  |  |  |  |  |  |  |  |  |  |  |  |  |  |  |  |  |  |  |  |  |  |  |  |  |  |  |  |  |  |  |  |  |  |  |  |  |  |  |  |  |  |  |  |  |  |  |  |  |  |  |  |  |  |  |  |  |  |  |  |  |  |  |  |  |  |  |  |  |  |  |  |  |  |  |  |  |  |  |  |  |  |  |  |  |  |  |  |  |  |  |  |  |  |  |  |  |  |  |  |  |  |  |  |  |  |  |  |  |  |  |  |  |  |  |  |  |  |  |  |  |  |  |  |  |  |  |  |  |  |  |  |  |  |  |  |  |  |  |  |  |  |  |  |  |  |  |  |  |  |  |  |  |  |  |  |  |  |  |  |  |  |  |  |  |  |  |  |  |  |  |  |  |  |  |  |  |  |  |  |  |  |  |  |  |  |  |  |  |  |  |  |  |  |  |  |  |  |  |  |  |  |  |  |  |  |  |  |  |  |  |  |  |  |  |  |  |  |  |  |  |  |  |  |  |  |  |  |  |  |  |  |  |  |  |  |  |  |  |  |  |  |  |  |  |  |  |  |  |  |  |  |  |  |  |  |  |  |  |  |  |  |  |  |  |  |  |  |  |  |  |  |  |  |  |  |  |  |  |  |  |  |  |  |  |  |  |  |  |  |  |  |  |  |  |  |  |  |  |  |  |  |  |  |  |  |  |  |  |  |  |  |  |  |  |  |  |  |  |  |  |  |  |  |  |  |  |  |  |  |  |  |  |  |  |  |  |  |  |  |  |  |  |  |  |  |  |  |  |  |  |  |  |  |  |  |  |  |  |  |  |  |  |  |  |  |  |  |  |  |  |  |  |  |  |  |  |  |  |  |  |  |  |  |  |  |  |  |  |  |  |  |  |  |  |  |  |  |  |  |  |  |  |  |  |  |  |  |  |  |  |  |  |  |  |  |  |  |  |  |  |  |  |  |  |  |  |  |  |  |  |  |  |  |  |  |  |  |  |  |  |  |  |  |  |  |  |  |  |  |  |  |  |  |  |  |  |  |  |  |  |  |  |  |  |  |  |  |  |  |  |  |  |  |  |  |  |  |  |  |  |  |  |  |  |  |  |  |  |  |  |  |  |  |  |  |  |  |  |  |  |  |  |  |  |  |  |  |  |  |  |  |  |  |  |  |  |  |  |  |  |  |  |  |  |  |  |  |  |  |  |  |  |  |  |  |  |  |  |  |  |  |  |  |  |  |  |  |  |  |  |  |  |  |  |  |  |  |  |  |  |  |  |  |  |  |  |  |  |  |  |  |  |  |  |  |  |  |  |  |  |  |  |  |  |  |  |  |  |  |  |  |  |  |  |  |  |  |  |  |  |  |  |  |  |  |  |  |  |  |  |  |  |  |  |  |  |  |  |  |  |  |  |  |  |  |  |  |  |  |  |  |  |  |  |  |  |  |  |  |  |  |  |  |  |  |  |  |  |  |  |  |  |  |  |  |  |  |  |  |  |  |  |  |  |  |  |  |  |  |  |  |  |  |  |  |  |  |  |  |  |  |  |  |  |  |  |  |  |  |  |  |  |  |  |  |  |  |  |  |  |  |  |  |  |  |  |  |  |  |  |  |  |  |  |  |  |  |  |  |  |  |  |  |  |  |  |  |  |  |  |  |  |  |  |  |  |  |  |  |  |  |  |  |  |  |  |  |  |  |  |  |  |  |  |  |  |  |  |  |  |  |  |  |  |  |  |  |  |  |  |  |  |  |  |  |  |  |  |  |  |  |  |  |  |  |  |  |  |  |  |  |  |  |  |  |  |  |  |  |  |  |  |  |  |  |  |  |  |  |  |  |  |  |  |  |  |  |  |  |  |  |  |  |  |  |  |  |  |  |  |  |  |  |  |  |  |  |  |  |  |  |  |  |  |  |  |  |  |  |  |  |  |  |  |  |  |  |  |  |  |  |  |  |  |  |  |  |  |  |  |  |  |  |  |  |  |  |  |  |  |  |  |  |  |  |  |  |  |  |  |  |  |  |  |  |  |  |  |  |  |  |  |  |  |  |  |  |  |  |  |  |  |  |  |  |  |  |  |  |  |  |  |  |  |  |  |  |  |  |  |  |  |  |  |  |  |  |  |  |  |  |  |  |  |  |  |  |  |  |  |  |  |  |  |  |  |  |  |  |  |  |  |  |  |  |  |  |  |  |  |  |  |  |  |  |  |  |  |  |  |  |  |  |  |  |  |  |  |  |  |  |  |  |  |  |  |  |  |  |  |  |  |  |  |  |  |  |  |  |  |  |  |  |  |  |  |  |  |  |  |  |  |  |  |  |  |  |  |  |  |  |  |  |  |  |  |  |  |  |  |  |  |  |  |  |  |  |  |  |  |  |  |  |  |  |  |  |  |  |  |  |  |  |  |  |  |  |  |  |  |  |  |  |  |  |  |  |  |  |  |  |  |  |  |  |  |  |  |  |  |  |  |  |  |  |  |  |  |  |  |  |  |  |  |  |  |  |  |  |  |  |  |  |  |  |  |  |  |  |  |  |  |  |  |  |  |  |  |  |  |  |  |  |  |  |  |  |  |  |  |  |  |  |  |  |  |  |  |  |  |  |  |  |  |  |  |  |  |  |  |  |  |  |  |  |  |  |  |  |  |  |  |  |  |  |  |  |  |  |  |  |  |  |  |  |  |  |  |  |  |  |  |  |  |  |  |  |  |  |  |  |  |  |  |  |  |  |  |  |  |  |  |  |  |  |  |  |  |  |  |  |  |  |  |  |  |  |  |  |  |  |  |  |  |  |  |  |  |  |  |  |  |  |  |  |  |  |  |  |  |  |  |  |  |  |  |  |  |  |  |  |  |  |  |  |  |  |  |  |  |  |  |  |  |  |  |  |  |  |  |  |  |  |  |  |  |  |  |  |  |  |  |  |  |  |  |  |  |  |  |  |  |  |  |  |  |  |  |  |  |  |  |  |  |  |  |  |  |  |  |  |  |  |  |  |  |  |  |  |  |  |  |  |  |  |  |  |  |  |  |  |  |  |  |  |  |  |  |  |  |  |  |  |  |  |  |  |  |  |  |  |  |  |  |  |  |  |  |  |  |  |  |  |  |  |  |  |  |  |  |  |  |  |  |  |  |  |  |  |  |  |  |  |  |  |  |  |  |  |  |  |  |  |  |  |  |  |  |  |  |  |  |  |  |  |  |  |  |  |  |  |  |  |  |  |  |  |  |  |  |  |  |  |  |  |  |  |  |  |  |  |  |  |  |  |  |  |  |  |  |  |  |  |  |  |  |  |  |  |  |  |  |  |  |  |  |  |  |  |  |  |  |  |  |  |  |  |  |  |  |  |  |  |  |  |  |  |  |  |  |  |  |  |  |  |  |  |  |  |  |  |  |  |  |  |  |  |  |  |  |  |  |  |  |  |  |  |  |  |  |  |  |  |  |  |  |  |  |  |  |  |  |  |  |  |  |  |  |  |  |  |  |  |  |  |  |  |  |  |  |  |  |  |  |  |  |  |  |  |  |  |  |  |  |  |  |  |  |  |  |  |  |  |  |  |  |  |  |  |  |  |  |  |  |  |  |  |  |  |  |  |  |  |  |  |  |  |  |  |  |  |  |  |  |  |  |  |  |  |  |  |  |  |  |  |  |  |  |  |  |  |  |  |  |  |  |  |  |  |  |  |  |  |  |  |  |  |  |  |  |  |  |  |  |  |  |  |  |  |  |  |  |  |  |  |  |  |  |  |  |  |  |  |  |  |  |  |  |  |  |  |  |  |  |  |  |  |  |  |  |  |  |  |  |  |  |  |  |  |  |  |  |  |  |  |  |  |  |  |  |  |  |  |  |  |  |  |  |  |  |  |  |  |  |  |  |  |  |  |  |  |  |  |  |  |  |  |  |  |  |  |  |  |  |  |  |  |  |  |  |  |  |  |  |  |  |  |  |  |  |  |  |  |  |  |  |  |  |  |  |  |  |  |  |  |  |  |  |  |  |  |  |  |  |  |  |  |  |  |  |  |  |  |  |  |  |  |  |  |  |  |  |  |  |  |  |  |  |  |  |  |  |  |  |  |  |  |  |  |  |  |  |  |  |  |  |  |  |  |  |  |  |  |  |  |  |  |  |  |  |  |  |  |  |  |  |  |  |  |  |  |  |  |  |  |  |  |  |  |  |  |  |  |  |  |  |  |  |  |  |  |  |  |  |  |  |  |  |  |  |  |  |  |  |  |  |  |  |  |  |  |  |  |  |  |  |  |  |  |  |  |  |  |  |  |  |  |  |  |  |  |  |  |  |  |  |  |  |  |  |  |  |  |  |  |  |  |  |  |  |  |  |  |  |  |  |  |  |  |  |  |  |  |  |  |  |  |  |  |  |  |  |  |  |  |  |  |  |  |  |  |  |  |  |  |  |  |  |  |  |  |  |  |  |  |  |  |  |  |  |  |  |  |  |  |  |  |  |  |  |  |  |  |  |  |  |  |  |  |  |  |  |  |  |  |  |  |  |  |  |  |  |  |  |  |  |  |  |  |  |  |  |  |  |  |  |  |  |  |  |  |  |  |  |  |  |  |  |  |  |  |  |  |  |  |  |  |  |  |  |  |  |  |  |  |  |  |  |  |  |  |  |  |  |  |  |  |  |  |  |  |  |  |  |  |  |  |  |  |  |  |  |  |  |  |  |  |  |  |  |  |  |  |  |  |  |  |  |  |  |  |  |  |  |  |  |  |  |  |  |  |  |  |  |  |  |  |  |  |  |  |  |  |  |  |  |  |  |  |  |  |  |  |  |  |  |  |  |  |  |  |  |  |  |  |  |  |  |  |  |  |  |  |  |  |  |  |  |  |  |  |  |  |  |  |  |  |  |  |  |  |  |  |  |  |  |  |  |  |  |  |  |  |  |  |  |  |  |  |  |  |  |  |  |  |  |  |  |  |  |  |  |  |  |  |  |  |  |  |  |  |  |  |  |  |  |  |  |  |  |  |  |  |  |  |  |  |  |  |  |  |  |  |  |  |  |  |  |  |  |  |  |  |  |  |  |  |  |  |  |  |  |  |  |  |  |  |  |  |  |  |  |  |  |  |  |  |  |  |  |  |  |  |  |  |  |  |  |  |  |  |  |  |  |  |  |  |  |  |  |  |  |  |  |  |  |  |  |  |  |  |  |  |  |  |  |  |  |  |  |  |  |  |  |  |  |  |  |  |  |  |  |  |  |  |  |  |  |  |  |  |  |  |  |  |  |  |  |  |  |  |  |  |  |  |  |  |  |  |  |  |  |  |  |  |  |  |  |  |  |  |  |  |  |  |  |  |  |  |  |  |  |  |  |  |  |  |  |  |  |  |  |  |  |  |  |  |  |  |  |  |  |  |  |  |  |  |  |  |  |  |  |  |  |  |  |  |  |  |  |  |  |  |  |  |  |  |  |  |  |  |  |  |  |  |  |  |  |  |  |  |  |  |  |  |  |  |  |  |  |  |  |  |  |  |  |  |  |  |  |  |  |  |  |  |  |  |  |  |  |  |  |  |  |  |  |  |  |  |  |  |  |  |  |  |  |  |  |  |  |  |  |  |  |  |  |  |  |  |  |  |  |  |  |  |  |  |  |  |  |  |  |  |  |  |  |  |  |  |  |  |  |  |  |  |  |  |  |  |  |  |  |  |  |  |  |  |  |  |  |  |  |  |  |  |  |  |  |  |  |  |  |  |  |  |  |  |  |  |  |  |  |  |  |  |  |  |  |  |  |  |  |  |  |  |  |  |  |  |  |  |  |  |  |  |  |  |  |  |  |  |  |  |  |  |  |  |  |  |  |  |  |  |  |  |  |  |  |  |  |  |  |  |  |  |  |  |  |  |  |  |
| --- | --- | --- | --- | --- | --- | --- | --- | --- | --- | --- | --- | --- | --- | --- | --- | --- | --- | --- | --- | --- | --- | --- | --- | --- | --- | --- | --- | --- | --- | --- | --- | --- | --- | --- | --- | --- | --- | --- | --- | --- | --- | --- | --- | --- | --- | --- | --- | --- | --- | --- | --- | --- | --- | --- | --- | --- | --- | --- | --- | --- | --- | --- | --- | --- | --- | --- | --- | --- | --- | --- | --- | --- | --- | --- | --- | --- | --- | --- | --- | --- | --- | --- | --- | --- | --- | --- | --- | --- | --- | --- | --- | --- | --- | --- | --- | --- | --- | --- | --- | --- | --- | --- | --- | --- | --- | --- | --- | --- | --- | --- | --- | --- | --- | --- | --- | --- | --- | --- | --- | --- | --- | --- | --- | --- | --- | --- | --- | --- | --- | --- | --- | --- | --- | --- | --- | --- | --- | --- | --- | --- | --- | --- | --- | --- | --- | --- | --- | --- | --- | --- | --- | --- | --- | --- | --- | --- | --- | --- | --- | --- | --- | --- | --- | --- | --- | --- | --- | --- | --- | --- | --- | --- | --- | --- | --- | --- | --- | --- | --- | --- | --- | --- | --- | --- | --- | --- | --- | --- | --- | --- | --- | --- | --- | --- | --- | --- | --- | --- | --- | --- | --- | --- | --- | --- | --- | --- | --- | --- | --- | --- | --- | --- | --- | --- | --- | --- | --- | --- | --- | --- | --- | --- | --- | --- | --- | --- | --- | --- | --- | --- | --- | --- | --- | --- | --- | --- | --- | --- | --- | --- | --- | --- | --- | --- | --- | --- | --- | --- | --- | --- | --- | --- | --- | --- | --- | --- | --- | --- | --- | --- | --- | --- | --- | --- | --- | --- | --- | --- | --- | --- | --- | --- | --- | --- | --- | --- | --- | --- | --- | --- | --- | --- | --- | --- | --- | --- | --- | --- | --- | --- | --- | --- | --- | --- | --- | --- | --- | --- | --- | --- | --- | --- | --- | --- | --- | --- | --- | --- | --- | --- | --- | --- | --- | --- | --- | --- | --- | --- | --- | --- | --- | --- | --- | --- | --- | --- | --- | --- | --- | --- | --- | --- | --- | --- | --- | --- | --- | --- | --- | --- | --- | --- | --- | --- | --- | --- | --- | --- | --- | --- | --- | --- | --- | --- | --- | --- | --- | --- | --- | --- | --- | --- | --- | --- | --- | --- | --- | --- | --- | --- | --- | --- | --- | --- | --- | --- | --- | --- | --- | --- | --- | --- | --- | --- | --- | --- | --- | --- | --- | --- | --- | --- | --- | --- | --- | --- | --- | --- | --- | --- | --- | --- | --- | --- | --- | --- | --- | --- | --- | --- | --- | --- | --- | --- | --- | --- | --- | --- | --- | --- | --- | --- | --- | --- | --- | --- | --- | --- | --- | --- | --- | --- | --- | --- | --- | --- | --- | --- | --- | --- | --- | --- | --- | --- | --- | --- | --- | --- | --- | --- | --- | --- | --- | --- | --- | --- | --- | --- | --- | --- | --- | --- | --- | --- | --- | --- | --- | --- | --- | --- | --- | --- | --- | --- | --- | --- | --- | --- | --- | --- | --- | --- | --- | --- | --- | --- | --- | --- | --- | --- | --- | --- | --- | --- | --- | --- | --- | --- | --- | --- | --- | --- | --- | --- | --- | --- | --- | --- | --- | --- | --- | --- | --- | --- | --- | --- | --- | --- | --- | --- | --- | --- | --- | --- | --- | --- | --- | --- | --- | --- | --- | --- | --- | --- | --- | --- | --- | --- | --- | --- | --- | --- | --- | --- | --- | --- | --- | --- | --- | --- | --- | --- | --- | --- | --- | --- | --- | --- | --- | --- | --- | --- | --- | --- | --- | --- | --- | --- | --- | --- | --- | --- | --- | --- | --- | --- | --- | --- | --- | --- | --- | --- | --- | --- | --- | --- | --- | --- | --- | --- | --- | --- | --- | --- | --- | --- | --- | --- | --- | --- | --- | --- | --- | --- | --- | --- | --- | --- | --- | --- | --- | --- | --- | --- | --- | --- | --- | --- | --- | --- | --- | --- | --- | --- | --- | --- | --- | --- | --- | --- | --- | --- | --- | --- | --- | --- | --- | --- | --- | --- | --- | --- | --- | --- | --- | --- | --- | --- | --- | --- | --- | --- | --- | --- | --- | --- | --- | --- | --- | --- | --- | --- | --- | --- | --- | --- | --- | --- | --- | --- | --- | --- | --- | --- | --- | --- | --- | --- | --- | --- | --- | --- | --- | --- | --- | --- | --- | --- | --- | --- | --- | --- | --- | --- | --- | --- | --- | --- | --- | --- | --- | --- | --- | --- | --- | --- | --- | --- | --- | --- | --- | --- | --- | --- | --- | --- | --- | --- | --- | --- | --- | --- | --- | --- | --- | --- | --- | --- | --- | --- | --- | --- | --- | --- | --- | --- | --- | --- | --- | --- | --- | --- | --- | --- | --- | --- | --- | --- | --- | --- | --- | --- | --- | --- | --- | --- | --- | --- | --- | --- | --- | --- | --- | --- | --- | --- | --- | --- | --- | --- | --- | --- | --- | --- | --- | --- | --- | --- | --- | --- | --- | --- | --- | --- | --- | --- | --- | --- | --- | --- | --- | --- | --- | --- | --- | --- | --- | --- | --- | --- | --- | --- | --- | --- | --- | --- | --- | --- | --- | --- | --- | --- | --- | --- | --- | --- | --- | --- | --- | --- | --- | --- | --- | --- | --- | --- | --- | --- | --- | --- | --- | --- | --- | --- | --- | --- | --- | --- | --- | --- | --- | --- | --- | --- | --- | --- | --- | --- | --- | --- | --- | --- | --- | --- | --- | --- | --- | --- | --- | --- | --- | --- | --- | --- | --- | --- | --- | --- | --- | --- | --- | --- | --- | --- | --- | --- | --- | --- | --- | --- | --- | --- | --- | --- | --- | --- | --- | --- | --- | --- | --- | --- | --- | --- | --- | --- | --- | --- | --- | --- | --- | --- | --- | --- | --- | --- | --- | --- | --- | --- | --- | --- | --- | --- | --- | --- | --- | --- | --- | --- | --- | --- | --- | --- | --- | --- | --- | --- | --- | --- | --- | --- | --- | --- | --- | --- | --- | --- | --- | --- | --- | --- | --- | --- | --- | --- | --- | --- | --- | --- | --- | --- | --- | --- | --- | --- | --- | --- | --- | --- | --- | --- | --- | --- | --- | --- | --- | --- | --- | --- | --- | --- | --- | --- | --- | --- | --- | --- | --- | --- | --- | --- | --- | --- | --- | --- | --- | --- | --- | --- | --- | --- | --- | --- | --- | --- | --- | --- | --- | --- | --- | --- | --- | --- | --- | --- | --- | --- | --- | --- | --- | --- | --- | --- | --- | --- | --- | --- | --- | --- | --- | --- | --- | --- | --- | --- | --- | --- | --- | --- | --- | --- | --- | --- | --- | --- | --- | --- | --- | --- | --- | --- | --- | --- | --- | --- | --- | --- | --- | --- | --- | --- | --- | --- | --- | --- | --- | --- | --- | --- | --- | --- | --- | --- | --- | --- | --- | --- | --- | --- | --- | --- | --- | --- | --- | --- | --- | --- | --- | --- | --- | --- | --- | --- | --- | --- | --- | --- | --- | --- | --- | --- | --- | --- | --- | --- | --- | --- | --- | --- | --- | --- | --- | --- | --- | --- | --- | --- | --- | --- | --- | --- | --- | --- | --- | --- | --- | --- | --- | --- | --- | --- | --- | --- | --- | --- | --- | --- | --- | --- | --- | --- | --- | --- | --- | --- | --- | --- | --- | --- | --- | --- | --- | --- | --- | --- | --- | --- | --- | --- | --- | --- | --- | --- | --- | --- | --- | --- | --- | --- | --- | --- | --- | --- | --- | --- | --- | --- | --- | --- | --- | --- | --- | --- | --- | --- | --- | --- | --- | --- | --- | --- | --- | --- | --- | --- | --- | --- | --- | --- | --- | --- | --- | --- | --- | --- | --- | --- | --- | --- | --- | --- | --- | --- | --- | --- | --- | --- | --- | --- | --- | --- | --- | --- | --- | --- | --- | --- | --- | --- | --- | --- | --- | --- | --- | --- | --- | --- | --- | --- | --- | --- | --- | --- | --- | --- | --- | --- | --- | --- | --- | --- | --- | --- | --- | --- | --- | --- | --- | --- | --- | --- | --- | --- | --- | --- | --- | --- | --- | --- | --- | --- | --- | --- | --- | --- | --- | --- | --- | --- | --- | --- | --- | --- | --- | --- | --- | --- | --- | --- | --- | --- | --- | --- | --- | --- | --- | --- | --- | --- | --- | --- | --- | --- | --- | --- | --- | --- | --- | --- | --- | --- | --- | --- | --- | --- | --- | --- | --- | --- | --- | --- | --- | --- | --- | --- | --- | --- | --- | --- | --- | --- | --- | --- | --- | --- | --- | --- | --- | --- | --- | --- | --- | --- | --- | --- | --- | --- | --- | --- | --- | --- | --- | --- | --- | --- | --- | --- | --- | --- | --- | --- | --- | --- | --- | --- | --- | --- | --- | --- | --- | --- | --- | --- | --- | --- | --- | --- | --- | --- | --- | --- | --- | --- | --- | --- | --- | --- | --- | --- | --- | --- | --- | --- | --- | --- | --- | --- | --- | --- | --- | --- | --- | --- | --- | --- | --- | --- | --- | --- | --- | --- | --- | --- | --- | --- | --- | --- | --- | --- | --- | --- | --- | --- | --- | --- | --- | --- | --- | --- | --- | --- | --- | --- | --- | --- | --- | --- | --- | --- | --- | --- | --- | --- | --- | --- | --- | --- | --- | --- | --- | --- | --- | --- | --- | --- | --- | --- | --- | --- | --- | --- | --- | --- | --- | --- | --- | --- | --- | --- | --- | --- | --- | --- | --- | --- | --- | --- | --- | --- | --- | --- | --- | --- | --- | --- | --- | --- | --- | --- | --- | --- | --- | --- | --- | --- | --- | --- | --- | --- | --- | --- | --- | --- | --- | --- | --- | --- | --- | --- | --- | --- | --- | --- | --- | --- | --- | --- | --- | --- | --- | --- | --- | --- | --- | --- | --- | --- | --- | --- | --- | --- | --- | --- | --- | --- | --- | --- | --- | --- | --- | --- | --- | --- | --- | --- | --- | --- | --- | --- | --- | --- | --- | --- | --- | --- | --- | --- | --- | --- | --- | --- | --- | --- | --- | --- | --- | --- | --- | --- | --- | --- | --- | --- | --- | --- | --- | --- | --- | --- | --- | --- | --- | --- | --- | --- | --- | --- | --- | --- | --- | --- | --- | --- | --- | --- | --- | --- | --- | --- | --- | --- | --- | --- | --- | --- | --- | --- | --- | --- | --- | --- | --- | --- | --- | --- | --- | --- | --- | --- | --- | --- | --- | --- | --- | --- | --- | --- | --- | --- | --- | --- | --- | --- | --- | --- | --- | --- | --- | --- | --- | --- | --- | --- | --- | --- | --- | --- | --- | --- | --- | --- | --- | --- | --- | --- | --- | --- | --- | --- | --- | --- | --- | --- | --- | --- | --- | --- | --- | --- | --- | --- | --- | --- | --- | --- | --- | --- | --- | --- | --- | --- | --- | --- | --- | --- | --- | --- | --- | --- | --- | --- | --- | --- | --- | --- | --- | --- | --- | --- | --- | --- | --- | --- | --- | --- | --- | --- | --- | --- | --- | --- | --- | --- | --- | --- | --- | --- | --- | --- | --- | --- | --- | --- | --- | --- | --- | --- | --- | --- | --- | --- | --- | --- | --- | --- | --- | --- | --- | --- | --- | --- | --- | --- | --- | --- | --- | --- | --- | --- | --- | --- | --- | --- | --- | --- | --- | --- | --- | --- | --- | --- | --- | --- | --- | --- | --- | --- | --- | --- | --- | --- | --- | --- | --- | --- | --- | --- | --- | --- | --- | --- | --- | --- | --- | --- | --- | --- | --- | --- | --- | --- | --- | --- | --- | --- | --- | --- | --- | --- | --- | --- | --- | --- | --- | --- | --- | --- | --- | --- | --- | --- | --- | --- | --- | --- | --- | --- | --- | --- | --- | --- | --- | --- | --- | --- | --- | --- | --- | --- | --- | --- | --- | --- | --- | --- | --- | --- | --- | --- | --- | --- | --- | --- | --- | --- | --- | --- | --- | --- | --- | --- | --- | --- | --- | --- | --- | --- | --- | --- | --- | --- | --- | --- | --- | --- | --- | --- | --- | --- | --- | --- | --- | --- | --- | --- | --- | --- | --- | --- | --- | --- | --- | --- | --- | --- | --- | --- | --- | --- | --- | --- | --- | --- | --- | --- | --- | --- | --- | --- | --- | --- | --- | --- | --- | --- | --- | --- | --- | --- | --- | --- | --- | --- | --- | --- | --- | --- | --- | --- | --- | --- | --- | --- | --- | --- | --- | --- | --- | --- | --- | --- | --- | --- | --- | --- | --- | --- | --- | --- | --- | --- | --- | --- | --- | --- | --- | --- | --- | --- | --- | --- | --- | --- | --- | --- | --- | --- | --- | --- | --- | --- | --- | --- | --- | --- | --- | --- | --- | --- | --- | --- | --- | --- | --- | --- | --- | --- | --- | --- | --- | --- | --- | --- | --- | --- | --- | --- | --- | --- | --- | --- | --- | --- | --- | --- | --- | --- | --- | --- | --- | --- | --- | --- | --- | --- | --- | --- | --- | --- | --- | --- | --- | --- | --- | --- | --- | --- | --- | --- | --- | --- | --- | --- | --- | --- | --- | --- | --- | --- | --- | --- | --- | --- | --- | --- | --- | --- | --- | --- | --- | --- | --- | --- | --- | --- | --- | --- | --- | --- | --- | --- | --- | --- | --- | --- | --- | --- | --- | --- | --- | --- | --- | --- | --- | --- | --- | --- | --- | --- | --- | --- | --- | --- | --- | --- | --- | --- | --- | --- | --- | --- | --- | --- | --- | --- | --- | --- | --- | --- | --- | --- | --- | --- | --- | --- | --- | --- | --- | --- | --- | --- | --- | --- | --- | --- | --- | --- | --- | --- | --- | --- | --- | --- | --- | --- | --- | --- | --- | --- | --- | --- | --- | --- | --- | --- | --- | --- | --- | --- | --- | --- | --- | --- | --- | --- | --- | --- | --- | --- | --- | --- | --- | --- | --- | --- | --- | --- | --- | --- | --- | --- | --- | --- | --- | --- | --- | --- | --- | --- | --- | --- | --- | --- | --- | --- | --- | --- | --- | --- | --- | --- | --- | --- | --- | --- | --- | --- | --- | --- | --- | --- | --- | --- | --- | --- | --- | --- | --- | --- | --- | --- | --- | --- | --- | --- | --- | --- | --- | --- | --- | --- | --- | --- | --- | --- | --- | --- | --- | --- | --- | --- | --- | --- | --- | --- | --- | --- | --- | --- | --- | --- | --- | --- | --- | --- | --- | --- | --- | --- | --- | --- | --- | --- | --- | --- | --- | --- | --- | --- | --- | --- | --- | --- | --- | --- | --- | --- | --- | --- | --- | --- | --- | --- | --- | --- | --- | --- | --- | --- | --- | --- | --- | --- | --- | --- | --- | --- | --- | --- | --- | --- | --- | --- | --- | --- | --- | --- | --- | --- | --- | --- | --- | --- | --- | --- | --- | --- | --- | --- | --- | --- | --- | --- | --- | --- | --- | --- | --- | --- | --- | --- | --- | --- | --- | --- | --- | --- | --- | --- | --- | --- | --- | --- | --- | --- | --- | --- | --- | --- | --- | --- | --- | --- | --- | --- | --- | --- | --- | --- | --- | --- | --- | --- | --- | --- | --- | --- | --- | --- | --- | --- | --- | --- | --- | --- | --- | --- | --- | --- | --- | --- | --- | --- | --- | --- | --- | --- | --- | --- | --- | --- | --- | --- | --- | --- | --- | --- | --- | --- | --- | --- | --- | --- | --- | --- | --- | --- | --- | --- | --- | --- | --- | --- | --- | --- | --- | --- | --- | --- | --- | --- | --- | --- | --- | --- | --- | --- | --- | --- | --- | --- | --- | --- | --- | --- | --- | --- | --- | --- | --- | --- | --- | --- | --- | --- | --- | --- | --- | --- | --- | --- | --- | --- | --- | --- | --- | --- | --- | --- | --- | --- | --- | --- | --- | --- | --- | --- | --- | --- | --- | --- | --- | --- | --- | --- | --- | --- | --- | --- | --- | --- | --- | --- | --- | --- | --- | --- | --- | --- | --- | --- | --- | --- | --- | --- | --- | --- | --- | --- | --- | --- | --- | --- | --- | --- | --- | --- | --- | --- | --- | --- | --- | --- | --- | --- | --- | --- | --- | --- | --- | --- | --- | --- | --- | --- | --- | --- | --- | --- | --- | --- | --- | --- | --- | --- | --- | --- | --- | --- | --- | --- | --- | --- | --- | --- | --- | --- | --- | --- | --- | --- | --- | --- | --- | --- | --- | --- | --- | --- | --- | --- | --- | --- | --- | --- | --- | --- | --- | --- | --- | --- | --- | --- | --- | --- | --- | --- | --- | --- | --- | --- | --- | --- | --- | --- | --- | --- | --- | --- | --- | --- | --- | --- | --- | --- | --- | --- | --- | --- | --- | --- | --- | --- | --- | --- | --- | --- | --- | --- | --- | --- | --- | --- | --- | --- | --- | --- | --- | --- | --- | --- | --- | --- | --- | --- | --- | --- | --- | --- | --- | --- | --- | --- | --- | --- | --- | --- | --- | --- | --- | --- | --- | --- | --- | --- | --- | --- | --- | --- | --- | --- | --- | --- | --- | --- | --- | --- | --- | --- | --- | --- | --- | --- | --- | --- | --- | --- | --- | --- | --- | --- | --- | --- | --- | --- | --- | --- | --- | --- | --- | --- | --- | --- | --- | --- | --- | --- | --- | --- | --- | --- | --- | --- | --- | --- | --- | --- | --- | --- | --- | --- | --- | --- | --- | --- | --- | --- | --- | --- | --- | --- | --- | --- | --- | --- | --- | --- | --- | --- | --- | --- | --- | --- | --- | --- | --- | --- | --- | --- | --- | --- | --- | --- | --- | --- | --- | --- | --- | --- | --- | --- | --- | --- | --- | --- | --- | --- | --- | --- | --- | --- | --- | --- | --- | --- | --- | --- | --- | --- | --- | --- | --- | --- | --- | --- | --- | --- | --- | --- | --- | --- | --- | --- | --- | --- | --- | --- | --- | --- | --- | --- | --- | --- | --- | --- | --- | --- | --- | --- | --- | --- | --- | --- | --- | --- | --- | --- | --- | --- | --- | --- | --- | --- | --- | --- | --- | --- | --- | --- | --- | --- | --- | --- | --- | --- | --- | --- | --- | --- | --- | --- | --- | --- | --- | --- | --- | --- | --- | --- | --- | --- | --- | --- | --- | --- | --- | --- | --- | --- | --- | --- | --- | --- | --- | --- | --- | --- | --- | --- |
| Actual | Predict  |  |  |  |  |  |  |  |  |  |  |  |  |  |  |  |  |  |  |  |  |  |  |  |  |  |  |  |  |  |  |  |  |  |  |  |  |  |  |  |  |  |  |  |  |  |  |  |  |  |  |  |  |  | | --- | --- | --- | --- | --- | --- | --- | --- | --- | --- | --- | --- | --- | --- | --- | --- | --- | --- | --- | --- | --- | --- | --- | --- | --- | --- | --- | --- | --- | --- | --- | --- | --- | --- | --- | --- | --- | --- | --- | --- | --- | --- | --- | --- | --- | --- | --- | --- | --- | --- | --- | --- | --- | |  | 0 | 1 | 2 | 3 | 4 | 5 | 6 | 7 | 8 | 9 | 10 | 11 | 12 | 13 | 14 | 15 | 16 | 17 | 18 | 19 | 20 | 21 | 22 | 23 | 24 | 25 | 26 | 27 | 28 | 29 | 30 | 31 | 32 | 33 | 34 | 35 | 36 | 37 | 38 | 39 | 40 | 41 | 42 | 43 | 44 | 45 | 46 | 47 | 48 | 49 | 50 | 51 | | 0 | 25 | 1 | 0 | 1 | 0 | 0 | 0 | 1 | 0 | 0 | 3 | 4 | 0 | 1 | 0 | 2 | 1 | 0 | 0 | 0 | 0 | 0 | 0 | 2 | 0 | 0 | 1 | 0 | 4 | 0 | 1 | 0 | 0 | 0 | 0 | 0 | 0 | 0 | 0 | 0 | 0 | 0 | 0 | 0 | 0 | 1 | 0 | 0 | 0 | 0 | 2 | 0 | | 1 | 0 | 32 | 0 | 0 | 0 | 0 | 1 | 0 | 0 | 0 | 0 | 0 | 0 | 0 | 0 | 0 | 0 | 0 | 0 | 0 | 0 | 0 | 1 | 0 | 0 | 0 | 0 | 0 | 0 | 0 | 0 | 0 | 0 | 0 | 0 | 0 | 0 | 0 | 0 | 0 | 0 | 0 | 0 | 0 | 0 | 0 | 0 | 0 | 0 | 0 | 0 | 0 | | 2 | 0 | 0 | 6 | 0 | 0 | 0 | 0 | 0 | 0 | 0 | 0 | 0 | 0 | 0 | 0 | 0 | 0 | 0 | 0 | 0 | 0 | 0 | 0 | 0 | 0 | 0 | 0 | 0 | 0 | 0 | 0 | 0 | 0 | 0 | 0 | 0 | 0 | 0 | 0 | 0 | 0 | 0 | 0 | 0 | 0 | 0 | 0 | 0 | 0 | 0 | 1 | 0 | | 3 | 0 | 0 | 0 | 5 | 0 | 0 | 0 | 0 | 0 | 0 | 0 | 0 | 0 | 0 | 0 | 0 | 0 | 0 | 0 | 0 | 0 | 0 | 0 | 0 | 0 | 0 | 0 | 0 | 1 | 0 | 1 | 0 | 0 | 0 | 0 | 0 | 0 | 0 | 0 | 0 | 0 | 0 | 0 | 0 | 0 | 0 | 0 | 0 | 0 | 0 | 0 | 0 | | 4 | 0 | 0 | 0 | 0 | 54 | 0 | 0 | 0 | 0 | 0 | 0 | 0 | 0 | 0 | 0 | 0 | 1 | 0 | 0 | 0 | 0 | 0 | 0 | 0 | 0 | 0 | 0 | 0 | 0 | 0 | 0 | 0 | 0 | 0 | 0 | 0 | 0 | 0 | 0 | 0 | 0 | 0 | 0 | 0 | 0 | 0 | 0 | 0 | 0 | 0 | 0 | 0 | | 5 | 1 | 2 | 0 | 0 | 0 | 1 | 0 | 0 | 0 | 0 | 0 | 0 | 0 | 0 | 0 | 0 | 0 | 0 | 0 | 0 | 0 | 0 | 1 | 0 | 1 | 0 | 0 | 0 | 0 | 0 | 0 | 0 | 0 | 0 | 0 | 0 | 0 | 0 | 0 | 0 | 0 | 0 | 0 | 0 | 0 | 0 | 0 | 0 | 0 | 0 | 0 | 0 | | 6 | 1 | 0 | 0 | 0 | 0 | 0 | 5 | 0 | 0 | 0 | 0 | 0 | 0 | 0 | 0 | 0 | 0 | 0 | 0 | 0 | 0 | 0 | 0 | 0 | 0 | 0 | 0 | 0 | 0 | 0 | 0 | 0 | 0 | 0 | 0 | 0 | 0 | 0 | 1 | 0 | 0 | 0 | 0 | 0 | 0 | 1 | 0 | 0 | 0 | 0 | 0 | 0 | | 7 | 0 | 0 | 0 | 0 | 0 | 0 | 0 | 4 | 0 | 0 | 0 | 0 | 1 | 0 | 0 | 0 | 0 | 0 | 0 | 0 | 0 | 0 | 0 | 0 | 0 | 0 | 0 | 0 | 0 | 0 | 0 | 0 | 0 | 0 | 0 | 0 | 0 | 0 | 0 | 0 | 0 | 0 | 0 | 0 | 0 | 0 | 0 | 0 | 0 | 0 | 0 | 0 | | 8 | 0 | 0 | 0 | 0 | 0 | 0 | 0 | 0 | 10 | 0 | 0 | 0 | 0 | 0 | 0 | 0 | 0 | 0 | 0 | 0 | 0 | 0 | 0 | 0 | 0 | 0 | 0 | 0 | 0 | 0 | 0 | 0 | 0 | 0 | 0 | 0 | 0 | 0 | 0 | 0 | 0 | 0 | 0 | 0 | 0 | 0 | 0 | 0 | 0 | 0 | 0 | 0 | | 9 | 0 | 1 | 0 | 0 | 0 | 0 | 0 | 0 | 0 | 5 | 0 | 0 | 0 | 0 | 0 | 0 | 0 | 0 | 0 | 0 | 0 | 0 | 0 | 0 | 0 | 0 | 0 | 0 | 0 | 0 | 0 | 0 | 0 | 0 | 0 | 0 | 0 | 0 | 0 | 0 | 0 | 0 | 0 | 0 | 0 | 0 | 0 | 0 | 0 | 0 | 0 | 0 | | 10 | 0 | 0 | 0 | 0 | 0 | 0 | 0 | 0 | 0 | 0 | 33 | 0 | 0 | 0 | 0 | 0 | 0 | 0 | 0 | 0 | 0 | 0 | 0 | 0 | 0 | 0 | 0 | 0 | 0 | 0 | 0 | 0 | 0 | 0 | 0 | 0 | 0 | 0 | 0 | 0 | 0 | 0 | 0 | 0 | 0 | 0 | 0 | 0 | 0 | 0 | 3 | 0 | | 11 | 10 | 1 | 0 | 0 | 0 | 0 | 1 | 0 | 0 | 0 | 0 | 9 | 6 | 0 | 0 | 1 | 0 | 0 | 0 | 1 | 5 | 0 | 0 | 2 | 1 | 0 | 2 | 0 | 2 | 2 | 0 | 0 | 0 | 0 | 0 | 0 | 0 | 0 | 1 | 0 | 0 | 0 | 1 | 1 | 0 | 1 | 0 | 0 | 0 | 0 | 0 | 0 | | 12 | 2 | 1 | 0 | 0 | 0 | 0 | 0 | 0 | 0 | 0 | 0 | 0 | 20 | 0 | 0 | 0 | 0 | 0 | 0 | 0 | 0 | 0 | 1 | 0 | 0 | 0 | 0 | 0 | 1 | 0 | 0 | 0 | 0 | 0 | 0 | 0 | 0 | 0 | 0 | 0 | 0 | 0 | 0 | 0 | 0 | 0 | 0 | 0 | 0 | 1 | 0 | 2 | | 13 | 2 | 0 | 0 | 0 | 0 | 0 | 0 | 0 | 0 | 0 | 0 | 1 | 0 | 21 | 0 | 0 | 0 | 0 | 0 | 0 | 0 | 0 | 0 | 0 | 0 | 0 | 0 | 0 | 0 | 0 | 0 | 0 | 0 | 0 | 0 | 0 | 0 | 0 | 0 | 0 | 0 | 0 | 0 | 0 | 0 | 0 | 0 | 0 | 0 | 0 | 0 | 1 | | 14 | 0 | 0 | 0 | 0 | 0 | 0 | 1 | 0 | 0 | 0 | 0 | 0 | 0 | 0 | 25 | 0 | 0 | 0 | 0 | 0 | 0 | 0 | 1 | 0 | 0 | 0 | 0 | 0 | 0 | 0 | 0 | 0 | 0 | 0 | 0 | 0 | 0 | 0 | 0 | 0 | 0 | 0 | 0 | 0 | 0 | 0 | 0 | 0 | 0 | 0 | 0 | 0 | | 15 | 1 | 0 | 0 | 0 | 0 | 0 | 0 | 0 | 0 | 0 | 0 | 3 | 0 | 0 | 0 | 21 | 0 | 0 | 0 | 0 | 0 | 0 | 0 | 0 | 0 | 0 | 0 | 0 | 0 | 0 | 0 | 0 | 0 | 0 | 0 | 0 | 0 | 0 | 0 | 0 | 0 | 0 | 0 | 0 | 0 | 1 | 0 | 0 | 0 | 0 | 0 | 0 | | 16 | 1 | 0 | 0 | 0 | 0 | 0 | 0 | 0 | 0 | 0 | 0 | 0 | 0 | 1 | 0 | 0 | 24 | 0 | 0 | 0 | 0 | 0 | 0 | 0 | 0 | 0 | 0 | 0 | 1 | 0 | 0 | 0 | 0 | 0 | 0 | 0 | 0 | 0 | 0 | 0 | 0 | 0 | 0 | 0 | 0 | 0 | 0 | 0 | 0 | 0 | 0 | 0 | | 17 | 1 | 0 | 0 | 0 | 0 | 0 | 0 | 0 | 0 | 0 | 0 | 2 | 0 | 0 | 0 | 0 | 0 | 3 | 0 | 0 | 0 | 0 | 0 | 1 | 0 | 0 | 0 | 0 | 0 | 0 | 0 | 0 | 0 | 0 | 0 | 0 | 0 | 0 | 0 | 0 | 0 | 0 | 0 | 0 | 0 | 0 | 0 | 0 | 0 | 0 | 0 | 0 | | 18 | 0 | 1 | 0 | 0 | 0 | 0 | 0 | 0 | 0 | 0 | 0 | 0 | 0 | 0 | 0 | 0 | 0 | 0 | 12 | 0 | 0 | 0 | 0 | 0 | 0 | 0 | 0 | 0 | 0 | 0 | 0 | 0 | 0 | 0 | 0 | 0 | 0 | 0 | 0 | 0 | 0 | 0 | 0 | 0 | 0 | 0 | 0 | 0 | 0 | 0 | 0 | 0 | | 19 | 0 | 0 | 0 | 0 | 0 | 0 | 0 | 0 | 0 | 0 | 0 | 0 | 0 | 0 | 0 | 0 | 0 | 0 | 0 | 62 | 0 | 0 | 0 | 0 | 0 | 0 | 0 | 0 | 0 | 0 | 0 | 0 | 0 | 0 | 0 | 0 | 0 | 0 | 0 | 0 | 0 | 0 | 0 | 0 | 0 | 0 | 0 | 0 | 0 | 0 | 0 | 0 | | 20 | 0 | 0 | 0 | 0 | 0 | 0 | 0 | 0 | 0 | 0 | 0 | 0 | 1 | 1 | 0 | 1 | 8 | 0 | 0 | 4 | 121 | 0 | 2 | 0 | 1 | 0 | 0 | 0 | 1 | 0 | 0 | 0 | 0 | 0 | 0 | 0 | 0 | 0 | 0 | 0 | 2 | 0 | 0 | 1 | 0 | 0 | 0 | 0 | 0 | 0 | 0 | 0 | | 21 | 0 | 0 | 0 | 0 | 0 | 0 | 0 | 0 | 0 | 0 | 0 | 0 | 0 | 0 | 0 | 0 | 0 | 0 | 0 | 0 | 0 | 14 | 1 | 0 | 0 | 0 | 0 | 0 | 0 | 0 | 0 | 0 | 0 | 0 | 0 | 0 | 0 | 0 | 0 | 0 | 0 | 0 | 0 | 0 | 0 | 0 | 0 | 0 | 0 | 0 | 0 | 0 | | 22 | 8 | 0 | 0 | 0 | 0 | 0 | 0 | 0 | 0 | 0 | 0 | 0 | 0 | 0 | 0 | 1 | 0 | 0 | 1 | 0 | 0 | 0 | 17 | 5 | 0 | 0 | 0 | 0 | 3 | 0 | 0 | 0 | 0 | 0 | 0 | 0 | 0 | 0 | 0 | 0 | 0 | 1 | 0 | 2 | 0 | 1 | 8 | 0 | 0 | 0 | 3 | 1 | | 23 | 0 | 0 | 0 | 0 | 0 | 0 | 1 | 0 | 0 | 0 | 0 | 0 | 0 | 0 | 0 | 0 | 0 | 0 | 0 | 0 | 0 | 0 | 0 | 11 | 0 | 0 | 1 | 0 | 15 | 0 | 4 | 0 | 0 | 0 | 0 | 1 | 0 | 0 | 0 | 0 | 1 | 1 | 0 | 0 | 0 | 0 | 1 | 0 | 0 | 0 | 3 | 0 | | 24 | 1 | 0 | 0 | 0 | 0 | 0 | 0 | 0 | 0 | 0 | 0 | 0 | 0 | 0 | 0 | 0 | 0 | 0 | 0 | 0 | 0 | 0 | 0 | 0 | 21 | 0 | 0 | 0 | 0 | 0 | 0 | 0 | 0 | 0 | 0 | 0 | 0 | 0 | 0 | 0 | 0 | 0 | 0 | 0 | 0 | 0 | 0 | 0 | 0 | 0 | 0 | 1 | | 25 | 0 | 0 | 0 | 0 | 0 | 0 | 0 | 0 | 0 | 0 | 0 | 0 | 0 | 0 | 0 | 0 | 0 | 0 | 0 | 0 | 0 | 0 | 0 | 0 | 0 | 11 | 0 | 0 | 0 | 0 | 0 | 0 | 0 | 0 | 0 | 0 | 0 | 0 | 0 | 0 | 0 | 0 | 0 | 0 | 0 | 0 | 0 | 0 | 0 | 0 | 0 | 0 | | 26 | 2 | 0 | 0 | 0 | 0 | 0 | 2 | 0 | 0 | 0 | 2 | 0 | 1 | 0 | 0 | 0 | 2 | 0 | 0 | 0 | 0 | 0 | 1 | 1 | 0 | 0 | 15 | 0 | 0 | 0 | 0 | 0 | 0 | 0 | 0 | 0 | 0 | 0 | 0 | 0 | 0 | 0 | 0 | 1 | 1 | 0 | 1 | 0 | 0 | 0 | 0 | 0 | | 27 | 0 | 1 | 0 | 0 | 0 | 0 | 0 | 0 | 0 | 0 | 0 | 0 | 0 | 0 | 0 | 0 | 0 | 0 | 0 | 0 | 0 | 0 | 0 | 0 | 0 | 0 | 1 | 12 | 0 | 0 | 0 | 0 | 0 | 0 | 0 | 0 | 0 | 0 | 0 | 0 | 0 | 0 | 0 | 0 | 0 | 0 | 0 | 0 | 0 | 0 | 0 | 0 | | 28 | 0 | 0 | 0 | 2 | 1 | 0 | 0 | 0 | 0 | 0 | 0 | 1 | 0 | 0 | 1 | 0 | 0 | 0 | 0 | 0 | 1 | 0 | 0 | 6 | 1 | 0 | 0 | 0 | 80 | 0 | 1 | 0 | 0 | 1 | 0 | 0 | 0 | 0 | 0 | 0 | 0 | 0 | 0 | 0 | 0 | 0 | 0 | 0 | 0 | 0 | 4 | 1 | | 29 | 1 | 0 | 0 | 0 | 0 | 0 | 0 | 0 | 0 | 0 | 0 | 0 | 0 | 0 | 0 | 0 | 0 | 0 | 0 | 0 | 0 | 0 | 0 | 0 | 1 | 0 | 0 | 0 | 0 | 4 | 0 | 0 | 0 | 1 | 0 | 0 | 0 | 0 | 0 | 0 | 0 | 0 | 0 | 0 | 0 | 0 | 0 | 0 | 0 | 0 | 0 | 0 | | 30 | 0 | 0 | 0 | 0 | 0 | 0 | 0 | 0 | 0 | 0 | 0 | 1 | 0 | 0 | 0 | 0 | 0 | 0 | 0 | 0 | 0 | 0 | 0 | 0 | 0 | 0 | 0 | 0 | 0 | 0 | 6 | 0 | 0 | 0 | 0 | 0 | 0 | 0 | 0 | 0 | 0 | 0 | 0 | 0 | 0 | 0 | 0 | 0 | 0 | 0 | 0 | 0 | | 31 | 0 | 0 | 0 | 0 | 0 | 0 | 0 | 0 | 0 | 0 | 0 | 0 | 0 | 0 | 0 | 0 | 0 | 0 | 0 | 0 | 0 | 0 | 0 | 0 | 0 | 0 | 0 | 0 | 0 | 0 | 0 | 10 | 0 | 0 | 0 | 0 | 0 | 0 | 0 | 0 | 0 | 0 | 0 | 0 | 0 | 0 | 0 | 0 | 0 | 0 | 0 | 0 | | 32 | 0 | 0 | 0 | 0 | 0 | 0 | 0 | 0 | 0 | 0 | 0 | 0 | 0 | 0 | 0 | 0 | 0 | 0 | 0 | 0 | 0 | 0 | 0 | 4 | 0 | 0 | 0 | 0 | 0 | 0 | 0 | 0 | 1 | 0 | 0 | 0 | 0 | 0 | 0 | 0 | 0 | 0 | 0 | 0 | 0 | 0 | 0 | 0 | 0 | 0 | 0 | 0 | | 33 | 0 | 0 | 0 | 0 | 0 | 0 | 0 | 0 | 0 | 0 | 0 | 0 | 0 | 0 | 0 | 0 | 0 | 0 | 0 | 0 | 0 | 0 | 0 | 0 | 0 | 0 | 0 | 0 | 0 | 0 | 0 | 0 | 0 | 5 | 0 | 0 | 0 | 0 | 0 | 0 | 0 | 0 | 0 | 0 | 0 | 0 | 0 | 0 | 0 | 0 | 0 | 0 | | 34 | 0 | 0 | 0 | 0 | 0 | 0 | 0 | 0 | 0 | 0 | 0 | 0 | 0 | 0 | 0 | 0 | 0 | 0 | 0 | 0 | 0 | 0 | 0 | 0 | 0 | 0 | 0 | 0 | 0 | 0 | 0 | 0 | 0 | 0 | 23 | 0 | 0 | 0 | 0 | 0 | 0 | 0 | 0 | 0 | 0 | 0 | 0 | 0 | 0 | 0 | 0 | 0 | | 35 | 0 | 0 | 0 | 0 | 0 | 0 | 0 | 0 | 0 | 0 | 0 | 0 | 0 | 0 | 0 | 0 | 0 | 0 | 0 | 0 | 0 | 0 | 0 | 0 | 0 | 0 | 0 | 0 | 0 | 0 | 0 | 0 | 0 | 0 | 0 | 70 | 0 | 0 | 0 | 0 | 0 | 0 | 0 | 0 | 0 | 0 | 0 | 0 | 0 | 0 | 0 | 0 | | 36 | 0 | 0 | 0 | 0 | 0 | 0 | 0 | 0 | 0 | 0 | 0 | 0 | 0 | 0 | 0 | 0 | 0 | 0 | 0 | 0 | 0 | 0 | 0 | 0 | 0 | 0 | 0 | 0 | 0 | 0 | 0 | 0 | 0 | 0 | 0 | 0 | 6 | 0 | 0 | 0 | 0 | 0 | 0 | 0 | 0 | 0 | 0 | 0 | 0 | 0 | 0 | 0 | | 37 | 1 | 0 | 0 | 0 | 0 | 0 | 0 | 0 | 0 | 0 | 0 | 0 | 0 | 0 | 0 | 0 | 0 | 0 | 0 | 0 | 0 | 0 | 0 | 0 | 0 | 0 | 0 | 0 | 0 | 0 | 0 | 0 | 0 | 0 | 0 | 0 | 0 | 7 | 0 | 0 | 0 | 0 | 0 | 0 | 0 | 0 | 0 | 0 | 0 | 0 | 0 | 2 | | 38 | 0 | 0 | 0 | 0 | 0 | 0 | 0 | 0 | 0 | 0 | 0 | 0 | 0 | 0 | 0 | 0 | 0 | 0 | 0 | 0 | 0 | 0 | 0 | 2 | 0 | 0 | 0 | 0 | 2 | 0 | 0 | 0 | 0 | 0 | 0 | 0 | 0 | 0 | 12 | 0 | 0 | 0 | 0 | 0 | 0 | 0 | 0 | 0 | 0 | 0 | 0 | 0 | | 39 | 0 | 0 | 0 | 0 | 0 | 0 | 0 | 0 | 0 | 0 | 0 | 0 | 0 | 0 | 0 | 0 | 0 | 0 | 0 | 0 | 0 | 0 | 1 | 0 | 0 | 0 | 0 | 0 | 1 | 0 | 0 | 0 | 0 | 0 | 0 | 0 | 0 | 0 | 0 | 4 | 0 | 0 | 0 | 0 | 0 | 0 | 0 | 0 | 0 | 0 | 0 | 0 | | 40 | 0 | 0 | 0 | 0 | 0 | 0 | 0 | 0 | 0 | 0 | 0 | 0 | 0 | 0 | 0 | 0 | 0 | 0 | 0 | 0 | 0 | 0 | 0 | 1 | 0 | 0 | 1 | 0 | 0 | 0 | 0 | 0 | 0 | 0 | 0 | 0 | 0 | 0 | 0 | 0 | 15 | 0 | 0 | 0 | 0 | 2 | 0 | 0 | 0 | 0 | 1 | 0 | | 41 | 4 | 0 | 0 | 0 | 0 | 0 | 0 | 0 | 0 | 0 | 0 | 0 | 0 | 0 | 0 | 0 | 0 | 0 | 0 | 0 | 0 | 0 | 0 | 0 | 0 | 0 | 0 | 0 | 0 | 0 | 0 | 0 | 0 | 0 | 0 | 0 | 0 | 0 | 0 | 0 | 2 | 19 | 0 | 0 | 0 | 0 | 0 | 0 | 0 | 0 | 0 | 0 | | 42 | 0 | 0 | 0 | 0 | 0 | 0 | 0 | 0 | 0 | 0 | 0 | 0 | 0 | 0 | 0 | 0 | 0 | 0 | 0 | 0 | 0 | 0 | 0 | 0 | 0 | 0 | 0 | 0 | 0 | 0 | 0 | 0 | 0 | 0 | 0 | 0 | 0 | 0 | 0 | 0 | 0 | 0 | 15 | 0 | 0 | 0 | 0 | 0 | 0 | 0 | 0 | 0 | | 43 | 0 | 0 | 0 | 0 | 0 | 0 | 0 | 0 | 0 | 0 | 0 | 1 | 0 | 0 | 0 | 0 | 0 | 0 | 0 | 0 | 0 | 0 | 0 | 0 | 0 | 0 | 0 | 0 | 0 | 0 | 0 | 0 | 0 | 0 | 0 | 0 | 0 | 0 | 0 | 0 | 0 | 0 | 0 | 4 | 0 | 0 | 1 | 1 | 0 | 0 | 0 | 0 | | 44 | 0 | 0 | 0 | 0 | 0 | 0 | 0 | 0 | 0 | 0 | 0 | 0 | 0 | 0 | 0 | 0 | 0 | 0 | 0 | 0 | 0 | 0 | 0 | 0 | 0 | 0 | 0 | 0 | 0 | 0 | 0 | 0 | 0 | 0 | 0 | 0 | 0 | 0 | 0 | 0 | 0 | 0 | 0 | 0 | 8 | 0 | 0 | 0 | 0 | 0 | 0 | 0 | | 45 | 0 | 0 | 0 | 0 | 0 | 0 | 0 | 0 | 0 | 0 | 0 | 0 | 0 | 0 | 0 | 0 | 0 | 0 | 0 | 0 | 0 | 0 | 0 | 0 | 0 | 0 | 0 | 0 | 0 | 0 | 0 | 0 | 0 | 0 | 0 | 0 | 0 | 0 | 0 | 0 | 0 | 0 | 0 | 0 | 0 | 48 | 0 | 3 | 0 | 0 | 0 | 0 | | 46 | 0 | 0 | 0 | 0 | 0 | 0 | 0 | 0 | 0 | 0 | 0 | 0 | 0 | 0 | 0 | 0 | 0 | 0 | 0 | 0 | 0 | 0 | 0 | 0 | 0 | 0 | 0 | 0 | 0 | 1 | 0 | 0 | 0 | 0 | 0 | 0 | 0 | 0 | 1 | 0 | 0 | 0 | 0 | 0 | 0 | 0 | 14 | 0 | 0 | 0 | 0 | 0 | | 47 | 0 | 0 | 0 | 0 | 0 | 0 | 0 | 0 | 0 | 0 | 0 | 0 | 0 | 0 | 0 | 0 | 0 | 0 | 0 | 0 | 0 | 0 | 0 | 0 | 0 | 0 | 0 | 0 | 0 | 0 | 0 | 0 | 0 | 0 | 0 | 0 | 0 | 0 | 0 | 0 | 0 | 0 | 0 | 0 | 0 | 0 | 0 | 14 | 0 | 0 | 0 | 0 | | 48 | 0 | 0 | 0 | 0 | 0 | 0 | 0 | 0 | 0 | 0 | 0 | 0 | 0 | 0 | 0 | 0 | 0 | 0 | 0 | 0 | 0 | 0 | 0 | 0 | 0 | 0 | 0 | 0 | 0 | 0 | 0 | 0 | 0 | 0 | 0 | 0 | 0 | 0 | 0 | 0 | 0 | 0 | 0 | 0 | 0 | 0 | 0 | 0 | 5 | 0 | 0 | 0 | | 49 | 0 | 0 | 0 | 0 | 0 | 0 | 0 | 0 | 0 | 0 | 0 | 0 | 0 | 0 | 0 | 0 | 0 | 0 | 0 | 0 | 0 | 0 | 0 | 0 | 1 | 0 | 0 | 0 | 0 | 0 | 0 | 0 | 0 | 0 | 0 | 0 | 0 | 0 | 0 | 0 | 0 | 0 | 0 | 0 | 0 | 0 | 0 | 0 | 0 | 7 | 0 | 0 | | 50 | 6 | 0 | 0 | 0 | 0 | 0 | 0 | 0 | 0 | 0 | 0 | 3 | 1 | 3 | 0 | 0 | 1 | 0 | 0 | 0 | 1 | 0 | 3 | 4 | 0 | 0 | 0 | 0 | 6 | 0 | 0 | 0 | 0 | 1 | 0 | 1 | 0 | 0 | 0 | 0 | 0 | 1 | 1 | 0 | 0 | 0 | 4 | 0 | 0 | 0 | 5 | 0 | | 51 | 0 | 0 | 0 | 0 | 0 | 0 | 0 | 0 | 0 | 0 | 0 | 0 | 0 | 0 | 0 | 0 | 0 | 0 | 0 | 0 | 0 | 0 | 0 | 0 | 0 | 0 | 0 | 0 | 0 | 0 | 0 | 0 | 0 | 0 | 0 | 0 | 0 | 0 | 0 | 0 | 0 | 0 | 0 | 0 | 0 | 0 | 0 | 0 | 0 | 0 | 0 | 11 | |

## Overall Statistics :

|  |  |
| --- | --- |
| 95% CI | (0.74176,0.78792) |
| ACC Macro | 0.99096 |
| ARI | 0.66163 |
| AUNP | 0.87823 |
| AUNU | 0.88399 |
| Bangdiwala B | 0.67821 |
| Bennett S | 0.76023 |
| CBA | 0.70515 |
| CSI | 0.56183 |
| Chi-Squared | 42730.63711 |
| Chi-Squared DF | 2601 |
| Conditional Entropy | 1.11916 |
| Cramer V | 0.80374 |
| Cross Entropy | 5.18212 |
| F1 Macro | 0.75847 |
| F1 Micro | 0.76484 |
| FNR Macro | 0.22734 |
| FNR Micro | 0.23516 |
| FPR Macro | 0.00468 |
| FPR Micro | 0.00461 |
| Gwet AC1 | 0.76033 |
| Hamming Loss | 0.23516 |
| Joint Entropy | 6.24315 |
| KL Divergence | 0.05812 |
| Kappa | 0.75526 |
| Kappa 95% CI | (0.73124,0.77928) |
| Kappa No Prevalence | 0.52968 |
| Kappa Standard Error | 0.01226 |
| Kappa Unbiased | 0.75515 |
| Krippendorff Alpha | 0.75525 |
| Lambda A | 0.7357 |
| Lambda B | 0.74765 |
| Mutual Information | 3.98268 |
| NIR | 0.11025 |
| Overall ACC | 0.76484 |
| Overall CEN | 0.16698 |
| Overall J | (34.18663,0.65744) |
| Overall MCC | 0.75591 |
| Overall MCEN | 0.2428 |
| Overall RACC | 0.03916 |
| Overall RACCU | 0.03958 |
| P-Value | None |
| PPV Macro | 0.78917 |
| PPV Micro | 0.76484 |
| Pearson C | 0.98516 |
| Phi-Squared | 32.94575 |
| RCI | 0.77726 |
| RR | 24.94231 |
| Reference Entropy | 5.12399 |
| Response Entropy | 5.10184 |
| SOA1(Landis & Koch) | Substantial |
| SOA2(Fleiss) | Excellent |
| SOA3(Altman) | Good |
| SOA4(Cicchetti) | Excellent |
| SOA5(Cramer) | Very Strong |
| SOA6(Matthews) | Strong |
| Scott PI | 0.75515 |
| Standard Error | 0.01178 |
| TNR Macro | 0.99532 |
| TNR Micro | 0.99539 |
| TPR Macro | 0.77266 |
| TPR Micro | 0.76484 |
| Zero-one Loss | 305 |

## Class Statistics :

|  |  |  |  |  |  |  |  |  |  |  |  |  |  |  |  |  |  |  |  |  |  |  |  |  |  |  |  |  |  |  |  |  |  |  |  |  |  |  |  |  |  |  |  |  |  |  |  |  |  |  |  |  |  |
| --- | --- | --- | --- | --- | --- | --- | --- | --- | --- | --- | --- | --- | --- | --- | --- | --- | --- | --- | --- | --- | --- | --- | --- | --- | --- | --- | --- | --- | --- | --- | --- | --- | --- | --- | --- | --- | --- | --- | --- | --- | --- | --- | --- | --- | --- | --- | --- | --- | --- | --- | --- | --- | --- |
| Class | 0 | 1 | 2 | 3 | 4 | 5 | 6 | 7 | 8 | 9 | 10 | 11 | 12 | 13 | 14 | 15 | 16 | 17 | 18 | 19 | 20 | 21 | 22 | 23 | 24 | 25 | 26 | 27 | 28 | 29 | 30 | 31 | 32 | 33 | 34 | 35 | 36 | 37 | 38 | 39 | 40 | 41 | 42 | 43 | 44 | 45 | 46 | 47 | 48 | 49 | 50 | 51 | Description |
| ACC | 0.94834 | 0.99229 | 0.99923 | 0.99614 | 0.99846 | 0.99614 | 0.99306 | 0.99846 | 1.0 | 0.99923 | 0.99383 | 0.95837 | 0.98612 | 0.99229 | 0.99769 | 0.99229 | 0.98766 | 0.99692 | 0.99846 | 0.99614 | 0.97764 | 0.99923 | 0.96453 | 0.95682 | 0.99383 | 1.0 | 0.98458 | 0.99846 | 0.95605 | 0.99537 | 0.99383 | 1.0 | 0.99692 | 0.99769 | 1.0 | 0.99846 | 1.0 | 0.99769 | 0.9946 | 0.99846 | 0.99229 | 0.99306 | 0.99846 | 0.99383 | 0.99923 | 0.99229 | 0.98689 | 0.99692 | 1.0 | 0.99846 | 0.95914 | 0.99383 | Accuracy |
| AGF | 0.6763 | 0.95225 | 0.93905 | 0.83262 | 0.99047 | 0.44652 | 0.76143 | 0.89408 | 1.0 | 0.92819 | 0.95087 | 0.45351 | 0.8364 | 0.90769 | 0.96515 | 0.89695 | 0.90791 | 0.69475 | 0.96039 | 0.99163 | 0.92206 | 0.97229 | 0.59688 | 0.52514 | 0.9383 | 1.0 | 0.73629 | 0.93875 | 0.87086 | 0.75505 | 0.85467 | 1.0 | 0.48735 | 0.94469 | 1.0 | 0.99699 | 1.0 | 0.86215 | 0.8702 | 0.84463 | 0.86433 | 0.88056 | 0.98677 | 0.73424 | 0.98765 | 0.96115 | 0.866 | 0.97229 | 1.0 | 0.93505 | 0.36194 | 0.93377 | Adjusted F-score |
| AGM | 0.82804 | 0.98019 | 0.96281 | 0.92072 | 0.99474 | 0.70344 | 0.89172 | 0.94655 | 1.0 | 0.95633 | 0.9755 | 0.7059 | 0.91615 | 0.95442 | 0.98035 | 0.94601 | 0.9636 | 0.82686 | 0.97971 | 0.99699 | 0.95326 | 0.98295 | 0.7783 | 0.74799 | 0.9741 | 1.0 | 0.85481 | 0.96271 | 0.92302 | 0.87603 | 0.95884 | 1.0 | 0.72307 | 0.99826 | 1.0 | 0.99879 | 1.0 | 0.91801 | 0.93092 | 0.90804 | 0.92969 | 0.93358 | 0.99883 | 0.87497 | 0.99942 | 0.98063 | 0.95892 | 0.99767 | 1.0 | 0.96704 | 0.66152 | 0.99534 | Adjusted geometric mean |
| AM | 17 | 6 | -1 | 1 | 0 | -5 | 3 | 0 | 0 | -1 | 2 | -22 | 2 | 2 | -1 | 0 | 10 | -4 | 0 | 5 | -15 | -1 | -22 | 0 | 4 | 0 | -8 | -2 | 17 | 0 | 6 | 0 | -4 | 3 | 0 | 2 | 0 | -3 | -1 | -2 | 0 | -3 | 2 | 2 | 1 | 4 | 13 | 4 | 0 | 0 | -19 | 8 | Difference between automatic and manual classification |
| AUC | 0.73316 | 0.96742 | 0.92857 | 0.85598 | 0.99051 | 0.58333 | 0.81017 | 0.89961 | 1.0 | 0.91667 | 0.95635 | 0.58934 | 0.8532 | 0.91764 | 0.96257 | 0.90188 | 0.93933 | 0.71429 | 0.96115 | 0.99798 | 0.92004 | 0.96667 | 0.66185 | 0.6299 | 0.95417 | 1.0 | 0.75625 | 0.92857 | 0.88454 | 0.78455 | 0.92586 | 1.0 | 0.6 | 0.99884 | 1.0 | 0.99919 | 1.0 | 0.85 | 0.87383 | 0.83333 | 0.87304 | 0.87882 | 0.99922 | 0.78378 | 0.99961 | 0.96778 | 0.93165 | 0.99844 | 1.0 | 0.93711 | 0.55421 | 0.99689 | Area under the ROC curve |
| AUCI | Good | Excellent | Excellent | Very Good | Excellent | Poor | Very Good | Very Good | Excellent | Excellent | Excellent | Poor | Very Good | Excellent | Excellent | Excellent | Excellent | Good | Excellent | Excellent | Excellent | Excellent | Fair | Fair | Excellent | Excellent | Good | Excellent | Very Good | Good | Excellent | Excellent | Fair | Excellent | Excellent | Excellent | Excellent | Very Good | Very Good | Very Good | Very Good | Very Good | Excellent | Good | Excellent | Excellent | Excellent | Excellent | Excellent | Excellent | Poor | Excellent | AUC value interpretation |
| AUPR | 0.43657 | 0.87059 | 0.92857 | 0.66964 | 0.98182 | 0.58333 | 0.53977 | 0.8 | 1.0 | 0.91667 | 0.89254 | 0.27574 | 0.69048 | 0.80889 | 0.94373 | 0.80769 | 0.76877 | 0.71429 | 0.92308 | 0.96269 | 0.89573 | 0.96667 | 0.45977 | 0.28205 | 0.84541 | 1.0 | 0.61576 | 0.92857 | 0.74188 | 0.57143 | 0.65934 | 1.0 | 0.6 | 0.8125 | 1.0 | 0.98611 | 1.0 | 0.85 | 0.775 | 0.83333 | 0.75 | 0.81182 | 0.94118 | 0.50794 | 0.94444 | 0.90695 | 0.67888 | 0.88889 | 1.0 | 0.875 | 0.17461 | 0.78947 | Area under the PR curve |
| BCD | 0.00655 | 0.00231 | 0.00039 | 0.00039 | 0.0 | 0.00193 | 0.00116 | 0.0 | 0.0 | 0.00039 | 0.00077 | 0.00848 | 0.00077 | 0.00077 | 0.00039 | 0.0 | 0.00386 | 0.00154 | 0.0 | 0.00193 | 0.00578 | 0.00039 | 0.00848 | 0.0 | 0.00154 | 0.0 | 0.00308 | 0.00077 | 0.00655 | 0.0 | 0.00231 | 0.0 | 0.00154 | 0.00116 | 0.0 | 0.00077 | 0.0 | 0.00116 | 0.00039 | 0.00077 | 0.0 | 0.00116 | 0.00077 | 0.00077 | 0.00039 | 0.00154 | 0.00501 | 0.00154 | 0.0 | 0.0 | 0.00732 | 0.00308 | Bray-Curtis dissimilarity |
| BM | 0.46632 | 0.93484 | 0.85714 | 0.71196 | 0.98101 | 0.16667 | 0.62035 | 0.79923 | 1.0 | 0.83333 | 0.9127 | 0.17869 | 0.70641 | 0.83528 | 0.92514 | 0.80376 | 0.87865 | 0.42857 | 0.9223 | 0.99595 | 0.84009 | 0.93333 | 0.3237 | 0.25979 | 0.90833 | 1.0 | 0.51251 | 0.85714 | 0.76909 | 0.5691 | 0.85172 | 1.0 | 0.2 | 0.99768 | 1.0 | 0.99837 | 1.0 | 0.7 | 0.74766 | 0.66667 | 0.74608 | 0.75764 | 0.99844 | 0.56755 | 0.99922 | 0.93556 | 0.86329 | 0.99688 | 1.0 | 0.87422 | 0.10842 | 0.99378 | Informedness or bookmaker informedness |
| CEN | 0.44427 | 0.12171 | 0.04266 | 0.17519 | 0.01848 | 0.25771 | 0.28579 | 0.09957 | 0 | 0.04713 | 0.0773 | 0.51066 | 0.22205 | 0.14483 | 0.04859 | 0.14483 | 0.16392 | 0.16917 | 0.05419 | 0.03143 | 0.10329 | 0.02511 | 0.39891 | 0.44482 | 0.13534 | 0 | 0.30837 | 0.05419 | 0.22517 | 0.22314 | 0.19914 | 0 | 0.05845 | 0.12798 | 0 | 0.01509 | 0 | 0.09047 | 0.14832 | 0.09957 | 0.17692 | 0.12752 | 0.04683 | 0.28101 | 0.03603 | 0.08557 | 0.20436 | 0.0714 | 0 | 0.07494 | 0.55011 | 0.17612 | Confusion entropy |
| DOR | 28.69048 | 2510.0 | None | 1072.5 | 67014.0 | None | 356.38889 | 5164.0 | None | None | 2763.2 | 18.26645 | 314.75 | 1107.75 | 15862.5 | 1063.44 | 773.53846 | None | 15396.0 | None | 901.21429 | None | 51.41667 | 17.25765 | 2219.0 | None | 225.35714 | None | 125.40541 | 572.0 | 1099.71429 | None | None | None | None | None | None | None | 1278.0 | None | 763.2 | 1339.5 | None | 342.66667 | None | 2832.0 | 590.8 | None | None | 9016.0 | 10.12255 | None | Diagnostic odds ratio |
| DP | 0.80369 | 1.87434 | None | 1.67075 | 2.6608 | None | 1.40695 | 2.04708 | None | None | 1.89735 | 0.69559 | 1.3772 | 1.67849 | 2.31579 | 1.66871 | 1.5925 | None | 2.30864 | None | 1.62908 | None | 0.94338 | 0.68198 | 1.84483 | None | 1.29721 | None | 1.15686 | 1.52023 | 1.67675 | None | None | None | None | None | None | None | 1.71272 | None | 1.58928 | 1.72397 | None | 1.39755 | None | 1.90324 | 1.52798 | None | None | 2.18051 | 0.55425 | None | Discriminant power |
| DPI | Poor | Limited | None | Limited | Fair | None | Limited | Fair | None | None | Limited | Poor | Limited | Limited | Fair | Limited | Limited | None | Fair | None | Limited | None | Poor | Poor | Limited | None | Limited | None | Limited | Limited | Limited | None | None | None | None | None | None | None | Limited | None | Limited | Limited | None | Limited | None | Limited | Limited | None | None | Fair | Poor | None | Discriminant power interpretation |
| ERR | 0.05166 | 0.00771 | 0.00077 | 0.00386 | 0.00154 | 0.00386 | 0.00694 | 0.00154 | 0.0 | 0.00077 | 0.00617 | 0.04163 | 0.01388 | 0.00771 | 0.00231 | 0.00771 | 0.01234 | 0.00308 | 0.00154 | 0.00386 | 0.02236 | 0.00077 | 0.03547 | 0.04318 | 0.00617 | 0.0 | 0.01542 | 0.00154 | 0.04395 | 0.00463 | 0.00617 | 0.0 | 0.00308 | 0.00231 | 0.0 | 0.00154 | 0.0 | 0.00231 | 0.0054 | 0.00154 | 0.00771 | 0.00694 | 0.00154 | 0.00617 | 0.00077 | 0.00771 | 0.01311 | 0.00308 | 0.0 | 0.00154 | 0.04086 | 0.00617 | Error rate |
| F0.5 | 0.39308 | 0.82474 | 0.96774 | 0.64103 | 0.98182 | 0.5 | 0.48077 | 0.8 | 1.0 | 0.96154 | 0.87766 | 0.30612 | 0.67568 | 0.78947 | 0.9542 | 0.80769 | 0.68571 | 0.78947 | 0.92308 | 0.93939 | 0.92366 | 0.98592 | 0.50898 | 0.28205 | 0.80153 | 1.0 | 0.66372 | 0.96774 | 0.70423 | 0.57143 | 0.50847 | 1.0 | 0.55556 | 0.67568 | 1.0 | 0.97765 | 1.0 | 0.92105 | 0.78947 | 0.90909 | 0.75 | 0.84071 | 0.90361 | 0.46512 | 0.90909 | 0.88561 | 0.5303 | 0.81395 | 1.0 | 0.875 | 0.1938 | 0.63218 | F0.5 score |
| F1 | 0.42735 | 0.86486 | 0.92308 | 0.66667 | 0.98182 | 0.28571 | 0.52632 | 0.8 | 1.0 | 0.90909 | 0.89189 | 0.25 | 0.68966 | 0.80769 | 0.9434 | 0.80769 | 0.75 | 0.6 | 0.92308 | 0.96124 | 0.89299 | 0.96552 | 0.425 | 0.28205 | 0.84 | 1.0 | 0.6 | 0.92308 | 0.73733 | 0.57143 | 0.6 | 1.0 | 0.33333 | 0.76923 | 1.0 | 0.98592 | 1.0 | 0.82353 | 0.77419 | 0.8 | 0.75 | 0.80851 | 0.9375 | 0.5 | 0.94118 | 0.90566 | 0.62222 | 0.875 | 1.0 | 0.875 | 0.15873 | 0.73333 | F1 score - harmonic mean of precision and sensitivity |
| F2 | 0.46816 | 0.90909 | 0.88235 | 0.69444 | 0.98182 | 0.2 | 0.5814 | 0.8 | 1.0 | 0.86207 | 0.90659 | 0.21127 | 0.70423 | 0.82677 | 0.93284 | 0.80769 | 0.82759 | 0.48387 | 0.92308 | 0.98413 | 0.86429 | 0.94595 | 0.36481 | 0.28205 | 0.88235 | 1.0 | 0.54745 | 0.88235 | 0.77369 | 0.57143 | 0.73171 | 1.0 | 0.2381 | 0.89286 | 1.0 | 0.99432 | 1.0 | 0.74468 | 0.75949 | 0.71429 | 0.75 | 0.77869 | 0.97403 | 0.54054 | 0.97561 | 0.92664 | 0.75269 | 0.94595 | 1.0 | 0.875 | 0.13441 | 0.87302 | F2 score |
| FDR | 0.62687 | 0.2 | 0.0 | 0.375 | 0.01818 | 0.0 | 0.54545 | 0.2 | 0.0 | 0.0 | 0.13158 | 0.64 | 0.33333 | 0.22222 | 0.03846 | 0.19231 | 0.35135 | 0.0 | 0.07692 | 0.07463 | 0.05469 | 0.0 | 0.41379 | 0.71795 | 0.22222 | 0.0 | 0.28571 | 0.0 | 0.31624 | 0.42857 | 0.53846 | 0.0 | 0.0 | 0.375 | 0.0 | 0.02778 | 0.0 | 0.0 | 0.2 | 0.0 | 0.25 | 0.13636 | 0.11765 | 0.55556 | 0.11111 | 0.12727 | 0.51724 | 0.22222 | 0.0 | 0.125 | 0.77273 | 0.42105 | False discovery rate |
| FN | 25 | 2 | 1 | 2 | 1 | 5 | 3 | 1 | 0 | 1 | 3 | 38 | 8 | 4 | 2 | 5 | 3 | 4 | 1 | 0 | 22 | 1 | 34 | 28 | 2 | 0 | 14 | 2 | 20 | 3 | 1 | 0 | 4 | 0 | 0 | 0 | 0 | 3 | 4 | 2 | 5 | 6 | 0 | 3 | 0 | 3 | 2 | 0 | 0 | 1 | 36 | 0 | False negative/miss/type 2 error |
| FNR | 0.5 | 0.05882 | 0.14286 | 0.28571 | 0.01818 | 0.83333 | 0.375 | 0.2 | 0.0 | 0.16667 | 0.08333 | 0.80851 | 0.28571 | 0.16 | 0.07407 | 0.19231 | 0.11111 | 0.57143 | 0.07692 | 0.0 | 0.15385 | 0.06667 | 0.66667 | 0.71795 | 0.08696 | 0.0 | 0.48276 | 0.14286 | 0.2 | 0.42857 | 0.14286 | 0.0 | 0.8 | 0.0 | 0.0 | 0.0 | 0.0 | 0.3 | 0.25 | 0.33333 | 0.25 | 0.24 | 0.0 | 0.42857 | 0.0 | 0.05882 | 0.125 | 0.0 | 0.0 | 0.125 | 0.87805 | 0.0 | Miss rate or false negative rate |
| FOR | 0.02033 | 0.00159 | 0.00077 | 0.00155 | 0.00081 | 0.00386 | 0.00233 | 0.00077 | 0.0 | 0.00077 | 0.00238 | 0.02987 | 0.00631 | 0.00315 | 0.00157 | 0.00393 | 0.00238 | 0.00309 | 0.00078 | 0.0 | 0.01882 | 0.00078 | 0.02681 | 0.02226 | 0.00157 | 0.0 | 0.01097 | 0.00156 | 0.01695 | 0.00233 | 0.00078 | 0.0 | 0.00309 | 0.0 | 0.0 | 0.0 | 0.0 | 0.00233 | 0.00312 | 0.00155 | 0.00392 | 0.00471 | 0.0 | 0.00233 | 0.0 | 0.00242 | 0.00158 | 0.0 | 0.0 | 0.00078 | 0.02824 | 0.0 | False omission rate |
| FP | 42 | 8 | 0 | 3 | 1 | 0 | 6 | 1 | 0 | 0 | 5 | 16 | 10 | 6 | 1 | 5 | 13 | 0 | 1 | 5 | 7 | 0 | 12 | 28 | 6 | 0 | 6 | 0 | 37 | 3 | 7 | 0 | 0 | 3 | 0 | 2 | 0 | 0 | 3 | 0 | 5 | 3 | 2 | 5 | 1 | 7 | 15 | 4 | 0 | 1 | 17 | 8 | False positive/type 1 error/false alarm |
| FPR | 0.03368 | 0.00633 | 0.0 | 0.00233 | 0.00081 | 0.0 | 0.00465 | 0.00077 | 0.0 | 0.0 | 0.00397 | 0.0128 | 0.00788 | 0.00472 | 0.00079 | 0.00393 | 0.01024 | 0.0 | 0.00078 | 0.00405 | 0.00607 | 0.0 | 0.00963 | 0.02226 | 0.00471 | 0.0 | 0.00473 | 0.0 | 0.03091 | 0.00233 | 0.00543 | 0.0 | 0.0 | 0.00232 | 0.0 | 0.00163 | 0.0 | 0.0 | 0.00234 | 0.0 | 0.00392 | 0.00236 | 0.00156 | 0.00388 | 0.00078 | 0.00562 | 0.01171 | 0.00312 | 0.0 | 0.00078 | 0.01354 | 0.00622 | Fall-out or false positive rate |
| G | 0.43193 | 0.86772 | 0.92582 | 0.66815 | 0.98182 | 0.40825 | 0.533 | 0.8 | 1.0 | 0.91287 | 0.89222 | 0.26256 | 0.69007 | 0.80829 | 0.94356 | 0.80769 | 0.75933 | 0.65465 | 0.92308 | 0.96196 | 0.89436 | 0.96609 | 0.44204 | 0.28205 | 0.8427 | 1.0 | 0.60783 | 0.92582 | 0.7396 | 0.57143 | 0.62897 | 1.0 | 0.44721 | 0.79057 | 1.0 | 0.98601 | 1.0 | 0.83666 | 0.7746 | 0.8165 | 0.75 | 0.81016 | 0.93934 | 0.50395 | 0.94281 | 0.90631 | 0.64993 | 0.88192 | 1.0 | 0.875 | 0.16648 | 0.76089 | G-measure geometric mean of precision and sensitivity |
| GI | 0.46632 | 0.93484 | 0.85714 | 0.71196 | 0.98101 | 0.16667 | 0.62035 | 0.79923 | 1.0 | 0.83333 | 0.9127 | 0.17869 | 0.70641 | 0.83528 | 0.92514 | 0.80376 | 0.87865 | 0.42857 | 0.9223 | 0.99595 | 0.84009 | 0.93333 | 0.3237 | 0.25979 | 0.90833 | 1.0 | 0.51251 | 0.85714 | 0.76909 | 0.5691 | 0.85172 | 1.0 | 0.2 | 0.99768 | 1.0 | 0.99837 | 1.0 | 0.7 | 0.74766 | 0.66667 | 0.74608 | 0.75764 | 0.99844 | 0.56755 | 0.99922 | 0.93556 | 0.86329 | 0.99688 | 1.0 | 0.87422 | 0.10842 | 0.99378 | Gini index |
| GM | 0.6951 | 0.96707 | 0.92582 | 0.84417 | 0.99047 | 0.40825 | 0.78873 | 0.89408 | 1.0 | 0.91287 | 0.95553 | 0.43479 | 0.84182 | 0.91435 | 0.96187 | 0.89695 | 0.93797 | 0.65465 | 0.96039 | 0.99797 | 0.91707 | 0.96609 | 0.57456 | 0.52514 | 0.95328 | 1.0 | 0.71749 | 0.92582 | 0.8805 | 0.75505 | 0.9233 | 1.0 | 0.44721 | 0.99884 | 1.0 | 0.99918 | 1.0 | 0.83666 | 0.86501 | 0.8165 | 0.86433 | 0.87075 | 0.99922 | 0.75446 | 0.99961 | 0.96741 | 0.92992 | 0.99844 | 1.0 | 0.93505 | 0.34684 | 0.99688 | G-mean geometric mean of specificity and sensitivity |
| IBA | 0.25785 | 0.88613 | 0.73469 | 0.51067 | 0.96398 | 0.02778 | 0.3917 | 0.64012 | 1.0 | 0.69444 | 0.84057 | 0.03862 | 0.51177 | 0.70622 | 0.85739 | 0.65297 | 0.79104 | 0.18367 | 0.85213 | 0.99998 | 0.71673 | 0.87111 | 0.11322 | 0.08392 | 0.834 | 1.0 | 0.26871 | 0.73469 | 0.64418 | 0.3271 | 0.73533 | 1.0 | 0.04 | 0.99999 | 1.0 | 1.0 | 1.0 | 0.49 | 0.56293 | 0.44444 | 0.56322 | 0.57803 | 1.0 | 0.32747 | 1.0 | 0.88609 | 0.76679 | 0.99999 | 1.0 | 0.76571 | 0.0163 | 0.99996 | Index of balanced accuracy |
| ICSI | -0.12687 | 0.74118 | 0.85714 | 0.33929 | 0.96364 | 0.16667 | 0.07955 | 0.6 | 1.0 | 0.83333 | 0.78509 | -0.44851 | 0.38095 | 0.61778 | 0.88746 | 0.61538 | 0.53754 | 0.42857 | 0.84615 | 0.92537 | 0.79147 | 0.93333 | -0.08046 | -0.4359 | 0.69082 | 1.0 | 0.23153 | 0.85714 | 0.48376 | 0.14286 | 0.31868 | 1.0 | 0.2 | 0.625 | 1.0 | 0.97222 | 1.0 | 0.7 | 0.55 | 0.66667 | 0.5 | 0.62364 | 0.88235 | 0.01587 | 0.88889 | 0.8139 | 0.35776 | 0.77778 | 1.0 | 0.75 | -0.65078 | 0.57895 | Individual classification success index |
| IS | 3.27487 | 4.93157 | 7.53361 | 6.85554 | 4.53313 | 7.756 | 6.20346 | 7.69711 | 7.01903 | 7.756 | 4.9675 | 3.31244 | 4.94865 | 5.33454 | 5.52949 | 5.3324 | 4.96158 | 7.53361 | 6.52505 | 4.27487 | 3.09995 | 6.43407 | 3.89802 | 3.22959 | 5.45483 | 6.88153 | 4.99755 | 6.53361 | 3.14867 | 6.72625 | 6.41813 | 7.01903 | 8.01903 | 7.34096 | 5.8174 | 4.17104 | 7.756 | 7.01903 | 6.01903 | 7.756 | 5.604 | 5.4856 | 6.2535 | 6.36368 | 7.17104 | 4.47214 | 5.29034 | 6.17104 | 8.01903 | 7.14832 | 2.84591 | 6.09304 | Information score |
| J | 0.27174 | 0.7619 | 0.85714 | 0.5 | 0.96429 | 0.16667 | 0.35714 | 0.66667 | 1.0 | 0.83333 | 0.80488 | 0.14286 | 0.52632 | 0.67742 | 0.89286 | 0.67742 | 0.6 | 0.42857 | 0.85714 | 0.92537 | 0.80667 | 0.93333 | 0.26984 | 0.16418 | 0.72414 | 1.0 | 0.42857 | 0.85714 | 0.58394 | 0.4 | 0.42857 | 1.0 | 0.2 | 0.625 | 1.0 | 0.97222 | 1.0 | 0.7 | 0.63158 | 0.66667 | 0.6 | 0.67857 | 0.88235 | 0.33333 | 0.88889 | 0.82759 | 0.45161 | 0.77778 | 1.0 | 0.77778 | 0.08621 | 0.57895 | Jaccard index |
| LS | 9.6791 | 30.51765 | 185.28571 | 115.80357 | 23.15306 | 216.16667 | 73.69318 | 207.52 | 129.7 | 216.16667 | 31.28728 | 9.93447 | 30.88095 | 40.35111 | 46.18946 | 40.29142 | 31.15916 | 185.28571 | 92.09467 | 19.35821 | 8.57392 | 86.46667 | 14.90805 | 9.38001 | 43.8599 | 117.90909 | 31.94581 | 92.64286 | 8.86838 | 105.87755 | 85.51648 | 129.7 | 259.4 | 162.125 | 56.3913 | 18.01389 | 216.16667 | 129.7 | 64.85 | 216.16667 | 48.6375 | 44.80545 | 76.29412 | 82.34921 | 144.11111 | 22.19465 | 39.13362 | 72.05556 | 259.4 | 141.85938 | 7.18958 | 68.26316 | Lift score |
| MCC | 0.40561 | 0.86394 | 0.92546 | 0.66624 | 0.98101 | 0.40746 | 0.52965 | 0.79923 | 1.0 | 0.91252 | 0.88906 | 0.24288 | 0.68299 | 0.80438 | 0.94239 | 0.80376 | 0.75355 | 0.65364 | 0.9223 | 0.96001 | 0.88223 | 0.96572 | 0.42553 | 0.25979 | 0.83967 | 1.0 | 0.60038 | 0.9251 | 0.71613 | 0.5691 | 0.62645 | 1.0 | 0.44652 | 0.78965 | 1.0 | 0.98521 | 1.0 | 0.83569 | 0.77188 | 0.81586 | 0.74608 | 0.8067 | 0.9386 | 0.50092 | 0.94244 | 0.90235 | 0.64451 | 0.88054 | 1.0 | 0.87422 | 0.1469 | 0.75852 | Matthews correlation coefficient |
| MCCI | Weak | Strong | Very Strong | Moderate | Very Strong | Weak | Moderate | Strong | Very Strong | Very Strong | Strong | Negligible | Moderate | Strong | Very Strong | Strong | Strong | Moderate | Very Strong | Very Strong | Strong | Very Strong | Weak | Negligible | Strong | Very Strong | Moderate | Very Strong | Strong | Moderate | Moderate | Very Strong | Weak | Strong | Very Strong | Very Strong | Very Strong | Strong | Strong | Strong | Strong | Strong | Very Strong | Moderate | Very Strong | Very Strong | Moderate | Strong | Very Strong | Strong | Negligible | Strong | Matthews correlation coefficient interpretation |
| MCEN | 0.52714 | 0.18528 | 0.06011 | 0.21896 | 0.03108 | 0.27288 | 0.34541 | 0.12914 | 0 | 0.06457 | 0.1146 | 0.55886 | 0.29561 | 0.20686 | 0.07719 | 0.20686 | 0.22162 | 0.1976 | 0.08152 | 0.04995 | 0.16189 | 0.03904 | 0.46883 | 0.49037 | 0.20085 | 0 | 0.39645 | 0.08152 | 0.31529 | 0.26874 | 0.24042 | 0 | 0.0386 | 0.1686 | 0 | 0.02569 | 0 | 0.11938 | 0.203 | 0.12914 | 0.24242 | 0.17806 | 0.07207 | 0.33321 | 0.05279 | 0.13391 | 0.25247 | 0.09929 | 0 | 0.10557 | 0.5812 | 0.23651 | Modified confusion entropy |
| MK | 0.35281 | 0.79841 | 0.99923 | 0.62345 | 0.98101 | 0.99614 | 0.45221 | 0.79923 | 1.0 | 0.99923 | 0.86604 | 0.33013 | 0.66035 | 0.77463 | 0.95996 | 0.80376 | 0.64627 | 0.99691 | 0.9223 | 0.92537 | 0.92649 | 0.99922 | 0.55939 | 0.25979 | 0.7762 | 1.0 | 0.70331 | 0.99844 | 0.66681 | 0.5691 | 0.46076 | 1.0 | 0.99691 | 0.625 | 1.0 | 0.97222 | 1.0 | 0.99767 | 0.79688 | 0.99845 | 0.74608 | 0.85893 | 0.88235 | 0.44212 | 0.88889 | 0.87031 | 0.48118 | 0.77778 | 1.0 | 0.87422 | 0.19904 | 0.57895 | Markedness |
| N | 1247 | 1263 | 1290 | 1290 | 1242 | 1291 | 1289 | 1292 | 1287 | 1291 | 1261 | 1250 | 1269 | 1272 | 1270 | 1271 | 1270 | 1290 | 1284 | 1235 | 1154 | 1282 | 1246 | 1258 | 1274 | 1286 | 1268 | 1283 | 1197 | 1290 | 1290 | 1287 | 1292 | 1292 | 1274 | 1227 | 1291 | 1287 | 1281 | 1291 | 1277 | 1272 | 1282 | 1290 | 1289 | 1246 | 1281 | 1283 | 1292 | 1289 | 1256 | 1286 | Condition negative |
| NLR | 0.51743 | 0.0592 | 0.14286 | 0.28638 | 0.0182 | 0.83333 | 0.37675 | 0.20015 | 0.0 | 0.16667 | 0.08367 | 0.81899 | 0.28798 | 0.16076 | 0.07413 | 0.19307 | 0.11226 | 0.57143 | 0.07698 | 0.0 | 0.15479 | 0.06667 | 0.67315 | 0.73429 | 0.08737 | 0.0 | 0.48505 | 0.14286 | 0.20638 | 0.42957 | 0.14364 | 0.0 | 0.8 | 0.0 | 0.0 | 0.0 | 0.0 | 0.3 | 0.25059 | 0.33333 | 0.25098 | 0.24057 | 0.0 | 0.43024 | 0.0 | 0.05916 | 0.12648 | 0.0 | 0.0 | 0.1251 | 0.8901 | 0.0 | Negative likelihood ratio |
| NLRI | Negligible | Good | Fair | Poor | Good | Negligible | Poor | Poor | Good | Fair | Good | Negligible | Poor | Fair | Good | Fair | Fair | Negligible | Good | Good | Fair | Good | Negligible | Negligible | Good | Good | Poor | Fair | Poor | Poor | Fair | Good | Negligible | Good | Good | Good | Good | Poor | Poor | Poor | Poor | Poor | Good | Poor | Good | Good | Fair | Good | Good | Fair | Negligible | Good | Negative likelihood ratio interpretation |
| NPV | 0.97967 | 0.99841 | 0.99923 | 0.99845 | 0.99919 | 0.99614 | 0.99767 | 0.99923 | 1.0 | 0.99923 | 0.99762 | 0.97013 | 0.99369 | 0.99685 | 0.99843 | 0.99607 | 0.99762 | 0.99691 | 0.99922 | 1.0 | 0.98118 | 0.99922 | 0.97319 | 0.97774 | 0.99843 | 1.0 | 0.98903 | 0.99844 | 0.98305 | 0.99767 | 0.99922 | 1.0 | 0.99691 | 1.0 | 1.0 | 1.0 | 1.0 | 0.99767 | 0.99688 | 0.99845 | 0.99608 | 0.99529 | 1.0 | 0.99767 | 1.0 | 0.99758 | 0.99842 | 1.0 | 1.0 | 0.99922 | 0.97176 | 1.0 | Negative predictive value |
| OC | 0.5 | 0.94118 | 1.0 | 0.71429 | 0.98182 | 1.0 | 0.625 | 0.8 | 1.0 | 1.0 | 0.91667 | 0.36 | 0.71429 | 0.84 | 0.96154 | 0.80769 | 0.88889 | 1.0 | 0.92308 | 1.0 | 0.94531 | 1.0 | 0.58621 | 0.28205 | 0.91304 | 1.0 | 0.71429 | 1.0 | 0.8 | 0.57143 | 0.85714 | 1.0 | 1.0 | 1.0 | 1.0 | 1.0 | 1.0 | 1.0 | 0.8 | 1.0 | 0.75 | 0.86364 | 1.0 | 0.57143 | 1.0 | 0.94118 | 0.875 | 1.0 | 1.0 | 0.875 | 0.22727 | 1.0 | Overlap coefficient |
| OOC | 0.43193 | 0.86772 | 0.92582 | 0.66815 | 0.98182 | 0.40825 | 0.533 | 0.8 | 1.0 | 0.91287 | 0.89222 | 0.26256 | 0.69007 | 0.80829 | 0.94356 | 0.80769 | 0.75933 | 0.65465 | 0.92308 | 0.96196 | 0.89436 | 0.96609 | 0.44204 | 0.28205 | 0.8427 | 1.0 | 0.60783 | 0.92582 | 0.7396 | 0.57143 | 0.62897 | 1.0 | 0.44721 | 0.79057 | 1.0 | 0.98601 | 1.0 | 0.83666 | 0.7746 | 0.8165 | 0.75 | 0.81016 | 0.93934 | 0.50395 | 0.94281 | 0.90631 | 0.64993 | 0.88192 | 1.0 | 0.875 | 0.16648 | 0.76089 | Otsuka-Ochiai coefficient |
| OP | 0.63032 | 0.96516 | 0.92231 | 0.83061 | 0.98969 | 0.28186 | 0.7645 | 0.88773 | 1.0 | 0.90832 | 0.95234 | 0.28328 | 0.8233 | 0.90768 | 0.95962 | 0.88786 | 0.93397 | 0.59692 | 0.95885 | 0.99412 | 0.89733 | 0.96475 | 0.46817 | 0.4046 | 0.95073 | 1.0 | 0.66853 | 0.92153 | 0.86047 | 0.72372 | 0.91961 | 1.0 | 0.33025 | 0.99652 | 1.0 | 0.99764 | 1.0 | 0.82122 | 0.85289 | 0.79846 | 0.85135 | 0.85786 | 0.99768 | 0.7229 | 0.99884 | 0.9648 | 0.92609 | 0.99535 | 1.0 | 0.93218 | 0.17918 | 0.99071 | Optimized precision |
| P | 50 | 34 | 7 | 7 | 55 | 6 | 8 | 5 | 10 | 6 | 36 | 47 | 28 | 25 | 27 | 26 | 27 | 7 | 13 | 62 | 143 | 15 | 51 | 39 | 23 | 11 | 29 | 14 | 100 | 7 | 7 | 10 | 5 | 5 | 23 | 70 | 6 | 10 | 16 | 6 | 20 | 25 | 15 | 7 | 8 | 51 | 16 | 14 | 5 | 8 | 41 | 11 | Condition positive or support |
| PLR | 14.84524 | 148.58824 | None | 307.14286 | 1219.41818 | None | 134.27083 | 1033.6 | None | None | 231.18333 | 14.96011 | 90.64286 | 178.08 | 1175.92593 | 205.31538 | 86.83761 | None | 1185.23077 | 247.0 | 139.49451 | None | 34.61111 | 12.67216 | 193.86957 | None | 109.31034 | None | 25.88108 | 245.71429 | 157.95918 | None | None | 430.66667 | None | 613.5 | None | None | 320.25 | None | 191.55 | 322.24 | 641.0 | 147.42857 | 1289.0 | 167.52941 | 74.725 | 320.75 | None | 1127.875 | 9.01004 | 160.75 | Positive likelihood ratio |
| PLRI | Good | Good | None | Good | Good | None | Good | Good | None | None | Good | Good | Good | Good | Good | Good | Good | None | Good | Good | Good | None | Good | Good | Good | None | Good | None | Good | Good | Good | None | None | Good | None | Good | None | None | Good | None | Good | Good | Good | Good | Good | Good | Good | Good | None | Good | Fair | Good | Positive likelihood ratio interpretation |
| POP | 1297 | 1297 | 1297 | 1297 | 1297 | 1297 | 1297 | 1297 | 1297 | 1297 | 1297 | 1297 | 1297 | 1297 | 1297 | 1297 | 1297 | 1297 | 1297 | 1297 | 1297 | 1297 | 1297 | 1297 | 1297 | 1297 | 1297 | 1297 | 1297 | 1297 | 1297 | 1297 | 1297 | 1297 | 1297 | 1297 | 1297 | 1297 | 1297 | 1297 | 1297 | 1297 | 1297 | 1297 | 1297 | 1297 | 1297 | 1297 | 1297 | 1297 | 1297 | 1297 | Population |
| PPV | 0.37313 | 0.8 | 1.0 | 0.625 | 0.98182 | 1.0 | 0.45455 | 0.8 | 1.0 | 1.0 | 0.86842 | 0.36 | 0.66667 | 0.77778 | 0.96154 | 0.80769 | 0.64865 | 1.0 | 0.92308 | 0.92537 | 0.94531 | 1.0 | 0.58621 | 0.28205 | 0.77778 | 1.0 | 0.71429 | 1.0 | 0.68376 | 0.57143 | 0.46154 | 1.0 | 1.0 | 0.625 | 1.0 | 0.97222 | 1.0 | 1.0 | 0.8 | 1.0 | 0.75 | 0.86364 | 0.88235 | 0.44444 | 0.88889 | 0.87273 | 0.48276 | 0.77778 | 1.0 | 0.875 | 0.22727 | 0.57895 | Precision or positive predictive value |
| PRE | 0.03855 | 0.02621 | 0.0054 | 0.0054 | 0.04241 | 0.00463 | 0.00617 | 0.00386 | 0.00771 | 0.00463 | 0.02776 | 0.03624 | 0.02159 | 0.01928 | 0.02082 | 0.02005 | 0.02082 | 0.0054 | 0.01002 | 0.0478 | 0.11025 | 0.01157 | 0.03932 | 0.03007 | 0.01773 | 0.00848 | 0.02236 | 0.01079 | 0.0771 | 0.0054 | 0.0054 | 0.00771 | 0.00386 | 0.00386 | 0.01773 | 0.05397 | 0.00463 | 0.00771 | 0.01234 | 0.00463 | 0.01542 | 0.01928 | 0.01157 | 0.0054 | 0.00617 | 0.03932 | 0.01234 | 0.01079 | 0.00386 | 0.00617 | 0.03161 | 0.00848 | Prevalence |
| Q | 0.93264 | 0.9992 | None | 0.99814 | 0.99997 | None | 0.9944 | 0.99961 | None | None | 0.99928 | 0.89619 | 0.99367 | 0.9982 | 0.99987 | 0.99812 | 0.99742 | None | 0.99987 | None | 0.99778 | None | 0.96184 | 0.89046 | 0.9991 | None | 0.99116 | None | 0.98418 | 0.99651 | 0.99818 | None | None | None | None | None | None | None | 0.99844 | None | 0.99738 | 0.99851 | None | 0.99418 | None | 0.99929 | 0.99662 | None | None | 0.99978 | 0.82019 | None | Yule Q - coefficient of colligation |
| QI | Strong | Strong | None | Strong | Strong | None | Strong | Strong | None | None | Strong | Strong | Strong | Strong | Strong | Strong | Strong | None | Strong | None | Strong | None | Strong | Strong | Strong | None | Strong | None | Strong | Strong | Strong | None | None | None | None | None | None | None | Strong | None | Strong | Strong | None | Strong | None | Strong | Strong | None | None | Strong | Strong | None | Yule Q interpretation |
| RACC | 0.00199 | 0.00081 | 2e-05 | 3e-05 | 0.0018 | 0.0 | 5e-05 | 1e-05 | 6e-05 | 2e-05 | 0.00081 | 0.0007 | 0.0005 | 0.0004 | 0.00042 | 0.0004 | 0.00059 | 1e-05 | 0.0001 | 0.00247 | 0.01088 | 0.00012 | 0.00088 | 0.0009 | 0.00037 | 7e-05 | 0.00036 | 0.0001 | 0.00696 | 3e-05 | 5e-05 | 6e-05 | 0.0 | 2e-05 | 0.00031 | 0.003 | 2e-05 | 4e-05 | 0.00014 | 1e-05 | 0.00024 | 0.00033 | 0.00015 | 4e-05 | 4e-05 | 0.00167 | 0.00028 | 0.00015 | 1e-05 | 4e-05 | 0.00054 | 0.00012 | Random accuracy |
| RACCU | 0.00203 | 0.00081 | 3e-05 | 3e-05 | 0.0018 | 1e-05 | 5e-05 | 1e-05 | 6e-05 | 2e-05 | 0.00081 | 0.00077 | 0.0005 | 0.0004 | 0.00042 | 0.0004 | 0.00061 | 1e-05 | 0.0001 | 0.00247 | 0.01091 | 0.00012 | 0.00095 | 0.0009 | 0.00037 | 7e-05 | 0.00037 | 0.0001 | 0.007 | 3e-05 | 6e-05 | 6e-05 | 1e-05 | 3e-05 | 0.00031 | 0.003 | 2e-05 | 4e-05 | 0.00014 | 1e-05 | 0.00024 | 0.00033 | 0.00015 | 4e-05 | 4e-05 | 0.00167 | 0.0003 | 0.00015 | 1e-05 | 4e-05 | 0.00059 | 0.00013 | Random accuracy unbiased |
| TN | 1205 | 1255 | 1290 | 1287 | 1241 | 1291 | 1283 | 1291 | 1287 | 1291 | 1256 | 1234 | 1259 | 1266 | 1269 | 1266 | 1257 | 1290 | 1283 | 1230 | 1147 | 1282 | 1234 | 1230 | 1268 | 1286 | 1262 | 1283 | 1160 | 1287 | 1283 | 1287 | 1292 | 1289 | 1274 | 1225 | 1291 | 1287 | 1278 | 1291 | 1272 | 1269 | 1280 | 1285 | 1288 | 1239 | 1266 | 1279 | 1292 | 1288 | 1239 | 1278 | True negative/correct rejection |
| TNR | 0.96632 | 0.99367 | 1.0 | 0.99767 | 0.99919 | 1.0 | 0.99535 | 0.99923 | 1.0 | 1.0 | 0.99603 | 0.9872 | 0.99212 | 0.99528 | 0.99921 | 0.99607 | 0.98976 | 1.0 | 0.99922 | 0.99595 | 0.99393 | 1.0 | 0.99037 | 0.97774 | 0.99529 | 1.0 | 0.99527 | 1.0 | 0.96909 | 0.99767 | 0.99457 | 1.0 | 1.0 | 0.99768 | 1.0 | 0.99837 | 1.0 | 1.0 | 0.99766 | 1.0 | 0.99608 | 0.99764 | 0.99844 | 0.99612 | 0.99922 | 0.99438 | 0.98829 | 0.99688 | 1.0 | 0.99922 | 0.98646 | 0.99378 | Specificity or true negative rate |
| TON | 1230 | 1257 | 1291 | 1289 | 1242 | 1296 | 1286 | 1292 | 1287 | 1292 | 1259 | 1272 | 1267 | 1270 | 1271 | 1271 | 1260 | 1294 | 1284 | 1230 | 1169 | 1283 | 1268 | 1258 | 1270 | 1286 | 1276 | 1285 | 1180 | 1290 | 1284 | 1287 | 1296 | 1289 | 1274 | 1225 | 1291 | 1290 | 1282 | 1293 | 1277 | 1275 | 1280 | 1288 | 1288 | 1242 | 1268 | 1279 | 1292 | 1289 | 1275 | 1278 | Test outcome negative |
| TOP | 67 | 40 | 6 | 8 | 55 | 1 | 11 | 5 | 10 | 5 | 38 | 25 | 30 | 27 | 26 | 26 | 37 | 3 | 13 | 67 | 128 | 14 | 29 | 39 | 27 | 11 | 21 | 12 | 117 | 7 | 13 | 10 | 1 | 8 | 23 | 72 | 6 | 7 | 15 | 4 | 20 | 22 | 17 | 9 | 9 | 55 | 29 | 18 | 5 | 8 | 22 | 19 | Test outcome positive |
| TP | 25 | 32 | 6 | 5 | 54 | 1 | 5 | 4 | 10 | 5 | 33 | 9 | 20 | 21 | 25 | 21 | 24 | 3 | 12 | 62 | 121 | 14 | 17 | 11 | 21 | 11 | 15 | 12 | 80 | 4 | 6 | 10 | 1 | 5 | 23 | 70 | 6 | 7 | 12 | 4 | 15 | 19 | 15 | 4 | 8 | 48 | 14 | 14 | 5 | 7 | 5 | 11 | True positive/hit |
| TPR | 0.5 | 0.94118 | 0.85714 | 0.71429 | 0.98182 | 0.16667 | 0.625 | 0.8 | 1.0 | 0.83333 | 0.91667 | 0.19149 | 0.71429 | 0.84 | 0.92593 | 0.80769 | 0.88889 | 0.42857 | 0.92308 | 1.0 | 0.84615 | 0.93333 | 0.33333 | 0.28205 | 0.91304 | 1.0 | 0.51724 | 0.85714 | 0.8 | 0.57143 | 0.85714 | 1.0 | 0.2 | 1.0 | 1.0 | 1.0 | 1.0 | 0.7 | 0.75 | 0.66667 | 0.75 | 0.76 | 1.0 | 0.57143 | 1.0 | 0.94118 | 0.875 | 1.0 | 1.0 | 0.875 | 0.12195 | 1.0 | Sensitivity, recall, hit rate, or true positive rate |
| Y | 0.46632 | 0.93484 | 0.85714 | 0.71196 | 0.98101 | 0.16667 | 0.62035 | 0.79923 | 1.0 | 0.83333 | 0.9127 | 0.17869 | 0.70641 | 0.83528 | 0.92514 | 0.80376 | 0.87865 | 0.42857 | 0.9223 | 0.99595 | 0.84009 | 0.93333 | 0.3237 | 0.25979 | 0.90833 | 1.0 | 0.51251 | 0.85714 | 0.76909 | 0.5691 | 0.85172 | 1.0 | 0.2 | 0.99768 | 1.0 | 0.99837 | 1.0 | 0.7 | 0.74766 | 0.66667 | 0.74608 | 0.75764 | 0.99844 | 0.56755 | 0.99922 | 0.93556 | 0.86329 | 0.99688 | 1.0 | 0.87422 | 0.10842 | 0.99378 | Youden index |
| dInd | 0.50113 | 0.05916 | 0.14286 | 0.28572 | 0.0182 | 0.83333 | 0.37503 | 0.2 | 0.0 | 0.16667 | 0.08343 | 0.80861 | 0.28582 | 0.16007 | 0.07408 | 0.19235 | 0.11158 | 0.57143 | 0.07693 | 0.00405 | 0.15397 | 0.06667 | 0.66674 | 0.71829 | 0.08708 | 0.0 | 0.48278 | 0.14286 | 0.20237 | 0.42858 | 0.14296 | 0.0 | 0.8 | 0.00232 | 0.0 | 0.00163 | 0.0 | 0.3 | 0.25001 | 0.33333 | 0.25003 | 0.24001 | 0.00156 | 0.42859 | 0.00078 | 0.05909 | 0.12555 | 0.00312 | 0.0 | 0.125 | 0.87815 | 0.00622 | Distance index |
| sInd | 0.64565 | 0.95817 | 0.89898 | 0.79796 | 0.98713 | 0.41074 | 0.73481 | 0.85858 | 1.0 | 0.88215 | 0.94101 | 0.42823 | 0.79789 | 0.88681 | 0.94762 | 0.86399 | 0.9211 | 0.59594 | 0.9456 | 0.99714 | 0.89113 | 0.95286 | 0.52855 | 0.49209 | 0.93842 | 1.0 | 0.65862 | 0.89898 | 0.8569 | 0.69695 | 0.89891 | 1.0 | 0.43431 | 0.99836 | 1.0 | 0.99885 | 1.0 | 0.78787 | 0.82322 | 0.7643 | 0.8232 | 0.83029 | 0.9989 | 0.69694 | 0.99945 | 0.95822 | 0.91122 | 0.9978 | 1.0 | 0.91161 | 0.37905 | 0.9956 | Similarity index |

Generated By PyCM Version 3.4
