# Supplementary material for: TopEC: prediction of Enzyme Commission classes by 3D graph neural networks and localized 3D protein descriptor
Source: Nat Commun. 2025 Mar 20;16:2737. doi: 10.1038/s41467-025-57324-5 (PMC11923149; doi:10.1038/s41467-025-57324-5)
Supplement: Supplementary file 3 — Supplementary Data 1 [file 41467_2025_57324_MOESM3_ESM.zip › Data_S1/table1/hierarchical/TopEC_distance_TEMP_2digs.html]

PyCM Report


# PyCM Report

## Dataset Type :

- Multi-Class Classification
- Imbalanced

Note 1 : Recommended statistics for this type of classification highlighted in aqua

Note 2 : The recommender system assumes that the input is the result of classification over the whole data rather than just a part of it.
If the confusion matrix is the result of test data classification, the recommendation is not valid.

## Confusion Matrix :

|  |  |  |  |  |  |  |  |  |  |  |  |  |  |  |  |  |  |  |  |  |  |  |  |  |  |  |  |  |  |  |  |  |  |  |  |  |  |  |  |  |  |  |  |  |  |  |  |  |  |  |  |  |  |  |  |  |  |  |  |  |  |  |  |  |  |  |  |  |  |  |  |  |  |  |  |  |  |  |  |  |  |  |  |  |  |  |  |  |  |  |  |  |  |  |  |  |  |  |  |  |  |  |  |  |  |  |  |  |  |  |  |  |  |  |  |  |  |  |  |  |  |  |  |  |  |  |  |  |  |  |  |  |  |  |  |  |  |  |  |  |  |  |  |  |  |  |  |  |  |  |  |  |  |  |  |  |  |  |  |  |  |  |  |  |  |  |  |  |  |  |  |  |  |  |  |  |  |  |  |  |  |  |  |  |  |  |  |  |  |  |  |  |  |  |  |  |  |  |  |  |  |  |  |  |  |  |  |  |  |  |  |  |  |  |  |  |  |  |  |  |  |  |  |  |  |  |  |  |  |  |  |  |  |  |  |  |  |  |  |  |  |  |  |  |  |  |  |  |  |  |  |  |  |  |  |  |  |  |  |  |  |  |  |  |  |  |  |  |  |  |  |  |  |  |  |  |  |  |  |  |  |  |  |  |  |  |  |  |  |  |  |  |  |  |  |  |  |  |  |  |  |  |  |  |  |  |  |  |  |  |  |  |  |  |  |  |  |  |  |  |  |  |  |  |  |  |  |  |  |  |  |  |  |  |  |  |  |  |  |  |  |  |  |  |  |  |  |  |  |  |  |  |  |  |  |  |  |  |  |  |  |  |  |  |  |  |  |  |  |  |  |  |  |  |  |  |  |  |  |  |  |  |  |  |  |  |  |  |  |  |  |  |  |  |  |  |  |  |  |  |  |  |  |  |  |  |  |  |  |  |  |  |  |  |  |  |  |  |  |  |  |  |  |  |  |  |  |  |  |  |  |  |  |  |  |  |  |  |  |  |  |  |  |  |  |  |  |  |  |  |  |  |  |  |  |  |  |  |  |  |  |  |  |  |  |  |  |  |  |  |  |  |  |  |  |  |  |  |  |  |  |  |  |  |  |  |  |  |  |  |  |  |  |  |  |  |  |  |  |  |  |  |  |  |  |  |  |  |  |  |  |  |  |  |  |  |  |  |  |  |  |  |  |  |  |  |  |  |  |  |  |  |  |  |  |  |  |  |  |  |  |  |  |  |  |  |  |  |  |  |  |  |  |  |  |  |  |  |  |  |  |  |  |  |  |  |  |  |  |  |  |  |  |  |  |  |  |  |  |  |  |  |  |  |  |  |  |  |  |  |  |  |  |  |  |  |  |  |  |  |  |  |  |  |  |  |  |  |  |  |  |  |  |  |  |  |  |  |  |  |  |  |  |  |  |  |  |  |  |  |  |  |  |  |  |  |  |  |  |  |  |  |  |  |  |  |  |  |  |  |  |  |  |  |  |  |  |  |  |  |  |  |  |  |  |  |  |  |  |  |  |  |  |  |  |  |  |  |  |  |  |  |  |  |  |  |  |  |  |  |  |  |  |  |  |  |  |  |  |  |  |  |  |  |  |  |  |  |  |  |  |  |  |  |  |  |  |  |  |  |  |  |  |  |  |  |  |  |  |  |  |  |  |  |  |  |  |  |  |  |  |  |  |  |  |  |  |  |  |  |  |  |  |  |  |  |  |  |  |  |  |  |  |  |  |  |  |  |  |  |  |  |  |  |  |  |  |  |  |  |  |  |  |  |  |  |  |  |  |  |  |  |  |  |  |  |  |  |  |  |  |  |  |  |  |  |  |  |  |  |  |  |  |  |  |  |  |  |  |  |  |  |  |  |  |  |  |  |  |  |  |  |  |  |  |  |  |  |  |  |  |  |  |  |  |  |  |  |  |  |  |  |  |  |  |  |  |  |  |  |  |  |  |  |  |  |  |  |  |  |  |  |  |  |  |  |  |  |  |  |  |  |  |  |  |  |  |  |  |  |  |  |  |  |  |  |  |  |  |  |  |  |  |  |  |  |  |  |  |  |  |  |  |  |  |  |  |  |  |  |  |  |  |  |  |  |  |  |  |  |  |  |  |  |  |  |  |  |  |  |  |  |  |  |  |  |  |  |  |  |  |  |  |  |  |  |  |  |  |  |  |  |  |  |  |  |  |  |  |  |  |  |  |  |  |  |  |  |  |  |  |  |  |  |  |  |  |  |  |  |  |  |  |  |  |  |  |  |  |  |  |  |  |  |  |  |  |  |  |  |  |  |  |  |  |  |  |  |  |  |  |  |  |  |  |
| --- | --- | --- | --- | --- | --- | --- | --- | --- | --- | --- | --- | --- | --- | --- | --- | --- | --- | --- | --- | --- | --- | --- | --- | --- | --- | --- | --- | --- | --- | --- | --- | --- | --- | --- | --- | --- | --- | --- | --- | --- | --- | --- | --- | --- | --- | --- | --- | --- | --- | --- | --- | --- | --- | --- | --- | --- | --- | --- | --- | --- | --- | --- | --- | --- | --- | --- | --- | --- | --- | --- | --- | --- | --- | --- | --- | --- | --- | --- | --- | --- | --- | --- | --- | --- | --- | --- | --- | --- | --- | --- | --- | --- | --- | --- | --- | --- | --- | --- | --- | --- | --- | --- | --- | --- | --- | --- | --- | --- | --- | --- | --- | --- | --- | --- | --- | --- | --- | --- | --- | --- | --- | --- | --- | --- | --- | --- | --- | --- | --- | --- | --- | --- | --- | --- | --- | --- | --- | --- | --- | --- | --- | --- | --- | --- | --- | --- | --- | --- | --- | --- | --- | --- | --- | --- | --- | --- | --- | --- | --- | --- | --- | --- | --- | --- | --- | --- | --- | --- | --- | --- | --- | --- | --- | --- | --- | --- | --- | --- | --- | --- | --- | --- | --- | --- | --- | --- | --- | --- | --- | --- | --- | --- | --- | --- | --- | --- | --- | --- | --- | --- | --- | --- | --- | --- | --- | --- | --- | --- | --- | --- | --- | --- | --- | --- | --- | --- | --- | --- | --- | --- | --- | --- | --- | --- | --- | --- | --- | --- | --- | --- | --- | --- | --- | --- | --- | --- | --- | --- | --- | --- | --- | --- | --- | --- | --- | --- | --- | --- | --- | --- | --- | --- | --- | --- | --- | --- | --- | --- | --- | --- | --- | --- | --- | --- | --- | --- | --- | --- | --- | --- | --- | --- | --- | --- | --- | --- | --- | --- | --- | --- | --- | --- | --- | --- | --- | --- | --- | --- | --- | --- | --- | --- | --- | --- | --- | --- | --- | --- | --- | --- | --- | --- | --- | --- | --- | --- | --- | --- | --- | --- | --- | --- | --- | --- | --- | --- | --- | --- | --- | --- | --- | --- | --- | --- | --- | --- | --- | --- | --- | --- | --- | --- | --- | --- | --- | --- | --- | --- | --- | --- | --- | --- | --- | --- | --- | --- | --- | --- | --- | --- | --- | --- | --- | --- | --- | --- | --- | --- | --- | --- | --- | --- | --- | --- | --- | --- | --- | --- | --- | --- | --- | --- | --- | --- | --- | --- | --- | --- | --- | --- | --- | --- | --- | --- | --- | --- | --- | --- | --- | --- | --- | --- | --- | --- | --- | --- | --- | --- | --- | --- | --- | --- | --- | --- | --- | --- | --- | --- | --- | --- | --- | --- | --- | --- | --- | --- | --- | --- | --- | --- | --- | --- | --- | --- | --- | --- | --- | --- | --- | --- | --- | --- | --- | --- | --- | --- | --- | --- | --- | --- | --- | --- | --- | --- | --- | --- | --- | --- | --- | --- | --- | --- | --- | --- | --- | --- | --- | --- | --- | --- | --- | --- | --- | --- | --- | --- | --- | --- | --- | --- | --- | --- | --- | --- | --- | --- | --- | --- | --- | --- | --- | --- | --- | --- | --- | --- | --- | --- | --- | --- | --- | --- | --- | --- | --- | --- | --- | --- | --- | --- | --- | --- | --- | --- | --- | --- | --- | --- | --- | --- | --- | --- | --- | --- | --- | --- | --- | --- | --- | --- | --- | --- | --- | --- | --- | --- | --- | --- | --- | --- | --- | --- | --- | --- | --- | --- | --- | --- | --- | --- | --- | --- | --- | --- | --- | --- | --- | --- | --- | --- | --- | --- | --- | --- | --- | --- | --- | --- | --- | --- | --- | --- | --- | --- | --- | --- | --- | --- | --- | --- | --- | --- | --- | --- | --- | --- | --- | --- | --- | --- | --- | --- | --- | --- | --- | --- | --- | --- | --- | --- | --- | --- | --- | --- | --- | --- | --- | --- | --- | --- | --- | --- | --- | --- | --- | --- | --- | --- | --- | --- | --- | --- | --- | --- | --- | --- | --- | --- | --- | --- | --- | --- | --- | --- | --- | --- | --- | --- | --- | --- | --- | --- | --- | --- | --- | --- | --- | --- | --- | --- | --- | --- | --- | --- | --- | --- | --- | --- | --- | --- | --- | --- | --- | --- | --- | --- | --- | --- | --- | --- | --- | --- | --- | --- | --- | --- | --- | --- | --- | --- | --- | --- | --- | --- | --- | --- | --- | --- | --- | --- | --- | --- | --- | --- | --- | --- | --- | --- | --- | --- | --- | --- | --- | --- | --- | --- | --- | --- | --- | --- | --- | --- | --- | --- | --- | --- | --- | --- | --- | --- | --- | --- | --- | --- | --- | --- | --- | --- | --- | --- | --- | --- | --- | --- | --- | --- | --- | --- | --- | --- | --- | --- | --- | --- | --- | --- | --- | --- | --- | --- | --- | --- | --- | --- | --- | --- | --- | --- | --- | --- | --- | --- | --- | --- | --- | --- | --- | --- | --- | --- | --- | --- | --- | --- | --- | --- | --- | --- | --- | --- | --- | --- | --- | --- | --- | --- | --- | --- | --- | --- | --- | --- | --- | --- | --- | --- | --- | --- | --- | --- | --- | --- | --- | --- | --- | --- | --- | --- | --- | --- | --- | --- | --- | --- | --- | --- | --- | --- | --- | --- | --- | --- | --- | --- | --- | --- | --- | --- | --- | --- | --- | --- | --- | --- | --- | --- | --- | --- | --- | --- | --- | --- | --- | --- | --- | --- | --- | --- | --- | --- | --- | --- | --- | --- | --- | --- | --- | --- | --- | --- | --- | --- | --- | --- | --- | --- | --- | --- | --- | --- | --- | --- | --- | --- | --- | --- | --- | --- | --- | --- | --- | --- | --- | --- | --- | --- | --- | --- | --- | --- | --- | --- | --- | --- | --- | --- | --- | --- | --- | --- | --- | --- | --- | --- | --- | --- | --- | --- | --- | --- | --- | --- | --- | --- | --- | --- | --- | --- | --- | --- | --- | --- | --- | --- | --- | --- | --- | --- | --- | --- | --- | --- | --- | --- | --- | --- | --- | --- | --- | --- | --- | --- | --- | --- | --- | --- | --- | --- | --- | --- | --- | --- | --- | --- | --- | --- | --- | --- | --- | --- | --- | --- | --- | --- | --- | --- | --- | --- | --- | --- | --- | --- | --- | --- | --- | --- | --- | --- | --- | --- | --- | --- | --- | --- | --- | --- | --- | --- | --- | --- | --- | --- | --- | --- | --- | --- | --- | --- | --- | --- | --- | --- | --- | --- | --- | --- | --- | --- | --- | --- | --- | --- | --- | --- | --- | --- | --- | --- | --- | --- | --- | --- | --- | --- | --- | --- | --- | --- | --- | --- | --- | --- | --- | --- | --- |
| Actual | Predict  |  |  |  |  |  |  |  |  |  |  |  |  |  |  |  |  |  |  |  |  |  |  |  |  |  |  |  |  |  |  |  |  | | --- | --- | --- | --- | --- | --- | --- | --- | --- | --- | --- | --- | --- | --- | --- | --- | --- | --- | --- | --- | --- | --- | --- | --- | --- | --- | --- | --- | --- | --- | --- | --- | |  | 0 | 1 | 2 | 3 | 4 | 5 | 6 | 7 | 8 | 9 | 10 | 11 | 12 | 13 | 14 | 15 | 16 | 17 | 18 | 19 | 20 | 21 | 22 | 23 | 24 | 25 | 26 | 27 | 28 | 29 | 30 | | 0 | 25 | 1 | 0 | 1 | 0 | 1 | 0 | 3 | 4 | 0 | 1 | 2 | 1 | 0 | 0 | 2 | 1 | 4 | 1 | 0 | 0 | 0 | 0 | 0 | 1 | 0 | 0 | 0 | 0 | 2 | 0 | | 1 | 0 | 32 | 0 | 0 | 0 | 1 | 0 | 0 | 0 | 0 | 0 | 0 | 0 | 0 | 1 | 0 | 0 | 0 | 0 | 0 | 0 | 0 | 0 | 0 | 0 | 0 | 0 | 0 | 0 | 0 | 0 | | 2 | 0 | 0 | 6 | 0 | 0 | 0 | 0 | 0 | 0 | 0 | 0 | 0 | 0 | 0 | 0 | 0 | 0 | 0 | 0 | 0 | 0 | 0 | 0 | 0 | 0 | 0 | 0 | 0 | 0 | 1 | 0 | | 3 | 0 | 0 | 0 | 59 | 0 | 0 | 0 | 0 | 0 | 0 | 0 | 0 | 1 | 0 | 0 | 0 | 0 | 1 | 1 | 0 | 0 | 0 | 0 | 0 | 0 | 0 | 0 | 0 | 0 | 0 | 0 | | 4 | 1 | 2 | 0 | 0 | 1 | 0 | 0 | 0 | 0 | 0 | 0 | 0 | 0 | 0 | 1 | 0 | 1 | 0 | 0 | 0 | 0 | 0 | 0 | 0 | 0 | 0 | 0 | 0 | 0 | 0 | 0 | | 5 | 1 | 0 | 0 | 0 | 0 | 9 | 0 | 0 | 0 | 1 | 0 | 0 | 0 | 0 | 0 | 0 | 0 | 0 | 0 | 0 | 1 | 0 | 0 | 0 | 1 | 0 | 0 | 0 | 0 | 0 | 0 | | 6 | 0 | 1 | 0 | 0 | 0 | 0 | 15 | 0 | 0 | 0 | 0 | 0 | 0 | 0 | 0 | 0 | 0 | 0 | 0 | 0 | 0 | 0 | 0 | 0 | 0 | 0 | 0 | 0 | 0 | 0 | 0 | | 7 | 0 | 0 | 0 | 0 | 0 | 0 | 0 | 33 | 0 | 0 | 0 | 0 | 0 | 0 | 0 | 0 | 0 | 0 | 0 | 0 | 0 | 0 | 0 | 0 | 0 | 0 | 0 | 0 | 0 | 3 | 0 | | 8 | 10 | 1 | 0 | 0 | 0 | 1 | 0 | 0 | 9 | 6 | 0 | 1 | 0 | 0 | 6 | 2 | 3 | 4 | 0 | 0 | 1 | 1 | 1 | 0 | 1 | 0 | 0 | 0 | 0 | 0 | 0 | | 9 | 2 | 1 | 0 | 0 | 0 | 0 | 0 | 0 | 0 | 20 | 0 | 0 | 0 | 0 | 1 | 0 | 0 | 1 | 0 | 0 | 0 | 0 | 0 | 0 | 0 | 0 | 0 | 0 | 1 | 0 | 2 | | 10 | 2 | 0 | 0 | 0 | 0 | 0 | 0 | 0 | 1 | 0 | 21 | 0 | 0 | 0 | 0 | 0 | 0 | 0 | 0 | 0 | 0 | 0 | 0 | 0 | 0 | 0 | 0 | 0 | 0 | 0 | 1 | | 11 | 1 | 0 | 0 | 0 | 0 | 1 | 0 | 0 | 3 | 0 | 0 | 46 | 0 | 0 | 1 | 0 | 0 | 0 | 0 | 0 | 0 | 0 | 0 | 0 | 1 | 0 | 0 | 0 | 0 | 0 | 0 | | 12 | 1 | 0 | 0 | 0 | 0 | 0 | 0 | 0 | 0 | 0 | 1 | 0 | 24 | 0 | 0 | 0 | 0 | 1 | 0 | 0 | 0 | 0 | 0 | 0 | 0 | 0 | 0 | 0 | 0 | 0 | 0 | | 13 | 1 | 0 | 0 | 0 | 0 | 0 | 0 | 0 | 2 | 0 | 0 | 0 | 0 | 3 | 0 | 1 | 0 | 0 | 0 | 0 | 0 | 0 | 0 | 0 | 0 | 0 | 0 | 0 | 0 | 0 | 0 | | 14 | 8 | 1 | 0 | 0 | 0 | 0 | 0 | 0 | 0 | 1 | 1 | 2 | 8 | 0 | 234 | 5 | 1 | 4 | 0 | 0 | 0 | 3 | 3 | 0 | 1 | 8 | 0 | 0 | 0 | 3 | 1 | | 15 | 0 | 0 | 0 | 0 | 0 | 1 | 0 | 0 | 0 | 0 | 0 | 0 | 0 | 0 | 0 | 11 | 1 | 15 | 4 | 1 | 0 | 2 | 0 | 0 | 0 | 1 | 0 | 0 | 0 | 3 | 0 | | 16 | 3 | 1 | 0 | 0 | 0 | 2 | 0 | 2 | 0 | 1 | 0 | 0 | 2 | 0 | 1 | 1 | 60 | 0 | 0 | 0 | 0 | 0 | 1 | 1 | 0 | 1 | 0 | 0 | 0 | 0 | 1 | | 17 | 1 | 0 | 0 | 3 | 0 | 0 | 0 | 0 | 1 | 0 | 0 | 1 | 0 | 0 | 1 | 6 | 2 | 84 | 1 | 2 | 0 | 0 | 0 | 0 | 0 | 0 | 0 | 0 | 0 | 4 | 1 | | 18 | 0 | 0 | 0 | 0 | 0 | 0 | 0 | 0 | 1 | 0 | 0 | 0 | 0 | 0 | 0 | 0 | 0 | 0 | 6 | 0 | 0 | 0 | 0 | 0 | 0 | 0 | 0 | 0 | 0 | 0 | 0 | | 19 | 0 | 0 | 0 | 0 | 0 | 0 | 0 | 0 | 0 | 0 | 0 | 0 | 0 | 0 | 0 | 4 | 0 | 0 | 0 | 115 | 0 | 0 | 0 | 0 | 0 | 0 | 0 | 0 | 0 | 0 | 0 | | 20 | 1 | 0 | 0 | 0 | 0 | 0 | 0 | 0 | 0 | 0 | 0 | 0 | 0 | 0 | 1 | 2 | 0 | 3 | 0 | 0 | 23 | 0 | 0 | 0 | 0 | 0 | 0 | 0 | 0 | 0 | 2 | | 21 | 4 | 0 | 0 | 0 | 0 | 0 | 0 | 0 | 0 | 0 | 0 | 0 | 0 | 0 | 0 | 1 | 1 | 0 | 0 | 0 | 0 | 51 | 0 | 0 | 2 | 0 | 0 | 0 | 0 | 1 | 0 | | 22 | 0 | 0 | 0 | 0 | 0 | 0 | 0 | 0 | 1 | 0 | 0 | 0 | 0 | 0 | 0 | 0 | 0 | 0 | 0 | 0 | 0 | 0 | 4 | 0 | 0 | 1 | 1 | 0 | 0 | 0 | 0 | | 23 | 0 | 0 | 0 | 0 | 0 | 0 | 0 | 0 | 0 | 0 | 0 | 0 | 0 | 0 | 0 | 0 | 0 | 0 | 0 | 0 | 0 | 0 | 0 | 8 | 0 | 0 | 0 | 0 | 0 | 0 | 0 | | 24 | 0 | 0 | 0 | 0 | 0 | 0 | 0 | 0 | 0 | 0 | 0 | 0 | 0 | 0 | 0 | 0 | 0 | 0 | 0 | 0 | 0 | 0 | 0 | 0 | 48 | 0 | 3 | 0 | 0 | 0 | 0 | | 25 | 0 | 0 | 0 | 0 | 0 | 0 | 0 | 0 | 0 | 0 | 0 | 0 | 0 | 0 | 0 | 0 | 0 | 1 | 0 | 0 | 1 | 0 | 0 | 0 | 0 | 14 | 0 | 0 | 0 | 0 | 0 | | 26 | 0 | 0 | 0 | 0 | 0 | 0 | 0 | 0 | 0 | 0 | 0 | 0 | 0 | 0 | 0 | 0 | 0 | 0 | 0 | 0 | 0 | 0 | 0 | 0 | 0 | 0 | 14 | 0 | 0 | 0 | 0 | | 27 | 0 | 0 | 0 | 0 | 0 | 0 | 0 | 0 | 0 | 0 | 0 | 0 | 0 | 0 | 0 | 0 | 0 | 0 | 0 | 0 | 0 | 0 | 0 | 0 | 0 | 0 | 0 | 5 | 0 | 0 | 0 | | 28 | 0 | 0 | 0 | 0 | 0 | 0 | 0 | 0 | 0 | 0 | 0 | 0 | 0 | 0 | 0 | 0 | 1 | 0 | 0 | 0 | 0 | 0 | 0 | 0 | 0 | 0 | 0 | 0 | 7 | 0 | 0 | | 29 | 6 | 0 | 0 | 0 | 0 | 0 | 0 | 0 | 3 | 1 | 3 | 0 | 1 | 0 | 4 | 4 | 0 | 6 | 0 | 2 | 0 | 2 | 0 | 0 | 0 | 4 | 0 | 0 | 0 | 5 | 0 | | 30 | 0 | 0 | 0 | 0 | 0 | 0 | 0 | 0 | 0 | 0 | 0 | 0 | 0 | 0 | 0 | 0 | 0 | 0 | 0 | 0 | 0 | 0 | 0 | 0 | 0 | 0 | 0 | 0 | 0 | 0 | 11 | |

## Overall Statistics :

|  |  |
| --- | --- |
| 95% CI | (0.75054,0.79611) |
| ACC Macro | 0.98538 |
| ARI | 0.69381 |
| AUNP | 0.88053 |
| AUNU | 0.87262 |
| Bangdiwala B | 0.72003 |
| Bennett S | 0.76577 |
| CBA | 0.66699 |
| CSI | 0.49268 |
| Chi-Squared | 22979.09687 |
| Chi-Squared DF | 900 |
| Conditional Entropy | 1.21131 |
| Cramer V | 0.76849 |
| Cross Entropy | 4.25976 |
| F1 Macro | 0.72314 |
| F1 Micro | 0.77332 |
| FNR Macro | 0.24705 |
| FNR Micro | 0.22668 |
| FPR Macro | 0.00771 |
| FPR Micro | 0.00756 |
| Gwet AC1 | 0.76614 |
| Hamming Loss | 0.22668 |
| Joint Entropy | 5.42756 |
| KL Divergence | 0.0435 |
| Kappa | 0.75401 |
| Kappa 95% CI | (0.72928,0.77874) |
| Kappa No Prevalence | 0.54665 |
| Kappa Standard Error | 0.01262 |
| Kappa Unbiased | 0.75389 |
| Krippendorff Alpha | 0.75398 |
| Lambda A | 0.70977 |
| Lambda B | 0.72562 |
| Mutual Information | 3.05673 |
| NIR | 0.21897 |
| Overall ACC | 0.77332 |
| Overall CEN | 0.19708 |
| Overall J | (18.97869,0.61222) |
| Overall MCC | 0.75477 |
| Overall MCEN | 0.28836 |
| Overall RACC | 0.07852 |
| Overall RACCU | 0.07898 |
| P-Value | None |
| PPV Macro | 0.73974 |
| PPV Micro | 0.77332 |
| Pearson C | 0.97292 |
| Phi-Squared | 17.71711 |
| RCI | 0.72499 |
| RR | 41.83871 |
| Reference Entropy | 4.21625 |
| Response Entropy | 4.26804 |
| SOA1(Landis & Koch) | Substantial |
| SOA2(Fleiss) | Excellent |
| SOA3(Altman) | Good |
| SOA4(Cicchetti) | Excellent |
| SOA5(Cramer) | Strong |
| SOA6(Matthews) | Strong |
| Scott PI | 0.75389 |
| Standard Error | 0.01163 |
| TNR Macro | 0.99229 |
| TNR Micro | 0.99244 |
| TPR Macro | 0.75295 |
| TPR Micro | 0.77332 |
| Zero-one Loss | 294 |

## Class Statistics :

|  |  |  |  |  |  |  |  |  |  |  |  |  |  |  |  |  |  |  |  |  |  |  |  |  |  |  |  |  |  |  |  |  |
| --- | --- | --- | --- | --- | --- | --- | --- | --- | --- | --- | --- | --- | --- | --- | --- | --- | --- | --- | --- | --- | --- | --- | --- | --- | --- | --- | --- | --- | --- | --- | --- | --- |
| Class | 0 | 1 | 2 | 3 | 4 | 5 | 6 | 7 | 8 | 9 | 10 | 11 | 12 | 13 | 14 | 15 | 16 | 17 | 18 | 19 | 20 | 21 | 22 | 23 | 24 | 25 | 26 | 27 | 28 | 29 | 30 | Description |
| ACC | 0.94834 | 0.99229 | 0.99923 | 0.9946 | 0.99614 | 0.99152 | 0.99923 | 0.99383 | 0.95837 | 0.98612 | 0.99229 | 0.98998 | 0.98766 | 0.99692 | 0.94834 | 0.95682 | 0.97841 | 0.95143 | 0.99383 | 0.99306 | 0.99075 | 0.98689 | 0.99383 | 0.99923 | 0.99229 | 0.98689 | 0.99692 | 1.0 | 0.99846 | 0.95914 | 0.99383 | Accuracy |
| AGF | 0.6763 | 0.95225 | 0.93905 | 0.97267 | 0.44652 | 0.81203 | 0.97405 | 0.95087 | 0.45351 | 0.8364 | 0.90769 | 0.93083 | 0.90791 | 0.69475 | 0.89907 | 0.52514 | 0.88394 | 0.86244 | 0.85467 | 0.98047 | 0.86149 | 0.92021 | 0.73424 | 0.98765 | 0.96115 | 0.866 | 0.97229 | 1.0 | 0.93505 | 0.36194 | 0.93377 | Adjusted F-score |
| AGM | 0.82804 | 0.98019 | 0.96281 | 0.98506 | 0.70344 | 0.91175 | 0.98402 | 0.9755 | 0.7059 | 0.91615 | 0.95442 | 0.96159 | 0.9636 | 0.82686 | 0.93653 | 0.74799 | 0.93315 | 0.91665 | 0.95884 | 0.988 | 0.92126 | 0.95537 | 0.87497 | 0.99942 | 0.98063 | 0.95892 | 0.99767 | 1.0 | 0.96704 | 0.66152 | 0.99534 | Adjusted geometric mean |
| AM | 17 | 6 | -1 | 1 | -5 | 3 | -1 | 2 | -22 | 2 | 2 | -1 | 10 | -4 | -33 | 0 | -6 | 17 | 6 | 1 | -6 | -1 | 2 | 1 | 4 | 13 | 4 | 0 | 0 | -19 | 8 | Difference between automatic and manual classification |
| AUC | 0.73316 | 0.96742 | 0.92857 | 0.97419 | 0.58333 | 0.84343 | 0.96875 | 0.95635 | 0.58934 | 0.8532 | 0.91764 | 0.93155 | 0.93933 | 0.71429 | 0.90358 | 0.6299 | 0.8851 | 0.87572 | 0.92586 | 0.98107 | 0.85819 | 0.92177 | 0.78378 | 0.99961 | 0.96778 | 0.93165 | 0.99844 | 1.0 | 0.93711 | 0.55421 | 0.99689 | Area under the ROC curve |
| AUCI | Good | Excellent | Excellent | Excellent | Poor | Very Good | Excellent | Excellent | Poor | Very Good | Excellent | Excellent | Excellent | Good | Excellent | Fair | Very Good | Very Good | Excellent | Excellent | Very Good | Excellent | Good | Excellent | Excellent | Excellent | Excellent | Excellent | Excellent | Poor | Excellent | AUC value interpretation |
| AUPR | 0.43657 | 0.87059 | 0.92857 | 0.94406 | 0.58333 | 0.6274 | 0.96875 | 0.89254 | 0.27574 | 0.69048 | 0.80889 | 0.87627 | 0.76877 | 0.71429 | 0.87811 | 0.28205 | 0.81215 | 0.73123 | 0.65934 | 0.96236 | 0.80168 | 0.8572 | 0.50794 | 0.94444 | 0.90695 | 0.67888 | 0.88889 | 1.0 | 0.875 | 0.17461 | 0.78947 | Area under the PR curve |
| BCD | 0.00655 | 0.00231 | 0.00039 | 0.00039 | 0.00193 | 0.00116 | 0.00039 | 0.00077 | 0.00848 | 0.00077 | 0.00077 | 0.00039 | 0.00386 | 0.00154 | 0.01272 | 0.0 | 0.00231 | 0.00655 | 0.00231 | 0.00039 | 0.00231 | 0.00039 | 0.00077 | 0.00039 | 0.00154 | 0.00501 | 0.00154 | 0.0 | 0.0 | 0.00732 | 0.00308 | Bray-Curtis dissimilarity |
| BM | 0.46632 | 0.93484 | 0.85714 | 0.94837 | 0.16667 | 0.68686 | 0.9375 | 0.9127 | 0.17869 | 0.70641 | 0.83528 | 0.8631 | 0.87865 | 0.42857 | 0.80716 | 0.25979 | 0.7702 | 0.75143 | 0.85172 | 0.96214 | 0.71638 | 0.84353 | 0.56755 | 0.99922 | 0.93556 | 0.86329 | 0.99688 | 1.0 | 0.87422 | 0.10842 | 0.99378 | Informedness or bookmaker informedness |
| CEN | 0.49786 | 0.13748 | 0.04819 | 0.0596 | 0.29111 | 0.30028 | 0.02706 | 0.08731 | 0.55179 | 0.25083 | 0.16359 | 0.12662 | 0.18517 | 0.19109 | 0.15058 | 0.49813 | 0.21088 | 0.26349 | 0.22495 | 0.04187 | 0.17963 | 0.14007 | 0.28828 | 0.0407 | 0.09666 | 0.23085 | 0.08065 | 0 | 0.08465 | 0.60194 | 0.19895 | Confusion entropy |
| DOR | 28.69048 | 2510.0 | None | 6052.41667 | None | 410.46429 | None | 2763.2 | 18.26645 | 314.75 | 1107.75 | 1355.90476 | 773.53846 | None | 274.19294 | 17.25765 | 387.91444 | 105.0 | 1099.71429 | 6744.75 | 1075.03704 | 870.54167 | 342.66667 | None | 2832.0 | 590.8 | None | None | 9016.0 | 10.12255 | None | Diagnostic odds ratio |
| DP | 0.80369 | 1.87434 | None | 2.08509 | None | 1.44077 | None | 1.89735 | 0.69559 | 1.3772 | 1.67849 | 1.72689 | 1.5925 | None | 1.34417 | 0.68198 | 1.42724 | 1.11434 | 1.67675 | 2.11102 | 1.67131 | 1.62079 | 1.39755 | None | 1.90324 | 1.52798 | None | None | 2.18051 | 0.55425 | None | Discriminant power |
| DPI | Poor | Limited | None | Fair | None | Limited | None | Limited | Poor | Limited | Limited | Limited | Limited | None | Limited | Poor | Limited | Limited | Limited | Fair | Limited | Limited | Limited | None | Limited | Limited | None | None | Fair | Poor | None | Discriminant power interpretation |
| ERR | 0.05166 | 0.00771 | 0.00077 | 0.0054 | 0.00386 | 0.00848 | 0.00077 | 0.00617 | 0.04163 | 0.01388 | 0.00771 | 0.01002 | 0.01234 | 0.00308 | 0.05166 | 0.04318 | 0.02159 | 0.04857 | 0.00617 | 0.00694 | 0.00925 | 0.01311 | 0.00617 | 0.00077 | 0.00771 | 0.01311 | 0.00308 | 0.0 | 0.00154 | 0.04086 | 0.00617 | Error rate |
| F0.5 | 0.39308 | 0.82474 | 0.96774 | 0.93949 | 0.5 | 0.58442 | 0.98684 | 0.87766 | 0.30612 | 0.67568 | 0.78947 | 0.88123 | 0.68571 | 0.78947 | 0.90839 | 0.28205 | 0.83102 | 0.69652 | 0.50847 | 0.95993 | 0.84559 | 0.86149 | 0.46512 | 0.90909 | 0.88561 | 0.5303 | 0.81395 | 1.0 | 0.875 | 0.1938 | 0.63218 | F0.5 score |
| F1 | 0.42735 | 0.86486 | 0.92308 | 0.944 | 0.28571 | 0.62069 | 0.96774 | 0.89189 | 0.25 | 0.68966 | 0.80769 | 0.87619 | 0.75 | 0.6 | 0.87477 | 0.28205 | 0.81081 | 0.72727 | 0.6 | 0.96234 | 0.7931 | 0.85714 | 0.5 | 0.94118 | 0.90566 | 0.62222 | 0.875 | 1.0 | 0.875 | 0.15873 | 0.73333 | F1 score - harmonic mean of precision and sensitivity |
| F2 | 0.46816 | 0.90909 | 0.88235 | 0.94855 | 0.2 | 0.66176 | 0.94937 | 0.90659 | 0.21127 | 0.70423 | 0.82677 | 0.87121 | 0.82759 | 0.48387 | 0.84355 | 0.28205 | 0.79156 | 0.76087 | 0.73171 | 0.96477 | 0.74675 | 0.85284 | 0.54054 | 0.97561 | 0.92664 | 0.75269 | 0.94595 | 1.0 | 0.875 | 0.13441 | 0.87302 | F2 score |
| FDR | 0.62687 | 0.2 | 0.0 | 0.06349 | 0.0 | 0.4375 | 0.0 | 0.13158 | 0.64 | 0.33333 | 0.22222 | 0.11538 | 0.35135 | 0.0 | 0.06773 | 0.71795 | 0.15493 | 0.32258 | 0.53846 | 0.04167 | 0.11538 | 0.13559 | 0.55556 | 0.11111 | 0.12727 | 0.51724 | 0.22222 | 0.0 | 0.125 | 0.77273 | 0.42105 | False discovery rate |
| FN | 25 | 2 | 1 | 3 | 5 | 4 | 1 | 3 | 38 | 8 | 4 | 7 | 3 | 4 | 50 | 28 | 17 | 23 | 1 | 4 | 9 | 9 | 3 | 0 | 3 | 2 | 0 | 0 | 1 | 36 | 0 | False negative/miss/type 2 error |
| FNR | 0.5 | 0.05882 | 0.14286 | 0.04839 | 0.83333 | 0.30769 | 0.0625 | 0.08333 | 0.80851 | 0.28571 | 0.16 | 0.13208 | 0.11111 | 0.57143 | 0.17606 | 0.71795 | 0.22078 | 0.21495 | 0.14286 | 0.03361 | 0.28125 | 0.15 | 0.42857 | 0.0 | 0.05882 | 0.125 | 0.0 | 0.0 | 0.125 | 0.87805 | 0.0 | Miss rate or false negative rate |
| FOR | 0.02033 | 0.00159 | 0.00077 | 0.00243 | 0.00386 | 0.00312 | 0.00078 | 0.00238 | 0.02987 | 0.00631 | 0.00315 | 0.00562 | 0.00238 | 0.00309 | 0.0478 | 0.02226 | 0.01387 | 0.01961 | 0.00078 | 0.0034 | 0.00708 | 0.00727 | 0.00233 | 0.0 | 0.00242 | 0.00158 | 0.0 | 0.0 | 0.00078 | 0.02824 | 0.0 | False omission rate |
| FP | 42 | 8 | 0 | 4 | 0 | 7 | 0 | 5 | 16 | 10 | 6 | 6 | 13 | 0 | 17 | 28 | 11 | 40 | 7 | 5 | 3 | 8 | 5 | 1 | 7 | 15 | 4 | 0 | 1 | 17 | 8 | False positive/type 1 error/false alarm |
| FPR | 0.03368 | 0.00633 | 0.0 | 0.00324 | 0.0 | 0.00545 | 0.0 | 0.00397 | 0.0128 | 0.00788 | 0.00472 | 0.00482 | 0.01024 | 0.0 | 0.01678 | 0.02226 | 0.00902 | 0.03361 | 0.00543 | 0.00424 | 0.00237 | 0.00647 | 0.00388 | 0.00078 | 0.00562 | 0.01171 | 0.00312 | 0.0 | 0.00078 | 0.01354 | 0.00622 | Fall-out or false positive rate |
| G | 0.43193 | 0.86772 | 0.92582 | 0.94403 | 0.40825 | 0.62404 | 0.96825 | 0.89222 | 0.26256 | 0.69007 | 0.80829 | 0.87623 | 0.75933 | 0.65465 | 0.87644 | 0.28205 | 0.81148 | 0.72925 | 0.62897 | 0.96235 | 0.79738 | 0.85717 | 0.50395 | 0.94281 | 0.90631 | 0.64993 | 0.88192 | 1.0 | 0.875 | 0.16648 | 0.76089 | G-measure geometric mean of precision and sensitivity |
| GI | 0.46632 | 0.93484 | 0.85714 | 0.94837 | 0.16667 | 0.68686 | 0.9375 | 0.9127 | 0.17869 | 0.70641 | 0.83528 | 0.8631 | 0.87865 | 0.42857 | 0.80716 | 0.25979 | 0.7702 | 0.75143 | 0.85172 | 0.96214 | 0.71638 | 0.84353 | 0.56755 | 0.99922 | 0.93556 | 0.86329 | 0.99688 | 1.0 | 0.87422 | 0.10842 | 0.99378 | Gini index |
| GM | 0.6951 | 0.96707 | 0.92582 | 0.97393 | 0.40825 | 0.82978 | 0.96825 | 0.95553 | 0.43479 | 0.84182 | 0.91435 | 0.92938 | 0.93797 | 0.65465 | 0.90006 | 0.52514 | 0.87875 | 0.87101 | 0.9233 | 0.98096 | 0.84679 | 0.91897 | 0.75446 | 0.99961 | 0.96741 | 0.92992 | 0.99844 | 1.0 | 0.93505 | 0.34684 | 0.99688 | G-mean geometric mean of specificity and sensitivity |
| IBA | 0.25785 | 0.88613 | 0.73469 | 0.90571 | 0.02778 | 0.48043 | 0.87891 | 0.84057 | 0.03862 | 0.51177 | 0.70622 | 0.75383 | 0.79104 | 0.18367 | 0.68109 | 0.08392 | 0.60867 | 0.62108 | 0.73533 | 0.93402 | 0.51708 | 0.72329 | 0.32747 | 1.0 | 0.88609 | 0.76679 | 0.99999 | 1.0 | 0.76571 | 0.0163 | 0.99996 | Index of balanced accuracy |
| ICSI | -0.12687 | 0.74118 | 0.85714 | 0.88812 | 0.16667 | 0.25481 | 0.9375 | 0.78509 | -0.44851 | 0.38095 | 0.61778 | 0.75254 | 0.53754 | 0.42857 | 0.75621 | -0.4359 | 0.62429 | 0.46247 | 0.31868 | 0.92472 | 0.60337 | 0.71441 | 0.01587 | 0.88889 | 0.8139 | 0.35776 | 0.77778 | 1.0 | 0.75 | -0.65078 | 0.57895 | Individual classification success index |
| IS | 3.27487 | 4.93157 | 7.53361 | 4.29213 | 7.756 | 5.81045 | 6.34096 | 4.9675 | 3.31244 | 4.94865 | 5.33454 | 4.43616 | 4.96158 | 7.53361 | 2.09004 | 3.22959 | 3.83132 | 3.03762 | 6.41813 | 3.38474 | 5.16409 | 4.22385 | 6.36368 | 7.17104 | 4.47214 | 5.29034 | 6.17104 | 8.01903 | 7.14832 | 2.84591 | 6.09304 | Information score |
| J | 0.27174 | 0.7619 | 0.85714 | 0.89394 | 0.16667 | 0.45 | 0.9375 | 0.80488 | 0.14286 | 0.52632 | 0.67742 | 0.77966 | 0.6 | 0.42857 | 0.77741 | 0.16418 | 0.68182 | 0.57143 | 0.42857 | 0.92742 | 0.65714 | 0.75 | 0.33333 | 0.88889 | 0.82759 | 0.45161 | 0.77778 | 1.0 | 0.77778 | 0.08621 | 0.57895 | Jaccard index |
| LS | 9.6791 | 30.51765 | 185.28571 | 19.59114 | 216.16667 | 56.12019 | 81.0625 | 31.28728 | 9.93447 | 30.88095 | 40.35111 | 21.64804 | 31.15916 | 185.28571 | 4.25759 | 9.38001 | 14.2345 | 8.21134 | 85.51648 | 10.44503 | 35.85457 | 18.68559 | 82.34921 | 144.11111 | 22.19465 | 39.13362 | 72.05556 | 259.4 | 141.85938 | 7.18958 | 68.26316 | Lift score |
| MCC | 0.40561 | 0.86394 | 0.92546 | 0.9412 | 0.40746 | 0.61985 | 0.96787 | 0.88906 | 0.24288 | 0.68299 | 0.80438 | 0.87101 | 0.75355 | 0.65364 | 0.84493 | 0.25979 | 0.80012 | 0.70307 | 0.62645 | 0.95853 | 0.79287 | 0.85031 | 0.50092 | 0.94244 | 0.90235 | 0.64451 | 0.88054 | 1.0 | 0.87422 | 0.1469 | 0.75852 | Matthews correlation coefficient |
| MCCI | Weak | Strong | Very Strong | Very Strong | Weak | Moderate | Very Strong | Strong | Negligible | Moderate | Strong | Strong | Strong | Moderate | Strong | Negligible | Strong | Strong | Moderate | Very Strong | Strong | Strong | Moderate | Very Strong | Very Strong | Moderate | Strong | Very Strong | Strong | Negligible | Strong | Matthews correlation coefficient interpretation |
| MCEN | 0.59039 | 0.20929 | 0.0679 | 0.09633 | 0.30825 | 0.38549 | 0.04232 | 0.12945 | 0.60266 | 0.33392 | 0.23366 | 0.19431 | 0.25035 | 0.22321 | 0.23637 | 0.54887 | 0.31426 | 0.36674 | 0.27158 | 0.06907 | 0.25537 | 0.21095 | 0.33753 | 0.05963 | 0.15127 | 0.28519 | 0.11216 | 0 | 0.11926 | 0.63537 | 0.26716 | Modified confusion entropy |
| MK | 0.35281 | 0.79841 | 0.99923 | 0.93408 | 0.99614 | 0.55938 | 0.99922 | 0.86604 | 0.33013 | 0.66035 | 0.77463 | 0.87899 | 0.64627 | 0.99691 | 0.88447 | 0.25979 | 0.8312 | 0.65781 | 0.46076 | 0.95493 | 0.87753 | 0.85714 | 0.44212 | 0.88889 | 0.87031 | 0.48118 | 0.77778 | 1.0 | 0.87422 | 0.19904 | 0.57895 | Markedness |
| N | 1247 | 1263 | 1290 | 1235 | 1291 | 1284 | 1281 | 1261 | 1250 | 1269 | 1272 | 1244 | 1270 | 1290 | 1013 | 1258 | 1220 | 1190 | 1290 | 1178 | 1265 | 1237 | 1290 | 1289 | 1246 | 1281 | 1283 | 1292 | 1289 | 1256 | 1286 | Condition negative |
| NLR | 0.51743 | 0.0592 | 0.14286 | 0.04854 | 0.83333 | 0.30938 | 0.0625 | 0.08367 | 0.81899 | 0.28798 | 0.16076 | 0.13272 | 0.11226 | 0.57143 | 0.17906 | 0.73429 | 0.22279 | 0.22243 | 0.14364 | 0.03376 | 0.28192 | 0.15098 | 0.43024 | 0.0 | 0.05916 | 0.12648 | 0.0 | 0.0 | 0.1251 | 0.8901 | 0.0 | Negative likelihood ratio |
| NLRI | Negligible | Good | Fair | Good | Negligible | Poor | Good | Good | Negligible | Poor | Fair | Fair | Fair | Negligible | Fair | Negligible | Poor | Poor | Fair | Good | Poor | Fair | Poor | Good | Good | Fair | Good | Good | Fair | Negligible | Good | Negative likelihood ratio interpretation |
| NPV | 0.97967 | 0.99841 | 0.99923 | 0.99757 | 0.99614 | 0.99688 | 0.99922 | 0.99762 | 0.97013 | 0.99369 | 0.99685 | 0.99438 | 0.99762 | 0.99691 | 0.9522 | 0.97774 | 0.98613 | 0.98039 | 0.99922 | 0.9966 | 0.99292 | 0.99273 | 0.99767 | 1.0 | 0.99758 | 0.99842 | 1.0 | 1.0 | 0.99922 | 0.97176 | 1.0 | Negative predictive value |
| OC | 0.5 | 0.94118 | 1.0 | 0.95161 | 1.0 | 0.69231 | 1.0 | 0.91667 | 0.36 | 0.71429 | 0.84 | 0.88462 | 0.88889 | 1.0 | 0.93227 | 0.28205 | 0.84507 | 0.78505 | 0.85714 | 0.96639 | 0.88462 | 0.86441 | 0.57143 | 1.0 | 0.94118 | 0.875 | 1.0 | 1.0 | 0.875 | 0.22727 | 1.0 | Overlap coefficient |
| OOC | 0.43193 | 0.86772 | 0.92582 | 0.94403 | 0.40825 | 0.62404 | 0.96825 | 0.89222 | 0.26256 | 0.69007 | 0.80829 | 0.87623 | 0.75933 | 0.65465 | 0.87644 | 0.28205 | 0.81148 | 0.72925 | 0.62897 | 0.96235 | 0.79738 | 0.85717 | 0.50395 | 0.94281 | 0.90631 | 0.64993 | 0.88192 | 1.0 | 0.875 | 0.16648 | 0.76089 | Otsuka-Ochiai coefficient |
| OP | 0.63032 | 0.96516 | 0.92231 | 0.97143 | 0.28186 | 0.81234 | 0.96697 | 0.95234 | 0.28328 | 0.8233 | 0.90768 | 0.92168 | 0.93397 | 0.59692 | 0.86021 | 0.4046 | 0.85879 | 0.84789 | 0.91961 | 0.97809 | 0.82827 | 0.90904 | 0.7229 | 0.99884 | 0.9648 | 0.92609 | 0.99535 | 1.0 | 0.93218 | 0.17918 | 0.99071 | Optimized precision |
| P | 50 | 34 | 7 | 62 | 6 | 13 | 16 | 36 | 47 | 28 | 25 | 53 | 27 | 7 | 284 | 39 | 77 | 107 | 7 | 119 | 32 | 60 | 7 | 8 | 51 | 16 | 14 | 5 | 8 | 41 | 11 | Condition positive or support |
| PLR | 14.84524 | 148.58824 | None | 293.81048 | None | 126.98901 | None | 231.18333 | 14.96011 | 90.64286 | 178.08 | 179.94969 | 86.83761 | None | 49.09735 | 12.67216 | 86.42267 | 23.35514 | 157.95918 | 227.68067 | 303.07292 | 131.43125 | 147.42857 | 1289.0 | 167.52941 | 74.725 | 320.75 | None | 1127.875 | 9.01004 | 160.75 | Positive likelihood ratio |
| PLRI | Good | Good | None | Good | None | Good | None | Good | Good | Good | Good | Good | Good | None | Good | Good | Good | Good | Good | Good | Good | Good | Good | Good | Good | Good | Good | None | Good | Fair | Good | Positive likelihood ratio interpretation |
| POP | 1297 | 1297 | 1297 | 1297 | 1297 | 1297 | 1297 | 1297 | 1297 | 1297 | 1297 | 1297 | 1297 | 1297 | 1297 | 1297 | 1297 | 1297 | 1297 | 1297 | 1297 | 1297 | 1297 | 1297 | 1297 | 1297 | 1297 | 1297 | 1297 | 1297 | 1297 | Population |
| PPV | 0.37313 | 0.8 | 1.0 | 0.93651 | 1.0 | 0.5625 | 1.0 | 0.86842 | 0.36 | 0.66667 | 0.77778 | 0.88462 | 0.64865 | 1.0 | 0.93227 | 0.28205 | 0.84507 | 0.67742 | 0.46154 | 0.95833 | 0.88462 | 0.86441 | 0.44444 | 0.88889 | 0.87273 | 0.48276 | 0.77778 | 1.0 | 0.875 | 0.22727 | 0.57895 | Precision or positive predictive value |
| PRE | 0.03855 | 0.02621 | 0.0054 | 0.0478 | 0.00463 | 0.01002 | 0.01234 | 0.02776 | 0.03624 | 0.02159 | 0.01928 | 0.04086 | 0.02082 | 0.0054 | 0.21897 | 0.03007 | 0.05937 | 0.0825 | 0.0054 | 0.09175 | 0.02467 | 0.04626 | 0.0054 | 0.00617 | 0.03932 | 0.01234 | 0.01079 | 0.00386 | 0.00617 | 0.03161 | 0.00848 | Prevalence |
| Q | 0.93264 | 0.9992 | None | 0.99967 | None | 0.99514 | None | 0.99928 | 0.89619 | 0.99367 | 0.9982 | 0.99853 | 0.99742 | None | 0.99273 | 0.89046 | 0.99486 | 0.98113 | 0.99818 | 0.9997 | 0.99814 | 0.99771 | 0.99418 | None | 0.99929 | 0.99662 | None | None | 0.99978 | 0.82019 | None | Yule Q - coefficient of colligation |
| QI | Strong | Strong | None | Strong | None | Strong | None | Strong | Strong | Strong | Strong | Strong | Strong | None | Strong | Strong | Strong | Strong | Strong | Strong | Strong | Strong | Strong | None | Strong | Strong | None | None | Strong | Strong | None | Yule Q interpretation |
| RACC | 0.00199 | 0.00081 | 2e-05 | 0.00232 | 0.0 | 0.00012 | 0.00014 | 0.00081 | 0.0007 | 0.0005 | 0.0004 | 0.00164 | 0.00059 | 1e-05 | 0.04238 | 0.0009 | 0.00325 | 0.00789 | 5e-05 | 0.00849 | 0.00049 | 0.0021 | 4e-05 | 4e-05 | 0.00167 | 0.00028 | 0.00015 | 1e-05 | 4e-05 | 0.00054 | 0.00012 | Random accuracy |
| RACCU | 0.00203 | 0.00081 | 3e-05 | 0.00232 | 1e-05 | 0.00012 | 0.00014 | 0.00081 | 0.00077 | 0.0005 | 0.0004 | 0.00164 | 0.00061 | 1e-05 | 0.04254 | 0.0009 | 0.00326 | 0.00793 | 6e-05 | 0.00849 | 0.0005 | 0.0021 | 4e-05 | 4e-05 | 0.00167 | 0.0003 | 0.00015 | 1e-05 | 4e-05 | 0.00059 | 0.00013 | Random accuracy unbiased |
| TN | 1205 | 1255 | 1290 | 1231 | 1291 | 1277 | 1281 | 1256 | 1234 | 1259 | 1266 | 1238 | 1257 | 1290 | 996 | 1230 | 1209 | 1150 | 1283 | 1173 | 1262 | 1229 | 1285 | 1288 | 1239 | 1266 | 1279 | 1292 | 1288 | 1239 | 1278 | True negative/correct rejection |
| TNR | 0.96632 | 0.99367 | 1.0 | 0.99676 | 1.0 | 0.99455 | 1.0 | 0.99603 | 0.9872 | 0.99212 | 0.99528 | 0.99518 | 0.98976 | 1.0 | 0.98322 | 0.97774 | 0.99098 | 0.96639 | 0.99457 | 0.99576 | 0.99763 | 0.99353 | 0.99612 | 0.99922 | 0.99438 | 0.98829 | 0.99688 | 1.0 | 0.99922 | 0.98646 | 0.99378 | Specificity or true negative rate |
| TON | 1230 | 1257 | 1291 | 1234 | 1296 | 1281 | 1282 | 1259 | 1272 | 1267 | 1270 | 1245 | 1260 | 1294 | 1046 | 1258 | 1226 | 1173 | 1284 | 1177 | 1271 | 1238 | 1288 | 1288 | 1242 | 1268 | 1279 | 1292 | 1289 | 1275 | 1278 | Test outcome negative |
| TOP | 67 | 40 | 6 | 63 | 1 | 16 | 15 | 38 | 25 | 30 | 27 | 52 | 37 | 3 | 251 | 39 | 71 | 124 | 13 | 120 | 26 | 59 | 9 | 9 | 55 | 29 | 18 | 5 | 8 | 22 | 19 | Test outcome positive |
| TP | 25 | 32 | 6 | 59 | 1 | 9 | 15 | 33 | 9 | 20 | 21 | 46 | 24 | 3 | 234 | 11 | 60 | 84 | 6 | 115 | 23 | 51 | 4 | 8 | 48 | 14 | 14 | 5 | 7 | 5 | 11 | True positive/hit |
| TPR | 0.5 | 0.94118 | 0.85714 | 0.95161 | 0.16667 | 0.69231 | 0.9375 | 0.91667 | 0.19149 | 0.71429 | 0.84 | 0.86792 | 0.88889 | 0.42857 | 0.82394 | 0.28205 | 0.77922 | 0.78505 | 0.85714 | 0.96639 | 0.71875 | 0.85 | 0.57143 | 1.0 | 0.94118 | 0.875 | 1.0 | 1.0 | 0.875 | 0.12195 | 1.0 | Sensitivity, recall, hit rate, or true positive rate |
| Y | 0.46632 | 0.93484 | 0.85714 | 0.94837 | 0.16667 | 0.68686 | 0.9375 | 0.9127 | 0.17869 | 0.70641 | 0.83528 | 0.8631 | 0.87865 | 0.42857 | 0.80716 | 0.25979 | 0.7702 | 0.75143 | 0.85172 | 0.96214 | 0.71638 | 0.84353 | 0.56755 | 0.99922 | 0.93556 | 0.86329 | 0.99688 | 1.0 | 0.87422 | 0.10842 | 0.99378 | Youden index |
| dInd | 0.50113 | 0.05916 | 0.14286 | 0.0485 | 0.83333 | 0.30774 | 0.0625 | 0.08343 | 0.80861 | 0.28582 | 0.16007 | 0.13216 | 0.11158 | 0.57143 | 0.17685 | 0.71829 | 0.22096 | 0.21757 | 0.14296 | 0.03388 | 0.28126 | 0.15014 | 0.42859 | 0.00078 | 0.05909 | 0.12555 | 0.00312 | 0.0 | 0.125 | 0.87815 | 0.00622 | Distance index |
| sInd | 0.64565 | 0.95817 | 0.89898 | 0.96571 | 0.41074 | 0.78239 | 0.95581 | 0.94101 | 0.42823 | 0.79789 | 0.88681 | 0.90655 | 0.9211 | 0.59594 | 0.87495 | 0.49209 | 0.84376 | 0.84616 | 0.89891 | 0.97604 | 0.80112 | 0.89384 | 0.69694 | 0.99945 | 0.95822 | 0.91122 | 0.9978 | 1.0 | 0.91161 | 0.37905 | 0.9956 | Similarity index |

Generated By PyCM Version 3.4
