# Supplementary material for: TopEC: prediction of Enzyme Commission classes by 3D graph neural networks and localized 3D protein descriptor
Source: Nat Commun. 2025 Mar 20;16:2737. doi: 10.1038/s41467-025-57324-5 (PMC11923149; doi:10.1038/s41467-025-57324-5)
Supplement: Supplementary file 3 — Supplementary Data 1 [file 41467_2025_57324_MOESM3_ESM.zip › Data_S1/table1/hierarchical/TopEC_distance_FOLD_3digs.html]

PyCM Report


# PyCM Report

## Dataset Type :

- Multi-Class Classification
- Imbalanced

Note 1 : Recommended statistics for this type of classification highlighted in aqua

Note 2 : The recommender system assumes that the input is the result of classification over the whole data rather than just a part of it.
If the confusion matrix is the result of test data classification, the recommendation is not valid.

## Confusion Matrix :

|  |  |  |  |  |  |  |  |  |  |  |  |  |  |  |  |  |  |  |  |  |  |  |  |  |  |  |  |  |  |  |  |  |  |  |  |  |  |  |  |  |  |  |  |  |  |  |  |  |  |  |  |  |  |  |  |  |  |  |  |  |  |  |  |  |  |  |  |  |  |  |  |  |  |  |  |  |  |  |  |  |  |  |  |  |  |  |  |  |  |  |  |  |  |  |  |  |  |  |  |  |  |  |  |  |  |  |  |  |  |  |  |  |  |  |  |  |  |  |  |  |  |  |  |  |  |  |  |  |  |  |  |  |  |  |  |  |  |  |  |  |  |  |  |  |  |  |  |  |  |  |  |  |  |  |  |  |  |  |  |  |  |  |  |  |  |  |  |  |  |  |  |  |  |  |  |  |  |  |  |  |  |  |  |  |  |  |  |  |  |  |  |  |  |  |  |  |  |  |  |  |  |  |  |  |  |  |  |  |  |  |  |  |  |  |  |  |  |  |  |  |  |  |  |  |  |  |  |  |  |  |  |  |  |  |  |  |  |  |  |  |  |  |  |  |  |  |  |  |  |  |  |  |  |  |  |  |  |  |  |  |  |  |  |  |  |  |  |  |  |  |  |  |  |  |  |  |  |  |  |  |  |  |  |  |  |  |  |  |  |  |  |  |  |  |  |  |  |  |  |  |  |  |  |  |  |  |  |  |  |  |  |  |  |  |  |  |  |  |  |  |  |  |  |  |  |  |  |  |  |  |  |  |  |  |  |  |  |  |  |  |  |  |  |  |  |  |  |  |  |  |  |  |  |  |  |  |  |  |  |  |  |  |  |  |  |  |  |  |  |  |  |  |  |  |  |  |  |  |  |  |  |  |  |  |  |  |  |  |  |  |  |  |  |  |  |  |  |  |  |  |  |  |  |  |  |  |  |  |  |  |  |  |  |  |  |  |  |  |  |  |  |  |  |  |  |  |  |  |  |  |  |  |  |  |  |  |  |  |  |  |  |  |  |  |  |  |  |  |  |  |  |  |  |  |  |  |  |  |  |  |  |  |  |  |  |  |  |  |  |  |  |  |  |  |  |  |  |  |  |  |  |  |  |  |  |  |  |  |  |  |  |  |  |  |  |  |  |  |  |  |  |  |  |  |  |  |  |  |  |  |  |  |  |  |  |  |  |  |  |  |  |  |  |  |  |  |  |  |  |  |  |  |  |  |  |  |  |  |  |  |  |  |  |  |  |  |  |  |  |  |  |  |  |  |  |  |  |  |  |  |  |  |  |  |  |  |  |  |  |  |  |  |  |  |  |  |  |  |  |  |  |  |  |  |  |  |  |  |  |  |  |  |  |  |  |  |  |  |  |  |  |  |  |  |  |  |  |  |  |  |  |  |  |  |  |  |  |  |  |  |  |  |  |  |  |  |  |  |  |  |  |  |  |  |  |  |  |  |  |  |  |  |  |  |  |  |  |  |  |  |  |  |  |  |  |  |  |  |  |  |  |  |  |  |  |  |  |  |  |  |  |  |  |  |  |  |  |  |  |  |  |  |  |  |  |  |  |  |  |  |  |  |  |  |  |  |  |  |  |  |  |  |  |  |  |  |  |  |  |  |  |  |  |  |  |  |  |  |  |  |  |  |  |  |  |  |  |  |  |  |  |  |  |  |  |  |  |  |  |  |  |  |  |  |  |  |  |  |  |  |  |  |  |  |  |  |  |  |  |  |  |  |  |  |  |  |  |  |  |  |  |  |  |  |  |  |  |  |  |  |  |  |  |  |  |  |  |  |  |  |  |  |  |  |  |  |  |  |  |  |  |  |  |  |  |  |  |  |  |  |  |  |  |  |  |  |  |  |  |  |  |  |  |  |  |  |  |  |  |  |  |  |  |  |  |  |  |  |  |  |  |  |  |  |  |  |  |  |  |  |  |  |  |  |  |  |  |  |  |  |  |  |  |  |  |  |  |  |  |  |  |  |  |  |  |  |  |  |  |  |  |  |  |  |  |  |  |  |  |  |  |  |  |  |  |  |  |  |  |  |  |  |  |  |  |  |  |  |  |  |  |  |  |  |  |  |  |  |  |  |  |  |  |  |  |  |  |  |  |  |  |  |  |  |  |  |  |  |  |  |  |  |  |  |  |  |  |  |  |  |  |  |  |  |  |  |  |  |  |  |  |  |  |  |  |  |  |  |  |  |  |  |  |  |  |  |  |  |  |  |  |  |  |  |  |  |  |  |  |  |  |  |  |  |  |  |  |  |  |  |  |  |  |  |  |  |  |  |  |  |  |  |  |  |  |  |  |  |  |  |  |  |  |  |  |  |  |  |  |  |  |  |  |  |  |  |  |  |  |  |  |  |  |  |  |  |  |  |  |  |  |  |  |  |  |  |  |  |  |  |  |  |  |  |  |  |  |  |  |  |  |  |  |  |  |  |  |  |  |  |  |  |  |  |  |  |  |  |  |  |  |  |  |  |  |  |  |  |  |  |  |  |  |  |  |  |  |  |  |  |  |  |  |  |  |  |  |  |  |  |  |  |  |  |  |  |  |  |  |  |  |  |  |  |  |  |  |  |  |  |  |  |  |  |  |  |  |  |  |  |  |  |  |  |  |  |  |  |  |  |  |  |  |  |  |  |  |  |  |  |  |  |  |  |  |  |  |  |  |  |  |  |  |  |  |  |  |  |  |  |  |  |  |  |  |  |  |  |  |  |  |  |  |  |  |  |  |  |  |  |  |  |  |  |  |  |  |  |  |  |  |  |  |  |  |  |  |  |  |  |  |  |  |  |  |  |  |  |  |  |  |  |  |  |  |  |  |  |  |  |  |  |  |  |  |  |  |  |  |  |  |  |  |  |  |  |  |  |  |  |  |  |  |  |  |  |  |  |  |  |  |  |  |  |  |  |  |  |  |  |  |  |  |  |  |  |  |  |  |  |  |  |  |  |  |  |  |  |  |  |  |  |  |  |  |  |  |  |  |  |  |  |  |  |  |  |  |  |  |  |  |  |  |  |  |  |  |  |  |  |  |  |  |  |  |  |  |  |  |  |  |  |  |  |  |  |  |  |  |  |  |  |  |  |  |  |  |  |  |  |  |  |  |  |  |  |  |  |  |  |  |  |  |  |  |  |  |  |  |  |  |  |  |  |  |  |  |  |  |  |  |  |  |  |  |  |  |  |  |  |  |  |  |  |  |  |  |  |  |  |  |  |  |  |  |  |  |  |  |  |  |  |  |  |  |  |  |  |  |  |  |  |  |  |  |  |  |  |  |  |  |  |  |  |  |  |  |  |  |  |  |  |  |  |  |  |  |  |  |  |  |  |  |  |  |  |  |  |  |  |  |  |  |  |  |  |  |  |  |  |  |  |  |  |  |  |  |  |  |  |  |  |  |  |  |  |  |  |  |  |  |  |  |  |  |  |  |  |  |  |  |  |  |  |  |  |  |  |  |  |  |  |  |  |  |  |  |  |  |  |  |  |  |  |  |  |  |  |  |  |  |  |  |  |  |  |  |  |  |  |  |  |  |  |  |  |  |  |  |  |  |  |  |  |  |  |  |  |  |  |  |  |  |  |  |  |  |  |  |  |  |  |  |  |  |  |  |  |  |  |  |  |  |  |  |  |  |  |  |  |  |  |  |  |  |  |  |  |  |  |  |  |  |  |  |  |  |  |  |  |  |  |  |  |  |  |  |  |  |  |  |  |  |  |  |  |  |  |  |  |  |  |  |  |  |  |  |  |  |  |  |  |  |  |  |  |  |  |  |  |  |  |  |  |  |  |  |  |  |  |  |  |  |  |  |  |  |  |  |  |  |  |  |  |  |  |  |  |  |  |  |  |  |  |  |  |  |  |  |  |  |  |  |  |  |  |  |  |  |  |  |  |  |  |  |  |  |  |  |  |  |  |  |  |  |  |  |  |  |  |  |  |  |  |  |  |  |  |  |  |  |  |  |  |  |  |  |  |  |  |  |  |  |  |  |  |  |  |  |  |  |  |  |  |  |  |  |  |  |  |  |  |  |  |  |  |  |  |  |  |  |  |  |  |  |  |  |  |  |  |  |  |  |  |  |  |  |  |  |  |  |  |  |  |  |  |  |  |  |  |  |  |  |  |  |  |  |  |  |  |  |  |  |  |  |  |  |  |  |  |  |  |  |  |  |  |  |  |  |  |  |  |  |  |  |  |  |  |  |  |  |  |  |  |  |  |  |  |  |  |  |  |  |  |  |  |  |  |  |  |  |  |  |  |  |  |  |  |  |  |  |  |  |  |  |  |  |  |  |  |  |  |  |  |  |  |  |  |  |  |  |  |  |  |  |  |  |  |  |  |  |  |  |  |  |  |  |  |  |  |  |  |  |  |  |  |  |  |  |  |  |  |  |  |  |  |  |  |  |  |  |  |  |  |  |  |  |  |  |  |  |  |  |  |  |  |  |  |  |  |  |  |  |  |  |  |  |  |  |  |  |  |  |  |  |  |  |  |  |  |  |  |  |  |  |  |  |  |  |  |  |  |  |  |  |  |  |  |  |  |  |  |  |  |  |  |  |  |  |  |  |  |  |  |  |  |  |  |  |  |  |  |  |  |  |  |  |  |  |  |  |  |  |  |  |  |  |  |  |  |  |  |  |  |  |  |  |  |  |  |  |  |  |  |  |  |  |  |  |  |  |  |  |  |  |  |  |  |  |  |  |  |  |  |  |  |  |  |  |  |  |  |  |  |  |  |  |  |  |  |  |  |  |  |  |  |  |  |  |  |  |  |  |  |  |  |  |  |  |  |  |  |  |  |  |  |  |  |  |  |  |  |  |  |  |  |  |  |  |  |  |  |  |  |  |  |  |  |  |  |  |  |  |  |  |  |  |  |  |  |  |  |  |  |  |  |  |  |  |  |  |  |  |  |  |  |  |  |  |  |  |  |  |  |  |  |  |  |  |  |  |  |  |  |  |  |  |  |  |  |  |  |  |  |  |  |  |  |  |  |  |  |  |  |  |  |  |  |  |  |  |  |  |  |  |  |  |  |  |  |  |  |  |  |  |  |  |  |  |  |  |  |  |  |  |  |  |  |  |  |  |  |  |  |  |  |  |  |  |  |  |  |  |  |  |  |  |  |  |  |  |  |  |  |  |  |  |  |  |  |  |  |  |  |  |  |  |  |  |  |  |  |  |  |  |  |  |  |  |  |  |  |  |  |  |  |  |  |  |  |  |  |  |  |  |  |  |  |  |  |  |  |  |  |  |  |  |  |  |  |  |  |  |  |  |  |  |  |  |  |  |  |  |  |  |  |  |  |  |  |  |  |  |  |  |  |  |  |  |  |  |  |  |  |  |  |  |  |  |  |  |  |  |  |  |  |  |  |  |  |  |  |  |  |  |  |  |  |  |  |  |  |  |  |  |  |  |  |  |  |  |  |  |  |  |  |  |  |  |  |  |  |  |  |  |  |  |  |  |  |  |  |  |  |  |  |  |  |  |  |  |  |  |  |  |  |  |  |  |  |  |  |  |  |  |  |  |  |  |  |  |  |  |  |  |  |  |  |  |  |  |  |  |  |  |  |  |  |  |  |  |  |  |  |  |  |  |  |  |  |  |  |  |  |  |  |  |  |  |  |  |  |  |  |  |  |  |  |  |  |  |  |  |  |  |  |  |  |  |  |  |  |  |  |  |  |  |  |  |  |  |  |  |  |  |  |  |  |  |  |  |  |  |  |  |  |  |  |  |  |  |  |  |  |  |  |  |  |  |  |  |  |  |  |  |  |  |  |  |  |  |  |  |  |  |  |  |  |  |  |  |  |  |  |  |  |  |  |  |  |  |  |  |  |  |  |  |  |  |  |  |  |  |  |  |  |  |  |  |  |  |  |  |  |  |  |  |  |  |  |  |  |  |  |  |  |  |  |  |  |  |  |  |  |  |  |  |  |  |  |  |  |  |  |  |  |  |  |  |  |  |  |  |  |  |  |  |  |  |  |  |  |  |  |  |  |  |  |  |  |  |  |  |  |  |  |  |  |  |  |  |  |  |  |  |  |  |  |  |  |  |  |  |  |  |  |  |  |  |  |  |  |  |  |  |  |  |  |  |  |  |  |  |  |  |  |  |  |  |  |  |  |  |  |  |  |  |  |  |  |  |  |  |  |  |  |  |  |  |  |  |  |  |  |  |  |  |  |  |  |  |  |  |  |  |  |  |  |  |  |  |  |  |  |  |  |  |  |  |  |  |  |  |  |  |  |  |  |  |  |  |  |  |  |  |  |  |  |  |  |  |  |  |  |  |  |  |  |  |  |  |  |  |  |  |  |  |  |  |  |  |  |  |  |  |  |  |  |  |  |
| --- | --- | --- | --- | --- | --- | --- | --- | --- | --- | --- | --- | --- | --- | --- | --- | --- | --- | --- | --- | --- | --- | --- | --- | --- | --- | --- | --- | --- | --- | --- | --- | --- | --- | --- | --- | --- | --- | --- | --- | --- | --- | --- | --- | --- | --- | --- | --- | --- | --- | --- | --- | --- | --- | --- | --- | --- | --- | --- | --- | --- | --- | --- | --- | --- | --- | --- | --- | --- | --- | --- | --- | --- | --- | --- | --- | --- | --- | --- | --- | --- | --- | --- | --- | --- | --- | --- | --- | --- | --- | --- | --- | --- | --- | --- | --- | --- | --- | --- | --- | --- | --- | --- | --- | --- | --- | --- | --- | --- | --- | --- | --- | --- | --- | --- | --- | --- | --- | --- | --- | --- | --- | --- | --- | --- | --- | --- | --- | --- | --- | --- | --- | --- | --- | --- | --- | --- | --- | --- | --- | --- | --- | --- | --- | --- | --- | --- | --- | --- | --- | --- | --- | --- | --- | --- | --- | --- | --- | --- | --- | --- | --- | --- | --- | --- | --- | --- | --- | --- | --- | --- | --- | --- | --- | --- | --- | --- | --- | --- | --- | --- | --- | --- | --- | --- | --- | --- | --- | --- | --- | --- | --- | --- | --- | --- | --- | --- | --- | --- | --- | --- | --- | --- | --- | --- | --- | --- | --- | --- | --- | --- | --- | --- | --- | --- | --- | --- | --- | --- | --- | --- | --- | --- | --- | --- | --- | --- | --- | --- | --- | --- | --- | --- | --- | --- | --- | --- | --- | --- | --- | --- | --- | --- | --- | --- | --- | --- | --- | --- | --- | --- | --- | --- | --- | --- | --- | --- | --- | --- | --- | --- | --- | --- | --- | --- | --- | --- | --- | --- | --- | --- | --- | --- | --- | --- | --- | --- | --- | --- | --- | --- | --- | --- | --- | --- | --- | --- | --- | --- | --- | --- | --- | --- | --- | --- | --- | --- | --- | --- | --- | --- | --- | --- | --- | --- | --- | --- | --- | --- | --- | --- | --- | --- | --- | --- | --- | --- | --- | --- | --- | --- | --- | --- | --- | --- | --- | --- | --- | --- | --- | --- | --- | --- | --- | --- | --- | --- | --- | --- | --- | --- | --- | --- | --- | --- | --- | --- | --- | --- | --- | --- | --- | --- | --- | --- | --- | --- | --- | --- | --- | --- | --- | --- | --- | --- | --- | --- | --- | --- | --- | --- | --- | --- | --- | --- | --- | --- | --- | --- | --- | --- | --- | --- | --- | --- | --- | --- | --- | --- | --- | --- | --- | --- | --- | --- | --- | --- | --- | --- | --- | --- | --- | --- | --- | --- | --- | --- | --- | --- | --- | --- | --- | --- | --- | --- | --- | --- | --- | --- | --- | --- | --- | --- | --- | --- | --- | --- | --- | --- | --- | --- | --- | --- | --- | --- | --- | --- | --- | --- | --- | --- | --- | --- | --- | --- | --- | --- | --- | --- | --- | --- | --- | --- | --- | --- | --- | --- | --- | --- | --- | --- | --- | --- | --- | --- | --- | --- | --- | --- | --- | --- | --- | --- | --- | --- | --- | --- | --- | --- | --- | --- | --- | --- | --- | --- | --- | --- | --- | --- | --- | --- | --- | --- | --- | --- | --- | --- | --- | --- | --- | --- | --- | --- | --- | --- | --- | --- | --- | --- | --- | --- | --- | --- | --- | --- | --- | --- | --- | --- | --- | --- | --- | --- | --- | --- | --- | --- | --- | --- | --- | --- | --- | --- | --- | --- | --- | --- | --- | --- | --- | --- | --- | --- | --- | --- | --- | --- | --- | --- | --- | --- | --- | --- | --- | --- | --- | --- | --- | --- | --- | --- | --- | --- | --- | --- | --- | --- | --- | --- | --- | --- | --- | --- | --- | --- | --- | --- | --- | --- | --- | --- | --- | --- | --- | --- | --- | --- | --- | --- | --- | --- | --- | --- | --- | --- | --- | --- | --- | --- | --- | --- | --- | --- | --- | --- | --- | --- | --- | --- | --- | --- | --- | --- | --- | --- | --- | --- | --- | --- | --- | --- | --- | --- | --- | --- | --- | --- | --- | --- | --- | --- | --- | --- | --- | --- | --- | --- | --- | --- | --- | --- | --- | --- | --- | --- | --- | --- | --- | --- | --- | --- | --- | --- | --- | --- | --- | --- | --- | --- | --- | --- | --- | --- | --- | --- | --- | --- | --- | --- | --- | --- | --- | --- | --- | --- | --- | --- | --- | --- | --- | --- | --- | --- | --- | --- | --- | --- | --- | --- | --- | --- | --- | --- | --- | --- | --- | --- | --- | --- | --- | --- | --- | --- | --- | --- | --- | --- | --- | --- | --- | --- | --- | --- | --- | --- | --- | --- | --- | --- | --- | --- | --- | --- | --- | --- | --- | --- | --- | --- | --- | --- | --- | --- | --- | --- | --- | --- | --- | --- | --- | --- | --- | --- | --- | --- | --- | --- | --- | --- | --- | --- | --- | --- | --- | --- | --- | --- | --- | --- | --- | --- | --- | --- | --- | --- | --- | --- | --- | --- | --- | --- | --- | --- | --- | --- | --- | --- | --- | --- | --- | --- | --- | --- | --- | --- | --- | --- | --- | --- | --- | --- | --- | --- | --- | --- | --- | --- | --- | --- | --- | --- | --- | --- | --- | --- | --- | --- | --- | --- | --- | --- | --- | --- | --- | --- | --- | --- | --- | --- | --- | --- | --- | --- | --- | --- | --- | --- | --- | --- | --- | --- | --- | --- | --- | --- | --- | --- | --- | --- | --- | --- | --- | --- | --- | --- | --- | --- | --- | --- | --- | --- | --- | --- | --- | --- | --- | --- | --- | --- | --- | --- | --- | --- | --- | --- | --- | --- | --- | --- | --- | --- | --- | --- | --- | --- | --- | --- | --- | --- | --- | --- | --- | --- | --- | --- | --- | --- | --- | --- | --- | --- | --- | --- | --- | --- | --- | --- | --- | --- | --- | --- | --- | --- | --- | --- | --- | --- | --- | --- | --- | --- | --- | --- | --- | --- | --- | --- | --- | --- | --- | --- | --- | --- | --- | --- | --- | --- | --- | --- | --- | --- | --- | --- | --- | --- | --- | --- | --- | --- | --- | --- | --- | --- | --- | --- | --- | --- | --- | --- | --- | --- | --- | --- | --- | --- | --- | --- | --- | --- | --- | --- | --- | --- | --- | --- | --- | --- | --- | --- | --- | --- | --- | --- | --- | --- | --- | --- | --- | --- | --- | --- | --- | --- | --- | --- | --- | --- | --- | --- | --- | --- | --- | --- | --- | --- | --- | --- | --- | --- | --- | --- | --- | --- | --- | --- | --- | --- | --- | --- | --- | --- | --- | --- | --- | --- | --- | --- | --- | --- | --- | --- | --- | --- | --- | --- | --- | --- | --- | --- | --- | --- | --- | --- | --- | --- | --- | --- | --- | --- | --- | --- | --- | --- | --- | --- | --- | --- | --- | --- | --- | --- | --- | --- | --- | --- | --- | --- | --- | --- | --- | --- | --- | --- | --- | --- | --- | --- | --- | --- | --- | --- | --- | --- | --- | --- | --- | --- | --- | --- | --- | --- | --- | --- | --- | --- | --- | --- | --- | --- | --- | --- | --- | --- | --- | --- | --- | --- | --- | --- | --- | --- | --- | --- | --- | --- | --- | --- | --- | --- | --- | --- | --- | --- | --- | --- | --- | --- | --- | --- | --- | --- | --- | --- | --- | --- | --- | --- | --- | --- | --- | --- | --- | --- | --- | --- | --- | --- | --- | --- | --- | --- | --- | --- | --- | --- | --- | --- | --- | --- | --- | --- | --- | --- | --- | --- | --- | --- | --- | --- | --- | --- | --- | --- | --- | --- | --- | --- | --- | --- | --- | --- | --- | --- | --- | --- | --- | --- | --- | --- | --- | --- | --- | --- | --- | --- | --- | --- | --- | --- | --- | --- | --- | --- | --- | --- | --- | --- | --- | --- | --- | --- | --- | --- | --- | --- | --- | --- | --- | --- | --- | --- | --- | --- | --- | --- | --- | --- | --- | --- | --- | --- | --- | --- | --- | --- | --- | --- | --- | --- | --- | --- | --- | --- | --- | --- | --- | --- | --- | --- | --- | --- | --- | --- | --- | --- | --- | --- | --- | --- | --- | --- | --- | --- | --- | --- | --- | --- | --- | --- | --- | --- | --- | --- | --- | --- | --- | --- | --- | --- | --- | --- | --- | --- | --- | --- | --- | --- | --- | --- | --- | --- | --- | --- | --- | --- | --- | --- | --- | --- | --- | --- | --- | --- | --- | --- | --- | --- | --- | --- | --- | --- | --- | --- | --- | --- | --- | --- | --- | --- | --- | --- | --- | --- | --- | --- | --- | --- | --- | --- | --- | --- | --- | --- | --- | --- | --- | --- | --- | --- | --- | --- | --- | --- | --- | --- | --- | --- | --- | --- | --- | --- | --- | --- | --- | --- | --- | --- | --- | --- | --- | --- | --- | --- | --- | --- | --- | --- | --- | --- | --- | --- | --- | --- | --- | --- | --- | --- | --- | --- | --- | --- | --- | --- | --- | --- | --- | --- | --- | --- | --- | --- | --- | --- | --- | --- | --- | --- | --- | --- | --- | --- | --- | --- | --- | --- | --- | --- | --- | --- | --- | --- | --- | --- | --- | --- | --- | --- | --- | --- | --- | --- | --- | --- | --- | --- | --- | --- | --- | --- | --- | --- | --- | --- | --- | --- | --- | --- | --- | --- | --- | --- | --- | --- | --- | --- | --- | --- | --- | --- | --- | --- | --- | --- | --- | --- | --- | --- | --- | --- | --- | --- | --- | --- | --- | --- | --- | --- | --- | --- | --- | --- | --- | --- | --- | --- | --- | --- | --- | --- | --- | --- | --- | --- | --- | --- | --- | --- | --- | --- | --- | --- | --- | --- | --- | --- | --- | --- | --- | --- | --- | --- | --- | --- | --- | --- | --- | --- | --- | --- | --- | --- | --- | --- | --- | --- | --- | --- | --- | --- | --- | --- | --- | --- | --- | --- | --- | --- | --- | --- | --- | --- | --- | --- | --- | --- | --- | --- | --- | --- | --- | --- | --- | --- | --- | --- | --- | --- | --- | --- | --- | --- | --- | --- | --- | --- | --- | --- | --- | --- | --- | --- | --- | --- | --- | --- | --- | --- | --- | --- | --- | --- | --- | --- | --- | --- | --- | --- | --- | --- | --- | --- | --- | --- | --- | --- | --- | --- | --- | --- | --- | --- | --- | --- | --- | --- | --- | --- | --- | --- | --- | --- | --- | --- | --- | --- | --- | --- | --- | --- | --- | --- | --- | --- | --- | --- | --- | --- | --- | --- | --- | --- | --- | --- | --- | --- | --- | --- | --- | --- | --- | --- | --- | --- | --- | --- | --- | --- | --- | --- | --- | --- | --- | --- | --- | --- | --- | --- | --- | --- | --- | --- | --- | --- | --- | --- | --- | --- | --- | --- | --- | --- | --- | --- | --- | --- | --- | --- | --- | --- | --- | --- | --- | --- | --- | --- | --- | --- | --- | --- | --- | --- | --- | --- | --- | --- | --- | --- | --- | --- | --- | --- | --- | --- | --- | --- | --- | --- | --- | --- | --- | --- | --- | --- | --- | --- | --- | --- | --- | --- | --- | --- | --- | --- | --- | --- | --- | --- | --- | --- | --- | --- | --- | --- | --- | --- | --- | --- | --- | --- | --- | --- | --- | --- | --- | --- | --- | --- | --- | --- | --- | --- | --- | --- | --- | --- | --- | --- | --- | --- | --- | --- | --- | --- | --- | --- | --- | --- | --- | --- | --- | --- | --- | --- | --- | --- | --- | --- | --- | --- | --- | --- | --- | --- | --- | --- | --- | --- | --- | --- | --- | --- | --- | --- | --- | --- | --- | --- | --- | --- | --- | --- | --- | --- | --- | --- | --- | --- | --- | --- | --- | --- | --- | --- | --- | --- | --- | --- | --- | --- | --- | --- | --- | --- | --- | --- | --- | --- | --- | --- | --- | --- | --- | --- | --- | --- | --- | --- | --- | --- | --- | --- | --- | --- | --- | --- | --- | --- | --- | --- | --- | --- | --- | --- | --- | --- | --- | --- | --- | --- | --- | --- | --- | --- | --- | --- | --- | --- | --- | --- | --- | --- | --- | --- | --- | --- | --- | --- | --- | --- | --- | --- | --- | --- | --- | --- | --- | --- | --- | --- | --- | --- | --- | --- | --- | --- | --- | --- | --- | --- | --- | --- | --- | --- | --- | --- | --- | --- | --- | --- | --- | --- | --- | --- | --- | --- | --- | --- | --- | --- | --- | --- | --- | --- | --- | --- | --- | --- | --- | --- | --- | --- | --- | --- | --- | --- | --- | --- | --- | --- | --- | --- | --- | --- | --- | --- | --- | --- | --- | --- | --- | --- | --- | --- | --- | --- | --- | --- | --- | --- | --- | --- | --- | --- | --- | --- | --- | --- | --- | --- | --- | --- | --- | --- | --- | --- | --- | --- | --- | --- | --- | --- | --- | --- | --- | --- | --- | --- | --- | --- | --- | --- | --- | --- | --- | --- | --- | --- | --- | --- | --- | --- | --- | --- | --- | --- | --- | --- | --- | --- | --- | --- | --- | --- | --- | --- | --- | --- | --- | --- | --- | --- | --- | --- | --- | --- | --- | --- | --- | --- | --- | --- | --- | --- | --- | --- | --- | --- | --- | --- | --- | --- | --- | --- | --- | --- | --- | --- | --- | --- | --- | --- | --- | --- | --- | --- | --- | --- | --- | --- | --- | --- | --- | --- | --- | --- | --- | --- | --- | --- | --- | --- | --- | --- | --- | --- | --- | --- | --- | --- | --- | --- | --- | --- | --- | --- | --- | --- | --- | --- | --- | --- | --- | --- | --- | --- | --- | --- | --- | --- | --- | --- | --- | --- | --- | --- | --- | --- | --- | --- | --- | --- | --- | --- | --- | --- | --- | --- | --- | --- | --- | --- | --- | --- | --- | --- | --- | --- | --- | --- | --- | --- | --- | --- | --- | --- | --- | --- | --- | --- | --- | --- | --- | --- | --- | --- | --- | --- | --- | --- | --- | --- | --- | --- | --- | --- | --- | --- | --- | --- | --- | --- | --- | --- | --- | --- | --- | --- | --- | --- | --- | --- | --- | --- | --- | --- | --- | --- | --- | --- | --- | --- | --- | --- | --- | --- | --- | --- | --- | --- | --- | --- | --- | --- | --- | --- | --- | --- | --- | --- | --- | --- | --- | --- | --- | --- | --- | --- | --- | --- | --- | --- | --- | --- | --- | --- | --- | --- | --- | --- | --- | --- | --- | --- | --- | --- | --- | --- | --- | --- | --- | --- | --- | --- | --- | --- | --- | --- | --- | --- | --- | --- | --- | --- | --- | --- | --- | --- | --- | --- | --- | --- | --- | --- | --- | --- | --- | --- | --- | --- | --- | --- | --- | --- | --- | --- | --- | --- | --- | --- | --- | --- | --- | --- | --- | --- | --- | --- | --- | --- | --- | --- | --- | --- | --- | --- | --- | --- | --- | --- | --- | --- | --- | --- | --- | --- | --- | --- | --- | --- | --- | --- | --- | --- | --- | --- | --- | --- | --- | --- | --- | --- | --- | --- | --- | --- | --- | --- | --- | --- | --- | --- | --- | --- | --- | --- | --- | --- | --- | --- | --- | --- | --- | --- | --- | --- | --- | --- | --- | --- | --- | --- | --- | --- | --- | --- | --- | --- | --- | --- | --- | --- | --- | --- | --- | --- | --- | --- | --- | --- | --- | --- | --- | --- | --- | --- | --- | --- | --- | --- | --- | --- | --- | --- | --- | --- | --- | --- | --- | --- | --- | --- | --- | --- | --- | --- | --- | --- | --- | --- | --- | --- | --- | --- | --- | --- | --- | --- | --- | --- | --- | --- | --- | --- | --- | --- | --- | --- | --- | --- | --- | --- | --- | --- | --- | --- | --- | --- | --- | --- | --- | --- | --- | --- | --- | --- | --- | --- | --- | --- | --- | --- | --- | --- | --- | --- | --- | --- | --- | --- | --- | --- | --- | --- | --- | --- | --- | --- | --- | --- | --- | --- | --- | --- | --- | --- | --- | --- | --- | --- | --- | --- | --- | --- | --- | --- | --- | --- | --- | --- | --- | --- | --- | --- | --- | --- | --- | --- | --- | --- | --- | --- | --- | --- | --- | --- | --- | --- | --- | --- | --- | --- | --- | --- | --- | --- | --- | --- | --- | --- | --- | --- | --- | --- | --- | --- | --- | --- | --- | --- | --- | --- | --- | --- | --- | --- | --- | --- | --- | --- | --- | --- | --- | --- | --- | --- | --- | --- | --- | --- | --- | --- | --- | --- | --- | --- | --- | --- | --- | --- | --- | --- | --- | --- | --- | --- | --- | --- | --- | --- | --- | --- | --- | --- | --- | --- | --- | --- | --- | --- | --- | --- | --- | --- | --- | --- | --- | --- | --- | --- | --- | --- | --- | --- | --- | --- | --- | --- | --- | --- | --- | --- | --- | --- | --- | --- | --- | --- | --- | --- | --- | --- | --- | --- | --- | --- | --- | --- | --- | --- | --- | --- | --- | --- | --- | --- | --- | --- | --- | --- | --- | --- | --- | --- | --- | --- | --- | --- | --- | --- | --- | --- | --- | --- | --- | --- | --- | --- | --- | --- | --- | --- | --- | --- | --- | --- | --- | --- | --- | --- | --- | --- | --- | --- | --- | --- | --- | --- | --- | --- | --- | --- | --- | --- | --- | --- | --- | --- | --- | --- | --- | --- | --- | --- | --- | --- | --- | --- | --- | --- | --- | --- | --- | --- | --- | --- | --- | --- | --- | --- | --- | --- | --- | --- | --- | --- | --- | --- | --- | --- | --- | --- | --- | --- | --- | --- | --- | --- | --- | --- | --- | --- | --- | --- | --- | --- | --- | --- | --- | --- | --- | --- | --- | --- | --- | --- | --- | --- | --- | --- | --- | --- | --- | --- | --- | --- | --- | --- | --- | --- | --- | --- | --- | --- | --- | --- | --- | --- | --- | --- | --- | --- | --- | --- | --- | --- | --- | --- | --- | --- | --- | --- | --- | --- | --- | --- | --- | --- | --- | --- | --- | --- | --- | --- | --- | --- | --- | --- | --- | --- | --- | --- | --- | --- | --- | --- | --- | --- | --- | --- | --- | --- | --- | --- | --- | --- | --- | --- | --- | --- | --- | --- | --- | --- | --- | --- | --- | --- | --- | --- | --- | --- | --- | --- | --- | --- | --- | --- | --- | --- | --- | --- | --- | --- | --- | --- | --- | --- | --- | --- | --- | --- | --- | --- | --- | --- | --- | --- | --- | --- | --- | --- |
| Actual | Predict  |  |  |  |  |  |  |  |  |  |  |  |  |  |  |  |  |  |  |  |  |  |  |  |  |  |  |  |  |  |  |  |  |  |  |  |  |  |  |  |  |  |  |  |  |  |  |  |  |  |  |  |  |  | | --- | --- | --- | --- | --- | --- | --- | --- | --- | --- | --- | --- | --- | --- | --- | --- | --- | --- | --- | --- | --- | --- | --- | --- | --- | --- | --- | --- | --- | --- | --- | --- | --- | --- | --- | --- | --- | --- | --- | --- | --- | --- | --- | --- | --- | --- | --- | --- | --- | --- | --- | --- | --- | |  | 0 | 1 | 2 | 3 | 4 | 5 | 6 | 7 | 8 | 9 | 10 | 11 | 12 | 13 | 14 | 15 | 16 | 17 | 18 | 19 | 20 | 21 | 22 | 23 | 24 | 25 | 26 | 27 | 28 | 29 | 30 | 31 | 32 | 33 | 34 | 35 | 36 | 37 | 38 | 39 | 40 | 41 | 42 | 43 | 44 | 45 | 46 | 47 | 48 | 49 | 50 | 51 | | 0 | 4 | 3 | 0 | 0 | 0 | 0 | 0 | 0 | 0 | 0 | 0 | 0 | 16 | 0 | 0 | 0 | 2 | 0 | 0 | 0 | 0 | 0 | 2 | 0 | 0 | 0 | 0 | 0 | 0 | 0 | 0 | 0 | 0 | 0 | 0 | 0 | 0 | 0 | 1 | 0 | 0 | 0 | 0 | 0 | 0 | 0 | 0 | 0 | 0 | 0 | 1 | 0 | | 1 | 4 | 19 | 0 | 0 | 0 | 0 | 0 | 0 | 0 | 0 | 0 | 0 | 3 | 0 | 0 | 0 | 0 | 0 | 0 | 0 | 0 | 0 | 0 | 0 | 0 | 0 | 0 | 0 | 1 | 0 | 0 | 0 | 0 | 0 | 0 | 0 | 0 | 1 | 0 | 0 | 0 | 0 | 0 | 0 | 0 | 0 | 0 | 0 | 0 | 0 | 1 | 0 | | 2 | 2 | 0 | 0 | 0 | 0 | 0 | 0 | 0 | 0 | 0 | 0 | 0 | 1 | 0 | 0 | 0 | 0 | 0 | 0 | 0 | 0 | 0 | 0 | 0 | 0 | 0 | 0 | 0 | 0 | 0 | 0 | 0 | 0 | 0 | 0 | 0 | 0 | 0 | 1 | 0 | 0 | 0 | 0 | 0 | 0 | 0 | 0 | 0 | 0 | 0 | 0 | 0 | | 3 | 0 | 0 | 0 | 3 | 0 | 0 | 0 | 0 | 0 | 0 | 0 | 0 | 1 | 2 | 0 | 0 | 0 | 0 | 0 | 0 | 0 | 0 | 0 | 0 | 0 | 0 | 0 | 0 | 0 | 0 | 0 | 0 | 0 | 0 | 0 | 0 | 0 | 0 | 0 | 0 | 0 | 0 | 0 | 0 | 0 | 0 | 0 | 0 | 0 | 0 | 2 | 0 | | 4 | 1 | 0 | 0 | 0 | 8 | 0 | 0 | 0 | 0 | 0 | 0 | 0 | 0 | 0 | 0 | 0 | 0 | 0 | 0 | 0 | 0 | 0 | 0 | 0 | 0 | 0 | 0 | 0 | 0 | 0 | 0 | 0 | 0 | 0 | 0 | 0 | 0 | 0 | 0 | 0 | 0 | 0 | 0 | 0 | 0 | 0 | 0 | 0 | 0 | 0 | 0 | 0 | | 5 | 0 | 0 | 0 | 0 | 0 | 1 | 0 | 0 | 0 | 0 | 0 | 0 | 0 | 0 | 0 | 0 | 2 | 0 | 0 | 0 | 0 | 0 | 2 | 0 | 0 | 0 | 1 | 0 | 0 | 0 | 0 | 0 | 0 | 0 | 0 | 0 | 0 | 0 | 0 | 0 | 0 | 0 | 0 | 0 | 0 | 0 | 0 | 0 | 0 | 0 | 0 | 0 | | 6 | 2 | 0 | 0 | 0 | 0 | 0 | 0 | 0 | 1 | 0 | 0 | 0 | 0 | 0 | 0 | 1 | 0 | 0 | 0 | 0 | 0 | 0 | 0 | 0 | 0 | 0 | 0 | 0 | 0 | 0 | 0 | 0 | 0 | 0 | 0 | 0 | 0 | 0 | 0 | 0 | 0 | 1 | 0 | 0 | 0 | 0 | 1 | 0 | 0 | 0 | 1 | 0 | | 7 | 0 | 0 | 0 | 0 | 0 | 0 | 0 | 1 | 0 | 0 | 0 | 0 | 0 | 0 | 0 | 0 | 0 | 0 | 0 | 0 | 0 | 0 | 0 | 1 | 0 | 0 | 0 | 0 | 0 | 0 | 0 | 0 | 0 | 0 | 0 | 0 | 0 | 0 | 1 | 0 | 1 | 0 | 0 | 0 | 0 | 0 | 0 | 0 | 0 | 0 | 0 | 0 | | 8 | 0 | 0 | 0 | 0 | 0 | 0 | 0 | 0 | 17 | 0 | 0 | 0 | 1 | 0 | 0 | 0 | 0 | 0 | 0 | 0 | 0 | 0 | 0 | 0 | 0 | 0 | 0 | 0 | 0 | 0 | 0 | 0 | 0 | 0 | 0 | 0 | 0 | 0 | 0 | 0 | 0 | 0 | 0 | 0 | 0 | 0 | 0 | 0 | 0 | 0 | 0 | 0 | | 9 | 0 | 0 | 0 | 0 | 0 | 0 | 0 | 0 | 0 | 5 | 0 | 0 | 1 | 0 | 0 | 0 | 0 | 0 | 0 | 0 | 0 | 0 | 0 | 2 | 0 | 0 | 0 | 0 | 0 | 0 | 0 | 0 | 0 | 0 | 0 | 0 | 0 | 0 | 0 | 0 | 0 | 0 | 0 | 0 | 0 | 0 | 0 | 0 | 0 | 0 | 0 | 1 | | 10 | 2 | 0 | 0 | 0 | 0 | 0 | 0 | 0 | 0 | 0 | 31 | 0 | 3 | 0 | 0 | 0 | 0 | 0 | 0 | 0 | 0 | 0 | 0 | 0 | 0 | 0 | 0 | 0 | 0 | 0 | 0 | 0 | 0 | 0 | 0 | 0 | 0 | 0 | 0 | 0 | 0 | 0 | 0 | 0 | 0 | 0 | 0 | 0 | 0 | 0 | 0 | 0 | | 11 | 0 | 0 | 0 | 0 | 0 | 0 | 0 | 0 | 0 | 0 | 0 | 2 | 5 | 3 | 0 | 0 | 0 | 0 | 0 | 0 | 0 | 0 | 2 | 0 | 0 | 0 | 0 | 0 | 0 | 0 | 0 | 0 | 0 | 0 | 0 | 0 | 0 | 0 | 0 | 0 | 0 | 0 | 0 | 0 | 0 | 0 | 0 | 0 | 0 | 0 | 11 | 0 | | 12 | 0 | 0 | 0 | 0 | 0 | 0 | 0 | 0 | 0 | 0 | 2 | 3 | 12 | 0 | 0 | 1 | 0 | 0 | 0 | 0 | 0 | 0 | 3 | 0 | 0 | 0 | 0 | 0 | 0 | 0 | 0 | 3 | 0 | 0 | 0 | 0 | 0 | 0 | 0 | 0 | 0 | 0 | 0 | 0 | 0 | 0 | 0 | 0 | 0 | 0 | 0 | 0 | | 13 | 0 | 0 | 0 | 0 | 0 | 0 | 0 | 0 | 0 | 0 | 0 | 0 | 0 | 26 | 0 | 0 | 0 | 0 | 0 | 0 | 0 | 0 | 1 | 0 | 0 | 0 | 0 | 0 | 0 | 0 | 0 | 0 | 0 | 0 | 0 | 0 | 0 | 0 | 0 | 0 | 0 | 0 | 0 | 0 | 0 | 0 | 0 | 0 | 0 | 0 | 0 | 0 | | 14 | 0 | 0 | 0 | 0 | 0 | 2 | 0 | 0 | 0 | 0 | 0 | 0 | 0 | 0 | 29 | 0 | 0 | 0 | 0 | 0 | 0 | 0 | 0 | 0 | 0 | 0 | 0 | 0 | 0 | 0 | 0 | 0 | 0 | 0 | 0 | 0 | 0 | 0 | 0 | 0 | 0 | 0 | 0 | 0 | 0 | 0 | 0 | 0 | 0 | 0 | 0 | 0 | | 15 | 1 | 0 | 0 | 0 | 0 | 1 | 0 | 0 | 0 | 0 | 0 | 0 | 2 | 0 | 0 | 4 | 0 | 0 | 0 | 0 | 0 | 0 | 0 | 0 | 0 | 0 | 0 | 1 | 0 | 0 | 0 | 0 | 0 | 0 | 0 | 0 | 0 | 0 | 0 | 0 | 0 | 0 | 0 | 0 | 0 | 0 | 0 | 0 | 0 | 0 | 2 | 0 | | 16 | 2 | 0 | 0 | 0 | 0 | 0 | 0 | 0 | 0 | 0 | 0 | 9 | 0 | 0 | 0 | 0 | 6 | 0 | 0 | 0 | 0 | 0 | 1 | 0 | 0 | 0 | 0 | 0 | 0 | 0 | 0 | 0 | 0 | 0 | 0 | 0 | 0 | 0 | 0 | 1 | 0 | 1 | 0 | 0 | 0 | 0 | 0 | 0 | 0 | 0 | 1 | 0 | | 17 | 1 | 0 | 0 | 0 | 0 | 0 | 0 | 0 | 0 | 0 | 0 | 1 | 0 | 0 | 0 | 0 | 0 | 1 | 0 | 0 | 0 | 0 | 0 | 0 | 0 | 0 | 0 | 0 | 0 | 0 | 0 | 0 | 0 | 0 | 0 | 0 | 0 | 0 | 0 | 0 | 0 | 0 | 0 | 0 | 0 | 0 | 0 | 0 | 0 | 0 | 0 | 0 | | 18 | 2 | 1 | 0 | 0 | 0 | 0 | 0 | 0 | 0 | 0 | 0 | 0 | 0 | 0 | 0 | 0 | 1 | 0 | 1 | 0 | 0 | 0 | 0 | 0 | 1 | 0 | 0 | 0 | 0 | 0 | 0 | 0 | 0 | 0 | 0 | 0 | 0 | 2 | 0 | 0 | 0 | 0 | 0 | 0 | 0 | 0 | 0 | 0 | 0 | 0 | 1 | 0 | | 19 | 0 | 0 | 0 | 0 | 0 | 0 | 0 | 0 | 0 | 0 | 0 | 7 | 3 | 0 | 0 | 1 | 0 | 0 | 3 | 30 | 9 | 0 | 0 | 0 | 0 | 0 | 0 | 0 | 0 | 0 | 0 | 0 | 0 | 0 | 0 | 0 | 0 | 0 | 0 | 0 | 0 | 0 | 0 | 0 | 0 | 0 | 0 | 0 | 0 | 0 | 0 | 0 | | 20 | 3 | 0 | 0 | 0 | 0 | 0 | 0 | 0 | 0 | 0 | 0 | 0 | 0 | 2 | 0 | 0 | 0 | 0 | 0 | 0 | 135 | 0 | 0 | 0 | 0 | 0 | 0 | 0 | 0 | 0 | 0 | 0 | 0 | 0 | 0 | 0 | 0 | 0 | 0 | 0 | 0 | 0 | 0 | 0 | 0 | 0 | 0 | 0 | 0 | 0 | 1 | 0 | | 21 | 0 | 0 | 0 | 0 | 0 | 0 | 0 | 0 | 0 | 0 | 0 | 0 | 1 | 0 | 0 | 0 | 0 | 0 | 0 | 0 | 0 | 11 | 0 | 0 | 0 | 0 | 0 | 0 | 0 | 0 | 0 | 0 | 0 | 0 | 0 | 0 | 0 | 0 | 3 | 0 | 0 | 0 | 0 | 0 | 0 | 0 | 0 | 0 | 0 | 0 | 0 | 0 | | 22 | 1 | 0 | 0 | 0 | 0 | 0 | 0 | 0 | 0 | 0 | 0 | 3 | 0 | 0 | 0 | 0 | 0 | 0 | 0 | 0 | 0 | 0 | 30 | 0 | 0 | 0 | 1 | 0 | 2 | 0 | 0 | 0 | 0 | 0 | 1 | 0 | 0 | 0 | 0 | 0 | 0 | 0 | 0 | 0 | 0 | 0 | 0 | 0 | 0 | 0 | 5 | 0 | | 23 | 0 | 0 | 0 | 0 | 0 | 0 | 0 | 0 | 0 | 0 | 0 | 0 | 0 | 0 | 0 | 0 | 0 | 0 | 0 | 0 | 0 | 0 | 0 | 0 | 0 | 0 | 0 | 0 | 0 | 0 | 5 | 0 | 0 | 0 | 0 | 0 | 0 | 0 | 0 | 0 | 0 | 0 | 0 | 0 | 0 | 10 | 5 | 0 | 0 | 0 | 2 | 0 | | 24 | 0 | 0 | 0 | 0 | 0 | 0 | 0 | 0 | 0 | 0 | 0 | 0 | 0 | 0 | 0 | 0 | 0 | 0 | 0 | 0 | 0 | 0 | 0 | 0 | 6 | 0 | 0 | 0 | 1 | 0 | 0 | 0 | 0 | 0 | 0 | 0 | 0 | 0 | 0 | 0 | 0 | 0 | 0 | 0 | 0 | 0 | 0 | 0 | 0 | 0 | 0 | 0 | | 25 | 4 | 0 | 0 | 0 | 0 | 0 | 0 | 0 | 0 | 0 | 0 | 0 | 0 | 0 | 0 | 4 | 0 | 0 | 0 | 0 | 0 | 0 | 0 | 0 | 1 | 0 | 0 | 1 | 1 | 0 | 0 | 0 | 0 | 0 | 0 | 0 | 0 | 0 | 0 | 0 | 0 | 0 | 0 | 0 | 0 | 0 | 0 | 0 | 0 | 0 | 0 | 0 | | 26 | 0 | 0 | 0 | 0 | 0 | 0 | 0 | 0 | 0 | 0 | 0 | 0 | 0 | 0 | 0 | 0 | 0 | 0 | 0 | 1 | 0 | 0 | 1 | 0 | 0 | 0 | 12 | 2 | 2 | 0 | 0 | 1 | 0 | 1 | 0 | 0 | 0 | 0 | 0 | 0 | 0 | 0 | 0 | 0 | 0 | 0 | 0 | 0 | 0 | 0 | 4 | 0 | | 27 | 0 | 0 | 0 | 0 | 0 | 0 | 0 | 0 | 0 | 0 | 0 | 0 | 0 | 1 | 0 | 0 | 0 | 0 | 0 | 0 | 0 | 0 | 0 | 0 | 0 | 0 | 0 | 9 | 0 | 0 | 0 | 0 | 0 | 0 | 0 | 0 | 0 | 0 | 0 | 0 | 0 | 0 | 0 | 0 | 0 | 0 | 0 | 0 | 0 | 0 | 0 | 0 | | 28 | 2 | 0 | 0 | 0 | 0 | 0 | 0 | 0 | 1 | 0 | 0 | 0 | 1 | 1 | 1 | 0 | 2 | 0 | 0 | 0 | 0 | 1 | 2 | 4 | 0 | 1 | 0 | 1 | 44 | 0 | 1 | 0 | 5 | 0 | 0 | 0 | 0 | 0 | 2 | 0 | 0 | 0 | 0 | 0 | 0 | 0 | 0 | 0 | 0 | 0 | 5 | 0 | | 29 | 0 | 0 | 0 | 0 | 0 | 0 | 0 | 0 | 0 | 0 | 0 | 0 | 0 | 0 | 0 | 0 | 0 | 0 | 0 | 0 | 0 | 0 | 0 | 0 | 0 | 0 | 0 | 0 | 0 | 7 | 0 | 0 | 0 | 0 | 0 | 0 | 0 | 0 | 0 | 0 | 0 | 0 | 0 | 0 | 0 | 0 | 0 | 0 | 0 | 0 | 0 | 0 | | 30 | 0 | 0 | 0 | 0 | 0 | 0 | 0 | 0 | 0 | 0 | 0 | 1 | 0 | 0 | 0 | 0 | 0 | 0 | 0 | 0 | 0 | 0 | 0 | 0 | 0 | 0 | 0 | 0 | 0 | 0 | 3 | 0 | 0 | 0 | 0 | 0 | 0 | 0 | 0 | 0 | 0 | 0 | 0 | 0 | 0 | 0 | 0 | 0 | 0 | 0 | 0 | 0 | | 31 | 0 | 0 | 0 | 0 | 0 | 0 | 0 | 0 | 0 | 0 | 0 | 0 | 0 | 0 | 0 | 0 | 0 | 0 | 0 | 0 | 0 | 0 | 0 | 0 | 0 | 0 | 0 | 0 | 0 | 0 | 0 | 4 | 0 | 0 | 0 | 0 | 0 | 0 | 0 | 0 | 0 | 0 | 0 | 0 | 0 | 0 | 0 | 0 | 0 | 0 | 0 | 0 | | 32 | 0 | 0 | 0 | 0 | 0 | 0 | 0 | 0 | 0 | 0 | 0 | 0 | 0 | 0 | 0 | 0 | 0 | 0 | 0 | 0 | 0 | 0 | 0 | 0 | 0 | 0 | 0 | 0 | 0 | 0 | 0 | 0 | 3 | 0 | 0 | 0 | 0 | 0 | 0 | 0 | 0 | 0 | 0 | 0 | 0 | 0 | 0 | 0 | 0 | 0 | 0 | 0 | | 33 | 0 | 0 | 0 | 0 | 0 | 0 | 0 | 0 | 0 | 0 | 0 | 0 | 0 | 0 | 0 | 0 | 0 | 0 | 0 | 0 | 0 | 0 | 0 | 0 | 0 | 0 | 0 | 0 | 0 | 0 | 0 | 0 | 0 | 7 | 0 | 0 | 0 | 0 | 0 | 0 | 0 | 0 | 0 | 0 | 0 | 0 | 0 | 0 | 0 | 0 | 0 | 0 | | 34 | 0 | 0 | 0 | 0 | 0 | 0 | 0 | 0 | 0 | 0 | 0 | 0 | 0 | 0 | 0 | 0 | 0 | 0 | 0 | 0 | 0 | 0 | 0 | 0 | 0 | 0 | 0 | 0 | 0 | 0 | 0 | 0 | 0 | 0 | 2 | 0 | 0 | 0 | 0 | 0 | 0 | 0 | 0 | 0 | 0 | 0 | 0 | 0 | 0 | 0 | 0 | 0 | | 35 | 0 | 0 | 0 | 0 | 0 | 0 | 0 | 0 | 0 | 0 | 0 | 0 | 0 | 0 | 0 | 0 | 0 | 0 | 0 | 0 | 0 | 0 | 1 | 0 | 0 | 0 | 0 | 0 | 0 | 0 | 0 | 0 | 0 | 0 | 0 | 103 | 0 | 0 | 0 | 0 | 0 | 0 | 0 | 0 | 0 | 0 | 0 | 0 | 0 | 0 | 0 | 0 | | 36 | 0 | 0 | 0 | 0 | 0 | 0 | 0 | 0 | 0 | 0 | 0 | 0 | 0 | 0 | 0 | 0 | 0 | 0 | 0 | 0 | 0 | 0 | 1 | 0 | 0 | 0 | 0 | 0 | 0 | 0 | 0 | 0 | 0 | 0 | 0 | 0 | 6 | 0 | 0 | 0 | 0 | 0 | 0 | 0 | 0 | 0 | 0 | 0 | 0 | 0 | 0 | 0 | | 37 | 0 | 0 | 0 | 0 | 0 | 0 | 0 | 0 | 0 | 0 | 0 | 0 | 0 | 0 | 0 | 0 | 0 | 0 | 0 | 0 | 0 | 0 | 0 | 0 | 0 | 0 | 0 | 0 | 0 | 0 | 0 | 0 | 0 | 0 | 0 | 0 | 0 | 10 | 0 | 0 | 0 | 0 | 0 | 0 | 0 | 0 | 0 | 0 | 0 | 0 | 0 | 0 | | 38 | 3 | 0 | 0 | 0 | 0 | 2 | 0 | 0 | 0 | 0 | 2 | 0 | 1 | 0 | 0 | 0 | 1 | 1 | 0 | 2 | 0 | 0 | 1 | 2 | 2 | 0 | 0 | 0 | 1 | 0 | 0 | 0 | 0 | 0 | 0 | 0 | 0 | 0 | 2 | 0 | 0 | 0 | 0 | 1 | 0 | 0 | 1 | 0 | 0 | 0 | 1 | 0 | | 39 | 0 | 0 | 0 | 0 | 0 | 0 | 0 | 0 | 0 | 0 | 0 | 0 | 0 | 0 | 0 | 0 | 0 | 0 | 0 | 0 | 0 | 0 | 0 | 0 | 0 | 0 | 0 | 0 | 0 | 0 | 0 | 0 | 0 | 0 | 0 | 0 | 0 | 0 | 0 | 6 | 0 | 0 | 0 | 0 | 0 | 0 | 0 | 0 | 0 | 0 | 0 | 0 | | 40 | 0 | 0 | 0 | 0 | 0 | 0 | 0 | 0 | 0 | 0 | 0 | 0 | 1 | 18 | 0 | 0 | 0 | 0 | 0 | 0 | 0 | 0 | 0 | 0 | 0 | 0 | 1 | 0 | 1 | 0 | 0 | 0 | 0 | 1 | 0 | 0 | 0 | 0 | 0 | 0 | 0 | 1 | 0 | 0 | 0 | 0 | 0 | 0 | 0 | 0 | 4 | 0 | | 41 | 0 | 0 | 0 | 0 | 0 | 0 | 0 | 0 | 0 | 0 | 0 | 0 | 0 | 0 | 0 | 1 | 0 | 0 | 0 | 0 | 0 | 0 | 1 | 0 | 0 | 0 | 0 | 0 | 0 | 0 | 0 | 0 | 0 | 0 | 0 | 0 | 0 | 0 | 0 | 0 | 3 | 0 | 0 | 0 | 0 | 0 | 0 | 0 | 0 | 0 | 1 | 0 | | 42 | 0 | 0 | 0 | 0 | 0 | 1 | 0 | 0 | 0 | 0 | 0 | 0 | 0 | 0 | 0 | 0 | 0 | 0 | 0 | 0 | 0 | 0 | 0 | 5 | 0 | 0 | 0 | 0 | 0 | 0 | 0 | 0 | 0 | 0 | 0 | 0 | 0 | 0 | 1 | 0 | 0 | 0 | 0 | 0 | 0 | 0 | 0 | 0 | 0 | 0 | 0 | 0 | | 43 | 4 | 1 | 0 | 0 | 0 | 0 | 0 | 0 | 0 | 0 | 0 | 0 | 0 | 0 | 0 | 0 | 0 | 0 | 0 | 0 | 0 | 0 | 0 | 0 | 0 | 0 | 0 | 2 | 1 | 0 | 0 | 0 | 0 | 0 | 0 | 0 | 0 | 0 | 0 | 0 | 0 | 0 | 0 | 0 | 0 | 0 | 0 | 0 | 0 | 0 | 0 | 0 | | 44 | 0 | 0 | 0 | 0 | 0 | 0 | 0 | 0 | 0 | 0 | 0 | 1 | 0 | 0 | 0 | 0 | 0 | 0 | 0 | 0 | 0 | 0 | 0 | 0 | 0 | 0 | 0 | 0 | 0 | 0 | 0 | 0 | 0 | 0 | 0 | 0 | 0 | 0 | 0 | 0 | 0 | 0 | 0 | 0 | 3 | 0 | 0 | 0 | 0 | 0 | 2 | 0 | | 45 | 0 | 0 | 0 | 0 | 0 | 0 | 0 | 0 | 0 | 0 | 0 | 1 | 0 | 0 | 0 | 0 | 0 | 0 | 0 | 0 | 0 | 0 | 0 | 1 | 0 | 0 | 0 | 0 | 0 | 0 | 0 | 0 | 0 | 0 | 0 | 0 | 0 | 0 | 0 | 0 | 1 | 0 | 0 | 0 | 0 | 10 | 0 | 0 | 0 | 2 | 0 | 0 | | 46 | 0 | 0 | 0 | 0 | 0 | 0 | 0 | 0 | 0 | 0 | 0 | 0 | 0 | 0 | 0 | 0 | 0 | 0 | 0 | 0 | 0 | 0 | 0 | 0 | 0 | 0 | 0 | 0 | 0 | 0 | 0 | 0 | 0 | 0 | 0 | 0 | 0 | 0 | 0 | 0 | 0 | 0 | 0 | 0 | 0 | 0 | 16 | 0 | 0 | 0 | 1 | 0 | | 47 | 3 | 1 | 0 | 0 | 0 | 0 | 0 | 0 | 0 | 0 | 0 | 0 | 1 | 0 | 0 | 0 | 0 | 0 | 0 | 0 | 0 | 0 | 0 | 1 | 0 | 0 | 0 | 0 | 1 | 0 | 0 | 0 | 0 | 0 | 0 | 0 | 0 | 0 | 0 | 0 | 0 | 0 | 0 | 0 | 0 | 1 | 0 | 7 | 0 | 0 | 0 | 0 | | 48 | 0 | 0 | 0 | 0 | 0 | 0 | 0 | 0 | 0 | 0 | 0 | 0 | 0 | 0 | 0 | 0 | 0 | 0 | 0 | 0 | 0 | 0 | 0 | 0 | 0 | 0 | 0 | 0 | 0 | 0 | 0 | 0 | 0 | 0 | 0 | 1 | 0 | 0 | 0 | 0 | 0 | 0 | 0 | 0 | 0 | 0 | 0 | 0 | 4 | 0 | 0 | 0 | | 49 | 1 | 0 | 0 | 0 | 0 | 0 | 0 | 1 | 0 | 0 | 0 | 4 | 0 | 4 | 0 | 0 | 0 | 0 | 0 | 0 | 0 | 0 | 0 | 0 | 0 | 0 | 0 | 0 | 0 | 0 | 0 | 0 | 0 | 0 | 0 | 0 | 0 | 0 | 0 | 0 | 0 | 0 | 0 | 0 | 0 | 0 | 0 | 0 | 0 | 0 | 0 | 0 | | 50 | 0 | 0 | 0 | 0 | 0 | 0 | 4 | 0 | 0 | 0 | 0 | 0 | 14 | 0 | 0 | 0 | 0 | 0 | 0 | 0 | 0 | 0 | 0 | 0 | 0 | 0 | 0 | 0 | 0 | 0 | 0 | 0 | 0 | 0 | 0 | 0 | 0 | 0 | 0 | 0 | 0 | 0 | 0 | 0 | 0 | 0 | 0 | 0 | 0 | 0 | 0 | 0 | | 51 | 0 | 0 | 0 | 0 | 0 | 0 | 0 | 0 | 0 | 0 | 0 | 0 | 0 | 0 | 0 | 0 | 0 | 0 | 0 | 0 | 0 | 0 | 0 | 0 | 0 | 0 | 0 | 0 | 0 | 0 | 0 | 0 | 0 | 0 | 0 | 0 | 0 | 0 | 0 | 0 | 0 | 0 | 0 | 0 | 0 | 0 | 0 | 0 | 0 | 0 | 1 | 6 | |

## Overall Statistics :

|  |  |
| --- | --- |
| 95% CI | (0.60008,0.65918) |
| ACC Macro | 0.98575 |
| ARI | 0.69167 |
| AUNP | 0.80984 |
| AUNU | 0.76688 |
| Bangdiwala B | 0.71792 |
| Bennett S | 0.62237 |
| CBA | 0.45276 |
| CSI | None |
| Chi-Squared | None |
| Chi-Squared DF | 2601 |
| Conditional Entropy | 1.22363 |
| Cramer V | None |
| Cross Entropy | 5.08414 |
| F1 Macro | 0.51614 |
| F1 Micro | 0.62963 |
| FNR Macro | 0.45892 |
| FNR Micro | 0.37037 |
| FPR Macro | 0.00731 |
| FPR Micro | 0.00726 |
| Gwet AC1 | 0.6226 |
| Hamming Loss | 0.37037 |
| Joint Entropy | 6.20096 |
| KL Divergence | None |
| Kappa | 0.61047 |
| Kappa 95% CI | (0.57939,0.64155) |
| Kappa No Prevalence | 0.25926 |
| Kappa Standard Error | 0.01586 |
| Kappa Unbiased | 0.60987 |
| Krippendorff Alpha | 0.61006 |
| Lambda A | 0.62373 |
| Lambda B | 0.6746 |
| Mutual Information | 3.56624 |
| NIR | 0.13743 |
| Overall ACC | 0.62963 |
| Overall CEN | 0.21009 |
| Overall J | (21.99159,0.42292) |
| Overall MCC | 0.61234 |
| Overall MCEN | 0.28367 |
| Overall RACC | 0.04919 |
| Overall RACCU | 0.05064 |
| P-Value | 0.0 |
| PPV Macro | None |
| PPV Micro | 0.62963 |
| Pearson C | None |
| Phi-Squared | None |
| RCI | 0.7165 |
| RR | 19.73077 |
| Reference Entropy | 4.97733 |
| Response Entropy | 4.78986 |
| SOA1(Landis & Koch) | Substantial |
| SOA2(Fleiss) | Intermediate to Good |
| SOA3(Altman) | Good |
| SOA4(Cicchetti) | Good |
| SOA5(Cramer) | None |
| SOA6(Matthews) | Moderate |
| Scott PI | 0.60987 |
| Standard Error | 0.01508 |
| TNR Macro | 0.99269 |
| TNR Micro | 0.99274 |
| TPR Macro | 0.54108 |
| TPR Micro | 0.62963 |
| Zero-one Loss | 380 |

## Class Statistics :

|  |  |  |  |  |  |  |  |  |  |  |  |  |  |  |  |  |  |  |  |  |  |  |  |  |  |  |  |  |  |  |  |  |  |  |  |  |  |  |  |  |  |  |  |  |  |  |  |  |  |  |  |  |  |
| --- | --- | --- | --- | --- | --- | --- | --- | --- | --- | --- | --- | --- | --- | --- | --- | --- | --- | --- | --- | --- | --- | --- | --- | --- | --- | --- | --- | --- | --- | --- | --- | --- | --- | --- | --- | --- | --- | --- | --- | --- | --- | --- | --- | --- | --- | --- | --- | --- | --- | --- | --- | --- | --- |
| Class | 0 | 1 | 2 | 3 | 4 | 5 | 6 | 7 | 8 | 9 | 10 | 11 | 12 | 13 | 14 | 15 | 16 | 17 | 18 | 19 | 20 | 21 | 22 | 23 | 24 | 25 | 26 | 27 | 28 | 29 | 30 | 31 | 32 | 33 | 34 | 35 | 36 | 37 | 38 | 39 | 40 | 41 | 42 | 43 | 44 | 45 | 46 | 47 | 48 | 49 | 50 | 51 | Description |
| ACC | 0.9386 | 0.98441 | 0.9961 | 0.99513 | 0.99903 | 0.98928 | 0.98928 | 0.9961 | 0.99708 | 0.9961 | 0.99123 | 0.95029 | 0.9347 | 0.96881 | 0.99708 | 0.98538 | 0.97758 | 0.99708 | 0.98928 | 0.97466 | 0.98538 | 0.99513 | 0.96979 | 0.96296 | 0.99513 | 0.9883 | 0.98538 | 0.9922 | 0.96004 | 1.0 | 0.99318 | 0.9961 | 0.99513 | 0.99805 | 0.99903 | 0.99805 | 0.99903 | 0.99708 | 0.97076 | 0.99903 | 0.96881 | 0.99123 | 0.99318 | 0.99123 | 0.99708 | 0.98441 | 0.9922 | 0.9922 | 0.99903 | 0.9883 | 0.93665 | 0.99805 | Accuracy |
| AGF | 0.35077 | 0.81704 | 0.0 | 0.65337 | 0.95309 | 0.40058 | 0.0 | 0.52638 | 0.96589 | 0.77964 | 0.92829 | 0.28071 | 0.60022 | 0.88443 | 0.96946 | 0.59549 | 0.54955 | 0.59709 | 0.35234 | 0.77491 | 0.97276 | 0.87254 | 0.8198 | 0.0 | 0.88782 | 0.0 | 0.73149 | 0.89545 | 0.7809 | 1.0 | 0.77384 | 0.91251 | 0.8656 | 0.97241 | 0.95337 | 0.99464 | 0.93897 | 0.971 | 0.30871 | 0.98364 | 0.0 | 0.0 | 0.0 | 0.0 | 0.74448 | 0.78325 | 0.93659 | 0.72049 | 0.91251 | 0.0 | 0.0 | 0.92537 | Adjusted F-score |
| AGM | 0.65878 | 0.89914 | 0 | 0.80543 | 0.97128 | 0.69972 | 0 | 0.7489 | 0.98432 | 0.87212 | 0.9604 | 0.62641 | 0.81474 | 0.96745 | 0.98261 | 0.79532 | 0.75984 | 0.78774 | 0.66348 | 0.8708 | 0.98106 | 0.92694 | 0.90299 | 0 | 0.95991 | 0 | 0.84981 | 0.96914 | 0.87339 | 1.0 | 0.92868 | 0.99707 | 0.99633 | 0.99853 | 0.99927 | 0.99666 | 0.96278 | 0.99779 | 0.63834 | 0.99927 | 0 | 0 | 0 | 0 | 0.85312 | 0.89993 | 0.97981 | 0.8404 | 0.94708 | 0 | 0 | 0.96207 | Adjusted geometric mean |
| AM | 13 | -4 | -4 | -5 | -1 | 1 | -3 | -2 | 1 | -4 | -1 | 9 | 43 | 30 | -1 | 1 | -7 | -1 | -5 | -20 | 3 | -3 | 5 | -6 | 3 | -10 | -9 | 6 | -19 | 0 | 5 | 4 | 5 | 2 | 1 | 0 | -1 | 3 | -12 | 1 | -22 | -3 | -7 | -7 | -3 | 6 | 6 | -8 | -1 | -8 | 29 | 0 | Difference between automatic and manual classification |
| AUC | 0.54991 | 0.82458 | 0.5 | 0.6875 | 0.94444 | 0.58039 | 0.49804 | 0.62451 | 0.97123 | 0.77778 | 0.92854 | 0.52852 | 0.72255 | 0.96597 | 0.96724 | 0.67788 | 0.63888 | 0.66618 | 0.55408 | 0.78148 | 0.97364 | 0.86617 | 0.83968 | 0.49203 | 0.92661 | 0.49951 | 0.7485 | 0.94656 | 0.79152 | 1.0 | 0.87206 | 0.99804 | 0.99756 | 0.99902 | 0.99951 | 0.99465 | 0.92857 | 0.99852 | 0.53899 | 0.99951 | 0.4975 | 0.49853 | 0.5 | 0.49951 | 0.75 | 0.82789 | 0.96712 | 0.73333 | 0.9 | 0.49902 | 0.47669 | 0.92808 | Area under the ROC curve |
| AUCI | Poor | Very Good | Poor | Fair | Excellent | Poor | Poor | Fair | Excellent | Good | Excellent | Poor | Good | Excellent | Excellent | Fair | Fair | Fair | Poor | Good | Excellent | Very Good | Very Good | Poor | Excellent | Poor | Good | Excellent | Good | Excellent | Very Good | Excellent | Excellent | Excellent | Excellent | Excellent | Excellent | Excellent | Poor | Excellent | Poor | Poor | Poor | Poor | Good | Very Good | Excellent | Good | Excellent | Poor | Poor | Excellent | AUC value interpretation |
| AUPR | 0.11658 | 0.70759 | None | 0.6875 | 0.94444 | 0.15476 | 0.0 | 0.375 | 0.91959 | 0.77778 | 0.87341 | 0.07473 | 0.33955 | 0.70955 | 0.95108 | 0.34848 | 0.35714 | 0.41667 | 0.18056 | 0.73756 | 0.94747 | 0.825 | 0.66134 | 0.0 | 0.72857 | 0.0 | 0.65 | 0.73125 | 0.6973 | 1.0 | 0.54167 | 0.75 | 0.6875 | 0.88889 | 0.83333 | 0.99038 | 0.92857 | 0.88462 | 0.13439 | 0.92857 | 0.0 | 0.0 | None | 0.0 | 0.75 | 0.57143 | 0.81841 | 0.73333 | 0.9 | 0.0 | 0.0 | 0.85714 | Area under the PR curve |
| BCD | 0.00634 | 0.00195 | 0.00195 | 0.00244 | 0.00049 | 0.00049 | 0.00146 | 0.00097 | 0.00049 | 0.00195 | 0.00049 | 0.00439 | 0.02096 | 0.01462 | 0.00049 | 0.00049 | 0.00341 | 0.00049 | 0.00244 | 0.00975 | 0.00146 | 0.00146 | 0.00244 | 0.00292 | 0.00146 | 0.00487 | 0.00439 | 0.00292 | 0.00926 | 0.0 | 0.00244 | 0.00195 | 0.00244 | 0.00097 | 0.00049 | 0.0 | 0.00049 | 0.00146 | 0.00585 | 0.00049 | 0.01072 | 0.00146 | 0.00341 | 0.00341 | 0.00146 | 0.00292 | 0.00292 | 0.0039 | 0.00049 | 0.0039 | 0.01413 | 0.0 | Bray-Curtis dissimilarity |
| BM | 0.09982 | 0.64915 | 0.0 | 0.375 | 0.88889 | 0.16078 | -0.00393 | 0.24902 | 0.94246 | 0.55556 | 0.85707 | 0.05705 | 0.44511 | 0.93193 | 0.93448 | 0.35575 | 0.27775 | 0.33236 | 0.10816 | 0.56295 | 0.94728 | 0.73234 | 0.67936 | -0.01594 | 0.85322 | -0.00099 | 0.49701 | 0.89311 | 0.58304 | 1.0 | 0.74413 | 0.99609 | 0.99511 | 0.99804 | 0.99902 | 0.9893 | 0.85714 | 0.99705 | 0.07798 | 0.99902 | -0.00501 | -0.00294 | 0.0 | -0.00098 | 0.5 | 0.65579 | 0.93424 | 0.46667 | 0.8 | -0.00197 | -0.04663 | 0.85616 | Informedness or bookmaker informedness |
| CEN | 0.55814 | 0.20696 | 0.22481 | 0.18117 | 0.03603 | 0.37704 | 0.38222 | 0.25827 | 0.0633 | 0.14162 | 0.09413 | 0.4707 | 0.45221 | 0.20115 | 0.0388 | 0.36395 | 0.34875 | 0.20879 | 0.36834 | 0.18715 | 0.04577 | 0.10557 | 0.281 | 0.46291 | 0.16254 | 0.33745 | 0.25855 | 0.1937 | 0.28376 | 0 | 0.16478 | 0.11971 | 0.07749 | 0.07494 | 0.0696 | 0.0111 | 0.04266 | 0.0754 | 0.57795 | 0.04266 | 0.33808 | 0.3959 | 0.17218 | 0.30856 | 0.12505 | 0.19774 | 0.11602 | 0.21064 | 0.05279 | 0.31247 | 0.56462 | 0.08152 | Confusion entropy |
| DOR | 4.03789 | 313.81667 | None | None | None | 33.8 | 0.0 | 340.33333 | 8551.0 | None | 1528.3 | 3.08889 | 17.21818 | 811.87097 | 14413.0 | 71.92857 | 49.85 | 511.0 | 42.25 | 421.73913 | 2190.0 | 2777.5 | 123.71795 | 0.0 | 1522.5 | 0.0 | 333.0 | 1297.28571 | 125.46667 | None | 508.0 | None | None | None | None | 94863.0 | None | None | 10.51852 | None | 0.0 | 0.0 | None | 0.0 | None | 181.81818 | 2290.28571 | None | None | 0.0 | 0.0 | 6108.0 | Diagnostic odds ratio |
| DP | 0.33419 | 1.37649 | None | None | None | 0.84294 | None | 1.39591 | 2.16783 | None | 1.75555 | 0.27004 | 0.68144 | 1.60408 | 2.29284 | 1.02376 | 0.93597 | 1.49323 | 0.89637 | 1.44726 | 1.84168 | 1.89859 | 1.15362 | None | 1.75464 | None | 1.3907 | 1.71631 | 1.15698 | None | 1.49182 | None | None | None | None | 2.74402 | None | None | 0.56343 | None | None | None | None | None | None | 1.2458 | 1.8524 | None | None | None | None | 2.08728 | Discriminant power |
| DPI | Poor | Limited | None | None | None | Poor | None | Limited | Fair | None | Limited | Poor | Poor | Limited | Fair | Limited | Poor | Limited | Poor | Limited | Limited | Limited | Limited | None | Limited | None | Limited | Limited | Limited | None | Limited | None | None | None | None | Fair | None | None | Poor | None | None | None | None | None | None | Limited | Limited | None | None | None | None | Fair | Discriminant power interpretation |
| ERR | 0.0614 | 0.01559 | 0.0039 | 0.00487 | 0.00097 | 0.01072 | 0.01072 | 0.0039 | 0.00292 | 0.0039 | 0.00877 | 0.04971 | 0.0653 | 0.03119 | 0.00292 | 0.01462 | 0.02242 | 0.00292 | 0.01072 | 0.02534 | 0.01462 | 0.00487 | 0.03021 | 0.03704 | 0.00487 | 0.0117 | 0.01462 | 0.0078 | 0.03996 | 0.0 | 0.00682 | 0.0039 | 0.00487 | 0.00195 | 0.00097 | 0.00195 | 0.00097 | 0.00292 | 0.02924 | 0.00097 | 0.03119 | 0.00877 | 0.00682 | 0.00877 | 0.00292 | 0.01559 | 0.0078 | 0.0078 | 0.00097 | 0.0117 | 0.06335 | 0.00195 | Error rate |
| F0.5 | 0.10152 | 0.73643 | 0.0 | 0.75 | 0.97561 | 0.14706 | 0.0 | 0.41667 | 0.90426 | 0.86207 | 0.88068 | 0.06623 | 0.20548 | 0.5098 | 0.96026 | 0.33898 | 0.38961 | 0.45455 | 0.2 | 0.81081 | 0.94142 | 0.87302 | 0.6383 | 0.0 | 0.6383 | 0.0 | 0.71429 | 0.60811 | 0.7483 | 1.0 | 0.375 | 0.55556 | 0.42857 | 0.81395 | 0.71429 | 0.99038 | 0.96774 | 0.80645 | 0.14925 | 0.88235 | 0.0 | 0.0 | 0.0 | 0.0 | 0.83333 | 0.50505 | 0.73394 | 0.81395 | 0.95238 | 0.0 | 0.0 | 0.85714 | F0.5 score |
| F1 | 0.11268 | 0.7037 | 0.0 | 0.54545 | 0.94118 | 0.15385 | 0.0 | 0.33333 | 0.91892 | 0.71429 | 0.87324 | 0.07273 | 0.26374 | 0.61905 | 0.95082 | 0.34783 | 0.34286 | 0.4 | 0.15385 | 0.69767 | 0.94737 | 0.81481 | 0.65934 | 0.0 | 0.70588 | 0.0 | 0.61538 | 0.69231 | 0.68217 | 1.0 | 0.46154 | 0.66667 | 0.54545 | 0.875 | 0.8 | 0.99038 | 0.92308 | 0.86957 | 0.11765 | 0.92308 | 0.0 | 0.0 | 0.0 | 0.0 | 0.66667 | 0.55556 | 0.8 | 0.63636 | 0.88889 | 0.0 | 0.0 | 0.85714 | F1 score - harmonic mean of precision and sensitivity |
| F2 | 0.12658 | 0.67376 | 0.0 | 0.42857 | 0.90909 | 0.16129 | 0.0 | 0.27778 | 0.93407 | 0.60976 | 0.86592 | 0.08065 | 0.3681 | 0.78788 | 0.94156 | 0.35714 | 0.30612 | 0.35714 | 0.125 | 0.61224 | 0.95339 | 0.76389 | 0.68182 | 0.0 | 0.78947 | 0.0 | 0.54054 | 0.80357 | 0.62678 | 1.0 | 0.6 | 0.83333 | 0.75 | 0.94595 | 0.90909 | 0.99038 | 0.88235 | 0.9434 | 0.09709 | 0.96774 | 0.0 | 0.0 | 0.0 | 0.0 | 0.55556 | 0.61728 | 0.87912 | 0.52239 | 0.83333 | 0.0 | 0.0 | 0.85714 | F2 score |
| FDR | 0.90476 | 0.24 | None | 0.0 | 0.0 | 0.85714 | 1.0 | 0.5 | 0.10526 | 0.0 | 0.11429 | 0.9375 | 0.8209 | 0.54386 | 0.03333 | 0.66667 | 0.57143 | 0.5 | 0.75 | 0.09091 | 0.0625 | 0.08333 | 0.375 | 1.0 | 0.4 | 1.0 | 0.2 | 0.4375 | 0.2 | 0.0 | 0.66667 | 0.5 | 0.625 | 0.22222 | 0.33333 | 0.00962 | 0.0 | 0.23077 | 0.81818 | 0.14286 | 1.0 | 1.0 | None | 1.0 | 0.0 | 0.52381 | 0.30435 | 0.0 | 0.0 | 1.0 | 1.0 | 0.14286 | False discovery rate |
| FN | 25 | 10 | 4 | 5 | 1 | 5 | 7 | 3 | 1 | 4 | 5 | 21 | 12 | 1 | 2 | 7 | 15 | 2 | 8 | 23 | 6 | 4 | 13 | 22 | 1 | 11 | 12 | 1 | 30 | 0 | 1 | 0 | 0 | 0 | 0 | 1 | 1 | 0 | 21 | 0 | 27 | 6 | 7 | 8 | 3 | 5 | 1 | 8 | 1 | 10 | 18 | 1 | False negative/miss/type 2 error |
| FNR | 0.86207 | 0.34483 | 1.0 | 0.625 | 0.11111 | 0.83333 | 1.0 | 0.75 | 0.05556 | 0.44444 | 0.13889 | 0.91304 | 0.5 | 0.03704 | 0.06452 | 0.63636 | 0.71429 | 0.66667 | 0.88889 | 0.43396 | 0.04255 | 0.26667 | 0.30233 | 1.0 | 0.14286 | 1.0 | 0.5 | 0.1 | 0.40541 | 0.0 | 0.25 | 0.0 | 0.0 | 0.0 | 0.0 | 0.00962 | 0.14286 | 0.0 | 0.91304 | 0.0 | 1.0 | 1.0 | 1.0 | 1.0 | 0.5 | 0.33333 | 0.05882 | 0.53333 | 0.2 | 1.0 | 1.0 | 0.14286 | Miss rate or false negative rate |
| FOR | 0.02541 | 0.00999 | 0.0039 | 0.00489 | 0.00098 | 0.00491 | 0.00685 | 0.00293 | 0.00099 | 0.00392 | 0.00505 | 0.02113 | 0.01251 | 0.00103 | 0.00201 | 0.0069 | 0.01482 | 0.00195 | 0.00783 | 0.02316 | 0.0068 | 0.00394 | 0.01329 | 0.02178 | 0.00098 | 0.01073 | 0.01187 | 0.00099 | 0.0309 | 0.0 | 0.00098 | 0.0 | 0.0 | 0.0 | 0.0 | 0.00108 | 0.00098 | 0.0 | 0.02069 | 0.0 | 0.02644 | 0.00587 | 0.00682 | 0.0078 | 0.00293 | 0.00498 | 0.001 | 0.00785 | 0.00098 | 0.00977 | 0.01839 | 0.00098 | False omission rate |
| FP | 38 | 6 | 0 | 0 | 0 | 6 | 4 | 1 | 2 | 0 | 4 | 30 | 55 | 31 | 1 | 8 | 8 | 1 | 3 | 3 | 9 | 1 | 18 | 16 | 4 | 1 | 3 | 7 | 11 | 0 | 6 | 4 | 5 | 2 | 1 | 1 | 0 | 3 | 9 | 1 | 5 | 3 | 0 | 1 | 0 | 11 | 7 | 0 | 0 | 2 | 47 | 1 | False positive/type 1 error/false alarm |
| FPR | 0.03811 | 0.00602 | 0.0 | 0.0 | 0.0 | 0.00588 | 0.00393 | 0.00098 | 0.00198 | 0.0 | 0.00404 | 0.02991 | 0.05489 | 0.03103 | 0.00101 | 0.00788 | 0.00796 | 0.00098 | 0.00295 | 0.00308 | 0.01017 | 0.00099 | 0.01831 | 0.01594 | 0.00393 | 0.00099 | 0.00299 | 0.00689 | 0.01155 | 0.0 | 0.00587 | 0.00391 | 0.00489 | 0.00196 | 0.00098 | 0.00108 | 0.0 | 0.00295 | 0.00897 | 0.00098 | 0.00501 | 0.00294 | 0.0 | 0.00098 | 0.0 | 0.01088 | 0.00694 | 0.0 | 0.0 | 0.00197 | 0.04663 | 0.00098 | Fall-out or false positive rate |
| G | 0.11461 | 0.70564 | None | 0.61237 | 0.94281 | 0.1543 | 0.0 | 0.35355 | 0.91925 | 0.74536 | 0.87333 | 0.07372 | 0.29925 | 0.66276 | 0.95095 | 0.34816 | 0.34993 | 0.40825 | 0.16667 | 0.71734 | 0.94742 | 0.81989 | 0.66034 | 0.0 | 0.71714 | 0.0 | 0.63246 | 0.71151 | 0.68969 | 1.0 | 0.5 | 0.70711 | 0.61237 | 0.88192 | 0.8165 | 0.99038 | 0.92582 | 0.87706 | 0.12574 | 0.92582 | 0.0 | 0.0 | None | 0.0 | 0.70711 | 0.56344 | 0.80915 | 0.68313 | 0.89443 | 0.0 | 0.0 | 0.85714 | G-measure geometric mean of precision and sensitivity |
| GI | 0.09982 | 0.64915 | 0.0 | 0.375 | 0.88889 | 0.16078 | -0.00393 | 0.24902 | 0.94246 | 0.55556 | 0.85707 | 0.05705 | 0.44511 | 0.93193 | 0.93448 | 0.35575 | 0.27775 | 0.33236 | 0.10816 | 0.56295 | 0.94728 | 0.73234 | 0.67936 | -0.01594 | 0.85322 | -0.00099 | 0.49701 | 0.89311 | 0.58304 | 1.0 | 0.74413 | 0.99609 | 0.99511 | 0.99804 | 0.99902 | 0.9893 | 0.85714 | 0.99705 | 0.07798 | 0.99902 | -0.00501 | -0.00294 | 0.0 | -0.00098 | 0.5 | 0.65579 | 0.93424 | 0.46667 | 0.8 | -0.00197 | -0.04663 | 0.85616 | Gini index |
| GM | 0.36424 | 0.80699 | 0.0 | 0.61237 | 0.94281 | 0.40705 | 0.0 | 0.49976 | 0.97086 | 0.74536 | 0.92608 | 0.29044 | 0.68743 | 0.96596 | 0.96672 | 0.60064 | 0.53239 | 0.57707 | 0.33284 | 0.75119 | 0.9735 | 0.85593 | 0.82759 | 0.0 | 0.924 | 0.0 | 0.70605 | 0.94541 | 0.76663 | 1.0 | 0.86348 | 0.99804 | 0.99755 | 0.99902 | 0.99951 | 0.99464 | 0.92582 | 0.99852 | 0.29356 | 0.99951 | 0.0 | 0.0 | 0.0 | 0.0 | 0.70711 | 0.81204 | 0.96677 | 0.68313 | 0.89443 | 0.0 | 0.0 | 0.92537 | G-mean geometric mean of specificity and sensitivity |
| IBA | 0.02336 | 0.43059 | 0.0 | 0.14062 | 0.79012 | 0.02859 | 0.0 | 0.06268 | 0.89208 | 0.30864 | 0.74198 | 0.00986 | 0.26222 | 0.92748 | 0.87519 | 0.13403 | 0.08324 | 0.11133 | 0.01264 | 0.32115 | 0.91702 | 0.53797 | 0.49038 | 0.0 | 0.73516 | 0.0 | 0.25074 | 0.81058 | 0.35625 | 1.0 | 0.56357 | 0.99998 | 0.99998 | 1.0 | 1.0 | 0.98087 | 0.73469 | 0.99999 | 0.00827 | 1.0 | 0.0 | 0.0 | 0.0 | 0.0 | 0.25 | 0.44678 | 0.88615 | 0.21778 | 0.64 | 0.0 | 0.0 | 0.73481 | Index of balanced accuracy |
| ICSI | -0.76683 | 0.41517 | None | 0.375 | 0.88889 | -0.69048 | -1.0 | -0.25 | 0.83918 | 0.55556 | 0.74683 | -0.85054 | -0.3209 | 0.4191 | 0.90215 | -0.30303 | -0.28571 | -0.16667 | -0.63889 | 0.47513 | 0.89495 | 0.65 | 0.32267 | -1.0 | 0.45714 | -1.0 | 0.3 | 0.4625 | 0.39459 | 1.0 | 0.08333 | 0.5 | 0.375 | 0.77778 | 0.66667 | 0.98077 | 0.85714 | 0.76923 | -0.73123 | 0.85714 | -1.0 | -1.0 | None | -1.0 | 0.5 | 0.14286 | 0.63683 | 0.46667 | 0.8 | -1.0 | -1.0 | 0.71429 | Individual classification success index |
| IS | 1.75252 | 4.74891 | None | 7.00282 | 6.83289 | 4.6105 | None | 7.00282 | 5.67243 | 6.83289 | 4.6578 | 1.47925 | 2.93673 | 4.11548 | 4.99971 | 4.95842 | 4.38811 | 7.41785 | 4.83289 | 4.13739 | 2.77015 | 5.97039 | 3.89848 | None | 6.45849 | None | 5.09592 | 5.85081 | 3.47143 | 7.19546 | 6.41785 | 7.00282 | 7.00282 | 6.83289 | 8.41785 | 3.28844 | 7.19546 | 6.30238 | 3.01982 | 7.19546 | None | None | None | None | 7.41785 | 5.02554 | 5.39179 | 6.09592 | 7.68089 | None | None | 6.97307 | Information score |
| J | 0.0597 | 0.54286 | 0.0 | 0.375 | 0.88889 | 0.08333 | 0.0 | 0.2 | 0.85 | 0.55556 | 0.775 | 0.03774 | 0.1519 | 0.44828 | 0.90625 | 0.21053 | 0.2069 | 0.25 | 0.08333 | 0.53571 | 0.9 | 0.6875 | 0.4918 | 0.0 | 0.54545 | 0.0 | 0.44444 | 0.52941 | 0.51765 | 1.0 | 0.3 | 0.5 | 0.375 | 0.77778 | 0.66667 | 0.98095 | 0.85714 | 0.76923 | 0.0625 | 0.85714 | 0.0 | 0.0 | 0.0 | 0.0 | 0.5 | 0.38462 | 0.66667 | 0.46667 | 0.8 | 0.0 | 0.0 | 0.75 | Jaccard index |
| LS | 3.36946 | 26.88828 | None | 128.25 | 114.0 | 24.42857 | 0.0 | 128.25 | 51.0 | 114.0 | 25.24286 | 2.78804 | 7.65672 | 17.33333 | 31.99355 | 31.09091 | 20.93878 | 171.0 | 28.5 | 17.59863 | 6.82181 | 62.7 | 14.91279 | 0.0 | 87.94286 | 0.0 | 34.2 | 57.7125 | 11.09189 | 146.57143 | 85.5 | 128.25 | 128.25 | 114.0 | 342.0 | 9.77053 | 146.57143 | 78.92308 | 8.11067 | 146.57143 | 0.0 | 0.0 | None | 0.0 | 171.0 | 32.57143 | 41.98465 | 68.4 | 205.2 | 0.0 | 0.0 | 125.63265 | Lift score |
| MCC | 0.08349 | 0.69776 | None | 0.61087 | 0.94235 | 0.14893 | -0.00519 | 0.35183 | 0.91778 | 0.74389 | 0.86879 | 0.04858 | 0.27231 | 0.65125 | 0.94945 | 0.34078 | 0.339 | 0.40685 | 0.16184 | 0.70621 | 0.93895 | 0.81757 | 0.64465 | -0.01863 | 0.71491 | -0.00325 | 0.62586 | 0.70816 | 0.66964 | 1.0 | 0.4973 | 0.70572 | 0.61087 | 0.88105 | 0.8161 | 0.9893 | 0.92537 | 0.87576 | 0.1121 | 0.92537 | -0.0115 | -0.00415 | None | -0.00277 | 0.70607 | 0.55589 | 0.80559 | 0.68044 | 0.89399 | -0.00438 | -0.02928 | 0.85616 | Matthews correlation coefficient |
| MCCI | Negligible | Moderate | None | Moderate | Very Strong | Negligible | Negligible | Weak | Very Strong | Strong | Strong | Negligible | Negligible | Moderate | Very Strong | Weak | Weak | Weak | Negligible | Strong | Very Strong | Strong | Moderate | Negligible | Strong | Negligible | Moderate | Strong | Moderate | Very Strong | Weak | Strong | Moderate | Strong | Strong | Very Strong | Very Strong | Strong | Negligible | Very Strong | Negligible | Negligible | None | Negligible | Strong | Moderate | Strong | Moderate | Strong | Negligible | Negligible | Strong | Matthews correlation coefficient interpretation |
| MCEN | 0.57967 | 0.27644 | 0.22481 | 0.20607 | 0.05279 | 0.39259 | 0.38222 | 0.27839 | 0.09716 | 0.17784 | 0.13916 | 0.48076 | 0.49497 | 0.24714 | 0.06088 | 0.40795 | 0.38865 | 0.22481 | 0.38317 | 0.24434 | 0.07308 | 0.1428 | 0.37525 | 0.46291 | 0.20842 | 0.33745 | 0.32929 | 0.25301 | 0.38713 | 0 | 0.17451 | 0.13573 | 0.06351 | 0.10557 | 0.07918 | 0.01917 | 0.06011 | 0.10492 | 0.60178 | 0.06011 | 0.33808 | 0.3959 | 0.17218 | 0.30856 | 0.14375 | 0.2305 | 0.15655 | 0.26477 | 0.0696 | 0.31247 | 0.56462 | 0.1124 | Modified confusion entropy |
| MK | 0.06983 | 0.75001 | None | 0.99511 | 0.99902 | 0.13795 | -0.00685 | 0.49707 | 0.89374 | 0.99608 | 0.88067 | 0.04137 | 0.16659 | 0.45511 | 0.96466 | 0.32643 | 0.41375 | 0.49805 | 0.24217 | 0.88593 | 0.9307 | 0.91272 | 0.61171 | -0.02178 | 0.59902 | -0.01073 | 0.78813 | 0.56151 | 0.7691 | 1.0 | 0.33235 | 0.5 | 0.375 | 0.77778 | 0.66667 | 0.9893 | 0.99902 | 0.76923 | 0.16113 | 0.85714 | -0.02644 | -0.00587 | None | -0.0078 | 0.99707 | 0.47122 | 0.69466 | 0.99215 | 0.99902 | -0.00977 | -0.01839 | 0.85616 | Markedness |
| N | 997 | 997 | 1022 | 1018 | 1017 | 1020 | 1019 | 1022 | 1008 | 1017 | 990 | 1003 | 1002 | 999 | 995 | 1015 | 1005 | 1023 | 1017 | 973 | 885 | 1011 | 983 | 1004 | 1019 | 1015 | 1002 | 1016 | 952 | 1019 | 1022 | 1022 | 1023 | 1019 | 1024 | 922 | 1019 | 1016 | 1003 | 1020 | 999 | 1020 | 1019 | 1018 | 1020 | 1011 | 1009 | 1011 | 1021 | 1016 | 1008 | 1019 | Condition negative |
| NLR | 0.89623 | 0.34692 | 1.0 | 0.625 | 0.11111 | 0.83826 | 1.00394 | 0.75073 | 0.05567 | 0.44444 | 0.13945 | 0.94119 | 0.52904 | 0.03822 | 0.06458 | 0.64142 | 0.72002 | 0.66732 | 0.89152 | 0.4353 | 0.04299 | 0.26693 | 0.30796 | 1.01619 | 0.14342 | 1.00099 | 0.5015 | 0.10069 | 0.41014 | 0.0 | 0.25148 | 0.0 | 0.0 | 0.0 | 0.0 | 0.00963 | 0.14286 | 0.0 | 0.92131 | 0.0 | 1.00503 | 1.00295 | 1.0 | 1.00098 | 0.5 | 0.337 | 0.05923 | 0.53333 | 0.2 | 1.00197 | 1.04891 | 0.143 | Negative likelihood ratio |
| NLRI | Negligible | Poor | Negligible | Negligible | Fair | Negligible | Negligible | Negligible | Good | Poor | Fair | Negligible | Negligible | Good | Good | Negligible | Negligible | Negligible | Negligible | Poor | Good | Poor | Poor | Negligible | Fair | Negligible | Negligible | Fair | Poor | Good | Poor | Good | Good | Good | Good | Good | Fair | Good | Negligible | Good | Negligible | Negligible | Negligible | Negligible | Negligible | Poor | Good | Negligible | Fair | Negligible | Negligible | Fair | Negative likelihood ratio interpretation |
| NPV | 0.97459 | 0.99001 | 0.9961 | 0.99511 | 0.99902 | 0.99509 | 0.99315 | 0.99707 | 0.99901 | 0.99608 | 0.99495 | 0.97887 | 0.98749 | 0.99897 | 0.99799 | 0.9931 | 0.98518 | 0.99805 | 0.99217 | 0.97684 | 0.9932 | 0.99606 | 0.98671 | 0.97822 | 0.99902 | 0.98927 | 0.98813 | 0.99901 | 0.9691 | 1.0 | 0.99902 | 1.0 | 1.0 | 1.0 | 1.0 | 0.99892 | 0.99902 | 1.0 | 0.97931 | 1.0 | 0.97356 | 0.99413 | 0.99318 | 0.9922 | 0.99707 | 0.99502 | 0.999 | 0.99215 | 0.99902 | 0.99023 | 0.98161 | 0.99902 | Negative predictive value |
| OC | 0.13793 | 0.76 | None | 1.0 | 1.0 | 0.16667 | 0.0 | 0.5 | 0.94444 | 1.0 | 0.88571 | 0.08696 | 0.5 | 0.96296 | 0.96667 | 0.36364 | 0.42857 | 0.5 | 0.25 | 0.90909 | 0.95745 | 0.91667 | 0.69767 | 0.0 | 0.85714 | 0.0 | 0.8 | 0.9 | 0.8 | 1.0 | 0.75 | 1.0 | 1.0 | 1.0 | 1.0 | 0.99038 | 1.0 | 1.0 | 0.18182 | 1.0 | 0.0 | 0.0 | None | 0.0 | 1.0 | 0.66667 | 0.94118 | 1.0 | 1.0 | 0.0 | 0.0 | 0.85714 | Overlap coefficient |
| OOC | 0.11461 | 0.70564 | None | 0.61237 | 0.94281 | 0.1543 | 0.0 | 0.35355 | 0.91925 | 0.74536 | 0.87333 | 0.07372 | 0.29925 | 0.66276 | 0.95095 | 0.34816 | 0.34993 | 0.40825 | 0.16667 | 0.71734 | 0.94742 | 0.81989 | 0.66034 | 0.0 | 0.71714 | 0.0 | 0.63246 | 0.71151 | 0.68969 | 1.0 | 0.5 | 0.70711 | 0.61237 | 0.88192 | 0.8165 | 0.99038 | 0.92582 | 0.87706 | 0.12574 | 0.92582 | 0.0 | 0.0 | None | 0.0 | 0.70711 | 0.56344 | 0.80915 | 0.68313 | 0.89443 | 0.0 | 0.0 | 0.85714 | Otsuka-Ochiai coefficient |
| OP | 0.18942 | 0.77896 | -0.0039 | 0.54058 | 0.9402 | 0.27644 | -0.01072 | 0.39641 | 0.9695 | 0.71039 | 0.91861 | 0.11482 | 0.62669 | 0.9657 | 0.96424 | 0.52181 | 0.4248 | 0.49744 | 0.18981 | 0.69898 | 0.96875 | 0.84176 | 0.80067 | -0.03704 | 0.92016 | -0.0117 | 0.65338 | 0.94302 | 0.71125 | 1.0 | 0.85321 | 0.99414 | 0.99268 | 0.99707 | 0.99854 | 0.99376 | 0.9221 | 0.9956 | 0.13209 | 0.99853 | -0.03119 | -0.00877 | -0.00682 | -0.00877 | 0.66374 | 0.78966 | 0.96538 | 0.62857 | 0.88791 | -0.0117 | -0.06335 | 0.92162 | Optimized precision |
| P | 29 | 29 | 4 | 8 | 9 | 6 | 7 | 4 | 18 | 9 | 36 | 23 | 24 | 27 | 31 | 11 | 21 | 3 | 9 | 53 | 141 | 15 | 43 | 22 | 7 | 11 | 24 | 10 | 74 | 7 | 4 | 4 | 3 | 7 | 2 | 104 | 7 | 10 | 23 | 6 | 27 | 6 | 7 | 8 | 6 | 15 | 17 | 15 | 5 | 10 | 18 | 7 | Condition positive or support |
| PLR | 3.61887 | 108.86782 | None | None | None | 28.33333 | 0.0 | 255.5 | 476.0 | None | 213.125 | 2.90725 | 9.10909 | 31.03226 | 930.80645 | 46.13636 | 35.89286 | 341.0 | 37.66667 | 183.58491 | 94.14894 | 741.4 | 38.10078 | 0.0 | 218.35714 | 0.0 | 167.0 | 130.62857 | 51.45946 | None | 127.75 | 255.5 | 204.6 | 509.5 | 1024.0 | 913.13462 | None | 338.66667 | 9.69082 | 1020.0 | 0.0 | 0.0 | None | 0.0 | None | 61.27273 | 135.66387 | None | None | 0.0 | 0.0 | 873.42857 | Positive likelihood ratio |
| PLRI | Poor | Good | None | None | None | Good | Negligible | Good | Good | None | Good | Poor | Fair | Good | Good | Good | Good | Good | Good | Good | Good | Good | Good | Negligible | Good | Negligible | Good | Good | Good | None | Good | Good | Good | Good | Good | Good | None | Good | Fair | Good | Negligible | Negligible | None | Negligible | None | Good | Good | None | None | Negligible | Negligible | Good | Positive likelihood ratio interpretation |
| POP | 1026 | 1026 | 1026 | 1026 | 1026 | 1026 | 1026 | 1026 | 1026 | 1026 | 1026 | 1026 | 1026 | 1026 | 1026 | 1026 | 1026 | 1026 | 1026 | 1026 | 1026 | 1026 | 1026 | 1026 | 1026 | 1026 | 1026 | 1026 | 1026 | 1026 | 1026 | 1026 | 1026 | 1026 | 1026 | 1026 | 1026 | 1026 | 1026 | 1026 | 1026 | 1026 | 1026 | 1026 | 1026 | 1026 | 1026 | 1026 | 1026 | 1026 | 1026 | 1026 | Population |
| PPV | 0.09524 | 0.76 | None | 1.0 | 1.0 | 0.14286 | 0.0 | 0.5 | 0.89474 | 1.0 | 0.88571 | 0.0625 | 0.1791 | 0.45614 | 0.96667 | 0.33333 | 0.42857 | 0.5 | 0.25 | 0.90909 | 0.9375 | 0.91667 | 0.625 | 0.0 | 0.6 | 0.0 | 0.8 | 0.5625 | 0.8 | 1.0 | 0.33333 | 0.5 | 0.375 | 0.77778 | 0.66667 | 0.99038 | 1.0 | 0.76923 | 0.18182 | 0.85714 | 0.0 | 0.0 | None | 0.0 | 1.0 | 0.47619 | 0.69565 | 1.0 | 1.0 | 0.0 | 0.0 | 0.85714 | Precision or positive predictive value |
| PRE | 0.02827 | 0.02827 | 0.0039 | 0.0078 | 0.00877 | 0.00585 | 0.00682 | 0.0039 | 0.01754 | 0.00877 | 0.03509 | 0.02242 | 0.02339 | 0.02632 | 0.03021 | 0.01072 | 0.02047 | 0.00292 | 0.00877 | 0.05166 | 0.13743 | 0.01462 | 0.04191 | 0.02144 | 0.00682 | 0.01072 | 0.02339 | 0.00975 | 0.07212 | 0.00682 | 0.0039 | 0.0039 | 0.00292 | 0.00682 | 0.00195 | 0.10136 | 0.00682 | 0.00975 | 0.02242 | 0.00585 | 0.02632 | 0.00585 | 0.00682 | 0.0078 | 0.00585 | 0.01462 | 0.01657 | 0.01462 | 0.00487 | 0.00975 | 0.01754 | 0.00682 | Prevalence |
| Q | 0.60301 | 0.99365 | None | None | None | 0.94253 | -1.0 | 0.99414 | 0.99977 | None | 0.99869 | 0.51087 | 0.89022 | 0.99754 | 0.99986 | 0.97258 | 0.96067 | 0.99609 | 0.95376 | 0.99527 | 0.99909 | 0.99928 | 0.98396 | -1.0 | 0.99869 | -1.0 | 0.99401 | 0.99846 | 0.98419 | None | 0.99607 | None | None | None | None | 0.99998 | None | None | 0.82637 | None | -1.0 | -1.0 | None | -1.0 | None | 0.98906 | 0.99913 | None | None | -1.0 | -1.0 | 0.99967 | Yule Q - coefficient of colligation |
| QI | Moderate | Strong | None | None | None | Strong | Negligible | Strong | Strong | None | Strong | Moderate | Strong | Strong | Strong | Strong | Strong | Strong | Strong | Strong | Strong | Strong | Strong | Negligible | Strong | Negligible | Strong | Strong | Strong | None | Strong | None | None | None | None | Strong | None | None | Strong | None | Negligible | Negligible | None | Negligible | None | Strong | Strong | None | None | Negligible | Negligible | Strong | Yule Q interpretation |
| RACC | 0.00116 | 0.00069 | 0.0 | 2e-05 | 7e-05 | 4e-05 | 3e-05 | 1e-05 | 0.00032 | 4e-05 | 0.0012 | 0.0007 | 0.00153 | 0.00146 | 0.00088 | 0.00013 | 0.00028 | 1e-05 | 3e-05 | 0.00166 | 0.01929 | 0.00017 | 0.00196 | 0.00033 | 7e-05 | 1e-05 | 0.00034 | 0.00015 | 0.00387 | 5e-05 | 3e-05 | 3e-05 | 2e-05 | 6e-05 | 1e-05 | 0.01027 | 4e-05 | 0.00012 | 0.00024 | 4e-05 | 0.00013 | 2e-05 | 0.0 | 1e-05 | 2e-05 | 0.0003 | 0.00037 | 0.0001 | 2e-05 | 2e-05 | 0.0008 | 5e-05 | Random accuracy |
| RACCU | 0.0012 | 0.00069 | 0.0 | 3e-05 | 7e-05 | 4e-05 | 3e-05 | 1e-05 | 0.00033 | 5e-05 | 0.0012 | 0.00072 | 0.00197 | 0.00168 | 0.00088 | 0.00013 | 0.00029 | 1e-05 | 4e-05 | 0.00176 | 0.01929 | 0.00017 | 0.00197 | 0.00034 | 7e-05 | 3e-05 | 0.00036 | 0.00016 | 0.00395 | 5e-05 | 4e-05 | 3e-05 | 3e-05 | 6e-05 | 1e-05 | 0.01027 | 4e-05 | 0.00013 | 0.00027 | 4e-05 | 0.00024 | 2e-05 | 1e-05 | 2e-05 | 2e-05 | 0.00031 | 0.00038 | 0.00011 | 2e-05 | 3e-05 | 0.001 | 5e-05 | Random accuracy unbiased |
| TN | 959 | 991 | 1022 | 1018 | 1017 | 1014 | 1015 | 1021 | 1006 | 1017 | 986 | 973 | 947 | 968 | 994 | 1007 | 997 | 1022 | 1014 | 970 | 876 | 1010 | 965 | 988 | 1015 | 1014 | 999 | 1009 | 941 | 1019 | 1016 | 1018 | 1018 | 1017 | 1023 | 921 | 1019 | 1013 | 994 | 1019 | 994 | 1017 | 1019 | 1017 | 1020 | 1000 | 1002 | 1011 | 1021 | 1014 | 961 | 1018 | True negative/correct rejection |
| TNR | 0.96189 | 0.99398 | 1.0 | 1.0 | 1.0 | 0.99412 | 0.99607 | 0.99902 | 0.99802 | 1.0 | 0.99596 | 0.97009 | 0.94511 | 0.96897 | 0.99899 | 0.99212 | 0.99204 | 0.99902 | 0.99705 | 0.99692 | 0.98983 | 0.99901 | 0.98169 | 0.98406 | 0.99607 | 0.99901 | 0.99701 | 0.99311 | 0.98845 | 1.0 | 0.99413 | 0.99609 | 0.99511 | 0.99804 | 0.99902 | 0.99892 | 1.0 | 0.99705 | 0.99103 | 0.99902 | 0.99499 | 0.99706 | 1.0 | 0.99902 | 1.0 | 0.98912 | 0.99306 | 1.0 | 1.0 | 0.99803 | 0.95337 | 0.99902 | Specificity or true negative rate |
| TON | 984 | 1001 | 1026 | 1023 | 1018 | 1019 | 1022 | 1024 | 1007 | 1021 | 991 | 994 | 959 | 969 | 996 | 1014 | 1012 | 1024 | 1022 | 993 | 882 | 1014 | 978 | 1010 | 1016 | 1025 | 1011 | 1010 | 971 | 1019 | 1017 | 1018 | 1018 | 1017 | 1023 | 922 | 1020 | 1013 | 1015 | 1019 | 1021 | 1023 | 1026 | 1025 | 1023 | 1005 | 1003 | 1019 | 1022 | 1024 | 979 | 1019 | Test outcome negative |
| TOP | 42 | 25 | 0 | 3 | 8 | 7 | 4 | 2 | 19 | 5 | 35 | 32 | 67 | 57 | 30 | 12 | 14 | 2 | 4 | 33 | 144 | 12 | 48 | 16 | 10 | 1 | 15 | 16 | 55 | 7 | 9 | 8 | 8 | 9 | 3 | 104 | 6 | 13 | 11 | 7 | 5 | 3 | 0 | 1 | 3 | 21 | 23 | 7 | 4 | 2 | 47 | 7 | Test outcome positive |
| TP | 4 | 19 | 0 | 3 | 8 | 1 | 0 | 1 | 17 | 5 | 31 | 2 | 12 | 26 | 29 | 4 | 6 | 1 | 1 | 30 | 135 | 11 | 30 | 0 | 6 | 0 | 12 | 9 | 44 | 7 | 3 | 4 | 3 | 7 | 2 | 103 | 6 | 10 | 2 | 6 | 0 | 0 | 0 | 0 | 3 | 10 | 16 | 7 | 4 | 0 | 0 | 6 | True positive/hit |
| TPR | 0.13793 | 0.65517 | 0.0 | 0.375 | 0.88889 | 0.16667 | 0.0 | 0.25 | 0.94444 | 0.55556 | 0.86111 | 0.08696 | 0.5 | 0.96296 | 0.93548 | 0.36364 | 0.28571 | 0.33333 | 0.11111 | 0.56604 | 0.95745 | 0.73333 | 0.69767 | 0.0 | 0.85714 | 0.0 | 0.5 | 0.9 | 0.59459 | 1.0 | 0.75 | 1.0 | 1.0 | 1.0 | 1.0 | 0.99038 | 0.85714 | 1.0 | 0.08696 | 1.0 | 0.0 | 0.0 | 0.0 | 0.0 | 0.5 | 0.66667 | 0.94118 | 0.46667 | 0.8 | 0.0 | 0.0 | 0.85714 | Sensitivity, recall, hit rate, or true positive rate |
| Y | 0.09982 | 0.64915 | 0.0 | 0.375 | 0.88889 | 0.16078 | -0.00393 | 0.24902 | 0.94246 | 0.55556 | 0.85707 | 0.05705 | 0.44511 | 0.93193 | 0.93448 | 0.35575 | 0.27775 | 0.33236 | 0.10816 | 0.56295 | 0.94728 | 0.73234 | 0.67936 | -0.01594 | 0.85322 | -0.00099 | 0.49701 | 0.89311 | 0.58304 | 1.0 | 0.74413 | 0.99609 | 0.99511 | 0.99804 | 0.99902 | 0.9893 | 0.85714 | 0.99705 | 0.07798 | 0.99902 | -0.00501 | -0.00294 | 0.0 | -0.00098 | 0.5 | 0.65579 | 0.93424 | 0.46667 | 0.8 | -0.00197 | -0.04663 | 0.85616 | Youden index |
| dInd | 0.86291 | 0.34488 | 1.0 | 0.625 | 0.11111 | 0.83335 | 1.00001 | 0.75 | 0.05559 | 0.44444 | 0.13895 | 0.91353 | 0.503 | 0.04832 | 0.06452 | 0.63641 | 0.71433 | 0.66667 | 0.88889 | 0.43397 | 0.04375 | 0.26667 | 0.30288 | 1.00013 | 0.14291 | 1.0 | 0.50001 | 0.10024 | 0.40557 | 0.0 | 0.25007 | 0.00391 | 0.00489 | 0.00196 | 0.00098 | 0.00968 | 0.14286 | 0.00295 | 0.91309 | 0.00098 | 1.00001 | 1.0 | 1.0 | 1.0 | 0.5 | 0.33351 | 0.05923 | 0.53333 | 0.2 | 1.0 | 1.00109 | 0.14286 | Distance index |
| sInd | 0.38983 | 0.75613 | 0.29289 | 0.55806 | 0.92143 | 0.41073 | 0.29289 | 0.46967 | 0.96069 | 0.68573 | 0.90175 | 0.35403 | 0.64432 | 0.96583 | 0.95437 | 0.54999 | 0.49489 | 0.52859 | 0.37146 | 0.69313 | 0.96906 | 0.81144 | 0.78583 | 0.2928 | 0.89895 | 0.29289 | 0.64644 | 0.92912 | 0.71322 | 1.0 | 0.82317 | 0.99723 | 0.99654 | 0.99861 | 0.99931 | 0.99316 | 0.89898 | 0.99791 | 0.35435 | 0.99931 | 0.29288 | 0.29289 | 0.29289 | 0.29289 | 0.64645 | 0.76417 | 0.95812 | 0.62288 | 0.85858 | 0.29289 | 0.29212 | 0.89898 | Similarity index |

Generated By PyCM Version 3.4
