# Supplementary material for: TopEC: prediction of Enzyme Commission classes by 3D graph neural networks and localized 3D protein descriptor
Source: Nat Commun. 2025 Mar 20;16:2737. doi: 10.1038/s41467-025-57324-5 (PMC11923149; doi:10.1038/s41467-025-57324-5)
Supplement: Supplementary file 3 — Supplementary Data 1 [file 41467_2025_57324_MOESM3_ESM.zip › Data_S1/table1/hierarchical/TopEC_distance_FOLD_2digs.html]

PyCM Report


# PyCM Report

## Dataset Type :

- Multi-Class Classification
- Imbalanced

Note 1 : Recommended statistics for this type of classification highlighted in aqua

Note 2 : The recommender system assumes that the input is the result of classification over the whole data rather than just a part of it.
If the confusion matrix is the result of test data classification, the recommendation is not valid.

## Confusion Matrix :

|  |  |  |  |  |  |  |  |  |  |  |  |  |  |  |  |  |  |  |  |  |  |  |  |  |  |  |  |  |  |  |  |  |  |  |  |  |  |  |  |  |  |  |  |  |  |  |  |  |  |  |  |  |  |  |  |  |  |  |  |  |  |  |  |  |  |  |  |  |  |  |  |  |  |  |  |  |  |  |  |  |  |  |  |  |  |  |  |  |  |  |  |  |  |  |  |  |  |  |  |  |  |  |  |  |  |  |  |  |  |  |  |  |  |  |  |  |  |  |  |  |  |  |  |  |  |  |  |  |  |  |  |  |  |  |  |  |  |  |  |  |  |  |  |  |  |  |  |  |  |  |  |  |  |  |  |  |  |  |  |  |  |  |  |  |  |  |  |  |  |  |  |  |  |  |  |  |  |  |  |  |  |  |  |  |  |  |  |  |  |  |  |  |  |  |  |  |  |  |  |  |  |  |  |  |  |  |  |  |  |  |  |  |  |  |  |  |  |  |  |  |  |  |  |  |  |  |  |  |  |  |  |  |  |  |  |  |  |  |  |  |  |  |  |  |  |  |  |  |  |  |  |  |  |  |  |  |  |  |  |  |  |  |  |  |  |  |  |  |  |  |  |  |  |  |  |  |  |  |  |  |  |  |  |  |  |  |  |  |  |  |  |  |  |  |  |  |  |  |  |  |  |  |  |  |  |  |  |  |  |  |  |  |  |  |  |  |  |  |  |  |  |  |  |  |  |  |  |  |  |  |  |  |  |  |  |  |  |  |  |  |  |  |  |  |  |  |  |  |  |  |  |  |  |  |  |  |  |  |  |  |  |  |  |  |  |  |  |  |  |  |  |  |  |  |  |  |  |  |  |  |  |  |  |  |  |  |  |  |  |  |  |  |  |  |  |  |  |  |  |  |  |  |  |  |  |  |  |  |  |  |  |  |  |  |  |  |  |  |  |  |  |  |  |  |  |  |  |  |  |  |  |  |  |  |  |  |  |  |  |  |  |  |  |  |  |  |  |  |  |  |  |  |  |  |  |  |  |  |  |  |  |  |  |  |  |  |  |  |  |  |  |  |  |  |  |  |  |  |  |  |  |  |  |  |  |  |  |  |  |  |  |  |  |  |  |  |  |  |  |  |  |  |  |  |  |  |  |  |  |  |  |  |  |  |  |  |  |  |  |  |  |  |  |  |  |  |  |  |  |  |  |  |  |  |  |  |  |  |  |  |  |  |  |  |  |  |  |  |  |  |  |  |  |  |  |  |  |  |  |  |  |  |  |  |  |  |  |  |  |  |  |  |  |  |  |  |  |  |  |  |  |  |  |  |  |  |  |  |  |  |  |  |  |  |  |  |  |  |  |  |  |  |  |  |  |  |  |  |  |  |  |  |  |  |  |  |  |  |  |  |  |  |  |  |  |  |  |  |  |  |  |  |  |  |  |  |  |  |  |  |  |  |  |  |  |  |  |  |  |  |  |  |  |  |  |  |  |  |  |  |  |  |  |  |  |  |  |  |  |  |  |  |  |  |  |  |  |  |  |  |  |  |  |  |  |  |  |  |  |  |  |  |  |  |  |  |  |  |  |  |  |  |  |  |  |  |  |  |  |  |  |  |  |  |  |  |  |  |  |  |  |  |  |  |  |  |  |  |  |  |  |  |  |  |  |  |  |  |  |  |  |  |  |  |  |  |  |  |  |  |  |  |  |  |  |  |  |  |  |  |  |  |  |  |  |  |  |  |  |  |  |  |  |  |  |  |  |  |  |  |  |  |  |  |  |  |  |  |  |  |  |  |  |  |  |  |  |  |  |  |  |  |  |  |  |  |  |  |  |  |  |  |  |  |  |  |  |  |  |  |  |  |  |  |  |  |  |  |  |  |  |  |  |  |  |  |  |  |  |  |  |  |  |  |  |  |  |  |  |  |  |  |  |  |  |  |  |  |  |  |  |  |  |  |  |  |  |  |  |  |  |  |  |  |  |  |  |  |  |  |  |  |  |  |  |  |  |  |  |  |  |  |  |  |  |  |  |  |  |  |  |  |  |  |  |  |  |  |  |  |  |  |  |  |  |  |  |  |  |  |  |  |  |  |  |  |  |  |  |  |  |  |  |  |  |  |  |  |  |  |  |  |  |  |  |  |  |  |  |  |  |  |  |  |  |  |  |  |  |  |  |  |  |  |  |  |  |  |  |  |  |  |  |  |  |  |  |  |  |  |  |  |  |  |  |  |  |  |  |  |  |  |  |  |  |  |  |  |  |  |  |  |  |  |  |  |  |  |  |  |  |  |  |  |  |  |  |  |  |  |  |  |  |  |  |
| --- | --- | --- | --- | --- | --- | --- | --- | --- | --- | --- | --- | --- | --- | --- | --- | --- | --- | --- | --- | --- | --- | --- | --- | --- | --- | --- | --- | --- | --- | --- | --- | --- | --- | --- | --- | --- | --- | --- | --- | --- | --- | --- | --- | --- | --- | --- | --- | --- | --- | --- | --- | --- | --- | --- | --- | --- | --- | --- | --- | --- | --- | --- | --- | --- | --- | --- | --- | --- | --- | --- | --- | --- | --- | --- | --- | --- | --- | --- | --- | --- | --- | --- | --- | --- | --- | --- | --- | --- | --- | --- | --- | --- | --- | --- | --- | --- | --- | --- | --- | --- | --- | --- | --- | --- | --- | --- | --- | --- | --- | --- | --- | --- | --- | --- | --- | --- | --- | --- | --- | --- | --- | --- | --- | --- | --- | --- | --- | --- | --- | --- | --- | --- | --- | --- | --- | --- | --- | --- | --- | --- | --- | --- | --- | --- | --- | --- | --- | --- | --- | --- | --- | --- | --- | --- | --- | --- | --- | --- | --- | --- | --- | --- | --- | --- | --- | --- | --- | --- | --- | --- | --- | --- | --- | --- | --- | --- | --- | --- | --- | --- | --- | --- | --- | --- | --- | --- | --- | --- | --- | --- | --- | --- | --- | --- | --- | --- | --- | --- | --- | --- | --- | --- | --- | --- | --- | --- | --- | --- | --- | --- | --- | --- | --- | --- | --- | --- | --- | --- | --- | --- | --- | --- | --- | --- | --- | --- | --- | --- | --- | --- | --- | --- | --- | --- | --- | --- | --- | --- | --- | --- | --- | --- | --- | --- | --- | --- | --- | --- | --- | --- | --- | --- | --- | --- | --- | --- | --- | --- | --- | --- | --- | --- | --- | --- | --- | --- | --- | --- | --- | --- | --- | --- | --- | --- | --- | --- | --- | --- | --- | --- | --- | --- | --- | --- | --- | --- | --- | --- | --- | --- | --- | --- | --- | --- | --- | --- | --- | --- | --- | --- | --- | --- | --- | --- | --- | --- | --- | --- | --- | --- | --- | --- | --- | --- | --- | --- | --- | --- | --- | --- | --- | --- | --- | --- | --- | --- | --- | --- | --- | --- | --- | --- | --- | --- | --- | --- | --- | --- | --- | --- | --- | --- | --- | --- | --- | --- | --- | --- | --- | --- | --- | --- | --- | --- | --- | --- | --- | --- | --- | --- | --- | --- | --- | --- | --- | --- | --- | --- | --- | --- | --- | --- | --- | --- | --- | --- | --- | --- | --- | --- | --- | --- | --- | --- | --- | --- | --- | --- | --- | --- | --- | --- | --- | --- | --- | --- | --- | --- | --- | --- | --- | --- | --- | --- | --- | --- | --- | --- | --- | --- | --- | --- | --- | --- | --- | --- | --- | --- | --- | --- | --- | --- | --- | --- | --- | --- | --- | --- | --- | --- | --- | --- | --- | --- | --- | --- | --- | --- | --- | --- | --- | --- | --- | --- | --- | --- | --- | --- | --- | --- | --- | --- | --- | --- | --- | --- | --- | --- | --- | --- | --- | --- | --- | --- | --- | --- | --- | --- | --- | --- | --- | --- | --- | --- | --- | --- | --- | --- | --- | --- | --- | --- | --- | --- | --- | --- | --- | --- | --- | --- | --- | --- | --- | --- | --- | --- | --- | --- | --- | --- | --- | --- | --- | --- | --- | --- | --- | --- | --- | --- | --- | --- | --- | --- | --- | --- | --- | --- | --- | --- | --- | --- | --- | --- | --- | --- | --- | --- | --- | --- | --- | --- | --- | --- | --- | --- | --- | --- | --- | --- | --- | --- | --- | --- | --- | --- | --- | --- | --- | --- | --- | --- | --- | --- | --- | --- | --- | --- | --- | --- | --- | --- | --- | --- | --- | --- | --- | --- | --- | --- | --- | --- | --- | --- | --- | --- | --- | --- | --- | --- | --- | --- | --- | --- | --- | --- | --- | --- | --- | --- | --- | --- | --- | --- | --- | --- | --- | --- | --- | --- | --- | --- | --- | --- | --- | --- | --- | --- | --- | --- | --- | --- | --- | --- | --- | --- | --- | --- | --- | --- | --- | --- | --- | --- | --- | --- | --- | --- | --- | --- | --- | --- | --- | --- | --- | --- | --- | --- | --- | --- | --- | --- | --- | --- | --- | --- | --- | --- | --- | --- | --- | --- | --- | --- | --- | --- | --- | --- | --- | --- | --- | --- | --- | --- | --- | --- | --- | --- | --- | --- | --- | --- | --- | --- | --- | --- | --- | --- | --- | --- | --- | --- | --- | --- | --- | --- | --- | --- | --- | --- | --- | --- | --- | --- | --- | --- | --- | --- | --- | --- | --- | --- | --- | --- | --- | --- | --- | --- | --- | --- | --- | --- | --- | --- | --- | --- | --- | --- | --- | --- | --- | --- | --- | --- | --- | --- | --- | --- | --- | --- | --- | --- | --- | --- | --- | --- | --- | --- | --- | --- | --- | --- | --- | --- | --- | --- | --- | --- | --- | --- | --- | --- | --- | --- | --- | --- | --- | --- | --- | --- | --- | --- | --- | --- | --- | --- | --- | --- | --- | --- | --- | --- | --- | --- | --- | --- | --- | --- | --- | --- | --- | --- | --- | --- | --- | --- | --- | --- | --- | --- | --- | --- | --- | --- | --- | --- | --- | --- | --- | --- | --- | --- | --- | --- | --- | --- | --- | --- | --- | --- | --- | --- | --- | --- | --- | --- | --- | --- | --- | --- | --- | --- | --- | --- | --- | --- | --- | --- | --- | --- | --- | --- | --- | --- | --- | --- | --- | --- | --- | --- | --- | --- | --- | --- | --- | --- | --- | --- | --- | --- | --- | --- | --- | --- | --- | --- | --- | --- | --- | --- | --- | --- | --- | --- | --- | --- | --- | --- | --- | --- | --- | --- | --- | --- | --- | --- | --- | --- | --- | --- | --- | --- | --- | --- | --- | --- | --- | --- | --- | --- | --- | --- | --- | --- | --- | --- | --- | --- | --- | --- | --- | --- | --- | --- | --- | --- | --- | --- | --- | --- | --- | --- | --- | --- | --- | --- | --- | --- | --- | --- | --- | --- | --- | --- | --- | --- | --- | --- | --- | --- | --- | --- | --- | --- | --- | --- | --- | --- | --- | --- | --- | --- | --- | --- | --- | --- | --- | --- | --- | --- | --- | --- | --- | --- | --- | --- | --- | --- | --- | --- | --- | --- | --- | --- | --- | --- | --- | --- | --- | --- | --- | --- | --- | --- | --- | --- | --- | --- | --- | --- | --- | --- | --- | --- | --- | --- | --- | --- | --- | --- | --- | --- | --- | --- | --- | --- | --- | --- | --- | --- | --- | --- | --- | --- | --- | --- | --- | --- | --- | --- | --- | --- | --- | --- | --- | --- | --- | --- | --- | --- | --- | --- | --- | --- | --- |
| Actual | Predict  |  |  |  |  |  |  |  |  |  |  |  |  |  |  |  |  |  |  |  |  |  |  |  |  |  |  |  |  |  |  |  |  | | --- | --- | --- | --- | --- | --- | --- | --- | --- | --- | --- | --- | --- | --- | --- | --- | --- | --- | --- | --- | --- | --- | --- | --- | --- | --- | --- | --- | --- | --- | --- | --- | |  | 0 | 1 | 2 | 3 | 4 | 5 | 6 | 7 | 8 | 9 | 10 | 11 | 12 | 13 | 14 | 15 | 16 | 17 | 18 | 19 | 20 | 21 | 22 | 23 | 24 | 25 | 26 | 27 | 28 | 29 | 30 | | 0 | 4 | 3 | 0 | 0 | 0 | 0 | 0 | 0 | 0 | 16 | 0 | 0 | 2 | 0 | 2 | 0 | 0 | 0 | 0 | 0 | 1 | 0 | 0 | 0 | 0 | 0 | 0 | 0 | 0 | 1 | 0 | | 1 | 4 | 19 | 0 | 0 | 0 | 0 | 0 | 0 | 0 | 3 | 0 | 0 | 0 | 0 | 0 | 0 | 0 | 1 | 0 | 0 | 1 | 0 | 0 | 0 | 0 | 0 | 0 | 0 | 0 | 1 | 0 | | 2 | 2 | 0 | 0 | 0 | 0 | 0 | 0 | 0 | 0 | 1 | 0 | 0 | 0 | 0 | 0 | 0 | 0 | 0 | 0 | 0 | 1 | 0 | 0 | 0 | 0 | 0 | 0 | 0 | 0 | 0 | 0 | | 3 | 1 | 0 | 0 | 11 | 0 | 0 | 0 | 0 | 0 | 1 | 2 | 0 | 0 | 0 | 0 | 0 | 0 | 0 | 0 | 0 | 0 | 0 | 0 | 0 | 0 | 0 | 0 | 0 | 0 | 2 | 0 | | 4 | 0 | 0 | 0 | 0 | 1 | 0 | 0 | 0 | 0 | 0 | 0 | 0 | 2 | 0 | 2 | 0 | 1 | 0 | 0 | 0 | 0 | 0 | 0 | 0 | 0 | 0 | 0 | 0 | 0 | 0 | 0 | | 5 | 2 | 0 | 0 | 0 | 0 | 1 | 1 | 0 | 0 | 0 | 0 | 1 | 0 | 0 | 0 | 1 | 0 | 0 | 0 | 0 | 1 | 2 | 0 | 0 | 0 | 1 | 0 | 0 | 0 | 1 | 0 | | 6 | 0 | 0 | 0 | 0 | 0 | 0 | 22 | 0 | 0 | 2 | 0 | 0 | 0 | 0 | 0 | 2 | 0 | 0 | 0 | 0 | 0 | 0 | 0 | 0 | 0 | 0 | 0 | 0 | 0 | 0 | 1 | | 7 | 2 | 0 | 0 | 0 | 0 | 0 | 0 | 31 | 0 | 3 | 0 | 0 | 0 | 0 | 0 | 0 | 0 | 0 | 0 | 0 | 0 | 0 | 0 | 0 | 0 | 0 | 0 | 0 | 0 | 0 | 0 | | 8 | 0 | 0 | 0 | 0 | 0 | 0 | 0 | 0 | 2 | 5 | 3 | 0 | 0 | 0 | 2 | 0 | 0 | 0 | 0 | 0 | 0 | 0 | 0 | 0 | 0 | 0 | 0 | 0 | 0 | 11 | 0 | | 9 | 0 | 0 | 0 | 0 | 0 | 0 | 0 | 2 | 3 | 12 | 0 | 1 | 0 | 0 | 3 | 0 | 0 | 0 | 0 | 3 | 0 | 0 | 0 | 0 | 0 | 0 | 0 | 0 | 0 | 0 | 0 | | 10 | 0 | 0 | 0 | 0 | 0 | 0 | 0 | 0 | 0 | 0 | 26 | 0 | 0 | 0 | 1 | 0 | 0 | 0 | 0 | 0 | 0 | 0 | 0 | 0 | 0 | 0 | 0 | 0 | 0 | 0 | 0 | | 11 | 1 | 0 | 0 | 0 | 3 | 0 | 0 | 0 | 0 | 2 | 0 | 33 | 0 | 0 | 0 | 0 | 1 | 0 | 0 | 0 | 0 | 0 | 0 | 0 | 0 | 0 | 0 | 0 | 0 | 2 | 0 | | 12 | 2 | 0 | 0 | 0 | 0 | 0 | 0 | 0 | 9 | 0 | 0 | 0 | 6 | 0 | 1 | 0 | 0 | 0 | 0 | 0 | 1 | 1 | 0 | 0 | 0 | 0 | 0 | 0 | 0 | 1 | 0 | | 13 | 1 | 0 | 0 | 0 | 0 | 0 | 0 | 0 | 1 | 0 | 0 | 0 | 0 | 1 | 0 | 0 | 0 | 0 | 0 | 0 | 0 | 0 | 0 | 0 | 0 | 0 | 0 | 0 | 0 | 0 | 0 | | 14 | 6 | 1 | 0 | 0 | 0 | 0 | 0 | 0 | 10 | 4 | 2 | 1 | 1 | 0 | 219 | 0 | 2 | 2 | 0 | 1 | 5 | 0 | 0 | 0 | 0 | 0 | 0 | 0 | 0 | 7 | 0 | | 15 | 0 | 0 | 0 | 0 | 0 | 0 | 0 | 0 | 0 | 0 | 0 | 0 | 0 | 0 | 0 | 0 | 0 | 0 | 5 | 0 | 0 | 0 | 0 | 0 | 10 | 5 | 0 | 0 | 0 | 2 | 0 | | 16 | 4 | 0 | 0 | 0 | 0 | 0 | 0 | 0 | 0 | 0 | 1 | 4 | 0 | 0 | 2 | 0 | 31 | 4 | 0 | 2 | 0 | 0 | 0 | 0 | 0 | 0 | 0 | 0 | 0 | 4 | 0 | | 17 | 2 | 0 | 0 | 0 | 0 | 0 | 1 | 0 | 0 | 1 | 1 | 1 | 2 | 0 | 3 | 4 | 2 | 51 | 1 | 5 | 2 | 0 | 0 | 0 | 0 | 0 | 0 | 0 | 0 | 5 | 0 | | 18 | 0 | 0 | 0 | 0 | 0 | 0 | 0 | 0 | 1 | 0 | 0 | 0 | 0 | 0 | 0 | 0 | 0 | 0 | 3 | 0 | 0 | 0 | 0 | 0 | 0 | 0 | 0 | 0 | 0 | 0 | 0 | | 19 | 0 | 0 | 0 | 0 | 0 | 0 | 0 | 0 | 0 | 0 | 0 | 0 | 0 | 0 | 2 | 0 | 0 | 0 | 0 | 125 | 0 | 0 | 0 | 0 | 0 | 0 | 0 | 0 | 0 | 0 | 0 | | 20 | 3 | 0 | 0 | 0 | 2 | 0 | 0 | 2 | 0 | 1 | 0 | 0 | 1 | 1 | 3 | 2 | 2 | 1 | 0 | 0 | 18 | 0 | 1 | 0 | 0 | 1 | 0 | 0 | 0 | 1 | 0 | | 21 | 0 | 0 | 0 | 0 | 1 | 0 | 0 | 0 | 0 | 1 | 18 | 1 | 0 | 0 | 1 | 5 | 1 | 1 | 0 | 1 | 1 | 4 | 0 | 0 | 0 | 0 | 0 | 0 | 0 | 5 | 0 | | 22 | 4 | 1 | 0 | 0 | 0 | 0 | 0 | 0 | 0 | 0 | 0 | 0 | 0 | 0 | 0 | 0 | 2 | 1 | 0 | 0 | 0 | 0 | 0 | 0 | 0 | 0 | 0 | 0 | 0 | 0 | 0 | | 23 | 0 | 0 | 0 | 0 | 0 | 0 | 0 | 0 | 1 | 0 | 0 | 0 | 0 | 0 | 0 | 0 | 0 | 0 | 0 | 0 | 0 | 0 | 0 | 3 | 0 | 0 | 0 | 0 | 0 | 2 | 0 | | 24 | 0 | 0 | 0 | 0 | 0 | 0 | 0 | 0 | 1 | 0 | 0 | 0 | 0 | 0 | 0 | 1 | 0 | 0 | 0 | 0 | 0 | 1 | 0 | 0 | 10 | 0 | 0 | 0 | 2 | 0 | 0 | | 25 | 0 | 0 | 0 | 0 | 0 | 0 | 0 | 0 | 0 | 0 | 0 | 0 | 0 | 0 | 0 | 0 | 0 | 0 | 0 | 0 | 0 | 0 | 0 | 0 | 0 | 16 | 0 | 0 | 0 | 1 | 0 | | 26 | 3 | 1 | 0 | 0 | 0 | 0 | 0 | 0 | 0 | 1 | 0 | 0 | 0 | 0 | 0 | 1 | 0 | 1 | 0 | 0 | 0 | 0 | 0 | 0 | 1 | 0 | 7 | 0 | 0 | 0 | 0 | | 27 | 0 | 0 | 0 | 0 | 0 | 0 | 0 | 0 | 0 | 0 | 0 | 0 | 0 | 0 | 0 | 0 | 0 | 0 | 0 | 1 | 0 | 0 | 0 | 0 | 0 | 0 | 0 | 4 | 0 | 0 | 0 | | 28 | 1 | 0 | 0 | 0 | 0 | 1 | 0 | 0 | 4 | 0 | 4 | 0 | 0 | 0 | 0 | 0 | 0 | 0 | 0 | 0 | 0 | 0 | 0 | 0 | 0 | 0 | 0 | 0 | 0 | 0 | 0 | | 29 | 0 | 0 | 0 | 0 | 0 | 4 | 0 | 0 | 0 | 14 | 0 | 0 | 0 | 0 | 0 | 0 | 0 | 0 | 0 | 0 | 0 | 0 | 0 | 0 | 0 | 0 | 0 | 0 | 0 | 0 | 0 | | 30 | 0 | 0 | 0 | 0 | 0 | 0 | 0 | 0 | 0 | 0 | 0 | 0 | 0 | 0 | 0 | 0 | 0 | 0 | 0 | 0 | 0 | 0 | 0 | 0 | 0 | 0 | 0 | 0 | 0 | 1 | 6 | |

## Overall Statistics :

|  |  |
| --- | --- |
| 95% CI | (0.61992,0.67833) |
| ACC Macro | 0.97736 |
| ARI | 0.67025 |
| AUNP | 0.81577 |
| AUNU | 0.73471 |
| Bangdiwala B | 0.70855 |
| Bennett S | 0.63743 |
| CBA | 0.42164 |
| CSI | None |
| Chi-Squared | None |
| Chi-Squared DF | 900 |
| Conditional Entropy | 1.39526 |
| Cramer V | None |
| Cross Entropy | 4.20382 |
| F1 Macro | 0.47837 |
| F1 Micro | 0.64912 |
| FNR Macro | 0.51869 |
| FNR Micro | 0.35088 |
| FPR Macro | 0.01189 |
| FPR Micro | 0.0117 |
| Gwet AC1 | 0.63824 |
| Hamming Loss | 0.35088 |
| Joint Entropy | 5.47517 |
| KL Divergence | None |
| Kappa | 0.61172 |
| Kappa 95% CI | (0.5794,0.64403) |
| Kappa No Prevalence | 0.29825 |
| Kappa Standard Error | 0.01649 |
| Kappa Unbiased | 0.61106 |
| Krippendorff Alpha | 0.61125 |
| Lambda A | 0.5817 |
| Lambda B | 0.63567 |
| Mutual Information | 2.61226 |
| NIR | 0.25439 |
| Overall ACC | 0.64912 |
| Overall CEN | 0.25176 |
| Overall J | (11.68907,0.37707) |
| Overall MCC | 0.61378 |
| Overall MCEN | 0.34285 |
| Overall RACC | 0.09634 |
| Overall RACCU | 0.09786 |
| P-Value | 0.0 |
| PPV Macro | None |
| PPV Micro | 0.64912 |
| Pearson C | None |
| Phi-Squared | None |
| RCI | 0.64027 |
| RR | 33.09677 |
| Reference Entropy | 4.07991 |
| Response Entropy | 4.00752 |
| SOA1(Landis & Koch) | Substantial |
| SOA2(Fleiss) | Intermediate to Good |
| SOA3(Altman) | Good |
| SOA4(Cicchetti) | Good |
| SOA5(Cramer) | None |
| SOA6(Matthews) | Moderate |
| Scott PI | 0.61106 |
| Standard Error | 0.0149 |
| TNR Macro | 0.98811 |
| TNR Micro | 0.9883 |
| TPR Macro | 0.48131 |
| TPR Micro | 0.64912 |
| Zero-one Loss | 360 |

## Class Statistics :

|  |  |  |  |  |  |  |  |  |  |  |  |  |  |  |  |  |  |  |  |  |  |  |  |  |  |  |  |  |  |  |  |  |
| --- | --- | --- | --- | --- | --- | --- | --- | --- | --- | --- | --- | --- | --- | --- | --- | --- | --- | --- | --- | --- | --- | --- | --- | --- | --- | --- | --- | --- | --- | --- | --- | --- |
| Class | 0 | 1 | 2 | 3 | 4 | 5 | 6 | 7 | 8 | 9 | 10 | 11 | 12 | 13 | 14 | 15 | 16 | 17 | 18 | 19 | 20 | 21 | 22 | 23 | 24 | 25 | 26 | 27 | 28 | 29 | 30 | Description |
| ACC | 0.9386 | 0.98441 | 0.9961 | 0.99415 | 0.98928 | 0.98538 | 0.99318 | 0.99123 | 0.95029 | 0.9347 | 0.96881 | 0.98246 | 0.97758 | 0.99708 | 0.93762 | 0.96296 | 0.96881 | 0.96004 | 0.99318 | 0.98538 | 0.96686 | 0.96101 | 0.99123 | 0.99708 | 0.98441 | 0.9922 | 0.9922 | 0.99903 | 0.9883 | 0.93665 | 0.99805 | Accuracy |
| AGF | 0.35077 | 0.81704 | 0.0 | 0.83241 | 0.40058 | 0.31483 | 0.91086 | 0.92829 | 0.28071 | 0.60022 | 0.88443 | 0.88234 | 0.54955 | 0.59709 | 0.90038 | 0.0 | 0.77975 | 0.80162 | 0.77384 | 0.98129 | 0.68694 | 0.33995 | 0.0 | 0.74448 | 0.78325 | 0.93659 | 0.72049 | 0.91251 | 0.0 | 0.0 | 0.92537 | Adjusted F-score |
| AGM | 0.65878 | 0.89914 | 0 | 0.90138 | 0.69972 | 0.64605 | 0.94924 | 0.9604 | 0.62641 | 0.81474 | 0.96745 | 0.93546 | 0.75984 | 0.78774 | 0.932 | 0 | 0.87535 | 0.88451 | 0.92868 | 0.9852 | 0.82783 | 0.649 | 0 | 0.85312 | 0.89993 | 0.97981 | 0.8404 | 0.94708 | 0 | 0 | 0.96207 | Adjusted geometric mean |
| AM | 13 | -4 | -4 | -6 | 1 | -5 | -3 | -1 | 9 | 43 | 30 | 0 | -7 | -1 | -20 | -6 | -10 | -19 | 5 | 11 | -8 | -32 | -7 | -3 | 6 | 6 | -8 | -1 | -8 | 29 | 0 | Difference between automatic and manual classification |
| AUC | 0.54991 | 0.82458 | 0.5 | 0.82353 | 0.58039 | 0.54299 | 0.90641 | 0.92854 | 0.52852 | 0.72255 | 0.96597 | 0.88828 | 0.63888 | 0.66618 | 0.90516 | 0.49203 | 0.79243 | 0.80899 | 0.87206 | 0.9849 | 0.72418 | 0.54797 | 0.49951 | 0.75 | 0.82789 | 0.96712 | 0.73333 | 0.9 | 0.49902 | 0.47669 | 0.92808 | Area under the ROC curve |
| AUCI | Poor | Very Good | Poor | Very Good | Poor | Poor | Excellent | Excellent | Poor | Good | Excellent | Very Good | Fair | Fair | Excellent | Poor | Good | Very Good | Very Good | Excellent | Good | Poor | Poor | Good | Very Good | Excellent | Good | Excellent | Poor | Poor | Excellent | AUC value interpretation |
| AUPR | 0.11658 | 0.70759 | None | 0.82353 | 0.15476 | 0.12879 | 0.86574 | 0.87341 | 0.07473 | 0.33955 | 0.70955 | 0.78571 | 0.35714 | 0.41667 | 0.8739 | 0.0 | 0.66712 | 0.72611 | 0.54167 | 0.94502 | 0.52109 | 0.3 | 0.0 | 0.75 | 0.57143 | 0.81841 | 0.73333 | 0.9 | 0.0 | 0.0 | 0.85714 | Area under the PR curve |
| BCD | 0.00634 | 0.00195 | 0.00195 | 0.00292 | 0.00049 | 0.00244 | 0.00146 | 0.00049 | 0.00439 | 0.02096 | 0.01462 | 0.0 | 0.00341 | 0.00049 | 0.00975 | 0.00292 | 0.00487 | 0.00926 | 0.00244 | 0.00536 | 0.0039 | 0.01559 | 0.00341 | 0.00146 | 0.00292 | 0.00292 | 0.0039 | 0.00049 | 0.0039 | 0.01413 | 0.0 | Bray-Curtis dissimilarity |
| BM | 0.09982 | 0.64915 | 0.0 | 0.64706 | 0.16078 | 0.08598 | 0.81281 | 0.85707 | 0.05705 | 0.44511 | 0.93193 | 0.77657 | 0.27775 | 0.33236 | 0.81032 | -0.01594 | 0.58486 | 0.61799 | 0.74413 | 0.96979 | 0.44837 | 0.09594 | -0.00098 | 0.5 | 0.65579 | 0.93424 | 0.46667 | 0.8 | -0.00197 | -0.04663 | 0.85616 | Informedness or bookmaker informedness |
| CEN | 0.6096 | 0.23378 | 0.25394 | 0.15021 | 0.39003 | 0.49107 | 0.11853 | 0.10633 | 0.50458 | 0.50106 | 0.22722 | 0.19813 | 0.39394 | 0.23585 | 0.15376 | 0.52291 | 0.29851 | 0.28363 | 0.18614 | 0.06413 | 0.42874 | 0.43424 | 0.34854 | 0.14126 | 0.22337 | 0.13106 | 0.23794 | 0.05963 | 0.35297 | 0.60744 | 0.09208 | Confusion entropy |
| DOR | 4.03789 | 313.81667 | None | None | 33.8 | 20.2 | 2193.4 | 1528.3 | 3.08889 | 17.21818 | 811.87097 | 397.22222 | 49.85 | 511.0 | 176.10065 | 0.0 | 129.23377 | 144.34545 | 508.0 | 4259.61538 | 64.21978 | 27.27778 | 0.0 | None | 181.81818 | 2290.28571 | None | None | 0.0 | 0.0 | 6108.0 | Diagnostic odds ratio |
| DP | 0.33419 | 1.37649 | None | None | 0.84294 | 0.71968 | 1.84205 | 1.75555 | 0.27004 | 0.68144 | 1.60408 | 1.43292 | 0.93597 | 1.49323 | 1.23815 | None | 1.16406 | 1.19054 | 1.49182 | 2.00098 | 0.99662 | 0.7916 | None | None | 1.2458 | 1.8524 | None | None | None | None | 2.08728 | Discriminant power |
| DPI | Poor | Limited | None | None | Poor | Poor | Limited | Limited | Poor | Poor | Limited | Limited | Poor | Limited | Limited | None | Limited | Limited | Limited | Fair | Poor | Poor | None | None | Limited | Limited | None | None | None | None | Fair | Discriminant power interpretation |
| ERR | 0.0614 | 0.01559 | 0.0039 | 0.00585 | 0.01072 | 0.01462 | 0.00682 | 0.00877 | 0.04971 | 0.0653 | 0.03119 | 0.01754 | 0.02242 | 0.00292 | 0.06238 | 0.03704 | 0.03119 | 0.03996 | 0.00682 | 0.01462 | 0.03314 | 0.03899 | 0.00877 | 0.00292 | 0.01559 | 0.0078 | 0.0078 | 0.00097 | 0.0117 | 0.06335 | 0.00195 | Error rate |
| F0.5 | 0.10152 | 0.73643 | 0.0 | 0.90164 | 0.14706 | 0.14286 | 0.89431 | 0.88068 | 0.06623 | 0.20548 | 0.5098 | 0.78571 | 0.38961 | 0.45455 | 0.89388 | 0.0 | 0.70455 | 0.77508 | 0.375 | 0.92047 | 0.55215 | 0.27778 | 0.0 | 0.83333 | 0.50505 | 0.73394 | 0.81395 | 0.95238 | 0.0 | 0.0 | 0.85714 | F0.5 score |
| F1 | 0.11268 | 0.7037 | 0.0 | 0.78571 | 0.15385 | 0.11765 | 0.86275 | 0.87324 | 0.07273 | 0.26374 | 0.61905 | 0.78571 | 0.34286 | 0.4 | 0.87251 | 0.0 | 0.65957 | 0.71329 | 0.46154 | 0.9434 | 0.51429 | 0.16667 | 0.0 | 0.66667 | 0.55556 | 0.8 | 0.63636 | 0.88889 | 0.0 | 0.0 | 0.85714 | F1 score - harmonic mean of precision and sensitivity |
| F2 | 0.12658 | 0.67376 | 0.0 | 0.6962 | 0.16129 | 0.1 | 0.83333 | 0.86592 | 0.08065 | 0.3681 | 0.78788 | 0.78571 | 0.30612 | 0.35714 | 0.85214 | 0.0 | 0.62 | 0.66062 | 0.6 | 0.96749 | 0.48128 | 0.11905 | 0.0 | 0.55556 | 0.61728 | 0.87912 | 0.52239 | 0.83333 | 0.0 | 0.0 | 0.85714 | F2 score |
| FDR | 0.90476 | 0.24 | None | 0.0 | 0.85714 | 0.83333 | 0.08333 | 0.11429 | 0.9375 | 0.8209 | 0.54386 | 0.21429 | 0.57143 | 0.5 | 0.09129 | 1.0 | 0.2619 | 0.17742 | 0.66667 | 0.0942 | 0.41935 | 0.5 | 1.0 | 0.0 | 0.52381 | 0.30435 | 0.0 | 0.0 | 1.0 | 1.0 | 0.14286 | False discovery rate |
| FN | 25 | 10 | 4 | 6 | 5 | 10 | 5 | 5 | 21 | 12 | 1 | 9 | 15 | 2 | 42 | 22 | 21 | 30 | 1 | 2 | 21 | 36 | 8 | 3 | 5 | 1 | 8 | 1 | 10 | 18 | 1 | False negative/miss/type 2 error |
| FNR | 0.86207 | 0.34483 | 1.0 | 0.35294 | 0.83333 | 0.90909 | 0.18519 | 0.13889 | 0.91304 | 0.5 | 0.03704 | 0.21429 | 0.71429 | 0.66667 | 0.16092 | 1.0 | 0.40385 | 0.37037 | 0.25 | 0.01575 | 0.53846 | 0.9 | 1.0 | 0.5 | 0.33333 | 0.05882 | 0.53333 | 0.2 | 1.0 | 1.0 | 0.14286 | Miss rate or false negative rate |
| FOR | 0.02541 | 0.00999 | 0.0039 | 0.00591 | 0.00491 | 0.0098 | 0.00499 | 0.00505 | 0.02113 | 0.01251 | 0.00103 | 0.00915 | 0.01482 | 0.00195 | 0.0535 | 0.02178 | 0.02134 | 0.03112 | 0.00098 | 0.00225 | 0.02111 | 0.03536 | 0.0078 | 0.00293 | 0.00498 | 0.001 | 0.00785 | 0.00098 | 0.00977 | 0.01839 | 0.00098 | False omission rate |
| FP | 38 | 6 | 0 | 0 | 6 | 5 | 2 | 4 | 30 | 55 | 31 | 9 | 8 | 1 | 22 | 16 | 11 | 11 | 6 | 13 | 13 | 4 | 1 | 0 | 11 | 7 | 0 | 0 | 2 | 47 | 1 | False positive/type 1 error/false alarm |
| FPR | 0.03811 | 0.00602 | 0.0 | 0.0 | 0.00588 | 0.00493 | 0.002 | 0.00404 | 0.02991 | 0.05489 | 0.03103 | 0.00915 | 0.00796 | 0.00098 | 0.02876 | 0.01594 | 0.01129 | 0.01164 | 0.00587 | 0.01446 | 0.01317 | 0.00406 | 0.00098 | 0.0 | 0.01088 | 0.00694 | 0.0 | 0.0 | 0.00197 | 0.04663 | 0.00098 | Fall-out or false positive rate |
| G | 0.11461 | 0.70564 | None | 0.8044 | 0.1543 | 0.12309 | 0.86424 | 0.87333 | 0.07372 | 0.29925 | 0.66276 | 0.78571 | 0.34993 | 0.40825 | 0.8732 | 0.0 | 0.66334 | 0.71967 | 0.5 | 0.94421 | 0.51768 | 0.22361 | 0.0 | 0.70711 | 0.56344 | 0.80915 | 0.68313 | 0.89443 | 0.0 | 0.0 | 0.85714 | G-measure geometric mean of precision and sensitivity |
| GI | 0.09982 | 0.64915 | 0.0 | 0.64706 | 0.16078 | 0.08598 | 0.81281 | 0.85707 | 0.05705 | 0.44511 | 0.93193 | 0.77657 | 0.27775 | 0.33236 | 0.81032 | -0.01594 | 0.58486 | 0.61799 | 0.74413 | 0.96979 | 0.44837 | 0.09594 | -0.00098 | 0.5 | 0.65579 | 0.93424 | 0.46667 | 0.8 | -0.00197 | -0.04663 | 0.85616 | Gini index |
| GM | 0.36424 | 0.80699 | 0.0 | 0.8044 | 0.40705 | 0.30077 | 0.90177 | 0.92608 | 0.29044 | 0.68743 | 0.96596 | 0.88234 | 0.53239 | 0.57707 | 0.90275 | 0.0 | 0.76774 | 0.78886 | 0.86348 | 0.9849 | 0.67488 | 0.31559 | 0.0 | 0.70711 | 0.81204 | 0.96677 | 0.68313 | 0.89443 | 0.0 | 0.0 | 0.92537 | G-mean geometric mean of specificity and sensitivity |
| IBA | 0.02336 | 0.43059 | 0.0 | 0.41869 | 0.02859 | 0.00867 | 0.66422 | 0.74198 | 0.00986 | 0.26222 | 0.92748 | 0.61882 | 0.08324 | 0.11133 | 0.70725 | 0.0 | 0.35804 | 0.39906 | 0.56357 | 0.96877 | 0.21621 | 0.01036 | 0.0 | 0.25 | 0.44678 | 0.88615 | 0.21778 | 0.64 | 0.0 | 0.0 | 0.73481 | Index of balanced accuracy |
| ICSI | -0.76683 | 0.41517 | None | 0.64706 | -0.69048 | -0.74242 | 0.73148 | 0.74683 | -0.85054 | -0.3209 | 0.4191 | 0.57143 | -0.28571 | -0.16667 | 0.74779 | -1.0 | 0.33425 | 0.45221 | 0.08333 | 0.89005 | 0.04218 | -0.4 | -1.0 | 0.5 | 0.14286 | 0.63683 | 0.46667 | 0.8 | -1.0 | -1.0 | 0.71429 | Individual classification success index |
| IS | 1.75252 | 4.74891 | None | 5.91535 | 4.6105 | 3.95842 | 5.1224 | 4.6578 | 1.47925 | 2.93673 | 4.11548 | 4.26257 | 4.38811 | 7.41785 | 1.83681 | None | 3.86425 | 3.38119 | 6.41785 | 2.87139 | 3.93314 | 3.68089 | None | 7.41785 | 5.02554 | 5.39179 | 6.09592 | 7.68089 | None | None | 6.97307 | Information score |
| J | 0.0597 | 0.54286 | 0.0 | 0.64706 | 0.08333 | 0.0625 | 0.75862 | 0.775 | 0.03774 | 0.1519 | 0.44828 | 0.64706 | 0.2069 | 0.25 | 0.77385 | 0.0 | 0.49206 | 0.55435 | 0.3 | 0.89286 | 0.34615 | 0.09091 | 0.0 | 0.5 | 0.38462 | 0.66667 | 0.46667 | 0.8 | 0.0 | 0.0 | 0.75 | Jaccard index |
| LS | 3.36946 | 26.88828 | None | 60.35294 | 24.42857 | 15.54545 | 34.83333 | 25.24286 | 2.78804 | 7.65672 | 17.33333 | 19.19388 | 20.93878 | 171.0 | 3.57218 | 0.0 | 14.56319 | 10.41935 | 85.5 | 7.3177 | 15.27543 | 12.825 | 0.0 | 171.0 | 32.57143 | 41.98465 | 68.4 | 205.2 | 0.0 | 0.0 | 125.63265 | Lift score |
| MCC | 0.08349 | 0.69776 | None | 0.80202 | 0.14893 | 0.11614 | 0.86083 | 0.86879 | 0.04858 | 0.27231 | 0.65125 | 0.77657 | 0.339 | 0.40685 | 0.83246 | -0.01863 | 0.64746 | 0.69937 | 0.4973 | 0.93608 | 0.50088 | 0.21114 | -0.00277 | 0.70607 | 0.55589 | 0.80559 | 0.68044 | 0.89399 | -0.00438 | -0.02928 | 0.85616 | Matthews correlation coefficient |
| MCCI | Negligible | Moderate | None | Strong | Negligible | Negligible | Strong | Strong | Negligible | Negligible | Moderate | Strong | Weak | Weak | Strong | Negligible | Moderate | Moderate | Weak | Very Strong | Moderate | Negligible | Negligible | Strong | Moderate | Strong | Moderate | Strong | Negligible | Negligible | Strong | Matthews correlation coefficient interpretation |
| MCEN | 0.63268 | 0.31227 | 0.25394 | 0.2044 | 0.40461 | 0.50788 | 0.17517 | 0.1572 | 0.51492 | 0.54788 | 0.27917 | 0.28331 | 0.43902 | 0.25394 | 0.24108 | 0.52291 | 0.39575 | 0.39286 | 0.19712 | 0.10469 | 0.52968 | 0.4544 | 0.34854 | 0.16238 | 0.26037 | 0.17684 | 0.29909 | 0.07862 | 0.35297 | 0.60744 | 0.12697 | Modified confusion entropy |
| MK | 0.06983 | 0.75001 | None | 0.99409 | 0.13795 | 0.15686 | 0.91168 | 0.88067 | 0.04137 | 0.16659 | 0.45511 | 0.77657 | 0.41375 | 0.49805 | 0.85521 | -0.02178 | 0.71675 | 0.79146 | 0.33235 | 0.90354 | 0.55954 | 0.46464 | -0.0078 | 0.99707 | 0.47122 | 0.69466 | 0.99215 | 0.99902 | -0.00977 | -0.01839 | 0.85616 | Markedness |
| N | 997 | 997 | 1022 | 1009 | 1020 | 1015 | 999 | 990 | 1003 | 1002 | 999 | 984 | 1005 | 1023 | 765 | 1004 | 974 | 945 | 1022 | 899 | 987 | 986 | 1018 | 1020 | 1011 | 1009 | 1011 | 1021 | 1016 | 1008 | 1019 | Condition negative |
| NLR | 0.89623 | 0.34692 | 1.0 | 0.35294 | 0.83826 | 0.91359 | 0.18556 | 0.13945 | 0.94119 | 0.52904 | 0.03822 | 0.21626 | 0.72002 | 0.66732 | 0.16568 | 1.01619 | 0.40846 | 0.37473 | 0.25148 | 0.01598 | 0.54565 | 0.90367 | 1.00098 | 0.5 | 0.337 | 0.05923 | 0.53333 | 0.2 | 1.00197 | 1.04891 | 0.143 | Negative likelihood ratio |
| NLRI | Negligible | Poor | Negligible | Poor | Negligible | Negligible | Fair | Fair | Negligible | Negligible | Good | Poor | Negligible | Negligible | Fair | Negligible | Poor | Poor | Poor | Good | Negligible | Negligible | Negligible | Negligible | Poor | Good | Negligible | Fair | Negligible | Negligible | Fair | Negative likelihood ratio interpretation |
| NPV | 0.97459 | 0.99001 | 0.9961 | 0.99409 | 0.99509 | 0.9902 | 0.99501 | 0.99495 | 0.97887 | 0.98749 | 0.99897 | 0.99085 | 0.98518 | 0.99805 | 0.9465 | 0.97822 | 0.97866 | 0.96888 | 0.99902 | 0.99775 | 0.97889 | 0.96464 | 0.9922 | 0.99707 | 0.99502 | 0.999 | 0.99215 | 0.99902 | 0.99023 | 0.98161 | 0.99902 | Negative predictive value |
| OC | 0.13793 | 0.76 | None | 1.0 | 0.16667 | 0.16667 | 0.91667 | 0.88571 | 0.08696 | 0.5 | 0.96296 | 0.78571 | 0.42857 | 0.5 | 0.90871 | 0.0 | 0.7381 | 0.82258 | 0.75 | 0.98425 | 0.58065 | 0.5 | 0.0 | 1.0 | 0.66667 | 0.94118 | 1.0 | 1.0 | 0.0 | 0.0 | 0.85714 | Overlap coefficient |
| OOC | 0.11461 | 0.70564 | None | 0.8044 | 0.1543 | 0.12309 | 0.86424 | 0.87333 | 0.07372 | 0.29925 | 0.66276 | 0.78571 | 0.34993 | 0.40825 | 0.8732 | 0.0 | 0.66334 | 0.71967 | 0.5 | 0.94421 | 0.51768 | 0.22361 | 0.0 | 0.70711 | 0.56344 | 0.80915 | 0.68313 | 0.89443 | 0.0 | 0.0 | 0.85714 | Otsuka-Ochiai coefficient |
| OP | 0.18942 | 0.77896 | -0.0039 | 0.77987 | 0.27644 | 0.1528 | 0.89213 | 0.91861 | 0.11482 | 0.62669 | 0.9657 | 0.86699 | 0.4248 | 0.49744 | 0.86462 | -0.03704 | 0.72112 | 0.73833 | 0.85321 | 0.98473 | 0.60418 | 0.1435 | -0.00877 | 0.66374 | 0.78966 | 0.96538 | 0.62857 | 0.88791 | -0.0117 | -0.06335 | 0.92162 | Optimized precision |
| P | 29 | 29 | 4 | 17 | 6 | 11 | 27 | 36 | 23 | 24 | 27 | 42 | 21 | 3 | 261 | 22 | 52 | 81 | 4 | 127 | 39 | 40 | 8 | 6 | 15 | 17 | 15 | 5 | 10 | 18 | 7 | Condition positive or support |
| PLR | 3.61887 | 108.86782 | None | None | 28.33333 | 18.45455 | 407.0 | 213.125 | 2.90725 | 9.10909 | 31.03226 | 85.90476 | 35.89286 | 341.0 | 29.17712 | 0.0 | 52.78671 | 54.09091 | 127.75 | 68.06481 | 35.04142 | 24.65 | 0.0 | None | 61.27273 | 135.66387 | None | None | 0.0 | 0.0 | 873.42857 | Positive likelihood ratio |
| PLRI | Poor | Good | None | None | Good | Good | Good | Good | Poor | Fair | Good | Good | Good | Good | Good | Negligible | Good | Good | Good | Good | Good | Good | Negligible | None | Good | Good | None | None | Negligible | Negligible | Good | Positive likelihood ratio interpretation |
| POP | 1026 | 1026 | 1026 | 1026 | 1026 | 1026 | 1026 | 1026 | 1026 | 1026 | 1026 | 1026 | 1026 | 1026 | 1026 | 1026 | 1026 | 1026 | 1026 | 1026 | 1026 | 1026 | 1026 | 1026 | 1026 | 1026 | 1026 | 1026 | 1026 | 1026 | 1026 | Population |
| PPV | 0.09524 | 0.76 | None | 1.0 | 0.14286 | 0.16667 | 0.91667 | 0.88571 | 0.0625 | 0.1791 | 0.45614 | 0.78571 | 0.42857 | 0.5 | 0.90871 | 0.0 | 0.7381 | 0.82258 | 0.33333 | 0.9058 | 0.58065 | 0.5 | 0.0 | 1.0 | 0.47619 | 0.69565 | 1.0 | 1.0 | 0.0 | 0.0 | 0.85714 | Precision or positive predictive value |
| PRE | 0.02827 | 0.02827 | 0.0039 | 0.01657 | 0.00585 | 0.01072 | 0.02632 | 0.03509 | 0.02242 | 0.02339 | 0.02632 | 0.04094 | 0.02047 | 0.00292 | 0.25439 | 0.02144 | 0.05068 | 0.07895 | 0.0039 | 0.12378 | 0.03801 | 0.03899 | 0.0078 | 0.00585 | 0.01462 | 0.01657 | 0.01462 | 0.00487 | 0.00975 | 0.01754 | 0.00682 | Prevalence |
| Q | 0.60301 | 0.99365 | None | None | 0.94253 | 0.90566 | 0.99909 | 0.99869 | 0.51087 | 0.89022 | 0.99754 | 0.99498 | 0.96067 | 0.99609 | 0.98871 | -1.0 | 0.98464 | 0.98624 | 0.99607 | 0.99953 | 0.96933 | 0.92927 | -1.0 | None | 0.98906 | 0.99913 | None | None | -1.0 | -1.0 | 0.99967 | Yule Q - coefficient of colligation |
| QI | Moderate | Strong | None | None | Strong | Strong | Strong | Strong | Moderate | Strong | Strong | Strong | Strong | Strong | Strong | Negligible | Strong | Strong | Strong | Strong | Strong | Strong | Negligible | None | Strong | Strong | None | None | Negligible | Negligible | Strong | Yule Q interpretation |
| RACC | 0.00116 | 0.00069 | 0.0 | 0.00018 | 4e-05 | 6e-05 | 0.00062 | 0.0012 | 0.0007 | 0.00153 | 0.00146 | 0.00168 | 0.00028 | 1e-05 | 0.05975 | 0.00033 | 0.00207 | 0.00477 | 3e-05 | 0.01665 | 0.00115 | 0.0003 | 1e-05 | 2e-05 | 0.0003 | 0.00037 | 0.0001 | 2e-05 | 2e-05 | 0.0008 | 5e-05 | Random accuracy |
| RACCU | 0.0012 | 0.00069 | 0.0 | 0.00019 | 4e-05 | 7e-05 | 0.00062 | 0.0012 | 0.00072 | 0.00197 | 0.00168 | 0.00168 | 0.00029 | 1e-05 | 0.05985 | 0.00034 | 0.0021 | 0.00486 | 4e-05 | 0.01668 | 0.00116 | 0.00055 | 2e-05 | 2e-05 | 0.00031 | 0.00038 | 0.00011 | 2e-05 | 3e-05 | 0.001 | 5e-05 | Random accuracy unbiased |
| TN | 959 | 991 | 1022 | 1009 | 1014 | 1010 | 997 | 986 | 973 | 947 | 968 | 975 | 997 | 1022 | 743 | 988 | 963 | 934 | 1016 | 886 | 974 | 982 | 1017 | 1020 | 1000 | 1002 | 1011 | 1021 | 1014 | 961 | 1018 | True negative/correct rejection |
| TNR | 0.96189 | 0.99398 | 1.0 | 1.0 | 0.99412 | 0.99507 | 0.998 | 0.99596 | 0.97009 | 0.94511 | 0.96897 | 0.99085 | 0.99204 | 0.99902 | 0.97124 | 0.98406 | 0.98871 | 0.98836 | 0.99413 | 0.98554 | 0.98683 | 0.99594 | 0.99902 | 1.0 | 0.98912 | 0.99306 | 1.0 | 1.0 | 0.99803 | 0.95337 | 0.99902 | Specificity or true negative rate |
| TON | 984 | 1001 | 1026 | 1015 | 1019 | 1020 | 1002 | 991 | 994 | 959 | 969 | 984 | 1012 | 1024 | 785 | 1010 | 984 | 964 | 1017 | 888 | 995 | 1018 | 1025 | 1023 | 1005 | 1003 | 1019 | 1022 | 1024 | 979 | 1019 | Test outcome negative |
| TOP | 42 | 25 | 0 | 11 | 7 | 6 | 24 | 35 | 32 | 67 | 57 | 42 | 14 | 2 | 241 | 16 | 42 | 62 | 9 | 138 | 31 | 8 | 1 | 3 | 21 | 23 | 7 | 4 | 2 | 47 | 7 | Test outcome positive |
| TP | 4 | 19 | 0 | 11 | 1 | 1 | 22 | 31 | 2 | 12 | 26 | 33 | 6 | 1 | 219 | 0 | 31 | 51 | 3 | 125 | 18 | 4 | 0 | 3 | 10 | 16 | 7 | 4 | 0 | 0 | 6 | True positive/hit |
| TPR | 0.13793 | 0.65517 | 0.0 | 0.64706 | 0.16667 | 0.09091 | 0.81481 | 0.86111 | 0.08696 | 0.5 | 0.96296 | 0.78571 | 0.28571 | 0.33333 | 0.83908 | 0.0 | 0.59615 | 0.62963 | 0.75 | 0.98425 | 0.46154 | 0.1 | 0.0 | 0.5 | 0.66667 | 0.94118 | 0.46667 | 0.8 | 0.0 | 0.0 | 0.85714 | Sensitivity, recall, hit rate, or true positive rate |
| Y | 0.09982 | 0.64915 | 0.0 | 0.64706 | 0.16078 | 0.08598 | 0.81281 | 0.85707 | 0.05705 | 0.44511 | 0.93193 | 0.77657 | 0.27775 | 0.33236 | 0.81032 | -0.01594 | 0.58486 | 0.61799 | 0.74413 | 0.96979 | 0.44837 | 0.09594 | -0.00098 | 0.5 | 0.65579 | 0.93424 | 0.46667 | 0.8 | -0.00197 | -0.04663 | 0.85616 | Youden index |
| dInd | 0.86291 | 0.34488 | 1.0 | 0.35294 | 0.83335 | 0.9091 | 0.1852 | 0.13895 | 0.91353 | 0.503 | 0.04832 | 0.21448 | 0.71433 | 0.66667 | 0.16347 | 1.00013 | 0.404 | 0.37055 | 0.25007 | 0.02138 | 0.53862 | 0.90001 | 1.0 | 0.5 | 0.33351 | 0.05923 | 0.53333 | 0.2 | 1.0 | 1.00109 | 0.14286 | Distance index |
| sInd | 0.38983 | 0.75613 | 0.29289 | 0.75043 | 0.41073 | 0.35717 | 0.86905 | 0.90175 | 0.35403 | 0.64432 | 0.96583 | 0.84834 | 0.49489 | 0.52859 | 0.88441 | 0.2928 | 0.71433 | 0.73798 | 0.82317 | 0.98488 | 0.61914 | 0.3636 | 0.29289 | 0.64645 | 0.76417 | 0.95812 | 0.62288 | 0.85858 | 0.29289 | 0.29212 | 0.89898 | Similarity index |

Generated By PyCM Version 3.4
