# Supplementary material for: TopEC: prediction of Enzyme Commission classes by 3D graph neural networks and localized 3D protein descriptor
Source: Nat Commun. 2025 Mar 20;16:2737. doi: 10.1038/s41467-025-57324-5 (PMC11923149; doi:10.1038/s41467-025-57324-5)
Supplement: Supplementary file 3 — Supplementary Data 1 [file 41467_2025_57324_MOESM3_ESM.zip › Data_S1/table1/hierarchical/TopEC_distance_FOLD_1digs.html]

PyCM Report


# PyCM Report

## Dataset Type :

- Multi-Class Classification
- Imbalanced

Note 1 : Recommended statistics for this type of classification highlighted in aqua

Note 2 : The recommender system assumes that the input is the result of classification over the whole data rather than just a part of it.
If the confusion matrix is the result of test data classification, the recommendation is not valid.

## Confusion Matrix :

|  |  |  |  |  |  |  |  |  |  |  |  |  |  |  |  |  |  |  |  |  |  |  |  |  |  |  |  |  |  |  |  |  |  |  |  |  |  |  |  |  |  |  |  |  |  |  |  |  |  |  |  |  |  |  |  |  |  |  |  |  |  |  |  |  |  |
| --- | --- | --- | --- | --- | --- | --- | --- | --- | --- | --- | --- | --- | --- | --- | --- | --- | --- | --- | --- | --- | --- | --- | --- | --- | --- | --- | --- | --- | --- | --- | --- | --- | --- | --- | --- | --- | --- | --- | --- | --- | --- | --- | --- | --- | --- | --- | --- | --- | --- | --- | --- | --- | --- | --- | --- | --- | --- | --- | --- | --- | --- | --- | --- | --- | --- |
| Actual | Predict  |  |  |  |  |  |  |  |  | | --- | --- | --- | --- | --- | --- | --- | --- | |  | 1 | 2 | 3 | 4 | 5 | 6 | 7 | | 1 | 104 | 37 | 11 | 0 | 1 | 0 | 6 | | 2 | 16 | 348 | 16 | 0 | 0 | 0 | 21 | | 3 | 15 | 45 | 271 | 11 | 6 | 0 | 17 | | 4 | 5 | 2 | 5 | 13 | 0 | 2 | 2 | | 5 | 4 | 1 | 3 | 1 | 27 | 0 | 1 | | 6 | 2 | 8 | 0 | 0 | 0 | 0 | 0 | | 7 | 4 | 14 | 0 | 0 | 0 | 0 | 7 | |

## Overall Statistics :

|  |  |
| --- | --- |
| 95% CI | (0.72401,0.77697) |
| ACC Macro | 0.92871 |
| ARI | 0.4951 |
| AUNP | 0.82738 |
| AUNU | 0.74122 |
| Bangdiwala B | 0.64201 |
| Bennett S | 0.7089 |
| CBA | 0.49557 |
| CSI | 0.07285 |
| Chi-Squared | 1895.26957 |
| Chi-Squared DF | 36 |
| Conditional Entropy | 1.14435 |
| Cramer V | 0.55486 |
| Cross Entropy | 2.0293 |
| F1 Macro | 0.53046 |
| F1 Micro | 0.75049 |
| FNR Macro | 0.46823 |
| FNR Micro | 0.24951 |
| FPR Macro | 0.04932 |
| FPR Micro | 0.04159 |
| Gwet AC1 | 0.71791 |
| Hamming Loss | 0.24951 |
| Joint Entropy | 3.13536 |
| KL Divergence | 0.03829 |
| Kappa | 0.64085 |
| Kappa 95% CI | (0.60274,0.67896) |
| Kappa No Prevalence | 0.50097 |
| Kappa Standard Error | 0.01945 |
| Kappa Unbiased | 0.63994 |
| Krippendorff Alpha | 0.64011 |
| Lambda A | 0.616 |
| Lambda B | 0.57793 |
| Mutual Information | 0.83659 |
| NIR | 0.39084 |
| Overall ACC | 0.75049 |
| Overall CEN | 0.3092 |
| Overall J | (2.89779,0.41397) |
| Overall MCC | 0.64412 |
| Overall MCEN | 0.42789 |
| Overall RACC | 0.30527 |
| Overall RACCU | 0.30703 |
| P-Value | -0.0 |
| PPV Macro | 0.54108 |
| PPV Micro | 0.75049 |
| Pearson C | 0.80547 |
| Phi-Squared | 1.84724 |
| RCI | 0.42018 |
| RR | 146.57143 |
| Reference Entropy | 1.99101 |
| Response Entropy | 1.98094 |
| SOA1(Landis & Koch) | Substantial |
| SOA2(Fleiss) | Intermediate to Good |
| SOA3(Altman) | Good |
| SOA4(Cicchetti) | Good |
| SOA5(Cramer) | Relatively Strong |
| SOA6(Matthews) | Moderate |
| Scott PI | 0.63994 |
| Standard Error | 0.01351 |
| TNR Macro | 0.95068 |
| TNR Micro | 0.95841 |
| TPR Macro | 0.53177 |
| TPR Micro | 0.75049 |
| Zero-one Loss | 256 |

## Class Statistics :

|  |  |  |  |  |  |  |  |  |
| --- | --- | --- | --- | --- | --- | --- | --- | --- |
| Class | 1 | 2 | 3 | 4 | 5 | 6 | 7 | Description |
| ACC | 0.90156 | 0.84405 | 0.87427 | 0.97271 | 0.98343 | 0.9883 | 0.93665 | Accuracy |
| AGF | 0.78824 | 0.86741 | 0.82354 | 0.67379 | 0.85716 | 0.0 | 0.47089 | Adjusted F-score |
| AGM | 0.86026 | 0.84079 | 0.88106 | 0.82442 | 0.92077 | 0 | 0.73212 | Adjusted geometric mean |
| AM | -9 | 54 | -59 | -4 | -3 | -8 | 29 | Difference between automatic and manual classification |
| AUC | 0.80052 | 0.84832 | 0.84476 | 0.71812 | 0.86133 | 0.49902 | 0.61652 | Area under the ROC curve |
| AUCI | Very Good | Very Good | Very Good | Good | Very Good | Poor | Fair | AUC value interpretation |
| AUPR | 0.67371 | 0.81633 | 0.81404 | 0.48414 | 0.76192 | 0.0 | 0.20481 | Area under the PR curve |
| BCD | 0.00439 | 0.02632 | 0.02875 | 0.00195 | 0.00146 | 0.0039 | 0.01413 | Bray-Curtis dissimilarity |
| BM | 0.60103 | 0.69663 | 0.68952 | 0.43624 | 0.72265 | -0.00197 | 0.23305 | Informedness or bookmaker informedness |
| CEN | 0.39439 | 0.26695 | 0.27609 | 0.48486 | 0.29969 | 0.34913 | 0.59746 | Confusion entropy |
| DOR | 33.74862 | 31.78699 | 51.56413 | 66.69271 | 378.77143 | 0.0 | 7.89362 | Diagnostic odds ratio |
| DP | 0.84257 | 0.82823 | 0.94407 | 1.00567 | 1.42153 | None | 0.49469 | Discriminant power |
| DPI | Poor | Poor | Poor | Limited | Limited | None | Poor | Discriminant power interpretation |
| ERR | 0.09844 | 0.15595 | 0.12573 | 0.02729 | 0.01657 | 0.0117 | 0.06335 | Error rate |
| F0.5 | 0.68511 | 0.78343 | 0.85274 | 0.50388 | 0.78035 | 0.0 | 0.14523 | F0.5 score |
| F1 | 0.67314 | 0.81308 | 0.80775 | 0.48148 | 0.76056 | 0.0 | 0.17722 | F1 score - harmonic mean of precision and sensitivity |
| F2 | 0.66158 | 0.84507 | 0.76727 | 0.46099 | 0.74176 | 0.0 | 0.22727 | F2 score |
| FDR | 0.30667 | 0.23516 | 0.11438 | 0.48 | 0.20588 | 1.0 | 0.87037 | False discovery rate |
| FN | 55 | 53 | 94 | 16 | 10 | 10 | 18 | False negative/miss/type 2 error |
| FNR | 0.34591 | 0.13217 | 0.25753 | 0.55172 | 0.27027 | 1.0 | 0.72 | Miss rate or false negative rate |
| FOR | 0.06279 | 0.09282 | 0.13056 | 0.01598 | 0.01008 | 0.00977 | 0.01852 | False omission rate |
| FP | 46 | 107 | 35 | 12 | 7 | 2 | 47 | False positive/type 1 error/false alarm |
| FPR | 0.05306 | 0.1712 | 0.05295 | 0.01204 | 0.00708 | 0.00197 | 0.04695 | Fall-out or false positive rate |
| G | 0.67342 | 0.81471 | 0.81089 | 0.48281 | 0.76124 | 0.0 | 0.19052 | G-measure geometric mean of precision and sensitivity |
| GI | 0.60103 | 0.69663 | 0.68952 | 0.43624 | 0.72265 | -0.00197 | 0.23305 | Gini index |
| GM | 0.78701 | 0.84809 | 0.83854 | 0.66549 | 0.85121 | 0.0 | 0.51658 | G-mean geometric mean of specificity and sensitivity |
| IBA | 0.43799 | 0.74733 | 0.5593 | 0.20386 | 0.53386 | 0.0 | 0.08725 | Index of balanced accuracy |
| ICSI | 0.34742 | 0.63267 | 0.62809 | -0.03172 | 0.52385 | -1.0 | -0.59037 | Individual classification success index |
| IS | 2.16155 | 0.96858 | 1.31582 | 4.20142 | 4.46079 | None | 2.41143 | Information score |
| J | 0.50732 | 0.68504 | 0.6775 | 0.31707 | 0.61364 | 0.0 | 0.09722 | Jaccard index |
| LS | 4.47396 | 1.95691 | 2.48944 | 18.39724 | 22.02067 | 0.0 | 5.32 | Lift score |
| MCC | 0.61561 | 0.68421 | 0.72155 | 0.4689 | 0.75272 | -0.00438 | 0.16092 | Matthews correlation coefficient |
| MCCI | Moderate | Moderate | Strong | Weak | Strong | Negligible | Negligible | Matthews correlation coefficient interpretation |
| MCEN | 0.51312 | 0.38369 | 0.396 | 0.56291 | 0.40919 | 0.34913 | 0.62184 | Modified confusion entropy |
| MK | 0.63055 | 0.67202 | 0.75507 | 0.50402 | 0.78404 | -0.00977 | 0.11111 | Markedness |
| N | 867 | 625 | 661 | 997 | 989 | 1016 | 1001 | Condition negative |
| NLR | 0.36529 | 0.15947 | 0.27193 | 0.55845 | 0.2722 | 1.00197 | 0.75547 | Negative likelihood ratio |
| NLRI | Poor | Fair | Poor | Negligible | Poor | Negligible | Negligible | Negative likelihood ratio interpretation |
| NPV | 0.93721 | 0.90718 | 0.86944 | 0.98402 | 0.98992 | 0.99023 | 0.98148 | Negative predictive value |
| OC | 0.69333 | 0.86783 | 0.88562 | 0.52 | 0.79412 | 0.0 | 0.28 | Overlap coefficient |
| OOC | 0.67342 | 0.81471 | 0.81089 | 0.48281 | 0.76124 | 0.0 | 0.19052 | Otsuka-Ochiai coefficient |
| OP | 0.71864 | 0.82105 | 0.75318 | 0.59694 | 0.83065 | -0.0117 | 0.39081 | Optimized precision |
| P | 159 | 401 | 365 | 29 | 37 | 10 | 25 | Condition positive or support |
| PLR | 12.32814 | 5.0691 | 14.022 | 37.24425 | 103.10039 | 0.0 | 5.9634 | Positive likelihood ratio |
| PLRI | Good | Fair | Good | Good | Good | Negligible | Fair | Positive likelihood ratio interpretation |
| POP | 1026 | 1026 | 1026 | 1026 | 1026 | 1026 | 1026 | Population |
| PPV | 0.69333 | 0.76484 | 0.88562 | 0.52 | 0.79412 | 0.0 | 0.12963 | Precision or positive predictive value |
| PRE | 0.15497 | 0.39084 | 0.35575 | 0.02827 | 0.03606 | 0.00975 | 0.02437 | Prevalence |
| Q | 0.94244 | 0.939 | 0.96195 | 0.97045 | 0.99473 | -1.0 | 0.77512 | Yule Q - coefficient of colligation |
| QI | Strong | Strong | Strong | Strong | Strong | Negligible | Strong | Yule Q interpretation |
| RACC | 0.02266 | 0.17332 | 0.1061 | 0.00069 | 0.0012 | 2e-05 | 0.00128 | Random accuracy |
| RACCU | 0.02268 | 0.17402 | 0.10693 | 0.00069 | 0.0012 | 3e-05 | 0.00148 | Random accuracy unbiased |
| TN | 821 | 518 | 626 | 985 | 982 | 1014 | 954 | True negative/correct rejection |
| TNR | 0.94694 | 0.8288 | 0.94705 | 0.98796 | 0.99292 | 0.99803 | 0.95305 | Specificity or true negative rate |
| TON | 876 | 571 | 720 | 1001 | 992 | 1024 | 972 | Test outcome negative |
| TOP | 150 | 455 | 306 | 25 | 34 | 2 | 54 | Test outcome positive |
| TP | 104 | 348 | 271 | 13 | 27 | 0 | 7 | True positive/hit |
| TPR | 0.65409 | 0.86783 | 0.74247 | 0.44828 | 0.72973 | 0.0 | 0.28 | Sensitivity, recall, hit rate, or true positive rate |
| Y | 0.60103 | 0.69663 | 0.68952 | 0.43624 | 0.72265 | -0.00197 | 0.23305 | Youden index |
| dInd | 0.34996 | 0.21628 | 0.26292 | 0.55186 | 0.27036 | 1.0 | 0.72153 | Distance index |
| sInd | 0.75254 | 0.84706 | 0.81409 | 0.60978 | 0.80882 | 0.29289 | 0.4898 | Similarity index |

Generated By PyCM Version 3.4
