# Supplementary material for: TopEC: prediction of Enzyme Commission classes by 3D graph neural networks and localized 3D protein descriptor
Source: Nat Commun. 2025 Mar 20;16:2737. doi: 10.1038/s41467-025-57324-5 (PMC11923149; doi:10.1038/s41467-025-57324-5)
Supplement: Supplementary file 3 — Supplementary Data 1 [file 41467_2025_57324_MOESM3_ESM.zip › Data_S1/table1/hierarchical/DeepFRI_TEMP_local.html]

DeepFRI\_HIER\_sites


# PyCM Report

## Dataset Type :

- Multi-Class Classification
- Imbalanced

Note 1 : Recommended statistics for this type of classification highlighted in aqua

Note 2 : The recommender system assumes that the input is the result of classification over the whole data rather than just a part of it.
If the confusion matrix is the result of test data classification, the recommendation is not valid.

## Confusion Matrix :

|  |  |  |  |  |  |  |  |  |  |  |  |  |  |  |  |  |  |  |  |  |  |  |  |  |  |  |  |  |  |  |  |  |  |  |  |  |  |  |  |  |  |  |  |  |  |  |  |  |  |  |  |  |  |  |  |  |  |  |  |  |  |  |  |  |  |  |  |  |  |  |  |  |  |  |  |  |  |  |  |  |  |  |  |  |  |  |  |  |  |  |  |  |  |  |  |  |  |  |  |  |  |  |  |  |  |  |  |  |  |  |  |  |  |  |  |  |  |  |  |  |  |  |  |  |  |  |  |  |  |  |  |  |  |  |  |  |  |  |  |  |  |  |  |  |  |  |  |  |  |  |  |  |  |  |  |  |  |  |  |  |  |  |  |  |  |  |  |  |  |  |  |  |  |  |  |  |  |  |  |  |  |  |  |  |  |  |  |  |  |  |  |  |  |  |  |  |  |  |  |  |  |  |  |  |  |  |  |  |  |  |  |  |  |  |  |  |  |  |  |  |  |  |  |  |  |  |  |  |  |  |  |  |  |  |  |  |  |  |  |  |  |  |  |  |  |  |  |  |  |  |  |  |  |  |  |  |  |  |  |  |  |  |  |  |  |  |  |  |  |  |  |  |  |  |  |  |  |  |  |  |  |  |  |  |  |  |  |  |  |  |  |  |  |  |  |  |  |  |  |  |  |  |  |  |  |  |  |  |  |  |  |  |  |  |  |  |  |  |  |  |  |  |  |  |  |  |  |  |  |  |  |  |  |  |  |  |  |  |  |  |  |  |  |  |  |  |  |  |  |  |  |  |  |  |  |  |  |  |  |  |  |  |  |  |  |  |  |  |  |  |  |  |  |  |  |  |  |  |  |  |  |  |  |  |  |  |  |  |  |  |  |  |  |  |  |  |  |  |  |  |  |  |  |  |  |  |  |  |  |  |  |  |  |  |  |  |  |  |  |  |  |  |  |  |  |  |  |  |  |  |  |  |  |  |  |  |  |  |  |  |  |  |  |  |  |  |  |  |  |  |  |  |  |  |  |  |  |  |  |  |  |  |  |  |  |  |  |  |  |  |  |  |  |  |  |  |  |  |  |  |  |  |  |  |  |  |  |  |  |  |  |  |  |  |  |  |  |  |  |  |  |  |  |  |  |  |  |  |  |  |  |  |  |  |  |  |  |  |  |  |  |  |  |  |  |  |  |  |  |  |  |  |  |  |  |  |  |  |  |  |  |  |  |  |  |  |  |  |  |  |  |  |  |  |  |  |  |  |  |  |  |  |  |  |  |  |  |  |  |  |  |  |  |  |  |  |  |  |  |  |  |  |  |  |  |  |  |  |  |  |  |  |  |  |  |  |  |  |  |  |  |  |  |  |  |  |  |  |  |  |  |  |  |  |  |  |  |  |  |  |  |  |  |  |  |  |  |  |  |  |  |  |  |  |  |  |  |  |  |  |  |  |  |  |  |  |  |  |  |  |  |  |  |  |  |  |  |  |  |  |  |  |  |  |  |  |  |  |  |  |  |  |  |  |  |  |  |  |  |  |  |  |  |  |  |  |  |  |  |  |  |  |  |  |  |  |  |  |  |  |  |  |  |  |  |  |  |  |  |  |  |  |  |  |  |  |  |  |  |  |  |  |  |  |  |  |  |  |  |  |  |  |  |  |  |  |  |  |  |  |  |  |  |  |  |  |  |  |  |  |  |  |  |  |  |  |  |  |  |  |  |  |  |  |  |  |  |  |  |  |  |  |  |  |  |  |  |  |  |  |  |  |  |  |  |  |  |  |  |  |  |  |  |  |  |  |  |  |  |  |  |  |  |  |  |  |  |  |  |  |  |  |  |  |  |  |  |  |  |  |  |  |  |  |  |  |  |  |  |  |  |  |  |  |  |  |  |  |  |  |  |  |  |  |  |  |  |  |  |  |  |  |  |  |  |  |  |  |  |  |  |  |  |  |  |  |  |  |  |  |  |  |  |  |  |  |  |  |  |  |  |  |  |  |  |  |  |  |  |  |  |  |  |  |  |  |  |  |  |  |  |  |  |  |  |  |  |  |  |  |  |  |  |  |  |  |  |  |  |  |  |  |  |  |  |  |  |  |  |  |  |  |  |  |  |  |  |  |  |  |  |  |  |  |  |  |  |  |  |  |  |  |  |  |  |  |  |  |  |  |  |  |  |  |  |  |  |  |  |  |  |  |  |  |  |  |  |  |  |  |  |  |  |  |  |  |  |  |  |  |  |  |  |  |  |  |  |  |  |  |  |  |  |  |  |  |  |  |  |  |  |  |  |  |  |  |  |  |  |  |  |  |  |  |  |  |  |  |  |  |  |  |  |  |  |  |  |  |  |  |  |  |  |  |  |  |  |  |  |  |  |  |  |  |  |  |  |  |  |  |  |  |  |  |  |  |  |  |  |  |  |  |  |  |  |  |  |  |  |  |  |  |  |  |  |  |  |  |  |  |  |  |  |  |  |  |  |  |  |  |  |  |  |  |  |  |  |  |  |  |  |  |  |  |  |  |  |  |  |  |  |  |  |  |  |  |  |  |  |  |  |  |  |  |  |  |  |  |  |  |  |  |  |  |  |  |  |  |  |  |  |  |  |  |  |  |  |  |  |  |  |  |  |  |  |  |  |  |  |  |  |  |  |  |  |  |  |  |  |  |  |  |  |  |  |  |  |  |  |  |  |  |  |  |  |  |  |  |  |  |  |  |  |  |  |  |  |  |  |  |  |  |  |  |  |  |  |  |  |  |  |  |  |  |  |  |  |  |  |  |  |  |  |  |  |  |  |  |  |  |  |  |  |  |  |  |  |  |  |  |  |  |  |  |  |  |  |  |  |  |  |  |  |  |  |  |  |  |  |  |  |  |  |  |  |  |  |  |  |  |  |  |  |  |  |  |  |  |  |  |  |  |  |  |  |  |  |  |  |  |  |  |  |  |  |  |  |  |  |  |  |  |  |  |  |  |  |  |  |  |  |  |  |  |  |  |  |  |  |  |  |  |  |  |  |  |  |  |  |  |  |  |  |  |  |  |  |  |  |  |  |  |  |  |  |  |  |  |  |  |  |  |  |  |  |  |  |  |  |  |  |  |  |  |  |  |  |  |  |  |  |  |  |  |  |  |  |  |  |  |  |  |  |  |  |  |  |  |  |  |  |  |  |  |  |  |  |  |  |  |  |  |  |  |  |  |  |  |  |  |  |  |  |  |  |  |  |  |  |  |  |  |  |  |  |  |  |  |  |  |  |  |  |  |  |  |  |  |  |  |  |  |  |  |  |  |  |  |  |  |  |  |  |  |  |  |  |  |  |  |  |  |  |  |  |  |  |  |  |  |  |  |  |  |  |  |  |  |  |  |  |  |  |  |  |  |  |  |  |  |  |  |  |  |  |  |  |  |  |  |  |  |  |  |  |  |  |  |  |  |  |  |  |  |  |  |  |  |  |  |  |  |  |  |  |  |  |  |  |  |  |  |  |  |  |  |  |  |  |  |  |  |  |  |  |  |  |  |  |  |  |  |  |  |  |  |  |  |  |  |  |  |  |  |  |  |  |  |  |  |  |  |  |  |  |  |  |  |  |  |  |  |  |  |  |  |  |  |  |  |  |  |  |  |  |  |  |  |  |  |  |  |  |  |  |  |  |  |  |  |  |  |  |  |  |  |  |  |  |  |  |  |  |  |  |  |  |  |  |  |  |  |  |  |  |  |  |  |  |  |  |  |  |  |  |  |  |  |  |  |  |  |  |  |  |  |  |  |  |  |  |  |  |  |  |  |  |  |  |  |  |  |  |  |  |  |  |  |  |  |  |  |  |  |  |  |  |  |  |  |  |  |  |  |  |  |  |  |  |  |  |  |  |  |  |  |  |  |  |  |  |  |  |  |  |  |  |  |  |  |  |  |  |  |  |  |  |  |  |  |  |  |  |  |  |  |  |  |  |  |  |  |  |  |  |  |  |  |  |  |  |  |  |  |  |  |  |  |  |  |  |  |  |  |  |  |  |  |  |  |  |  |  |  |  |  |  |  |  |  |  |  |  |  |  |  |  |  |  |  |  |  |  |  |  |  |  |  |  |  |  |  |  |  |  |  |  |  |  |  |  |  |  |  |  |  |  |  |  |  |  |  |  |  |  |  |  |  |  |  |  |  |  |  |  |  |  |  |  |  |  |  |  |  |  |  |  |  |  |  |  |  |  |  |  |  |  |  |  |  |  |  |  |  |  |  |  |  |  |  |  |  |  |  |  |  |  |  |  |  |  |  |  |  |  |  |  |  |  |  |  |  |  |  |  |  |  |  |  |  |  |  |  |  |  |  |  |  |  |  |  |  |  |  |  |  |  |  |  |  |  |  |  |  |  |  |  |  |  |  |  |  |  |  |  |  |  |  |  |  |  |  |  |  |  |  |  |  |  |  |  |  |  |  |  |  |  |  |  |  |  |  |  |  |  |  |  |  |  |  |  |  |  |  |  |  |  |  |  |  |  |  |  |  |  |  |  |  |  |  |  |  |  |  |  |  |  |  |  |  |  |  |  |  |  |  |  |  |  |  |  |  |  |  |  |  |  |  |  |  |  |  |  |  |  |  |  |  |  |  |  |  |  |  |  |  |  |  |  |  |  |  |  |  |  |  |  |  |  |  |  |  |  |  |  |  |  |  |  |  |  |  |  |  |  |  |  |  |  |  |  |  |  |  |  |  |  |  |  |  |  |  |  |  |  |  |  |  |  |  |  |  |  |  |  |  |  |  |  |  |  |  |  |  |  |  |  |  |  |  |  |  |  |  |  |  |  |  |  |  |  |  |  |  |  |  |  |  |  |  |  |  |  |  |  |  |  |  |  |  |  |  |  |  |  |  |  |  |  |  |  |  |  |  |  |  |  |  |  |  |  |  |  |  |  |  |  |  |  |  |  |  |  |  |  |  |  |  |  |  |  |  |  |  |  |  |  |  |  |  |  |  |  |  |  |  |  |  |  |  |  |  |  |  |  |  |  |  |  |  |  |  |  |  |  |  |  |  |  |  |  |  |  |  |  |  |  |  |  |  |  |  |  |  |  |  |  |  |  |  |  |  |  |  |  |  |  |  |  |  |  |  |  |  |  |  |  |  |  |  |  |  |  |  |  |  |  |  |  |  |  |  |  |  |  |  |  |  |  |  |  |  |  |  |  |  |  |  |  |  |  |  |  |  |  |  |  |  |  |  |  |  |  |  |  |  |  |  |  |  |  |  |  |  |  |  |  |  |  |  |  |  |  |  |  |  |  |  |  |  |  |  |  |  |  |  |  |  |  |  |  |  |  |  |  |  |  |  |  |  |  |  |  |  |  |  |  |  |  |  |  |  |  |  |  |  |  |  |  |  |  |  |  |  |  |  |  |  |  |  |  |  |  |  |  |  |  |  |  |  |  |  |  |  |  |  |  |  |  |  |  |  |  |  |  |  |  |  |  |  |  |  |  |  |  |  |  |  |  |  |  |  |  |  |  |  |  |  |  |  |  |  |  |  |  |  |  |  |  |  |  |  |  |  |  |  |  |  |  |  |  |  |  |  |  |  |  |  |  |  |  |  |  |  |  |  |  |  |  |  |  |  |  |  |  |  |  |  |  |  |  |  |  |  |  |  |  |  |  |  |  |  |  |  |  |  |  |  |  |  |  |  |  |  |  |  |  |  |  |  |  |  |  |  |  |  |  |  |  |  |  |  |  |  |  |  |  |  |  |  |  |  |  |  |  |  |  |  |  |  |  |  |  |  |  |  |  |  |  |  |  |  |  |  |  |  |  |  |  |  |  |  |  |  |  |  |  |  |  |  |  |  |  |  |  |  |  |  |  |  |  |  |  |  |  |  |  |  |  |  |  |  |  |  |  |  |  |  |  |  |  |  |  |  |  |  |  |  |  |  |  |  |  |  |  |  |  |  |  |  |  |  |  |  |  |  |  |  |  |  |  |  |  |  |  |  |  |  |  |  |  |  |  |  |  |  |  |  |  |  |  |  |  |  |  |  |  |  |  |  |  |  |  |  |  |  |  |  |  |  |  |  |  |  |  |  |  |  |  |  |  |  |  |  |  |  |  |  |  |  |  |  |  |  |  |  |  |  |  |  |  |  |  |  |  |  |  |  |  |  |  |  |  |  |  |  |  |  |  |  |  |  |  |  |  |  |  |  |  |  |  |  |  |  |  |  |  |  |  |  |  |  |  |  |  |  |  |  |  |  |  |  |  |  |  |  |  |  |  |  |  |  |  |  |  |  |  |  |  |  |  |  |  |  |  |  |  |  |  |  |  |  |  |  |  |  |  |  |  |  |  |  |  |  |  |  |  |  |  |  |
| --- | --- | --- | --- | --- | --- | --- | --- | --- | --- | --- | --- | --- | --- | --- | --- | --- | --- | --- | --- | --- | --- | --- | --- | --- | --- | --- | --- | --- | --- | --- | --- | --- | --- | --- | --- | --- | --- | --- | --- | --- | --- | --- | --- | --- | --- | --- | --- | --- | --- | --- | --- | --- | --- | --- | --- | --- | --- | --- | --- | --- | --- | --- | --- | --- | --- | --- | --- | --- | --- | --- | --- | --- | --- | --- | --- | --- | --- | --- | --- | --- | --- | --- | --- | --- | --- | --- | --- | --- | --- | --- | --- | --- | --- | --- | --- | --- | --- | --- | --- | --- | --- | --- | --- | --- | --- | --- | --- | --- | --- | --- | --- | --- | --- | --- | --- | --- | --- | --- | --- | --- | --- | --- | --- | --- | --- | --- | --- | --- | --- | --- | --- | --- | --- | --- | --- | --- | --- | --- | --- | --- | --- | --- | --- | --- | --- | --- | --- | --- | --- | --- | --- | --- | --- | --- | --- | --- | --- | --- | --- | --- | --- | --- | --- | --- | --- | --- | --- | --- | --- | --- | --- | --- | --- | --- | --- | --- | --- | --- | --- | --- | --- | --- | --- | --- | --- | --- | --- | --- | --- | --- | --- | --- | --- | --- | --- | --- | --- | --- | --- | --- | --- | --- | --- | --- | --- | --- | --- | --- | --- | --- | --- | --- | --- | --- | --- | --- | --- | --- | --- | --- | --- | --- | --- | --- | --- | --- | --- | --- | --- | --- | --- | --- | --- | --- | --- | --- | --- | --- | --- | --- | --- | --- | --- | --- | --- | --- | --- | --- | --- | --- | --- | --- | --- | --- | --- | --- | --- | --- | --- | --- | --- | --- | --- | --- | --- | --- | --- | --- | --- | --- | --- | --- | --- | --- | --- | --- | --- | --- | --- | --- | --- | --- | --- | --- | --- | --- | --- | --- | --- | --- | --- | --- | --- | --- | --- | --- | --- | --- | --- | --- | --- | --- | --- | --- | --- | --- | --- | --- | --- | --- | --- | --- | --- | --- | --- | --- | --- | --- | --- | --- | --- | --- | --- | --- | --- | --- | --- | --- | --- | --- | --- | --- | --- | --- | --- | --- | --- | --- | --- | --- | --- | --- | --- | --- | --- | --- | --- | --- | --- | --- | --- | --- | --- | --- | --- | --- | --- | --- | --- | --- | --- | --- | --- | --- | --- | --- | --- | --- | --- | --- | --- | --- | --- | --- | --- | --- | --- | --- | --- | --- | --- | --- | --- | --- | --- | --- | --- | --- | --- | --- | --- | --- | --- | --- | --- | --- | --- | --- | --- | --- | --- | --- | --- | --- | --- | --- | --- | --- | --- | --- | --- | --- | --- | --- | --- | --- | --- | --- | --- | --- | --- | --- | --- | --- | --- | --- | --- | --- | --- | --- | --- | --- | --- | --- | --- | --- | --- | --- | --- | --- | --- | --- | --- | --- | --- | --- | --- | --- | --- | --- | --- | --- | --- | --- | --- | --- | --- | --- | --- | --- | --- | --- | --- | --- | --- | --- | --- | --- | --- | --- | --- | --- | --- | --- | --- | --- | --- | --- | --- | --- | --- | --- | --- | --- | --- | --- | --- | --- | --- | --- | --- | --- | --- | --- | --- | --- | --- | --- | --- | --- | --- | --- | --- | --- | --- | --- | --- | --- | --- | --- | --- | --- | --- | --- | --- | --- | --- | --- | --- | --- | --- | --- | --- | --- | --- | --- | --- | --- | --- | --- | --- | --- | --- | --- | --- | --- | --- | --- | --- | --- | --- | --- | --- | --- | --- | --- | --- | --- | --- | --- | --- | --- | --- | --- | --- | --- | --- | --- | --- | --- | --- | --- | --- | --- | --- | --- | --- | --- | --- | --- | --- | --- | --- | --- | --- | --- | --- | --- | --- | --- | --- | --- | --- | --- | --- | --- | --- | --- | --- | --- | --- | --- | --- | --- | --- | --- | --- | --- | --- | --- | --- | --- | --- | --- | --- | --- | --- | --- | --- | --- | --- | --- | --- | --- | --- | --- | --- | --- | --- | --- | --- | --- | --- | --- | --- | --- | --- | --- | --- | --- | --- | --- | --- | --- | --- | --- | --- | --- | --- | --- | --- | --- | --- | --- | --- | --- | --- | --- | --- | --- | --- | --- | --- | --- | --- | --- | --- | --- | --- | --- | --- | --- | --- | --- | --- | --- | --- | --- | --- | --- | --- | --- | --- | --- | --- | --- | --- | --- | --- | --- | --- | --- | --- | --- | --- | --- | --- | --- | --- | --- | --- | --- | --- | --- | --- | --- | --- | --- | --- | --- | --- | --- | --- | --- | --- | --- | --- | --- | --- | --- | --- | --- | --- | --- | --- | --- | --- | --- | --- | --- | --- | --- | --- | --- | --- | --- | --- | --- | --- | --- | --- | --- | --- | --- | --- | --- | --- | --- | --- | --- | --- | --- | --- | --- | --- | --- | --- | --- | --- | --- | --- | --- | --- | --- | --- | --- | --- | --- | --- | --- | --- | --- | --- | --- | --- | --- | --- | --- | --- | --- | --- | --- | --- | --- | --- | --- | --- | --- | --- | --- | --- | --- | --- | --- | --- | --- | --- | --- | --- | --- | --- | --- | --- | --- | --- | --- | --- | --- | --- | --- | --- | --- | --- | --- | --- | --- | --- | --- | --- | --- | --- | --- | --- | --- | --- | --- | --- | --- | --- | --- | --- | --- | --- | --- | --- | --- | --- | --- | --- | --- | --- | --- | --- | --- | --- | --- | --- | --- | --- | --- | --- | --- | --- | --- | --- | --- | --- | --- | --- | --- | --- | --- | --- | --- | --- | --- | --- | --- | --- | --- | --- | --- | --- | --- | --- | --- | --- | --- | --- | --- | --- | --- | --- | --- | --- | --- | --- | --- | --- | --- | --- | --- | --- | --- | --- | --- | --- | --- | --- | --- | --- | --- | --- | --- | --- | --- | --- | --- | --- | --- | --- | --- | --- | --- | --- | --- | --- | --- | --- | --- | --- | --- | --- | --- | --- | --- | --- | --- | --- | --- | --- | --- | --- | --- | --- | --- | --- | --- | --- | --- | --- | --- | --- | --- | --- | --- | --- | --- | --- | --- | --- | --- | --- | --- | --- | --- | --- | --- | --- | --- | --- | --- | --- | --- | --- | --- | --- | --- | --- | --- | --- | --- | --- | --- | --- | --- | --- | --- | --- | --- | --- | --- | --- | --- | --- | --- | --- | --- | --- | --- | --- | --- | --- | --- | --- | --- | --- | --- | --- | --- | --- | --- | --- | --- | --- | --- | --- | --- | --- | --- | --- | --- | --- | --- | --- | --- | --- | --- | --- | --- | --- | --- | --- | --- | --- | --- | --- | --- | --- | --- | --- | --- | --- | --- | --- | --- | --- | --- | --- | --- | --- | --- | --- | --- | --- | --- | --- | --- | --- | --- | --- | --- | --- | --- | --- | --- | --- | --- | --- | --- | --- | --- | --- | --- | --- | --- | --- | --- | --- | --- | --- | --- | --- | --- | --- | --- | --- | --- | --- | --- | --- | --- | --- | --- | --- | --- | --- | --- | --- | --- | --- | --- | --- | --- | --- | --- | --- | --- | --- | --- | --- | --- | --- | --- | --- | --- | --- | --- | --- | --- | --- | --- | --- | --- | --- | --- | --- | --- | --- | --- | --- | --- | --- | --- | --- | --- | --- | --- | --- | --- | --- | --- | --- | --- | --- | --- | --- | --- | --- | --- | --- | --- | --- | --- | --- | --- | --- | --- | --- | --- | --- | --- | --- | --- | --- | --- | --- | --- | --- | --- | --- | --- | --- | --- | --- | --- | --- | --- | --- | --- | --- | --- | --- | --- | --- | --- | --- | --- | --- | --- | --- | --- | --- | --- | --- | --- | --- | --- | --- | --- | --- | --- | --- | --- | --- | --- | --- | --- | --- | --- | --- | --- | --- | --- | --- | --- | --- | --- | --- | --- | --- | --- | --- | --- | --- | --- | --- | --- | --- | --- | --- | --- | --- | --- | --- | --- | --- | --- | --- | --- | --- | --- | --- | --- | --- | --- | --- | --- | --- | --- | --- | --- | --- | --- | --- | --- | --- | --- | --- | --- | --- | --- | --- | --- | --- | --- | --- | --- | --- | --- | --- | --- | --- | --- | --- | --- | --- | --- | --- | --- | --- | --- | --- | --- | --- | --- | --- | --- | --- | --- | --- | --- | --- | --- | --- | --- | --- | --- | --- | --- | --- | --- | --- | --- | --- | --- | --- | --- | --- | --- | --- | --- | --- | --- | --- | --- | --- | --- | --- | --- | --- | --- | --- | --- | --- | --- | --- | --- | --- | --- | --- | --- | --- | --- | --- | --- | --- | --- | --- | --- | --- | --- | --- | --- | --- | --- | --- | --- | --- | --- | --- | --- | --- | --- | --- | --- | --- | --- | --- | --- | --- | --- | --- | --- | --- | --- | --- | --- | --- | --- | --- | --- | --- | --- | --- | --- | --- | --- | --- | --- | --- | --- | --- | --- | --- | --- | --- | --- | --- | --- | --- | --- | --- | --- | --- | --- | --- | --- | --- | --- | --- | --- | --- | --- | --- | --- | --- | --- | --- | --- | --- | --- | --- | --- | --- | --- | --- | --- | --- | --- | --- | --- | --- | --- | --- | --- | --- | --- | --- | --- | --- | --- | --- | --- | --- | --- | --- | --- | --- | --- | --- | --- | --- | --- | --- | --- | --- | --- | --- | --- | --- | --- | --- | --- | --- | --- | --- | --- | --- | --- | --- | --- | --- | --- | --- | --- | --- | --- | --- | --- | --- | --- | --- | --- | --- | --- | --- | --- | --- | --- | --- | --- | --- | --- | --- | --- | --- | --- | --- | --- | --- | --- | --- | --- | --- | --- | --- | --- | --- | --- | --- | --- | --- | --- | --- | --- | --- | --- | --- | --- | --- | --- | --- | --- | --- | --- | --- | --- | --- | --- | --- | --- | --- | --- | --- | --- | --- | --- | --- | --- | --- | --- | --- | --- | --- | --- | --- | --- | --- | --- | --- | --- | --- | --- | --- | --- | --- | --- | --- | --- | --- | --- | --- | --- | --- | --- | --- | --- | --- | --- | --- | --- | --- | --- | --- | --- | --- | --- | --- | --- | --- | --- | --- | --- | --- | --- | --- | --- | --- | --- | --- | --- | --- | --- | --- | --- | --- | --- | --- | --- | --- | --- | --- | --- | --- | --- | --- | --- | --- | --- | --- | --- | --- | --- | --- | --- | --- | --- | --- | --- | --- | --- | --- | --- | --- | --- | --- | --- | --- | --- | --- | --- | --- | --- | --- | --- | --- | --- | --- | --- | --- | --- | --- | --- | --- | --- | --- | --- | --- | --- | --- | --- | --- | --- | --- | --- | --- | --- | --- | --- | --- | --- | --- | --- | --- | --- | --- | --- | --- | --- | --- | --- | --- | --- | --- | --- | --- | --- | --- | --- | --- | --- | --- | --- | --- | --- | --- | --- | --- | --- | --- | --- | --- | --- | --- | --- | --- | --- | --- | --- | --- | --- | --- | --- | --- | --- | --- | --- | --- | --- | --- | --- | --- | --- | --- | --- | --- | --- | --- | --- | --- | --- | --- | --- | --- | --- | --- | --- | --- | --- | --- | --- | --- | --- | --- | --- | --- | --- | --- | --- | --- | --- | --- | --- | --- | --- | --- | --- | --- | --- | --- | --- | --- | --- | --- | --- | --- | --- | --- | --- | --- | --- | --- | --- | --- | --- | --- | --- | --- | --- | --- | --- | --- | --- | --- | --- | --- | --- | --- | --- | --- | --- | --- | --- | --- | --- | --- | --- | --- | --- | --- | --- | --- | --- | --- | --- | --- | --- | --- | --- | --- | --- | --- | --- | --- | --- | --- | --- | --- | --- | --- | --- | --- | --- | --- | --- | --- | --- | --- | --- | --- | --- | --- | --- | --- | --- | --- | --- | --- | --- | --- | --- | --- | --- | --- | --- | --- | --- | --- | --- | --- | --- | --- | --- | --- | --- | --- | --- | --- | --- | --- | --- | --- | --- | --- | --- | --- | --- | --- | --- | --- | --- | --- | --- | --- | --- | --- | --- | --- | --- | --- | --- | --- | --- | --- | --- | --- | --- | --- | --- | --- | --- | --- | --- | --- | --- | --- | --- | --- | --- | --- | --- | --- | --- | --- | --- | --- | --- | --- | --- | --- | --- | --- | --- | --- | --- | --- | --- | --- | --- | --- | --- | --- | --- | --- | --- | --- | --- | --- | --- | --- | --- | --- | --- | --- | --- | --- | --- | --- | --- | --- | --- | --- | --- | --- | --- | --- | --- | --- | --- | --- | --- | --- | --- | --- | --- | --- | --- | --- | --- | --- | --- | --- | --- | --- | --- | --- | --- | --- | --- | --- | --- | --- | --- | --- | --- | --- | --- | --- | --- | --- | --- | --- | --- | --- | --- | --- | --- | --- | --- | --- | --- | --- | --- | --- | --- | --- | --- | --- | --- | --- | --- | --- | --- | --- | --- | --- | --- | --- | --- | --- | --- | --- | --- | --- | --- | --- | --- | --- | --- | --- | --- | --- | --- | --- | --- | --- | --- | --- | --- | --- | --- | --- | --- | --- | --- | --- | --- | --- | --- | --- | --- | --- | --- | --- | --- | --- | --- | --- | --- | --- | --- | --- | --- | --- | --- | --- | --- | --- | --- | --- | --- | --- | --- | --- | --- | --- | --- | --- | --- | --- | --- | --- | --- | --- | --- | --- | --- | --- | --- | --- | --- | --- | --- | --- | --- | --- | --- | --- | --- | --- | --- | --- | --- | --- | --- | --- | --- | --- | --- | --- | --- | --- | --- | --- | --- | --- | --- | --- | --- | --- | --- | --- | --- | --- | --- | --- | --- | --- | --- | --- | --- | --- | --- | --- | --- | --- | --- | --- | --- | --- | --- | --- | --- | --- | --- | --- | --- | --- | --- | --- | --- | --- | --- | --- | --- | --- | --- | --- | --- | --- | --- | --- | --- | --- | --- | --- | --- | --- | --- | --- | --- | --- | --- | --- | --- | --- | --- | --- | --- | --- | --- | --- | --- | --- | --- | --- | --- | --- | --- | --- | --- | --- | --- | --- | --- | --- | --- | --- | --- | --- | --- | --- | --- | --- | --- | --- | --- | --- | --- | --- | --- | --- | --- | --- | --- | --- | --- | --- | --- | --- | --- | --- | --- | --- | --- | --- | --- | --- | --- | --- | --- | --- | --- | --- | --- | --- | --- | --- | --- | --- | --- | --- | --- | --- | --- | --- | --- | --- | --- | --- | --- | --- | --- | --- | --- | --- | --- | --- | --- | --- | --- | --- | --- | --- | --- | --- | --- | --- | --- | --- | --- | --- | --- | --- | --- | --- | --- | --- | --- | --- | --- | --- | --- | --- | --- | --- | --- | --- | --- | --- | --- | --- | --- | --- | --- | --- | --- | --- | --- | --- | --- | --- | --- | --- | --- | --- | --- | --- | --- | --- | --- | --- | --- | --- | --- | --- | --- | --- | --- | --- | --- | --- | --- | --- | --- | --- | --- | --- | --- | --- | --- | --- | --- | --- | --- | --- | --- | --- | --- | --- | --- | --- | --- | --- | --- | --- | --- | --- | --- | --- | --- | --- | --- | --- | --- | --- | --- | --- | --- | --- | --- | --- | --- | --- | --- | --- | --- | --- | --- | --- | --- | --- | --- | --- | --- | --- | --- | --- | --- | --- | --- | --- | --- | --- | --- | --- | --- | --- | --- | --- | --- | --- | --- | --- | --- | --- | --- | --- | --- | --- | --- | --- | --- | --- | --- | --- | --- | --- | --- | --- | --- | --- | --- | --- | --- | --- | --- | --- | --- | --- | --- | --- | --- | --- | --- | --- | --- | --- | --- | --- | --- | --- | --- | --- | --- | --- | --- | --- | --- | --- | --- | --- | --- | --- | --- | --- | --- | --- | --- | --- | --- | --- | --- | --- | --- | --- | --- | --- | --- | --- | --- | --- | --- | --- | --- | --- | --- | --- | --- | --- | --- | --- | --- | --- | --- | --- | --- | --- | --- | --- | --- | --- | --- | --- | --- | --- | --- | --- | --- | --- | --- | --- | --- | --- | --- | --- | --- | --- | --- | --- | --- | --- | --- | --- | --- | --- | --- | --- | --- | --- | --- | --- | --- | --- | --- | --- | --- | --- | --- | --- | --- | --- | --- | --- | --- | --- | --- | --- | --- | --- | --- | --- | --- | --- | --- | --- | --- | --- | --- | --- | --- | --- | --- | --- | --- | --- | --- | --- | --- | --- | --- | --- | --- | --- | --- | --- | --- | --- | --- | --- | --- | --- | --- | --- | --- | --- | --- | --- | --- | --- | --- | --- | --- | --- | --- | --- | --- | --- | --- | --- | --- | --- | --- | --- | --- | --- | --- | --- | --- | --- | --- | --- | --- | --- | --- | --- | --- | --- | --- | --- | --- | --- | --- | --- | --- | --- | --- | --- | --- | --- | --- | --- | --- | --- | --- | --- | --- | --- | --- | --- | --- | --- | --- | --- | --- | --- | --- | --- | --- | --- | --- | --- | --- | --- | --- | --- | --- | --- | --- | --- | --- | --- | --- | --- | --- | --- | --- | --- | --- | --- | --- | --- | --- | --- | --- | --- | --- | --- | --- | --- | --- | --- | --- | --- | --- | --- | --- | --- | --- | --- | --- | --- | --- | --- | --- | --- | --- | --- | --- | --- | --- | --- | --- | --- | --- | --- | --- | --- | --- | --- | --- | --- | --- | --- | --- | --- | --- | --- | --- | --- | --- | --- | --- | --- | --- | --- | --- | --- | --- | --- | --- | --- | --- | --- | --- | --- | --- | --- | --- | --- | --- | --- | --- | --- | --- | --- | --- | --- | --- | --- | --- | --- | --- | --- | --- | --- | --- | --- | --- | --- | --- | --- | --- | --- | --- | --- | --- | --- | --- | --- | --- | --- | --- | --- | --- | --- | --- | --- | --- | --- | --- | --- | --- | --- | --- | --- | --- | --- | --- | --- | --- | --- | --- | --- | --- | --- | --- | --- | --- | --- | --- | --- | --- | --- | --- | --- | --- | --- | --- | --- | --- | --- | --- | --- | --- | --- | --- | --- | --- | --- | --- | --- | --- | --- | --- | --- | --- | --- | --- | --- | --- | --- | --- | --- | --- | --- | --- | --- | --- | --- | --- | --- | --- | --- | --- | --- | --- | --- | --- | --- | --- | --- | --- | --- | --- | --- | --- | --- | --- | --- | --- | --- | --- | --- | --- | --- | --- | --- | --- | --- | --- | --- | --- | --- | --- | --- | --- | --- | --- |
| Actual | Predict  |  |  |  |  |  |  |  |  |  |  |  |  |  |  |  |  |  |  |  |  |  |  |  |  |  |  |  |  |  |  |  |  |  |  |  |  |  |  |  |  |  |  |  |  |  |  |  |  |  |  |  |  |  | | --- | --- | --- | --- | --- | --- | --- | --- | --- | --- | --- | --- | --- | --- | --- | --- | --- | --- | --- | --- | --- | --- | --- | --- | --- | --- | --- | --- | --- | --- | --- | --- | --- | --- | --- | --- | --- | --- | --- | --- | --- | --- | --- | --- | --- | --- | --- | --- | --- | --- | --- | --- | --- | |  | 0 | 1 | 2 | 3 | 4 | 5 | 6 | 7 | 8 | 9 | 10 | 11 | 12 | 13 | 14 | 15 | 16 | 17 | 18 | 19 | 20 | 21 | 22 | 23 | 24 | 25 | 26 | 27 | 28 | 29 | 30 | 31 | 32 | 33 | 34 | 35 | 36 | 37 | 38 | 39 | 40 | 41 | 42 | 43 | 44 | 45 | 46 | 47 | 48 | 49 | 50 | 51 | | 0 | 22 | 1 | 0 | 0 | 0 | 0 | 0 | 0 | 0 | 0 | 0 | 0 | 0 | 0 | 0 | 0 | 0 | 0 | 0 | 0 | 0 | 0 | 0 | 0 | 0 | 0 | 0 | 0 | 0 | 0 | 0 | 0 | 0 | 0 | 0 | 0 | 0 | 0 | 0 | 0 | 0 | 0 | 0 | 0 | 0 | 0 | 0 | 0 | 0 | 0 | 0 | 0 | | 1 | 0 | 71 | 2 | 0 | 1 | 2 | 0 | 0 | 0 | 0 | 0 | 2 | 1 | 1 | 1 | 0 | 0 | 2 | 11 | 0 | 0 | 0 | 1 | 0 | 0 | 0 | 0 | 2 | 3 | 0 | 0 | 0 | 0 | 0 | 0 | 0 | 0 | 0 | 0 | 0 | 0 | 0 | 0 | 0 | 0 | 0 | 0 | 0 | 0 | 0 | 0 | 0 | | 2 | 0 | 0 | 70 | 0 | 0 | 0 | 0 | 0 | 0 | 0 | 0 | 0 | 0 | 0 | 0 | 0 | 0 | 0 | 0 | 0 | 0 | 0 | 0 | 0 | 0 | 0 | 0 | 0 | 0 | 0 | 0 | 0 | 0 | 0 | 0 | 0 | 0 | 0 | 0 | 0 | 0 | 0 | 0 | 0 | 0 | 0 | 0 | 0 | 0 | 0 | 0 | 0 | | 3 | 0 | 3 | 4 | 19 | 1 | 9 | 0 | 0 | 0 | 0 | 0 | 0 | 0 | 0 | 0 | 0 | 0 | 0 | 0 | 0 | 0 | 0 | 0 | 0 | 0 | 0 | 0 | 0 | 0 | 0 | 0 | 0 | 0 | 0 | 0 | 0 | 0 | 0 | 0 | 0 | 0 | 0 | 0 | 0 | 0 | 0 | 0 | 0 | 0 | 0 | 0 | 0 | | 4 | 0 | 0 | 0 | 0 | 127 | 0 | 0 | 0 | 0 | 0 | 0 | 2 | 0 | 7 | 0 | 0 | 0 | 0 | 1 | 0 | 0 | 0 | 6 | 0 | 0 | 0 | 0 | 0 | 0 | 0 | 0 | 0 | 0 | 0 | 0 | 0 | 0 | 0 | 0 | 0 | 0 | 0 | 0 | 0 | 0 | 0 | 0 | 0 | 0 | 0 | 0 | 0 | | 5 | 0 | 0 | 0 | 0 | 1 | 10 | 0 | 0 | 0 | 0 | 0 | 2 | 0 | 1 | 0 | 0 | 0 | 0 | 1 | 0 | 0 | 0 | 7 | 0 | 0 | 0 | 0 | 0 | 0 | 0 | 0 | 0 | 0 | 0 | 0 | 0 | 1 | 0 | 0 | 0 | 0 | 0 | 0 | 2 | 0 | 0 | 0 | 0 | 0 | 0 | 0 | 0 | | 6 | 0 | 0 | 0 | 0 | 0 | 0 | 6 | 0 | 0 | 0 | 0 | 0 | 0 | 0 | 0 | 0 | 0 | 0 | 0 | 0 | 0 | 0 | 0 | 0 | 0 | 0 | 0 | 0 | 0 | 0 | 0 | 0 | 0 | 0 | 0 | 0 | 0 | 0 | 0 | 0 | 0 | 0 | 0 | 0 | 0 | 0 | 0 | 0 | 0 | 0 | 0 | 0 | | 7 | 0 | 0 | 0 | 0 | 0 | 0 | 0 | 11 | 0 | 0 | 0 | 0 | 0 | 0 | 0 | 0 | 0 | 0 | 0 | 0 | 0 | 0 | 0 | 0 | 0 | 0 | 0 | 0 | 0 | 0 | 0 | 0 | 0 | 0 | 0 | 0 | 0 | 0 | 0 | 0 | 0 | 0 | 0 | 0 | 0 | 0 | 0 | 0 | 0 | 0 | 0 | 0 | | 8 | 0 | 0 | 1 | 0 | 13 | 0 | 0 | 0 | 15 | 0 | 0 | 0 | 4 | 0 | 0 | 0 | 0 | 0 | 1 | 0 | 0 | 0 | 0 | 0 | 0 | 0 | 0 | 0 | 0 | 0 | 0 | 0 | 0 | 0 | 0 | 0 | 0 | 0 | 0 | 0 | 0 | 0 | 0 | 0 | 0 | 0 | 0 | 0 | 0 | 0 | 0 | 0 | | 9 | 0 | 0 | 0 | 0 | 0 | 0 | 0 | 0 | 0 | 46 | 0 | 0 | 0 | 3 | 0 | 0 | 0 | 0 | 2 | 0 | 0 | 0 | 0 | 0 | 0 | 0 | 0 | 0 | 0 | 0 | 0 | 0 | 0 | 0 | 0 | 0 | 0 | 0 | 0 | 0 | 0 | 0 | 0 | 0 | 0 | 0 | 0 | 0 | 0 | 0 | 0 | 0 | | 10 | 0 | 0 | 0 | 0 | 0 | 0 | 0 | 0 | 0 | 0 | 7 | 0 | 2 | 1 | 0 | 0 | 0 | 1 | 3 | 0 | 0 | 0 | 0 | 0 | 0 | 0 | 0 | 0 | 0 | 0 | 0 | 0 | 0 | 0 | 0 | 0 | 0 | 0 | 0 | 0 | 0 | 0 | 0 | 0 | 0 | 0 | 0 | 0 | 0 | 0 | 0 | 0 | | 11 | 0 | 1 | 0 | 0 | 0 | 0 | 0 | 0 | 0 | 0 | 0 | 19 | 0 | 1 | 0 | 0 | 0 | 0 | 5 | 0 | 0 | 0 | 0 | 0 | 0 | 0 | 0 | 0 | 0 | 1 | 0 | 0 | 0 | 0 | 0 | 0 | 0 | 0 | 0 | 0 | 0 | 0 | 0 | 0 | 0 | 0 | 0 | 0 | 0 | 0 | 0 | 0 | | 12 | 0 | 0 | 1 | 0 | 2 | 0 | 0 | 0 | 0 | 0 | 0 | 0 | 0 | 0 | 0 | 0 | 1 | 2 | 1 | 0 | 0 | 0 | 1 | 0 | 0 | 0 | 0 | 0 | 0 | 0 | 0 | 0 | 0 | 0 | 0 | 0 | 0 | 0 | 0 | 0 | 0 | 0 | 0 | 0 | 0 | 0 | 0 | 0 | 0 | 0 | 0 | 0 | | 13 | 0 | 1 | 0 | 0 | 13 | 0 | 0 | 0 | 0 | 0 | 0 | 1 | 0 | 12 | 0 | 0 | 0 | 3 | 7 | 0 | 3 | 0 | 4 | 0 | 0 | 0 | 0 | 0 | 0 | 0 | 0 | 0 | 0 | 0 | 3 | 0 | 0 | 0 | 0 | 0 | 0 | 0 | 0 | 0 | 0 | 0 | 0 | 0 | 0 | 0 | 0 | 0 | | 14 | 0 | 0 | 0 | 0 | 0 | 0 | 0 | 0 | 0 | 0 | 0 | 1 | 0 | 0 | 22 | 0 | 0 | 0 | 0 | 0 | 0 | 0 | 0 | 0 | 0 | 0 | 0 | 0 | 0 | 0 | 0 | 0 | 0 | 0 | 0 | 0 | 0 | 0 | 0 | 0 | 0 | 0 | 0 | 0 | 0 | 0 | 0 | 0 | 0 | 0 | 0 | 0 | | 15 | 0 | 0 | 0 | 0 | 2 | 0 | 0 | 0 | 0 | 0 | 0 | 0 | 0 | 3 | 0 | 22 | 0 | 0 | 0 | 0 | 0 | 0 | 0 | 0 | 0 | 0 | 0 | 0 | 0 | 0 | 0 | 0 | 0 | 0 | 0 | 0 | 0 | 0 | 0 | 0 | 0 | 0 | 0 | 0 | 0 | 0 | 0 | 0 | 0 | 0 | 0 | 0 | | 16 | 0 | 0 | 0 | 0 | 1 | 0 | 0 | 0 | 0 | 0 | 0 | 0 | 0 | 0 | 0 | 0 | 14 | 0 | 0 | 0 | 0 | 0 | 0 | 0 | 0 | 0 | 0 | 0 | 0 | 0 | 0 | 0 | 0 | 0 | 0 | 0 | 0 | 0 | 0 | 0 | 0 | 0 | 0 | 0 | 0 | 0 | 0 | 0 | 0 | 0 | 0 | 0 | | 17 | 0 | 10 | 2 | 0 | 3 | 0 | 0 | 0 | 0 | 0 | 0 | 6 | 0 | 2 | 0 | 0 | 0 | 5 | 9 | 1 | 1 | 0 | 0 | 0 | 0 | 0 | 0 | 0 | 0 | 0 | 0 | 0 | 0 | 0 | 0 | 0 | 0 | 0 | 0 | 0 | 0 | 0 | 0 | 0 | 0 | 0 | 0 | 0 | 0 | 0 | 0 | 0 | | 18 | 0 | 1 | 0 | 3 | 6 | 0 | 0 | 0 | 0 | 0 | 0 | 0 | 0 | 7 | 0 | 0 | 0 | 6 | 24 | 0 | 1 | 0 | 1 | 0 | 0 | 0 | 0 | 0 | 0 | 0 | 0 | 0 | 0 | 0 | 1 | 0 | 0 | 0 | 0 | 0 | 0 | 0 | 0 | 0 | 0 | 0 | 0 | 0 | 0 | 0 | 0 | 0 | | 19 | 0 | 0 | 0 | 0 | 0 | 0 | 0 | 0 | 0 | 0 | 0 | 0 | 0 | 0 | 0 | 0 | 0 | 0 | 1 | 1 | 0 | 0 | 2 | 0 | 0 | 0 | 0 | 1 | 0 | 0 | 0 | 0 | 0 | 0 | 0 | 0 | 0 | 0 | 0 | 0 | 0 | 0 | 0 | 0 | 0 | 0 | 0 | 0 | 0 | 0 | 0 | 0 | | 20 | 0 | 0 | 0 | 0 | 1 | 0 | 0 | 0 | 0 | 0 | 0 | 0 | 0 | 8 | 0 | 0 | 0 | 1 | 4 | 0 | 8 | 0 | 0 | 1 | 0 | 0 | 1 | 2 | 0 | 0 | 0 | 0 | 0 | 0 | 0 | 0 | 0 | 0 | 0 | 0 | 0 | 0 | 0 | 0 | 0 | 0 | 0 | 0 | 0 | 0 | 0 | 0 | | 21 | 0 | 0 | 2 | 0 | 0 | 0 | 0 | 0 | 0 | 0 | 0 | 0 | 0 | 2 | 0 | 0 | 0 | 0 | 2 | 0 | 0 | 0 | 1 | 0 | 0 | 0 | 0 | 0 | 0 | 0 | 0 | 0 | 0 | 0 | 0 | 0 | 0 | 0 | 0 | 0 | 0 | 0 | 0 | 0 | 0 | 0 | 0 | 0 | 0 | 0 | 0 | 0 | | 22 | 0 | 0 | 0 | 0 | 0 | 0 | 0 | 0 | 0 | 0 | 0 | 0 | 0 | 0 | 0 | 0 | 0 | 0 | 0 | 0 | 0 | 0 | 62 | 0 | 0 | 0 | 0 | 0 | 0 | 0 | 0 | 0 | 0 | 0 | 0 | 0 | 0 | 0 | 0 | 0 | 0 | 0 | 0 | 0 | 0 | 0 | 0 | 0 | 0 | 0 | 0 | 0 | | 23 | 0 | 0 | 0 | 0 | 5 | 0 | 0 | 0 | 0 | 0 | 0 | 1 | 0 | 8 | 0 | 0 | 0 | 0 | 2 | 0 | 0 | 0 | 3 | 9 | 0 | 0 | 0 | 0 | 0 | 0 | 0 | 0 | 0 | 0 | 0 | 0 | 0 | 0 | 0 | 0 | 0 | 0 | 0 | 0 | 0 | 0 | 0 | 0 | 0 | 0 | 0 | 0 | | 24 | 0 | 0 | 0 | 0 | 1 | 0 | 0 | 0 | 0 | 0 | 0 | 0 | 0 | 0 | 0 | 0 | 0 | 6 | 7 | 0 | 0 | 0 | 0 | 0 | 1 | 1 | 0 | 0 | 0 | 0 | 0 | 0 | 0 | 0 | 0 | 0 | 0 | 0 | 0 | 0 | 0 | 0 | 0 | 0 | 0 | 0 | 0 | 0 | 0 | 0 | 0 | 0 | | 25 | 0 | 0 | 0 | 0 | 3 | 0 | 0 | 0 | 0 | 0 | 0 | 0 | 0 | 1 | 0 | 0 | 0 | 2 | 0 | 0 | 0 | 0 | 0 | 0 | 0 | 19 | 0 | 0 | 0 | 0 | 0 | 0 | 0 | 0 | 0 | 0 | 0 | 0 | 0 | 0 | 0 | 0 | 0 | 0 | 0 | 0 | 0 | 0 | 0 | 0 | 0 | 0 | | 26 | 0 | 0 | 0 | 0 | 2 | 0 | 0 | 0 | 0 | 0 | 0 | 0 | 0 | 4 | 0 | 0 | 0 | 0 | 0 | 0 | 0 | 0 | 7 | 0 | 0 | 0 | 0 | 0 | 0 | 0 | 0 | 0 | 0 | 0 | 0 | 0 | 0 | 0 | 0 | 0 | 0 | 0 | 0 | 0 | 0 | 0 | 0 | 0 | 0 | 0 | 0 | 0 | | 27 | 0 | 0 | 0 | 0 | 11 | 0 | 0 | 0 | 0 | 0 | 0 | 0 | 0 | 1 | 0 | 1 | 0 | 0 | 4 | 0 | 1 | 0 | 7 | 0 | 0 | 0 | 0 | 4 | 0 | 0 | 0 | 0 | 0 | 0 | 0 | 0 | 0 | 0 | 0 | 0 | 0 | 0 | 0 | 0 | 0 | 0 | 0 | 0 | 0 | 0 | 0 | 0 | | 28 | 0 | 2 | 0 | 0 | 0 | 0 | 0 | 0 | 0 | 0 | 0 | 0 | 0 | 0 | 0 | 0 | 0 | 0 | 1 | 0 | 0 | 0 | 0 | 0 | 0 | 0 | 0 | 0 | 4 | 0 | 0 | 0 | 0 | 0 | 0 | 0 | 0 | 0 | 0 | 0 | 0 | 0 | 0 | 0 | 0 | 0 | 0 | 0 | 0 | 0 | 0 | 0 | | 29 | 0 | 1 | 0 | 0 | 0 | 0 | 0 | 0 | 0 | 0 | 0 | 0 | 0 | 0 | 0 | 0 | 0 | 0 | 0 | 0 | 0 | 0 | 2 | 0 | 0 | 0 | 0 | 0 | 0 | 5 | 0 | 0 | 0 | 0 | 0 | 0 | 0 | 0 | 0 | 0 | 0 | 0 | 0 | 0 | 0 | 0 | 0 | 0 | 0 | 0 | 0 | 0 | | 30 | 0 | 0 | 0 | 0 | 1 | 0 | 0 | 0 | 0 | 0 | 0 | 0 | 0 | 1 | 0 | 0 | 0 | 1 | 1 | 0 | 0 | 0 | 0 | 0 | 0 | 0 | 0 | 0 | 0 | 0 | 16 | 0 | 0 | 0 | 0 | 0 | 0 | 0 | 0 | 0 | 0 | 0 | 0 | 0 | 0 | 0 | 0 | 0 | 0 | 0 | 0 | 0 | | 31 | 0 | 0 | 10 | 0 | 4 | 0 | 0 | 0 | 0 | 0 | 0 | 1 | 0 | 7 | 0 | 0 | 0 | 15 | 1 | 0 | 0 | 0 | 1 | 0 | 0 | 0 | 0 | 1 | 0 | 0 | 0 | 5 | 0 | 1 | 5 | 0 | 0 | 0 | 0 | 0 | 0 | 0 | 0 | 0 | 0 | 0 | 0 | 0 | 0 | 0 | 0 | 0 | | 32 | 0 | 0 | 2 | 0 | 0 | 0 | 0 | 0 | 0 | 0 | 0 | 0 | 0 | 4 | 0 | 0 | 0 | 4 | 2 | 0 | 0 | 0 | 0 | 0 | 0 | 0 | 1 | 1 | 0 | 0 | 0 | 1 | 0 | 0 | 0 | 0 | 0 | 0 | 0 | 0 | 0 | 0 | 0 | 0 | 0 | 0 | 0 | 0 | 0 | 0 | 0 | 0 | | 33 | 0 | 1 | 0 | 0 | 0 | 0 | 0 | 0 | 0 | 0 | 0 | 0 | 0 | 0 | 0 | 0 | 0 | 0 | 1 | 0 | 0 | 0 | 0 | 0 | 0 | 0 | 0 | 0 | 0 | 0 | 0 | 0 | 0 | 5 | 0 | 0 | 0 | 0 | 0 | 0 | 0 | 0 | 0 | 0 | 0 | 0 | 0 | 0 | 0 | 0 | 0 | 0 | | 34 | 0 | 0 | 0 | 0 | 0 | 0 | 0 | 0 | 0 | 0 | 0 | 0 | 0 | 0 | 0 | 0 | 0 | 0 | 4 | 0 | 0 | 0 | 0 | 0 | 0 | 0 | 0 | 0 | 0 | 0 | 0 | 0 | 0 | 0 | 51 | 0 | 0 | 0 | 0 | 0 | 0 | 0 | 0 | 0 | 0 | 0 | 0 | 0 | 0 | 0 | 0 | 0 | | 35 | 0 | 0 | 0 | 0 | 0 | 0 | 0 | 0 | 0 | 0 | 0 | 0 | 0 | 0 | 0 | 0 | 0 | 0 | 2 | 0 | 0 | 0 | 0 | 0 | 0 | 0 | 0 | 0 | 0 | 0 | 0 | 0 | 0 | 0 | 0 | 8 | 0 | 0 | 0 | 0 | 0 | 0 | 0 | 0 | 0 | 0 | 0 | 0 | 0 | 0 | 0 | 0 | | 36 | 0 | 0 | 0 | 0 | 0 | 0 | 0 | 0 | 0 | 0 | 0 | 1 | 0 | 0 | 0 | 0 | 0 | 0 | 0 | 0 | 0 | 0 | 0 | 0 | 0 | 0 | 0 | 0 | 0 | 0 | 0 | 0 | 0 | 0 | 0 | 0 | 10 | 0 | 0 | 0 | 0 | 0 | 0 | 0 | 0 | 0 | 0 | 0 | 0 | 0 | 0 | 0 | | 37 | 0 | 0 | 0 | 0 | 0 | 0 | 0 | 0 | 0 | 0 | 0 | 0 | 0 | 0 | 0 | 0 | 0 | 0 | 0 | 0 | 0 | 0 | 0 | 0 | 1 | 0 | 0 | 0 | 0 | 0 | 0 | 0 | 0 | 0 | 0 | 0 | 0 | 6 | 0 | 0 | 0 | 0 | 0 | 0 | 0 | 0 | 0 | 0 | 0 | 0 | 0 | 0 | | 38 | 0 | 0 | 4 | 0 | 0 | 0 | 0 | 0 | 0 | 0 | 0 | 0 | 0 | 0 | 0 | 0 | 0 | 0 | 0 | 0 | 0 | 0 | 0 | 0 | 0 | 0 | 0 | 0 | 0 | 0 | 0 | 0 | 0 | 0 | 0 | 0 | 0 | 0 | 1 | 0 | 0 | 0 | 0 | 0 | 0 | 0 | 0 | 0 | 0 | 0 | 0 | 0 | | 39 | 0 | 0 | 0 | 0 | 0 | 0 | 0 | 0 | 0 | 0 | 0 | 0 | 0 | 0 | 0 | 0 | 0 | 0 | 0 | 0 | 0 | 0 | 0 | 0 | 0 | 0 | 0 | 0 | 0 | 0 | 0 | 0 | 0 | 0 | 0 | 0 | 0 | 0 | 0 | 10 | 0 | 0 | 0 | 0 | 0 | 0 | 0 | 0 | 0 | 0 | 0 | 0 | | 40 | 0 | 0 | 0 | 0 | 4 | 0 | 0 | 0 | 0 | 0 | 0 | 0 | 0 | 0 | 0 | 0 | 0 | 0 | 0 | 0 | 0 | 0 | 0 | 0 | 0 | 0 | 0 | 0 | 0 | 0 | 0 | 0 | 0 | 0 | 0 | 0 | 0 | 0 | 0 | 1 | 0 | 0 | 0 | 0 | 0 | 0 | 0 | 0 | 0 | 0 | 0 | 0 | | 41 | 0 | 0 | 0 | 0 | 0 | 0 | 0 | 0 | 0 | 0 | 0 | 0 | 0 | 4 | 0 | 0 | 0 | 0 | 0 | 0 | 0 | 0 | 1 | 0 | 0 | 0 | 0 | 0 | 0 | 0 | 0 | 0 | 0 | 0 | 1 | 0 | 0 | 0 | 0 | 0 | 0 | 0 | 0 | 0 | 0 | 0 | 0 | 0 | 0 | 0 | 0 | 0 | | 42 | 0 | 0 | 0 | 0 | 0 | 0 | 0 | 0 | 0 | 0 | 0 | 0 | 0 | 6 | 0 | 0 | 0 | 0 | 1 | 0 | 0 | 0 | 0 | 0 | 0 | 0 | 0 | 0 | 0 | 0 | 0 | 0 | 0 | 0 | 0 | 0 | 0 | 0 | 0 | 0 | 0 | 0 | 0 | 0 | 0 | 0 | 0 | 0 | 0 | 0 | 0 | 0 | | 43 | 0 | 0 | 0 | 0 | 0 | 0 | 0 | 0 | 0 | 0 | 0 | 0 | 0 | 0 | 0 | 0 | 0 | 0 | 0 | 0 | 0 | 0 | 3 | 0 | 0 | 0 | 0 | 0 | 0 | 0 | 0 | 0 | 0 | 0 | 0 | 0 | 0 | 0 | 0 | 0 | 0 | 0 | 0 | 11 | 0 | 0 | 0 | 0 | 0 | 0 | 0 | 0 | | 44 | 0 | 4 | 0 | 0 | 0 | 0 | 0 | 0 | 0 | 0 | 0 | 6 | 0 | 11 | 0 | 0 | 2 | 1 | 5 | 0 | 0 | 0 | 1 | 0 | 0 | 0 | 0 | 2 | 7 | 0 | 1 | 0 | 0 | 0 | 0 | 0 | 1 | 0 | 0 | 0 | 0 | 0 | 0 | 0 | 0 | 0 | 0 | 0 | 0 | 0 | 0 | 0 | | 45 | 0 | 0 | 0 | 0 | 2 | 0 | 0 | 0 | 0 | 0 | 0 | 0 | 0 | 4 | 0 | 0 | 0 | 0 | 0 | 0 | 0 | 0 | 0 | 0 | 0 | 0 | 0 | 0 | 0 | 0 | 0 | 0 | 0 | 0 | 0 | 0 | 0 | 0 | 0 | 0 | 0 | 0 | 0 | 0 | 0 | 0 | 0 | 0 | 0 | 0 | 0 | 0 | | 46 | 0 | 0 | 0 | 0 | 0 | 0 | 0 | 0 | 0 | 0 | 0 | 0 | 0 | 0 | 0 | 0 | 0 | 0 | 0 | 0 | 0 | 0 | 0 | 0 | 0 | 0 | 0 | 0 | 0 | 0 | 0 | 0 | 0 | 0 | 0 | 0 | 0 | 0 | 0 | 0 | 0 | 0 | 0 | 0 | 0 | 0 | 8 | 0 | 0 | 0 | 0 | 0 | | 47 | 0 | 0 | 0 | 0 | 3 | 0 | 0 | 0 | 0 | 0 | 0 | 0 | 0 | 1 | 0 | 0 | 0 | 0 | 0 | 0 | 0 | 0 | 0 | 0 | 0 | 0 | 0 | 0 | 0 | 0 | 0 | 0 | 0 | 0 | 0 | 0 | 0 | 0 | 0 | 0 | 0 | 0 | 0 | 0 | 0 | 0 | 0 | 2 | 0 | 0 | 0 | 0 | | 48 | 0 | 2 | 0 | 0 | 0 | 0 | 0 | 0 | 0 | 0 | 0 | 0 | 0 | 1 | 0 | 0 | 0 | 0 | 4 | 0 | 0 | 0 | 0 | 0 | 0 | 0 | 0 | 0 | 0 | 0 | 0 | 0 | 0 | 0 | 0 | 0 | 0 | 0 | 0 | 0 | 0 | 0 | 0 | 0 | 0 | 0 | 0 | 0 | 9 | 0 | 0 | 0 | | 49 | 0 | 0 | 0 | 0 | 1 | 0 | 0 | 0 | 0 | 0 | 0 | 0 | 0 | 2 | 0 | 0 | 0 | 0 | 0 | 0 | 0 | 0 | 0 | 0 | 0 | 0 | 0 | 0 | 5 | 0 | 0 | 0 | 0 | 0 | 0 | 0 | 2 | 0 | 0 | 0 | 0 | 0 | 0 | 0 | 0 | 0 | 0 | 0 | 0 | 0 | 0 | 0 | | 50 | 0 | 0 | 0 | 0 | 1 | 0 | 0 | 0 | 0 | 0 | 0 | 0 | 0 | 0 | 0 | 0 | 0 | 3 | 0 | 0 | 0 | 0 | 0 | 0 | 0 | 0 | 0 | 0 | 0 | 0 | 0 | 0 | 0 | 0 | 0 | 0 | 0 | 0 | 0 | 0 | 0 | 0 | 0 | 0 | 0 | 0 | 0 | 0 | 0 | 0 | 3 | 0 | | 51 | 0 | 0 | 0 | 0 | 0 | 0 | 0 | 0 | 0 | 0 | 0 | 0 | 0 | 0 | 0 | 0 | 0 | 0 | 0 | 0 | 0 | 0 | 0 | 0 | 0 | 0 | 0 | 0 | 0 | 0 | 0 | 0 | 0 | 0 | 0 | 0 | 0 | 0 | 0 | 0 | 0 | 0 | 0 | 0 | 0 | 0 | 0 | 0 | 0 | 0 | 0 | 5 | |

## Overall Statistics :

|  |  |
| --- | --- |
| 95% CI | (0.57864,0.63184) |
| ACC Macro | 0.98482 |
| ARI | 0.43774 |
| AUNP | 0.79237 |
| AUNU | 0.75562 |
| Bangdiwala B | 0.49836 |
| Bennett S | 0.5975 |
| CBA | 0.4706 |
| CSI | None |
| Chi-Squared | None |
| Chi-Squared DF | 2601 |
| Conditional Entropy | 1.34744 |
| Cramer V | None |
| Cross Entropy | 4.79807 |
| F1 Macro | 0.53221 |
| F1 Micro | 0.60524 |
| FNR Macro | 0.48078 |
| FNR Micro | 0.39476 |
| FPR Macro | 0.00799 |
| FPR Micro | 0.00774 |
| Gwet AC1 | 0.59775 |
| Hamming Loss | 0.39476 |
| Joint Entropy | 6.47144 |
| KL Divergence | None |
| Kappa | 0.58559 |
| Kappa 95% CI | (0.55767,0.61352) |
| Kappa No Prevalence | 0.21049 |
| Kappa Standard Error | 0.01425 |
| Kappa Unbiased | 0.58426 |
| Krippendorff Alpha | 0.58442 |
| Lambda A | 0.57192 |
| Lambda B | 0.6057 |
| Mutual Information | 3.13907 |
| NIR | 0.11025 |
| Overall ACC | 0.60524 |
| Overall CEN | 0.24992 |
| Overall J | (23.22346,0.44661) |
| Overall MCC | 0.58942 |
| Overall MCEN | 0.32985 |
| Overall RACC | 0.04742 |
| Overall RACCU | 0.05047 |
| P-Value | None |
| PPV Macro | None |
| PPV Micro | 0.60524 |
| Pearson C | None |
| Phi-Squared | None |
| RCI | 0.61262 |
| RR | 24.94231 |
| Reference Entropy | 5.12399 |
| Response Entropy | 4.48651 |
| SOA1(Landis & Koch) | Moderate |
| SOA2(Fleiss) | Intermediate to Good |
| SOA3(Altman) | Moderate |
| SOA4(Cicchetti) | Fair |
| SOA5(Cramer) | None |
| SOA6(Matthews) | Moderate |
| Scott PI | 0.58426 |
| Standard Error | 0.01357 |
| TNR Macro | 0.99201 |
| TNR Micro | 0.99226 |
| TPR Macro | 0.51922 |
| TPR Micro | 0.60524 |
| Zero-one Loss | 512 |

## Class Statistics :

|  |  |  |  |  |  |  |  |  |  |  |  |  |  |  |  |  |  |  |  |  |  |  |  |  |  |  |  |  |  |  |  |  |  |  |  |  |  |  |  |  |  |  |  |  |  |  |  |  |  |  |  |  |  |
| --- | --- | --- | --- | --- | --- | --- | --- | --- | --- | --- | --- | --- | --- | --- | --- | --- | --- | --- | --- | --- | --- | --- | --- | --- | --- | --- | --- | --- | --- | --- | --- | --- | --- | --- | --- | --- | --- | --- | --- | --- | --- | --- | --- | --- | --- | --- | --- | --- | --- | --- | --- | --- | --- |
| Class | 0 | 1 | 2 | 3 | 4 | 5 | 6 | 7 | 8 | 9 | 10 | 11 | 12 | 13 | 14 | 15 | 16 | 17 | 18 | 19 | 20 | 21 | 22 | 23 | 24 | 25 | 26 | 27 | 28 | 29 | 30 | 31 | 32 | 33 | 34 | 35 | 36 | 37 | 38 | 39 | 40 | 41 | 42 | 43 | 44 | 45 | 46 | 47 | 48 | 49 | 50 | 51 | Description |
| ACC | 0.99923 | 0.95682 | 0.97841 | 0.98458 | 0.92444 | 0.97995 | 1.0 | 1.0 | 0.98535 | 0.99614 | 0.9946 | 0.9761 | 0.98843 | 0.90285 | 0.99846 | 0.99537 | 0.99692 | 0.93755 | 0.91596 | 0.99614 | 0.9815 | 0.9946 | 0.96299 | 0.98458 | 0.98766 | 0.9946 | 0.98843 | 0.97379 | 0.98612 | 0.99692 | 0.99614 | 0.96376 | 0.98843 | 0.99769 | 0.98921 | 0.99846 | 0.99614 | 0.99923 | 0.99692 | 0.99923 | 0.99614 | 0.99537 | 0.9946 | 0.99614 | 0.96839 | 0.99537 | 1.0 | 0.99692 | 0.9946 | 0.99229 | 0.99692 | 1.0 | Accuracy |
| AGF | 0.98199 | 0.83417 | 0.96001 | 0.75226 | 0.88966 | 0.63924 | 1.0 | 1.0 | 0.70056 | 0.95763 | 0.74373 | 0.79234 | 0.0 | 0.44529 | 0.97764 | 0.91484 | 0.95294 | 0.34156 | 0.6154 | 0.4761 | 0.57867 | 0.0 | 0.92681 | 0.60368 | 0.27394 | 0.88801 | 0.0 | 0.39038 | 0.65095 | 0.81029 | 0.90695 | 0.34002 | 0.0 | 0.85689 | 0.95061 | 0.9123 | 0.9279 | 0.93905 | 0.48735 | 0.99007 | 0.0 | 0.0 | 0.0 | 0.89183 | 0.0 | 0.0 | 1.0 | 0.61941 | 0.78343 | 0.0 | 0.69475 | 1.0 | Adjusted F-score |
| AGM | 0.98891 | 0.90236 | 0.98301 | 0.85971 | 0.91802 | 0.80877 | 1.0 | 1.0 | 0.82988 | 0.97435 | 0.85276 | 0.90577 | 0 | 0.70281 | 0.98833 | 0.95025 | 0.98122 | 0.65231 | 0.79881 | 0.7226 | 0.7721 | 0 | 0.97099 | 0.78061 | 0.62224 | 0.9347 | 0 | 0.67797 | 0.86963 | 0.89442 | 0.94624 | 0.64921 | 0 | 0.92182 | 0.97515 | 0.94701 | 0.97434 | 0.96281 | 0.72307 | 0.99942 | 0 | 0 | 0 | 0.94177 | 0 | 0 | 1.0 | 0.78819 | 0.87422 | 0 | 0.82686 | 1.0 | Adjusted geometric mean |
| AM | -1 | -2 | 28 | -14 | 66 | -4 | 0 | 0 | -19 | -5 | -7 | 15 | -1 | 56 | 0 | -4 | 2 | 13 | 57 | -3 | -12 | -7 | 48 | -18 | -14 | -5 | -11 | -16 | 12 | -2 | -3 | -45 | -15 | -1 | 6 | -2 | 3 | -1 | -4 | 1 | -5 | -6 | -7 | -1 | -41 | -6 | 0 | -4 | -7 | -10 | -4 | 0 | Difference between automatic and manual classification |
| AUC | 0.97826 | 0.84372 | 0.98859 | 0.7627 | 0.90853 | 0.69568 | 1.0 | 1.0 | 0.72059 | 0.95098 | 0.75 | 0.8428 | 0.49728 | 0.59126 | 0.97787 | 0.90701 | 0.9655 | 0.54542 | 0.70672 | 0.59961 | 0.65149 | 0.5 | 0.98057 | 0.66032 | 0.53086 | 0.87961 | 0.49922 | 0.56542 | 0.7799 | 0.81211 | 0.89961 | 0.54862 | 0.5 | 0.85676 | 0.95961 | 0.9 | 0.95299 | 0.92857 | 0.6 | 0.99961 | 0.5 | 0.5 | 0.5 | 0.89208 | 0.5 | 0.5 | 1.0 | 0.66667 | 0.78125 | 0.5 | 0.71429 | 1.0 | Area under the ROC curve |
| AUCI | Excellent | Very Good | Excellent | Good | Excellent | Fair | Excellent | Excellent | Good | Excellent | Good | Very Good | Poor | Poor | Excellent | Excellent | Excellent | Poor | Good | Poor | Fair | Poor | Excellent | Fair | Poor | Very Good | Poor | Poor | Good | Very Good | Very Good | Poor | Poor | Very Good | Excellent | Excellent | Excellent | Excellent | Fair | Excellent | Poor | Poor | Poor | Very Good | Poor | Poor | Excellent | Fair | Good | Poor | Good | Excellent | AUC value interpretation |
| AUPR | 0.97826 | 0.71724 | 0.85714 | 0.69571 | 0.74788 | 0.4381 | 1.0 | 1.0 | 0.72059 | 0.95098 | 0.75 | 0.57804 | 0.0 | 0.18591 | 0.95652 | 0.88567 | 0.87843 | 0.11218 | 0.35215 | 0.35 | 0.43956 | None | 0.78182 | 0.61071 | 0.28125 | 0.855 | 0.0 | 0.22281 | 0.39098 | 0.72917 | 0.87059 | 0.46569 | None | 0.77381 | 0.88167 | 0.9 | 0.81169 | 0.92857 | 0.6 | 0.95455 | None | None | None | 0.81593 | None | None | 1.0 | 0.66667 | 0.78125 | None | 0.71429 | 1.0 | Area under the PR curve |
| BCD | 0.00039 | 0.00077 | 0.01079 | 0.0054 | 0.02544 | 0.00154 | 0.0 | 0.0 | 0.00732 | 0.00193 | 0.0027 | 0.00578 | 0.00039 | 0.02159 | 0.0 | 0.00154 | 0.00077 | 0.00501 | 0.02197 | 0.00116 | 0.00463 | 0.0027 | 0.0185 | 0.00694 | 0.0054 | 0.00193 | 0.00424 | 0.00617 | 0.00463 | 0.00077 | 0.00116 | 0.01735 | 0.00578 | 0.00039 | 0.00231 | 0.00077 | 0.00116 | 0.00039 | 0.00154 | 0.00039 | 0.00193 | 0.00231 | 0.0027 | 0.00039 | 0.01581 | 0.00231 | 0.0 | 0.00154 | 0.0027 | 0.00386 | 0.00154 | 0.0 | Bray-Curtis dissimilarity |
| BM | 0.95652 | 0.68744 | 0.97718 | 0.5254 | 0.81705 | 0.39135 | 1.0 | 1.0 | 0.44118 | 0.90196 | 0.5 | 0.68559 | -0.00543 | 0.18252 | 0.95574 | 0.81403 | 0.93099 | 0.09084 | 0.41344 | 0.19923 | 0.30297 | 0.0 | 0.96113 | 0.32064 | 0.06172 | 0.75921 | -0.00156 | 0.13083 | 0.5598 | 0.62422 | 0.79922 | 0.09724 | 0.0 | 0.71351 | 0.91922 | 0.8 | 0.90598 | 0.85714 | 0.2 | 0.99922 | 0.0 | 0.0 | 0.0 | 0.78416 | 0.0 | 0.0 | 1.0 | 0.33333 | 0.5625 | 0.0 | 0.42857 | 1.0 | Informedness or bookmaker informedness |
| CEN | 0.01829 | 0.24559 | 0.1336 | 0.18377 | 0.25215 | 0.29137 | 0 | 0 | 0.15468 | 0.04055 | 0.17122 | 0.30568 | 0.44565 | 0.61225 | 0.03599 | 0.08125 | 0.0843 | 0.54017 | 0.53822 | 0.25771 | 0.33335 | 0.29228 | 0.22697 | 0.24687 | 0.26275 | 0.10554 | 0.28927 | 0.39835 | 0.26857 | 0.14162 | 0.10551 | 0.37615 | 0.3857 | 0.12798 | 0.09257 | 0.05279 | 0.12721 | 0.04266 | 0.05845 | 0.03135 | 0.1082 | 0.18758 | 0.08867 | 0.09447 | 0.44901 | 0.13763 | 0 | 0.13573 | 0.13493 | 0.26392 | 0.12788 | 0 | Confusion entropy |
| DOR | None | 106.09195 | None | 468.66667 | 103.76829 | 76.42424 | None | None | None | None | None | 128.7663 | 0.0 | 4.36672 | 28006.0 | 5583.6 | 5968.66667 | 3.78911 | 12.94532 | 322.75 | 93.7037 | None | None | 600.63158 | 85.33333 | 4024.83333 | 0.0 | 22.38222 | 113.33333 | 2146.66667 | 5104.0 | 135.32609 | None | 3222.5 | 1570.8 | None | 3205.0 | None | None | None | None | None | None | 2348.5 | None | None | None | None | None | None | None | None | Diagnostic odds ratio |
| DP | None | 1.11682 | None | 1.47252 | 1.11151 | 1.03828 | None | None | None | None | None | 1.16319 | None | 0.35294 | 2.4519 | 2.06578 | 2.08175 | 0.31896 | 0.61314 | 1.38321 | 1.08709 | None | None | 1.53193 | 1.06468 | 1.9874 | None | 0.74424 | 1.13263 | 1.8369 | 2.04428 | 1.17509 | None | 1.93417 | 1.76211 | None | 1.93286 | None | None | None | None | None | None | 1.85841 | None | None | None | None | None | None | None | None | Discriminant power |
| DPI | None | Limited | None | Limited | Limited | Limited | None | None | None | None | None | Limited | None | Poor | Fair | Fair | Fair | Poor | Poor | Limited | Limited | None | None | Limited | Limited | Limited | None | Poor | Limited | Limited | Fair | Limited | None | Limited | Limited | None | Limited | None | None | None | None | None | None | Limited | None | None | None | None | None | None | None | None | Discriminant power interpretation |
| ERR | 0.00077 | 0.04318 | 0.02159 | 0.01542 | 0.07556 | 0.02005 | 0.0 | 0.0 | 0.01465 | 0.00386 | 0.0054 | 0.0239 | 0.01157 | 0.09715 | 0.00154 | 0.00463 | 0.00308 | 0.06245 | 0.08404 | 0.00386 | 0.0185 | 0.0054 | 0.03701 | 0.01542 | 0.01234 | 0.0054 | 0.01157 | 0.02621 | 0.01388 | 0.00308 | 0.00386 | 0.03624 | 0.01157 | 0.00231 | 0.01079 | 0.00154 | 0.00386 | 0.00077 | 0.00308 | 0.00077 | 0.00386 | 0.00463 | 0.0054 | 0.00386 | 0.03161 | 0.00463 | 0.0 | 0.00308 | 0.0054 | 0.00771 | 0.00308 | 0.0 | Error rate |
| F0.5 | 0.99099 | 0.72154 | 0.75758 | 0.76613 | 0.64862 | 0.45872 | 1.0 | 1.0 | 0.79787 | 0.97872 | 0.83333 | 0.48718 | 0.0 | 0.13072 | 0.95652 | 0.92437 | 0.84337 | 0.10121 | 0.25105 | 0.38462 | 0.4878 | 0.0 | 0.61753 | 0.66176 | 0.20833 | 0.90476 | 0.0 | 0.24691 | 0.24096 | 0.78125 | 0.90909 | 0.33333 | 0.0 | 0.80645 | 0.85284 | 0.95238 | 0.74627 | 0.96774 | 0.55556 | 0.92593 | 0.0 | 0.0 | 0.0 | 0.83333 | 0.0 | 0.0 | 1.0 | 0.71429 | 0.86538 | 0.0 | 0.78947 | 1.0 | F0.5 score |
| F1 | 0.97778 | 0.71717 | 0.83333 | 0.65517 | 0.72159 | 0.43478 | 1.0 | 1.0 | 0.61224 | 0.94845 | 0.66667 | 0.55072 | 0.0 | 0.16 | 0.95652 | 0.88 | 0.875 | 0.10989 | 0.30573 | 0.28571 | 0.4 | 0.0 | 0.72093 | 0.47368 | 0.11111 | 0.84444 | 0.0 | 0.19048 | 0.30769 | 0.71429 | 0.86486 | 0.17544 | 0.0 | 0.76923 | 0.87931 | 0.88889 | 0.8 | 0.92308 | 0.33333 | 0.95238 | 0.0 | 0.0 | 0.0 | 0.81481 | 0.0 | 0.0 | 1.0 | 0.5 | 0.72 | 0.0 | 0.6 | 1.0 | F1 score - harmonic mean of precision and sensitivity |
| F2 | 0.96491 | 0.71285 | 0.92593 | 0.57229 | 0.81306 | 0.41322 | 1.0 | 1.0 | 0.49669 | 0.92 | 0.55556 | 0.63333 | 0.0 | 0.20619 | 0.95652 | 0.83969 | 0.90909 | 0.12019 | 0.39088 | 0.22727 | 0.33898 | 0.0 | 0.86592 | 0.36885 | 0.07576 | 0.79167 | 0.0 | 0.15504 | 0.42553 | 0.65789 | 0.82474 | 0.11905 | 0.0 | 0.73529 | 0.90747 | 0.83333 | 0.86207 | 0.88235 | 0.2381 | 0.98039 | 0.0 | 0.0 | 0.0 | 0.7971 | 0.0 | 0.0 | 1.0 | 0.38462 | 0.61644 | 0.0 | 0.48387 | 1.0 | F2 score |
| FDR | 0.0 | 0.27551 | 0.28571 | 0.13636 | 0.39234 | 0.52381 | 0.0 | 0.0 | 0.0 | 0.0 | 0.0 | 0.54762 | 1.0 | 0.8835 | 0.04348 | 0.04348 | 0.17647 | 0.90385 | 0.7757 | 0.5 | 0.42857 | None | 0.43636 | 0.1 | 0.5 | 0.05 | 1.0 | 0.69231 | 0.78947 | 0.16667 | 0.05882 | 0.16667 | None | 0.16667 | 0.16393 | 0.0 | 0.28571 | 0.0 | 0.0 | 0.09091 | None | None | None | 0.15385 | None | None | 0.0 | 0.0 | 0.0 | None | 0.0 | 0.0 | False discovery rate |
| FN | 1 | 29 | 0 | 17 | 16 | 15 | 0 | 0 | 19 | 5 | 7 | 8 | 8 | 35 | 1 | 5 | 1 | 34 | 26 | 4 | 18 | 7 | 0 | 19 | 15 | 6 | 13 | 25 | 3 | 3 | 4 | 46 | 15 | 2 | 4 | 2 | 1 | 1 | 4 | 0 | 5 | 6 | 7 | 3 | 41 | 6 | 0 | 4 | 7 | 10 | 4 | 0 | False negative/miss/type 2 error |
| FNR | 0.04348 | 0.29 | 0.0 | 0.47222 | 0.11189 | 0.6 | 0.0 | 0.0 | 0.55882 | 0.09804 | 0.5 | 0.2963 | 1.0 | 0.74468 | 0.04348 | 0.18519 | 0.06667 | 0.87179 | 0.52 | 0.8 | 0.69231 | 1.0 | 0.0 | 0.67857 | 0.9375 | 0.24 | 1.0 | 0.86207 | 0.42857 | 0.375 | 0.2 | 0.90196 | 1.0 | 0.28571 | 0.07273 | 0.2 | 0.09091 | 0.14286 | 0.8 | 0.0 | 1.0 | 1.0 | 1.0 | 0.21429 | 1.0 | 1.0 | 0.0 | 0.66667 | 0.4375 | 1.0 | 0.57143 | 0.0 | Miss rate or false negative rate |
| FOR | 0.00078 | 0.02419 | 0.0 | 0.01333 | 0.01471 | 0.01176 | 0.0 | 0.0 | 0.01482 | 0.004 | 0.00543 | 0.00637 | 0.0062 | 0.02931 | 0.00078 | 0.00392 | 0.00078 | 0.02731 | 0.02185 | 0.00309 | 0.01403 | 0.0054 | 0.0 | 0.01476 | 0.01158 | 0.0047 | 0.01004 | 0.01947 | 0.00235 | 0.00232 | 0.00313 | 0.03563 | 0.01157 | 0.00155 | 0.00324 | 0.00155 | 0.00078 | 0.00077 | 0.00309 | 0.0 | 0.00386 | 0.00463 | 0.0054 | 0.00234 | 0.03161 | 0.00463 | 0.0 | 0.00309 | 0.00543 | 0.00771 | 0.00309 | 0.0 | False omission rate |
| FP | 0 | 27 | 28 | 3 | 82 | 11 | 0 | 0 | 0 | 0 | 0 | 23 | 7 | 91 | 1 | 1 | 3 | 47 | 83 | 1 | 6 | 0 | 48 | 1 | 1 | 1 | 2 | 9 | 15 | 1 | 1 | 1 | 0 | 1 | 10 | 0 | 4 | 0 | 0 | 1 | 0 | 0 | 0 | 2 | 0 | 0 | 0 | 0 | 0 | 0 | 0 | 0 | False positive/type 1 error/false alarm |
| FPR | 0.0 | 0.02256 | 0.02282 | 0.00238 | 0.07106 | 0.00865 | 0.0 | 0.0 | 0.0 | 0.0 | 0.0 | 0.01811 | 0.00543 | 0.0728 | 0.00078 | 0.00079 | 0.00234 | 0.03736 | 0.06656 | 0.00077 | 0.00472 | 0.0 | 0.03887 | 0.00079 | 0.00078 | 0.00079 | 0.00156 | 0.0071 | 0.01163 | 0.00078 | 0.00078 | 0.0008 | 0.0 | 0.00078 | 0.00805 | 0.0 | 0.00311 | 0.0 | 0.0 | 0.00078 | 0.0 | 0.0 | 0.0 | 0.00156 | 0.0 | 0.0 | 0.0 | 0.0 | 0.0 | 0.0 | 0.0 | 0.0 | Fall-out or false positive rate |
| G | 0.97802 | 0.71721 | 0.84515 | 0.67514 | 0.73462 | 0.43644 | 1.0 | 1.0 | 0.66421 | 0.94972 | 0.70711 | 0.56422 | 0.0 | 0.17247 | 0.95652 | 0.88283 | 0.87671 | 0.11103 | 0.32812 | 0.31623 | 0.41931 | None | 0.75076 | 0.53785 | 0.17678 | 0.84971 | 0.0 | 0.20601 | 0.34684 | 0.72169 | 0.86772 | 0.28583 | None | 0.77152 | 0.88049 | 0.89443 | 0.80582 | 0.92582 | 0.44721 | 0.95346 | None | None | None | 0.81537 | None | None | 1.0 | 0.57735 | 0.75 | None | 0.65465 | 1.0 | G-measure geometric mean of precision and sensitivity |
| GI | 0.95652 | 0.68744 | 0.97718 | 0.5254 | 0.81705 | 0.39135 | 1.0 | 1.0 | 0.44118 | 0.90196 | 0.5 | 0.68559 | -0.00543 | 0.18252 | 0.95574 | 0.81403 | 0.93099 | 0.09084 | 0.41344 | 0.19923 | 0.30297 | 0.0 | 0.96113 | 0.32064 | 0.06172 | 0.75921 | -0.00156 | 0.13083 | 0.5598 | 0.62422 | 0.79922 | 0.09724 | 0.0 | 0.71351 | 0.91922 | 0.8 | 0.90598 | 0.85714 | 0.2 | 0.99922 | 0.0 | 0.0 | 0.0 | 0.78416 | 0.0 | 0.0 | 1.0 | 0.33333 | 0.5625 | 0.0 | 0.42857 | 1.0 | Gini index |
| GM | 0.97802 | 0.83306 | 0.98852 | 0.72562 | 0.9083 | 0.62971 | 1.0 | 1.0 | 0.66421 | 0.94972 | 0.70711 | 0.83124 | 0.0 | 0.48655 | 0.97764 | 0.90232 | 0.96496 | 0.35131 | 0.66937 | 0.44704 | 0.55339 | 0.0 | 0.98037 | 0.56672 | 0.2499 | 0.87144 | 0.0 | 0.37007 | 0.75152 | 0.79026 | 0.89408 | 0.31299 | 0.0 | 0.84483 | 0.95907 | 0.89443 | 0.95198 | 0.92582 | 0.44721 | 0.99961 | 0.0 | 0.0 | 0.0 | 0.88571 | 0.0 | 0.0 | 1.0 | 0.57735 | 0.75 | 0.0 | 0.65465 | 1.0 | G-mean geometric mean of specificity and sensitivity |
| IBA | 0.91493 | 0.50838 | 0.99948 | 0.27914 | 0.79132 | 0.16205 | 1.0 | 1.0 | 0.19464 | 0.81353 | 0.25 | 0.49874 | 0.0 | 0.07768 | 0.91497 | 0.66404 | 0.87125 | 0.02043 | 0.24489 | 0.04012 | 0.09567 | 0.0 | 0.99849 | 0.10349 | 0.00395 | 0.57774 | 0.0 | 0.01986 | 0.3293 | 0.39081 | 0.64012 | 0.00968 | 0.0 | 0.51036 | 0.86032 | 0.64 | 0.82669 | 0.73469 | 0.04 | 1.0 | 0.0 | 0.0 | 0.0 | 0.61761 | 0.0 | 0.0 | 1.0 | 0.11111 | 0.31641 | 0.0 | 0.18367 | 1.0 | Index of balanced accuracy |
| ICSI | 0.95652 | 0.43449 | 0.71429 | 0.39141 | 0.49577 | -0.12381 | 1.0 | 1.0 | 0.44118 | 0.90196 | 0.5 | 0.15608 | -1.0 | -0.62818 | 0.91304 | 0.77134 | 0.75686 | -0.77564 | -0.2957 | -0.3 | -0.12088 | None | 0.56364 | 0.22143 | -0.4375 | 0.71 | -1.0 | -0.55438 | -0.21805 | 0.45833 | 0.74118 | -0.06863 | None | 0.54762 | 0.76334 | 0.8 | 0.62338 | 0.85714 | 0.2 | 0.90909 | None | None | None | 0.63187 | None | None | 1.0 | 0.33333 | 0.5625 | None | 0.42857 | 1.0 | Individual classification success index |
| IS | 5.8174 | 3.23214 | 3.72625 | 4.95953 | 2.46242 | 4.62672 | 7.756 | 6.88153 | 5.2535 | 4.66854 | 6.53361 | 4.44169 | None | 1.68484 | 5.75327 | 5.52194 | 6.15396 | 1.67705 | 2.5406 | 7.01903 | 4.83317 | None | 3.5596 | 5.3816 | 5.34096 | 5.62311 | None | 3.78254 | 5.28568 | 7.07793 | 5.93157 | 4.4055 | None | 7.27057 | 4.30129 | 7.01903 | 6.3961 | 7.53361 | 8.01903 | 6.88153 | None | None | None | 6.2926 | None | None | 7.34096 | 7.756 | 6.34096 | None | 7.53361 | 8.01903 | Information score |
| J | 0.95652 | 0.55906 | 0.71429 | 0.48718 | 0.56444 | 0.27778 | 1.0 | 1.0 | 0.44118 | 0.90196 | 0.5 | 0.38 | 0.0 | 0.08696 | 0.91667 | 0.78571 | 0.77778 | 0.05814 | 0.18045 | 0.16667 | 0.25 | 0.0 | 0.56364 | 0.31034 | 0.05882 | 0.73077 | 0.0 | 0.10526 | 0.18182 | 0.55556 | 0.7619 | 0.09615 | 0.0 | 0.625 | 0.78462 | 0.8 | 0.66667 | 0.85714 | 0.2 | 0.90909 | 0.0 | 0.0 | 0.0 | 0.6875 | 0.0 | 0.0 | 1.0 | 0.33333 | 0.5625 | 0.0 | 0.42857 | 1.0 | Jaccard index |
| LS | 56.3913 | 9.39663 | 13.23469 | 31.1149 | 5.51139 | 24.70476 | 216.16667 | 117.90909 | 38.14706 | 25.43137 | 92.64286 | 21.73104 | 0.0 | 3.21504 | 53.93951 | 45.94847 | 71.20784 | 3.19773 | 5.81832 | 129.7 | 28.50549 | None | 11.79091 | 41.68929 | 40.53125 | 49.286 | 0.0 | 13.76127 | 39.00752 | 135.10417 | 61.03529 | 21.19281 | None | 154.40476 | 19.71595 | 129.7 | 84.22078 | 185.28571 | 259.4 | 117.90909 | None | None | None | 78.39011 | None | None | 162.125 | 216.16667 | 81.0625 | None | 185.28571 | 259.4 | Lift score |
| MCC | 0.97764 | 0.69384 | 0.83546 | 0.66839 | 0.69604 | 0.42633 | 1.0 | 1.0 | 0.65927 | 0.94782 | 0.70519 | 0.55297 | -0.0058 | 0.12615 | 0.95574 | 0.88059 | 0.8752 | 0.07908 | 0.28931 | 0.31464 | 0.41095 | None | 0.73602 | 0.53277 | 0.17362 | 0.84716 | -0.00395 | 0.19419 | 0.34138 | 0.72023 | 0.86586 | 0.27851 | None | 0.77038 | 0.87496 | 0.89373 | 0.804 | 0.92546 | 0.44652 | 0.95309 | None | None | None | 0.81344 | None | None | 1.0 | 0.57646 | 0.74796 | None | 0.65364 | 1.0 | Matthews correlation coefficient |
| MCCI | Very Strong | Moderate | Strong | Moderate | Moderate | Weak | Very Strong | Very Strong | Moderate | Very Strong | Strong | Moderate | Negligible | Negligible | Very Strong | Strong | Strong | Negligible | Negligible | Weak | Weak | None | Strong | Moderate | Negligible | Strong | Negligible | Negligible | Weak | Strong | Strong | Negligible | None | Strong | Strong | Strong | Strong | Very Strong | Weak | Very Strong | None | None | None | Strong | None | None | Very Strong | Moderate | Strong | None | Moderate | Very Strong | Matthews correlation coefficient interpretation |
| MCEN | 0.02948 | 0.34055 | 0.19574 | 0.2293 | 0.35232 | 0.33403 | 0 | 0 | 0.17877 | 0.0635 | 0.21299 | 0.37866 | 0.44565 | 0.64903 | 0.05726 | 0.11823 | 0.12223 | 0.56007 | 0.60595 | 0.27288 | 0.38051 | 0.29228 | 0.31273 | 0.28318 | 0.26658 | 0.15072 | 0.28927 | 0.42092 | 0.28785 | 0.17784 | 0.15673 | 0.39438 | 0.3857 | 0.1686 | 0.13822 | 0.0696 | 0.17519 | 0.06011 | 0.0386 | 0.04713 | 0.1082 | 0.18758 | 0.08867 | 0.12407 | 0.44901 | 0.13763 | 0 | 0.1395 | 0.1686 | 0.26392 | 0.13862 | 0 | Modified confusion entropy |
| MK | 0.99922 | 0.7003 | 0.71429 | 0.8503 | 0.59295 | 0.46443 | 1.0 | 1.0 | 0.98518 | 0.996 | 0.99457 | 0.44601 | -0.0062 | 0.08719 | 0.95574 | 0.9526 | 0.82275 | 0.06884 | 0.20245 | 0.49691 | 0.5574 | None | 0.56364 | 0.88524 | 0.48842 | 0.9453 | -0.01004 | 0.28822 | 0.20818 | 0.83101 | 0.93805 | 0.7977 | None | 0.83178 | 0.83283 | 0.99845 | 0.71351 | 0.99923 | 0.99691 | 0.90909 | None | None | None | 0.84382 | None | None | 1.0 | 0.99691 | 0.99457 | None | 0.99691 | 1.0 | Markedness |
| N | 1274 | 1197 | 1227 | 1261 | 1154 | 1272 | 1291 | 1286 | 1263 | 1246 | 1283 | 1270 | 1289 | 1250 | 1274 | 1270 | 1282 | 1258 | 1247 | 1292 | 1271 | 1290 | 1235 | 1269 | 1281 | 1272 | 1284 | 1268 | 1290 | 1289 | 1277 | 1246 | 1282 | 1290 | 1242 | 1287 | 1286 | 1290 | 1292 | 1287 | 1292 | 1291 | 1290 | 1283 | 1256 | 1291 | 1289 | 1291 | 1281 | 1287 | 1290 | 1292 | Condition negative |
| NLR | 0.04348 | 0.29669 | 0.0 | 0.47335 | 0.12045 | 0.60523 | 0.0 | 0.0 | 0.55882 | 0.09804 | 0.5 | 0.30176 | 1.00546 | 0.80315 | 0.04351 | 0.18533 | 0.06682 | 0.90563 | 0.55708 | 0.80062 | 0.69559 | 1.0 | 0.0 | 0.67911 | 0.93823 | 0.24019 | 1.00156 | 0.86823 | 0.43361 | 0.37529 | 0.20016 | 0.90269 | 1.0 | 0.28594 | 0.07332 | 0.2 | 0.09119 | 0.14286 | 0.8 | 0.0 | 1.0 | 1.0 | 1.0 | 0.21462 | 1.0 | 1.0 | 0.0 | 0.66667 | 0.4375 | 1.0 | 0.57143 | 0.0 | Negative likelihood ratio |
| NLRI | Good | Poor | Good | Poor | Fair | Negligible | Good | Good | Negligible | Good | Negligible | Poor | Negligible | Negligible | Good | Fair | Good | Negligible | Negligible | Negligible | Negligible | Negligible | Good | Negligible | Negligible | Poor | Negligible | Negligible | Poor | Poor | Poor | Negligible | Negligible | Poor | Good | Fair | Good | Fair | Negligible | Good | Negligible | Negligible | Negligible | Poor | Negligible | Negligible | Good | Negligible | Poor | Negligible | Negligible | Good | Negative likelihood ratio interpretation |
| NPV | 0.99922 | 0.97581 | 1.0 | 0.98667 | 0.98529 | 0.98824 | 1.0 | 1.0 | 0.98518 | 0.996 | 0.99457 | 0.99363 | 0.9938 | 0.97069 | 0.99922 | 0.99608 | 0.99922 | 0.97269 | 0.97815 | 0.99691 | 0.98597 | 0.9946 | 1.0 | 0.98524 | 0.98842 | 0.9953 | 0.98996 | 0.98053 | 0.99765 | 0.99768 | 0.99688 | 0.96437 | 0.98843 | 0.99845 | 0.99676 | 0.99845 | 0.99922 | 0.99923 | 0.99691 | 1.0 | 0.99614 | 0.99537 | 0.9946 | 0.99766 | 0.96839 | 0.99537 | 1.0 | 0.99691 | 0.99457 | 0.99229 | 0.99691 | 1.0 | Negative predictive value |
| OC | 1.0 | 0.72449 | 1.0 | 0.86364 | 0.88811 | 0.47619 | 1.0 | 1.0 | 1.0 | 1.0 | 1.0 | 0.7037 | 0.0 | 0.25532 | 0.95652 | 0.95652 | 0.93333 | 0.12821 | 0.48 | 0.5 | 0.57143 | None | 1.0 | 0.9 | 0.5 | 0.95 | 0.0 | 0.30769 | 0.57143 | 0.83333 | 0.94118 | 0.83333 | None | 0.83333 | 0.92727 | 1.0 | 0.90909 | 1.0 | 1.0 | 1.0 | None | None | None | 0.84615 | None | None | 1.0 | 1.0 | 1.0 | None | 1.0 | 1.0 | Overlap coefficient |
| OOC | 0.97802 | 0.71721 | 0.84515 | 0.67514 | 0.73462 | 0.43644 | 1.0 | 1.0 | 0.66421 | 0.94972 | 0.70711 | 0.56422 | 0.0 | 0.17247 | 0.95652 | 0.88283 | 0.87671 | 0.11103 | 0.32812 | 0.31623 | 0.41931 | None | 0.75076 | 0.53785 | 0.17678 | 0.84971 | 0.0 | 0.20601 | 0.34684 | 0.72169 | 0.86772 | 0.28583 | None | 0.77152 | 0.88049 | 0.89443 | 0.80582 | 0.92582 | 0.44721 | 0.95346 | None | None | None | 0.81537 | None | None | 1.0 | 0.57735 | 0.75 | None | 0.65465 | 1.0 | Otsuka-Ochiai coefficient |
| OP | 0.97701 | 0.79833 | 0.96687 | 0.67657 | 0.90197 | 0.55493 | 1.0 | 1.0 | 0.5976 | 0.9446 | 0.66127 | 0.81106 | -0.01157 | 0.33468 | 0.97663 | 0.89372 | 0.9636 | 0.1726 | 0.59515 | 0.32969 | 0.45379 | -0.0054 | 0.94317 | 0.47136 | 0.1054 | 0.85863 | -0.01157 | 0.21773 | 0.71882 | 0.76651 | 0.88542 | 0.14246 | -0.01157 | 0.8314 | 0.95551 | 0.88735 | 0.95008 | 0.92231 | 0.33025 | 0.99884 | -0.00386 | -0.00463 | -0.0054 | 0.87691 | -0.03161 | -0.00463 | 1.0 | 0.49692 | 0.7146 | -0.00771 | 0.59692 | 1.0 | Optimized precision |
| P | 23 | 100 | 70 | 36 | 143 | 25 | 6 | 11 | 34 | 51 | 14 | 27 | 8 | 47 | 23 | 27 | 15 | 39 | 50 | 5 | 26 | 7 | 62 | 28 | 16 | 25 | 13 | 29 | 7 | 8 | 20 | 51 | 15 | 7 | 55 | 10 | 11 | 7 | 5 | 10 | 5 | 6 | 7 | 14 | 41 | 6 | 8 | 6 | 16 | 10 | 7 | 5 | Condition positive or support |
| PLR | None | 31.47667 | 43.82143 | 221.84259 | 12.49855 | 46.25455 | None | None | None | None | None | 38.85668 | 0.0 | 3.50713 | 1218.6087 | 1034.81481 | 398.84444 | 3.43153 | 7.21157 | 258.4 | 65.17949 | None | 25.72917 | 407.89286 | 80.0625 | 966.72 | 0.0 | 19.43295 | 49.14286 | 805.625 | 1021.6 | 122.15686 | None | 921.42857 | 115.16727 | None | 292.27273 | None | None | 1287.0 | None | None | None | 504.03571 | None | None | None | None | None | None | None | None | Positive likelihood ratio |
| PLRI | None | Good | Good | Good | Good | Good | None | None | None | None | None | Good | Negligible | Poor | Good | Good | Good | Poor | Fair | Good | Good | None | Good | Good | Good | Good | Negligible | Good | Good | Good | Good | Good | None | Good | Good | None | Good | None | None | Good | None | None | None | Good | None | None | None | None | None | None | None | None | Positive likelihood ratio interpretation |
| POP | 1297 | 1297 | 1297 | 1297 | 1297 | 1297 | 1297 | 1297 | 1297 | 1297 | 1297 | 1297 | 1297 | 1297 | 1297 | 1297 | 1297 | 1297 | 1297 | 1297 | 1297 | 1297 | 1297 | 1297 | 1297 | 1297 | 1297 | 1297 | 1297 | 1297 | 1297 | 1297 | 1297 | 1297 | 1297 | 1297 | 1297 | 1297 | 1297 | 1297 | 1297 | 1297 | 1297 | 1297 | 1297 | 1297 | 1297 | 1297 | 1297 | 1297 | 1297 | 1297 | Population |
| PPV | 1.0 | 0.72449 | 0.71429 | 0.86364 | 0.60766 | 0.47619 | 1.0 | 1.0 | 1.0 | 1.0 | 1.0 | 0.45238 | 0.0 | 0.1165 | 0.95652 | 0.95652 | 0.82353 | 0.09615 | 0.2243 | 0.5 | 0.57143 | None | 0.56364 | 0.9 | 0.5 | 0.95 | 0.0 | 0.30769 | 0.21053 | 0.83333 | 0.94118 | 0.83333 | None | 0.83333 | 0.83607 | 1.0 | 0.71429 | 1.0 | 1.0 | 0.90909 | None | None | None | 0.84615 | None | None | 1.0 | 1.0 | 1.0 | None | 1.0 | 1.0 | Precision or positive predictive value |
| PRE | 0.01773 | 0.0771 | 0.05397 | 0.02776 | 0.11025 | 0.01928 | 0.00463 | 0.00848 | 0.02621 | 0.03932 | 0.01079 | 0.02082 | 0.00617 | 0.03624 | 0.01773 | 0.02082 | 0.01157 | 0.03007 | 0.03855 | 0.00386 | 0.02005 | 0.0054 | 0.0478 | 0.02159 | 0.01234 | 0.01928 | 0.01002 | 0.02236 | 0.0054 | 0.00617 | 0.01542 | 0.03932 | 0.01157 | 0.0054 | 0.04241 | 0.00771 | 0.00848 | 0.0054 | 0.00386 | 0.00771 | 0.00386 | 0.00463 | 0.0054 | 0.01079 | 0.03161 | 0.00463 | 0.00617 | 0.00463 | 0.01234 | 0.00771 | 0.0054 | 0.00386 | Prevalence |
| Q | None | 0.98132 | None | 0.99574 | 0.98091 | 0.97417 | None | None | None | None | None | 0.98459 | -1.0 | 0.62733 | 0.99993 | 0.99964 | 0.99966 | 0.58239 | 0.85658 | 0.99382 | 0.97888 | None | None | 0.99668 | 0.97683 | 0.9995 | -1.0 | 0.91446 | 0.98251 | 0.99907 | 0.99961 | 0.98533 | None | 0.99938 | 0.99873 | None | 0.99938 | None | None | None | None | None | None | 0.99915 | None | None | None | None | None | None | None | None | Yule Q - coefficient of colligation |
| QI | None | Strong | None | Strong | Strong | Strong | None | None | None | None | None | Strong | Negligible | Moderate | Strong | Strong | Strong | Moderate | Strong | Strong | Strong | None | None | Strong | Strong | Strong | Negligible | Strong | Strong | Strong | Strong | Strong | None | Strong | Strong | None | Strong | None | None | None | None | None | None | Strong | None | None | None | None | None | None | None | None | Yule Q interpretation |
| RACC | 0.0003 | 0.00583 | 0.00408 | 0.00047 | 0.01777 | 0.00031 | 2e-05 | 7e-05 | 0.0003 | 0.00139 | 6e-05 | 0.00067 | 3e-05 | 0.00288 | 0.00031 | 0.00037 | 0.00015 | 0.00121 | 0.00318 | 1e-05 | 0.00022 | 0.0 | 0.00405 | 0.00017 | 2e-05 | 0.0003 | 2e-05 | 0.00022 | 8e-05 | 3e-05 | 0.0002 | 0.00018 | 0.0 | 2e-05 | 0.00199 | 5e-05 | 9e-05 | 2e-05 | 0.0 | 7e-05 | 0.0 | 0.0 | 0.0 | 0.00011 | 0.0 | 0.0 | 4e-05 | 1e-05 | 9e-05 | 0.0 | 1e-05 | 1e-05 | Random accuracy |
| RACCU | 0.0003 | 0.00583 | 0.00419 | 0.0005 | 0.01841 | 0.00031 | 2e-05 | 7e-05 | 0.00036 | 0.0014 | 7e-05 | 0.00071 | 3e-05 | 0.00334 | 0.00031 | 0.00037 | 0.00015 | 0.00123 | 0.00366 | 1e-05 | 0.00024 | 1e-05 | 0.0044 | 0.00021 | 5e-05 | 0.0003 | 3e-05 | 0.00026 | 0.0001 | 3e-05 | 0.0002 | 0.00048 | 3e-05 | 3e-05 | 0.002 | 5e-05 | 9e-05 | 3e-05 | 1e-05 | 7e-05 | 0.0 | 1e-05 | 1e-05 | 0.00011 | 0.00025 | 1e-05 | 4e-05 | 1e-05 | 9e-05 | 1e-05 | 1e-05 | 1e-05 | Random accuracy unbiased |
| TN | 1274 | 1170 | 1199 | 1258 | 1072 | 1261 | 1291 | 1286 | 1263 | 1246 | 1283 | 1247 | 1282 | 1159 | 1273 | 1269 | 1279 | 1211 | 1164 | 1291 | 1265 | 1290 | 1187 | 1268 | 1280 | 1271 | 1282 | 1259 | 1275 | 1288 | 1276 | 1245 | 1282 | 1289 | 1232 | 1287 | 1282 | 1290 | 1292 | 1286 | 1292 | 1291 | 1290 | 1281 | 1256 | 1291 | 1289 | 1291 | 1281 | 1287 | 1290 | 1292 | True negative/correct rejection |
| TNR | 1.0 | 0.97744 | 0.97718 | 0.99762 | 0.92894 | 0.99135 | 1.0 | 1.0 | 1.0 | 1.0 | 1.0 | 0.98189 | 0.99457 | 0.9272 | 0.99922 | 0.99921 | 0.99766 | 0.96264 | 0.93344 | 0.99923 | 0.99528 | 1.0 | 0.96113 | 0.99921 | 0.99922 | 0.99921 | 0.99844 | 0.9929 | 0.98837 | 0.99922 | 0.99922 | 0.9992 | 1.0 | 0.99922 | 0.99195 | 1.0 | 0.99689 | 1.0 | 1.0 | 0.99922 | 1.0 | 1.0 | 1.0 | 0.99844 | 1.0 | 1.0 | 1.0 | 1.0 | 1.0 | 1.0 | 1.0 | 1.0 | Specificity or true negative rate |
| TON | 1275 | 1199 | 1199 | 1275 | 1088 | 1276 | 1291 | 1286 | 1282 | 1251 | 1290 | 1255 | 1290 | 1194 | 1274 | 1274 | 1280 | 1245 | 1190 | 1295 | 1283 | 1297 | 1187 | 1287 | 1295 | 1277 | 1295 | 1284 | 1278 | 1291 | 1280 | 1291 | 1297 | 1291 | 1236 | 1289 | 1283 | 1291 | 1296 | 1286 | 1297 | 1297 | 1297 | 1284 | 1297 | 1297 | 1289 | 1295 | 1288 | 1297 | 1294 | 1292 | Test outcome negative |
| TOP | 22 | 98 | 98 | 22 | 209 | 21 | 6 | 11 | 15 | 46 | 7 | 42 | 7 | 103 | 23 | 23 | 17 | 52 | 107 | 2 | 14 | 0 | 110 | 10 | 2 | 20 | 2 | 13 | 19 | 6 | 17 | 6 | 0 | 6 | 61 | 8 | 14 | 6 | 1 | 11 | 0 | 0 | 0 | 13 | 0 | 0 | 8 | 2 | 9 | 0 | 3 | 5 | Test outcome positive |
| TP | 22 | 71 | 70 | 19 | 127 | 10 | 6 | 11 | 15 | 46 | 7 | 19 | 0 | 12 | 22 | 22 | 14 | 5 | 24 | 1 | 8 | 0 | 62 | 9 | 1 | 19 | 0 | 4 | 4 | 5 | 16 | 5 | 0 | 5 | 51 | 8 | 10 | 6 | 1 | 10 | 0 | 0 | 0 | 11 | 0 | 0 | 8 | 2 | 9 | 0 | 3 | 5 | True positive/hit |
| TPR | 0.95652 | 0.71 | 1.0 | 0.52778 | 0.88811 | 0.4 | 1.0 | 1.0 | 0.44118 | 0.90196 | 0.5 | 0.7037 | 0.0 | 0.25532 | 0.95652 | 0.81481 | 0.93333 | 0.12821 | 0.48 | 0.2 | 0.30769 | 0.0 | 1.0 | 0.32143 | 0.0625 | 0.76 | 0.0 | 0.13793 | 0.57143 | 0.625 | 0.8 | 0.09804 | 0.0 | 0.71429 | 0.92727 | 0.8 | 0.90909 | 0.85714 | 0.2 | 1.0 | 0.0 | 0.0 | 0.0 | 0.78571 | 0.0 | 0.0 | 1.0 | 0.33333 | 0.5625 | 0.0 | 0.42857 | 1.0 | Sensitivity, recall, hit rate, or true positive rate |
| Y | 0.95652 | 0.68744 | 0.97718 | 0.5254 | 0.81705 | 0.39135 | 1.0 | 1.0 | 0.44118 | 0.90196 | 0.5 | 0.68559 | -0.00543 | 0.18252 | 0.95574 | 0.81403 | 0.93099 | 0.09084 | 0.41344 | 0.19923 | 0.30297 | 0.0 | 0.96113 | 0.32064 | 0.06172 | 0.75921 | -0.00156 | 0.13083 | 0.5598 | 0.62422 | 0.79922 | 0.09724 | 0.0 | 0.71351 | 0.91922 | 0.8 | 0.90598 | 0.85714 | 0.2 | 0.99922 | 0.0 | 0.0 | 0.0 | 0.78416 | 0.0 | 0.0 | 1.0 | 0.33333 | 0.5625 | 0.0 | 0.42857 | 1.0 | Youden index |
| dInd | 0.04348 | 0.29088 | 0.02282 | 0.47223 | 0.13254 | 0.60006 | 0.0 | 0.0 | 0.55882 | 0.09804 | 0.5 | 0.29685 | 1.00001 | 0.74823 | 0.04349 | 0.18519 | 0.06671 | 0.8726 | 0.52424 | 0.8 | 0.69232 | 1.0 | 0.03887 | 0.67857 | 0.9375 | 0.24 | 1.0 | 0.8621 | 0.42873 | 0.375 | 0.2 | 0.90196 | 1.0 | 0.28572 | 0.07317 | 0.2 | 0.09096 | 0.14286 | 0.8 | 0.00078 | 1.0 | 1.0 | 1.0 | 0.21429 | 1.0 | 1.0 | 0.0 | 0.66667 | 0.4375 | 1.0 | 0.57143 | 0.0 | Distance index |
| sInd | 0.96926 | 0.79432 | 0.98386 | 0.66608 | 0.90628 | 0.57569 | 1.0 | 1.0 | 0.60485 | 0.93068 | 0.64645 | 0.7901 | 0.29288 | 0.47092 | 0.96925 | 0.86905 | 0.95283 | 0.38298 | 0.6293 | 0.43431 | 0.51045 | 0.29289 | 0.97252 | 0.52018 | 0.33709 | 0.83029 | 0.29289 | 0.3904 | 0.69684 | 0.73483 | 0.85858 | 0.36222 | 0.29289 | 0.79797 | 0.94826 | 0.85858 | 0.93568 | 0.89898 | 0.43431 | 0.99945 | 0.29289 | 0.29289 | 0.29289 | 0.84847 | 0.29289 | 0.29289 | 1.0 | 0.5286 | 0.69064 | 0.29289 | 0.59594 | 1.0 | Similarity index |

Generated By PyCM Version 3.1
