# Supplementary material for: TopEC: prediction of Enzyme Commission classes by 3D graph neural networks and localized 3D protein descriptor
Source: Nat Commun. 2025 Mar 20;16:2737. doi: 10.1038/s41467-025-57324-5 (PMC11923149; doi:10.1038/s41467-025-57324-5)
Supplement: Supplementary file 3 — Supplementary Data 1 [file 41467_2025_57324_MOESM3_ESM.zip › Data_S1/table1/hierarchical/DeepFRI_TEMP.html]

DeepFRI\_HIER


# PyCM Report

## Dataset Type :

- Multi-Class Classification
- Imbalanced

Note 1 : Recommended statistics for this type of classification highlighted in aqua

Note 2 : The recommender system assumes that the input is the result of classification over the whole data rather than just a part of it.
If the confusion matrix is the result of test data classification, the recommendation is not valid.

## Confusion Matrix :

|  |  |  |  |  |  |  |  |  |  |  |  |  |  |  |  |  |  |  |  |  |  |  |  |  |  |  |  |  |  |  |  |  |  |  |  |  |  |  |  |  |  |  |  |  |  |  |  |  |  |  |  |  |  |  |  |  |  |  |  |  |  |  |  |  |  |  |  |  |  |  |  |  |  |  |  |  |  |  |  |  |  |  |  |  |  |  |  |  |  |  |  |  |  |  |  |  |  |  |  |  |  |  |  |  |  |  |  |  |  |  |  |  |  |  |  |  |  |  |  |  |  |  |  |  |  |  |  |  |  |  |  |  |  |  |  |  |  |  |  |  |  |  |  |  |  |  |  |  |  |  |  |  |  |  |  |  |  |  |  |  |  |  |  |  |  |  |  |  |  |  |  |  |  |  |  |  |  |  |  |  |  |  |  |  |  |  |  |  |  |  |  |  |  |  |  |  |  |  |  |  |  |  |  |  |  |  |  |  |  |  |  |  |  |  |  |  |  |  |  |  |  |  |  |  |  |  |  |  |  |  |  |  |  |  |  |  |  |  |  |  |  |  |  |  |  |  |  |  |  |  |  |  |  |  |  |  |  |  |  |  |  |  |  |  |  |  |  |  |  |  |  |  |  |  |  |  |  |  |  |  |  |  |  |  |  |  |  |  |  |  |  |  |  |  |  |  |  |  |  |  |  |  |  |  |  |  |  |  |  |  |  |  |  |  |  |  |  |  |  |  |  |  |  |  |  |  |  |  |  |  |  |  |  |  |  |  |  |  |  |  |  |  |  |  |  |  |  |  |  |  |  |  |  |  |  |  |  |  |  |  |  |  |  |  |  |  |  |  |  |  |  |  |  |  |  |  |  |  |  |  |  |  |  |  |  |  |  |  |  |  |  |  |  |  |  |  |  |  |  |  |  |  |  |  |  |  |  |  |  |  |  |  |  |  |  |  |  |  |  |  |  |  |  |  |  |  |  |  |  |  |  |  |  |  |  |  |  |  |  |  |  |  |  |  |  |  |  |  |  |  |  |  |  |  |  |  |  |  |  |  |  |  |  |  |  |  |  |  |  |  |  |  |  |  |  |  |  |  |  |  |  |  |  |  |  |  |  |  |  |  |  |  |  |  |  |  |  |  |  |  |  |  |  |  |  |  |  |  |  |  |  |  |  |  |  |  |  |  |  |  |  |  |  |  |  |  |  |  |  |  |  |  |  |  |  |  |  |  |  |  |  |  |  |  |  |  |  |  |  |  |  |  |  |  |  |  |  |  |  |  |  |  |  |  |  |  |  |  |  |  |  |  |  |  |  |  |  |  |  |  |  |  |  |  |  |  |  |  |  |  |  |  |  |  |  |  |  |  |  |  |  |  |  |  |  |  |  |  |  |  |  |  |  |  |  |  |  |  |  |  |  |  |  |  |  |  |  |  |  |  |  |  |  |  |  |  |  |  |  |  |  |  |  |  |  |  |  |  |  |  |  |  |  |  |  |  |  |  |  |  |  |  |  |  |  |  |  |  |  |  |  |  |  |  |  |  |  |  |  |  |  |  |  |  |  |  |  |  |  |  |  |  |  |  |  |  |  |  |  |  |  |  |  |  |  |  |  |  |  |  |  |  |  |  |  |  |  |  |  |  |  |  |  |  |  |  |  |  |  |  |  |  |  |  |  |  |  |  |  |  |  |  |  |  |  |  |  |  |  |  |  |  |  |  |  |  |  |  |  |  |  |  |  |  |  |  |  |  |  |  |  |  |  |  |  |  |  |  |  |  |  |  |  |  |  |  |  |  |  |  |  |  |  |  |  |  |  |  |  |  |  |  |  |  |  |  |  |  |  |  |  |  |  |  |  |  |  |  |  |  |  |  |  |  |  |  |  |  |  |  |  |  |  |  |  |  |  |  |  |  |  |  |  |  |  |  |  |  |  |  |  |  |  |  |  |  |  |  |  |  |  |  |  |  |  |  |  |  |  |  |  |  |  |  |  |  |  |  |  |  |  |  |  |  |  |  |  |  |  |  |  |  |  |  |  |  |  |  |  |  |  |  |  |  |  |  |  |  |  |  |  |  |  |  |  |  |  |  |  |  |  |  |  |  |  |  |  |  |  |  |  |  |  |  |  |  |  |  |  |  |  |  |  |  |  |  |  |  |  |  |  |  |  |  |  |  |  |  |  |  |  |  |  |  |  |  |  |  |  |  |  |  |  |  |  |  |  |  |  |  |  |  |  |  |  |  |  |  |  |  |  |  |  |  |  |  |  |  |  |  |  |  |  |  |  |  |  |  |  |  |  |  |  |  |  |  |  |  |  |  |  |  |  |  |  |  |  |  |  |  |  |  |  |  |  |  |  |  |  |  |  |  |  |  |  |  |  |  |  |  |  |  |  |  |  |  |  |  |  |  |  |  |  |  |  |  |  |  |  |  |  |  |  |  |  |  |  |  |  |  |  |  |  |  |  |  |  |  |  |  |  |  |  |  |  |  |  |  |  |  |  |  |  |  |  |  |  |  |  |  |  |  |  |  |  |  |  |  |  |  |  |  |  |  |  |  |  |  |  |  |  |  |  |  |  |  |  |  |  |  |  |  |  |  |  |  |  |  |  |  |  |  |  |  |  |  |  |  |  |  |  |  |  |  |  |  |  |  |  |  |  |  |  |  |  |  |  |  |  |  |  |  |  |  |  |  |  |  |  |  |  |  |  |  |  |  |  |  |  |  |  |  |  |  |  |  |  |  |  |  |  |  |  |  |  |  |  |  |  |  |  |  |  |  |  |  |  |  |  |  |  |  |  |  |  |  |  |  |  |  |  |  |  |  |  |  |  |  |  |  |  |  |  |  |  |  |  |  |  |  |  |  |  |  |  |  |  |  |  |  |  |  |  |  |  |  |  |  |  |  |  |  |  |  |  |  |  |  |  |  |  |  |  |  |  |  |  |  |  |  |  |  |  |  |  |  |  |  |  |  |  |  |  |  |  |  |  |  |  |  |  |  |  |  |  |  |  |  |  |  |  |  |  |  |  |  |  |  |  |  |  |  |  |  |  |  |  |  |  |  |  |  |  |  |  |  |  |  |  |  |  |  |  |  |  |  |  |  |  |  |  |  |  |  |  |  |  |  |  |  |  |  |  |  |  |  |  |  |  |  |  |  |  |  |  |  |  |  |  |  |  |  |  |  |  |  |  |  |  |  |  |  |  |  |  |  |  |  |  |  |  |  |  |  |  |  |  |  |  |  |  |  |  |  |  |  |  |  |  |  |  |  |  |  |  |  |  |  |  |  |  |  |  |  |  |  |  |  |  |  |  |  |  |  |  |  |  |  |  |  |  |  |  |  |  |  |  |  |  |  |  |  |  |  |  |  |  |  |  |  |  |  |  |  |  |  |  |  |  |  |  |  |  |  |  |  |  |  |  |  |  |  |  |  |  |  |  |  |  |  |  |  |  |  |  |  |  |  |  |  |  |  |  |  |  |  |  |  |  |  |  |  |  |  |  |  |  |  |  |  |  |  |  |  |  |  |  |  |  |  |  |  |  |  |  |  |  |  |  |  |  |  |  |  |  |  |  |  |  |  |  |  |  |  |  |  |  |  |  |  |  |  |  |  |  |  |  |  |  |  |  |  |  |  |  |  |  |  |  |  |  |  |  |  |  |  |  |  |  |  |  |  |  |  |  |  |  |  |  |  |  |  |  |  |  |  |  |  |  |  |  |  |  |  |  |  |  |  |  |  |  |  |  |  |  |  |  |  |  |  |  |  |  |  |  |  |  |  |  |  |  |  |  |  |  |  |  |  |  |  |  |  |  |  |  |  |  |  |  |  |  |  |  |  |  |  |  |  |  |  |  |  |  |  |  |  |  |  |  |  |  |  |  |  |  |  |  |  |  |  |  |  |  |  |  |  |  |  |  |  |  |  |  |  |  |  |  |  |  |  |  |  |  |  |  |  |  |  |  |  |  |  |  |  |  |  |  |  |  |  |  |  |  |  |  |  |  |  |  |  |  |  |  |  |  |  |  |  |  |  |  |  |  |  |  |  |  |  |  |  |  |  |  |  |  |  |  |  |  |  |  |  |  |  |  |  |  |  |  |  |  |  |  |  |  |  |  |  |  |  |  |  |  |  |  |  |  |  |  |  |  |  |  |  |  |  |  |  |  |  |  |  |  |  |  |  |  |  |  |  |  |  |  |  |  |  |  |  |  |  |  |  |  |  |  |  |  |  |  |  |  |  |  |  |  |  |  |  |  |  |  |  |  |  |  |  |  |  |  |  |  |  |  |  |  |  |  |  |  |  |  |  |  |  |  |  |  |  |  |  |  |  |  |  |  |  |  |  |  |  |  |  |  |  |  |  |  |  |  |  |  |  |  |  |  |  |  |  |  |  |  |  |  |  |  |  |  |  |  |  |  |  |  |  |  |  |  |  |  |  |  |  |  |  |  |  |  |  |  |  |  |  |  |  |  |  |  |  |  |  |  |  |  |  |  |  |  |  |  |  |  |  |  |  |  |  |  |  |  |  |  |  |  |  |  |  |  |  |  |  |  |  |  |  |  |  |  |  |  |  |  |  |  |  |  |  |  |  |  |  |  |  |  |  |  |  |  |  |  |  |  |  |  |  |  |  |  |  |  |  |  |  |  |  |  |  |  |  |  |  |  |  |  |  |  |  |  |  |  |  |  |  |  |  |  |  |  |  |  |  |  |  |  |  |  |  |  |  |  |  |  |  |  |  |  |  |  |  |  |  |  |  |  |  |  |  |  |  |  |  |  |  |  |  |  |  |  |  |  |  |  |  |  |  |  |  |  |  |  |  |  |  |  |  |  |  |  |  |  |  |  |  |  |  |  |  |  |  |  |  |  |  |  |  |  |  |  |  |  |  |  |  |  |  |  |  |  |  |  |  |  |  |  |  |  |  |  |  |  |  |  |  |  |  |  |  |  |  |  |  |  |  |  |  |  |  |  |  |  |  |  |  |  |  |  |  |  |  |  |  |  |  |  |  |  |  |  |  |  |  |  |  |  |  |  |  |  |  |  |  |  |  |  |  |  |  |  |  |  |  |  |  |  |  |  |  |  |  |  |  |  |  |  |  |  |  |  |  |  |  |  |  |  |  |  |  |  |  |  |  |  |  |  |  |  |  |  |  |  |  |  |  |  |  |  |  |  |  |  |  |  |  |  |  |  |  |  |  |  |  |  |  |  |  |  |  |  |  |  |  |  |  |  |  |  |  |  |  |  |  |  |  |  |  |  |  |  |  |  |  |  |  |  |  |  |  |  |  |  |  |  |  |  |  |  |  |  |  |  |  |  |  |  |  |  |  |  |  |  |  |  |  |  |  |  |  |  |  |  |  |  |  |  |  |  |  |  |  |  |  |  |  |  |  |  |  |  |  |  |  |  |  |  |  |  |  |  |  |  |  |  |  |  |  |  |  |  |  |  |  |  |  |  |  |  |  |  |  |  |  |  |  |  |  |  |  |  |  |  |  |  |  |  |  |  |  |  |  |  |  |  |  |  |  |  |  |  |  |  |  |  |  |  |  |  |  |  |  |  |  |  |  |  |  |  |  |  |  |  |  |  |  |  |  |  |  |  |  |  |  |  |  |  |  |  |  |  |  |  |  |  |  |  |  |  |  |  |  |  |  |  |  |  |  |  |  |  |  |  |  |  |  |  |  |  |  |  |  |  |  |  |  |  |  |  |  |  |  |  |  |  |  |  |  |  |  |  |  |  |  |  |  |  |  |  |  |  |  |  |  |  |  |  |  |  |  |  |  |  |  |  |  |  |  |  |  |  |  |  |  |  |  |  |  |  |  |  |  |  |  |  |  |  |  |  |  |  |  |  |  |  |  |  |  |  |  |  |  |  |  |  |  |  |  |  |  |  |  |  |  |  |  |  |  |  |  |  |  |  |  |  |  |  |  |  |  |  |  |  |  |  |  |  |  |  |  |  |  |  |  |  |  |  |  |  |  |  |  |  |  |  |  |  |  |  |  |  |  |  |  |  |  |  |  |  |  |  |  |  |  |  |  |  |  |  |  |  |  |  |  |  |  |  |  |  |  |  |  |  |  |  |  |  |  |  |  |  |  |  |  |  |  |  |  |  |  |  |  |  |  |  |  |  |  |  |  |  |  |  |  |  |  |  |  |  |  |  |  |  |  |  |  |  |  |  |  |  |  |  |  |  |  |  |  |  |  |  |  |  |  |  |  |  |  |  |  |  |  |  |  |  |  |  |  |  |  |  |  |  |  |  |  |  |  |  |  |  |  |  |  |  |  |  |  |  |  |  |  |  |  |  |  |  |  |  |  |  |  |  |  |  |  |  |  |  |  |  |  |  |  |
| --- | --- | --- | --- | --- | --- | --- | --- | --- | --- | --- | --- | --- | --- | --- | --- | --- | --- | --- | --- | --- | --- | --- | --- | --- | --- | --- | --- | --- | --- | --- | --- | --- | --- | --- | --- | --- | --- | --- | --- | --- | --- | --- | --- | --- | --- | --- | --- | --- | --- | --- | --- | --- | --- | --- | --- | --- | --- | --- | --- | --- | --- | --- | --- | --- | --- | --- | --- | --- | --- | --- | --- | --- | --- | --- | --- | --- | --- | --- | --- | --- | --- | --- | --- | --- | --- | --- | --- | --- | --- | --- | --- | --- | --- | --- | --- | --- | --- | --- | --- | --- | --- | --- | --- | --- | --- | --- | --- | --- | --- | --- | --- | --- | --- | --- | --- | --- | --- | --- | --- | --- | --- | --- | --- | --- | --- | --- | --- | --- | --- | --- | --- | --- | --- | --- | --- | --- | --- | --- | --- | --- | --- | --- | --- | --- | --- | --- | --- | --- | --- | --- | --- | --- | --- | --- | --- | --- | --- | --- | --- | --- | --- | --- | --- | --- | --- | --- | --- | --- | --- | --- | --- | --- | --- | --- | --- | --- | --- | --- | --- | --- | --- | --- | --- | --- | --- | --- | --- | --- | --- | --- | --- | --- | --- | --- | --- | --- | --- | --- | --- | --- | --- | --- | --- | --- | --- | --- | --- | --- | --- | --- | --- | --- | --- | --- | --- | --- | --- | --- | --- | --- | --- | --- | --- | --- | --- | --- | --- | --- | --- | --- | --- | --- | --- | --- | --- | --- | --- | --- | --- | --- | --- | --- | --- | --- | --- | --- | --- | --- | --- | --- | --- | --- | --- | --- | --- | --- | --- | --- | --- | --- | --- | --- | --- | --- | --- | --- | --- | --- | --- | --- | --- | --- | --- | --- | --- | --- | --- | --- | --- | --- | --- | --- | --- | --- | --- | --- | --- | --- | --- | --- | --- | --- | --- | --- | --- | --- | --- | --- | --- | --- | --- | --- | --- | --- | --- | --- | --- | --- | --- | --- | --- | --- | --- | --- | --- | --- | --- | --- | --- | --- | --- | --- | --- | --- | --- | --- | --- | --- | --- | --- | --- | --- | --- | --- | --- | --- | --- | --- | --- | --- | --- | --- | --- | --- | --- | --- | --- | --- | --- | --- | --- | --- | --- | --- | --- | --- | --- | --- | --- | --- | --- | --- | --- | --- | --- | --- | --- | --- | --- | --- | --- | --- | --- | --- | --- | --- | --- | --- | --- | --- | --- | --- | --- | --- | --- | --- | --- | --- | --- | --- | --- | --- | --- | --- | --- | --- | --- | --- | --- | --- | --- | --- | --- | --- | --- | --- | --- | --- | --- | --- | --- | --- | --- | --- | --- | --- | --- | --- | --- | --- | --- | --- | --- | --- | --- | --- | --- | --- | --- | --- | --- | --- | --- | --- | --- | --- | --- | --- | --- | --- | --- | --- | --- | --- | --- | --- | --- | --- | --- | --- | --- | --- | --- | --- | --- | --- | --- | --- | --- | --- | --- | --- | --- | --- | --- | --- | --- | --- | --- | --- | --- | --- | --- | --- | --- | --- | --- | --- | --- | --- | --- | --- | --- | --- | --- | --- | --- | --- | --- | --- | --- | --- | --- | --- | --- | --- | --- | --- | --- | --- | --- | --- | --- | --- | --- | --- | --- | --- | --- | --- | --- | --- | --- | --- | --- | --- | --- | --- | --- | --- | --- | --- | --- | --- | --- | --- | --- | --- | --- | --- | --- | --- | --- | --- | --- | --- | --- | --- | --- | --- | --- | --- | --- | --- | --- | --- | --- | --- | --- | --- | --- | --- | --- | --- | --- | --- | --- | --- | --- | --- | --- | --- | --- | --- | --- | --- | --- | --- | --- | --- | --- | --- | --- | --- | --- | --- | --- | --- | --- | --- | --- | --- | --- | --- | --- | --- | --- | --- | --- | --- | --- | --- | --- | --- | --- | --- | --- | --- | --- | --- | --- | --- | --- | --- | --- | --- | --- | --- | --- | --- | --- | --- | --- | --- | --- | --- | --- | --- | --- | --- | --- | --- | --- | --- | --- | --- | --- | --- | --- | --- | --- | --- | --- | --- | --- | --- | --- | --- | --- | --- | --- | --- | --- | --- | --- | --- | --- | --- | --- | --- | --- | --- | --- | --- | --- | --- | --- | --- | --- | --- | --- | --- | --- | --- | --- | --- | --- | --- | --- | --- | --- | --- | --- | --- | --- | --- | --- | --- | --- | --- | --- | --- | --- | --- | --- | --- | --- | --- | --- | --- | --- | --- | --- | --- | --- | --- | --- | --- | --- | --- | --- | --- | --- | --- | --- | --- | --- | --- | --- | --- | --- | --- | --- | --- | --- | --- | --- | --- | --- | --- | --- | --- | --- | --- | --- | --- | --- | --- | --- | --- | --- | --- | --- | --- | --- | --- | --- | --- | --- | --- | --- | --- | --- | --- | --- | --- | --- | --- | --- | --- | --- | --- | --- | --- | --- | --- | --- | --- | --- | --- | --- | --- | --- | --- | --- | --- | --- | --- | --- | --- | --- | --- | --- | --- | --- | --- | --- | --- | --- | --- | --- | --- | --- | --- | --- | --- | --- | --- | --- | --- | --- | --- | --- | --- | --- | --- | --- | --- | --- | --- | --- | --- | --- | --- | --- | --- | --- | --- | --- | --- | --- | --- | --- | --- | --- | --- | --- | --- | --- | --- | --- | --- | --- | --- | --- | --- | --- | --- | --- | --- | --- | --- | --- | --- | --- | --- | --- | --- | --- | --- | --- | --- | --- | --- | --- | --- | --- | --- | --- | --- | --- | --- | --- | --- | --- | --- | --- | --- | --- | --- | --- | --- | --- | --- | --- | --- | --- | --- | --- | --- | --- | --- | --- | --- | --- | --- | --- | --- | --- | --- | --- | --- | --- | --- | --- | --- | --- | --- | --- | --- | --- | --- | --- | --- | --- | --- | --- | --- | --- | --- | --- | --- | --- | --- | --- | --- | --- | --- | --- | --- | --- | --- | --- | --- | --- | --- | --- | --- | --- | --- | --- | --- | --- | --- | --- | --- | --- | --- | --- | --- | --- | --- | --- | --- | --- | --- | --- | --- | --- | --- | --- | --- | --- | --- | --- | --- | --- | --- | --- | --- | --- | --- | --- | --- | --- | --- | --- | --- | --- | --- | --- | --- | --- | --- | --- | --- | --- | --- | --- | --- | --- | --- | --- | --- | --- | --- | --- | --- | --- | --- | --- | --- | --- | --- | --- | --- | --- | --- | --- | --- | --- | --- | --- | --- | --- | --- | --- | --- | --- | --- | --- | --- | --- | --- | --- | --- | --- | --- | --- | --- | --- | --- | --- | --- | --- | --- | --- | --- | --- | --- | --- | --- | --- | --- | --- | --- | --- | --- | --- | --- | --- | --- | --- | --- | --- | --- | --- | --- | --- | --- | --- | --- | --- | --- | --- | --- | --- | --- | --- | --- | --- | --- | --- | --- | --- | --- | --- | --- | --- | --- | --- | --- | --- | --- | --- | --- | --- | --- | --- | --- | --- | --- | --- | --- | --- | --- | --- | --- | --- | --- | --- | --- | --- | --- | --- | --- | --- | --- | --- | --- | --- | --- | --- | --- | --- | --- | --- | --- | --- | --- | --- | --- | --- | --- | --- | --- | --- | --- | --- | --- | --- | --- | --- | --- | --- | --- | --- | --- | --- | --- | --- | --- | --- | --- | --- | --- | --- | --- | --- | --- | --- | --- | --- | --- | --- | --- | --- | --- | --- | --- | --- | --- | --- | --- | --- | --- | --- | --- | --- | --- | --- | --- | --- | --- | --- | --- | --- | --- | --- | --- | --- | --- | --- | --- | --- | --- | --- | --- | --- | --- | --- | --- | --- | --- | --- | --- | --- | --- | --- | --- | --- | --- | --- | --- | --- | --- | --- | --- | --- | --- | --- | --- | --- | --- | --- | --- | --- | --- | --- | --- | --- | --- | --- | --- | --- | --- | --- | --- | --- | --- | --- | --- | --- | --- | --- | --- | --- | --- | --- | --- | --- | --- | --- | --- | --- | --- | --- | --- | --- | --- | --- | --- | --- | --- | --- | --- | --- | --- | --- | --- | --- | --- | --- | --- | --- | --- | --- | --- | --- | --- | --- | --- | --- | --- | --- | --- | --- | --- | --- | --- | --- | --- | --- | --- | --- | --- | --- | --- | --- | --- | --- | --- | --- | --- | --- | --- | --- | --- | --- | --- | --- | --- | --- | --- | --- | --- | --- | --- | --- | --- | --- | --- | --- | --- | --- | --- | --- | --- | --- | --- | --- | --- | --- | --- | --- | --- | --- | --- | --- | --- | --- | --- | --- | --- | --- | --- | --- | --- | --- | --- | --- | --- | --- | --- | --- | --- | --- | --- | --- | --- | --- | --- | --- | --- | --- | --- | --- | --- | --- | --- | --- | --- | --- | --- | --- | --- | --- | --- | --- | --- | --- | --- | --- | --- | --- | --- | --- | --- | --- | --- | --- | --- | --- | --- | --- | --- | --- | --- | --- | --- | --- | --- | --- | --- | --- | --- | --- | --- | --- | --- | --- | --- | --- | --- | --- | --- | --- | --- | --- | --- | --- | --- | --- | --- | --- | --- | --- | --- | --- | --- | --- | --- | --- | --- | --- | --- | --- | --- | --- | --- | --- | --- | --- | --- | --- | --- | --- | --- | --- | --- | --- | --- | --- | --- | --- | --- | --- | --- | --- | --- | --- | --- | --- | --- | --- | --- | --- | --- | --- | --- | --- | --- | --- | --- | --- | --- | --- | --- | --- | --- | --- | --- | --- | --- | --- | --- | --- | --- | --- | --- | --- | --- | --- | --- | --- | --- | --- | --- | --- | --- | --- | --- | --- | --- | --- | --- | --- | --- | --- | --- | --- | --- | --- | --- | --- | --- | --- | --- | --- | --- | --- | --- | --- | --- | --- | --- | --- | --- | --- | --- | --- | --- | --- | --- | --- | --- | --- | --- | --- | --- | --- | --- | --- | --- | --- | --- | --- | --- | --- | --- | --- | --- | --- | --- | --- | --- | --- | --- | --- | --- | --- | --- | --- | --- | --- | --- | --- | --- | --- | --- | --- | --- | --- | --- | --- | --- | --- | --- | --- | --- | --- | --- | --- | --- | --- | --- | --- | --- | --- | --- | --- | --- | --- | --- | --- | --- | --- | --- | --- | --- | --- | --- | --- | --- | --- | --- | --- | --- | --- | --- | --- | --- | --- | --- | --- | --- | --- | --- | --- | --- | --- | --- | --- | --- | --- | --- | --- | --- | --- | --- | --- | --- | --- | --- | --- | --- | --- | --- | --- | --- | --- | --- | --- | --- | --- | --- | --- | --- | --- | --- | --- | --- | --- | --- | --- | --- | --- | --- | --- | --- | --- | --- | --- | --- | --- | --- | --- | --- | --- | --- | --- | --- | --- | --- | --- | --- | --- | --- | --- | --- | --- | --- | --- | --- | --- | --- | --- | --- | --- | --- | --- | --- | --- | --- | --- | --- | --- | --- | --- | --- | --- | --- | --- | --- | --- | --- | --- | --- | --- | --- | --- | --- | --- | --- | --- | --- | --- | --- | --- | --- | --- | --- | --- | --- | --- | --- | --- | --- | --- | --- | --- | --- | --- | --- | --- | --- | --- | --- | --- | --- | --- | --- | --- | --- | --- | --- | --- | --- | --- | --- | --- | --- | --- | --- | --- | --- | --- | --- | --- | --- | --- | --- | --- | --- | --- | --- | --- | --- | --- | --- | --- | --- | --- | --- | --- | --- | --- | --- | --- | --- | --- | --- | --- | --- | --- | --- | --- | --- | --- | --- | --- | --- | --- | --- | --- | --- | --- | --- | --- | --- | --- | --- | --- | --- | --- | --- | --- | --- | --- | --- | --- | --- | --- | --- | --- | --- | --- | --- | --- | --- | --- | --- | --- | --- | --- | --- | --- | --- | --- | --- | --- | --- | --- | --- | --- | --- | --- | --- | --- | --- | --- | --- | --- | --- | --- | --- | --- | --- | --- | --- | --- | --- | --- | --- | --- | --- | --- | --- | --- | --- | --- | --- | --- | --- | --- | --- | --- | --- | --- | --- | --- | --- | --- | --- | --- | --- | --- | --- | --- | --- | --- | --- | --- | --- | --- | --- | --- | --- | --- | --- | --- | --- | --- | --- | --- | --- | --- | --- | --- | --- | --- | --- | --- | --- | --- | --- | --- | --- | --- | --- | --- | --- | --- | --- | --- | --- | --- | --- | --- | --- | --- | --- | --- | --- | --- | --- | --- | --- | --- | --- | --- | --- | --- | --- | --- | --- | --- | --- | --- | --- | --- | --- | --- | --- | --- | --- | --- | --- | --- | --- | --- | --- | --- | --- | --- | --- | --- | --- | --- | --- | --- | --- | --- | --- | --- | --- | --- | --- | --- | --- | --- | --- | --- | --- | --- | --- | --- | --- | --- | --- | --- | --- | --- | --- | --- | --- | --- | --- | --- | --- | --- | --- | --- | --- | --- | --- | --- | --- | --- | --- | --- | --- | --- | --- | --- | --- | --- | --- | --- | --- | --- | --- | --- | --- | --- | --- | --- | --- | --- | --- | --- | --- | --- | --- | --- | --- | --- | --- | --- | --- | --- | --- | --- | --- | --- | --- | --- | --- | --- | --- | --- | --- | --- | --- | --- | --- | --- | --- | --- | --- | --- | --- | --- | --- | --- | --- | --- | --- | --- | --- | --- | --- | --- | --- | --- | --- | --- | --- | --- | --- | --- | --- | --- | --- | --- | --- | --- | --- | --- | --- | --- | --- | --- | --- | --- | --- | --- | --- | --- | --- | --- | --- | --- | --- | --- | --- | --- | --- | --- | --- | --- | --- | --- | --- | --- | --- | --- | --- | --- | --- | --- | --- | --- | --- | --- | --- | --- | --- | --- | --- | --- | --- | --- | --- | --- | --- | --- | --- | --- | --- | --- | --- | --- | --- | --- | --- | --- | --- | --- | --- | --- | --- | --- | --- | --- | --- | --- | --- | --- | --- | --- | --- | --- | --- | --- | --- | --- | --- | --- | --- | --- | --- | --- | --- | --- | --- | --- | --- | --- | --- | --- | --- | --- | --- | --- | --- | --- | --- | --- | --- | --- | --- | --- | --- | --- | --- | --- | --- | --- | --- | --- | --- | --- | --- | --- | --- | --- | --- | --- | --- | --- | --- | --- | --- | --- | --- | --- | --- | --- | --- | --- | --- | --- | --- | --- | --- | --- | --- | --- | --- | --- | --- | --- | --- | --- | --- | --- | --- | --- | --- | --- | --- | --- | --- | --- | --- | --- | --- | --- | --- | --- | --- | --- | --- | --- | --- | --- | --- | --- | --- | --- | --- | --- | --- | --- | --- | --- | --- | --- | --- | --- | --- | --- | --- | --- | --- | --- | --- | --- | --- | --- | --- | --- | --- | --- | --- | --- | --- | --- | --- | --- | --- | --- | --- | --- | --- | --- | --- | --- | --- | --- | --- | --- | --- | --- | --- | --- | --- | --- | --- | --- | --- | --- | --- | --- | --- | --- | --- | --- | --- | --- | --- | --- | --- | --- | --- | --- | --- | --- | --- | --- | --- | --- | --- | --- | --- | --- | --- | --- | --- | --- | --- | --- | --- | --- | --- | --- | --- | --- | --- | --- | --- | --- | --- | --- | --- | --- | --- | --- | --- | --- | --- | --- | --- | --- | --- | --- | --- | --- | --- | --- | --- | --- | --- | --- | --- | --- | --- | --- | --- | --- | --- | --- | --- | --- | --- | --- | --- | --- | --- | --- | --- | --- | --- | --- | --- | --- | --- | --- | --- | --- | --- | --- | --- | --- | --- | --- | --- | --- | --- | --- | --- | --- | --- | --- | --- | --- | --- | --- | --- | --- | --- | --- | --- | --- | --- | --- | --- | --- | --- | --- | --- | --- | --- | --- | --- | --- | --- | --- | --- | --- | --- | --- | --- | --- | --- | --- | --- | --- | --- | --- | --- | --- | --- | --- | --- | --- | --- | --- | --- | --- | --- | --- | --- | --- | --- | --- | --- | --- | --- | --- | --- | --- | --- | --- | --- | --- | --- | --- | --- | --- | --- | --- | --- | --- | --- | --- | --- | --- | --- | --- | --- | --- | --- | --- | --- | --- | --- | --- | --- | --- | --- | --- | --- | --- | --- | --- | --- | --- | --- | --- | --- | --- | --- | --- | --- | --- | --- | --- | --- | --- | --- | --- | --- | --- | --- | --- | --- | --- | --- | --- | --- | --- | --- | --- | --- | --- | --- | --- | --- | --- | --- | --- | --- | --- | --- | --- | --- | --- | --- | --- | --- | --- | --- | --- | --- | --- | --- | --- | --- | --- | --- | --- | --- | --- | --- | --- | --- | --- | --- | --- | --- | --- | --- | --- | --- | --- | --- | --- | --- | --- | --- | --- | --- | --- | --- | --- | --- | --- | --- | --- | --- | --- | --- | --- | --- | --- | --- | --- | --- | --- | --- | --- | --- | --- | --- | --- | --- | --- | --- | --- | --- | --- | --- | --- | --- | --- | --- | --- | --- | --- | --- | --- | --- | --- | --- | --- | --- | --- | --- | --- | --- | --- | --- | --- | --- | --- | --- | --- | --- | --- | --- | --- | --- | --- | --- | --- | --- | --- | --- | --- | --- | --- | --- | --- | --- | --- | --- | --- | --- | --- | --- | --- | --- | --- | --- | --- | --- | --- | --- | --- | --- | --- | --- | --- | --- | --- | --- | --- | --- | --- | --- | --- | --- | --- | --- | --- | --- | --- | --- | --- | --- | --- | --- | --- | --- | --- | --- | --- | --- | --- | --- | --- | --- | --- | --- | --- | --- | --- | --- | --- | --- | --- | --- | --- | --- | --- | --- | --- | --- | --- | --- | --- | --- | --- | --- | --- | --- | --- | --- | --- | --- | --- | --- | --- | --- | --- | --- | --- | --- | --- | --- | --- | --- | --- | --- | --- | --- | --- | --- | --- | --- | --- | --- | --- | --- | --- | --- | --- | --- | --- | --- | --- | --- | --- | --- | --- | --- | --- | --- | --- | --- | --- | --- | --- | --- | --- | --- | --- | --- | --- | --- | --- | --- | --- | --- | --- | --- | --- | --- | --- | --- | --- | --- | --- | --- | --- | --- | --- | --- | --- | --- | --- | --- | --- | --- | --- | --- | --- | --- | --- | --- | --- | --- | --- | --- | --- | --- | --- | --- | --- | --- | --- | --- | --- | --- | --- | --- | --- | --- | --- | --- | --- | --- | --- | --- | --- | --- | --- | --- | --- | --- | --- | --- | --- | --- | --- | --- | --- | --- |
| Actual | Predict  |  |  |  |  |  |  |  |  |  |  |  |  |  |  |  |  |  |  |  |  |  |  |  |  |  |  |  |  |  |  |  |  |  |  |  |  |  |  |  |  |  |  |  |  |  |  |  |  |  |  |  |  |  | | --- | --- | --- | --- | --- | --- | --- | --- | --- | --- | --- | --- | --- | --- | --- | --- | --- | --- | --- | --- | --- | --- | --- | --- | --- | --- | --- | --- | --- | --- | --- | --- | --- | --- | --- | --- | --- | --- | --- | --- | --- | --- | --- | --- | --- | --- | --- | --- | --- | --- | --- | --- | --- | |  | 0 | 1 | 2 | 3 | 4 | 5 | 6 | 7 | 8 | 9 | 10 | 11 | 12 | 13 | 14 | 15 | 16 | 17 | 18 | 19 | 20 | 21 | 22 | 23 | 24 | 25 | 26 | 27 | 28 | 29 | 30 | 31 | 32 | 33 | 34 | 35 | 36 | 37 | 38 | 39 | 40 | 41 | 42 | 43 | 44 | 45 | 46 | 47 | 48 | 49 | 50 | 51 | | 0 | 22 | 1 | 0 | 0 | 0 | 0 | 0 | 0 | 0 | 0 | 0 | 0 | 0 | 0 | 0 | 0 | 0 | 0 | 0 | 0 | 0 | 0 | 0 | 0 | 0 | 0 | 0 | 0 | 0 | 0 | 0 | 0 | 0 | 0 | 0 | 0 | 0 | 0 | 0 | 0 | 0 | 0 | 0 | 0 | 0 | 0 | 0 | 0 | 0 | 0 | 0 | 0 | | 1 | 0 | 15 | 0 | 11 | 1 | 0 | 6 | 0 | 0 | 0 | 0 | 4 | 2 | 0 | 0 | 0 | 0 | 0 | 0 | 0 | 0 | 0 | 0 | 0 | 0 | 0 | 0 | 0 | 0 | 0 | 0 | 0 | 0 | 0 | 0 | 0 | 0 | 0 | 0 | 0 | 0 | 0 | 0 | 0 | 0 | 0 | 0 | 0 | 0 | 0 | 0 | 0 | | 2 | 0 | 7 | 0 | 9 | 2 | 9 | 2 | 0 | 0 | 0 | 0 | 0 | 0 | 0 | 0 | 0 | 0 | 0 | 0 | 0 | 0 | 0 | 0 | 0 | 7 | 0 | 0 | 0 | 0 | 0 | 0 | 0 | 0 | 0 | 0 | 0 | 0 | 0 | 0 | 0 | 0 | 0 | 0 | 0 | 0 | 0 | 0 | 0 | 0 | 0 | 0 | 0 | | 3 | 0 | 8 | 0 | 13 | 11 | 0 | 2 | 0 | 0 | 0 | 0 | 0 | 0 | 0 | 0 | 0 | 0 | 7 | 0 | 0 | 0 | 0 | 4 | 0 | 0 | 0 | 0 | 0 | 0 | 0 | 0 | 0 | 0 | 0 | 0 | 0 | 0 | 0 | 0 | 2 | 0 | 0 | 0 | 0 | 0 | 0 | 0 | 0 | 0 | 0 | 0 | 0 | | 4 | 0 | 0 | 0 | 2 | 117 | 0 | 0 | 0 | 0 | 0 | 0 | 0 | 0 | 0 | 2 | 0 | 0 | 0 | 0 | 0 | 0 | 0 | 22 | 0 | 0 | 0 | 0 | 0 | 0 | 0 | 0 | 0 | 0 | 0 | 0 | 0 | 0 | 0 | 0 | 0 | 0 | 0 | 0 | 0 | 0 | 0 | 0 | 0 | 0 | 0 | 0 | 0 | | 5 | 0 | 0 | 0 | 3 | 3 | 7 | 0 | 0 | 0 | 0 | 0 | 1 | 0 | 0 | 0 | 0 | 0 | 1 | 0 | 0 | 0 | 0 | 10 | 0 | 0 | 0 | 0 | 0 | 0 | 0 | 0 | 0 | 0 | 0 | 0 | 0 | 0 | 0 | 0 | 0 | 0 | 0 | 0 | 0 | 0 | 0 | 0 | 0 | 0 | 0 | 0 | 0 | | 6 | 0 | 19 | 0 | 5 | 2 | 0 | 70 | 0 | 0 | 0 | 0 | 0 | 2 | 1 | 0 | 0 | 0 | 1 | 0 | 0 | 0 | 0 | 0 | 0 | 0 | 0 | 0 | 0 | 0 | 0 | 0 | 0 | 0 | 0 | 0 | 0 | 0 | 0 | 0 | 0 | 0 | 0 | 0 | 0 | 0 | 0 | 0 | 0 | 0 | 0 | 0 | 0 | | 7 | 0 | 6 | 0 | 0 | 0 | 0 | 0 | 0 | 0 | 0 | 0 | 0 | 0 | 0 | 0 | 0 | 0 | 0 | 0 | 0 | 0 | 0 | 0 | 0 | 0 | 0 | 0 | 0 | 0 | 0 | 0 | 0 | 0 | 0 | 0 | 0 | 0 | 0 | 0 | 0 | 0 | 0 | 0 | 0 | 0 | 0 | 0 | 0 | 0 | 0 | 0 | 0 | | 8 | 0 | 0 | 0 | 0 | 0 | 0 | 0 | 0 | 11 | 0 | 0 | 0 | 0 | 0 | 0 | 0 | 0 | 0 | 0 | 0 | 0 | 0 | 0 | 0 | 0 | 0 | 0 | 0 | 0 | 0 | 0 | 0 | 0 | 0 | 0 | 0 | 0 | 0 | 0 | 0 | 0 | 0 | 0 | 0 | 0 | 0 | 0 | 0 | 0 | 0 | 0 | 0 | | 9 | 0 | 9 | 0 | 5 | 5 | 0 | 0 | 0 | 0 | 10 | 0 | 0 | 0 | 0 | 0 | 0 | 0 | 5 | 0 | 0 | 0 | 0 | 0 | 0 | 0 | 0 | 0 | 0 | 0 | 0 | 0 | 0 | 0 | 0 | 0 | 0 | 0 | 0 | 0 | 0 | 0 | 0 | 0 | 0 | 0 | 0 | 0 | 0 | 0 | 0 | 0 | 0 | | 10 | 0 | 1 | 0 | 3 | 0 | 0 | 3 | 0 | 0 | 0 | 42 | 0 | 0 | 0 | 0 | 0 | 0 | 2 | 0 | 0 | 0 | 0 | 0 | 0 | 0 | 0 | 0 | 0 | 0 | 0 | 0 | 0 | 0 | 0 | 0 | 0 | 0 | 0 | 0 | 0 | 0 | 0 | 0 | 0 | 0 | 0 | 0 | 0 | 0 | 0 | 0 | 0 | | 11 | 0 | 4 | 0 | 2 | 0 | 0 | 0 | 0 | 0 | 0 | 0 | 7 | 0 | 0 | 0 | 0 | 0 | 1 | 0 | 0 | 0 | 0 | 0 | 0 | 0 | 0 | 0 | 0 | 0 | 0 | 0 | 0 | 0 | 0 | 0 | 0 | 0 | 0 | 0 | 0 | 0 | 0 | 0 | 0 | 0 | 0 | 0 | 0 | 0 | 0 | 0 | 0 | | 12 | 0 | 0 | 0 | 0 | 0 | 0 | 0 | 0 | 0 | 0 | 0 | 0 | 70 | 0 | 0 | 0 | 0 | 0 | 0 | 0 | 0 | 0 | 0 | 0 | 0 | 0 | 0 | 0 | 0 | 0 | 0 | 0 | 0 | 0 | 0 | 0 | 0 | 0 | 0 | 0 | 0 | 0 | 0 | 0 | 0 | 0 | 0 | 0 | 0 | 0 | 0 | 0 | | 13 | 0 | 0 | 0 | 0 | 0 | 0 | 10 | 0 | 0 | 0 | 0 | 0 | 0 | 12 | 1 | 0 | 0 | 0 | 0 | 0 | 0 | 0 | 0 | 0 | 0 | 0 | 0 | 0 | 0 | 0 | 0 | 0 | 0 | 0 | 0 | 0 | 0 | 0 | 0 | 0 | 0 | 0 | 0 | 0 | 0 | 0 | 0 | 0 | 0 | 0 | 0 | 0 | | 14 | 0 | 2 | 0 | 7 | 0 | 0 | 3 | 0 | 0 | 0 | 0 | 0 | 0 | 0 | 14 | 0 | 0 | 0 | 0 | 0 | 0 | 0 | 0 | 0 | 1 | 0 | 0 | 0 | 0 | 0 | 0 | 0 | 0 | 0 | 0 | 0 | 0 | 0 | 0 | 0 | 0 | 0 | 0 | 0 | 0 | 0 | 0 | 0 | 0 | 0 | 0 | 0 | | 15 | 0 | 0 | 0 | 17 | 5 | 0 | 0 | 0 | 0 | 0 | 0 | 0 | 0 | 0 | 0 | 5 | 0 | 0 | 0 | 0 | 0 | 0 | 0 | 0 | 0 | 0 | 0 | 0 | 0 | 0 | 0 | 0 | 0 | 0 | 0 | 0 | 0 | 0 | 0 | 0 | 0 | 0 | 0 | 0 | 0 | 0 | 0 | 0 | 0 | 0 | 0 | 0 | | 16 | 0 | 0 | 0 | 1 | 0 | 0 | 0 | 0 | 0 | 0 | 0 | 0 | 0 | 0 | 0 | 0 | 14 | 0 | 0 | 0 | 0 | 0 | 0 | 0 | 0 | 0 | 0 | 0 | 0 | 0 | 0 | 0 | 0 | 0 | 0 | 0 | 0 | 0 | 0 | 0 | 0 | 0 | 0 | 0 | 0 | 0 | 0 | 0 | 0 | 0 | 0 | 0 | | 17 | 0 | 0 | 0 | 1 | 2 | 0 | 0 | 0 | 0 | 0 | 0 | 0 | 0 | 0 | 0 | 0 | 1 | 1 | 0 | 0 | 0 | 0 | 1 | 0 | 0 | 0 | 2 | 0 | 0 | 0 | 0 | 0 | 0 | 0 | 0 | 0 | 0 | 0 | 0 | 0 | 0 | 0 | 0 | 0 | 0 | 0 | 0 | 0 | 0 | 0 | 0 | 0 | | 18 | 0 | 0 | 0 | 4 | 0 | 0 | 0 | 0 | 0 | 0 | 0 | 0 | 3 | 0 | 0 | 0 | 0 | 0 | 0 | 0 | 0 | 0 | 0 | 0 | 0 | 0 | 0 | 0 | 0 | 0 | 0 | 0 | 0 | 0 | 0 | 0 | 0 | 0 | 0 | 0 | 0 | 0 | 0 | 0 | 0 | 0 | 0 | 0 | 0 | 0 | 0 | 0 | | 19 | 0 | 1 | 0 | 9 | 6 | 1 | 1 | 0 | 0 | 0 | 0 | 0 | 0 | 0 | 0 | 0 | 0 | 0 | 0 | 0 | 0 | 0 | 0 | 0 | 10 | 0 | 0 | 0 | 0 | 0 | 0 | 0 | 0 | 0 | 0 | 0 | 0 | 0 | 0 | 0 | 0 | 0 | 0 | 0 | 0 | 0 | 0 | 0 | 0 | 0 | 0 | 0 | | 20 | 0 | 5 | 0 | 2 | 7 | 0 | 0 | 0 | 0 | 1 | 0 | 0 | 0 | 0 | 0 | 0 | 0 | 0 | 0 | 0 | 0 | 1 | 0 | 0 | 0 | 0 | 0 | 0 | 0 | 0 | 0 | 0 | 0 | 0 | 0 | 0 | 0 | 0 | 0 | 0 | 0 | 0 | 0 | 0 | 0 | 0 | 0 | 0 | 0 | 0 | 0 | 0 | | 21 | 0 | 10 | 0 | 4 | 0 | 0 | 0 | 0 | 0 | 0 | 0 | 0 | 0 | 0 | 0 | 0 | 0 | 0 | 0 | 0 | 0 | 6 | 0 | 0 | 0 | 0 | 0 | 0 | 0 | 0 | 0 | 0 | 0 | 0 | 0 | 0 | 0 | 0 | 0 | 0 | 0 | 0 | 0 | 0 | 0 | 0 | 0 | 0 | 0 | 0 | 0 | 0 | | 22 | 0 | 0 | 0 | 0 | 1 | 0 | 0 | 0 | 0 | 0 | 0 | 0 | 0 | 0 | 0 | 0 | 0 | 0 | 0 | 0 | 0 | 0 | 61 | 0 | 0 | 0 | 0 | 0 | 0 | 0 | 0 | 0 | 0 | 0 | 0 | 0 | 0 | 0 | 0 | 0 | 0 | 0 | 0 | 0 | 0 | 0 | 0 | 0 | 0 | 0 | 0 | 0 | | 23 | 0 | 0 | 0 | 1 | 0 | 0 | 0 | 0 | 0 | 0 | 0 | 0 | 0 | 0 | 0 | 0 | 0 | 5 | 0 | 0 | 0 | 0 | 7 | 0 | 0 | 0 | 0 | 0 | 0 | 0 | 0 | 0 | 0 | 0 | 0 | 0 | 0 | 0 | 0 | 0 | 0 | 0 | 0 | 0 | 0 | 0 | 0 | 0 | 0 | 0 | 0 | 0 | | 24 | 0 | 15 | 0 | 14 | 5 | 0 | 1 | 0 | 0 | 0 | 0 | 7 | 2 | 0 | 0 | 0 | 0 | 3 | 0 | 0 | 0 | 0 | 0 | 0 | 3 | 0 | 0 | 0 | 0 | 0 | 0 | 0 | 0 | 0 | 0 | 0 | 0 | 0 | 0 | 0 | 0 | 0 | 0 | 0 | 0 | 0 | 0 | 0 | 0 | 0 | 0 | 0 | | 25 | 0 | 19 | 0 | 7 | 0 | 3 | 1 | 0 | 0 | 0 | 0 | 2 | 10 | 0 | 0 | 0 | 0 | 3 | 0 | 0 | 0 | 0 | 0 | 0 | 0 | 1 | 1 | 0 | 0 | 0 | 0 | 0 | 0 | 0 | 0 | 0 | 0 | 0 | 4 | 0 | 0 | 0 | 0 | 0 | 0 | 0 | 0 | 0 | 0 | 0 | 0 | 0 | | 26 | 0 | 0 | 0 | 0 | 0 | 0 | 0 | 0 | 0 | 0 | 0 | 0 | 0 | 0 | 0 | 0 | 0 | 0 | 0 | 0 | 0 | 0 | 0 | 0 | 0 | 0 | 10 | 0 | 0 | 0 | 0 | 0 | 0 | 0 | 0 | 0 | 0 | 0 | 0 | 0 | 0 | 0 | 0 | 0 | 0 | 0 | 0 | 0 | 0 | 0 | 0 | 0 | | 27 | 0 | 2 | 0 | 8 | 3 | 0 | 0 | 0 | 0 | 0 | 0 | 0 | 2 | 0 | 0 | 0 | 0 | 0 | 0 | 0 | 0 | 0 | 0 | 0 | 0 | 0 | 0 | 0 | 0 | 0 | 0 | 0 | 0 | 0 | 0 | 0 | 0 | 0 | 0 | 0 | 0 | 0 | 0 | 0 | 0 | 0 | 0 | 0 | 0 | 0 | 0 | 0 | | 28 | 0 | 4 | 0 | 2 | 1 | 0 | 0 | 0 | 0 | 0 | 0 | 0 | 0 | 0 | 0 | 0 | 0 | 0 | 0 | 0 | 0 | 0 | 0 | 0 | 0 | 0 | 0 | 0 | 0 | 0 | 0 | 0 | 0 | 0 | 0 | 0 | 0 | 0 | 0 | 0 | 0 | 0 | 0 | 0 | 0 | 0 | 0 | 0 | 0 | 0 | 0 | 0 | | 29 | 0 | 7 | 0 | 4 | 7 | 0 | 0 | 0 | 0 | 0 | 0 | 0 | 0 | 0 | 0 | 1 | 0 | 2 | 0 | 0 | 0 | 0 | 6 | 0 | 0 | 0 | 1 | 0 | 0 | 0 | 1 | 0 | 0 | 0 | 0 | 0 | 0 | 0 | 0 | 0 | 0 | 0 | 0 | 0 | 0 | 0 | 0 | 0 | 0 | 0 | 0 | 0 | | 30 | 0 | 0 | 0 | 15 | 2 | 0 | 0 | 0 | 0 | 3 | 0 | 0 | 1 | 0 | 0 | 0 | 0 | 0 | 0 | 0 | 0 | 0 | 1 | 0 | 0 | 0 | 0 | 0 | 0 | 0 | 4 | 0 | 0 | 0 | 0 | 0 | 0 | 0 | 0 | 0 | 0 | 0 | 0 | 0 | 0 | 0 | 0 | 0 | 0 | 0 | 0 | 0 | | 31 | 0 | 2 | 0 | 8 | 0 | 0 | 0 | 0 | 0 | 0 | 0 | 0 | 0 | 0 | 0 | 0 | 0 | 0 | 0 | 0 | 0 | 0 | 0 | 0 | 0 | 0 | 0 | 0 | 0 | 0 | 0 | 0 | 0 | 0 | 0 | 0 | 0 | 0 | 0 | 0 | 0 | 0 | 0 | 0 | 0 | 0 | 0 | 0 | 0 | 0 | 0 | 0 | | 32 | 0 | 1 | 0 | 1 | 0 | 0 | 0 | 0 | 0 | 0 | 0 | 0 | 0 | 0 | 2 | 0 | 0 | 0 | 0 | 0 | 0 | 0 | 0 | 0 | 0 | 0 | 0 | 0 | 0 | 0 | 0 | 0 | 7 | 0 | 0 | 0 | 0 | 0 | 0 | 0 | 0 | 0 | 0 | 0 | 0 | 0 | 0 | 0 | 0 | 0 | 0 | 0 | | 33 | 0 | 2 | 0 | 3 | 20 | 0 | 0 | 0 | 0 | 0 | 0 | 0 | 0 | 0 | 0 | 0 | 0 | 0 | 0 | 0 | 0 | 0 | 0 | 0 | 0 | 0 | 0 | 0 | 0 | 0 | 0 | 0 | 0 | 0 | 0 | 0 | 0 | 0 | 0 | 0 | 0 | 0 | 0 | 0 | 0 | 0 | 0 | 0 | 0 | 0 | 0 | 0 | | 34 | 0 | 0 | 0 | 0 | 1 | 0 | 0 | 0 | 0 | 0 | 0 | 0 | 0 | 0 | 0 | 0 | 0 | 0 | 0 | 0 | 0 | 0 | 0 | 0 | 0 | 0 | 0 | 0 | 0 | 0 | 0 | 0 | 0 | 0 | 6 | 0 | 0 | 0 | 0 | 0 | 0 | 0 | 0 | 0 | 0 | 0 | 0 | 0 | 0 | 0 | 0 | 0 | | 35 | 0 | 1 | 0 | 0 | 0 | 0 | 0 | 0 | 0 | 0 | 0 | 0 | 0 | 0 | 0 | 0 | 0 | 4 | 0 | 0 | 0 | 0 | 0 | 0 | 0 | 0 | 0 | 0 | 0 | 0 | 0 | 0 | 0 | 0 | 0 | 0 | 0 | 0 | 0 | 0 | 0 | 0 | 0 | 0 | 0 | 0 | 0 | 0 | 0 | 0 | 0 | 0 | | 36 | 0 | 4 | 0 | 0 | 0 | 0 | 0 | 0 | 0 | 0 | 0 | 0 | 0 | 0 | 0 | 0 | 0 | 0 | 0 | 0 | 0 | 0 | 0 | 0 | 0 | 0 | 1 | 0 | 0 | 0 | 0 | 0 | 0 | 0 | 0 | 0 | 0 | 0 | 0 | 0 | 0 | 0 | 0 | 0 | 0 | 0 | 0 | 0 | 0 | 0 | 0 | 0 | | 37 | 0 | 0 | 0 | 5 | 0 | 0 | 0 | 0 | 0 | 0 | 0 | 0 | 0 | 0 | 0 | 0 | 0 | 1 | 0 | 0 | 0 | 0 | 0 | 0 | 0 | 0 | 0 | 0 | 0 | 0 | 0 | 0 | 0 | 0 | 0 | 0 | 0 | 0 | 0 | 0 | 0 | 0 | 0 | 0 | 0 | 0 | 0 | 0 | 0 | 0 | 0 | 0 | | 38 | 0 | 0 | 0 | 7 | 0 | 0 | 0 | 0 | 0 | 0 | 0 | 0 | 0 | 0 | 0 | 0 | 0 | 0 | 0 | 0 | 0 | 0 | 26 | 0 | 0 | 0 | 0 | 0 | 0 | 0 | 0 | 0 | 0 | 0 | 0 | 0 | 0 | 0 | 22 | 0 | 0 | 0 | 0 | 0 | 0 | 0 | 0 | 0 | 0 | 0 | 0 | 0 | | 39 | 0 | 1 | 0 | 4 | 1 | 0 | 0 | 0 | 0 | 0 | 0 | 0 | 0 | 0 | 0 | 0 | 0 | 1 | 0 | 0 | 0 | 0 | 0 | 0 | 0 | 0 | 0 | 0 | 0 | 0 | 0 | 0 | 0 | 0 | 0 | 0 | 0 | 0 | 0 | 0 | 0 | 0 | 0 | 0 | 0 | 0 | 0 | 0 | 0 | 0 | 0 | 0 | | 40 | 0 | 6 | 0 | 15 | 7 | 0 | 4 | 0 | 0 | 0 | 0 | 0 | 0 | 0 | 0 | 0 | 2 | 0 | 0 | 0 | 0 | 0 | 6 | 0 | 0 | 0 | 0 | 0 | 0 | 0 | 0 | 0 | 0 | 0 | 0 | 0 | 0 | 0 | 0 | 1 | 0 | 0 | 0 | 0 | 0 | 0 | 0 | 0 | 0 | 0 | 0 | 0 | | 41 | 0 | 0 | 0 | 4 | 2 | 0 | 0 | 0 | 0 | 0 | 0 | 0 | 0 | 0 | 0 | 0 | 0 | 0 | 0 | 0 | 0 | 0 | 0 | 0 | 0 | 0 | 0 | 0 | 0 | 0 | 0 | 0 | 0 | 0 | 0 | 0 | 0 | 0 | 0 | 0 | 0 | 0 | 0 | 0 | 0 | 0 | 0 | 0 | 0 | 0 | 0 | 0 | | 42 | 0 | 0 | 0 | 8 | 0 | 0 | 0 | 0 | 0 | 0 | 0 | 0 | 0 | 0 | 0 | 0 | 0 | 0 | 0 | 0 | 0 | 0 | 0 | 0 | 0 | 0 | 0 | 0 | 0 | 0 | 0 | 0 | 0 | 0 | 0 | 0 | 0 | 0 | 0 | 0 | 0 | 0 | 0 | 0 | 0 | 0 | 0 | 0 | 0 | 0 | 0 | 0 | | 43 | 0 | 2 | 0 | 4 | 0 | 0 | 0 | 0 | 0 | 0 | 0 | 0 | 0 | 0 | 0 | 0 | 0 | 0 | 0 | 0 | 0 | 0 | 0 | 0 | 0 | 0 | 0 | 0 | 0 | 0 | 0 | 0 | 0 | 0 | 0 | 0 | 0 | 0 | 0 | 0 | 0 | 0 | 0 | 0 | 0 | 0 | 0 | 0 | 0 | 0 | 0 | 0 | | 44 | 0 | 6 | 0 | 7 | 0 | 0 | 2 | 0 | 0 | 0 | 0 | 1 | 0 | 0 | 0 | 0 | 0 | 0 | 0 | 0 | 0 | 0 | 0 | 0 | 0 | 0 | 0 | 0 | 0 | 0 | 0 | 0 | 0 | 0 | 0 | 0 | 0 | 0 | 0 | 0 | 0 | 0 | 0 | 0 | 0 | 0 | 0 | 0 | 0 | 0 | 0 | 0 | | 45 | 0 | 0 | 0 | 1 | 0 | 0 | 0 | 0 | 0 | 0 | 0 | 0 | 0 | 0 | 0 | 0 | 0 | 0 | 0 | 0 | 0 | 0 | 13 | 0 | 0 | 0 | 0 | 0 | 0 | 0 | 0 | 0 | 0 | 0 | 0 | 0 | 0 | 0 | 0 | 0 | 0 | 0 | 0 | 0 | 0 | 0 | 0 | 0 | 0 | 0 | 0 | 0 | | 46 | 1 | 0 | 0 | 0 | 0 | 0 | 1 | 0 | 0 | 0 | 0 | 0 | 0 | 0 | 0 | 0 | 5 | 0 | 0 | 0 | 0 | 0 | 1 | 0 | 0 | 0 | 0 | 0 | 0 | 0 | 0 | 0 | 0 | 0 | 0 | 0 | 0 | 0 | 0 | 0 | 0 | 0 | 0 | 0 | 0 | 0 | 0 | 0 | 0 | 0 | 0 | 0 | | 47 | 0 | 1 | 0 | 0 | 0 | 0 | 1 | 0 | 0 | 0 | 0 | 0 | 0 | 0 | 0 | 0 | 0 | 0 | 0 | 0 | 0 | 0 | 3 | 0 | 0 | 0 | 0 | 0 | 0 | 0 | 0 | 0 | 0 | 0 | 0 | 0 | 0 | 0 | 0 | 0 | 0 | 0 | 0 | 0 | 0 | 0 | 0 | 0 | 0 | 0 | 0 | 0 | | 48 | 0 | 1 | 0 | 1 | 0 | 0 | 0 | 0 | 0 | 0 | 0 | 0 | 0 | 0 | 0 | 0 | 0 | 0 | 0 | 0 | 0 | 0 | 1 | 0 | 0 | 0 | 0 | 0 | 0 | 0 | 0 | 0 | 2 | 0 | 0 | 0 | 0 | 0 | 5 | 0 | 0 | 0 | 0 | 0 | 0 | 0 | 0 | 0 | 0 | 0 | 0 | 0 | | 49 | 0 | 2 | 0 | 0 | 0 | 1 | 0 | 0 | 0 | 0 | 0 | 0 | 0 | 0 | 0 | 0 | 0 | 0 | 0 | 0 | 0 | 0 | 4 | 0 | 0 | 0 | 0 | 0 | 0 | 0 | 0 | 0 | 0 | 0 | 0 | 0 | 0 | 0 | 0 | 0 | 0 | 0 | 0 | 0 | 0 | 0 | 0 | 0 | 0 | 0 | 0 | 0 | | 50 | 0 | 0 | 0 | 0 | 0 | 0 | 0 | 0 | 0 | 0 | 0 | 0 | 0 | 0 | 0 | 0 | 0 | 0 | 0 | 0 | 0 | 0 | 0 | 0 | 0 | 0 | 0 | 0 | 0 | 0 | 0 | 0 | 0 | 0 | 0 | 0 | 0 | 0 | 0 | 0 | 0 | 0 | 0 | 0 | 0 | 0 | 0 | 0 | 0 | 0 | 5 | 0 | | 51 | 0 | 0 | 0 | 0 | 1 | 1 | 0 | 0 | 0 | 0 | 0 | 0 | 0 | 0 | 0 | 0 | 0 | 0 | 0 | 0 | 0 | 0 | 5 | 0 | 0 | 0 | 0 | 0 | 0 | 0 | 0 | 0 | 0 | 0 | 0 | 0 | 0 | 0 | 0 | 0 | 0 | 0 | 0 | 0 | 0 | 0 | 0 | 0 | 0 | 0 | 0 | 0 | |

## Overall Statistics :

|  |  |
| --- | --- |
| 95% CI | (0.40098,0.45484) |
| ACC Macro | 0.978 |
| ARI | 0.26479 |
| AUNP | 0.7 |
| AUNU | 0.63585 |
| Bangdiwala B | 0.3758 |
| Bennett S | 0.41669 |
| CBA | 0.23327 |
| CSI | None |
| Chi-Squared | None |
| Chi-Squared DF | 2601 |
| Conditional Entropy | 1.40236 |
| Cramer V | None |
| Cross Entropy | 3.67629 |
| F1 Macro | 0.27063 |
| F1 Micro | 0.42791 |
| FNR Macro | 0.71677 |
| FNR Micro | 0.57209 |
| FPR Macro | 0.01154 |
| FPR Micro | 0.01122 |
| Gwet AC1 | 0.41718 |
| Hamming Loss | 0.57209 |
| Joint Entropy | 6.52635 |
| KL Divergence | None |
| Kappa | 0.39792 |
| Kappa 95% CI | (0.36958,0.42626) |
| Kappa No Prevalence | -0.14418 |
| Kappa Standard Error | 0.01446 |
| Kappa Unbiased | 0.39074 |
| Krippendorff Alpha | 0.39097 |
| Lambda A | 0.37868 |
| Lambda B | 0.53981 |
| Mutual Information | 2.33218 |
| NIR | 0.11025 |
| Overall ACC | 0.42791 |
| Overall CEN | 0.31615 |
| Overall J | (11.32048,0.2177) |
| Overall MCC | 0.40778 |
| Overall MCEN | 0.37886 |
| Overall RACC | 0.04981 |
| Overall RACCU | 0.06102 |
| P-Value | None |
| PPV Macro | None |
| PPV Micro | 0.42791 |
| Pearson C | None |
| Phi-Squared | None |
| RCI | 0.45515 |
| RR | 24.94231 |
| Reference Entropy | 5.12399 |
| Response Entropy | 3.73454 |
| SOA1(Landis & Koch) | Fair |
| SOA2(Fleiss) | Poor |
| SOA3(Altman) | Fair |
| SOA4(Cicchetti) | Poor |
| SOA5(Cramer) | None |
| SOA6(Matthews) | Weak |
| Scott PI | 0.39074 |
| Standard Error | 0.01374 |
| TNR Macro | 0.98846 |
| TNR Micro | 0.98878 |
| TPR Macro | 0.28323 |
| TPR Micro | 0.42791 |
| Zero-one Loss | 742 |

## Class Statistics :

|  |  |  |  |  |  |  |  |  |  |  |  |  |  |  |  |  |  |  |  |  |  |  |  |  |  |  |  |  |  |  |  |  |  |  |  |  |  |  |  |  |  |  |  |  |  |  |  |  |  |  |  |  |  |
| --- | --- | --- | --- | --- | --- | --- | --- | --- | --- | --- | --- | --- | --- | --- | --- | --- | --- | --- | --- | --- | --- | --- | --- | --- | --- | --- | --- | --- | --- | --- | --- | --- | --- | --- | --- | --- | --- | --- | --- | --- | --- | --- | --- | --- | --- | --- | --- | --- | --- | --- | --- | --- | --- |
| Class | 0 | 1 | 2 | 3 | 4 | 5 | 6 | 7 | 8 | 9 | 10 | 11 | 12 | 13 | 14 | 15 | 16 | 17 | 18 | 19 | 20 | 21 | 22 | 23 | 24 | 25 | 26 | 27 | 28 | 29 | 30 | 31 | 32 | 33 | 34 | 35 | 36 | 37 | 38 | 39 | 40 | 41 | 42 | 43 | 44 | 45 | 46 | 47 | 48 | 49 | 50 | 51 | Description |
| ACC | 0.99846 | 0.86662 | 0.97224 | 0.8165 | 0.90671 | 0.97456 | 0.94834 | 0.99537 | 1.0 | 0.97841 | 0.99306 | 0.98304 | 0.98304 | 0.99075 | 0.98612 | 0.98227 | 0.99306 | 0.96685 | 0.9946 | 0.97841 | 0.98766 | 0.98843 | 0.91442 | 0.98998 | 0.94988 | 0.96145 | 0.99614 | 0.98843 | 0.9946 | 0.97764 | 0.98227 | 0.99229 | 0.99537 | 0.98072 | 0.99923 | 0.99614 | 0.99614 | 0.99537 | 0.96762 | 0.99229 | 0.96839 | 0.99537 | 0.99383 | 0.99537 | 0.98766 | 0.98921 | 0.99383 | 0.99614 | 0.99229 | 0.9946 | 1.0 | 0.9946 | Accuracy |
| AGF | 0.97764 | 0.47377 | 0.0 | 0.3882 | 0.84802 | 0.53195 | 0.81984 | 0.0 | 1.0 | 0.57282 | 0.92128 | 0.6676 | 0.96821 | 0.75327 | 0.73909 | 0.46504 | 0.92307 | 0.26783 | 0.0 | 0.0 | 0.0 | 0.58461 | 0.84467 | 0.0 | 0.25627 | 0.15373 | 0.95309 | 0.0 | 0.0 | 0.0 | 0.42538 | 0.0 | 0.8115 | 0.0 | 0.93905 | 0.0 | 0.0 | 0.0 | 0.65456 | 0.0 | 0.0 | 0.0 | 0.0 | 0.0 | 0.0 | 0.0 | 0.0 | 0.0 | 0.0 | 0.0 | 1.0 | 0.0 | Adjusted F-score |
| AGM | 0.98833 | 0.72964 | 0 | 0.65567 | 0.8906 | 0.75487 | 0.89344 | 0 | 1.0 | 0.76613 | 0.95281 | 0.84486 | 0.98666 | 0.85938 | 0.8559 | 0.71169 | 0.97833 | 0.65936 | 0 | 0 | 0 | 0.77161 | 0.92925 | 0 | 0.60707 | 0.56139 | 0.99709 | 0 | 0 | 0 | 0.69257 | 0 | 0.89735 | 0 | 0.96281 | 0 | 0 | 0 | 0.80753 | 0 | 0 | 0 | 0 | 0 | 0 | 0 | 0 | 0 | 0 | 0 | 1.0 | 0 | Adjusted geometric mean |
| AM | 0 | 125 | -36 | 170 | 69 | -3 | 7 | -6 | 0 | -20 | -9 | 8 | 22 | -10 | -8 | -21 | 7 | 29 | -7 | -28 | -16 | -13 | 109 | -13 | -29 | -50 | 5 | -15 | -7 | -29 | -21 | -10 | -2 | -25 | -1 | -5 | -5 | -6 | -24 | -4 | -41 | -6 | -8 | -6 | -16 | -14 | -8 | -5 | -10 | -7 | 0 | -7 | Difference between automatic and manual classification |
| AUC | 0.97787 | 0.63309 | 0.5 | 0.5567 | 0.86793 | 0.6341 | 0.83454 | 0.5 | 1.0 | 0.64548 | 0.91176 | 0.74415 | 0.99104 | 0.76048 | 0.75729 | 0.5922 | 0.96355 | 0.54854 | 0.5 | 0.5 | 0.5 | 0.64961 | 0.9474 | 0.5 | 0.52278 | 0.5098 | 0.99806 | 0.5 | 0.5 | 0.5 | 0.57653 | 0.5 | 0.8174 | 0.5 | 0.92857 | 0.5 | 0.5 | 0.5 | 0.69638 | 0.49884 | 0.5 | 0.5 | 0.5 | 0.5 | 0.5 | 0.5 | 0.5 | 0.5 | 0.5 | 0.5 | 1.0 | 0.5 | Area under the ROC curve |
| AUCI | Excellent | Fair | Poor | Poor | Very Good | Fair | Very Good | Poor | Excellent | Fair | Excellent | Good | Excellent | Good | Good | Poor | Excellent | Poor | Poor | Poor | Poor | Fair | Excellent | Poor | Poor | Poor | Excellent | Poor | Poor | Poor | Poor | Poor | Very Good | Poor | Excellent | Poor | Poor | Poor | Fair | Poor | Poor | Poor | Poor | Poor | Poor | Poor | Poor | Poor | Poor | Poor | Excellent | Poor | AUC value interpretation |
| AUPR | 0.95652 | 0.23804 | None | 0.16825 | 0.68503 | 0.29909 | 0.6771 | None | 1.0 | 0.5042 | 0.91176 | 0.40909 | 0.88043 | 0.72241 | 0.62768 | 0.50926 | 0.78485 | 0.07601 | None | None | None | 0.57857 | 0.6703 | None | 0.10143 | 0.5098 | 0.83333 | None | None | None | 0.47692 | None | 0.70707 | None | 0.92857 | None | None | None | 0.55484 | 0.0 | None | None | None | None | None | None | None | None | None | None | 1.0 | None | Area under the PR curve |
| BCD | 0.0 | 0.04819 | 0.01388 | 0.06554 | 0.0266 | 0.00116 | 0.0027 | 0.00231 | 0.0 | 0.00771 | 0.00347 | 0.00308 | 0.00848 | 0.00386 | 0.00308 | 0.0081 | 0.0027 | 0.01118 | 0.0027 | 0.01079 | 0.00617 | 0.00501 | 0.04202 | 0.00501 | 0.01118 | 0.01928 | 0.00193 | 0.00578 | 0.0027 | 0.01118 | 0.0081 | 0.00386 | 0.00077 | 0.00964 | 0.00039 | 0.00193 | 0.00193 | 0.00231 | 0.00925 | 0.00154 | 0.01581 | 0.00231 | 0.00308 | 0.00231 | 0.00617 | 0.0054 | 0.00308 | 0.00193 | 0.00386 | 0.0027 | 0.0 | 0.0027 | Bray-Curtis dissimilarity |
| BM | 0.95574 | 0.26617 | 0.0 | 0.1134 | 0.73586 | 0.26821 | 0.66909 | 0.0 | 1.0 | 0.29095 | 0.82353 | 0.48831 | 0.98207 | 0.52095 | 0.51458 | 0.1844 | 0.92709 | 0.09707 | 0.0 | 0.0 | 0.0 | 0.29922 | 0.8948 | 0.0 | 0.04557 | 0.01961 | 0.99611 | 0.0 | 0.0 | 0.0 | 0.15306 | 0.0 | 0.63481 | 0.0 | 0.85714 | 0.0 | 0.0 | 0.0 | 0.39275 | -0.00233 | 0.0 | 0.0 | 0.0 | 0.0 | 0.0 | 0.0 | 0.0 | 0.0 | 0.0 | 0.0 | 1.0 | 0.0 | Informedness or bookmaker informedness |
| CEN | 0.03599 | 0.60784 | 0.35701 | 0.69829 | 0.28271 | 0.34211 | 0.25116 | 0.0 | 0 | 0.27559 | 0.07629 | 0.30843 | 0.10685 | 0.11998 | 0.22486 | 0.15861 | 0.13478 | 0.55875 | 0.14766 | 0.30695 | 0.28793 | 0.1671 | 0.31405 | 0.19419 | 0.41571 | 0.38024 | 0.12721 | 0.25826 | 0.20664 | 0.39312 | 0.2349 | 0.1082 | 0.16434 | 0.1373 | 0.04266 | 0.1082 | 0.1082 | 0.09742 | 0.18896 | 0.34799 | 0.36695 | 0.13763 | 0.0 | 0.13763 | 0.2514 | 0.05564 | 0.23212 | 0.20547 | 0.29389 | 0.20664 | 0 | 0.17218 | Confusion entropy |
| DOR | 28006.0 | 4.65185 | None | 1.9605 | 50.16316 | 32.58889 | 73.15315 | None | None | 131.14583 | None | 84.53333 | None | 1388.72727 | 272.46154 | 288.40909 | 2229.5 | 4.97222 | None | None | None | 546.85714 | 623.86364 | None | 4.35816 | None | None | None | None | None | 230.90909 | None | 1123.5 | None | None | None | None | None | 91.33333 | 0.0 | None | None | None | None | None | None | None | None | None | None | None | None | Diagnostic odds ratio |
| DP | 2.4519 | 0.36808 | None | 0.16119 | 0.93747 | 0.8342 | 1.02781 | None | None | 1.16758 | None | 1.06243 | None | 1.73262 | 1.34265 | 1.35627 | 1.84596 | 0.38403 | None | None | None | 1.50947 | 1.54101 | None | 0.35247 | None | None | None | None | None | 1.30303 | None | 1.68187 | None | None | None | None | None | 1.08095 | None | None | None | None | None | None | None | None | None | None | None | None | None | Discriminant power |
| DPI | Fair | Poor | None | Poor | Poor | Poor | Limited | None | None | Limited | None | Limited | None | Limited | Limited | Limited | Limited | Poor | None | None | None | Limited | Limited | None | Poor | None | None | None | None | None | Limited | None | Limited | None | None | None | None | None | Limited | None | None | None | None | None | None | None | None | None | None | None | None | None | Discriminant power interpretation |
| ERR | 0.00154 | 0.13338 | 0.02776 | 0.1835 | 0.09329 | 0.02544 | 0.05166 | 0.00463 | 0.0 | 0.02159 | 0.00694 | 0.01696 | 0.01696 | 0.00925 | 0.01388 | 0.01773 | 0.00694 | 0.03315 | 0.0054 | 0.02159 | 0.01234 | 0.01157 | 0.08558 | 0.01002 | 0.05012 | 0.03855 | 0.00386 | 0.01157 | 0.0054 | 0.02236 | 0.01773 | 0.00771 | 0.00463 | 0.01928 | 0.00077 | 0.00386 | 0.00386 | 0.00463 | 0.03238 | 0.00771 | 0.03161 | 0.00463 | 0.00617 | 0.00463 | 0.01234 | 0.01079 | 0.00617 | 0.00386 | 0.00771 | 0.0054 | 0.0 | 0.0054 | Error rate |
| F0.5 | 0.95652 | 0.10791 | 0.0 | 0.07104 | 0.59031 | 0.30973 | 0.66288 | 0.0 | 1.0 | 0.55556 | 0.9589 | 0.34314 | 0.79909 | 0.8 | 0.67961 | 0.4902 | 0.67961 | 0.03205 | 0.0 | 0.0 | 0.0 | 0.625 | 0.40885 | 0.0 | 0.11194 | 0.09091 | 0.71429 | 0.0 | 0.0 | 0.0 | 0.43478 | 0.0 | 0.74468 | 0.0 | 0.96774 | 0.0 | 0.0 | 0.0 | 0.61453 | 0.0 | 0.0 | 0.0 | 0.0 | 0.0 | 0.0 | 0.0 | 0.0 | 0.0 | 0.0 | 0.0 | 1.0 | 0.0 | F0.5 score |
| F1 | 0.95652 | 0.14778 | 0.0 | 0.09848 | 0.65915 | 0.29787 | 0.67633 | 0.0 | 1.0 | 0.41667 | 0.90323 | 0.38889 | 0.8642 | 0.66667 | 0.6087 | 0.30303 | 0.75676 | 0.04444 | 0.0 | 0.0 | 0.0 | 0.44444 | 0.52361 | 0.0 | 0.08451 | 0.03846 | 0.8 | 0.0 | 0.0 | 0.0 | 0.25806 | 0.0 | 0.7 | 0.0 | 0.92308 | 0.0 | 0.0 | 0.0 | 0.51163 | 0.0 | 0.0 | 0.0 | 0.0 | 0.0 | 0.0 | 0.0 | 0.0 | 0.0 | 0.0 | 0.0 | 1.0 | 0.0 | F1 score - harmonic mean of precision and sensitivity |
| F2 | 0.95652 | 0.23438 | 0.0 | 0.16049 | 0.74617 | 0.28689 | 0.69034 | 0.0 | 1.0 | 0.33333 | 0.85366 | 0.44872 | 0.94086 | 0.57143 | 0.55118 | 0.2193 | 0.85366 | 0.07246 | 0.0 | 0.0 | 0.0 | 0.34483 | 0.72792 | 0.0 | 0.06787 | 0.02439 | 0.90909 | 0.0 | 0.0 | 0.0 | 0.18349 | 0.0 | 0.66038 | 0.0 | 0.88235 | 0.0 | 0.0 | 0.0 | 0.43825 | 0.0 | 0.0 | 0.0 | 0.0 | 0.0 | 0.0 | 0.0 | 0.0 | 0.0 | 0.0 | 0.0 | 1.0 | 0.0 | F2 score |
| FDR | 0.04348 | 0.90854 | None | 0.94009 | 0.44811 | 0.68182 | 0.34579 | None | 0.0 | 0.28571 | 0.0 | 0.68182 | 0.23913 | 0.07692 | 0.26316 | 0.16667 | 0.36364 | 0.97297 | None | None | None | 0.14286 | 0.64327 | None | 0.85714 | 0.0 | 0.33333 | None | None | None | 0.2 | None | 0.22222 | None | 0.0 | None | None | None | 0.29032 | 1.0 | None | None | None | None | None | None | None | None | None | None | 0.0 | None | False discovery rate |
| FN | 1 | 24 | 36 | 34 | 26 | 18 | 30 | 6 | 0 | 24 | 9 | 7 | 0 | 11 | 13 | 22 | 1 | 7 | 7 | 28 | 16 | 14 | 1 | 13 | 47 | 50 | 0 | 15 | 7 | 29 | 22 | 10 | 4 | 25 | 1 | 5 | 5 | 6 | 33 | 7 | 41 | 6 | 8 | 6 | 16 | 14 | 8 | 5 | 10 | 7 | 0 | 7 | False negative/miss/type 2 error |
| FNR | 0.04348 | 0.61538 | 1.0 | 0.7234 | 0.18182 | 0.72 | 0.3 | 1.0 | 0.0 | 0.70588 | 0.17647 | 0.5 | 0.0 | 0.47826 | 0.48148 | 0.81481 | 0.06667 | 0.875 | 1.0 | 1.0 | 1.0 | 0.7 | 0.01613 | 1.0 | 0.94 | 0.98039 | 0.0 | 1.0 | 1.0 | 1.0 | 0.84615 | 1.0 | 0.36364 | 1.0 | 0.14286 | 1.0 | 1.0 | 1.0 | 0.6 | 1.0 | 1.0 | 1.0 | 1.0 | 1.0 | 1.0 | 1.0 | 1.0 | 1.0 | 1.0 | 1.0 | 0.0 | 1.0 | Miss rate or false negative rate |
| FOR | 0.00078 | 0.02118 | 0.02776 | 0.03148 | 0.02396 | 0.01412 | 0.02521 | 0.00463 | 0.0 | 0.01871 | 0.00717 | 0.00549 | 0.0 | 0.00857 | 0.01017 | 0.01704 | 0.00078 | 0.00556 | 0.0054 | 0.02159 | 0.01234 | 0.01085 | 0.00089 | 0.01002 | 0.03683 | 0.03858 | 0.0 | 0.01157 | 0.0054 | 0.02236 | 0.01703 | 0.00771 | 0.00311 | 0.01928 | 0.00077 | 0.00386 | 0.00386 | 0.00463 | 0.02607 | 0.00541 | 0.03161 | 0.00463 | 0.00617 | 0.00463 | 0.01234 | 0.01079 | 0.00617 | 0.00386 | 0.00771 | 0.0054 | 0.0 | 0.0054 | False omission rate |
| FP | 1 | 149 | 0 | 204 | 95 | 15 | 37 | 0 | 0 | 4 | 0 | 15 | 22 | 1 | 5 | 1 | 8 | 36 | 0 | 0 | 0 | 1 | 110 | 0 | 18 | 0 | 5 | 0 | 0 | 0 | 1 | 0 | 2 | 0 | 0 | 0 | 0 | 0 | 9 | 3 | 0 | 0 | 0 | 0 | 0 | 0 | 0 | 0 | 0 | 0 | 0 | 0 | False positive/type 1 error/false alarm |
| FPR | 0.00078 | 0.11844 | 0.0 | 0.1632 | 0.08232 | 0.01179 | 0.03091 | 0.0 | 0.0 | 0.00317 | 0.0 | 0.01169 | 0.01793 | 0.00078 | 0.00394 | 0.00079 | 0.00624 | 0.02793 | 0.0 | 0.0 | 0.0 | 0.00078 | 0.08907 | 0.0 | 0.01443 | 0.0 | 0.00389 | 0.0 | 0.0 | 0.0 | 0.00079 | 0.0 | 0.00156 | 0.0 | 0.0 | 0.0 | 0.0 | 0.0 | 0.00725 | 0.00233 | 0.0 | 0.0 | 0.0 | 0.0 | 0.0 | 0.0 | 0.0 | 0.0 | 0.0 | 0.0 | 0.0 | 0.0 | Fall-out or false positive rate |
| G | 0.95652 | 0.18756 | None | 0.12873 | 0.67197 | 0.29848 | 0.67672 | None | 1.0 | 0.45835 | 0.90749 | 0.39886 | 0.87228 | 0.69398 | 0.61812 | 0.39284 | 0.77067 | 0.05812 | None | None | None | 0.50709 | 0.59243 | None | 0.09258 | 0.14003 | 0.8165 | None | None | None | 0.35082 | None | 0.70353 | None | 0.92582 | None | None | None | 0.5328 | 0.0 | None | None | None | None | None | None | None | None | None | None | 1.0 | None | G-measure geometric mean of precision and sensitivity |
| GI | 0.95574 | 0.26617 | 0.0 | 0.1134 | 0.73586 | 0.26821 | 0.66909 | 0.0 | 1.0 | 0.29095 | 0.82353 | 0.48831 | 0.98207 | 0.52095 | 0.51458 | 0.1844 | 0.92709 | 0.09707 | 0.0 | 0.0 | 0.0 | 0.29922 | 0.8948 | 0.0 | 0.04557 | 0.01961 | 0.99611 | 0.0 | 0.0 | 0.0 | 0.15306 | 0.0 | 0.63481 | 0.0 | 0.85714 | 0.0 | 0.0 | 0.0 | 0.39275 | -0.00233 | 0.0 | 0.0 | 0.0 | 0.0 | 0.0 | 0.0 | 0.0 | 0.0 | 0.0 | 0.0 | 1.0 | 0.0 | Gini index |
| GM | 0.97764 | 0.58229 | 0.0 | 0.4811 | 0.8665 | 0.52602 | 0.82363 | 0.0 | 1.0 | 0.54147 | 0.90749 | 0.70296 | 0.99099 | 0.72203 | 0.71866 | 0.43016 | 0.96307 | 0.34858 | 0.0 | 0.0 | 0.0 | 0.54751 | 0.9467 | 0.0 | 0.24317 | 0.14003 | 0.99806 | 0.0 | 0.0 | 0.0 | 0.39208 | 0.0 | 0.7971 | 0.0 | 0.92582 | 0.0 | 0.0 | 0.0 | 0.63016 | 0.0 | 0.0 | 0.0 | 0.0 | 0.0 | 0.0 | 0.0 | 0.0 | 0.0 | 0.0 | 0.0 | 1.0 | 0.0 | G-mean geometric mean of specificity and sensitivity |
| IBA | 0.91497 | 0.17057 | 0.0 | 0.10179 | 0.67612 | 0.08074 | 0.49582 | 0.0 | 1.0 | 0.08716 | 0.6782 | 0.25285 | 0.99968 | 0.27241 | 0.26984 | 0.03441 | 0.87146 | 0.01858 | 0.0 | 0.0 | 0.0 | 0.09016 | 0.96161 | 0.0 | 0.0044 | 0.00038 | 0.99998 | 0.0 | 0.0 | 0.0 | 0.02377 | 0.0 | 0.40532 | 0.0 | 0.73469 | 0.0 | 0.0 | 0.0 | 0.16172 | 0.0 | 0.0 | 0.0 | 0.0 | 0.0 | 0.0 | 0.0 | 0.0 | 0.0 | 0.0 | 0.0 | 1.0 | 0.0 | Index of balanced accuracy |
| ICSI | 0.91304 | -0.52392 | None | -0.6635 | 0.37007 | -0.40182 | 0.35421 | None | 1.0 | 0.0084 | 0.82353 | -0.18182 | 0.76087 | 0.44482 | 0.25536 | 0.01852 | 0.5697 | -0.84797 | None | None | None | 0.15714 | 0.3406 | None | -0.79714 | 0.01961 | 0.66667 | None | None | None | -0.04615 | None | 0.41414 | None | 0.85714 | None | None | None | 0.10968 | -1.0 | None | None | None | None | None | None | None | None | None | None | 1.0 | None | Individual classification success index |
| IS | 5.75327 | 1.6049 | None | 0.72526 | 2.32354 | 4.04503 | 3.08492 | None | 6.88153 | 4.76807 | 4.66854 | 4.88153 | 3.8174 | 5.70192 | 5.1455 | 5.32304 | 5.782 | 2.13151 | None | None | None | 5.79664 | 2.89965 | None | 1.88975 | 4.66854 | 6.43407 | None | None | None | 5.31859 | None | 6.51896 | None | 7.53361 | None | None | None | 4.06484 | None | None | None | None | None | None | None | None | None | None | None | 8.01903 | None | Information score |
| J | 0.91667 | 0.07979 | 0.0 | 0.05179 | 0.4916 | 0.175 | 0.51095 | 0.0 | 1.0 | 0.26316 | 0.82353 | 0.24138 | 0.76087 | 0.5 | 0.4375 | 0.17857 | 0.6087 | 0.02273 | 0.0 | 0.0 | 0.0 | 0.28571 | 0.35465 | 0.0 | 0.04412 | 0.01961 | 0.66667 | 0.0 | 0.0 | 0.0 | 0.14815 | 0.0 | 0.53846 | 0.0 | 0.85714 | 0.0 | 0.0 | 0.0 | 0.34375 | 0.0 | 0.0 | 0.0 | 0.0 | 0.0 | 0.0 | 0.0 | 0.0 | 0.0 | 0.0 | 0.0 | 1.0 | 0.0 | Jaccard index |
| LS | 53.93951 | 3.04174 | None | 1.6532 | 5.00557 | 16.50727 | 8.48505 | None | 117.90909 | 27.2479 | 25.43137 | 29.47727 | 14.09783 | 52.05351 | 35.39571 | 40.03086 | 55.02424 | 4.38176 | None | None | None | 55.58571 | 7.46246 | None | 3.70571 | 25.43137 | 86.46667 | None | None | None | 39.90769 | None | 91.70707 | None | 185.28571 | None | None | None | 16.73548 | 0.0 | None | None | None | None | None | None | None | None | None | None | 259.4 | None | Lift score |
| MCC | 0.95574 | 0.13677 | None | 0.05678 | 0.62328 | 0.28557 | 0.64873 | None | 1.0 | 0.44987 | 0.90423 | 0.39076 | 0.86442 | 0.69023 | 0.6115 | 0.38797 | 0.76762 | 0.04565 | None | None | None | 0.50321 | 0.56427 | None | 0.06951 | 0.1373 | 0.81491 | None | None | None | 0.34618 | None | 0.70126 | None | 0.92546 | None | None | None | 0.51816 | -0.00355 | None | None | None | None | None | None | None | None | None | None | 1.0 | None | Matthews correlation coefficient |
| MCCI | Very Strong | Negligible | None | Negligible | Moderate | Negligible | Moderate | None | Very Strong | Weak | Very Strong | Weak | Strong | Moderate | Moderate | Weak | Strong | Negligible | None | None | None | Moderate | Moderate | None | Negligible | Negligible | Strong | None | None | None | Weak | None | Strong | None | Very Strong | None | None | None | Moderate | Negligible | None | None | None | None | None | None | None | None | None | None | Very Strong | None | Matthews correlation coefficient interpretation |
| MCEN | 0.05726 | 0.64107 | 0.35701 | 0.7241 | 0.37774 | 0.37322 | 0.33585 | 0.0 | 0 | 0.3109 | 0.1162 | 0.34741 | 0.1589 | 0.13613 | 0.2791 | 0.15775 | 0.1766 | 0.5667 | 0.14766 | 0.30695 | 0.28793 | 0.17603 | 0.38307 | 0.19419 | 0.42513 | 0.38358 | 0.17519 | 0.25826 | 0.20664 | 0.39312 | 0.24426 | 0.1082 | 0.20985 | 0.1373 | 0.06011 | 0.1082 | 0.1082 | 0.09742 | 0.21199 | 0.34799 | 0.36695 | 0.13763 | 0.0 | 0.13763 | 0.2514 | 0.05564 | 0.23212 | 0.20547 | 0.29389 | 0.20664 | 0 | 0.17218 | Modified confusion entropy |
| MK | 0.95574 | 0.07028 | None | 0.02843 | 0.52792 | 0.30406 | 0.629 | None | 1.0 | 0.69558 | 0.99283 | 0.31269 | 0.76087 | 0.91451 | 0.72667 | 0.81629 | 0.63558 | 0.02147 | None | None | None | 0.84629 | 0.35584 | None | 0.10602 | 0.96142 | 0.66667 | None | None | None | 0.78297 | None | 0.77467 | None | 0.99923 | None | None | None | 0.68361 | -0.00541 | None | None | None | None | None | None | None | None | None | None | 1.0 | None | Markedness |
| N | 1274 | 1258 | 1261 | 1250 | 1154 | 1272 | 1197 | 1291 | 1286 | 1263 | 1246 | 1283 | 1227 | 1274 | 1270 | 1270 | 1282 | 1289 | 1290 | 1269 | 1281 | 1277 | 1235 | 1284 | 1247 | 1246 | 1287 | 1282 | 1290 | 1268 | 1271 | 1287 | 1286 | 1272 | 1290 | 1292 | 1292 | 1291 | 1242 | 1290 | 1256 | 1291 | 1289 | 1291 | 1281 | 1283 | 1289 | 1292 | 1287 | 1290 | 1292 | 1290 | Condition negative |
| NLR | 0.04351 | 0.69806 | 1.0 | 0.86449 | 0.19813 | 0.72859 | 0.30957 | 1.0 | 0.0 | 0.70813 | 0.17647 | 0.50591 | 0.0 | 0.47864 | 0.48338 | 0.81546 | 0.06709 | 0.90014 | 1.0 | 1.0 | 1.0 | 0.70055 | 0.01771 | 1.0 | 0.95377 | 0.98039 | 0.0 | 1.0 | 1.0 | 1.0 | 0.84682 | 1.0 | 0.3642 | 1.0 | 0.14286 | 1.0 | 1.0 | 1.0 | 0.60438 | 1.00233 | 1.0 | 1.0 | 1.0 | 1.0 | 1.0 | 1.0 | 1.0 | 1.0 | 1.0 | 1.0 | 0.0 | 1.0 | Negative likelihood ratio |
| NLRI | Good | Negligible | Negligible | Negligible | Fair | Negligible | Poor | Negligible | Good | Negligible | Fair | Negligible | Good | Poor | Poor | Negligible | Good | Negligible | Negligible | Negligible | Negligible | Negligible | Good | Negligible | Negligible | Negligible | Good | Negligible | Negligible | Negligible | Negligible | Negligible | Poor | Negligible | Fair | Negligible | Negligible | Negligible | Negligible | Negligible | Negligible | Negligible | Negligible | Negligible | Negligible | Negligible | Negligible | Negligible | Negligible | Negligible | Good | Negligible | Negative likelihood ratio interpretation |
| NPV | 0.99922 | 0.97882 | 0.97224 | 0.96852 | 0.97604 | 0.98588 | 0.97479 | 0.99537 | 1.0 | 0.98129 | 0.99283 | 0.99451 | 1.0 | 0.99143 | 0.98983 | 0.98296 | 0.99922 | 0.99444 | 0.9946 | 0.97841 | 0.98766 | 0.98915 | 0.99911 | 0.98998 | 0.96317 | 0.96142 | 1.0 | 0.98843 | 0.9946 | 0.97764 | 0.98297 | 0.99229 | 0.99689 | 0.98072 | 0.99923 | 0.99614 | 0.99614 | 0.99537 | 0.97393 | 0.99459 | 0.96839 | 0.99537 | 0.99383 | 0.99537 | 0.98766 | 0.98921 | 0.99383 | 0.99614 | 0.99229 | 0.9946 | 1.0 | 0.9946 | Negative predictive value |
| OC | 0.95652 | 0.38462 | None | 0.2766 | 0.81818 | 0.31818 | 0.7 | None | 1.0 | 0.71429 | 1.0 | 0.5 | 1.0 | 0.92308 | 0.73684 | 0.83333 | 0.93333 | 0.125 | None | None | None | 0.85714 | 0.98387 | None | 0.14286 | 1.0 | 1.0 | None | None | None | 0.8 | None | 0.77778 | None | 1.0 | None | None | None | 0.70968 | 0.0 | None | None | None | None | None | None | None | None | None | None | 1.0 | None | Overlap coefficient |
| OOC | 0.95652 | 0.18756 | None | 0.12873 | 0.67197 | 0.29848 | 0.67672 | None | 1.0 | 0.45835 | 0.90749 | 0.39886 | 0.87228 | 0.69398 | 0.61812 | 0.39284 | 0.77067 | 0.05812 | None | None | None | 0.50709 | 0.59243 | None | 0.09258 | 0.14003 | 0.8165 | None | None | None | 0.35082 | None | 0.70353 | None | 0.92582 | None | None | None | 0.5328 | 0.0 | None | None | None | None | None | None | None | None | None | None | 1.0 | None | Otsuka-Ochiai coefficient |
| OP | 0.97663 | 0.47414 | -0.02776 | 0.31335 | 0.84939 | 0.41612 | 0.78712 | -0.00463 | 1.0 | 0.43407 | 0.89629 | 0.65494 | 0.97399 | 0.67682 | 0.67082 | 0.29497 | 0.9617 | 0.19473 | -0.0054 | -0.02159 | -0.01234 | 0.45025 | 0.87592 | -0.01002 | 0.06465 | -9e-05 | 0.9942 | -0.01157 | -0.0054 | -0.02236 | 0.24912 | -0.00771 | 0.77389 | -0.01928 | 0.92231 | -0.00386 | -0.00386 | -0.00463 | 0.54202 | -0.00771 | -0.03161 | -0.00463 | -0.00617 | -0.00463 | -0.01234 | -0.01079 | -0.00617 | -0.00386 | -0.00771 | -0.0054 | 1.0 | -0.0054 | Optimized precision |
| P | 23 | 39 | 36 | 47 | 143 | 25 | 100 | 6 | 11 | 34 | 51 | 14 | 70 | 23 | 27 | 27 | 15 | 8 | 7 | 28 | 16 | 20 | 62 | 13 | 50 | 51 | 10 | 15 | 7 | 29 | 26 | 10 | 11 | 25 | 7 | 5 | 5 | 6 | 55 | 7 | 41 | 6 | 8 | 6 | 16 | 14 | 8 | 5 | 10 | 7 | 5 | 7 | Condition positive or support |
| PLR | 1218.6087 | 3.24729 | None | 1.69483 | 9.93876 | 23.744 | 22.64595 | None | None | 92.86765 | None | 42.76667 | 55.77273 | 664.69565 | 131.7037 | 235.18519 | 149.56667 | 4.47569 | None | None | None | 383.1 | 11.04619 | None | 4.15667 | None | 257.4 | None | None | None | 195.53846 | None | 409.18182 | None | None | None | None | None | 55.2 | 0.0 | None | None | None | None | None | None | None | None | None | None | None | None | Positive likelihood ratio |
| PLRI | Good | Poor | None | Poor | Fair | Good | Good | None | None | Good | None | Good | Good | Good | Good | Good | Good | Poor | None | None | None | Good | Good | None | Poor | None | Good | None | None | None | Good | None | Good | None | None | None | None | None | Good | Negligible | None | None | None | None | None | None | None | None | None | None | None | None | Positive likelihood ratio interpretation |
| POP | 1297 | 1297 | 1297 | 1297 | 1297 | 1297 | 1297 | 1297 | 1297 | 1297 | 1297 | 1297 | 1297 | 1297 | 1297 | 1297 | 1297 | 1297 | 1297 | 1297 | 1297 | 1297 | 1297 | 1297 | 1297 | 1297 | 1297 | 1297 | 1297 | 1297 | 1297 | 1297 | 1297 | 1297 | 1297 | 1297 | 1297 | 1297 | 1297 | 1297 | 1297 | 1297 | 1297 | 1297 | 1297 | 1297 | 1297 | 1297 | 1297 | 1297 | 1297 | 1297 | Population |
| PPV | 0.95652 | 0.09146 | None | 0.05991 | 0.55189 | 0.31818 | 0.65421 | None | 1.0 | 0.71429 | 1.0 | 0.31818 | 0.76087 | 0.92308 | 0.73684 | 0.83333 | 0.63636 | 0.02703 | None | None | None | 0.85714 | 0.35673 | None | 0.14286 | 1.0 | 0.66667 | None | None | None | 0.8 | None | 0.77778 | None | 1.0 | None | None | None | 0.70968 | 0.0 | None | None | None | None | None | None | None | None | None | None | 1.0 | None | Precision or positive predictive value |
| PRE | 0.01773 | 0.03007 | 0.02776 | 0.03624 | 0.11025 | 0.01928 | 0.0771 | 0.00463 | 0.00848 | 0.02621 | 0.03932 | 0.01079 | 0.05397 | 0.01773 | 0.02082 | 0.02082 | 0.01157 | 0.00617 | 0.0054 | 0.02159 | 0.01234 | 0.01542 | 0.0478 | 0.01002 | 0.03855 | 0.03932 | 0.00771 | 0.01157 | 0.0054 | 0.02236 | 0.02005 | 0.00771 | 0.00848 | 0.01928 | 0.0054 | 0.00386 | 0.00386 | 0.00463 | 0.04241 | 0.0054 | 0.03161 | 0.00463 | 0.00617 | 0.00463 | 0.01234 | 0.01079 | 0.00617 | 0.00386 | 0.00771 | 0.0054 | 0.00386 | 0.0054 | Prevalence |
| Q | 0.99993 | 0.64613 | None | 0.32444 | 0.96091 | 0.94046 | 0.97303 | None | None | 0.98487 | None | 0.97662 | None | 0.99856 | 0.99269 | 0.99309 | 0.9991 | 0.66512 | None | None | None | 0.99635 | 0.9968 | None | 0.62674 | None | None | None | None | None | 0.99138 | None | 0.99822 | None | None | None | None | None | 0.97834 | -1.0 | None | None | None | None | None | None | None | None | None | None | None | None | Yule Q - coefficient of colligation |
| QI | Strong | Moderate | None | Weak | Strong | Strong | Strong | None | None | Strong | None | Strong | None | Strong | Strong | Strong | Strong | Moderate | None | None | None | Strong | Strong | None | Moderate | None | None | None | None | None | Strong | None | Strong | None | None | None | None | None | Strong | Negligible | None | None | None | None | None | None | None | None | None | None | None | None | Yule Q interpretation |
| RACC | 0.00031 | 0.0038 | 0.0 | 0.00606 | 0.01802 | 0.00033 | 0.00636 | 0.0 | 7e-05 | 0.00028 | 0.00127 | 0.00018 | 0.00383 | 0.00018 | 0.0003 | 0.0001 | 0.0002 | 0.00018 | 0.0 | 0.0 | 0.0 | 8e-05 | 0.0063 | 0.0 | 0.00062 | 3e-05 | 9e-05 | 0.0 | 0.0 | 0.0 | 8e-05 | 0.0 | 6e-05 | 0.0 | 2e-05 | 0.0 | 0.0 | 0.0 | 0.00101 | 1e-05 | 0.0 | 0.0 | 0.0 | 0.0 | 0.0 | 0.0 | 0.0 | 0.0 | 0.0 | 0.0 | 1e-05 | 0.0 | Random accuracy |
| RACCU | 0.00031 | 0.00612 | 0.00019 | 0.01036 | 0.01873 | 0.00033 | 0.00637 | 1e-05 | 7e-05 | 0.00034 | 0.00129 | 0.00019 | 0.0039 | 0.00019 | 0.00031 | 0.00016 | 0.0002 | 0.0003 | 1e-05 | 0.00012 | 4e-05 | 0.00011 | 0.00807 | 3e-05 | 0.00075 | 0.0004 | 9e-05 | 3e-05 | 1e-05 | 0.00012 | 0.00014 | 1e-05 | 6e-05 | 9e-05 | 3e-05 | 0.0 | 0.0 | 1e-05 | 0.0011 | 1e-05 | 0.00025 | 1e-05 | 1e-05 | 1e-05 | 4e-05 | 3e-05 | 1e-05 | 0.0 | 1e-05 | 1e-05 | 1e-05 | 1e-05 | Random accuracy unbiased |
| TN | 1273 | 1109 | 1261 | 1046 | 1059 | 1257 | 1160 | 1291 | 1286 | 1259 | 1246 | 1268 | 1205 | 1273 | 1265 | 1269 | 1274 | 1253 | 1290 | 1269 | 1281 | 1276 | 1125 | 1284 | 1229 | 1246 | 1282 | 1282 | 1290 | 1268 | 1270 | 1287 | 1284 | 1272 | 1290 | 1292 | 1292 | 1291 | 1233 | 1287 | 1256 | 1291 | 1289 | 1291 | 1281 | 1283 | 1289 | 1292 | 1287 | 1290 | 1292 | 1290 | True negative/correct rejection |
| TNR | 0.99922 | 0.88156 | 1.0 | 0.8368 | 0.91768 | 0.98821 | 0.96909 | 1.0 | 1.0 | 0.99683 | 1.0 | 0.98831 | 0.98207 | 0.99922 | 0.99606 | 0.99921 | 0.99376 | 0.97207 | 1.0 | 1.0 | 1.0 | 0.99922 | 0.91093 | 1.0 | 0.98557 | 1.0 | 0.99611 | 1.0 | 1.0 | 1.0 | 0.99921 | 1.0 | 0.99844 | 1.0 | 1.0 | 1.0 | 1.0 | 1.0 | 0.99275 | 0.99767 | 1.0 | 1.0 | 1.0 | 1.0 | 1.0 | 1.0 | 1.0 | 1.0 | 1.0 | 1.0 | 1.0 | 1.0 | Specificity or true negative rate |
| TON | 1274 | 1133 | 1297 | 1080 | 1085 | 1275 | 1190 | 1297 | 1286 | 1283 | 1255 | 1275 | 1205 | 1284 | 1278 | 1291 | 1275 | 1260 | 1297 | 1297 | 1297 | 1290 | 1126 | 1297 | 1276 | 1296 | 1282 | 1297 | 1297 | 1297 | 1292 | 1297 | 1288 | 1297 | 1291 | 1297 | 1297 | 1297 | 1266 | 1294 | 1297 | 1297 | 1297 | 1297 | 1297 | 1297 | 1297 | 1297 | 1297 | 1297 | 1292 | 1297 | Test outcome negative |
| TOP | 23 | 164 | 0 | 217 | 212 | 22 | 107 | 0 | 11 | 14 | 42 | 22 | 92 | 13 | 19 | 6 | 22 | 37 | 0 | 0 | 0 | 7 | 171 | 0 | 21 | 1 | 15 | 0 | 0 | 0 | 5 | 0 | 9 | 0 | 6 | 0 | 0 | 0 | 31 | 3 | 0 | 0 | 0 | 0 | 0 | 0 | 0 | 0 | 0 | 0 | 5 | 0 | Test outcome positive |
| TP | 22 | 15 | 0 | 13 | 117 | 7 | 70 | 0 | 11 | 10 | 42 | 7 | 70 | 12 | 14 | 5 | 14 | 1 | 0 | 0 | 0 | 6 | 61 | 0 | 3 | 1 | 10 | 0 | 0 | 0 | 4 | 0 | 7 | 0 | 6 | 0 | 0 | 0 | 22 | 0 | 0 | 0 | 0 | 0 | 0 | 0 | 0 | 0 | 0 | 0 | 5 | 0 | True positive/hit |
| TPR | 0.95652 | 0.38462 | 0.0 | 0.2766 | 0.81818 | 0.28 | 0.7 | 0.0 | 1.0 | 0.29412 | 0.82353 | 0.5 | 1.0 | 0.52174 | 0.51852 | 0.18519 | 0.93333 | 0.125 | 0.0 | 0.0 | 0.0 | 0.3 | 0.98387 | 0.0 | 0.06 | 0.01961 | 1.0 | 0.0 | 0.0 | 0.0 | 0.15385 | 0.0 | 0.63636 | 0.0 | 0.85714 | 0.0 | 0.0 | 0.0 | 0.4 | 0.0 | 0.0 | 0.0 | 0.0 | 0.0 | 0.0 | 0.0 | 0.0 | 0.0 | 0.0 | 0.0 | 1.0 | 0.0 | Sensitivity, recall, hit rate, or true positive rate |
| Y | 0.95574 | 0.26617 | 0.0 | 0.1134 | 0.73586 | 0.26821 | 0.66909 | 0.0 | 1.0 | 0.29095 | 0.82353 | 0.48831 | 0.98207 | 0.52095 | 0.51458 | 0.1844 | 0.92709 | 0.09707 | 0.0 | 0.0 | 0.0 | 0.29922 | 0.8948 | 0.0 | 0.04557 | 0.01961 | 0.99611 | 0.0 | 0.0 | 0.0 | 0.15306 | 0.0 | 0.63481 | 0.0 | 0.85714 | 0.0 | 0.0 | 0.0 | 0.39275 | -0.00233 | 0.0 | 0.0 | 0.0 | 0.0 | 0.0 | 0.0 | 0.0 | 0.0 | 0.0 | 0.0 | 1.0 | 0.0 | Youden index |
| dInd | 0.04349 | 0.62668 | 1.0 | 0.74158 | 0.19959 | 0.7201 | 0.30159 | 1.0 | 0.0 | 0.70589 | 0.17647 | 0.50014 | 0.01793 | 0.47826 | 0.4815 | 0.81482 | 0.06696 | 0.87545 | 1.0 | 1.0 | 1.0 | 0.7 | 0.09052 | 1.0 | 0.94011 | 0.98039 | 0.00389 | 1.0 | 1.0 | 1.0 | 0.84615 | 1.0 | 0.36364 | 1.0 | 0.14286 | 1.0 | 1.0 | 1.0 | 0.60004 | 1.0 | 1.0 | 1.0 | 1.0 | 1.0 | 1.0 | 1.0 | 1.0 | 1.0 | 1.0 | 1.0 | 0.0 | 1.0 | Distance index |
| sInd | 0.96925 | 0.55687 | 0.29289 | 0.47562 | 0.85887 | 0.49081 | 0.78674 | 0.29289 | 1.0 | 0.50086 | 0.87522 | 0.64635 | 0.98732 | 0.66182 | 0.65953 | 0.42384 | 0.95265 | 0.38097 | 0.29289 | 0.29289 | 0.29289 | 0.50502 | 0.93599 | 0.29289 | 0.33524 | 0.30676 | 0.99725 | 0.29289 | 0.29289 | 0.29289 | 0.40168 | 0.29289 | 0.74287 | 0.29289 | 0.89898 | 0.29289 | 0.29289 | 0.29289 | 0.5757 | 0.29289 | 0.29289 | 0.29289 | 0.29289 | 0.29289 | 0.29289 | 0.29289 | 0.29289 | 0.29289 | 0.29289 | 0.29289 | 1.0 | 0.29289 | Similarity index |

Generated By PyCM Version 3.1
