# Supplementary material for: TopEC: prediction of Enzyme Commission classes by 3D graph neural networks and localized 3D protein descriptor
Source: Nat Commun. 2025 Mar 20;16:2737. doi: 10.1038/s41467-025-57324-5 (PMC11923149; doi:10.1038/s41467-025-57324-5)
Supplement: Supplementary file 3 — Supplementary Data 1 [file 41467_2025_57324_MOESM3_ESM.zip › Data_S1/table1/hierarchical/DeepFRI_FOLD_local.html]

PyCM Report


# PyCM Report

## Dataset Type :

- Multi-Class Classification
- Imbalanced

Note 1 : Recommended statistics for this type of classification highlighted in aqua

Note 2 : The recommender system assumes that the input is the result of classification over the whole data rather than just a part of it.
If the confusion matrix is the result of test data classification, the recommendation is not valid.

## Confusion Matrix :

|  |  |  |  |  |  |  |  |  |  |  |  |  |  |  |  |  |  |  |  |  |  |  |  |  |  |  |  |  |  |  |  |  |  |  |  |  |  |  |  |  |  |  |  |  |  |  |  |  |  |  |  |  |  |  |  |  |  |  |  |  |  |  |  |  |  |  |  |  |  |  |  |  |  |  |  |  |  |  |  |  |  |  |  |  |  |  |  |  |  |  |  |  |  |  |  |  |  |  |  |  |  |  |  |  |  |  |  |  |  |  |  |  |  |  |  |  |  |  |  |  |  |  |  |  |  |  |  |  |  |  |  |  |  |  |  |  |  |  |  |  |  |  |  |  |  |  |  |  |  |  |  |  |  |  |  |  |  |  |  |  |  |  |  |  |  |  |  |  |  |  |  |  |  |  |  |  |  |  |  |  |  |  |  |  |  |  |  |  |  |  |  |  |  |  |  |  |  |  |  |  |  |  |  |  |  |  |  |  |  |  |  |  |  |  |  |  |  |  |  |  |  |  |  |  |  |  |  |  |  |  |  |  |  |  |  |  |  |  |  |  |  |  |  |  |  |  |  |  |  |  |  |  |  |  |  |  |  |  |  |  |  |  |  |  |  |  |  |  |  |  |  |  |  |  |  |  |  |  |  |  |  |  |  |  |  |  |  |  |  |  |  |  |  |  |  |  |  |  |  |  |  |  |  |  |  |  |  |  |  |  |  |  |  |  |  |  |  |  |  |  |  |  |  |  |  |  |  |  |  |  |  |  |  |  |  |  |  |  |  |  |  |  |  |  |  |  |  |  |  |  |  |  |  |  |  |  |  |  |  |  |  |  |  |  |  |  |  |  |  |  |  |  |  |  |  |  |  |  |  |  |  |  |  |  |  |  |  |  |  |  |  |  |  |  |  |  |  |  |  |  |  |  |  |  |  |  |  |  |  |  |  |  |  |  |  |  |  |  |  |  |  |  |  |  |  |  |  |  |  |  |  |  |  |  |  |  |  |  |  |  |  |  |  |  |  |  |  |  |  |  |  |  |  |  |  |  |  |  |  |  |  |  |  |  |  |  |  |  |  |  |  |  |  |  |  |  |  |  |  |  |  |  |  |  |  |  |  |  |  |  |  |  |  |  |  |  |  |  |  |  |  |  |  |  |  |  |  |  |  |  |  |  |  |  |  |  |  |  |  |  |  |  |  |  |  |  |  |  |  |  |  |  |  |  |  |  |  |  |  |  |  |  |  |  |  |  |  |  |  |  |  |  |  |  |  |  |  |  |  |  |  |  |  |  |  |  |  |  |  |  |  |  |  |  |  |  |  |  |  |  |  |  |  |  |  |  |  |  |  |  |  |  |  |  |  |  |  |  |  |  |  |  |  |  |  |  |  |  |  |  |  |  |  |  |  |  |  |  |  |  |  |  |  |  |  |  |  |  |  |  |  |  |  |  |  |  |  |  |  |  |  |  |  |  |  |  |  |  |  |  |  |  |  |  |  |  |  |  |  |  |  |  |  |  |  |  |  |  |  |  |  |  |  |  |  |  |  |  |  |  |  |  |  |  |  |  |  |  |  |  |  |  |  |  |  |  |  |  |  |  |  |  |  |  |  |  |  |  |  |  |  |  |  |  |  |  |  |  |  |  |  |  |  |  |  |  |  |  |  |  |  |  |  |  |  |  |  |  |  |  |  |  |  |  |  |  |  |  |  |  |  |  |  |  |  |  |  |  |  |  |  |  |  |  |  |  |  |  |  |  |  |  |  |  |  |  |  |  |  |  |  |  |  |  |  |  |  |  |  |  |  |  |  |  |  |  |  |  |  |  |  |  |  |  |  |  |  |  |  |  |  |  |  |  |  |  |  |  |  |  |  |  |  |  |  |  |  |  |  |  |  |  |  |  |  |  |  |  |  |  |  |  |  |  |  |  |  |  |  |  |  |  |  |  |  |  |  |  |  |  |  |  |  |  |  |  |  |  |  |  |  |  |  |  |  |  |  |  |  |  |  |  |  |  |  |  |  |  |  |  |  |  |  |  |  |  |  |  |  |  |  |  |  |  |  |  |  |  |  |  |  |  |  |  |  |  |  |  |  |  |  |  |  |  |  |  |  |  |  |  |  |  |  |  |  |  |  |  |  |  |  |  |  |  |  |  |  |  |  |  |  |  |  |  |  |  |  |  |  |  |  |  |  |  |  |  |  |  |  |  |  |  |  |  |  |  |  |  |  |  |  |  |  |  |  |  |  |  |  |  |  |  |  |  |  |  |  |  |  |  |  |  |  |  |  |  |  |  |  |  |  |  |  |  |  |  |  |  |  |  |  |  |  |  |  |  |  |  |  |  |  |  |  |  |  |  |  |  |  |  |  |  |  |  |  |  |  |  |  |  |  |  |  |  |  |  |  |  |  |  |  |  |  |  |  |  |  |  |  |  |  |  |  |  |  |  |  |  |  |  |  |  |  |  |  |  |  |  |  |  |  |  |  |  |  |  |  |  |  |  |  |  |  |  |  |  |  |  |  |  |  |  |  |  |  |  |  |  |  |  |  |  |  |  |  |  |  |  |  |  |  |  |  |  |  |  |  |  |  |  |  |  |  |  |  |  |  |  |  |  |  |  |  |  |  |  |  |  |  |  |  |  |  |  |  |  |  |  |  |  |  |  |  |  |  |  |  |  |  |  |  |  |  |  |  |  |  |  |  |  |  |  |  |  |  |  |  |  |  |  |  |  |  |  |  |  |  |  |  |  |  |  |  |  |  |  |  |  |  |  |  |  |  |  |  |  |  |  |  |  |  |  |  |  |  |  |  |  |  |  |  |  |  |  |  |  |  |  |  |  |  |  |  |  |  |  |  |  |  |  |  |  |  |  |  |  |  |  |  |  |  |  |  |  |  |  |  |  |  |  |  |  |  |  |  |  |  |  |  |  |  |  |  |  |  |  |  |  |  |  |  |  |  |  |  |  |  |  |  |  |  |  |  |  |  |  |  |  |  |  |  |  |  |  |  |  |  |  |  |  |  |  |  |  |  |  |  |  |  |  |  |  |  |  |  |  |  |  |  |  |  |  |  |  |  |  |  |  |  |  |  |  |  |  |  |  |  |  |  |  |  |  |  |  |  |  |  |  |  |  |  |  |  |  |  |  |  |  |  |  |  |  |  |  |  |  |  |  |  |  |  |  |  |  |  |  |  |  |  |  |  |  |  |  |  |  |  |  |  |  |  |  |  |  |  |  |  |  |  |  |  |  |  |  |  |  |  |  |  |  |  |  |  |  |  |  |  |  |  |  |  |  |  |  |  |  |  |  |  |  |  |  |  |  |  |  |  |  |  |  |  |  |  |  |  |  |  |  |  |  |  |  |  |  |  |  |  |  |  |  |  |  |  |  |  |  |  |  |  |  |  |  |  |  |  |  |  |  |  |  |  |  |  |  |  |  |  |  |  |  |  |  |  |  |  |  |  |  |  |  |  |  |  |  |  |  |  |  |  |  |  |  |  |  |  |  |  |  |  |  |  |  |  |  |  |  |  |  |  |  |  |  |  |  |  |  |  |  |  |  |  |  |  |  |  |  |  |  |  |  |  |  |  |  |  |  |  |  |  |  |  |  |  |  |  |  |  |  |  |  |  |  |  |  |  |  |  |  |  |  |  |  |  |  |  |  |  |  |  |  |  |  |  |  |  |  |  |  |  |  |  |  |  |  |  |  |  |  |  |  |  |  |  |  |  |  |  |  |  |  |  |  |  |  |  |  |  |  |  |  |  |  |  |  |  |  |  |  |  |  |  |  |  |  |  |  |  |  |  |  |  |  |  |  |  |  |  |  |  |  |  |  |  |  |  |  |  |  |  |  |  |  |  |  |  |  |  |  |  |  |  |  |  |  |  |  |  |  |  |  |  |  |  |  |  |  |  |  |  |  |  |  |  |  |  |  |  |  |  |  |  |  |  |  |  |  |  |  |  |  |  |  |  |  |  |  |  |  |  |  |  |  |  |  |  |  |  |  |  |  |  |  |  |  |  |  |  |  |  |  |  |  |  |  |  |  |  |  |  |  |  |  |  |  |  |  |  |  |  |  |  |  |  |  |  |  |  |  |  |  |  |  |  |  |  |  |  |  |  |  |  |  |  |  |  |  |  |  |  |  |  |  |  |  |  |  |  |  |  |  |  |  |  |  |  |  |  |  |  |  |  |  |  |  |  |  |  |  |  |  |  |  |  |  |  |  |  |  |  |  |  |  |  |  |  |  |  |  |  |  |  |  |  |  |  |  |  |  |  |  |  |  |  |  |  |  |  |  |  |  |  |  |  |  |  |  |  |  |  |  |  |  |  |  |  |  |  |  |  |  |  |  |  |  |  |  |  |  |  |  |  |  |  |  |  |  |  |  |  |  |  |  |  |  |  |  |  |  |  |  |  |  |  |  |  |  |  |  |  |  |  |  |  |  |  |  |  |  |  |  |  |  |  |  |  |  |  |  |  |  |  |  |  |  |  |  |  |  |  |  |  |  |  |  |  |  |  |  |  |  |  |  |  |  |  |  |  |  |  |  |  |  |  |  |  |  |  |  |  |  |  |  |  |  |  |  |  |  |  |  |  |  |  |  |  |  |  |  |  |  |  |  |  |  |  |  |  |  |  |  |  |  |  |  |  |  |  |  |  |  |  |  |  |  |  |  |  |  |  |  |  |  |  |  |  |  |  |  |  |  |  |  |  |  |  |  |  |  |  |  |  |  |  |  |  |  |  |  |  |  |  |  |  |  |  |  |  |  |  |  |  |  |  |  |  |  |  |  |  |  |  |  |  |  |  |  |  |  |  |  |  |  |  |  |  |  |  |  |  |  |  |  |  |  |  |  |  |  |  |  |  |  |  |  |  |  |  |  |  |  |  |  |  |  |  |  |  |  |  |  |  |  |  |  |  |  |  |  |  |  |  |  |  |  |  |  |  |  |  |  |  |  |  |  |  |  |  |  |  |  |  |  |  |  |  |  |  |  |  |  |  |  |  |  |  |  |  |  |  |  |  |  |  |  |  |  |  |  |  |  |  |  |  |  |  |  |  |  |  |  |  |  |  |  |  |  |  |  |  |  |  |  |  |  |  |  |  |  |  |  |  |  |  |  |  |  |  |  |  |  |  |  |  |  |  |  |  |  |  |  |  |  |  |  |  |  |  |  |  |  |  |  |  |  |  |  |  |  |  |  |  |  |  |  |  |  |  |  |  |  |  |  |  |  |  |  |  |  |  |  |  |  |  |  |  |  |  |  |  |  |  |  |  |  |  |  |  |  |  |  |  |  |  |  |  |  |  |  |  |  |  |  |  |  |  |  |  |  |  |  |  |  |  |  |  |  |  |  |  |  |  |  |  |  |  |  |  |  |  |  |  |  |  |  |  |  |  |  |  |  |  |  |  |  |  |  |  |  |  |  |  |  |  |  |  |  |  |  |  |  |  |  |  |  |  |  |  |  |  |  |  |  |  |  |  |  |  |  |  |  |  |  |  |  |  |  |  |  |  |  |  |  |  |  |  |  |  |  |  |  |  |  |  |  |  |  |  |  |  |  |  |  |  |  |  |  |  |  |  |  |  |  |  |  |  |  |  |  |  |  |  |  |  |  |  |  |  |  |  |  |  |  |  |  |  |  |  |  |  |  |  |  |  |  |  |  |  |  |  |  |  |  |  |  |  |  |  |  |  |  |  |  |  |  |  |  |  |  |  |  |  |  |  |  |  |  |  |  |  |  |  |  |  |  |  |  |  |  |  |  |  |  |  |  |  |  |  |  |  |  |  |  |  |  |  |  |  |  |  |  |  |  |  |  |  |  |  |  |  |  |  |  |  |  |  |  |  |  |  |  |  |  |  |  |  |  |  |  |  |  |  |  |  |  |  |  |  |  |  |  |  |  |  |  |  |  |  |  |  |  |  |  |  |  |  |  |  |  |  |  |  |  |  |  |  |  |  |  |  |  |  |  |  |  |  |  |  |  |  |  |  |  |  |  |  |  |  |  |  |  |  |  |  |  |  |  |  |  |  |  |  |  |  |  |  |  |  |  |  |  |  |  |  |  |  |  |  |  |  |  |  |  |  |  |  |  |  |  |  |  |  |  |  |  |  |  |  |  |  |  |  |  |  |  |  |  |  |  |  |  |  |  |  |  |  |  |  |  |  |  |  |  |  |  |  |  |  |  |  |  |  |  |  |  |  |  |  |  |  |  |  |  |  |  |  |  |  |  |  |  |  |  |  |  |  |  |  |  |  |  |  |  |  |  |  |  |  |  |  |  |  |  |  |  |  |  |  |  |  |  |  |  |  |  |  |  |  |  |  |  |  |  |  |  |  |  |  |  |  |  |
| --- | --- | --- | --- | --- | --- | --- | --- | --- | --- | --- | --- | --- | --- | --- | --- | --- | --- | --- | --- | --- | --- | --- | --- | --- | --- | --- | --- | --- | --- | --- | --- | --- | --- | --- | --- | --- | --- | --- | --- | --- | --- | --- | --- | --- | --- | --- | --- | --- | --- | --- | --- | --- | --- | --- | --- | --- | --- | --- | --- | --- | --- | --- | --- | --- | --- | --- | --- | --- | --- | --- | --- | --- | --- | --- | --- | --- | --- | --- | --- | --- | --- | --- | --- | --- | --- | --- | --- | --- | --- | --- | --- | --- | --- | --- | --- | --- | --- | --- | --- | --- | --- | --- | --- | --- | --- | --- | --- | --- | --- | --- | --- | --- | --- | --- | --- | --- | --- | --- | --- | --- | --- | --- | --- | --- | --- | --- | --- | --- | --- | --- | --- | --- | --- | --- | --- | --- | --- | --- | --- | --- | --- | --- | --- | --- | --- | --- | --- | --- | --- | --- | --- | --- | --- | --- | --- | --- | --- | --- | --- | --- | --- | --- | --- | --- | --- | --- | --- | --- | --- | --- | --- | --- | --- | --- | --- | --- | --- | --- | --- | --- | --- | --- | --- | --- | --- | --- | --- | --- | --- | --- | --- | --- | --- | --- | --- | --- | --- | --- | --- | --- | --- | --- | --- | --- | --- | --- | --- | --- | --- | --- | --- | --- | --- | --- | --- | --- | --- | --- | --- | --- | --- | --- | --- | --- | --- | --- | --- | --- | --- | --- | --- | --- | --- | --- | --- | --- | --- | --- | --- | --- | --- | --- | --- | --- | --- | --- | --- | --- | --- | --- | --- | --- | --- | --- | --- | --- | --- | --- | --- | --- | --- | --- | --- | --- | --- | --- | --- | --- | --- | --- | --- | --- | --- | --- | --- | --- | --- | --- | --- | --- | --- | --- | --- | --- | --- | --- | --- | --- | --- | --- | --- | --- | --- | --- | --- | --- | --- | --- | --- | --- | --- | --- | --- | --- | --- | --- | --- | --- | --- | --- | --- | --- | --- | --- | --- | --- | --- | --- | --- | --- | --- | --- | --- | --- | --- | --- | --- | --- | --- | --- | --- | --- | --- | --- | --- | --- | --- | --- | --- | --- | --- | --- | --- | --- | --- | --- | --- | --- | --- | --- | --- | --- | --- | --- | --- | --- | --- | --- | --- | --- | --- | --- | --- | --- | --- | --- | --- | --- | --- | --- | --- | --- | --- | --- | --- | --- | --- | --- | --- | --- | --- | --- | --- | --- | --- | --- | --- | --- | --- | --- | --- | --- | --- | --- | --- | --- | --- | --- | --- | --- | --- | --- | --- | --- | --- | --- | --- | --- | --- | --- | --- | --- | --- | --- | --- | --- | --- | --- | --- | --- | --- | --- | --- | --- | --- | --- | --- | --- | --- | --- | --- | --- | --- | --- | --- | --- | --- | --- | --- | --- | --- | --- | --- | --- | --- | --- | --- | --- | --- | --- | --- | --- | --- | --- | --- | --- | --- | --- | --- | --- | --- | --- | --- | --- | --- | --- | --- | --- | --- | --- | --- | --- | --- | --- | --- | --- | --- | --- | --- | --- | --- | --- | --- | --- | --- | --- | --- | --- | --- | --- | --- | --- | --- | --- | --- | --- | --- | --- | --- | --- | --- | --- | --- | --- | --- | --- | --- | --- | --- | --- | --- | --- | --- | --- | --- | --- | --- | --- | --- | --- | --- | --- | --- | --- | --- | --- | --- | --- | --- | --- | --- | --- | --- | --- | --- | --- | --- | --- | --- | --- | --- | --- | --- | --- | --- | --- | --- | --- | --- | --- | --- | --- | --- | --- | --- | --- | --- | --- | --- | --- | --- | --- | --- | --- | --- | --- | --- | --- | --- | --- | --- | --- | --- | --- | --- | --- | --- | --- | --- | --- | --- | --- | --- | --- | --- | --- | --- | --- | --- | --- | --- | --- | --- | --- | --- | --- | --- | --- | --- | --- | --- | --- | --- | --- | --- | --- | --- | --- | --- | --- | --- | --- | --- | --- | --- | --- | --- | --- | --- | --- | --- | --- | --- | --- | --- | --- | --- | --- | --- | --- | --- | --- | --- | --- | --- | --- | --- | --- | --- | --- | --- | --- | --- | --- | --- | --- | --- | --- | --- | --- | --- | --- | --- | --- | --- | --- | --- | --- | --- | --- | --- | --- | --- | --- | --- | --- | --- | --- | --- | --- | --- | --- | --- | --- | --- | --- | --- | --- | --- | --- | --- | --- | --- | --- | --- | --- | --- | --- | --- | --- | --- | --- | --- | --- | --- | --- | --- | --- | --- | --- | --- | --- | --- | --- | --- | --- | --- | --- | --- | --- | --- | --- | --- | --- | --- | --- | --- | --- | --- | --- | --- | --- | --- | --- | --- | --- | --- | --- | --- | --- | --- | --- | --- | --- | --- | --- | --- | --- | --- | --- | --- | --- | --- | --- | --- | --- | --- | --- | --- | --- | --- | --- | --- | --- | --- | --- | --- | --- | --- | --- | --- | --- | --- | --- | --- | --- | --- | --- | --- | --- | --- | --- | --- | --- | --- | --- | --- | --- | --- | --- | --- | --- | --- | --- | --- | --- | --- | --- | --- | --- | --- | --- | --- | --- | --- | --- | --- | --- | --- | --- | --- | --- | --- | --- | --- | --- | --- | --- | --- | --- | --- | --- | --- | --- | --- | --- | --- | --- | --- | --- | --- | --- | --- | --- | --- | --- | --- | --- | --- | --- | --- | --- | --- | --- | --- | --- | --- | --- | --- | --- | --- | --- | --- | --- | --- | --- | --- | --- | --- | --- | --- | --- | --- | --- | --- | --- | --- | --- | --- | --- | --- | --- | --- | --- | --- | --- | --- | --- | --- | --- | --- | --- | --- | --- | --- | --- | --- | --- | --- | --- | --- | --- | --- | --- | --- | --- | --- | --- | --- | --- | --- | --- | --- | --- | --- | --- | --- | --- | --- | --- | --- | --- | --- | --- | --- | --- | --- | --- | --- | --- | --- | --- | --- | --- | --- | --- | --- | --- | --- | --- | --- | --- | --- | --- | --- | --- | --- | --- | --- | --- | --- | --- | --- | --- | --- | --- | --- | --- | --- | --- | --- | --- | --- | --- | --- | --- | --- | --- | --- | --- | --- | --- | --- | --- | --- | --- | --- | --- | --- | --- | --- | --- | --- | --- | --- | --- | --- | --- | --- | --- | --- | --- | --- | --- | --- | --- | --- | --- | --- | --- | --- | --- | --- | --- | --- | --- | --- | --- | --- | --- | --- | --- | --- | --- | --- | --- | --- | --- | --- | --- | --- | --- | --- | --- | --- | --- | --- | --- | --- | --- | --- | --- | --- | --- | --- | --- | --- | --- | --- | --- | --- | --- | --- | --- | --- | --- | --- | --- | --- | --- | --- | --- | --- | --- | --- | --- | --- | --- | --- | --- | --- | --- | --- | --- | --- | --- | --- | --- | --- | --- | --- | --- | --- | --- | --- | --- | --- | --- | --- | --- | --- | --- | --- | --- | --- | --- | --- | --- | --- | --- | --- | --- | --- | --- | --- | --- | --- | --- | --- | --- | --- | --- | --- | --- | --- | --- | --- | --- | --- | --- | --- | --- | --- | --- | --- | --- | --- | --- | --- | --- | --- | --- | --- | --- | --- | --- | --- | --- | --- | --- | --- | --- | --- | --- | --- | --- | --- | --- | --- | --- | --- | --- | --- | --- | --- | --- | --- | --- | --- | --- | --- | --- | --- | --- | --- | --- | --- | --- | --- | --- | --- | --- | --- | --- | --- | --- | --- | --- | --- | --- | --- | --- | --- | --- | --- | --- | --- | --- | --- | --- | --- | --- | --- | --- | --- | --- | --- | --- | --- | --- | --- | --- | --- | --- | --- | --- | --- | --- | --- | --- | --- | --- | --- | --- | --- | --- | --- | --- | --- | --- | --- | --- | --- | --- | --- | --- | --- | --- | --- | --- | --- | --- | --- | --- | --- | --- | --- | --- | --- | --- | --- | --- | --- | --- | --- | --- | --- | --- | --- | --- | --- | --- | --- | --- | --- | --- | --- | --- | --- | --- | --- | --- | --- | --- | --- | --- | --- | --- | --- | --- | --- | --- | --- | --- | --- | --- | --- | --- | --- | --- | --- | --- | --- | --- | --- | --- | --- | --- | --- | --- | --- | --- | --- | --- | --- | --- | --- | --- | --- | --- | --- | --- | --- | --- | --- | --- | --- | --- | --- | --- | --- | --- | --- | --- | --- | --- | --- | --- | --- | --- | --- | --- | --- | --- | --- | --- | --- | --- | --- | --- | --- | --- | --- | --- | --- | --- | --- | --- | --- | --- | --- | --- | --- | --- | --- | --- | --- | --- | --- | --- | --- | --- | --- | --- | --- | --- | --- | --- | --- | --- | --- | --- | --- | --- | --- | --- | --- | --- | --- | --- | --- | --- | --- | --- | --- | --- | --- | --- | --- | --- | --- | --- | --- | --- | --- | --- | --- | --- | --- | --- | --- | --- | --- | --- | --- | --- | --- | --- | --- | --- | --- | --- | --- | --- | --- | --- | --- | --- | --- | --- | --- | --- | --- | --- | --- | --- | --- | --- | --- | --- | --- | --- | --- | --- | --- | --- | --- | --- | --- | --- | --- | --- | --- | --- | --- | --- | --- | --- | --- | --- | --- | --- | --- | --- | --- | --- | --- | --- | --- | --- | --- | --- | --- | --- | --- | --- | --- | --- | --- | --- | --- | --- | --- | --- | --- | --- | --- | --- | --- | --- | --- | --- | --- | --- | --- | --- | --- | --- | --- | --- | --- | --- | --- | --- | --- | --- | --- | --- | --- | --- | --- | --- | --- | --- | --- | --- | --- | --- | --- | --- | --- | --- | --- | --- | --- | --- | --- | --- | --- | --- | --- | --- | --- | --- | --- | --- | --- | --- | --- | --- | --- | --- | --- | --- | --- | --- | --- | --- | --- | --- | --- | --- | --- | --- | --- | --- | --- | --- | --- | --- | --- | --- | --- | --- | --- | --- | --- | --- | --- | --- | --- | --- | --- | --- | --- | --- | --- | --- | --- | --- | --- | --- | --- | --- | --- | --- | --- | --- | --- | --- | --- | --- | --- | --- | --- | --- | --- | --- | --- | --- | --- | --- | --- | --- | --- | --- | --- | --- | --- | --- | --- | --- | --- | --- | --- | --- | --- | --- | --- | --- | --- | --- | --- | --- | --- | --- | --- | --- | --- | --- | --- | --- | --- | --- | --- | --- | --- | --- | --- | --- | --- | --- | --- | --- | --- | --- | --- | --- | --- | --- | --- | --- | --- | --- | --- | --- | --- | --- | --- | --- | --- | --- | --- | --- | --- | --- | --- | --- | --- | --- | --- | --- | --- | --- | --- | --- | --- | --- | --- | --- | --- | --- | --- | --- | --- | --- | --- | --- | --- | --- | --- | --- | --- | --- | --- | --- | --- | --- | --- | --- | --- | --- | --- | --- | --- | --- | --- | --- | --- | --- | --- | --- | --- | --- | --- | --- | --- | --- | --- | --- | --- | --- | --- | --- | --- | --- | --- | --- | --- | --- | --- | --- | --- | --- | --- | --- | --- | --- | --- | --- | --- | --- | --- | --- | --- | --- | --- | --- | --- | --- | --- | --- | --- | --- | --- | --- | --- | --- | --- | --- | --- | --- | --- | --- | --- | --- | --- | --- | --- | --- | --- | --- | --- | --- | --- | --- | --- | --- | --- | --- | --- | --- | --- | --- | --- | --- | --- | --- | --- | --- | --- | --- | --- | --- | --- | --- | --- | --- | --- | --- | --- | --- | --- | --- | --- | --- | --- | --- | --- | --- | --- | --- | --- | --- | --- | --- | --- | --- | --- | --- | --- | --- | --- | --- | --- | --- | --- | --- | --- | --- | --- | --- | --- | --- | --- | --- | --- | --- | --- | --- | --- | --- | --- | --- | --- | --- | --- | --- | --- | --- | --- | --- | --- | --- | --- | --- | --- | --- | --- | --- | --- | --- | --- | --- | --- | --- | --- | --- | --- | --- | --- | --- | --- | --- | --- | --- | --- | --- | --- | --- | --- | --- | --- | --- | --- | --- | --- | --- | --- | --- | --- | --- | --- | --- | --- | --- | --- | --- | --- | --- | --- | --- | --- | --- | --- | --- | --- | --- | --- | --- | --- | --- | --- | --- | --- | --- | --- | --- | --- | --- | --- | --- | --- | --- | --- | --- | --- | --- | --- | --- | --- | --- | --- | --- | --- | --- | --- | --- | --- | --- | --- | --- | --- | --- | --- | --- | --- | --- | --- | --- | --- | --- | --- | --- | --- | --- | --- | --- | --- | --- | --- | --- | --- | --- | --- | --- | --- | --- | --- | --- | --- | --- | --- | --- | --- | --- | --- | --- | --- | --- | --- | --- | --- | --- | --- | --- | --- | --- | --- | --- | --- | --- | --- | --- | --- | --- | --- | --- | --- | --- | --- | --- | --- | --- | --- | --- | --- | --- | --- | --- | --- | --- | --- | --- | --- | --- | --- | --- | --- | --- | --- | --- | --- | --- | --- | --- | --- | --- | --- | --- | --- | --- | --- | --- | --- | --- | --- | --- | --- | --- | --- | --- | --- | --- | --- | --- | --- | --- | --- | --- | --- | --- | --- | --- | --- | --- | --- | --- | --- | --- | --- | --- | --- | --- | --- | --- | --- | --- | --- | --- | --- | --- | --- | --- | --- | --- | --- | --- | --- | --- | --- | --- | --- | --- | --- | --- | --- | --- | --- | --- | --- | --- | --- | --- | --- | --- | --- | --- | --- | --- | --- | --- | --- | --- | --- | --- | --- | --- | --- | --- | --- | --- | --- | --- | --- | --- | --- | --- | --- | --- | --- | --- | --- | --- | --- | --- | --- | --- | --- | --- | --- | --- | --- | --- | --- | --- | --- | --- | --- | --- | --- | --- | --- | --- | --- | --- | --- | --- | --- | --- | --- | --- | --- | --- | --- | --- | --- | --- | --- | --- | --- | --- | --- | --- | --- | --- | --- | --- | --- | --- | --- | --- | --- | --- | --- | --- | --- | --- | --- | --- | --- | --- | --- | --- | --- | --- | --- | --- | --- | --- | --- | --- | --- | --- | --- | --- | --- | --- | --- | --- | --- | --- | --- | --- | --- | --- | --- | --- | --- | --- | --- | --- | --- | --- | --- | --- | --- | --- | --- | --- | --- | --- | --- | --- | --- | --- | --- | --- | --- | --- | --- | --- | --- | --- | --- | --- | --- | --- | --- | --- | --- | --- | --- | --- | --- | --- | --- | --- | --- | --- | --- | --- | --- | --- | --- | --- | --- | --- | --- | --- | --- | --- | --- | --- | --- | --- | --- | --- | --- | --- | --- | --- | --- | --- | --- | --- | --- | --- | --- | --- | --- | --- | --- | --- | --- | --- | --- | --- | --- | --- | --- | --- | --- | --- | --- | --- | --- | --- | --- | --- | --- | --- | --- | --- | --- | --- | --- | --- | --- | --- | --- | --- | --- | --- | --- | --- | --- | --- | --- | --- | --- | --- | --- | --- | --- | --- | --- | --- | --- | --- | --- | --- | --- | --- | --- | --- | --- | --- | --- | --- | --- | --- | --- | --- | --- | --- | --- | --- | --- | --- | --- | --- | --- | --- | --- | --- | --- | --- | --- | --- | --- | --- | --- | --- | --- | --- | --- | --- | --- | --- | --- | --- | --- | --- | --- | --- | --- | --- | --- | --- | --- | --- | --- | --- | --- | --- | --- | --- | --- | --- | --- | --- | --- | --- | --- | --- | --- | --- | --- | --- | --- | --- | --- | --- | --- | --- | --- | --- | --- | --- | --- | --- | --- | --- | --- | --- | --- | --- | --- | --- | --- | --- | --- | --- | --- | --- | --- | --- | --- | --- | --- | --- | --- | --- | --- | --- | --- | --- | --- | --- | --- | --- | --- | --- | --- | --- | --- | --- | --- | --- | --- | --- | --- | --- | --- | --- | --- | --- | --- | --- | --- | --- | --- | --- | --- | --- | --- | --- | --- | --- | --- | --- | --- | --- | --- | --- | --- | --- | --- | --- | --- | --- | --- | --- | --- | --- | --- | --- | --- | --- | --- | --- | --- | --- | --- | --- | --- | --- | --- | --- | --- | --- | --- | --- | --- | --- | --- | --- | --- | --- | --- | --- | --- | --- | --- | --- | --- | --- | --- | --- | --- | --- | --- | --- | --- | --- | --- | --- | --- | --- | --- | --- | --- | --- | --- | --- | --- | --- | --- | --- | --- | --- | --- | --- | --- | --- | --- | --- | --- | --- | --- | --- | --- | --- | --- | --- | --- | --- | --- | --- | --- | --- | --- | --- | --- | --- | --- | --- | --- | --- | --- | --- | --- | --- | --- | --- | --- | --- | --- | --- | --- | --- | --- | --- | --- | --- | --- | --- | --- | --- | --- | --- | --- | --- | --- | --- | --- | --- | --- | --- | --- | --- | --- | --- | --- | --- | --- | --- | --- | --- | --- | --- | --- | --- | --- | --- | --- | --- | --- | --- | --- | --- | --- | --- | --- | --- | --- | --- | --- | --- | --- | --- | --- | --- | --- | --- | --- | --- | --- | --- | --- | --- | --- | --- | --- | --- | --- | --- | --- | --- | --- | --- | --- | --- | --- | --- | --- | --- | --- | --- | --- | --- | --- | --- | --- | --- | --- | --- | --- | --- | --- | --- | --- | --- | --- | --- | --- | --- | --- | --- | --- | --- | --- | --- | --- | --- | --- | --- | --- | --- | --- | --- | --- | --- | --- | --- | --- | --- | --- | --- | --- | --- | --- | --- | --- | --- | --- | --- | --- | --- | --- | --- | --- | --- | --- | --- | --- | --- | --- | --- | --- | --- | --- | --- | --- | --- | --- | --- | --- | --- | --- | --- | --- | --- | --- | --- | --- | --- | --- | --- | --- | --- | --- | --- | --- | --- | --- | --- | --- | --- | --- | --- | --- | --- | --- | --- | --- | --- | --- | --- | --- | --- | --- | --- | --- | --- | --- | --- | --- | --- | --- | --- | --- | --- | --- | --- | --- | --- | --- | --- | --- | --- | --- | --- | --- | --- | --- | --- | --- | --- | --- | --- | --- | --- | --- | --- | --- | --- | --- | --- | --- | --- | --- | --- | --- | --- | --- | --- | --- | --- | --- | --- | --- | --- | --- | --- | --- | --- | --- | --- | --- | --- | --- | --- | --- | --- | --- | --- | --- | --- | --- | --- | --- | --- | --- | --- | --- | --- | --- | --- | --- | --- | --- | --- | --- | --- | --- | --- | --- | --- | --- | --- | --- | --- | --- | --- | --- | --- | --- | --- |
| Actual | Predict  |  |  |  |  |  |  |  |  |  |  |  |  |  |  |  |  |  |  |  |  |  |  |  |  |  |  |  |  |  |  |  |  |  |  |  |  |  |  |  |  |  |  |  |  |  |  |  |  |  |  |  |  |  | | --- | --- | --- | --- | --- | --- | --- | --- | --- | --- | --- | --- | --- | --- | --- | --- | --- | --- | --- | --- | --- | --- | --- | --- | --- | --- | --- | --- | --- | --- | --- | --- | --- | --- | --- | --- | --- | --- | --- | --- | --- | --- | --- | --- | --- | --- | --- | --- | --- | --- | --- | --- | --- | |  | 8 | 9 | 11 | 12 | 20 | 27 | 33 | 34 | 42 | 43 | 53 | 58 | 81 | 82 | 86 | 93 | 96 | 108 | 127 | 131 | 133 | 136 | 138 | 140 | 143 | 144 | 145 | 148 | 158 | 160 | 161 | 163 | 166 | 176 | 181 | 182 | 190 | 195 | 200 | 203 | 213 | 214 | 215 | 216 | 222 | 225 | 227 | 228 | 236 | 240 | 271 | 274 | | 8 | 3 | 0 | 0 | 0 | 2 | 0 | 0 | 0 | 0 | 0 | 0 | 0 | 0 | 0 | 0 | 0 | 0 | 0 | 0 | 0 | 0 | 0 | 0 | 0 | 0 | 0 | 0 | 0 | 0 | 0 | 0 | 0 | 0 | 0 | 0 | 0 | 0 | 1 | 0 | 0 | 0 | 0 | 0 | 0 | 0 | 0 | 0 | 0 | 0 | 0 | 0 | 0 | | 9 | 0 | 0 | 0 | 0 | 0 | 0 | 0 | 0 | 0 | 0 | 0 | 0 | 0 | 0 | 0 | 0 | 0 | 0 | 0 | 0 | 0 | 0 | 0 | 0 | 0 | 0 | 0 | 0 | 0 | 0 | 0 | 0 | 0 | 0 | 0 | 10 | 0 | 0 | 0 | 0 | 0 | 0 | 0 | 0 | 0 | 0 | 0 | 0 | 0 | 0 | 0 | 0 | | 11 | 0 | 0 | 5 | 0 | 0 | 1 | 0 | 0 | 0 | 0 | 0 | 0 | 0 | 0 | 0 | 0 | 0 | 0 | 0 | 0 | 0 | 0 | 0 | 0 | 0 | 0 | 0 | 0 | 0 | 0 | 0 | 0 | 0 | 0 | 0 | 17 | 0 | 0 | 0 | 0 | 0 | 0 | 0 | 0 | 0 | 0 | 0 | 0 | 0 | 0 | 0 | 0 | | 12 | 0 | 0 | 0 | 0 | 0 | 0 | 0 | 3 | 0 | 0 | 0 | 0 | 0 | 0 | 0 | 0 | 0 | 0 | 0 | 0 | 0 | 0 | 0 | 0 | 0 | 0 | 0 | 0 | 0 | 0 | 0 | 0 | 0 | 0 | 0 | 4 | 0 | 0 | 0 | 18 | 0 | 0 | 0 | 2 | 0 | 0 | 0 | 0 | 0 | 0 | 0 | 0 | | 20 | 0 | 0 | 0 | 0 | 0 | 8 | 0 | 0 | 0 | 0 | 0 | 0 | 0 | 1 | 0 | 0 | 0 | 0 | 0 | 0 | 0 | 0 | 0 | 0 | 0 | 0 | 0 | 0 | 0 | 0 | 0 | 0 | 0 | 0 | 0 | 0 | 0 | 0 | 0 | 0 | 0 | 0 | 2 | 0 | 0 | 0 | 0 | 0 | 0 | 0 | 0 | 0 | | 27 | 0 | 0 | 0 | 0 | 0 | 20 | 0 | 0 | 0 | 1 | 0 | 0 | 0 | 0 | 0 | 0 | 0 | 0 | 0 | 0 | 0 | 0 | 0 | 0 | 0 | 0 | 0 | 0 | 0 | 0 | 0 | 6 | 0 | 0 | 0 | 0 | 0 | 0 | 0 | 0 | 0 | 0 | 0 | 0 | 0 | 0 | 0 | 0 | 0 | 0 | 0 | 0 | | 33 | 0 | 0 | 0 | 0 | 0 | 0 | 0 | 0 | 0 | 0 | 0 | 0 | 0 | 0 | 0 | 1 | 0 | 0 | 0 | 0 | 0 | 0 | 0 | 0 | 0 | 0 | 0 | 0 | 0 | 0 | 0 | 0 | 0 | 0 | 1 | 0 | 0 | 1 | 0 | 0 | 0 | 0 | 0 | 0 | 0 | 0 | 0 | 0 | 0 | 0 | 0 | 0 | | 34 | 2 | 0 | 0 | 0 | 0 | 0 | 0 | 18 | 0 | 0 | 0 | 0 | 0 | 0 | 0 | 0 | 0 | 0 | 0 | 0 | 0 | 2 | 0 | 0 | 0 | 0 | 0 | 0 | 0 | 0 | 0 | 0 | 0 | 0 | 0 | 0 | 0 | 0 | 0 | 0 | 0 | 0 | 0 | 0 | 0 | 0 | 0 | 0 | 0 | 0 | 0 | 0 | | 42 | 0 | 0 | 0 | 0 | 1 | 2 | 0 | 2 | 0 | 0 | 0 | 0 | 0 | 0 | 0 | 0 | 0 | 0 | 0 | 0 | 1 | 3 | 0 | 0 | 0 | 0 | 0 | 0 | 0 | 0 | 0 | 0 | 0 | 0 | 0 | 0 | 0 | 0 | 0 | 0 | 0 | 0 | 0 | 0 | 0 | 0 | 0 | 0 | 0 | 0 | 0 | 0 | | 43 | 0 | 0 | 0 | 0 | 0 | 0 | 0 | 0 | 0 | 26 | 5 | 0 | 0 | 0 | 0 | 0 | 0 | 0 | 0 | 0 | 0 | 0 | 0 | 0 | 0 | 0 | 0 | 0 | 0 | 0 | 0 | 0 | 0 | 0 | 0 | 0 | 0 | 0 | 0 | 0 | 0 | 0 | 0 | 0 | 0 | 0 | 0 | 0 | 0 | 0 | 0 | 0 | | 53 | 0 | 0 | 0 | 0 | 0 | 18 | 0 | 3 | 4 | 0 | 11 | 0 | 0 | 0 | 0 | 0 | 0 | 0 | 0 | 0 | 0 | 0 | 0 | 0 | 0 | 0 | 0 | 0 | 0 | 0 | 0 | 0 | 0 | 0 | 0 | 0 | 0 | 0 | 0 | 0 | 0 | 0 | 0 | 0 | 0 | 0 | 0 | 0 | 0 | 0 | 0 | 0 | | 58 | 0 | 0 | 5 | 0 | 0 | 0 | 0 | 3 | 0 | 0 | 0 | 0 | 0 | 0 | 0 | 0 | 0 | 0 | 0 | 0 | 0 | 0 | 0 | 0 | 0 | 0 | 0 | 0 | 0 | 0 | 0 | 0 | 0 | 0 | 0 | 0 | 0 | 0 | 0 | 0 | 0 | 0 | 0 | 0 | 0 | 0 | 0 | 0 | 0 | 0 | 0 | 0 | | 81 | 0 | 0 | 0 | 0 | 0 | 0 | 0 | 69 | 0 | 0 | 0 | 0 | 27 | 0 | 0 | 0 | 0 | 0 | 0 | 0 | 0 | 8 | 0 | 0 | 0 | 0 | 0 | 0 | 0 | 0 | 0 | 0 | 0 | 0 | 0 | 0 | 0 | 0 | 0 | 0 | 0 | 0 | 0 | 0 | 0 | 0 | 0 | 0 | 0 | 0 | 0 | 0 | | 82 | 0 | 0 | 0 | 0 | 0 | 0 | 0 | 4 | 0 | 0 | 0 | 0 | 0 | 0 | 0 | 0 | 0 | 0 | 0 | 0 | 0 | 0 | 0 | 0 | 0 | 0 | 0 | 0 | 0 | 0 | 0 | 0 | 0 | 0 | 0 | 0 | 0 | 0 | 0 | 0 | 0 | 0 | 0 | 0 | 0 | 0 | 0 | 0 | 0 | 0 | 0 | 0 | | 86 | 0 | 0 | 13 | 0 | 0 | 0 | 0 | 0 | 1 | 0 | 1 | 0 | 0 | 0 | 0 | 0 | 0 | 0 | 0 | 0 | 0 | 0 | 0 | 0 | 0 | 0 | 0 | 0 | 0 | 0 | 0 | 0 | 0 | 0 | 0 | 0 | 0 | 0 | 0 | 0 | 0 | 0 | 0 | 0 | 0 | 0 | 0 | 0 | 0 | 0 | 0 | 0 | | 93 | 0 | 0 | 0 | 0 | 0 | 0 | 0 | 0 | 0 | 0 | 0 | 0 | 0 | 0 | 0 | 8 | 0 | 0 | 0 | 0 | 0 | 0 | 0 | 0 | 0 | 0 | 0 | 0 | 0 | 0 | 0 | 0 | 0 | 0 | 0 | 0 | 0 | 0 | 0 | 0 | 0 | 0 | 0 | 0 | 0 | 0 | 0 | 0 | 0 | 0 | 0 | 0 | | 96 | 0 | 0 | 0 | 0 | 0 | 0 | 0 | 0 | 0 | 9 | 0 | 0 | 0 | 0 | 0 | 0 | 0 | 0 | 0 | 0 | 0 | 0 | 0 | 0 | 0 | 0 | 0 | 0 | 0 | 0 | 0 | 0 | 0 | 0 | 0 | 0 | 0 | 0 | 0 | 0 | 0 | 0 | 0 | 0 | 0 | 0 | 0 | 0 | 0 | 0 | 0 | 0 | | 108 | 0 | 0 | 0 | 0 | 0 | 0 | 0 | 12 | 0 | 0 | 0 | 0 | 3 | 0 | 0 | 0 | 0 | 1 | 0 | 0 | 0 | 0 | 0 | 0 | 0 | 0 | 0 | 0 | 0 | 0 | 0 | 0 | 0 | 0 | 0 | 0 | 0 | 1 | 0 | 0 | 0 | 0 | 0 | 0 | 0 | 0 | 0 | 0 | 0 | 0 | 0 | 0 | | 127 | 0 | 0 | 0 | 0 | 0 | 41 | 0 | 1 | 0 | 1 | 0 | 0 | 0 | 0 | 0 | 0 | 0 | 0 | 28 | 0 | 66 | 0 | 0 | 0 | 0 | 0 | 0 | 0 | 0 | 0 | 0 | 0 | 0 | 0 | 0 | 0 | 0 | 0 | 0 | 0 | 0 | 0 | 4 | 0 | 0 | 0 | 0 | 0 | 0 | 0 | 0 | 0 | | 131 | 0 | 0 | 0 | 0 | 0 | 0 | 0 | 0 | 0 | 0 | 0 | 0 | 0 | 0 | 0 | 0 | 0 | 0 | 0 | 0 | 0 | 0 | 0 | 0 | 0 | 0 | 0 | 0 | 0 | 0 | 0 | 0 | 0 | 0 | 0 | 0 | 0 | 23 | 0 | 0 | 0 | 0 | 0 | 0 | 0 | 0 | 0 | 0 | 0 | 0 | 0 | 0 | | 133 | 0 | 0 | 0 | 0 | 0 | 33 | 0 | 0 | 0 | 0 | 0 | 0 | 0 | 0 | 0 | 17 | 0 | 0 | 0 | 0 | 0 | 0 | 0 | 0 | 0 | 0 | 0 | 0 | 0 | 0 | 0 | 0 | 0 | 0 | 3 | 0 | 0 | 0 | 0 | 0 | 0 | 0 | 0 | 0 | 0 | 0 | 0 | 0 | 0 | 0 | 0 | 0 | | 136 | 0 | 0 | 3 | 0 | 0 | 0 | 0 | 10 | 0 | 0 | 1 | 0 | 0 | 0 | 0 | 0 | 0 | 0 | 0 | 0 | 0 | 15 | 0 | 0 | 0 | 0 | 0 | 0 | 0 | 0 | 0 | 0 | 0 | 0 | 0 | 0 | 0 | 0 | 0 | 0 | 0 | 0 | 0 | 0 | 0 | 0 | 0 | 0 | 0 | 0 | 0 | 0 | | 138 | 0 | 0 | 0 | 0 | 0 | 7 | 0 | 3 | 0 | 0 | 0 | 0 | 0 | 0 | 0 | 2 | 0 | 0 | 0 | 0 | 0 | 1 | 2 | 0 | 0 | 0 | 0 | 0 | 0 | 0 | 0 | 0 | 0 | 0 | 0 | 0 | 0 | 0 | 0 | 0 | 0 | 0 | 0 | 0 | 0 | 0 | 0 | 0 | 0 | 0 | 0 | 0 | | 140 | 0 | 0 | 0 | 0 | 0 | 0 | 0 | 4 | 0 | 0 | 0 | 0 | 0 | 1 | 0 | 0 | 0 | 0 | 0 | 0 | 0 | 0 | 0 | 0 | 0 | 0 | 0 | 0 | 0 | 0 | 0 | 0 | 0 | 0 | 0 | 0 | 0 | 0 | 0 | 0 | 0 | 0 | 0 | 0 | 0 | 0 | 0 | 0 | 0 | 0 | 0 | 0 | | 143 | 0 | 0 | 1 | 0 | 0 | 0 | 0 | 1 | 2 | 0 | 0 | 0 | 0 | 0 | 0 | 0 | 0 | 0 | 0 | 0 | 0 | 0 | 0 | 0 | 0 | 0 | 0 | 0 | 0 | 0 | 0 | 0 | 0 | 0 | 0 | 0 | 0 | 0 | 0 | 0 | 0 | 0 | 0 | 0 | 0 | 0 | 0 | 0 | 0 | 0 | 0 | 0 | | 144 | 0 | 0 | 0 | 0 | 0 | 0 | 0 | 1 | 0 | 0 | 0 | 0 | 0 | 0 | 0 | 0 | 0 | 0 | 0 | 0 | 0 | 0 | 1 | 0 | 0 | 0 | 0 | 0 | 0 | 0 | 0 | 0 | 0 | 0 | 0 | 0 | 0 | 0 | 0 | 0 | 0 | 0 | 1 | 0 | 0 | 0 | 0 | 0 | 0 | 0 | 0 | 0 | | 145 | 0 | 0 | 0 | 0 | 0 | 0 | 0 | 1 | 0 | 0 | 0 | 0 | 0 | 0 | 0 | 0 | 0 | 0 | 0 | 0 | 0 | 0 | 0 | 0 | 0 | 0 | 3 | 0 | 0 | 0 | 0 | 0 | 0 | 0 | 0 | 0 | 0 | 0 | 0 | 0 | 0 | 0 | 0 | 0 | 0 | 0 | 0 | 0 | 0 | 0 | 0 | 0 | | 148 | 0 | 0 | 0 | 0 | 0 | 3 | 0 | 0 | 0 | 0 | 0 | 0 | 0 | 0 | 0 | 0 | 0 | 0 | 0 | 0 | 0 | 0 | 0 | 0 | 0 | 0 | 0 | 0 | 0 | 0 | 0 | 0 | 0 | 0 | 0 | 2 | 0 | 0 | 2 | 0 | 0 | 0 | 0 | 0 | 0 | 0 | 0 | 0 | 0 | 0 | 0 | 0 | | 158 | 0 | 0 | 0 | 0 | 0 | 0 | 0 | 12 | 0 | 0 | 0 | 0 | 0 | 0 | 0 | 0 | 0 | 0 | 0 | 0 | 0 | 2 | 0 | 0 | 0 | 0 | 0 | 0 | 0 | 0 | 0 | 0 | 0 | 0 | 0 | 1 | 0 | 0 | 0 | 0 | 0 | 0 | 0 | 0 | 0 | 0 | 0 | 0 | 0 | 0 | 0 | 0 | | 160 | 0 | 0 | 0 | 0 | 0 | 0 | 0 | 1 | 0 | 0 | 0 | 0 | 0 | 0 | 0 | 0 | 0 | 0 | 0 | 0 | 0 | 0 | 0 | 0 | 0 | 0 | 0 | 0 | 0 | 0 | 0 | 0 | 0 | 0 | 0 | 6 | 0 | 0 | 0 | 0 | 0 | 0 | 0 | 0 | 0 | 0 | 0 | 0 | 0 | 0 | 0 | 0 | | 161 | 0 | 0 | 0 | 0 | 0 | 0 | 0 | 7 | 0 | 0 | 0 | 0 | 0 | 0 | 0 | 0 | 0 | 0 | 0 | 0 | 0 | 0 | 0 | 0 | 0 | 0 | 0 | 0 | 0 | 0 | 0 | 0 | 0 | 0 | 0 | 0 | 0 | 0 | 0 | 0 | 0 | 0 | 0 | 0 | 0 | 0 | 0 | 0 | 0 | 0 | 0 | 0 | | 163 | 0 | 0 | 2 | 0 | 0 | 0 | 0 | 2 | 0 | 3 | 0 | 0 | 0 | 0 | 0 | 0 | 0 | 0 | 7 | 0 | 0 | 0 | 1 | 0 | 0 | 0 | 0 | 0 | 0 | 0 | 0 | 6 | 0 | 0 | 0 | 0 | 0 | 0 | 0 | 0 | 0 | 0 | 0 | 0 | 0 | 0 | 0 | 0 | 0 | 0 | 0 | 0 | | 166 | 0 | 0 | 0 | 0 | 0 | 0 | 0 | 0 | 0 | 0 | 0 | 0 | 0 | 0 | 0 | 0 | 0 | 0 | 0 | 0 | 0 | 0 | 0 | 0 | 0 | 0 | 0 | 0 | 0 | 0 | 0 | 0 | 7 | 0 | 0 | 0 | 0 | 0 | 0 | 0 | 0 | 0 | 0 | 0 | 0 | 0 | 0 | 0 | 0 | 0 | 0 | 0 | | 176 | 0 | 0 | 0 | 0 | 0 | 0 | 0 | 0 | 0 | 0 | 1 | 0 | 0 | 0 | 0 | 0 | 0 | 0 | 0 | 0 | 0 | 0 | 0 | 0 | 0 | 0 | 0 | 0 | 0 | 0 | 0 | 0 | 0 | 2 | 0 | 3 | 0 | 0 | 0 | 0 | 0 | 0 | 0 | 0 | 0 | 1 | 0 | 0 | 0 | 0 | 0 | 0 | | 181 | 0 | 0 | 0 | 0 | 0 | 1 | 0 | 0 | 0 | 0 | 0 | 0 | 0 | 0 | 0 | 0 | 0 | 0 | 0 | 0 | 0 | 0 | 0 | 0 | 0 | 0 | 0 | 0 | 0 | 0 | 0 | 0 | 0 | 0 | 8 | 0 | 0 | 0 | 0 | 0 | 0 | 0 | 0 | 0 | 0 | 0 | 0 | 0 | 0 | 0 | 0 | 0 | | 182 | 0 | 0 | 1 | 0 | 0 | 2 | 0 | 23 | 0 | 1 | 0 | 0 | 0 | 0 | 0 | 0 | 0 | 0 | 0 | 0 | 0 | 0 | 0 | 0 | 0 | 0 | 0 | 0 | 0 | 0 | 0 | 0 | 0 | 0 | 0 | 47 | 0 | 0 | 0 | 0 | 0 | 0 | 0 | 0 | 0 | 0 | 0 | 0 | 0 | 0 | 0 | 0 | | 190 | 0 | 0 | 0 | 0 | 0 | 0 | 0 | 0 | 0 | 0 | 0 | 0 | 0 | 0 | 0 | 0 | 0 | 0 | 0 | 0 | 0 | 0 | 0 | 0 | 0 | 0 | 0 | 0 | 0 | 0 | 0 | 0 | 0 | 0 | 0 | 0 | 0 | 10 | 0 | 0 | 0 | 0 | 0 | 0 | 0 | 0 | 0 | 0 | 0 | 0 | 0 | 0 | | 195 | 0 | 0 | 6 | 0 | 0 | 8 | 0 | 0 | 0 | 0 | 0 | 0 | 0 | 0 | 0 | 0 | 0 | 0 | 0 | 0 | 1 | 0 | 0 | 0 | 0 | 0 | 0 | 0 | 0 | 0 | 0 | 0 | 0 | 0 | 1 | 0 | 0 | 8 | 0 | 0 | 0 | 0 | 0 | 0 | 0 | 0 | 0 | 0 | 0 | 0 | 0 | 0 | | 200 | 0 | 0 | 0 | 0 | 0 | 0 | 0 | 0 | 0 | 4 | 0 | 0 | 0 | 0 | 0 | 0 | 0 | 0 | 0 | 0 | 0 | 0 | 0 | 0 | 0 | 0 | 0 | 0 | 0 | 0 | 0 | 0 | 0 | 0 | 0 | 0 | 0 | 0 | 0 | 0 | 0 | 0 | 2 | 0 | 0 | 0 | 0 | 0 | 0 | 0 | 0 | 0 | | 203 | 0 | 0 | 0 | 0 | 0 | 0 | 0 | 6 | 0 | 0 | 0 | 0 | 0 | 0 | 0 | 0 | 0 | 0 | 0 | 0 | 0 | 0 | 0 | 0 | 0 | 0 | 0 | 0 | 0 | 0 | 0 | 0 | 0 | 0 | 0 | 0 | 0 | 0 | 0 | 0 | 0 | 0 | 0 | 0 | 0 | 0 | 0 | 0 | 0 | 0 | 0 | 0 | | 213 | 0 | 0 | 0 | 0 | 0 | 11 | 0 | 10 | 0 | 0 | 0 | 0 | 0 | 0 | 0 | 0 | 0 | 0 | 3 | 0 | 0 | 0 | 0 | 0 | 0 | 0 | 0 | 0 | 0 | 0 | 0 | 0 | 0 | 0 | 0 | 0 | 0 | 0 | 0 | 0 | 0 | 0 | 0 | 0 | 0 | 0 | 0 | 0 | 0 | 0 | 0 | 0 | | 214 | 0 | 0 | 0 | 0 | 0 | 0 | 0 | 3 | 0 | 0 | 0 | 0 | 0 | 0 | 0 | 0 | 0 | 0 | 0 | 0 | 0 | 0 | 0 | 0 | 0 | 0 | 0 | 0 | 0 | 0 | 0 | 0 | 0 | 0 | 0 | 0 | 0 | 0 | 0 | 0 | 0 | 1 | 0 | 0 | 0 | 0 | 0 | 0 | 0 | 0 | 0 | 0 | | 215 | 0 | 0 | 0 | 0 | 0 | 12 | 0 | 8 | 0 | 0 | 1 | 0 | 17 | 1 | 0 | 0 | 0 | 0 | 0 | 0 | 1 | 0 | 0 | 0 | 0 | 0 | 0 | 0 | 0 | 0 | 0 | 0 | 0 | 2 | 0 | 0 | 0 | 0 | 0 | 0 | 0 | 0 | 1 | 0 | 0 | 0 | 0 | 0 | 0 | 0 | 0 | 0 | | 216 | 0 | 0 | 0 | 0 | 0 | 0 | 0 | 0 | 0 | 0 | 0 | 0 | 0 | 0 | 0 | 0 | 0 | 0 | 0 | 0 | 0 | 0 | 0 | 0 | 0 | 0 | 0 | 0 | 0 | 0 | 0 | 0 | 0 | 0 | 0 | 0 | 0 | 18 | 0 | 0 | 0 | 0 | 0 | 0 | 0 | 0 | 0 | 0 | 0 | 0 | 0 | 0 | | 222 | 0 | 0 | 0 | 0 | 0 | 10 | 0 | 0 | 0 | 0 | 0 | 0 | 0 | 0 | 0 | 0 | 0 | 0 | 0 | 0 | 0 | 0 | 0 | 0 | 0 | 0 | 0 | 0 | 0 | 0 | 0 | 0 | 0 | 0 | 0 | 0 | 0 | 0 | 0 | 0 | 0 | 0 | 0 | 0 | 0 | 0 | 0 | 0 | 0 | 0 | 0 | 0 | | 225 | 0 | 0 | 0 | 0 | 0 | 0 | 0 | 0 | 0 | 0 | 0 | 0 | 0 | 0 | 0 | 0 | 0 | 0 | 0 | 0 | 0 | 0 | 0 | 0 | 0 | 0 | 0 | 0 | 0 | 0 | 0 | 0 | 0 | 0 | 0 | 0 | 0 | 0 | 0 | 0 | 0 | 0 | 1 | 0 | 0 | 6 | 0 | 0 | 0 | 0 | 0 | 0 | | 227 | 0 | 0 | 0 | 0 | 0 | 0 | 0 | 0 | 0 | 0 | 0 | 0 | 0 | 0 | 0 | 0 | 0 | 0 | 0 | 0 | 0 | 0 | 0 | 0 | 0 | 0 | 0 | 0 | 0 | 0 | 0 | 0 | 0 | 2 | 0 | 0 | 0 | 0 | 0 | 0 | 0 | 0 | 0 | 0 | 0 | 0 | 0 | 0 | 0 | 0 | 0 | 0 | | 228 | 0 | 0 | 0 | 0 | 0 | 0 | 0 | 2 | 0 | 0 | 0 | 0 | 0 | 2 | 0 | 0 | 0 | 0 | 0 | 0 | 0 | 2 | 0 | 0 | 0 | 0 | 0 | 0 | 0 | 0 | 0 | 0 | 0 | 0 | 0 | 0 | 0 | 0 | 0 | 0 | 0 | 1 | 0 | 0 | 0 | 0 | 0 | 0 | 0 | 0 | 0 | 0 | | 236 | 0 | 0 | 3 | 0 | 0 | 0 | 0 | 3 | 0 | 0 | 0 | 0 | 0 | 0 | 0 | 0 | 0 | 0 | 0 | 0 | 0 | 0 | 0 | 0 | 0 | 0 | 0 | 0 | 0 | 0 | 0 | 0 | 0 | 0 | 0 | 0 | 0 | 0 | 0 | 0 | 0 | 0 | 0 | 0 | 0 | 0 | 0 | 0 | 0 | 0 | 0 | 0 | | 240 | 0 | 0 | 3 | 0 | 0 | 0 | 0 | 0 | 0 | 0 | 0 | 0 | 0 | 0 | 0 | 0 | 0 | 0 | 22 | 0 | 0 | 2 | 0 | 0 | 0 | 0 | 0 | 0 | 1 | 0 | 0 | 0 | 0 | 0 | 0 | 1 | 0 | 0 | 0 | 0 | 0 | 0 | 0 | 0 | 0 | 0 | 0 | 0 | 0 | 0 | 0 | 0 | | 271 | 0 | 0 | 0 | 0 | 0 | 3 | 0 | 0 | 0 | 1 | 3 | 0 | 0 | 0 | 0 | 0 | 0 | 0 | 0 | 0 | 0 | 0 | 0 | 0 | 0 | 0 | 0 | 0 | 0 | 0 | 0 | 0 | 0 | 0 | 0 | 0 | 0 | 0 | 11 | 0 | 0 | 0 | 0 | 0 | 0 | 0 | 0 | 0 | 0 | 0 | 0 | 0 | | 274 | 0 | 0 | 0 | 0 | 0 | 7 | 0 | 0 | 0 | 0 | 0 | 0 | 0 | 0 | 0 | 0 | 0 | 0 | 0 | 0 | 0 | 0 | 0 | 0 | 0 | 0 | 0 | 0 | 0 | 0 | 0 | 0 | 0 | 0 | 0 | 4 | 0 | 0 | 0 | 0 | 0 | 0 | 0 | 0 | 0 | 0 | 0 | 0 | 0 | 0 | 0 | 0 | |

## Overall Statistics :

|  |  |
| --- | --- |
| 95% CI | (0.2202,0.273) |
| ACC Macro | 0.971 |
| ARI | 0.2279 |
| AUNP | 0.6094 |
| AUNU | 0.5973 |
| Bangdiwala B | 0.1418 |
| Bennett S | 0.2318 |
| CBA | 0.1488 |
| CSI | None |
| Chi-Squared | None |
| Chi-Squared DF | 2601 |
| Conditional Entropy | 1.2322 |
| Cramer V | None |
| Cross Entropy | 3.8436 |
| F1 Macro | 0.1766 |
| F1 Micro | 0.2466 |
| FNR Macro | 0.7904 |
| FNR Micro | 0.7534 |
| FPR Macro | 0.015 |
| FPR Micro | 0.0148 |
| Gwet AC1 | 0.2324 |
| Hamming Loss | 0.7534 |
| Joint Entropy | 6.2095 |
| KL Divergence | None |
| Kappa | 0.2159 |
| Kappa 95% CI | (0.1885,0.2434) |
| Kappa No Prevalence | -0.5068 |
| Kappa Standard Error | 0.014 |
| Kappa Unbiased | 0.1999 |
| Krippendorff Alpha | 0.2003 |
| Lambda A | 0.3672 |
| Lambda B | 0.5455 |
| Mutual Information | 2.5814 |
| NIR | 0.1374 |
| Overall ACC | 0.2466 |
| Overall CEN | 0.2888 |
| Overall J | (6.7138,0.1291) |
| Overall MCC | 0.2251 |
| Overall MCEN | 0.3105 |
| Overall RACC | 0.0391 |
| Overall RACCU | 0.0584 |
| P-Value | 0.0 |
| PPV Macro | None |
| PPV Micro | 0.2466 |
| Pearson C | None |
| Phi-Squared | None |
| RCI | 0.5186 |
| RR | 19.7308 |
| Reference Entropy | 4.9773 |
| Response Entropy | 3.8136 |
| SOA1(Landis & Koch) | Fair |
| SOA2(Fleiss) | Poor |
| SOA3(Altman) | Fair |
| SOA4(Cicchetti) | Poor |
| SOA5(Cramer) | None |
| SOA6(Matthews) | Negligible |
| Scott PI | 0.1999 |
| Standard Error | 0.0135 |
| TNR Macro | 0.985 |
| TNR Micro | 0.9852 |
| TPR Macro | 0.2096 |
| TPR Micro | 0.2466 |
| Zero-one Loss | 773 |

## Class Statistics :

|  |  |  |  |  |  |  |  |  |  |  |  |  |  |  |  |  |  |  |  |  |  |  |  |  |  |  |  |  |  |  |  |  |  |  |  |  |  |  |  |  |  |  |  |  |  |  |  |  |  |  |  |  |  |
| --- | --- | --- | --- | --- | --- | --- | --- | --- | --- | --- | --- | --- | --- | --- | --- | --- | --- | --- | --- | --- | --- | --- | --- | --- | --- | --- | --- | --- | --- | --- | --- | --- | --- | --- | --- | --- | --- | --- | --- | --- | --- | --- | --- | --- | --- | --- | --- | --- | --- | --- | --- | --- | --- |
| Class | 8 | 9 | 11 | 12 | 20 | 27 | 33 | 34 | 42 | 43 | 53 | 58 | 81 | 82 | 86 | 93 | 96 | 108 | 127 | 131 | 133 | 136 | 138 | 140 | 143 | 144 | 145 | 148 | 158 | 160 | 161 | 163 | 166 | 176 | 181 | 182 | 190 | 195 | 200 | 203 | 213 | 214 | 215 | 216 | 222 | 225 | 227 | 228 | 236 | 240 | 271 | 274 | Description |
| ACC | 0.9951 | 0.9903 | 0.9464 | 0.9737 | 0.9864 | 0.8304 | 0.9971 | 0.807 | 0.9844 | 0.9756 | 0.9639 | 0.9922 | 0.9055 | 0.9912 | 0.9854 | 0.9805 | 0.9912 | 0.9844 | 0.8587 | 0.9776 | 0.8811 | 0.9669 | 0.9854 | 0.9951 | 0.9961 | 0.9971 | 0.999 | 0.9932 | 0.9844 | 0.9932 | 0.9932 | 0.9795 | 1.0 | 0.9912 | 0.9942 | 0.9269 | 0.9903 | 0.9318 | 0.9815 | 0.9766 | 0.9766 | 0.9961 | 0.9493 | 0.9805 | 0.9903 | 0.9981 | 0.9981 | 0.9932 | 0.9942 | 0.9717 | 0.9825 | 0.9893 | Accuracy |
| AGF | 0.7182 | 0.0 | 0.4271 | 0.0 | 0.0 | 0.569 | 0.0 | 0.5341 | 0.0 | 0.8709 | 0.5674 | 0.0 | 0.5214 | 0.0 | 0.0 | 0.8149 | 0.0 | 0.2675 | 0.4489 | 0.0 | 0.0 | 0.6993 | 0.3932 | 0.0 | 0.0 | 0.0 | 0.8882 | 0.0 | 0.0 | 0.0 | 0.0 | 0.5554 | 1.0 | 0.541 | 0.9027 | 0.7622 | 0.0 | 0.497 | 0.0 | 0.0 | 0.0 | 0.5264 | 0.1624 | 0.0 | 0.0 | 0.9254 | 0.0 | 0.0 | 0.0 | 0.0 | 0.0 | 0.0 | Adjusted F-score |
| AGM | 0.8518 | 0 | 0.7075 | 0 | 0 | 0.8088 | 0 | 0.8096 | 0 | 0.9427 | 0.7647 | 0 | 0.7285 | 0 | 0 | 0.9853 | 0 | 0.6181 | 0.6813 | 0 | 0 | 0.844 | 0.6791 | 0 | 0 | 0 | 0.9329 | 0 | 0 | 0 | 0 | 0.7611 | 1.0 | 0.764 | 0.9677 | 0.8599 | 0 | 0.7516 | 0 | 0 | 0 | 0.7489 | 0.5618 | 0 | 0 | 0.9621 | 0 | 0 | 0 | 0 | 0 | 0 | Adjusted geometric mean |
| AM | -1 | -10 | 19 | -27 | -8 | 160 | -3 | 190 | -2 | 15 | -13 | -8 | -57 | 1 | -15 | 20 | -9 | -16 | -81 | -23 | 16 | 6 | -11 | -5 | -4 | -3 | -1 | -7 | -14 | -7 | -7 | -9 | 0 | -1 | 4 | 21 | -10 | 38 | 7 | 12 | -24 | -2 | -32 | -16 | -10 | 0 | -2 | -7 | -6 | -29 | -18 | -11 | Difference between automatic and manual classification |
| AUC | 0.749 | 0.5 | 0.5903 | 0.5 | 0.4985 | 0.7868 | 0.5 | 0.8125 | 0.4966 | 0.9093 | 0.6467 | 0.5 | 0.619 | 0.4976 | 0.5 | 0.9902 | 0.5 | 0.5294 | 0.5812 | 0.5 | 0.4645 | 0.7486 | 0.5657 | 0.5 | 0.5 | 0.5 | 0.875 | 0.5 | 0.4995 | 0.5 | 0.5 | 0.6399 | 1.0 | 0.6409 | 0.942 | 0.7924 | 0.5 | 0.6397 | 0.4936 | 0.4912 | 0.5 | 0.6245 | 0.5065 | 0.499 | 0.5 | 0.9281 | 0.5 | 0.5 | 0.5 | 0.5 | 0.5 | 0.5 | Area under the ROC curve |
| AUCI | Good | Poor | Poor | Poor | Poor | Good | Poor | Very Good | Poor | Excellent | Fair | Poor | Fair | Poor | Poor | Excellent | Poor | Poor | Poor | Poor | Poor | Good | Poor | Poor | Poor | Poor | Very Good | Poor | Poor | Poor | Poor | Fair | Excellent | Fair | Excellent | Good | Poor | Fair | Poor | Poor | Poor | Fair | Poor | Poor | Poor | Excellent | Poor | Poor | Poor | Poor | Poor | Poor | AUC value interpretation |
| AUPR | 0.55 | None | 0.1682 | None | 0.0 | 0.4238 | None | 0.4515 | 0.0 | 0.702 | 0.3919 | None | 0.417 | 0.0 | None | 0.6429 | None | 0.5294 | 0.3326 | None | 0.0 | 0.4729 | 0.3167 | None | None | None | 0.875 | None | 0.0 | None | None | 0.3929 | 1.0 | 0.3095 | 0.7521 | 0.5649 | None | 0.2312 | 0.0 | 0.0 | None | 0.375 | 0.0571 | 0.0 | None | 0.8571 | None | None | None | None | None | None | Area under the PR curve |
| BCD | 0.0005 | 0.0049 | 0.0093 | 0.0132 | 0.0039 | 0.078 | 0.0015 | 0.0926 | 0.001 | 0.0073 | 0.0063 | 0.0039 | 0.0278 | 0.0005 | 0.0073 | 0.0097 | 0.0044 | 0.0078 | 0.0395 | 0.0112 | 0.0078 | 0.0029 | 0.0054 | 0.0024 | 0.0019 | 0.0015 | 0.0005 | 0.0034 | 0.0068 | 0.0034 | 0.0034 | 0.0044 | 0.0 | 0.0005 | 0.0019 | 0.0102 | 0.0049 | 0.0185 | 0.0034 | 0.0058 | 0.0117 | 0.001 | 0.0156 | 0.0078 | 0.0049 | 0.0 | 0.001 | 0.0034 | 0.0029 | 0.0141 | 0.0088 | 0.0054 | Bray-Curtis dissimilarity |
| BM | 0.498 | 0.0 | 0.1805 | 0.0 | -0.003 | 0.5736 | 0.0 | 0.625 | -0.0069 | 0.8186 | 0.2934 | 0.0 | 0.2379 | -0.0049 | 0.0 | 0.9804 | 0.0 | 0.0588 | 0.1624 | 0.0 | -0.0709 | 0.4972 | 0.1314 | 0.0 | 0.0 | 0.0 | 0.75 | 0.0 | -0.001 | 0.0 | 0.0 | 0.2797 | 1.0 | 0.2818 | 0.884 | 0.5847 | 0.0 | 0.2794 | -0.0127 | -0.0176 | 0.0 | 0.249 | 0.0131 | -0.002 | 0.0 | 0.8562 | 0.0 | 0.0 | 0.0 | 0.0 | 0.0 | 0.0 | Informedness or bookmaker informedness |
| CEN | 0.1812 | 0.0 | 0.3926 | 0.2141 | 0.2709 | 0.4589 | 0.2375 | 0.491 | 0.4238 | 0.202 | 0.2883 | 0.143 | 0.181 | 0.3086 | 0.1049 | 0.1329 | 0.0 | 0.1577 | 0.2686 | 0.0 | 0.253 | 0.3027 | 0.2943 | 0.1082 | 0.2248 | 0.2375 | 0.0601 | 0.2333 | 0.1778 | 0.0887 | 0.0 | 0.2816 | 0 | 0.283 | 0.1499 | 0.2732 | 0.0 | 0.3451 | 0.2418 | 0.1216 | 0.2124 | 0.1395 | 0.4128 | 0.0703 | 0.0 | 0.0815 | 0.0 | 0.2923 | 0.1499 | 0.1861 | 0.2289 | 0.1417 | Confusion entropy |
| DOR | 509.0 | None | 7.2523 | None | 0.0 | 14.2344 | None | 18.7887 | 0.0 | 253.5 | 35.86 | None | 15.8143 | 0.0 | None | None | None | None | 6.6051 | None | 0.0 | 52.3393 | 77.6154 | None | None | None | None | None | 0.0 | None | None | 66.6 | None | 101.5 | 1619.2 | 32.784 | None | 8.7778 | 0.0 | 0.0 | None | 340.3333 | 2.3167 | 0.0 | None | 6108.0 | None | None | None | None | None | None | Diagnostic odds ratio |
| DP | 1.4923 | None | 0.4744 | None | None | 0.6359 | None | 0.7023 | None | 1.3254 | 0.8571 | None | 0.6611 | None | None | None | None | None | 0.452 | None | None | 0.9476 | 1.042 | None | None | None | None | None | None | None | None | 1.0053 | None | 1.1062 | 1.7694 | 0.8356 | None | 0.5201 | None | None | None | 1.3959 | 0.2012 | None | None | 2.0873 | None | None | None | None | None | None | Discriminant power |
| DPI | Limited | None | Poor | None | None | Poor | None | Poor | None | Limited | Poor | None | Poor | None | None | None | None | None | Poor | None | None | Poor | Limited | None | None | None | None | None | None | None | None | Limited | None | Limited | Limited | Poor | None | Poor | None | None | None | Limited | Poor | None | None | Fair | None | None | None | None | None | None | Discriminant power interpretation |
| ERR | 0.0049 | 0.0097 | 0.0536 | 0.0263 | 0.0136 | 0.1696 | 0.0029 | 0.193 | 0.0156 | 0.0244 | 0.0361 | 0.0078 | 0.0945 | 0.0088 | 0.0146 | 0.0195 | 0.0088 | 0.0156 | 0.1413 | 0.0224 | 0.1189 | 0.0331 | 0.0146 | 0.0049 | 0.0039 | 0.0029 | 0.001 | 0.0068 | 0.0156 | 0.0068 | 0.0068 | 0.0205 | 0.0 | 0.0088 | 0.0058 | 0.0731 | 0.0097 | 0.0682 | 0.0185 | 0.0234 | 0.0234 | 0.0039 | 0.0507 | 0.0195 | 0.0097 | 0.0019 | 0.0019 | 0.0068 | 0.0058 | 0.0283 | 0.0175 | 0.0107 | Error rate |
| F0.5 | 0.5769 | 0.0 | 0.1309 | 0.0 | 0.0 | 0.129 | 0.0 | 0.1034 | 0.0 | 0.6047 | 0.4297 | 0.0 | 0.4623 | 0.0 | 0.0 | 0.3333 | 0.0 | 0.2381 | 0.3675 | 0.0 | 0.0 | 0.4438 | 0.3226 | 0.0 | 0.0 | 0.0 | 0.9375 | 0.0 | 0.0 | 0.0 | 0.0 | 0.4348 | 1.0 | 0.3226 | 0.6557 | 0.5176 | 0.0 | 0.1471 | 0.0 | 0.0 | 0.0 | 0.4167 | 0.0575 | 0.0 | 0.0 | 0.8571 | 0.0 | 0.0 | 0.0 | 0.0 | 0.0 | 0.0 | F0.5 score |
| F1 | 0.5455 | 0.0 | 0.1538 | 0.0 | 0.0 | 0.1869 | 0.0 | 0.1538 | 0.0 | 0.6753 | 0.3729 | 0.0 | 0.3576 | 0.0 | 0.0 | 0.4444 | 0.0 | 0.1111 | 0.2786 | 0.0 | 0.0 | 0.4688 | 0.2105 | 0.0 | 0.0 | 0.0 | 0.8571 | 0.0 | 0.0 | 0.0 | 0.0 | 0.3636 | 1.0 | 0.3077 | 0.7273 | 0.5562 | 0.0 | 0.186 | 0.0 | 0.0 | 0.0 | 0.3333 | 0.037 | 0.0 | 0.0 | 0.8571 | 0.0 | 0.0 | 0.0 | 0.0 | 0.0 | 0.0 | F1 score - harmonic mean of precision and sensitivity |
| F2 | 0.5172 | 0.0 | 0.1866 | 0.0 | 0.0 | 0.339 | 0.0 | 0.3 | 0.0 | 0.7647 | 0.3293 | 0.0 | 0.2916 | 0.0 | 0.0 | 0.6667 | 0.0 | 0.0725 | 0.2244 | 0.0 | 0.0 | 0.4967 | 0.1562 | 0.0 | 0.0 | 0.0 | 0.7895 | 0.0 | 0.0 | 0.0 | 0.0 | 0.3125 | 1.0 | 0.2941 | 0.8163 | 0.601 | 0.0 | 0.2532 | 0.0 | 0.0 | 0.0 | 0.2778 | 0.0273 | 0.0 | 0.0 | 0.8571 | 0.0 | 0.0 | 0.0 | 0.0 | 0.0 | 0.0 | F2 score |
| FDR | 0.4 | None | 0.881 | None | 1.0 | 0.893 | None | 0.9151 | 1.0 | 0.4348 | 0.5217 | None | 0.4255 | 1.0 | None | 0.7143 | None | 0.0 | 0.5333 | None | 1.0 | 0.5714 | 0.5 | None | None | None | 0.0 | None | 1.0 | None | None | 0.5 | 0.0 | 0.6667 | 0.3846 | 0.5053 | None | 0.871 | 1.0 | 1.0 | None | 0.5 | 0.9091 | 1.0 | None | 0.1429 | None | None | None | None | None | None | False discovery rate |
| FN | 3 | 10 | 18 | 27 | 11 | 7 | 3 | 4 | 9 | 5 | 25 | 8 | 77 | 4 | 15 | 0 | 9 | 16 | 113 | 23 | 53 | 14 | 13 | 5 | 4 | 3 | 1 | 7 | 15 | 7 | 7 | 15 | 0 | 5 | 1 | 27 | 10 | 16 | 6 | 6 | 24 | 3 | 42 | 18 | 10 | 1 | 2 | 7 | 6 | 29 | 18 | 11 | False negative/miss/type 2 error |
| FNR | 0.5 | 1.0 | 0.7826 | 1.0 | 1.0 | 0.2593 | 1.0 | 0.1818 | 1.0 | 0.1613 | 0.6944 | 1.0 | 0.7404 | 1.0 | 1.0 | 0.0 | 1.0 | 0.9412 | 0.8014 | 1.0 | 1.0 | 0.4828 | 0.8667 | 1.0 | 1.0 | 1.0 | 0.25 | 1.0 | 1.0 | 1.0 | 1.0 | 0.7143 | 0.0 | 0.7143 | 0.1111 | 0.3649 | 1.0 | 0.6667 | 1.0 | 1.0 | 1.0 | 0.75 | 0.9767 | 1.0 | 1.0 | 0.1429 | 1.0 | 1.0 | 1.0 | 1.0 | 1.0 | 1.0 | Miss rate or false negative rate |
| FOR | 0.0029 | 0.0097 | 0.0183 | 0.0263 | 0.0108 | 0.0083 | 0.0029 | 0.0049 | 0.0088 | 0.0051 | 0.0249 | 0.0078 | 0.0787 | 0.0039 | 0.0146 | 0.0 | 0.0088 | 0.0156 | 0.117 | 0.0224 | 0.0554 | 0.0141 | 0.0127 | 0.0049 | 0.0039 | 0.0029 | 0.001 | 0.0068 | 0.0146 | 0.0068 | 0.0068 | 0.0148 | 0.0 | 0.0049 | 0.001 | 0.029 | 0.0097 | 0.0166 | 0.0059 | 0.006 | 0.0234 | 0.0029 | 0.0414 | 0.0176 | 0.0097 | 0.001 | 0.0019 | 0.0068 | 0.0058 | 0.0283 | 0.0175 | 0.0107 | False omission rate |
| FP | 2 | 0 | 37 | 0 | 3 | 167 | 0 | 194 | 7 | 20 | 12 | 0 | 20 | 5 | 0 | 20 | 0 | 0 | 32 | 0 | 69 | 20 | 2 | 0 | 0 | 0 | 0 | 0 | 1 | 0 | 0 | 6 | 0 | 4 | 5 | 48 | 0 | 54 | 13 | 18 | 0 | 1 | 10 | 2 | 0 | 1 | 0 | 0 | 0 | 0 | 0 | 0 | False positive/type 1 error/false alarm |
| FPR | 0.002 | 0.0 | 0.0369 | 0.0 | 0.003 | 0.1672 | 0.0 | 0.1932 | 0.0069 | 0.0201 | 0.0121 | 0.0 | 0.0217 | 0.0049 | 0.0 | 0.0196 | 0.0 | 0.0 | 0.0362 | 0.0 | 0.0709 | 0.0201 | 0.002 | 0.0 | 0.0 | 0.0 | 0.0 | 0.0 | 0.001 | 0.0 | 0.0 | 0.006 | 0.0 | 0.0039 | 0.0049 | 0.0504 | 0.0 | 0.0539 | 0.0127 | 0.0176 | 0.0 | 0.001 | 0.0102 | 0.002 | 0.0 | 0.001 | 0.0 | 0.0 | 0.0 | 0.0 | 0.0 | 0.0 | Fall-out or false positive rate |
| G | 0.5477 | None | 0.1609 | None | 0.0 | 0.2815 | None | 0.2636 | 0.0 | 0.6885 | 0.3823 | None | 0.3862 | 0.0 | None | 0.5345 | None | 0.2425 | 0.3044 | None | 0.0 | 0.4708 | 0.2582 | None | None | None | 0.866 | None | 0.0 | None | None | 0.378 | 1.0 | 0.3086 | 0.7396 | 0.5606 | None | 0.2074 | 0.0 | 0.0 | None | 0.3536 | 0.046 | 0.0 | None | 0.8571 | None | None | None | None | None | None | G-measure geometric mean of precision and sensitivity |
| GI | 0.498 | 0.0 | 0.1805 | 0.0 | -0.003 | 0.5736 | 0.0 | 0.625 | -0.0069 | 0.8186 | 0.2934 | 0.0 | 0.2379 | -0.0049 | 0.0 | 0.9804 | 0.0 | 0.0588 | 0.1624 | 0.0 | -0.0709 | 0.4972 | 0.1314 | 0.0 | 0.0 | 0.0 | 0.75 | 0.0 | -0.001 | 0.0 | 0.0 | 0.2797 | 1.0 | 0.2818 | 0.884 | 0.5847 | 0.0 | 0.2794 | -0.0127 | -0.0176 | 0.0 | 0.249 | 0.0131 | -0.002 | 0.0 | 0.8562 | 0.0 | 0.0 | 0.0 | 0.0 | 0.0 | 0.0 | Gini index |
| GM | 0.7064 | 0.0 | 0.4576 | 0.0 | 0.0 | 0.7854 | 0.0 | 0.8125 | 0.0 | 0.9066 | 0.5494 | 0.0 | 0.504 | 0.0 | 0.0 | 0.9901 | 0.0 | 0.2425 | 0.4375 | 0.0 | 0.0 | 0.7119 | 0.3648 | 0.0 | 0.0 | 0.0 | 0.866 | 0.0 | 0.0 | 0.0 | 0.0 | 0.5329 | 1.0 | 0.5335 | 0.9405 | 0.7766 | 0.0 | 0.5616 | 0.0 | 0.0 | 0.0 | 0.4998 | 0.1517 | 0.0 | 0.0 | 0.9254 | 0.0 | 0.0 | 0.0 | 0.0 | 0.0 | 0.0 | G-mean geometric mean of specificity and sensitivity |
| IBA | 0.2505 | 0.0 | 0.0532 | 0.0 | 0.0 | 0.5601 | 0.0 | 0.6676 | 0.0 | 0.7058 | 0.0959 | 0.0 | 0.0714 | 0.0 | 0.0 | 0.9996 | 0.0 | 0.0035 | 0.0449 | 0.0 | 0.0 | 0.2723 | 0.018 | 0.0 | 0.0 | 0.0 | 0.5625 | 0.0 | 0.0 | 0.0 | 0.0 | 0.0828 | 1.0 | 0.0824 | 0.7906 | 0.4135 | 0.0 | 0.1221 | 0.0 | 0.0 | 0.0 | 0.0627 | 0.0008 | 0.0 | 0.0 | 0.7348 | 0.0 | 0.0 | 0.0 | 0.0 | 0.0 | 0.0 | Index of balanced accuracy |
| ICSI | 0.1 | None | -0.6636 | None | -1.0 | -0.1523 | None | -0.0969 | -1.0 | 0.4039 | -0.2162 | None | -0.1659 | -1.0 | None | 0.2857 | None | 0.0588 | -0.3348 | None | -1.0 | -0.0542 | -0.3667 | None | None | None | 0.75 | None | -1.0 | None | None | -0.2143 | 1.0 | -0.381 | 0.5043 | 0.1299 | None | -0.5376 | -1.0 | -1.0 | None | -0.25 | -0.8858 | -1.0 | None | 0.7143 | None | None | None | None | None | None | Individual classification success index |
| IS | 6.6809 | None | 2.4089 | None | None | 2.023 | None | 1.9854 | None | 4.2255 | 3.7688 | None | 2.5027 | None | None | 5.1955 | None | 5.9154 | 1.7637 | None | None | 3.9224 | 5.0959 | None | None | None | 8.0028 | None | None | None | None | 4.6105 | 7.1955 | 5.6105 | 6.1325 | 2.7781 | None | 2.4637 | None | None | None | 7.0028 | 1.1171 | None | None | 6.9731 | None | None | None | None | None | None | Information score |
| J | 0.375 | 0.0 | 0.0833 | 0.0 | 0.0 | 0.1031 | 0.0 | 0.0833 | 0.0 | 0.5098 | 0.2292 | 0.0 | 0.2177 | 0.0 | 0.0 | 0.2857 | 0.0 | 0.0588 | 0.1618 | 0.0 | 0.0 | 0.3061 | 0.1176 | 0.0 | 0.0 | 0.0 | 0.75 | 0.0 | 0.0 | 0.0 | 0.0 | 0.2222 | 1.0 | 0.1818 | 0.5714 | 0.3852 | 0.0 | 0.1026 | 0.0 | 0.0 | 0.0 | 0.2 | 0.0189 | 0.0 | 0.0 | 0.75 | 0.0 | 0.0 | 0.0 | 0.0 | 0.0 | 0.0 | Jaccard index |
| LS | 102.6 | None | 5.3106 | None | 0.0 | 4.0642 | None | 3.9597 | 0.0 | 18.7069 | 13.6304 | None | 5.6673 | 0.0 | None | 36.6429 | None | 60.3529 | 3.3957 | None | 0.0 | 15.1626 | 34.2 | None | None | None | 256.5 | None | 0.0 | None | None | 24.4286 | 146.5714 | 48.8571 | 70.1538 | 6.8595 | None | 5.5161 | 0.0 | 0.0 | None | 128.25 | 2.1691 | 0.0 | None | 125.6327 | None | None | None | None | None | None | Lift score |
| MCC | 0.5453 | None | 0.1349 | None | -0.0056 | 0.2378 | None | 0.2236 | -0.0078 | 0.6771 | 0.3647 | None | 0.3435 | -0.0044 | None | 0.5292 | None | 0.2406 | 0.2383 | None | -0.0627 | 0.4539 | 0.253 | None | None | None | 0.8656 | None | -0.0038 | None | None | 0.3684 | 1.0 | 0.3042 | 0.737 | 0.5218 | None | 0.1773 | -0.0087 | -0.0102 | None | 0.3518 | 0.0255 | -0.0059 | None | 0.8562 | None | None | None | None | None | None | Matthews correlation coefficient |
| MCCI | Moderate | None | Negligible | None | Negligible | Negligible | None | Negligible | Negligible | Moderate | Weak | None | Weak | Negligible | None | Moderate | None | Negligible | Negligible | None | Negligible | Weak | Negligible | None | None | None | Strong | None | Negligible | None | None | Weak | Very Strong | Weak | Strong | Moderate | None | Negligible | Negligible | Negligible | None | Weak | Negligible | Negligible | None | Strong | None | None | None | None | None | None | Matthews correlation coefficient interpretation |
| MCEN | 0.2061 | 0.0 | 0.4094 | 0.2141 | 0.2709 | 0.4871 | 0.2375 | 0.5161 | 0.4238 | 0.2613 | 0.3199 | 0.143 | 0.1871 | 0.3086 | 0.1049 | 0.132 | 0.0 | 0.1554 | 0.2849 | 0.0 | 0.253 | 0.3553 | 0.3077 | 0.1082 | 0.2248 | 0.2375 | 0.0749 | 0.2333 | 0.1778 | 0.0887 | 0.0 | 0.3105 | 0 | 0.3049 | 0.1936 | 0.3351 | 0.0 | 0.3615 | 0.2418 | 0.1216 | 0.2124 | 0.1359 | 0.4167 | 0.0703 | 0.0 | 0.1124 | 0.0 | 0.2923 | 0.1499 | 0.1861 | 0.2289 | 0.1417 | Modified confusion entropy |
| MK | 0.5971 | None | 0.1008 | None | -0.0108 | 0.0986 | None | 0.08 | -0.0088 | 0.5601 | 0.4533 | None | 0.4958 | -0.0039 | None | 0.2857 | None | 0.9844 | 0.3497 | None | -0.0554 | 0.4144 | 0.4873 | None | None | None | 0.999 | None | -0.0146 | None | None | 0.4852 | 1.0 | 0.3284 | 0.6144 | 0.4657 | None | 0.1124 | -0.0059 | -0.006 | None | 0.4971 | 0.0495 | -0.0176 | None | 0.8562 | None | None | None | None | None | None | Markedness |
| N | 1020 | 1016 | 1003 | 999 | 1015 | 999 | 1023 | 1004 | 1017 | 995 | 990 | 1018 | 922 | 1022 | 1011 | 1018 | 1017 | 1009 | 885 | 1003 | 973 | 997 | 1011 | 1021 | 1022 | 1023 | 1022 | 1019 | 1011 | 1019 | 1019 | 1005 | 1019 | 1019 | 1017 | 952 | 1016 | 1002 | 1020 | 1020 | 1002 | 1022 | 983 | 1008 | 1016 | 1019 | 1024 | 1019 | 1020 | 997 | 1008 | 1015 | Condition negative |
| NLR | 0.501 | 1.0 | 0.8126 | 1.0 | 1.003 | 0.3113 | 1.0 | 0.2254 | 1.0069 | 0.1646 | 0.703 | 1.0 | 0.7568 | 1.0049 | 1.0 | 0.0 | 1.0 | 0.9412 | 0.8315 | 1.0 | 1.0763 | 0.4926 | 0.8684 | 1.0 | 1.0 | 1.0 | 0.25 | 1.0 | 1.001 | 1.0 | 1.0 | 0.7186 | 0.0 | 0.7171 | 0.1117 | 0.3842 | 1.0 | 0.7046 | 1.0129 | 1.018 | 1.0 | 0.7507 | 0.9868 | 1.002 | 1.0 | 0.143 | 1.0 | 1.0 | 1.0 | 1.0 | 1.0 | 1.0 | Negative likelihood ratio |
| NLRI | Negligible | Negligible | Negligible | Negligible | Negligible | Poor | Negligible | Poor | Negligible | Fair | Negligible | Negligible | Negligible | Negligible | Negligible | Good | Negligible | Negligible | Negligible | Negligible | Negligible | Poor | Negligible | Negligible | Negligible | Negligible | Poor | Negligible | Negligible | Negligible | Negligible | Negligible | Good | Negligible | Fair | Poor | Negligible | Negligible | Negligible | Negligible | Negligible | Negligible | Negligible | Negligible | Negligible | Fair | Negligible | Negligible | Negligible | Negligible | Negligible | Negligible | Negative likelihood ratio interpretation |
| NPV | 0.9971 | 0.9903 | 0.9817 | 0.9737 | 0.9892 | 0.9917 | 0.9971 | 0.9951 | 0.9912 | 0.9949 | 0.9751 | 0.9922 | 0.9213 | 0.9961 | 0.9854 | 1.0 | 0.9912 | 0.9844 | 0.883 | 0.9776 | 0.9446 | 0.9859 | 0.9873 | 0.9951 | 0.9961 | 0.9971 | 0.999 | 0.9932 | 0.9854 | 0.9932 | 0.9932 | 0.9852 | 1.0 | 0.9951 | 0.999 | 0.971 | 0.9903 | 0.9834 | 0.9941 | 0.994 | 0.9766 | 0.9971 | 0.9586 | 0.9824 | 0.9903 | 0.999 | 0.9981 | 0.9932 | 0.9942 | 0.9717 | 0.9825 | 0.9893 | Negative predictive value |
| OC | 0.6 | None | 0.2174 | None | 0.0 | 0.7407 | None | 0.8182 | 0.0 | 0.8387 | 0.4783 | None | 0.5745 | 0.0 | None | 1.0 | None | 1.0 | 0.4667 | None | 0.0 | 0.5172 | 0.5 | None | None | None | 1.0 | None | 0.0 | None | None | 0.5 | 1.0 | 0.3333 | 0.8889 | 0.6351 | None | 0.3333 | 0.0 | 0.0 | None | 0.5 | 0.0909 | 0.0 | None | 0.8571 | None | None | None | None | None | None | Overlap coefficient |
| OOC | 0.5477 | None | 0.1609 | None | 0.0 | 0.2815 | None | 0.2636 | 0.0 | 0.6885 | 0.3823 | None | 0.3862 | 0.0 | None | 0.5345 | None | 0.2425 | 0.3044 | None | 0.0 | 0.4708 | 0.2582 | None | None | None | 0.866 | None | 0.0 | None | None | 0.378 | 1.0 | 0.3086 | 0.7396 | 0.5606 | None | 0.2074 | 0.0 | 0.0 | None | 0.3536 | 0.046 | 0.0 | None | 0.8571 | None | None | None | None | None | None | Otsuka-Ochiai coefficient |
| OP | 0.6627 | -0.0097 | 0.3147 | -0.0263 | -0.0136 | 0.7719 | -0.0029 | 0.8 | -0.0156 | 0.898 | 0.4364 | -0.0078 | 0.3249 | -0.0088 | -0.0146 | 0.9706 | -0.0088 | 0.0955 | 0.2003 | -0.0224 | -0.1189 | 0.6578 | 0.2211 | -0.0049 | -0.0039 | -0.0029 | 0.8562 | -0.0068 | -0.0156 | -0.0068 | -0.0068 | 0.426 | 1.0 | 0.437 | 0.9378 | 0.7285 | -0.0097 | 0.4528 | -0.0185 | -0.0234 | -0.0234 | 0.3964 | -0.0048 | -0.0195 | -0.0097 | 0.9216 | -0.0019 | -0.0068 | -0.0058 | -0.0283 | -0.0175 | -0.0107 | Optimized precision |
| P | 6 | 10 | 23 | 27 | 11 | 27 | 3 | 22 | 9 | 31 | 36 | 8 | 104 | 4 | 15 | 8 | 9 | 17 | 141 | 23 | 53 | 29 | 15 | 5 | 4 | 3 | 4 | 7 | 15 | 7 | 7 | 21 | 7 | 7 | 9 | 74 | 10 | 24 | 6 | 6 | 24 | 4 | 43 | 18 | 10 | 7 | 2 | 7 | 6 | 29 | 18 | 11 | Condition positive or support |
| PLR | 255.0 | None | 5.8931 | None | 0.0 | 4.4311 | None | 4.2343 | 0.0 | 41.7258 | 25.2083 | None | 11.9683 | 0.0 | None | 50.9 | None | None | 5.492 | None | 0.0 | 25.7845 | 67.4 | None | None | None | None | None | 0.0 | None | None | 47.8571 | None | 72.7857 | 180.8 | 12.5968 | None | 6.1852 | 0.0 | 0.0 | None | 255.5 | 2.286 | 0.0 | None | 873.4286 | None | None | None | None | None | None | Positive likelihood ratio |
| PLRI | Good | None | Fair | None | Negligible | Poor | None | Poor | Negligible | Good | Good | None | Good | Negligible | None | Good | None | None | Fair | None | Negligible | Good | Good | None | None | None | None | None | Negligible | None | None | Good | None | Good | Good | Good | None | Fair | Negligible | Negligible | None | Good | Poor | Negligible | None | Good | None | None | None | None | None | None | Positive likelihood ratio interpretation |
| POP | 1026 | 1026 | 1026 | 1026 | 1026 | 1026 | 1026 | 1026 | 1026 | 1026 | 1026 | 1026 | 1026 | 1026 | 1026 | 1026 | 1026 | 1026 | 1026 | 1026 | 1026 | 1026 | 1026 | 1026 | 1026 | 1026 | 1026 | 1026 | 1026 | 1026 | 1026 | 1026 | 1026 | 1026 | 1026 | 1026 | 1026 | 1026 | 1026 | 1026 | 1026 | 1026 | 1026 | 1026 | 1026 | 1026 | 1026 | 1026 | 1026 | 1026 | 1026 | 1026 | Population |
| PPV | 0.6 | None | 0.119 | None | 0.0 | 0.107 | None | 0.0849 | 0.0 | 0.5652 | 0.4783 | None | 0.5745 | 0.0 | None | 0.2857 | None | 1.0 | 0.4667 | None | 0.0 | 0.4286 | 0.5 | None | None | None | 1.0 | None | 0.0 | None | None | 0.5 | 1.0 | 0.3333 | 0.6154 | 0.4947 | None | 0.129 | 0.0 | 0.0 | None | 0.5 | 0.0909 | 0.0 | None | 0.8571 | None | None | None | None | None | None | Precision or positive predictive value |
| PRE | 0.0058 | 0.0097 | 0.0224 | 0.0263 | 0.0107 | 0.0263 | 0.0029 | 0.0214 | 0.0088 | 0.0302 | 0.0351 | 0.0078 | 0.1014 | 0.0039 | 0.0146 | 0.0078 | 0.0088 | 0.0166 | 0.1374 | 0.0224 | 0.0517 | 0.0283 | 0.0146 | 0.0049 | 0.0039 | 0.0029 | 0.0039 | 0.0068 | 0.0146 | 0.0068 | 0.0068 | 0.0205 | 0.0068 | 0.0068 | 0.0088 | 0.0721 | 0.0097 | 0.0234 | 0.0058 | 0.0058 | 0.0234 | 0.0039 | 0.0419 | 0.0175 | 0.0097 | 0.0068 | 0.0019 | 0.0068 | 0.0058 | 0.0283 | 0.0175 | 0.0107 | Prevalence |
| Q | 0.9961 | None | 0.7576 | None | -1.0 | 0.8687 | None | 0.8989 | -1.0 | 0.9921 | 0.9457 | None | 0.8811 | -1.0 | None | None | None | None | 0.737 | None | -1.0 | 0.9625 | 0.9746 | None | None | None | None | None | -1.0 | None | None | 0.9704 | None | 0.9805 | 0.9988 | 0.9408 | None | 0.7955 | -1.0 | -1.0 | None | 0.9941 | 0.397 | -1.0 | None | 0.9997 | None | None | None | None | None | None | Yule Q - coefficient of colligation |
| QI | Strong | None | Strong | None | Negligible | Strong | None | Strong | Negligible | Strong | Strong | None | Strong | Negligible | None | None | None | None | Moderate | None | Negligible | Strong | Strong | None | None | None | None | None | Negligible | None | None | Strong | None | Strong | Strong | Strong | None | Strong | Negligible | Negligible | None | Strong | Weak | Negligible | None | Strong | None | None | None | None | None | None | Yule Q interpretation |
| RACC | 0.0 | 0.0 | 0.0009 | 0.0 | 0.0 | 0.0048 | 0.0 | 0.0044 | 0.0001 | 0.0014 | 0.0008 | 0.0 | 0.0046 | 0.0 | 0.0 | 0.0002 | 0.0 | 0.0 | 0.008 | 0.0 | 0.0035 | 0.001 | 0.0001 | 0.0 | 0.0 | 0.0 | 0.0 | 0.0 | 0.0 | 0.0 | 0.0 | 0.0002 | 0.0 | 0.0 | 0.0001 | 0.0067 | 0.0 | 0.0014 | 0.0001 | 0.0001 | 0.0 | 0.0 | 0.0004 | 0.0 | 0.0 | 0.0 | 0.0 | 0.0 | 0.0 | 0.0 | 0.0 | 0.0 | Random accuracy |
| RACCU | 0.0 | 0.0 | 0.001 | 0.0002 | 0.0 | 0.0109 | 0.0 | 0.013 | 0.0001 | 0.0014 | 0.0008 | 0.0 | 0.0054 | 0.0 | 0.0001 | 0.0003 | 0.0 | 0.0001 | 0.0096 | 0.0001 | 0.0035 | 0.001 | 0.0001 | 0.0 | 0.0 | 0.0 | 0.0 | 0.0 | 0.0001 | 0.0 | 0.0 | 0.0003 | 0.0 | 0.0 | 0.0001 | 0.0068 | 0.0 | 0.0018 | 0.0001 | 0.0001 | 0.0001 | 0.0 | 0.0007 | 0.0001 | 0.0 | 0.0 | 0.0 | 0.0 | 0.0 | 0.0002 | 0.0001 | 0.0 | Random accuracy unbiased |
| TN | 1018 | 1016 | 966 | 999 | 1012 | 832 | 1023 | 810 | 1010 | 975 | 978 | 1018 | 902 | 1017 | 1011 | 998 | 1017 | 1009 | 853 | 1003 | 904 | 977 | 1009 | 1021 | 1022 | 1023 | 1022 | 1019 | 1010 | 1019 | 1019 | 999 | 1019 | 1015 | 1012 | 904 | 1016 | 948 | 1007 | 1002 | 1002 | 1021 | 973 | 1006 | 1016 | 1018 | 1024 | 1019 | 1020 | 997 | 1008 | 1015 | True negative/correct rejection |
| TNR | 0.998 | 1.0 | 0.9631 | 1.0 | 0.997 | 0.8328 | 1.0 | 0.8068 | 0.9931 | 0.9799 | 0.9879 | 1.0 | 0.9783 | 0.9951 | 1.0 | 0.9804 | 1.0 | 1.0 | 0.9638 | 1.0 | 0.9291 | 0.9799 | 0.998 | 1.0 | 1.0 | 1.0 | 1.0 | 1.0 | 0.999 | 1.0 | 1.0 | 0.994 | 1.0 | 0.9961 | 0.9951 | 0.9496 | 1.0 | 0.9461 | 0.9873 | 0.9824 | 1.0 | 0.999 | 0.9898 | 0.998 | 1.0 | 0.999 | 1.0 | 1.0 | 1.0 | 1.0 | 1.0 | 1.0 | Specificity or true negative rate |
| TON | 1021 | 1026 | 984 | 1026 | 1023 | 839 | 1026 | 814 | 1019 | 980 | 1003 | 1026 | 979 | 1021 | 1026 | 998 | 1026 | 1025 | 966 | 1026 | 957 | 991 | 1022 | 1026 | 1026 | 1026 | 1023 | 1026 | 1025 | 1026 | 1026 | 1014 | 1019 | 1020 | 1013 | 931 | 1026 | 964 | 1013 | 1008 | 1026 | 1024 | 1015 | 1024 | 1026 | 1019 | 1026 | 1026 | 1026 | 1026 | 1026 | 1026 | Test outcome negative |
| TOP | 5 | 0 | 42 | 0 | 3 | 187 | 0 | 212 | 7 | 46 | 23 | 0 | 47 | 5 | 0 | 28 | 0 | 1 | 60 | 0 | 69 | 35 | 4 | 0 | 0 | 0 | 3 | 0 | 1 | 0 | 0 | 12 | 7 | 6 | 13 | 95 | 0 | 62 | 13 | 18 | 0 | 2 | 11 | 2 | 0 | 7 | 0 | 0 | 0 | 0 | 0 | 0 | Test outcome positive |
| TP | 3 | 0 | 5 | 0 | 0 | 20 | 0 | 18 | 0 | 26 | 11 | 0 | 27 | 0 | 0 | 8 | 0 | 1 | 28 | 0 | 0 | 15 | 2 | 0 | 0 | 0 | 3 | 0 | 0 | 0 | 0 | 6 | 7 | 2 | 8 | 47 | 0 | 8 | 0 | 0 | 0 | 1 | 1 | 0 | 0 | 6 | 0 | 0 | 0 | 0 | 0 | 0 | True positive/hit |
| TPR | 0.5 | 0.0 | 0.2174 | 0.0 | 0.0 | 0.7407 | 0.0 | 0.8182 | 0.0 | 0.8387 | 0.3056 | 0.0 | 0.2596 | 0.0 | 0.0 | 1.0 | 0.0 | 0.0588 | 0.1986 | 0.0 | 0.0 | 0.5172 | 0.1333 | 0.0 | 0.0 | 0.0 | 0.75 | 0.0 | 0.0 | 0.0 | 0.0 | 0.2857 | 1.0 | 0.2857 | 0.8889 | 0.6351 | 0.0 | 0.3333 | 0.0 | 0.0 | 0.0 | 0.25 | 0.0233 | 0.0 | 0.0 | 0.8571 | 0.0 | 0.0 | 0.0 | 0.0 | 0.0 | 0.0 | Sensitivity, recall, hit rate, or true positive rate |
| Y | 0.498 | 0.0 | 0.1805 | 0.0 | -0.003 | 0.5736 | 0.0 | 0.625 | -0.0069 | 0.8186 | 0.2934 | 0.0 | 0.2379 | -0.0049 | 0.0 | 0.9804 | 0.0 | 0.0588 | 0.1624 | 0.0 | -0.0709 | 0.4972 | 0.1314 | 0.0 | 0.0 | 0.0 | 0.75 | 0.0 | -0.001 | 0.0 | 0.0 | 0.2797 | 1.0 | 0.2818 | 0.884 | 0.5847 | 0.0 | 0.2794 | -0.0127 | -0.0176 | 0.0 | 0.249 | 0.0131 | -0.002 | 0.0 | 0.8562 | 0.0 | 0.0 | 0.0 | 0.0 | 0.0 | 0.0 | Youden index |
| dInd | 0.5 | 1.0 | 0.7835 | 1.0 | 1.0 | 0.3085 | 1.0 | 0.2653 | 1.0 | 0.1625 | 0.6946 | 1.0 | 0.7407 | 1.0 | 1.0 | 0.0196 | 1.0 | 0.9412 | 0.8022 | 1.0 | 1.0025 | 0.4832 | 0.8667 | 1.0 | 1.0 | 1.0 | 0.25 | 1.0 | 1.0 | 1.0 | 1.0 | 0.7143 | 0.0 | 0.7143 | 0.1112 | 0.3683 | 1.0 | 0.6688 | 1.0001 | 1.0002 | 1.0 | 0.75 | 0.9768 | 1.0 | 1.0 | 0.1429 | 1.0 | 1.0 | 1.0 | 1.0 | 1.0 | 1.0 | Distance index |
| sInd | 0.6464 | 0.2929 | 0.446 | 0.2929 | 0.2929 | 0.7819 | 0.2929 | 0.8124 | 0.2929 | 0.8851 | 0.5089 | 0.2929 | 0.4762 | 0.2929 | 0.2929 | 0.9861 | 0.2929 | 0.3345 | 0.4327 | 0.2929 | 0.2911 | 0.6583 | 0.3872 | 0.2929 | 0.2929 | 0.2929 | 0.8232 | 0.2929 | 0.2929 | 0.2929 | 0.2929 | 0.4949 | 1.0 | 0.4949 | 0.9214 | 0.7395 | 0.2929 | 0.5271 | 0.2928 | 0.2928 | 0.2929 | 0.4697 | 0.3093 | 0.2929 | 0.2929 | 0.899 | 0.2929 | 0.2929 | 0.2929 | 0.2929 | 0.2929 | 0.2929 | Similarity index |

Generated By PyCM Version 3.4
