# Supplementary material for: TopEC: prediction of Enzyme Commission classes by 3D graph neural networks and localized 3D protein descriptor
Source: Nat Commun. 2025 Mar 20;16:2737. doi: 10.1038/s41467-025-57324-5 (PMC11923149; doi:10.1038/s41467-025-57324-5)
Supplement: Supplementary file 3 — Supplementary Data 1 [file 41467_2025_57324_MOESM3_ESM.zip › Data_S1/table1/hierarchical/DeepFRI_FOLD.html]

PyCM Report


# PyCM Report

## Dataset Type :

- Multi-Class Classification
- Imbalanced

Note 1 : Recommended statistics for this type of classification highlighted in aqua

Note 2 : The recommender system assumes that the input is the result of classification over the whole data rather than just a part of it.
If the confusion matrix is the result of test data classification, the recommendation is not valid.

## Confusion Matrix :

|  |  |  |  |  |  |  |  |  |  |  |  |  |  |  |  |  |  |  |  |  |  |  |  |  |  |  |  |  |  |  |  |  |  |  |  |  |  |  |  |  |  |  |  |  |  |  |  |  |  |  |  |  |  |  |  |  |  |  |  |  |  |  |  |  |  |  |  |  |  |  |  |  |  |  |  |  |  |  |  |  |  |  |  |  |  |  |  |  |  |  |  |  |  |  |  |  |  |  |  |  |  |  |  |  |  |  |  |  |  |  |  |  |  |  |  |  |  |  |  |  |  |  |  |  |  |  |  |  |  |  |  |  |  |  |  |  |  |  |  |  |  |  |  |  |  |  |  |  |  |  |  |  |  |  |  |  |  |  |  |  |  |  |  |  |  |  |  |  |  |  |  |  |  |  |  |  |  |  |  |  |  |  |  |  |  |  |  |  |  |  |  |  |  |  |  |  |  |  |  |  |  |  |  |  |  |  |  |  |  |  |  |  |  |  |  |  |  |  |  |  |  |  |  |  |  |  |  |  |  |  |  |  |  |  |  |  |  |  |  |  |  |  |  |  |  |  |  |  |  |  |  |  |  |  |  |  |  |  |  |  |  |  |  |  |  |  |  |  |  |  |  |  |  |  |  |  |  |  |  |  |  |  |  |  |  |  |  |  |  |  |  |  |  |  |  |  |  |  |  |  |  |  |  |  |  |  |  |  |  |  |  |  |  |  |  |  |  |  |  |  |  |  |  |  |  |  |  |  |  |  |  |  |  |  |  |  |  |  |  |  |  |  |  |  |  |  |  |  |  |  |  |  |  |  |  |  |  |  |  |  |  |  |  |  |  |  |  |  |  |  |  |  |  |  |  |  |  |  |  |  |  |  |  |  |  |  |  |  |  |  |  |  |  |  |  |  |  |  |  |  |  |  |  |  |  |  |  |  |  |  |  |  |  |  |  |  |  |  |  |  |  |  |  |  |  |  |  |  |  |  |  |  |  |  |  |  |  |  |  |  |  |  |  |  |  |  |  |  |  |  |  |  |  |  |  |  |  |  |  |  |  |  |  |  |  |  |  |  |  |  |  |  |  |  |  |  |  |  |  |  |  |  |  |  |  |  |  |  |  |  |  |  |  |  |  |  |  |  |  |  |  |  |  |  |  |  |  |  |  |  |  |  |  |  |  |  |  |  |  |  |  |  |  |  |  |  |  |  |  |  |  |  |  |  |  |  |  |  |  |  |  |  |  |  |  |  |  |  |  |  |  |  |  |  |  |  |  |  |  |  |  |  |  |  |  |  |  |  |  |  |  |  |  |  |  |  |  |  |  |  |  |  |  |  |  |  |  |  |  |  |  |  |  |  |  |  |  |  |  |  |  |  |  |  |  |  |  |  |  |  |  |  |  |  |  |  |  |  |  |  |  |  |  |  |  |  |  |  |  |  |  |  |  |  |  |  |  |  |  |  |  |  |  |  |  |  |  |  |  |  |  |  |  |  |  |  |  |  |  |  |  |  |  |  |  |  |  |  |  |  |  |  |  |  |  |  |  |  |  |  |  |  |  |  |  |  |  |  |  |  |  |  |  |  |  |  |  |  |  |  |  |  |  |  |  |  |  |  |  |  |  |  |  |  |  |  |  |  |  |  |  |  |  |  |  |  |  |  |  |  |  |  |  |  |  |  |  |  |  |  |  |  |  |  |  |  |  |  |  |  |  |  |  |  |  |  |  |  |  |  |  |  |  |  |  |  |  |  |  |  |  |  |  |  |  |  |  |  |  |  |  |  |  |  |  |  |  |  |  |  |  |  |  |  |  |  |  |  |  |  |  |  |  |  |  |  |  |  |  |  |  |  |  |  |  |  |  |  |  |  |  |  |  |  |  |  |  |  |  |  |  |  |  |  |  |  |  |  |  |  |  |  |  |  |  |  |  |  |  |  |  |  |  |  |  |  |  |  |  |  |  |  |  |  |  |  |  |  |  |  |  |  |  |  |  |  |  |  |  |  |  |  |  |  |  |  |  |  |  |  |  |  |  |  |  |  |  |  |  |  |  |  |  |  |  |  |  |  |  |  |  |  |  |  |  |  |  |  |  |  |  |  |  |  |  |  |  |  |  |  |  |  |  |  |  |  |  |  |  |  |  |  |  |  |  |  |  |  |  |  |  |  |  |  |  |  |  |  |  |  |  |  |  |  |  |  |  |  |  |  |  |  |  |  |  |  |  |  |  |  |  |  |  |  |  |  |  |  |  |  |  |  |  |  |  |  |  |  |  |  |  |  |  |  |  |  |  |  |  |  |  |  |  |  |  |  |  |  |  |  |  |  |  |  |  |  |  |  |  |  |  |  |  |  |  |  |  |  |  |  |  |  |  |  |  |  |  |  |  |  |  |  |  |  |  |  |  |  |  |  |  |  |  |  |  |  |  |  |  |  |  |  |  |  |  |  |  |  |  |  |  |  |  |  |  |  |  |  |  |  |  |  |  |  |  |  |  |  |  |  |  |  |  |  |  |  |  |  |  |  |  |  |  |  |  |  |  |  |  |  |  |  |  |  |  |  |  |  |  |  |  |  |  |  |  |  |  |  |  |  |  |  |  |  |  |  |  |  |  |  |  |  |  |  |  |  |  |  |  |  |  |  |  |  |  |  |  |  |  |  |  |  |  |  |  |  |  |  |  |  |  |  |  |  |  |  |  |  |  |  |  |  |  |  |  |  |  |  |  |  |  |  |  |  |  |  |  |  |  |  |  |  |  |  |  |  |  |  |  |  |  |  |  |  |  |  |  |  |  |  |  |  |  |  |  |  |  |  |  |  |  |  |  |  |  |  |  |  |  |  |  |  |  |  |  |  |  |  |  |  |  |  |  |  |  |  |  |  |  |  |  |  |  |  |  |  |  |  |  |  |  |  |  |  |  |  |  |  |  |  |  |  |  |  |  |  |  |  |  |  |  |  |  |  |  |  |  |  |  |  |  |  |  |  |  |  |  |  |  |  |  |  |  |  |  |  |  |  |  |  |  |  |  |  |  |  |  |  |  |  |  |  |  |  |  |  |  |  |  |  |  |  |  |  |  |  |  |  |  |  |  |  |  |  |  |  |  |  |  |  |  |  |  |  |  |  |  |  |  |  |  |  |  |  |  |  |  |  |  |  |  |  |  |  |  |  |  |  |  |  |  |  |  |  |  |  |  |  |  |  |  |  |  |  |  |  |  |  |  |  |  |  |  |  |  |  |  |  |  |  |  |  |  |  |  |  |  |  |  |  |  |  |  |  |  |  |  |  |  |  |  |  |  |  |  |  |  |  |  |  |  |  |  |  |  |  |  |  |  |  |  |  |  |  |  |  |  |  |  |  |  |  |  |  |  |  |  |  |  |  |  |  |  |  |  |  |  |  |  |  |  |  |  |  |  |  |  |  |  |  |  |  |  |  |  |  |  |  |  |  |  |  |  |  |  |  |  |  |  |  |  |  |  |  |  |  |  |  |  |  |  |  |  |  |  |  |  |  |  |  |  |  |  |  |  |  |  |  |  |  |  |  |  |  |  |  |  |  |  |  |  |  |  |  |  |  |  |  |  |  |  |  |  |  |  |  |  |  |  |  |  |  |  |  |  |  |  |  |  |  |  |  |  |  |  |  |  |  |  |  |  |  |  |  |  |  |  |  |  |  |  |  |  |  |  |  |  |  |  |  |  |  |  |  |  |  |  |  |  |  |  |  |  |  |  |  |  |  |  |  |  |  |  |  |  |  |  |  |  |  |  |  |  |  |  |  |  |  |  |  |  |  |  |  |  |  |  |  |  |  |  |  |  |  |  |  |  |  |  |  |  |  |  |  |  |  |  |  |  |  |  |  |  |  |  |  |  |  |  |  |  |  |  |  |  |  |  |  |  |  |  |  |  |  |  |  |  |  |  |  |  |  |  |  |  |  |  |  |  |  |  |  |  |  |  |  |  |  |  |  |  |  |  |  |  |  |  |  |  |  |  |  |  |  |  |  |  |  |  |  |  |  |  |  |  |  |  |  |  |  |  |  |  |  |  |  |  |  |  |  |  |  |  |  |  |  |  |  |  |  |  |  |  |  |  |  |  |  |  |  |  |  |  |  |  |  |  |  |  |  |  |  |  |  |  |  |  |  |  |  |  |  |  |  |  |  |  |  |  |  |  |  |  |  |  |  |  |  |  |  |  |  |  |  |  |  |  |  |  |  |  |  |  |  |  |  |  |  |  |  |  |  |  |  |  |  |  |  |  |  |  |  |  |  |  |  |  |  |  |  |  |  |  |  |  |  |  |  |  |  |  |  |  |  |  |  |  |  |  |  |  |  |  |  |  |  |  |  |  |  |  |  |  |  |  |  |  |  |  |  |  |  |  |  |  |  |  |  |  |  |  |  |  |  |  |  |  |  |  |  |  |  |  |  |  |  |  |  |  |  |  |  |  |  |  |  |  |  |  |  |  |  |  |  |  |  |  |  |  |  |  |  |  |  |  |  |  |  |  |  |  |  |  |  |  |  |  |  |  |  |  |  |  |  |  |  |  |  |  |  |  |  |  |  |  |  |  |  |  |  |  |  |  |  |  |  |  |  |  |  |  |  |  |  |  |  |  |  |  |  |  |  |  |  |  |  |  |  |  |  |  |  |  |  |  |  |  |  |  |  |  |  |  |  |  |  |  |  |  |  |  |  |  |  |  |  |  |  |  |  |  |  |  |  |  |  |  |  |  |  |  |  |  |  |  |  |  |  |  |  |  |  |  |  |  |  |  |  |  |  |  |  |  |  |  |  |  |  |  |  |  |  |  |  |  |  |  |  |  |  |  |  |  |  |  |  |  |  |  |  |  |  |  |  |  |  |  |  |  |  |  |  |  |  |  |  |  |  |  |  |  |  |  |  |  |  |  |  |  |  |  |  |  |  |  |  |  |  |  |  |  |  |  |  |  |  |  |  |  |  |  |  |  |  |  |  |  |  |  |  |  |  |  |  |  |  |  |  |  |  |  |  |  |  |  |  |  |  |  |  |  |  |  |  |  |  |  |  |  |  |  |  |  |  |  |  |  |  |  |  |  |  |  |  |  |  |  |  |  |  |  |  |  |  |  |  |  |  |  |  |  |  |  |  |  |  |  |  |  |  |  |  |  |  |  |  |  |  |  |  |  |  |  |  |  |  |  |  |  |  |  |  |  |  |  |  |  |  |  |  |  |  |  |  |  |  |  |  |  |  |  |  |  |  |  |  |  |  |  |  |  |  |  |  |  |  |  |  |  |  |  |  |  |  |  |  |  |  |  |  |  |  |  |  |  |  |  |  |  |  |  |  |  |  |  |  |  |  |  |  |  |  |  |  |  |  |  |  |  |  |  |  |  |  |  |  |  |  |  |  |  |  |  |  |  |  |  |  |  |  |  |  |  |  |  |  |  |  |  |  |  |  |  |  |  |  |  |  |  |  |  |  |  |  |  |  |  |  |  |  |  |  |  |  |  |  |  |  |  |  |  |  |  |  |  |  |  |  |  |  |  |  |  |  |  |  |  |  |  |  |  |  |  |  |  |  |  |  |  |  |  |  |  |  |  |  |  |  |  |  |  |  |  |  |  |  |  |  |  |  |  |  |  |  |  |  |  |  |  |  |  |  |  |  |  |  |  |  |  |  |  |  |  |  |  |  |  |  |  |  |  |  |  |  |  |  |  |  |  |  |  |  |  |  |  |  |  |  |  |  |  |  |  |  |  |  |  |  |  |  |  |  |  |  |  |  |  |  |  |  |  |  |  |  |  |  |  |  |  |  |  |  |  |  |  |  |  |  |  |  |  |  |  |  |  |  |  |  |  |  |  |  |  |  |  |  |  |  |  |  |  |  |  |  |  |  |  |  |  |  |  |  |  |  |  |  |  |  |  |  |  |  |  |  |  |  |  |  |  |  |  |  |  |  |  |  |  |  |  |  |  |  |  |  |  |  |  |  |  |  |  |  |  |  |  |  |  |  |  |  |  |  |  |  |  |  |  |  |  |  |  |  |  |  |  |  |  |  |  |  |  |  |  |  |  |  |  |  |  |  |  |  |  |  |  |  |  |  |  |  |  |  |  |  |  |  |  |  |  |  |  |  |  |  |  |  |  |  |  |  |  |  |  |  |  |  |  |  |  |  |  |  |  |  |  |  |  |  |  |  |  |  |  |  |  |  |  |  |  |  |  |  |  |  |  |  |  |  |  |  |  |  |  |  |  |  |  |  |  |  |  |  |  |  |  |  |  |  |  |  |  |  |  |  |  |  |  |  |  |  |  |  |  |  |  |  |  |  |  |  |  |  |  |  |  |  |  |  |  |  |  |  |  |  |  |  |  |  |  |  |
| --- | --- | --- | --- | --- | --- | --- | --- | --- | --- | --- | --- | --- | --- | --- | --- | --- | --- | --- | --- | --- | --- | --- | --- | --- | --- | --- | --- | --- | --- | --- | --- | --- | --- | --- | --- | --- | --- | --- | --- | --- | --- | --- | --- | --- | --- | --- | --- | --- | --- | --- | --- | --- | --- | --- | --- | --- | --- | --- | --- | --- | --- | --- | --- | --- | --- | --- | --- | --- | --- | --- | --- | --- | --- | --- | --- | --- | --- | --- | --- | --- | --- | --- | --- | --- | --- | --- | --- | --- | --- | --- | --- | --- | --- | --- | --- | --- | --- | --- | --- | --- | --- | --- | --- | --- | --- | --- | --- | --- | --- | --- | --- | --- | --- | --- | --- | --- | --- | --- | --- | --- | --- | --- | --- | --- | --- | --- | --- | --- | --- | --- | --- | --- | --- | --- | --- | --- | --- | --- | --- | --- | --- | --- | --- | --- | --- | --- | --- | --- | --- | --- | --- | --- | --- | --- | --- | --- | --- | --- | --- | --- | --- | --- | --- | --- | --- | --- | --- | --- | --- | --- | --- | --- | --- | --- | --- | --- | --- | --- | --- | --- | --- | --- | --- | --- | --- | --- | --- | --- | --- | --- | --- | --- | --- | --- | --- | --- | --- | --- | --- | --- | --- | --- | --- | --- | --- | --- | --- | --- | --- | --- | --- | --- | --- | --- | --- | --- | --- | --- | --- | --- | --- | --- | --- | --- | --- | --- | --- | --- | --- | --- | --- | --- | --- | --- | --- | --- | --- | --- | --- | --- | --- | --- | --- | --- | --- | --- | --- | --- | --- | --- | --- | --- | --- | --- | --- | --- | --- | --- | --- | --- | --- | --- | --- | --- | --- | --- | --- | --- | --- | --- | --- | --- | --- | --- | --- | --- | --- | --- | --- | --- | --- | --- | --- | --- | --- | --- | --- | --- | --- | --- | --- | --- | --- | --- | --- | --- | --- | --- | --- | --- | --- | --- | --- | --- | --- | --- | --- | --- | --- | --- | --- | --- | --- | --- | --- | --- | --- | --- | --- | --- | --- | --- | --- | --- | --- | --- | --- | --- | --- | --- | --- | --- | --- | --- | --- | --- | --- | --- | --- | --- | --- | --- | --- | --- | --- | --- | --- | --- | --- | --- | --- | --- | --- | --- | --- | --- | --- | --- | --- | --- | --- | --- | --- | --- | --- | --- | --- | --- | --- | --- | --- | --- | --- | --- | --- | --- | --- | --- | --- | --- | --- | --- | --- | --- | --- | --- | --- | --- | --- | --- | --- | --- | --- | --- | --- | --- | --- | --- | --- | --- | --- | --- | --- | --- | --- | --- | --- | --- | --- | --- | --- | --- | --- | --- | --- | --- | --- | --- | --- | --- | --- | --- | --- | --- | --- | --- | --- | --- | --- | --- | --- | --- | --- | --- | --- | --- | --- | --- | --- | --- | --- | --- | --- | --- | --- | --- | --- | --- | --- | --- | --- | --- | --- | --- | --- | --- | --- | --- | --- | --- | --- | --- | --- | --- | --- | --- | --- | --- | --- | --- | --- | --- | --- | --- | --- | --- | --- | --- | --- | --- | --- | --- | --- | --- | --- | --- | --- | --- | --- | --- | --- | --- | --- | --- | --- | --- | --- | --- | --- | --- | --- | --- | --- | --- | --- | --- | --- | --- | --- | --- | --- | --- | --- | --- | --- | --- | --- | --- | --- | --- | --- | --- | --- | --- | --- | --- | --- | --- | --- | --- | --- | --- | --- | --- | --- | --- | --- | --- | --- | --- | --- | --- | --- | --- | --- | --- | --- | --- | --- | --- | --- | --- | --- | --- | --- | --- | --- | --- | --- | --- | --- | --- | --- | --- | --- | --- | --- | --- | --- | --- | --- | --- | --- | --- | --- | --- | --- | --- | --- | --- | --- | --- | --- | --- | --- | --- | --- | --- | --- | --- | --- | --- | --- | --- | --- | --- | --- | --- | --- | --- | --- | --- | --- | --- | --- | --- | --- | --- | --- | --- | --- | --- | --- | --- | --- | --- | --- | --- | --- | --- | --- | --- | --- | --- | --- | --- | --- | --- | --- | --- | --- | --- | --- | --- | --- | --- | --- | --- | --- | --- | --- | --- | --- | --- | --- | --- | --- | --- | --- | --- | --- | --- | --- | --- | --- | --- | --- | --- | --- | --- | --- | --- | --- | --- | --- | --- | --- | --- | --- | --- | --- | --- | --- | --- | --- | --- | --- | --- | --- | --- | --- | --- | --- | --- | --- | --- | --- | --- | --- | --- | --- | --- | --- | --- | --- | --- | --- | --- | --- | --- | --- | --- | --- | --- | --- | --- | --- | --- | --- | --- | --- | --- | --- | --- | --- | --- | --- | --- | --- | --- | --- | --- | --- | --- | --- | --- | --- | --- | --- | --- | --- | --- | --- | --- | --- | --- | --- | --- | --- | --- | --- | --- | --- | --- | --- | --- | --- | --- | --- | --- | --- | --- | --- | --- | --- | --- | --- | --- | --- | --- | --- | --- | --- | --- | --- | --- | --- | --- | --- | --- | --- | --- | --- | --- | --- | --- | --- | --- | --- | --- | --- | --- | --- | --- | --- | --- | --- | --- | --- | --- | --- | --- | --- | --- | --- | --- | --- | --- | --- | --- | --- | --- | --- | --- | --- | --- | --- | --- | --- | --- | --- | --- | --- | --- | --- | --- | --- | --- | --- | --- | --- | --- | --- | --- | --- | --- | --- | --- | --- | --- | --- | --- | --- | --- | --- | --- | --- | --- | --- | --- | --- | --- | --- | --- | --- | --- | --- | --- | --- | --- | --- | --- | --- | --- | --- | --- | --- | --- | --- | --- | --- | --- | --- | --- | --- | --- | --- | --- | --- | --- | --- | --- | --- | --- | --- | --- | --- | --- | --- | --- | --- | --- | --- | --- | --- | --- | --- | --- | --- | --- | --- | --- | --- | --- | --- | --- | --- | --- | --- | --- | --- | --- | --- | --- | --- | --- | --- | --- | --- | --- | --- | --- | --- | --- | --- | --- | --- | --- | --- | --- | --- | --- | --- | --- | --- | --- | --- | --- | --- | --- | --- | --- | --- | --- | --- | --- | --- | --- | --- | --- | --- | --- | --- | --- | --- | --- | --- | --- | --- | --- | --- | --- | --- | --- | --- | --- | --- | --- | --- | --- | --- | --- | --- | --- | --- | --- | --- | --- | --- | --- | --- | --- | --- | --- | --- | --- | --- | --- | --- | --- | --- | --- | --- | --- | --- | --- | --- | --- | --- | --- | --- | --- | --- | --- | --- | --- | --- | --- | --- | --- | --- | --- | --- | --- | --- | --- | --- | --- | --- | --- | --- | --- | --- | --- | --- | --- | --- | --- | --- | --- | --- | --- | --- | --- | --- | --- | --- | --- | --- | --- | --- | --- | --- | --- | --- | --- | --- | --- | --- | --- | --- | --- | --- | --- | --- | --- | --- | --- | --- | --- | --- | --- | --- | --- | --- | --- | --- | --- | --- | --- | --- | --- | --- | --- | --- | --- | --- | --- | --- | --- | --- | --- | --- | --- | --- | --- | --- | --- | --- | --- | --- | --- | --- | --- | --- | --- | --- | --- | --- | --- | --- | --- | --- | --- | --- | --- | --- | --- | --- | --- | --- | --- | --- | --- | --- | --- | --- | --- | --- | --- | --- | --- | --- | --- | --- | --- | --- | --- | --- | --- | --- | --- | --- | --- | --- | --- | --- | --- | --- | --- | --- | --- | --- | --- | --- | --- | --- | --- | --- | --- | --- | --- | --- | --- | --- | --- | --- | --- | --- | --- | --- | --- | --- | --- | --- | --- | --- | --- | --- | --- | --- | --- | --- | --- | --- | --- | --- | --- | --- | --- | --- | --- | --- | --- | --- | --- | --- | --- | --- | --- | --- | --- | --- | --- | --- | --- | --- | --- | --- | --- | --- | --- | --- | --- | --- | --- | --- | --- | --- | --- | --- | --- | --- | --- | --- | --- | --- | --- | --- | --- | --- | --- | --- | --- | --- | --- | --- | --- | --- | --- | --- | --- | --- | --- | --- | --- | --- | --- | --- | --- | --- | --- | --- | --- | --- | --- | --- | --- | --- | --- | --- | --- | --- | --- | --- | --- | --- | --- | --- | --- | --- | --- | --- | --- | --- | --- | --- | --- | --- | --- | --- | --- | --- | --- | --- | --- | --- | --- | --- | --- | --- | --- | --- | --- | --- | --- | --- | --- | --- | --- | --- | --- | --- | --- | --- | --- | --- | --- | --- | --- | --- | --- | --- | --- | --- | --- | --- | --- | --- | --- | --- | --- | --- | --- | --- | --- | --- | --- | --- | --- | --- | --- | --- | --- | --- | --- | --- | --- | --- | --- | --- | --- | --- | --- | --- | --- | --- | --- | --- | --- | --- | --- | --- | --- | --- | --- | --- | --- | --- | --- | --- | --- | --- | --- | --- | --- | --- | --- | --- | --- | --- | --- | --- | --- | --- | --- | --- | --- | --- | --- | --- | --- | --- | --- | --- | --- | --- | --- | --- | --- | --- | --- | --- | --- | --- | --- | --- | --- | --- | --- | --- | --- | --- | --- | --- | --- | --- | --- | --- | --- | --- | --- | --- | --- | --- | --- | --- | --- | --- | --- | --- | --- | --- | --- | --- | --- | --- | --- | --- | --- | --- | --- | --- | --- | --- | --- | --- | --- | --- | --- | --- | --- | --- | --- | --- | --- | --- | --- | --- | --- | --- | --- | --- | --- | --- | --- | --- | --- | --- | --- | --- | --- | --- | --- | --- | --- | --- | --- | --- | --- | --- | --- | --- | --- | --- | --- | --- | --- | --- | --- | --- | --- | --- | --- | --- | --- | --- | --- | --- | --- | --- | --- | --- | --- | --- | --- | --- | --- | --- | --- | --- | --- | --- | --- | --- | --- | --- | --- | --- | --- | --- | --- | --- | --- | --- | --- | --- | --- | --- | --- | --- | --- | --- | --- | --- | --- | --- | --- | --- | --- | --- | --- | --- | --- | --- | --- | --- | --- | --- | --- | --- | --- | --- | --- | --- | --- | --- | --- | --- | --- | --- | --- | --- | --- | --- | --- | --- | --- | --- | --- | --- | --- | --- | --- | --- | --- | --- | --- | --- | --- | --- | --- | --- | --- | --- | --- | --- | --- | --- | --- | --- | --- | --- | --- | --- | --- | --- | --- | --- | --- | --- | --- | --- | --- | --- | --- | --- | --- | --- | --- | --- | --- | --- | --- | --- | --- | --- | --- | --- | --- | --- | --- | --- | --- | --- | --- | --- | --- | --- | --- | --- | --- | --- | --- | --- | --- | --- | --- | --- | --- | --- | --- | --- | --- | --- | --- | --- | --- | --- | --- | --- | --- | --- | --- | --- | --- | --- | --- | --- | --- | --- | --- | --- | --- | --- | --- | --- | --- | --- | --- | --- | --- | --- | --- | --- | --- | --- | --- | --- | --- | --- | --- | --- | --- | --- | --- | --- | --- | --- | --- | --- | --- | --- | --- | --- | --- | --- | --- | --- | --- | --- | --- | --- | --- | --- | --- | --- | --- | --- | --- | --- | --- | --- | --- | --- | --- | --- | --- | --- | --- | --- | --- | --- | --- | --- | --- | --- | --- | --- | --- | --- | --- | --- | --- | --- | --- | --- | --- | --- | --- | --- | --- | --- | --- | --- | --- | --- | --- | --- | --- | --- | --- | --- | --- | --- | --- | --- | --- | --- | --- | --- | --- | --- | --- | --- | --- | --- | --- | --- | --- | --- | --- | --- | --- | --- | --- | --- | --- | --- | --- | --- | --- | --- | --- | --- | --- | --- | --- | --- | --- | --- | --- | --- | --- | --- | --- | --- | --- | --- | --- | --- | --- | --- | --- | --- | --- | --- | --- | --- | --- | --- | --- | --- | --- | --- | --- | --- | --- | --- | --- | --- | --- | --- | --- | --- | --- | --- | --- | --- | --- | --- | --- | --- | --- | --- | --- | --- | --- | --- | --- | --- | --- | --- | --- | --- | --- | --- | --- | --- | --- | --- | --- | --- | --- | --- | --- | --- | --- | --- | --- | --- | --- | --- | --- | --- | --- | --- | --- | --- | --- | --- | --- | --- | --- | --- | --- | --- | --- | --- | --- | --- | --- | --- | --- | --- | --- | --- | --- | --- | --- | --- | --- | --- | --- | --- | --- | --- | --- | --- | --- | --- | --- | --- | --- | --- | --- | --- | --- | --- | --- | --- | --- | --- | --- | --- | --- | --- | --- | --- | --- | --- | --- | --- | --- | --- | --- | --- | --- | --- | --- | --- | --- | --- | --- | --- | --- | --- | --- | --- | --- | --- | --- | --- | --- | --- | --- | --- | --- | --- | --- | --- | --- | --- | --- | --- | --- | --- | --- | --- | --- | --- | --- | --- | --- | --- | --- | --- | --- | --- | --- | --- | --- | --- | --- | --- | --- | --- | --- | --- | --- | --- | --- | --- | --- | --- | --- | --- | --- | --- | --- | --- | --- | --- | --- | --- | --- | --- | --- | --- | --- | --- | --- | --- | --- | --- | --- | --- | --- | --- | --- | --- | --- | --- | --- | --- | --- | --- | --- | --- | --- | --- | --- | --- | --- | --- | --- | --- | --- | --- | --- | --- | --- | --- | --- | --- | --- | --- | --- | --- | --- | --- | --- | --- | --- | --- | --- | --- | --- | --- | --- | --- | --- | --- | --- | --- | --- | --- | --- | --- | --- | --- | --- | --- | --- | --- | --- | --- | --- | --- | --- | --- | --- | --- | --- | --- | --- | --- | --- | --- | --- | --- | --- | --- | --- | --- | --- | --- | --- | --- | --- | --- | --- | --- | --- | --- | --- | --- | --- | --- | --- | --- | --- | --- | --- | --- | --- | --- | --- | --- | --- | --- | --- | --- | --- | --- | --- | --- | --- | --- | --- | --- | --- | --- | --- | --- | --- | --- | --- | --- | --- | --- | --- | --- | --- | --- | --- | --- | --- | --- | --- | --- | --- | --- | --- | --- | --- | --- | --- | --- | --- | --- | --- | --- | --- | --- | --- | --- | --- | --- | --- | --- | --- | --- | --- | --- | --- | --- | --- | --- | --- | --- | --- | --- | --- | --- | --- | --- | --- | --- | --- | --- | --- | --- | --- | --- | --- | --- | --- | --- | --- | --- | --- | --- | --- | --- | --- | --- | --- | --- | --- | --- | --- | --- | --- | --- | --- | --- | --- | --- | --- | --- | --- | --- | --- | --- | --- | --- | --- | --- | --- | --- | --- | --- | --- | --- | --- | --- | --- | --- | --- | --- | --- | --- | --- | --- | --- | --- | --- | --- | --- | --- | --- | --- | --- | --- | --- | --- | --- | --- | --- | --- | --- | --- | --- | --- | --- | --- | --- | --- | --- | --- | --- | --- | --- | --- | --- | --- | --- | --- | --- | --- | --- | --- | --- | --- | --- | --- | --- | --- | --- | --- | --- | --- | --- | --- | --- | --- | --- | --- | --- | --- | --- | --- | --- | --- | --- | --- | --- | --- | --- | --- | --- | --- | --- | --- | --- | --- | --- | --- | --- | --- | --- | --- | --- | --- | --- | --- | --- | --- | --- | --- | --- | --- | --- | --- | --- | --- | --- | --- | --- | --- | --- | --- | --- | --- | --- | --- | --- | --- | --- | --- | --- | --- | --- | --- | --- | --- | --- | --- | --- | --- | --- | --- | --- | --- | --- | --- | --- | --- | --- | --- | --- | --- | --- | --- | --- | --- | --- | --- | --- | --- | --- | --- | --- | --- | --- | --- | --- | --- | --- | --- | --- | --- | --- | --- | --- | --- | --- | --- | --- | --- | --- | --- | --- | --- | --- | --- | --- | --- | --- | --- | --- | --- | --- | --- | --- | --- | --- | --- | --- | --- | --- | --- | --- | --- | --- | --- | --- | --- | --- | --- | --- | --- | --- | --- | --- | --- | --- | --- | --- | --- | --- | --- | --- | --- | --- | --- | --- | --- | --- | --- | --- | --- | --- | --- | --- | --- | --- | --- | --- | --- | --- | --- | --- | --- | --- | --- | --- | --- | --- | --- | --- | --- | --- | --- | --- | --- | --- | --- | --- | --- | --- | --- | --- | --- | --- | --- | --- | --- | --- | --- | --- | --- | --- | --- | --- | --- | --- | --- | --- | --- | --- | --- | --- | --- | --- | --- | --- | --- | --- | --- | --- | --- | --- | --- | --- | --- | --- | --- | --- | --- | --- | --- | --- | --- | --- | --- | --- | --- | --- | --- | --- | --- | --- | --- | --- | --- | --- | --- | --- | --- | --- | --- | --- | --- | --- | --- | --- | --- | --- | --- | --- | --- | --- | --- | --- | --- | --- | --- | --- | --- | --- | --- | --- | --- | --- | --- | --- | --- | --- | --- | --- | --- | --- | --- | --- | --- | --- | --- | --- | --- | --- | --- | --- | --- | --- | --- | --- | --- | --- | --- | --- | --- | --- | --- | --- | --- | --- | --- | --- | --- | --- | --- | --- | --- | --- | --- | --- | --- | --- | --- | --- | --- | --- | --- | --- | --- | --- | --- | --- | --- | --- | --- | --- | --- | --- | --- | --- | --- | --- | --- | --- | --- | --- | --- | --- | --- | --- | --- | --- | --- | --- | --- | --- | --- | --- | --- | --- | --- | --- | --- | --- | --- | --- | --- | --- | --- | --- | --- | --- | --- | --- | --- | --- | --- | --- | --- | --- | --- | --- | --- | --- | --- | --- | --- | --- | --- | --- | --- | --- | --- | --- | --- | --- | --- | --- | --- | --- | --- | --- | --- | --- | --- | --- | --- | --- | --- | --- | --- | --- | --- | --- | --- | --- | --- | --- | --- | --- | --- | --- | --- | --- | --- | --- | --- | --- | --- | --- | --- | --- | --- | --- | --- | --- | --- | --- | --- | --- | --- | --- | --- | --- | --- | --- | --- | --- | --- | --- | --- | --- | --- | --- | --- | --- | --- | --- | --- | --- | --- | --- | --- | --- | --- | --- | --- | --- | --- | --- | --- | --- | --- | --- | --- | --- | --- | --- | --- | --- | --- | --- | --- | --- | --- | --- | --- | --- | --- | --- | --- | --- | --- | --- | --- | --- | --- | --- | --- | --- | --- | --- | --- | --- | --- | --- | --- | --- | --- | --- | --- | --- | --- | --- | --- | --- | --- | --- | --- | --- | --- | --- | --- | --- | --- | --- | --- | --- | --- | --- | --- | --- | --- | --- | --- | --- | --- | --- | --- | --- | --- | --- | --- | --- | --- | --- | --- | --- | --- | --- | --- | --- | --- | --- | --- | --- | --- | --- | --- | --- | --- | --- |
| Actual | Predict  |  |  |  |  |  |  |  |  |  |  |  |  |  |  |  |  |  |  |  |  |  |  |  |  |  |  |  |  |  |  |  |  |  |  |  |  |  |  |  |  |  |  |  |  |  |  |  |  |  |  |  |  |  | | --- | --- | --- | --- | --- | --- | --- | --- | --- | --- | --- | --- | --- | --- | --- | --- | --- | --- | --- | --- | --- | --- | --- | --- | --- | --- | --- | --- | --- | --- | --- | --- | --- | --- | --- | --- | --- | --- | --- | --- | --- | --- | --- | --- | --- | --- | --- | --- | --- | --- | --- | --- | --- | |  | 8 | 9 | 11 | 12 | 20 | 27 | 33 | 34 | 42 | 43 | 53 | 58 | 81 | 82 | 86 | 93 | 96 | 108 | 127 | 131 | 133 | 136 | 138 | 140 | 143 | 144 | 145 | 148 | 158 | 160 | 161 | 163 | 166 | 176 | 181 | 182 | 190 | 195 | 200 | 203 | 213 | 214 | 215 | 216 | 222 | 225 | 227 | 228 | 236 | 240 | 271 | 274 | | 8 | 0 | 0 | 0 | 0 | 0 | 0 | 0 | 2 | 0 | 0 | 0 | 0 | 0 | 1 | 0 | 0 | 0 | 0 | 0 | 0 | 0 | 3 | 0 | 0 | 0 | 0 | 0 | 0 | 0 | 0 | 0 | 0 | 0 | 0 | 0 | 0 | 0 | 0 | 0 | 0 | 0 | 0 | 0 | 0 | 0 | 0 | 0 | 0 | 0 | 0 | 0 | 0 | | 9 | 0 | 0 | 0 | 0 | 0 | 0 | 0 | 0 | 0 | 0 | 0 | 0 | 0 | 0 | 0 | 0 | 0 | 0 | 10 | 0 | 0 | 0 | 0 | 0 | 0 | 0 | 0 | 0 | 0 | 0 | 0 | 0 | 0 | 0 | 0 | 0 | 0 | 0 | 0 | 0 | 0 | 0 | 0 | 0 | 0 | 0 | 0 | 0 | 0 | 0 | 0 | 0 | | 11 | 0 | 0 | 5 | 0 | 0 | 0 | 0 | 0 | 0 | 0 | 0 | 0 | 0 | 0 | 0 | 0 | 0 | 0 | 0 | 0 | 0 | 0 | 0 | 0 | 0 | 0 | 0 | 0 | 0 | 0 | 0 | 0 | 0 | 0 | 0 | 17 | 0 | 0 | 0 | 0 | 0 | 0 | 1 | 0 | 0 | 0 | 0 | 0 | 0 | 0 | 0 | 0 | | 12 | 0 | 0 | 0 | 0 | 0 | 0 | 0 | 3 | 0 | 0 | 0 | 0 | 0 | 0 | 0 | 0 | 0 | 0 | 0 | 0 | 0 | 0 | 0 | 0 | 0 | 0 | 0 | 0 | 0 | 0 | 0 | 0 | 0 | 0 | 0 | 4 | 0 | 0 | 2 | 0 | 0 | 0 | 18 | 0 | 0 | 0 | 0 | 0 | 0 | 0 | 0 | 0 | | 20 | 0 | 0 | 0 | 0 | 0 | 0 | 0 | 0 | 0 | 0 | 0 | 0 | 0 | 1 | 0 | 0 | 0 | 0 | 0 | 0 | 0 | 0 | 0 | 0 | 0 | 0 | 0 | 0 | 0 | 0 | 0 | 0 | 0 | 0 | 0 | 0 | 0 | 0 | 0 | 0 | 0 | 0 | 10 | 0 | 0 | 0 | 0 | 0 | 0 | 0 | 0 | 0 | | 27 | 0 | 0 | 0 | 0 | 0 | 13 | 0 | 0 | 0 | 5 | 0 | 0 | 0 | 0 | 0 | 0 | 0 | 0 | 2 | 0 | 0 | 0 | 0 | 0 | 0 | 0 | 0 | 0 | 0 | 0 | 0 | 0 | 0 | 0 | 0 | 0 | 0 | 0 | 0 | 0 | 0 | 0 | 7 | 0 | 0 | 0 | 0 | 0 | 0 | 0 | 0 | 0 | | 33 | 0 | 0 | 0 | 0 | 0 | 0 | 0 | 0 | 0 | 0 | 0 | 0 | 0 | 0 | 0 | 0 | 0 | 0 | 0 | 0 | 1 | 0 | 0 | 0 | 0 | 0 | 0 | 0 | 0 | 0 | 0 | 0 | 0 | 0 | 0 | 0 | 0 | 0 | 0 | 0 | 0 | 0 | 2 | 0 | 0 | 0 | 0 | 0 | 0 | 0 | 0 | 0 | | 34 | 0 | 0 | 0 | 0 | 0 | 0 | 0 | 22 | 0 | 0 | 0 | 0 | 0 | 0 | 0 | 0 | 0 | 0 | 0 | 0 | 0 | 0 | 0 | 0 | 0 | 0 | 0 | 0 | 0 | 0 | 0 | 0 | 0 | 0 | 0 | 0 | 0 | 0 | 0 | 0 | 0 | 0 | 0 | 0 | 0 | 0 | 0 | 0 | 0 | 0 | 0 | 0 | | 42 | 0 | 0 | 0 | 0 | 0 | 3 | 0 | 6 | 0 | 0 | 0 | 0 | 0 | 0 | 0 | 0 | 0 | 0 | 0 | 0 | 0 | 0 | 0 | 0 | 0 | 0 | 0 | 0 | 0 | 0 | 0 | 0 | 0 | 0 | 0 | 0 | 0 | 0 | 0 | 0 | 0 | 0 | 0 | 0 | 0 | 0 | 0 | 0 | 0 | 0 | 0 | 0 | | 43 | 0 | 0 | 0 | 0 | 0 | 0 | 0 | 0 | 0 | 26 | 5 | 0 | 0 | 0 | 0 | 0 | 0 | 0 | 0 | 0 | 0 | 0 | 0 | 0 | 0 | 0 | 0 | 0 | 0 | 0 | 0 | 0 | 0 | 0 | 0 | 0 | 0 | 0 | 0 | 0 | 0 | 0 | 0 | 0 | 0 | 0 | 0 | 0 | 0 | 0 | 0 | 0 | | 53 | 0 | 0 | 0 | 0 | 0 | 0 | 0 | 0 | 0 | 18 | 0 | 0 | 0 | 0 | 0 | 0 | 0 | 0 | 0 | 0 | 0 | 11 | 0 | 0 | 0 | 0 | 0 | 0 | 0 | 0 | 0 | 0 | 0 | 0 | 0 | 0 | 0 | 0 | 0 | 0 | 0 | 0 | 7 | 0 | 0 | 0 | 0 | 0 | 0 | 0 | 0 | 0 | | 58 | 0 | 0 | 0 | 0 | 0 | 0 | 0 | 6 | 0 | 0 | 0 | 0 | 0 | 0 | 0 | 0 | 0 | 0 | 0 | 0 | 0 | 2 | 0 | 0 | 0 | 0 | 0 | 0 | 0 | 0 | 0 | 0 | 0 | 0 | 0 | 0 | 0 | 0 | 0 | 0 | 0 | 0 | 0 | 0 | 0 | 0 | 0 | 0 | 0 | 0 | 0 | 0 | | 81 | 0 | 0 | 0 | 0 | 0 | 0 | 0 | 104 | 0 | 0 | 0 | 0 | 0 | 0 | 0 | 0 | 0 | 0 | 0 | 0 | 0 | 0 | 0 | 0 | 0 | 0 | 0 | 0 | 0 | 0 | 0 | 0 | 0 | 0 | 0 | 0 | 0 | 0 | 0 | 0 | 0 | 0 | 0 | 0 | 0 | 0 | 0 | 0 | 0 | 0 | 0 | 0 | | 82 | 0 | 0 | 0 | 0 | 0 | 0 | 0 | 1 | 0 | 0 | 0 | 0 | 0 | 1 | 0 | 0 | 0 | 0 | 0 | 0 | 0 | 0 | 0 | 0 | 0 | 0 | 0 | 0 | 0 | 0 | 0 | 0 | 0 | 0 | 0 | 0 | 0 | 0 | 0 | 0 | 0 | 0 | 2 | 0 | 0 | 0 | 0 | 0 | 0 | 0 | 0 | 0 | | 86 | 0 | 0 | 13 | 0 | 0 | 0 | 0 | 1 | 0 | 0 | 0 | 0 | 0 | 0 | 0 | 0 | 0 | 0 | 0 | 0 | 0 | 0 | 0 | 0 | 0 | 0 | 0 | 0 | 0 | 0 | 0 | 0 | 0 | 0 | 0 | 0 | 0 | 1 | 0 | 0 | 0 | 0 | 0 | 0 | 0 | 0 | 0 | 0 | 0 | 0 | 0 | 0 | | 93 | 0 | 0 | 0 | 0 | 0 | 0 | 0 | 0 | 0 | 0 | 0 | 0 | 0 | 0 | 0 | 0 | 0 | 0 | 0 | 0 | 0 | 0 | 0 | 0 | 0 | 0 | 0 | 0 | 0 | 0 | 0 | 0 | 0 | 0 | 8 | 0 | 0 | 0 | 0 | 0 | 0 | 0 | 0 | 0 | 0 | 0 | 0 | 0 | 0 | 0 | 0 | 0 | | 96 | 0 | 0 | 0 | 0 | 0 | 0 | 0 | 0 | 0 | 0 | 0 | 0 | 0 | 0 | 0 | 0 | 0 | 0 | 0 | 0 | 0 | 0 | 0 | 0 | 0 | 0 | 0 | 0 | 0 | 0 | 0 | 0 | 0 | 0 | 0 | 0 | 0 | 0 | 0 | 0 | 0 | 0 | 9 | 0 | 0 | 0 | 0 | 0 | 0 | 0 | 0 | 0 | | 108 | 0 | 0 | 0 | 0 | 0 | 0 | 0 | 17 | 0 | 0 | 0 | 0 | 0 | 0 | 0 | 0 | 0 | 0 | 0 | 0 | 0 | 0 | 0 | 0 | 0 | 0 | 0 | 0 | 0 | 0 | 0 | 0 | 0 | 0 | 0 | 0 | 0 | 0 | 0 | 0 | 0 | 0 | 0 | 0 | 0 | 0 | 0 | 0 | 0 | 0 | 0 | 0 | | 127 | 0 | 0 | 0 | 0 | 0 | 22 | 0 | 0 | 0 | 0 | 0 | 0 | 0 | 0 | 0 | 0 | 0 | 0 | 40 | 0 | 0 | 0 | 0 | 0 | 0 | 0 | 0 | 0 | 0 | 0 | 0 | 0 | 0 | 0 | 0 | 0 | 0 | 71 | 0 | 0 | 0 | 0 | 8 | 0 | 0 | 0 | 0 | 0 | 0 | 0 | 0 | 0 | | 131 | 0 | 0 | 0 | 0 | 0 | 0 | 0 | 0 | 0 | 0 | 0 | 0 | 0 | 0 | 0 | 0 | 0 | 0 | 0 | 0 | 0 | 0 | 0 | 0 | 0 | 0 | 0 | 0 | 0 | 0 | 0 | 0 | 0 | 0 | 0 | 0 | 0 | 0 | 0 | 0 | 0 | 0 | 23 | 0 | 0 | 0 | 0 | 0 | 0 | 0 | 0 | 0 | | 133 | 0 | 0 | 0 | 0 | 0 | 8 | 0 | 0 | 0 | 0 | 0 | 0 | 0 | 0 | 0 | 0 | 0 | 0 | 0 | 0 | 0 | 0 | 0 | 0 | 0 | 0 | 0 | 0 | 0 | 0 | 0 | 0 | 0 | 0 | 0 | 0 | 0 | 0 | 0 | 0 | 0 | 0 | 45 | 0 | 0 | 0 | 0 | 0 | 0 | 0 | 0 | 0 | | 136 | 0 | 0 | 13 | 0 | 0 | 0 | 0 | 0 | 0 | 0 | 1 | 0 | 0 | 0 | 0 | 0 | 0 | 0 | 0 | 0 | 0 | 15 | 0 | 0 | 0 | 0 | 0 | 0 | 0 | 0 | 0 | 0 | 0 | 0 | 0 | 0 | 0 | 0 | 0 | 0 | 0 | 0 | 0 | 0 | 0 | 0 | 0 | 0 | 0 | 0 | 0 | 0 | | 138 | 0 | 0 | 0 | 0 | 0 | 4 | 0 | 6 | 0 | 0 | 0 | 0 | 0 | 0 | 0 | 0 | 0 | 0 | 1 | 0 | 0 | 0 | 0 | 0 | 0 | 0 | 0 | 0 | 0 | 0 | 0 | 0 | 0 | 0 | 0 | 0 | 0 | 4 | 0 | 0 | 0 | 0 | 0 | 0 | 0 | 0 | 0 | 0 | 0 | 0 | 0 | 0 | | 140 | 0 | 0 | 0 | 0 | 0 | 0 | 0 | 4 | 0 | 0 | 0 | 0 | 0 | 1 | 0 | 0 | 0 | 0 | 0 | 0 | 0 | 0 | 0 | 0 | 0 | 0 | 0 | 0 | 0 | 0 | 0 | 0 | 0 | 0 | 0 | 0 | 0 | 0 | 0 | 0 | 0 | 0 | 0 | 0 | 0 | 0 | 0 | 0 | 0 | 0 | 0 | 0 | | 143 | 0 | 0 | 1 | 0 | 0 | 0 | 0 | 3 | 0 | 0 | 0 | 0 | 0 | 0 | 0 | 0 | 0 | 0 | 0 | 0 | 0 | 0 | 0 | 0 | 0 | 0 | 0 | 0 | 0 | 0 | 0 | 0 | 0 | 0 | 0 | 0 | 0 | 0 | 0 | 0 | 0 | 0 | 0 | 0 | 0 | 0 | 0 | 0 | 0 | 0 | 0 | 0 | | 144 | 0 | 0 | 0 | 0 | 0 | 0 | 0 | 2 | 0 | 0 | 0 | 0 | 0 | 0 | 0 | 0 | 0 | 0 | 0 | 0 | 0 | 0 | 0 | 0 | 0 | 0 | 0 | 0 | 0 | 0 | 0 | 0 | 0 | 0 | 0 | 0 | 0 | 0 | 1 | 0 | 0 | 0 | 0 | 0 | 0 | 0 | 0 | 0 | 0 | 0 | 0 | 0 | | 145 | 0 | 0 | 0 | 0 | 0 | 0 | 0 | 1 | 0 | 0 | 3 | 0 | 0 | 0 | 0 | 0 | 0 | 0 | 0 | 0 | 0 | 0 | 0 | 0 | 0 | 0 | 0 | 0 | 0 | 0 | 0 | 0 | 0 | 0 | 0 | 0 | 0 | 0 | 0 | 0 | 0 | 0 | 0 | 0 | 0 | 0 | 0 | 0 | 0 | 0 | 0 | 0 | | 148 | 0 | 0 | 0 | 0 | 0 | 1 | 0 | 0 | 0 | 0 | 0 | 0 | 0 | 0 | 0 | 0 | 0 | 0 | 0 | 0 | 0 | 0 | 0 | 0 | 0 | 0 | 0 | 0 | 0 | 0 | 0 | 0 | 0 | 0 | 0 | 0 | 0 | 0 | 0 | 0 | 0 | 0 | 6 | 0 | 0 | 0 | 0 | 0 | 0 | 0 | 0 | 0 | | 158 | 0 | 0 | 0 | 0 | 0 | 0 | 0 | 2 | 0 | 0 | 0 | 0 | 0 | 0 | 0 | 0 | 0 | 0 | 0 | 0 | 0 | 0 | 0 | 0 | 0 | 0 | 0 | 0 | 0 | 0 | 0 | 0 | 0 | 0 | 0 | 10 | 0 | 0 | 0 | 0 | 0 | 0 | 3 | 0 | 0 | 0 | 0 | 0 | 0 | 0 | 0 | 0 | | 160 | 0 | 0 | 0 | 0 | 0 | 0 | 0 | 0 | 0 | 0 | 0 | 0 | 0 | 0 | 0 | 0 | 0 | 0 | 0 | 0 | 0 | 0 | 0 | 0 | 0 | 0 | 0 | 0 | 0 | 0 | 0 | 0 | 0 | 0 | 0 | 6 | 0 | 0 | 0 | 0 | 0 | 0 | 1 | 0 | 0 | 0 | 0 | 0 | 0 | 0 | 0 | 0 | | 161 | 0 | 0 | 0 | 0 | 0 | 0 | 0 | 4 | 0 | 0 | 0 | 0 | 0 | 0 | 0 | 0 | 0 | 0 | 0 | 0 | 0 | 0 | 0 | 0 | 0 | 0 | 0 | 0 | 0 | 0 | 0 | 0 | 0 | 0 | 0 | 0 | 0 | 3 | 0 | 0 | 0 | 0 | 0 | 0 | 0 | 0 | 0 | 0 | 0 | 0 | 0 | 0 | | 163 | 0 | 0 | 3 | 0 | 0 | 0 | 0 | 3 | 0 | 0 | 0 | 0 | 0 | 0 | 0 | 0 | 0 | 0 | 4 | 0 | 0 | 0 | 0 | 0 | 0 | 0 | 0 | 0 | 0 | 0 | 0 | 11 | 0 | 0 | 0 | 0 | 0 | 0 | 0 | 0 | 0 | 0 | 0 | 0 | 0 | 0 | 0 | 0 | 0 | 0 | 0 | 0 | | 166 | 0 | 0 | 0 | 0 | 0 | 0 | 0 | 0 | 0 | 0 | 0 | 0 | 0 | 0 | 0 | 0 | 0 | 0 | 0 | 0 | 0 | 0 | 0 | 0 | 0 | 0 | 0 | 0 | 0 | 0 | 0 | 0 | 7 | 0 | 0 | 0 | 0 | 0 | 0 | 0 | 0 | 0 | 0 | 0 | 0 | 0 | 0 | 0 | 0 | 0 | 0 | 0 | | 176 | 0 | 0 | 0 | 0 | 0 | 0 | 0 | 0 | 0 | 0 | 1 | 0 | 0 | 0 | 0 | 0 | 0 | 0 | 0 | 0 | 0 | 0 | 0 | 0 | 0 | 0 | 0 | 0 | 0 | 0 | 0 | 1 | 0 | 2 | 0 | 3 | 0 | 0 | 0 | 0 | 0 | 0 | 0 | 0 | 0 | 0 | 0 | 0 | 0 | 0 | 0 | 0 | | 181 | 0 | 0 | 0 | 0 | 0 | 0 | 0 | 0 | 0 | 0 | 0 | 0 | 0 | 0 | 0 | 0 | 0 | 0 | 0 | 0 | 0 | 0 | 0 | 0 | 0 | 0 | 0 | 0 | 0 | 0 | 0 | 0 | 0 | 0 | 9 | 0 | 0 | 0 | 0 | 0 | 0 | 0 | 0 | 0 | 0 | 0 | 0 | 0 | 0 | 0 | 0 | 0 | | 182 | 0 | 0 | 0 | 0 | 0 | 0 | 0 | 26 | 1 | 0 | 0 | 0 | 0 | 9 | 0 | 0 | 0 | 0 | 0 | 0 | 0 | 0 | 0 | 0 | 0 | 0 | 0 | 0 | 0 | 0 | 0 | 0 | 0 | 0 | 0 | 35 | 0 | 0 | 0 | 0 | 0 | 0 | 3 | 0 | 0 | 0 | 0 | 0 | 0 | 0 | 0 | 0 | | 190 | 0 | 0 | 0 | 0 | 0 | 0 | 0 | 10 | 0 | 0 | 0 | 0 | 0 | 0 | 0 | 0 | 0 | 0 | 0 | 0 | 0 | 0 | 0 | 0 | 0 | 0 | 0 | 0 | 0 | 0 | 0 | 0 | 0 | 0 | 0 | 0 | 0 | 0 | 0 | 0 | 0 | 0 | 0 | 0 | 0 | 0 | 0 | 0 | 0 | 0 | 0 | 0 | | 195 | 0 | 0 | 1 | 0 | 0 | 0 | 0 | 14 | 0 | 0 | 0 | 0 | 0 | 0 | 0 | 0 | 0 | 0 | 0 | 0 | 0 | 0 | 0 | 0 | 0 | 0 | 0 | 0 | 0 | 0 | 0 | 0 | 0 | 0 | 0 | 1 | 0 | 0 | 0 | 0 | 0 | 0 | 8 | 0 | 0 | 0 | 0 | 0 | 0 | 0 | 0 | 0 | | 200 | 0 | 0 | 1 | 0 | 0 | 0 | 0 | 0 | 0 | 0 | 0 | 0 | 0 | 0 | 0 | 0 | 0 | 0 | 0 | 0 | 0 | 0 | 0 | 0 | 0 | 0 | 0 | 0 | 0 | 0 | 0 | 0 | 0 | 0 | 0 | 0 | 0 | 0 | 0 | 0 | 0 | 0 | 5 | 0 | 0 | 0 | 0 | 0 | 0 | 0 | 0 | 0 | | 203 | 0 | 0 | 0 | 0 | 0 | 0 | 0 | 6 | 0 | 0 | 0 | 0 | 0 | 0 | 0 | 0 | 0 | 0 | 0 | 0 | 0 | 0 | 0 | 0 | 0 | 0 | 0 | 0 | 0 | 0 | 0 | 0 | 0 | 0 | 0 | 0 | 0 | 0 | 0 | 0 | 0 | 0 | 0 | 0 | 0 | 0 | 0 | 0 | 0 | 0 | 0 | 0 | | 213 | 0 | 0 | 0 | 0 | 0 | 0 | 0 | 18 | 0 | 0 | 0 | 0 | 0 | 0 | 0 | 0 | 0 | 0 | 0 | 0 | 3 | 0 | 0 | 0 | 0 | 0 | 0 | 0 | 0 | 0 | 0 | 0 | 0 | 0 | 0 | 0 | 0 | 0 | 0 | 0 | 0 | 0 | 3 | 0 | 0 | 0 | 0 | 0 | 0 | 0 | 0 | 0 | | 214 | 0 | 0 | 0 | 0 | 0 | 0 | 0 | 1 | 0 | 0 | 0 | 0 | 0 | 0 | 0 | 0 | 0 | 0 | 0 | 0 | 0 | 3 | 0 | 0 | 0 | 0 | 0 | 0 | 0 | 0 | 0 | 0 | 0 | 0 | 0 | 0 | 0 | 0 | 0 | 0 | 0 | 0 | 0 | 0 | 0 | 0 | 0 | 0 | 0 | 0 | 0 | 0 | | 215 | 0 | 0 | 0 | 0 | 0 | 0 | 0 | 17 | 0 | 0 | 0 | 0 | 17 | 0 | 0 | 0 | 0 | 0 | 0 | 0 | 0 | 0 | 0 | 0 | 0 | 0 | 0 | 0 | 0 | 0 | 0 | 0 | 0 | 1 | 0 | 0 | 0 | 0 | 0 | 0 | 0 | 0 | 8 | 0 | 0 | 0 | 0 | 0 | 0 | 0 | 0 | 0 | | 216 | 0 | 0 | 0 | 0 | 0 | 0 | 0 | 0 | 0 | 0 | 0 | 0 | 0 | 0 | 0 | 0 | 0 | 0 | 0 | 0 | 0 | 0 | 0 | 0 | 0 | 0 | 0 | 0 | 0 | 0 | 0 | 0 | 0 | 0 | 0 | 0 | 0 | 0 | 0 | 0 | 0 | 0 | 18 | 0 | 0 | 0 | 0 | 0 | 0 | 0 | 0 | 0 | | 222 | 0 | 0 | 0 | 0 | 0 | 7 | 0 | 0 | 0 | 0 | 0 | 0 | 0 | 0 | 0 | 0 | 0 | 0 | 0 | 0 | 0 | 0 | 0 | 0 | 0 | 0 | 0 | 0 | 0 | 0 | 0 | 0 | 0 | 0 | 0 | 0 | 0 | 0 | 0 | 0 | 0 | 0 | 3 | 0 | 0 | 0 | 0 | 0 | 0 | 0 | 0 | 0 | | 225 | 0 | 0 | 0 | 0 | 0 | 0 | 0 | 0 | 0 | 0 | 0 | 0 | 0 | 0 | 0 | 0 | 0 | 0 | 0 | 0 | 0 | 0 | 0 | 0 | 0 | 0 | 0 | 0 | 0 | 0 | 0 | 0 | 0 | 0 | 0 | 0 | 0 | 0 | 0 | 0 | 0 | 0 | 1 | 0 | 0 | 6 | 0 | 0 | 0 | 0 | 0 | 0 | | 227 | 0 | 0 | 0 | 0 | 0 | 0 | 0 | 0 | 0 | 0 | 0 | 0 | 0 | 0 | 0 | 0 | 0 | 0 | 0 | 0 | 0 | 0 | 0 | 0 | 0 | 0 | 0 | 0 | 0 | 0 | 0 | 0 | 0 | 2 | 0 | 0 | 0 | 0 | 0 | 0 | 0 | 0 | 0 | 0 | 0 | 0 | 0 | 0 | 0 | 0 | 0 | 0 | | 228 | 0 | 0 | 1 | 0 | 0 | 0 | 0 | 4 | 0 | 0 | 0 | 0 | 0 | 2 | 0 | 0 | 0 | 0 | 0 | 0 | 0 | 0 | 0 | 0 | 0 | 0 | 0 | 0 | 0 | 0 | 0 | 0 | 0 | 0 | 0 | 0 | 0 | 0 | 0 | 0 | 0 | 0 | 0 | 0 | 0 | 0 | 0 | 0 | 0 | 0 | 0 | 0 | | 236 | 0 | 0 | 2 | 0 | 0 | 0 | 0 | 4 | 0 | 0 | 0 | 0 | 0 | 0 | 0 | 0 | 0 | 0 | 0 | 0 | 0 | 0 | 0 | 0 | 0 | 0 | 0 | 0 | 0 | 0 | 0 | 0 | 0 | 0 | 0 | 0 | 0 | 0 | 0 | 0 | 0 | 0 | 0 | 0 | 0 | 0 | 0 | 0 | 0 | 0 | 0 | 0 | | 240 | 0 | 0 | 1 | 0 | 0 | 0 | 0 | 3 | 0 | 0 | 0 | 0 | 0 | 0 | 0 | 0 | 0 | 0 | 21 | 0 | 0 | 3 | 0 | 0 | 0 | 0 | 0 | 0 | 0 | 0 | 0 | 1 | 0 | 0 | 0 | 0 | 0 | 0 | 0 | 0 | 0 | 0 | 0 | 0 | 0 | 0 | 0 | 0 | 0 | 0 | 0 | 0 | | 271 | 0 | 0 | 0 | 0 | 0 | 0 | 0 | 0 | 0 | 0 | 0 | 0 | 0 | 0 | 0 | 0 | 0 | 0 | 0 | 0 | 0 | 0 | 0 | 0 | 0 | 0 | 0 | 0 | 0 | 0 | 0 | 0 | 0 | 0 | 0 | 0 | 0 | 0 | 15 | 0 | 0 | 0 | 0 | 0 | 0 | 0 | 0 | 0 | 0 | 0 | 3 | 0 | | 274 | 0 | 0 | 0 | 0 | 0 | 0 | 0 | 7 | 0 | 0 | 0 | 0 | 0 | 0 | 0 | 0 | 0 | 0 | 0 | 0 | 0 | 0 | 0 | 0 | 0 | 0 | 0 | 0 | 0 | 0 | 4 | 0 | 0 | 0 | 0 | 0 | 0 | 0 | 0 | 0 | 0 | 0 | 0 | 0 | 0 | 0 | 0 | 0 | 0 | 0 | 0 | 0 | |

## Overall Statistics :

|  |  |
| --- | --- |
| 95% CI | (0.1735,0.2222) |
| ACC Macro | 0.9691 |
| ARI | 0.2259 |
| AUNP | 0.5836 |
| AUNU | 0.5697 |
| Bangdiwala B | 0.1149 |
| Bennett S | 0.1821 |
| CBA | 0.1071 |
| CSI | None |
| Chi-Squared | None |
| Chi-Squared DF | 2601 |
| Conditional Entropy | 0.9783 |
| Cramer V | None |
| Cross Entropy | 3.4178 |
| F1 Macro | 0.1268 |
| F1 Micro | 0.1979 |
| FNR Macro | 0.8446 |
| FNR Micro | 0.8021 |
| FPR Macro | 0.016 |
| FPR Micro | 0.0157 |
| Gwet AC1 | 0.1829 |
| Hamming Loss | 0.8021 |
| Joint Entropy | 5.9557 |
| KL Divergence | None |
| Kappa | 0.1649 |
| Kappa 95% CI | (0.1395,0.1903) |
| Kappa No Prevalence | -0.6043 |
| Kappa Standard Error | 0.0129 |
| Kappa Unbiased | 0.1388 |
| Krippendorff Alpha | 0.1392 |
| Lambda A | 0.3638 |
| Lambda B | 0.5857 |
| Mutual Information | 2.3979 |
| NIR | 0.1374 |
| Overall ACC | 0.1979 |
| Overall CEN | 0.2887 |
| Overall J | (4.9652,0.0955) |
| Overall MCC | 0.1758 |
| Overall MCEN | 0.3058 |
| Overall RACC | 0.0394 |
| Overall RACCU | 0.0686 |
| P-Value | 0.0 |
| PPV Macro | None |
| PPV Micro | 0.1979 |
| Pearson C | None |
| Phi-Squared | None |
| RCI | 0.4818 |
| RR | 19.7308 |
| Reference Entropy | 4.9773 |
| Response Entropy | 3.3763 |
| SOA1(Landis & Koch) | Slight |
| SOA2(Fleiss) | Poor |
| SOA3(Altman) | Poor |
| SOA4(Cicchetti) | Poor |
| SOA5(Cramer) | None |
| SOA6(Matthews) | Negligible |
| Scott PI | 0.1388 |
| Standard Error | 0.0124 |
| TNR Macro | 0.984 |
| TNR Micro | 0.9843 |
| TPR Macro | 0.1554 |
| TPR Micro | 0.1979 |
| Zero-one Loss | 823 |

## Class Statistics :

|  |  |  |  |  |  |  |  |  |  |  |  |  |  |  |  |  |  |  |  |  |  |  |  |  |  |  |  |  |  |  |  |  |  |  |  |  |  |  |  |  |  |  |  |  |  |  |  |  |  |  |  |  |  |
| --- | --- | --- | --- | --- | --- | --- | --- | --- | --- | --- | --- | --- | --- | --- | --- | --- | --- | --- | --- | --- | --- | --- | --- | --- | --- | --- | --- | --- | --- | --- | --- | --- | --- | --- | --- | --- | --- | --- | --- | --- | --- | --- | --- | --- | --- | --- | --- | --- | --- | --- | --- | --- | --- |
| Class | 8 | 9 | 11 | 12 | 20 | 27 | 33 | 34 | 42 | 43 | 53 | 58 | 81 | 82 | 86 | 93 | 96 | 108 | 127 | 131 | 133 | 136 | 138 | 140 | 143 | 144 | 145 | 148 | 158 | 160 | 161 | 163 | 166 | 176 | 181 | 182 | 190 | 195 | 200 | 203 | 213 | 214 | 215 | 216 | 222 | 225 | 227 | 228 | 236 | 240 | 271 | 274 | Description |
| ACC | 0.9942 | 0.9903 | 0.9474 | 0.9737 | 0.9893 | 0.9425 | 0.9971 | 0.732 | 0.9903 | 0.9727 | 0.9552 | 0.9922 | 0.8821 | 0.9834 | 0.9854 | 0.9922 | 0.9912 | 0.9834 | 0.8645 | 0.9776 | 0.9444 | 0.9649 | 0.9854 | 0.9951 | 0.9961 | 0.9971 | 0.9961 | 0.9932 | 0.9854 | 0.9932 | 0.9893 | 0.9883 | 1.0 | 0.9922 | 0.9922 | 0.922 | 0.9903 | 0.8996 | 0.9766 | 0.9942 | 0.9766 | 0.9961 | 0.7875 | 0.9825 | 0.9903 | 0.999 | 0.9981 | 0.9932 | 0.9942 | 0.9717 | 0.9854 | 0.9893 | Accuracy |
| AGF | 0.0 | 0.0 | 0.4288 | 0.0 | 0.0 | 0.6192 | 0.0 | 0.5154 | 0.0 | 0.863 | 0.0 | 0.0 | 0.0 | 0.4006 | 0.0 | 0.0 | 0.0 | 0.0 | 0.5311 | 0.0 | 0.0 | 0.6946 | 0.0 | 0.0 | 0.0 | 0.0 | 0.0 | 0.0 | 0.0 | 0.0 | 0.0 | 0.7499 | 1.0 | 0.5492 | 0.9207 | 0.6715 | 0.0 | 0.0 | 0.0 | 0.0 | 0.0 | 0.0 | 0.3193 | 0.0 | 0.0 | 0.939 | 0.0 | 0.0 | 0.0 | 0.0 | 0.4446 | 0.0 | Adjusted F-score |
| AGM | 0 | 0 | 0.7081 | 0 | 0 | 0.8147 | 0 | 0.7898 | 0 | 0.9405 | 0 | 0 | 0 | 0.741 | 0 | 0 | 0 | 0 | 0.723 | 0 | 0 | 0.8427 | 0 | 0 | 0 | 0 | 0 | 0 | 0 | 0 | 0 | 0.8591 | 1.0 | 0.7646 | 0.9941 | 0.8095 | 0 | 0 | 0 | 0 | 0 | 0 | 0.5969 | 0 | 0 | 0.9628 | 0 | 0 | 0 | 0 | 0.7015 | 0 | Adjusted geometric mean |
| AM | -6 | -10 | 18 | -27 | -11 | 31 | -3 | 275 | -8 | 18 | -26 | -8 | -87 | 11 | -15 | -8 | -9 | -17 | -63 | -23 | -49 | 8 | -15 | -5 | -4 | -3 | -4 | -7 | -15 | -7 | -3 | -8 | 0 | -2 | 8 | 2 | -10 | 55 | 12 | -6 | -24 | -4 | 148 | -18 | -10 | -1 | -2 | -7 | -6 | -29 | -15 | -11 | Difference between automatic and manual classification |
| AUC | 0.5 | 0.5 | 0.5907 | 0.5 | 0.5 | 0.7182 | 0.5 | 0.863 | 0.4995 | 0.9078 | 0.4949 | 0.5 | 0.4908 | 0.6182 | 0.5 | 0.5 | 0.5 | 0.5 | 0.6204 | 0.5 | 0.4979 | 0.7476 | 0.5 | 0.5 | 0.5 | 0.5 | 0.5 | 0.5 | 0.5 | 0.5 | 0.498 | 0.7609 | 1.0 | 0.6414 | 0.9961 | 0.715 | 0.5 | 0.4606 | 0.4912 | 0.5 | 0.5 | 0.5 | 0.4999 | 0.5 | 0.5 | 0.9286 | 0.5 | 0.5 | 0.5 | 0.5 | 0.5833 | 0.5 | Area under the ROC curve |
| AUCI | Poor | Poor | Poor | Poor | Poor | Good | Poor | Very Good | Poor | Excellent | Poor | Poor | Poor | Fair | Poor | Poor | Poor | Poor | Fair | Poor | Poor | Good | Poor | Poor | Poor | Poor | Poor | Poor | Poor | Poor | Poor | Good | Excellent | Fair | Excellent | Good | Poor | Poor | Poor | Poor | Poor | Poor | Poor | Poor | Poor | Excellent | Poor | Poor | Poor | Poor | Poor | Poor | AUC value interpretation |
| AUPR | None | None | 0.1697 | None | None | 0.3528 | None | 0.537 | 0.0 | 0.6847 | 0.0 | None | 0.0 | 0.1583 | None | None | None | None | 0.3983 | None | 0.0 | 0.4613 | None | None | None | None | None | None | None | None | 0.0 | 0.685 | 1.0 | 0.3429 | 0.7647 | 0.4667 | None | 0.0 | 0.0 | None | None | None | 0.114 | None | None | 0.9286 | None | None | None | None | 0.5833 | None | Area under the PR curve |
| BCD | 0.0029 | 0.0049 | 0.0088 | 0.0132 | 0.0054 | 0.0151 | 0.0015 | 0.134 | 0.0039 | 0.0088 | 0.0127 | 0.0039 | 0.0424 | 0.0054 | 0.0073 | 0.0039 | 0.0044 | 0.0083 | 0.0307 | 0.0112 | 0.0239 | 0.0039 | 0.0073 | 0.0024 | 0.0019 | 0.0015 | 0.0019 | 0.0034 | 0.0073 | 0.0034 | 0.0015 | 0.0039 | 0.0 | 0.001 | 0.0039 | 0.001 | 0.0049 | 0.0268 | 0.0058 | 0.0029 | 0.0117 | 0.0019 | 0.0721 | 0.0088 | 0.0049 | 0.0005 | 0.001 | 0.0034 | 0.0029 | 0.0141 | 0.0073 | 0.0054 | Bray-Curtis dissimilarity |
| BM | 0.0 | 0.0 | 0.1815 | 0.0 | 0.0 | 0.4364 | 0.0 | 0.7261 | -0.001 | 0.8156 | -0.0101 | 0.0 | -0.0184 | 0.2363 | 0.0 | 0.0 | 0.0 | 0.0 | 0.2408 | 0.0 | -0.0041 | 0.4952 | 0.0 | 0.0 | 0.0 | 0.0 | 0.0 | 0.0 | 0.0 | 0.0 | -0.0039 | 0.5218 | 1.0 | 0.2828 | 0.9921 | 0.4299 | 0.0 | -0.0788 | -0.0176 | 0.0 | 0.0 | 0.0 | -0.0001 | 0.0 | 0.0 | 0.8571 | 0.0 | 0.0 | 0.0 | 0.0 | 0.1667 | 0.0 | Informedness or bookmaker informedness |
| CEN | 0.2187 | 0.0 | 0.3549 | 0.2141 | 0.0659 | 0.3357 | 0.1376 | 0.4834 | 0.1942 | 0.1475 | 0.342 | 0.1216 | 0.0878 | 0.313 | 0.1049 | 0.0 | 0.0 | 0.0 | 0.2645 | 0.0 | 0.1488 | 0.2615 | 0.2707 | 0.1082 | 0.1216 | 0.1376 | 0.1216 | 0.0887 | 0.1861 | 0.0887 | 0.2357 | 0.1919 | 0 | 0.2738 | 0.0784 | 0.2916 | 0.0 | 0.2357 | 0.2362 | 0.0 | 0.1591 | 0.1216 | 0.5612 | 0.0 | 0.1321 | 0.0427 | 0.0 | 0.2066 | 0.1376 | 0.2022 | 0.052 | 0.1417 | Confusion entropy |
| DOR | None | None | 7.4614 | None | None | 19.6857 | None | None | 0.0 | 219.7565 | 0.0 | None | 0.0 | 24.0 | None | None | None | None | 8.8275 | None | 0.0 | 47.4838 | None | None | None | None | None | None | None | None | 0.0 | 551.65 | None | 135.4667 | None | 19.9406 | None | 0.0 | 0.0 | None | None | None | 0.9992 | None | None | None | None | None | None | None | None | None | Diagnostic odds ratio |
| DP | None | None | 0.4812 | None | None | 0.7135 | None | None | None | 1.2912 | None | None | None | 0.761 | None | None | None | None | 0.5215 | None | None | 0.9243 | None | None | None | None | None | None | None | None | None | 1.5116 | None | 1.1753 | None | 0.7166 | None | None | None | None | None | None | -0.0002 | None | None | None | None | None | None | None | None | None | Discriminant power |
| DPI | None | None | Poor | None | None | Poor | None | None | None | Limited | None | None | None | Poor | None | None | None | None | Poor | None | None | Poor | None | None | None | None | None | None | None | None | None | Limited | None | Limited | None | Poor | None | None | None | None | None | None | Poor | None | None | None | None | None | None | None | None | None | Discriminant power interpretation |
| ERR | 0.0058 | 0.0097 | 0.0526 | 0.0263 | 0.0107 | 0.0575 | 0.0029 | 0.268 | 0.0097 | 0.0273 | 0.0448 | 0.0078 | 0.1179 | 0.0166 | 0.0146 | 0.0078 | 0.0088 | 0.0166 | 0.1355 | 0.0224 | 0.0556 | 0.0351 | 0.0146 | 0.0049 | 0.0039 | 0.0029 | 0.0039 | 0.0068 | 0.0146 | 0.0068 | 0.0107 | 0.0117 | 0.0 | 0.0078 | 0.0078 | 0.078 | 0.0097 | 0.1004 | 0.0234 | 0.0058 | 0.0234 | 0.0039 | 0.2125 | 0.0175 | 0.0097 | 0.001 | 0.0019 | 0.0068 | 0.0058 | 0.0283 | 0.0146 | 0.0107 | Error rate |
| F0.5 | 0.0 | 0.0 | 0.1337 | 0.0 | 0.0 | 0.251 | 0.0 | 0.0909 | 0.0 | 0.5727 | 0.0 | 0.0 | 0.0 | 0.0781 | 0.0 | 0.0 | 0.0 | 0.0 | 0.4415 | 0.0 | 0.0 | 0.4237 | 0.0 | 0.0 | 0.0 | 0.0 | 0.0 | 0.0 | 0.0 | 0.0 | 0.0 | 0.7534 | 1.0 | 0.3704 | 0.5844 | 0.463 | 0.0 | 0.0 | 0.0 | 0.0 | 0.0 | 0.0 | 0.0496 | 0.0 | 0.0 | 0.9677 | 0.0 | 0.0 | 0.0 | 0.0 | 0.5 | 0.0 | F0.5 score |
| F1 | 0.0 | 0.0 | 0.1562 | 0.0 | 0.0 | 0.3059 | 0.0 | 0.1379 | 0.0 | 0.65 | 0.0 | 0.0 | 0.0 | 0.1053 | 0.0 | 0.0 | 0.0 | 0.0 | 0.3653 | 0.0 | 0.0 | 0.4545 | 0.0 | 0.0 | 0.0 | 0.0 | 0.0 | 0.0 | 0.0 | 0.0 | 0.0 | 0.6471 | 1.0 | 0.3333 | 0.6923 | 0.4667 | 0.0 | 0.0 | 0.0 | 0.0 | 0.0 | 0.0 | 0.0684 | 0.0 | 0.0 | 0.9231 | 0.0 | 0.0 | 0.0 | 0.0 | 0.2857 | 0.0 | F1 score - harmonic mean of precision and sensitivity |
| F2 | 0.0 | 0.0 | 0.188 | 0.0 | 0.0 | 0.3916 | 0.0 | 0.2857 | 0.0 | 0.7514 | 0.0 | 0.0 | 0.0 | 0.1613 | 0.0 | 0.0 | 0.0 | 0.0 | 0.3115 | 0.0 | 0.0 | 0.4902 | 0.0 | 0.0 | 0.0 | 0.0 | 0.0 | 0.0 | 0.0 | 0.0 | 0.0 | 0.567 | 1.0 | 0.303 | 0.8491 | 0.4704 | 0.0 | 0.0 | 0.0 | 0.0 | 0.0 | 0.0 | 0.1102 | 0.0 | 0.0 | 0.8824 | 0.0 | 0.0 | 0.0 | 0.0 | 0.2 | 0.0 | F2 score |
| FDR | None | None | 0.878 | None | None | 0.7759 | None | 0.9259 | 1.0 | 0.4694 | 1.0 | None | 1.0 | 0.9333 | None | None | None | None | 0.4872 | None | 1.0 | 0.5946 | None | None | None | None | None | None | None | None | 1.0 | 0.1538 | 0.0 | 0.6 | 0.4706 | 0.5395 | None | 1.0 | 1.0 | None | None | None | 0.9581 | None | None | 0.0 | None | None | None | None | 0.0 | None | False discovery rate |
| FN | 6 | 10 | 18 | 27 | 11 | 14 | 3 | 0 | 9 | 5 | 36 | 8 | 104 | 3 | 15 | 8 | 9 | 17 | 101 | 23 | 53 | 14 | 15 | 5 | 4 | 3 | 4 | 7 | 15 | 7 | 7 | 10 | 0 | 5 | 0 | 39 | 10 | 24 | 6 | 6 | 24 | 4 | 35 | 18 | 10 | 1 | 2 | 7 | 6 | 29 | 15 | 11 | False negative/miss/type 2 error |
| FNR | 1.0 | 1.0 | 0.7826 | 1.0 | 1.0 | 0.5185 | 1.0 | 0.0 | 1.0 | 0.1613 | 1.0 | 1.0 | 1.0 | 0.75 | 1.0 | 1.0 | 1.0 | 1.0 | 0.7163 | 1.0 | 1.0 | 0.4828 | 1.0 | 1.0 | 1.0 | 1.0 | 1.0 | 1.0 | 1.0 | 1.0 | 1.0 | 0.4762 | 0.0 | 0.7143 | 0.0 | 0.527 | 1.0 | 1.0 | 1.0 | 1.0 | 1.0 | 1.0 | 0.814 | 1.0 | 1.0 | 0.1429 | 1.0 | 1.0 | 1.0 | 1.0 | 0.8333 | 1.0 | Miss rate or false negative rate |
| FOR | 0.0058 | 0.0097 | 0.0183 | 0.0263 | 0.0107 | 0.0145 | 0.0029 | 0.0 | 0.0088 | 0.0051 | 0.0354 | 0.0078 | 0.1031 | 0.003 | 0.0146 | 0.0078 | 0.0088 | 0.0166 | 0.1065 | 0.0224 | 0.0519 | 0.0142 | 0.0146 | 0.0049 | 0.0039 | 0.0029 | 0.0039 | 0.0068 | 0.0146 | 0.0068 | 0.0068 | 0.0099 | 0.0 | 0.0049 | 0.0 | 0.0411 | 0.0097 | 0.0253 | 0.006 | 0.0058 | 0.0234 | 0.0039 | 0.0419 | 0.0175 | 0.0097 | 0.001 | 0.0019 | 0.0068 | 0.0058 | 0.0283 | 0.0147 | 0.0107 | False omission rate |
| FP | 0 | 0 | 36 | 0 | 0 | 45 | 0 | 275 | 1 | 23 | 10 | 0 | 17 | 14 | 0 | 0 | 0 | 0 | 38 | 0 | 4 | 22 | 0 | 0 | 0 | 0 | 0 | 0 | 0 | 0 | 4 | 2 | 0 | 3 | 8 | 41 | 0 | 79 | 18 | 0 | 0 | 0 | 183 | 0 | 0 | 0 | 0 | 0 | 0 | 0 | 0 | 0 | False positive/type 1 error/false alarm |
| FPR | 0.0 | 0.0 | 0.0359 | 0.0 | 0.0 | 0.045 | 0.0 | 0.2739 | 0.001 | 0.0231 | 0.0101 | 0.0 | 0.0184 | 0.0137 | 0.0 | 0.0 | 0.0 | 0.0 | 0.0429 | 0.0 | 0.0041 | 0.0221 | 0.0 | 0.0 | 0.0 | 0.0 | 0.0 | 0.0 | 0.0 | 0.0 | 0.0039 | 0.002 | 0.0 | 0.0029 | 0.0079 | 0.0431 | 0.0 | 0.0788 | 0.0176 | 0.0 | 0.0 | 0.0 | 0.1862 | 0.0 | 0.0 | 0.0 | 0.0 | 0.0 | 0.0 | 0.0 | 0.0 | 0.0 | Fall-out or false positive rate |
| G | None | None | 0.1628 | None | None | 0.3285 | None | 0.2722 | 0.0 | 0.6671 | 0.0 | None | 0.0 | 0.1291 | None | None | None | None | 0.3814 | None | 0.0 | 0.4579 | None | None | None | None | None | None | None | None | 0.0 | 0.6658 | 1.0 | 0.3381 | 0.7276 | 0.4667 | None | 0.0 | 0.0 | None | None | None | 0.0883 | None | None | 0.9258 | None | None | None | None | 0.4082 | None | G-measure geometric mean of precision and sensitivity |
| GI | 0.0 | 0.0 | 0.1815 | 0.0 | 0.0 | 0.4364 | 0.0 | 0.7261 | -0.001 | 0.8156 | -0.0101 | 0.0 | -0.0184 | 0.2363 | 0.0 | 0.0 | 0.0 | 0.0 | 0.2408 | 0.0 | -0.0041 | 0.4952 | 0.0 | 0.0 | 0.0 | 0.0 | 0.0 | 0.0 | 0.0 | 0.0 | -0.0039 | 0.5218 | 1.0 | 0.2828 | 0.9921 | 0.4299 | 0.0 | -0.0788 | -0.0176 | 0.0 | 0.0 | 0.0 | -0.0001 | 0.0 | 0.0 | 0.8571 | 0.0 | 0.0 | 0.0 | 0.0 | 0.1667 | 0.0 | Gini index |
| GM | 0.0 | 0.0 | 0.4578 | 0.0 | 0.0 | 0.6781 | 0.0 | 0.8521 | 0.0 | 0.9052 | 0.0 | 0.0 | 0.0 | 0.4966 | 0.0 | 0.0 | 0.0 | 0.0 | 0.5211 | 0.0 | 0.0 | 0.7112 | 0.0 | 0.0 | 0.0 | 0.0 | 0.0 | 0.0 | 0.0 | 0.0 | 0.0 | 0.723 | 1.0 | 0.5337 | 0.9961 | 0.6728 | 0.0 | 0.0 | 0.0 | 0.0 | 0.0 | 0.0 | 0.3891 | 0.0 | 0.0 | 0.9258 | 0.0 | 0.0 | 0.0 | 0.0 | 0.4082 | 0.0 | G-mean geometric mean of specificity and sensitivity |
| IBA | 0.0 | 0.0 | 0.0531 | 0.0 | 0.0 | 0.2421 | 0.0 | 0.925 | 0.0 | 0.7061 | 0.0 | 0.0 | 0.0 | 0.065 | 0.0 | 0.0 | 0.0 | 0.0 | 0.0887 | 0.0 | 0.0 | 0.2728 | 0.0 | 0.0 | 0.0 | 0.0 | 0.0 | 0.0 | 0.0 | 0.0 | 0.0 | 0.2749 | 1.0 | 0.0822 | 0.9999 | 0.2336 | 0.0 | 0.0 | 0.0 | 0.0 | 0.0 | 0.0 | 0.0564 | 0.0 | 0.0 | 0.7347 | 0.0 | 0.0 | 0.0 | 0.0 | 0.0278 | 0.0 | Index of balanced accuracy |
| ICSI | None | None | -0.6607 | None | None | -0.2944 | None | 0.0741 | -1.0 | 0.3693 | -1.0 | None | -1.0 | -0.6833 | None | None | None | None | -0.2035 | None | -1.0 | -0.0774 | None | None | None | None | None | None | None | None | -1.0 | 0.37 | 1.0 | -0.3143 | 0.5294 | -0.0665 | None | -1.0 | -1.0 | None | None | None | -0.7721 | None | None | 0.8571 | None | None | None | None | 0.1667 | None | Individual classification success index |
| IS | None | None | 2.4436 | None | None | 3.0904 | None | 1.7885 | None | 4.1343 | None | None | None | 4.0959 | None | None | None | None | 1.8998 | None | None | 3.8423 | None | None | None | None | None | None | None | None | None | 5.3695 | 7.1955 | 5.8735 | 5.9154 | 2.6747 | None | None | None | None | None | None | -0.0009 | None | None | 7.1955 | None | None | None | None | 5.8329 | None | Information score |
| J | 0.0 | 0.0 | 0.0847 | 0.0 | 0.0 | 0.1806 | 0.0 | 0.0741 | 0.0 | 0.4815 | 0.0 | 0.0 | 0.0 | 0.0556 | 0.0 | 0.0 | 0.0 | 0.0 | 0.2235 | 0.0 | 0.0 | 0.2941 | 0.0 | 0.0 | 0.0 | 0.0 | 0.0 | 0.0 | 0.0 | 0.0 | 0.0 | 0.4783 | 1.0 | 0.2 | 0.5294 | 0.3043 | 0.0 | 0.0 | 0.0 | 0.0 | 0.0 | 0.0 | 0.0354 | 0.0 | 0.0 | 0.8571 | 0.0 | 0.0 | 0.0 | 0.0 | 0.1667 | 0.0 | Jaccard index |
| LS | None | None | 5.4401 | None | None | 8.5172 | None | 3.4545 | 0.0 | 17.5616 | 0.0 | None | 0.0 | 17.1 | None | None | None | None | 3.7316 | None | 0.0 | 14.343 | None | None | None | None | None | None | None | None | 0.0 | 41.3407 | 146.5714 | 58.6286 | 60.3529 | 6.3851 | None | 0.0 | 0.0 | None | None | None | 0.9994 | None | None | 146.5714 | None | None | None | None | 57.0 | None | Lift score |
| MCC | None | None | 0.1372 | None | None | 0.3025 | None | 0.2319 | -0.0029 | 0.6547 | -0.0189 | None | -0.0436 | 0.1227 | None | None | None | None | 0.3127 | None | -0.0146 | 0.4402 | None | None | None | None | None | None | None | None | -0.0052 | 0.6606 | 1.0 | 0.3343 | 0.7247 | 0.4247 | None | -0.0447 | -0.0102 | None | None | None | -0.0001 | None | None | 0.9254 | None | None | None | None | 0.4052 | None | Matthews correlation coefficient |
| MCCI | None | None | Negligible | None | None | Weak | None | Negligible | Negligible | Moderate | Negligible | None | Negligible | Negligible | None | None | None | None | Weak | None | Negligible | Weak | None | None | None | None | None | None | None | None | Negligible | Moderate | Very Strong | Weak | Strong | Weak | None | Negligible | Negligible | None | None | None | Negligible | None | None | Very Strong | None | None | None | None | Weak | None | Matthews correlation coefficient interpretation |
| MCEN | 0.2187 | 0.0 | 0.3689 | 0.2141 | 0.0659 | 0.3669 | 0.1376 | 0.5049 | 0.1942 | 0.1745 | 0.342 | 0.1216 | 0.0878 | 0.3194 | 0.1049 | 0.0 | 0.0 | 0.0 | 0.2897 | 0.0 | 0.1488 | 0.2991 | 0.2707 | 0.1082 | 0.1216 | 0.1376 | 0.1216 | 0.0887 | 0.1861 | 0.0887 | 0.2357 | 0.2396 | 0 | 0.2971 | 0.0767 | 0.3404 | 0.0 | 0.2357 | 0.2362 | 0.0 | 0.1591 | 0.1216 | 0.5738 | 0.0 | 0.1321 | 0.0601 | 0.0 | 0.2066 | 0.1376 | 0.2022 | 0.0329 | 0.1417 | Modified confusion entropy |
| MK | None | None | 0.1037 | None | None | 0.2097 | None | 0.0741 | -0.0088 | 0.5255 | -0.0354 | None | -0.1031 | 0.0637 | None | None | None | None | 0.4063 | None | -0.0519 | 0.3912 | None | None | None | None | None | None | None | None | -0.0068 | 0.8363 | 1.0 | 0.3951 | 0.5294 | 0.4195 | None | -0.0253 | -0.006 | None | None | None | -0.0 | None | None | 0.999 | None | None | None | None | 0.9853 | None | Markedness |
| N | 1020 | 1016 | 1003 | 999 | 1015 | 999 | 1023 | 1004 | 1017 | 995 | 990 | 1018 | 922 | 1022 | 1011 | 1018 | 1017 | 1009 | 885 | 1003 | 973 | 997 | 1011 | 1021 | 1022 | 1023 | 1022 | 1019 | 1011 | 1019 | 1019 | 1005 | 1019 | 1019 | 1017 | 952 | 1016 | 1002 | 1020 | 1020 | 1002 | 1022 | 983 | 1008 | 1016 | 1019 | 1024 | 1019 | 1020 | 997 | 1008 | 1015 | Condition negative |
| NLR | 1.0 | 1.0 | 0.8117 | 1.0 | 1.0 | 0.543 | 1.0 | 0.0 | 1.001 | 0.1651 | 1.0102 | 1.0 | 1.0188 | 0.7604 | 1.0 | 1.0 | 1.0 | 1.0 | 0.7484 | 1.0 | 1.0041 | 0.4937 | 1.0 | 1.0 | 1.0 | 1.0 | 1.0 | 1.0 | 1.0 | 1.0 | 1.0039 | 0.4771 | 0.0 | 0.7164 | 0.0 | 0.5507 | 1.0 | 1.0856 | 1.018 | 1.0 | 1.0 | 1.0 | 1.0001 | 1.0 | 1.0 | 0.1429 | 1.0 | 1.0 | 1.0 | 1.0 | 0.8333 | 1.0 | Negative likelihood ratio |
| NLRI | Negligible | Negligible | Negligible | Negligible | Negligible | Negligible | Negligible | Good | Negligible | Fair | Negligible | Negligible | Negligible | Negligible | Negligible | Negligible | Negligible | Negligible | Negligible | Negligible | Negligible | Poor | Negligible | Negligible | Negligible | Negligible | Negligible | Negligible | Negligible | Negligible | Negligible | Poor | Good | Negligible | Good | Negligible | Negligible | Negligible | Negligible | Negligible | Negligible | Negligible | Negligible | Negligible | Negligible | Fair | Negligible | Negligible | Negligible | Negligible | Negligible | Negligible | Negative likelihood ratio interpretation |
| NPV | 0.9942 | 0.9903 | 0.9817 | 0.9737 | 0.9893 | 0.9855 | 0.9971 | 1.0 | 0.9912 | 0.9949 | 0.9646 | 0.9922 | 0.8969 | 0.997 | 0.9854 | 0.9922 | 0.9912 | 0.9834 | 0.8935 | 0.9776 | 0.9481 | 0.9858 | 0.9854 | 0.9951 | 0.9961 | 0.9971 | 0.9961 | 0.9932 | 0.9854 | 0.9932 | 0.9932 | 0.9901 | 1.0 | 0.9951 | 1.0 | 0.9589 | 0.9903 | 0.9747 | 0.994 | 0.9942 | 0.9766 | 0.9961 | 0.9581 | 0.9825 | 0.9903 | 0.999 | 0.9981 | 0.9932 | 0.9942 | 0.9717 | 0.9853 | 0.9893 | Negative predictive value |
| OC | None | None | 0.2174 | None | None | 0.4815 | None | 1.0 | 0.0 | 0.8387 | 0.0 | None | 0.0 | 0.25 | None | None | None | None | 0.5128 | None | 0.0 | 0.5172 | None | None | None | None | None | None | None | None | 0.0 | 0.8462 | 1.0 | 0.4 | 1.0 | 0.473 | None | 0.0 | 0.0 | None | None | None | 0.186 | None | None | 1.0 | None | None | None | None | 1.0 | None | Overlap coefficient |
| OOC | None | None | 0.1628 | None | None | 0.3285 | None | 0.2722 | 0.0 | 0.6671 | 0.0 | None | 0.0 | 0.1291 | None | None | None | None | 0.3814 | None | 0.0 | 0.4579 | None | None | None | None | None | None | None | None | 0.0 | 0.6658 | 1.0 | 0.3381 | 0.7276 | 0.4667 | None | 0.0 | 0.0 | None | None | None | 0.0883 | None | None | 0.9258 | None | None | None | None | 0.4082 | None | Otsuka-Ochiai coefficient |
| OP | -0.0058 | -0.0097 | 0.3154 | -0.0263 | -0.0107 | 0.6129 | -0.0029 | 0.5733 | -0.0097 | 0.8966 | -0.0448 | -0.0078 | -0.1179 | 0.3879 | -0.0146 | -0.0078 | -0.0088 | -0.0166 | 0.3218 | -0.0224 | -0.0556 | 0.6568 | -0.0146 | -0.0049 | -0.0039 | -0.0029 | -0.0039 | -0.0068 | -0.0146 | -0.0068 | -0.0107 | 0.6767 | 1.0 | 0.4377 | 0.9883 | 0.5836 | -0.0097 | -0.1004 | -0.0234 | -0.0058 | -0.0234 | -0.0039 | 0.1597 | -0.0175 | -0.0097 | 0.9221 | -0.0019 | -0.0068 | -0.0058 | -0.0283 | 0.2711 | -0.0107 | Optimized precision |
| P | 6 | 10 | 23 | 27 | 11 | 27 | 3 | 22 | 9 | 31 | 36 | 8 | 104 | 4 | 15 | 8 | 9 | 17 | 141 | 23 | 53 | 29 | 15 | 5 | 4 | 3 | 4 | 7 | 15 | 7 | 7 | 21 | 7 | 7 | 9 | 74 | 10 | 24 | 6 | 6 | 24 | 4 | 43 | 18 | 10 | 7 | 2 | 7 | 6 | 29 | 18 | 11 | Condition positive or support |
| PLR | None | None | 6.0568 | None | None | 10.6889 | None | 3.6509 | 0.0 | 36.2833 | 0.0 | None | 0.0 | 18.25 | None | None | None | None | 6.6069 | None | 0.0 | 23.4404 | None | None | None | None | None | None | None | None | 0.0 | 263.2143 | None | 97.0476 | 127.125 | 10.9822 | None | 0.0 | 0.0 | None | None | None | 0.9994 | None | None | None | None | None | None | None | None | None | Positive likelihood ratio |
| PLRI | None | None | Fair | None | None | Good | None | Poor | Negligible | Good | Negligible | None | Negligible | Good | None | None | None | None | Fair | None | Negligible | Good | None | None | None | None | None | None | None | None | Negligible | Good | None | Good | Good | Good | None | Negligible | Negligible | None | None | None | Negligible | None | None | None | None | None | None | None | None | None | Positive likelihood ratio interpretation |
| POP | 1026 | 1026 | 1026 | 1026 | 1026 | 1026 | 1026 | 1026 | 1026 | 1026 | 1026 | 1026 | 1026 | 1026 | 1026 | 1026 | 1026 | 1026 | 1026 | 1026 | 1026 | 1026 | 1026 | 1026 | 1026 | 1026 | 1026 | 1026 | 1026 | 1026 | 1026 | 1026 | 1026 | 1026 | 1026 | 1026 | 1026 | 1026 | 1026 | 1026 | 1026 | 1026 | 1026 | 1026 | 1026 | 1026 | 1026 | 1026 | 1026 | 1026 | 1026 | 1026 | Population |
| PPV | None | None | 0.122 | None | None | 0.2241 | None | 0.0741 | 0.0 | 0.5306 | 0.0 | None | 0.0 | 0.0667 | None | None | None | None | 0.5128 | None | 0.0 | 0.4054 | None | None | None | None | None | None | None | None | 0.0 | 0.8462 | 1.0 | 0.4 | 0.5294 | 0.4605 | None | 0.0 | 0.0 | None | None | None | 0.0419 | None | None | 1.0 | None | None | None | None | 1.0 | None | Precision or positive predictive value |
| PRE | 0.0058 | 0.0097 | 0.0224 | 0.0263 | 0.0107 | 0.0263 | 0.0029 | 0.0214 | 0.0088 | 0.0302 | 0.0351 | 0.0078 | 0.1014 | 0.0039 | 0.0146 | 0.0078 | 0.0088 | 0.0166 | 0.1374 | 0.0224 | 0.0517 | 0.0283 | 0.0146 | 0.0049 | 0.0039 | 0.0029 | 0.0039 | 0.0068 | 0.0146 | 0.0068 | 0.0068 | 0.0205 | 0.0068 | 0.0068 | 0.0088 | 0.0721 | 0.0097 | 0.0234 | 0.0058 | 0.0058 | 0.0234 | 0.0039 | 0.0419 | 0.0175 | 0.0097 | 0.0068 | 0.0019 | 0.0068 | 0.0058 | 0.0283 | 0.0175 | 0.0107 | Prevalence |
| Q | None | None | 0.7636 | None | None | 0.9033 | None | None | -1.0 | 0.9909 | -1.0 | None | -1.0 | 0.92 | None | None | None | None | 0.7965 | None | -1.0 | 0.9587 | None | None | None | None | None | None | None | None | -1.0 | 0.9964 | None | 0.9853 | None | 0.9045 | None | -1.0 | -1.0 | None | None | None | -0.0004 | None | None | None | None | None | None | None | None | None | Yule Q - coefficient of colligation |
| QI | None | None | Strong | None | None | Strong | None | None | Negligible | Strong | Negligible | None | Negligible | Strong | None | None | None | None | Strong | None | Negligible | Strong | None | None | None | None | None | None | None | None | Negligible | Strong | None | Strong | None | Strong | None | Negligible | Negligible | None | None | None | Negligible | None | None | None | None | None | None | None | None | None | Yule Q interpretation |
| RACC | 0.0 | 0.0 | 0.0009 | 0.0 | 0.0 | 0.0015 | 0.0 | 0.0062 | 0.0 | 0.0014 | 0.0003 | 0.0 | 0.0017 | 0.0001 | 0.0 | 0.0 | 0.0 | 0.0 | 0.0104 | 0.0 | 0.0002 | 0.001 | 0.0 | 0.0 | 0.0 | 0.0 | 0.0 | 0.0 | 0.0 | 0.0 | 0.0 | 0.0003 | 0.0 | 0.0 | 0.0001 | 0.0053 | 0.0 | 0.0018 | 0.0001 | 0.0 | 0.0 | 0.0 | 0.0078 | 0.0 | 0.0 | 0.0 | 0.0 | 0.0 | 0.0 | 0.0 | 0.0001 | 0.0 | Random accuracy |
| RACCU | 0.0 | 0.0 | 0.001 | 0.0002 | 0.0 | 0.0017 | 0.0 | 0.0242 | 0.0 | 0.0015 | 0.0005 | 0.0 | 0.0035 | 0.0001 | 0.0001 | 0.0 | 0.0 | 0.0001 | 0.0114 | 0.0001 | 0.0008 | 0.001 | 0.0001 | 0.0 | 0.0 | 0.0 | 0.0 | 0.0 | 0.0001 | 0.0 | 0.0 | 0.0003 | 0.0 | 0.0 | 0.0002 | 0.0053 | 0.0 | 0.0025 | 0.0001 | 0.0 | 0.0001 | 0.0 | 0.013 | 0.0001 | 0.0 | 0.0 | 0.0 | 0.0 | 0.0 | 0.0002 | 0.0001 | 0.0 | Random accuracy unbiased |
| TN | 1020 | 1016 | 967 | 999 | 1015 | 954 | 1023 | 729 | 1016 | 972 | 980 | 1018 | 905 | 1008 | 1011 | 1018 | 1017 | 1009 | 847 | 1003 | 969 | 975 | 1011 | 1021 | 1022 | 1023 | 1022 | 1019 | 1011 | 1019 | 1015 | 1003 | 1019 | 1016 | 1009 | 911 | 1016 | 923 | 1002 | 1020 | 1002 | 1022 | 800 | 1008 | 1016 | 1019 | 1024 | 1019 | 1020 | 997 | 1008 | 1015 | True negative/correct rejection |
| TNR | 1.0 | 1.0 | 0.9641 | 1.0 | 1.0 | 0.955 | 1.0 | 0.7261 | 0.999 | 0.9769 | 0.9899 | 1.0 | 0.9816 | 0.9863 | 1.0 | 1.0 | 1.0 | 1.0 | 0.9571 | 1.0 | 0.9959 | 0.9779 | 1.0 | 1.0 | 1.0 | 1.0 | 1.0 | 1.0 | 1.0 | 1.0 | 0.9961 | 0.998 | 1.0 | 0.9971 | 0.9921 | 0.9569 | 1.0 | 0.9212 | 0.9824 | 1.0 | 1.0 | 1.0 | 0.8138 | 1.0 | 1.0 | 1.0 | 1.0 | 1.0 | 1.0 | 1.0 | 1.0 | 1.0 | Specificity or true negative rate |
| TON | 1026 | 1026 | 985 | 1026 | 1026 | 968 | 1026 | 729 | 1025 | 977 | 1016 | 1026 | 1009 | 1011 | 1026 | 1026 | 1026 | 1026 | 948 | 1026 | 1022 | 989 | 1026 | 1026 | 1026 | 1026 | 1026 | 1026 | 1026 | 1026 | 1022 | 1013 | 1019 | 1021 | 1009 | 950 | 1026 | 947 | 1008 | 1026 | 1026 | 1026 | 835 | 1026 | 1026 | 1020 | 1026 | 1026 | 1026 | 1026 | 1023 | 1026 | Test outcome negative |
| TOP | 0 | 0 | 41 | 0 | 0 | 58 | 0 | 297 | 1 | 49 | 10 | 0 | 17 | 15 | 0 | 0 | 0 | 0 | 78 | 0 | 4 | 37 | 0 | 0 | 0 | 0 | 0 | 0 | 0 | 0 | 4 | 13 | 7 | 5 | 17 | 76 | 0 | 79 | 18 | 0 | 0 | 0 | 191 | 0 | 0 | 6 | 0 | 0 | 0 | 0 | 3 | 0 | Test outcome positive |
| TP | 0 | 0 | 5 | 0 | 0 | 13 | 0 | 22 | 0 | 26 | 0 | 0 | 0 | 1 | 0 | 0 | 0 | 0 | 40 | 0 | 0 | 15 | 0 | 0 | 0 | 0 | 0 | 0 | 0 | 0 | 0 | 11 | 7 | 2 | 9 | 35 | 0 | 0 | 0 | 0 | 0 | 0 | 8 | 0 | 0 | 6 | 0 | 0 | 0 | 0 | 3 | 0 | True positive/hit |
| TPR | 0.0 | 0.0 | 0.2174 | 0.0 | 0.0 | 0.4815 | 0.0 | 1.0 | 0.0 | 0.8387 | 0.0 | 0.0 | 0.0 | 0.25 | 0.0 | 0.0 | 0.0 | 0.0 | 0.2837 | 0.0 | 0.0 | 0.5172 | 0.0 | 0.0 | 0.0 | 0.0 | 0.0 | 0.0 | 0.0 | 0.0 | 0.0 | 0.5238 | 1.0 | 0.2857 | 1.0 | 0.473 | 0.0 | 0.0 | 0.0 | 0.0 | 0.0 | 0.0 | 0.186 | 0.0 | 0.0 | 0.8571 | 0.0 | 0.0 | 0.0 | 0.0 | 0.1667 | 0.0 | Sensitivity, recall, hit rate, or true positive rate |
| Y | 0.0 | 0.0 | 0.1815 | 0.0 | 0.0 | 0.4364 | 0.0 | 0.7261 | -0.001 | 0.8156 | -0.0101 | 0.0 | -0.0184 | 0.2363 | 0.0 | 0.0 | 0.0 | 0.0 | 0.2408 | 0.0 | -0.0041 | 0.4952 | 0.0 | 0.0 | 0.0 | 0.0 | 0.0 | 0.0 | 0.0 | 0.0 | -0.0039 | 0.5218 | 1.0 | 0.2828 | 0.9921 | 0.4299 | 0.0 | -0.0788 | -0.0176 | 0.0 | 0.0 | 0.0 | -0.0001 | 0.0 | 0.0 | 0.8571 | 0.0 | 0.0 | 0.0 | 0.0 | 0.1667 | 0.0 | Youden index |
| dInd | 1.0 | 1.0 | 0.7834 | 1.0 | 1.0 | 0.5205 | 1.0 | 0.2739 | 1.0 | 0.1629 | 1.0001 | 1.0 | 1.0002 | 0.7501 | 1.0 | 1.0 | 1.0 | 1.0 | 0.7176 | 1.0 | 1.0 | 0.4833 | 1.0 | 1.0 | 1.0 | 1.0 | 1.0 | 1.0 | 1.0 | 1.0 | 1.0 | 0.4762 | 0.0 | 0.7143 | 0.0079 | 0.5288 | 1.0 | 1.0031 | 1.0002 | 1.0 | 1.0 | 1.0 | 0.835 | 1.0 | 1.0 | 0.1429 | 1.0 | 1.0 | 1.0 | 1.0 | 0.8333 | 1.0 | Distance index |
| sInd | 0.2929 | 0.2929 | 0.446 | 0.2929 | 0.2929 | 0.632 | 0.2929 | 0.8063 | 0.2929 | 0.8848 | 0.2929 | 0.2929 | 0.2928 | 0.4696 | 0.2929 | 0.2929 | 0.2929 | 0.2929 | 0.4926 | 0.2929 | 0.2929 | 0.6583 | 0.2929 | 0.2929 | 0.2929 | 0.2929 | 0.2929 | 0.2929 | 0.2929 | 0.2929 | 0.2929 | 0.6633 | 1.0 | 0.4949 | 0.9944 | 0.6261 | 0.2929 | 0.2907 | 0.2928 | 0.2929 | 0.2929 | 0.2929 | 0.4096 | 0.2929 | 0.2929 | 0.899 | 0.2929 | 0.2929 | 0.2929 | 0.2929 | 0.4107 | 0.2929 | Similarity index |

Generated By PyCM Version 3.4
