# Supplementary material for: TopEC: prediction of Enzyme Commission classes by 3D graph neural networks and localized 3D protein descriptor
Source: Nat Commun. 2025 Mar 20;16:2737. doi: 10.1038/s41467-025-57324-5 (PMC11923149; doi:10.1038/s41467-025-57324-5)
Supplement: Supplementary file 3 — Supplementary Data 1 [file 41467_2025_57324_MOESM3_ESM.zip › Data_S1/table1/mainclass/TopEC_distances_angles/TopEnzyme_TEMP.html]

PyCM Report


# PyCM Report

## Dataset Type :

- Multi-Class Classification
- Imbalanced

Note 1 : Recommended statistics for this type of classification highlighted in aqua

Note 2 : The recommender system assumes that the input is the result of classification over the whole data rather than just a part of it.
If the confusion matrix is the result of test data classification, the recommendation is not valid.

## Confusion Matrix :

|  |  |  |  |  |  |  |  |  |  |  |  |  |  |  |  |  |  |  |  |  |  |  |  |  |  |  |  |  |  |  |  |  |  |  |  |  |  |  |  |  |  |  |  |  |  |  |  |  |  |  |  |  |  |  |  |  |  |  |  |  |  |  |  |  |  |
| --- | --- | --- | --- | --- | --- | --- | --- | --- | --- | --- | --- | --- | --- | --- | --- | --- | --- | --- | --- | --- | --- | --- | --- | --- | --- | --- | --- | --- | --- | --- | --- | --- | --- | --- | --- | --- | --- | --- | --- | --- | --- | --- | --- | --- | --- | --- | --- | --- | --- | --- | --- | --- | --- | --- | --- | --- | --- | --- | --- | --- | --- | --- | --- | --- | --- |
| Actual | Predict  |  |  |  |  |  |  |  |  | | --- | --- | --- | --- | --- | --- | --- | --- | |  | 0 | 1 | 2 | 3 | 4 | 5 | 6 | | 0 | 145 | 46 | 20 | 0 | 1 | 2 | 4 | | 1 | 32 | 148 | 37 | 1 | 1 | 5 | 9 | | 2 | 27 | 48 | 131 | 3 | 1 | 0 | 6 | | 3 | 19 | 33 | 16 | 2 | 1 | 2 | 0 | | 4 | 10 | 9 | 4 | 0 | 7 | 1 | 1 | | 5 | 7 | 9 | 4 | 0 | 0 | 11 | 0 | | 6 | 12 | 31 | 9 | 0 | 0 | 1 | 39 | |

## Overall Statistics :

|  |  |
| --- | --- |
| 95% CI | (0.50701,0.57232) |
| ACC Macro | 0.86848 |
| ARI | 0.18529 |
| AUNP | 0.6982 |
| AUNU | 0.66629 |
| Bangdiwala B | 0.33386 |
| Bennett S | 0.46294 |
| CBA | 0.37855 |
| CSI | -0.04466 |
| Chi-Squared | 859.91146 |
| Chi-Squared DF | 36 |
| Conditional Entropy | 1.61655 |
| Cramer V | 0.40017 |
| Cross Entropy | 2.71829 |
| F1 Macro | 0.43655 |
| F1 Micro | 0.53966 |
| FNR Macro | 0.58118 |
| FNR Micro | 0.46034 |
| FPR Macro | 0.08623 |
| FPR Micro | 0.07672 |
| Gwet AC1 | 0.47248 |
| Hamming Loss | 0.46034 |
| Joint Entropy | 4.08544 |
| KL Divergence | 0.2494 |
| Kappa | 0.40136 |
| Kappa 95% CI | (0.35889,0.44382) |
| Kappa No Prevalence | 0.07933 |
| Kappa Standard Error | 0.02167 |
| Kappa Unbiased | 0.39755 |
| Krippendorff Alpha | 0.39789 |
| Lambda A | 0.37915 |
| Lambda B | 0.338 |
| Mutual Information | 0.44366 |
| NIR | 0.26034 |
| Overall ACC | 0.53966 |
| Overall CEN | 0.49881 |
| Overall J | (2.06665,0.29524) |
| Overall MCC | 0.40694 |
| Overall MCEN | 0.60756 |
| Overall RACC | 0.23103 |
| Overall RACCU | 0.23589 |
| P-Value | -0.0 |
| PPV Macro | 0.53652 |
| PPV Micro | 0.53966 |
| Pearson C | 0.7 |
| Phi-Squared | 0.96079 |
| RCI | 0.1797 |
| RR | 127.85714 |
| Reference Entropy | 2.46889 |
| Response Entropy | 2.0602 |
| SOA1(Landis & Koch) | Moderate |
| SOA2(Fleiss) | Intermediate to Good |
| SOA3(Altman) | Moderate |
| SOA4(Cicchetti) | Fair |
| SOA5(Cramer) | Relatively Strong |
| SOA6(Matthews) | Weak |
| Scott PI | 0.39755 |
| Standard Error | 0.01666 |
| TNR Macro | 0.91377 |
| TNR Micro | 0.92328 |
| TPR Macro | 0.41882 |
| TPR Micro | 0.53966 |
| Zero-one Loss | 412 |

## Class Statistics :

|  |  |  |  |  |  |  |  |  |
| --- | --- | --- | --- | --- | --- | --- | --- | --- |
| Class | 0 | 1 | 2 | 3 | 4 | 5 | 6 | Description |
| ACC | 0.79888 | 0.70838 | 0.80447 | 0.9162 | 0.9676 | 0.96536 | 0.91844 | Accuracy |
| AGF | 0.7522 | 0.69712 | 0.72579 | 0.17706 | 0.49583 | 0.60732 | 0.6566 | Adjusted F-score |
| AGM | 0.78865 | 0.70467 | 0.78664 | 0.56248 | 0.72618 | 0.78609 | 0.80001 | Adjusted geometric mean |
| AM | 34 | 91 | 5 | -67 | -21 | -9 | -33 | Difference between automatic and manual classification |
| AUC | 0.75354 | 0.68467 | 0.73697 | 0.51127 | 0.60706 | 0.67105 | 0.6995 | Area under the ROC curve |
| AUCI | Good | Fair | Good | Poor | Fair | Fair | Fair | AUC value interpretation |
| AUPR | 0.62027 | 0.54599 | 0.59962 | 0.18037 | 0.42756 | 0.42742 | 0.54246 | Area under the PR curve |
| BCD | 0.01899 | 0.05084 | 0.00279 | 0.03743 | 0.01173 | 0.00503 | 0.01844 | Bray-Curtis dissimilarity |
| BM | 0.50709 | 0.36933 | 0.47393 | 0.02253 | 0.21412 | 0.34211 | 0.39901 | Informedness or bookmaker informedness |
| CEN | 0.46427 | 0.54515 | 0.46633 | 0.54677 | 0.56835 | 0.5567 | 0.46411 | Confusion entropy |
| DOR | 10.58123 | 4.80802 | 10.08614 | 5.76056 | 60.13 | 42.65 | 28.80849 | Diagnostic odds ratio |
| DP | 0.56486 | 0.37599 | 0.55338 | 0.41927 | 0.98086 | 0.89862 | 0.80468 | Discriminant power |
| DPI | Poor | Poor | Poor | Poor | Poor | Poor | Poor | Discriminant power interpretation |
| ERR | 0.20112 | 0.29162 | 0.19553 | 0.0838 | 0.0324 | 0.03464 | 0.08156 | Error rate |
| F0.5 | 0.59135 | 0.48398 | 0.59545 | 0.10309 | 0.46053 | 0.46218 | 0.59451 | F0.5 score |
| F1 | 0.61702 | 0.53142 | 0.59954 | 0.05063 | 0.32558 | 0.41509 | 0.51656 | F1 score - harmonic mean of precision and sensitivity |
| F2 | 0.64502 | 0.58917 | 0.60369 | 0.03356 | 0.2518 | 0.37671 | 0.45667 | F2 score |
| FDR | 0.4246 | 0.54321 | 0.40724 | 0.66667 | 0.36364 | 0.5 | 0.33898 | False discovery rate |
| FN | 73 | 85 | 85 | 71 | 25 | 20 | 53 | False negative/miss/type 2 error |
| FNR | 0.33486 | 0.36481 | 0.39352 | 0.9726 | 0.78125 | 0.64516 | 0.57609 | Miss rate or false negative rate |
| FOR | 0.11353 | 0.14886 | 0.12611 | 0.07987 | 0.02828 | 0.02291 | 0.0634 | False omission rate |
| FP | 107 | 176 | 90 | 4 | 4 | 11 | 20 | False positive/type 1 error/false alarm |
| FPR | 0.15805 | 0.26586 | 0.13255 | 0.00487 | 0.00463 | 0.01273 | 0.02491 | Fall-out or false positive rate |
| G | 0.61864 | 0.53866 | 0.59958 | 0.09556 | 0.3731 | 0.42121 | 0.52935 | G-measure geometric mean of precision and sensitivity |
| GI | 0.50709 | 0.36933 | 0.47393 | 0.02253 | 0.21412 | 0.34211 | 0.39901 | Gini index |
| GM | 0.74834 | 0.68288 | 0.72532 | 0.16512 | 0.46662 | 0.59188 | 0.64293 | G-mean geometric mean of specificity and sensitivity |
| IBA | 0.461 | 0.42018 | 0.3888 | 0.00088 | 0.04864 | 0.12877 | 0.18552 | Index of balanced accuracy |
| ICSI | 0.24053 | 0.09198 | 0.19924 | -0.63927 | -0.14489 | -0.14516 | 0.08493 | Individual classification success index |
| IS | 1.24019 | 0.81116 | 1.29638 | 2.03096 | 4.15367 | 3.85155 | 2.68494 | Information score |
| J | 0.44615 | 0.36186 | 0.4281 | 0.02597 | 0.19444 | 0.2619 | 0.34821 | Jaccard index |
| LS | 2.36229 | 1.75462 | 2.45611 | 4.08676 | 17.7983 | 14.43548 | 6.43055 | Lift score |
| MCC | 0.48395 | 0.33724 | 0.47028 | 0.07557 | 0.36083 | 0.404 | 0.48832 | Matthews correlation coefficient |
| MCCI | Weak | Weak | Weak | Negligible | Weak | Weak | Weak | Matthews correlation coefficient interpretation |
| MCEN | 0.58919 | 0.6631 | 0.58396 | 0.55092 | 0.62126 | 0.63341 | 0.54735 | Modified confusion entropy |
| MK | 0.46187 | 0.30793 | 0.46665 | 0.25347 | 0.60808 | 0.47709 | 0.59762 | Markedness |
| N | 677 | 662 | 679 | 822 | 863 | 864 | 803 | Condition negative |
| NLR | 0.39772 | 0.49692 | 0.45365 | 0.97736 | 0.78489 | 0.65348 | 0.5908 | Negative likelihood ratio |
| NLRI | Poor | Poor | Poor | Negligible | Negligible | Negligible | Negligible | Negative likelihood ratio interpretation |
| NPV | 0.88647 | 0.85114 | 0.87389 | 0.92013 | 0.97172 | 0.97709 | 0.9366 | Negative predictive value |
| OC | 0.66514 | 0.63519 | 0.60648 | 0.33333 | 0.63636 | 0.5 | 0.66102 | Overlap coefficient |
| OOC | 0.61864 | 0.53866 | 0.59958 | 0.09556 | 0.3731 | 0.42121 | 0.52935 | Otsuka-Ochiai coefficient |
| OP | 0.68156 | 0.63612 | 0.62741 | -0.03021 | 0.32794 | 0.49414 | 0.52446 | Optimized precision |
| P | 218 | 233 | 216 | 73 | 32 | 31 | 92 | Condition positive or support |
| PLR | 4.20839 | 2.38919 | 4.57557 | 5.63014 | 47.19531 | 27.87097 | 17.02011 | Positive likelihood ratio |
| PLRI | Poor | Poor | Poor | Fair | Good | Good | Good | Positive likelihood ratio interpretation |
| POP | 895 | 895 | 895 | 895 | 895 | 895 | 895 | Population |
| PPV | 0.5754 | 0.45679 | 0.59276 | 0.33333 | 0.63636 | 0.5 | 0.66102 | Precision or positive predictive value |
| PRE | 0.24358 | 0.26034 | 0.24134 | 0.08156 | 0.03575 | 0.03464 | 0.10279 | Prevalence |
| Q | 0.82731 | 0.65565 | 0.81959 | 0.70417 | 0.96728 | 0.95418 | 0.93291 | Yule Q - coefficient of colligation |
| QI | Strong | Moderate | Strong | Moderate | Strong | Strong | Strong | Yule Q interpretation |
| RACC | 0.06858 | 0.09424 | 0.05959 | 0.00055 | 0.00044 | 0.00085 | 0.00678 | Random accuracy |
| RACCU | 0.06894 | 0.09683 | 0.0596 | 0.00195 | 0.00058 | 0.00088 | 0.00712 | Random accuracy unbiased |
| TN | 570 | 486 | 589 | 818 | 859 | 853 | 783 | True negative/correct rejection |
| TNR | 0.84195 | 0.73414 | 0.86745 | 0.99513 | 0.99537 | 0.98727 | 0.97509 | Specificity or true negative rate |
| TON | 643 | 571 | 674 | 889 | 884 | 873 | 836 | Test outcome negative |
| TOP | 252 | 324 | 221 | 6 | 11 | 22 | 59 | Test outcome positive |
| TP | 145 | 148 | 131 | 2 | 7 | 11 | 39 | True positive/hit |
| TPR | 0.66514 | 0.63519 | 0.60648 | 0.0274 | 0.21875 | 0.35484 | 0.42391 | Sensitivity, recall, hit rate, or true positive rate |
| Y | 0.50709 | 0.36933 | 0.47393 | 0.02253 | 0.21412 | 0.34211 | 0.39901 | Youden index |
| dInd | 0.37029 | 0.4514 | 0.41524 | 0.97261 | 0.78126 | 0.64529 | 0.57663 | Distance index |
| sInd | 0.73817 | 0.68081 | 0.70638 | 0.31226 | 0.44756 | 0.54371 | 0.59226 | Similarity index |

Generated By PyCM Version 3.3
