# Supplementary material for: TopEC: prediction of Enzyme Commission classes by 3D graph neural networks and localized 3D protein descriptor
Source: Nat Commun. 2025 Mar 20;16:2737. doi: 10.1038/s41467-025-57324-5 (PMC11923149; doi:10.1038/s41467-025-57324-5)
Supplement: Supplementary file 3 — Supplementary Data 1 [file 41467_2025_57324_MOESM3_ESM.zip › Data_S1/table1/mainclass/TopEC_distances_angles/TopEnzyme_FOLD.html]

PyCM Report


# PyCM Report

## Dataset Type :

- Multi-Class Classification
- Imbalanced

Note 1 : Recommended statistics for this type of classification highlighted in aqua

Note 2 : The recommender system assumes that the input is the result of classification over the whole data rather than just a part of it.
If the confusion matrix is the result of test data classification, the recommendation is not valid.

## Confusion Matrix :

|  |  |  |  |  |  |  |  |  |  |  |  |  |  |  |  |  |  |  |  |  |  |  |  |  |  |  |  |  |  |  |  |  |  |  |  |  |  |  |  |  |  |  |  |  |  |  |  |  |  |  |  |  |  |  |  |  |  |  |  |  |  |  |  |  |  |
| --- | --- | --- | --- | --- | --- | --- | --- | --- | --- | --- | --- | --- | --- | --- | --- | --- | --- | --- | --- | --- | --- | --- | --- | --- | --- | --- | --- | --- | --- | --- | --- | --- | --- | --- | --- | --- | --- | --- | --- | --- | --- | --- | --- | --- | --- | --- | --- | --- | --- | --- | --- | --- | --- | --- | --- | --- | --- | --- | --- | --- | --- | --- | --- | --- | --- |
| Actual | Predict  |  |  |  |  |  |  |  |  | | --- | --- | --- | --- | --- | --- | --- | --- | |  | 0 | 1 | 2 | 3 | 4 | 5 | 6 | | 0 | 111 | 40 | 16 | 0 | 0 | 0 | 3 | | 1 | 15 | 200 | 24 | 0 | 0 | 5 | 1 | | 2 | 24 | 39 | 112 | 1 | 0 | 3 | 0 | | 3 | 20 | 37 | 15 | 7 | 1 | 2 | 1 | | 4 | 19 | 30 | 23 | 2 | 6 | 2 | 3 | | 5 | 10 | 44 | 23 | 2 | 3 | 10 | 1 | | 6 | 1 | 12 | 2 | 0 | 0 | 0 | 23 | |

## Overall Statistics :

|  |  |
| --- | --- |
| 95% CI | (0.49244,0.55795) |
| ACC Macro | 0.86434 |
| ARI | 0.20454 |
| AUNP | 0.69213 |
| AUNU | 0.66764 |
| Bangdiwala B | 0.37243 |
| Bennett S | 0.44606 |
| CBA | 0.34874 |
| CSI | -0.01532 |
| Chi-Squared | 925.96877 |
| Chi-Squared DF | 36 |
| Conditional Entropy | 1.47234 |
| Cramer V | 0.41572 |
| Cross Entropy | 3.09419 |
| F1 Macro | 0.41307 |
| F1 Micro | 0.5252 |
| FNR Macro | 0.57676 |
| FNR Micro | 0.4748 |
| FPR Macro | 0.08796 |
| FPR Micro | 0.07913 |
| Gwet AC1 | 0.45572 |
| Hamming Loss | 0.4748 |
| Joint Entropy | 4.07983 |
| KL Divergence | 0.48671 |
| Kappa | 0.39065 |
| Kappa 95% CI | (0.34861,0.43268) |
| Kappa No Prevalence | 0.05039 |
| Kappa Standard Error | 0.02145 |
| Kappa Unbiased | 0.38002 |
| Krippendorff Alpha | 0.38037 |
| Lambda A | 0.34568 |
| Lambda B | 0.31568 |
| Mutual Information | 0.48392 |
| NIR | 0.27436 |
| Overall ACC | 0.5252 |
| Overall CEN | 0.488 |
| Overall J | (2.00472,0.28639) |
| Overall MCC | 0.40605 |
| Overall MCEN | 0.59126 |
| Overall RACC | 0.22081 |
| Overall RACCU | 0.23416 |
| P-Value | -0.0 |
| PPV Macro | 0.56144 |
| PPV Micro | 0.5252 |
| Pearson C | 0.71349 |
| Phi-Squared | 1.03692 |
| RCI | 0.18559 |
| RR | 127.57143 |
| Reference Entropy | 2.60748 |
| Response Entropy | 1.95627 |
| SOA1(Landis & Koch) | Fair |
| SOA2(Fleiss) | Poor |
| SOA3(Altman) | Fair |
| SOA4(Cicchetti) | Poor |
| SOA5(Cramer) | Relatively Strong |
| SOA6(Matthews) | Weak |
| Scott PI | 0.38002 |
| Standard Error | 0.01671 |
| TNR Macro | 0.91204 |
| TNR Micro | 0.92087 |
| TPR Macro | 0.42324 |
| TPR Micro | 0.5252 |
| Zero-one Loss | 424 |

## Class Statistics :

|  |  |  |  |  |  |  |  |  |
| --- | --- | --- | --- | --- | --- | --- | --- | --- |
| Class | 0 | 1 | 2 | 3 | 4 | 5 | 6 | Description |
| ACC | 0.83427 | 0.7234 | 0.80963 | 0.90929 | 0.90705 | 0.89362 | 0.97312 | Accuracy |
| AGF | 0.75633 | 0.78598 | 0.73237 | 0.30739 | 0.28177 | 0.34163 | 0.7842 | Adjusted F-score |
| AGM | 0.81047 | 0.72379 | 0.78683 | 0.62451 | 0.6118 | 0.63711 | 0.87933 | Adjusted geometric mean |
| AM | 30 | 157 | 36 | -71 | -75 | -71 | -6 | Difference between automatic and manual classification |
| AUC | 0.76492 | 0.7523 | 0.74072 | 0.53908 | 0.53282 | 0.54626 | 0.79737 | Area under the ROC curve |
| AUCI | Good | Good | Good | Poor | Poor | Poor | Good | AUC value interpretation |
| AUPR | 0.60397 | 0.65692 | 0.57331 | 0.33384 | 0.33529 | 0.28104 | 0.66201 | Area under the PR curve |
| BCD | 0.0168 | 0.08791 | 0.02016 | 0.03975 | 0.04199 | 0.03975 | 0.00336 | Bray-Curtis dissimilarity |
| BM | 0.52984 | 0.5046 | 0.48144 | 0.07816 | 0.06564 | 0.09253 | 0.59474 | Informedness or bookmaker informedness |
| CEN | 0.46139 | 0.46732 | 0.49707 | 0.55309 | 0.58675 | 0.59603 | 0.3689 | Confusion entropy |
| DOR | 13.40202 | 9.81298 | 9.91624 | 14.82895 | 15.26582 | 7.91165 | 144.13333 | Diagnostic odds ratio |
| DP | 0.62144 | 0.54681 | 0.54932 | 0.64567 | 0.65262 | 0.49524 | 1.19019 | Discriminant power |
| DPI | Poor | Poor | Poor | Poor | Poor | Poor | Limited | Discriminant power interpretation |
| ERR | 0.16573 | 0.2766 | 0.19037 | 0.09071 | 0.09295 | 0.10638 | 0.02688 | Error rate |
| F0.5 | 0.57216 | 0.53967 | 0.53898 | 0.26718 | 0.24 | 0.27624 | 0.69277 | F0.5 score |
| F1 | 0.6 | 0.61824 | 0.56853 | 0.14737 | 0.12632 | 0.17391 | 0.65714 | F1 score - harmonic mean of precision and sensitivity |
| F2 | 0.63068 | 0.72359 | 0.6015 | 0.10174 | 0.08571 | 0.1269 | 0.625 | F2 score |
| FDR | 0.445 | 0.50249 | 0.47907 | 0.41667 | 0.4 | 0.54545 | 0.28125 | False discovery rate |
| FN | 59 | 45 | 67 | 76 | 79 | 83 | 15 | False negative/miss/type 2 error |
| FNR | 0.34706 | 0.18367 | 0.3743 | 0.91566 | 0.92941 | 0.89247 | 0.39474 | Miss rate or false negative rate |
| FOR | 0.08514 | 0.09165 | 0.09882 | 0.08627 | 0.08947 | 0.09529 | 0.01742 | False omission rate |
| FP | 89 | 202 | 103 | 5 | 4 | 12 | 9 | False positive/type 1 error/false alarm |
| FPR | 0.1231 | 0.31173 | 0.14426 | 0.00617 | 0.00495 | 0.015 | 0.01053 | Fall-out or false positive rate |
| G | 0.60198 | 0.63729 | 0.57092 | 0.2218 | 0.2058 | 0.22108 | 0.65957 | G-measure geometric mean of precision and sensitivity |
| GI | 0.52984 | 0.5046 | 0.48144 | 0.07816 | 0.06564 | 0.09253 | 0.59474 | Gini index |
| GM | 0.75668 | 0.74957 | 0.73174 | 0.28951 | 0.26503 | 0.32544 | 0.77388 | G-mean geometric mean of specificity and sensitivity |
| IBA | 0.44433 | 0.6338 | 0.41226 | 0.00759 | 0.00531 | 0.01298 | 0.36879 | Index of balanced accuracy |
| ICSI | 0.20794 | 0.31384 | 0.14663 | -0.33233 | -0.32941 | -0.43793 | 0.32401 | Individual classification success index |
| IS | 1.54369 | 0.85868 | 1.37786 | 2.64987 | 2.65616 | 2.12585 | 4.07815 | Information score |
| J | 0.42857 | 0.44743 | 0.39716 | 0.07955 | 0.06742 | 0.09524 | 0.48936 | Jaccard index |
| LS | 2.91538 | 1.81338 | 2.59883 | 6.2761 | 6.30353 | 4.36461 | 16.89062 | Lift score |
| MCC | 0.49895 | 0.45255 | 0.4508 | 0.19711 | 0.18306 | 0.18232 | 0.64584 | Matthews correlation coefficient |
| MCCI | Weak | Weak | Weak | Negligible | Negligible | Negligible | Moderate | Matthews correlation coefficient interpretation |
| MCEN | 0.5771 | 0.59419 | 0.61335 | 0.56873 | 0.60182 | 0.61967 | 0.46756 | Modified confusion entropy |
| MK | 0.46986 | 0.40586 | 0.42211 | 0.49707 | 0.51053 | 0.35925 | 0.70133 | Markedness |
| N | 723 | 648 | 714 | 810 | 808 | 800 | 855 | Condition negative |
| NLR | 0.39578 | 0.26686 | 0.4374 | 0.92135 | 0.93404 | 0.90606 | 0.39894 | Negative likelihood ratio |
| NLRI | Poor | Poor | Poor | Negligible | Negligible | Negligible | Poor | Negative likelihood ratio interpretation |
| NPV | 0.91486 | 0.90835 | 0.90118 | 0.91373 | 0.91053 | 0.90471 | 0.98258 | Negative predictive value |
| OC | 0.65294 | 0.81633 | 0.6257 | 0.58333 | 0.6 | 0.45455 | 0.71875 | Overlap coefficient |
| OOC | 0.60198 | 0.63729 | 0.57092 | 0.2218 | 0.2058 | 0.22108 | 0.65957 | Otsuka-Ochiai coefficient |
| OP | 0.68787 | 0.6383 | 0.65435 | 0.06574 | 0.03954 | 0.09046 | 0.7322 | Optimized precision |
| P | 170 | 245 | 179 | 83 | 85 | 93 | 38 | Condition positive or support |
| PLR | 5.30423 | 2.61871 | 4.33737 | 13.66265 | 14.25882 | 7.16846 | 57.5 | Positive likelihood ratio |
| PLRI | Fair | Poor | Poor | Good | Good | Fair | Good | Positive likelihood ratio interpretation |
| POP | 893 | 893 | 893 | 893 | 893 | 893 | 893 | Population |
| PPV | 0.555 | 0.49751 | 0.52093 | 0.58333 | 0.6 | 0.45455 | 0.71875 | Precision or positive predictive value |
| PRE | 0.19037 | 0.27436 | 0.20045 | 0.09295 | 0.09518 | 0.10414 | 0.04255 | Prevalence |
| Q | 0.86113 | 0.81504 | 0.81679 | 0.87365 | 0.87704 | 0.77557 | 0.98622 | Yule Q - coefficient of colligation |
| QI | Strong | Strong | Strong | Strong | Strong | Strong | Strong | Yule Q interpretation |
| RACC | 0.04264 | 0.12351 | 0.04826 | 0.00125 | 0.00107 | 0.00257 | 0.00152 | Random accuracy |
| RACCU | 0.04292 | 0.13123 | 0.04867 | 0.00283 | 0.00283 | 0.00415 | 0.00154 | Random accuracy unbiased |
| TN | 634 | 446 | 611 | 805 | 804 | 788 | 846 | True negative/correct rejection |
| TNR | 0.8769 | 0.68827 | 0.85574 | 0.99383 | 0.99505 | 0.985 | 0.98947 | Specificity or true negative rate |
| TON | 693 | 491 | 678 | 881 | 883 | 871 | 861 | Test outcome negative |
| TOP | 200 | 402 | 215 | 12 | 10 | 22 | 32 | Test outcome positive |
| TP | 111 | 200 | 112 | 7 | 6 | 10 | 23 | True positive/hit |
| TPR | 0.65294 | 0.81633 | 0.6257 | 0.08434 | 0.07059 | 0.10753 | 0.60526 | Sensitivity, recall, hit rate, or true positive rate |
| Y | 0.52984 | 0.5046 | 0.48144 | 0.07816 | 0.06564 | 0.09253 | 0.59474 | Youden index |
| dInd | 0.36824 | 0.36182 | 0.40114 | 0.91568 | 0.92942 | 0.8926 | 0.39488 | Distance index |
| sInd | 0.73961 | 0.74416 | 0.71635 | 0.35251 | 0.3428 | 0.36884 | 0.72078 | Similarity index |

Generated By PyCM Version 3.3
