# Supplementary material for: TopEC: prediction of Enzyme Commission classes by 3D graph neural networks and localized 3D protein descriptor
Source: Nat Commun. 2025 Mar 20;16:2737. doi: 10.1038/s41467-025-57324-5 (PMC11923149; doi:10.1038/s41467-025-57324-5)
Supplement: Supplementary file 3 — Supplementary Data 1 [file 41467_2025_57324_MOESM3_ESM.zip › Data_S1/table1/mainclass/TopEC_distances_angles/Combined_TEMP.html]

PyCM Report


# PyCM Report

## Dataset Type :

- Multi-Class Classification
- Imbalanced

Note 1 : Recommended statistics for this type of classification highlighted in aqua

Note 2 : The recommender system assumes that the input is the result of classification over the whole data rather than just a part of it.
If the confusion matrix is the result of test data classification, the recommendation is not valid.

## Confusion Matrix :

|  |  |  |  |  |  |  |  |  |  |  |  |  |  |  |  |  |  |  |  |  |  |  |  |  |  |  |  |  |  |  |  |  |  |  |  |  |  |  |  |  |  |  |  |  |  |  |  |  |  |  |  |  |  |  |  |  |  |  |  |  |  |  |  |  |  |
| --- | --- | --- | --- | --- | --- | --- | --- | --- | --- | --- | --- | --- | --- | --- | --- | --- | --- | --- | --- | --- | --- | --- | --- | --- | --- | --- | --- | --- | --- | --- | --- | --- | --- | --- | --- | --- | --- | --- | --- | --- | --- | --- | --- | --- | --- | --- | --- | --- | --- | --- | --- | --- | --- | --- | --- | --- | --- | --- | --- | --- | --- | --- | --- | --- | --- |
| Actual | Predict  |  |  |  |  |  |  |  |  | | --- | --- | --- | --- | --- | --- | --- | --- | |  | 0 | 1 | 2 | 3 | 4 | 5 | 6 | | 0 | 472 | 106 | 31 | 20 | 3 | 0 | 9 | | 1 | 35 | 813 | 76 | 30 | 9 | 6 | 18 | | 2 | 24 | 120 | 709 | 11 | 2 | 6 | 14 | | 3 | 19 | 45 | 23 | 107 | 2 | 1 | 1 | | 4 | 6 | 29 | 7 | 3 | 60 | 2 | 2 | | 5 | 4 | 17 | 5 | 2 | 2 | 52 | 7 | | 6 | 16 | 40 | 42 | 6 | 1 | 1 | 62 | |

## Overall Statistics :

|  |  |
| --- | --- |
| 95% CI | (0.7236,0.75463) |
| ACC Macro | 0.92546 |
| ARI | 0.46906 |
| AUNP | 0.82419 |
| AUNU | 0.78949 |
| Bangdiwala B | 0.59054 |
| Bennett S | 0.69564 |
| CBA | 0.60991 |
| CSI | 0.34048 |
| Chi-Squared | 7131.37162 |
| Chi-Squared DF | 36 |
| Conditional Entropy | 1.24221 |
| Cramer V | 0.62141 |
| Cross Entropy | 2.33527 |
| F1 Macro | 0.66232 |
| F1 Micro | 0.73912 |
| FNR Macro | 0.37079 |
| FNR Micro | 0.26088 |
| FPR Macro | 0.05023 |
| FPR Micro | 0.04348 |
| Gwet AC1 | 0.70201 |
| Hamming Loss | 0.26088 |
| Joint Entropy | 3.55904 |
| KL Divergence | 0.01843 |
| Kappa | 0.65138 |
| Kappa 95% CI | (0.63065,0.67211) |
| Kappa No Prevalence | 0.47823 |
| Kappa Standard Error | 0.01058 |
| Kappa Unbiased | 0.65086 |
| Krippendorff Alpha | 0.65091 |
| Lambda A | 0.61597 |
| Lambda B | 0.57914 |
| Mutual Information | 0.92947 |
| NIR | 0.32066 |
| Overall ACC | 0.73912 |
| Overall CEN | 0.35065 |
| Overall J | (3.54447,0.50635) |
| Overall MCC | 0.65348 |
| Overall MCEN | 0.48261 |
| Overall RACC | 0.25166 |
| Overall RACCU | 0.25279 |
| P-Value | None |
| PPV Macro | 0.71127 |
| PPV Micro | 0.73912 |
| Pearson C | 0.83577 |
| Phi-Squared | 2.31688 |
| RCI | 0.40118 |
| RR | 439.71429 |
| Reference Entropy | 2.31684 |
| Response Entropy | 2.17168 |
| SOA1(Landis & Koch) | Substantial |
| SOA2(Fleiss) | Intermediate to Good |
| SOA3(Altman) | Good |
| SOA4(Cicchetti) | Good |
| SOA5(Cramer) | Strong |
| SOA6(Matthews) | Moderate |
| Scott PI | 0.65086 |
| Standard Error | 0.00791 |
| TNR Macro | 0.94977 |
| TNR Micro | 0.95652 |
| TPR Macro | 0.62921 |
| TPR Micro | 0.73912 |
| Zero-one Loss | 803 |

## Class Statistics :

|  |  |  |  |  |  |  |  |  |
| --- | --- | --- | --- | --- | --- | --- | --- | --- |
| Class | 0 | 1 | 2 | 3 | 4 | 5 | 6 | Description |
| ACC | 0.91131 | 0.82749 | 0.88272 | 0.94704 | 0.97791 | 0.98278 | 0.94899 | Accuracy |
| AGF | 0.83934 | 0.84157 | 0.8566 | 0.73102 | 0.75773 | 0.77879 | 0.61823 | Adjusted F-score |
| AGM | 0.89162 | 0.82761 | 0.88109 | 0.8463 | 0.86429 | 0.87678 | 0.78697 | Adjusted geometric mean |
| AM | -65 | 183 | 7 | -19 | -30 | -21 | -55 | Difference between automatic and manual classification |
| AUC | 0.84684 | 0.82649 | 0.85814 | 0.7577 | 0.77203 | 0.78946 | 0.67576 | Area under the ROC curve |
| AUCI | Very Good | Very Good | Very Good | Good | Good | Good | Fair | AUC value interpretation |
| AUPR | 0.7779 | 0.75929 | 0.79709 | 0.56908 | 0.65498 | 0.67449 | 0.45886 | Area under the PR curve |
| BCD | 0.01056 | 0.02973 | 0.00114 | 0.00309 | 0.00487 | 0.00341 | 0.00893 | Bray-Curtis dissimilarity |
| BM | 0.69367 | 0.65298 | 0.71628 | 0.5154 | 0.54406 | 0.57892 | 0.35152 | Informedness or bookmaker informedness |
| CEN | 0.31029 | 0.35533 | 0.2916 | 0.49364 | 0.42974 | 0.42792 | 0.57545 | Confusion entropy |
| DOR | 62.65225 | 22.69458 | 43.71383 | 45.85714 | 190.11815 | 261.14189 | 32.78912 | Diagnostic odds ratio |
| DP | 0.9907 | 0.74756 | 0.90452 | 0.91598 | 1.25649 | 1.33249 | 0.83567 | Discriminant power |
| DPI | Poor | Poor | Poor | Poor | Limited | Limited | Poor | Discriminant power interpretation |
| ERR | 0.08869 | 0.17251 | 0.11728 | 0.05296 | 0.02209 | 0.01722 | 0.05101 | Error rate |
| F0.5 | 0.80136 | 0.71731 | 0.7952 | 0.58534 | 0.70588 | 0.72022 | 0.5 | F0.5 score |
| F1 | 0.77568 | 0.75382 | 0.79708 | 0.56764 | 0.6383 | 0.66242 | 0.44128 | F1 score - harmonic mean of precision and sensitivity |
| F2 | 0.75159 | 0.79426 | 0.79896 | 0.55098 | 0.58252 | 0.61321 | 0.3949 | F2 score |
| FDR | 0.18056 | 0.30513 | 0.20605 | 0.40223 | 0.24051 | 0.23529 | 0.45133 | False discovery rate |
| FN | 169 | 174 | 177 | 91 | 49 | 37 | 106 | False negative/miss/type 2 error |
| FNR | 0.26365 | 0.17629 | 0.19977 | 0.4596 | 0.44954 | 0.41573 | 0.63095 | Miss rate or false negative rate |
| FOR | 0.06755 | 0.09119 | 0.08101 | 0.03139 | 0.01634 | 0.01229 | 0.03575 | False omission rate |
| FP | 104 | 357 | 184 | 72 | 19 | 16 | 51 | False positive/type 1 error/false alarm |
| FPR | 0.04268 | 0.17073 | 0.08394 | 0.025 | 0.0064 | 0.00535 | 0.01753 | Fall-out or false positive rate |
| G | 0.77679 | 0.75655 | 0.79708 | 0.56836 | 0.64658 | 0.66843 | 0.44998 | G-measure geometric mean of precision and sensitivity |
| GI | 0.69367 | 0.65298 | 0.71628 | 0.5154 | 0.54406 | 0.57892 | 0.35152 | Gini index |
| GM | 0.8396 | 0.82648 | 0.85619 | 0.72587 | 0.73955 | 0.76233 | 0.60215 | G-mean geometric mean of specificity and sensitivity |
| IBA | 0.54915 | 0.67928 | 0.64814 | 0.29791 | 0.30457 | 0.34265 | 0.14016 | Index of balanced accuracy |
| ICSI | 0.55579 | 0.51858 | 0.59418 | 0.13817 | 0.30995 | 0.34898 | -0.08228 | Individual classification success index |
| IS | 1.97632 | 1.11569 | 1.46374 | 3.21607 | 4.4227 | 4.72502 | 3.32948 | Information score |
| J | 0.63356 | 0.60491 | 0.66262 | 0.3963 | 0.46875 | 0.49524 | 0.28311 | Jaccard index |
| LS | 3.93487 | 2.16699 | 2.75822 | 9.29253 | 21.44699 | 26.44679 | 10.05247 | Lift score |
| MCC | 0.7222 | 0.62784 | 0.71461 | 0.54029 | 0.63586 | 0.65999 | 0.42462 | Matthews correlation coefficient |
| MCCI | Strong | Moderate | Strong | Moderate | Moderate | Moderate | Weak | Matthews correlation coefficient interpretation |
| MCEN | 0.4345 | 0.49506 | 0.41579 | 0.60817 | 0.54899 | 0.55812 | 0.66645 | Modified confusion entropy |
| MK | 0.7519 | 0.60368 | 0.71295 | 0.56638 | 0.74315 | 0.75241 | 0.51292 | Markedness |
| N | 2437 | 2091 | 2192 | 2880 | 2969 | 2989 | 2910 | Condition negative |
| NLR | 0.2754 | 0.21259 | 0.21808 | 0.47138 | 0.45244 | 0.41797 | 0.64221 | Negative likelihood ratio |
| NLRI | Poor | Poor | Poor | Poor | Poor | Poor | Negligible | Negative likelihood ratio interpretation |
| NPV | 0.93245 | 0.90881 | 0.91899 | 0.96861 | 0.98366 | 0.98771 | 0.96425 | Negative predictive value |
| OC | 0.81944 | 0.82371 | 0.80023 | 0.59777 | 0.75949 | 0.76471 | 0.54867 | Overlap coefficient |
| OOC | 0.77679 | 0.75655 | 0.79708 | 0.56836 | 0.64658 | 0.66843 | 0.44998 | Otsuka-Ochiai coefficient |
| OP | 0.78084 | 0.82412 | 0.81523 | 0.66026 | 0.69091 | 0.72287 | 0.49511 | Optimized precision |
| P | 641 | 987 | 886 | 198 | 109 | 89 | 168 | Condition positive or support |
| PLR | 17.25465 | 4.82458 | 9.53312 | 21.61616 | 86.01642 | 109.14888 | 21.05742 | Positive likelihood ratio |
| PLRI | Good | Poor | Fair | Good | Good | Good | Good | Positive likelihood ratio interpretation |
| POP | 3078 | 3078 | 3078 | 3078 | 3078 | 3078 | 3078 | Population |
| PPV | 0.81944 | 0.69487 | 0.79395 | 0.59777 | 0.75949 | 0.76471 | 0.54867 | Precision or positive predictive value |
| PRE | 0.20825 | 0.32066 | 0.28785 | 0.06433 | 0.03541 | 0.02891 | 0.05458 | Prevalence |
| Q | 0.96858 | 0.91559 | 0.95527 | 0.95732 | 0.98954 | 0.99237 | 0.94081 | Yule Q - coefficient of colligation |
| QI | Strong | Strong | Strong | Strong | Strong | Strong | Strong | Yule Q interpretation |
| RACC | 0.03897 | 0.12189 | 0.08351 | 0.00374 | 0.00091 | 0.00064 | 0.002 | Random accuracy |
| RACCU | 0.03908 | 0.12277 | 0.08351 | 0.00375 | 0.00093 | 0.00065 | 0.00208 | Random accuracy unbiased |
| TN | 2333 | 1734 | 2008 | 2808 | 2950 | 2973 | 2859 | True negative/correct rejection |
| TNR | 0.95732 | 0.82927 | 0.91606 | 0.975 | 0.9936 | 0.99465 | 0.98247 | Specificity or true negative rate |
| TON | 2502 | 1908 | 2185 | 2899 | 2999 | 3010 | 2965 | Test outcome negative |
| TOP | 576 | 1170 | 893 | 179 | 79 | 68 | 113 | Test outcome positive |
| TP | 472 | 813 | 709 | 107 | 60 | 52 | 62 | True positive/hit |
| TPR | 0.73635 | 0.82371 | 0.80023 | 0.5404 | 0.55046 | 0.58427 | 0.36905 | Sensitivity, recall, hit rate, or true positive rate |
| Y | 0.69367 | 0.65298 | 0.71628 | 0.5154 | 0.54406 | 0.57892 | 0.35152 | Youden index |
| dInd | 0.26708 | 0.24541 | 0.21669 | 0.46028 | 0.44959 | 0.41576 | 0.6312 | Distance index |
| sInd | 0.81114 | 0.82647 | 0.84677 | 0.67454 | 0.68209 | 0.70601 | 0.55368 | Similarity index |

Generated By PyCM Version 3.3
