# Supplementary material for: TopEC: prediction of Enzyme Commission classes by 3D graph neural networks and localized 3D protein descriptor
Source: Nat Commun. 2025 Mar 20;16:2737. doi: 10.1038/s41467-025-57324-5 (PMC11923149; doi:10.1038/s41467-025-57324-5)
Supplement: Supplementary file 3 — Supplementary Data 1 [file 41467_2025_57324_MOESM3_ESM.zip › Data_S1/table1/mainclass/TopEC_distances_angles/Combined_FOLD.html]

PyCM Report


# PyCM Report

## Dataset Type :

- Multi-Class Classification
- Imbalanced

Note 1 : Recommended statistics for this type of classification highlighted in aqua

Note 2 : The recommender system assumes that the input is the result of classification over the whole data rather than just a part of it.
If the confusion matrix is the result of test data classification, the recommendation is not valid.

## Confusion Matrix :

|  |  |  |  |  |  |  |  |  |  |  |  |  |  |  |  |  |  |  |  |  |  |  |  |  |  |  |  |  |  |  |  |  |  |  |  |  |  |  |  |  |  |  |  |  |  |  |  |  |  |  |  |  |  |  |  |  |  |  |  |  |  |  |  |  |  |
| --- | --- | --- | --- | --- | --- | --- | --- | --- | --- | --- | --- | --- | --- | --- | --- | --- | --- | --- | --- | --- | --- | --- | --- | --- | --- | --- | --- | --- | --- | --- | --- | --- | --- | --- | --- | --- | --- | --- | --- | --- | --- | --- | --- | --- | --- | --- | --- | --- | --- | --- | --- | --- | --- | --- | --- | --- | --- | --- | --- | --- | --- | --- | --- | --- | --- |
| Actual | Predict  |  |  |  |  |  |  |  |  | | --- | --- | --- | --- | --- | --- | --- | --- | |  | 0 | 1 | 2 | 3 | 4 | 5 | 6 | | 0 | 467 | 70 | 32 | 4 | 4 | 0 | 0 | | 1 | 128 | 854 | 59 | 8 | 0 | 1 | 2 | | 2 | 52 | 59 | 448 | 6 | 1 | 7 | 9 | | 3 | 69 | 77 | 53 | 9 | 0 | 0 | 7 | | 4 | 70 | 93 | 70 | 6 | 14 | 3 | 0 | | 5 | 56 | 50 | 23 | 2 | 1 | 9 | 0 | | 6 | 12 | 14 | 21 | 1 | 0 | 0 | 7 | |

## Overall Statistics :

|  |  |
| --- | --- |
| 95% CI | (0.61056,0.64587) |
| ACC Macro | 0.89378 |
| ARI | 0.35143 |
| AUNP | 0.74891 |
| AUNU | 0.65546 |
| Bangdiwala B | 0.52182 |
| Bennett S | 0.56625 |
| CBA | 0.31011 |
| CSI | -0.10833 |
| Chi-Squared | 2579.5209 |
| Chi-Squared DF | 36 |
| Conditional Entropy | 1.21869 |
| Cramer V | 0.3865 |
| Cross Entropy | 2.80723 |
| F1 Macro | 0.36587 |
| F1 Micro | 0.62821 |
| FNR Macro | 0.61735 |
| FNR Micro | 0.37179 |
| FPR Macro | 0.07174 |
| FPR Micro | 0.06196 |
| Gwet AC1 | 0.57691 |
| Hamming Loss | 0.37179 |
| Joint Entropy | 3.59295 |
| KL Divergence | 0.43297 |
| Kappa | 0.49367 |
| Kappa 95% CI | (0.46962,0.51771) |
| Kappa No Prevalence | 0.25643 |
| Kappa Standard Error | 0.01227 |
| Kappa Unbiased | 0.48899 |
| Krippendorff Alpha | 0.48908 |
| Lambda A | 0.41512 |
| Lambda B | 0.48104 |
| Mutual Information | 0.562 |
| NIR | 0.36553 |
| Overall ACC | 0.62821 |
| Overall CEN | 0.40966 |
| Overall J | (1.86703,0.26672) |
| Overall MCC | 0.504 |
| Overall MCEN | 0.52953 |
| Overall RACC | 0.26573 |
| Overall RACCU | 0.27245 |
| P-Value | None |
| PPV Macro | 0.50902 |
| PPV Micro | 0.62821 |
| Pearson C | 0.6875 |
| Phi-Squared | 0.89629 |
| RCI | 0.2367 |
| RR | 411.14286 |
| Reference Entropy | 2.37426 |
| Response Entropy | 1.78069 |
| SOA1(Landis & Koch) | Moderate |
| SOA2(Fleiss) | Intermediate to Good |
| SOA3(Altman) | Moderate |
| SOA4(Cicchetti) | Fair |
| SOA5(Cramer) | Moderate |
| SOA6(Matthews) | Moderate |
| Scott PI | 0.48899 |
| Standard Error | 0.00901 |
| TNR Macro | 0.92826 |
| TNR Micro | 0.93804 |
| TPR Macro | 0.38265 |
| TPR Micro | 0.62821 |
| Zero-one Loss | 1070 |

## Class Statistics :

|  |  |  |  |  |  |  |  |  |
| --- | --- | --- | --- | --- | --- | --- | --- | --- |
| Class | 0 | 1 | 2 | 3 | 4 | 5 | 6 | Description |
| ACC | 0.82731 | 0.80507 | 0.86379 | 0.91904 | 0.91383 | 0.95031 | 0.97707 | Accuracy |
| AGF | 0.82445 | 0.82448 | 0.8276 | 0.2172 | 0.24981 | 0.27226 | 0.37517 | Adjusted F-score |
| AGM | 0.82553 | 0.80443 | 0.85368 | 0.58145 | 0.59787 | 0.61472 | 0.67154 | Adjusted geometric mean |
| AM | 277 | 165 | 124 | -179 | -236 | -121 | -30 | Difference between automatic and manual classification |
| AUC | 0.82059 | 0.8065 | 0.8287 | 0.51586 | 0.5262 | 0.52991 | 0.56045 | Area under the ROC curve |
| AUCI | Very Good | Very Good | Very Good | Poor | Poor | Poor | Poor | AUC value interpretation |
| AUPR | 0.6781 | 0.75676 | 0.70216 | 0.14593 | 0.37734 | 0.25691 | 0.20364 | Area under the PR curve |
| BCD | 0.04812 | 0.02867 | 0.02154 | 0.0311 | 0.041 | 0.02102 | 0.00521 | Bray-Curtis dissimilarity |
| BM | 0.64117 | 0.61299 | 0.65739 | 0.03172 | 0.0524 | 0.05981 | 0.1209 | Informedness or bookmaker informedness |
| CEN | 0.42762 | 0.34208 | 0.4129 | 0.628 | 0.52191 | 0.53791 | 0.62241 | Confusion entropy |
| DOR | 20.9969 | 17.38323 | 26.40935 | 4.26537 | 25.22314 | 16.89669 | 22.72569 | Diagnostic odds ratio |
| DP | 0.72894 | 0.68372 | 0.78386 | 0.34731 | 0.77285 | 0.67692 | 0.74789 | Discriminant power |
| DPI | Poor | Poor | Poor | Poor | Poor | Poor | Poor | Discriminant power interpretation |
| ERR | 0.17269 | 0.19493 | 0.13621 | 0.08096 | 0.08617 | 0.04969 | 0.02293 | Error rate |
| F0.5 | 0.58477 | 0.72128 | 0.65766 | 0.12535 | 0.20833 | 0.20362 | 0.22581 | F0.5 score |
| F1 | 0.65269 | 0.75275 | 0.69565 | 0.07171 | 0.10145 | 0.1118 | 0.175 | F1 score - harmonic mean of precision and sensitivity |
| F2 | 0.73846 | 0.7871 | 0.7383 | 0.05022 | 0.06705 | 0.07705 | 0.14286 | F2 score |
| FDR | 0.45316 | 0.29827 | 0.36544 | 0.75 | 0.3 | 0.55 | 0.72 | False discovery rate |
| FN | 110 | 198 | 134 | 206 | 242 | 132 | 48 | False negative/miss/type 2 error |
| FNR | 0.19064 | 0.18821 | 0.23024 | 0.95814 | 0.94531 | 0.93617 | 0.87273 | Miss rate or false negative rate |
| FOR | 0.05435 | 0.11921 | 0.06169 | 0.07248 | 0.08467 | 0.04619 | 0.01682 | False omission rate |
| FP | 387 | 363 | 258 | 27 | 6 | 11 | 18 | False positive/type 1 error/false alarm |
| FPR | 0.16819 | 0.1988 | 0.11237 | 0.01014 | 0.00229 | 0.00402 | 0.00638 | Fall-out or false positive rate |
| G | 0.66527 | 0.75475 | 0.6989 | 0.1023 | 0.19566 | 0.16948 | 0.18878 | G-measure geometric mean of precision and sensitivity |
| GI | 0.64117 | 0.61299 | 0.65739 | 0.03172 | 0.0524 | 0.05981 | 0.1209 | Gini index |
| GM | 0.82051 | 0.80648 | 0.8266 | 0.20356 | 0.23359 | 0.25214 | 0.35561 | G-mean geometric mean of specificity and sensitivity |
| IBA | 0.65812 | 0.65729 | 0.60273 | 0.00215 | 0.00311 | 0.00431 | 0.0169 | Index of balanced accuracy |
| ICSI | 0.3562 | 0.51351 | 0.40432 | -0.70814 | -0.24531 | -0.48617 | -0.59273 | Individual classification success index |
| IS | 1.44761 | 0.94091 | 1.64981 | 1.74266 | 2.97628 | 3.1993 | 3.87299 | Information score |
| J | 0.48444 | 0.60353 | 0.53333 | 0.03719 | 0.05344 | 0.05921 | 0.09589 | Jaccard index |
| LS | 2.72756 | 1.91974 | 3.13791 | 3.34651 | 7.86953 | 9.18511 | 14.65164 | Lift score |
| MCC | 0.56193 | 0.59756 | 0.61367 | 0.07504 | 0.17956 | 0.15541 | 0.17837 | Matthews correlation coefficient |
| MCCI | Moderate | Moderate | Moderate | Negligible | Negligible | Negligible | Negligible | Matthews correlation coefficient interpretation |
| MCEN | 0.55281 | 0.47319 | 0.55284 | 0.6372 | 0.52997 | 0.54798 | 0.64878 | Modified confusion entropy |
| MK | 0.49249 | 0.58252 | 0.57287 | 0.17752 | 0.61533 | 0.40381 | 0.26318 | Markedness |
| N | 2301 | 1826 | 2296 | 2663 | 2622 | 2737 | 2823 | Condition negative |
| NLR | 0.22919 | 0.23491 | 0.25939 | 0.96795 | 0.94748 | 0.93995 | 0.87833 | Negative likelihood ratio |
| NLRI | Poor | Poor | Poor | Negligible | Negligible | Negligible | Negligible | Negative likelihood ratio interpretation |
| NPV | 0.94565 | 0.88079 | 0.93831 | 0.92752 | 0.91533 | 0.95381 | 0.98318 | Negative predictive value |
| OC | 0.80936 | 0.81179 | 0.76976 | 0.25 | 0.7 | 0.45 | 0.28 | Overlap coefficient |
| OOC | 0.66527 | 0.75475 | 0.6989 | 0.1023 | 0.19566 | 0.16948 | 0.18878 | Otsuka-Ochiai coefficient |
| OP | 0.81363 | 0.79851 | 0.79268 | 0.00019 | 0.01776 | 0.07077 | 0.20416 | Optimized precision |
| P | 577 | 1052 | 582 | 215 | 256 | 141 | 55 | Condition positive or support |
| PLR | 4.81223 | 4.08353 | 6.85026 | 4.12868 | 23.89844 | 15.88201 | 19.96061 | Positive likelihood ratio |
| PLRI | Poor | Poor | Fair | Poor | Good | Good | Good | Positive likelihood ratio interpretation |
| POP | 2878 | 2878 | 2878 | 2878 | 2878 | 2878 | 2878 | Population |
| PPV | 0.54684 | 0.70173 | 0.63456 | 0.25 | 0.7 | 0.45 | 0.28 | Precision or positive predictive value |
| PRE | 0.20049 | 0.36553 | 0.20222 | 0.0747 | 0.08895 | 0.04899 | 0.01911 | Prevalence |
| Q | 0.90908 | 0.89121 | 0.92703 | 0.62016 | 0.92373 | 0.88825 | 0.9157 | Yule Q - coefficient of colligation |
| QI | Strong | Strong | Strong | Moderate | Strong | Strong | Strong | Yule Q interpretation |
| RACC | 0.05949 | 0.15457 | 0.04961 | 0.00093 | 0.00062 | 0.00034 | 0.00017 | Random accuracy |
| RACCU | 0.06181 | 0.15539 | 0.05007 | 0.0019 | 0.0023 | 0.00078 | 0.00019 | Random accuracy unbiased |
| TN | 1914 | 1463 | 2038 | 2636 | 2616 | 2726 | 2805 | True negative/correct rejection |
| TNR | 0.83181 | 0.8012 | 0.88763 | 0.98986 | 0.99771 | 0.99598 | 0.99362 | Specificity or true negative rate |
| TON | 2024 | 1661 | 2172 | 2842 | 2858 | 2858 | 2853 | Test outcome negative |
| TOP | 854 | 1217 | 706 | 36 | 20 | 20 | 25 | Test outcome positive |
| TP | 467 | 854 | 448 | 9 | 14 | 9 | 7 | True positive/hit |
| TPR | 0.80936 | 0.81179 | 0.76976 | 0.04186 | 0.05469 | 0.06383 | 0.12727 | Sensitivity, recall, hit rate, or true positive rate |
| Y | 0.64117 | 0.61299 | 0.65739 | 0.03172 | 0.0524 | 0.05981 | 0.1209 | Youden index |
| dInd | 0.25423 | 0.27376 | 0.2562 | 0.95819 | 0.94532 | 0.93618 | 0.87275 | Distance index |
| sInd | 0.82023 | 0.80642 | 0.81884 | 0.32246 | 0.33156 | 0.33802 | 0.38287 | Similarity index |

Generated By PyCM Version 3.3

/html>
