# Supplementary material for: TopEC: prediction of Enzyme Commission classes by 3D graph neural networks and localized 3D protein descriptor
Source: Nat Commun. 2025 Mar 20;16:2737. doi: 10.1038/s41467-025-57324-5 (PMC11923149; doi:10.1038/s41467-025-57324-5)
Supplement: Supplementary file 3 — Supplementary Data 1 [file 41467_2025_57324_MOESM3_ESM.zip › Data_S1/table1/mainclass/TopEC_distances_angles/BindingMOAD_TEMP.html]

PyCM Report


# PyCM Report

## Dataset Type :

- Multi-Class Classification
- Imbalanced

Note 1 : Recommended statistics for this type of classification highlighted in aqua

Note 2 : The recommender system assumes that the input is the result of classification over the whole data rather than just a part of it.
If the confusion matrix is the result of test data classification, the recommendation is not valid.

## Confusion Matrix :

|  |  |  |  |  |  |  |  |  |  |  |  |  |  |  |  |  |  |  |  |  |  |  |  |  |  |  |  |  |  |  |  |  |  |  |  |  |  |  |  |  |  |  |  |  |  |  |  |  |  |  |  |  |  |  |  |  |  |  |  |  |  |  |  |  |  |
| --- | --- | --- | --- | --- | --- | --- | --- | --- | --- | --- | --- | --- | --- | --- | --- | --- | --- | --- | --- | --- | --- | --- | --- | --- | --- | --- | --- | --- | --- | --- | --- | --- | --- | --- | --- | --- | --- | --- | --- | --- | --- | --- | --- | --- | --- | --- | --- | --- | --- | --- | --- | --- | --- | --- | --- | --- | --- | --- | --- | --- | --- | --- | --- | --- | --- |
| Actual | Predict  |  |  |  |  |  |  |  |  | | --- | --- | --- | --- | --- | --- | --- | --- | |  | 0 | 1 | 2 | 3 | 4 | 5 | 6 | | 0 | 349 | 56 | 15 | 3 | 0 | 0 | 0 | | 1 | 25 | 660 | 59 | 9 | 0 | 1 | 0 | | 2 | 18 | 52 | 597 | 3 | 0 | 0 | 0 | | 3 | 16 | 19 | 15 | 73 | 0 | 0 | 2 | | 4 | 7 | 18 | 2 | 1 | 49 | 0 | 0 | | 5 | 3 | 19 | 5 | 3 | 0 | 28 | 0 | | 6 | 5 | 24 | 41 | 2 | 1 | 2 | 1 | |

## Overall Statistics :

|  |  |
| --- | --- |
| 95% CI | (0.78823,0.82148) |
| ACC Macro | 0.94424 |
| ARI | 0.58371 |
| AUNP | 0.8614 |
| AUNU | 0.78789 |
| Bangdiwala B | 0.69466 |
| Bennett S | 0.77233 |
| CBA | 0.59043 |
| CSI | 0.38823 |
| Chi-Squared | 5915.60667 |
| Chi-Squared DF | 36 |
| Conditional Entropy | 0.84884 |
| Cramer V | 0.67204 |
| Cross Entropy | 2.35862 |
| F1 Macro | 0.65604 |
| F1 Micro | 0.80486 |
| FNR Macro | 0.38461 |
| FNR Micro | 0.19514 |
| FPR Macro | 0.0396 |
| FPR Micro | 0.03252 |
| Gwet AC1 | 0.77823 |
| Hamming Loss | 0.19514 |
| Joint Entropy | 3.07453 |
| KL Divergence | 0.13293 |
| Kappa | 0.72949 |
| Kappa 95% CI | (0.70644,0.75253) |
| Kappa No Prevalence | 0.60971 |
| Kappa Standard Error | 0.01176 |
| Kappa Unbiased | 0.72908 |
| Krippendorff Alpha | 0.72914 |
| Lambda A | 0.70259 |
| Lambda B | 0.71086 |
| Mutual Information | 1.08897 |
| NIR | 0.3454 |
| Overall ACC | 0.80486 |
| Overall CEN | 0.26162 |
| Overall J | (3.74267,0.53467) |
| Overall MCC | 0.73207 |
| Overall MCEN | 0.37581 |
| Overall RACC | 0.27861 |
| Overall RACCU | 0.2797 |
| P-Value | None |
| PPV Macro | 0.77284 |
| PPV Micro | 0.80486 |
| Pearson C | 0.85466 |
| Phi-Squared | 2.70985 |
| RCI | 0.48927 |
| RR | 311.85714 |
| Reference Entropy | 2.22569 |
| Response Entropy | 1.93781 |
| SOA1(Landis & Koch) | Substantial |
| SOA2(Fleiss) | Intermediate to Good |
| SOA3(Altman) | Good |
| SOA4(Cicchetti) | Good |
| SOA5(Cramer) | Strong |
| SOA6(Matthews) | Strong |
| Scott PI | 0.72908 |
| Standard Error | 0.00848 |
| TNR Macro | 0.9604 |
| TNR Micro | 0.96748 |
| TPR Macro | 0.61539 |
| TPR Micro | 0.80486 |
| Zero-one Loss | 426 |

## Class Statistics :

|  |  |  |  |  |  |  |  |  |
| --- | --- | --- | --- | --- | --- | --- | --- | --- |
| Class | 0 | 1 | 2 | 3 | 4 | 5 | 6 | Description |
| ACC | 0.9322 | 0.87082 | 0.9038 | 0.96656 | 0.98672 | 0.98488 | 0.96473 | Accuracy |
| AGF | 0.88903 | 0.8848 | 0.90721 | 0.77522 | 0.82285 | 0.72541 | 0.12583 | Adjusted F-score |
| AGM | 0.91979 | 0.87052 | 0.90399 | 0.87166 | 0.89672 | 0.8444 | 0.54902 | Adjusted geometric mean |
| AM | 0 | 94 | 64 | -31 | -27 | -27 | -73 | Difference between automatic and manual classification |
| AUC | 0.89151 | 0.87189 | 0.90025 | 0.7869 | 0.81794 | 0.74067 | 0.5061 | Area under the ROC curve |
| AUCI | Very Good | Very Good | Excellent | Good | Very Good | Good | Poor | AUC value interpretation |
| AUPR | 0.82506 | 0.82682 | 0.8522 | 0.6803 | 0.80818 | 0.69299 | 0.17325 | Area under the PR curve |
| BCD | 0.0 | 0.02153 | 0.01466 | 0.0071 | 0.00618 | 0.00618 | 0.01672 | Bray-Curtis dissimilarity |
| BM | 0.78301 | 0.74377 | 0.8005 | 0.5738 | 0.63589 | 0.48135 | 0.01221 | Informedness or bookmaker informedness |
| CEN | 0.25042 | 0.26853 | 0.22288 | 0.40434 | 0.23273 | 0.34435 | 0.48756 | Confusion entropy |
| DOR | 107.45325 | 46.34789 | 82.13899 | 136.17308 | 3683.75 | 660.17778 | 14.03333 | Diagnostic odds ratio |
| DP | 1.11987 | 0.91853 | 1.05555 | 1.17659 | 1.9662 | 1.55456 | 0.63246 | Discriminant power |
| DPI | Limited | Poor | Limited | Limited | Limited | Limited | Poor | Discriminant power interpretation |
| ERR | 0.0678 | 0.12918 | 0.0962 | 0.03344 | 0.01328 | 0.01512 | 0.03527 | Error rate |
| F0.5 | 0.82506 | 0.79595 | 0.82779 | 0.72854 | 0.88448 | 0.76923 | 0.05682 | F0.5 score |
| F1 | 0.82506 | 0.82397 | 0.85043 | 0.66667 | 0.77165 | 0.62921 | 0.02532 | F1 score - harmonic mean of precision and sensitivity |
| F2 | 0.82506 | 0.85404 | 0.87434 | 0.61448 | 0.68436 | 0.53232 | 0.01629 | F2 score |
| FDR | 0.17494 | 0.2217 | 0.18665 | 0.2234 | 0.02 | 0.09677 | 0.66667 | False discovery rate |
| FN | 74 | 94 | 73 | 52 | 28 | 30 | 75 | False negative/miss/type 2 error |
| FNR | 0.17494 | 0.12467 | 0.10896 | 0.416 | 0.36364 | 0.51724 | 0.98684 | Miss rate or false negative rate |
| FOR | 0.04205 | 0.07041 | 0.05038 | 0.02489 | 0.01313 | 0.01394 | 0.0344 | False omission rate |
| FP | 74 | 188 | 137 | 21 | 1 | 3 | 2 | False positive/type 1 error/false alarm |
| FPR | 0.04205 | 0.13156 | 0.09055 | 0.0102 | 0.00047 | 0.00141 | 0.00095 | Fall-out or false positive rate |
| G | 0.82506 | 0.82539 | 0.85131 | 0.67345 | 0.78971 | 0.66033 | 0.06623 | G-measure geometric mean of precision and sensitivity |
| GI | 0.78301 | 0.74377 | 0.8005 | 0.5738 | 0.63589 | 0.48135 | 0.01221 | Gini index |
| GM | 0.88903 | 0.87188 | 0.9002 | 0.76029 | 0.79753 | 0.69432 | 0.11465 | G-mean geometric mean of specificity and sensitivity |
| IBA | 0.68533 | 0.76541 | 0.79545 | 0.34347 | 0.40507 | 0.23341 | 0.00019 | Index of balanced accuracy |
| ICSI | 0.65012 | 0.65363 | 0.7044 | 0.3606 | 0.61636 | 0.38598 | -0.65351 | Individual classification success index |
| IS | 2.09015 | 1.17208 | 1.40603 | 3.76155 | 4.79616 | 5.08727 | 3.25921 | Information score |
| J | 0.70221 | 0.70064 | 0.73978 | 0.5 | 0.62821 | 0.45902 | 0.01282 | Jaccard index |
| LS | 4.25793 | 2.25336 | 2.65007 | 13.56247 | 27.78364 | 33.99555 | 9.57456 | Lift score |
| MCC | 0.78301 | 0.72561 | 0.78151 | 0.65675 | 0.78411 | 0.65426 | 0.06041 | Matthews correlation coefficient |
| MCCI | Strong | Strong | Strong | Moderate | Strong | Moderate | Negligible | Matthews correlation coefficient interpretation |
| MCEN | 0.36252 | 0.39271 | 0.32978 | 0.52493 | 0.306 | 0.42017 | 0.48875 | Modified confusion entropy |
| MK | 0.78301 | 0.70789 | 0.76297 | 0.7517 | 0.96687 | 0.88929 | 0.29893 | Markedness |
| N | 1760 | 1429 | 1513 | 2058 | 2106 | 2125 | 2107 | Condition negative |
| NLR | 0.18262 | 0.14355 | 0.1198 | 0.42029 | 0.36381 | 0.51797 | 0.98778 | Negative likelihood ratio |
| NLRI | Fair | Fair | Fair | Poor | Poor | Negligible | Negligible | Negative likelihood ratio interpretation |
| NPV | 0.95795 | 0.92959 | 0.94962 | 0.97511 | 0.98687 | 0.98606 | 0.9656 | Negative predictive value |
| OC | 0.82506 | 0.87533 | 0.89104 | 0.7766 | 0.98 | 0.90323 | 0.33333 | Overlap coefficient |
| OOC | 0.82506 | 0.82539 | 0.85131 | 0.67345 | 0.78971 | 0.66033 | 0.06623 | Otsuka-Ochiai coefficient |
| OP | 0.85767 | 0.86687 | 0.89358 | 0.70871 | 0.76472 | 0.63667 | -0.00927 | Optimized precision |
| P | 423 | 754 | 670 | 125 | 77 | 58 | 76 | Condition positive or support |
| PLR | 19.62303 | 6.65345 | 9.84052 | 57.232 | 1340.18182 | 341.95402 | 13.86184 | Positive likelihood ratio |
| PLRI | Good | Fair | Fair | Good | Good | Good | Good | Positive likelihood ratio interpretation |
| POP | 2183 | 2183 | 2183 | 2183 | 2183 | 2183 | 2183 | Population |
| PPV | 0.82506 | 0.7783 | 0.81335 | 0.7766 | 0.98 | 0.90323 | 0.33333 | Precision or positive predictive value |
| PRE | 0.19377 | 0.3454 | 0.30692 | 0.05726 | 0.03527 | 0.02657 | 0.03481 | Prevalence |
| Q | 0.98156 | 0.95776 | 0.97594 | 0.98542 | 0.99946 | 0.99698 | 0.86696 | Yule Q - coefficient of colligation |
| QI | Strong | Strong | Strong | Strong | Strong | Strong | Strong | Yule Q interpretation |
| RACC | 0.03755 | 0.13417 | 0.1032 | 0.00247 | 0.00081 | 0.00038 | 5e-05 | Random accuracy |
| RACCU | 0.03755 | 0.13463 | 0.10341 | 0.00252 | 0.00085 | 0.00042 | 0.00033 | Random accuracy unbiased |
| TN | 1686 | 1241 | 1376 | 2037 | 2105 | 2122 | 2105 | True negative/correct rejection |
| TNR | 0.95795 | 0.86844 | 0.90945 | 0.9898 | 0.99953 | 0.99859 | 0.99905 | Specificity or true negative rate |
| TON | 1760 | 1335 | 1449 | 2089 | 2133 | 2152 | 2180 | Test outcome negative |
| TOP | 423 | 848 | 734 | 94 | 50 | 31 | 3 | Test outcome positive |
| TP | 349 | 660 | 597 | 73 | 49 | 28 | 1 | True positive/hit |
| TPR | 0.82506 | 0.87533 | 0.89104 | 0.584 | 0.63636 | 0.48276 | 0.01316 | Sensitivity, recall, hit rate, or true positive rate |
| Y | 0.78301 | 0.74377 | 0.8005 | 0.5738 | 0.63589 | 0.48135 | 0.01221 | Youden index |
| dInd | 0.17992 | 0.18125 | 0.14167 | 0.41613 | 0.36364 | 0.51724 | 0.98684 | Distance index |
| sInd | 0.87278 | 0.87184 | 0.89982 | 0.70576 | 0.74287 | 0.63425 | 0.3022 | Similarity index |

Generated By PyCM Version 3.3
