# Supplementary material for: TopEC: prediction of Enzyme Commission classes by 3D graph neural networks and localized 3D protein descriptor
Source: Nat Commun. 2025 Mar 20;16:2737. doi: 10.1038/s41467-025-57324-5 (PMC11923149; doi:10.1038/s41467-025-57324-5)
Supplement: Supplementary file 3 — Supplementary Data 1 [file 41467_2025_57324_MOESM3_ESM.zip › Data_S1/table1/mainclass/TopEC_distances_angles/BindingMOAD_FOLD.html]

PyCM Report


# PyCM Report

## Dataset Type :

- Multi-Class Classification
- Imbalanced

Note 1 : Recommended statistics for this type of classification highlighted in aqua

Note 2 : The recommender system assumes that the input is the result of classification over the whole data rather than just a part of it.
If the confusion matrix is the result of test data classification, the recommendation is not valid.

## Confusion Matrix :

|  |  |  |  |  |  |  |  |  |  |  |  |  |  |  |  |  |  |  |  |  |  |  |  |  |  |  |  |  |  |  |  |  |  |  |  |  |  |  |  |  |  |  |  |  |  |  |  |  |  |  |  |  |  |  |  |  |  |  |  |  |  |  |  |  |  |
| --- | --- | --- | --- | --- | --- | --- | --- | --- | --- | --- | --- | --- | --- | --- | --- | --- | --- | --- | --- | --- | --- | --- | --- | --- | --- | --- | --- | --- | --- | --- | --- | --- | --- | --- | --- | --- | --- | --- | --- | --- | --- | --- | --- | --- | --- | --- | --- | --- | --- | --- | --- | --- | --- | --- | --- | --- | --- | --- | --- | --- | --- | --- | --- | --- | --- |
| Actual | Predict  |  |  |  |  |  |  |  |  | | --- | --- | --- | --- | --- | --- | --- | --- | |  | 0 | 1 | 2 | 3 | 4 | 5 | 6 | | 0 | 265 | 106 | 15 | 17 | 0 | 3 | 1 | | 1 | 25 | 764 | 14 | 3 | 0 | 0 | 1 | | 2 | 37 | 60 | 249 | 42 | 1 | 8 | 6 | | 3 | 18 | 77 | 5 | 30 | 0 | 0 | 2 | | 4 | 44 | 79 | 21 | 12 | 1 | 13 | 1 | | 5 | 6 | 29 | 2 | 0 | 0 | 10 | 1 | | 6 | 2 | 10 | 4 | 1 | 0 | 0 | 0 | |

## Overall Statistics :

|  |  |
| --- | --- |
| 95% CI | (0.64371,0.68526) |
| ACC Macro | 0.90414 |
| ARI | 0.4001 |
| AUNP | 0.75591 |
| AUNU | 0.65493 |
| Bangdiwala B | 0.59229 |
| Bennett S | 0.60856 |
| CBA | 0.34136 |
| CSI | -0.15903 |
| Chi-Squared | 1977.79743 |
| Chi-Squared DF | 36 |
| Conditional Entropy | 1.11039 |
| Cramer V | 0.40751 |
| Cross Entropy | 2.69757 |
| F1 Macro | 0.3796 |
| F1 Micro | 0.66448 |
| FNR Macro | 0.62041 |
| FNR Micro | 0.33552 |
| FPR Macro | 0.06974 |
| FPR Micro | 0.05592 |
| Gwet AC1 | 0.62132 |
| Hamming Loss | 0.33552 |
| Joint Entropy | 3.32745 |
| KL Divergence | 0.48051 |
| Kappa | 0.51571 |
| Kappa 95% CI | (0.48573,0.54569) |
| Kappa No Prevalence | 0.32897 |
| Kappa Standard Error | 0.0153 |
| Kappa Unbiased | 0.50945 |
| Krippendorff Alpha | 0.50957 |
| Lambda A | 0.45246 |
| Lambda B | 0.40465 |
| Mutual Information | 0.61604 |
| NIR | 0.40655 |
| Overall ACC | 0.66448 |
| Overall CEN | 0.36813 |
| Overall J | (1.97203,0.28172) |
| Overall MCC | 0.53161 |
| Overall MCEN | 0.48477 |
| Overall RACC | 0.3072 |
| Overall RACCU | 0.31604 |
| P-Value | None |
| PPV Macro | 0.46138 |
| PPV Micro | 0.66448 |
| Pearson C | 0.70646 |
| Phi-Squared | 0.99637 |
| RCI | 0.27786 |
| RR | 283.57143 |
| Reference Entropy | 2.21706 |
| Response Entropy | 1.72643 |
| SOA1(Landis & Koch) | Moderate |
| SOA2(Fleiss) | Intermediate to Good |
| SOA3(Altman) | Moderate |
| SOA4(Cicchetti) | Fair |
| SOA5(Cramer) | Relatively Strong |
| SOA6(Matthews) | Moderate |
| Scott PI | 0.50945 |
| Standard Error | 0.0106 |
| TNR Macro | 0.93026 |
| TNR Micro | 0.94408 |
| TPR Macro | 0.37959 |
| TPR Micro | 0.66448 |
| Zero-one Loss | 666 |

## Class Statistics :

|  |  |  |  |  |  |  |  |  |
| --- | --- | --- | --- | --- | --- | --- | --- | --- |
| Class | 0 | 1 | 2 | 3 | 4 | 5 | 6 | Description |
| ACC | 0.86196 | 0.79647 | 0.89169 | 0.91083 | 0.91385 | 0.96877 | 0.98539 | Accuracy |
| AGF | 0.77237 | 0.88106 | 0.77124 | 0.47408 | 0.08234 | 0.46609 | 0.0 | Adjusted F-score |
| AGM | 0.83617 | 0.76682 | 0.85532 | 0.70478 | 0.51718 | 0.71734 | 0 | Adjusted geometric mean |
| AM | -10 | 318 | -93 | -27 | -169 | -14 | -5 | Difference between automatic and manual classification |
| AUC | 0.78373 | 0.82013 | 0.78965 | 0.5934 | 0.50265 | 0.59797 | 0.49695 | Area under the ROC curve |
| AUCI | Good | Very Good | Good | Poor | Poor | Poor | Poor | AUC value interpretation |
| AUPR | 0.65931 | 0.81291 | 0.71055 | 0.25649 | 0.25292 | 0.25123 | 0.0 | Area under the PR curve |
| BCD | 0.00252 | 0.0801 | 0.02343 | 0.0068 | 0.04257 | 0.00353 | 0.00126 | Bray-Curtis dissimilarity |
| BM | 0.56746 | 0.64026 | 0.57931 | 0.1868 | 0.0053 | 0.19594 | -0.0061 | Informedness or bookmaker informedness |
| CEN | 0.40133 | 0.29073 | 0.39076 | 0.56655 | 0.56392 | 0.54059 | 0.77096 | Confusion entropy |
| DOR | 20.44334 | 40.21053 | 40.31605 | 6.97255 | 10.66471 | 20.97588 | 0.0 | Diagnostic odds ratio |
| DP | 0.72255 | 0.88452 | 0.88515 | 0.46499 | 0.56674 | 0.7287 | None | Discriminant power |
| DPI | Poor | Poor | Poor | Poor | Poor | Poor | None | Discriminant power interpretation |
| ERR | 0.13804 | 0.20353 | 0.10831 | 0.08917 | 0.08615 | 0.03123 | 0.01461 | Error rate |
| F0.5 | 0.66416 | 0.7198 | 0.75776 | 0.27174 | 0.02793 | 0.27174 | 0.0 | F0.5 score |
| F1 | 0.6592 | 0.79089 | 0.69846 | 0.25316 | 0.01156 | 0.2439 | 0.0 | F1 score - harmonic mean of precision and sensitivity |
| F2 | 0.65432 | 0.87756 | 0.64776 | 0.23697 | 0.00729 | 0.22124 | 0.0 | F2 score |
| FDR | 0.33249 | 0.32089 | 0.19677 | 0.71429 | 0.5 | 0.70588 | 1.0 | False discovery rate |
| FN | 142 | 43 | 154 | 102 | 170 | 38 | 17 | False negative/miss/type 2 error |
| FNR | 0.34889 | 0.05328 | 0.38213 | 0.77273 | 0.99415 | 0.79167 | 1.0 | Miss rate or false negative rate |
| FOR | 0.08942 | 0.05 | 0.09194 | 0.05426 | 0.08573 | 0.01948 | 0.00862 | False omission rate |
| FP | 132 | 361 | 61 | 75 | 1 | 24 | 12 | False positive/type 1 error/false alarm |
| FPR | 0.08365 | 0.30645 | 0.03856 | 0.04047 | 0.00055 | 0.01239 | 0.0061 | Fall-out or false positive rate |
| G | 0.65925 | 0.80183 | 0.70448 | 0.25482 | 0.05407 | 0.24754 | 0.0 | G-measure geometric mean of precision and sensitivity |
| GI | 0.56746 | 0.64026 | 0.57931 | 0.1868 | 0.0053 | 0.19594 | -0.0061 | Gini index |
| GM | 0.77243 | 0.8103 | 0.77074 | 0.46698 | 0.07645 | 0.4536 | 0.0 | G-mean geometric mean of specificity and sensitivity |
| IBA | 0.43839 | 0.82282 | 0.38994 | 0.05839 | 4e-05 | 0.04541 | 0.0 | Index of balanced accuracy |
| ICSI | 0.31861 | 0.62583 | 0.42109 | -0.48701 | -0.49415 | -0.49755 | -1.0 | Individual classification success index |
| IS | 1.70289 | 0.74022 | 1.98416 | 2.10317 | 2.53707 | 3.60443 | None | Information score |
| J | 0.49165 | 0.65411 | 0.53664 | 0.14493 | 0.00581 | 0.13889 | 0.0 | Jaccard index |
| LS | 3.25553 | 1.67043 | 3.95634 | 4.29654 | 5.80409 | 12.16299 | 0.0 | Lift score |
| MCC | 0.57275 | 0.63466 | 0.64191 | 0.20793 | 0.04684 | 0.23198 | -0.00725 | Matthews correlation coefficient |
| MCCI | Moderate | Moderate | Moderate | Negligible | Negligible | Negligible | Negligible | Matthews correlation coefficient interpretation |
| MCEN | 0.51684 | 0.41085 | 0.52035 | 0.60208 | 0.56488 | 0.57061 | 0.77096 | Modified confusion entropy |
| MK | 0.57809 | 0.62911 | 0.71129 | 0.23146 | 0.41427 | 0.27464 | -0.00862 | Markedness |
| N | 1578 | 1178 | 1582 | 1853 | 1814 | 1937 | 1968 | Condition negative |
| NLR | 0.38074 | 0.07683 | 0.39746 | 0.80532 | 0.9947 | 0.8016 | 1.00613 | Negative likelihood ratio |
| NLRI | Poor | Good | Poor | Negligible | Negligible | Negligible | Negligible | Negative likelihood ratio interpretation |
| NPV | 0.91058 | 0.95 | 0.90806 | 0.94574 | 0.91427 | 0.98052 | 0.99138 | Negative predictive value |
| OC | 0.66751 | 0.94672 | 0.80323 | 0.28571 | 0.5 | 0.29412 | 0.0 | Overlap coefficient |
| OOC | 0.65925 | 0.80183 | 0.70448 | 0.25482 | 0.05407 | 0.24754 | 0.0 | Otsuka-Ochiai coefficient |
| OP | 0.69275 | 0.64213 | 0.67414 | 0.29383 | -0.07451 | 0.31717 | -0.01461 | Optimized precision |
| P | 407 | 807 | 403 | 132 | 171 | 48 | 17 | Condition positive or support |
| PLR | 7.78367 | 3.08928 | 16.024 | 5.61515 | 10.60819 | 16.81424 | 0.0 | Positive likelihood ratio |
| PLRI | Fair | Poor | Good | Fair | Good | Good | Negligible | Positive likelihood ratio interpretation |
| POP | 1985 | 1985 | 1985 | 1985 | 1985 | 1985 | 1985 | Population |
| PPV | 0.66751 | 0.67911 | 0.80323 | 0.28571 | 0.5 | 0.29412 | 0.0 | Precision or positive predictive value |
| PRE | 0.20504 | 0.40655 | 0.20302 | 0.0665 | 0.08615 | 0.02418 | 0.00856 | Prevalence |
| Q | 0.90673 | 0.95147 | 0.95159 | 0.74914 | 0.82854 | 0.90899 | -1.0 | Yule Q - coefficient of colligation |
| QI | Strong | Strong | Strong | Moderate | Strong | Strong | Negligible | Yule Q interpretation |
| RACC | 0.04101 | 0.23041 | 0.03171 | 0.00352 | 9e-05 | 0.00041 | 5e-05 | Random accuracy |
| RACCU | 0.04101 | 0.23683 | 0.03226 | 0.00356 | 0.0019 | 0.00043 | 5e-05 | Random accuracy unbiased |
| TN | 1446 | 817 | 1521 | 1778 | 1813 | 1913 | 1956 | True negative/correct rejection |
| TNR | 0.91635 | 0.69355 | 0.96144 | 0.95953 | 0.99945 | 0.98761 | 0.9939 | Specificity or true negative rate |
| TON | 1588 | 860 | 1675 | 1880 | 1983 | 1951 | 1973 | Test outcome negative |
| TOP | 397 | 1125 | 310 | 105 | 2 | 34 | 12 | Test outcome positive |
| TP | 265 | 764 | 249 | 30 | 1 | 10 | 0 | True positive/hit |
| TPR | 0.65111 | 0.94672 | 0.61787 | 0.22727 | 0.00585 | 0.20833 | 0.0 | Sensitivity, recall, hit rate, or true positive rate |
| Y | 0.56746 | 0.64026 | 0.57931 | 0.1868 | 0.0053 | 0.19594 | -0.0061 | Youden index |
| dInd | 0.35878 | 0.31105 | 0.38407 | 0.77379 | 0.99415 | 0.79176 | 1.00002 | Distance index |
| sInd | 0.7463 | 0.78005 | 0.72842 | 0.45285 | 0.29703 | 0.44014 | 0.29288 | Similarity index |

Generated By PyCM Version 3.3
