# Supplementary material for: TopEC: prediction of Enzyme Commission classes by 3D graph neural networks and localized 3D protein descriptor
Source: Nat Commun. 2025 Mar 20;16:2737. doi: 10.1038/s41467-025-57324-5 (PMC11923149; doi:10.1038/s41467-025-57324-5)
Supplement: Supplementary file 3 — Supplementary Data 1 [file 41467_2025_57324_MOESM3_ESM.zip › Data_S1/table1/mainclass/TopEC_distances/TopEnzyme_TEMP.html]

PyCM Report


# PyCM Report

## Dataset Type :

- Multi-Class Classification
- Imbalanced

Note 1 : Recommended statistics for this type of classification highlighted in aqua

Note 2 : The recommender system assumes that the input is the result of classification over the whole data rather than just a part of it.
If the confusion matrix is the result of test data classification, the recommendation is not valid.

## Confusion Matrix :

|  |  |  |  |  |  |  |  |  |  |  |  |  |  |  |  |  |  |  |  |  |  |  |  |  |  |  |  |  |  |  |  |  |  |  |  |  |  |  |  |  |  |  |  |  |  |  |  |  |  |  |  |  |  |  |  |  |  |  |  |  |  |  |  |  |  |
| --- | --- | --- | --- | --- | --- | --- | --- | --- | --- | --- | --- | --- | --- | --- | --- | --- | --- | --- | --- | --- | --- | --- | --- | --- | --- | --- | --- | --- | --- | --- | --- | --- | --- | --- | --- | --- | --- | --- | --- | --- | --- | --- | --- | --- | --- | --- | --- | --- | --- | --- | --- | --- | --- | --- | --- | --- | --- | --- | --- | --- | --- | --- | --- | --- | --- |
| Actual | Predict  |  |  |  |  |  |  |  |  | | --- | --- | --- | --- | --- | --- | --- | --- | |  | 0 | 1 | 2 | 3 | 4 | 5 | 6 | | 0 | 134 | 27 | 43 | 4 | 2 | 5 | 3 | | 1 | 51 | 104 | 57 | 8 | 3 | 5 | 5 | | 2 | 35 | 27 | 141 | 4 | 5 | 3 | 1 | | 3 | 18 | 22 | 17 | 8 | 6 | 2 | 0 | | 4 | 9 | 2 | 10 | 1 | 7 | 3 | 0 | | 5 | 6 | 6 | 4 | 0 | 1 | 14 | 0 | | 6 | 15 | 14 | 16 | 2 | 0 | 1 | 44 | |

## Overall Statistics :

|  |  |
| --- | --- |
| 95% CI | (0.47227,0.53778) |
| ACC Macro | 0.85858 |
| ARI | 0.15559 |
| AUNP | 0.68076 |
| AUNU | 0.66668 |
| Bangdiwala B | 0.28864 |
| Bennett S | 0.42253 |
| CBA | 0.38097 |
| CSI | -0.09731 |
| Chi-Squared | 814.20639 |
| Chi-Squared DF | 36 |
| Conditional Entropy | 1.83156 |
| Cramer V | 0.38939 |
| Cross Entropy | 2.56033 |
| F1 Macro | 0.43479 |
| F1 Micro | 0.50503 |
| FNR Macro | 0.57543 |
| FNR Micro | 0.49497 |
| FPR Macro | 0.09121 |
| FPR Micro | 0.0825 |
| Gwet AC1 | 0.4315 |
| Hamming Loss | 0.49497 |
| Joint Entropy | 4.30046 |
| KL Divergence | 0.09144 |
| Kappa | 0.36531 |
| Kappa 95% CI | (0.32331,0.40731) |
| Kappa No Prevalence | 0.01006 |
| Kappa Standard Error | 0.02143 |
| Kappa Unbiased | 0.36216 |
| Krippendorff Alpha | 0.36251 |
| Lambda A | 0.33082 |
| Lambda B | 0.29819 |
| Mutual Information | 0.40985 |
| NIR | 0.26034 |
| Overall ACC | 0.50503 |
| Overall CEN | 0.54169 |
| Overall J | (2.02877,0.28982) |
| Overall MCC | 0.36912 |
| Overall MCEN | 0.6503 |
| Overall RACC | 0.22014 |
| Overall RACCU | 0.22399 |
| P-Value | -0.0 |
| PPV Macro | 0.47812 |
| PPV Micro | 0.50503 |
| Pearson C | 0.69019 |
| Phi-Squared | 0.90973 |
| RCI | 0.16601 |
| RR | 127.85714 |
| Reference Entropy | 2.46889 |
| Response Entropy | 2.24141 |
| SOA1(Landis & Koch) | Fair |
| SOA2(Fleiss) | Poor |
| SOA3(Altman) | Fair |
| SOA4(Cicchetti) | Poor |
| SOA5(Cramer) | Moderate |
| SOA6(Matthews) | Weak |
| Scott PI | 0.36216 |
| Standard Error | 0.01671 |
| TNR Macro | 0.90879 |
| TNR Micro | 0.9175 |
| TPR Macro | 0.42457 |
| TPR Micro | 0.50503 |
| Zero-one Loss | 443 |

## Class Statistics :

|  |  |  |  |  |  |  |  |  |
| --- | --- | --- | --- | --- | --- | --- | --- | --- |
| Class | 0 | 1 | 2 | 3 | 4 | 5 | 6 | Description |
| ACC | 0.75642 | 0.74637 | 0.75196 | 0.90615 | 0.95307 | 0.95978 | 0.93631 | Accuracy |
| AGF | 0.70781 | 0.61365 | 0.72383 | 0.34241 | 0.47336 | 0.66096 | 0.70525 | Adjusted F-score |
| AGM | 0.74518 | 0.71671 | 0.74464 | 0.63823 | 0.71698 | 0.81854 | 0.83008 | Adjusted geometric mean |
| AM | 50 | -31 | 72 | -46 | -8 | 2 | -39 | Difference between automatic and manual classification |
| AUC | 0.70837 | 0.64916 | 0.71814 | 0.54324 | 0.59953 | 0.71481 | 0.73353 | Area under the ROC curve |
| AUCI | Good | Fair | Good | Poor | Poor | Good | Good | AUC value interpretation |
| AUPR | 0.55734 | 0.4806 | 0.57118 | 0.20294 | 0.25521 | 0.43793 | 0.65422 | Area under the PR curve |
| BCD | 0.02793 | 0.01732 | 0.04022 | 0.0257 | 0.00447 | 0.00112 | 0.02179 | Bray-Curtis dissimilarity |
| BM | 0.41675 | 0.29832 | 0.43628 | 0.08647 | 0.19905 | 0.42962 | 0.46705 | Informedness or bookmaker informedness |
| CEN | 0.52288 | 0.57084 | 0.50928 | 0.71387 | 0.70498 | 0.62 | 0.41357 | Confusion entropy |
| DOR | 6.46429 | 4.63977 | 6.80381 | 5.20162 | 13.93412 | 36.62539 | 80.87037 | Diagnostic odds ratio |
| DP | 0.44686 | 0.36746 | 0.45912 | 0.39483 | 0.63076 | 0.86216 | 1.05182 | Discriminant power |
| DPI | Poor | Poor | Poor | Poor | Poor | Poor | Limited | Discriminant power interpretation |
| ERR | 0.24358 | 0.25363 | 0.24804 | 0.09385 | 0.04693 | 0.04022 | 0.06369 | Error rate |
| F0.5 | 0.51938 | 0.49952 | 0.51535 | 0.22099 | 0.27344 | 0.42945 | 0.72368 | F0.5 score |
| F1 | 0.55144 | 0.47816 | 0.55952 | 0.16 | 0.25 | 0.4375 | 0.6069 | F1 score - harmonic mean of precision and sensitivity |
| F2 | 0.58772 | 0.45855 | 0.61198 | 0.12539 | 0.23026 | 0.44586 | 0.52257 | F2 score |
| FDR | 0.5 | 0.48515 | 0.51042 | 0.7037 | 0.70833 | 0.57576 | 0.16981 | False discovery rate |
| FN | 84 | 129 | 75 | 65 | 25 | 17 | 48 | False negative/miss/type 2 error |
| FNR | 0.38532 | 0.55365 | 0.34722 | 0.89041 | 0.78125 | 0.54839 | 0.52174 | Miss rate or false negative rate |
| FOR | 0.13397 | 0.18615 | 0.12356 | 0.07488 | 0.0287 | 0.01972 | 0.05701 | False omission rate |
| FP | 134 | 98 | 147 | 19 | 17 | 19 | 9 | False positive/type 1 error/false alarm |
| FPR | 0.19793 | 0.14804 | 0.21649 | 0.02311 | 0.0197 | 0.02199 | 0.01121 | Fall-out or false positive rate |
| G | 0.55438 | 0.47938 | 0.56532 | 0.1802 | 0.25259 | 0.43771 | 0.63012 | G-measure geometric mean of precision and sensitivity |
| GI | 0.41675 | 0.29832 | 0.43628 | 0.08647 | 0.19905 | 0.42962 | 0.46705 | Gini index |
| GM | 0.70215 | 0.61666 | 0.71516 | 0.32719 | 0.46308 | 0.66459 | 0.68768 | G-mean geometric mean of specificity and sensitivity |
| IBA | 0.40063 | 0.22603 | 0.44459 | 0.01421 | 0.05113 | 0.20918 | 0.23147 | Index of balanced accuracy |
| ICSI | 0.11468 | -0.0388 | 0.14236 | -0.59411 | -0.48958 | -0.12414 | 0.30845 | Individual classification success index |
| IS | 1.03756 | 0.98379 | 1.02048 | 1.86103 | 3.02814 | 3.61451 | 3.01369 | Information score |
| J | 0.38068 | 0.3142 | 0.38843 | 0.08696 | 0.14286 | 0.28 | 0.43564 | Jaccard index |
| LS | 2.05275 | 1.97765 | 2.0286 | 3.63267 | 8.15755 | 12.24829 | 8.07629 | Lift score |
| MCC | 0.39057 | 0.31314 | 0.39961 | 0.13837 | 0.22879 | 0.41688 | 0.60093 | Matthews correlation coefficient |
| MCCI | Weak | Weak | Weak | Negligible | Negligible | Weak | Moderate | Matthews correlation coefficient interpretation |
| MCEN | 0.64154 | 0.67479 | 0.62633 | 0.74531 | 0.75964 | 0.72207 | 0.51162 | Modified confusion entropy |
| MK | 0.36603 | 0.3287 | 0.36602 | 0.22141 | 0.26296 | 0.40452 | 0.77318 | Markedness |
| N | 677 | 662 | 679 | 822 | 863 | 864 | 803 | Condition negative |
| NLR | 0.48041 | 0.64985 | 0.44317 | 0.91148 | 0.79695 | 0.56072 | 0.52765 | Negative likelihood ratio |
| NLRI | Poor | Negligible | Poor | Negligible | Negligible | Negligible | Negligible | Negative likelihood ratio interpretation |
| NPV | 0.86603 | 0.81385 | 0.87644 | 0.92512 | 0.9713 | 0.98028 | 0.94299 | Negative predictive value |
| OC | 0.61468 | 0.51485 | 0.65278 | 0.2963 | 0.29167 | 0.45161 | 0.83019 | Overlap coefficient |
| OOC | 0.55438 | 0.47938 | 0.56532 | 0.1802 | 0.25259 | 0.43771 | 0.63012 | Otsuka-Ochiai coefficient |
| OP | 0.62416 | 0.43395 | 0.66094 | 0.10788 | 0.31794 | 0.59157 | 0.58832 | Optimized precision |
| P | 218 | 233 | 216 | 73 | 32 | 31 | 92 | Condition positive or support |
| PLR | 3.1055 | 3.01515 | 3.01521 | 4.74117 | 11.10478 | 20.5365 | 42.6715 | Positive likelihood ratio |
| PLRI | Poor | Poor | Poor | Poor | Good | Good | Good | Positive likelihood ratio interpretation |
| POP | 895 | 895 | 895 | 895 | 895 | 895 | 895 | Population |
| PPV | 0.5 | 0.51485 | 0.48958 | 0.2963 | 0.29167 | 0.42424 | 0.83019 | Precision or positive predictive value |
| PRE | 0.24358 | 0.26034 | 0.24134 | 0.08156 | 0.03575 | 0.03464 | 0.10279 | Prevalence |
| Q | 0.73206 | 0.64538 | 0.74371 | 0.6775 | 0.86608 | 0.94684 | 0.97557 | Yule Q - coefficient of colligation |
| QI | Moderate | Moderate | Moderate | Moderate | Strong | Strong | Strong | Yule Q interpretation |
| RACC | 0.07294 | 0.05876 | 0.07766 | 0.00246 | 0.00096 | 0.00128 | 0.00609 | Random accuracy |
| RACCU | 0.07372 | 0.05906 | 0.07928 | 0.00312 | 0.00098 | 0.00128 | 0.00656 | Random accuracy unbiased |
| TN | 543 | 564 | 532 | 803 | 846 | 845 | 794 | True negative/correct rejection |
| TNR | 0.80207 | 0.85196 | 0.78351 | 0.97689 | 0.9803 | 0.97801 | 0.98879 | Specificity or true negative rate |
| TON | 627 | 693 | 607 | 868 | 871 | 862 | 842 | Test outcome negative |
| TOP | 268 | 202 | 288 | 27 | 24 | 33 | 53 | Test outcome positive |
| TP | 134 | 104 | 141 | 8 | 7 | 14 | 44 | True positive/hit |
| TPR | 0.61468 | 0.44635 | 0.65278 | 0.10959 | 0.21875 | 0.45161 | 0.47826 | Sensitivity, recall, hit rate, or true positive rate |
| Y | 0.41675 | 0.29832 | 0.43628 | 0.08647 | 0.19905 | 0.42962 | 0.46705 | Youden index |
| dInd | 0.43319 | 0.5731 | 0.40919 | 0.89071 | 0.7815 | 0.54883 | 0.52186 | Distance index |
| sInd | 0.69369 | 0.59476 | 0.71066 | 0.37017 | 0.4474 | 0.61192 | 0.63099 | Similarity index |

Generated By PyCM Version 3.3
