# Supplementary material for: TopEC: prediction of Enzyme Commission classes by 3D graph neural networks and localized 3D protein descriptor
Source: Nat Commun. 2025 Mar 20;16:2737. doi: 10.1038/s41467-025-57324-5 (PMC11923149; doi:10.1038/s41467-025-57324-5)
Supplement: Supplementary file 3 — Supplementary Data 1 [file 41467_2025_57324_MOESM3_ESM.zip › Data_S1/table1/mainclass/TopEC_distances/TopEnzyme_FOLD.html]

PyCM Report


# PyCM Report

## Dataset Type :

- Multi-Class Classification
- Imbalanced

Note 1 : Recommended statistics for this type of classification highlighted in aqua

Note 2 : The recommender system assumes that the input is the result of classification over the whole data rather than just a part of it.
If the confusion matrix is the result of test data classification, the recommendation is not valid.

## Confusion Matrix :

|  |  |  |  |  |  |  |  |  |  |  |  |  |  |  |  |  |  |  |  |  |  |  |  |  |  |  |  |  |  |  |  |  |  |  |  |  |  |  |  |  |  |  |  |  |  |  |  |  |  |  |  |  |  |  |  |  |  |  |  |  |  |  |  |  |  |
| --- | --- | --- | --- | --- | --- | --- | --- | --- | --- | --- | --- | --- | --- | --- | --- | --- | --- | --- | --- | --- | --- | --- | --- | --- | --- | --- | --- | --- | --- | --- | --- | --- | --- | --- | --- | --- | --- | --- | --- | --- | --- | --- | --- | --- | --- | --- | --- | --- | --- | --- | --- | --- | --- | --- | --- | --- | --- | --- | --- | --- | --- | --- | --- | --- | --- |
| Actual | Predict  |  |  |  |  |  |  |  |  | | --- | --- | --- | --- | --- | --- | --- | --- | |  | 0 | 1 | 2 | 3 | 4 | 5 | 6 | | 0 | 106 | 31 | 15 | 18 | 0 | 0 | 0 | | 1 | 32 | 179 | 8 | 26 | 0 | 0 | 0 | | 2 | 31 | 37 | 90 | 21 | 0 | 0 | 0 | | 3 | 28 | 22 | 12 | 21 | 0 | 0 | 0 | | 4 | 21 | 23 | 14 | 24 | 3 | 0 | 0 | | 5 | 28 | 40 | 15 | 8 | 0 | 2 | 0 | | 6 | 2 | 31 | 4 | 1 | 0 | 0 | 0 | |

## Overall Statistics :

|  |  |
| --- | --- |
| 95% CI | (0.41642,0.48167) |
| ACC Macro | 0.84259 |
| ARI | 0.14974 |
| AUNP | 0.65171 |
| AUNU | 0.60501 |
| Bangdiwala B | 0.30544 |
| Bennett S | 0.35722 |
| CBA | 0.23666 |
| CSI | None |
| Chi-Squared | None |
| Chi-Squared DF | 36 |
| Conditional Entropy | 1.59921 |
| Cramer V | None |
| Cross Entropy | 3.1782 |
| F1 Macro | 0.27833 |
| F1 Micro | 0.44905 |
| FNR Macro | 0.69046 |
| FNR Micro | 0.55095 |
| FPR Macro | 0.09951 |
| FPR Micro | 0.09183 |
| Gwet AC1 | 0.3673 |
| Hamming Loss | 0.55095 |
| Joint Entropy | 4.2067 |
| KL Divergence | None |
| Kappa | 0.30011 |
| Kappa 95% CI | (0.25867,0.34156) |
| Kappa No Prevalence | -0.1019 |
| Kappa Standard Error | 0.02114 |
| Kappa Unbiased | 0.2893 |
| Krippendorff Alpha | 0.2897 |
| Lambda A | 0.24846 |
| Lambda B | 0.25472 |
| Mutual Information | 0.31891 |
| NIR | 0.27436 |
| Overall ACC | 0.44905 |
| Overall CEN | 0.53948 |
| Overall J | (1.29419,0.18488) |
| Overall MCC | 0.31033 |
| Overall MCEN | 0.63217 |
| Overall RACC | 0.2128 |
| Overall RACCU | 0.22477 |
| P-Value | -0.0 |
| PPV Macro | None |
| PPV Micro | 0.44905 |
| Pearson C | None |
| Phi-Squared | None |
| RCI | 0.1223 |
| RR | 127.57143 |
| Reference Entropy | 2.60748 |
| Response Entropy | 1.91812 |
| SOA1(Landis & Koch) | Fair |
| SOA2(Fleiss) | Poor |
| SOA3(Altman) | Fair |
| SOA4(Cicchetti) | Poor |
| SOA5(Cramer) | None |
| SOA6(Matthews) | Weak |
| Scott PI | 0.2893 |
| Standard Error | 0.01664 |
| TNR Macro | 0.90049 |
| TNR Micro | 0.90817 |
| TPR Macro | 0.30954 |
| TPR Micro | 0.44905 |
| Zero-one Loss | 492 |

## Class Statistics :

|  |  |  |  |  |  |  |  |  |
| --- | --- | --- | --- | --- | --- | --- | --- | --- |
| Class | 0 | 1 | 2 | 3 | 4 | 5 | 6 | Description |
| ACC | 0.76932 | 0.72004 | 0.82419 | 0.82083 | 0.90817 | 0.8981 | 0.95745 | Accuracy |
| AGF | 0.70873 | 0.74737 | 0.67463 | 0.46064 | 0.20112 | 0.15655 | 0.0 | Adjusted F-score |
| AGM | 0.75069 | 0.72025 | 0.77679 | 0.66537 | 0.57364 | 0.54989 | 0 | Adjusted geometric mean |
| AM | 78 | 118 | -21 | 36 | -82 | -91 | -38 | Difference between automatic and manual classification |
| AUC | 0.71356 | 0.72333 | 0.70378 | 0.56601 | 0.51765 | 0.51075 | 0.5 | Area under the ROC curve |
| AUCI | Good | Good | Good | Poor | Poor | Poor | Poor | AUC value interpretation |
| AUPR | 0.52547 | 0.61186 | 0.53621 | 0.21474 | 0.51765 | 0.51075 | None | Area under the PR curve |
| BCD | 0.04367 | 0.06607 | 0.01176 | 0.02016 | 0.04591 | 0.05095 | 0.02128 | Bray-Curtis dissimilarity |
| BM | 0.42713 | 0.44666 | 0.40756 | 0.13202 | 0.03529 | 0.02151 | 0.0 | Informedness or bookmaker informedness |
| CEN | 0.55351 | 0.50046 | 0.52492 | 0.7264 | 0.53903 | 0.49261 | 0.2631 | Confusion entropy |
| DOR | 6.77663 | 6.83926 | 9.60674 | 2.46083 | None | None | None | Diagnostic odds ratio |
| DP | 0.45816 | 0.46036 | 0.54172 | 0.21561 | None | None | None | Discriminant power |
| DPI | Poor | Poor | Poor | Poor | None | None | None | Discriminant power interpretation |
| ERR | 0.23068 | 0.27996 | 0.17581 | 0.17917 | 0.09183 | 0.1019 | 0.04255 | Error rate |
| F0.5 | 0.45611 | 0.5274 | 0.55487 | 0.18784 | 0.15464 | 0.09901 | 0.0 | F0.5 score |
| F1 | 0.50718 | 0.58882 | 0.53412 | 0.20792 | 0.06818 | 0.04211 | 0.0 | F1 score - harmonic mean of precision and sensitivity |
| F2 | 0.57112 | 0.66642 | 0.51487 | 0.23282 | 0.04373 | 0.02674 | 0.0 | F2 score |
| FDR | 0.57258 | 0.50689 | 0.43038 | 0.82353 | 0.0 | 0.0 | None | False discovery rate |
| FN | 64 | 66 | 89 | 62 | 82 | 91 | 38 | False negative/miss/type 2 error |
| FNR | 0.37647 | 0.26939 | 0.49721 | 0.74699 | 0.96471 | 0.97849 | 1.0 | Miss rate or false negative rate |
| FOR | 0.09922 | 0.12453 | 0.12109 | 0.0801 | 0.09213 | 0.10213 | 0.04255 | False omission rate |
| FP | 142 | 184 | 68 | 98 | 0 | 0 | 0 | False positive/type 1 error/false alarm |
| FPR | 0.1964 | 0.28395 | 0.09524 | 0.12099 | 0.0 | 0.0 | 0.0 | Fall-out or false positive rate |
| G | 0.51624 | 0.60023 | 0.53516 | 0.2113 | 0.18787 | 0.14665 | None | G-measure geometric mean of precision and sensitivity |
| GI | 0.42713 | 0.44666 | 0.40756 | 0.13202 | 0.03529 | 0.02151 | 0.0 | Gini index |
| GM | 0.70786 | 0.72329 | 0.67447 | 0.47159 | 0.18787 | 0.14665 | 0.0 | G-mean geometric mean of specificity and sensitivity |
| IBA | 0.41084 | 0.53077 | 0.27205 | 0.08318 | 0.00125 | 0.00046 | 0.0 | Index of balanced accuracy |
| ICSI | 0.05095 | 0.22373 | 0.07241 | -0.57052 | 0.03529 | 0.02151 | None | Individual classification success index |
| IS | 1.16685 | 0.84587 | 1.50677 | 0.92498 | 3.39313 | 3.26336 | None | Information score |
| J | 0.33974 | 0.41725 | 0.36437 | 0.11602 | 0.03529 | 0.02151 | 0.0 | Jaccard index |
| LS | 2.24521 | 1.79735 | 2.84174 | 1.89865 | 10.50588 | 9.60215 | None | Lift score |
| MCC | 0.37441 | 0.40575 | 0.42755 | 0.1128 | 0.179 | 0.13896 | None | Matthews correlation coefficient |
| MCCI | Weak | Weak | Weak | Negligible | Negligible | Negligible | None | Matthews correlation coefficient interpretation |
| MCEN | 0.66385 | 0.6275 | 0.63671 | 0.77163 | 0.54459 | 0.49483 | 0.2631 | Modified confusion entropy |
| MK | 0.32819 | 0.36858 | 0.44853 | 0.09637 | 0.90787 | 0.89787 | None | Markedness |
| N | 723 | 648 | 714 | 810 | 808 | 800 | 855 | Condition negative |
| NLR | 0.46848 | 0.37621 | 0.54954 | 0.8498 | 0.96471 | 0.97849 | 1.0 | Negative likelihood ratio |
| NLRI | Poor | Poor | Negligible | Negligible | Negligible | Negligible | Negligible | Negative likelihood ratio interpretation |
| NPV | 0.90078 | 0.87547 | 0.87891 | 0.9199 | 0.90787 | 0.89787 | 0.95745 | Negative predictive value |
| OC | 0.62353 | 0.73061 | 0.56962 | 0.25301 | 1.0 | 1.0 | None | Overlap coefficient |
| OOC | 0.51624 | 0.60023 | 0.53516 | 0.2113 | 0.18787 | 0.14665 | None | Otsuka-Ochiai coefficient |
| OP | 0.64314 | 0.70998 | 0.53861 | 0.26784 | -0.02364 | -0.0598 | -0.04255 | Optimized precision |
| P | 170 | 245 | 179 | 83 | 85 | 93 | 38 | Condition positive or support |
| PLR | 3.17473 | 2.57303 | 5.27933 | 2.09122 | None | None | None | Positive likelihood ratio |
| PLRI | Poor | Poor | Fair | Poor | None | None | None | Positive likelihood ratio interpretation |
| POP | 893 | 893 | 893 | 893 | 893 | 893 | 893 | Population |
| PPV | 0.42742 | 0.49311 | 0.56962 | 0.17647 | 1.0 | 1.0 | None | Precision or positive predictive value |
| PRE | 0.19037 | 0.27436 | 0.20045 | 0.09295 | 0.09518 | 0.10414 | 0.04255 | Prevalence |
| Q | 0.74282 | 0.74487 | 0.81144 | 0.4221 | None | None | None | Yule Q - coefficient of colligation |
| QI | Moderate | Moderate | Strong | Weak | None | None | None | Yule Q interpretation |
| RACC | 0.05287 | 0.11152 | 0.03547 | 0.01239 | 0.00032 | 0.00023 | 0.0 | Random accuracy |
| RACCU | 0.05478 | 0.11589 | 0.0356 | 0.01279 | 0.00243 | 0.00283 | 0.00045 | Random accuracy unbiased |
| TN | 581 | 464 | 646 | 712 | 808 | 800 | 855 | True negative/correct rejection |
| TNR | 0.8036 | 0.71605 | 0.90476 | 0.87901 | 1.0 | 1.0 | 1.0 | Specificity or true negative rate |
| TON | 645 | 530 | 735 | 774 | 890 | 891 | 893 | Test outcome negative |
| TOP | 248 | 363 | 158 | 119 | 3 | 2 | 0 | Test outcome positive |
| TP | 106 | 179 | 90 | 21 | 3 | 2 | 0 | True positive/hit |
| TPR | 0.62353 | 0.73061 | 0.50279 | 0.25301 | 0.03529 | 0.02151 | 0.0 | Sensitivity, recall, hit rate, or true positive rate |
| Y | 0.42713 | 0.44666 | 0.40756 | 0.13202 | 0.03529 | 0.02151 | 0.0 | Youden index |
| dInd | 0.42462 | 0.3914 | 0.50625 | 0.75672 | 0.96471 | 0.97849 | 1.0 | Distance index |
| sInd | 0.69975 | 0.72324 | 0.64203 | 0.46492 | 0.31785 | 0.3081 | 0.29289 | Similarity index |

Generated By PyCM Version 3.3
