# Supplementary material for: TopEC: prediction of Enzyme Commission classes by 3D graph neural networks and localized 3D protein descriptor
Source: Nat Commun. 2025 Mar 20;16:2737. doi: 10.1038/s41467-025-57324-5 (PMC11923149; doi:10.1038/s41467-025-57324-5)
Supplement: Supplementary file 3 — Supplementary Data 1 [file 41467_2025_57324_MOESM3_ESM.zip › Data_S1/table1/mainclass/TopEC_distances/Combined_TEMP.html]

PyCM Report


# PyCM Report

## Dataset Type :

- Multi-Class Classification
- Imbalanced

Note 1 : Recommended statistics for this type of classification highlighted in aqua

Note 2 : The recommender system assumes that the input is the result of classification over the whole data rather than just a part of it.
If the confusion matrix is the result of test data classification, the recommendation is not valid.

## Confusion Matrix :

|  |  |  |  |  |  |  |  |  |  |  |  |  |  |  |  |  |  |  |  |  |  |  |  |  |  |  |  |  |  |  |  |  |  |  |  |  |  |  |  |  |  |  |  |  |  |  |  |  |  |  |  |  |  |  |  |  |  |  |  |  |  |  |  |  |  |
| --- | --- | --- | --- | --- | --- | --- | --- | --- | --- | --- | --- | --- | --- | --- | --- | --- | --- | --- | --- | --- | --- | --- | --- | --- | --- | --- | --- | --- | --- | --- | --- | --- | --- | --- | --- | --- | --- | --- | --- | --- | --- | --- | --- | --- | --- | --- | --- | --- | --- | --- | --- | --- | --- | --- | --- | --- | --- | --- | --- | --- | --- | --- | --- | --- | --- |
| Actual | Predict  |  |  |  |  |  |  |  |  | | --- | --- | --- | --- | --- | --- | --- | --- | |  | 0 | 1 | 2 | 3 | 4 | 5 | 6 | | 0 | 464 | 83 | 45 | 22 | 12 | 10 | 5 | | 1 | 67 | 767 | 100 | 26 | 6 | 10 | 11 | | 2 | 31 | 76 | 740 | 17 | 6 | 6 | 10 | | 3 | 22 | 27 | 34 | 95 | 14 | 4 | 2 | | 4 | 8 | 16 | 13 | 3 | 68 | 1 | 0 | | 5 | 8 | 22 | 8 | 0 | 2 | 49 | 0 | | 6 | 20 | 38 | 53 | 4 | 3 | 4 | 46 | |

## Overall Statistics :

|  |  |
| --- | --- |
| 95% CI | (0.70838,0.73996) |
| ACC Macro | 0.92119 |
| ARI | 0.45984 |
| AUNP | 0.81723 |
| AUNU | 0.77848 |
| Bangdiwala B | 0.58075 |
| Bennett S | 0.6782 |
| CBA | 0.59018 |
| CSI | 0.26995 |
| Chi-Squared | 6209.6037 |
| Chi-Squared DF | 36 |
| Conditional Entropy | 1.32847 |
| Cramer V | 0.57986 |
| Cross Entropy | 2.34206 |
| F1 Macro | 0.62427 |
| F1 Micro | 0.72417 |
| FNR Macro | 0.39083 |
| FNR Micro | 0.27583 |
| FPR Macro | 0.05222 |
| FPR Micro | 0.04597 |
| Gwet AC1 | 0.68474 |
| Hamming Loss | 0.27583 |
| Joint Entropy | 3.6453 |
| KL Divergence | 0.02523 |
| Kappa | 0.63278 |
| Kappa 95% CI | (0.61176,0.6538) |
| Kappa No Prevalence | 0.44834 |
| Kappa Standard Error | 0.01072 |
| Kappa Unbiased | 0.63247 |
| Krippendorff Alpha | 0.63253 |
| Lambda A | 0.59397 |
| Lambda B | 0.58907 |
| Mutual Information | 0.86422 |
| NIR | 0.32066 |
| Overall ACC | 0.72417 |
| Overall CEN | 0.37233 |
| Overall J | (3.27504,0.46786) |
| Overall MCC | 0.6339 |
| Overall MCEN | 0.5095 |
| Overall RACC | 0.24888 |
| Overall RACCU | 0.2495 |
| P-Value | None |
| PPV Macro | 0.66077 |
| PPV Micro | 0.72417 |
| Pearson C | 0.81767 |
| Phi-Squared | 2.01742 |
| RCI | 0.37302 |
| RR | 439.71429 |
| Reference Entropy | 2.31684 |
| Response Entropy | 2.19268 |
| SOA1(Landis & Koch) | Substantial |
| SOA2(Fleiss) | Intermediate to Good |
| SOA3(Altman) | Good |
| SOA4(Cicchetti) | Good |
| SOA5(Cramer) | Relatively Strong |
| SOA6(Matthews) | Moderate |
| Scott PI | 0.63247 |
| Standard Error | 0.00806 |
| TNR Macro | 0.94778 |
| TNR Micro | 0.95403 |
| TPR Macro | 0.60917 |
| TPR Micro | 0.72417 |
| Zero-one Loss | 849 |

## Class Statistics :

|  |  |  |  |  |  |  |  |  |
| --- | --- | --- | --- | --- | --- | --- | --- | --- |
| Class | 0 | 1 | 2 | 3 | 4 | 5 | 6 | Description |
| ACC | 0.89181 | 0.8434 | 0.87037 | 0.94314 | 0.97271 | 0.97563 | 0.95127 | Accuracy |
| AGF | 0.823 | 0.82765 | 0.86643 | 0.69196 | 0.78288 | 0.74132 | 0.54558 | Adjusted F-score |
| AGM | 0.873 | 0.84478 | 0.86996 | 0.82464 | 0.88299 | 0.86113 | 0.74897 | Adjusted geometric mean |
| AM | -21 | 42 | 107 | -31 | 2 | -5 | -94 | Difference between automatic and manual classification |
| AUC | 0.82993 | 0.8259 | 0.8599 | 0.7274 | 0.80469 | 0.76943 | 0.63209 | Area under the ROC curve |
| AUCI | Very Good | Very Good | Very Good | Good | Very Good | Good | Fair | AUC value interpretation |
| AUPR | 0.73613 | 0.76124 | 0.79022 | 0.52433 | 0.61823 | 0.56695 | 0.44772 | Area under the PR curve |
| BCD | 0.00341 | 0.00682 | 0.01738 | 0.00504 | 0.00032 | 0.00081 | 0.01527 | Bray-Curtis dissimilarity |
| BM | 0.65986 | 0.6518 | 0.71979 | 0.4548 | 0.60937 | 0.53885 | 0.26419 | Informedness or bookmaker informedness |
| CEN | 0.37058 | 0.34831 | 0.31429 | 0.55348 | 0.48013 | 0.50233 | 0.56797 | Confusion entropy |
| DOR | 38.33058 | 24.33801 | 38.84509 | 35.97087 | 112.85763 | 103.39 | 38.80913 | Diagnostic odds ratio |
| DP | 0.87305 | 0.7643 | 0.87625 | 0.85784 | 1.13162 | 1.11064 | 0.87603 | Discriminant power |
| DPI | Poor | Poor | Poor | Poor | Limited | Limited | Poor | Discriminant power interpretation |
| ERR | 0.10819 | 0.1566 | 0.12963 | 0.05686 | 0.02729 | 0.02437 | 0.04873 | Error rate |
| F0.5 | 0.74335 | 0.75152 | 0.76163 | 0.5485 | 0.61483 | 0.57647 | 0.49569 | F0.5 score |
| F1 | 0.73592 | 0.76091 | 0.78765 | 0.52055 | 0.61818 | 0.56647 | 0.38017 | F1 score - harmonic mean of precision and sensitivity |
| F2 | 0.72864 | 0.77054 | 0.81552 | 0.49531 | 0.62157 | 0.55682 | 0.30831 | F2 score |
| FDR | 0.25161 | 0.25462 | 0.25478 | 0.43114 | 0.38739 | 0.41667 | 0.37838 | False discovery rate |
| FN | 177 | 220 | 146 | 103 | 41 | 40 | 122 | False negative/miss/type 2 error |
| FNR | 0.27613 | 0.2229 | 0.16479 | 0.5202 | 0.37615 | 0.44944 | 0.72619 | Miss rate or false negative rate |
| FOR | 0.07201 | 0.10737 | 0.07002 | 0.03538 | 0.01382 | 0.01336 | 0.04061 | False omission rate |
| FP | 156 | 262 | 253 | 72 | 43 | 35 | 28 | False positive/type 1 error/false alarm |
| FPR | 0.06401 | 0.1253 | 0.11542 | 0.025 | 0.01448 | 0.01171 | 0.00962 | Fall-out or false positive rate |
| G | 0.73603 | 0.76108 | 0.78893 | 0.52244 | 0.61821 | 0.56671 | 0.41256 | G-measure geometric mean of precision and sensitivity |
| GI | 0.65986 | 0.6518 | 0.71979 | 0.4548 | 0.60937 | 0.53885 | 0.26419 | Gini index |
| GM | 0.82312 | 0.82446 | 0.85954 | 0.68396 | 0.7841 | 0.73764 | 0.52074 | G-mean geometric mean of specificity and sensitivity |
| IBA | 0.53382 | 0.61339 | 0.70234 | 0.23615 | 0.39246 | 0.30594 | 0.07686 | Index of balanced accuracy |
| ICSI | 0.47226 | 0.52249 | 0.58043 | 0.04866 | 0.23647 | 0.1339 | -0.10457 | Individual classification success index |
| IS | 1.84545 | 1.21693 | 1.37235 | 3.14457 | 4.11264 | 4.33444 | 3.50957 | Information score |
| J | 0.58218 | 0.61409 | 0.64969 | 0.35185 | 0.44737 | 0.39516 | 0.23469 | Jaccard index |
| LS | 3.59366 | 2.32451 | 2.58891 | 8.84322 | 17.29928 | 20.17416 | 11.389 | Lift score |
| MCC | 0.66807 | 0.64487 | 0.69714 | 0.49257 | 0.60406 | 0.55419 | 0.39178 | Matthews correlation coefficient |
| MCCI | Moderate | Moderate | Moderate | Weak | Moderate | Moderate | Weak | Matthews correlation coefficient interpretation |
| MCEN | 0.50918 | 0.48786 | 0.44791 | 0.66958 | 0.6127 | 0.61977 | 0.63634 | Modified confusion entropy |
| MK | 0.67638 | 0.63801 | 0.67519 | 0.53348 | 0.59879 | 0.56997 | 0.58101 | Markedness |
| N | 2437 | 2091 | 2192 | 2880 | 2969 | 2989 | 2910 | Condition negative |
| NLR | 0.29502 | 0.25483 | 0.18629 | 0.53354 | 0.38167 | 0.45476 | 0.73325 | Negative likelihood ratio |
| NLRI | Poor | Poor | Fair | Negligible | Poor | Poor | Negligible | Negative likelihood ratio interpretation |
| NPV | 0.92799 | 0.89263 | 0.92998 | 0.96462 | 0.98618 | 0.98664 | 0.95939 | Negative predictive value |
| OC | 0.74839 | 0.7771 | 0.83521 | 0.56886 | 0.62385 | 0.58333 | 0.62162 | Overlap coefficient |
| OOC | 0.73603 | 0.76108 | 0.78893 | 0.52244 | 0.61821 | 0.56671 | 0.41256 | Otsuka-Ochiai coefficient |
| OP | 0.76402 | 0.78432 | 0.84167 | 0.60275 | 0.74799 | 0.69118 | 0.38445 | Optimized precision |
| P | 641 | 987 | 886 | 198 | 109 | 89 | 168 | Condition positive or support |
| PLR | 11.30813 | 6.20199 | 7.23632 | 19.19192 | 43.07489 | 47.01798 | 28.45663 | Positive likelihood ratio |
| PLRI | Good | Fair | Fair | Good | Good | Good | Good | Positive likelihood ratio interpretation |
| POP | 3078 | 3078 | 3078 | 3078 | 3078 | 3078 | 3078 | Population |
| PPV | 0.74839 | 0.74538 | 0.74522 | 0.56886 | 0.61261 | 0.58333 | 0.62162 | Precision or positive predictive value |
| PRE | 0.20825 | 0.32066 | 0.28785 | 0.06433 | 0.03541 | 0.02891 | 0.05458 | Prevalence |
| Q | 0.94915 | 0.92107 | 0.94981 | 0.9459 | 0.98243 | 0.98084 | 0.94976 | Yule Q - coefficient of colligation |
| QI | Strong | Strong | Strong | Strong | Strong | Strong | Strong | Yule Q interpretation |
| RACC | 0.04195 | 0.1072 | 0.09286 | 0.00349 | 0.00128 | 0.00079 | 0.00131 | Random accuracy |
| RACCU | 0.04196 | 0.10725 | 0.09317 | 0.00352 | 0.00128 | 0.00079 | 0.00155 | Random accuracy unbiased |
| TN | 2281 | 1829 | 1939 | 2808 | 2926 | 2954 | 2882 | True negative/correct rejection |
| TNR | 0.93599 | 0.8747 | 0.88458 | 0.975 | 0.98552 | 0.98829 | 0.99038 | Specificity or true negative rate |
| TON | 2458 | 2049 | 2085 | 2911 | 2967 | 2994 | 3004 | Test outcome negative |
| TOP | 620 | 1029 | 993 | 167 | 111 | 84 | 74 | Test outcome positive |
| TP | 464 | 767 | 740 | 95 | 68 | 49 | 46 | True positive/hit |
| TPR | 0.72387 | 0.7771 | 0.83521 | 0.4798 | 0.62385 | 0.55056 | 0.27381 | Sensitivity, recall, hit rate, or true positive rate |
| Y | 0.65986 | 0.6518 | 0.71979 | 0.4548 | 0.60937 | 0.53885 | 0.26419 | Youden index |
| dInd | 0.28345 | 0.2557 | 0.20119 | 0.5208 | 0.37643 | 0.44959 | 0.72625 | Distance index |
| sInd | 0.79957 | 0.81919 | 0.85774 | 0.63174 | 0.73383 | 0.68209 | 0.48646 | Similarity index |

Generated By PyCM Version 3.3
