# Supplementary material for: TopEC: prediction of Enzyme Commission classes by 3D graph neural networks and localized 3D protein descriptor
Source: Nat Commun. 2025 Mar 20;16:2737. doi: 10.1038/s41467-025-57324-5 (PMC11923149; doi:10.1038/s41467-025-57324-5)
Supplement: Supplementary file 3 — Supplementary Data 1 [file 41467_2025_57324_MOESM3_ESM.zip › Data_S1/table1/mainclass/TopEC_distances/Combined_FOLD.html]

PyCM Report


# PyCM Report

## Dataset Type :

- Multi-Class Classification
- Imbalanced

Note 1 : Recommended statistics for this type of classification highlighted in aqua

Note 2 : The recommender system assumes that the input is the result of classification over the whole data rather than just a part of it.
If the confusion matrix is the result of test data classification, the recommendation is not valid.

## Confusion Matrix :

|  |  |  |  |  |  |  |  |  |  |  |  |  |  |  |  |  |  |  |  |  |  |  |  |  |  |  |  |  |  |  |  |  |  |  |  |  |  |  |  |  |  |  |  |  |  |  |  |  |  |  |  |  |  |  |  |  |  |  |  |  |  |  |  |  |  |
| --- | --- | --- | --- | --- | --- | --- | --- | --- | --- | --- | --- | --- | --- | --- | --- | --- | --- | --- | --- | --- | --- | --- | --- | --- | --- | --- | --- | --- | --- | --- | --- | --- | --- | --- | --- | --- | --- | --- | --- | --- | --- | --- | --- | --- | --- | --- | --- | --- | --- | --- | --- | --- | --- | --- | --- | --- | --- | --- | --- | --- | --- | --- | --- | --- | --- |
| Actual | Predict  |  |  |  |  |  |  |  |  | | --- | --- | --- | --- | --- | --- | --- | --- | |  | 0 | 1 | 2 | 3 | 4 | 5 | 6 | | 0 | 384 | 99 | 47 | 46 | 0 | 0 | 1 | | 1 | 93 | 855 | 55 | 47 | 0 | 1 | 1 | | 2 | 74 | 42 | 437 | 26 | 0 | 0 | 3 | | 3 | 81 | 77 | 34 | 22 | 1 | 0 | 0 | | 4 | 101 | 54 | 68 | 32 | 0 | 1 | 0 | | 5 | 43 | 51 | 18 | 23 | 0 | 6 | 0 | | 6 | 7 | 29 | 17 | 2 | 0 | 0 | 0 | |

## Overall Statistics :

|  |  |
| --- | --- |
| 95% CI | (0.57412,0.61003) |
| ACC Macro | 0.88345 |
| ARI | 0.33462 |
| AUNP | 0.73038 |
| AUNU | 0.63105 |
| Bangdiwala B | 0.49554 |
| Bennett S | 0.52409 |
| CBA | 0.2843 |
| CSI | -0.27424 |
| Chi-Squared | 2236.27243 |
| Chi-Squared DF | 36 |
| Conditional Entropy | 1.33255 |
| Cramer V | 0.35987 |
| Cross Entropy | 3.1592 |
| F1 Macro | 0.31479 |
| F1 Micro | 0.59208 |
| FNR Macro | 0.66086 |
| FNR Micro | 0.40792 |
| FPR Macro | 0.07703 |
| FPR Micro | 0.06799 |
| Gwet AC1 | 0.53518 |
| Hamming Loss | 0.40792 |
| Joint Entropy | 3.70681 |
| KL Divergence | 0.78494 |
| Kappa | 0.44824 |
| Kappa 95% CI | (0.42396,0.47253) |
| Kappa No Prevalence | 0.18416 |
| Kappa Standard Error | 0.01239 |
| Kappa Unbiased | 0.4446 |
| Krippendorff Alpha | 0.44469 |
| Lambda A | 0.37295 |
| Lambda B | 0.43746 |
| Mutual Information | 0.50423 |
| NIR | 0.36553 |
| Overall ACC | 0.59208 |
| Overall CEN | 0.44475 |
| Overall J | (1.63292,0.23327) |
| Overall MCC | 0.45487 |
| Overall MCEN | 0.5675 |
| Overall RACC | 0.26068 |
| Overall RACCU | 0.26554 |
| P-Value | None |
| PPV Macro | 0.38662 |
| PPV Micro | 0.59208 |
| Pearson C | 0.66126 |
| Phi-Squared | 0.77702 |
| RCI | 0.21237 |
| RR | 411.14286 |
| Reference Entropy | 2.37426 |
| Response Entropy | 1.83678 |
| SOA1(Landis & Koch) | Moderate |
| SOA2(Fleiss) | Intermediate to Good |
| SOA3(Altman) | Moderate |
| SOA4(Cicchetti) | Fair |
| SOA5(Cramer) | Moderate |
| SOA6(Matthews) | Weak |
| Scott PI | 0.4446 |
| Standard Error | 0.00916 |
| TNR Macro | 0.92297 |
| TNR Micro | 0.93201 |
| TPR Macro | 0.33914 |
| TPR Micro | 0.59208 |
| Zero-one Loss | 1174 |

## Class Statistics :

|  |  |  |  |  |  |  |  |  |
| --- | --- | --- | --- | --- | --- | --- | --- | --- |
| Class | 0 | 1 | 2 | 3 | 4 | 5 | 6 | Description |
| ACC | 0.7943 | 0.80924 | 0.86657 | 0.87179 | 0.9107 | 0.9524 | 0.97915 | Accuracy |
| AGF | 0.74368 | 0.82687 | 0.8208 | 0.31081 | 0.0 | 0.22461 | 0.0 | Adjusted F-score |
| AGM | 0.77942 | 0.80891 | 0.85379 | 0.6094 | 0 | 0.59278 | 0 | Adjusted geometric mean |
| AM | 206 | 155 | 94 | -17 | -255 | -133 | -50 | Difference between automatic and manual classification |
| AUC | 0.74605 | 0.80998 | 0.82338 | 0.51812 | 0.49981 | 0.52091 | 0.49911 | Area under the ROC curve |
| AUCI | Good | Very Good | Very Good | Poor | Poor | Poor | Poor | AUC value interpretation |
| AUPR | 0.57797 | 0.76055 | 0.69865 | 0.10672 | 0.0 | 0.39628 | 0.0 | Area under the PR curve |
| BCD | 0.03579 | 0.02693 | 0.01633 | 0.00295 | 0.0443 | 0.02311 | 0.00869 | Bray-Curtis dissimilarity |
| BM | 0.49211 | 0.61997 | 0.64677 | 0.03623 | -0.00038 | 0.04182 | -0.00177 | Informedness or bookmaker informedness |
| CEN | 0.50954 | 0.34861 | 0.4059 | 0.76602 | 0.54297 | 0.53786 | 0.54691 | Confusion entropy |
| DOR | 9.48444 | 18.17418 | 25.9388 | 1.61075 | 0.0 | 60.77778 | 0.0 | Diagnostic odds ratio |
| DP | 0.53865 | 0.69437 | 0.77955 | 0.11414 | None | 0.98343 | None | Discriminant power |
| DPI | Poor | Poor | Poor | Poor | None | Poor | None | Discriminant power interpretation |
| ERR | 0.2057 | 0.19076 | 0.13343 | 0.12821 | 0.0893 | 0.0476 | 0.02085 | Error rate |
| F0.5 | 0.51766 | 0.72704 | 0.66494 | 0.10924 | 0.0 | 0.17341 | 0.0 | F0.5 score |
| F1 | 0.56471 | 0.75697 | 0.69475 | 0.10654 | 0.0 | 0.08054 | 0.0 | F1 score - harmonic mean of precision and sensitivity |
| F2 | 0.62116 | 0.78947 | 0.72736 | 0.10397 | 0.0 | 0.05245 | 0.0 | F2 score |
| FDR | 0.50958 | 0.29163 | 0.35355 | 0.88889 | 1.0 | 0.25 | 1.0 | False discovery rate |
| FN | 193 | 197 | 145 | 193 | 256 | 135 | 55 | False negative/miss/type 2 error |
| FNR | 0.33449 | 0.18726 | 0.24914 | 0.89767 | 1.0 | 0.95745 | 1.0 | Miss rate or false negative rate |
| FOR | 0.09212 | 0.11789 | 0.06585 | 0.07201 | 0.08898 | 0.04704 | 0.01914 | False omission rate |
| FP | 399 | 352 | 239 | 176 | 1 | 2 | 5 | False positive/type 1 error/false alarm |
| FPR | 0.1734 | 0.19277 | 0.10409 | 0.06609 | 0.00038 | 0.00073 | 0.00177 | Fall-out or false positive rate |
| G | 0.5713 | 0.75876 | 0.6967 | 0.10663 | 0.0 | 0.17865 | 0.0 | G-measure geometric mean of precision and sensitivity |
| GI | 0.49211 | 0.61997 | 0.64677 | 0.03623 | -0.00038 | 0.04182 | -0.00177 | Gini index |
| GM | 0.74169 | 0.80998 | 0.82018 | 0.30913 | 0.0 | 0.20621 | 0.0 | G-mean geometric mean of specificity and sensitivity |
| IBA | 0.46149 | 0.65968 | 0.57513 | 0.01609 | 0.0 | 0.00184 | 0.0 | Index of balanced accuracy |
| ICSI | 0.15593 | 0.52111 | 0.39731 | -0.78656 | -1.0 | -0.20745 | -1.0 | Individual classification success index |
| IS | 1.29052 | 0.9545 | 1.67659 | 0.57273 | None | 3.93626 | None | Information score |
| J | 0.39344 | 0.60897 | 0.53228 | 0.05627 | 0.0 | 0.04196 | 0.0 | Jaccard index |
| LS | 2.44616 | 1.93791 | 3.1967 | 1.48734 | 0.0 | 15.30851 | 0.0 | Lift score |
| MCC | 0.44273 | 0.60504 | 0.61279 | 0.03764 | -0.00583 | 0.17146 | -0.00582 | Matthews correlation coefficient |
| MCCI | Weak | Moderate | Moderate | Negligible | Negligible | Negligible | Negligible | Matthews correlation coefficient interpretation |
| MCEN | 0.62903 | 0.48607 | 0.54163 | 0.78833 | 0.54297 | 0.54458 | 0.54691 | Modified confusion entropy |
| MK | 0.3983 | 0.59047 | 0.5806 | 0.0391 | -0.08898 | 0.70296 | -0.01914 | Markedness |
| N | 2301 | 1826 | 2296 | 2663 | 2622 | 2737 | 2823 | Condition negative |
| NLR | 0.40466 | 0.23198 | 0.27809 | 0.9612 | 1.00038 | 0.95815 | 1.00177 | Negative likelihood ratio |
| NLRI | Poor | Poor | Poor | Negligible | Negligible | Negligible | Negligible | Negative likelihood ratio interpretation |
| NPV | 0.90788 | 0.88211 | 0.93415 | 0.92799 | 0.91102 | 0.95296 | 0.98086 | Negative predictive value |
| OC | 0.66551 | 0.81274 | 0.75086 | 0.11111 | 0.0 | 0.75 | 0.0 | Overlap coefficient |
| OOC | 0.5713 | 0.75876 | 0.6967 | 0.10663 | 0.0 | 0.17865 | 0.0 | Otsuka-Ochiai coefficient |
| OP | 0.68634 | 0.80584 | 0.77849 | 0.06928 | -0.0893 | 0.03409 | -0.02085 | Optimized precision |
| P | 577 | 1052 | 582 | 215 | 256 | 141 | 55 | Condition positive or support |
| PLR | 3.83795 | 4.21608 | 7.21327 | 1.54826 | 0.0 | 58.23404 | 0.0 | Positive likelihood ratio |
| PLRI | Poor | Poor | Fair | Poor | Negligible | Good | Negligible | Positive likelihood ratio interpretation |
| POP | 2878 | 2878 | 2878 | 2878 | 2878 | 2878 | 2878 | Population |
| PPV | 0.49042 | 0.70837 | 0.64645 | 0.11111 | 0.0 | 0.75 | 0.0 | Precision or positive predictive value |
| PRE | 0.20049 | 0.36553 | 0.20222 | 0.0747 | 0.08895 | 0.04899 | 0.01911 | Prevalence |
| Q | 0.80924 | 0.89569 | 0.92576 | 0.23394 | -1.0 | 0.96763 | -1.0 | Yule Q - coefficient of colligation |
| QI | Strong | Strong | Strong | Negligible | Negligible | Strong | Negligible | Yule Q interpretation |
| RACC | 0.05455 | 0.1533 | 0.0475 | 0.00514 | 3e-05 | 0.00014 | 3e-05 | Random accuracy |
| RACCU | 0.05583 | 0.15402 | 0.04777 | 0.00515 | 0.00199 | 0.00067 | 0.00011 | Random accuracy unbiased |
| TN | 1902 | 1474 | 2057 | 2487 | 2621 | 2735 | 2818 | True negative/correct rejection |
| TNR | 0.8266 | 0.80723 | 0.89591 | 0.93391 | 0.99962 | 0.99927 | 0.99823 | Specificity or true negative rate |
| TON | 2095 | 1671 | 2202 | 2680 | 2877 | 2870 | 2873 | Test outcome negative |
| TOP | 783 | 1207 | 676 | 198 | 1 | 8 | 5 | Test outcome positive |
| TP | 384 | 855 | 437 | 22 | 0 | 6 | 0 | True positive/hit |
| TPR | 0.66551 | 0.81274 | 0.75086 | 0.10233 | 0.0 | 0.04255 | 0.0 | Sensitivity, recall, hit rate, or true positive rate |
| Y | 0.49211 | 0.61997 | 0.64677 | 0.03623 | -0.00038 | 0.04182 | -0.00177 | Youden index |
| dInd | 0.37676 | 0.26875 | 0.27001 | 0.9001 | 1.0 | 0.95745 | 1.0 | Distance index |
| sInd | 0.73359 | 0.80996 | 0.80907 | 0.36353 | 0.29289 | 0.32298 | 0.29289 | Similarity index |

Generated By PyCM Version 3.3
