# Supplementary material for: TopEC: prediction of Enzyme Commission classes by 3D graph neural networks and localized 3D protein descriptor
Source: Nat Commun. 2025 Mar 20;16:2737. doi: 10.1038/s41467-025-57324-5 (PMC11923149; doi:10.1038/s41467-025-57324-5)
Supplement: Supplementary file 3 — Supplementary Data 1 [file 41467_2025_57324_MOESM3_ESM.zip › Data_S1/table1/mainclass/TopEC_distances/BindingMOAD_TEMP.html]

PyCM Report


# PyCM Report

## Dataset Type :

- Multi-Class Classification
- Imbalanced

Note 1 : Recommended statistics for this type of classification highlighted in aqua

Note 2 : The recommender system assumes that the input is the result of classification over the whole data rather than just a part of it.
If the confusion matrix is the result of test data classification, the recommendation is not valid.

## Confusion Matrix :

|  |  |  |  |  |  |  |  |  |  |  |  |  |  |  |  |  |  |  |  |  |  |  |  |  |  |  |  |  |  |  |  |  |  |  |  |  |  |  |  |  |  |  |  |  |  |  |  |  |  |  |  |  |  |  |  |  |  |  |  |  |  |  |  |  |  |
| --- | --- | --- | --- | --- | --- | --- | --- | --- | --- | --- | --- | --- | --- | --- | --- | --- | --- | --- | --- | --- | --- | --- | --- | --- | --- | --- | --- | --- | --- | --- | --- | --- | --- | --- | --- | --- | --- | --- | --- | --- | --- | --- | --- | --- | --- | --- | --- | --- | --- | --- | --- | --- | --- | --- | --- | --- | --- | --- | --- | --- | --- | --- | --- | --- | --- |
| Actual | Predict  |  |  |  |  |  |  |  |  | | --- | --- | --- | --- | --- | --- | --- | --- | |  | 0 | 1 | 2 | 3 | 4 | 5 | 6 | | 0 | 335 | 56 | 21 | 3 | 0 | 7 | 1 | | 1 | 28 | 629 | 75 | 4 | 3 | 11 | 4 | | 2 | 23 | 51 | 578 | 0 | 5 | 12 | 1 | | 3 | 16 | 17 | 17 | 69 | 1 | 5 | 0 | | 4 | 9 | 4 | 7 | 3 | 54 | 0 | 0 | | 5 | 1 | 19 | 2 | 0 | 1 | 32 | 3 | | 6 | 10 | 16 | 31 | 0 | 3 | 10 | 6 | |

## Overall Statistics :

|  |  |
| --- | --- |
| 95% CI | (0.76274,0.79749) |
| ACC Macro | 0.93718 |
| ARI | 0.54873 |
| AUNP | 0.84945 |
| AUNU | 0.79084 |
| Bangdiwala B | 0.66146 |
| Bennett S | 0.74347 |
| CBA | 0.58924 |
| CSI | 0.32093 |
| Chi-Squared | 5243.55295 |
| Chi-Squared DF | 36 |
| Conditional Entropy | 1.04654 |
| Cramer V | 0.63272 |
| Cross Entropy | 2.2789 |
| F1 Macro | 0.63774 |
| F1 Micro | 0.78012 |
| FNR Macro | 0.37531 |
| FNR Micro | 0.21988 |
| FPR Macro | 0.04301 |
| FPR Micro | 0.03665 |
| Gwet AC1 | 0.74968 |
| Hamming Loss | 0.21988 |
| Joint Entropy | 3.27223 |
| KL Divergence | 0.0532 |
| Kappa | 0.69885 |
| Kappa 95% CI | (0.67505,0.72264) |
| Kappa No Prevalence | 0.56024 |
| Kappa Standard Error | 0.01214 |
| Kappa Unbiased | 0.6986 |
| Krippendorff Alpha | 0.69867 |
| Lambda A | 0.6641 |
| Lambda B | 0.6729 |
| Mutual Information | 1.01814 |
| NIR | 0.3454 |
| Overall ACC | 0.78012 |
| Overall CEN | 0.29966 |
| Overall J | (3.53748,0.50535) |
| Overall MCC | 0.70012 |
| Overall MCEN | 0.42511 |
| Overall RACC | 0.26987 |
| Overall RACCU | 0.27048 |
| P-Value | None |
| PPV Macro | 0.69624 |
| PPV Micro | 0.78012 |
| Pearson C | 0.84027 |
| Phi-Squared | 2.40199 |
| RCI | 0.45745 |
| RR | 311.85714 |
| Reference Entropy | 2.22569 |
| Response Entropy | 2.06467 |
| SOA1(Landis & Koch) | Substantial |
| SOA2(Fleiss) | Intermediate to Good |
| SOA3(Altman) | Good |
| SOA4(Cicchetti) | Good |
| SOA5(Cramer) | Strong |
| SOA6(Matthews) | Strong |
| Scott PI | 0.6986 |
| Standard Error | 0.00886 |
| TNR Macro | 0.95699 |
| TNR Micro | 0.96335 |
| TPR Macro | 0.62469 |
| TPR Micro | 0.78012 |
| Zero-one Loss | 480 |

## Class Statistics :

|  |  |  |  |  |  |  |  |  |
| --- | --- | --- | --- | --- | --- | --- | --- | --- |
| Class | 0 | 1 | 2 | 3 | 4 | 5 | 6 | Description |
| ACC | 0.91984 | 0.86807 | 0.88777 | 0.96977 | 0.98351 | 0.96748 | 0.96381 | Accuracy |
| AGF | 0.86766 | 0.86463 | 0.88711 | 0.76324 | 0.8443 | 0.71448 | 0.30253 | Adjusted F-score |
| AGM | 0.90466 | 0.87007 | 0.88808 | 0.86441 | 0.91291 | 0.85521 | 0.63172 | Adjusted geometric mean |
| AM | -1 | 38 | 61 | -46 | -10 | 19 | -61 | Difference between automatic and manual classification |
| AUC | 0.87127 | 0.86008 | 0.88078 | 0.77357 | 0.84756 | 0.76527 | 0.53734 | Area under the ROC curve |
| AUCI | Very Good | Very Good | Very Good | Good | Very Good | Good | Poor | AUC value interpretation |
| AUPR | 0.7929 | 0.8142 | 0.82669 | 0.71271 | 0.75363 | 0.48365 | 0.23947 | Area under the PR curve |
| BCD | 0.00023 | 0.0087 | 0.01397 | 0.01054 | 0.00229 | 0.00435 | 0.01397 | Bray-Curtis dissimilarity |
| BM | 0.74253 | 0.72015 | 0.76156 | 0.54714 | 0.69513 | 0.53055 | 0.07468 | Informedness or bookmaker informedness |
| CEN | 0.29553 | 0.28037 | 0.26057 | 0.37505 | 0.34105 | 0.55672 | 0.65165 | Confusion entropy |
| DOR | 73.20468 | 39.0829 | 55.84541 | 252.34286 | 378.0 | 56.88889 | 19.98095 | Diagnostic odds ratio |
| DP | 1.02797 | 0.87771 | 0.96316 | 1.32429 | 1.42105 | 0.9676 | 0.71707 | Discriminant power |
| DPI | Limited | Poor | Poor | Limited | Limited | Poor | Poor | Discriminant power interpretation |
| ERR | 0.08016 | 0.13193 | 0.11223 | 0.03023 | 0.01649 | 0.03252 | 0.03619 | Error rate |
| F0.5 | 0.79346 | 0.80189 | 0.80412 | 0.78231 | 0.78261 | 0.43716 | 0.22059 | F0.5 score |
| F1 | 0.7929 | 0.81371 | 0.82512 | 0.67647 | 0.75 | 0.47407 | 0.13187 | F1 score - harmonic mean of precision and sensitivity |
| F2 | 0.79234 | 0.82589 | 0.84726 | 0.59585 | 0.72 | 0.5178 | 0.09404 | F2 score |
| FDR | 0.20616 | 0.20581 | 0.2093 | 0.12658 | 0.19403 | 0.58442 | 0.6 | False discovery rate |
| FN | 88 | 125 | 92 | 56 | 23 | 26 | 70 | False negative/miss/type 2 error |
| FNR | 0.20804 | 0.16578 | 0.13731 | 0.448 | 0.2987 | 0.44828 | 0.92105 | Miss rate or false negative rate |
| FOR | 0.04997 | 0.08986 | 0.06336 | 0.02662 | 0.01087 | 0.01235 | 0.03229 | False omission rate |
| FP | 87 | 163 | 153 | 10 | 13 | 45 | 9 | False positive/type 1 error/false alarm |
| FPR | 0.04943 | 0.11407 | 0.10112 | 0.00486 | 0.00617 | 0.02118 | 0.00427 | Fall-out or false positive rate |
| G | 0.7929 | 0.81396 | 0.82591 | 0.69435 | 0.75182 | 0.47884 | 0.1777 | G-measure geometric mean of precision and sensitivity |
| GI | 0.74253 | 0.72015 | 0.76156 | 0.54714 | 0.69513 | 0.53055 | 0.07468 | Gini index |
| GM | 0.86765 | 0.85969 | 0.8806 | 0.74116 | 0.83485 | 0.73487 | 0.28038 | G-mean geometric mean of specificity and sensitivity |
| IBA | 0.63341 | 0.70084 | 0.74739 | 0.30589 | 0.49309 | 0.30939 | 0.00654 | Index of balanced accuracy |
| ICSI | 0.5858 | 0.62841 | 0.65338 | 0.42542 | 0.50727 | -0.03269 | -0.52105 | Individual classification success index |
| IS | 2.0345 | 1.20124 | 1.36528 | 3.93106 | 4.51411 | 3.96733 | 3.52224 | Information score |
| J | 0.65686 | 0.68593 | 0.70231 | 0.51111 | 0.6 | 0.31068 | 0.07059 | Jaccard index |
| LS | 4.09681 | 2.29936 | 2.57626 | 15.25337 | 22.84978 | 15.64174 | 11.48947 | Lift score |
| MCC | 0.7432 | 0.7122 | 0.74425 | 0.68068 | 0.74343 | 0.46253 | 0.16571 | Matthews correlation coefficient |
| MCCI | Strong | Strong | Strong | Moderate | Strong | Weak | Negligible | Matthews correlation coefficient interpretation |
| MCEN | 0.41992 | 0.40666 | 0.37984 | 0.48552 | 0.47002 | 0.65463 | 0.67213 | Modified confusion entropy |
| MK | 0.74387 | 0.70433 | 0.72734 | 0.8468 | 0.7951 | 0.40324 | 0.36771 | Markedness |
| N | 1760 | 1429 | 1513 | 2058 | 2106 | 2125 | 2107 | Condition negative |
| NLR | 0.21886 | 0.18713 | 0.15276 | 0.45019 | 0.30056 | 0.45797 | 0.925 | Negative likelihood ratio |
| NLRI | Poor | Fair | Fair | Poor | Poor | Poor | Negligible | Negative likelihood ratio interpretation |
| NPV | 0.95003 | 0.91014 | 0.93664 | 0.97338 | 0.98913 | 0.98765 | 0.96771 | Negative predictive value |
| OC | 0.79384 | 0.83422 | 0.86269 | 0.87342 | 0.80597 | 0.55172 | 0.4 | Overlap coefficient |
| OOC | 0.7929 | 0.81396 | 0.82591 | 0.69435 | 0.75182 | 0.47884 | 0.1777 | Otsuka-Ochiai coefficient |
| OP | 0.82881 | 0.83801 | 0.86722 | 0.68334 | 0.81094 | 0.68843 | 0.11073 | Optimized precision |
| P | 423 | 754 | 670 | 125 | 77 | 58 | 76 | Condition positive or support |
| PLR | 16.0213 | 7.31348 | 8.53101 | 113.6016 | 113.61039 | 26.05364 | 18.48246 | Positive likelihood ratio |
| PLRI | Good | Fair | Fair | Good | Good | Good | Good | Positive likelihood ratio interpretation |
| POP | 2183 | 2183 | 2183 | 2183 | 2183 | 2183 | 2183 | Population |
| PPV | 0.79384 | 0.79419 | 0.7907 | 0.87342 | 0.80597 | 0.41558 | 0.4 | Precision or positive predictive value |
| PRE | 0.19377 | 0.3454 | 0.30692 | 0.05726 | 0.03527 | 0.02657 | 0.03481 | Prevalence |
| Q | 0.97305 | 0.9501 | 0.96482 | 0.99211 | 0.99472 | 0.96545 | 0.90468 | Yule Q - coefficient of colligation |
| QI | Strong | Strong | Strong | Strong | Strong | Strong | Strong | Yule Q interpretation |
| RACC | 0.03746 | 0.12531 | 0.10277 | 0.00207 | 0.00108 | 0.00094 | 0.00024 | Random accuracy |
| RACCU | 0.03746 | 0.12539 | 0.10297 | 0.00218 | 0.00109 | 0.00096 | 0.00043 | Random accuracy unbiased |
| TN | 1673 | 1266 | 1360 | 2048 | 2093 | 2080 | 2098 | True negative/correct rejection |
| TNR | 0.95057 | 0.88593 | 0.89888 | 0.99514 | 0.99383 | 0.97882 | 0.99573 | Specificity or true negative rate |
| TON | 1761 | 1391 | 1452 | 2104 | 2116 | 2106 | 2168 | Test outcome negative |
| TOP | 422 | 792 | 731 | 79 | 67 | 77 | 15 | Test outcome positive |
| TP | 335 | 629 | 578 | 69 | 54 | 32 | 6 | True positive/hit |
| TPR | 0.79196 | 0.83422 | 0.86269 | 0.552 | 0.7013 | 0.55172 | 0.07895 | Sensitivity, recall, hit rate, or true positive rate |
| Y | 0.74253 | 0.72015 | 0.76156 | 0.54714 | 0.69513 | 0.53055 | 0.07468 | Youden index |
| dInd | 0.21383 | 0.20123 | 0.17053 | 0.44803 | 0.29877 | 0.44878 | 0.92106 | Distance index |
| sInd | 0.8488 | 0.85771 | 0.87942 | 0.6832 | 0.78874 | 0.68267 | 0.34871 | Similarity index |

Generated By PyCM Version 3.3
