# Supplementary material for: TopEC: prediction of Enzyme Commission classes by 3D graph neural networks and localized 3D protein descriptor
Source: Nat Commun. 2025 Mar 20;16:2737. doi: 10.1038/s41467-025-57324-5 (PMC11923149; doi:10.1038/s41467-025-57324-5)
Supplement: Supplementary file 3 — Supplementary Data 1 [file 41467_2025_57324_MOESM3_ESM.zip › Data_S1/table1/mainclass/TopEC_distances/BindingMOAD_FOLD.html]

PyCM Report


# PyCM Report

## Dataset Type :

- Multi-Class Classification
- Imbalanced

Note 1 : Recommended statistics for this type of classification highlighted in aqua

Note 2 : The recommender system assumes that the input is the result of classification over the whole data rather than just a part of it.
If the confusion matrix is the result of test data classification, the recommendation is not valid.

## Confusion Matrix :

|  |  |  |  |  |  |  |  |  |  |  |  |  |  |  |  |  |  |  |  |  |  |  |  |  |  |  |  |  |  |  |  |  |  |  |  |  |  |  |  |  |  |  |  |  |  |  |  |  |  |  |  |  |  |  |  |  |  |  |  |  |  |  |  |  |  |
| --- | --- | --- | --- | --- | --- | --- | --- | --- | --- | --- | --- | --- | --- | --- | --- | --- | --- | --- | --- | --- | --- | --- | --- | --- | --- | --- | --- | --- | --- | --- | --- | --- | --- | --- | --- | --- | --- | --- | --- | --- | --- | --- | --- | --- | --- | --- | --- | --- | --- | --- | --- | --- | --- | --- | --- | --- | --- | --- | --- | --- | --- | --- | --- | --- | --- |
| Actual | Predict  |  |  |  |  |  |  |  |  | | --- | --- | --- | --- | --- | --- | --- | --- | |  | 0 | 1 | 2 | 3 | 4 | 5 | 6 | | 0 | 329 | 38 | 30 | 3 | 0 | 4 | 3 | | 1 | 125 | 586 | 73 | 4 | 2 | 2 | 15 | | 2 | 59 | 30 | 303 | 6 | 0 | 2 | 3 | | 3 | 64 | 36 | 18 | 0 | 0 | 0 | 14 | | 4 | 112 | 19 | 33 | 4 | 1 | 2 | 0 | | 5 | 15 | 7 | 10 | 1 | 0 | 15 | 0 | | 6 | 4 | 4 | 9 | 0 | 0 | 0 | 0 | |

## Overall Statistics :

|  |  |
| --- | --- |
| 95% CI | (0.60033,0.643) |
| ACC Macro | 0.8919 |
| ARI | 0.32677 |
| AUNP | 0.75148 |
| AUNU | 0.65055 |
| Bangdiwala B | 0.51015 |
| Bennett S | 0.55861 |
| CBA | 0.30653 |
| CSI | -0.22097 |
| Chi-Squared | 1942.22348 |
| Chi-Squared DF | 36 |
| Conditional Entropy | 1.26006 |
| Cramer V | 0.40383 |
| Cross Entropy | 2.7789 |
| F1 Macro | 0.35279 |
| F1 Micro | 0.62166 |
| FNR Macro | 0.6279 |
| FNR Micro | 0.37834 |
| FPR Macro | 0.07101 |
| FPR Micro | 0.06306 |
| Gwet AC1 | 0.57005 |
| Hamming Loss | 0.37834 |
| Joint Entropy | 3.47712 |
| KL Divergence | 0.56184 |
| Kappa | 0.4814 |
| Kappa 95% CI | (0.45215,0.51064) |
| Kappa No Prevalence | 0.24332 |
| Kappa Standard Error | 0.01492 |
| Kappa Unbiased | 0.47475 |
| Krippendorff Alpha | 0.47488 |
| Lambda A | 0.38115 |
| Lambda B | 0.55178 |
| Mutual Information | 0.55301 |
| NIR | 0.40655 |
| Overall ACC | 0.62166 |
| Overall CEN | 0.39922 |
| Overall J | (1.83176,0.26168) |
| Overall MCC | 0.49428 |
| Overall MCEN | 0.51192 |
| Overall RACC | 0.27047 |
| Overall RACCU | 0.27971 |
| P-Value | None |
| PPV Macro | 0.40692 |
| PPV Micro | 0.62166 |
| Pearson C | 0.70325 |
| Phi-Squared | 0.97845 |
| RCI | 0.24944 |
| RR | 283.57143 |
| Reference Entropy | 2.21706 |
| Response Entropy | 1.81308 |
| SOA1(Landis & Koch) | Moderate |
| SOA2(Fleiss) | Intermediate to Good |
| SOA3(Altman) | Moderate |
| SOA4(Cicchetti) | Fair |
| SOA5(Cramer) | Relatively Strong |
| SOA6(Matthews) | Weak |
| Scott PI | 0.47475 |
| Standard Error | 0.01089 |
| TNR Macro | 0.92899 |
| TNR Micro | 0.93694 |
| TPR Macro | 0.3721 |
| TPR Micro | 0.62166 |
| Zero-one Loss | 751 |

## Class Statistics :

|  |  |  |  |  |  |  |  |  |
| --- | --- | --- | --- | --- | --- | --- | --- | --- |
| Class | 0 | 1 | 2 | 3 | 4 | 5 | 6 | Description |
| ACC | 0.76977 | 0.82116 | 0.86247 | 0.92443 | 0.91335 | 0.97834 | 0.9738 | Accuracy |
| AGF | 0.79462 | 0.78806 | 0.81915 | 0.0 | 0.08227 | 0.58361 | 0.0 | Adjusted F-score |
| AGM | 0.77313 | 0.83351 | 0.85039 | 0 | 0.5169 | 0.77353 | 0 | Adjusted geometric mean |
| AM | 301 | -87 | 73 | -114 | -168 | -23 | 18 | Difference between automatic and manual classification |
| AUC | 0.78409 | 0.8062 | 0.82125 | 0.49514 | 0.50237 | 0.65367 | 0.49111 | Area under the ROC curve |
| AUCI | Good | Very Good | Very Good | Poor | Poor | Fair | Poor | AUC value interpretation |
| AUPR | 0.63652 | 0.77002 | 0.69421 | 0.0 | 0.16959 | 0.45625 | 0.0 | Area under the PR curve |
| BCD | 0.07582 | 0.02191 | 0.01839 | 0.02872 | 0.04232 | 0.00579 | 0.00453 | Bray-Curtis dissimilarity |
| BM | 0.56818 | 0.61239 | 0.64251 | -0.00971 | 0.00475 | 0.30734 | -0.01778 | Informedness or bookmaker informedness |
| CEN | 0.45811 | 0.31426 | 0.39699 | 0.65008 | 0.41455 | 0.53759 | 0.69992 | Confusion entropy |
| DOR | 13.34385 | 20.65861 | 24.67786 | 0.0 | 5.32941 | 87.59091 | 0.0 | Diagnostic odds ratio |
| DP | 0.6204 | 0.72505 | 0.76762 | None | 0.40064 | 1.07093 | None | Discriminant power |
| DPI | Poor | Poor | Poor | None | Poor | Limited | None | Discriminant power interpretation |
| ERR | 0.23023 | 0.17884 | 0.13753 | 0.07557 | 0.08665 | 0.02166 | 0.0262 | Error rate |
| F0.5 | 0.50787 | 0.79468 | 0.6567 | 0.0 | 0.02732 | 0.50676 | 0.0 | F0.5 score |
| F1 | 0.59013 | 0.76752 | 0.68942 | 0.0 | 0.01149 | 0.41096 | 0.0 | F1 score - harmonic mean of precision and sensitivity |
| F2 | 0.7042 | 0.74215 | 0.72557 | 0.0 | 0.00728 | 0.34562 | 0.0 | F2 score |
| FDR | 0.53531 | 0.18611 | 0.36345 | 1.0 | 0.66667 | 0.4 | 1.0 | False discovery rate |
| FN | 78 | 221 | 100 | 132 | 170 | 33 | 17 | False negative/miss/type 2 error |
| FNR | 0.19165 | 0.27385 | 0.24814 | 1.0 | 0.99415 | 0.6875 | 1.0 | Miss rate or false negative rate |
| FOR | 0.06108 | 0.1747 | 0.06627 | 0.06711 | 0.08577 | 0.01684 | 0.00872 | False omission rate |
| FP | 379 | 134 | 173 | 18 | 2 | 10 | 35 | False positive/type 1 error/false alarm |
| FPR | 0.24018 | 0.11375 | 0.10936 | 0.00971 | 0.0011 | 0.00516 | 0.01778 | Fall-out or false positive rate |
| G | 0.61289 | 0.76877 | 0.69181 | 0.0 | 0.04415 | 0.43301 | 0.0 | G-measure geometric mean of precision and sensitivity |
| GI | 0.56818 | 0.61239 | 0.64251 | -0.00971 | 0.00475 | 0.30734 | -0.01778 | Gini index |
| GM | 0.78371 | 0.80221 | 0.81832 | 0.0 | 0.07643 | 0.55757 | 0.0 | G-mean geometric mean of specificity and sensitivity |
| IBA | 0.64401 | 0.54051 | 0.57671 | 0.0 | 4e-05 | 0.09876 | 0.0 | Index of balanced accuracy |
| ICSI | 0.27304 | 0.54004 | 0.38842 | -1.0 | -0.66082 | -0.0875 | -1.0 | Individual classification success index |
| IS | 1.18038 | 1.0014 | 1.64864 | None | 1.95211 | 4.633 | None | Information score |
| J | 0.41858 | 0.62274 | 0.52604 | 0.0 | 0.00578 | 0.25862 | 0.0 | Jaccard index |
| LS | 2.26636 | 2.00194 | 3.13539 | 0.0 | 3.8694 | 24.8125 | 0.0 | Lift score |
| MCC | 0.47887 | 0.62565 | 0.60532 | -0.02553 | 0.03428 | 0.42335 | -0.01245 | Matthews correlation coefficient |
| MCCI | Weak | Moderate | Moderate | Negligible | Negligible | Weak | Negligible | Matthews correlation coefficient interpretation |
| MCEN | 0.56805 | 0.43647 | 0.5252 | 0.65008 | 0.41464 | 0.60799 | 0.69992 | Modified confusion entropy |
| MK | 0.40361 | 0.63919 | 0.57029 | -0.06711 | 0.24756 | 0.58316 | -0.00872 | Markedness |
| N | 1578 | 1178 | 1582 | 1853 | 1814 | 1937 | 1968 | Condition negative |
| NLR | 0.25222 | 0.309 | 0.27861 | 1.00981 | 0.99525 | 0.69107 | 1.01811 | Negative likelihood ratio |
| NLRI | Poor | Poor | Poor | Negligible | Negligible | Negligible | Negligible | Negative likelihood ratio interpretation |
| NPV | 0.93892 | 0.8253 | 0.93373 | 0.93289 | 0.91423 | 0.98316 | 0.99128 | Negative predictive value |
| OC | 0.80835 | 0.81389 | 0.75186 | 0.0 | 0.33333 | 0.6 | 0.0 | Overlap coefficient |
| OOC | 0.61289 | 0.76877 | 0.69181 | 0.0 | 0.04415 | 0.43301 | 0.0 | Otsuka-Ochiai coefficient |
| OP | 0.73883 | 0.72186 | 0.77797 | -0.07557 | -0.07501 | 0.45641 | -0.0262 | Optimized precision |
| P | 407 | 807 | 403 | 132 | 171 | 48 | 17 | Condition positive or support |
| PLR | 3.36565 | 6.38358 | 6.8754 | 0.0 | 5.30409 | 60.53125 | 0.0 | Positive likelihood ratio |
| PLRI | Poor | Fair | Fair | Negligible | Fair | Good | Negligible | Positive likelihood ratio interpretation |
| POP | 1985 | 1985 | 1985 | 1985 | 1985 | 1985 | 1985 | Population |
| PPV | 0.46469 | 0.81389 | 0.63655 | 0.0 | 0.33333 | 0.6 | 0.0 | Precision or positive predictive value |
| PRE | 0.20504 | 0.40655 | 0.20302 | 0.0665 | 0.08615 | 0.02418 | 0.00856 | Prevalence |
| Q | 0.86057 | 0.90766 | 0.92211 | -1.0 | 0.68401 | 0.97742 | -1.0 | Yule Q - coefficient of colligation |
| QI | Strong | Strong | Strong | Negligible | Moderate | Strong | Negligible | Yule Q interpretation |
| RACC | 0.07313 | 0.14746 | 0.04868 | 0.0006 | 0.00013 | 0.0003 | 0.00015 | Random accuracy |
| RACCU | 0.07888 | 0.14794 | 0.04902 | 0.00143 | 0.00192 | 0.00034 | 0.00017 | Random accuracy unbiased |
| TN | 1199 | 1044 | 1409 | 1835 | 1812 | 1927 | 1933 | True negative/correct rejection |
| TNR | 0.75982 | 0.88625 | 0.89064 | 0.99029 | 0.9989 | 0.99484 | 0.98222 | Specificity or true negative rate |
| TON | 1277 | 1265 | 1509 | 1967 | 1982 | 1960 | 1950 | Test outcome negative |
| TOP | 708 | 720 | 476 | 18 | 3 | 25 | 35 | Test outcome positive |
| TP | 329 | 586 | 303 | 0 | 1 | 15 | 0 | True positive/hit |
| TPR | 0.80835 | 0.72615 | 0.75186 | 0.0 | 0.00585 | 0.3125 | 0.0 | Sensitivity, recall, hit rate, or true positive rate |
| Y | 0.56818 | 0.61239 | 0.64251 | -0.00971 | 0.00475 | 0.30734 | -0.01778 | Youden index |
| dInd | 0.30727 | 0.29654 | 0.27117 | 1.00005 | 0.99415 | 0.68752 | 1.00016 | Distance index |
| sInd | 0.78273 | 0.79032 | 0.80826 | 0.29286 | 0.29703 | 0.51385 | 0.29278 | Similarity index |

Generated By PyCM Version 3.3
