# Supplementary material for: TopEC: prediction of Enzyme Commission classes by 3D graph neural networks and localized 3D protein descriptor
Source: Nat Commun. 2025 Mar 20;16:2737. doi: 10.1038/s41467-025-57324-5 (PMC11923149; doi:10.1038/s41467-025-57324-5)
Supplement: Supplementary file 3 — Supplementary Data 1 [file 41467_2025_57324_MOESM3_ESM.zip › Data_S1/table1/mainclass/DeepFRI/local/TopEnzyme_TEMP.html]

DeepFRI\_TopM\_TEMP\_sites


# PyCM Report

## Dataset Type :

- Multi-Class Classification
- Imbalanced

Note 1 : Recommended statistics for this type of classification highlighted in aqua

Note 2 : The recommender system assumes that the input is the result of classification over the whole data rather than just a part of it.
If the confusion matrix is the result of test data classification, the recommendation is not valid.

## Confusion Matrix :

|  |  |  |  |  |  |  |  |  |  |  |  |  |  |  |  |  |  |  |  |  |  |  |  |  |  |  |  |  |  |  |  |  |  |  |  |  |  |  |  |  |  |  |  |  |  |  |  |  |  |  |  |  |  |  |  |  |  |  |  |  |  |  |  |  |  |
| --- | --- | --- | --- | --- | --- | --- | --- | --- | --- | --- | --- | --- | --- | --- | --- | --- | --- | --- | --- | --- | --- | --- | --- | --- | --- | --- | --- | --- | --- | --- | --- | --- | --- | --- | --- | --- | --- | --- | --- | --- | --- | --- | --- | --- | --- | --- | --- | --- | --- | --- | --- | --- | --- | --- | --- | --- | --- | --- | --- | --- | --- | --- | --- | --- | --- |
| Actual | Predict  |  |  |  |  |  |  |  |  | | --- | --- | --- | --- | --- | --- | --- | --- | |  | 0 | 1 | 2 | 3 | 4 | 5 | 6 | | 0 | 58 | 49 | 91 | 14 | 2 | 0 | 4 | | 1 | 38 | 186 | 74 | 15 | 1 | 11 | 7 | | 2 | 24 | 59 | 112 | 5 | 1 | 9 | 5 | | 3 | 12 | 26 | 28 | 6 | 0 | 1 | 0 | | 4 | 6 | 10 | 8 | 2 | 2 | 4 | 0 | | 5 | 11 | 11 | 4 | 0 | 0 | 5 | 0 | | 6 | 29 | 13 | 8 | 1 | 0 | 1 | 40 | |

## Overall Statistics :

|  |  |
| --- | --- |
| 95% CI | (0.38127,0.4425) |
| ACC Macro | 0.83197 |
| ARI | 0.09419 |
| AUNP | 0.61403 |
| AUNU | 0.594 |
| Bangdiwala B | 0.22138 |
| Bennett S | 0.31386 |
| CBA | 0.26812 |
| CSI | -0.33824 |
| Chi-Squared | 488.51277 |
| Chi-Squared DF | 36 |
| Conditional Entropy | 1.87773 |
| Cramer V | 0.28634 |
| Cross Entropy | 2.5018 |
| F1 Macro | 0.30865 |
| F1 Micro | 0.41188 |
| FNR Macro | 0.70172 |
| FNR Micro | 0.58812 |
| FPR Macro | 0.11028 |
| FPR Micro | 0.09802 |
| Gwet AC1 | 0.32701 |
| Hamming Loss | 0.58812 |
| Joint Entropy | 4.27503 |
| KL Divergence | 0.1045 |
| Kappa | 0.22719 |
| Kappa 95% CI | (0.18697,0.26742) |
| Kappa No Prevalence | -0.17623 |
| Kappa Standard Error | 0.02052 |
| Kappa Unbiased | 0.22278 |
| Krippendorff Alpha | 0.22317 |
| Lambda A | 0.13918 |
| Lambda B | 0.19405 |
| Mutual Information | 0.25542 |
| NIR | 0.33434 |
| Overall ACC | 0.41188 |
| Overall CEN | 0.58229 |
| Overall J | (1.37347,0.19621) |
| Overall MCC | 0.22993 |
| Overall MCEN | 0.66945 |
| Overall RACC | 0.23899 |
| Overall RACCU | 0.24331 |
| P-Value | 0.0 |
| PPV Macro | 0.36348 |
| PPV Micro | 0.41188 |
| Pearson C | 0.57423 |
| Phi-Squared | 0.49196 |
| RCI | 0.10654 |
| RR | 141.85714 |
| Reference Entropy | 2.3973 |
| Response Entropy | 2.13315 |
| SOA1(Landis & Koch) | Fair |
| SOA2(Fleiss) | Poor |
| SOA3(Altman) | Fair |
| SOA4(Cicchetti) | Poor |
| SOA5(Cramer) | Moderate |
| SOA6(Matthews) | Negligible |
| Scott PI | 0.22278 |
| Standard Error | 0.01562 |
| TNR Macro | 0.88972 |
| TNR Micro | 0.90198 |
| TPR Macro | 0.29828 |
| TPR Micro | 0.41188 |
| Zero-one Loss | 584 |

## Class Statistics :

|  |  |  |  |  |  |  |  |  |
| --- | --- | --- | --- | --- | --- | --- | --- | --- |
| Class | 0 | 1 | 2 | 3 | 4 | 5 | 6 | Description |
| ACC | 0.71803 | 0.68379 | 0.68177 | 0.89527 | 0.96576 | 0.94763 | 0.93152 | Accuracy |
| AGF | 0.47347 | 0.6509 | 0.62206 | 0.28942 | 0.26971 | 0.39615 | 0.67005 | Adjusted F-score |
| AGM | 0.63681 | 0.68615 | 0.6639 | 0.60737 | 0.61655 | 0.67999 | 0.80989 | Adjusted geometric mean |
| AM | -40 | 22 | 110 | -30 | -26 | 0 | -36 | Difference between automatic and manual classification |
| AUC | 0.55561 | 0.65304 | 0.62358 | 0.52099 | 0.52917 | 0.56713 | 0.70851 | Area under the ROC curve |
| AUCI | Poor | Fair | Fair | Poor | Poor | Poor | Good | AUC value interpretation |
| AUPR | 0.29595 | 0.54283 | 0.43277 | 0.11086 | 0.19792 | 0.16129 | 0.57453 | Area under the PR curve |
| BCD | 0.02014 | 0.01108 | 0.05539 | 0.01511 | 0.01309 | 0.0 | 0.01813 | Bray-Curtis dissimilarity |
| BM | 0.11122 | 0.30608 | 0.24715 | 0.04197 | 0.05834 | 0.13426 | 0.41702 | Informedness or bookmaker informedness |
| CEN | 0.66119 | 0.53654 | 0.57705 | 0.69225 | 0.68782 | 0.67904 | 0.44856 | Confusion entropy |
| DOR | 1.97865 | 3.7385 | 2.88436 | 2.13715 | 15.95 | 6.92308 | 42.54808 | Diagnostic odds ratio |
| DP | 0.1634 | 0.31574 | 0.25364 | 0.18185 | 0.66312 | 0.46328 | 0.89805 | Discriminant power |
| DPI | Poor | Poor | Poor | Poor | Poor | Poor | Poor | Discriminant power interpretation |
| ERR | 0.28197 | 0.31621 | 0.31823 | 0.10473 | 0.03424 | 0.05237 | 0.06848 | Error rate |
| F0.5 | 0.31183 | 0.53204 | 0.36964 | 0.12245 | 0.17857 | 0.16129 | 0.63291 | F0.5 score |
| F1 | 0.29293 | 0.54227 | 0.41481 | 0.10345 | 0.10526 | 0.16129 | 0.54054 | F1 score - harmonic mean of precision and sensitivity |
| F2 | 0.27619 | 0.55291 | 0.47257 | 0.08955 | 0.07463 | 0.16129 | 0.4717 | F2 score |
| FDR | 0.67416 | 0.47458 | 0.65538 | 0.86047 | 0.66667 | 0.83871 | 0.28571 | False discovery rate |
| FN | 160 | 146 | 103 | 67 | 30 | 26 | 52 | False negative/miss/type 2 error |
| FNR | 0.73394 | 0.43976 | 0.47907 | 0.91781 | 0.9375 | 0.83871 | 0.56522 | Miss rate or false negative rate |
| FOR | 0.19632 | 0.22848 | 0.15419 | 0.07053 | 0.0304 | 0.02703 | 0.0555 | False omission rate |
| FP | 120 | 168 | 213 | 37 | 4 | 26 | 16 | False positive/type 1 error/false alarm |
| FPR | 0.15484 | 0.25416 | 0.27378 | 0.04022 | 0.00416 | 0.02703 | 0.01776 | Fall-out or false positive rate |
| G | 0.29444 | 0.54255 | 0.4237 | 0.10709 | 0.14434 | 0.16129 | 0.55728 | G-measure geometric mean of precision and sensitivity |
| GI | 0.11122 | 0.30608 | 0.24715 | 0.04197 | 0.05834 | 0.13426 | 0.41702 | Gini index |
| GM | 0.47419 | 0.64641 | 0.61507 | 0.28087 | 0.24948 | 0.39615 | 0.6535 | G-mean geometric mean of specificity and sensitivity |
| IBA | 0.09464 | 0.3403 | 0.30065 | 0.00966 | 0.00415 | 0.02955 | 0.19326 | Index of balanced accuracy |
| ICSI | -0.4081 | 0.08566 | -0.13445 | -0.77827 | -0.60417 | -0.67742 | 0.14907 | Individual classification success index |
| IS | 0.56971 | 0.65216 | 0.67052 | 0.92452 | 3.37069 | 2.36919 | 2.94666 | Information score |
| J | 0.1716 | 0.372 | 0.26168 | 0.05455 | 0.05556 | 0.08772 | 0.37037 | Jaccard index |
| LS | 1.48423 | 1.57152 | 1.59164 | 1.89806 | 10.34375 | 5.16649 | 7.70963 | Lift score |
| MCC | 0.12002 | 0.30148 | 0.21694 | 0.05382 | 0.13294 | 0.13426 | 0.52415 | Matthews correlation coefficient |
| MCCI | Negligible | Weak | Negligible | Negligible | Negligible | Negligible | Moderate | Matthews correlation coefficient interpretation |
| MCEN | 0.72186 | 0.65621 | 0.65899 | 0.7098 | 0.70548 | 0.70773 | 0.53486 | Modified confusion entropy |
| MK | 0.12952 | 0.29694 | 0.19042 | 0.06901 | 0.30294 | 0.13426 | 0.65879 | Markedness |
| N | 775 | 661 | 778 | 920 | 961 | 962 | 901 | Condition negative |
| NLR | 0.86841 | 0.58962 | 0.65967 | 0.95627 | 0.94142 | 0.86201 | 0.57544 | Negative likelihood ratio |
| NLRI | Negligible | Negligible | Negligible | Negligible | Negligible | Negligible | Negligible | Negative likelihood ratio interpretation |
| NPV | 0.80368 | 0.77152 | 0.84581 | 0.92947 | 0.9696 | 0.97297 | 0.9445 | Negative predictive value |
| OC | 0.32584 | 0.56024 | 0.52093 | 0.13953 | 0.33333 | 0.16129 | 0.71429 | Overlap coefficient |
| OOC | 0.29444 | 0.54255 | 0.4237 | 0.10709 | 0.14434 | 0.16129 | 0.55728 | Otsuka-Ochiai coefficient |
| OP | 0.19688 | 0.54168 | 0.51716 | 0.05303 | 0.08387 | 0.23203 | 0.54518 | Optimized precision |
| P | 218 | 332 | 215 | 73 | 32 | 31 | 92 | Condition positive or support |
| PLR | 1.71827 | 2.20428 | 1.90274 | 2.04369 | 15.01563 | 5.96774 | 24.4837 | Positive likelihood ratio |
| PLRI | Poor | Poor | Poor | Poor | Good | Fair | Good | Positive likelihood ratio interpretation |
| POP | 993 | 993 | 993 | 993 | 993 | 993 | 993 | Population |
| PPV | 0.32584 | 0.52542 | 0.34462 | 0.13953 | 0.33333 | 0.16129 | 0.71429 | Precision or positive predictive value |
| PRE | 0.21954 | 0.33434 | 0.21652 | 0.07351 | 0.03223 | 0.03122 | 0.09265 | Prevalence |
| Q | 0.32855 | 0.57793 | 0.48511 | 0.36248 | 0.88201 | 0.74757 | 0.95407 | Yule Q - coefficient of colligation |
| QI | Weak | Moderate | Weak | Weak | Strong | Moderate | Strong | Yule Q interpretation |
| RACC | 0.03935 | 0.11919 | 0.07086 | 0.00318 | 0.00019 | 0.00097 | 0.00522 | Random accuracy |
| RACCU | 0.03976 | 0.11931 | 0.07393 | 0.00341 | 0.00037 | 0.00097 | 0.00555 | Random accuracy unbiased |
| TN | 655 | 493 | 565 | 883 | 957 | 936 | 885 | True negative/correct rejection |
| TNR | 0.84516 | 0.74584 | 0.72622 | 0.95978 | 0.99584 | 0.97297 | 0.98224 | Specificity or true negative rate |
| TON | 815 | 639 | 668 | 950 | 987 | 962 | 937 | Test outcome negative |
| TOP | 178 | 354 | 325 | 43 | 6 | 31 | 56 | Test outcome positive |
| TP | 58 | 186 | 112 | 6 | 2 | 5 | 40 | True positive/hit |
| TPR | 0.26606 | 0.56024 | 0.52093 | 0.08219 | 0.0625 | 0.16129 | 0.43478 | Sensitivity, recall, hit rate, or true positive rate |
| Y | 0.11122 | 0.30608 | 0.24715 | 0.04197 | 0.05834 | 0.13426 | 0.41702 | Youden index |
| dInd | 0.7501 | 0.50792 | 0.55178 | 0.91869 | 0.93751 | 0.83915 | 0.5655 | Distance index |
| sInd | 0.4696 | 0.64084 | 0.60983 | 0.35039 | 0.33708 | 0.40663 | 0.60013 | Similarity index |

Generated By PyCM Version 3.1
