# Supplementary material for: TopEC: prediction of Enzyme Commission classes by 3D graph neural networks and localized 3D protein descriptor
Source: Nat Commun. 2025 Mar 20;16:2737. doi: 10.1038/s41467-025-57324-5 (PMC11923149; doi:10.1038/s41467-025-57324-5)
Supplement: Supplementary file 3 — Supplementary Data 1 [file 41467_2025_57324_MOESM3_ESM.zip › Data_S1/table1/mainclass/DeepFRI/local/TopEnzyme_FOLD.html]

DeepFRI\_TopM\_FOLD\_sites


# PyCM Report

## Dataset Type :

- Multi-Class Classification
- Imbalanced

Note 1 : Recommended statistics for this type of classification highlighted in aqua

Note 2 : The recommender system assumes that the input is the result of classification over the whole data rather than just a part of it.
If the confusion matrix is the result of test data classification, the recommendation is not valid.

## Confusion Matrix :

|  |  |  |  |  |  |  |  |  |  |  |  |  |  |  |  |  |  |  |  |  |  |  |  |  |  |  |  |  |  |  |  |  |  |  |  |  |  |  |  |  |  |  |  |  |  |  |  |  |  |  |  |  |  |  |  |  |  |  |  |  |  |  |  |  |  |
| --- | --- | --- | --- | --- | --- | --- | --- | --- | --- | --- | --- | --- | --- | --- | --- | --- | --- | --- | --- | --- | --- | --- | --- | --- | --- | --- | --- | --- | --- | --- | --- | --- | --- | --- | --- | --- | --- | --- | --- | --- | --- | --- | --- | --- | --- | --- | --- | --- | --- | --- | --- | --- | --- | --- | --- | --- | --- | --- | --- | --- | --- | --- | --- | --- | --- |
| Actual | Predict  |  |  |  |  |  |  |  |  | | --- | --- | --- | --- | --- | --- | --- | --- | |  | 0 | 1 | 2 | 3 | 4 | 5 | 6 | | 0 | 25 | 82 | 37 | 22 | 0 | 0 | 4 | | 1 | 16 | 174 | 39 | 16 | 0 | 0 | 0 | | 2 | 9 | 57 | 97 | 16 | 0 | 0 | 0 | | 3 | 9 | 41 | 22 | 9 | 0 | 0 | 2 | | 4 | 12 | 37 | 21 | 14 | 1 | 0 | 0 | | 5 | 1 | 60 | 16 | 14 | 0 | 2 | 0 | | 6 | 0 | 34 | 1 | 1 | 0 | 0 | 2 | |

## Overall Statistics :

|  |  |
| --- | --- |
| 95% CI | (0.31592,0.37837) |
| ACC Macro | 0.81347 |
| ARI | 0.05083 |
| AUNP | 0.57755 |
| AUNU | 0.55347 |
| Bangdiwala B | 0.22323 |
| Bennett S | 0.23834 |
| CBA | 0.15798 |
| CSI | -0.27663 |
| Chi-Squared | 203.00161 |
| Chi-Squared DF | 36 |
| Conditional Entropy | 1.55394 |
| Cramer V | 0.19465 |
| Cross Entropy | 3.76569 |
| F1 Macro | 0.20134 |
| F1 Micro | 0.34714 |
| FNR Macro | 0.77236 |
| FNR Micro | 0.65286 |
| FPR Macro | 0.1207 |
| FPR Micro | 0.10881 |
| Gwet AC1 | 0.25445 |
| Hamming Loss | 0.65286 |
| Joint Entropy | 4.16142 |
| KL Divergence | 1.15821 |
| Kappa | 0.15548 |
| Kappa 95% CI | (0.11509,0.19587) |
| Kappa No Prevalence | -0.30571 |
| Kappa Standard Error | 0.02061 |
| Kappa Unbiased | 0.1248 |
| Krippendorff Alpha | 0.12529 |
| Lambda A | 0.12346 |
| Lambda B | 0.09804 |
| Mutual Information | 0.15244 |
| NIR | 0.27436 |
| Overall ACC | 0.34714 |
| Overall CEN | 0.57473 |
| Overall J | (0.86904,0.12415) |
| Overall MCC | 0.16881 |
| Overall MCEN | 0.64185 |
| Overall RACC | 0.22695 |
| Overall RACCU | 0.25405 |
| P-Value | 0.0 |
| PPV Macro | 0.49573 |
| PPV Micro | 0.34714 |
| Pearson C | 0.43037 |
| Phi-Squared | 0.22733 |
| RCI | 0.05846 |
| RR | 127.57143 |
| Reference Entropy | 2.60748 |
| Response Entropy | 1.70638 |
| SOA1(Landis & Koch) | Slight |
| SOA2(Fleiss) | Poor |
| SOA3(Altman) | Poor |
| SOA4(Cicchetti) | Poor |
| SOA5(Cramer) | Weak |
| SOA6(Matthews) | Negligible |
| Scott PI | 0.1248 |
| Standard Error | 0.01593 |
| TNR Macro | 0.8793 |
| TNR Micro | 0.89119 |
| TPR Macro | 0.22764 |
| TPR Micro | 0.34714 |
| Zero-one Loss | 583 |

## Class Statistics :

|  |  |  |  |  |  |  |  |  |
| --- | --- | --- | --- | --- | --- | --- | --- | --- |
| Class | 0 | 1 | 2 | 3 | 4 | 5 | 6 | Description |
| ACC | 0.78499 | 0.57223 | 0.75588 | 0.82419 | 0.90594 | 0.8981 | 0.95297 | Accuracy |
| AGF | 0.37445 | 0.66248 | 0.6636 | 0.31002 | 0.11635 | 0.15655 | 0.2457 | Adjusted F-score |
| AGM | 0.62323 | 0.57087 | 0.72773 | 0.59048 | 0.53196 | 0.54989 | 0.60249 | Adjusted geometric mean |
| AM | -98 | 240 | 54 | 9 | -84 | -91 | -30 | Difference between automatic and manual classification |
| AUC | 0.54103 | 0.61513 | 0.67571 | 0.50298 | 0.50588 | 0.51075 | 0.52281 | Area under the ROC curve |
| AUCI | Poor | Fair | Fair | Poor | Poor | Poor | Poor | AUC value interpretation |
| AUPR | 0.24714 | 0.53448 | 0.4791 | 0.10313 | 0.50588 | 0.51075 | 0.15132 | Area under the PR curve |
| BCD | 0.05487 | 0.13438 | 0.03024 | 0.00504 | 0.04703 | 0.05095 | 0.0168 | Bray-Curtis dissimilarity |
| BM | 0.08205 | 0.23027 | 0.35142 | 0.00596 | 0.01176 | 0.02151 | 0.04561 | Informedness or bookmaker informedness |
| CEN | 0.61802 | 0.57537 | 0.55208 | 0.77918 | 0.51408 | 0.37038 | 0.29723 | Confusion entropy |
| DOR | 2.47982 | 2.65559 | 5.02744 | 1.06529 | None | None | 7.86111 | Diagnostic odds ratio |
| DP | 0.21746 | 0.23385 | 0.38667 | 0.01514 | None | None | 0.49371 | Discriminant power |
| DPI | Poor | Poor | Poor | Poor | None | None | Poor | Discriminant power interpretation |
| ERR | 0.21501 | 0.42777 | 0.24412 | 0.17581 | 0.09406 | 0.1019 | 0.04703 | Error rate |
| F0.5 | 0.27293 | 0.39817 | 0.43654 | 0.09978 | 0.05618 | 0.09901 | 0.14286 | F0.5 score |
| F1 | 0.20661 | 0.47671 | 0.47087 | 0.10286 | 0.02326 | 0.04211 | 0.08696 | F1 score - harmonic mean of precision and sensitivity |
| F2 | 0.16622 | 0.59386 | 0.51106 | 0.10613 | 0.01466 | 0.02674 | 0.0625 | F2 score |
| FDR | 0.65278 | 0.64124 | 0.58369 | 0.90217 | 0.0 | 0.0 | 0.75 | False discovery rate |
| FN | 145 | 71 | 82 | 74 | 84 | 91 | 36 | False negative/miss/type 2 error |
| FNR | 0.85294 | 0.2898 | 0.4581 | 0.89157 | 0.98824 | 0.97849 | 0.94737 | Miss rate or false negative rate |
| FOR | 0.17661 | 0.17402 | 0.12424 | 0.09238 | 0.09417 | 0.10213 | 0.04068 | False omission rate |
| FP | 47 | 311 | 136 | 83 | 0 | 0 | 6 | False positive/type 1 error/false alarm |
| FPR | 0.06501 | 0.47994 | 0.19048 | 0.10247 | 0.0 | 0.0 | 0.00702 | Fall-out or false positive rate |
| G | 0.22597 | 0.50477 | 0.47497 | 0.10299 | 0.10847 | 0.14665 | 0.11471 | G-measure geometric mean of precision and sensitivity |
| GI | 0.08205 | 0.23027 | 0.35142 | 0.00596 | 0.01176 | 0.02151 | 0.04561 | Gini index |
| GM | 0.37081 | 0.60774 | 0.66233 | 0.31197 | 0.10847 | 0.14665 | 0.22861 | G-mean geometric mean of specificity and sensitivity |
| IBA | 0.02916 | 0.43958 | 0.32128 | 0.02053 | 0.00014 | 0.00046 | 0.00312 | Index of balanced accuracy |
| ICSI | -0.50572 | 0.06897 | -0.04179 | -0.79374 | 0.01176 | 0.02151 | -0.69737 | Individual classification success index |
| IS | 0.86706 | 0.38698 | 1.05443 | 0.07384 | 3.39313 | 3.26336 | 2.55459 | Information score |
| J | 0.11521 | 0.31295 | 0.30794 | 0.05422 | 0.01176 | 0.02151 | 0.04545 | Jaccard index |
| LS | 1.82394 | 1.30765 | 2.07689 | 1.05251 | 10.50588 | 9.60215 | 5.875 | Lift score |
| MCC | 0.11832 | 0.20625 | 0.32037 | 0.0057 | 0.10323 | 0.13896 | 0.09771 | Matthews correlation coefficient |
| MCCI | Negligible | Negligible | Weak | Negligible | Negligible | Negligible | Negligible | Matthews correlation coefficient interpretation |
| MCEN | 0.65039 | 0.68016 | 0.64732 | 0.80132 | 0.51548 | 0.36997 | 0.29367 | Modified confusion entropy |
| MK | 0.17061 | 0.18474 | 0.29207 | 0.00544 | 0.90583 | 0.89787 | 0.20932 | Markedness |
| N | 723 | 648 | 714 | 810 | 808 | 800 | 855 | Condition negative |
| NLR | 0.91224 | 0.55723 | 0.56589 | 0.99335 | 0.98824 | 0.97849 | 0.95406 | Negative likelihood ratio |
| NLRI | Negligible | Negligible | Negligible | Negligible | Negligible | Negligible | Negligible | Negative likelihood ratio interpretation |
| NPV | 0.82339 | 0.82598 | 0.87576 | 0.90762 | 0.90583 | 0.89787 | 0.95932 | Negative predictive value |
| OC | 0.34722 | 0.7102 | 0.5419 | 0.10843 | 1.0 | 1.0 | 0.25 | Overlap coefficient |
| OOC | 0.22597 | 0.50477 | 0.47497 | 0.10299 | 0.10847 | 0.14665 | 0.11471 | Otsuka-Ochiai coefficient |
| OP | 0.05681 | 0.41767 | 0.55785 | 0.03977 | -0.07081 | -0.0598 | 0.05364 | Optimized precision |
| P | 170 | 245 | 179 | 83 | 85 | 93 | 38 | Condition positive or support |
| PLR | 2.2622 | 1.47978 | 2.84497 | 1.05821 | None | None | 7.5 | Positive likelihood ratio |
| PLRI | Poor | Poor | Poor | Poor | None | None | Fair | Positive likelihood ratio interpretation |
| POP | 893 | 893 | 893 | 893 | 893 | 893 | 893 | Population |
| PPV | 0.34722 | 0.35876 | 0.41631 | 0.09783 | 1.0 | 1.0 | 0.25 | Precision or positive predictive value |
| PRE | 0.19037 | 0.27436 | 0.20045 | 0.09295 | 0.09518 | 0.10414 | 0.04255 | Prevalence |
| Q | 0.42526 | 0.45289 | 0.66818 | 0.03161 | None | None | 0.77429 | Yule Q - coefficient of colligation |
| QI | Weak | Weak | Moderate | Negligible | None | None | Strong | Yule Q interpretation |
| RACC | 0.01535 | 0.14901 | 0.0523 | 0.00958 | 0.00011 | 0.00023 | 0.00038 | Random accuracy |
| RACCU | 0.01836 | 0.16706 | 0.05321 | 0.0096 | 0.00232 | 0.00283 | 0.00066 | Random accuracy unbiased |
| TN | 676 | 337 | 578 | 727 | 808 | 800 | 849 | True negative/correct rejection |
| TNR | 0.93499 | 0.52006 | 0.80952 | 0.89753 | 1.0 | 1.0 | 0.99298 | Specificity or true negative rate |
| TON | 821 | 408 | 660 | 801 | 892 | 891 | 885 | Test outcome negative |
| TOP | 72 | 485 | 233 | 92 | 1 | 2 | 8 | Test outcome positive |
| TP | 25 | 174 | 97 | 9 | 1 | 2 | 2 | True positive/hit |
| TPR | 0.14706 | 0.7102 | 0.5419 | 0.10843 | 0.01176 | 0.02151 | 0.05263 | Sensitivity, recall, hit rate, or true positive rate |
| Y | 0.08205 | 0.23027 | 0.35142 | 0.00596 | 0.01176 | 0.02151 | 0.04561 | Youden index |
| dInd | 0.85541 | 0.56064 | 0.49612 | 0.89744 | 0.98824 | 0.97849 | 0.94739 | Distance index |
| sInd | 0.39513 | 0.60356 | 0.64919 | 0.36542 | 0.30121 | 0.3081 | 0.33009 | Similarity index |

Generated By PyCM Version 3.1
