# Supplementary material for: TopEC: prediction of Enzyme Commission classes by 3D graph neural networks and localized 3D protein descriptor
Source: Nat Commun. 2025 Mar 20;16:2737. doi: 10.1038/s41467-025-57324-5 (PMC11923149; doi:10.1038/s41467-025-57324-5)
Supplement: Supplementary file 3 — Supplementary Data 1 [file 41467_2025_57324_MOESM3_ESM.zip › Data_S1/table1/mainclass/DeepFRI/local/Combined_TEMP.html]

DeepFRI\_Both\_TEMP\_sites


# PyCM Report

## Dataset Type :

- Multi-Class Classification
- Imbalanced

Note 1 : Recommended statistics for this type of classification highlighted in aqua

Note 2 : The recommender system assumes that the input is the result of classification over the whole data rather than just a part of it.
If the confusion matrix is the result of test data classification, the recommendation is not valid.

## Confusion Matrix :

|  |  |  |  |  |  |  |  |  |  |  |  |  |  |  |  |  |  |  |  |  |  |  |  |  |  |  |  |  |  |  |  |  |  |  |  |  |  |  |  |  |  |  |  |  |  |  |  |  |  |  |  |  |  |  |  |  |  |  |  |  |  |  |  |  |  |
| --- | --- | --- | --- | --- | --- | --- | --- | --- | --- | --- | --- | --- | --- | --- | --- | --- | --- | --- | --- | --- | --- | --- | --- | --- | --- | --- | --- | --- | --- | --- | --- | --- | --- | --- | --- | --- | --- | --- | --- | --- | --- | --- | --- | --- | --- | --- | --- | --- | --- | --- | --- | --- | --- | --- | --- | --- | --- | --- | --- | --- | --- | --- | --- | --- | --- |
| Actual | Predict  |  |  |  |  |  |  |  |  | | --- | --- | --- | --- | --- | --- | --- | --- | |  | 0 | 1 | 2 | 3 | 4 | 5 | 6 | | 0 | 300 | 199 | 116 | 9 | 7 | 5 | 5 | | 1 | 162 | 643 | 126 | 18 | 8 | 19 | 10 | | 2 | 101 | 245 | 513 | 11 | 0 | 9 | 5 | | 3 | 44 | 81 | 21 | 41 | 0 | 1 | 0 | | 4 | 35 | 34 | 14 | 5 | 19 | 2 | 0 | | 5 | 15 | 52 | 11 | 0 | 0 | 11 | 0 | | 6 | 29 | 50 | 45 | 2 | 0 | 5 | 37 | |

## Overall Statistics :

|  |  |
| --- | --- |
| 95% CI | (0.49258,0.52798) |
| ACC Macro | 0.86008 |
| ARI | 0.1389 |
| AUNP | 0.66437 |
| AUNU | 0.6261 |
| Bangdiwala B | 0.30715 |
| Bennett S | 0.42866 |
| CBA | 0.32099 |
| CSI | -0.16147 |
| Chi-Squared | 1870.98873 |
| Chi-Squared DF | 36 |
| Conditional Entropy | 1.66066 |
| Cramer V | 0.31897 |
| Cross Entropy | 2.41697 |
| F1 Macro | 0.37953 |
| F1 Micro | 0.51028 |
| FNR Macro | 0.6519 |
| FNR Micro | 0.48972 |
| FPR Macro | 0.09589 |
| FPR Micro | 0.08162 |
| Gwet AC1 | 0.44249 |
| Hamming Loss | 0.48972 |
| Joint Entropy | 3.97259 |
| KL Divergence | 0.10504 |
| Kappa | 0.33197 |
| Kappa 95% CI | (0.30783,0.35612) |
| Kappa No Prevalence | 0.02055 |
| Kappa Standard Error | 0.01232 |
| Kappa Unbiased | 0.3287 |
| Krippendorff Alpha | 0.32881 |
| Lambda A | 0.28187 |
| Lambda B | 0.21011 |
| Mutual Information | 0.28325 |
| NIR | 0.3217 |
| Overall ACC | 0.51028 |
| Overall CEN | 0.51174 |
| Overall J | (1.71466,0.24495) |
| Overall MCC | 0.33563 |
| Overall MCEN | 0.61231 |
| Overall RACC | 0.26691 |
| Overall RACCU | 0.27048 |
| P-Value | None |
| PPV Macro | 0.49043 |
| PPV Micro | 0.51028 |
| Pearson C | 0.61567 |
| Phi-Squared | 0.61044 |
| RCI | 0.12252 |
| RR | 437.85714 |
| Reference Entropy | 2.31193 |
| Response Entropy | 1.94391 |
| SOA1(Landis & Koch) | Fair |
| SOA2(Fleiss) | Poor |
| SOA3(Altman) | Fair |
| SOA4(Cicchetti) | Poor |
| SOA5(Cramer) | Moderate |
| SOA6(Matthews) | Weak |
| Scott PI | 0.3287 |
| Standard Error | 0.00903 |
| TNR Macro | 0.90411 |
| TNR Micro | 0.91838 |
| TPR Macro | 0.3481 |
| TPR Micro | 0.51028 |
| Zero-one Loss | 1501 |

## Class Statistics :

|  |  |  |  |  |  |  |  |  |
| --- | --- | --- | --- | --- | --- | --- | --- | --- |
| Class | 0 | 1 | 2 | 3 | 4 | 5 | 6 | Description |
| ACC | 0.76281 | 0.67243 | 0.77031 | 0.93736 | 0.96574 | 0.96117 | 0.95073 | Accuracy |
| AGF | 0.62761 | 0.69 | 0.6994 | 0.4839 | 0.44396 | 0.36282 | 0.49449 | Adjusted F-score |
| AGM | 0.72156 | 0.67304 | 0.76196 | 0.7156 | 0.70045 | 0.66298 | 0.72298 | Adjusted geometric mean |
| AM | 45 | 318 | -38 | -102 | -75 | -37 | -111 | Difference between automatic and manual classification |
| AUC | 0.65439 | 0.66709 | 0.71382 | 0.60122 | 0.58462 | 0.55491 | 0.60667 | Area under the ROC curve |
| AUCI | Fair | Fair | Good | Fair | Poor | Poor | Fair | AUC value interpretation |
| AUPR | 0.45267 | 0.57261 | 0.59335 | 0.34741 | 0.36657 | 0.16757 | 0.43468 | Area under the PR curve |
| BCD | 0.00734 | 0.05188 | 0.0062 | 0.01664 | 0.01223 | 0.00604 | 0.01811 | Bray-Curtis dissimilarity |
| BM | 0.30878 | 0.33419 | 0.42763 | 0.20244 | 0.16924 | 0.10982 | 0.21333 | Informedness or bookmaker informedness |
| CEN | 0.56219 | 0.51103 | 0.44697 | 0.56358 | 0.5632 | 0.63715 | 0.54502 | Confusion entropy |
| DOR | 4.64498 | 4.02153 | 7.67364 | 17.55283 | 41.39185 | 10.09537 | 40.62939 | Diagnostic odds ratio |
| DP | 0.36773 | 0.33322 | 0.48793 | 0.68604 | 0.89145 | 0.5536 | 0.887 | Discriminant power |
| DPI | Poor | Poor | Poor | Poor | Poor | Poor | Poor | Discriminant power interpretation |
| ERR | 0.23719 | 0.32757 | 0.22969 | 0.06264 | 0.03426 | 0.03883 | 0.04927 | Error rate |
| F0.5 | 0.44313 | 0.51838 | 0.60098 | 0.38534 | 0.38776 | 0.18519 | 0.46717 | F0.5 score |
| F1 | 0.45215 | 0.56157 | 0.59306 | 0.29927 | 0.26573 | 0.15603 | 0.32889 | F1 score - harmonic mean of precision and sensitivity |
| F2 | 0.46154 | 0.61261 | 0.58535 | 0.24463 | 0.20213 | 0.1348 | 0.25377 | F2 score |
| FDR | 0.56268 | 0.5069 | 0.39362 | 0.52326 | 0.44118 | 0.78846 | 0.35088 | False discovery rate |
| FN | 341 | 343 | 371 | 147 | 90 | 78 | 131 | False negative/miss/type 2 error |
| FNR | 0.53198 | 0.34787 | 0.41968 | 0.78191 | 0.82569 | 0.8764 | 0.77976 | Miss rate or false negative rate |
| FOR | 0.14334 | 0.19478 | 0.16719 | 0.04935 | 0.02969 | 0.02589 | 0.04355 | False omission rate |
| FP | 386 | 661 | 333 | 45 | 15 | 41 | 20 | False positive/type 1 error/false alarm |
| FPR | 0.15924 | 0.31794 | 0.15268 | 0.01564 | 0.00507 | 0.01378 | 0.0069 | Fall-out or false positive rate |
| G | 0.45241 | 0.56707 | 0.59321 | 0.32245 | 0.31211 | 0.16169 | 0.3781 | G-measure geometric mean of precision and sensitivity |
| GI | 0.30878 | 0.33419 | 0.42763 | 0.20244 | 0.16924 | 0.10982 | 0.21333 | Gini index |
| GM | 0.62729 | 0.66693 | 0.70122 | 0.46333 | 0.41645 | 0.34913 | 0.46767 | G-mean geometric mean of specificity and sensitivity |
| IBA | 0.24682 | 0.43148 | 0.36042 | 0.05017 | 0.03111 | 0.01674 | 0.04968 | Index of balanced accuracy |
| ICSI | -0.09466 | 0.14523 | 0.1867 | -0.30517 | -0.26686 | -0.66487 | -0.13064 | Individual classification success index |
| IS | 1.06424 | 0.61617 | 1.07207 | 2.95837 | 3.97395 | 2.86493 | 3.56592 | Information score |
| J | 0.29211 | 0.39041 | 0.42153 | 0.17597 | 0.15323 | 0.08462 | 0.19681 | Jaccard index |
| LS | 2.09107 | 1.53281 | 2.10245 | 7.77245 | 15.71371 | 7.285 | 11.84263 | Lift score |
| MCC | 0.30129 | 0.31575 | 0.43337 | 0.29415 | 0.29925 | 0.14279 | 0.35943 | Matthews correlation coefficient |
| MCCI | Weak | Weak | Weak | Negligible | Negligible | Negligible | Weak | Matthews correlation coefficient interpretation |
| MCEN | 0.65341 | 0.62968 | 0.55349 | 0.60899 | 0.60091 | 0.66114 | 0.59422 | Modified confusion entropy |
| MK | 0.29398 | 0.29832 | 0.43919 | 0.4274 | 0.52913 | 0.18565 | 0.60557 | Markedness |
| N | 2424 | 2079 | 2181 | 2877 | 2956 | 2976 | 2897 | Condition negative |
| NLR | 0.63274 | 0.51003 | 0.49531 | 0.79434 | 0.8299 | 0.88865 | 0.78518 | Negative likelihood ratio |
| NLRI | Negligible | Negligible | Poor | Negligible | Negligible | Negligible | Negligible | Negative likelihood ratio interpretation |
| NPV | 0.85666 | 0.80522 | 0.83281 | 0.95065 | 0.97031 | 0.97411 | 0.95645 | Negative predictive value |
| OC | 0.46802 | 0.65213 | 0.60638 | 0.47674 | 0.55882 | 0.21154 | 0.64912 | Overlap coefficient |
| OOC | 0.45241 | 0.56707 | 0.59321 | 0.32245 | 0.31211 | 0.16169 | 0.3781 | Otsuka-Ochiai coefficient |
| OP | 0.47801 | 0.65 | 0.58329 | 0.30009 | 0.26391 | 0.18391 | 0.31376 | Optimized precision |
| P | 641 | 986 | 884 | 188 | 109 | 89 | 168 | Condition positive or support |
| PLR | 2.93906 | 2.0511 | 3.80081 | 13.94291 | 34.35107 | 8.97122 | 31.90149 | Positive likelihood ratio |
| PLRI | Poor | Poor | Poor | Good | Good | Fair | Good | Positive likelihood ratio interpretation |
| POP | 3065 | 3065 | 3065 | 3065 | 3065 | 3065 | 3065 | Population |
| PPV | 0.43732 | 0.4931 | 0.60638 | 0.47674 | 0.55882 | 0.21154 | 0.64912 | Precision or positive predictive value |
| PRE | 0.20914 | 0.3217 | 0.28842 | 0.06134 | 0.03556 | 0.02904 | 0.05481 | Prevalence |
| Q | 0.6457 | 0.60172 | 0.76942 | 0.8922 | 0.95282 | 0.81974 | 0.95196 | Yule Q - coefficient of colligation |
| QI | Moderate | Moderate | Strong | Strong | Strong | Strong | Strong | Yule Q interpretation |
| RACC | 0.04681 | 0.13687 | 0.07961 | 0.00172 | 0.00039 | 0.00049 | 0.00102 | Random accuracy |
| RACCU | 0.04686 | 0.13956 | 0.07965 | 0.002 | 0.00054 | 0.00053 | 0.00135 | Random accuracy unbiased |
| TN | 2038 | 1418 | 1848 | 2832 | 2941 | 2935 | 2877 | True negative/correct rejection |
| TNR | 0.84076 | 0.68206 | 0.84732 | 0.98436 | 0.99493 | 0.98622 | 0.9931 | Specificity or true negative rate |
| TON | 2379 | 1761 | 2219 | 2979 | 3031 | 3013 | 3008 | Test outcome negative |
| TOP | 686 | 1304 | 846 | 86 | 34 | 52 | 57 | Test outcome positive |
| TP | 300 | 643 | 513 | 41 | 19 | 11 | 37 | True positive/hit |
| TPR | 0.46802 | 0.65213 | 0.58032 | 0.21809 | 0.17431 | 0.1236 | 0.22024 | Sensitivity, recall, hit rate, or true positive rate |
| Y | 0.30878 | 0.33419 | 0.42763 | 0.20244 | 0.16924 | 0.10982 | 0.21333 | Youden index |
| dInd | 0.5553 | 0.47128 | 0.44659 | 0.78207 | 0.8257 | 0.87651 | 0.77979 | Distance index |
| sInd | 0.60734 | 0.66676 | 0.68421 | 0.44699 | 0.41614 | 0.38021 | 0.4486 | Similarity index |

Generated By PyCM Version 3.1
