# Supplementary material for: TopEC: prediction of Enzyme Commission classes by 3D graph neural networks and localized 3D protein descriptor
Source: Nat Commun. 2025 Mar 20;16:2737. doi: 10.1038/s41467-025-57324-5 (PMC11923149; doi:10.1038/s41467-025-57324-5)
Supplement: Supplementary file 3 — Supplementary Data 1 [file 41467_2025_57324_MOESM3_ESM.zip › Data_S1/table1/mainclass/DeepFRI/local/Combined_FOLD.html]

DeepFRI\_Both\_FOLD\_sites


# PyCM Report

## Dataset Type :

- Multi-Class Classification
- Imbalanced

Note 1 : Recommended statistics for this type of classification highlighted in aqua

Note 2 : The recommender system assumes that the input is the result of classification over the whole data rather than just a part of it.
If the confusion matrix is the result of test data classification, the recommendation is not valid.

## Confusion Matrix :

|  |  |  |  |  |  |  |  |  |  |  |  |  |  |  |  |  |  |  |  |  |  |  |  |  |  |  |  |  |  |  |  |  |  |  |  |  |  |  |  |  |  |  |  |  |  |  |  |  |  |  |  |  |  |  |  |  |  |  |  |  |  |  |  |  |  |
| --- | --- | --- | --- | --- | --- | --- | --- | --- | --- | --- | --- | --- | --- | --- | --- | --- | --- | --- | --- | --- | --- | --- | --- | --- | --- | --- | --- | --- | --- | --- | --- | --- | --- | --- | --- | --- | --- | --- | --- | --- | --- | --- | --- | --- | --- | --- | --- | --- | --- | --- | --- | --- | --- | --- | --- | --- | --- | --- | --- | --- | --- | --- | --- | --- | --- |
| Actual | Predict  |  |  |  |  |  |  |  |  | | --- | --- | --- | --- | --- | --- | --- | --- | |  | 0 | 1 | 2 | 3 | 4 | 5 | 6 | | 0 | 364 | 159 | 48 | 6 | 0 | 0 | 0 | | 1 | 174 | 840 | 33 | 3 | 0 | 1 | 0 | | 2 | 48 | 138 | 372 | 14 | 0 | 9 | 0 | | 3 | 69 | 88 | 23 | 1 | 0 | 6 | 28 | | 4 | 145 | 97 | 12 | 1 | 0 | 1 | 0 | | 5 | 33 | 59 | 23 | 0 | 0 | 26 | 0 | | 6 | 11 | 42 | 2 | 0 | 0 | 0 | 0 | |

## Overall Statistics :

|  |  |
| --- | --- |
| 95% CI | (0.53922,0.57552) |
| ACC Macro | 0.87353 |
| ARI | 0.23514 |
| AUNP | 0.69258 |
| AUNU | 0.61747 |
| Bangdiwala B | 0.42604 |
| Bennett S | 0.4836 |
| CBA | 0.26442 |
| CSI | None |
| Chi-Squared | None |
| Chi-Squared DF | 36 |
| Conditional Entropy | 1.23917 |
| Cramer V | None |
| Cross Entropy | 2.1651 |
| F1 Macro | 0.30891 |
| F1 Micro | 0.55737 |
| FNR Macro | 0.67723 |
| FNR Micro | 0.44263 |
| FPR Macro | 0.08783 |
| FPR Micro | 0.07377 |
| Gwet AC1 | 0.49769 |
| Hamming Loss | 0.44263 |
| Joint Entropy | 3.61378 |
| KL Divergence | None |
| Kappa | 0.38754 |
| Kappa 95% CI | (0.36242,0.41266) |
| Kappa No Prevalence | 0.11474 |
| Kappa Standard Error | 0.01282 |
| Kappa Unbiased | 0.37907 |
| Krippendorff Alpha | 0.37918 |
| Lambda A | 0.32493 |
| Lambda B | 0.33517 |
| Mutual Information | 0.44101 |
| NIR | 0.36544 |
| Overall ACC | 0.55737 |
| Overall CEN | 0.43653 |
| Overall J | (1.54242,0.22035) |
| Overall MCC | 0.40019 |
| Overall MCEN | 0.53719 |
| Overall RACC | 0.27729 |
| Overall RACCU | 0.28715 |
| P-Value | None |
| PPV Macro | None |
| PPV Micro | 0.55737 |
| Pearson C | None |
| Phi-Squared | None |
| RCI | 0.18572 |
| RR | 410.85714 |
| Reference Entropy | 2.37461 |
| Response Entropy | 1.68018 |
| SOA1(Landis & Koch) | Fair |
| SOA2(Fleiss) | Poor |
| SOA3(Altman) | Fair |
| SOA4(Cicchetti) | Poor |
| SOA5(Cramer) | None |
| SOA6(Matthews) | Weak |
| Scott PI | 0.37907 |
| Standard Error | 0.00926 |
| TNR Macro | 0.91217 |
| TNR Micro | 0.92623 |
| TPR Macro | 0.32277 |
| TPR Micro | 0.55737 |
| Zero-one Loss | 1273 |

## Class Statistics :

|  |  |  |  |  |  |  |  |  |
| --- | --- | --- | --- | --- | --- | --- | --- | --- |
| Class | 0 | 1 | 2 | 3 | 4 | 5 | 6 | Description |
| ACC | 0.75904 | 0.72392 | 0.8783 | 0.91725 | 0.91099 | 0.9541 | 0.97114 | Accuracy |
| AGF | 0.70968 | 0.77906 | 0.7753 | 0.07278 | 0.0 | 0.45487 | 0.0 | Adjusted F-score |
| AGM | 0.74413 | 0.7154 | 0.8477 | 0.51151 | 0 | 0.70382 | 0 | Adjusted geometric mean |
| AM | 267 | 372 | -68 | -190 | -256 | -98 | -27 | Difference between automatic and manual classification |
| AUC | 0.71103 | 0.73989 | 0.78942 | 0.49782 | 0.5 | 0.58909 | 0.49504 | Area under the ROC curve |
| AUCI | Good | Good | Good | Poor | Poor | Poor | Poor | AUC value interpretation |
| AUPR | 0.53106 | 0.69477 | 0.68271 | 0.02233 | None | 0.39452 | 0.0 | Area under the PR curve |
| BCD | 0.04642 | 0.06467 | 0.01182 | 0.03303 | 0.04451 | 0.01704 | 0.00469 | Bray-Curtis dissimilarity |
| BM | 0.42206 | 0.47979 | 0.57884 | -0.00437 | 0.0 | 0.17818 | -0.00993 | Informedness or bookmaker informedness |
| CEN | 0.50665 | 0.40119 | 0.38625 | 0.65574 | 0.35271 | 0.50253 | 0.43015 | Confusion entropy |
| DOR | 6.4761 | 8.48105 | 27.19088 | 0.51343 | None | 36.14731 | 0.0 | Diagnostic odds ratio |
| DP | 0.4473 | 0.51188 | 0.79084 | -0.15962 | None | 0.85901 | None | Discriminant power |
| DPI | Poor | Poor | Poor | Poor | None | Poor | None | Discriminant power interpretation |
| ERR | 0.24096 | 0.27608 | 0.1217 | 0.08275 | 0.08901 | 0.0459 | 0.02886 | Error rate |
| F0.5 | 0.46041 | 0.62287 | 0.70642 | 0.01587 | 0.0 | 0.41534 | 0.0 | F0.5 score |
| F1 | 0.51232 | 0.67906 | 0.68007 | 0.00833 | 0.0 | 0.28261 | 0.0 | F1 score - harmonic mean of precision and sensitivity |
| F2 | 0.57741 | 0.7464 | 0.65562 | 0.00565 | 0.0 | 0.21417 | 0.0 | F2 score |
| FDR | 0.56872 | 0.4097 | 0.27485 | 0.96 | None | 0.39535 | 1.0 | False discovery rate |
| FN | 213 | 211 | 209 | 214 | 256 | 115 | 55 | False negative/miss/type 2 error |
| FNR | 0.36915 | 0.20076 | 0.35972 | 0.99535 | 1.0 | 0.8156 | 1.0 | Miss rate or false negative rate |
| FOR | 0.10482 | 0.14522 | 0.08845 | 0.07506 | 0.08901 | 0.04059 | 0.01931 | False omission rate |
| FP | 480 | 583 | 141 | 24 | 0 | 17 | 28 | False positive/type 1 error/false alarm |
| FPR | 0.20879 | 0.31945 | 0.06144 | 0.00902 | 0.0 | 0.00622 | 0.00993 | Fall-out or false positive rate |
| G | 0.52161 | 0.68687 | 0.68139 | 0.01364 | None | 0.33391 | 0.0 | G-measure geometric mean of precision and sensitivity |
| GI | 0.42206 | 0.47979 | 0.57884 | -0.00437 | 0.0 | 0.17818 | -0.00993 | Gini index |
| GM | 0.7065 | 0.73751 | 0.7752 | 0.06789 | 0.0 | 0.42808 | 0.0 | G-mean geometric mean of specificity and sensitivity |
| IBA | 0.41909 | 0.60848 | 0.42169 | 6e-05 | 0.0 | 0.03493 | 0.0 | Index of balanced accuracy |
| ICSI | 0.06213 | 0.38954 | 0.36542 | -0.95535 | None | -0.21095 | -1.0 | Individual classification success index |
| IS | 1.10412 | 0.69183 | 1.8438 | -0.9022 | None | 3.62447 | None | Information score |
| J | 0.34437 | 0.51408 | 0.51524 | 0.00418 | 0.0 | 0.16456 | 0.0 | Jaccard index |
| LS | 2.14967 | 1.61533 | 3.58954 | 0.53507 | None | 12.33317 | 0.0 | Lift score |
| MCC | 0.37119 | 0.46211 | 0.60708 | -0.01238 | None | 0.31702 | -0.01384 | Matthews correlation coefficient |
| MCCI | Weak | Weak | Moderate | Negligible | None | Weak | Negligible | Matthews correlation coefficient interpretation |
| MCEN | 0.60305 | 0.52632 | 0.50419 | 0.65681 | 0.35271 | 0.53401 | 0.43015 | Modified confusion entropy |
| MK | 0.32646 | 0.44509 | 0.6367 | -0.03506 | None | 0.56406 | -0.01931 | Markedness |
| N | 2299 | 1825 | 2295 | 2661 | 2620 | 2735 | 2821 | Condition negative |
| NLR | 0.46656 | 0.295 | 0.38327 | 1.00441 | 1.0 | 0.8207 | 1.01003 | Negative likelihood ratio |
| NLRI | Poor | Poor | Poor | Negligible | Negligible | Negligible | Negligible | Negative likelihood ratio interpretation |
| NPV | 0.89518 | 0.85478 | 0.91155 | 0.92494 | 0.91099 | 0.95941 | 0.98069 | Negative predictive value |
| OC | 0.63085 | 0.79924 | 0.72515 | 0.04 | None | 0.60465 | 0.0 | Overlap coefficient |
| OOC | 0.52161 | 0.68687 | 0.68139 | 0.01364 | None | 0.33391 | 0.0 | Otsuka-Ochiai coefficient |
| OP | 0.64627 | 0.64371 | 0.68938 | -0.07341 | -0.08901 | 0.26712 | -0.02886 | Optimized precision |
| P | 577 | 1051 | 581 | 215 | 256 | 141 | 55 | Condition positive or support |
| PLR | 3.0215 | 2.50191 | 10.4215 | 0.5157 | None | 29.66625 | 0.0 | Positive likelihood ratio |
| PLRI | Poor | Poor | Good | Negligible | None | Good | Negligible | Positive likelihood ratio interpretation |
| POP | 2876 | 2876 | 2876 | 2876 | 2876 | 2876 | 2876 | Population |
| PPV | 0.43128 | 0.5903 | 0.72515 | 0.04 | None | 0.60465 | 0.0 | Precision or positive predictive value |
| PRE | 0.20063 | 0.36544 | 0.20202 | 0.07476 | 0.08901 | 0.04903 | 0.01912 | Prevalence |
| Q | 0.73248 | 0.78905 | 0.92906 | -0.3215 | None | 0.94616 | -1.0 | Yule Q - coefficient of colligation |
| QI | Moderate | Strong | Strong | Negligible | None | Strong | Negligible | Yule Q interpretation |
| RACC | 0.05888 | 0.18081 | 0.03603 | 0.00065 | 0.0 | 0.00073 | 0.00019 | Random accuracy |
| RACCU | 0.06103 | 0.185 | 0.03617 | 0.00174 | 0.00198 | 0.00102 | 0.00021 | Random accuracy unbiased |
| TN | 1819 | 1242 | 2154 | 2637 | 2620 | 2718 | 2793 | True negative/correct rejection |
| TNR | 0.79121 | 0.68055 | 0.93856 | 0.99098 | 1.0 | 0.99378 | 0.99007 | Specificity or true negative rate |
| TON | 2032 | 1453 | 2363 | 2851 | 2876 | 2833 | 2848 | Test outcome negative |
| TOP | 844 | 1423 | 513 | 25 | 0 | 43 | 28 | Test outcome positive |
| TP | 364 | 840 | 372 | 1 | 0 | 26 | 0 | True positive/hit |
| TPR | 0.63085 | 0.79924 | 0.64028 | 0.00465 | 0.0 | 0.1844 | 0.0 | Sensitivity, recall, hit rate, or true positive rate |
| Y | 0.42206 | 0.47979 | 0.57884 | -0.00437 | 0.0 | 0.17818 | -0.00993 | Youden index |
| dInd | 0.4241 | 0.3773 | 0.36493 | 0.99539 | 1.0 | 0.81563 | 1.00005 | Distance index |
| sInd | 0.70011 | 0.73321 | 0.74195 | 0.29615 | 0.29289 | 0.42326 | 0.29286 | Similarity index |

Generated By PyCM Version 3.1
