# Supplementary material for: TopEC: prediction of Enzyme Commission classes by 3D graph neural networks and localized 3D protein descriptor
Source: Nat Commun. 2025 Mar 20;16:2737. doi: 10.1038/s41467-025-57324-5 (PMC11923149; doi:10.1038/s41467-025-57324-5)
Supplement: Supplementary file 3 — Supplementary Data 1 [file 41467_2025_57324_MOESM3_ESM.zip › Data_S1/table1/mainclass/DeepFRI/local/BindingMOAD_TEMP.html]

DeepFRI\_PDB\_TEMP\_sites


# PyCM Report

## Dataset Type :

- Multi-Class Classification
- Imbalanced

Note 1 : Recommended statistics for this type of classification highlighted in aqua

Note 2 : The recommender system assumes that the input is the result of classification over the whole data rather than just a part of it.
If the confusion matrix is the result of test data classification, the recommendation is not valid.

## Confusion Matrix :

|  |  |  |  |  |  |  |  |  |  |  |  |  |  |  |  |  |  |  |  |  |  |  |  |  |  |  |  |  |  |  |  |  |  |  |  |  |  |  |  |  |  |  |  |  |  |  |  |  |  |  |  |  |  |  |  |  |  |  |  |  |  |  |  |  |  |
| --- | --- | --- | --- | --- | --- | --- | --- | --- | --- | --- | --- | --- | --- | --- | --- | --- | --- | --- | --- | --- | --- | --- | --- | --- | --- | --- | --- | --- | --- | --- | --- | --- | --- | --- | --- | --- | --- | --- | --- | --- | --- | --- | --- | --- | --- | --- | --- | --- | --- | --- | --- | --- | --- | --- | --- | --- | --- | --- | --- | --- | --- | --- | --- | --- | --- |
| Actual | Predict  |  |  |  |  |  |  |  |  | | --- | --- | --- | --- | --- | --- | --- | --- | |  | 0 | 1 | 2 | 3 | 4 | 5 | 6 | | 0 | 250 | 94 | 66 | 12 | 0 | 1 | 0 | | 1 | 170 | 504 | 66 | 4 | 4 | 6 | 0 | | 2 | 124 | 120 | 417 | 5 | 0 | 1 | 3 | | 3 | 33 | 21 | 21 | 49 | 0 | 0 | 1 | | 4 | 22 | 13 | 3 | 0 | 30 | 0 | 9 | | 5 | 10 | 30 | 11 | 0 | 0 | 7 | 0 | | 6 | 7 | 28 | 30 | 1 | 0 | 5 | 5 | |

## Overall Statistics :

|  |  |
| --- | --- |
| 95% CI | (0.55739,0.59882) |
| ACC Macro | 0.87946 |
| ARI | 0.2187 |
| AUNP | 0.71141 |
| AUNU | 0.66234 |
| Bangdiwala B | 0.38079 |
| Bennett S | 0.50779 |
| CBA | 0.37408 |
| CSI | -0.03465 |
| Chi-Squared | 2427.79834 |
| Chi-Squared DF | 36 |
| Conditional Entropy | 1.48357 |
| Cramer V | 0.43053 |
| Cross Entropy | 2.32504 |
| F1 Macro | 0.4431 |
| F1 Micro | 0.5781 |
| FNR Macro | 0.59287 |
| FNR Micro | 0.4219 |
| FPR Macro | 0.08245 |
| FPR Micro | 0.07032 |
| Gwet AC1 | 0.52011 |
| Hamming Loss | 0.4219 |
| Joint Entropy | 3.70926 |
| KL Divergence | 0.09935 |
| Kappa | 0.42039 |
| Kappa 95% CI | (0.39193,0.44885) |
| Kappa No Prevalence | 0.15621 |
| Kappa Standard Error | 0.01452 |
| Kappa Unbiased | 0.41816 |
| Krippendorff Alpha | 0.4183 |
| Lambda A | 0.35829 |
| Lambda B | 0.36417 |
| Mutual Information | 0.4503 |
| NIR | 0.3454 |
| Overall ACC | 0.5781 |
| Overall CEN | 0.44456 |
| Overall J | (2.13177,0.30454) |
| Overall MCC | 0.4238 |
| Overall MCEN | 0.54801 |
| Overall RACC | 0.2721 |
| Overall RACCU | 0.27489 |
| P-Value | None |
| PPV Macro | 0.55821 |
| PPV Micro | 0.5781 |
| Pearson C | 0.72564 |
| Phi-Squared | 1.11214 |
| RCI | 0.20232 |
| RR | 311.85714 |
| Reference Entropy | 2.22569 |
| Response Entropy | 1.93387 |
| SOA1(Landis & Koch) | Moderate |
| SOA2(Fleiss) | Intermediate to Good |
| SOA3(Altman) | Moderate |
| SOA4(Cicchetti) | Fair |
| SOA5(Cramer) | Relatively Strong |
| SOA6(Matthews) | Weak |
| Scott PI | 0.41816 |
| Standard Error | 0.01057 |
| TNR Macro | 0.91755 |
| TNR Micro | 0.92968 |
| TPR Macro | 0.40713 |
| TPR Micro | 0.5781 |
| Zero-one Loss | 921 |

## Class Statistics :

|  |  |  |  |  |  |  |  |  |
| --- | --- | --- | --- | --- | --- | --- | --- | --- |
| Class | 0 | 1 | 2 | 3 | 4 | 5 | 6 | Description |
| ACC | 0.75309 | 0.7453 | 0.79386 | 0.95511 | 0.97664 | 0.97068 | 0.96152 | Accuracy |
| AGF | 0.68572 | 0.731 | 0.73125 | 0.64479 | 0.6563 | 0.36891 | 0.27477 | Adjusted F-score |
| AGM | 0.73233 | 0.74894 | 0.79063 | 0.80062 | 0.80749 | 0.66575 | 0.61823 | Adjusted geometric mean |
| AM | 193 | 56 | -56 | -54 | -43 | -38 | -58 | Difference between automatic and manual classification |
| AUC | 0.69153 | 0.72715 | 0.74609 | 0.69066 | 0.69386 | 0.55729 | 0.52981 | Area under the ROC curve |
| AUCI | Fair | Good | Good | Fair | Fair | Poor | Poor | AUC value interpretation |
| AUPR | 0.49843 | 0.64533 | 0.65077 | 0.54107 | 0.63598 | 0.23534 | 0.17178 | Area under the PR curve |
| BCD | 0.04421 | 0.01283 | 0.01283 | 0.01237 | 0.00985 | 0.0087 | 0.01328 | Bray-Curtis dissimilarity |
| BM | 0.38306 | 0.4543 | 0.49218 | 0.38131 | 0.38771 | 0.11457 | 0.05962 | Informedness or bookmaker informedness |
| CEN | 0.51114 | 0.4178 | 0.40392 | 0.47345 | 0.39961 | 0.56027 | 0.60604 | Confusion entropy |
| DOR | 5.50396 | 7.39859 | 11.01045 | 59.66746 | 335.42553 | 22.29864 | 11.34345 | Diagnostic odds ratio |
| DP | 0.40836 | 0.47919 | 0.57438 | 0.97902 | 1.39243 | 0.74334 | 0.58151 | Discriminant power |
| DPI | Poor | Poor | Poor | Poor | Limited | Poor | Poor | Discriminant power interpretation |
| ERR | 0.24691 | 0.2547 | 0.20614 | 0.04489 | 0.02336 | 0.02932 | 0.03848 | Error rate |
| F0.5 | 0.43298 | 0.63095 | 0.66699 | 0.59902 | 0.70423 | 0.25362 | 0.16892 | F0.5 score |
| F1 | 0.48123 | 0.6445 | 0.64953 | 0.5 | 0.54054 | 0.17949 | 0.10638 | F1 score - harmonic mean of precision and sensitivity |
| F2 | 0.54159 | 0.65865 | 0.63297 | 0.42907 | 0.4386 | 0.13889 | 0.07764 | F2 score |
| FDR | 0.59416 | 0.37778 | 0.32085 | 0.30986 | 0.11765 | 0.65 | 0.72222 | False discovery rate |
| FN | 173 | 250 | 253 | 76 | 47 | 51 | 71 | False negative/miss/type 2 error |
| FNR | 0.40898 | 0.33156 | 0.37761 | 0.608 | 0.61039 | 0.87931 | 0.93421 | Miss rate or false negative rate |
| FOR | 0.1104 | 0.18208 | 0.16125 | 0.03598 | 0.02187 | 0.02358 | 0.03279 | False omission rate |
| FP | 366 | 306 | 197 | 22 | 4 | 13 | 13 | False positive/type 1 error/false alarm |
| FPR | 0.20795 | 0.21414 | 0.1302 | 0.01069 | 0.0019 | 0.00612 | 0.00617 | Fall-out or false positive rate |
| G | 0.48976 | 0.64491 | 0.65015 | 0.52013 | 0.58632 | 0.20553 | 0.13518 | G-measure geometric mean of precision and sensitivity |
| GI | 0.38306 | 0.4543 | 0.49218 | 0.38131 | 0.38771 | 0.11457 | 0.05962 | Gini index |
| GM | 0.68419 | 0.72478 | 0.73576 | 0.62274 | 0.62359 | 0.34634 | 0.2557 | G-mean geometric mean of specificity and sensitivity |
| IBA | 0.37401 | 0.46361 | 0.40742 | 0.15617 | 0.15225 | 0.01521 | 0.0047 | Index of balanced accuracy |
| ICSI | -0.00314 | 0.29066 | 0.30154 | 0.08214 | 0.27196 | -0.52931 | -0.65643 | Individual classification success index |
| IS | 1.06658 | 0.84918 | 1.14589 | 3.59127 | 4.64474 | 3.71954 | 2.99617 | Information score |
| J | 0.31686 | 0.47547 | 0.48097 | 0.33333 | 0.37037 | 0.09859 | 0.05618 | Jaccard index |
| LS | 2.09446 | 1.80147 | 2.21282 | 12.05262 | 25.01528 | 13.17328 | 7.9788 | Lift score |
| MCC | 0.33641 | 0.44716 | 0.50488 | 0.49944 | 0.5776 | 0.19339 | 0.12085 | Matthews correlation coefficient |
| MCCI | Weak | Weak | Moderate | Weak | Moderate | Negligible | Negligible | Matthews correlation coefficient interpretation |
| MCEN | 0.59742 | 0.53435 | 0.51616 | 0.55408 | 0.46778 | 0.5814 | 0.61933 | Modified confusion entropy |
| MK | 0.29544 | 0.44014 | 0.5179 | 0.65416 | 0.86048 | 0.32642 | 0.24498 | Markedness |
| N | 1760 | 1429 | 1513 | 2058 | 2106 | 2125 | 2107 | Condition negative |
| NLR | 0.51636 | 0.42191 | 0.43414 | 0.61457 | 0.61155 | 0.88472 | 0.94001 | Negative likelihood ratio |
| NLRI | Negligible | Poor | Poor | Negligible | Negligible | Negligible | Negligible | Negative likelihood ratio interpretation |
| NPV | 0.8896 | 0.81792 | 0.83875 | 0.96402 | 0.97813 | 0.97642 | 0.96721 | Negative predictive value |
| OC | 0.59102 | 0.66844 | 0.67915 | 0.69014 | 0.88235 | 0.35 | 0.27778 | Overlap coefficient |
| OOC | 0.48976 | 0.64491 | 0.65015 | 0.52013 | 0.58632 | 0.20553 | 0.13518 | Otsuka-Ochiai coefficient |
| OP | 0.60774 | 0.66456 | 0.62806 | 0.52268 | 0.53815 | 0.18725 | 0.0857 | Optimized precision |
| P | 423 | 754 | 670 | 125 | 77 | 58 | 76 | Condition positive or support |
| PLR | 2.84205 | 3.12155 | 4.78007 | 36.66982 | 205.12987 | 19.72812 | 10.66296 | Positive likelihood ratio |
| PLRI | Poor | Poor | Poor | Good | Good | Good | Good | Positive likelihood ratio interpretation |
| POP | 2183 | 2183 | 2183 | 2183 | 2183 | 2183 | 2183 | Population |
| PPV | 0.40584 | 0.62222 | 0.67915 | 0.69014 | 0.88235 | 0.35 | 0.27778 | Precision or positive predictive value |
| PRE | 0.19377 | 0.3454 | 0.30692 | 0.05726 | 0.03527 | 0.02657 | 0.03481 | Prevalence |
| Q | 0.6925 | 0.76186 | 0.83348 | 0.96703 | 0.99406 | 0.91416 | 0.83797 | Yule Q - coefficient of colligation |
| QI | Moderate | Strong | Strong | Strong | Strong | Strong | Strong | Yule Q interpretation |
| RACC | 0.05468 | 0.12816 | 0.08632 | 0.00186 | 0.00055 | 0.00024 | 0.00029 | Random accuracy |
| RACCU | 0.05663 | 0.12832 | 0.08649 | 0.00202 | 0.00065 | 0.00032 | 0.00046 | Random accuracy unbiased |
| TN | 1394 | 1123 | 1316 | 2036 | 2102 | 2112 | 2094 | True negative/correct rejection |
| TNR | 0.79205 | 0.78586 | 0.8698 | 0.98931 | 0.9981 | 0.99388 | 0.99383 | Specificity or true negative rate |
| TON | 1567 | 1373 | 1569 | 2112 | 2149 | 2163 | 2165 | Test outcome negative |
| TOP | 616 | 810 | 614 | 71 | 34 | 20 | 18 | Test outcome positive |
| TP | 250 | 504 | 417 | 49 | 30 | 7 | 5 | True positive/hit |
| TPR | 0.59102 | 0.66844 | 0.62239 | 0.392 | 0.38961 | 0.12069 | 0.06579 | Sensitivity, recall, hit rate, or true positive rate |
| Y | 0.38306 | 0.4543 | 0.49218 | 0.38131 | 0.38771 | 0.11457 | 0.05962 | Youden index |
| dInd | 0.45882 | 0.3947 | 0.39943 | 0.60809 | 0.61039 | 0.87933 | 0.93423 | Distance index |
| sInd | 0.67557 | 0.7209 | 0.71756 | 0.57001 | 0.56839 | 0.37822 | 0.3394 | Similarity index |

Generated By PyCM Version 3.1
