# Supplementary material for: TopEC: prediction of Enzyme Commission classes by 3D graph neural networks and localized 3D protein descriptor
Source: Nat Commun. 2025 Mar 20;16:2737. doi: 10.1038/s41467-025-57324-5 (PMC11923149; doi:10.1038/s41467-025-57324-5)
Supplement: Supplementary file 3 — Supplementary Data 1 [file 41467_2025_57324_MOESM3_ESM.zip › Data_S1/table1/mainclass/DeepFRI/local/BindingMOAD_FOLD.html]

DeepFRI\_PDB\_FOLD\_sites


# PyCM Report

## Dataset Type :

- Multi-Class Classification
- Imbalanced

Note 1 : Recommended statistics for this type of classification highlighted in aqua

Note 2 : The recommender system assumes that the input is the result of classification over the whole data rather than just a part of it.
If the confusion matrix is the result of test data classification, the recommendation is not valid.

## Confusion Matrix :

|  |  |  |  |  |  |  |  |  |  |  |  |  |  |  |  |  |  |  |  |  |  |  |  |  |  |  |  |  |  |  |  |  |  |  |  |  |  |  |  |  |  |  |  |  |  |  |  |  |  |  |  |  |  |  |  |  |  |  |  |  |  |  |  |  |  |
| --- | --- | --- | --- | --- | --- | --- | --- | --- | --- | --- | --- | --- | --- | --- | --- | --- | --- | --- | --- | --- | --- | --- | --- | --- | --- | --- | --- | --- | --- | --- | --- | --- | --- | --- | --- | --- | --- | --- | --- | --- | --- | --- | --- | --- | --- | --- | --- | --- | --- | --- | --- | --- | --- | --- | --- | --- | --- | --- | --- | --- | --- | --- | --- | --- | --- |
| Actual | Predict  |  |  |  |  |  |  |  |  | | --- | --- | --- | --- | --- | --- | --- | --- | |  | 0 | 1 | 2 | 3 | 4 | 5 | 6 | | 0 | 180 | 173 | 31 | 0 | 0 | 0 | 23 | | 1 | 76 | 681 | 47 | 0 | 0 | 0 | 0 | | 2 | 58 | 72 | 272 | 0 | 0 | 0 | 0 | | 3 | 27 | 87 | 18 | 0 | 0 | 0 | 0 | | 4 | 83 | 55 | 28 | 5 | 0 | 0 | 0 | | 5 | 8 | 20 | 14 | 0 | 0 | 0 | 0 | | 6 | 1 | 15 | 1 | 0 | 0 | 0 | 0 | |

## Overall Statistics :

|  |  |
| --- | --- |
| 95% CI | (0.55186,0.59548) |
| ACC Macro | 0.87819 |
| ARI | 0.25435 |
| AUNP | 0.68772 |
| AUNU | 0.59581 |
| Bangdiwala B | 0.46379 |
| Bennett S | 0.50262 |
| CBA | 0.24213 |
| CSI | None |
| Chi-Squared | None |
| Chi-Squared DF | 36 |
| Conditional Entropy | 1.14368 |
| Cramer V | None |
| Cross Entropy | 1.88606 |
| F1 Macro | 0.25884 |
| F1 Micro | 0.57367 |
| FNR Macro | 0.71916 |
| FNR Micro | 0.42633 |
| FPR Macro | 0.08922 |
| FPR Micro | 0.07105 |
| Gwet AC1 | 0.51954 |
| Hamming Loss | 0.42633 |
| Joint Entropy | 3.35223 |
| KL Divergence | None |
| Kappa | 0.37748 |
| Kappa 95% CI | (0.34563,0.40933) |
| Kappa No Prevalence | 0.14734 |
| Kappa Standard Error | 0.01625 |
| Kappa Unbiased | 0.36937 |
| Krippendorff Alpha | 0.36953 |
| Lambda A | 0.30487 |
| Lambda B | 0.2695 |
| Mutual Information | 0.37361 |
| NIR | 0.40709 |
| Overall ACC | 0.57367 |
| Overall CEN | 0.40929 |
| Overall J | (1.33096,0.19014) |
| Overall MCC | 0.38963 |
| Overall MCEN | 0.50732 |
| Overall RACC | 0.31516 |
| Overall RACCU | 0.32396 |
| P-Value | None |
| PPV Macro | None |
| PPV Micro | 0.57367 |
| Pearson C | None |
| Phi-Squared | None |
| RCI | 0.16917 |
| RR | 282.14286 |
| Reference Entropy | 2.20856 |
| Response Entropy | 1.51729 |
| SOA1(Landis & Koch) | Fair |
| SOA2(Fleiss) | Poor |
| SOA3(Altman) | Fair |
| SOA4(Cicchetti) | Poor |
| SOA5(Cramer) | None |
| SOA6(Matthews) | Weak |
| Scott PI | 0.36937 |
| Standard Error | 0.01113 |
| TNR Macro | 0.91078 |
| TNR Micro | 0.92895 |
| TPR Macro | 0.28084 |
| TPR Micro | 0.57367 |
| Zero-one Loss | 842 |

## Class Statistics :

|  |  |  |  |  |  |  |  |  |
| --- | --- | --- | --- | --- | --- | --- | --- | --- |
| Class | 0 | 1 | 2 | 3 | 4 | 5 | 6 | Description |
| ACC | 0.75696 | 0.72405 | 0.8638 | 0.93063 | 0.91342 | 0.97873 | 0.97975 | Accuracy |
| AGF | 0.60922 | 0.79606 | 0.78543 | 0.0 | 0.0 | 0.0 | 0.0 | Adjusted F-score |
| AGM | 0.71064 | 0.70016 | 0.84136 | 0 | 0 | 0 | 0 | Adjusted geometric mean |
| AM | 26 | 299 | 9 | -127 | -171 | -42 | 6 | Difference between automatic and manual classification |
| AUC | 0.64045 | 0.74332 | 0.79413 | 0.49864 | 0.5 | 0.5 | 0.49413 | Area under the ROC curve |
| AUCI | Fair | Good | Good | Poor | Poor | Poor | Poor | AUC value interpretation |
| AUPR | 0.42898 | 0.73221 | 0.66921 | 0.0 | None | None | 0.0 | Area under the PR curve |
| BCD | 0.00658 | 0.0757 | 0.00228 | 0.03215 | 0.04329 | 0.01063 | 0.00152 | Bray-Curtis dissimilarity |
| BM | 0.28091 | 0.48664 | 0.58825 | -0.00271 | 0.0 | 0.0 | -0.01175 | Informedness or bookmaker informedness |
| CEN | 0.53893 | 0.35823 | 0.39074 | 0.40079 | 0.44881 | 0.41666 | 0.3503 | Confusion entropy |
| DOR | 4.12147 | 9.82678 | 21.58539 | 0.0 | None | None | 0.0 | Diagnostic odds ratio |
| DP | 0.3391 | 0.54715 | 0.73556 | None | None | None | None | Discriminant power |
| DPI | Poor | Poor | Poor | None | None | None | None | Discriminant power interpretation |
| ERR | 0.24304 | 0.27595 | 0.1362 | 0.06937 | 0.08658 | 0.02127 | 0.02025 | Error rate |
| F0.5 | 0.42076 | 0.6528 | 0.66471 | 0.0 | 0.0 | 0.0 | 0.0 | F0.5 score |
| F1 | 0.42857 | 0.71421 | 0.66913 | 0.0 | 0.0 | 0.0 | 0.0 | F1 score - harmonic mean of precision and sensitivity |
| F2 | 0.43668 | 0.78838 | 0.6736 | 0.0 | 0.0 | 0.0 | 0.0 | F2 score |
| FDR | 0.5843 | 0.38259 | 0.3382 | 1.0 | None | None | 1.0 | False discovery rate |
| FN | 227 | 123 | 130 | 132 | 171 | 42 | 17 | False negative/miss/type 2 error |
| FNR | 0.55774 | 0.15299 | 0.32338 | 1.0 | 1.0 | 1.0 | 1.0 | Miss rate or false negative rate |
| FOR | 0.14721 | 0.14106 | 0.08312 | 0.06701 | 0.08658 | 0.02127 | 0.00871 | False omission rate |
| FP | 253 | 422 | 139 | 5 | 0 | 0 | 23 | False positive/type 1 error/false alarm |
| FPR | 0.16135 | 0.36038 | 0.08837 | 0.00271 | 0.0 | 0.0 | 0.01175 | Fall-out or false positive rate |
| G | 0.42878 | 0.72315 | 0.66917 | 0.0 | None | None | 0.0 | G-measure geometric mean of precision and sensitivity |
| GI | 0.28091 | 0.48664 | 0.58825 | -0.00271 | 0.0 | 0.0 | -0.01175 | Gini index |
| GM | 0.60902 | 0.73605 | 0.78538 | 0.0 | 0.0 | 0.0 | 0.0 | G-mean geometric mean of specificity and sensitivity |
| IBA | 0.22388 | 0.65413 | 0.47186 | 0.0 | 0.0 | 0.0 | 0.0 | Index of balanced accuracy |
| ICSI | -0.14204 | 0.46442 | 0.33842 | -1.0 | None | None | -1.0 | Individual classification success index |
| IS | 1.01238 | 0.60088 | 1.70105 | None | None | None | None | Information score |
| J | 0.27273 | 0.55546 | 0.50277 | 0.0 | 0.0 | 0.0 | 0.0 | Jaccard index |
| LS | 2.01724 | 1.51664 | 3.25138 | 0.0 | None | None | 0.0 | Lift score |
| MCC | 0.27463 | 0.48147 | 0.58345 | -0.01348 | None | None | -0.01011 | Matthews correlation coefficient |
| MCCI | Negligible | Weak | Moderate | Negligible | None | None | Negligible | Matthews correlation coefficient interpretation |
| MCEN | 0.61533 | 0.47818 | 0.50569 | 0.40079 | 0.44881 | 0.41666 | 0.3503 | Modified confusion entropy |
| MK | 0.26849 | 0.47635 | 0.57868 | -0.06701 | None | None | -0.00871 | Markedness |
| N | 1568 | 1171 | 1573 | 1843 | 1804 | 1933 | 1958 | Condition negative |
| NLR | 0.66505 | 0.23918 | 0.35473 | 1.00272 | 1.0 | 1.0 | 1.01189 | Negative likelihood ratio |
| NLRI | Negligible | Poor | Poor | Negligible | Negligible | Negligible | Negligible | Negative likelihood ratio interpretation |
| NPV | 0.85279 | 0.85894 | 0.91688 | 0.93299 | 0.91342 | 0.97873 | 0.99129 | Negative predictive value |
| OC | 0.44226 | 0.84701 | 0.67662 | 0.0 | None | None | 0.0 | Overlap coefficient |
| OOC | 0.42878 | 0.72315 | 0.66917 | 0.0 | None | None | 0.0 | Otsuka-Ochiai coefficient |
| OP | 0.4475 | 0.58455 | 0.71583 | -0.06937 | -0.08658 | -0.02127 | -0.02025 | Optimized precision |
| P | 407 | 804 | 402 | 132 | 171 | 42 | 17 | Condition positive or support |
| PLR | 2.74097 | 2.35037 | 7.65697 | 0.0 | None | None | 0.0 | Positive likelihood ratio |
| PLRI | Poor | Poor | Fair | Negligible | None | None | Negligible | Positive likelihood ratio interpretation |
| POP | 1975 | 1975 | 1975 | 1975 | 1975 | 1975 | 1975 | Population |
| PPV | 0.4157 | 0.61741 | 0.6618 | 0.0 | None | None | 0.0 | Precision or positive predictive value |
| PRE | 0.20608 | 0.40709 | 0.20354 | 0.06684 | 0.08658 | 0.02127 | 0.00861 | Prevalence |
| Q | 0.60949 | 0.81527 | 0.91145 | -1.0 | None | None | -1.0 | Yule Q - coefficient of colligation |
| QI | Moderate | Strong | Strong | Negligible | None | None | Negligible | Yule Q interpretation |
| RACC | 0.04518 | 0.22735 | 0.04236 | 0.00017 | 0.0 | 0.0 | 0.0001 | Random accuracy |
| RACCU | 0.04522 | 0.23308 | 0.04236 | 0.0012 | 0.00187 | 0.00011 | 0.0001 | Random accuracy unbiased |
| TN | 1315 | 749 | 1434 | 1838 | 1804 | 1933 | 1935 | True negative/correct rejection |
| TNR | 0.83865 | 0.63962 | 0.91163 | 0.99729 | 1.0 | 1.0 | 0.98825 | Specificity or true negative rate |
| TON | 1542 | 872 | 1564 | 1970 | 1975 | 1975 | 1952 | Test outcome negative |
| TOP | 433 | 1103 | 411 | 5 | 0 | 0 | 23 | Test outcome positive |
| TP | 180 | 681 | 272 | 0 | 0 | 0 | 0 | True positive/hit |
| TPR | 0.44226 | 0.84701 | 0.67662 | 0.0 | 0.0 | 0.0 | 0.0 | Sensitivity, recall, hit rate, or true positive rate |
| Y | 0.28091 | 0.48664 | 0.58825 | -0.00271 | 0.0 | 0.0 | -0.01175 | Youden index |
| dInd | 0.58061 | 0.3915 | 0.33524 | 1.0 | 1.0 | 1.0 | 1.00007 | Distance index |
| sInd | 0.58945 | 0.72317 | 0.76295 | 0.29289 | 0.29289 | 0.29289 | 0.29284 | Similarity index |

Generated By PyCM Version 3.1
