# Supplementary material for: TopEC: prediction of Enzyme Commission classes by 3D graph neural networks and localized 3D protein descriptor
Source: Nat Commun. 2025 Mar 20;16:2737. doi: 10.1038/s41467-025-57324-5 (PMC11923149; doi:10.1038/s41467-025-57324-5)
Supplement: Supplementary file 3 — Supplementary Data 1 [file 41467_2025_57324_MOESM3_ESM.zip › Data_S1/table1/mainclass/DeepFRI/full_struc/TopEnzyme_TEMP.html]

DeepFRI\_TopM\_TEMP


# PyCM Report

## Dataset Type :

- Multi-Class Classification
- Imbalanced

Note 1 : Recommended statistics for this type of classification highlighted in aqua

Note 2 : The recommender system assumes that the input is the result of classification over the whole data rather than just a part of it.
If the confusion matrix is the result of test data classification, the recommendation is not valid.

## Confusion Matrix :

|  |  |  |  |  |  |  |  |  |  |  |  |  |  |  |  |  |  |  |  |  |  |  |  |  |  |  |  |  |  |  |  |  |  |  |  |  |  |  |  |  |  |  |  |  |  |  |  |  |  |  |  |  |  |  |  |  |  |  |  |  |  |  |  |  |  |
| --- | --- | --- | --- | --- | --- | --- | --- | --- | --- | --- | --- | --- | --- | --- | --- | --- | --- | --- | --- | --- | --- | --- | --- | --- | --- | --- | --- | --- | --- | --- | --- | --- | --- | --- | --- | --- | --- | --- | --- | --- | --- | --- | --- | --- | --- | --- | --- | --- | --- | --- | --- | --- | --- | --- | --- | --- | --- | --- | --- | --- | --- | --- | --- | --- | --- |
| Actual | Predict  |  |  |  |  |  |  |  |  | | --- | --- | --- | --- | --- | --- | --- | --- | |  | 0 | 1 | 2 | 3 | 4 | 5 | 6 | | 0 | 59 | 96 | 52 | 7 | 0 | 0 | 4 | | 1 | 33 | 152 | 34 | 2 | 0 | 1 | 10 | | 2 | 13 | 95 | 97 | 4 | 0 | 0 | 6 | | 3 | 9 | 40 | 21 | 2 | 0 | 1 | 0 | | 4 | 3 | 19 | 5 | 5 | 0 | 0 | 0 | | 5 | 4 | 26 | 0 | 0 | 0 | 1 | 0 | | 6 | 15 | 29 | 4 | 1 | 0 | 0 | 43 | |

## Overall Statistics :

|  |  |
| --- | --- |
| 95% CI | (0.36433,0.4285) |
| ACC Macro | 0.82755 |
| ARI | 0.05223 |
| AUNP | 0.60148 |
| AUNU | 0.57907 |
| Bangdiwala B | 0.20037 |
| Bennett S | 0.29582 |
| CBA | 0.22592 |
| CSI | None |
| Chi-Squared | None |
| Chi-Squared DF | 36 |
| Conditional Entropy | 1.58682 |
| Cramer V | None |
| Cross Entropy | 2.53336 |
| F1 Macro | 0.26915 |
| F1 Micro | 0.39642 |
| FNR Macro | 0.728 |
| FNR Micro | 0.60358 |
| FPR Macro | 0.11386 |
| FPR Micro | 0.1006 |
| Gwet AC1 | 0.31105 |
| Hamming Loss | 0.60358 |
| Joint Entropy | 4.05677 |
| KL Divergence | None |
| Kappa | 0.20907 |
| Kappa 95% CI | (0.16703,0.25111) |
| Kappa No Prevalence | -0.20717 |
| Kappa Standard Error | 0.02145 |
| Kappa Unbiased | 0.18816 |
| Krippendorff Alpha | 0.18861 |
| Lambda A | 0.19213 |
| Lambda B | 0.0367 |
| Mutual Information | 0.23918 |
| NIR | 0.2598 |
| Overall ACC | 0.39642 |
| Overall CEN | 0.54277 |
| Overall J | (1.21208,0.17315) |
| Overall MCC | 0.22151 |
| Overall MCEN | 0.61387 |
| Overall RACC | 0.23687 |
| Overall RACCU | 0.25652 |
| P-Value | 0.0 |
| PPV Macro | None |
| PPV Micro | 0.39642 |
| Pearson C | None |
| Phi-Squared | None |
| RCI | 0.09684 |
| RR | 127.57143 |
| Reference Entropy | 2.46995 |
| Response Entropy | 1.826 |
| SOA1(Landis & Koch) | Fair |
| SOA2(Fleiss) | Poor |
| SOA3(Altman) | Fair |
| SOA4(Cicchetti) | Poor |
| SOA5(Cramer) | None |
| SOA6(Matthews) | Negligible |
| Scott PI | 0.18816 |
| Standard Error | 0.01637 |
| TNR Macro | 0.88614 |
| TNR Micro | 0.8994 |
| TPR Macro | 0.272 |
| TPR Micro | 0.39642 |
| Zero-one Loss | 539 |

## Class Statistics :

|  |  |  |  |  |  |  |  |  |
| --- | --- | --- | --- | --- | --- | --- | --- | --- |
| Class | 0 | 1 | 2 | 3 | 4 | 5 | 6 | Description |
| ACC | 0.73572 | 0.56887 | 0.73796 | 0.89922 | 0.96417 | 0.96417 | 0.92273 | Accuracy |
| AGF | 0.48612 | 0.63729 | 0.61138 | 0.17234 | 0.0 | 0.19566 | 0.68753 | Adjusted F-score |
| AGM | 0.66025 | 0.57044 | 0.70535 | 0.55288 | 0 | 0.58131 | 0.81691 | Adjusted geometric mean |
| AM | -82 | 225 | -2 | -52 | -32 | -28 | -29 | Difference between automatic and manual classification |
| AUC | 0.57828 | 0.59688 | 0.64004 | 0.50211 | 0.5 | 0.51497 | 0.72121 | Area under the ROC curve |
| AUCI | Poor | Poor | Fair | Poor | Poor | Poor | Good | AUC value interpretation |
| AUPR | 0.35223 | 0.49389 | 0.45328 | 0.06132 | None | 0.1828 | 0.57497 | Area under the PR curve |
| BCD | 0.04591 | 0.12598 | 0.00112 | 0.02912 | 0.01792 | 0.01568 | 0.01624 | Bray-Curtis dissimilarity |
| BM | 0.15657 | 0.19375 | 0.28007 | 0.00423 | 0.0 | 0.02994 | 0.44242 | Informedness or bookmaker informedness |
| CEN | 0.57338 | 0.58391 | 0.50086 | 0.63803 | 0.44731 | 0.26735 | 0.42802 | Confusion entropy |
| DOR | 2.88181 | 2.2177 | 3.98261 | 1.18755 | None | 14.33333 | 34.26837 | Diagnostic odds ratio |
| DP | 0.25343 | 0.19071 | 0.33089 | 0.04116 | None | 0.63753 | 0.84623 | Discriminant power |
| DPI | Poor | Poor | Poor | Poor | None | Poor | Poor | Discriminant power interpretation |
| ERR | 0.26428 | 0.43113 | 0.26204 | 0.10078 | 0.03583 | 0.03583 | 0.07727 | Error rate |
| F0.5 | 0.38714 | 0.36893 | 0.45455 | 0.06369 | 0.0 | 0.11628 | 0.625 | F0.5 score |
| F1 | 0.33333 | 0.44122 | 0.45327 | 0.04255 | 0.0 | 0.05882 | 0.55484 | F1 score - harmonic mean of precision and sensitivity |
| F2 | 0.29266 | 0.54874 | 0.452 | 0.03195 | 0.0 | 0.03937 | 0.49884 | F2 score |
| FDR | 0.56618 | 0.6674 | 0.5446 | 0.90476 | None | 0.66667 | 0.31746 | False discovery rate |
| FN | 159 | 80 | 118 | 71 | 32 | 30 | 49 | False negative/miss/type 2 error |
| FNR | 0.72936 | 0.34483 | 0.54884 | 0.9726 | 1.0 | 0.96774 | 0.53261 | Miss rate or false negative rate |
| FOR | 0.21004 | 0.18349 | 0.17353 | 0.08142 | 0.03583 | 0.03371 | 0.05904 | False omission rate |
| FP | 77 | 305 | 116 | 19 | 0 | 2 | 20 | False positive/type 1 error/false alarm |
| FPR | 0.11407 | 0.46142 | 0.17109 | 0.02317 | 0.0 | 0.00232 | 0.02497 | Fall-out or false positive rate |
| G | 0.34265 | 0.46681 | 0.45328 | 0.05108 | None | 0.1037 | 0.56481 | G-measure geometric mean of precision and sensitivity |
| GI | 0.15657 | 0.19375 | 0.28007 | 0.00423 | 0.0 | 0.02994 | 0.44242 | Gini index |
| GM | 0.48966 | 0.59402 | 0.61153 | 0.16359 | 0.0 | 0.1794 | 0.67507 | G-mean geometric mean of specificity and sensitivity |
| IBA | 0.09224 | 0.394 | 0.23271 | 0.00135 | 0.0 | 0.00111 | 0.22438 | Index of balanced accuracy |
| ICSI | -0.29553 | -0.01222 | -0.09344 | -0.87736 | None | -0.63441 | 0.14993 | Individual classification success index |
| IS | 0.82951 | 0.35641 | 0.91953 | 0.22037 | None | 3.26336 | 2.72794 | Information score |
| J | 0.2 | 0.28305 | 0.29305 | 0.02174 | 0.0 | 0.0303 | 0.38393 | Jaccard index |
| LS | 1.77708 | 1.28024 | 1.89149 | 1.16504 | None | 9.60215 | 6.62509 | Lift score |
| MCC | 0.18718 | 0.16998 | 0.28097 | 0.00764 | None | 0.09471 | 0.52522 | Matthews correlation coefficient |
| MCCI | Negligible | Negligible | Negligible | Negligible | None | Negligible | Moderate | Matthews correlation coefficient interpretation |
| MCEN | 0.62936 | 0.67728 | 0.57452 | 0.64343 | 0.44731 | 0.26381 | 0.51179 | Modified confusion entropy |
| MK | 0.22378 | 0.14912 | 0.28187 | 0.01382 | None | 0.29963 | 0.6235 | Markedness |
| N | 675 | 661 | 678 | 820 | 861 | 862 | 801 | Condition negative |
| NLR | 0.82327 | 0.64026 | 0.66212 | 0.99567 | 1.0 | 0.96999 | 0.54625 | Negative likelihood ratio |
| NLRI | Negligible | Negligible | Negligible | Negligible | Negligible | Negligible | Negligible | Negative likelihood ratio interpretation |
| NPV | 0.78996 | 0.81651 | 0.82647 | 0.91858 | 0.96417 | 0.96629 | 0.94096 | Negative predictive value |
| OC | 0.43382 | 0.65517 | 0.4554 | 0.09524 | None | 0.33333 | 0.68254 | Overlap coefficient |
| OOC | 0.34265 | 0.46681 | 0.45328 | 0.05108 | None | 0.1037 | 0.56481 | Otsuka-Ochiai coefficient |
| OP | 0.20373 | 0.4712 | 0.44286 | -0.04622 | -0.03583 | 0.02681 | 0.5708 | Optimized precision |
| P | 218 | 232 | 215 | 73 | 32 | 31 | 92 | Condition positive or support |
| PLR | 2.37251 | 1.4199 | 2.63697 | 1.18241 | None | 13.90323 | 18.71902 | Positive likelihood ratio |
| PLRI | Poor | Poor | Poor | Poor | None | Good | Good | Positive likelihood ratio interpretation |
| POP | 893 | 893 | 893 | 893 | 893 | 893 | 893 | Population |
| PPV | 0.43382 | 0.3326 | 0.4554 | 0.09524 | None | 0.33333 | 0.68254 | Precision or positive predictive value |
| PRE | 0.24412 | 0.2598 | 0.24076 | 0.08175 | 0.03583 | 0.03471 | 0.10302 | Prevalence |
| Q | 0.48478 | 0.37844 | 0.5986 | 0.08573 | None | 0.86957 | 0.94329 | Yule Q - coefficient of colligation |
| QI | Weak | Weak | Moderate | Negligible | None | Strong | Strong | Yule Q interpretation |
| RACC | 0.03718 | 0.13295 | 0.05743 | 0.00192 | 0.0 | 0.00012 | 0.00727 | Random accuracy |
| RACCU | 0.03929 | 0.14882 | 0.05743 | 0.00277 | 0.00032 | 0.00036 | 0.00753 | Random accuracy unbiased |
| TN | 598 | 356 | 562 | 801 | 861 | 860 | 781 | True negative/correct rejection |
| TNR | 0.88593 | 0.53858 | 0.82891 | 0.97683 | 1.0 | 0.99768 | 0.97503 | Specificity or true negative rate |
| TON | 757 | 436 | 680 | 872 | 893 | 890 | 830 | Test outcome negative |
| TOP | 136 | 457 | 213 | 21 | 0 | 3 | 63 | Test outcome positive |
| TP | 59 | 152 | 97 | 2 | 0 | 1 | 43 | True positive/hit |
| TPR | 0.27064 | 0.65517 | 0.45116 | 0.0274 | 0.0 | 0.03226 | 0.46739 | Sensitivity, recall, hit rate, or true positive rate |
| Y | 0.15657 | 0.19375 | 0.28007 | 0.00423 | 0.0 | 0.02994 | 0.44242 | Youden index |
| dInd | 0.73822 | 0.57604 | 0.57489 | 0.97288 | 1.0 | 0.96774 | 0.53319 | Distance index |
| sInd | 0.478 | 0.59268 | 0.59349 | 0.31207 | 0.29289 | 0.3157 | 0.62298 | Similarity index |

Generated By PyCM Version 3.1
