# Supplementary material for: TopEC: prediction of Enzyme Commission classes by 3D graph neural networks and localized 3D protein descriptor
Source: Nat Commun. 2025 Mar 20;16:2737. doi: 10.1038/s41467-025-57324-5 (PMC11923149; doi:10.1038/s41467-025-57324-5)
Supplement: Supplementary file 3 — Supplementary Data 1 [file 41467_2025_57324_MOESM3_ESM.zip › Data_S1/table1/mainclass/DeepFRI/full_struc/TopEnzyme_FOLD.html]

DeepFRI\_TopM\_FOLD


# PyCM Report

## Dataset Type :

- Multi-Class Classification
- Imbalanced

Note 1 : Recommended statistics for this type of classification highlighted in aqua

Note 2 : The recommender system assumes that the input is the result of classification over the whole data rather than just a part of it.
If the confusion matrix is the result of test data classification, the recommendation is not valid.

## Confusion Matrix :

|  |  |  |  |  |  |  |  |  |  |  |  |  |  |  |  |  |  |  |  |  |  |  |  |  |  |  |  |  |  |  |  |  |  |  |  |  |  |  |  |  |  |  |  |  |  |  |  |  |  |  |  |  |  |  |  |  |  |  |  |  |  |  |  |  |  |
| --- | --- | --- | --- | --- | --- | --- | --- | --- | --- | --- | --- | --- | --- | --- | --- | --- | --- | --- | --- | --- | --- | --- | --- | --- | --- | --- | --- | --- | --- | --- | --- | --- | --- | --- | --- | --- | --- | --- | --- | --- | --- | --- | --- | --- | --- | --- | --- | --- | --- | --- | --- | --- | --- | --- | --- | --- | --- | --- | --- | --- | --- | --- | --- | --- | --- |
| Actual | Predict  |  |  |  |  |  |  |  |  | | --- | --- | --- | --- | --- | --- | --- | --- | |  | 0 | 1 | 2 | 3 | 4 | 5 | 6 | | 0 | 39 | 72 | 48 | 7 | 0 | 0 | 4 | | 1 | 24 | 147 | 61 | 13 | 0 | 0 | 0 | | 2 | 8 | 45 | 111 | 9 | 0 | 6 | 0 | | 3 | 13 | 33 | 23 | 5 | 0 | 6 | 3 | | 4 | 13 | 36 | 28 | 6 | 1 | 1 | 0 | | 5 | 8 | 45 | 20 | 6 | 0 | 14 | 0 | | 6 | 2 | 31 | 4 | 0 | 0 | 0 | 1 | |

## Overall Statistics :

|  |  |
| --- | --- |
| 95% CI | (0.3247,0.38751) |
| ACC Macro | 0.81603 |
| ARI | 0.0516 |
| AUNP | 0.58443 |
| AUNU | 0.56195 |
| Bangdiwala B | 0.20051 |
| Bennett S | 0.24879 |
| CBA | 0.17342 |
| CSI | -0.34989 |
| Chi-Squared | 226.40532 |
| Chi-Squared DF | 36 |
| Conditional Entropy | 1.68718 |
| Cramer V | 0.20556 |
| Cross Entropy | 3.35802 |
| F1 Macro | 0.2253 |
| F1 Micro | 0.3561 |
| FNR Macro | 0.75737 |
| FNR Micro | 0.6439 |
| FPR Macro | 0.11873 |
| FPR Micro | 0.10732 |
| Gwet AC1 | 0.2629 |
| Hamming Loss | 0.6439 |
| Joint Entropy | 4.29467 |
| KL Divergence | 0.75054 |
| Kappa | 0.17119 |
| Kappa 95% CI | (0.13076,0.21161) |
| Kappa No Prevalence | -0.28779 |
| Kappa Standard Error | 0.02063 |
| Kappa Unbiased | 0.15132 |
| Krippendorff Alpha | 0.1518 |
| Lambda A | 0.12963 |
| Lambda B | 0.13636 |
| Mutual Information | 0.16839 |
| NIR | 0.27436 |
| Overall ACC | 0.3561 |
| Overall CEN | 0.59419 |
| Overall J | (0.96598,0.138) |
| Overall MCC | 0.18058 |
| Overall MCEN | 0.66612 |
| Overall RACC | 0.22311 |
| Overall RACCU | 0.24129 |
| P-Value | 0.0 |
| PPV Macro | 0.40748 |
| PPV Micro | 0.3561 |
| Pearson C | 0.44973 |
| Phi-Squared | 0.25353 |
| RCI | 0.06458 |
| RR | 127.57143 |
| Reference Entropy | 2.60748 |
| Response Entropy | 1.85558 |
| SOA1(Landis & Koch) | Slight |
| SOA2(Fleiss) | Poor |
| SOA3(Altman) | Poor |
| SOA4(Cicchetti) | Poor |
| SOA5(Cramer) | Moderate |
| SOA6(Matthews) | Negligible |
| Scott PI | 0.15132 |
| Standard Error | 0.01602 |
| TNR Macro | 0.88127 |
| TNR Micro | 0.89268 |
| TPR Macro | 0.24263 |
| TPR Micro | 0.3561 |
| Zero-one Loss | 575 |

## Class Statistics :

|  |  |  |  |  |  |  |  |  |
| --- | --- | --- | --- | --- | --- | --- | --- | --- |
| Class | 0 | 1 | 2 | 3 | 4 | 5 | 6 | Description |
| ACC | 0.77716 | 0.59686 | 0.71781 | 0.86674 | 0.90594 | 0.89698 | 0.95073 | Accuracy |
| AGF | 0.45809 | 0.62867 | 0.68437 | 0.24612 | 0.11635 | 0.40237 | 0.17363 | Adjusted F-score |
| AGM | 0.65725 | 0.59693 | 0.70682 | 0.57696 | 0.53196 | 0.66784 | 0.56766 | Adjusted geometric mean |
| AM | -63 | 164 | 116 | -37 | -84 | -66 | -30 | Difference between automatic and manual classification |
| AUC | 0.56768 | 0.59784 | 0.6812 | 0.50481 | 0.50588 | 0.56714 | 0.50906 | Area under the ROC curve |
| AUCI | Poor | Poor | Fair | Poor | Poor | Poor | Poor | AUC value interpretation |
| AUPR | 0.29695 | 0.47971 | 0.49819 | 0.08447 | 0.50588 | 0.33453 | 0.07566 | Area under the PR curve |
| BCD | 0.03527 | 0.09183 | 0.06495 | 0.02072 | 0.04703 | 0.03695 | 0.0168 | Bray-Curtis dissimilarity |
| BM | 0.13536 | 0.19568 | 0.36241 | 0.00962 | 0.01176 | 0.13429 | 0.01813 | Informedness or bookmaker informedness |
| CEN | 0.62276 | 0.59821 | 0.56604 | 0.79607 | 0.50426 | 0.53774 | 0.40448 | Confusion entropy |
| DOR | 2.86765 | 2.20992 | 4.70189 | 1.20231 | None | 10.72833 | 3.27413 | Diagnostic odds ratio |
| DP | 0.25225 | 0.18987 | 0.37064 | 0.04412 | None | 0.56816 | 0.28399 | Discriminant power |
| DPI | Poor | Poor | Poor | Poor | None | Poor | Poor | Discriminant power interpretation |
| ERR | 0.22284 | 0.40314 | 0.28219 | 0.13326 | 0.09406 | 0.10302 | 0.04927 | Error rate |
| F0.5 | 0.32609 | 0.39075 | 0.40839 | 0.09363 | 0.05618 | 0.34826 | 0.07143 | F0.5 score |
| F1 | 0.28159 | 0.44954 | 0.46835 | 0.07752 | 0.02326 | 0.23333 | 0.04348 | F1 score - harmonic mean of precision and sensitivity |
| F2 | 0.24778 | 0.52916 | 0.54896 | 0.06614 | 0.01466 | 0.17544 | 0.03125 | F2 score |
| FDR | 0.63551 | 0.64059 | 0.62373 | 0.8913 | 0.0 | 0.48148 | 0.875 | False discovery rate |
| FN | 131 | 98 | 68 | 78 | 84 | 79 | 37 | False negative/miss/type 2 error |
| FNR | 0.77059 | 0.4 | 0.37989 | 0.93976 | 0.98824 | 0.84946 | 0.97368 | Miss rate or false negative rate |
| FOR | 0.16667 | 0.20248 | 0.11371 | 0.09209 | 0.09417 | 0.09122 | 0.04181 | False omission rate |
| FP | 68 | 262 | 184 | 41 | 0 | 13 | 7 | False positive/type 1 error/false alarm |
| FPR | 0.09405 | 0.40432 | 0.2577 | 0.05062 | 0.0 | 0.01625 | 0.00819 | Fall-out or false positive rate |
| G | 0.28917 | 0.46438 | 0.48304 | 0.08092 | 0.10847 | 0.27939 | 0.05735 | G-measure geometric mean of precision and sensitivity |
| GI | 0.13536 | 0.19568 | 0.36241 | 0.00962 | 0.01176 | 0.13429 | 0.01813 | Gini index |
| GM | 0.45589 | 0.59784 | 0.67846 | 0.23915 | 0.10847 | 0.38483 | 0.16156 | G-mean geometric mean of specificity and sensitivity |
| IBA | 0.06723 | 0.35895 | 0.40406 | 0.00634 | 0.00014 | 0.0247 | 0.0009 | Index of balanced accuracy |
| ICSI | -0.4061 | -0.04059 | -0.00362 | -0.83106 | 0.01176 | -0.33094 | -0.84868 | Individual classification success index |
| IS | 0.93706 | 0.38959 | 0.90855 | 0.22584 | 3.39313 | 2.31582 | 1.55459 | Information score |
| J | 0.16387 | 0.28994 | 0.30579 | 0.04032 | 0.01176 | 0.13208 | 0.02222 | Jaccard index |
| LS | 1.91462 | 1.31002 | 1.87715 | 1.16946 | 10.50588 | 4.97889 | 2.9375 | Lift score |
| MCC | 0.16364 | 0.17524 | 0.30847 | 0.01264 | 0.10323 | 0.23954 | 0.03884 | Matthews correlation coefficient |
| MCCI | Negligible | Negligible | Weak | Negligible | Negligible | Negligible | Negligible | Matthews correlation coefficient interpretation |
| MCEN | 0.67375 | 0.69891 | 0.66459 | 0.81291 | 0.50554 | 0.56543 | 0.40482 | Modified confusion entropy |
| MK | 0.19782 | 0.15693 | 0.26256 | 0.01661 | 0.90583 | 0.42729 | 0.08319 | Markedness |
| N | 723 | 648 | 714 | 810 | 808 | 800 | 855 | Condition negative |
| NLR | 0.85059 | 0.6715 | 0.51177 | 0.98986 | 0.98824 | 0.86349 | 0.98172 | Negative likelihood ratio |
| NLRI | Negligible | Negligible | Negligible | Negligible | Negligible | Negligible | Negligible | Negative likelihood ratio interpretation |
| NPV | 0.83333 | 0.79752 | 0.88629 | 0.90791 | 0.90583 | 0.90878 | 0.95819 | Negative predictive value |
| OC | 0.36449 | 0.6 | 0.62011 | 0.1087 | 1.0 | 0.51852 | 0.125 | Overlap coefficient |
| OOC | 0.28917 | 0.46438 | 0.48304 | 0.08092 | 0.10847 | 0.27939 | 0.05735 | Otsuka-Ochiai coefficient |
| OP | 0.18128 | 0.59325 | 0.62812 | -0.01393 | -0.07081 | 0.16241 | 0.00242 | Optimized precision |
| P | 170 | 245 | 179 | 83 | 85 | 93 | 38 | Condition positive or support |
| PLR | 2.43919 | 1.48397 | 2.4063 | 1.19013 | None | 9.26385 | 3.21429 | Positive likelihood ratio |
| PLRI | Poor | Poor | Poor | Poor | None | Fair | Poor | Positive likelihood ratio interpretation |
| POP | 893 | 893 | 893 | 893 | 893 | 893 | 893 | Population |
| PPV | 0.36449 | 0.35941 | 0.37627 | 0.1087 | 1.0 | 0.51852 | 0.125 | Precision or positive predictive value |
| PRE | 0.19037 | 0.27436 | 0.20045 | 0.09295 | 0.09518 | 0.10414 | 0.04255 | Prevalence |
| Q | 0.48289 | 0.37693 | 0.64924 | 0.09186 | None | 0.82947 | 0.53207 | Yule Q - coefficient of colligation |
| QI | Weak | Weak | Moderate | Negligible | None | Strong | Moderate | Yule Q interpretation |
| RACC | 0.02281 | 0.12566 | 0.06622 | 0.00479 | 0.00011 | 0.00315 | 0.00038 | Random accuracy |
| RACCU | 0.02405 | 0.13409 | 0.07044 | 0.00522 | 0.00232 | 0.00451 | 0.00066 | Random accuracy unbiased |
| TN | 655 | 386 | 530 | 769 | 808 | 787 | 848 | True negative/correct rejection |
| TNR | 0.90595 | 0.59568 | 0.7423 | 0.94938 | 1.0 | 0.98375 | 0.99181 | Specificity or true negative rate |
| TON | 786 | 484 | 598 | 847 | 892 | 866 | 885 | Test outcome negative |
| TOP | 107 | 409 | 295 | 46 | 1 | 27 | 8 | Test outcome positive |
| TP | 39 | 147 | 111 | 5 | 1 | 14 | 1 | True positive/hit |
| TPR | 0.22941 | 0.6 | 0.62011 | 0.06024 | 0.01176 | 0.15054 | 0.02632 | Sensitivity, recall, hit rate, or true positive rate |
| Y | 0.13536 | 0.19568 | 0.36241 | 0.00962 | 0.01176 | 0.13429 | 0.01813 | Youden index |
| dInd | 0.77631 | 0.56875 | 0.45905 | 0.94112 | 0.98824 | 0.84962 | 0.97372 | Distance index |
| sInd | 0.45107 | 0.59783 | 0.6754 | 0.33453 | 0.30121 | 0.39923 | 0.31148 | Similarity index |

Generated By PyCM Version 3.1
