# Supplementary material for: TopEC: prediction of Enzyme Commission classes by 3D graph neural networks and localized 3D protein descriptor
Source: Nat Commun. 2025 Mar 20;16:2737. doi: 10.1038/s41467-025-57324-5 (PMC11923149; doi:10.1038/s41467-025-57324-5)
Supplement: Supplementary file 3 — Supplementary Data 1 [file 41467_2025_57324_MOESM3_ESM.zip › Data_S1/table1/mainclass/DeepFRI/full_struc/Combined_TEMP.html]

DeepFRI\_Both\_TEMP


# PyCM Report

## Dataset Type :

- Multi-Class Classification
- Imbalanced

Note 1 : Recommended statistics for this type of classification highlighted in aqua

Note 2 : The recommender system assumes that the input is the result of classification over the whole data rather than just a part of it.
If the confusion matrix is the result of test data classification, the recommendation is not valid.

## Confusion Matrix :

|  |  |  |  |  |  |  |  |  |  |  |  |  |  |  |  |  |  |  |  |  |  |  |  |  |  |  |  |  |  |  |  |  |  |  |  |  |  |  |  |  |  |  |  |  |  |  |  |  |  |  |  |  |  |  |  |  |  |  |  |  |  |  |  |  |  |
| --- | --- | --- | --- | --- | --- | --- | --- | --- | --- | --- | --- | --- | --- | --- | --- | --- | --- | --- | --- | --- | --- | --- | --- | --- | --- | --- | --- | --- | --- | --- | --- | --- | --- | --- | --- | --- | --- | --- | --- | --- | --- | --- | --- | --- | --- | --- | --- | --- | --- | --- | --- | --- | --- | --- | --- | --- | --- | --- | --- | --- | --- | --- | --- | --- | --- |
| Actual | Predict  |  |  |  |  |  |  |  |  | | --- | --- | --- | --- | --- | --- | --- | --- | |  | 0 | 1 | 2 | 3 | 4 | 5 | 6 | | 0 | 260 | 237 | 94 | 33 | 7 | 4 | 6 | | 1 | 126 | 661 | 141 | 31 | 10 | 9 | 8 | | 2 | 62 | 283 | 507 | 13 | 6 | 10 | 4 | | 3 | 34 | 69 | 30 | 65 | 0 | 0 | 0 | | 4 | 7 | 51 | 14 | 5 | 23 | 9 | 0 | | 5 | 5 | 61 | 6 | 5 | 0 | 12 | 0 | | 6 | 18 | 54 | 49 | 8 | 0 | 5 | 34 | |

## Overall Statistics :

|  |  |
| --- | --- |
| 95% CI | (0.49013,0.52547) |
| ACC Macro | 0.85937 |
| ARI | 0.13411 |
| AUNP | 0.66174 |
| AUNU | 0.63206 |
| Bangdiwala B | 0.3048 |
| Bennett S | 0.42577 |
| CBA | 0.33169 |
| CSI | -0.15602 |
| Chi-Squared | 1985.29406 |
| Chi-Squared DF | 36 |
| Conditional Entropy | 1.66679 |
| Cramer V | 0.32798 |
| Cross Entropy | 2.41978 |
| F1 Macro | 0.39025 |
| F1 Micro | 0.5078 |
| FNR Macro | 0.63923 |
| FNR Micro | 0.4922 |
| FPR Macro | 0.09664 |
| FPR Micro | 0.08203 |
| Gwet AC1 | 0.43985 |
| Hamming Loss | 0.4922 |
| Joint Entropy | 3.98402 |
| KL Divergence | 0.10256 |
| Kappa | 0.32928 |
| Kappa 95% CI | (0.3052,0.35335) |
| Kappa No Prevalence | 0.0156 |
| Kappa Standard Error | 0.01228 |
| Kappa Unbiased | 0.32382 |
| Krippendorff Alpha | 0.32393 |
| Lambda A | 0.2756 |
| Lambda B | 0.1488 |
| Mutual Information | 0.29768 |
| NIR | 0.32055 |
| Overall ACC | 0.5078 |
| Overall CEN | 0.51066 |
| Overall J | (1.76085,0.25155) |
| Overall MCC | 0.33518 |
| Overall MCEN | 0.60939 |
| Overall RACC | 0.26617 |
| Overall RACCU | 0.27209 |
| P-Value | None |
| PPV Macro | 0.48321 |
| PPV Micro | 0.5078 |
| Pearson C | 0.6263 |
| Phi-Squared | 0.64541 |
| RCI | 0.12847 |
| RR | 439.42857 |
| Reference Entropy | 2.31722 |
| Response Entropy | 1.96448 |
| SOA1(Landis & Koch) | Fair |
| SOA2(Fleiss) | Poor |
| SOA3(Altman) | Fair |
| SOA4(Cicchetti) | Poor |
| SOA5(Cramer) | Moderate |
| SOA6(Matthews) | Weak |
| Scott PI | 0.32382 |
| Standard Error | 0.00901 |
| TNR Macro | 0.90336 |
| TNR Micro | 0.91797 |
| TPR Macro | 0.36077 |
| TPR Micro | 0.5078 |
| Zero-one Loss | 1514 |

## Class Statistics :

|  |  |  |  |  |  |  |  |  |
| --- | --- | --- | --- | --- | --- | --- | --- | --- |
| Class | 0 | 1 | 2 | 3 | 4 | 5 | 6 | Description |
| ACC | 0.79421 | 0.64889 | 0.76853 | 0.92588 | 0.96456 | 0.96294 | 0.95059 | Accuracy |
| AGF | 0.6029 | 0.68663 | 0.69475 | 0.57155 | 0.48248 | 0.38048 | 0.47554 | Adjusted F-score |
| AGM | 0.7327 | 0.64806 | 0.75952 | 0.7585 | 0.72009 | 0.67169 | 0.71349 | Adjusted geometric mean |
| AM | -129 | 430 | -44 | -38 | -63 | -40 | -116 | Difference between automatic and manual classification |
| AUC | 0.65106 | 0.65457 | 0.71022 | 0.64764 | 0.60163 | 0.56122 | 0.5981 | Area under the ROC curve |
| AUCI | Fair | Fair | Good | Fair | Fair | Poor | Poor | AUC value interpretation |
| AUPR | 0.45671 | 0.5686 | 0.58787 | 0.36727 | 0.3555 | 0.18986 | 0.42811 | Area under the PR curve |
| BCD | 0.02097 | 0.0699 | 0.00715 | 0.00618 | 0.01024 | 0.0065 | 0.01886 | Bray-Curtis dissimilarity |
| BM | 0.30213 | 0.30914 | 0.42044 | 0.29527 | 0.20326 | 0.12244 | 0.19619 | Informedness or bookmaker informedness |
| CEN | 0.53596 | 0.51624 | 0.4466 | 0.60563 | 0.58008 | 0.60636 | 0.55624 | Confusion entropy |
| DOR | 5.91155 | 3.59627 | 7.4573 | 14.31698 | 34.23256 | 12.42541 | 40.73798 | Diagnostic odds ratio |
| DP | 0.42546 | 0.30646 | 0.48108 | 0.63725 | 0.84598 | 0.60333 | 0.88764 | Discriminant power |
| DPI | Poor | Poor | Poor | Poor | Poor | Poor | Poor | Discriminant power interpretation |
| ERR | 0.20579 | 0.35111 | 0.23147 | 0.07412 | 0.03544 | 0.03706 | 0.04941 | Error rate |
| F0.5 | 0.48345 | 0.49699 | 0.59661 | 0.38783 | 0.39249 | 0.21053 | 0.45213 | F0.5 score |
| F1 | 0.451 | 0.55037 | 0.58749 | 0.36313 | 0.29677 | 0.17391 | 0.30909 | F1 score - harmonic mean of precision and sensitivity |
| F2 | 0.42263 | 0.6166 | 0.57864 | 0.34139 | 0.23859 | 0.14815 | 0.23481 | F2 score |
| FDR | 0.49219 | 0.53319 | 0.39715 | 0.59375 | 0.5 | 0.7551 | 0.34615 | False discovery rate |
| FN | 381 | 325 | 378 | 133 | 86 | 77 | 134 | False negative/miss/type 2 error |
| FNR | 0.59438 | 0.32961 | 0.42712 | 0.67172 | 0.78899 | 0.86517 | 0.79762 | Miss rate or false negative rate |
| FOR | 0.1486 | 0.19578 | 0.16913 | 0.04561 | 0.02838 | 0.02544 | 0.04431 | False omission rate |
| FP | 252 | 755 | 334 | 95 | 23 | 37 | 18 | False positive/type 1 error/false alarm |
| FPR | 0.10349 | 0.36124 | 0.15244 | 0.03301 | 0.00775 | 0.01239 | 0.00619 | Fall-out or false positive rate |
| G | 0.45385 | 0.55941 | 0.58768 | 0.36519 | 0.32481 | 0.18171 | 0.36377 | G-measure geometric mean of precision and sensitivity |
| GI | 0.30213 | 0.30914 | 0.42044 | 0.29527 | 0.20326 | 0.12244 | 0.19619 | Gini index |
| GM | 0.60302 | 0.65438 | 0.69681 | 0.56342 | 0.45757 | 0.36491 | 0.44847 | G-mean geometric mean of specificity and sensitivity |
| IBA | 0.18513 | 0.44176 | 0.35218 | 0.11469 | 0.0458 | 0.0196 | 0.04195 | Index of balanced accuracy |
| ICSI | -0.08657 | 0.13719 | 0.17574 | -0.26547 | -0.28899 | -0.62027 | -0.14377 | Individual classification success index |
| IS | 1.28503 | 0.5423 | 1.06719 | 2.65792 | 3.81866 | 3.08136 | 3.58155 | Information score |
| J | 0.29115 | 0.37967 | 0.41591 | 0.22184 | 0.17424 | 0.09524 | 0.1828 | Jaccard index |
| LS | 2.43687 | 1.45629 | 2.09534 | 6.31124 | 14.11009 | 8.46411 | 11.97161 | Lift score |
| MCC | 0.32944 | 0.28946 | 0.42703 | 0.32632 | 0.30961 | 0.16393 | 0.34581 | Matthews correlation coefficient |
| MCCI | Weak | Negligible | Weak | Weak | Weak | Negligible | Weak | Matthews correlation coefficient interpretation |
| MCEN | 0.61911 | 0.6319 | 0.5506 | 0.67724 | 0.62777 | 0.63098 | 0.60271 | Modified confusion entropy |
| MK | 0.35922 | 0.27102 | 0.43373 | 0.36064 | 0.47162 | 0.21946 | 0.60953 | Markedness |
| N | 2435 | 2090 | 2191 | 2878 | 2967 | 2987 | 2908 | Condition negative |
| NLR | 0.663 | 0.51603 | 0.50394 | 0.69465 | 0.79515 | 0.87602 | 0.80259 | Negative likelihood ratio |
| NLRI | Negligible | Negligible | Negligible | Negligible | Negligible | Negligible | Negligible | Negative likelihood ratio interpretation |
| NPV | 0.8514 | 0.80422 | 0.83087 | 0.95439 | 0.97162 | 0.97456 | 0.95569 | Negative predictive value |
| OC | 0.50781 | 0.67039 | 0.60285 | 0.40625 | 0.5 | 0.2449 | 0.65385 | Overlap coefficient |
| OOC | 0.45385 | 0.55941 | 0.58768 | 0.36519 | 0.32481 | 0.18171 | 0.36377 | Otsuka-Ochiai coefficient |
| OP | 0.41722 | 0.62473 | 0.57516 | 0.43277 | 0.31529 | 0.20318 | 0.28896 | Optimized precision |
| P | 641 | 986 | 885 | 198 | 109 | 89 | 168 | Condition positive or support |
| PLR | 3.91935 | 1.85577 | 3.75803 | 9.94524 | 27.22018 | 10.88491 | 32.69577 | Positive likelihood ratio |
| PLRI | Poor | Poor | Poor | Fair | Good | Good | Good | Positive likelihood ratio interpretation |
| POP | 3076 | 3076 | 3076 | 3076 | 3076 | 3076 | 3076 | Population |
| PPV | 0.50781 | 0.46681 | 0.60285 | 0.40625 | 0.5 | 0.2449 | 0.65385 | Precision or positive predictive value |
| PRE | 0.20839 | 0.32055 | 0.28771 | 0.06437 | 0.03544 | 0.02893 | 0.05462 | Prevalence |
| Q | 0.71063 | 0.56486 | 0.76352 | 0.86943 | 0.94323 | 0.85103 | 0.95208 | Yule Q - coefficient of colligation |
| QI | Moderate | Moderate | Strong | Strong | Strong | Strong | Strong | Yule Q interpretation |
| RACC | 0.03469 | 0.14756 | 0.07866 | 0.00335 | 0.00053 | 0.00046 | 0.00092 | Random accuracy |
| RACCU | 0.03513 | 0.15245 | 0.07871 | 0.00339 | 0.00063 | 0.0005 | 0.00128 | Random accuracy unbiased |
| TN | 2183 | 1335 | 1857 | 2783 | 2944 | 2950 | 2890 | True negative/correct rejection |
| TNR | 0.89651 | 0.63876 | 0.84756 | 0.96699 | 0.99225 | 0.98761 | 0.99381 | Specificity or true negative rate |
| TON | 2564 | 1660 | 2235 | 2916 | 3030 | 3027 | 3024 | Test outcome negative |
| TOP | 512 | 1416 | 841 | 160 | 46 | 49 | 52 | Test outcome positive |
| TP | 260 | 661 | 507 | 65 | 23 | 12 | 34 | True positive/hit |
| TPR | 0.40562 | 0.67039 | 0.57288 | 0.32828 | 0.21101 | 0.13483 | 0.20238 | Sensitivity, recall, hit rate, or true positive rate |
| Y | 0.30213 | 0.30914 | 0.42044 | 0.29527 | 0.20326 | 0.12244 | 0.19619 | Youden index |
| dInd | 0.60333 | 0.48902 | 0.45351 | 0.67253 | 0.78903 | 0.86526 | 0.79764 | Distance index |
| sInd | 0.57338 | 0.65421 | 0.67932 | 0.52445 | 0.44207 | 0.38817 | 0.43598 | Similarity index |

Generated By PyCM Version 3.1
