# Supplementary material for: TopEC: prediction of Enzyme Commission classes by 3D graph neural networks and localized 3D protein descriptor
Source: Nat Commun. 2025 Mar 20;16:2737. doi: 10.1038/s41467-025-57324-5 (PMC11923149; doi:10.1038/s41467-025-57324-5)
Supplement: Supplementary file 3 — Supplementary Data 1 [file 41467_2025_57324_MOESM3_ESM.zip › Data_S1/table1/mainclass/DeepFRI/full_struc/Combined_FOLD.html]

DeepFRI\_Both\_FOLD


# PyCM Report

## Dataset Type :

- Multi-Class Classification
- Imbalanced

Note 1 : Recommended statistics for this type of classification highlighted in aqua

Note 2 : The recommender system assumes that the input is the result of classification over the whole data rather than just a part of it.
If the confusion matrix is the result of test data classification, the recommendation is not valid.

## Confusion Matrix :

|  |  |  |  |  |  |  |  |  |  |  |  |  |  |  |  |  |  |  |  |  |  |  |  |  |  |  |  |  |  |  |  |  |  |  |  |  |  |  |  |  |  |  |  |  |  |  |  |  |  |  |  |  |  |  |  |  |  |  |  |  |  |  |  |  |  |
| --- | --- | --- | --- | --- | --- | --- | --- | --- | --- | --- | --- | --- | --- | --- | --- | --- | --- | --- | --- | --- | --- | --- | --- | --- | --- | --- | --- | --- | --- | --- | --- | --- | --- | --- | --- | --- | --- | --- | --- | --- | --- | --- | --- | --- | --- | --- | --- | --- | --- | --- | --- | --- | --- | --- | --- | --- | --- | --- | --- | --- | --- | --- | --- | --- | --- |
| Actual | Predict  |  |  |  |  |  |  |  |  | | --- | --- | --- | --- | --- | --- | --- | --- | |  | 0 | 1 | 2 | 3 | 4 | 5 | 6 | | 0 | 331 | 176 | 61 | 0 | 1 | 0 | 8 | | 1 | 208 | 792 | 45 | 2 | 0 | 4 | 0 | | 2 | 63 | 108 | 397 | 5 | 3 | 5 | 0 | | 3 | 93 | 87 | 28 | 0 | 0 | 6 | 1 | | 4 | 168 | 69 | 17 | 0 | 1 | 1 | 0 | | 5 | 31 | 56 | 25 | 0 | 2 | 27 | 0 | | 6 | 12 | 38 | 5 | 0 | 0 | 0 | 0 | |

## Overall Statistics :

|  |  |
| --- | --- |
| 95% CI | (0.52003,0.55647) |
| ACC Macro | 0.86807 |
| ARI | 0.21465 |
| AUNP | 0.68223 |
| AUNU | 0.61217 |
| Bangdiwala B | 0.39573 |
| Bennett S | 0.46129 |
| CBA | 0.26305 |
| CSI | -0.33912 |
| Chi-Squared | 1940.27974 |
| Chi-Squared DF | 36 |
| Conditional Entropy | 1.27554 |
| Cramer V | 0.33532 |
| Cross Entropy | 3.08849 |
| F1 Macro | 0.29985 |
| F1 Micro | 0.53825 |
| FNR Macro | 0.68487 |
| FNR Micro | 0.46175 |
| FPR Macro | 0.09079 |
| FPR Micro | 0.07696 |
| Gwet AC1 | 0.47554 |
| Hamming Loss | 0.46175 |
| Joint Entropy | 3.65015 |
| KL Divergence | 0.71388 |
| Kappa | 0.36443 |
| Kappa 95% CI | (0.33935,0.38951) |
| Kappa No Prevalence | 0.0765 |
| Kappa Standard Error | 0.0128 |
| Kappa Unbiased | 0.35638 |
| Krippendorff Alpha | 0.35649 |
| Lambda A | 0.28055 |
| Lambda B | 0.35419 |
| Mutual Information | 0.38862 |
| NIR | 0.36544 |
| Overall ACC | 0.53825 |
| Overall CEN | 0.44904 |
| Overall J | (1.4838,0.21197) |
| Overall MCC | 0.37517 |
| Overall MCEN | 0.54706 |
| Overall RACC | 0.27348 |
| Overall RACCU | 0.28257 |
| P-Value | None |
| PPV Macro | 0.34575 |
| PPV Micro | 0.53825 |
| Pearson C | 0.63471 |
| Phi-Squared | 0.67465 |
| RCI | 0.16366 |
| RR | 410.85714 |
| Reference Entropy | 2.37461 |
| Response Entropy | 1.66417 |
| SOA1(Landis & Koch) | Fair |
| SOA2(Fleiss) | Poor |
| SOA3(Altman) | Fair |
| SOA4(Cicchetti) | Poor |
| SOA5(Cramer) | Moderate |
| SOA6(Matthews) | Weak |
| Scott PI | 0.35638 |
| Standard Error | 0.0093 |
| TNR Macro | 0.90921 |
| TNR Micro | 0.92304 |
| TPR Macro | 0.31513 |
| TPR Micro | 0.53825 |
| Zero-one Loss | 1328 |

## Class Statistics :

|  |  |  |  |  |  |  |  |  |
| --- | --- | --- | --- | --- | --- | --- | --- | --- |
| Class | 0 | 1 | 2 | 3 | 4 | 5 | 6 | Description |
| ACC | 0.71453 | 0.72427 | 0.87309 | 0.92281 | 0.90925 | 0.9548 | 0.97775 | Accuracy |
| AGF | 0.66035 | 0.75895 | 0.79335 | 0.0 | 0.06706 | 0.46362 | 0.0 | Adjusted F-score |
| AGM | 0.69765 | 0.7213 | 0.85007 | 0 | 0.50829 | 0.70822 | 0 | Adjusted geometric mean |
| AM | 329 | 275 | -3 | -208 | -249 | -98 | -46 | Difference between automatic and manual classification |
| AUC | 0.66177 | 0.73048 | 0.80222 | 0.49868 | 0.50081 | 0.59282 | 0.4984 | Area under the ROC curve |
| AUCI | Fair | Good | Very Good | Poor | Poor | Poor | Poor | AUC value interpretation |
| AUPR | 0.4695 | 0.67543 | 0.68508 | 0.0 | 0.07338 | 0.4097 | 0.0 | Area under the PR curve |
| BCD | 0.0572 | 0.04781 | 0.00052 | 0.03616 | 0.04329 | 0.01704 | 0.008 | Bray-Curtis dissimilarity |
| BM | 0.32355 | 0.46097 | 0.60444 | -0.00263 | 0.00162 | 0.18564 | -0.00319 | Informedness or bookmaker informedness |
| CEN | 0.5502 | 0.40903 | 0.39481 | 0.50003 | 0.38025 | 0.52462 | 0.46178 | Confusion entropy |
| DOR | 4.03425 | 7.39282 | 25.19992 | 0.0 | 1.7085 | 40.24836 | 0.0 | Diagnostic odds ratio |
| DP | 0.33397 | 0.479 | 0.77263 | None | 0.12825 | 0.88474 | None | Discriminant power |
| DPI | Poor | Poor | Poor | None | Poor | Poor | None | Discriminant power interpretation |
| ERR | 0.28547 | 0.27573 | 0.12691 | 0.07719 | 0.09075 | 0.0452 | 0.02225 | Error rate |
| F0.5 | 0.39395 | 0.62313 | 0.68614 | 0.0 | 0.01761 | 0.43131 | 0.0 | F0.5 score |
| F1 | 0.44639 | 0.66639 | 0.68507 | 0.0 | 0.0076 | 0.29348 | 0.0 | F1 score - harmonic mean of precision and sensitivity |
| F2 | 0.51493 | 0.71609 | 0.68401 | 0.0 | 0.00485 | 0.22241 | 0.0 | F2 score |
| FDR | 0.63466 | 0.40271 | 0.31315 | 1.0 | 0.85714 | 0.37209 | 1.0 | False discovery rate |
| FN | 246 | 259 | 184 | 215 | 255 | 114 | 55 | False negative/miss/type 2 error |
| FNR | 0.42634 | 0.24643 | 0.3167 | 1.0 | 0.99609 | 0.80851 | 1.0 | Miss rate or false negative rate |
| FOR | 0.12487 | 0.1671 | 0.08007 | 0.07494 | 0.08888 | 0.04024 | 0.01918 | False omission rate |
| FP | 575 | 534 | 181 | 7 | 6 | 16 | 9 | False positive/type 1 error/false alarm |
| FPR | 0.25011 | 0.2926 | 0.07887 | 0.00263 | 0.00229 | 0.00585 | 0.00319 | Fall-out or false positive rate |
| G | 0.4578 | 0.67089 | 0.68508 | 0.0 | 0.02362 | 0.34675 | 0.0 | G-measure geometric mean of precision and sensitivity |
| GI | 0.32355 | 0.46097 | 0.60444 | -0.00263 | 0.00162 | 0.18564 | -0.00319 | Gini index |
| GM | 0.65588 | 0.73012 | 0.79336 | 0.0 | 0.06243 | 0.43631 | 0.0 | G-mean geometric mean of specificity and sensitivity |
| IBA | 0.35437 | 0.55768 | 0.47972 | 0.0 | 2e-05 | 0.03757 | 0.0 | Index of balanced accuracy |
| ICSI | -0.061 | 0.35085 | 0.37016 | -1.0 | -0.85324 | -0.1806 | -1.0 | Individual classification success index |
| IS | 0.86474 | 0.70879 | 1.76552 | None | 0.68249 | 3.67892 | None | Information score |
| J | 0.28733 | 0.49968 | 0.521 | 0.0 | 0.00382 | 0.17197 | 0.0 | Jaccard index |
| LS | 1.82101 | 1.63444 | 3.39997 | 0.0 | 1.60491 | 12.80752 | 0.0 | Lift score |
| MCC | 0.27893 | 0.44531 | 0.60561 | -0.01404 | 0.00934 | 0.33029 | -0.00782 | Matthews correlation coefficient |
| MCCI | Negligible | Weak | Moderate | Negligible | Negligible | Weak | Negligible | Matthews correlation coefficient interpretation |
| MCEN | 0.63585 | 0.53183 | 0.51967 | 0.50003 | 0.38018 | 0.56196 | 0.46178 | Modified confusion entropy |
| MK | 0.24047 | 0.43019 | 0.60678 | -0.07494 | 0.05398 | 0.58767 | -0.01918 | Markedness |
| N | 2299 | 1825 | 2295 | 2661 | 2620 | 2735 | 2821 | Condition negative |
| NLR | 0.56854 | 0.34836 | 0.34381 | 1.00264 | 0.99838 | 0.81327 | 1.0032 | Negative likelihood ratio |
| NLRI | Negligible | Poor | Poor | Negligible | Negligible | Negligible | Negligible | Negative likelihood ratio interpretation |
| NPV | 0.87513 | 0.8329 | 0.91993 | 0.92506 | 0.91112 | 0.95976 | 0.98082 | Negative predictive value |
| OC | 0.57366 | 0.75357 | 0.68685 | 0.0 | 0.14286 | 0.62791 | 0.0 | Overlap coefficient |
| OOC | 0.4578 | 0.67089 | 0.68508 | 0.0 | 0.02362 | 0.34675 | 0.0 | Otsuka-Ochiai coefficient |
| OP | 0.58138 | 0.69267 | 0.72486 | -0.07719 | -0.08295 | 0.27781 | -0.02225 | Optimized precision |
| P | 577 | 1051 | 581 | 215 | 256 | 141 | 55 | Condition positive or support |
| PLR | 2.29363 | 2.5754 | 8.664 | 0.0 | 1.70573 | 32.73271 | 0.0 | Positive likelihood ratio |
| PLRI | Poor | Poor | Fair | Negligible | Poor | Good | Negligible | Positive likelihood ratio interpretation |
| POP | 2876 | 2876 | 2876 | 2876 | 2876 | 2876 | 2876 | Population |
| PPV | 0.36534 | 0.59729 | 0.68685 | 0.0 | 0.14286 | 0.62791 | 0.0 | Precision or positive predictive value |
| PRE | 0.20063 | 0.36544 | 0.20202 | 0.07476 | 0.08901 | 0.04903 | 0.01912 | Prevalence |
| Q | 0.60272 | 0.7617 | 0.92366 | -1.0 | 0.26158 | 0.95151 | -1.0 | Yule Q - coefficient of colligation |
| QI | Moderate | Strong | Strong | Negligible | Weak | Strong | Negligible | Yule Q interpretation |
| RACC | 0.0632 | 0.16849 | 0.0406 | 0.00018 | 0.00022 | 0.00073 | 6e-05 | Random accuracy |
| RACCU | 0.06647 | 0.17077 | 0.0406 | 0.00149 | 0.00209 | 0.00102 | 0.00012 | Random accuracy unbiased |
| TN | 1724 | 1291 | 2114 | 2654 | 2614 | 2719 | 2812 | True negative/correct rejection |
| TNR | 0.74989 | 0.7074 | 0.92113 | 0.99737 | 0.99771 | 0.99415 | 0.99681 | Specificity or true negative rate |
| TON | 1970 | 1550 | 2298 | 2869 | 2869 | 2833 | 2867 | Test outcome negative |
| TOP | 906 | 1326 | 578 | 7 | 7 | 43 | 9 | Test outcome positive |
| TP | 331 | 792 | 397 | 0 | 1 | 27 | 0 | True positive/hit |
| TPR | 0.57366 | 0.75357 | 0.6833 | 0.0 | 0.00391 | 0.19149 | 0.0 | Sensitivity, recall, hit rate, or true positive rate |
| Y | 0.32355 | 0.46097 | 0.60444 | -0.00263 | 0.00162 | 0.18564 | -0.00319 | Youden index |
| dInd | 0.49429 | 0.38255 | 0.32637 | 1.0 | 0.9961 | 0.80853 | 1.00001 | Distance index |
| sInd | 0.65048 | 0.7295 | 0.76922 | 0.29289 | 0.29565 | 0.42828 | 0.29289 | Similarity index |

Generated By PyCM Version 3.1
