# Supplementary material for: TopEC: prediction of Enzyme Commission classes by 3D graph neural networks and localized 3D protein descriptor
Source: Nat Commun. 2025 Mar 20;16:2737. doi: 10.1038/s41467-025-57324-5 (PMC11923149; doi:10.1038/s41467-025-57324-5)
Supplement: Supplementary file 3 — Supplementary Data 1 [file 41467_2025_57324_MOESM3_ESM.zip › Data_S1/table1/mainclass/DeepFRI/full_struc/BindingMOAD_TEMP.html]

DeepFRI\_PDB\_TEMP


# PyCM Report

## Dataset Type :

- Multi-Class Classification
- Imbalanced

Note 1 : Recommended statistics for this type of classification highlighted in aqua

Note 2 : The recommender system assumes that the input is the result of classification over the whole data rather than just a part of it.
If the confusion matrix is the result of test data classification, the recommendation is not valid.

## Confusion Matrix :

|  |  |  |  |  |  |  |  |  |  |  |  |  |  |  |  |  |  |  |  |  |  |  |  |  |  |  |  |  |  |  |  |  |  |  |  |  |  |  |  |  |  |  |  |  |  |  |  |  |  |  |  |  |  |  |  |  |  |  |  |  |  |  |  |  |  |
| --- | --- | --- | --- | --- | --- | --- | --- | --- | --- | --- | --- | --- | --- | --- | --- | --- | --- | --- | --- | --- | --- | --- | --- | --- | --- | --- | --- | --- | --- | --- | --- | --- | --- | --- | --- | --- | --- | --- | --- | --- | --- | --- | --- | --- | --- | --- | --- | --- | --- | --- | --- | --- | --- | --- | --- | --- | --- | --- | --- | --- | --- | --- | --- | --- | --- |
| Actual | Predict  |  |  |  |  |  |  |  |  | | --- | --- | --- | --- | --- | --- | --- | --- | |  | 0 | 1 | 2 | 3 | 4 | 5 | 6 | | 0 | 238 | 98 | 66 | 18 | 2 | 1 | 0 | | 1 | 132 | 498 | 116 | 4 | 1 | 3 | 0 | | 2 | 84 | 145 | 425 | 14 | 0 | 1 | 1 | | 3 | 38 | 15 | 15 | 53 | 0 | 4 | 0 | | 4 | 8 | 18 | 26 | 0 | 24 | 1 | 0 | | 5 | 3 | 23 | 21 | 4 | 0 | 7 | 0 | | 6 | 4 | 32 | 33 | 1 | 0 | 5 | 1 | |

## Overall Statistics :

|  |  |
| --- | --- |
| 95% CI | (0.55001,0.59154) |
| ACC Macro | 0.87736 |
| ARI | 0.20196 |
| AUNP | 0.70178 |
| AUNU | 0.65218 |
| Bangdiwala B | 0.3688 |
| Bennett S | 0.49924 |
| CBA | 0.36359 |
| CSI | -0.04665 |
| Chi-Squared | 2011.52025 |
| Chi-Squared DF | 36 |
| Conditional Entropy | 1.50046 |
| Cramer V | 0.39189 |
| Cross Entropy | 2.40388 |
| F1 Macro | 0.41525 |
| F1 Micro | 0.57077 |
| FNR Macro | 0.61043 |
| FNR Micro | 0.42923 |
| FPR Macro | 0.0852 |
| FPR Micro | 0.07154 |
| Gwet AC1 | 0.51218 |
| Hamming Loss | 0.42923 |
| Joint Entropy | 3.72615 |
| KL Divergence | 0.17819 |
| Kappa | 0.40545 |
| Kappa 95% CI | (0.37669,0.43421) |
| Kappa No Prevalence | 0.14155 |
| Kappa Standard Error | 0.01467 |
| Kappa Unbiased | 0.40441 |
| Krippendorff Alpha | 0.40455 |
| Lambda A | 0.3443 |
| Lambda B | 0.3449 |
| Mutual Information | 0.39538 |
| NIR | 0.3454 |
| Overall ACC | 0.57077 |
| Overall CEN | 0.4546 |
| Overall J | (1.97986,0.28284) |
| Overall MCC | 0.40707 |
| Overall MCEN | 0.55844 |
| Overall RACC | 0.27807 |
| Overall RACCU | 0.27932 |
| P-Value | None |
| PPV Macro | 0.56378 |
| PPV Micro | 0.57077 |
| Pearson C | 0.6925 |
| Phi-Squared | 0.92145 |
| RCI | 0.17764 |
| RR | 311.85714 |
| Reference Entropy | 2.22569 |
| Response Entropy | 1.89584 |
| SOA1(Landis & Koch) | Moderate |
| SOA2(Fleiss) | Intermediate to Good |
| SOA3(Altman) | Moderate |
| SOA4(Cicchetti) | Fair |
| SOA5(Cramer) | Moderate |
| SOA6(Matthews) | Weak |
| Scott PI | 0.40441 |
| Standard Error | 0.01059 |
| TNR Macro | 0.9148 |
| TNR Micro | 0.92846 |
| TPR Macro | 0.38957 |
| TPR Micro | 0.57077 |
| Zero-one Loss | 937 |

## Class Statistics :

|  |  |  |  |  |  |  |  |  |
| --- | --- | --- | --- | --- | --- | --- | --- | --- |
| Class | 0 | 1 | 2 | 3 | 4 | 5 | 6 | Description |
| ACC | 0.79203 | 0.7311 | 0.76088 | 0.94824 | 0.97435 | 0.96977 | 0.96519 | Accuracy |
| AGF | 0.69039 | 0.72069 | 0.72258 | 0.65729 | 0.59248 | 0.36741 | 0.12604 | Adjusted F-score |
| AGM | 0.76037 | 0.73453 | 0.75959 | 0.80741 | 0.77428 | 0.6652 | 0.54927 | Adjusted geometric mean |
| AM | 84 | 75 | 32 | -31 | -50 | -36 | -74 | Difference between automatic and manual classification |
| AUC | 0.7049 | 0.71442 | 0.72562 | 0.70204 | 0.65513 | 0.55682 | 0.50634 | Area under the ROC curve |
| AUCI | Good | Good | Good | Good | Fair | Poor | Poor | AUC value interpretation |
| AUPR | 0.51604 | 0.6306 | 0.61987 | 0.49391 | 0.60029 | 0.21944 | 0.25658 | Area under the PR curve |
| BCD | 0.01924 | 0.01718 | 0.00733 | 0.0071 | 0.01145 | 0.00825 | 0.01695 | Bray-Curtis dissimilarity |
| BM | 0.40981 | 0.42885 | 0.45125 | 0.40408 | 0.31026 | 0.11363 | 0.01268 | Informedness or bookmaker informedness |
| CEN | 0.49481 | 0.42533 | 0.4422 | 0.52166 | 0.40756 | 0.64104 | 0.47068 | Confusion entropy |
| DOR | 7.13067 | 6.45303 | 7.74037 | 36.21308 | 317.43396 | 19.30719 | 28.08 | Diagnostic odds ratio |
| DP | 0.47036 | 0.44645 | 0.49 | 0.85945 | 1.37923 | 0.70885 | 0.79854 | Discriminant power |
| DPI | Poor | Poor | Poor | Poor | Limited | Poor | Poor | Discriminant power interpretation |
| ERR | 0.20797 | 0.2689 | 0.23912 | 0.05176 | 0.02565 | 0.03023 | 0.03481 | Error rate |
| F0.5 | 0.48552 | 0.61179 | 0.61098 | 0.52894 | 0.64865 | 0.23973 | 0.05952 | F0.5 score |
| F1 | 0.51183 | 0.62919 | 0.61953 | 0.48402 | 0.46154 | 0.175 | 0.02564 | F1 score - harmonic mean of precision and sensitivity |
| F2 | 0.54116 | 0.64759 | 0.62833 | 0.44613 | 0.35821 | 0.1378 | 0.01634 | F2 score |
| FDR | 0.53057 | 0.39928 | 0.39459 | 0.43617 | 0.11111 | 0.68182 | 0.5 | False discovery rate |
| FN | 185 | 256 | 245 | 72 | 53 | 51 | 75 | False negative/miss/type 2 error |
| FNR | 0.43735 | 0.33952 | 0.36567 | 0.576 | 0.68831 | 0.87931 | 0.98684 | Miss rate or false negative rate |
| FOR | 0.11038 | 0.18907 | 0.16543 | 0.03447 | 0.02458 | 0.0236 | 0.03439 | False omission rate |
| FP | 269 | 331 | 277 | 41 | 3 | 15 | 1 | False positive/type 1 error/false alarm |
| FPR | 0.15284 | 0.23163 | 0.18308 | 0.01992 | 0.00142 | 0.00706 | 0.00047 | Fall-out or false positive rate |
| G | 0.51393 | 0.62989 | 0.6197 | 0.48894 | 0.52636 | 0.19596 | 0.08111 | G-measure geometric mean of precision and sensitivity |
| GI | 0.40981 | 0.42885 | 0.45125 | 0.40408 | 0.31026 | 0.11363 | 0.01268 | Gini index |
| GM | 0.6904 | 0.71238 | 0.71986 | 0.64463 | 0.55789 | 0.34618 | 0.11468 | G-mean geometric mean of specificity and sensitivity |
| IBA | 0.34104 | 0.45274 | 0.42358 | 0.18447 | 0.09745 | 0.01531 | 0.00018 | Index of balanced accuracy |
| ICSI | 0.03208 | 0.2612 | 0.23974 | -0.01217 | 0.20058 | -0.56113 | -0.48684 | Individual classification success index |
| IS | 1.27656 | 0.79845 | 0.98007 | 3.29964 | 4.65538 | 3.58204 | 3.84417 | Information score |
| J | 0.34393 | 0.45899 | 0.44879 | 0.31928 | 0.3 | 0.09589 | 0.01299 | Jaccard index |
| LS | 2.4226 | 1.73923 | 1.97256 | 9.84672 | 25.20058 | 11.97571 | 14.36184 | Lift score |
| MCC | 0.38359 | 0.42016 | 0.44558 | 0.4625 | 0.51784 | 0.18296 | 0.07685 | Matthews correlation coefficient |
| MCCI | Weak | Weak | Weak | Weak | Moderate | Negligible | Negligible | Matthews correlation coefficient interpretation |
| MCEN | 0.58695 | 0.5383 | 0.55842 | 0.61231 | 0.45592 | 0.66919 | 0.47167 | Modified confusion entropy |
| MK | 0.35905 | 0.41165 | 0.43998 | 0.52936 | 0.86431 | 0.29458 | 0.46561 | Markedness |
| N | 1760 | 1429 | 1513 | 2058 | 2106 | 2125 | 2107 | Condition negative |
| NLR | 0.51626 | 0.44187 | 0.44762 | 0.58771 | 0.68929 | 0.88556 | 0.98731 | Negative likelihood ratio |
| NLRI | Negligible | Poor | Poor | Negligible | Negligible | Negligible | Negligible | Negative likelihood ratio interpretation |
| NPV | 0.88962 | 0.81093 | 0.83457 | 0.96553 | 0.97542 | 0.9764 | 0.96561 | Negative predictive value |
| OC | 0.56265 | 0.66048 | 0.63433 | 0.56383 | 0.88889 | 0.31818 | 0.5 | Overlap coefficient |
| OOC | 0.51393 | 0.62989 | 0.6197 | 0.48894 | 0.52636 | 0.19596 | 0.08111 | Otsuka-Ochiai coefficient |
| OP | 0.59022 | 0.65559 | 0.63506 | 0.55219 | 0.45011 | 0.18652 | -0.00883 | Optimized precision |
| P | 423 | 754 | 670 | 125 | 77 | 58 | 76 | Condition positive or support |
| PLR | 3.68126 | 2.85143 | 3.46476 | 21.28273 | 218.80519 | 17.0977 | 27.72368 | Positive likelihood ratio |
| PLRI | Poor | Poor | Poor | Good | Good | Good | Good | Positive likelihood ratio interpretation |
| POP | 2183 | 2183 | 2183 | 2183 | 2183 | 2183 | 2183 | Population |
| PPV | 0.46943 | 0.60072 | 0.60541 | 0.56383 | 0.88889 | 0.31818 | 0.5 | Precision or positive predictive value |
| PRE | 0.19377 | 0.3454 | 0.30692 | 0.05726 | 0.03527 | 0.02657 | 0.03481 | Prevalence |
| Q | 0.75402 | 0.73165 | 0.77118 | 0.94626 | 0.99372 | 0.90151 | 0.93122 | Yule Q - coefficient of colligation |
| QI | Strong | Moderate | Strong | Strong | Strong | Strong | Strong | Yule Q interpretation |
| RACC | 0.045 | 0.13117 | 0.0987 | 0.00247 | 0.00044 | 0.00027 | 3e-05 | Random accuracy |
| RACCU | 0.04537 | 0.13146 | 0.09875 | 0.00252 | 0.00057 | 0.00034 | 0.00032 | Random accuracy unbiased |
| TN | 1491 | 1098 | 1236 | 2017 | 2103 | 2110 | 2106 | True negative/correct rejection |
| TNR | 0.84716 | 0.76837 | 0.81692 | 0.98008 | 0.99858 | 0.99294 | 0.99953 | Specificity or true negative rate |
| TON | 1676 | 1354 | 1481 | 2089 | 2156 | 2161 | 2181 | Test outcome negative |
| TOP | 507 | 829 | 702 | 94 | 27 | 22 | 2 | Test outcome positive |
| TP | 238 | 498 | 425 | 53 | 24 | 7 | 1 | True positive/hit |
| TPR | 0.56265 | 0.66048 | 0.63433 | 0.424 | 0.31169 | 0.12069 | 0.01316 | Sensitivity, recall, hit rate, or true positive rate |
| Y | 0.40981 | 0.42885 | 0.45125 | 0.40408 | 0.31026 | 0.11363 | 0.01268 | Youden index |
| dInd | 0.46329 | 0.41101 | 0.40894 | 0.57634 | 0.68831 | 0.87934 | 0.98684 | Distance index |
| sInd | 0.6724 | 0.70937 | 0.71083 | 0.59246 | 0.51329 | 0.37821 | 0.3022 | Similarity index |

Generated By PyCM Version 3.1
