# Supplementary material for: TopEC: prediction of Enzyme Commission classes by 3D graph neural networks and localized 3D protein descriptor
Source: Nat Commun. 2025 Mar 20;16:2737. doi: 10.1038/s41467-025-57324-5 (PMC11923149; doi:10.1038/s41467-025-57324-5)
Supplement: Supplementary file 3 — Supplementary Data 1 [file 41467_2025_57324_MOESM3_ESM.zip › Data_S1/table1/mainclass/DeepFRI/full_struc/BindingMOAD_FOLD.html]

DeepFRI\_PDB\_FOLD


# PyCM Report

## Dataset Type :

- Multi-Class Classification
- Imbalanced

Note 1 : Recommended statistics for this type of classification highlighted in aqua

Note 2 : The recommender system assumes that the input is the result of classification over the whole data rather than just a part of it.
If the confusion matrix is the result of test data classification, the recommendation is not valid.

## Confusion Matrix :

|  |  |  |  |  |  |  |  |  |  |  |  |  |  |  |  |  |  |  |  |  |  |  |  |  |  |  |  |  |  |  |  |  |  |  |  |  |  |  |  |  |  |  |  |  |  |  |  |  |  |  |  |  |  |  |  |  |  |  |  |  |  |  |  |  |  |
| --- | --- | --- | --- | --- | --- | --- | --- | --- | --- | --- | --- | --- | --- | --- | --- | --- | --- | --- | --- | --- | --- | --- | --- | --- | --- | --- | --- | --- | --- | --- | --- | --- | --- | --- | --- | --- | --- | --- | --- | --- | --- | --- | --- | --- | --- | --- | --- | --- | --- | --- | --- | --- | --- | --- | --- | --- | --- | --- | --- | --- | --- | --- | --- | --- | --- |
| Actual | Predict  |  |  |  |  |  |  |  |  | | --- | --- | --- | --- | --- | --- | --- | --- | |  | 0 | 1 | 2 | 3 | 4 | 5 | 6 | | 0 | 209 | 130 | 68 | 0 | 0 | 0 | 0 | | 1 | 105 | 640 | 54 | 6 | 0 | 0 | 1 | | 2 | 9 | 69 | 321 | 0 | 0 | 0 | 3 | | 3 | 34 | 37 | 60 | 1 | 0 | 0 | 0 | | 4 | 88 | 47 | 35 | 0 | 0 | 0 | 1 | | 5 | 8 | 23 | 17 | 0 | 0 | 0 | 0 | | 6 | 3 | 4 | 10 | 0 | 0 | 0 | 0 | |

## Overall Statistics :

|  |  |
| --- | --- |
| 95% CI | (0.56888,0.61216) |
| ACC Macro | 0.88301 |
| ARI | 0.28218 |
| AUNP | 0.70989 |
| AUNU | 0.60953 |
| Bangdiwala B | 0.47169 |
| Bennett S | 0.52227 |
| CBA | 0.24396 |
| CSI | None |
| Chi-Squared | None |
| Chi-Squared DF | 36 |
| Conditional Entropy | 1.15394 |
| Cramer V | None |
| Cross Entropy | 1.85022 |
| F1 Macro | 0.27023 |
| F1 Micro | 0.59052 |
| FNR Macro | 0.69805 |
| FNR Micro | 0.40948 |
| FPR Macro | 0.08289 |
| FPR Micro | 0.06825 |
| Gwet AC1 | 0.53697 |
| Hamming Loss | 0.40948 |
| Joint Entropy | 3.37141 |
| KL Divergence | None |
| Kappa | 0.41508 |
| Kappa 95% CI | (0.38417,0.446) |
| Kappa No Prevalence | 0.18104 |
| Kappa Standard Error | 0.01577 |
| Kappa Unbiased | 0.40988 |
| Krippendorff Alpha | 0.41003 |
| Lambda A | 0.31691 |
| Lambda B | 0.38819 |
| Mutual Information | 0.40894 |
| NIR | 0.40645 |
| Overall ACC | 0.59052 |
| Overall CEN | 0.40319 |
| Overall J | (1.3972,0.1996) |
| Overall MCC | 0.42371 |
| Overall MCEN | 0.50426 |
| Overall RACC | 0.29993 |
| Overall RACCU | 0.3061 |
| P-Value | None |
| PPV Macro | None |
| PPV Micro | 0.59052 |
| Pearson C | None |
| Phi-Squared | None |
| RCI | 0.18442 |
| RR | 283.28571 |
| Reference Entropy | 2.21748 |
| Response Entropy | 1.56288 |
| SOA1(Landis & Koch) | Moderate |
| SOA2(Fleiss) | Intermediate to Good |
| SOA3(Altman) | Moderate |
| SOA4(Cicchetti) | Fair |
| SOA5(Cramer) | None |
| SOA6(Matthews) | Weak |
| Scott PI | 0.40988 |
| Standard Error | 0.01104 |
| TNR Macro | 0.91711 |
| TNR Micro | 0.93175 |
| TPR Macro | 0.30195 |
| TPR Micro | 0.59052 |
| Zero-one Loss | 812 |

## Class Statistics :

|  |  |  |  |  |  |  |  |  |
| --- | --- | --- | --- | --- | --- | --- | --- | --- |
| Class | 0 | 1 | 2 | 3 | 4 | 5 | 6 | Description |
| ACC | 0.77559 | 0.75996 | 0.83611 | 0.93091 | 0.91377 | 0.97579 | 0.98891 | Accuracy |
| AGF | 0.65851 | 0.7912 | 0.82509 | 0.09401 | 0.0 | 0.0 | 0.0 | Adjusted F-score |
| AGM | 0.74007 | 0.7543 | 0.83236 | 0.52617 | 0 | 0 | 0 | Adjusted geometric mean |
| AM | 49 | 144 | 163 | -125 | -171 | -48 | -12 | Difference between automatic and manual classification |
| AUC | 0.67839 | 0.76533 | 0.82209 | 0.50217 | 0.5 | 0.5 | 0.49873 | Area under the ROC curve |
| AUCI | Fair | Good | Very Good | Poor | Poor | Poor | Poor | AUC value interpretation |
| AUPR | 0.48592 | 0.73386 | 0.68332 | 0.07522 | None | None | 0.0 | Area under the PR curve |
| BCD | 0.01236 | 0.03631 | 0.0411 | 0.03152 | 0.04312 | 0.0121 | 0.00303 | Bray-Curtis dissimilarity |
| BM | 0.35679 | 0.53066 | 0.64417 | 0.00433 | 0.0 | 0.0 | -0.00254 | Informedness or bookmaker informedness |
| CEN | 0.48795 | 0.34929 | 0.40587 | 0.48092 | 0.4232 | 0.40998 | 0.60072 | Confusion entropy |
| DOR | 5.67949 | 10.78274 | 21.71509 | 2.34733 | None | None | 0.0 | Diagnostic odds ratio |
| DP | 0.41587 | 0.56937 | 0.737 | 0.20431 | None | None | None | Discriminant power |
| DPI | Poor | Poor | Poor | Poor | None | None | None | Discriminant power interpretation |
| ERR | 0.22441 | 0.24004 | 0.16389 | 0.06909 | 0.08623 | 0.02421 | 0.01109 | Error rate |
| F0.5 | 0.4684 | 0.69475 | 0.60293 | 0.03125 | 0.0 | 0.0 | 0.0 | F0.5 score |
| F1 | 0.48436 | 0.72893 | 0.66391 | 0.01439 | 0.0 | 0.0 | 0.0 | F1 score - harmonic mean of precision and sensitivity |
| F2 | 0.50144 | 0.76665 | 0.73861 | 0.00935 | 0.0 | 0.0 | 0.0 | F2 score |
| FDR | 0.54167 | 0.32632 | 0.43186 | 0.85714 | None | None | 1.0 | False discovery rate |
| FN | 198 | 166 | 81 | 131 | 171 | 48 | 17 | False negative/miss/type 2 error |
| FNR | 0.48649 | 0.20596 | 0.20149 | 0.99242 | 1.0 | 1.0 | 1.0 | Miss rate or false negative rate |
| FOR | 0.12967 | 0.1607 | 0.05712 | 0.0663 | 0.08623 | 0.02421 | 0.00859 | False omission rate |
| FP | 247 | 310 | 244 | 6 | 0 | 0 | 5 | False positive/type 1 error/false alarm |
| FPR | 0.15673 | 0.26338 | 0.15433 | 0.00324 | 0.0 | 0.0 | 0.00254 | Fall-out or false positive rate |
| G | 0.48514 | 0.73139 | 0.67355 | 0.0329 | None | None | 0.0 | G-measure geometric mean of precision and sensitivity |
| GI | 0.35679 | 0.53066 | 0.64417 | 0.00433 | 0.0 | 0.0 | -0.00254 | Gini index |
| GM | 0.65805 | 0.76479 | 0.82175 | 0.0869 | 0.0 | 0.0 | 0.0 | G-mean geometric mean of specificity and sensitivity |
| IBA | 0.29024 | 0.6185 | 0.64343 | 8e-05 | 0.0 | 0.0 | 0.0 | Index of balanced accuracy |
| ICSI | -0.02815 | 0.46773 | 0.36665 | -0.84957 | None | None | -1.0 | Individual classification success index |
| IS | 1.15905 | 0.72898 | 1.48674 | 1.10172 | None | None | None | Information score |
| J | 0.31957 | 0.57348 | 0.4969 | 0.00725 | 0.0 | 0.0 | 0.0 | Jaccard index |
| LS | 2.23311 | 1.65746 | 2.80255 | 2.1461 | None | None | 0.0 | Lift score |
| MCC | 0.34244 | 0.52175 | 0.57375 | 0.01822 | None | None | -0.00468 | Matthews correlation coefficient |
| MCCI | Weak | Moderate | Moderate | Negligible | None | None | Negligible | Matthews correlation coefficient interpretation |
| MCEN | 0.56796 | 0.47179 | 0.52588 | 0.48152 | 0.4232 | 0.40998 | 0.60072 | Modified confusion entropy |
| MK | 0.32867 | 0.51299 | 0.51102 | 0.07656 | None | None | -0.00859 | Markedness |
| N | 1576 | 1177 | 1581 | 1851 | 1812 | 1935 | 1966 | Condition negative |
| NLR | 0.5769 | 0.2796 | 0.23826 | 0.99565 | 1.0 | 1.0 | 1.00255 | Negative likelihood ratio |
| NLRI | Negligible | Poor | Poor | Negligible | Negligible | Negligible | Negligible | Negative likelihood ratio interpretation |
| NPV | 0.87033 | 0.8393 | 0.94288 | 0.9337 | 0.91377 | 0.97579 | 0.99141 | Negative predictive value |
| OC | 0.51351 | 0.79404 | 0.79851 | 0.14286 | None | None | 0.0 | Overlap coefficient |
| OOC | 0.48514 | 0.73139 | 0.67355 | 0.0329 | None | None | 0.0 | Otsuka-Ochiai coefficient |
| OP | 0.53255 | 0.72244 | 0.80742 | -0.054 | -0.08623 | -0.02421 | -0.01109 | Optimized precision |
| P | 407 | 806 | 402 | 132 | 171 | 48 | 17 | Condition positive or support |
| PLR | 3.27651 | 3.01481 | 5.17394 | 2.33712 | None | None | 0.0 | Positive likelihood ratio |
| PLRI | Poor | Poor | Fair | Poor | None | None | Negligible | Positive likelihood ratio interpretation |
| POP | 1983 | 1983 | 1983 | 1983 | 1983 | 1983 | 1983 | Population |
| PPV | 0.45833 | 0.67368 | 0.56814 | 0.14286 | None | None | 0.0 | Precision or positive predictive value |
| PRE | 0.20524 | 0.40645 | 0.20272 | 0.06657 | 0.08623 | 0.02421 | 0.00857 | Prevalence |
| Q | 0.70058 | 0.83026 | 0.91195 | 0.40251 | None | None | -1.0 | Yule Q - coefficient of colligation |
| QI | Moderate | Strong | Strong | Weak | None | None | Negligible | Yule Q interpretation |
| RACC | 0.0472 | 0.19472 | 0.05776 | 0.00023 | 0.0 | 0.0 | 2e-05 | Random accuracy |
| RACCU | 0.04735 | 0.19604 | 0.05945 | 0.00123 | 0.00186 | 0.00015 | 3e-05 | Random accuracy unbiased |
| TN | 1329 | 867 | 1337 | 1845 | 1812 | 1935 | 1961 | True negative/correct rejection |
| TNR | 0.84327 | 0.73662 | 0.84567 | 0.99676 | 1.0 | 1.0 | 0.99746 | Specificity or true negative rate |
| TON | 1527 | 1033 | 1418 | 1976 | 1983 | 1983 | 1978 | Test outcome negative |
| TOP | 456 | 950 | 565 | 7 | 0 | 0 | 5 | Test outcome positive |
| TP | 209 | 640 | 321 | 1 | 0 | 0 | 0 | True positive/hit |
| TPR | 0.51351 | 0.79404 | 0.79851 | 0.00758 | 0.0 | 0.0 | 0.0 | Sensitivity, recall, hit rate, or true positive rate |
| Y | 0.35679 | 0.53066 | 0.64417 | 0.00433 | 0.0 | 0.0 | -0.00254 | Youden index |
| dInd | 0.51111 | 0.33435 | 0.25381 | 0.99243 | 1.0 | 1.0 | 1.0 | Distance index |
| sInd | 0.63859 | 0.76358 | 0.82053 | 0.29825 | 0.29289 | 0.29289 | 0.29289 | Similarity index |

Generated By PyCM Version 3.1
