# Supplementary material for: TopEC: prediction of Enzyme Commission classes by 3D graph neural networks and localized 3D protein descriptor
Source: Nat Commun. 2025 Mar 20;16:2737. doi: 10.1038/s41467-025-57324-5 (PMC11923149; doi:10.1038/s41467-025-57324-5)
Supplement: Supplementary file 3 — Supplementary Data 1 [file 41467_2025_57324_MOESM3_ESM.zip › Data_S1/table1/mainclass/EnzyNet/local/TopEnzyme_TEMP_wflips.html]

TopM\_TEMP\_enzynet\_wflips\_sites


# PyCM Report

## Dataset Type :

- Multi-Class Classification
- Imbalanced

Note 1 : Recommended statistics for this type of classification highlighted in aqua

Note 2 : The recommender system assumes that the input is the result of classification over the whole data rather than just a part of it.
If the confusion matrix is the result of test data classification, the recommendation is not valid.

## Confusion Matrix :

|  |  |  |  |  |  |  |  |  |  |  |  |  |  |  |  |  |  |  |  |  |  |  |  |  |  |  |  |  |  |  |  |  |  |  |  |  |  |  |  |  |  |  |  |  |  |  |  |  |  |  |  |  |  |  |  |  |  |  |  |  |  |  |  |  |  |
| --- | --- | --- | --- | --- | --- | --- | --- | --- | --- | --- | --- | --- | --- | --- | --- | --- | --- | --- | --- | --- | --- | --- | --- | --- | --- | --- | --- | --- | --- | --- | --- | --- | --- | --- | --- | --- | --- | --- | --- | --- | --- | --- | --- | --- | --- | --- | --- | --- | --- | --- | --- | --- | --- | --- | --- | --- | --- | --- | --- | --- | --- | --- | --- | --- | --- |
| Actual | Predict  |  |  |  |  |  |  |  |  | | --- | --- | --- | --- | --- | --- | --- | --- | |  | 0 | 1 | 2 | 3 | 4 | 5 | 6 | | 0 | 94 | 50 | 62 | 5 | 2 | 2 | 1 | | 1 | 23 | 108 | 89 | 9 | 1 | 2 | 0 | | 2 | 29 | 44 | 137 | 3 | 0 | 1 | 0 | | 3 | 14 | 22 | 24 | 10 | 0 | 1 | 1 | | 4 | 6 | 9 | 11 | 2 | 4 | 0 | 0 | | 5 | 5 | 10 | 7 | 1 | 0 | 8 | 0 | | 6 | 26 | 22 | 35 | 1 | 0 | 0 | 8 | |

## Overall Statistics :

|  |  |
| --- | --- |
| 95% CI | (0.38268,0.44746) |
| ACC Macro | 0.83288 |
| ARI | 0.07146 |
| AUNP | 0.61575 |
| AUNU | 0.59866 |
| Bangdiwala B | 0.21249 |
| Bennett S | 0.31759 |
| CBA | 0.261 |
| CSI | -0.18925 |
| Chi-Squared | 403.85593 |
| Chi-Squared DF | 36 |
| Conditional Entropy | 1.72313 |
| Cramer V | 0.27516 |
| Cross Entropy | 2.81558 |
| F1 Macro | 0.32497 |
| F1 Micro | 0.41507 |
| FNR Macro | 0.69289 |
| FNR Micro | 0.58493 |
| FPR Macro | 0.10979 |
| FPR Micro | 0.09749 |
| Gwet AC1 | 0.33099 |
| Hamming Loss | 0.58493 |
| Joint Entropy | 4.19323 |
| KL Divergence | 0.34549 |
| Kappa | 0.2351 |
| Kappa 95% CI | (0.19275,0.27746) |
| Kappa No Prevalence | -0.16985 |
| Kappa Standard Error | 0.02161 |
| Kappa Unbiased | 0.22433 |
| Krippendorff Alpha | 0.22477 |
| Lambda A | 0.20852 |
| Lambda B | 0.10305 |
| Mutual Information | 0.19743 |
| NIR | 0.26097 |
| Overall ACC | 0.41507 |
| Overall CEN | 0.56103 |
| Overall J | (1.4054,0.20077) |
| Overall MCC | 0.2424 |
| Overall MCEN | 0.64117 |
| Overall RACC | 0.23529 |
| Overall RACCU | 0.24591 |
| P-Value | -0.0 |
| PPV Macro | 0.50364 |
| PPV Micro | 0.41507 |
| Pearson C | 0.55891 |
| Phi-Squared | 0.45428 |
| RCI | 0.07993 |
| RR | 127.0 |
| Reference Entropy | 2.4701 |
| Response Entropy | 1.92056 |
| SOA1(Landis & Koch) | Fair |
| SOA2(Fleiss) | Poor |
| SOA3(Altman) | Fair |
| SOA4(Cicchetti) | Poor |
| SOA5(Cramer) | Moderate |
| SOA6(Matthews) | Negligible |
| Scott PI | 0.22433 |
| Standard Error | 0.01653 |
| TNR Macro | 0.89021 |
| TNR Micro | 0.90251 |
| TPR Macro | 0.30711 |
| TPR Micro | 0.41507 |
| Zero-one Loss | 520 |

## Class Statistics :

|  |  |  |  |  |  |  |  |  |
| --- | --- | --- | --- | --- | --- | --- | --- | --- |
| Class | 0 | 1 | 2 | 3 | 4 | 5 | 6 | Description |
| ACC | 0.74691 | 0.68391 | 0.65692 | 0.90664 | 0.96513 | 0.96738 | 0.90326 | Accuracy |
| AGF | 0.60572 | 0.59908 | 0.67268 | 0.38317 | 0.37982 | 0.53229 | 0.31229 | Adjusted F-score |
| AGM | 0.71045 | 0.66568 | 0.65591 | 0.65828 | 0.66882 | 0.7453 | 0.62682 | Adjusted geometric mean |
| AM | -19 | 33 | 151 | -41 | -25 | -17 | -82 | Difference between automatic and manual classification |
| AUC | 0.64107 | 0.61328 | 0.6512 | 0.55659 | 0.56075 | 0.62554 | 0.54222 | Area under the ROC curve |
| AUCI | Fair | Fair | Fair | Poor | Poor | Fair | Poor | AUC value interpretation |
| AUPR | 0.45617 | 0.43653 | 0.50776 | 0.23073 | 0.34821 | 0.41475 | 0.44348 | Area under the PR curve |
| BCD | 0.01069 | 0.01856 | 0.08493 | 0.02306 | 0.01406 | 0.00956 | 0.04612 | Bray-Curtis dissimilarity |
| BM | 0.28214 | 0.22655 | 0.30241 | 0.11319 | 0.1215 | 0.25107 | 0.08445 | Informedness or bookmaker informedness |
| CEN | 0.56783 | 0.57566 | 0.53982 | 0.66794 | 0.55613 | 0.56274 | 0.4758 | Confusion entropy |
| DOR | 4.26389 | 2.77378 | 3.48821 | 6.11367 | 40.66667 | 49.3913 | 37.85714 | Diagnostic odds ratio |
| DP | 0.34723 | 0.24428 | 0.29915 | 0.43351 | 0.88722 | 0.93376 | 0.87008 | Discriminant power |
| DPI | Poor | Poor | Poor | Poor | Poor | Poor | Poor | Discriminant power interpretation |
| ERR | 0.25309 | 0.31609 | 0.34308 | 0.09336 | 0.03487 | 0.03262 | 0.09674 | Error rate |
| F0.5 | 0.46813 | 0.41796 | 0.4092 | 0.2551 | 0.33333 | 0.45977 | 0.30303 | F0.5 score |
| F1 | 0.45521 | 0.43461 | 0.47323 | 0.19417 | 0.20513 | 0.35556 | 0.15686 | F1 score - harmonic mean of precision and sensitivity |
| F2 | 0.44298 | 0.45264 | 0.56102 | 0.15674 | 0.14815 | 0.28986 | 0.10582 | F2 score |
| FDR | 0.52284 | 0.59245 | 0.62466 | 0.67742 | 0.42857 | 0.42857 | 0.2 | False discovery rate |
| FN | 122 | 124 | 77 | 62 | 28 | 23 | 84 | False negative/miss/type 2 error |
| FNR | 0.56481 | 0.53448 | 0.35981 | 0.86111 | 0.875 | 0.74194 | 0.91304 | Miss rate or false negative rate |
| FOR | 0.1763 | 0.19872 | 0.14695 | 0.07226 | 0.03175 | 0.02629 | 0.09556 | False omission rate |
| FP | 103 | 157 | 228 | 21 | 3 | 6 | 2 | False positive/type 1 error/false alarm |
| FPR | 0.15305 | 0.23896 | 0.33778 | 0.0257 | 0.0035 | 0.00699 | 0.00251 | Fall-out or false positive rate |
| G | 0.45569 | 0.43557 | 0.49019 | 0.21167 | 0.26726 | 0.38401 | 0.26375 | G-measure geometric mean of precision and sensitivity |
| GI | 0.28214 | 0.22655 | 0.30241 | 0.11319 | 0.1215 | 0.25107 | 0.08445 | Gini index |
| GM | 0.60711 | 0.59521 | 0.65111 | 0.36786 | 0.35293 | 0.50622 | 0.29451 | G-mean geometric mean of specificity and sensitivity |
| IBA | 0.21681 | 0.24958 | 0.4146 | 0.02227 | 0.01601 | 0.06792 | 0.00776 | Index of balanced accuracy |
| ICSI | -0.08766 | -0.12694 | 0.01553 | -0.53853 | -0.30357 | -0.17051 | -0.11304 | Individual classification success index |
| IS | 0.97369 | 0.6431 | 0.64085 | 1.99385 | 3.98868 | 4.03449 | 2.95055 | Information score |
| J | 0.29467 | 0.27763 | 0.30995 | 0.10753 | 0.11429 | 0.21622 | 0.08511 | Jaccard index |
| LS | 1.96386 | 1.56168 | 1.55925 | 3.98297 | 15.875 | 16.3871 | 7.73043 | Lift score |
| MCC | 0.29135 | 0.21751 | 0.26281 | 0.16832 | 0.25607 | 0.36996 | 0.2439 | Matthews correlation coefficient |
| MCCI | Negligible | Negligible | Negligible | Negligible | Negligible | Weak | Negligible | Matthews correlation coefficient interpretation |
| MCEN | 0.66185 | 0.66425 | 0.63216 | 0.70308 | 0.58112 | 0.62267 | 0.48622 | Modified confusion entropy |
| MK | 0.30086 | 0.20883 | 0.2284 | 0.25032 | 0.53968 | 0.54514 | 0.70444 | Markedness |
| N | 673 | 657 | 675 | 817 | 857 | 858 | 797 | Condition negative |
| NLR | 0.66688 | 0.70231 | 0.54334 | 0.88383 | 0.87807 | 0.74716 | 0.91534 | Negative likelihood ratio |
| NLRI | Negligible | Negligible | Negligible | Negligible | Negligible | Negligible | Negligible | Negative likelihood ratio interpretation |
| NPV | 0.8237 | 0.80128 | 0.85305 | 0.92774 | 0.96825 | 0.97371 | 0.90444 | Negative predictive value |
| OC | 0.47716 | 0.46552 | 0.64019 | 0.32258 | 0.57143 | 0.57143 | 0.8 | Overlap coefficient |
| OOC | 0.45569 | 0.43557 | 0.49019 | 0.21167 | 0.26726 | 0.38401 | 0.26375 | Otsuka-Ochiai coefficient |
| OP | 0.42575 | 0.44298 | 0.64 | 0.15617 | 0.18805 | 0.37993 | 0.06363 | Optimized precision |
| P | 216 | 232 | 214 | 72 | 32 | 31 | 92 | Condition positive or support |
| PLR | 2.84349 | 1.94806 | 1.89529 | 5.40344 | 35.70833 | 36.90323 | 34.65217 | Positive likelihood ratio |
| PLRI | Poor | Poor | Poor | Fair | Good | Good | Good | Positive likelihood ratio interpretation |
| POP | 889 | 889 | 889 | 889 | 889 | 889 | 889 | Population |
| PPV | 0.47716 | 0.40755 | 0.37534 | 0.32258 | 0.57143 | 0.57143 | 0.8 | Precision or positive predictive value |
| PRE | 0.24297 | 0.26097 | 0.24072 | 0.08099 | 0.036 | 0.03487 | 0.10349 | Prevalence |
| Q | 0.62005 | 0.47003 | 0.55439 | 0.71885 | 0.952 | 0.96031 | 0.94853 | Yule Q - coefficient of colligation |
| QI | Moderate | Weak | Moderate | Moderate | Strong | Strong | Strong | Yule Q interpretation |
| RACC | 0.05384 | 0.07779 | 0.09883 | 0.00282 | 0.00028 | 0.00055 | 0.00116 | Random accuracy |
| RACCU | 0.05396 | 0.07814 | 0.10605 | 0.00336 | 0.00048 | 0.00064 | 0.00329 | Random accuracy unbiased |
| TN | 570 | 500 | 447 | 796 | 854 | 852 | 795 | True negative/correct rejection |
| TNR | 0.84695 | 0.76104 | 0.66222 | 0.9743 | 0.9965 | 0.99301 | 0.99749 | Specificity or true negative rate |
| TON | 692 | 624 | 524 | 858 | 882 | 875 | 879 | Test outcome negative |
| TOP | 197 | 265 | 365 | 31 | 7 | 14 | 10 | Test outcome positive |
| TP | 94 | 108 | 137 | 10 | 4 | 8 | 8 | True positive/hit |
| TPR | 0.43519 | 0.46552 | 0.64019 | 0.13889 | 0.125 | 0.25806 | 0.08696 | Sensitivity, recall, hit rate, or true positive rate |
| Y | 0.28214 | 0.22655 | 0.30241 | 0.11319 | 0.1215 | 0.25107 | 0.08445 | Youden index |
| dInd | 0.58518 | 0.58547 | 0.49352 | 0.86149 | 0.87501 | 0.74197 | 0.91305 | Distance index |
| sInd | 0.58621 | 0.58601 | 0.65103 | 0.39083 | 0.38128 | 0.47535 | 0.35438 | Similarity index |

Generated By PyCM Version 3.1
