# Supplementary material for: TopEC: prediction of Enzyme Commission classes by 3D graph neural networks and localized 3D protein descriptor
Source: Nat Commun. 2025 Mar 20;16:2737. doi: 10.1038/s41467-025-57324-5 (PMC11923149; doi:10.1038/s41467-025-57324-5)
Supplement: Supplementary file 3 — Supplementary Data 1 [file 41467_2025_57324_MOESM3_ESM.zip › Data_S1/table1/mainclass/EnzyNet/local/TopEnzyme_TEMP_flips.html]

TopM\_TEMP\_enzynet\_flips\_sites


# PyCM Report

## Dataset Type :

- Multi-Class Classification
- Imbalanced

Note 1 : Recommended statistics for this type of classification highlighted in aqua

Note 2 : The recommender system assumes that the input is the result of classification over the whole data rather than just a part of it.
If the confusion matrix is the result of test data classification, the recommendation is not valid.

## Confusion Matrix :

|  |  |  |  |  |  |  |  |  |  |  |  |  |  |  |  |  |  |  |  |  |  |  |  |  |  |  |  |  |  |  |  |  |  |  |  |  |  |  |  |  |  |  |  |  |  |  |  |  |  |  |  |  |  |  |  |  |  |  |  |  |  |  |  |  |  |
| --- | --- | --- | --- | --- | --- | --- | --- | --- | --- | --- | --- | --- | --- | --- | --- | --- | --- | --- | --- | --- | --- | --- | --- | --- | --- | --- | --- | --- | --- | --- | --- | --- | --- | --- | --- | --- | --- | --- | --- | --- | --- | --- | --- | --- | --- | --- | --- | --- | --- | --- | --- | --- | --- | --- | --- | --- | --- | --- | --- | --- | --- | --- | --- | --- | --- |
| Actual | Predict  |  |  |  |  |  |  |  |  | | --- | --- | --- | --- | --- | --- | --- | --- | |  | 0 | 1 | 2 | 3 | 4 | 5 | 6 | | 0 | 89 | 51 | 67 | 4 | 3 | 1 | 1 | | 1 | 16 | 111 | 96 | 7 | 0 | 2 | 0 | | 2 | 28 | 38 | 144 | 3 | 0 | 1 | 0 | | 3 | 12 | 23 | 28 | 8 | 0 | 0 | 1 | | 4 | 7 | 11 | 10 | 1 | 3 | 0 | 0 | | 5 | 5 | 11 | 7 | 1 | 0 | 7 | 0 | | 6 | 25 | 25 | 33 | 0 | 0 | 0 | 9 | |

## Overall Statistics :

|  |  |
| --- | --- |
| 95% CI | (0.38491,0.44974) |
| ACC Macro | 0.83352 |
| ARI | 0.07437 |
| AUNP | 0.61626 |
| AUNU | 0.5946 |
| Bangdiwala B | 0.2195 |
| Bennett S | 0.32021 |
| CBA | 0.24652 |
| CSI | -0.1923 |
| Chi-Squared | 400.9631 |
| Chi-Squared DF | 36 |
| Conditional Entropy | 1.6504 |
| Cramer V | 0.27417 |
| Cross Entropy | 2.85359 |
| F1 Macro | 0.3147 |
| F1 Micro | 0.41732 |
| FNR Macro | 0.70116 |
| FNR Micro | 0.58268 |
| FPR Macro | 0.10964 |
| FPR Micro | 0.09711 |
| Gwet AC1 | 0.33416 |
| Hamming Loss | 0.58268 |
| Joint Entropy | 4.1205 |
| KL Divergence | 0.3835 |
| Kappa | 0.23594 |
| Kappa 95% CI | (0.19344,0.27845) |
| Kappa No Prevalence | -0.16535 |
| Kappa Standard Error | 0.02169 |
| Kappa Unbiased | 0.22247 |
| Krippendorff Alpha | 0.2229 |
| Lambda A | 0.21157 |
| Lambda B | 0.08333 |
| Mutual Information | 0.20923 |
| NIR | 0.26097 |
| Overall ACC | 0.41732 |
| Overall CEN | 0.54664 |
| Overall J | (1.36076,0.19439) |
| Overall MCC | 0.2452 |
| Overall MCEN | 0.62395 |
| Overall RACC | 0.23739 |
| Overall RACCU | 0.25061 |
| P-Value | -0.0 |
| PPV Macro | 0.50886 |
| PPV Micro | 0.41732 |
| Pearson C | 0.55752 |
| Phi-Squared | 0.45103 |
| RCI | 0.0847 |
| RR | 127.0 |
| Reference Entropy | 2.4701 |
| Response Entropy | 1.85963 |
| SOA1(Landis & Koch) | Fair |
| SOA2(Fleiss) | Poor |
| SOA3(Altman) | Fair |
| SOA4(Cicchetti) | Poor |
| SOA5(Cramer) | Moderate |
| SOA6(Matthews) | Negligible |
| Scott PI | 0.22247 |
| Standard Error | 0.01654 |
| TNR Macro | 0.89036 |
| TNR Micro | 0.90289 |
| TPR Macro | 0.29884 |
| TPR Micro | 0.41732 |
| Zero-one Loss | 518 |

## Class Statistics :

|  |  |  |  |  |  |  |  |  |
| --- | --- | --- | --- | --- | --- | --- | --- | --- |
| Class | 0 | 1 | 2 | 3 | 4 | 5 | 6 | Description |
| ACC | 0.75253 | 0.68504 | 0.65017 | 0.91001 | 0.964 | 0.9685 | 0.90439 | Accuracy |
| AGF | 0.59363 | 0.60679 | 0.684 | 0.34648 | 0.33001 | 0.50332 | 0.33095 | Adjusted F-score |
| AGM | 0.71047 | 0.66841 | 0.65137 | 0.64151 | 0.64474 | 0.73009 | 0.63624 | Adjusted geometric mean |
| AM | -34 | 38 | 171 | -48 | -26 | -20 | -81 | Difference between automatic and manual classification |
| AUC | 0.63692 | 0.61822 | 0.65793 | 0.54576 | 0.54512 | 0.61057 | 0.54766 | Area under the ROC curve |
| AUCI | Fair | Fair | Fair | Poor | Poor | Fair | Poor | AUC value interpretation |
| AUPR | 0.45052 | 0.44478 | 0.52346 | 0.22222 | 0.29688 | 0.43109 | 0.458 | Area under the PR curve |
| BCD | 0.01912 | 0.02137 | 0.09618 | 0.027 | 0.01462 | 0.01125 | 0.04556 | Bray-Curtis dissimilarity |
| BM | 0.27385 | 0.23644 | 0.31586 | 0.09153 | 0.09025 | 0.22114 | 0.09532 | Informedness or bookmaker informedness |
| CEN | 0.56251 | 0.56081 | 0.52999 | 0.6181 | 0.53039 | 0.52913 | 0.45956 | Confusion entropy |
| DOR | 4.3705 | 2.87323 | 3.70456 | 6.25781 | 29.44828 | 62.27083 | 43.10241 | Diagnostic odds ratio |
| DP | 0.35314 | 0.25271 | 0.31356 | 0.43909 | 0.80994 | 0.98924 | 0.90115 | Discriminant power |
| DPI | Poor | Poor | Poor | Poor | Poor | Poor | Poor | Discriminant power interpretation |
| ERR | 0.24747 | 0.31496 | 0.34983 | 0.08999 | 0.036 | 0.0315 | 0.09561 | Error rate |
| F0.5 | 0.4714 | 0.42302 | 0.41049 | 0.2381 | 0.26786 | 0.46667 | 0.33088 | F0.5 score |
| F1 | 0.44724 | 0.44223 | 0.4808 | 0.16667 | 0.15789 | 0.33333 | 0.17476 | F1 score - harmonic mean of precision and sensitivity |
| F2 | 0.42543 | 0.46327 | 0.58018 | 0.12821 | 0.11194 | 0.25926 | 0.11873 | F2 score |
| FDR | 0.51099 | 0.58889 | 0.62597 | 0.66667 | 0.5 | 0.36364 | 0.18182 | False discovery rate |
| FN | 127 | 121 | 70 | 64 | 29 | 24 | 83 | False negative/miss/type 2 error |
| FNR | 0.58796 | 0.52155 | 0.3271 | 0.88889 | 0.90625 | 0.77419 | 0.90217 | Miss rate or false negative rate |
| FOR | 0.17963 | 0.19548 | 0.13889 | 0.07399 | 0.03284 | 0.02733 | 0.09453 | False omission rate |
| FP | 93 | 159 | 241 | 16 | 3 | 4 | 2 | False positive/type 1 error/false alarm |
| FPR | 0.13819 | 0.24201 | 0.35704 | 0.01958 | 0.0035 | 0.00466 | 0.00251 | Fall-out or false positive rate |
| G | 0.44888 | 0.4435 | 0.50168 | 0.19245 | 0.21651 | 0.37907 | 0.28291 | G-measure geometric mean of precision and sensitivity |
| GI | 0.27385 | 0.23644 | 0.31586 | 0.09153 | 0.09025 | 0.22114 | 0.09532 | Gini index |
| GM | 0.5959 | 0.60221 | 0.65776 | 0.33005 | 0.30565 | 0.47408 | 0.31238 | G-mean geometric mean of specificity and sensitivity |
| IBA | 0.19538 | 0.26128 | 0.4456 | 0.01424 | 0.00909 | 0.0518 | 0.00979 | Index of balanced accuracy |
| ICSI | -0.09895 | -0.11044 | 0.04692 | -0.55556 | -0.40625 | -0.13783 | -0.08399 | Individual classification success index |
| IS | 1.00909 | 0.65566 | 0.63578 | 2.04115 | 3.79604 | 4.18977 | 2.98297 | Information score |
| J | 0.28803 | 0.28389 | 0.31648 | 0.09091 | 0.08571 | 0.2 | 0.09574 | Jaccard index |
| LS | 2.01264 | 1.57534 | 1.55378 | 4.11574 | 13.89062 | 18.24927 | 7.90613 | Lift score |
| MCC | 0.29107 | 0.2258 | 0.27253 | 0.15407 | 0.20533 | 0.36699 | 0.26263 | Matthews correlation coefficient |
| MCCI | Negligible | Negligible | Negligible | Negligible | Negligible | Weak | Negligible | Matthews correlation coefficient interpretation |
| MCEN | 0.65201 | 0.648 | 0.6221 | 0.64246 | 0.54559 | 0.57626 | 0.47029 | Modified confusion entropy |
| MK | 0.30938 | 0.21563 | 0.23514 | 0.25934 | 0.46716 | 0.60903 | 0.72365 | Markedness |
| N | 673 | 657 | 675 | 817 | 857 | 858 | 797 | Condition negative |
| NLR | 0.68224 | 0.68807 | 0.50874 | 0.90664 | 0.90943 | 0.77782 | 0.90444 | Negative likelihood ratio |
| NLRI | Negligible | Negligible | Negligible | Negligible | Negligible | Negligible | Negligible | Negative likelihood ratio interpretation |
| NPV | 0.82037 | 0.80452 | 0.86111 | 0.92601 | 0.96716 | 0.97267 | 0.90547 | Negative predictive value |
| OC | 0.48901 | 0.47845 | 0.6729 | 0.33333 | 0.5 | 0.63636 | 0.81818 | Overlap coefficient |
| OOC | 0.44888 | 0.4435 | 0.50168 | 0.19245 | 0.21651 | 0.37907 | 0.28291 | Otsuka-Ochiai coefficient |
| OP | 0.39945 | 0.45895 | 0.62742 | 0.1136 | 0.13598 | 0.33833 | 0.08301 | Optimized precision |
| P | 216 | 232 | 214 | 72 | 32 | 31 | 92 | Condition positive or support |
| PLR | 2.98173 | 1.97698 | 1.88467 | 5.67361 | 26.78125 | 48.43548 | 38.9837 | Positive likelihood ratio |
| PLRI | Poor | Poor | Poor | Fair | Good | Good | Good | Positive likelihood ratio interpretation |
| POP | 889 | 889 | 889 | 889 | 889 | 889 | 889 | Population |
| PPV | 0.48901 | 0.41111 | 0.37403 | 0.33333 | 0.5 | 0.63636 | 0.81818 | Precision or positive predictive value |
| PRE | 0.24297 | 0.26097 | 0.24072 | 0.08099 | 0.036 | 0.03487 | 0.10349 | Prevalence |
| Q | 0.6276 | 0.48363 | 0.57488 | 0.72443 | 0.93431 | 0.96839 | 0.95465 | Yule Q - coefficient of colligation |
| QI | Moderate | Weak | Moderate | Moderate | Strong | Strong | Strong | Yule Q interpretation |
| RACC | 0.04974 | 0.07926 | 0.10425 | 0.00219 | 0.00024 | 0.00043 | 0.00128 | Random accuracy |
| RACCU | 0.05011 | 0.07972 | 0.1135 | 0.00292 | 0.00046 | 0.00056 | 0.00336 | Random accuracy unbiased |
| TN | 580 | 498 | 434 | 801 | 854 | 854 | 795 | True negative/correct rejection |
| TNR | 0.86181 | 0.75799 | 0.64296 | 0.98042 | 0.9965 | 0.99534 | 0.99749 | Specificity or true negative rate |
| TON | 707 | 619 | 504 | 865 | 883 | 878 | 878 | Test outcome negative |
| TOP | 182 | 270 | 385 | 24 | 6 | 11 | 11 | Test outcome positive |
| TP | 89 | 111 | 144 | 8 | 3 | 7 | 9 | True positive/hit |
| TPR | 0.41204 | 0.47845 | 0.6729 | 0.11111 | 0.09375 | 0.22581 | 0.09783 | Sensitivity, recall, hit rate, or true positive rate |
| Y | 0.27385 | 0.23644 | 0.31586 | 0.09153 | 0.09025 | 0.22114 | 0.09532 | Youden index |
| dInd | 0.60398 | 0.57496 | 0.48422 | 0.8891 | 0.90626 | 0.77421 | 0.90218 | Distance index |
| sInd | 0.57292 | 0.59344 | 0.6576 | 0.37131 | 0.35918 | 0.45255 | 0.36206 | Similarity index |

Generated By PyCM Version 3.1
