# Supplementary material for: TopEC: prediction of Enzyme Commission classes by 3D graph neural networks and localized 3D protein descriptor
Source: Nat Commun. 2025 Mar 20;16:2737. doi: 10.1038/s41467-025-57324-5 (PMC11923149; doi:10.1038/s41467-025-57324-5)
Supplement: Supplementary file 3 — Supplementary Data 1 [file 41467_2025_57324_MOESM3_ESM.zip › Data_S1/table1/mainclass/EnzyNet/local/TopEnzyme_TEMP.html]

TopM\_TEMP\_enzynet\_none\_sites


# PyCM Report

## Dataset Type :

- Multi-Class Classification
- Imbalanced

Note 1 : Recommended statistics for this type of classification highlighted in aqua

Note 2 : The recommender system assumes that the input is the result of classification over the whole data rather than just a part of it.
If the confusion matrix is the result of test data classification, the recommendation is not valid.

## Confusion Matrix :

|  |  |  |  |  |  |  |  |  |  |  |  |  |  |  |  |  |  |  |  |  |  |  |  |  |  |  |  |  |  |  |  |  |  |  |  |  |  |  |  |  |  |  |  |  |  |  |  |  |  |  |  |  |  |  |  |  |  |  |  |  |  |  |  |  |  |
| --- | --- | --- | --- | --- | --- | --- | --- | --- | --- | --- | --- | --- | --- | --- | --- | --- | --- | --- | --- | --- | --- | --- | --- | --- | --- | --- | --- | --- | --- | --- | --- | --- | --- | --- | --- | --- | --- | --- | --- | --- | --- | --- | --- | --- | --- | --- | --- | --- | --- | --- | --- | --- | --- | --- | --- | --- | --- | --- | --- | --- | --- | --- | --- | --- | --- |
| Actual | Predict  |  |  |  |  |  |  |  |  | | --- | --- | --- | --- | --- | --- | --- | --- | |  | 0 | 1 | 2 | 3 | 4 | 5 | 6 | | 0 | 68 | 62 | 60 | 18 | 1 | 6 | 1 | | 1 | 35 | 103 | 70 | 15 | 3 | 6 | 0 | | 2 | 33 | 49 | 118 | 6 | 2 | 6 | 0 | | 3 | 14 | 20 | 20 | 14 | 1 | 3 | 0 | | 4 | 6 | 8 | 6 | 5 | 7 | 0 | 0 | | 5 | 5 | 12 | 5 | 3 | 1 | 5 | 0 | | 6 | 27 | 18 | 33 | 4 | 1 | 2 | 7 | |

## Overall Statistics :

|  |  |
| --- | --- |
| 95% CI | (0.33061,0.3938) |
| ACC Macro | 0.81777 |
| ARI | 0.04774 |
| AUNP | 0.58804 |
| AUNU | 0.5812 |
| Bangdiwala B | 0.16627 |
| Bennett S | 0.25591 |
| CBA | 0.24604 |
| CSI | -0.31632 |
| Chi-Squared | 252.80304 |
| Chi-Squared DF | 36 |
| Conditional Entropy | 1.98997 |
| Cramer V | 0.2177 |
| Cross Entropy | 2.74565 |
| F1 Macro | 0.28565 |
| F1 Micro | 0.3622 |
| FNR Macro | 0.71989 |
| FNR Micro | 0.6378 |
| FPR Macro | 0.1177 |
| FPR Micro | 0.1063 |
| Gwet AC1 | 0.26836 |
| Hamming Loss | 0.6378 |
| Joint Entropy | 4.46007 |
| KL Divergence | 0.27555 |
| Kappa | 0.17777 |
| Kappa 95% CI | (0.13704,0.2185) |
| Kappa No Prevalence | -0.27559 |
| Kappa Standard Error | 0.02078 |
| Kappa Unbiased | 0.17123 |
| Krippendorff Alpha | 0.1717 |
| Lambda A | 0.1446 |
| Lambda B | 0.08666 |
| Mutual Information | 0.13547 |
| NIR | 0.26097 |
| Overall ACC | 0.3622 |
| Overall CEN | 0.6261 |
| Overall J | (1.20087,0.17155) |
| Overall MCC | 0.18078 |
| Overall MCEN | 0.70407 |
| Overall RACC | 0.22431 |
| Overall RACCU | 0.23043 |
| P-Value | 0.0 |
| PPV Macro | 0.40358 |
| PPV Micro | 0.3622 |
| Pearson C | 0.47054 |
| Phi-Squared | 0.28437 |
| RCI | 0.05485 |
| RR | 127.0 |
| Reference Entropy | 2.4701 |
| Response Entropy | 2.12545 |
| SOA1(Landis & Koch) | Slight |
| SOA2(Fleiss) | Poor |
| SOA3(Altman) | Poor |
| SOA4(Cicchetti) | Poor |
| SOA5(Cramer) | Moderate |
| SOA6(Matthews) | Negligible |
| Scott PI | 0.17123 |
| Standard Error | 0.01612 |
| TNR Macro | 0.8823 |
| TNR Micro | 0.8937 |
| TPR Macro | 0.28011 |
| TPR Micro | 0.3622 |
| Zero-one Loss | 567 |

## Class Statistics :

|  |  |  |  |  |  |  |  |  |
| --- | --- | --- | --- | --- | --- | --- | --- | --- |
| Class | 0 | 1 | 2 | 3 | 4 | 5 | 6 | Description |
| ACC | 0.69854 | 0.66479 | 0.67379 | 0.87739 | 0.96175 | 0.94488 | 0.90326 | Accuracy |
| AGF | 0.50696 | 0.57887 | 0.63817 | 0.42972 | 0.48679 | 0.39952 | 0.29281 | Adjusted F-score |
| AGM | 0.6435 | 0.64587 | 0.66385 | 0.6715 | 0.72257 | 0.67957 | 0.61748 | Adjusted geometric mean |
| AM | -28 | 40 | 98 | -7 | -16 | -3 | -84 | Difference between automatic and manual classification |
| AUC | 0.56825 | 0.59337 | 0.632 | 0.56601 | 0.60412 | 0.56724 | 0.53742 | Area under the ROC curve |
| AUCI | Poor | Poor | Fair | Poor | Fair | Poor | Poor | AUC value interpretation |
| AUPR | 0.33826 | 0.41132 | 0.4648 | 0.20491 | 0.32812 | 0.16993 | 0.47554 | Area under the PR curve |
| BCD | 0.01575 | 0.0225 | 0.05512 | 0.00394 | 0.009 | 0.00169 | 0.04724 | Bray-Curtis dissimilarity |
| BM | 0.13651 | 0.18674 | 0.26399 | 0.13202 | 0.20825 | 0.13448 | 0.07483 | Informedness or bookmaker informedness |
| CEN | 0.65694 | 0.6171 | 0.57443 | 0.75175 | 0.67704 | 0.77523 | 0.53408 | Confusion entropy |
| DOR | 2.11734 | 2.30558 | 3.04757 | 3.62542 | 26.38222 | 6.98161 | 65.55294 | Diagnostic odds ratio |
| DP | 0.17962 | 0.20001 | 0.26682 | 0.30839 | 0.78361 | 0.4653 | 1.00154 | Discriminant power |
| DPI | Poor | Poor | Poor | Poor | Poor | Poor | Limited | Discriminant power interpretation |
| ERR | 0.30146 | 0.33521 | 0.32621 | 0.12261 | 0.03825 | 0.05512 | 0.09674 | Error rate |
| F0.5 | 0.35124 | 0.39015 | 0.40356 | 0.21084 | 0.36458 | 0.17483 | 0.28226 | F0.5 score |
| F1 | 0.33663 | 0.40873 | 0.44867 | 0.20438 | 0.29167 | 0.16949 | 0.14 | F1 score - harmonic mean of precision and sensitivity |
| F2 | 0.32319 | 0.42917 | 0.50514 | 0.1983 | 0.24306 | 0.16447 | 0.09309 | F2 score |
| FDR | 0.6383 | 0.62132 | 0.62179 | 0.78462 | 0.5625 | 0.82143 | 0.125 | False discovery rate |
| FN | 148 | 129 | 96 | 58 | 25 | 26 | 85 | False negative/miss/type 2 error |
| FNR | 0.68519 | 0.55603 | 0.4486 | 0.80556 | 0.78125 | 0.83871 | 0.92391 | Miss rate or false negative rate |
| FOR | 0.21113 | 0.20908 | 0.16638 | 0.07039 | 0.02864 | 0.0302 | 0.09648 | False omission rate |
| FP | 120 | 169 | 194 | 51 | 9 | 23 | 1 | False positive/type 1 error/false alarm |
| FPR | 0.17831 | 0.25723 | 0.28741 | 0.06242 | 0.0105 | 0.02681 | 0.00125 | Fall-out or false positive rate |
| G | 0.33745 | 0.41002 | 0.45667 | 0.20465 | 0.30936 | 0.16971 | 0.25802 | G-measure geometric mean of precision and sensitivity |
| GI | 0.13651 | 0.18674 | 0.26399 | 0.13202 | 0.20825 | 0.13448 | 0.07483 | Gini index |
| GM | 0.50861 | 0.57425 | 0.62684 | 0.42697 | 0.46524 | 0.39619 | 0.27567 | G-mean geometric mean of specificity and sensitivity |
| IBA | 0.12756 | 0.23123 | 0.32959 | 0.04683 | 0.04962 | 0.02952 | 0.00588 | Index of balanced accuracy |
| ICSI | -0.32348 | -0.17736 | -0.07039 | -0.59017 | -0.34375 | -0.66014 | -0.04891 | Individual classification success index |
| IS | 0.57403 | 0.5371 | 0.65181 | 1.4111 | 3.60339 | 2.35642 | 3.07983 | Information score |
| J | 0.20238 | 0.25686 | 0.28922 | 0.11382 | 0.17073 | 0.09259 | 0.07527 | Jaccard index |
| LS | 1.48867 | 1.45105 | 1.57114 | 2.6594 | 12.1543 | 5.12097 | 8.45516 | Lift score |
| MCC | 0.14337 | 0.17796 | 0.23648 | 0.13836 | 0.2918 | 0.14126 | 0.24137 | Matthews correlation coefficient |
| MCCI | Negligible | Negligible | Negligible | Negligible | Negligible | Negligible | Negligible | Matthews correlation coefficient interpretation |
| MCEN | 0.73073 | 0.70724 | 0.6679 | 0.79887 | 0.74003 | 0.81468 | 0.54727 | Modified confusion entropy |
| MK | 0.15058 | 0.1696 | 0.21183 | 0.145 | 0.40886 | 0.14837 | 0.77852 | Markedness |
| N | 673 | 657 | 675 | 817 | 857 | 858 | 797 | Condition negative |
| NLR | 0.83387 | 0.7486 | 0.62953 | 0.85919 | 0.78954 | 0.86181 | 0.92507 | Negative likelihood ratio |
| NLRI | Negligible | Negligible | Negligible | Negligible | Negligible | Negligible | Negligible | Negative likelihood ratio interpretation |
| NPV | 0.78887 | 0.79092 | 0.83362 | 0.92961 | 0.97136 | 0.9698 | 0.90352 | Negative predictive value |
| OC | 0.3617 | 0.44397 | 0.5514 | 0.21538 | 0.4375 | 0.17857 | 0.875 | Overlap coefficient |
| OOC | 0.33745 | 0.41002 | 0.45667 | 0.20465 | 0.30936 | 0.16971 | 0.25802 | Otsuka-Ochiai coefficient |
| OP | 0.25254 | 0.413 | 0.54627 | 0.22093 | 0.32385 | 0.22922 | 0.04484 | Optimized precision |
| P | 216 | 232 | 214 | 72 | 32 | 31 | 92 | Condition positive or support |
| PLR | 1.76559 | 1.72595 | 1.91854 | 3.11492 | 20.82986 | 6.01683 | 60.6413 | Positive likelihood ratio |
| PLRI | Poor | Poor | Poor | Poor | Good | Fair | Good | Positive likelihood ratio interpretation |
| POP | 889 | 889 | 889 | 889 | 889 | 889 | 889 | Population |
| PPV | 0.3617 | 0.37868 | 0.37821 | 0.21538 | 0.4375 | 0.17857 | 0.875 | Precision or positive predictive value |
| PRE | 0.24297 | 0.26097 | 0.24072 | 0.08099 | 0.036 | 0.03487 | 0.10349 | Prevalence |
| Q | 0.35843 | 0.39496 | 0.50588 | 0.56761 | 0.92696 | 0.74942 | 0.96995 | Yule Q - coefficient of colligation |
| QI | Weak | Weak | Moderate | Moderate | Strong | Moderate | Strong | Yule Q interpretation |
| RACC | 0.05138 | 0.07985 | 0.08448 | 0.00592 | 0.00065 | 0.0011 | 0.00093 | Random accuracy |
| RACCU | 0.05163 | 0.08035 | 0.08752 | 0.00594 | 0.00073 | 0.0011 | 0.00316 | Random accuracy unbiased |
| TN | 553 | 488 | 481 | 766 | 848 | 835 | 796 | True negative/correct rejection |
| TNR | 0.82169 | 0.74277 | 0.71259 | 0.93758 | 0.9895 | 0.97319 | 0.99875 | Specificity or true negative rate |
| TON | 701 | 617 | 577 | 824 | 873 | 861 | 881 | Test outcome negative |
| TOP | 188 | 272 | 312 | 65 | 16 | 28 | 8 | Test outcome positive |
| TP | 68 | 103 | 118 | 14 | 7 | 5 | 7 | True positive/hit |
| TPR | 0.31481 | 0.44397 | 0.5514 | 0.19444 | 0.21875 | 0.16129 | 0.07609 | Sensitivity, recall, hit rate, or true positive rate |
| Y | 0.13651 | 0.18674 | 0.26399 | 0.13202 | 0.20825 | 0.13448 | 0.07483 | Youden index |
| dInd | 0.70801 | 0.61265 | 0.53277 | 0.80797 | 0.78132 | 0.83914 | 0.92391 | Distance index |
| sInd | 0.49936 | 0.56679 | 0.62328 | 0.42868 | 0.44752 | 0.40664 | 0.34669 | Similarity index |

Generated By PyCM Version 3.1
