# Supplementary material for: TopEC: prediction of Enzyme Commission classes by 3D graph neural networks and localized 3D protein descriptor
Source: Nat Commun. 2025 Mar 20;16:2737. doi: 10.1038/s41467-025-57324-5 (PMC11923149; doi:10.1038/s41467-025-57324-5)
Supplement: Supplementary file 3 — Supplementary Data 1 [file 41467_2025_57324_MOESM3_ESM.zip › Data_S1/table1/mainclass/EnzyNet/local/TopEnzyme_FOLD_wflips.html]

TopM\_FOLD\_enzynet\_wflips\_sites


# PyCM Report

## Dataset Type :

- Multi-Class Classification
- Imbalanced

Note 1 : Recommended statistics for this type of classification highlighted in aqua

Note 2 : The recommender system assumes that the input is the result of classification over the whole data rather than just a part of it.
If the confusion matrix is the result of test data classification, the recommendation is not valid.

## Confusion Matrix :

|  |  |  |  |  |  |  |  |  |  |  |  |  |  |  |  |  |  |  |  |  |  |  |  |  |  |  |  |  |  |  |  |  |  |  |  |  |  |  |  |  |  |  |  |  |  |  |  |  |  |  |  |  |  |  |  |  |  |  |  |  |  |  |  |  |  |
| --- | --- | --- | --- | --- | --- | --- | --- | --- | --- | --- | --- | --- | --- | --- | --- | --- | --- | --- | --- | --- | --- | --- | --- | --- | --- | --- | --- | --- | --- | --- | --- | --- | --- | --- | --- | --- | --- | --- | --- | --- | --- | --- | --- | --- | --- | --- | --- | --- | --- | --- | --- | --- | --- | --- | --- | --- | --- | --- | --- | --- | --- | --- | --- | --- | --- |
| Actual | Predict  |  |  |  |  |  |  |  |  | | --- | --- | --- | --- | --- | --- | --- | --- | |  | 0 | 1 | 2 | 3 | 4 | 5 | 6 | | 0 | 86 | 40 | 39 | 1 | 0 | 3 | 1 | | 1 | 31 | 163 | 47 | 2 | 0 | 2 | 0 | | 2 | 19 | 49 | 107 | 1 | 0 | 3 | 0 | | 3 | 14 | 40 | 24 | 3 | 2 | 0 | 0 | | 4 | 10 | 30 | 33 | 6 | 5 | 0 | 1 | | 5 | 12 | 36 | 23 | 2 | 0 | 20 | 0 | | 6 | 1 | 17 | 10 | 0 | 0 | 0 | 10 | |

## Overall Statistics :

|  |  |
| --- | --- |
| 95% CI | (0.40864,0.47378) |
| ACC Macro | 0.84035 |
| ARI | 0.10218 |
| AUNP | 0.63819 |
| AUNU | 0.61561 |
| Bangdiwala B | 0.25983 |
| Bennett S | 0.34808 |
| CBA | 0.26901 |
| CSI | -0.12658 |
| Chi-Squared | 588.40672 |
| Chi-Squared DF | 36 |
| Conditional Entropy | 1.60784 |
| Cramer V | 0.33139 |
| Cross Entropy | 3.12511 |
| F1 Macro | 0.34157 |
| F1 Micro | 0.44121 |
| FNR Macro | 0.66541 |
| FNR Micro | 0.55879 |
| FPR Macro | 0.10337 |
| FPR Micro | 0.09313 |
| Gwet AC1 | 0.35959 |
| Hamming Loss | 0.55879 |
| Joint Entropy | 4.21533 |
| KL Divergence | 0.51763 |
| Kappa | 0.28198 |
| Kappa 95% CI | (0.24014,0.32383) |
| Kappa No Prevalence | -0.11758 |
| Kappa Standard Error | 0.02135 |
| Kappa Unbiased | 0.26923 |
| Krippendorff Alpha | 0.26963 |
| Lambda A | 0.23457 |
| Lambda B | 0.20656 |
| Mutual Information | 0.29597 |
| NIR | 0.27436 |
| Overall ACC | 0.44121 |
| Overall CEN | 0.5424 |
| Overall J | (1.52978,0.21854) |
| Overall MCC | 0.29335 |
| Overall MCEN | 0.62851 |
| Overall RACC | 0.22176 |
| Overall RACCU | 0.23534 |
| P-Value | -0.0 |
| PPV Macro | 0.53882 |
| PPV Micro | 0.44121 |
| Pearson C | 0.63023 |
| Phi-Squared | 0.65891 |
| RCI | 0.11351 |
| RR | 127.57143 |
| Reference Entropy | 2.60748 |
| Response Entropy | 1.90382 |
| SOA1(Landis & Koch) | Fair |
| SOA2(Fleiss) | Poor |
| SOA3(Altman) | Fair |
| SOA4(Cicchetti) | Poor |
| SOA5(Cramer) | Moderate |
| SOA6(Matthews) | Negligible |
| Scott PI | 0.26923 |
| Standard Error | 0.01662 |
| TNR Macro | 0.89663 |
| TNR Micro | 0.90687 |
| TPR Macro | 0.33459 |
| TPR Micro | 0.44121 |
| Zero-one Loss | 499 |

## Class Statistics :

|  |  |  |  |  |  |  |  |  |
| --- | --- | --- | --- | --- | --- | --- | --- | --- |
| Class | 0 | 1 | 2 | 3 | 4 | 5 | 6 | Description |
| ACC | 0.80851 | 0.67077 | 0.72228 | 0.89698 | 0.90817 | 0.90929 | 0.96641 | Accuracy |
| AGF | 0.66702 | 0.69431 | 0.67583 | 0.19977 | 0.25829 | 0.48207 | 0.54492 | Adjusted F-score |
| AGM | 0.7622 | 0.67065 | 0.70773 | 0.56754 | 0.60101 | 0.71119 | 0.74975 | Adjusted geometric mean |
| AM | 3 | 130 | 104 | -68 | -78 | -65 | -26 | Difference between automatic and manual classification |
| AUC | 0.69278 | 0.66907 | 0.67563 | 0.51066 | 0.52817 | 0.60253 | 0.63041 | Area under the ROC curve |
| AUCI | Fair | Fair | Fair | Poor | Poor | Fair | Fair | AUC value interpretation |
| AUPR | 0.5015 | 0.54999 | 0.48793 | 0.11807 | 0.38655 | 0.46467 | 0.54825 | Area under the PR curve |
| BCD | 0.00168 | 0.07279 | 0.05823 | 0.03807 | 0.04367 | 0.03639 | 0.01456 | Bray-Curtis dissimilarity |
| BM | 0.38555 | 0.33815 | 0.35127 | 0.02133 | 0.05635 | 0.20505 | 0.26082 | Informedness or bookmaker informedness |
| CEN | 0.53071 | 0.54207 | 0.57581 | 0.60008 | 0.51705 | 0.49274 | 0.37161 | Confusion entropy |
| DOR | 7.4844 | 4.08813 | 4.54277 | 2.49375 | 25.1875 | 27.12329 | 152.32143 | Diagnostic odds ratio |
| DP | 0.48195 | 0.33715 | 0.3624 | 0.2188 | 0.77251 | 0.79024 | 1.20342 | Discriminant power |
| DPI | Poor | Poor | Poor | Poor | Poor | Poor | Limited | Discriminant power interpretation |
| ERR | 0.19149 | 0.32923 | 0.27772 | 0.10302 | 0.09183 | 0.09071 | 0.03359 | Error rate |
| F0.5 | 0.49884 | 0.46705 | 0.40809 | 0.1049 | 0.22124 | 0.4878 | 0.5814 | F0.5 score |
| F1 | 0.50146 | 0.52581 | 0.4632 | 0.06122 | 0.1087 | 0.33058 | 0.4 | F1 score - harmonic mean of precision and sensitivity |
| F2 | 0.5041 | 0.60148 | 0.53554 | 0.04323 | 0.07205 | 0.25 | 0.30488 | F2 score |
| FDR | 0.50289 | 0.56533 | 0.62191 | 0.8 | 0.28571 | 0.28571 | 0.16667 | False discovery rate |
| FN | 84 | 82 | 72 | 80 | 80 | 73 | 28 | False negative/miss/type 2 error |
| FNR | 0.49412 | 0.33469 | 0.40223 | 0.96386 | 0.94118 | 0.78495 | 0.73684 | Miss rate or false negative rate |
| FOR | 0.11667 | 0.1583 | 0.11803 | 0.09112 | 0.09029 | 0.08439 | 0.03178 | False omission rate |
| FP | 87 | 212 | 176 | 12 | 2 | 8 | 2 | False positive/type 1 error/false alarm |
| FPR | 0.12033 | 0.32716 | 0.2465 | 0.01481 | 0.00248 | 0.01 | 0.00234 | Fall-out or false positive rate |
| G | 0.50148 | 0.53776 | 0.47541 | 0.08502 | 0.20498 | 0.39193 | 0.46829 | G-measure geometric mean of precision and sensitivity |
| GI | 0.38555 | 0.33815 | 0.35127 | 0.02133 | 0.05635 | 0.20505 | 0.26082 | Gini index |
| GM | 0.66709 | 0.66906 | 0.67113 | 0.1887 | 0.24224 | 0.46141 | 0.51239 | G-mean geometric mean of specificity and sensitivity |
| IBA | 0.27867 | 0.44427 | 0.38027 | 0.00181 | 0.0036 | 0.04791 | 0.0697 | Index of balanced accuracy |
| ICSI | 0.00299 | 0.09997 | -0.02414 | -0.76386 | -0.22689 | -0.07066 | 0.09649 | Individual classification success index |
| IS | 1.38476 | 0.66386 | 0.91551 | 1.10555 | 2.9077 | 2.77793 | 4.29155 | Information score |
| J | 0.33463 | 0.35667 | 0.30141 | 0.03158 | 0.05747 | 0.19802 | 0.25 | Jaccard index |
| LS | 2.61129 | 1.58432 | 1.88623 | 2.15181 | 7.5042 | 6.85868 | 19.58333 | Lift score |
| MCC | 0.38299 | 0.3057 | 0.30224 | 0.04819 | 0.18751 | 0.35939 | 0.45723 | Matthews correlation coefficient |
| MCCI | Weak | Weak | Weak | Negligible | Negligible | Weak | Weak | Matthews correlation coefficient interpretation |
| MCEN | 0.631 | 0.65644 | 0.6753 | 0.60691 | 0.52557 | 0.532 | 0.39716 | Modified confusion entropy |
| MK | 0.38044 | 0.27637 | 0.26006 | 0.10888 | 0.62399 | 0.62989 | 0.80155 | Markedness |
| N | 723 | 648 | 714 | 810 | 808 | 800 | 855 | Condition negative |
| NLR | 0.56171 | 0.49743 | 0.53382 | 0.97835 | 0.94351 | 0.79287 | 0.73857 | Negative likelihood ratio |
| NLRI | Negligible | Poor | Negligible | Negligible | Negligible | Negligible | Negligible | Negative likelihood ratio interpretation |
| NPV | 0.88333 | 0.8417 | 0.88197 | 0.90888 | 0.90971 | 0.91561 | 0.96822 | Negative predictive value |
| OC | 0.50588 | 0.66531 | 0.59777 | 0.2 | 0.71429 | 0.71429 | 0.83333 | Overlap coefficient |
| OOC | 0.50148 | 0.53776 | 0.47541 | 0.08502 | 0.20498 | 0.39193 | 0.46829 | Otsuka-Ochiai coefficient |
| OP | 0.53874 | 0.66514 | 0.60703 | -0.03224 | 0.01955 | 0.26621 | 0.38385 | Optimized precision |
| P | 170 | 245 | 179 | 83 | 85 | 93 | 38 | Condition positive or support |
| PLR | 4.20406 | 2.03358 | 2.42503 | 2.43976 | 23.76471 | 21.50538 | 112.5 | Positive likelihood ratio |
| PLRI | Poor | Poor | Poor | Poor | Good | Good | Good | Positive likelihood ratio interpretation |
| POP | 893 | 893 | 893 | 893 | 893 | 893 | 893 | Population |
| PPV | 0.49711 | 0.43467 | 0.37809 | 0.2 | 0.71429 | 0.71429 | 0.83333 | Precision or positive predictive value |
| PRE | 0.19037 | 0.27436 | 0.20045 | 0.09295 | 0.09518 | 0.10414 | 0.04255 | Prevalence |
| Q | 0.76427 | 0.60693 | 0.63917 | 0.42755 | 0.92363 | 0.92888 | 0.98696 | Yule Q - coefficient of colligation |
| QI | Strong | Moderate | Moderate | Weak | Strong | Strong | Strong | Yule Q interpretation |
| RACC | 0.03688 | 0.11521 | 0.06352 | 0.00156 | 0.00075 | 0.00327 | 0.00057 | Random accuracy |
| RACCU | 0.03688 | 0.12051 | 0.06691 | 0.00301 | 0.00265 | 0.00459 | 0.00078 | Random accuracy unbiased |
| TN | 636 | 436 | 538 | 798 | 806 | 792 | 853 | True negative/correct rejection |
| TNR | 0.87967 | 0.67284 | 0.7535 | 0.98519 | 0.99752 | 0.99 | 0.99766 | Specificity or true negative rate |
| TON | 720 | 518 | 610 | 878 | 886 | 865 | 881 | Test outcome negative |
| TOP | 173 | 375 | 283 | 15 | 7 | 28 | 12 | Test outcome positive |
| TP | 86 | 163 | 107 | 3 | 5 | 20 | 10 | True positive/hit |
| TPR | 0.50588 | 0.66531 | 0.59777 | 0.03614 | 0.05882 | 0.21505 | 0.26316 | Sensitivity, recall, hit rate, or true positive rate |
| Y | 0.38555 | 0.33815 | 0.35127 | 0.02133 | 0.05635 | 0.20505 | 0.26082 | Youden index |
| dInd | 0.50856 | 0.46803 | 0.47176 | 0.96397 | 0.94118 | 0.78501 | 0.73685 | Distance index |
| sInd | 0.64039 | 0.66905 | 0.66642 | 0.31837 | 0.33449 | 0.44491 | 0.47897 | Similarity index |

Generated By PyCM Version 3.1
