# Supplementary material for: TopEC: prediction of Enzyme Commission classes by 3D graph neural networks and localized 3D protein descriptor
Source: Nat Commun. 2025 Mar 20;16:2737. doi: 10.1038/s41467-025-57324-5 (PMC11923149; doi:10.1038/s41467-025-57324-5)
Supplement: Supplementary file 3 — Supplementary Data 1 [file 41467_2025_57324_MOESM3_ESM.zip › Data_S1/table1/mainclass/EnzyNet/local/TopEnzyme_FOLD_flips.html]

TopM\_FOLD\_enzynet\_flips\_sites


# PyCM Report

## Dataset Type :

- Multi-Class Classification
- Imbalanced

Note 1 : Recommended statistics for this type of classification highlighted in aqua

Note 2 : The recommender system assumes that the input is the result of classification over the whole data rather than just a part of it.
If the confusion matrix is the result of test data classification, the recommendation is not valid.

## Confusion Matrix :

|  |  |  |  |  |  |  |  |  |  |  |  |  |  |  |  |  |  |  |  |  |  |  |  |  |  |  |  |  |  |  |  |  |  |  |  |  |  |  |  |  |  |  |  |  |  |  |  |  |  |  |  |  |  |  |  |  |  |  |  |  |  |  |  |  |  |
| --- | --- | --- | --- | --- | --- | --- | --- | --- | --- | --- | --- | --- | --- | --- | --- | --- | --- | --- | --- | --- | --- | --- | --- | --- | --- | --- | --- | --- | --- | --- | --- | --- | --- | --- | --- | --- | --- | --- | --- | --- | --- | --- | --- | --- | --- | --- | --- | --- | --- | --- | --- | --- | --- | --- | --- | --- | --- | --- | --- | --- | --- | --- | --- | --- | --- |
| Actual | Predict  |  |  |  |  |  |  |  |  | | --- | --- | --- | --- | --- | --- | --- | --- | |  | 0 | 1 | 2 | 3 | 4 | 5 | 6 | | 0 | 86 | 38 | 42 | 1 | 0 | 2 | 1 | | 1 | 28 | 170 | 43 | 2 | 0 | 2 | 0 | | 2 | 22 | 51 | 102 | 0 | 0 | 4 | 0 | | 3 | 12 | 40 | 24 | 6 | 1 | 0 | 0 | | 4 | 10 | 30 | 33 | 8 | 3 | 0 | 1 | | 5 | 14 | 39 | 24 | 2 | 0 | 14 | 0 | | 6 | 1 | 19 | 11 | 0 | 0 | 0 | 7 | |

## Overall Statistics :

|  |  |
| --- | --- |
| 95% CI | (0.40198,0.467) |
| ACC Macro | 0.83843 |
| ARI | 0.10145 |
| AUNP | 0.63363 |
| AUNU | 0.60566 |
| Bangdiwala B | 0.26329 |
| Bennett S | 0.34024 |
| CBA | 0.24919 |
| CSI | -0.14374 |
| Chi-Squared | 484.27474 |
| Chi-Squared DF | 36 |
| Conditional Entropy | 1.58657 |
| Cramer V | 0.30064 |
| Cross Entropy | 3.2158 |
| F1 Macro | 0.31589 |
| F1 Micro | 0.43449 |
| FNR Macro | 0.68401 |
| FNR Micro | 0.56551 |
| FPR Macro | 0.10468 |
| FPR Micro | 0.09425 |
| Gwet AC1 | 0.35228 |
| Hamming Loss | 0.56551 |
| Joint Entropy | 4.19405 |
| KL Divergence | 0.60832 |
| Kappa | 0.27144 |
| Kappa 95% CI | (0.22955,0.31332) |
| Kappa No Prevalence | -0.13102 |
| Kappa Standard Error | 0.02137 |
| Kappa Unbiased | 0.25743 |
| Krippendorff Alpha | 0.25784 |
| Lambda A | 0.22377 |
| Lambda B | 0.20158 |
| Mutual Information | 0.27094 |
| NIR | 0.27436 |
| Overall ACC | 0.43449 |
| Overall CEN | 0.54263 |
| Overall J | (1.40011,0.20002) |
| Overall MCC | 0.28336 |
| Overall MCEN | 0.62709 |
| Overall RACC | 0.2238 |
| Overall RACCU | 0.23845 |
| P-Value | -0.0 |
| PPV Macro | 0.54027 |
| PPV Micro | 0.43449 |
| Pearson C | 0.59297 |
| Phi-Squared | 0.5423 |
| RCI | 0.10391 |
| RR | 127.57143 |
| Reference Entropy | 2.60748 |
| Response Entropy | 1.85751 |
| SOA1(Landis & Koch) | Fair |
| SOA2(Fleiss) | Poor |
| SOA3(Altman) | Fair |
| SOA4(Cicchetti) | Poor |
| SOA5(Cramer) | Moderate |
| SOA6(Matthews) | Negligible |
| Scott PI | 0.25743 |
| Standard Error | 0.01659 |
| TNR Macro | 0.89532 |
| TNR Micro | 0.90575 |
| TPR Macro | 0.31599 |
| TPR Micro | 0.43449 |
| Zero-one Loss | 505 |

## Class Statistics :

|  |  |  |  |  |  |  |  |  |
| --- | --- | --- | --- | --- | --- | --- | --- | --- |
| Class | 0 | 1 | 2 | 3 | 4 | 5 | 6 | Description |
| ACC | 0.80851 | 0.67301 | 0.71557 | 0.89922 | 0.90705 | 0.90258 | 0.96305 | Accuracy |
| AGF | 0.66702 | 0.70816 | 0.65889 | 0.28124 | 0.20079 | 0.40525 | 0.45951 | Adjusted F-score |
| AGM | 0.7622 | 0.67337 | 0.69795 | 0.60785 | 0.57299 | 0.67144 | 0.70699 | Adjusted geometric mean |
| AM | 3 | 142 | 100 | -64 | -81 | -71 | -29 | Difference between automatic and manual classification |
| AUC | 0.69278 | 0.6795 | 0.66097 | 0.52812 | 0.51703 | 0.57027 | 0.59094 | Area under the ROC curve |
| AUCI | Fair | Fair | Fair | Poor | Poor | Poor | Poor | AUC value interpretation |
| AUPR | 0.5015 | 0.56658 | 0.46771 | 0.19404 | 0.39265 | 0.39345 | 0.48099 | Area under the PR curve |
| BCD | 0.00168 | 0.07951 | 0.05599 | 0.03583 | 0.04535 | 0.03975 | 0.01624 | Bray-Curtis dissimilarity |
| BM | 0.38555 | 0.359 | 0.32193 | 0.05624 | 0.03406 | 0.14054 | 0.18187 | Informedness or bookmaker informedness |
| CEN | 0.52862 | 0.53367 | 0.58794 | 0.56495 | 0.52214 | 0.51443 | 0.38302 | Confusion entropy |
| DOR | 7.4844 | 4.502 | 4.01893 | 4.77722 | 29.52439 | 17.5443 | 96.30645 | Diagnostic odds ratio |
| DP | 0.48195 | 0.36024 | 0.33306 | 0.37445 | 0.81055 | 0.68593 | 1.09365 | Discriminant power |
| DPI | Poor | Poor | Poor | Poor | Poor | Poor | Limited | Discriminant power interpretation |
| ERR | 0.19149 | 0.32699 | 0.28443 | 0.10078 | 0.09295 | 0.09742 | 0.03695 | Error rate |
| F0.5 | 0.49884 | 0.47407 | 0.39382 | 0.18868 | 0.14851 | 0.38674 | 0.47297 | F0.5 score |
| F1 | 0.50146 | 0.53797 | 0.44541 | 0.11765 | 0.06742 | 0.24348 | 0.29787 | F1 score - harmonic mean of precision and sensitivity |
| F2 | 0.5041 | 0.6218 | 0.51256 | 0.08547 | 0.0436 | 0.17766 | 0.21739 | F2 score |
| FDR | 0.50289 | 0.56072 | 0.63441 | 0.68421 | 0.25 | 0.36364 | 0.22222 | False discovery rate |
| FN | 84 | 75 | 77 | 77 | 82 | 79 | 31 | False negative/miss/type 2 error |
| FNR | 0.49412 | 0.30612 | 0.43017 | 0.92771 | 0.96471 | 0.84946 | 0.81579 | Miss rate or false negative rate |
| FOR | 0.11667 | 0.14822 | 0.12541 | 0.0881 | 0.09224 | 0.0907 | 0.03507 | False omission rate |
| FP | 87 | 217 | 177 | 13 | 1 | 8 | 2 | False positive/type 1 error/false alarm |
| FPR | 0.12033 | 0.33488 | 0.2479 | 0.01605 | 0.00124 | 0.01 | 0.00234 | Fall-out or false positive rate |
| G | 0.50148 | 0.55209 | 0.45643 | 0.15109 | 0.1627 | 0.30951 | 0.37852 | G-measure geometric mean of precision and sensitivity |
| GI | 0.38555 | 0.359 | 0.32193 | 0.05624 | 0.03406 | 0.14054 | 0.18187 | Gini index |
| GM | 0.66709 | 0.67935 | 0.65465 | 0.2667 | 0.18775 | 0.38605 | 0.4287 | G-mean geometric mean of specificity and sensitivity |
| IBA | 0.27867 | 0.47478 | 0.35046 | 0.00628 | 0.00129 | 0.02393 | 0.03428 | Index of balanced accuracy |
| ICSI | 0.00299 | 0.13315 | -0.06458 | -0.61192 | -0.21471 | -0.2131 | -0.03801 | Individual classification success index |
| IS | 1.38476 | 0.67908 | 0.867 | 1.76451 | 2.97809 | 2.61128 | 4.19202 | Information score |
| J | 0.33463 | 0.36797 | 0.28652 | 0.0625 | 0.03488 | 0.13861 | 0.175 | Jaccard index |
| LS | 2.61129 | 1.60112 | 1.82387 | 3.39759 | 7.87941 | 6.11046 | 18.27778 | Lift score |
| MCC | 0.38299 | 0.32325 | 0.27807 | 0.11316 | 0.14967 | 0.27692 | 0.36753 | Matthews correlation coefficient |
| MCCI | Weak | Weak | Negligible | Negligible | Negligible | Negligible | Weak | Matthews correlation coefficient interpretation |
| MCEN | 0.62823 | 0.65034 | 0.68406 | 0.57739 | 0.52704 | 0.54074 | 0.39651 | Modified confusion entropy |
| MK | 0.38044 | 0.29106 | 0.24018 | 0.22769 | 0.65776 | 0.54566 | 0.74271 | Markedness |
| N | 723 | 648 | 714 | 810 | 808 | 800 | 855 | Condition negative |
| NLR | 0.56171 | 0.46025 | 0.57195 | 0.94284 | 0.9659 | 0.85804 | 0.8177 | Negative likelihood ratio |
| NLRI | Negligible | Poor | Negligible | Negligible | Negligible | Negligible | Negligible | Negative likelihood ratio interpretation |
| NPV | 0.88333 | 0.85178 | 0.87459 | 0.9119 | 0.90776 | 0.9093 | 0.96493 | Negative predictive value |
| OC | 0.50588 | 0.69388 | 0.56983 | 0.31579 | 0.75 | 0.63636 | 0.77778 | Overlap coefficient |
| OOC | 0.50148 | 0.55209 | 0.45643 | 0.15109 | 0.1627 | 0.30951 | 0.37852 | Otsuka-Ochiai coefficient |
| OP | 0.53874 | 0.65185 | 0.57769 | 0.0361 | -0.02468 | 0.16655 | 0.27477 | Optimized precision |
| P | 170 | 245 | 179 | 83 | 85 | 93 | 38 | Condition positive or support |
| PLR | 4.20406 | 2.07204 | 2.29865 | 4.50417 | 28.51765 | 15.05376 | 78.75 | Positive likelihood ratio |
| PLRI | Poor | Poor | Poor | Poor | Good | Good | Good | Positive likelihood ratio interpretation |
| POP | 893 | 893 | 893 | 893 | 893 | 893 | 893 | Population |
| PPV | 0.49711 | 0.43928 | 0.36559 | 0.31579 | 0.75 | 0.63636 | 0.77778 | Precision or positive predictive value |
| PRE | 0.19037 | 0.27436 | 0.20045 | 0.09295 | 0.09518 | 0.10414 | 0.04255 | Prevalence |
| Q | 0.76427 | 0.6365 | 0.60151 | 0.65381 | 0.93448 | 0.89215 | 0.97945 | Yule Q - coefficient of colligation |
| QI | Strong | Moderate | Moderate | Moderate | Strong | Strong | Strong | Yule Q interpretation |
| RACC | 0.03688 | 0.1189 | 0.06263 | 0.00198 | 0.00043 | 0.00257 | 0.00043 | Random accuracy |
| RACCU | 0.03688 | 0.12522 | 0.06576 | 0.00326 | 0.00248 | 0.00415 | 0.00069 | Random accuracy unbiased |
| TN | 636 | 431 | 537 | 797 | 807 | 792 | 853 | True negative/correct rejection |
| TNR | 0.87967 | 0.66512 | 0.7521 | 0.98395 | 0.99876 | 0.99 | 0.99766 | Specificity or true negative rate |
| TON | 720 | 506 | 614 | 874 | 889 | 871 | 884 | Test outcome negative |
| TOP | 173 | 387 | 279 | 19 | 4 | 22 | 9 | Test outcome positive |
| TP | 86 | 170 | 102 | 6 | 3 | 14 | 7 | True positive/hit |
| TPR | 0.50588 | 0.69388 | 0.56983 | 0.07229 | 0.03529 | 0.15054 | 0.18421 | Sensitivity, recall, hit rate, or true positive rate |
| Y | 0.38555 | 0.359 | 0.32193 | 0.05624 | 0.03406 | 0.14054 | 0.18187 | Youden index |
| dInd | 0.50856 | 0.45371 | 0.49649 | 0.92785 | 0.96471 | 0.84952 | 0.81579 | Distance index |
| sInd | 0.64039 | 0.67918 | 0.64893 | 0.34391 | 0.31785 | 0.3993 | 0.42315 | Similarity index |

Generated By PyCM Version 3.1
