# Supplementary material for: TopEC: prediction of Enzyme Commission classes by 3D graph neural networks and localized 3D protein descriptor
Source: Nat Commun. 2025 Mar 20;16:2737. doi: 10.1038/s41467-025-57324-5 (PMC11923149; doi:10.1038/s41467-025-57324-5)
Supplement: Supplementary file 3 — Supplementary Data 1 [file 41467_2025_57324_MOESM3_ESM.zip › Data_S1/table1/mainclass/EnzyNet/local/TopEnzyme_FOLD.html]

TopM\_FOLD\_enzynet\_none\_sites


# PyCM Report

## Dataset Type :

- Multi-Class Classification
- Imbalanced

Note 1 : Recommended statistics for this type of classification highlighted in aqua

Note 2 : The recommender system assumes that the input is the result of classification over the whole data rather than just a part of it.
If the confusion matrix is the result of test data classification, the recommendation is not valid.

## Confusion Matrix :

|  |  |  |  |  |  |  |  |  |  |  |  |  |  |  |  |  |  |  |  |  |  |  |  |  |  |  |  |  |  |  |  |  |  |  |  |  |  |  |  |  |  |  |  |  |  |  |  |  |  |  |  |  |  |  |  |  |  |  |  |  |  |  |  |  |  |
| --- | --- | --- | --- | --- | --- | --- | --- | --- | --- | --- | --- | --- | --- | --- | --- | --- | --- | --- | --- | --- | --- | --- | --- | --- | --- | --- | --- | --- | --- | --- | --- | --- | --- | --- | --- | --- | --- | --- | --- | --- | --- | --- | --- | --- | --- | --- | --- | --- | --- | --- | --- | --- | --- | --- | --- | --- | --- | --- | --- | --- | --- | --- | --- | --- | --- |
| Actual | Predict  |  |  |  |  |  |  |  |  | | --- | --- | --- | --- | --- | --- | --- | --- | |  | 0 | 1 | 2 | 3 | 4 | 5 | 6 | | 0 | 71 | 52 | 36 | 5 | 0 | 5 | 1 | | 1 | 40 | 145 | 54 | 2 | 0 | 3 | 1 | | 2 | 23 | 53 | 79 | 9 | 3 | 12 | 0 | | 3 | 23 | 28 | 20 | 5 | 5 | 2 | 0 | | 4 | 12 | 28 | 22 | 8 | 12 | 1 | 2 | | 5 | 12 | 37 | 22 | 5 | 0 | 17 | 0 | | 6 | 6 | 9 | 8 | 0 | 1 | 0 | 14 | |

## Overall Statistics :

|  |  |
| --- | --- |
| 95% CI | (0.3522,0.416) |
| ACC Macro | 0.82403 |
| ARI | 0.06947 |
| AUNP | 0.60643 |
| AUNU | 0.60117 |
| Bangdiwala B | 0.19368 |
| Bennett S | 0.28145 |
| CBA | 0.26744 |
| CSI | -0.25084 |
| Chi-Squared | 488.88419 |
| Chi-Squared DF | 36 |
| Conditional Entropy | 1.90145 |
| Cramer V | 0.30207 |
| Cross Entropy | 2.83617 |
| F1 Macro | 0.33246 |
| F1 Micro | 0.3841 |
| FNR Macro | 0.68522 |
| FNR Micro | 0.6159 |
| FPR Macro | 0.11245 |
| FPR Micro | 0.10265 |
| Gwet AC1 | 0.29225 |
| Hamming Loss | 0.6159 |
| Joint Entropy | 4.50893 |
| KL Divergence | 0.22869 |
| Kappa | 0.217 |
| Kappa 95% CI | (0.17645,0.25756) |
| Kappa No Prevalence | -0.2318 |
| Kappa Standard Error | 0.02069 |
| Kappa Unbiased | 0.20903 |
| Krippendorff Alpha | 0.20947 |
| Lambda A | 0.15741 |
| Lambda B | 0.09242 |
| Mutual Information | 0.23125 |
| NIR | 0.27436 |
| Overall ACC | 0.3841 |
| Overall CEN | 0.61491 |
| Overall J | (1.45291,0.20756) |
| Overall MCC | 0.22188 |
| Overall MCEN | 0.69866 |
| Overall RACC | 0.21341 |
| Overall RACCU | 0.22133 |
| P-Value | 0.0 |
| PPV Macro | 0.43438 |
| PPV Micro | 0.3841 |
| Pearson C | 0.59479 |
| Phi-Squared | 0.54746 |
| RCI | 0.08869 |
| RR | 127.57143 |
| Reference Entropy | 2.60748 |
| Response Entropy | 2.1327 |
| SOA1(Landis & Koch) | Fair |
| SOA2(Fleiss) | Poor |
| SOA3(Altman) | Fair |
| SOA4(Cicchetti) | Poor |
| SOA5(Cramer) | Moderate |
| SOA6(Matthews) | Negligible |
| Scott PI | 0.20903 |
| Standard Error | 0.01628 |
| TNR Macro | 0.88755 |
| TNR Micro | 0.89735 |
| TPR Macro | 0.31478 |
| TPR Micro | 0.3841 |
| Zero-one Loss | 550 |

## Class Statistics :

|  |  |  |  |  |  |  |  |  |
| --- | --- | --- | --- | --- | --- | --- | --- | --- |
| Class | 0 | 1 | 2 | 3 | 4 | 5 | 6 | Description |
| ACC | 0.75924 | 0.65622 | 0.70661 | 0.88018 | 0.90817 | 0.88914 | 0.96865 | Accuracy |
| AGF | 0.59191 | 0.6533 | 0.58559 | 0.25064 | 0.39314 | 0.43623 | 0.63428 | Adjusted F-score |
| AGM | 0.70284 | 0.65395 | 0.66809 | 0.58498 | 0.66588 | 0.6812 | 0.7962 | Adjusted geometric mean |
| AM | 17 | 107 | 62 | -49 | -64 | -53 | -20 | Difference between automatic and manual classification |
| AUC | 0.6286 | 0.6362 | 0.60723 | 0.51222 | 0.56502 | 0.57702 | 0.68187 | Area under the ROC curve |
| AUCI | Fair | Fair | Fair | Poor | Poor | Poor | Fair | AUC value interpretation |
| AUPR | 0.39866 | 0.50188 | 0.38457 | 0.10365 | 0.3563 | 0.3039 | 0.5731 | Area under the PR curve |
| BCD | 0.00952 | 0.05991 | 0.03471 | 0.02744 | 0.03583 | 0.02968 | 0.0112 | Bray-Curtis dissimilarity |
| BM | 0.2572 | 0.27239 | 0.21445 | 0.02444 | 0.13004 | 0.15405 | 0.36374 | Informedness or bookmaker informedness |
| CEN | 0.62108 | 0.56543 | 0.66128 | 0.75978 | 0.61471 | 0.61158 | 0.46109 | Confusion entropy |
| DOR | 3.75279 | 3.08913 | 2.69185 | 1.72635 | 14.59361 | 7.55664 | 124.10417 | Diagnostic odds ratio |
| DP | 0.31666 | 0.27006 | 0.2371 | 0.13074 | 0.64184 | 0.48425 | 1.15436 | Discriminant power |
| DPI | Poor | Poor | Poor | Poor | Poor | Poor | Limited | Discriminant power interpretation |
| ERR | 0.24076 | 0.34378 | 0.29339 | 0.11982 | 0.09183 | 0.11086 | 0.03135 | Error rate |
| F0.5 | 0.38671 | 0.4386 | 0.34558 | 0.11416 | 0.35503 | 0.33597 | 0.63636 | F0.5 score |
| F1 | 0.39776 | 0.48576 | 0.37619 | 0.08547 | 0.22642 | 0.25564 | 0.5 | F1 score - harmonic mean of precision and sensitivity |
| F2 | 0.40946 | 0.54429 | 0.41275 | 0.06831 | 0.1662 | 0.20631 | 0.41176 | F2 score |
| FDR | 0.62032 | 0.58807 | 0.6722 | 0.85294 | 0.42857 | 0.575 | 0.22222 | False discovery rate |
| FN | 99 | 100 | 100 | 78 | 73 | 76 | 24 | False negative/miss/type 2 error |
| FNR | 0.58235 | 0.40816 | 0.55866 | 0.93976 | 0.85882 | 0.8172 | 0.63158 | Miss rate or false negative rate |
| FOR | 0.14023 | 0.18484 | 0.15337 | 0.0908 | 0.08372 | 0.0891 | 0.02743 | False omission rate |
| FP | 116 | 207 | 162 | 29 | 9 | 23 | 4 | False positive/type 1 error/false alarm |
| FPR | 0.16044 | 0.31944 | 0.22689 | 0.0358 | 0.01114 | 0.02875 | 0.00468 | Fall-out or false positive rate |
| G | 0.39821 | 0.49376 | 0.38036 | 0.09412 | 0.28403 | 0.27873 | 0.5353 | G-measure geometric mean of precision and sensitivity |
| GI | 0.2572 | 0.27239 | 0.21445 | 0.02444 | 0.13004 | 0.15405 | 0.36374 | Gini index |
| GM | 0.59215 | 0.63465 | 0.58413 | 0.24101 | 0.37364 | 0.42136 | 0.60556 | G-mean geometric mean of specificity and sensitivity |
| IBA | 0.2027 | 0.36704 | 0.228 | 0.00558 | 0.02126 | 0.03756 | 0.13681 | Index of balanced accuracy |
| ICSI | -0.20267 | 0.00377 | -0.23086 | -0.7927 | -0.28739 | -0.3922 | 0.1462 | Individual classification success index |
| IS | 0.99598 | 0.58636 | 0.70959 | 0.66194 | 2.58577 | 2.02889 | 4.19202 | Information score |
| J | 0.24825 | 0.3208 | 0.23167 | 0.04464 | 0.12766 | 0.14655 | 0.33333 | Jaccard index |
| LS | 1.99443 | 1.50145 | 1.63534 | 1.58221 | 6.00336 | 4.08091 | 18.27778 | Lift score |
| MCC | 0.24817 | 0.24871 | 0.19341 | 0.03708 | 0.25184 | 0.22747 | 0.52243 | Matthews correlation coefficient |
| MCCI | Negligible | Negligible | Negligible | Negligible | Negligible | Negligible | Moderate | Matthews correlation coefficient interpretation |
| MCEN | 0.70819 | 0.67076 | 0.75005 | 0.77691 | 0.651 | 0.65424 | 0.5376 | Modified confusion entropy |
| MK | 0.23945 | 0.22709 | 0.17443 | 0.05626 | 0.48771 | 0.3359 | 0.75035 | Markedness |
| N | 723 | 648 | 714 | 810 | 808 | 800 | 855 | Condition negative |
| NLR | 0.69364 | 0.59975 | 0.72261 | 0.97465 | 0.8685 | 0.84139 | 0.63455 | Negative likelihood ratio |
| NLRI | Negligible | Negligible | Negligible | Negligible | Negligible | Negligible | Negligible | Negative likelihood ratio interpretation |
| NPV | 0.85977 | 0.81516 | 0.84663 | 0.9092 | 0.91628 | 0.9109 | 0.97257 | Negative predictive value |
| OC | 0.41765 | 0.59184 | 0.44134 | 0.14706 | 0.57143 | 0.425 | 0.77778 | Overlap coefficient |
| OOC | 0.39821 | 0.49376 | 0.38036 | 0.09412 | 0.28403 | 0.27873 | 0.5353 | Otsuka-Ochiai coefficient |
| OP | 0.42364 | 0.58649 | 0.43342 | -0.00221 | 0.15804 | 0.20593 | 0.50895 | Optimized precision |
| P | 170 | 245 | 179 | 83 | 85 | 93 | 38 | Condition positive or support |
| PLR | 2.60309 | 1.85271 | 1.94517 | 1.68259 | 12.67451 | 6.35811 | 78.75 | Positive likelihood ratio |
| PLRI | Poor | Poor | Poor | Poor | Good | Fair | Good | Positive likelihood ratio interpretation |
| POP | 893 | 893 | 893 | 893 | 893 | 893 | 893 | Population |
| PPV | 0.37968 | 0.41193 | 0.3278 | 0.14706 | 0.57143 | 0.425 | 0.77778 | Precision or positive predictive value |
| PRE | 0.19037 | 0.27436 | 0.20045 | 0.09295 | 0.09518 | 0.10414 | 0.04255 | Prevalence |
| Q | 0.57919 | 0.5109 | 0.45827 | 0.26642 | 0.87174 | 0.76626 | 0.98401 | Yule Q - coefficient of colligation |
| QI | Moderate | Moderate | Weak | Weak | Strong | Strong | Strong | Yule Q interpretation |
| RACC | 0.03986 | 0.10814 | 0.0541 | 0.00354 | 0.00224 | 0.00466 | 0.00086 | Random accuracy |
| RACCU | 0.03996 | 0.11173 | 0.0553 | 0.00429 | 0.00352 | 0.00555 | 0.00098 | Random accuracy unbiased |
| TN | 607 | 441 | 552 | 781 | 799 | 777 | 851 | True negative/correct rejection |
| TNR | 0.83956 | 0.68056 | 0.77311 | 0.9642 | 0.98886 | 0.97125 | 0.99532 | Specificity or true negative rate |
| TON | 706 | 541 | 652 | 859 | 872 | 853 | 875 | Test outcome negative |
| TOP | 187 | 352 | 241 | 34 | 21 | 40 | 18 | Test outcome positive |
| TP | 71 | 145 | 79 | 5 | 12 | 17 | 14 | True positive/hit |
| TPR | 0.41765 | 0.59184 | 0.44134 | 0.06024 | 0.14118 | 0.1828 | 0.36842 | Sensitivity, recall, hit rate, or true positive rate |
| Y | 0.2572 | 0.27239 | 0.21445 | 0.02444 | 0.13004 | 0.15405 | 0.36374 | Youden index |
| dInd | 0.60405 | 0.51831 | 0.60298 | 0.94044 | 0.8589 | 0.81771 | 0.6316 | Distance index |
| sInd | 0.57287 | 0.6335 | 0.57363 | 0.33501 | 0.39267 | 0.42179 | 0.55339 | Similarity index |

Generated By PyCM Version 3.1
