# Supplementary material for: TopEC: prediction of Enzyme Commission classes by 3D graph neural networks and localized 3D protein descriptor
Source: Nat Commun. 2025 Mar 20;16:2737. doi: 10.1038/s41467-025-57324-5 (PMC11923149; doi:10.1038/s41467-025-57324-5)
Supplement: Supplementary file 3 — Supplementary Data 1 [file 41467_2025_57324_MOESM3_ESM.zip › Data_S1/table1/mainclass/EnzyNet/local/Combined_TEMP_wflips.html]

Both\_TEMP\_enzynet\_wflips\_sites


# PyCM Report

## Dataset Type :

- Multi-Class Classification
- Imbalanced

Note 1 : Recommended statistics for this type of classification highlighted in aqua

Note 2 : The recommender system assumes that the input is the result of classification over the whole data rather than just a part of it.
If the confusion matrix is the result of test data classification, the recommendation is not valid.

## Confusion Matrix :

|  |  |  |  |  |  |  |  |  |  |  |  |  |  |  |  |  |  |  |  |  |  |  |  |  |  |  |  |  |  |  |  |  |  |  |  |  |  |  |  |  |  |  |  |  |  |  |  |  |  |  |  |  |  |  |  |  |  |  |  |  |  |  |  |  |  |
| --- | --- | --- | --- | --- | --- | --- | --- | --- | --- | --- | --- | --- | --- | --- | --- | --- | --- | --- | --- | --- | --- | --- | --- | --- | --- | --- | --- | --- | --- | --- | --- | --- | --- | --- | --- | --- | --- | --- | --- | --- | --- | --- | --- | --- | --- | --- | --- | --- | --- | --- | --- | --- | --- | --- | --- | --- | --- | --- | --- | --- | --- | --- | --- | --- | --- |
| Actual | Predict  |  |  |  |  |  |  |  |  | | --- | --- | --- | --- | --- | --- | --- | --- | |  | 0 | 1 | 2 | 3 | 4 | 5 | 6 | | 0 | 369 | 112 | 146 | 8 | 2 | 2 | 0 | | 1 | 114 | 622 | 243 | 3 | 1 | 0 | 3 | | 2 | 76 | 152 | 635 | 6 | 0 | 0 | 15 | | 3 | 42 | 36 | 64 | 55 | 0 | 0 | 0 | | 4 | 33 | 26 | 25 | 2 | 23 | 0 | 0 | | 5 | 13 | 39 | 28 | 0 | 0 | 9 | 0 | | 6 | 47 | 62 | 52 | 2 | 1 | 0 | 4 | |

## Overall Statistics :

|  |  |
| --- | --- |
| 95% CI | (0.54136,0.57648) |
| ACC Macro | 0.87398 |
| ARI | 0.19172 |
| AUNP | 0.69559 |
| AUNU | 0.63807 |
| Bangdiwala B | 0.36396 |
| Bennett S | 0.48541 |
| CBA | 0.32458 |
| CSI | -0.03226 |
| Chi-Squared | 2582.56274 |
| Chi-Squared DF | 36 |
| Conditional Entropy | 1.4504 |
| Cramer V | 0.37432 |
| Cross Entropy | 2.54632 |
| F1 Macro | 0.39138 |
| F1 Micro | 0.55892 |
| FNR Macro | 0.63689 |
| FNR Micro | 0.44108 |
| FPR Macro | 0.08698 |
| FPR Micro | 0.07351 |
| Gwet AC1 | 0.49827 |
| Hamming Loss | 0.44108 |
| Joint Entropy | 3.76729 |
| KL Divergence | 0.22943 |
| Kappa | 0.39517 |
| Kappa 95% CI | (0.37109,0.41925) |
| Kappa No Prevalence | 0.11784 |
| Kappa Standard Error | 0.01228 |
| Kappa Unbiased | 0.39183 |
| Krippendorff Alpha | 0.39193 |
| Lambda A | 0.3557 |
| Lambda B | 0.33582 |
| Mutual Information | 0.36591 |
| NIR | 0.32096 |
| Overall ACC | 0.55892 |
| Overall CEN | 0.4627 |
| Overall J | (1.83958,0.2628) |
| Overall MCC | 0.40019 |
| Overall MCEN | 0.56572 |
| Overall RACC | 0.27074 |
| Overall RACCU | 0.27474 |
| P-Value | None |
| PPV Macro | 0.60464 |
| PPV Micro | 0.55892 |
| Pearson C | 0.67581 |
| Phi-Squared | 0.84068 |
| RCI | 0.15793 |
| RR | 438.85714 |
| Reference Entropy | 2.31689 |
| Response Entropy | 1.8163 |
| SOA1(Landis & Koch) | Fair |
| SOA2(Fleiss) | Poor |
| SOA3(Altman) | Fair |
| SOA4(Cicchetti) | Poor |
| SOA5(Cramer) | Moderate |
| SOA6(Matthews) | Weak |
| Scott PI | 0.39183 |
| Standard Error | 0.00896 |
| TNR Macro | 0.91302 |
| TNR Micro | 0.92649 |
| TPR Macro | 0.36311 |
| TPR Micro | 0.55892 |
| Zero-one Loss | 1355 |

## Class Statistics :

|  |  |  |  |  |  |  |  |  |
| --- | --- | --- | --- | --- | --- | --- | --- | --- |
| Class | 0 | 1 | 2 | 3 | 4 | 5 | 6 | Description |
| ACC | 0.80632 | 0.74251 | 0.7373 | 0.94694 | 0.9707 | 0.97331 | 0.94076 | Accuracy |
| AGF | 0.70776 | 0.71249 | 0.75091 | 0.55287 | 0.49262 | 0.34644 | 0.16593 | Adjusted F-score |
| AGM | 0.77765 | 0.74349 | 0.73712 | 0.75185 | 0.72398 | 0.6536 | 0.56201 | Adjusted geometric mean |
| AM | 55 | 63 | 309 | -121 | -82 | -78 | -146 | Difference between automatic and manual classification |
| AUC | 0.72194 | 0.71307 | 0.73165 | 0.63594 | 0.60483 | 0.55023 | 0.50881 | Area under the ROC curve |
| AUCI | Good | Good | Good | Fair | Fair | Poor | Poor | AUC value interpretation |
| AUPR | 0.55458 | 0.61189 | 0.6253 | 0.50144 | 0.53143 | 0.45965 | 0.10281 | Area under the PR curve |
| BCD | 0.00895 | 0.01025 | 0.05029 | 0.01969 | 0.01335 | 0.0127 | 0.02376 | Bray-Curtis dissimilarity |
| BM | 0.44388 | 0.42613 | 0.4633 | 0.27188 | 0.20966 | 0.10045 | 0.01761 | Informedness or bookmaker informedness |
| CEN | 0.49811 | 0.44299 | 0.44805 | 0.48458 | 0.46989 | 0.42944 | 0.56626 | Confusion entropy |
| DOR | 8.86441 | 6.63907 | 7.44951 | 52.63917 | 197.84012 | 167.68125 | 3.91057 | Diagnostic odds ratio |
| DP | 0.52247 | 0.45325 | 0.48083 | 0.94901 | 1.26602 | 1.22642 | 0.32652 | Discriminant power |
| DPI | Poor | Poor | Poor | Poor | Limited | Limited | Poor | Discriminant power interpretation |
| ERR | 0.19368 | 0.25749 | 0.2627 | 0.05306 | 0.0293 | 0.02669 | 0.05924 | Error rate |
| F0.5 | 0.54026 | 0.60015 | 0.56135 | 0.5489 | 0.52995 | 0.33835 | 0.07812 | F0.5 score |
| F1 | 0.55364 | 0.6113 | 0.61146 | 0.40293 | 0.33824 | 0.18 | 0.04211 | F1 score - harmonic mean of precision and sensitivity |
| F2 | 0.56769 | 0.62287 | 0.67139 | 0.31829 | 0.24838 | 0.12262 | 0.02882 | F2 score |
| FDR | 0.4683 | 0.40705 | 0.46773 | 0.27632 | 0.14815 | 0.18182 | 0.81818 | False discovery rate |
| FN | 270 | 364 | 249 | 142 | 86 | 80 | 164 | False negative/miss/type 2 error |
| FNR | 0.42254 | 0.36917 | 0.28167 | 0.72081 | 0.78899 | 0.89888 | 0.97619 | Miss rate or false negative rate |
| FOR | 0.11354 | 0.17993 | 0.13252 | 0.0474 | 0.02824 | 0.02614 | 0.05377 | False omission rate |
| FP | 325 | 427 | 558 | 21 | 4 | 2 | 18 | False positive/type 1 error/false alarm |
| FPR | 0.13358 | 0.2047 | 0.25503 | 0.0073 | 0.00135 | 0.00067 | 0.0062 | Fall-out or false positive rate |
| G | 0.55411 | 0.6116 | 0.61834 | 0.44949 | 0.42397 | 0.28764 | 0.0658 | G-measure geometric mean of precision and sensitivity |
| GI | 0.44388 | 0.42613 | 0.4633 | 0.27188 | 0.20966 | 0.10045 | 0.01761 | Gini index |
| GM | 0.70734 | 0.70831 | 0.73153 | 0.52645 | 0.45905 | 0.31789 | 0.15382 | G-mean geometric mean of specificity and sensitivity |
| IBA | 0.35575 | 0.41919 | 0.52087 | 0.0794 | 0.04475 | 0.01029 | 0.00071 | Index of balanced accuracy |
| ICSI | 0.10917 | 0.22378 | 0.2506 | 0.00287 | 0.06286 | -0.08069 | -0.79437 | Individual classification success index |
| IS | 1.35398 | 0.88549 | 0.88729 | 3.49634 | 4.58545 | 4.81972 | 1.73321 | Information score |
| J | 0.38278 | 0.4402 | 0.44036 | 0.25229 | 0.20354 | 0.0989 | 0.02151 | Jaccard index |
| LS | 2.55616 | 1.84739 | 1.8497 | 11.28507 | 24.00815 | 28.24106 | 3.32468 | Lift score |
| MCC | 0.43083 | 0.41952 | 0.43036 | 0.4288 | 0.41554 | 0.28207 | 0.04749 | Matthews correlation coefficient |
| MCCI | Weak | Weak | Weak | Weak | Weak | Negligible | Negligible | Matthews correlation coefficient interpretation |
| MCEN | 0.60827 | 0.55582 | 0.56317 | 0.53915 | 0.50615 | 0.43772 | 0.57006 | Modified confusion entropy |
| MK | 0.41816 | 0.41301 | 0.39975 | 0.67629 | 0.82361 | 0.79205 | 0.12805 | Markedness |
| N | 2433 | 2086 | 2188 | 2875 | 2963 | 2983 | 2904 | Condition negative |
| NLR | 0.48768 | 0.46419 | 0.3781 | 0.72612 | 0.79006 | 0.89948 | 0.98228 | Negative likelihood ratio |
| NLRI | Poor | Poor | Poor | Negligible | Negligible | Negligible | Negligible | Negative likelihood ratio interpretation |
| NPV | 0.88646 | 0.82007 | 0.86748 | 0.9526 | 0.97176 | 0.97386 | 0.94623 | Negative predictive value |
| OC | 0.57746 | 0.63083 | 0.71833 | 0.72368 | 0.85185 | 0.81818 | 0.18182 | Overlap coefficient |
| OOC | 0.55411 | 0.6116 | 0.61834 | 0.44949 | 0.42397 | 0.28764 | 0.0658 | Otsuka-Ochiai coefficient |
| OP | 0.60619 | 0.62719 | 0.71909 | 0.38595 | 0.31958 | 0.15709 | -0.01245 | Optimized precision |
| P | 639 | 986 | 884 | 197 | 109 | 89 | 168 | Condition positive or support |
| PLR | 4.32299 | 3.08177 | 2.81666 | 38.22214 | 156.30505 | 150.82584 | 3.84127 | Positive likelihood ratio |
| PLRI | Poor | Poor | Poor | Good | Good | Good | Poor | Positive likelihood ratio interpretation |
| POP | 3072 | 3072 | 3072 | 3072 | 3072 | 3072 | 3072 | Population |
| PPV | 0.5317 | 0.59295 | 0.53227 | 0.72368 | 0.85185 | 0.81818 | 0.18182 | Precision or positive predictive value |
| PRE | 0.20801 | 0.32096 | 0.28776 | 0.06413 | 0.03548 | 0.02897 | 0.05469 | Prevalence |
| Q | 0.79725 | 0.73819 | 0.7633 | 0.96271 | 0.98994 | 0.98814 | 0.59272 | Yule Q - coefficient of colligation |
| QI | Strong | Moderate | Strong | Strong | Strong | Strong | Moderate | Yule Q interpretation |
| RACC | 0.04699 | 0.1096 | 0.11175 | 0.00159 | 0.00031 | 0.0001 | 0.00039 | Random accuracy |
| RACCU | 0.04707 | 0.1097 | 0.11428 | 0.00197 | 0.00049 | 0.00026 | 0.00096 | Random accuracy unbiased |
| TN | 2108 | 1659 | 1630 | 2854 | 2959 | 2981 | 2886 | True negative/correct rejection |
| TNR | 0.86642 | 0.7953 | 0.74497 | 0.9927 | 0.99865 | 0.99933 | 0.9938 | Specificity or true negative rate |
| TON | 2378 | 2023 | 1879 | 2996 | 3045 | 3061 | 3050 | Test outcome negative |
| TOP | 694 | 1049 | 1193 | 76 | 27 | 11 | 22 | Test outcome positive |
| TP | 369 | 622 | 635 | 55 | 23 | 9 | 4 | True positive/hit |
| TPR | 0.57746 | 0.63083 | 0.71833 | 0.27919 | 0.21101 | 0.10112 | 0.02381 | Sensitivity, recall, hit rate, or true positive rate |
| Y | 0.44388 | 0.42613 | 0.4633 | 0.27188 | 0.20966 | 0.10045 | 0.01761 | Youden index |
| dInd | 0.44315 | 0.42212 | 0.37997 | 0.72085 | 0.78899 | 0.89888 | 0.97621 | Distance index |
| sInd | 0.68665 | 0.70152 | 0.73132 | 0.49028 | 0.4421 | 0.3644 | 0.30972 | Similarity index |

Generated By PyCM Version 3.1
