# Supplementary material for: TopEC: prediction of Enzyme Commission classes by 3D graph neural networks and localized 3D protein descriptor
Source: Nat Commun. 2025 Mar 20;16:2737. doi: 10.1038/s41467-025-57324-5 (PMC11923149; doi:10.1038/s41467-025-57324-5)
Supplement: Supplementary file 3 — Supplementary Data 1 [file 41467_2025_57324_MOESM3_ESM.zip › Data_S1/table1/mainclass/EnzyNet/local/Combined_TEMP_flips.html]

Both\_TEMP\_enzynet\_flips\_sites


# PyCM Report

## Dataset Type :

- Multi-Class Classification
- Imbalanced

Note 1 : Recommended statistics for this type of classification highlighted in aqua

Note 2 : The recommender system assumes that the input is the result of classification over the whole data rather than just a part of it.
If the confusion matrix is the result of test data classification, the recommendation is not valid.

## Confusion Matrix :

|  |  |  |  |  |  |  |  |  |  |  |  |  |  |  |  |  |  |  |  |  |  |  |  |  |  |  |  |  |  |  |  |  |  |  |  |  |  |  |  |  |  |  |  |  |  |  |  |  |  |  |  |  |  |  |  |  |  |  |  |  |  |  |  |  |  |
| --- | --- | --- | --- | --- | --- | --- | --- | --- | --- | --- | --- | --- | --- | --- | --- | --- | --- | --- | --- | --- | --- | --- | --- | --- | --- | --- | --- | --- | --- | --- | --- | --- | --- | --- | --- | --- | --- | --- | --- | --- | --- | --- | --- | --- | --- | --- | --- | --- | --- | --- | --- | --- | --- | --- | --- | --- | --- | --- | --- | --- | --- | --- | --- | --- | --- |
| Actual | Predict  |  |  |  |  |  |  |  |  | | --- | --- | --- | --- | --- | --- | --- | --- | |  | 0 | 1 | 2 | 3 | 4 | 5 | 6 | | 0 | 370 | 115 | 147 | 5 | 1 | 1 | 0 | | 1 | 121 | 612 | 247 | 3 | 0 | 0 | 3 | | 2 | 75 | 152 | 642 | 3 | 1 | 0 | 11 | | 3 | 39 | 40 | 67 | 50 | 1 | 0 | 0 | | 4 | 35 | 23 | 30 | 2 | 19 | 0 | 0 | | 5 | 15 | 40 | 26 | 0 | 0 | 8 | 0 | | 6 | 50 | 61 | 50 | 2 | 1 | 0 | 4 | |

## Overall Statistics :

|  |  |
| --- | --- |
| 95% CI | (0.53744,0.57259) |
| ACC Macro | 0.87286 |
| ARI | 0.18665 |
| AUNP | 0.69242 |
| AUNU | 0.63233 |
| Bangdiwala B | 0.36098 |
| Bennett S | 0.48085 |
| CBA | 0.31206 |
| CSI | -0.02617 |
| Chi-Squared | 2426.2028 |
| Chi-Squared DF | 36 |
| Conditional Entropy | 1.43491 |
| Cramer V | 0.36281 |
| Cross Entropy | 2.58562 |
| F1 Macro | 0.37759 |
| F1 Micro | 0.55501 |
| FNR Macro | 0.64746 |
| FNR Micro | 0.44499 |
| FPR Macro | 0.08788 |
| FPR Micro | 0.07416 |
| Gwet AC1 | 0.49399 |
| Hamming Loss | 0.44499 |
| Joint Entropy | 3.75181 |
| KL Divergence | 0.26872 |
| Kappa | 0.38877 |
| Kappa 95% CI | (0.36463,0.41291) |
| Kappa No Prevalence | 0.11003 |
| Kappa Standard Error | 0.01232 |
| Kappa Unbiased | 0.38503 |
| Krippendorff Alpha | 0.38513 |
| Lambda A | 0.34803 |
| Lambda B | 0.33172 |
| Mutual Information | 0.34967 |
| NIR | 0.32096 |
| Overall ACC | 0.55501 |
| Overall CEN | 0.46199 |
| Overall J | (1.76909,0.25273) |
| Overall MCC | 0.39424 |
| Overall MCEN | 0.56332 |
| Overall RACC | 0.27199 |
| Overall RACCU | 0.27641 |
| P-Value | None |
| PPV Macro | 0.62129 |
| PPV Micro | 0.55501 |
| Pearson C | 0.66428 |
| Phi-Squared | 0.78978 |
| RCI | 0.15092 |
| RR | 438.85714 |
| Reference Entropy | 2.31689 |
| Response Entropy | 1.78458 |
| SOA1(Landis & Koch) | Fair |
| SOA2(Fleiss) | Poor |
| SOA3(Altman) | Fair |
| SOA4(Cicchetti) | Poor |
| SOA5(Cramer) | Moderate |
| SOA6(Matthews) | Weak |
| Scott PI | 0.38503 |
| Standard Error | 0.00897 |
| TNR Macro | 0.91212 |
| TNR Micro | 0.92584 |
| TPR Macro | 0.35254 |
| TPR Micro | 0.55501 |
| Zero-one Loss | 1367 |

## Class Statistics :

|  |  |  |  |  |  |  |  |  |
| --- | --- | --- | --- | --- | --- | --- | --- | --- |
| Class | 0 | 1 | 2 | 3 | 4 | 5 | 6 | Description |
| ACC | 0.80339 | 0.73796 | 0.73665 | 0.94727 | 0.9694 | 0.97331 | 0.94206 | Accuracy |
| AGF | 0.70714 | 0.70548 | 0.75418 | 0.53031 | 0.44945 | 0.32749 | 0.16644 | Adjusted F-score |
| AGM | 0.77543 | 0.73881 | 0.73657 | 0.74048 | 0.70269 | 0.64457 | 0.56273 | Adjusted geometric mean |
| AM | 66 | 57 | 325 | -132 | -86 | -80 | -150 | Difference between automatic and manual classification |
| AUC | 0.72067 | 0.70704 | 0.73355 | 0.62429 | 0.58648 | 0.54478 | 0.50949 | Area under the ROC curve |
| AUCI | Good | Good | Good | Fair | Poor | Poor | Poor | AUC value interpretation |
| AUPR | 0.55193 | 0.60373 | 0.62863 | 0.51152 | 0.5002 | 0.48939 | 0.12302 | Area under the PR curve |
| BCD | 0.01074 | 0.00928 | 0.0529 | 0.02148 | 0.014 | 0.01302 | 0.02441 | Bray-Curtis dissimilarity |
| BM | 0.44134 | 0.41407 | 0.4671 | 0.24859 | 0.17296 | 0.08955 | 0.01899 | Informedness or bookmaker informedness |
| CEN | 0.49582 | 0.44732 | 0.44421 | 0.48002 | 0.48477 | 0.42329 | 0.55639 | Confusion entropy |
| DOR | 8.6141 | 6.28348 | 7.58437 | 64.85261 | 156.16944 | 294.51852 | 5.03484 | Diagnostic odds ratio |
| DP | 0.51561 | 0.44007 | 0.48513 | 0.99897 | 1.20939 | 1.36129 | 0.38703 | Discriminant power |
| DPI | Poor | Poor | Poor | Poor | Limited | Limited | Poor | Discriminant power interpretation |
| ERR | 0.19661 | 0.26204 | 0.26335 | 0.05273 | 0.0306 | 0.02669 | 0.05794 | Error rate |
| F0.5 | 0.53484 | 0.59325 | 0.56119 | 0.54705 | 0.47264 | 0.32 | 0.08333 | F0.5 score |
| F1 | 0.5506 | 0.60325 | 0.61347 | 0.38168 | 0.28788 | 0.16327 | 0.04301 | F1 score - harmonic mean of precision and sensitivity |
| F2 | 0.56731 | 0.6136 | 0.6765 | 0.29308 | 0.20697 | 0.10959 | 0.02899 | F2 score |
| FDR | 0.47518 | 0.41323 | 0.46898 | 0.23077 | 0.17391 | 0.11111 | 0.77778 | False discovery rate |
| FN | 269 | 374 | 242 | 147 | 90 | 81 | 164 | False negative/miss/type 2 error |
| FNR | 0.42097 | 0.37931 | 0.27376 | 0.74619 | 0.82569 | 0.91011 | 0.97619 | Miss rate or false negative rate |
| FOR | 0.11365 | 0.18433 | 0.1299 | 0.04889 | 0.02952 | 0.02644 | 0.0537 | False omission rate |
| FP | 335 | 431 | 567 | 15 | 4 | 1 | 14 | False positive/type 1 error/false alarm |
| FPR | 0.13769 | 0.20662 | 0.25914 | 0.00522 | 0.00135 | 0.00034 | 0.00482 | Fall-out or false positive rate |
| G | 0.55126 | 0.60349 | 0.62101 | 0.44186 | 0.37947 | 0.28267 | 0.07274 | G-measure geometric mean of precision and sensitivity |
| GI | 0.44134 | 0.41407 | 0.4671 | 0.24859 | 0.17296 | 0.08955 | 0.01899 | Gini index |
| GM | 0.70661 | 0.70174 | 0.73352 | 0.50248 | 0.41722 | 0.29976 | 0.15393 | G-mean geometric mean of specificity and sensitivity |
| IBA | 0.35786 | 0.4074 | 0.53018 | 0.0654 | 0.03058 | 0.00811 | 0.00068 | Index of balanced accuracy |
| ICSI | 0.10385 | 0.20746 | 0.25726 | 0.02304 | 0.0004 | -0.02122 | -0.75397 | Individual classification success index |
| IS | 1.33519 | 0.87038 | 0.88389 | 3.5844 | 4.54114 | 4.9393 | 2.02272 | Information score |
| J | 0.37988 | 0.4319 | 0.44245 | 0.23585 | 0.16814 | 0.08889 | 0.02198 | Jaccard index |
| LS | 2.52309 | 1.82815 | 1.84535 | 11.99531 | 23.28201 | 30.68165 | 4.06349 | Lift score |
| MCC | 0.42599 | 0.40822 | 0.43286 | 0.42317 | 0.37118 | 0.27791 | 0.05657 | Matthews correlation coefficient |
| MCCI | Weak | Weak | Weak | Weak | Weak | Negligible | Negligible | Matthews correlation coefficient interpretation |
| MCEN | 0.60382 | 0.55844 | 0.55856 | 0.52811 | 0.51425 | 0.4297 | 0.56006 | Modified confusion entropy |
| MK | 0.41118 | 0.40244 | 0.40112 | 0.72034 | 0.79657 | 0.86244 | 0.16852 | Markedness |
| N | 2433 | 2086 | 2188 | 2875 | 2963 | 2983 | 2904 | Condition negative |
| NLR | 0.48819 | 0.47809 | 0.36951 | 0.75011 | 0.8268 | 0.91042 | 0.98092 | Negative likelihood ratio |
| NLRI | Poor | Poor | Poor | Negligible | Negligible | Negligible | Negligible | Negative likelihood ratio interpretation |
| NPV | 0.88635 | 0.81567 | 0.8701 | 0.95111 | 0.97048 | 0.97356 | 0.9463 | Negative predictive value |
| OC | 0.57903 | 0.62069 | 0.72624 | 0.76923 | 0.82609 | 0.88889 | 0.22222 | Overlap coefficient |
| OOC | 0.55126 | 0.60349 | 0.62101 | 0.44186 | 0.37947 | 0.28267 | 0.07274 | Otsuka-Ochiai coefficient |
| OP | 0.60685 | 0.61583 | 0.72669 | 0.35382 | 0.26662 | 0.13831 | -0.01121 | Optimized precision |
| P | 639 | 986 | 884 | 197 | 109 | 89 | 168 | Condition positive or support |
| PLR | 4.20531 | 3.00408 | 2.80251 | 48.64636 | 129.12156 | 268.13483 | 4.93878 | Positive likelihood ratio |
| PLRI | Poor | Poor | Poor | Good | Good | Good | Poor | Positive likelihood ratio interpretation |
| POP | 3072 | 3072 | 3072 | 3072 | 3072 | 3072 | 3072 | Population |
| PPV | 0.52482 | 0.58677 | 0.53102 | 0.76923 | 0.82609 | 0.88889 | 0.22222 | Precision or positive predictive value |
| PRE | 0.20801 | 0.32096 | 0.28776 | 0.06413 | 0.03548 | 0.02897 | 0.05469 | Prevalence |
| Q | 0.79197 | 0.72541 | 0.76702 | 0.96963 | 0.98727 | 0.99323 | 0.66859 | Yule Q - coefficient of colligation |
| QI | Strong | Moderate | Strong | Strong | Strong | Strong | Moderate | Yule Q interpretation |
| RACC | 0.04774 | 0.10897 | 0.11325 | 0.00136 | 0.00027 | 8e-05 | 0.00032 | Random accuracy |
| RACCU | 0.04785 | 0.10906 | 0.11605 | 0.00182 | 0.00046 | 0.00025 | 0.00092 | Random accuracy unbiased |
| TN | 2098 | 1655 | 1621 | 2860 | 2959 | 2982 | 2890 | True negative/correct rejection |
| TNR | 0.86231 | 0.79338 | 0.74086 | 0.99478 | 0.99865 | 0.99966 | 0.99518 | Specificity or true negative rate |
| TON | 2367 | 2029 | 1863 | 3007 | 3049 | 3063 | 3054 | Test outcome negative |
| TOP | 705 | 1043 | 1209 | 65 | 23 | 9 | 18 | Test outcome positive |
| TP | 370 | 612 | 642 | 50 | 19 | 8 | 4 | True positive/hit |
| TPR | 0.57903 | 0.62069 | 0.72624 | 0.25381 | 0.17431 | 0.08989 | 0.02381 | Sensitivity, recall, hit rate, or true positive rate |
| Y | 0.44134 | 0.41407 | 0.4671 | 0.24859 | 0.17296 | 0.08955 | 0.01899 | Youden index |
| dInd | 0.44292 | 0.43193 | 0.37696 | 0.74621 | 0.82569 | 0.91011 | 0.9762 | Distance index |
| sInd | 0.68681 | 0.69458 | 0.73345 | 0.47235 | 0.41615 | 0.35645 | 0.30972 | Similarity index |

Generated By PyCM Version 3.1
