# Supplementary material for: TopEC: prediction of Enzyme Commission classes by 3D graph neural networks and localized 3D protein descriptor
Source: Nat Commun. 2025 Mar 20;16:2737. doi: 10.1038/s41467-025-57324-5 (PMC11923149; doi:10.1038/s41467-025-57324-5)
Supplement: Supplementary file 3 — Supplementary Data 1 [file 41467_2025_57324_MOESM3_ESM.zip › Data_S1/table1/mainclass/EnzyNet/local/Combined_TEMP.html]

Both\_TEMP\_enzynet\_none\_sites


# PyCM Report

## Dataset Type :

- Multi-Class Classification
- Imbalanced

Note 1 : Recommended statistics for this type of classification highlighted in aqua

Note 2 : The recommender system assumes that the input is the result of classification over the whole data rather than just a part of it.
If the confusion matrix is the result of test data classification, the recommendation is not valid.

## Confusion Matrix :

|  |  |  |  |  |  |  |  |  |  |  |  |  |  |  |  |  |  |  |  |  |  |  |  |  |  |  |  |  |  |  |  |  |  |  |  |  |  |  |  |  |  |  |  |  |  |  |  |  |  |  |  |  |  |  |  |  |  |  |  |  |  |  |  |  |  |
| --- | --- | --- | --- | --- | --- | --- | --- | --- | --- | --- | --- | --- | --- | --- | --- | --- | --- | --- | --- | --- | --- | --- | --- | --- | --- | --- | --- | --- | --- | --- | --- | --- | --- | --- | --- | --- | --- | --- | --- | --- | --- | --- | --- | --- | --- | --- | --- | --- | --- | --- | --- | --- | --- | --- | --- | --- | --- | --- | --- | --- | --- | --- | --- | --- | --- |
| Actual | Predict  |  |  |  |  |  |  |  |  | | --- | --- | --- | --- | --- | --- | --- | --- | |  | 0 | 1 | 2 | 3 | 4 | 5 | 6 | | 0 | 347 | 119 | 138 | 22 | 8 | 5 | 0 | | 1 | 123 | 613 | 209 | 21 | 6 | 10 | 4 | | 2 | 73 | 145 | 616 | 25 | 3 | 7 | 15 | | 3 | 37 | 30 | 58 | 70 | 1 | 1 | 0 | | 4 | 30 | 27 | 20 | 2 | 30 | 0 | 0 | | 5 | 10 | 26 | 26 | 3 | 0 | 24 | 0 | | 6 | 39 | 52 | 64 | 2 | 0 | 1 | 10 | |

## Overall Statistics :

|  |  |
| --- | --- |
| 95% CI | (0.53907,0.57421) |
| ACC Macro | 0.87333 |
| ARI | 0.19484 |
| AUNP | 0.69918 |
| AUNU | 0.65855 |
| Bangdiwala B | 0.35834 |
| Bennett S | 0.48275 |
| CBA | 0.37667 |
| CSI | -0.07845 |
| Chi-Squared | 2565.42644 |
| Chi-Squared DF | 36 |
| Conditional Entropy | 1.62224 |
| Cramer V | 0.37307 |
| Cross Entropy | 2.42804 |
| F1 Macro | 0.42902 |
| F1 Micro | 0.55664 |
| FNR Macro | 0.59695 |
| FNR Micro | 0.44336 |
| FPR Macro | 0.08595 |
| FPR Micro | 0.07389 |
| Gwet AC1 | 0.49458 |
| Hamming Loss | 0.44336 |
| Joint Entropy | 3.93913 |
| KL Divergence | 0.11115 |
| Kappa | 0.40018 |
| Kappa 95% CI | (0.37641,0.42394) |
| Kappa No Prevalence | 0.11328 |
| Kappa Standard Error | 0.01213 |
| Kappa Unbiased | 0.39825 |
| Krippendorff Alpha | 0.39835 |
| Lambda A | 0.34947 |
| Lambda B | 0.32715 |
| Mutual Information | 0.37156 |
| NIR | 0.32096 |
| Overall ACC | 0.55664 |
| Overall CEN | 0.48957 |
| Overall J | (2.00723,0.28675) |
| Overall MCC | 0.40304 |
| Overall MCEN | 0.60126 |
| Overall RACC | 0.26085 |
| Overall RACCU | 0.26322 |
| P-Value | None |
| PPV Macro | 0.5185 |
| PPV Micro | 0.55664 |
| Pearson C | 0.67459 |
| Phi-Squared | 0.8351 |
| RCI | 0.16037 |
| RR | 438.85714 |
| Reference Entropy | 2.31689 |
| Response Entropy | 1.9938 |
| SOA1(Landis & Koch) | Moderate |
| SOA2(Fleiss) | Intermediate to Good |
| SOA3(Altman) | Moderate |
| SOA4(Cicchetti) | Fair |
| SOA5(Cramer) | Moderate |
| SOA6(Matthews) | Weak |
| Scott PI | 0.39825 |
| Standard Error | 0.00896 |
| TNR Macro | 0.91405 |
| TNR Micro | 0.92611 |
| TPR Macro | 0.40305 |
| TPR Micro | 0.55664 |
| Zero-one Loss | 1362 |

## Class Statistics :

|  |  |  |  |  |  |  |  |  |
| --- | --- | --- | --- | --- | --- | --- | --- | --- |
| Class | 0 | 1 | 2 | 3 | 4 | 5 | 6 | Description |
| ACC | 0.80339 | 0.7487 | 0.74512 | 0.93424 | 0.96842 | 0.97103 | 0.94238 | Accuracy |
| AGF | 0.68816 | 0.71077 | 0.74479 | 0.60011 | 0.55049 | 0.53985 | 0.26124 | Adjusted F-score |
| AGM | 0.76924 | 0.74938 | 0.74437 | 0.7747 | 0.75422 | 0.75109 | 0.60777 | Adjusted geometric mean |
| AM | 20 | 26 | 247 | -52 | -61 | -41 | -139 | Difference between automatic and manual classification |
| AUC | 0.7074 | 0.71521 | 0.73073 | 0.66462 | 0.63458 | 0.63081 | 0.52649 | Area under the ROC curve |
| AUCI | Good | Good | Good | Fair | Fair | Fair | Poor | AUC value interpretation |
| AUPR | 0.5348 | 0.61372 | 0.62074 | 0.41904 | 0.45011 | 0.38483 | 0.20218 | Area under the PR curve |
| BCD | 0.00326 | 0.00423 | 0.0402 | 0.00846 | 0.00993 | 0.00667 | 0.02262 | Bray-Curtis dissimilarity |
| BM | 0.4148 | 0.43043 | 0.46146 | 0.32924 | 0.26915 | 0.26162 | 0.05298 | Informedness or bookmaker informedness |
| CEN | 0.52143 | 0.46001 | 0.46768 | 0.57541 | 0.53174 | 0.57997 | 0.55781 | Confusion entropy |
| DOR | 8.07854 | 6.94854 | 7.4668 | 20.57743 | 62.1308 | 45.52308 | 9.61026 | Diagnostic odds ratio |
| DP | 0.50024 | 0.46416 | 0.48138 | 0.72411 | 0.9887 | 0.91423 | 0.54181 | Discriminant power |
| DPI | Poor | Poor | Poor | Poor | Poor | Poor | Poor | Discriminant power interpretation |
| ERR | 0.19661 | 0.2513 | 0.25488 | 0.06576 | 0.03158 | 0.02897 | 0.05762 | Error rate |
| F0.5 | 0.52977 | 0.60886 | 0.56953 | 0.45045 | 0.49834 | 0.42705 | 0.17606 | F0.5 score |
| F1 | 0.53467 | 0.61361 | 0.61141 | 0.40936 | 0.38217 | 0.35036 | 0.10152 | F1 score - harmonic mean of precision and sensitivity |
| F2 | 0.53966 | 0.61844 | 0.65995 | 0.37513 | 0.30992 | 0.29703 | 0.07133 | F2 score |
| FDR | 0.47344 | 0.39427 | 0.45535 | 0.51724 | 0.375 | 0.5 | 0.65517 | False discovery rate |
| FN | 292 | 373 | 268 | 127 | 79 | 65 | 158 | False negative/miss/type 2 error |
| FNR | 0.45696 | 0.3783 | 0.30317 | 0.64467 | 0.72477 | 0.73034 | 0.94048 | Miss rate or false negative rate |
| FOR | 0.12101 | 0.18107 | 0.13807 | 0.04339 | 0.02612 | 0.02149 | 0.05192 | False omission rate |
| FP | 312 | 399 | 515 | 75 | 18 | 24 | 19 | False positive/type 1 error/false alarm |
| FPR | 0.12824 | 0.19128 | 0.23537 | 0.02609 | 0.00607 | 0.00805 | 0.00654 | Fall-out or false positive rate |
| G | 0.53473 | 0.61367 | 0.61606 | 0.41417 | 0.41475 | 0.36719 | 0.14327 | G-measure geometric mean of precision and sensitivity |
| GI | 0.4148 | 0.43043 | 0.46146 | 0.32924 | 0.26915 | 0.26162 | 0.05298 | Gini index |
| GM | 0.68804 | 0.70907 | 0.72994 | 0.58827 | 0.52303 | 0.5172 | 0.24318 | G-mean geometric mean of specificity and sensitivity |
| IBA | 0.31778 | 0.40876 | 0.49669 | 0.13199 | 0.07695 | 0.07429 | 0.00391 | Index of balanced accuracy |
| ICSI | 0.06959 | 0.22744 | 0.24148 | -0.16191 | -0.09977 | -0.23034 | -0.59565 | Individual classification success index |
| IS | 1.33995 | 0.91627 | 0.92046 | 2.91228 | 4.13871 | 4.10923 | 2.65659 | Information score |
| J | 0.36488 | 0.4426 | 0.44031 | 0.25735 | 0.23622 | 0.21239 | 0.05348 | Jaccard index |
| LS | 2.53142 | 1.88723 | 1.89272 | 7.52809 | 17.61468 | 17.25843 | 6.30542 | Lift score |
| MCC | 0.41015 | 0.42754 | 0.43315 | 0.38034 | 0.40148 | 0.35382 | 0.12457 | Matthews correlation coefficient |
| MCCI | Weak | Weak | Weak | Weak | Weak | Weak | Negligible | Matthews correlation coefficient interpretation |
| MCEN | 0.63218 | 0.58141 | 0.59143 | 0.65506 | 0.59217 | 0.64211 | 0.5678 | Modified confusion entropy |
| MK | 0.40554 | 0.42466 | 0.40658 | 0.43937 | 0.59888 | 0.47851 | 0.29291 | Markedness |
| N | 2433 | 2086 | 2188 | 2875 | 2963 | 2983 | 2904 | Condition negative |
| NLR | 0.52418 | 0.46777 | 0.39649 | 0.66194 | 0.7292 | 0.73626 | 0.94667 | Negative likelihood ratio |
| NLRI | Negligible | Poor | Poor | Negligible | Negligible | Negligible | Negligible | Negative likelihood ratio interpretation |
| NPV | 0.87899 | 0.81893 | 0.86193 | 0.95661 | 0.97388 | 0.97851 | 0.94808 | Negative predictive value |
| OC | 0.54304 | 0.6217 | 0.69683 | 0.48276 | 0.625 | 0.5 | 0.34483 | Overlap coefficient |
| OOC | 0.53473 | 0.61367 | 0.61606 | 0.41417 | 0.41475 | 0.36719 | 0.14327 | Otsuka-Ochiai coefficient |
| OP | 0.57104 | 0.61795 | 0.69873 | 0.46888 | 0.40215 | 0.39852 | 0.05544 | Optimized precision |
| P | 639 | 986 | 884 | 197 | 109 | 89 | 168 | Condition positive or support |
| PLR | 4.23464 | 3.25031 | 2.96052 | 13.62098 | 45.30581 | 33.51685 | 9.09774 | Positive likelihood ratio |
| PLRI | Poor | Poor | Poor | Good | Good | Good | Fair | Positive likelihood ratio interpretation |
| POP | 3072 | 3072 | 3072 | 3072 | 3072 | 3072 | 3072 | Population |
| PPV | 0.52656 | 0.60573 | 0.54465 | 0.48276 | 0.625 | 0.5 | 0.34483 | Precision or positive predictive value |
| PRE | 0.20801 | 0.32096 | 0.28776 | 0.06413 | 0.03548 | 0.02897 | 0.05469 | Prevalence |
| Q | 0.7797 | 0.74838 | 0.76378 | 0.90731 | 0.96832 | 0.95701 | 0.8115 | Yule Q - coefficient of colligation |
| QI | Strong | Moderate | Strong | Strong | Strong | Strong | Strong | Yule Q interpretation |
| RACC | 0.04462 | 0.10573 | 0.10594 | 0.00303 | 0.00055 | 0.00045 | 0.00052 | Random accuracy |
| RACCU | 0.04463 | 0.10575 | 0.10756 | 0.0031 | 0.00065 | 0.0005 | 0.00103 | Random accuracy unbiased |
| TN | 2121 | 1687 | 1673 | 2800 | 2945 | 2959 | 2885 | True negative/correct rejection |
| TNR | 0.87176 | 0.80872 | 0.76463 | 0.97391 | 0.99393 | 0.99195 | 0.99346 | Specificity or true negative rate |
| TON | 2413 | 2060 | 1941 | 2927 | 3024 | 3024 | 3043 | Test outcome negative |
| TOP | 659 | 1012 | 1131 | 145 | 48 | 48 | 29 | Test outcome positive |
| TP | 347 | 613 | 616 | 70 | 30 | 24 | 10 | True positive/hit |
| TPR | 0.54304 | 0.6217 | 0.69683 | 0.35533 | 0.27523 | 0.26966 | 0.05952 | Sensitivity, recall, hit rate, or true positive rate |
| Y | 0.4148 | 0.43043 | 0.46146 | 0.32924 | 0.26915 | 0.26162 | 0.05298 | Youden index |
| dInd | 0.47462 | 0.4239 | 0.38381 | 0.6452 | 0.7248 | 0.73038 | 0.9405 | Distance index |
| sInd | 0.6644 | 0.70025 | 0.7286 | 0.54378 | 0.48749 | 0.48354 | 0.33497 | Similarity index |

Generated By PyCM Version 3.1
