# Supplementary material for: TopEC: prediction of Enzyme Commission classes by 3D graph neural networks and localized 3D protein descriptor
Source: Nat Commun. 2025 Mar 20;16:2737. doi: 10.1038/s41467-025-57324-5 (PMC11923149; doi:10.1038/s41467-025-57324-5)
Supplement: Supplementary file 3 — Supplementary Data 1 [file 41467_2025_57324_MOESM3_ESM.zip › Data_S1/table1/mainclass/EnzyNet/local/Combined_FOLD_wflips.html]

Both\_FOLD\_enzynet\_wflips\_sites


# PyCM Report

## Dataset Type :

- Multi-Class Classification
- Imbalanced

Note 1 : Recommended statistics for this type of classification highlighted in aqua

Note 2 : The recommender system assumes that the input is the result of classification over the whole data rather than just a part of it.
If the confusion matrix is the result of test data classification, the recommendation is not valid.

## Confusion Matrix :

|  |  |  |  |  |  |  |  |  |  |  |  |  |  |  |  |  |  |  |  |  |  |  |  |  |  |  |  |  |  |  |  |  |  |  |  |  |  |  |  |  |  |  |  |  |  |  |  |  |  |  |  |  |  |  |  |  |  |  |  |  |  |  |  |  |  |
| --- | --- | --- | --- | --- | --- | --- | --- | --- | --- | --- | --- | --- | --- | --- | --- | --- | --- | --- | --- | --- | --- | --- | --- | --- | --- | --- | --- | --- | --- | --- | --- | --- | --- | --- | --- | --- | --- | --- | --- | --- | --- | --- | --- | --- | --- | --- | --- | --- | --- | --- | --- | --- | --- | --- | --- | --- | --- | --- | --- | --- | --- | --- | --- | --- | --- |
| Actual | Predict  |  |  |  |  |  |  |  |  | | --- | --- | --- | --- | --- | --- | --- | --- | |  | 0 | 1 | 2 | 3 | 4 | 5 | 6 | | 0 | 303 | 91 | 176 | 7 | 0 | 0 | 0 | | 1 | 51 | 805 | 194 | 2 | 0 | 0 | 0 | | 2 | 57 | 132 | 386 | 7 | 0 | 0 | 0 | | 3 | 47 | 65 | 94 | 8 | 1 | 0 | 0 | | 4 | 54 | 60 | 121 | 18 | 3 | 0 | 0 | | 5 | 24 | 48 | 62 | 0 | 0 | 7 | 0 | | 6 | 2 | 21 | 32 | 0 | 0 | 0 | 0 | |

## Overall Statistics :

|  |  |
| --- | --- |
| 95% CI | (0.50712,0.54361) |
| ACC Macro | 0.86439 |
| ARI | 0.20606 |
| AUNP | 0.68031 |
| AUNU | 0.60091 |
| Bangdiwala B | 0.3992 |
| Bennett S | 0.44626 |
| CBA | 0.23499 |
| CSI | None |
| Chi-Squared | None |
| Chi-Squared DF | 36 |
| Conditional Entropy | 1.31489 |
| Cramer V | None |
| Cross Entropy | 2.95205 |
| F1 Macro | 0.27145 |
| F1 Micro | 0.52536 |
| FNR Macro | 0.70684 |
| FNR Micro | 0.47464 |
| FPR Macro | 0.09134 |
| FPR Micro | 0.07911 |
| Gwet AC1 | 0.46067 |
| Hamming Loss | 0.47464 |
| Joint Entropy | 3.68915 |
| KL Divergence | None |
| Kappa | 0.35084 |
| Kappa 95% CI | (0.32589,0.37579) |
| Kappa No Prevalence | 0.05073 |
| Kappa Standard Error | 0.01273 |
| Kappa Unbiased | 0.34055 |
| Krippendorff Alpha | 0.34066 |
| Lambda A | 0.25739 |
| Lambda B | 0.35085 |
| Mutual Information | 0.31615 |
| NIR | 0.36553 |
| Overall ACC | 0.52536 |
| Overall CEN | 0.46125 |
| Overall J | (1.3207,0.18867) |
| Overall MCC | 0.36348 |
| Overall MCEN | 0.55898 |
| Overall RACC | 0.26885 |
| Overall RACCU | 0.28026 |
| P-Value | None |
| PPV Macro | None |
| PPV Micro | 0.52536 |
| Pearson C | None |
| Phi-Squared | None |
| RCI | 0.13316 |
| RR | 411.14286 |
| Reference Entropy | 2.37426 |
| Response Entropy | 1.63104 |
| SOA1(Landis & Koch) | Fair |
| SOA2(Fleiss) | Poor |
| SOA3(Altman) | Fair |
| SOA4(Cicchetti) | Poor |
| SOA5(Cramer) | None |
| SOA6(Matthews) | Weak |
| Scott PI | 0.34055 |
| Standard Error | 0.00931 |
| TNR Macro | 0.90866 |
| TNR Micro | 0.92089 |
| TPR Macro | 0.29316 |
| TPR Micro | 0.52536 |
| Zero-one Loss | 1366 |

## Class Statistics :

|  |  |  |  |  |  |  |  |  |
| --- | --- | --- | --- | --- | --- | --- | --- | --- |
| Class | 0 | 1 | 2 | 3 | 4 | 5 | 6 | Description |
| ACC | 0.82314 | 0.76928 | 0.69597 | 0.91626 | 0.91174 | 0.95344 | 0.98089 | Accuracy |
| AGF | 0.68671 | 0.78613 | 0.69402 | 0.204 | 0.11638 | 0.24287 | 0.0 | Adjusted F-score |
| AGM | 0.7805 | 0.76966 | 0.69268 | 0.57401 | 0.53318 | 0.60165 | 0 | Adjusted geometric mean |
| AM | -39 | 170 | 483 | -173 | -252 | -134 | -55 | Difference between automatic and manual classification |
| AUC | 0.7115 | 0.76842 | 0.68375 | 0.51222 | 0.50567 | 0.52482 | 0.5 | Area under the ROC curve |
| AUCI | Good | Good | Fair | Poor | Poor | Poor | Poor | AUC value interpretation |
| AUPR | 0.54416 | 0.71198 | 0.51284 | 0.11384 | 0.38086 | 0.52482 | None | Area under the PR curve |
| BCD | 0.00678 | 0.02953 | 0.08391 | 0.03006 | 0.04378 | 0.02328 | 0.00956 | Bray-Curtis dissimilarity |
| BM | 0.423 | 0.53684 | 0.3675 | 0.02444 | 0.01134 | 0.04965 | 0.0 | Informedness or bookmaker informedness |
| CEN | 0.48087 | 0.36927 | 0.5586 | 0.59082 | 0.4938 | 0.41236 | 0.32325 | Confusion entropy |
| DOR | 9.72198 | 11.01219 | 4.68999 | 2.98835 | 31.07905 | None | None | Diagnostic odds ratio |
| DP | 0.54458 | 0.57442 | 0.37004 | 0.26212 | 0.82284 | None | None | Discriminant power |
| DPI | Poor | Poor | Poor | Poor | Poor | None | None | Discriminant power interpretation |
| ERR | 0.17686 | 0.23072 | 0.30403 | 0.08374 | 0.08826 | 0.04656 | 0.01911 | Error rate |
| F0.5 | 0.55515 | 0.67761 | 0.3986 | 0.10444 | 0.05515 | 0.2071 | 0.0 | F0.5 score |
| F1 | 0.5435 | 0.708 | 0.46873 | 0.06226 | 0.02308 | 0.09459 | 0.0 | F1 score - harmonic mean of precision and sensitivity |
| F2 | 0.53233 | 0.74125 | 0.56882 | 0.04435 | 0.01459 | 0.0613 | 0.0 | F2 score |
| FDR | 0.4368 | 0.34124 | 0.63756 | 0.80952 | 0.25 | 0.0 | None | False discovery rate |
| FN | 274 | 247 | 196 | 207 | 253 | 134 | 55 | False negative/miss/type 2 error |
| FNR | 0.47487 | 0.23479 | 0.33677 | 0.96279 | 0.98828 | 0.95035 | 1.0 | Miss rate or false negative rate |
| FOR | 0.11709 | 0.14915 | 0.10811 | 0.07299 | 0.08803 | 0.04667 | 0.01911 | False omission rate |
| FP | 235 | 417 | 679 | 34 | 1 | 0 | 0 | False positive/type 1 error/false alarm |
| FPR | 0.10213 | 0.22837 | 0.29573 | 0.01277 | 0.00038 | 0.0 | 0.0 | Fall-out or false positive rate |
| G | 0.54383 | 0.70999 | 0.49029 | 0.08419 | 0.09375 | 0.22281 | None | G-measure geometric mean of precision and sensitivity |
| GI | 0.423 | 0.53684 | 0.3675 | 0.02444 | 0.01134 | 0.04965 | 0.0 | Gini index |
| GM | 0.68666 | 0.76841 | 0.68344 | 0.19166 | 0.10823 | 0.22281 | 0.0 | G-mean geometric mean of specificity and sensitivity |
| IBA | 0.29575 | 0.58667 | 0.44792 | 0.00184 | 0.00014 | 0.00246 | 0.0 | Index of balanced accuracy |
| ICSI | 0.08833 | 0.42397 | 0.02567 | -0.77231 | -0.23828 | 0.04965 | None | Individual classification success index |
| IS | 1.49013 | 0.84975 | 0.84179 | 1.35034 | 3.07581 | 4.3513 | None | Information score |
| J | 0.37315 | 0.54799 | 0.30611 | 0.03213 | 0.01167 | 0.04965 | 0.0 | Jaccard index |
| LS | 2.80915 | 1.80219 | 1.79228 | 2.54972 | 8.43164 | 20.41135 | None | Lift score |
| MCC | 0.4344 | 0.52304 | 0.30572 | 0.05359 | 0.08663 | 0.21755 | None | Matthews correlation coefficient |
| MCCI | Weak | Moderate | Weak | Negligible | Negligible | Negligible | None | Matthews correlation coefficient interpretation |
| MCEN | 0.58032 | 0.49214 | 0.65502 | 0.59748 | 0.49494 | 0.4143 | 0.32325 | Modified confusion entropy |
| MK | 0.4461 | 0.5096 | 0.25433 | 0.11749 | 0.66197 | 0.95333 | None | Markedness |
| N | 2301 | 1826 | 2296 | 2663 | 2622 | 2737 | 2823 | Condition negative |
| NLR | 0.52888 | 0.30428 | 0.47818 | 0.97524 | 0.98866 | 0.95035 | 1.0 | Negative likelihood ratio |
| NLRI | Negligible | Poor | Poor | Negligible | Negligible | Negligible | Negligible | Negative likelihood ratio interpretation |
| NPV | 0.88291 | 0.85085 | 0.89189 | 0.92701 | 0.91197 | 0.95333 | 0.98089 | Negative predictive value |
| OC | 0.5632 | 0.76521 | 0.66323 | 0.19048 | 0.75 | 1.0 | None | Overlap coefficient |
| OOC | 0.54383 | 0.70999 | 0.49029 | 0.08419 | 0.09375 | 0.22281 | None | Otsuka-Ochiai coefficient |
| OP | 0.5612 | 0.7651 | 0.66596 | -0.0111 | -0.06508 | 0.04803 | -0.01911 | Optimized precision |
| P | 577 | 1052 | 582 | 215 | 256 | 141 | 55 | Condition positive or support |
| PLR | 5.1418 | 3.35077 | 2.24268 | 2.91436 | 30.72656 | None | None | Positive likelihood ratio |
| PLRI | Fair | Poor | Poor | Poor | Good | None | None | Positive likelihood ratio interpretation |
| POP | 2878 | 2878 | 2878 | 2878 | 2878 | 2878 | 2878 | Population |
| PPV | 0.5632 | 0.65876 | 0.36244 | 0.19048 | 0.75 | 1.0 | None | Precision or positive predictive value |
| PRE | 0.20049 | 0.36553 | 0.20222 | 0.0747 | 0.08895 | 0.04899 | 0.01911 | Prevalence |
| Q | 0.81347 | 0.8335 | 0.64851 | 0.49854 | 0.93765 | None | None | Yule Q - coefficient of colligation |
| QI | Strong | Strong | Moderate | Weak | Strong | None | None | Yule Q interpretation |
| RACC | 0.03748 | 0.1552 | 0.07483 | 0.00109 | 0.00012 | 0.00012 | 0.0 | Random accuracy |
| RACCU | 0.03752 | 0.15608 | 0.08187 | 0.00199 | 0.00204 | 0.00066 | 9e-05 | Random accuracy unbiased |
| TN | 2066 | 1409 | 1617 | 2629 | 2621 | 2737 | 2823 | True negative/correct rejection |
| TNR | 0.89787 | 0.77163 | 0.70427 | 0.98723 | 0.99962 | 1.0 | 1.0 | Specificity or true negative rate |
| TON | 2340 | 1656 | 1813 | 2836 | 2874 | 2871 | 2878 | Test outcome negative |
| TOP | 538 | 1222 | 1065 | 42 | 4 | 7 | 0 | Test outcome positive |
| TP | 303 | 805 | 386 | 8 | 3 | 7 | 0 | True positive/hit |
| TPR | 0.52513 | 0.76521 | 0.66323 | 0.03721 | 0.01172 | 0.04965 | 0.0 | Sensitivity, recall, hit rate, or true positive rate |
| Y | 0.423 | 0.53684 | 0.3675 | 0.02444 | 0.01134 | 0.04965 | 0.0 | Youden index |
| dInd | 0.48573 | 0.32753 | 0.44819 | 0.96288 | 0.98828 | 0.95035 | 1.0 | Distance index |
| sInd | 0.65654 | 0.7684 | 0.68308 | 0.31914 | 0.30118 | 0.328 | 0.29289 | Similarity index |

Generated By PyCM Version 3.1
