# Supplementary material for: TopEC: prediction of Enzyme Commission classes by 3D graph neural networks and localized 3D protein descriptor
Source: Nat Commun. 2025 Mar 20;16:2737. doi: 10.1038/s41467-025-57324-5 (PMC11923149; doi:10.1038/s41467-025-57324-5)
Supplement: Supplementary file 3 — Supplementary Data 1 [file 41467_2025_57324_MOESM3_ESM.zip › Data_S1/table1/mainclass/EnzyNet/local/Combined_FOLD_flips.html]

Both\_FOLD\_enzynet\_flips\_sites


# PyCM Report

## Dataset Type :

- Multi-Class Classification
- Imbalanced

Note 1 : Recommended statistics for this type of classification highlighted in aqua

Note 2 : The recommender system assumes that the input is the result of classification over the whole data rather than just a part of it.
If the confusion matrix is the result of test data classification, the recommendation is not valid.

## Confusion Matrix :

|  |  |  |  |  |  |  |  |  |  |  |  |  |  |  |  |  |  |  |  |  |  |  |  |  |  |  |  |  |  |  |  |  |  |  |  |  |  |  |  |  |  |  |  |  |  |  |  |  |  |  |  |  |  |  |  |  |  |  |  |  |  |  |  |  |  |
| --- | --- | --- | --- | --- | --- | --- | --- | --- | --- | --- | --- | --- | --- | --- | --- | --- | --- | --- | --- | --- | --- | --- | --- | --- | --- | --- | --- | --- | --- | --- | --- | --- | --- | --- | --- | --- | --- | --- | --- | --- | --- | --- | --- | --- | --- | --- | --- | --- | --- | --- | --- | --- | --- | --- | --- | --- | --- | --- | --- | --- | --- | --- | --- | --- | --- |
| Actual | Predict  |  |  |  |  |  |  |  |  | | --- | --- | --- | --- | --- | --- | --- | --- | |  | 0 | 1 | 2 | 3 | 4 | 5 | 6 | | 0 | 302 | 85 | 182 | 8 | 0 | 0 | 0 | | 1 | 59 | 798 | 194 | 1 | 0 | 0 | 0 | | 2 | 61 | 144 | 375 | 2 | 0 | 0 | 0 | | 3 | 48 | 69 | 91 | 6 | 1 | 0 | 0 | | 4 | 62 | 58 | 115 | 21 | 0 | 0 | 0 | | 5 | 27 | 44 | 65 | 0 | 0 | 5 | 0 | | 6 | 3 | 19 | 33 | 0 | 0 | 0 | 0 | |

## Overall Statistics :

|  |  |
| --- | --- |
| 95% CI | (0.49807,0.53459) |
| ACC Macro | 0.86181 |
| ARI | 0.19869 |
| AUNP | 0.67449 |
| AUNU | 0.59562 |
| Bangdiwala B | 0.38995 |
| Bennett S | 0.43572 |
| CBA | 0.22828 |
| CSI | None |
| Chi-Squared | None |
| Chi-Squared DF | 36 |
| Conditional Entropy | 1.31616 |
| Cramer V | None |
| Cross Entropy | 3.15681 |
| F1 Macro | 0.25825 |
| F1 Micro | 0.51633 |
| FNR Macro | 0.71576 |
| FNR Micro | 0.48367 |
| FPR Macro | 0.093 |
| FPR Micro | 0.08061 |
| Gwet AC1 | 0.45039 |
| Hamming Loss | 0.48367 |
| Joint Entropy | 3.69042 |
| KL Divergence | None |
| Kappa | 0.33839 |
| Kappa 95% CI | (0.31341,0.36336) |
| Kappa No Prevalence | 0.03266 |
| Kappa Standard Error | 0.01274 |
| Kappa Unbiased | 0.32815 |
| Krippendorff Alpha | 0.32827 |
| Lambda A | 0.24644 |
| Lambda B | 0.33835 |
| Mutual Information | 0.30216 |
| NIR | 0.36553 |
| Overall ACC | 0.51633 |
| Overall CEN | 0.46581 |
| Overall J | (1.2602,0.18003) |
| Overall MCC | 0.35029 |
| Overall MCEN | 0.56215 |
| Overall RACC | 0.26895 |
| Overall RACCU | 0.28009 |
| P-Value | None |
| PPV Macro | None |
| PPV Micro | 0.51633 |
| Pearson C | None |
| Phi-Squared | None |
| RCI | 0.12726 |
| RR | 411.14286 |
| Reference Entropy | 2.37426 |
| Response Entropy | 1.61832 |
| SOA1(Landis & Koch) | Fair |
| SOA2(Fleiss) | Poor |
| SOA3(Altman) | Fair |
| SOA4(Cicchetti) | Poor |
| SOA5(Cramer) | None |
| SOA6(Matthews) | Weak |
| Scott PI | 0.32815 |
| Standard Error | 0.00932 |
| TNR Macro | 0.907 |
| TNR Micro | 0.91939 |
| TPR Macro | 0.28424 |
| TPR Micro | 0.51633 |
| Zero-one Loss | 1392 |

## Class Statistics :

|  |  |  |  |  |  |  |  |  |
| --- | --- | --- | --- | --- | --- | --- | --- | --- |
| Class | 0 | 1 | 2 | 3 | 4 | 5 | 6 | Description |
| ACC | 0.81411 | 0.76616 | 0.6918 | 0.91626 | 0.9107 | 0.95274 | 0.98089 | Accuracy |
| AGF | 0.68137 | 0.78159 | 0.68343 | 0.17703 | 0.0 | 0.20556 | 0.0 | Adjusted F-score |
| AGM | 0.77273 | 0.76686 | 0.68692 | 0.56107 | 0 | 0.58396 | 0 | Adjusted geometric mean |
| AM | -15 | 165 | 473 | -177 | -255 | -136 | -55 | Difference between automatic and manual classification |
| AUC | 0.7052 | 0.76455 | 0.67408 | 0.50795 | 0.49981 | 0.51773 | 0.5 | Area under the ROC curve |
| AUCI | Good | Good | Fair | Poor | Poor | Poor | Poor | AUC value interpretation |
| AUPR | 0.53038 | 0.70713 | 0.49989 | 0.0929 | 0.0 | 0.51773 | None | Area under the PR curve |
| BCD | 0.00261 | 0.02867 | 0.08218 | 0.03075 | 0.0443 | 0.02363 | 0.00956 | Bray-Curtis dissimilarity |
| BM | 0.4104 | 0.52909 | 0.34816 | 0.01589 | -0.00038 | 0.03546 | 0.0 | Informedness or bookmaker informedness |
| CEN | 0.49414 | 0.37146 | 0.56163 | 0.57761 | 0.5091 | 0.41606 | 0.33496 | Confusion entropy |
| DOR | 8.62073 | 10.54992 | 4.3052 | 2.36035 | 0.0 | None | None | Diagnostic odds ratio |
| DP | 0.51579 | 0.56415 | 0.34954 | 0.20563 | None | None | None | Discriminant power |
| DPI | Poor | Poor | Poor | Poor | None | None | None | Discriminant power interpretation |
| ERR | 0.18589 | 0.23384 | 0.3082 | 0.08374 | 0.0893 | 0.04726 | 0.01911 | Error rate |
| F0.5 | 0.53451 | 0.67399 | 0.39046 | 0.08174 | 0.0 | 0.15528 | 0.0 | F0.5 score |
| F1 | 0.53029 | 0.70339 | 0.45816 | 0.04743 | 0.0 | 0.06849 | 0.0 | F1 score - harmonic mean of precision and sensitivity |
| F2 | 0.52613 | 0.73548 | 0.55424 | 0.03341 | 0.0 | 0.04394 | 0.0 | F2 score |
| FDR | 0.46263 | 0.34429 | 0.64455 | 0.84211 | 1.0 | 0.0 | None | False discovery rate |
| FN | 275 | 254 | 207 | 209 | 256 | 136 | 55 | False negative/miss/type 2 error |
| FNR | 0.4766 | 0.24144 | 0.35567 | 0.97209 | 1.0 | 0.96454 | 1.0 | Miss rate or false negative rate |
| FOR | 0.11874 | 0.15292 | 0.11355 | 0.07359 | 0.08898 | 0.04734 | 0.01911 | False omission rate |
| FP | 260 | 419 | 680 | 32 | 1 | 0 | 0 | False positive/type 1 error/false alarm |
| FPR | 0.11299 | 0.22946 | 0.29617 | 0.01202 | 0.00038 | 0.0 | 0.0 | Fall-out or false positive rate |
| G | 0.53034 | 0.70526 | 0.47857 | 0.06638 | 0.0 | 0.18831 | None | G-measure geometric mean of precision and sensitivity |
| GI | 0.4104 | 0.52909 | 0.34816 | 0.01589 | -0.00038 | 0.03546 | 0.0 | Gini index |
| GM | 0.68136 | 0.76452 | 0.67342 | 0.16605 | 0.0 | 0.18831 | 0.0 | G-mean geometric mean of specificity and sensitivity |
| IBA | 0.29545 | 0.57749 | 0.42652 | 0.0011 | 0.0 | 0.00126 | 0.0 | Index of balanced accuracy |
| ICSI | 0.06076 | 0.41427 | -0.00022 | -0.8142 | -1.0 | 0.03546 | None | Individual classification success index |
| IS | 1.4224 | 0.84306 | 0.8137 | 1.07969 | None | 4.3513 | None | Information score |
| J | 0.36081 | 0.54249 | 0.29715 | 0.02429 | 0.0 | 0.03546 | 0.0 | Jaccard index |
| LS | 2.68031 | 1.79386 | 1.75771 | 2.11359 | 0.0 | 20.41135 | None | Lift score |
| MCC | 0.41449 | 0.51577 | 0.29021 | 0.0366 | -0.00583 | 0.1838 | None | Matthews correlation coefficient |
| MCCI | Weak | Moderate | Negligible | Negligible | Negligible | Negligible | None | Matthews correlation coefficient interpretation |
| MCEN | 0.59318 | 0.49318 | 0.65492 | 0.58221 | 0.5091 | 0.41728 | 0.33496 | Modified confusion entropy |
| MK | 0.41863 | 0.50279 | 0.2419 | 0.0843 | -0.08898 | 0.95266 | None | Markedness |
| N | 2301 | 1826 | 2296 | 2663 | 2622 | 2737 | 2823 | Condition negative |
| NLR | 0.53732 | 0.31335 | 0.50533 | 0.98392 | 1.00038 | 0.96454 | 1.0 | Negative likelihood ratio |
| NLRI | Negligible | Poor | Negligible | Negligible | Negligible | Negligible | Negligible | Negative likelihood ratio interpretation |
| NPV | 0.88126 | 0.84708 | 0.88645 | 0.92641 | 0.91102 | 0.95266 | 0.98089 | Negative predictive value |
| OC | 0.53737 | 0.75856 | 0.64433 | 0.15789 | 0.0 | 1.0 | None | Overlap coefficient |
| OOC | 0.53034 | 0.70526 | 0.47857 | 0.06638 | 0.0 | 0.18831 | None | Otsuka-Ochiai coefficient |
| OP | 0.5563 | 0.75832 | 0.64766 | -0.0288 | -0.0893 | 0.02124 | -0.01911 | Optimized precision |
| P | 577 | 1052 | 582 | 215 | 256 | 141 | 55 | Condition positive or support |
| PLR | 4.63206 | 3.30578 | 2.17556 | 2.32238 | 0.0 | None | None | Positive likelihood ratio |
| PLRI | Poor | Poor | Poor | Poor | Negligible | None | None | Positive likelihood ratio interpretation |
| POP | 2878 | 2878 | 2878 | 2878 | 2878 | 2878 | 2878 | Population |
| PPV | 0.53737 | 0.65571 | 0.35545 | 0.15789 | 0.0 | 1.0 | None | Precision or positive predictive value |
| PRE | 0.20049 | 0.36553 | 0.20222 | 0.0747 | 0.08895 | 0.04899 | 0.01911 | Prevalence |
| Q | 0.79212 | 0.82684 | 0.62301 | 0.40482 | -1.0 | None | None | Yule Q - coefficient of colligation |
| QI | Strong | Strong | Moderate | Weak | Negligible | None | None | Yule Q interpretation |
| RACC | 0.03915 | 0.15457 | 0.07413 | 0.00099 | 3e-05 | 9e-05 | 0.0 | Random accuracy |
| RACCU | 0.03916 | 0.15539 | 0.08088 | 0.00193 | 0.00199 | 0.00064 | 9e-05 | Random accuracy unbiased |
| TN | 2041 | 1407 | 1616 | 2631 | 2621 | 2737 | 2823 | True negative/correct rejection |
| TNR | 0.88701 | 0.77054 | 0.70383 | 0.98798 | 0.99962 | 1.0 | 1.0 | Specificity or true negative rate |
| TON | 2316 | 1661 | 1823 | 2840 | 2877 | 2873 | 2878 | Test outcome negative |
| TOP | 562 | 1217 | 1055 | 38 | 1 | 5 | 0 | Test outcome positive |
| TP | 302 | 798 | 375 | 6 | 0 | 5 | 0 | True positive/hit |
| TPR | 0.5234 | 0.75856 | 0.64433 | 0.02791 | 0.0 | 0.03546 | 0.0 | Sensitivity, recall, hit rate, or true positive rate |
| Y | 0.4104 | 0.52909 | 0.34816 | 0.01589 | -0.00038 | 0.03546 | 0.0 | Youden index |
| dInd | 0.48981 | 0.33309 | 0.46284 | 0.97217 | 1.0 | 0.96454 | 1.0 | Distance index |
| sInd | 0.65365 | 0.76447 | 0.67273 | 0.31257 | 0.29289 | 0.31797 | 0.29289 | Similarity index |

Generated By PyCM Version 3.1
