# Supplementary material for: TopEC: prediction of Enzyme Commission classes by 3D graph neural networks and localized 3D protein descriptor
Source: Nat Commun. 2025 Mar 20;16:2737. doi: 10.1038/s41467-025-57324-5 (PMC11923149; doi:10.1038/s41467-025-57324-5)
Supplement: Supplementary file 3 — Supplementary Data 1 [file 41467_2025_57324_MOESM3_ESM.zip › Data_S1/table1/mainclass/EnzyNet/local/Combined_FOLD.html]

Both\_FOLD\_enzynet\_none\_sites


# PyCM Report

## Dataset Type :

- Multi-Class Classification
- Imbalanced

Note 1 : Recommended statistics for this type of classification highlighted in aqua

Note 2 : The recommender system assumes that the input is the result of classification over the whole data rather than just a part of it.
If the confusion matrix is the result of test data classification, the recommendation is not valid.

## Confusion Matrix :

|  |  |  |  |  |  |  |  |  |  |  |  |  |  |  |  |  |  |  |  |  |  |  |  |  |  |  |  |  |  |  |  |  |  |  |  |  |  |  |  |  |  |  |  |  |  |  |  |  |  |  |  |  |  |  |  |  |  |  |  |  |  |  |  |  |  |
| --- | --- | --- | --- | --- | --- | --- | --- | --- | --- | --- | --- | --- | --- | --- | --- | --- | --- | --- | --- | --- | --- | --- | --- | --- | --- | --- | --- | --- | --- | --- | --- | --- | --- | --- | --- | --- | --- | --- | --- | --- | --- | --- | --- | --- | --- | --- | --- | --- | --- | --- | --- | --- | --- | --- | --- | --- | --- | --- | --- | --- | --- | --- | --- | --- | --- |
| Actual | Predict  |  |  |  |  |  |  |  |  | | --- | --- | --- | --- | --- | --- | --- | --- | |  | 0 | 1 | 2 | 3 | 4 | 5 | 6 | | 0 | 260 | 128 | 163 | 18 | 0 | 7 | 1 | | 1 | 68 | 781 | 190 | 8 | 0 | 5 | 0 | | 2 | 76 | 128 | 348 | 24 | 3 | 3 | 0 | | 3 | 44 | 63 | 85 | 19 | 3 | 1 | 0 | | 4 | 49 | 53 | 116 | 21 | 14 | 3 | 0 | | 5 | 39 | 39 | 53 | 1 | 1 | 8 | 0 | | 6 | 5 | 23 | 27 | 0 | 0 | 0 | 0 | |

## Overall Statistics :

|  |  |
| --- | --- |
| 95% CI | (0.47861,0.51514) |
| ACC Macro | 0.85625 |
| ARI | 0.18349 |
| AUNP | 0.66353 |
| AUNU | 0.59413 |
| Bangdiwala B | 0.36486 |
| Bennett S | 0.41302 |
| CBA | 0.23537 |
| CSI | -0.33711 |
| Chi-Squared | 1142.96194 |
| Chi-Squared DF | 36 |
| Conditional Entropy | 1.52418 |
| Cramer V | 0.25727 |
| Cross Entropy | 2.8052 |
| F1 Macro | 0.27424 |
| F1 Micro | 0.49687 |
| FNR Macro | 0.71561 |
| FNR Micro | 0.50313 |
| FPR Macro | 0.09613 |
| FPR Micro | 0.08385 |
| Gwet AC1 | 0.42748 |
| Hamming Loss | 0.50313 |
| Joint Entropy | 3.89844 |
| KL Divergence | 0.43093 |
| Kappa | 0.31596 |
| Kappa 95% CI | (0.29112,0.34079) |
| Kappa No Prevalence | -0.00625 |
| Kappa Standard Error | 0.01267 |
| Kappa Unbiased | 0.30817 |
| Krippendorff Alpha | 0.30829 |
| Lambda A | 0.2103 |
| Lambda B | 0.2736 |
| Mutual Information | 0.26018 |
| NIR | 0.36553 |
| Overall ACC | 0.49687 |
| Overall CEN | 0.50779 |
| Overall J | (1.28422,0.18346) |
| Overall MCC | 0.32402 |
| Overall MCEN | 0.6094 |
| Overall RACC | 0.26448 |
| Overall RACCU | 0.27276 |
| P-Value | None |
| PPV Macro | 0.3785 |
| PPV Micro | 0.49687 |
| Pearson C | 0.53315 |
| Phi-Squared | 0.39714 |
| RCI | 0.10958 |
| RR | 411.14286 |
| Reference Entropy | 2.37426 |
| Response Entropy | 1.78436 |
| SOA1(Landis & Koch) | Fair |
| SOA2(Fleiss) | Poor |
| SOA3(Altman) | Fair |
| SOA4(Cicchetti) | Poor |
| SOA5(Cramer) | Moderate |
| SOA6(Matthews) | Weak |
| Scott PI | 0.30817 |
| Standard Error | 0.00932 |
| TNR Macro | 0.90387 |
| TNR Micro | 0.91615 |
| TPR Macro | 0.28439 |
| TPR Micro | 0.49687 |
| Zero-one Loss | 1448 |

## Class Statistics :

|  |  |  |  |  |  |  |  |  |
| --- | --- | --- | --- | --- | --- | --- | --- | --- |
| Class | 0 | 1 | 2 | 3 | 4 | 5 | 6 | Description |
| ACC | 0.79222 | 0.75504 | 0.6984 | 0.90688 | 0.91348 | 0.94719 | 0.98054 | Accuracy |
| AGF | 0.62899 | 0.76886 | 0.66494 | 0.30611 | 0.24967 | 0.25504 | 0.0 | Adjusted F-score |
| AGM | 0.73955 | 0.75619 | 0.68717 | 0.61991 | 0.59766 | 0.60573 | 0 | Adjusted geometric mean |
| AM | -36 | 163 | 400 | -124 | -235 | -114 | -54 | Difference between automatic and manual classification |
| AUC | 0.66424 | 0.75236 | 0.6609 | 0.53067 | 0.52601 | 0.5249 | 0.49982 | Area under the ROC curve |
| AUCI | Fair | Good | Fair | Poor | Poor | Poor | Poor | AUC value interpretation |
| AUPR | 0.4656 | 0.6926 | 0.47616 | 0.14858 | 0.36068 | 0.17652 | 0.0 | Area under the PR curve |
| BCD | 0.00625 | 0.02832 | 0.06949 | 0.02154 | 0.04083 | 0.01981 | 0.00938 | Bray-Curtis dissimilarity |
| BM | 0.32849 | 0.50472 | 0.32181 | 0.06133 | 0.05202 | 0.0498 | -0.00035 | Informedness or bookmaker informedness |
| CEN | 0.56022 | 0.39229 | 0.59063 | 0.67941 | 0.54338 | 0.60939 | 0.40436 | Confusion entropy |
| DOR | 5.89602 | 9.24339 | 3.89857 | 3.48845 | 21.61157 | 8.60467 | 0.0 | Diagnostic odds ratio |
| DP | 0.42483 | 0.53249 | 0.32578 | 0.29917 | 0.73585 | 0.51535 | None | Discriminant power |
| DPI | Poor | Poor | Poor | Poor | Poor | Poor | None | Discriminant power interpretation |
| ERR | 0.20778 | 0.24496 | 0.3016 | 0.09312 | 0.08652 | 0.05281 | 0.01946 | Error rate |
| F0.5 | 0.47428 | 0.66052 | 0.38581 | 0.16408 | 0.20588 | 0.16064 | 0.0 | F0.5 score |
| F1 | 0.46512 | 0.68902 | 0.44501 | 0.12418 | 0.10108 | 0.09524 | 0.0 | F1 score - harmonic mean of precision and sensitivity |
| F2 | 0.4563 | 0.72008 | 0.52568 | 0.09989 | 0.06699 | 0.06768 | 0.0 | F2 score |
| FDR | 0.51941 | 0.3572 | 0.64562 | 0.79121 | 0.33333 | 0.7037 | 1.0 | False discovery rate |
| FN | 317 | 271 | 234 | 196 | 242 | 133 | 55 | False negative/miss/type 2 error |
| FNR | 0.54939 | 0.2576 | 0.40206 | 0.91163 | 0.94531 | 0.94326 | 1.0 | Miss rate or false negative rate |
| FOR | 0.13564 | 0.16296 | 0.12342 | 0.07033 | 0.0847 | 0.04665 | 0.01912 | False omission rate |
| FP | 281 | 434 | 634 | 72 | 7 | 19 | 1 | False positive/type 1 error/false alarm |
| FPR | 0.12212 | 0.23768 | 0.27613 | 0.02704 | 0.00267 | 0.00694 | 0.00035 | Fall-out or false positive rate |
| G | 0.46536 | 0.6908 | 0.46032 | 0.13584 | 0.19094 | 0.12966 | 0.0 | G-measure geometric mean of precision and sensitivity |
| GI | 0.32849 | 0.50472 | 0.32181 | 0.06133 | 0.05202 | 0.0498 | -0.00035 | Gini index |
| GM | 0.62895 | 0.75229 | 0.6579 | 0.29323 | 0.23354 | 0.23737 | 0.0 | G-mean geometric mean of specificity and sensitivity |
| IBA | 0.22656 | 0.55467 | 0.37832 | 0.00992 | 0.00313 | 0.00359 | 0.0 | Index of balanced accuracy |
| ICSI | -0.0688 | 0.38519 | -0.04768 | -0.70284 | -0.27865 | -0.64697 | -1.0 | Individual classification success index |
| IS | 1.26131 | 0.81437 | 0.80934 | 1.48279 | 2.90589 | 2.59641 | None | Information score |
| J | 0.30303 | 0.52557 | 0.28618 | 0.0662 | 0.05323 | 0.05 | 0.0 | Jaccard index |
| LS | 2.39713 | 1.75853 | 1.75241 | 2.79489 | 7.49479 | 6.04781 | 0.0 | Lift score |
| MCC | 0.33662 | 0.49212 | 0.27263 | 0.09216 | 0.17399 | 0.1115 | -0.0026 | Matthews correlation coefficient |
| MCCI | Weak | Weak | Negligible | Negligible | Negligible | Negligible | Negligible | Matthews correlation coefficient interpretation |
| MCEN | 0.65574 | 0.51783 | 0.68736 | 0.70029 | 0.55254 | 0.62121 | 0.40436 | Modified confusion entropy |
| MK | 0.34495 | 0.47984 | 0.23096 | 0.13846 | 0.58196 | 0.24965 | -0.01912 | Markedness |
| N | 2301 | 1826 | 2296 | 2663 | 2622 | 2737 | 2823 | Condition negative |
| NLR | 0.62582 | 0.33792 | 0.55544 | 0.93696 | 0.94784 | 0.94986 | 1.00035 | Negative likelihood ratio |
| NLRI | Negligible | Poor | Negligible | Negligible | Negligible | Negligible | Negligible | Negative likelihood ratio interpretation |
| NPV | 0.86436 | 0.83704 | 0.87658 | 0.92967 | 0.9153 | 0.95335 | 0.98088 | Negative predictive value |
| OC | 0.48059 | 0.7424 | 0.59794 | 0.20879 | 0.66667 | 0.2963 | 0.0 | Overlap coefficient |
| OOC | 0.46536 | 0.6908 | 0.46032 | 0.13584 | 0.19094 | 0.12966 | 0.0 | Otsuka-Ochiai coefficient |
| OP | 0.47059 | 0.7418 | 0.60313 | 0.07341 | 0.01745 | 0.05528 | -0.01946 | Optimized precision |
| P | 577 | 1052 | 582 | 215 | 256 | 141 | 55 | Condition positive or support |
| PLR | 3.68984 | 3.12353 | 2.1654 | 3.26854 | 20.48437 | 8.1732 | 0.0 | Positive likelihood ratio |
| PLRI | Poor | Poor | Poor | Poor | Good | Fair | Negligible | Positive likelihood ratio interpretation |
| POP | 2878 | 2878 | 2878 | 2878 | 2878 | 2878 | 2878 | Population |
| PPV | 0.48059 | 0.6428 | 0.35438 | 0.20879 | 0.66667 | 0.2963 | 0.0 | Precision or positive predictive value |
| PRE | 0.20049 | 0.36553 | 0.20222 | 0.0747 | 0.08895 | 0.04899 | 0.01911 | Prevalence |
| Q | 0.70998 | 0.80475 | 0.59172 | 0.55441 | 0.91155 | 0.79177 | -1.0 | Yule Q - coefficient of colligation |
| QI | Moderate | Strong | Moderate | Moderate | Strong | Strong | Negligible | Yule Q interpretation |
| RACC | 0.03769 | 0.15432 | 0.069 | 0.00236 | 0.00065 | 0.00046 | 1e-05 | Random accuracy |
| RACCU | 0.03773 | 0.15512 | 0.07383 | 0.00283 | 0.00232 | 0.00085 | 9e-05 | Random accuracy unbiased |
| TN | 2020 | 1392 | 1662 | 2591 | 2615 | 2718 | 2822 | True negative/correct rejection |
| TNR | 0.87788 | 0.76232 | 0.72387 | 0.97296 | 0.99733 | 0.99306 | 0.99965 | Specificity or true negative rate |
| TON | 2337 | 1663 | 1896 | 2787 | 2857 | 2851 | 2877 | Test outcome negative |
| TOP | 541 | 1215 | 982 | 91 | 21 | 27 | 1 | Test outcome positive |
| TP | 260 | 781 | 348 | 19 | 14 | 8 | 0 | True positive/hit |
| TPR | 0.45061 | 0.7424 | 0.59794 | 0.08837 | 0.05469 | 0.05674 | 0.0 | Sensitivity, recall, hit rate, or true positive rate |
| Y | 0.32849 | 0.50472 | 0.32181 | 0.06133 | 0.05202 | 0.0498 | -0.00035 | Youden index |
| dInd | 0.5628 | 0.3505 | 0.48775 | 0.91203 | 0.94532 | 0.94329 | 1.0 | Distance index |
| sInd | 0.60204 | 0.75216 | 0.65511 | 0.3551 | 0.33156 | 0.33299 | 0.29289 | Similarity index |

Generated By PyCM Version 3.1
