# Supplementary material for: TopEC: prediction of Enzyme Commission classes by 3D graph neural networks and localized 3D protein descriptor
Source: Nat Commun. 2025 Mar 20;16:2737. doi: 10.1038/s41467-025-57324-5 (PMC11923149; doi:10.1038/s41467-025-57324-5)
Supplement: Supplementary file 3 — Supplementary Data 1 [file 41467_2025_57324_MOESM3_ESM.zip › Data_S1/table1/mainclass/EnzyNet/local/BindingMOAD_TEMP_wflips.html]

PDB\_TEMP\_enzynet\_wflips\_sites


# PyCM Report

## Dataset Type :

- Multi-Class Classification
- Imbalanced

Note 1 : Recommended statistics for this type of classification highlighted in aqua

Note 2 : The recommender system assumes that the input is the result of classification over the whole data rather than just a part of it.
If the confusion matrix is the result of test data classification, the recommendation is not valid.

## Confusion Matrix :

|  |  |  |  |  |  |  |  |  |  |  |  |  |  |  |  |  |  |  |  |  |  |  |  |  |  |  |  |  |  |  |  |  |  |  |  |  |  |  |  |  |  |  |  |  |  |  |  |  |  |  |  |  |  |  |  |  |  |  |  |  |  |  |  |  |  |
| --- | --- | --- | --- | --- | --- | --- | --- | --- | --- | --- | --- | --- | --- | --- | --- | --- | --- | --- | --- | --- | --- | --- | --- | --- | --- | --- | --- | --- | --- | --- | --- | --- | --- | --- | --- | --- | --- | --- | --- | --- | --- | --- | --- | --- | --- | --- | --- | --- | --- | --- | --- | --- | --- | --- | --- | --- | --- | --- | --- | --- | --- | --- | --- | --- | --- |
| Actual | Predict  |  |  |  |  |  |  |  |  | | --- | --- | --- | --- | --- | --- | --- | --- | |  | 0 | 1 | 2 | 3 | 4 | 5 | 6 | | 0 | 288 | 55 | 76 | 3 | 0 | 1 | 0 | | 1 | 86 | 521 | 142 | 1 | 0 | 0 | 4 | | 2 | 57 | 110 | 487 | 0 | 1 | 0 | 15 | | 3 | 26 | 20 | 26 | 53 | 0 | 0 | 0 | | 4 | 23 | 13 | 23 | 0 | 18 | 0 | 0 | | 5 | 8 | 24 | 12 | 0 | 0 | 14 | 0 | | 6 | 16 | 31 | 28 | 0 | 0 | 0 | 1 | |

## Overall Statistics :

|  |  |
| --- | --- |
| 95% CI | (0.61286,0.65329) |
| ACC Macro | 0.89516 |
| ARI | 0.27581 |
| AUNP | 0.74271 |
| AUNU | 0.67832 |
| Bangdiwala B | 0.44403 |
| Bennett S | 0.57192 |
| CBA | 0.39574 |
| CSI | 0.1042 |
| Chi-Squared | 2998.62677 |
| Chi-Squared DF | 36 |
| Conditional Entropy | 1.32558 |
| Cramer V | 0.47847 |
| Cross Entropy | 2.34345 |
| F1 Macro | 0.47577 |
| F1 Micro | 0.63307 |
| FNR Macro | 0.56986 |
| FNR Micro | 0.36693 |
| FPR Macro | 0.07351 |
| FPR Micro | 0.06115 |
| Gwet AC1 | 0.58327 |
| Hamming Loss | 0.36693 |
| Joint Entropy | 3.55127 |
| KL Divergence | 0.11776 |
| Kappa | 0.48958 |
| Kappa 95% CI | (0.46145,0.5177) |
| Kappa No Prevalence | 0.26615 |
| Kappa Standard Error | 0.01435 |
| Kappa Unbiased | 0.48826 |
| Krippendorff Alpha | 0.48837 |
| Lambda A | 0.44927 |
| Lambda B | 0.45572 |
| Mutual Information | 0.53206 |
| NIR | 0.3454 |
| Overall ACC | 0.63307 |
| Overall CEN | 0.4065 |
| Overall J | (2.35598,0.33657) |
| Overall MCC | 0.49247 |
| Overall MCEN | 0.51646 |
| Overall RACC | 0.28114 |
| Overall RACCU | 0.28299 |
| P-Value | None |
| PPV Macro | 0.67406 |
| PPV Micro | 0.63307 |
| Pearson C | 0.76073 |
| Phi-Squared | 1.37363 |
| RCI | 0.23905 |
| RR | 311.85714 |
| Reference Entropy | 2.22569 |
| Response Entropy | 1.85764 |
| SOA1(Landis & Koch) | Moderate |
| SOA2(Fleiss) | Intermediate to Good |
| SOA3(Altman) | Moderate |
| SOA4(Cicchetti) | Fair |
| SOA5(Cramer) | Relatively Strong |
| SOA6(Matthews) | Weak |
| Scott PI | 0.48826 |
| Standard Error | 0.01032 |
| TNR Macro | 0.92649 |
| TNR Micro | 0.93885 |
| TPR Macro | 0.43014 |
| TPR Micro | 0.63307 |
| Zero-one Loss | 801 |

## Class Statistics :

|  |  |  |  |  |  |  |  |  |
| --- | --- | --- | --- | --- | --- | --- | --- | --- |
| Class | 0 | 1 | 2 | 3 | 4 | 5 | 6 | Description |
| ACC | 0.83921 | 0.77737 | 0.77554 | 0.96519 | 0.97251 | 0.97939 | 0.95694 | Accuracy |
| AGF | 0.77282 | 0.75634 | 0.77324 | 0.68015 | 0.51881 | 0.52797 | 0.12237 | Adjusted F-score |
| AGM | 0.81946 | 0.78133 | 0.77587 | 0.81917 | 0.73682 | 0.74194 | 0.54482 | Adjusted geometric mean |
| AM | 81 | 20 | 124 | -68 | -58 | -43 | -56 | Difference between automatic and manual classification |
| AUC | 0.77906 | 0.75697 | 0.76198 | 0.71103 | 0.61665 | 0.62045 | 0.50207 | Area under the ROC curve |
| AUCI | Good | Good | Good | Good | Fair | Fair | Poor | AUC value interpretation |
| AUPR | 0.62614 | 0.68205 | 0.67011 | 0.67691 | 0.59057 | 0.58736 | 0.03158 | Area under the PR curve |
| BCD | 0.01855 | 0.00458 | 0.0284 | 0.01557 | 0.01328 | 0.00985 | 0.01283 | Bray-Curtis dissimilarity |
| BM | 0.55812 | 0.51393 | 0.52396 | 0.42206 | 0.23329 | 0.24091 | 0.00414 | Informedness or bookmaker informedness |
| CEN | 0.44012 | 0.38384 | 0.40425 | 0.36014 | 0.40362 | 0.38778 | 0.5817 | Confusion entropy |
| DOR | 15.24938 | 10.39366 | 10.4541 | 377.99306 | 642.20339 | 675.81818 | 1.46526 | Diagnostic odds ratio |
| DP | 0.65236 | 0.56057 | 0.56196 | 1.42104 | 1.54795 | 1.56017 | 0.09147 | Discriminant power |
| DPI | Poor | Poor | Poor | Limited | Limited | Limited | Poor | Discriminant power interpretation |
| ERR | 0.16079 | 0.22263 | 0.22446 | 0.03481 | 0.02749 | 0.02061 | 0.04306 | Error rate |
| F0.5 | 0.59041 | 0.67662 | 0.63313 | 0.75071 | 0.58824 | 0.59322 | 0.03205 | F0.5 score |
| F1 | 0.62136 | 0.68194 | 0.6653 | 0.58242 | 0.375 | 0.38356 | 0.02083 | F1 score - harmonic mean of precision and sensitivity |
| F2 | 0.65574 | 0.68734 | 0.70092 | 0.47576 | 0.27523 | 0.2834 | 0.01543 | F2 score |
| FDR | 0.42857 | 0.32687 | 0.38665 | 0.07018 | 0.05263 | 0.06667 | 0.95 | False discovery rate |
| FN | 135 | 233 | 183 | 72 | 59 | 44 | 75 | False negative/miss/type 2 error |
| FNR | 0.31915 | 0.30902 | 0.27313 | 0.576 | 0.76623 | 0.75862 | 0.98684 | Miss rate or false negative rate |
| FOR | 0.08041 | 0.16537 | 0.13175 | 0.03387 | 0.02726 | 0.0203 | 0.03467 | False omission rate |
| FP | 216 | 253 | 307 | 4 | 1 | 1 | 19 | False positive/type 1 error/false alarm |
| FPR | 0.12273 | 0.17705 | 0.20291 | 0.00194 | 0.00047 | 0.00047 | 0.00902 | Fall-out or false positive rate |
| G | 0.62374 | 0.682 | 0.6677 | 0.62789 | 0.4706 | 0.47464 | 0.02565 | G-measure geometric mean of precision and sensitivity |
| GI | 0.55812 | 0.51393 | 0.52396 | 0.42206 | 0.23329 | 0.24091 | 0.00414 | Gini index |
| GM | 0.77285 | 0.75409 | 0.76117 | 0.65052 | 0.48338 | 0.49119 | 0.11419 | G-mean geometric mean of specificity and sensitivity |
| IBA | 0.47997 | 0.4936 | 0.53869 | 0.18025 | 0.05473 | 0.05835 | 0.00029 | Index of balanced accuracy |
| ICSI | 0.25228 | 0.36411 | 0.34022 | 0.35382 | 0.18113 | 0.17471 | -0.93684 | Individual classification success index |
| IS | 1.56023 | 0.96263 | 0.99886 | 4.02134 | 4.74731 | 5.13458 | 0.52224 | Information score |
| J | 0.4507 | 0.51738 | 0.49846 | 0.41085 | 0.23077 | 0.23729 | 0.01053 | Jaccard index |
| LS | 2.949 | 1.94885 | 1.99842 | 16.23846 | 26.85851 | 35.12874 | 1.43618 | Lift score |
| MCC | 0.5235 | 0.51084 | 0.50233 | 0.61493 | 0.46331 | 0.469 | 0.00797 | Matthews correlation coefficient |
| MCCI | Moderate | Moderate | Moderate | Moderate | Weak | Weak | Negligible | Matthews correlation coefficient interpretation |
| MCEN | 0.55625 | 0.50145 | 0.52413 | 0.4265 | 0.43249 | 0.41444 | 0.58366 | Modified confusion entropy |
| MK | 0.49102 | 0.50776 | 0.4816 | 0.89596 | 0.9201 | 0.91304 | 0.01533 | Markedness |
| N | 1760 | 1429 | 1513 | 2058 | 2106 | 2125 | 2107 | Condition negative |
| NLR | 0.3638 | 0.3755 | 0.34266 | 0.57712 | 0.7666 | 0.75898 | 0.99582 | Negative likelihood ratio |
| NLRI | Poor | Poor | Poor | Negligible | Negligible | Negligible | Negligible | Negative likelihood ratio interpretation |
| NPV | 0.91959 | 0.83463 | 0.86825 | 0.96613 | 0.97274 | 0.9797 | 0.96533 | Negative predictive value |
| OC | 0.68085 | 0.69098 | 0.72687 | 0.92982 | 0.94737 | 0.93333 | 0.05 | Overlap coefficient |
| OOC | 0.62374 | 0.682 | 0.6677 | 0.62789 | 0.4706 | 0.47464 | 0.02565 | Otsuka-Ochiai coefficient |
| OP | 0.71315 | 0.6902 | 0.72946 | 0.56151 | 0.35161 | 0.36842 | -0.01685 | Optimized precision |
| P | 423 | 754 | 670 | 125 | 77 | 58 | 76 | Condition positive or support |
| PLR | 5.54768 | 3.90282 | 3.58224 | 218.148 | 492.31169 | 512.93103 | 1.45914 | Positive likelihood ratio |
| PLRI | Fair | Poor | Poor | Good | Good | Good | Poor | Positive likelihood ratio interpretation |
| POP | 2183 | 2183 | 2183 | 2183 | 2183 | 2183 | 2183 | Population |
| PPV | 0.57143 | 0.67313 | 0.61335 | 0.92982 | 0.94737 | 0.93333 | 0.05 | Precision or positive predictive value |
| PRE | 0.19377 | 0.3454 | 0.30692 | 0.05726 | 0.03527 | 0.02657 | 0.03481 | Prevalence |
| Q | 0.87692 | 0.82446 | 0.82539 | 0.99472 | 0.99689 | 0.99704 | 0.18873 | Yule Q - coefficient of colligation |
| QI | Strong | Strong | Strong | Strong | Strong | Strong | Negligible | Yule Q interpretation |
| RACC | 0.04474 | 0.12246 | 0.11163 | 0.0015 | 0.00031 | 0.00018 | 0.00032 | Random accuracy |
| RACCU | 0.04508 | 0.12248 | 0.11244 | 0.00174 | 0.00048 | 0.00028 | 0.00048 | Random accuracy unbiased |
| TN | 1544 | 1176 | 1206 | 2054 | 2105 | 2124 | 2088 | True negative/correct rejection |
| TNR | 0.87727 | 0.82295 | 0.79709 | 0.99806 | 0.99953 | 0.99953 | 0.99098 | Specificity or true negative rate |
| TON | 1679 | 1409 | 1389 | 2126 | 2164 | 2168 | 2163 | Test outcome negative |
| TOP | 504 | 774 | 794 | 57 | 19 | 15 | 20 | Test outcome positive |
| TP | 288 | 521 | 487 | 53 | 18 | 14 | 1 | True positive/hit |
| TPR | 0.68085 | 0.69098 | 0.72687 | 0.424 | 0.23377 | 0.24138 | 0.01316 | Sensitivity, recall, hit rate, or true positive rate |
| Y | 0.55812 | 0.51393 | 0.52396 | 0.42206 | 0.23329 | 0.24091 | 0.00414 | Youden index |
| dInd | 0.34193 | 0.35614 | 0.34026 | 0.576 | 0.76623 | 0.75862 | 0.98688 | Distance index |
| sInd | 0.75822 | 0.74817 | 0.7594 | 0.5927 | 0.45819 | 0.46357 | 0.30217 | Similarity index |

Generated By PyCM Version 3.1
