# Supplementary material for: TopEC: prediction of Enzyme Commission classes by 3D graph neural networks and localized 3D protein descriptor
Source: Nat Commun. 2025 Mar 20;16:2737. doi: 10.1038/s41467-025-57324-5 (PMC11923149; doi:10.1038/s41467-025-57324-5)
Supplement: Supplementary file 3 — Supplementary Data 1 [file 41467_2025_57324_MOESM3_ESM.zip › Data_S1/table1/mainclass/EnzyNet/local/BindingMOAD_TEMP_flips.html]

PDB\_TEMP\_enzynet\_flips\_sites


# PyCM Report

## Dataset Type :

- Multi-Class Classification
- Imbalanced

Note 1 : Recommended statistics for this type of classification highlighted in aqua

Note 2 : The recommender system assumes that the input is the result of classification over the whole data rather than just a part of it.
If the confusion matrix is the result of test data classification, the recommendation is not valid.

## Confusion Matrix :

|  |  |  |  |  |  |  |  |  |  |  |  |  |  |  |  |  |  |  |  |  |  |  |  |  |  |  |  |  |  |  |  |  |  |  |  |  |  |  |  |  |  |  |  |  |  |  |  |  |  |  |  |  |  |  |  |  |  |  |  |  |  |  |  |  |  |
| --- | --- | --- | --- | --- | --- | --- | --- | --- | --- | --- | --- | --- | --- | --- | --- | --- | --- | --- | --- | --- | --- | --- | --- | --- | --- | --- | --- | --- | --- | --- | --- | --- | --- | --- | --- | --- | --- | --- | --- | --- | --- | --- | --- | --- | --- | --- | --- | --- | --- | --- | --- | --- | --- | --- | --- | --- | --- | --- | --- | --- | --- | --- | --- | --- | --- |
| Actual | Predict  |  |  |  |  |  |  |  |  | | --- | --- | --- | --- | --- | --- | --- | --- | |  | 0 | 1 | 2 | 3 | 4 | 5 | 6 | | 0 | 285 | 62 | 71 | 4 | 0 | 1 | 0 | | 1 | 92 | 519 | 139 | 1 | 0 | 0 | 3 | | 2 | 60 | 104 | 490 | 0 | 1 | 0 | 15 | | 3 | 25 | 20 | 29 | 51 | 0 | 0 | 0 | | 4 | 24 | 13 | 26 | 0 | 14 | 0 | 0 | | 5 | 8 | 32 | 12 | 0 | 0 | 6 | 0 | | 6 | 20 | 28 | 28 | 0 | 0 | 0 | 0 | |

## Overall Statistics :

|  |  |
| --- | --- |
| 95% CI | (0.60498,0.64559) |
| ACC Macro | 0.89294 |
| ARI | 0.26872 |
| AUNP | 0.73757 |
| AUNU | 0.66156 |
| Bangdiwala B | 0.44075 |
| Bennett S | 0.56283 |
| CBA | 0.3616 |
| CSI | 0.04654 |
| Chi-Squared | 2534.52283 |
| Chi-Squared DF | 36 |
| Conditional Entropy | 1.32316 |
| Cramer V | 0.43989 |
| Cross Entropy | 2.38283 |
| F1 Macro | 0.42962 |
| F1 Micro | 0.62529 |
| FNR Macro | 0.6019 |
| FNR Micro | 0.37471 |
| FPR Macro | 0.07498 |
| FPR Micro | 0.06245 |
| Gwet AC1 | 0.57456 |
| Hamming Loss | 0.37471 |
| Joint Entropy | 3.54885 |
| KL Divergence | 0.15714 |
| Kappa | 0.4777 |
| Kappa 95% CI | (0.44939,0.506) |
| Kappa No Prevalence | 0.25057 |
| Kappa Standard Error | 0.01444 |
| Kappa Unbiased | 0.4762 |
| Krippendorff Alpha | 0.47632 |
| Lambda A | 0.43807 |
| Lambda B | 0.45821 |
| Mutual Information | 0.49788 |
| NIR | 0.3454 |
| Overall ACC | 0.62529 |
| Overall CEN | 0.41189 |
| Overall J | (2.12551,0.30364) |
| Overall MCC | 0.48084 |
| Overall MCEN | 0.5219 |
| Overall RACC | 0.28258 |
| Overall RACCU | 0.28462 |
| P-Value | None |
| PPV Macro | 0.64844 |
| PPV Micro | 0.62529 |
| Pearson C | 0.73298 |
| Phi-Squared | 1.16103 |
| RCI | 0.2237 |
| RR | 311.85714 |
| Reference Entropy | 2.22569 |
| Response Entropy | 1.82104 |
| SOA1(Landis & Koch) | Moderate |
| SOA2(Fleiss) | Intermediate to Good |
| SOA3(Altman) | Moderate |
| SOA4(Cicchetti) | Fair |
| SOA5(Cramer) | Relatively Strong |
| SOA6(Matthews) | Weak |
| Scott PI | 0.4762 |
| Standard Error | 0.01036 |
| TNR Macro | 0.92502 |
| TNR Micro | 0.93755 |
| TPR Macro | 0.3981 |
| TPR Micro | 0.62529 |
| Zero-one Loss | 818 |

## Class Statistics :

|  |  |  |  |  |  |  |  |  |
| --- | --- | --- | --- | --- | --- | --- | --- | --- |
| Class | 0 | 1 | 2 | 3 | 4 | 5 | 6 | Description |
| ACC | 0.83188 | 0.77371 | 0.77783 | 0.96381 | 0.97068 | 0.97572 | 0.95694 | Accuracy |
| AGF | 0.76561 | 0.75341 | 0.77637 | 0.66751 | 0.46003 | 0.35086 | 0.0 | Adjusted F-score |
| AGM | 0.81213 | 0.77763 | 0.77817 | 0.81247 | 0.70777 | 0.65598 | 0 | Adjusted geometric mean |
| AM | 91 | 24 | 125 | -69 | -62 | -51 | -58 | Difference between automatic and manual classification |
| AUC | 0.77182 | 0.75354 | 0.76488 | 0.70279 | 0.59067 | 0.55149 | 0.49573 | Area under the ROC curve |
| AUCI | Good | Good | Good | Good | Poor | Poor | Poor | AUC value interpretation |
| AUPR | 0.61412 | 0.67771 | 0.67385 | 0.65936 | 0.55758 | 0.4803 | 0.0 | Area under the PR curve |
| BCD | 0.02084 | 0.0055 | 0.02863 | 0.0158 | 0.0142 | 0.01168 | 0.01328 | Bray-Curtis dissimilarity |
| BM | 0.54365 | 0.50708 | 0.52976 | 0.40557 | 0.18134 | 0.10298 | -0.00854 | Informedness or bookmaker informedness |
| CEN | 0.45192 | 0.38919 | 0.40439 | 0.37152 | 0.41584 | 0.39552 | 0.58496 | Confusion entropy |
| DOR | 13.8072 | 9.97667 | 10.78179 | 282.98108 | 467.77778 | 245.07692 | 0.0 | Diagnostic odds ratio |
| DP | 0.62857 | 0.55077 | 0.56935 | 1.35172 | 1.47207 | 1.31729 | None | Discriminant power |
| DPI | Poor | Poor | Poor | Limited | Limited | Limited | None | Discriminant power interpretation |
| ERR | 0.16812 | 0.22629 | 0.22217 | 0.03619 | 0.02932 | 0.02428 | 0.04306 | Error rate |
| F0.5 | 0.57483 | 0.67124 | 0.63636 | 0.73066 | 0.51095 | 0.34884 | 0.0 | F0.5 score |
| F1 | 0.60832 | 0.67755 | 0.66894 | 0.56354 | 0.30435 | 0.18462 | 0.0 | F1 score - harmonic mean of precision and sensitivity |
| F2 | 0.64597 | 0.68397 | 0.70504 | 0.45863 | 0.21672 | 0.12552 | 0.0 | F2 score |
| FDR | 0.44553 | 0.3329 | 0.38365 | 0.08929 | 0.06667 | 0.14286 | 1.0 | False discovery rate |
| FN | 138 | 235 | 180 | 74 | 63 | 52 | 76 | False negative/miss/type 2 error |
| FNR | 0.32624 | 0.31167 | 0.26866 | 0.592 | 0.81818 | 0.89655 | 1.0 | Miss rate or false negative rate |
| FOR | 0.08268 | 0.16726 | 0.12968 | 0.03479 | 0.02906 | 0.0239 | 0.0351 | False omission rate |
| FP | 229 | 259 | 305 | 5 | 1 | 1 | 18 | False positive/type 1 error/false alarm |
| FPR | 0.13011 | 0.18125 | 0.20159 | 0.00243 | 0.00047 | 0.00047 | 0.00854 | Fall-out or false positive rate |
| G | 0.61121 | 0.67763 | 0.67139 | 0.60957 | 0.41194 | 0.29778 | 0.0 | G-measure geometric mean of precision and sensitivity |
| GI | 0.54365 | 0.50708 | 0.52976 | 0.40557 | 0.18134 | 0.10298 | -0.00854 | Gini index |
| GM | 0.76557 | 0.75071 | 0.76414 | 0.63797 | 0.4263 | 0.32156 | 0.0 | G-mean geometric mean of specificity and sensitivity |
| IBA | 0.47114 | 0.49007 | 0.54475 | 0.16705 | 0.03313 | 0.01075 | 0.0 | Index of balanced accuracy |
| ICSI | 0.22823 | 0.35542 | 0.3477 | 0.31871 | 0.11515 | -0.03941 | -1.0 | Individual classification success index |
| IS | 1.51678 | 0.94964 | 1.00591 | 3.99138 | 4.72577 | 5.01172 | None | Information score |
| J | 0.43712 | 0.51234 | 0.50256 | 0.39231 | 0.17949 | 0.10169 | 0.0 | Jaccard index |
| LS | 2.86151 | 1.93139 | 2.0082 | 15.90471 | 26.46061 | 32.26108 | 0.0 | Lift score |
| MCC | 0.50645 | 0.50345 | 0.50776 | 0.59603 | 0.40495 | 0.29293 | -0.01732 | Matthews correlation coefficient |
| MCCI | Moderate | Moderate | Moderate | Moderate | Weak | Negligible | Negligible | Matthews correlation coefficient interpretation |
| MCEN | 0.56731 | 0.50741 | 0.52611 | 0.43633 | 0.43597 | 0.40073 | 0.58496 | Modified confusion entropy |
| MK | 0.47179 | 0.49984 | 0.48667 | 0.87592 | 0.90427 | 0.83325 | -0.0351 | Markedness |
| N | 1760 | 1429 | 1513 | 2058 | 2106 | 2125 | 2107 | Condition negative |
| NLR | 0.37504 | 0.38066 | 0.33649 | 0.59344 | 0.81857 | 0.89697 | 1.00862 | Negative likelihood ratio |
| NLRI | Poor | Poor | Poor | Negligible | Negligible | Negligible | Negligible | Negative likelihood ratio interpretation |
| NPV | 0.91732 | 0.83274 | 0.87032 | 0.96521 | 0.97094 | 0.9761 | 0.9649 | Negative predictive value |
| OC | 0.67376 | 0.68833 | 0.73134 | 0.91071 | 0.93333 | 0.85714 | 0.0 | Overlap coefficient |
| OOC | 0.61121 | 0.67763 | 0.67139 | 0.60957 | 0.41194 | 0.29778 | 0.0 | Otsuka-Ochiai coefficient |
| OP | 0.70483 | 0.68716 | 0.73398 | 0.54436 | 0.2785 | 0.1633 | -0.04306 | Optimized precision |
| P | 423 | 754 | 670 | 125 | 77 | 58 | 76 | Condition positive or support |
| PLR | 5.17823 | 3.79777 | 3.62794 | 167.9328 | 382.90909 | 219.82759 | 0.0 | Positive likelihood ratio |
| PLRI | Fair | Poor | Poor | Good | Good | Good | Negligible | Positive likelihood ratio interpretation |
| POP | 2183 | 2183 | 2183 | 2183 | 2183 | 2183 | 2183 | Population |
| PPV | 0.55447 | 0.6671 | 0.61635 | 0.91071 | 0.93333 | 0.85714 | 0.0 | Precision or positive predictive value |
| PRE | 0.19377 | 0.3454 | 0.30692 | 0.05726 | 0.03527 | 0.02657 | 0.03481 | Prevalence |
| Q | 0.86493 | 0.8178 | 0.83025 | 0.99296 | 0.99573 | 0.99187 | -1.0 | Yule Q - coefficient of colligation |
| QI | Strong | Strong | Strong | Strong | Strong | Strong | Negligible | Yule Q interpretation |
| RACC | 0.04562 | 0.1231 | 0.11177 | 0.00147 | 0.00024 | 9e-05 | 0.00029 | Random accuracy |
| RACCU | 0.04606 | 0.12313 | 0.11259 | 0.00172 | 0.00044 | 0.00022 | 0.00046 | Random accuracy unbiased |
| TN | 1531 | 1170 | 1208 | 2053 | 2105 | 2124 | 2089 | True negative/correct rejection |
| TNR | 0.86989 | 0.81875 | 0.79841 | 0.99757 | 0.99953 | 0.99953 | 0.99146 | Specificity or true negative rate |
| TON | 1669 | 1405 | 1388 | 2127 | 2168 | 2176 | 2165 | Test outcome negative |
| TOP | 514 | 778 | 795 | 56 | 15 | 7 | 18 | Test outcome positive |
| TP | 285 | 519 | 490 | 51 | 14 | 6 | 0 | True positive/hit |
| TPR | 0.67376 | 0.68833 | 0.73134 | 0.408 | 0.18182 | 0.10345 | 0.0 | Sensitivity, recall, hit rate, or true positive rate |
| Y | 0.54365 | 0.50708 | 0.52976 | 0.40557 | 0.18134 | 0.10298 | -0.00854 | Youden index |
| dInd | 0.35123 | 0.36054 | 0.33588 | 0.592 | 0.81818 | 0.89655 | 1.00004 | Distance index |
| sInd | 0.75164 | 0.74506 | 0.7625 | 0.58139 | 0.42146 | 0.36604 | 0.29287 | Similarity index |

Generated By PyCM Version 3.1
