# Supplementary material for: TopEC: prediction of Enzyme Commission classes by 3D graph neural networks and localized 3D protein descriptor
Source: Nat Commun. 2025 Mar 20;16:2737. doi: 10.1038/s41467-025-57324-5 (PMC11923149; doi:10.1038/s41467-025-57324-5)
Supplement: Supplementary file 3 — Supplementary Data 1 [file 41467_2025_57324_MOESM3_ESM.zip › Data_S1/table1/mainclass/EnzyNet/local/BindingMOAD_TEMP.html]

PDB\_TEMP\_enzynet\_none\_sites


# PyCM Report

## Dataset Type :

- Multi-Class Classification
- Imbalanced

Note 1 : Recommended statistics for this type of classification highlighted in aqua

Note 2 : The recommender system assumes that the input is the result of classification over the whole data rather than just a part of it.
If the confusion matrix is the result of test data classification, the recommendation is not valid.

## Confusion Matrix :

|  |  |  |  |  |  |  |  |  |  |  |  |  |  |  |  |  |  |  |  |  |  |  |  |  |  |  |  |  |  |  |  |  |  |  |  |  |  |  |  |  |  |  |  |  |  |  |  |  |  |  |  |  |  |  |  |  |  |  |  |  |  |  |  |  |  |
| --- | --- | --- | --- | --- | --- | --- | --- | --- | --- | --- | --- | --- | --- | --- | --- | --- | --- | --- | --- | --- | --- | --- | --- | --- | --- | --- | --- | --- | --- | --- | --- | --- | --- | --- | --- | --- | --- | --- | --- | --- | --- | --- | --- | --- | --- | --- | --- | --- | --- | --- | --- | --- | --- | --- | --- | --- | --- | --- | --- | --- | --- | --- | --- | --- | --- |
| Actual | Predict  |  |  |  |  |  |  |  |  | | --- | --- | --- | --- | --- | --- | --- | --- | |  | 0 | 1 | 2 | 3 | 4 | 5 | 6 | | 0 | 264 | 68 | 78 | 10 | 1 | 1 | 1 | | 1 | 88 | 503 | 141 | 10 | 5 | 3 | 4 | | 2 | 65 | 123 | 463 | 2 | 1 | 1 | 15 | | 3 | 23 | 23 | 16 | 62 | 1 | 0 | 0 | | 4 | 17 | 13 | 22 | 0 | 24 | 1 | 0 | | 5 | 5 | 23 | 12 | 1 | 0 | 17 | 0 | | 6 | 20 | 25 | 27 | 1 | 0 | 0 | 3 | |

## Overall Statistics :

|  |  |
| --- | --- |
| 95% CI | (0.59156,0.63244) |
| ACC Macro | 0.88914 |
| ARI | 0.24761 |
| AUNP | 0.73014 |
| AUNU | 0.68449 |
| Bangdiwala B | 0.41191 |
| Bennett S | 0.54734 |
| CBA | 0.42065 |
| CSI | 0.03818 |
| Chi-Squared | 2723.95867 |
| Chi-Squared DF | 36 |
| Conditional Entropy | 1.47081 |
| Cramer V | 0.45603 |
| Cross Entropy | 2.28941 |
| F1 Macro | 0.48521 |
| F1 Micro | 0.612 |
| FNR Macro | 0.55392 |
| FNR Micro | 0.388 |
| FPR Macro | 0.0771 |
| FPR Micro | 0.06467 |
| Gwet AC1 | 0.55885 |
| Hamming Loss | 0.388 |
| Joint Entropy | 3.69651 |
| KL Divergence | 0.06372 |
| Kappa | 0.46408 |
| Kappa 95% CI | (0.43585,0.49232) |
| Kappa No Prevalence | 0.224 |
| Kappa Standard Error | 0.01441 |
| Kappa Unbiased | 0.46332 |
| Krippendorff Alpha | 0.46345 |
| Lambda A | 0.41568 |
| Lambda B | 0.41851 |
| Mutual Information | 0.48227 |
| NIR | 0.3454 |
| Overall ACC | 0.612 |
| Overall CEN | 0.43653 |
| Overall J | (2.37531,0.33933) |
| Overall MCC | 0.46559 |
| Overall MCEN | 0.54988 |
| Overall RACC | 0.27601 |
| Overall RACCU | 0.27704 |
| P-Value | None |
| PPV Macro | 0.59211 |
| PPV Micro | 0.612 |
| Pearson C | 0.74506 |
| Phi-Squared | 1.24781 |
| RCI | 0.21668 |
| RR | 311.85714 |
| Reference Entropy | 2.22569 |
| Response Entropy | 1.95309 |
| SOA1(Landis & Koch) | Moderate |
| SOA2(Fleiss) | Intermediate to Good |
| SOA3(Altman) | Moderate |
| SOA4(Cicchetti) | Fair |
| SOA5(Cramer) | Relatively Strong |
| SOA6(Matthews) | Weak |
| Scott PI | 0.46332 |
| Standard Error | 0.01043 |
| TNR Macro | 0.9229 |
| TNR Micro | 0.93533 |
| TPR Macro | 0.44608 |
| TPR Micro | 0.612 |
| Zero-one Loss | 847 |

## Class Statistics :

|  |  |  |  |  |  |  |  |  |
| --- | --- | --- | --- | --- | --- | --- | --- | --- |
| Class | 0 | 1 | 2 | 3 | 4 | 5 | 6 | Description |
| ACC | 0.8273 | 0.75905 | 0.76958 | 0.96015 | 0.97206 | 0.97847 | 0.9574 | Accuracy |
| AGF | 0.73934 | 0.73662 | 0.75379 | 0.71766 | 0.58796 | 0.57277 | 0.21104 | Adjusted F-score |
| AGM | 0.80047 | 0.76309 | 0.76963 | 0.84 | 0.77277 | 0.76583 | 0.5871 | Adjusted geometric mean |
| AM | 59 | 24 | 89 | -39 | -45 | -35 | -53 | Difference between automatic and manual classification |
| AUC | 0.75012 | 0.73733 | 0.7477 | 0.74217 | 0.65394 | 0.64514 | 0.51499 | Area under the ROC curve |
| AUCI | Good | Good | Good | Good | Fair | Fair | Poor | AUC value interpretation |
| AUPR | 0.58592 | 0.65682 | 0.65053 | 0.60847 | 0.53084 | 0.51612 | 0.08495 | Area under the PR curve |
| BCD | 0.01351 | 0.0055 | 0.02038 | 0.00893 | 0.01031 | 0.00802 | 0.01214 | Bray-Curtis dissimilarity |
| BM | 0.50025 | 0.47467 | 0.49541 | 0.48434 | 0.30789 | 0.29028 | 0.02998 | Informedness or bookmaker informedness |
| CEN | 0.47279 | 0.41748 | 0.41683 | 0.43787 | 0.47484 | 0.46334 | 0.61708 | Confusion entropy |
| DOR | 11.7445 | 8.40945 | 9.19622 | 83.40476 | 118.75472 | 146.43496 | 4.28836 | Diagnostic odds ratio |
| DP | 0.58983 | 0.50985 | 0.53127 | 1.05921 | 1.14381 | 1.19398 | 0.3486 | Discriminant power |
| DPI | Poor | Poor | Poor | Limited | Limited | Limited | Poor | Discriminant power interpretation |
| ERR | 0.1727 | 0.24095 | 0.23042 | 0.03985 | 0.02794 | 0.02153 | 0.0426 | Error rate |
| F0.5 | 0.56146 | 0.65054 | 0.62466 | 0.66098 | 0.58537 | 0.56667 | 0.08929 | F0.5 score |
| F1 | 0.58343 | 0.65666 | 0.64801 | 0.58768 | 0.44037 | 0.41975 | 0.06061 | F1 score - harmonic mean of precision and sensitivity |
| F2 | 0.60718 | 0.66289 | 0.67316 | 0.52901 | 0.35294 | 0.33333 | 0.04587 | F2 score |
| FDR | 0.45228 | 0.35347 | 0.38999 | 0.27907 | 0.25 | 0.26087 | 0.86957 | False discovery rate |
| FN | 159 | 251 | 207 | 63 | 53 | 41 | 73 | False negative/miss/type 2 error |
| FNR | 0.37589 | 0.33289 | 0.30896 | 0.504 | 0.68831 | 0.7069 | 0.96053 | Miss rate or false negative rate |
| FOR | 0.09347 | 0.17865 | 0.14537 | 0.03004 | 0.02464 | 0.01898 | 0.0338 | False omission rate |
| FP | 218 | 275 | 296 | 24 | 8 | 6 | 20 | False positive/type 1 error/false alarm |
| FPR | 0.12386 | 0.19244 | 0.19564 | 0.01166 | 0.0038 | 0.00282 | 0.00949 | Fall-out or false positive rate |
| G | 0.58467 | 0.65674 | 0.64927 | 0.59798 | 0.48349 | 0.46545 | 0.07175 | G-measure geometric mean of precision and sensitivity |
| GI | 0.50025 | 0.47467 | 0.49541 | 0.48434 | 0.30789 | 0.29028 | 0.02998 | Gini index |
| GM | 0.73947 | 0.73398 | 0.74555 | 0.70015 | 0.55723 | 0.54063 | 0.19773 | G-mean geometric mean of specificity and sensitivity |
| IBA | 0.409 | 0.46306 | 0.49286 | 0.24886 | 0.09796 | 0.08649 | 0.00191 | Index of balanced accuracy |
| ICSI | 0.17183 | 0.31364 | 0.30106 | 0.21693 | 0.06169 | 0.03223 | -0.83009 | Individual classification success index |
| IS | 1.49909 | 0.90446 | 0.99099 | 3.65424 | 4.41027 | 4.79802 | 1.90557 | Information score |
| J | 0.41186 | 0.48882 | 0.4793 | 0.41611 | 0.28235 | 0.26562 | 0.03125 | Jaccard index |
| LS | 2.82664 | 1.87185 | 1.98755 | 12.59033 | 21.26299 | 27.81934 | 3.74657 | Lift score |
| MCC | 0.47669 | 0.47126 | 0.47978 | 0.57847 | 0.47258 | 0.45721 | 0.05383 | Matthews correlation coefficient |
| MCCI | Weak | Weak | Weak | Moderate | Weak | Weak | Negligible | Matthews correlation coefficient interpretation |
| MCEN | 0.58588 | 0.53968 | 0.53457 | 0.53831 | 0.53709 | 0.5168 | 0.62437 | Modified confusion entropy |
| MK | 0.45424 | 0.46788 | 0.46465 | 0.69089 | 0.72536 | 0.72015 | 0.09664 | Markedness |
| N | 1760 | 1429 | 1513 | 2058 | 2106 | 2125 | 2107 | Condition negative |
| NLR | 0.42903 | 0.41222 | 0.3841 | 0.50995 | 0.69094 | 0.7089 | 0.96973 | Negative likelihood ratio |
| NLRI | Poor | Poor | Poor | Negligible | Negligible | Negligible | Negligible | Negative likelihood ratio interpretation |
| NPV | 0.90653 | 0.82135 | 0.85463 | 0.96996 | 0.97536 | 0.98102 | 0.9662 | Negative predictive value |
| OC | 0.62411 | 0.66711 | 0.69104 | 0.72093 | 0.75 | 0.73913 | 0.13043 | Overlap coefficient |
| OOC | 0.58467 | 0.65674 | 0.64927 | 0.59798 | 0.48349 | 0.46545 | 0.07175 | Otsuka-Ochiai coefficient |
| OP | 0.65931 | 0.66381 | 0.69381 | 0.62846 | 0.44868 | 0.4328 | 0.03405 | Optimized precision |
| P | 423 | 754 | 670 | 125 | 77 | 58 | 76 | Condition positive or support |
| PLR | 5.03871 | 3.46654 | 3.53227 | 42.532 | 82.05195 | 103.80747 | 4.15855 | Positive likelihood ratio |
| PLRI | Fair | Poor | Poor | Good | Good | Good | Poor | Positive likelihood ratio interpretation |
| POP | 2183 | 2183 | 2183 | 2183 | 2183 | 2183 | 2183 | Population |
| PPV | 0.54772 | 0.64653 | 0.61001 | 0.72093 | 0.75 | 0.73913 | 0.13043 | Precision or positive predictive value |
| PRE | 0.19377 | 0.3454 | 0.30692 | 0.05726 | 0.03527 | 0.02657 | 0.03481 | Prevalence |
| Q | 0.84307 | 0.78745 | 0.80385 | 0.9763 | 0.9833 | 0.98643 | 0.62181 | Yule Q - coefficient of colligation |
| QI | Strong | Strong | Strong | Strong | Strong | Strong | Moderate | Yule Q interpretation |
| RACC | 0.04278 | 0.1231 | 0.10671 | 0.00226 | 0.00052 | 0.00028 | 0.00037 | Random accuracy |
| RACCU | 0.04297 | 0.12313 | 0.10713 | 0.00234 | 0.00062 | 0.00034 | 0.00051 | Random accuracy unbiased |
| TN | 1542 | 1154 | 1217 | 2034 | 2098 | 2119 | 2087 | True negative/correct rejection |
| TNR | 0.87614 | 0.80756 | 0.80436 | 0.98834 | 0.9962 | 0.99718 | 0.99051 | Specificity or true negative rate |
| TON | 1701 | 1405 | 1424 | 2097 | 2151 | 2160 | 2160 | Test outcome negative |
| TOP | 482 | 778 | 759 | 86 | 32 | 23 | 23 | Test outcome positive |
| TP | 264 | 503 | 463 | 62 | 24 | 17 | 3 | True positive/hit |
| TPR | 0.62411 | 0.66711 | 0.69104 | 0.496 | 0.31169 | 0.2931 | 0.03947 | Sensitivity, recall, hit rate, or true positive rate |
| Y | 0.50025 | 0.47467 | 0.49541 | 0.48434 | 0.30789 | 0.29028 | 0.02998 | Youden index |
| dInd | 0.39577 | 0.38451 | 0.36569 | 0.50413 | 0.68832 | 0.7069 | 0.96057 | Distance index |
| sInd | 0.72015 | 0.72811 | 0.74142 | 0.64352 | 0.51328 | 0.50014 | 0.32077 | Similarity index |

Generated By PyCM Version 3.1
