# Supplementary material for: TopEC: prediction of Enzyme Commission classes by 3D graph neural networks and localized 3D protein descriptor
Source: Nat Commun. 2025 Mar 20;16:2737. doi: 10.1038/s41467-025-57324-5 (PMC11923149; doi:10.1038/s41467-025-57324-5)
Supplement: Supplementary file 3 — Supplementary Data 1 [file 41467_2025_57324_MOESM3_ESM.zip › Data_S1/table1/mainclass/EnzyNet/local/BindingMOAD_FOLD_wflips.html]

PDB\_FOLD\_enzynet\_wflips\_sites


# PyCM Report

## Dataset Type :

- Multi-Class Classification
- Imbalanced

Note 1 : Recommended statistics for this type of classification highlighted in aqua

Note 2 : The recommender system assumes that the input is the result of classification over the whole data rather than just a part of it.
If the confusion matrix is the result of test data classification, the recommendation is not valid.

## Confusion Matrix :

|  |  |  |  |  |  |  |  |  |  |  |  |  |  |  |  |  |  |  |  |  |  |  |  |  |  |  |  |  |  |  |  |  |  |  |  |  |  |  |  |  |  |  |  |  |  |  |  |  |  |  |  |  |  |  |  |  |  |  |  |  |  |  |  |  |  |
| --- | --- | --- | --- | --- | --- | --- | --- | --- | --- | --- | --- | --- | --- | --- | --- | --- | --- | --- | --- | --- | --- | --- | --- | --- | --- | --- | --- | --- | --- | --- | --- | --- | --- | --- | --- | --- | --- | --- | --- | --- | --- | --- | --- | --- | --- | --- | --- | --- | --- | --- | --- | --- | --- | --- | --- | --- | --- | --- | --- | --- | --- | --- | --- | --- | --- |
| Actual | Predict  |  |  |  |  |  |  |  |  | | --- | --- | --- | --- | --- | --- | --- | --- | |  | 0 | 1 | 2 | 3 | 4 | 5 | 6 | | 0 | 200 | 82 | 122 | 2 | 1 | 0 | 0 | | 1 | 22 | 641 | 143 | 1 | 0 | 0 | 0 | | 2 | 51 | 109 | 236 | 7 | 0 | 0 | 0 | | 3 | 27 | 61 | 43 | 1 | 0 | 0 | 0 | | 4 | 36 | 61 | 71 | 1 | 2 | 0 | 0 | | 5 | 5 | 20 | 23 | 0 | 0 | 0 | 0 | | 6 | 2 | 9 | 6 | 0 | 0 | 0 | 0 | |

## Overall Statistics :

|  |  |
| --- | --- |
| 95% CI | (0.52217,0.56599) |
| ACC Macro | 0.86974 |
| ARI | 0.21678 |
| AUNP | 0.67733 |
| AUNU | 0.58895 |
| Bangdiwala B | 0.42409 |
| Bennett S | 0.46809 |
| CBA | 0.21846 |
| CSI | None |
| Chi-Squared | None |
| Chi-Squared DF | 36 |
| Conditional Entropy | 1.25095 |
| Cramer V | None |
| Cross Entropy | 2.55851 |
| F1 Macro | 0.24817 |
| F1 Micro | 0.54408 |
| FNR Macro | 0.72992 |
| FNR Micro | 0.45592 |
| FPR Macro | 0.09219 |
| FPR Micro | 0.07599 |
| Gwet AC1 | 0.48502 |
| Hamming Loss | 0.45592 |
| Joint Entropy | 3.46801 |
| KL Divergence | None |
| Kappa | 0.34573 |
| Kappa 95% CI | (0.31429,0.37718) |
| Kappa No Prevalence | 0.08816 |
| Kappa Standard Error | 0.01604 |
| Kappa Unbiased | 0.33739 |
| Krippendorff Alpha | 0.33755 |
| Lambda A | 0.23684 |
| Lambda B | 0.25749 |
| Mutual Information | 0.2744 |
| NIR | 0.40655 |
| Overall ACC | 0.54408 |
| Overall CEN | 0.43803 |
| Overall J | (1.23113,0.17588) |
| Overall MCC | 0.35605 |
| Overall MCEN | 0.53439 |
| Overall RACC | 0.30316 |
| Overall RACCU | 0.31194 |
| P-Value | None |
| PPV Macro | None |
| PPV Micro | 0.54408 |
| Pearson C | None |
| Phi-Squared | None |
| RCI | 0.12377 |
| RR | 283.57143 |
| Reference Entropy | 2.21706 |
| Response Entropy | 1.52535 |
| SOA1(Landis & Koch) | Fair |
| SOA2(Fleiss) | Poor |
| SOA3(Altman) | Fair |
| SOA4(Cicchetti) | Poor |
| SOA5(Cramer) | None |
| SOA6(Matthews) | Weak |
| Scott PI | 0.33739 |
| Standard Error | 0.01118 |
| TNR Macro | 0.90781 |
| TNR Micro | 0.92401 |
| TPR Macro | 0.27008 |
| TPR Micro | 0.54408 |
| Zero-one Loss | 905 |

## Class Statistics :

|  |  |  |  |  |  |  |  |  |
| --- | --- | --- | --- | --- | --- | --- | --- | --- |
| Class | 0 | 1 | 2 | 3 | 4 | 5 | 6 | Description |
| ACC | 0.82368 | 0.74408 | 0.71033 | 0.92846 | 0.91436 | 0.97582 | 0.99144 | Accuracy |
| AGF | 0.66849 | 0.78324 | 0.66485 | 0.09355 | 0.11638 | 0.0 | 0.0 | Adjusted F-score |
| AGM | 0.77517 | 0.73548 | 0.69598 | 0.52482 | 0.53372 | 0 | 0 | Adjusted geometric mean |
| AM | -64 | 176 | 241 | -120 | -168 | -48 | -17 | Difference between automatic and manual classification |
| AUC | 0.70039 | 0.75199 | 0.66385 | 0.50082 | 0.50557 | 0.5 | 0.5 | Area under the ROC curve |
| AUCI | Good | Good | Fair | Poor | Poor | Poor | Poor | AUC value interpretation |
| AUPR | 0.53725 | 0.72319 | 0.47603 | 0.04545 | 0.33918 | None | None | Area under the PR curve |
| BCD | 0.01612 | 0.04433 | 0.06071 | 0.03023 | 0.04232 | 0.01209 | 0.00428 | Bray-Curtis dissimilarity |
| BM | 0.40078 | 0.50398 | 0.32771 | 0.00164 | 0.01114 | 0.0 | 0.0 | Informedness or bookmaker informedness |
| CEN | 0.46802 | 0.35371 | 0.54958 | 0.52881 | 0.45012 | 0.38348 | 0.38474 | Confusion entropy |
| DOR | 9.69562 | 9.43909 | 4.06634 | 1.27828 | 21.45562 | None | None | Diagnostic odds ratio |
| DP | 0.54393 | 0.53751 | 0.33587 | 0.05879 | 0.73412 | None | None | Discriminant power |
| DPI | Poor | Poor | Poor | Poor | Poor | None | None | Discriminant power interpretation |
| ERR | 0.17632 | 0.25592 | 0.28967 | 0.07154 | 0.08564 | 0.02418 | 0.00856 | Error rate |
| F0.5 | 0.56211 | 0.6763 | 0.39611 | 0.02778 | 0.05464 | 0.0 | 0.0 | F0.5 score |
| F1 | 0.53333 | 0.7162 | 0.45081 | 0.01389 | 0.02299 | 0.0 | 0.0 | F1 score - harmonic mean of precision and sensitivity |
| F2 | 0.50736 | 0.7611 | 0.52305 | 0.00926 | 0.01456 | 0.0 | 0.0 | F2 score |
| FDR | 0.41691 | 0.34791 | 0.63354 | 0.91667 | 0.33333 | None | None | False discovery rate |
| FN | 207 | 166 | 167 | 131 | 169 | 48 | 17 | False negative/miss/type 2 error |
| FNR | 0.5086 | 0.2057 | 0.41439 | 0.99242 | 0.9883 | 1.0 | 1.0 | Miss rate or false negative rate |
| FOR | 0.12607 | 0.16567 | 0.12453 | 0.0664 | 0.08527 | 0.02418 | 0.00856 | False omission rate |
| FP | 143 | 342 | 408 | 11 | 1 | 0 | 0 | False positive/type 1 error/false alarm |
| FPR | 0.09062 | 0.29032 | 0.2579 | 0.00594 | 0.00055 | 0.0 | 0.0 | Fall-out or false positive rate |
| G | 0.53529 | 0.71969 | 0.46325 | 0.02513 | 0.0883 | None | None | G-measure geometric mean of precision and sensitivity |
| GI | 0.40078 | 0.50398 | 0.32771 | 0.00164 | 0.01114 | 0.0 | 0.0 | Gini index |
| GM | 0.66848 | 0.7508 | 0.65923 | 0.08678 | 0.10812 | 0.0 | 0.0 | G-mean geometric mean of specificity and sensitivity |
| IBA | 0.26009 | 0.6114 | 0.36657 | 0.0001 | 0.00014 | 0.0 | 0.0 | Index of balanced accuracy |
| ICSI | 0.07449 | 0.44639 | -0.04793 | -0.90909 | -0.32164 | None | None | Individual classification success index |
| IS | 1.50783 | 0.68163 | 0.85201 | 0.32557 | 2.95211 | None | None | Information score |
| J | 0.36364 | 0.55788 | 0.291 | 0.00699 | 0.01163 | 0.0 | 0.0 | Jaccard index |
| LS | 2.84382 | 1.60395 | 1.80502 | 1.25316 | 7.73879 | None | None | Lift score |
| MCC | 0.42798 | 0.49512 | 0.28157 | 0.00527 | 0.0805 | None | None | Matthews correlation coefficient |
| MCCI | Weak | Weak | Negligible | Negligible | Negligible | None | None | Matthews correlation coefficient interpretation |
| MCEN | 0.55878 | 0.47216 | 0.63663 | 0.52973 | 0.45075 | 0.38348 | 0.38474 | Modified confusion entropy |
| MK | 0.45702 | 0.48642 | 0.24193 | 0.01694 | 0.5814 | None | None | Markedness |
| N | 1578 | 1178 | 1582 | 1853 | 1814 | 1937 | 1968 | Condition negative |
| NLR | 0.55928 | 0.28985 | 0.55841 | 0.99835 | 0.98885 | 1.0 | 1.0 | Negative likelihood ratio |
| NLRI | Negligible | Poor | Negligible | Negligible | Negligible | Negligible | Negligible | Negative likelihood ratio interpretation |
| NPV | 0.87393 | 0.83433 | 0.87547 | 0.9336 | 0.91473 | 0.97582 | 0.99144 | Negative predictive value |
| OC | 0.58309 | 0.7943 | 0.58561 | 0.08333 | 0.66667 | None | None | Overlap coefficient |
| OOC | 0.53529 | 0.71969 | 0.46325 | 0.02513 | 0.0883 | None | None | Otsuka-Ochiai coefficient |
| OP | 0.52529 | 0.68781 | 0.59246 | -0.05641 | -0.06251 | -0.02418 | -0.00856 | Optimized precision |
| P | 407 | 807 | 403 | 132 | 171 | 48 | 17 | Condition positive or support |
| PLR | 5.42259 | 2.73592 | 2.27067 | 1.27617 | 21.21637 | None | None | Positive likelihood ratio |
| PLRI | Fair | Poor | Poor | Poor | Good | None | None | Positive likelihood ratio interpretation |
| POP | 1985 | 1985 | 1985 | 1985 | 1985 | 1985 | 1985 | Population |
| PPV | 0.58309 | 0.65209 | 0.36646 | 0.08333 | 0.66667 | None | None | Precision or positive predictive value |
| PRE | 0.20504 | 0.40655 | 0.20302 | 0.0665 | 0.08615 | 0.02418 | 0.00856 | Prevalence |
| Q | 0.81301 | 0.80841 | 0.60524 | 0.12214 | 0.91094 | None | None | Yule Q - coefficient of colligation |
| QI | Strong | Strong | Moderate | Negligible | Strong | None | None | Yule Q interpretation |
| RACC | 0.03543 | 0.20133 | 0.06587 | 0.0004 | 0.00013 | 0.0 | 0.0 | Random accuracy |
| RACCU | 0.03569 | 0.20329 | 0.06955 | 0.00132 | 0.00192 | 0.00015 | 2e-05 | Random accuracy unbiased |
| TN | 1435 | 836 | 1174 | 1842 | 1813 | 1937 | 1968 | True negative/correct rejection |
| TNR | 0.90938 | 0.70968 | 0.7421 | 0.99406 | 0.99945 | 1.0 | 1.0 | Specificity or true negative rate |
| TON | 1642 | 1002 | 1341 | 1973 | 1982 | 1985 | 1985 | Test outcome negative |
| TOP | 343 | 983 | 644 | 12 | 3 | 0 | 0 | Test outcome positive |
| TP | 200 | 641 | 236 | 1 | 2 | 0 | 0 | True positive/hit |
| TPR | 0.4914 | 0.7943 | 0.58561 | 0.00758 | 0.0117 | 0.0 | 0.0 | Sensitivity, recall, hit rate, or true positive rate |
| Y | 0.40078 | 0.50398 | 0.32771 | 0.00164 | 0.01114 | 0.0 | 0.0 | Youden index |
| dInd | 0.51661 | 0.35581 | 0.48809 | 0.99244 | 0.9883 | 1.0 | 1.0 | Distance index |
| sInd | 0.6347 | 0.74841 | 0.65487 | 0.29824 | 0.30116 | 0.29289 | 0.29289 | Similarity index |

Generated By PyCM Version 3.1
