# Supplementary material for: TopEC: prediction of Enzyme Commission classes by 3D graph neural networks and localized 3D protein descriptor
Source: Nat Commun. 2025 Mar 20;16:2737. doi: 10.1038/s41467-025-57324-5 (PMC11923149; doi:10.1038/s41467-025-57324-5)
Supplement: Supplementary file 3 — Supplementary Data 1 [file 41467_2025_57324_MOESM3_ESM.zip › Data_S1/table1/mainclass/EnzyNet/local/BindingMOAD_FOLD_flips.html]

PDB\_FOLD\_enzynet\_flips\_sites


# PyCM Report

## Dataset Type :

- Multi-Class Classification
- Imbalanced

Note 1 : Recommended statistics for this type of classification highlighted in aqua

Note 2 : The recommender system assumes that the input is the result of classification over the whole data rather than just a part of it.
If the confusion matrix is the result of test data classification, the recommendation is not valid.

## Confusion Matrix :

|  |  |  |  |  |  |  |  |  |  |  |  |  |  |  |  |  |  |  |  |  |  |  |  |  |  |  |  |  |  |  |  |  |  |  |  |  |  |  |  |  |  |  |  |  |  |  |  |  |  |  |  |  |  |  |  |  |  |  |  |  |  |  |  |  |  |
| --- | --- | --- | --- | --- | --- | --- | --- | --- | --- | --- | --- | --- | --- | --- | --- | --- | --- | --- | --- | --- | --- | --- | --- | --- | --- | --- | --- | --- | --- | --- | --- | --- | --- | --- | --- | --- | --- | --- | --- | --- | --- | --- | --- | --- | --- | --- | --- | --- | --- | --- | --- | --- | --- | --- | --- | --- | --- | --- | --- | --- | --- | --- | --- | --- | --- |
| Actual | Predict  |  |  |  |  |  |  |  |  | | --- | --- | --- | --- | --- | --- | --- | --- | |  | 0 | 1 | 2 | 3 | 4 | 5 | 6 | | 0 | 202 | 80 | 122 | 3 | 0 | 0 | 0 | | 1 | 25 | 637 | 145 | 0 | 0 | 0 | 0 | | 2 | 45 | 108 | 244 | 6 | 0 | 0 | 0 | | 3 | 27 | 62 | 42 | 1 | 0 | 0 | 0 | | 4 | 37 | 61 | 71 | 1 | 1 | 0 | 0 | | 5 | 8 | 22 | 18 | 0 | 0 | 0 | 0 | | 6 | 2 | 10 | 5 | 0 | 0 | 0 | 0 | |

## Overall Statistics :

|  |  |
| --- | --- |
| 95% CI | (0.5247,0.5685) |
| ACC Macro | 0.87046 |
| ARI | 0.2158 |
| AUNP | 0.67872 |
| AUNU | 0.59014 |
| Bangdiwala B | 0.42386 |
| Bennett S | 0.47103 |
| CBA | 0.21955 |
| CSI | None |
| Chi-Squared | None |
| Chi-Squared DF | 36 |
| Conditional Entropy | 1.24145 |
| Cramer V | None |
| Cross Entropy | 2.70125 |
| F1 Macro | 0.24855 |
| F1 Micro | 0.5466 |
| FNR Macro | 0.72792 |
| FNR Micro | 0.4534 |
| FPR Macro | 0.09179 |
| FPR Micro | 0.07557 |
| Gwet AC1 | 0.48786 |
| Hamming Loss | 0.4534 |
| Joint Entropy | 3.4585 |
| KL Divergence | None |
| Kappa | 0.34946 |
| Kappa 95% CI | (0.31804,0.38088) |
| Kappa No Prevalence | 0.0932 |
| Kappa Standard Error | 0.01603 |
| Kappa Unbiased | 0.3411 |
| Krippendorff Alpha | 0.34127 |
| Lambda A | 0.24024 |
| Lambda B | 0.26667 |
| Mutual Information | 0.27479 |
| NIR | 0.40655 |
| Overall ACC | 0.5466 |
| Overall CEN | 0.43549 |
| Overall J | (1.23614,0.17659) |
| Overall MCC | 0.35995 |
| Overall MCEN | 0.53152 |
| Overall RACC | 0.30304 |
| Overall RACCU | 0.31188 |
| P-Value | None |
| PPV Macro | None |
| PPV Micro | 0.5466 |
| Pearson C | None |
| Phi-Squared | None |
| RCI | 0.12395 |
| RR | 283.57143 |
| Reference Entropy | 2.21706 |
| Response Entropy | 1.51624 |
| SOA1(Landis & Koch) | Fair |
| SOA2(Fleiss) | Poor |
| SOA3(Altman) | Fair |
| SOA4(Cicchetti) | Poor |
| SOA5(Cramer) | None |
| SOA6(Matthews) | Weak |
| Scott PI | 0.3411 |
| Standard Error | 0.01117 |
| TNR Macro | 0.90821 |
| TNR Micro | 0.92443 |
| TPR Macro | 0.27208 |
| TPR Micro | 0.5466 |
| Zero-one Loss | 900 |

## Class Statistics :

|  |  |  |  |  |  |  |  |  |
| --- | --- | --- | --- | --- | --- | --- | --- | --- |
| Class | 0 | 1 | 2 | 3 | 4 | 5 | 6 | Description |
| ACC | 0.82418 | 0.74156 | 0.71688 | 0.92897 | 0.91436 | 0.97582 | 0.99144 | Accuracy |
| AGF | 0.67158 | 0.7797 | 0.6776 | 0.09364 | 0.0824 | 0.0 | 0.0 | Adjusted F-score |
| AGM | 0.77662 | 0.73341 | 0.70434 | 0.52509 | 0.51745 | 0 | 0 | Adjusted geometric mean |
| AM | -61 | 173 | 244 | -121 | -170 | -48 | -17 | Difference between automatic and manual classification |
| AUC | 0.70253 | 0.74909 | 0.67536 | 0.50109 | 0.50292 | 0.5 | 0.5 | Area under the ROC curve |
| AUCI | Good | Good | Fair | Poor | Poor | Poor | Poor | AUC value interpretation |
| AUPR | 0.54006 | 0.71967 | 0.49129 | 0.04924 | 0.50292 | None | None | Area under the PR curve |
| BCD | 0.01537 | 0.04358 | 0.06146 | 0.03048 | 0.04282 | 0.01209 | 0.00428 | Bray-Curtis dissimilarity |
| BM | 0.40506 | 0.49817 | 0.35072 | 0.00218 | 0.00585 | 0.0 | 0.0 | Informedness or bookmaker informedness |
| CEN | 0.47002 | 0.35705 | 0.53444 | 0.51742 | 0.44 | 0.41209 | 0.37178 | Confusion entropy |
| DOR | 9.8126 | 9.12185 | 4.48954 | 1.40687 | None | None | None | Diagnostic odds ratio |
| DP | 0.5468 | 0.52932 | 0.35958 | 0.08174 | None | None | None | Discriminant power |
| DPI | Poor | Poor | Poor | Poor | None | None | None | Discriminant power interpretation |
| ERR | 0.17582 | 0.25844 | 0.28312 | 0.07103 | 0.08564 | 0.02418 | 0.00856 | Error rate |
| F0.5 | 0.56393 | 0.67379 | 0.40789 | 0.02841 | 0.02857 | 0.0 | 0.0 | F0.5 score |
| F1 | 0.53652 | 0.71293 | 0.46476 | 0.01399 | 0.01163 | 0.0 | 0.0 | F1 score - harmonic mean of precision and sensitivity |
| F2 | 0.51165 | 0.75689 | 0.54006 | 0.00928 | 0.0073 | 0.0 | 0.0 | F2 score |
| FDR | 0.41618 | 0.35 | 0.62287 | 0.90909 | 0.0 | None | None | False discovery rate |
| FN | 205 | 170 | 159 | 131 | 170 | 48 | 17 | False negative/miss/type 2 error |
| FNR | 0.50369 | 0.21066 | 0.39454 | 0.99242 | 0.99415 | 1.0 | 1.0 | Miss rate or false negative rate |
| FOR | 0.12508 | 0.16915 | 0.11883 | 0.06636 | 0.08569 | 0.02418 | 0.00856 | False omission rate |
| FP | 144 | 343 | 403 | 10 | 0 | 0 | 0 | False positive/type 1 error/false alarm |
| FPR | 0.09125 | 0.29117 | 0.25474 | 0.0054 | 0.0 | 0.0 | 0.0 | Fall-out or false positive rate |
| G | 0.53829 | 0.71629 | 0.47784 | 0.02624 | 0.07647 | None | None | G-measure geometric mean of precision and sensitivity |
| GI | 0.40506 | 0.49817 | 0.35072 | 0.00218 | 0.00585 | 0.0 | 0.0 | Gini index |
| GM | 0.67158 | 0.748 | 0.67173 | 0.0868 | 0.07647 | 0.0 | 0.0 | G-mean geometric mean of specificity and sensitivity |
| IBA | 0.26501 | 0.60456 | 0.38814 | 0.0001 | 3e-05 | 0.0 | 0.0 | Index of balanced accuracy |
| ICSI | 0.08013 | 0.43934 | -0.01742 | -0.90152 | 0.00585 | None | None | Individual classification success index |
| IS | 1.50962 | 0.67701 | 0.8934 | 0.4511 | 3.53707 | None | None | Information score |
| J | 0.36661 | 0.55391 | 0.30273 | 0.00704 | 0.00585 | 0.0 | 0.0 | Jaccard index |
| LS | 2.84735 | 1.59882 | 1.85755 | 1.36708 | 11.60819 | None | None | Lift score |
| MCC | 0.43106 | 0.48943 | 0.30098 | 0.00731 | 0.07312 | None | None | Matthews correlation coefficient |
| MCCI | Weak | Weak | Weak | Negligible | Negligible | None | None | Matthews correlation coefficient interpretation |
| MCEN | 0.56272 | 0.4757 | 0.62202 | 0.51826 | 0.44024 | 0.41209 | 0.37178 | Modified confusion entropy |
| MK | 0.45874 | 0.48085 | 0.25829 | 0.02455 | 0.91431 | None | None | Markedness |
| N | 1578 | 1178 | 1582 | 1853 | 1814 | 1937 | 1968 | Condition negative |
| NLR | 0.55426 | 0.29719 | 0.5294 | 0.99781 | 0.99415 | 1.0 | 1.0 | Negative likelihood ratio |
| NLRI | Negligible | Poor | Negligible | Negligible | Negligible | Negligible | Negligible | Negative likelihood ratio interpretation |
| NPV | 0.87492 | 0.83085 | 0.88117 | 0.93364 | 0.91431 | 0.97582 | 0.99144 | Negative predictive value |
| OC | 0.58382 | 0.78934 | 0.60546 | 0.09091 | 1.0 | None | None | Overlap coefficient |
| OOC | 0.53829 | 0.71629 | 0.47784 | 0.02624 | 0.07647 | None | None | Otsuka-Ochiai coefficient |
| OP | 0.53065 | 0.68782 | 0.61338 | -0.05591 | -0.07401 | -0.02418 | -0.00856 | Optimized precision |
| P | 407 | 807 | 403 | 132 | 171 | 48 | 17 | Condition positive or support |
| PLR | 5.43878 | 2.71092 | 2.37676 | 1.40379 | None | None | None | Positive likelihood ratio |
| PLRI | Fair | Poor | Poor | Poor | None | None | None | Positive likelihood ratio interpretation |
| POP | 1985 | 1985 | 1985 | 1985 | 1985 | 1985 | 1985 | Population |
| PPV | 0.58382 | 0.65 | 0.37713 | 0.09091 | 1.0 | None | None | Precision or positive predictive value |
| PRE | 0.20504 | 0.40655 | 0.20302 | 0.0665 | 0.08615 | 0.02418 | 0.00856 | Prevalence |
| Q | 0.81503 | 0.80241 | 0.63567 | 0.16905 | None | None | None | Yule Q - coefficient of colligation |
| QI | Strong | Strong | Moderate | Negligible | None | None | None | Yule Q interpretation |
| RACC | 0.03574 | 0.20071 | 0.06617 | 0.00037 | 4e-05 | 0.0 | 0.0 | Random accuracy |
| RACCU | 0.03598 | 0.20261 | 0.06995 | 0.0013 | 0.00188 | 0.00015 | 2e-05 | Random accuracy unbiased |
| TN | 1434 | 835 | 1179 | 1843 | 1814 | 1937 | 1968 | True negative/correct rejection |
| TNR | 0.90875 | 0.70883 | 0.74526 | 0.9946 | 1.0 | 1.0 | 1.0 | Specificity or true negative rate |
| TON | 1639 | 1005 | 1338 | 1974 | 1984 | 1985 | 1985 | Test outcome negative |
| TOP | 346 | 980 | 647 | 11 | 1 | 0 | 0 | Test outcome positive |
| TP | 202 | 637 | 244 | 1 | 1 | 0 | 0 | True positive/hit |
| TPR | 0.49631 | 0.78934 | 0.60546 | 0.00758 | 0.00585 | 0.0 | 0.0 | Sensitivity, recall, hit rate, or true positive rate |
| Y | 0.40506 | 0.49817 | 0.35072 | 0.00218 | 0.00585 | 0.0 | 0.0 | Youden index |
| dInd | 0.51189 | 0.35938 | 0.46963 | 0.99244 | 0.99415 | 1.0 | 1.0 | Distance index |
| sInd | 0.63804 | 0.74588 | 0.66792 | 0.29824 | 0.29703 | 0.29289 | 0.29289 | Similarity index |

Generated By PyCM Version 3.1
