# Supplementary material for: TopEC: prediction of Enzyme Commission classes by 3D graph neural networks and localized 3D protein descriptor
Source: Nat Commun. 2025 Mar 20;16:2737. doi: 10.1038/s41467-025-57324-5 (PMC11923149; doi:10.1038/s41467-025-57324-5)
Supplement: Supplementary file 3 — Supplementary Data 1 [file 41467_2025_57324_MOESM3_ESM.zip › Data_S1/table1/mainclass/EnzyNet/local/BindingMOAD_FOLD.html]

PDB\_FOLD\_Enzy\_sites\_no\_aug


# PyCM Report

## Dataset Type :

- Multi-Class Classification
- Imbalanced

Note 1 : Recommended statistics for this type of classification highlighted in aqua

Note 2 : The recommender system assumes that the input is the result of classification over the whole data rather than just a part of it.
If the confusion matrix is the result of test data classification, the recommendation is not valid.

## Confusion Matrix :

|  |  |  |  |  |  |  |  |  |  |  |  |  |  |  |  |  |  |  |  |  |  |  |  |  |  |  |  |  |  |  |  |  |  |  |  |  |  |  |  |  |  |  |  |  |  |  |  |  |  |  |  |  |  |  |  |  |  |  |  |  |  |  |  |  |  |
| --- | --- | --- | --- | --- | --- | --- | --- | --- | --- | --- | --- | --- | --- | --- | --- | --- | --- | --- | --- | --- | --- | --- | --- | --- | --- | --- | --- | --- | --- | --- | --- | --- | --- | --- | --- | --- | --- | --- | --- | --- | --- | --- | --- | --- | --- | --- | --- | --- | --- | --- | --- | --- | --- | --- | --- | --- | --- | --- | --- | --- | --- | --- | --- | --- | --- |
| Actual | Predict  |  |  |  |  |  |  |  |  | | --- | --- | --- | --- | --- | --- | --- | --- | |  | 0 | 1 | 2 | 3 | 4 | 5 | 6 | | 0 | 185 | 103 | 108 | 4 | 3 | 1 | 3 | | 1 | 38 | 625 | 137 | 2 | 0 | 4 | 1 | | 2 | 69 | 119 | 193 | 15 | 6 | 1 | 0 | | 3 | 32 | 47 | 46 | 4 | 2 | 1 | 0 | | 4 | 32 | 59 | 66 | 2 | 8 | 3 | 1 | | 5 | 7 | 12 | 28 | 1 | 0 | 0 | 0 | | 6 | 2 | 10 | 5 | 0 | 0 | 0 | 0 | |

## Overall Statistics :

|  |  |
| --- | --- |
| 95% CI | (0.48934,0.53333) |
| ACC Macro | 0.86038 |
| ARI | 0.19478 |
| AUNP | 0.65779 |
| AUNU | 0.57861 |
| Bangdiwala B | 0.3924 |
| Bennett S | 0.42989 |
| CBA | 0.21481 |
| CSI | -0.45317 |
| Chi-Squared | 661.7716 |
| Chi-Squared DF | 36 |
| Conditional Entropy | 1.45164 |
| Cramer V | 0.23572 |
| Cross Entropy | 2.52188 |
| F1 Macro | 0.24377 |
| F1 Micro | 0.51134 |
| FNR Macro | 0.745 |
| FNR Micro | 0.48866 |
| FPR Macro | 0.09777 |
| FPR Micro | 0.08144 |
| Gwet AC1 | 0.44733 |
| Hamming Loss | 0.48866 |
| Joint Entropy | 3.66869 |
| KL Divergence | 0.30483 |
| Kappa | 0.30298 |
| Kappa 95% CI | (0.27161,0.33434) |
| Kappa No Prevalence | 0.02267 |
| Kappa Standard Error | 0.016 |
| Kappa Unbiased | 0.29675 |
| Krippendorff Alpha | 0.29692 |
| Lambda A | 0.19185 |
| Lambda B | 0.17723 |
| Mutual Information | 0.23166 |
| NIR | 0.40655 |
| Overall ACC | 0.51134 |
| Overall CEN | 0.4849 |
| Overall J | (1.16833,0.1669) |
| Overall MCC | 0.30927 |
| Overall MCEN | 0.58582 |
| Overall RACC | 0.29893 |
| Overall RACCU | 0.30514 |
| P-Value | None |
| PPV Macro | 0.29183 |
| PPV Micro | 0.51134 |
| Pearson C | 0.50003 |
| Phi-Squared | 0.33339 |
| RCI | 0.10449 |
| RR | 283.57143 |
| Reference Entropy | 2.21706 |
| Response Entropy | 1.68329 |
| SOA1(Landis & Koch) | Fair |
| SOA2(Fleiss) | Poor |
| SOA3(Altman) | Fair |
| SOA4(Cicchetti) | Poor |
| SOA5(Cramer) | Moderate |
| SOA6(Matthews) | Weak |
| Scott PI | 0.29675 |
| Standard Error | 0.01122 |
| TNR Macro | 0.90223 |
| TNR Micro | 0.91856 |
| TPR Macro | 0.255 |
| TPR Micro | 0.51134 |
| Zero-one Loss | 970 |

## Class Statistics :

|  |  |  |  |  |  |  |  |  |
| --- | --- | --- | --- | --- | --- | --- | --- | --- |
| Class | 0 | 1 | 2 | 3 | 4 | 5 | 6 | Description |
| ACC | 0.79748 | 0.73199 | 0.69773 | 0.92343 | 0.91234 | 0.97078 | 0.98892 | Accuracy |
| AGF | 0.63452 | 0.76806 | 0.60368 | 0.18434 | 0.23022 | 0.0 | 0.0 | Adjusted F-score |
| AGM | 0.7459 | 0.7248 | 0.66846 | 0.566 | 0.58727 | 0 | 0 | Adjusted geometric mean |
| AM | -42 | 168 | 180 | -104 | -152 | -38 | -12 | Difference between automatic and manual classification |
| AUC | 0.67024 | 0.73868 | 0.61619 | 0.50868 | 0.52036 | 0.49742 | 0.49873 | Area under the ROC curve |
| AUCI | Fair | Good | Fair | Poor | Poor | Poor | Poor | AUC value interpretation |
| AUPR | 0.4807 | 0.70775 | 0.40498 | 0.08658 | 0.23392 | 0.0 | 0.0 | Area under the PR curve |
| BCD | 0.01058 | 0.04232 | 0.04534 | 0.0262 | 0.03829 | 0.00957 | 0.00302 | Bray-Curtis dissimilarity |
| BM | 0.34048 | 0.47736 | 0.23238 | 0.01735 | 0.04072 | -0.00516 | -0.00254 | Informedness or bookmaker informedness |
| CEN | 0.52676 | 0.37095 | 0.60824 | 0.63665 | 0.56101 | 0.62392 | 0.58988 | Confusion entropy |
| DOR | 6.47222 | 8.12402 | 2.80899 | 2.38151 | 8.04462 | 0.0 | 0.0 | Diagnostic odds ratio |
| DP | 0.44716 | 0.50158 | 0.2473 | 0.20777 | 0.49923 | None | None | Discriminant power |
| DPI | Poor | Poor | Poor | Poor | Poor | None | None | Discriminant power interpretation |
| ERR | 0.20252 | 0.26801 | 0.30227 | 0.07657 | 0.08766 | 0.02922 | 0.01108 | Error rate |
| F0.5 | 0.49545 | 0.6639 | 0.35283 | 0.08197 | 0.16194 | 0.0 | 0.0 | F0.5 score |
| F1 | 0.47927 | 0.70146 | 0.39148 | 0.05 | 0.08421 | 0.0 | 0.0 | F1 score - harmonic mean of precision and sensitivity |
| F2 | 0.46412 | 0.74352 | 0.43964 | 0.03597 | 0.0569 | 0.0 | 0.0 | F2 score |
| FDR | 0.49315 | 0.35897 | 0.66895 | 0.85714 | 0.57895 | 1.0 | 1.0 | False discovery rate |
| FN | 222 | 182 | 210 | 128 | 163 | 48 | 17 | False negative/miss/type 2 error |
| FNR | 0.54545 | 0.22553 | 0.52109 | 0.9697 | 0.95322 | 1.0 | 1.0 | Miss rate or false negative rate |
| FOR | 0.13704 | 0.1802 | 0.14979 | 0.06541 | 0.08291 | 0.0243 | 0.00859 | False omission rate |
| FP | 180 | 350 | 390 | 24 | 11 | 10 | 5 | False positive/type 1 error/false alarm |
| FPR | 0.11407 | 0.29711 | 0.24652 | 0.01295 | 0.00606 | 0.00516 | 0.00254 | Fall-out or false positive rate |
| G | 0.47999 | 0.7046 | 0.39817 | 0.0658 | 0.14035 | 0.0 | 0.0 | G-measure geometric mean of precision and sensitivity |
| GI | 0.34048 | 0.47736 | 0.23238 | 0.01735 | 0.04072 | -0.00516 | -0.00254 | Gini index |
| GM | 0.63458 | 0.73781 | 0.6007 | 0.17295 | 0.21564 | 0.0 | 0.0 | G-mean geometric mean of specificity and sensitivity |
| IBA | 0.22898 | 0.58334 | 0.26177 | 0.00129 | 0.00246 | 0.0 | 0.0 | Index of balanced accuracy |
| ICSI | -0.03861 | 0.4155 | -0.19005 | -0.82684 | -0.53216 | -1.0 | -1.0 | Individual classification success index |
| IS | 1.30567 | 0.65695 | 0.70539 | 1.10317 | 2.28914 | None | None | Information score |
| J | 0.31516 | 0.54019 | 0.24338 | 0.02564 | 0.04396 | 0.0 | 0.0 | Jaccard index |
| LS | 2.47198 | 1.57675 | 1.63059 | 2.14827 | 4.88766 | 0.0 | 0.0 | Lift score |
| MCC | 0.35484 | 0.46902 | 0.20524 | 0.03666 | 0.11734 | -0.0112 | -0.00467 | Matthews correlation coefficient |
| MCCI | Weak | Weak | Negligible | Negligible | Negligible | Negligible | Negligible | Matthews correlation coefficient interpretation |
| MCEN | 0.61727 | 0.49142 | 0.68995 | 0.64305 | 0.56912 | 0.62392 | 0.58988 | Modified confusion entropy |
| MK | 0.36981 | 0.46083 | 0.18126 | 0.07745 | 0.33814 | -0.0243 | -0.00859 | Markedness |
| N | 1578 | 1178 | 1582 | 1853 | 1814 | 1937 | 1968 | Condition negative |
| NLR | 0.61568 | 0.32086 | 0.69158 | 0.98242 | 0.95903 | 1.00519 | 1.00255 | Negative likelihood ratio |
| NLRI | Negligible | Poor | Negligible | Negligible | Negligible | Negligible | Negligible | Negative likelihood ratio interpretation |
| NPV | 0.86296 | 0.8198 | 0.85021 | 0.93459 | 0.91709 | 0.9757 | 0.99141 | Negative predictive value |
| OC | 0.50685 | 0.77447 | 0.47891 | 0.14286 | 0.42105 | 0.0 | 0.0 | Overlap coefficient |
| OOC | 0.47999 | 0.7046 | 0.39817 | 0.0658 | 0.14035 | 0.0 | 0.0 | Otsuka-Ochiai coefficient |
| OP | 0.47567 | 0.68353 | 0.47494 | -0.017 | 0.00225 | -0.02922 | -0.01108 | Optimized precision |
| P | 407 | 807 | 403 | 132 | 171 | 48 | 17 | Condition positive or support |
| PLR | 3.98485 | 2.60666 | 1.94265 | 2.33965 | 7.71505 | 0.0 | 0.0 | Positive likelihood ratio |
| PLRI | Poor | Poor | Poor | Poor | Fair | Negligible | Negligible | Positive likelihood ratio interpretation |
| POP | 1985 | 1985 | 1985 | 1985 | 1985 | 1985 | 1985 | Population |
| PPV | 0.50685 | 0.64103 | 0.33105 | 0.14286 | 0.42105 | 0.0 | 0.0 | Precision or positive predictive value |
| PRE | 0.20504 | 0.40655 | 0.20302 | 0.0665 | 0.08615 | 0.02418 | 0.00856 | Prevalence |
| Q | 0.73234 | 0.7808 | 0.47493 | 0.40855 | 0.77887 | -1.0 | -1.0 | Yule Q - coefficient of colligation |
| QI | Moderate | Strong | Weak | Weak | Strong | Negligible | Negligible | Yule Q interpretation |
| RACC | 0.0377 | 0.19969 | 0.05963 | 0.00094 | 0.00082 | 0.00012 | 2e-05 | Random accuracy |
| RACCU | 0.03781 | 0.20148 | 0.06168 | 0.00162 | 0.00229 | 0.00021 | 3e-05 | Random accuracy unbiased |
| TN | 1398 | 828 | 1192 | 1829 | 1803 | 1927 | 1963 | True negative/correct rejection |
| TNR | 0.88593 | 0.70289 | 0.75348 | 0.98705 | 0.99394 | 0.99484 | 0.99746 | Specificity or true negative rate |
| TON | 1620 | 1010 | 1402 | 1957 | 1966 | 1975 | 1980 | Test outcome negative |
| TOP | 365 | 975 | 583 | 28 | 19 | 10 | 5 | Test outcome positive |
| TP | 185 | 625 | 193 | 4 | 8 | 0 | 0 | True positive/hit |
| TPR | 0.45455 | 0.77447 | 0.47891 | 0.0303 | 0.04678 | 0.0 | 0.0 | Sensitivity, recall, hit rate, or true positive rate |
| Y | 0.34048 | 0.47736 | 0.23238 | 0.01735 | 0.04072 | -0.00516 | -0.00254 | Youden index |
| dInd | 0.55725 | 0.37301 | 0.57646 | 0.96978 | 0.95324 | 1.00001 | 1.0 | Distance index |
| sInd | 0.60596 | 0.73624 | 0.59238 | 0.31426 | 0.32596 | 0.29288 | 0.29289 | Similarity index |

Generated By PyCM Version 3.1
