# Supplementary material for: TopEC: prediction of Enzyme Commission classes by 3D graph neural networks and localized 3D protein descriptor
Source: Nat Commun. 2025 Mar 20;16:2737. doi: 10.1038/s41467-025-57324-5 (PMC11923149; doi:10.1038/s41467-025-57324-5)
Supplement: Supplementary file 3 — Supplementary Data 1 [file 41467_2025_57324_MOESM3_ESM.zip › Data_S1/table1/mainclass/EnzyNet/full_struc/TopEnzyme_TEMP_wflips.html]

PyCM Report


# PyCM Report

## Dataset Type :

- Multi-Class Classification
- Imbalanced

Note 1 : Recommended statistics for this type of classification highlighted in aqua

Note 2 : The recommender system assumes that the input is the result of classification over the whole data rather than just a part of it.
If the confusion matrix is the result of test data classification, the recommendation is not valid.

## Confusion Matrix :

|  |  |  |  |  |  |  |  |  |  |  |  |  |  |  |  |  |  |  |  |  |  |  |  |  |  |  |  |  |  |  |  |  |  |  |  |  |  |  |  |  |  |  |  |  |  |  |  |  |  |  |  |  |  |  |  |  |  |  |  |  |  |  |  |  |  |
| --- | --- | --- | --- | --- | --- | --- | --- | --- | --- | --- | --- | --- | --- | --- | --- | --- | --- | --- | --- | --- | --- | --- | --- | --- | --- | --- | --- | --- | --- | --- | --- | --- | --- | --- | --- | --- | --- | --- | --- | --- | --- | --- | --- | --- | --- | --- | --- | --- | --- | --- | --- | --- | --- | --- | --- | --- | --- | --- | --- | --- | --- | --- | --- | --- | --- |
| Actual | Predict  |  |  |  |  |  |  |  |  | | --- | --- | --- | --- | --- | --- | --- | --- | |  | 0 | 1 | 2 | 3 | 4 | 5 | 6 | | 0 | 86 | 73 | 49 | 56 | 1 | 2 | 0 | | 1 | 31 | 129 | 63 | 4 | 1 | 4 | 0 | | 2 | 25 | 45 | 133 | 3 | 0 | 8 | 0 | | 3 | 15 | 18 | 30 | 7 | 0 | 1 | 1 | | 4 | 4 | 6 | 10 | 1 | 3 | 8 | 0 | | 5 | 8 | 11 | 7 | 0 | 0 | 5 | 0 | | 6 | 18 | 34 | 24 | 2 | 0 | 9 | 5 | |

## Overall Statistics :

|  |  |
| --- | --- |
| 95% CI | (0.36029,0.42269) |
| ACC Macro | 0.82614 |
| ARI | 0.07953 |
| AUNP | 0.60956 |
| AUNU | 0.58038 |
| Bangdiwala B | 0.2113 |
| Bennett S | 0.29007 |
| CBA | 0.21862 |
| CSI | -0.30577 |
| Chi-Squared | 406.27167 |
| Chi-Squared DF | 36 |
| Conditional Entropy | 1.83255 |
| Cramer V | 0.26839 |
| Cross Entropy | 2.81504 |
| F1 Macro | 0.26562 |
| F1 Micro | 0.39149 |
| FNR Macro | 0.72768 |
| FNR Micro | 0.60851 |
| FPR Macro | 0.11156 |
| FPR Micro | 0.10142 |
| Gwet AC1 | 0.30232 |
| Hamming Loss | 0.60851 |
| Joint Entropy | 4.27299 |
| KL Divergence | 0.3746 |
| Kappa | 0.21578 |
| Kappa 95% CI | (0.17556,0.25599) |
| Kappa No Prevalence | -0.21702 |
| Kappa Standard Error | 0.02052 |
| Kappa Unbiased | 0.20651 |
| Krippendorff Alpha | 0.20693 |
| Lambda A | 0.22883 |
| Lambda B | 0.1875 |
| Mutual Information | 0.24504 |
| NIR | 0.28404 |
| Overall ACC | 0.39149 |
| Overall CEN | 0.57947 |
| Overall J | (1.14867,0.1641) |
| Overall MCC | 0.22112 |
| Overall MCEN | 0.65889 |
| Overall RACC | 0.22406 |
| Overall RACCU | 0.23312 |
| P-Value | 0.0 |
| PPV Macro | 0.42191 |
| PPV Micro | 0.39149 |
| Pearson C | 0.54934 |
| Phi-Squared | 0.4322 |
| RCI | 0.10041 |
| RR | 134.28571 |
| Reference Entropy | 2.44044 |
| Response Entropy | 2.0776 |
| SOA1(Landis & Koch) | Fair |
| SOA2(Fleiss) | Poor |
| SOA3(Altman) | Fair |
| SOA4(Cicchetti) | Poor |
| SOA5(Cramer) | Moderate |
| SOA6(Matthews) | Negligible |
| Scott PI | 0.20651 |
| Standard Error | 0.01592 |
| TNR Macro | 0.88844 |
| TNR Micro | 0.89858 |
| TPR Macro | 0.27232 |
| TPR Micro | 0.39149 |
| Zero-one Loss | 572 |

## Class Statistics :

|  |  |  |  |  |  |  |  |  |
| --- | --- | --- | --- | --- | --- | --- | --- | --- |
| Class | 0 | 1 | 2 | 3 | 4 | 5 | 6 | Description |
| ACC | 0.7 | 0.69149 | 0.71915 | 0.86064 | 0.96702 | 0.9383 | 0.90638 | Accuracy |
| AGF | 0.51568 | 0.64927 | 0.69146 | 0.29944 | 0.33154 | 0.38808 | 0.24851 | Adjusted F-score |
| AGM | 0.65953 | 0.681 | 0.71061 | 0.59941 | 0.64583 | 0.67486 | 0.5962 | Adjusted geometric mean |
| AM | -80 | 84 | 102 | 1 | -27 | 6 | -86 | Difference between automatic and manual classification |
| AUC | 0.58601 | 0.64596 | 0.68471 | 0.51059 | 0.54577 | 0.56304 | 0.52658 | Area under the ROC curve |
| AUCI | Poor | Fair | Fair | Poor | Poor | Poor | Poor | AUC value interpretation |
| AUPR | 0.391 | 0.48213 | 0.52119 | 0.09656 | 0.34688 | 0.14821 | 0.44384 | Area under the PR curve |
| BCD | 0.04255 | 0.04468 | 0.05426 | 0.00053 | 0.01436 | 0.00319 | 0.04574 | Bray-Curtis dissimilarity |
| BM | 0.17202 | 0.29191 | 0.36943 | 0.02119 | 0.09155 | 0.12609 | 0.05317 | Informedness or bookmaker informedness |
| CEN | 0.61414 | 0.55569 | 0.54492 | 0.61508 | 0.60888 | 0.75823 | 0.55075 | Confusion entropy |
| DOR | 2.69088 | 3.48938 | 4.87209 | 1.30862 | 46.86207 | 5.27043 | 48.67816 | Diagnostic odds ratio |
| DP | 0.23701 | 0.29923 | 0.37916 | 0.0644 | 0.92117 | 0.39797 | 0.93028 | Discriminant power |
| DPI | Poor | Poor | Poor | Poor | Poor | Poor | Poor | Discriminant power interpretation |
| ERR | 0.3 | 0.30851 | 0.28085 | 0.13936 | 0.03298 | 0.0617 | 0.09362 | Error rate |
| F0.5 | 0.42365 | 0.43115 | 0.44993 | 0.09615 | 0.28846 | 0.13966 | 0.21552 | F0.5 score |
| F1 | 0.37885 | 0.4708 | 0.50189 | 0.09655 | 0.16216 | 0.14706 | 0.10204 | F1 score - harmonic mean of precision and sensitivity |
| F2 | 0.34263 | 0.51849 | 0.56741 | 0.09695 | 0.11278 | 0.15528 | 0.06684 | F2 score |
| FDR | 0.54011 | 0.59177 | 0.57911 | 0.90411 | 0.4 | 0.86486 | 0.16667 | False discovery rate |
| FN | 181 | 103 | 81 | 65 | 29 | 26 | 87 | False negative/miss/type 2 error |
| FNR | 0.6779 | 0.44397 | 0.3785 | 0.90278 | 0.90625 | 0.83871 | 0.94565 | Miss rate or false negative rate |
| FOR | 0.24037 | 0.16506 | 0.12981 | 0.07497 | 0.03102 | 0.02879 | 0.09315 | False omission rate |
| FP | 101 | 187 | 183 | 66 | 2 | 32 | 1 | False positive/type 1 error/false alarm |
| FPR | 0.15007 | 0.26412 | 0.25207 | 0.07604 | 0.0022 | 0.0352 | 0.00118 | Fall-out or false positive rate |
| G | 0.38488 | 0.47643 | 0.51145 | 0.09655 | 0.23717 | 0.14763 | 0.21281 | G-measure geometric mean of precision and sensitivity |
| GI | 0.17202 | 0.29191 | 0.36943 | 0.02119 | 0.09155 | 0.12609 | 0.05317 | Gini index |
| GM | 0.52322 | 0.63967 | 0.68179 | 0.29972 | 0.30585 | 0.39448 | 0.23299 | G-mean geometric mean of specificity and sensitivity |
| IBA | 0.12926 | 0.33559 | 0.40606 | 0.01556 | 0.00898 | 0.03058 | 0.00301 | Index of balanced accuracy |
| ICSI | -0.21801 | -0.03574 | 0.04238 | -0.80689 | -0.30625 | -0.70357 | -0.11232 | Individual classification success index |
| IS | 0.69519 | 0.72598 | 0.88655 | 0.32412 | 4.13955 | 2.0348 | 3.08992 | Information score |
| J | 0.2337 | 0.30788 | 0.33501 | 0.05072 | 0.08824 | 0.07937 | 0.05376 | Jaccard index |
| LS | 1.6191 | 1.65403 | 1.84875 | 1.2519 | 17.625 | 4.09765 | 8.51449 | Lift score |
| MCC | 0.19433 | 0.26642 | 0.32792 | 0.02105 | 0.22823 | 0.11579 | 0.19838 | Matthews correlation coefficient |
| MCCI | Negligible | Negligible | Weak | Negligible | Negligible | Negligible | Negligible | Matthews correlation coefficient interpretation |
| MCEN | 0.6929 | 0.65202 | 0.65015 | 0.62738 | 0.63158 | 0.79011 | 0.56042 | Modified confusion entropy |
| MK | 0.21952 | 0.24316 | 0.29108 | 0.02092 | 0.56898 | 0.10634 | 0.74019 | Markedness |
| N | 673 | 708 | 726 | 868 | 908 | 909 | 848 | Condition negative |
| NLR | 0.7976 | 0.60332 | 0.50607 | 0.97707 | 0.90825 | 0.86931 | 0.94677 | Negative likelihood ratio |
| NLRI | Negligible | Negligible | Negligible | Negligible | Negligible | Negligible | Negligible | Negative likelihood ratio interpretation |
| NPV | 0.75963 | 0.83494 | 0.87019 | 0.92503 | 0.96898 | 0.97121 | 0.90685 | Negative predictive value |
| OC | 0.45989 | 0.55603 | 0.6215 | 0.09722 | 0.6 | 0.16129 | 0.83333 | Overlap coefficient |
| OOC | 0.38488 | 0.47643 | 0.51145 | 0.09655 | 0.23717 | 0.14763 | 0.21281 | Otsuka-Ochiai coefficient |
| OP | 0.24964 | 0.55228 | 0.62682 | 0.05105 | 0.1388 | 0.22476 | 0.00959 | Optimized precision |
| P | 267 | 232 | 214 | 72 | 32 | 31 | 92 | Condition positive or support |
| PLR | 2.14625 | 2.1052 | 2.4656 | 1.27862 | 42.5625 | 4.58165 | 46.08696 | Positive likelihood ratio |
| PLRI | Poor | Poor | Poor | Poor | Good | Poor | Good | Positive likelihood ratio interpretation |
| POP | 940 | 940 | 940 | 940 | 940 | 940 | 940 | Population |
| PPV | 0.45989 | 0.40823 | 0.42089 | 0.09589 | 0.6 | 0.13514 | 0.83333 | Precision or positive predictive value |
| PRE | 0.28404 | 0.24681 | 0.22766 | 0.0766 | 0.03404 | 0.03298 | 0.09787 | Prevalence |
| Q | 0.45812 | 0.5545 | 0.65941 | 0.13368 | 0.95821 | 0.68104 | 0.95974 | Yule Q - coefficient of colligation |
| QI | Weak | Moderate | Moderate | Negligible | Strong | Moderate | Strong | Yule Q interpretation |
| RACC | 0.05651 | 0.08297 | 0.07653 | 0.00595 | 0.00018 | 0.0013 | 0.00062 | Random accuracy |
| RACCU | 0.05832 | 0.08497 | 0.07948 | 0.00595 | 0.00039 | 0.00131 | 0.00272 | Random accuracy unbiased |
| TN | 572 | 521 | 543 | 802 | 906 | 877 | 847 | True negative/correct rejection |
| TNR | 0.84993 | 0.73588 | 0.74793 | 0.92396 | 0.9978 | 0.9648 | 0.99882 | Specificity or true negative rate |
| TON | 753 | 624 | 624 | 867 | 935 | 903 | 934 | Test outcome negative |
| TOP | 187 | 316 | 316 | 73 | 5 | 37 | 6 | Test outcome positive |
| TP | 86 | 129 | 133 | 7 | 3 | 5 | 5 | True positive/hit |
| TPR | 0.3221 | 0.55603 | 0.6215 | 0.09722 | 0.09375 | 0.16129 | 0.05435 | Sensitivity, recall, hit rate, or true positive rate |
| Y | 0.17202 | 0.29191 | 0.36943 | 0.02119 | 0.09155 | 0.12609 | 0.05317 | Youden index |
| dInd | 0.69432 | 0.51659 | 0.45476 | 0.90597 | 0.90625 | 0.83945 | 0.94565 | Distance index |
| sInd | 0.50904 | 0.63471 | 0.67844 | 0.35938 | 0.35918 | 0.40642 | 0.33132 | Similarity index |

Generated By PyCM Version 3.2
