# Supplementary material for: TopEC: prediction of Enzyme Commission classes by 3D graph neural networks and localized 3D protein descriptor
Source: Nat Commun. 2025 Mar 20;16:2737. doi: 10.1038/s41467-025-57324-5 (PMC11923149; doi:10.1038/s41467-025-57324-5)
Supplement: Supplementary file 3 — Supplementary Data 1 [file 41467_2025_57324_MOESM3_ESM.zip › Data_S1/table1/mainclass/EnzyNet/full_struc/TopEnzyme_TEMP_flips.html]

PyCM Report


# PyCM Report

## Dataset Type :

- Multi-Class Classification
- Imbalanced

Note 1 : Recommended statistics for this type of classification highlighted in aqua

Note 2 : The recommender system assumes that the input is the result of classification over the whole data rather than just a part of it.
If the confusion matrix is the result of test data classification, the recommendation is not valid.

## Confusion Matrix :

|  |  |  |  |  |  |  |  |  |  |  |  |  |  |  |  |  |  |  |  |  |  |  |  |  |  |  |  |  |  |  |  |  |  |  |  |  |  |  |  |  |  |  |  |  |  |  |  |  |  |  |  |  |  |  |  |  |  |  |  |  |  |  |  |  |  |
| --- | --- | --- | --- | --- | --- | --- | --- | --- | --- | --- | --- | --- | --- | --- | --- | --- | --- | --- | --- | --- | --- | --- | --- | --- | --- | --- | --- | --- | --- | --- | --- | --- | --- | --- | --- | --- | --- | --- | --- | --- | --- | --- | --- | --- | --- | --- | --- | --- | --- | --- | --- | --- | --- | --- | --- | --- | --- | --- | --- | --- | --- | --- | --- | --- | --- |
| Actual | Predict  |  |  |  |  |  |  |  |  | | --- | --- | --- | --- | --- | --- | --- | --- | |  | 0 | 1 | 2 | 3 | 4 | 5 | 6 | | 0 | 87 | 71 | 83 | 6 | 0 | 4 | 0 | | 1 | 34 | 133 | 58 | 3 | 2 | 2 | 0 | | 2 | 26 | 43 | 135 | 2 | 0 | 8 | 0 | | 3 | 13 | 21 | 31 | 5 | 0 | 1 | 1 | | 4 | 3 | 7 | 12 | 1 | 2 | 7 | 0 | | 5 | 7 | 12 | 7 | 0 | 0 | 5 | 0 | | 6 | 17 | 32 | 26 | 1 | 1 | 8 | 7 | |

## Overall Statistics :

|  |  |
| --- | --- |
| 95% CI | (0.37311,0.43641) |
| ACC Macro | 0.82993 |
| ARI | 0.07003 |
| AUNP | 0.61179 |
| AUNU | 0.58169 |
| Bangdiwala B | 0.21842 |
| Bennett S | 0.30556 |
| CBA | 0.21399 |
| CSI | -0.30266 |
| Chi-Squared | 289.61823 |
| Chi-Squared DF | 36 |
| Conditional Entropy | 1.74038 |
| Cramer V | 0.22856 |
| Cross Entropy | 2.87797 |
| F1 Macro | 0.26683 |
| F1 Micro | 0.40476 |
| FNR Macro | 0.72571 |
| FNR Micro | 0.59524 |
| FPR Macro | 0.11092 |
| FPR Micro | 0.09921 |
| Gwet AC1 | 0.31919 |
| Hamming Loss | 0.59524 |
| Joint Entropy | 4.19108 |
| KL Divergence | 0.42726 |
| Kappa | 0.2232 |
| Kappa 95% CI | (0.1819,0.26451) |
| Kappa No Prevalence | -0.19048 |
| Kappa Standard Error | 0.02107 |
| Kappa Unbiased | 0.21071 |
| Krippendorff Alpha | 0.21114 |
| Lambda A | 0.18871 |
| Lambda B | 0.15734 |
| Mutual Information | 0.17583 |
| NIR | 0.27165 |
| Overall ACC | 0.40476 |
| Overall CEN | 0.5671 |
| Overall J | (1.15247,0.16464) |
| Overall MCC | 0.23101 |
| Overall MCEN | 0.64685 |
| Overall RACC | 0.23373 |
| Overall RACCU | 0.24586 |
| P-Value | 0.0 |
| PPV Macro | 0.42305 |
| PPV Micro | 0.40476 |
| Pearson C | 0.48851 |
| Phi-Squared | 0.31344 |
| RCI | 0.07175 |
| RR | 132.0 |
| Reference Entropy | 2.4507 |
| Response Entropy | 1.91621 |
| SOA1(Landis & Koch) | Fair |
| SOA2(Fleiss) | Poor |
| SOA3(Altman) | Fair |
| SOA4(Cicchetti) | Poor |
| SOA5(Cramer) | Moderate |
| SOA6(Matthews) | Negligible |
| Scott PI | 0.21071 |
| Standard Error | 0.01615 |
| TNR Macro | 0.88908 |
| TNR Micro | 0.90079 |
| TPR Macro | 0.27429 |
| TPR Micro | 0.40476 |
| Zero-one Loss | 550 |

## Class Statistics :

|  |  |  |  |  |  |  |  |  |
| --- | --- | --- | --- | --- | --- | --- | --- | --- |
| Class | 0 | 1 | 2 | 3 | 4 | 5 | 6 | Description |
| ACC | 0.71429 | 0.69156 | 0.67965 | 0.91342 | 0.96429 | 0.93939 | 0.90693 | Accuracy |
| AGF | 0.53757 | 0.65844 | 0.67781 | 0.27671 | 0.27049 | 0.39051 | 0.2933 | Adjusted F-score |
| AGM | 0.67311 | 0.68332 | 0.67597 | 0.60846 | 0.61653 | 0.67573 | 0.61829 | Adjusted geometric mean |
| AM | -64 | 87 | 138 | -54 | -27 | 4 | -84 | Difference between automatic and manual classification |
| AUC | 0.59901 | 0.65224 | 0.6626 | 0.52709 | 0.52957 | 0.56385 | 0.53744 | Area under the ROC curve |
| AUCI | Poor | Fair | Fair | Poor | Poor | Poor | Poor | AUC value interpretation |
| AUPR | 0.40593 | 0.4951 | 0.50718 | 0.17361 | 0.23125 | 0.15207 | 0.47554 | Area under the PR curve |
| BCD | 0.03463 | 0.04708 | 0.07468 | 0.02922 | 0.01461 | 0.00216 | 0.04545 | Bray-Curtis dissimilarity |
| BM | 0.19803 | 0.30449 | 0.32521 | 0.05419 | 0.05914 | 0.1277 | 0.07489 | Informedness or bookmaker informedness |
| CEN | 0.56686 | 0.55121 | 0.55095 | 0.62966 | 0.62449 | 0.75446 | 0.54582 | Confusion entropy |
| DOR | 3.0397 | 3.65472 | 3.88234 | 4.8163 | 19.75556 | 5.53205 | 68.43529 | Diagnostic odds ratio |
| DP | 0.2662 | 0.31032 | 0.32478 | 0.3764 | 0.71435 | 0.40957 | 1.01184 | Discriminant power |
| DPI | Poor | Poor | Poor | Poor | Poor | Poor | Limited | Discriminant power interpretation |
| ERR | 0.28571 | 0.30844 | 0.32035 | 0.08658 | 0.03571 | 0.06061 | 0.09307 | Error rate |
| F0.5 | 0.43544 | 0.44098 | 0.41615 | 0.17361 | 0.19231 | 0.1462 | 0.28226 | F0.5 score |
| F1 | 0.39726 | 0.48276 | 0.47703 | 0.11111 | 0.10811 | 0.15152 | 0.14 | F1 score - harmonic mean of precision and sensitivity |
| F2 | 0.36524 | 0.53328 | 0.55877 | 0.0817 | 0.07519 | 0.15723 | 0.09309 | F2 score |
| FDR | 0.53476 | 0.58307 | 0.61648 | 0.72222 | 0.6 | 0.85714 | 0.125 | False discovery rate |
| FN | 164 | 99 | 79 | 67 | 30 | 26 | 85 | False negative/miss/type 2 error |
| FNR | 0.65339 | 0.42672 | 0.36916 | 0.93056 | 0.9375 | 0.83871 | 0.92391 | Miss rate or false negative rate |
| FOR | 0.22252 | 0.16364 | 0.13811 | 0.07395 | 0.03264 | 0.02925 | 0.09279 | False omission rate |
| FP | 100 | 186 | 217 | 13 | 3 | 30 | 1 | False positive/type 1 error/false alarm |
| FPR | 0.14859 | 0.26879 | 0.30563 | 0.01526 | 0.00336 | 0.03359 | 0.0012 | Fall-out or false positive rate |
| G | 0.40157 | 0.48889 | 0.49188 | 0.13889 | 0.15811 | 0.15179 | 0.25802 | G-measure geometric mean of precision and sensitivity |
| GI | 0.19803 | 0.30449 | 0.32521 | 0.05419 | 0.05914 | 0.1277 | 0.07489 | Gini index |
| GM | 0.54324 | 0.64745 | 0.66184 | 0.2615 | 0.24958 | 0.39481 | 0.27567 | G-mean geometric mean of specificity and sensitivity |
| IBA | 0.14614 | 0.35298 | 0.41021 | 0.00579 | 0.0041 | 0.03038 | 0.00587 | Index of balanced accuracy |
| ICSI | -0.18815 | -0.0098 | 0.01436 | -0.65278 | -0.5375 | -0.69585 | -0.04891 | Individual classification success index |
| IS | 0.77625 | 0.73164 | 0.72767 | 1.83383 | 3.52982 | 2.0902 | 3.13554 | Information score |
| J | 0.24786 | 0.31818 | 0.31323 | 0.05882 | 0.05714 | 0.08197 | 0.07527 | Jaccard index |
| LS | 1.71268 | 1.66052 | 1.65596 | 3.56481 | 11.55 | 4.25806 | 8.78804 | Lift score |
| MCC | 0.21924 | 0.27771 | 0.28251 | 0.10509 | 0.14739 | 0.12045 | 0.24202 | Matthews correlation coefficient |
| MCCI | Negligible | Negligible | Negligible | Negligible | Negligible | Negligible | Negligible | Matthews correlation coefficient interpretation |
| MCEN | 0.64034 | 0.6508 | 0.64821 | 0.64505 | 0.63909 | 0.7872 | 0.55989 | Modified confusion entropy |
| MK | 0.24272 | 0.25329 | 0.24541 | 0.20383 | 0.36736 | 0.11361 | 0.78221 | Markedness |
| N | 673 | 692 | 710 | 852 | 892 | 893 | 832 | Condition negative |
| NLR | 0.76742 | 0.58358 | 0.53165 | 0.94497 | 0.94066 | 0.86787 | 0.92502 | Negative likelihood ratio |
| NLRI | Negligible | Negligible | Negligible | Negligible | Negligible | Negligible | Negligible | Negative likelihood ratio interpretation |
| NPV | 0.77748 | 0.83636 | 0.86189 | 0.92605 | 0.96736 | 0.97075 | 0.90721 | Negative predictive value |
| OC | 0.46524 | 0.57328 | 0.63084 | 0.27778 | 0.4 | 0.16129 | 0.875 | Overlap coefficient |
| OOC | 0.40157 | 0.48889 | 0.49188 | 0.13889 | 0.15811 | 0.15179 | 0.25802 | Otsuka-Ochiai coefficient |
| OP | 0.29293 | 0.57049 | 0.63172 | 0.04517 | 0.08231 | 0.22545 | 0.0485 | Optimized precision |
| P | 251 | 232 | 214 | 72 | 32 | 31 | 92 | Condition positive or support |
| PLR | 2.33271 | 2.13283 | 2.06404 | 4.55128 | 18.58333 | 4.80108 | 63.30435 | Positive likelihood ratio |
| PLRI | Poor | Poor | Poor | Poor | Good | Poor | Good | Positive likelihood ratio interpretation |
| POP | 924 | 924 | 924 | 924 | 924 | 924 | 924 | Population |
| PPV | 0.46524 | 0.41693 | 0.38352 | 0.27778 | 0.4 | 0.14286 | 0.875 | Precision or positive predictive value |
| PRE | 0.27165 | 0.25108 | 0.2316 | 0.07792 | 0.03463 | 0.03355 | 0.09957 | Prevalence |
| Q | 0.50491 | 0.57033 | 0.59036 | 0.65614 | 0.90364 | 0.69382 | 0.9712 | Yule Q - coefficient of colligation |
| QI | Moderate | Moderate | Moderate | Moderate | Strong | Moderate | Strong | Yule Q interpretation |
| RACC | 0.05498 | 0.08668 | 0.08823 | 0.00152 | 0.00019 | 0.00127 | 0.00086 | Random accuracy |
| RACCU | 0.05618 | 0.0889 | 0.09381 | 0.00237 | 0.0004 | 0.00128 | 0.00293 | Random accuracy unbiased |
| TN | 573 | 506 | 493 | 839 | 889 | 863 | 831 | True negative/correct rejection |
| TNR | 0.85141 | 0.73121 | 0.69437 | 0.98474 | 0.99664 | 0.96641 | 0.9988 | Specificity or true negative rate |
| TON | 737 | 605 | 572 | 906 | 919 | 889 | 916 | Test outcome negative |
| TOP | 187 | 319 | 352 | 18 | 5 | 35 | 8 | Test outcome positive |
| TP | 87 | 133 | 135 | 5 | 2 | 5 | 7 | True positive/hit |
| TPR | 0.34661 | 0.57328 | 0.63084 | 0.06944 | 0.0625 | 0.16129 | 0.07609 | Sensitivity, recall, hit rate, or true positive rate |
| Y | 0.19803 | 0.30449 | 0.32521 | 0.05419 | 0.05914 | 0.1277 | 0.07489 | Youden index |
| dInd | 0.67007 | 0.50432 | 0.47926 | 0.93068 | 0.93751 | 0.83938 | 0.92391 | Distance index |
| sInd | 0.52619 | 0.64339 | 0.66111 | 0.34191 | 0.33708 | 0.40647 | 0.34669 | Similarity index |

Generated By PyCM Version 3.2
