# Supplementary material for: TopEC: prediction of Enzyme Commission classes by 3D graph neural networks and localized 3D protein descriptor
Source: Nat Commun. 2025 Mar 20;16:2737. doi: 10.1038/s41467-025-57324-5 (PMC11923149; doi:10.1038/s41467-025-57324-5)
Supplement: Supplementary file 3 — Supplementary Data 1 [file 41467_2025_57324_MOESM3_ESM.zip › Data_S1/table1/mainclass/EnzyNet/full_struc/TopEnzyme_TEMP.html]

PyCM Report


# PyCM Report

## Dataset Type :

- Multi-Class Classification
- Imbalanced

Note 1 : Recommended statistics for this type of classification highlighted in aqua

Note 2 : The recommender system assumes that the input is the result of classification over the whole data rather than just a part of it.
If the confusion matrix is the result of test data classification, the recommendation is not valid.

## Confusion Matrix :

|  |  |  |  |  |  |  |  |  |  |  |  |  |  |  |  |  |  |  |  |  |  |  |  |  |  |  |  |  |  |  |  |  |  |  |  |  |  |  |  |  |  |  |  |  |  |  |  |  |  |  |  |  |  |  |  |  |  |  |  |  |  |  |  |  |  |
| --- | --- | --- | --- | --- | --- | --- | --- | --- | --- | --- | --- | --- | --- | --- | --- | --- | --- | --- | --- | --- | --- | --- | --- | --- | --- | --- | --- | --- | --- | --- | --- | --- | --- | --- | --- | --- | --- | --- | --- | --- | --- | --- | --- | --- | --- | --- | --- | --- | --- | --- | --- | --- | --- | --- | --- | --- | --- | --- | --- | --- | --- | --- | --- | --- | --- |
| Actual | Predict  |  |  |  |  |  |  |  |  | | --- | --- | --- | --- | --- | --- | --- | --- | |  | 0 | 1 | 2 | 3 | 4 | 5 | 6 | | 0 | 90 | 66 | 43 | 10 | 3 | 4 | 0 | | 1 | 45 | 98 | 71 | 10 | 3 | 5 | 0 | | 2 | 31 | 46 | 112 | 7 | 3 | 15 | 0 | | 3 | 14 | 15 | 26 | 11 | 1 | 3 | 2 | | 4 | 4 | 8 | 6 | 2 | 6 | 5 | 1 | | 5 | 9 | 10 | 7 | 1 | 0 | 4 | 0 | | 6 | 25 | 25 | 18 | 5 | 1 | 9 | 9 | |

## Overall Statistics :

|  |  |
| --- | --- |
| 95% CI | (0.33944,0.40296) |
| ACC Macro | 0.82034 |
| ARI | 0.0586 |
| AUNP | 0.59525 |
| AUNU | 0.58001 |
| Bangdiwala B | 0.17313 |
| Bennett S | 0.2664 |
| CBA | 0.24304 |
| CSI | -0.35217 |
| Chi-Squared | 255.43782 |
| Chi-Squared DF | 36 |
| Conditional Entropy | 2.01878 |
| Cramer V | 0.21883 |
| Cross Entropy | 2.68605 |
| F1 Macro | 0.28102 |
| F1 Micro | 0.3712 |
| FNR Macro | 0.72435 |
| FNR Micro | 0.6288 |
| FPR Macro | 0.11564 |
| FPR Micro | 0.1048 |
| Gwet AC1 | 0.27824 |
| Hamming Loss | 0.6288 |
| Joint Entropy | 4.48888 |
| KL Divergence | 0.21596 |
| Kappa | 0.19082 |
| Kappa 95% CI | (0.14995,0.23169) |
| Kappa No Prevalence | -0.25759 |
| Kappa Standard Error | 0.02085 |
| Kappa Unbiased | 0.18633 |
| Krippendorff Alpha | 0.18678 |
| Lambda A | 0.16591 |
| Lambda B | 0.14191 |
| Mutual Information | 0.15764 |
| NIR | 0.26097 |
| Overall ACC | 0.3712 |
| Overall CEN | 0.62751 |
| Overall J | (1.18895,0.16985) |
| Overall MCC | 0.19306 |
| Overall MCEN | 0.70918 |
| Overall RACC | 0.22292 |
| Overall RACCU | 0.22721 |
| P-Value | 0.0 |
| PPV Macro | 0.37218 |
| PPV Micro | 0.3712 |
| Pearson C | 0.47244 |
| Phi-Squared | 0.28733 |
| RCI | 0.06382 |
| RR | 127.0 |
| Reference Entropy | 2.4701 |
| Response Entropy | 2.17642 |
| SOA1(Landis & Koch) | Slight |
| SOA2(Fleiss) | Poor |
| SOA3(Altman) | Poor |
| SOA4(Cicchetti) | Poor |
| SOA5(Cramer) | Moderate |
| SOA6(Matthews) | Negligible |
| Scott PI | 0.18633 |
| Standard Error | 0.0162 |
| TNR Macro | 0.88436 |
| TNR Micro | 0.8952 |
| TPR Macro | 0.27565 |
| TPR Micro | 0.3712 |
| Zero-one Loss | 559 |

## Class Statistics :

|  |  |  |  |  |  |  |  |  |
| --- | --- | --- | --- | --- | --- | --- | --- | --- |
| Class | 0 | 1 | 2 | 3 | 4 | 5 | 6 | Description |
| ACC | 0.71429 | 0.65804 | 0.69291 | 0.89201 | 0.95838 | 0.92351 | 0.90326 | Accuracy |
| AGF | 0.58103 | 0.56357 | 0.6323 | 0.39205 | 0.4488 | 0.3379 | 0.33046 | Adjusted F-score |
| AGM | 0.67951 | 0.63677 | 0.67758 | 0.65765 | 0.70359 | 0.64603 | 0.63555 | Adjusted geometric mean |
| AM | 2 | 36 | 69 | -26 | -15 | 14 | -80 | Difference between automatic and manual classification |
| AUC | 0.61324 | 0.58183 | 0.63502 | 0.55497 | 0.58733 | 0.54062 | 0.54703 | Area under the ROC curve |
| AUCI | Fair | Poor | Fair | Poor | Poor | Poor | Poor | AUC value interpretation |
| AUPR | 0.41476 | 0.39404 | 0.45956 | 0.19595 | 0.27022 | 0.10896 | 0.42391 | Area under the PR curve |
| BCD | 0.00112 | 0.02025 | 0.03881 | 0.01462 | 0.00844 | 0.00787 | 0.04499 | Bray-Curtis dissimilarity |
| BM | 0.22647 | 0.16366 | 0.27003 | 0.10994 | 0.17466 | 0.08125 | 0.09406 | Informedness or bookmaker informedness |
| CEN | 0.60452 | 0.61776 | 0.59056 | 0.75684 | 0.75349 | 0.80876 | 0.60844 | Confusion entropy |
| DOR | 3.04129 | 2.09508 | 3.23633 | 4.02904 | 17.74825 | 2.95212 | 28.6988 | Diagnostic odds ratio |
| DP | 0.26632 | 0.17709 | 0.28121 | 0.33367 | 0.6887 | 0.2592 | 0.80376 | Discriminant power |
| DPI | Poor | Poor | Poor | Poor | Poor | Poor | Poor | Discriminant power interpretation |
| ERR | 0.28571 | 0.34196 | 0.30709 | 0.10799 | 0.04162 | 0.07649 | 0.09674 | Error rate |
| F0.5 | 0.4136 | 0.37577 | 0.41605 | 0.21484 | 0.3 | 0.09479 | 0.32143 | F0.5 score |
| F1 | 0.41475 | 0.392 | 0.4507 | 0.18644 | 0.2449 | 0.10526 | 0.17308 | F1 score - harmonic mean of precision and sensitivity |
| F2 | 0.4159 | 0.4097 | 0.49166 | 0.16467 | 0.2069 | 0.11834 | 0.11842 | F2 score |
| FDR | 0.58716 | 0.63433 | 0.60424 | 0.76087 | 0.64706 | 0.91111 | 0.25 | False discovery rate |
| FN | 126 | 134 | 102 | 61 | 26 | 27 | 83 | False negative/miss/type 2 error |
| FNR | 0.58333 | 0.57759 | 0.47664 | 0.84722 | 0.8125 | 0.87097 | 0.90217 | Miss rate or false negative rate |
| FOR | 0.18778 | 0.21578 | 0.16832 | 0.07236 | 0.02982 | 0.03199 | 0.09464 | False omission rate |
| FP | 128 | 170 | 171 | 35 | 11 | 41 | 3 | False positive/type 1 error/false alarm |
| FPR | 0.19019 | 0.25875 | 0.25333 | 0.04284 | 0.01284 | 0.04779 | 0.00376 | Fall-out or false positive rate |
| G | 0.41475 | 0.39302 | 0.45511 | 0.19114 | 0.25725 | 0.1071 | 0.27087 | G-measure geometric mean of precision and sensitivity |
| GI | 0.22647 | 0.16366 | 0.27003 | 0.10994 | 0.17466 | 0.08125 | 0.09406 | Gini index |
| GM | 0.58088 | 0.55957 | 0.62512 | 0.3824 | 0.43022 | 0.35052 | 0.31218 | G-mean geometric mean of specificity and sensitivity |
| IBA | 0.20477 | 0.21328 | 0.30352 | 0.02861 | 0.03708 | 0.02172 | 0.0099 | Index of balanced accuracy |
| ICSI | -0.17049 | -0.21191 | -0.08088 | -0.60809 | -0.45956 | -0.78208 | -0.15217 | Individual classification success index |
| IS | 0.76482 | 0.48668 | 0.71727 | 1.56198 | 3.29354 | 1.34999 | 2.85744 | Information score |
| J | 0.26163 | 0.24378 | 0.29091 | 0.1028 | 0.13953 | 0.05556 | 0.09474 | Jaccard index |
| LS | 1.69916 | 1.40122 | 1.64407 | 2.9526 | 9.80515 | 2.5491 | 7.24728 | Lift score |
| MCC | 0.22577 | 0.15662 | 0.24782 | 0.1354 | 0.23757 | 0.06799 | 0.24828 | Matthews correlation coefficient |
| MCCI | Negligible | Negligible | Negligible | Negligible | Negligible | Negligible | Negligible | Matthews correlation coefficient interpretation |
| MCEN | 0.69362 | 0.70196 | 0.6895 | 0.79931 | 0.8134 | 0.83314 | 0.6331 | Modified confusion entropy |
| MK | 0.22506 | 0.14989 | 0.22744 | 0.16677 | 0.32312 | 0.0569 | 0.65536 | Markedness |
| N | 673 | 657 | 675 | 817 | 857 | 858 | 797 | Condition negative |
| NLR | 0.72034 | 0.77921 | 0.63835 | 0.88514 | 0.82306 | 0.91468 | 0.90558 | Negative likelihood ratio |
| NLRI | Negligible | Negligible | Negligible | Negligible | Negligible | Negligible | Negligible | Negative likelihood ratio interpretation |
| NPV | 0.81222 | 0.78422 | 0.83168 | 0.92764 | 0.97018 | 0.96801 | 0.90536 | Negative predictive value |
| OC | 0.41667 | 0.42241 | 0.52336 | 0.23913 | 0.35294 | 0.12903 | 0.75 | Overlap coefficient |
| OOC | 0.41475 | 0.39302 | 0.45511 | 0.19114 | 0.25725 | 0.1071 | 0.27087 | Otsuka-Ochiai coefficient |
| OP | 0.39374 | 0.38405 | 0.51709 | 0.1673 | 0.27762 | 0.16218 | 0.08209 | Optimized precision |
| P | 216 | 232 | 214 | 72 | 32 | 31 | 92 | Condition positive or support |
| PLR | 2.19076 | 1.63251 | 2.06591 | 3.56627 | 14.60795 | 2.70024 | 25.98913 | Positive likelihood ratio |
| PLRI | Poor | Poor | Poor | Poor | Good | Poor | Good | Positive likelihood ratio interpretation |
| POP | 889 | 889 | 889 | 889 | 889 | 889 | 889 | Population |
| PPV | 0.41284 | 0.36567 | 0.39576 | 0.23913 | 0.35294 | 0.08889 | 0.75 | Precision or positive predictive value |
| PRE | 0.24297 | 0.26097 | 0.24072 | 0.08099 | 0.036 | 0.03487 | 0.10349 | Prevalence |
| Q | 0.50511 | 0.35381 | 0.52789 | 0.60231 | 0.89332 | 0.49394 | 0.93266 | Yule Q - coefficient of colligation |
| QI | Moderate | Weak | Moderate | Moderate | Strong | Weak | Strong | Yule Q interpretation |
| RACC | 0.05958 | 0.07867 | 0.07663 | 0.00419 | 0.00069 | 0.00177 | 0.0014 | Random accuracy |
| RACCU | 0.05958 | 0.07908 | 0.07814 | 0.0044 | 0.00076 | 0.00183 | 0.00342 | Random accuracy unbiased |
| TN | 545 | 487 | 504 | 782 | 846 | 817 | 794 | True negative/correct rejection |
| TNR | 0.80981 | 0.74125 | 0.74667 | 0.95716 | 0.98716 | 0.95221 | 0.99624 | Specificity or true negative rate |
| TON | 671 | 621 | 606 | 843 | 872 | 844 | 877 | Test outcome negative |
| TOP | 218 | 268 | 283 | 46 | 17 | 45 | 12 | Test outcome positive |
| TP | 90 | 98 | 112 | 11 | 6 | 4 | 9 | True positive/hit |
| TPR | 0.41667 | 0.42241 | 0.52336 | 0.15278 | 0.1875 | 0.12903 | 0.09783 | Sensitivity, recall, hit rate, or true positive rate |
| Y | 0.22647 | 0.16366 | 0.27003 | 0.10994 | 0.17466 | 0.08125 | 0.09406 | Youden index |
| dInd | 0.61356 | 0.6329 | 0.53978 | 0.8483 | 0.8126 | 0.87228 | 0.90218 | Distance index |
| sInd | 0.56615 | 0.55247 | 0.61832 | 0.40016 | 0.4254 | 0.38321 | 0.36206 | Similarity index |

Generated By PyCM Version 3.2
