# Supplementary material for: TopEC: prediction of Enzyme Commission classes by 3D graph neural networks and localized 3D protein descriptor
Source: Nat Commun. 2025 Mar 20;16:2737. doi: 10.1038/s41467-025-57324-5 (PMC11923149; doi:10.1038/s41467-025-57324-5)
Supplement: Supplementary file 3 — Supplementary Data 1 [file 41467_2025_57324_MOESM3_ESM.zip › Data_S1/table1/mainclass/EnzyNet/full_struc/TopEnzyme_FOLD_wflips.html]

PyCM Report


# PyCM Report

## Dataset Type :

- Multi-Class Classification
- Imbalanced

Note 1 : Recommended statistics for this type of classification highlighted in aqua

Note 2 : The recommender system assumes that the input is the result of classification over the whole data rather than just a part of it.
If the confusion matrix is the result of test data classification, the recommendation is not valid.

## Confusion Matrix :

|  |  |  |  |  |  |  |  |  |  |  |  |  |  |  |  |  |  |  |  |  |  |  |  |  |  |  |  |  |  |  |  |  |  |  |  |  |  |  |  |  |  |  |  |  |  |  |  |  |  |  |  |  |  |  |  |  |  |  |  |  |  |  |  |  |  |
| --- | --- | --- | --- | --- | --- | --- | --- | --- | --- | --- | --- | --- | --- | --- | --- | --- | --- | --- | --- | --- | --- | --- | --- | --- | --- | --- | --- | --- | --- | --- | --- | --- | --- | --- | --- | --- | --- | --- | --- | --- | --- | --- | --- | --- | --- | --- | --- | --- | --- | --- | --- | --- | --- | --- | --- | --- | --- | --- | --- | --- | --- | --- | --- | --- | --- |
| Actual | Predict  |  |  |  |  |  |  |  |  | | --- | --- | --- | --- | --- | --- | --- | --- | |  | 0 | 1 | 2 | 3 | 4 | 5 | 6 | | 0 | 69 | 62 | 38 | 1 | 0 | 0 | 0 | | 1 | 32 | 165 | 45 | 2 | 0 | 1 | 0 | | 2 | 12 | 79 | 84 | 2 | 1 | 1 | 0 | | 3 | 7 | 46 | 18 | 11 | 0 | 0 | 1 | | 4 | 7 | 41 | 20 | 13 | 0 | 3 | 1 | | 5 | 9 | 55 | 22 | 3 | 0 | 3 | 1 | | 6 | 0 | 36 | 1 | 0 | 0 | 0 | 1 | |

## Overall Statistics :

|  |  |
| --- | --- |
| 95% CI | (0.34118,0.40462) |
| ACC Macro | 0.82083 |
| ARI | 0.04413 |
| AUNP | 0.58822 |
| AUNU | 0.56544 |
| Bangdiwala B | 0.21044 |
| Bennett S | 0.26838 |
| CBA | 0.18662 |
| CSI | -0.43926 |
| Chi-Squared | 271.16883 |
| Chi-Squared DF | 36 |
| Conditional Entropy | 1.48359 |
| Cramer V | 0.22497 |
| Cross Entropy | 3.57399 |
| F1 Macro | 0.23068 |
| F1 Micro | 0.3729 |
| FNR Macro | 0.75147 |
| FNR Micro | 0.6271 |
| FPR Macro | 0.11765 |
| FPR Micro | 0.10452 |
| Gwet AC1 | 0.28442 |
| Hamming Loss | 0.6271 |
| Joint Entropy | 4.09108 |
| KL Divergence | 0.9665 |
| Kappa | 0.18194 |
| Kappa 95% CI | (0.14056,0.22332) |
| Kappa No Prevalence | -0.2542 |
| Kappa Standard Error | 0.02111 |
| Kappa Unbiased | 0.15473 |
| Krippendorff Alpha | 0.1552 |
| Lambda A | 0.14043 |
| Lambda B | 0.02934 |
| Mutual Information | 0.19068 |
| NIR | 0.27436 |
| Overall ACC | 0.3729 |
| Overall CEN | 0.55035 |
| Overall J | (1.00453,0.1435) |
| Overall MCC | 0.19641 |
| Overall MCEN | 0.61728 |
| Overall RACC | 0.23343 |
| Overall RACCU | 0.25811 |
| P-Value | 0.0 |
| PPV Macro | 0.3122 |
| PPV Micro | 0.3729 |
| Pearson C | 0.48263 |
| Phi-Squared | 0.30366 |
| RCI | 0.07313 |
| RR | 127.57143 |
| Reference Entropy | 2.60748 |
| Response Entropy | 1.67427 |
| SOA1(Landis & Koch) | Slight |
| SOA2(Fleiss) | Poor |
| SOA3(Altman) | Poor |
| SOA4(Cicchetti) | Poor |
| SOA5(Cramer) | Moderate |
| SOA6(Matthews) | Negligible |
| Scott PI | 0.15473 |
| Standard Error | 0.01618 |
| TNR Macro | 0.88235 |
| TNR Micro | 0.89548 |
| TPR Macro | 0.24853 |
| TPR Micro | 0.3729 |
| Zero-one Loss | 560 |

## Class Statistics :

|  |  |  |  |  |  |  |  |  |
| --- | --- | --- | --- | --- | --- | --- | --- | --- |
| Class | 0 | 1 | 2 | 3 | 4 | 5 | 6 | Description |
| ACC | 0.81187 | 0.55319 | 0.73236 | 0.89586 | 0.9037 | 0.89362 | 0.95521 | Accuracy |
| AGF | 0.60803 | 0.63708 | 0.61304 | 0.37433 | 0.0 | 0.19014 | 0.17594 | Adjusted F-score |
| AGM | 0.74129 | 0.55236 | 0.69482 | 0.6517 | 0 | 0.56402 | 0.57014 | Adjusted geometric mean |
| AM | -34 | 239 | 49 | -51 | -84 | -85 | -34 | Difference between automatic and manual classification |
| AUC | 0.65661 | 0.59059 | 0.6338 | 0.5533 | 0.49938 | 0.513 | 0.5114 | Area under the ROC curve |
| AUCI | Fair | Poor | Fair | Poor | Poor | Poor | Poor | AUC value interpretation |
| AUPR | 0.45662 | 0.50719 | 0.41885 | 0.23814 | 0.0 | 0.20363 | 0.13816 | Area under the PR curve |
| BCD | 0.01904 | 0.13382 | 0.02744 | 0.02856 | 0.04703 | 0.04759 | 0.01904 | Bray-Curtis dissimilarity |
| BM | 0.31321 | 0.18119 | 0.26759 | 0.1066 | -0.00124 | 0.02601 | 0.02281 | Informedness or bookmaker informedness |
| CEN | 0.49937 | 0.58937 | 0.56384 | 0.56025 | 0.56453 | 0.49273 | 0.19642 | Confusion entropy |
| DOR | 6.68893 | 2.12716 | 3.5 | 5.74008 | 0.0 | 5.3 | 7.67568 | Diagnostic odds ratio |
| DP | 0.45504 | 0.18073 | 0.29996 | 0.41841 | None | 0.39931 | 0.48799 | Discriminant power |
| DPI | Poor | Poor | Poor | Poor | None | Poor | Poor | Discriminant power interpretation |
| ERR | 0.18813 | 0.44681 | 0.26764 | 0.10414 | 0.0963 | 0.10638 | 0.04479 | Error rate |
| F0.5 | 0.48319 | 0.37827 | 0.38497 | 0.26066 | 0.0 | 0.12 | 0.09259 | F0.5 score |
| F1 | 0.45098 | 0.45267 | 0.41278 | 0.1913 | 0.0 | 0.05941 | 0.04762 | F1 score - harmonic mean of precision and sensitivity |
| F2 | 0.42279 | 0.56352 | 0.44492 | 0.1511 | 0.0 | 0.03947 | 0.03205 | F2 score |
| FDR | 0.49265 | 0.65909 | 0.63158 | 0.65625 | 1.0 | 0.625 | 0.75 | False discovery rate |
| FN | 101 | 80 | 95 | 72 | 85 | 90 | 37 | False negative/miss/type 2 error |
| FNR | 0.59412 | 0.32653 | 0.53073 | 0.86747 | 1.0 | 0.96774 | 0.97368 | Miss rate or false negative rate |
| FOR | 0.13342 | 0.1956 | 0.14286 | 0.08362 | 0.09529 | 0.10169 | 0.04162 | False omission rate |
| FP | 67 | 319 | 144 | 21 | 1 | 5 | 3 | False positive/type 1 error/false alarm |
| FPR | 0.09267 | 0.49228 | 0.20168 | 0.02593 | 0.00124 | 0.00625 | 0.00351 | Fall-out or false positive rate |
| G | 0.45379 | 0.47916 | 0.4158 | 0.21344 | 0.0 | 0.10999 | 0.08111 | G-measure geometric mean of precision and sensitivity |
| GI | 0.31321 | 0.18119 | 0.26759 | 0.1066 | -0.00124 | 0.02601 | 0.02281 | Gini index |
| GM | 0.60685 | 0.58475 | 0.61207 | 0.3593 | 0.0 | 0.17904 | 0.16194 | G-mean geometric mean of specificity and sensitivity |
| IBA | 0.1836 | 0.39861 | 0.25136 | 0.02046 | 0.0 | 0.00123 | 0.00078 | Index of balanced accuracy |
| ICSI | -0.08676 | 0.01438 | -0.16231 | -0.52372 | -1.0 | -0.59274 | -0.72368 | Individual classification success index |
| IS | 1.41419 | 0.31334 | 0.87813 | 1.88691 | None | 1.84832 | 2.55459 | Information score |
| J | 0.29114 | 0.29255 | 0.26006 | 0.10577 | 0.0 | 0.03061 | 0.02439 | Jaccard index |
| LS | 2.6651 | 1.24258 | 1.83799 | 3.69842 | 0.0 | 3.60081 | 5.875 | Lift score |
| MCC | 0.34223 | 0.16226 | 0.24568 | 0.16652 | -0.01086 | 0.08431 | 0.06894 | Matthews correlation coefficient |
| MCCI | Weak | Negligible | Negligible | Negligible | Negligible | Negligible | Negligible | Matthews correlation coefficient interpretation |
| MCEN | 0.57186 | 0.68873 | 0.64164 | 0.58333 | 0.56453 | 0.49605 | 0.19175 | Modified confusion entropy |
| MK | 0.37393 | 0.14531 | 0.22556 | 0.26013 | -0.09529 | 0.27331 | 0.20838 | Markedness |
| N | 723 | 648 | 714 | 810 | 808 | 800 | 855 | Condition negative |
| NLR | 0.6548 | 0.64314 | 0.6648 | 0.89056 | 1.00124 | 0.97383 | 0.97711 | Negative likelihood ratio |
| NLRI | Negligible | Negligible | Negligible | Negligible | Negligible | Negligible | Negligible | Negative likelihood ratio interpretation |
| NPV | 0.86658 | 0.8044 | 0.85714 | 0.91638 | 0.90471 | 0.89831 | 0.95838 | Negative predictive value |
| OC | 0.50735 | 0.67347 | 0.46927 | 0.34375 | 0.0 | 0.375 | 0.25 | Overlap coefficient |
| OOC | 0.45379 | 0.47916 | 0.4158 | 0.21344 | 0.0 | 0.10999 | 0.08111 | Otsuka-Ochiai coefficient |
| OP | 0.43002 | 0.41286 | 0.47278 | 0.13538 | -0.0963 | -0.0435 | 0.00667 | Optimized precision |
| P | 170 | 245 | 179 | 83 | 85 | 93 | 38 | Condition positive or support |
| PLR | 4.37989 | 1.36805 | 2.32682 | 5.11188 | 0.0 | 5.16129 | 7.5 | Positive likelihood ratio |
| PLRI | Poor | Poor | Poor | Fair | Negligible | Fair | Fair | Positive likelihood ratio interpretation |
| POP | 893 | 893 | 893 | 893 | 893 | 893 | 893 | Population |
| PPV | 0.50735 | 0.34091 | 0.36842 | 0.34375 | 0.0 | 0.375 | 0.25 | Precision or positive predictive value |
| PRE | 0.19037 | 0.27436 | 0.20045 | 0.09295 | 0.09518 | 0.10414 | 0.04255 | Prevalence |
| Q | 0.73989 | 0.36044 | 0.55556 | 0.70327 | -1.0 | 0.68254 | 0.76947 | Yule Q - coefficient of colligation |
| QI | Moderate | Weak | Moderate | Moderate | Negligible | Moderate | Strong | Yule Q interpretation |
| RACC | 0.02899 | 0.1487 | 0.05118 | 0.00333 | 0.00011 | 0.00093 | 0.00019 | Random accuracy |
| RACCU | 0.02935 | 0.16661 | 0.05193 | 0.00415 | 0.00232 | 0.0032 | 0.00055 | Random accuracy unbiased |
| TN | 656 | 329 | 570 | 789 | 807 | 795 | 852 | True negative/correct rejection |
| TNR | 0.90733 | 0.50772 | 0.79832 | 0.97407 | 0.99876 | 0.99375 | 0.99649 | Specificity or true negative rate |
| TON | 757 | 409 | 665 | 861 | 892 | 885 | 889 | Test outcome negative |
| TOP | 136 | 484 | 228 | 32 | 1 | 8 | 4 | Test outcome positive |
| TP | 69 | 165 | 84 | 11 | 0 | 3 | 1 | True positive/hit |
| TPR | 0.40588 | 0.67347 | 0.46927 | 0.13253 | 0.0 | 0.03226 | 0.02632 | Sensitivity, recall, hit rate, or true positive rate |
| Y | 0.31321 | 0.18119 | 0.26759 | 0.1066 | -0.00124 | 0.02601 | 0.02281 | Youden index |
| dInd | 0.6013 | 0.59073 | 0.56775 | 0.86786 | 1.0 | 0.96776 | 0.97369 | Distance index |
| sInd | 0.57482 | 0.58229 | 0.59854 | 0.38633 | 0.29289 | 0.31569 | 0.3115 | Similarity index |

Generated By PyCM Version 3.2
