# Supplementary material for: TopEC: prediction of Enzyme Commission classes by 3D graph neural networks and localized 3D protein descriptor
Source: Nat Commun. 2025 Mar 20;16:2737. doi: 10.1038/s41467-025-57324-5 (PMC11923149; doi:10.1038/s41467-025-57324-5)
Supplement: Supplementary file 3 — Supplementary Data 1 [file 41467_2025_57324_MOESM3_ESM.zip › Data_S1/table1/mainclass/EnzyNet/full_struc/TopEnzyme_FOLD_flips.html]

PyCM Report


# PyCM Report

## Dataset Type :

- Multi-Class Classification
- Imbalanced

Note 1 : Recommended statistics for this type of classification highlighted in aqua

Note 2 : The recommender system assumes that the input is the result of classification over the whole data rather than just a part of it.
If the confusion matrix is the result of test data classification, the recommendation is not valid.

## Confusion Matrix :

|  |  |  |  |  |  |  |  |  |  |  |  |  |  |  |  |  |  |  |  |  |  |  |  |  |  |  |  |  |  |  |  |  |  |  |  |  |  |  |  |  |  |  |  |  |  |  |  |  |  |  |  |  |  |  |  |  |  |  |  |  |  |  |  |  |  |
| --- | --- | --- | --- | --- | --- | --- | --- | --- | --- | --- | --- | --- | --- | --- | --- | --- | --- | --- | --- | --- | --- | --- | --- | --- | --- | --- | --- | --- | --- | --- | --- | --- | --- | --- | --- | --- | --- | --- | --- | --- | --- | --- | --- | --- | --- | --- | --- | --- | --- | --- | --- | --- | --- | --- | --- | --- | --- | --- | --- | --- | --- | --- | --- | --- | --- |
| Actual | Predict  |  |  |  |  |  |  |  |  | | --- | --- | --- | --- | --- | --- | --- | --- | |  | 0 | 1 | 2 | 3 | 4 | 5 | 6 | | 0 | 65 | 60 | 44 | 1 | 0 | 0 | 0 | | 1 | 34 | 165 | 44 | 2 | 0 | 0 | 0 | | 2 | 14 | 70 | 91 | 2 | 1 | 1 | 0 | | 3 | 9 | 44 | 19 | 10 | 0 | 0 | 1 | | 4 | 6 | 47 | 20 | 10 | 0 | 2 | 0 | | 5 | 8 | 55 | 25 | 3 | 0 | 1 | 1 | | 6 | 0 | 36 | 0 | 20 | 0 | 0 | 2 | |

## Overall Statistics :

|  |  |
| --- | --- |
| 95% CI | (0.33458,0.39707) |
| ACC Macro | 0.81881 |
| ARI | 0.05447 |
| AUNP | 0.58865 |
| AUNU | 0.5648 |
| Bangdiwala B | 0.21172 |
| Bennett S | 0.26013 |
| CBA | 0.18121 |
| CSI | -0.44477 |
| Chi-Squared | 337.16694 |
| Chi-Squared DF | 36 |
| Conditional Entropy | 1.48296 |
| Cramer V | 0.24809 |
| Cross Entropy | 3.73487 |
| F1 Macro | 0.22157 |
| F1 Micro | 0.36583 |
| FNR Macro | 0.75287 |
| FNR Micro | 0.63417 |
| FPR Macro | 0.11753 |
| FPR Micro | 0.1057 |
| Gwet AC1 | 0.27512 |
| Hamming Loss | 0.63417 |
| Joint Entropy | 4.12629 |
| KL Divergence | 1.09154 |
| Kappa | 0.18095 |
| Kappa 95% CI | (0.1406,0.2213) |
| Kappa No Prevalence | -0.26835 |
| Kappa Standard Error | 0.02059 |
| Kappa Unbiased | 0.15537 |
| Krippendorff Alpha | 0.15583 |
| Lambda A | 0.1512 |
| Lambda B | 0.05963 |
| Mutual Information | 0.22668 |
| NIR | 0.26835 |
| Overall ACC | 0.36583 |
| Overall CEN | 0.55299 |
| Overall J | (0.96726,0.13818) |
| Overall MCC | 0.19432 |
| Overall MCEN | 0.61914 |
| Overall RACC | 0.22572 |
| Overall RACCU | 0.24917 |
| P-Value | 0.0 |
| PPV Macro | 0.3081 |
| PPV Micro | 0.36583 |
| Pearson C | 0.51932 |
| Phi-Squared | 0.3693 |
| RCI | 0.08575 |
| RR | 130.42857 |
| Reference Entropy | 2.64334 |
| Response Entropy | 1.70964 |
| SOA1(Landis & Koch) | Slight |
| SOA2(Fleiss) | Poor |
| SOA3(Altman) | Poor |
| SOA4(Cicchetti) | Poor |
| SOA5(Cramer) | Moderate |
| SOA6(Matthews) | Negligible |
| Scott PI | 0.15537 |
| Standard Error | 0.01594 |
| TNR Macro | 0.88247 |
| TNR Micro | 0.8943 |
| TPR Macro | 0.24713 |
| TPR Micro | 0.36583 |
| Zero-one Loss | 579 |

## Class Statistics :

|  |  |  |  |  |  |  |  |  |
| --- | --- | --- | --- | --- | --- | --- | --- | --- |
| Class | 0 | 1 | 2 | 3 | 4 | 5 | 6 | Description |
| ACC | 0.80723 | 0.57065 | 0.73713 | 0.87842 | 0.90581 | 0.89595 | 0.93647 | Accuracy |
| AGF | 0.58949 | 0.64643 | 0.63593 | 0.34851 | 0.0 | 0.11041 | 0.2006 | Adjusted F-score |
| AGM | 0.73001 | 0.57114 | 0.70532 | 0.632 | 0 | 0.52597 | 0.57825 | Adjusted geometric mean |
| AM | -34 | 232 | 64 | -35 | -84 | -89 | -54 | Difference between automatic and manual classification |
| AUC | 0.6434 | 0.6032 | 0.65065 | 0.53735 | 0.4994 | 0.50355 | 0.51607 | Area under the ROC curve |
| AUCI | Fair | Fair | Fair | Poor | Poor | Poor | Poor | AUC value interpretation |
| AUPR | 0.43015 | 0.50969 | 0.44143 | 0.16441 | 0.0 | 0.13038 | 0.26724 | Area under the PR curve |
| BCD | 0.01862 | 0.12705 | 0.03505 | 0.01917 | 0.046 | 0.04874 | 0.02957 | Bray-Curtis dissimilarity |
| BM | 0.28679 | 0.2064 | 0.3013 | 0.0747 | -0.00121 | 0.00709 | 0.03214 | Informedness or bookmaker informedness |
| CEN | 0.51445 | 0.58661 | 0.55857 | 0.64485 | 0.50088 | 0.46634 | 0.32748 | Confusion entropy |
| DOR | 5.85915 | 2.35337 | 3.95948 | 2.85508 | 0.0 | 2.96014 | 15.23214 | Diagnostic odds ratio |
| DP | 0.42333 | 0.20492 | 0.3295 | 0.2512 | None | 0.25985 | 0.65209 | Discriminant power |
| DPI | Poor | Poor | Poor | Poor | None | Poor | Poor | Discriminant power interpretation |
| ERR | 0.19277 | 0.42935 | 0.26287 | 0.12158 | 0.09419 | 0.10405 | 0.06353 | Error rate |
| F0.5 | 0.45518 | 0.38319 | 0.39531 | 0.18182 | 0.0 | 0.04587 | 0.13514 | F0.5 score |
| F1 | 0.42484 | 0.45706 | 0.43128 | 0.15267 | 0.0 | 0.02062 | 0.06452 | F1 score - harmonic mean of precision and sensitivity |
| F2 | 0.39828 | 0.56623 | 0.47445 | 0.13158 | 0.0 | 0.0133 | 0.04237 | F2 score |
| FDR | 0.52206 | 0.65409 | 0.62551 | 0.79167 | 1.0 | 0.75 | 0.5 | False discovery rate |
| FN | 105 | 80 | 88 | 73 | 85 | 92 | 56 | False negative/miss/type 2 error |
| FNR | 0.61765 | 0.32653 | 0.49162 | 0.87952 | 1.0 | 0.98925 | 0.96552 | Miss rate or false negative rate |
| FOR | 0.13514 | 0.18349 | 0.13134 | 0.08439 | 0.0932 | 0.10121 | 0.06161 | False omission rate |
| FP | 71 | 312 | 152 | 38 | 1 | 3 | 2 | False positive/type 1 error/false alarm |
| FPR | 0.09556 | 0.46707 | 0.20708 | 0.04578 | 0.00121 | 0.00366 | 0.00234 | Fall-out or false positive rate |
| G | 0.42748 | 0.48266 | 0.43633 | 0.15843 | 0.0 | 0.05185 | 0.13131 | G-measure geometric mean of precision and sensitivity |
| GI | 0.28679 | 0.2064 | 0.3013 | 0.0747 | -0.00121 | 0.00709 | 0.03214 | Gini index |
| GM | 0.58806 | 0.5991 | 0.6349 | 0.33907 | 0.0 | 0.10351 | 0.18548 | G-mean geometric mean of specificity and sensitivity |
| IBA | 0.16527 | 0.40936 | 0.28841 | 0.01911 | 0.0 | 0.00015 | 0.00127 | Index of balanced accuracy |
| ICSI | -0.13971 | 0.01938 | -0.11713 | -0.67118 | -1.0 | -0.73925 | -0.46552 | Individual classification success index |
| IS | 1.35999 | 0.36631 | 0.93364 | 1.1964 | None | 1.29531 | 2.97649 | Information score |
| J | 0.26971 | 0.29623 | 0.27492 | 0.08264 | 0.0 | 0.01042 | 0.03333 | Jaccard index |
| LS | 2.56683 | 1.28905 | 1.91009 | 2.29167 | 0.0 | 2.4543 | 7.87069 | Lift score |
| MCC | 0.31355 | 0.1831 | 0.27066 | 0.09622 | -0.01061 | 0.03249 | 0.11871 | Matthews correlation coefficient |
| MCCI | Weak | Negligible | Negligible | Negligible | Negligible | Negligible | Negligible | Matthews correlation coefficient interpretation |
| MCEN | 0.58303 | 0.6869 | 0.64126 | 0.66883 | 0.50088 | 0.46707 | 0.32564 | Modified confusion entropy |
| MK | 0.34281 | 0.16243 | 0.24314 | 0.12394 | -0.0932 | 0.14879 | 0.43839 | Markedness |
| N | 743 | 668 | 734 | 830 | 828 | 820 | 855 | Condition negative |
| NLR | 0.6829 | 0.6127 | 0.62002 | 0.92172 | 1.00121 | 0.99288 | 0.96778 | Negative likelihood ratio |
| NLRI | Negligible | Negligible | Negligible | Negligible | Negligible | Negligible | Negligible | Negative likelihood ratio interpretation |
| NPV | 0.86486 | 0.81651 | 0.86866 | 0.91561 | 0.9068 | 0.89879 | 0.93839 | Negative predictive value |
| OC | 0.47794 | 0.67347 | 0.50838 | 0.20833 | 0.0 | 0.25 | 0.5 | Overlap coefficient |
| OOC | 0.42748 | 0.48266 | 0.43633 | 0.15843 | 0.0 | 0.05185 | 0.13131 | Otsuka-Ochiai coefficient |
| OP | 0.4015 | 0.45416 | 0.51847 | 0.10264 | -0.09419 | -0.0827 | 0.00329 | Optimized precision |
| P | 170 | 245 | 179 | 83 | 85 | 93 | 58 | Condition positive or support |
| PLR | 4.00124 | 1.44192 | 2.45494 | 2.63158 | 0.0 | 2.93907 | 14.74138 | Positive likelihood ratio |
| PLRI | Poor | Poor | Poor | Poor | Negligible | Poor | Good | Positive likelihood ratio interpretation |
| POP | 913 | 913 | 913 | 913 | 913 | 913 | 913 | Population |
| PPV | 0.47794 | 0.34591 | 0.37449 | 0.20833 | 0.0 | 0.25 | 0.5 | Precision or positive predictive value |
| PRE | 0.1862 | 0.26835 | 0.19606 | 0.09091 | 0.0931 | 0.10186 | 0.06353 | Prevalence |
| Q | 0.70842 | 0.40358 | 0.59673 | 0.4812 | -1.0 | 0.49497 | 0.87679 | Yule Q - coefficient of colligation |
| QI | Moderate | Weak | Moderate | Weak | Negligible | Weak | Strong | Yule Q interpretation |
| RACC | 0.02774 | 0.1402 | 0.05218 | 0.00478 | 0.0001 | 0.00045 | 0.00028 | Random accuracy |
| RACCU | 0.02808 | 0.15634 | 0.05341 | 0.00515 | 0.00222 | 0.00282 | 0.00115 | Random accuracy unbiased |
| TN | 672 | 356 | 582 | 792 | 827 | 817 | 853 | True negative/correct rejection |
| TNR | 0.90444 | 0.53293 | 0.79292 | 0.95422 | 0.99879 | 0.99634 | 0.99766 | Specificity or true negative rate |
| TON | 777 | 436 | 670 | 865 | 912 | 909 | 909 | Test outcome negative |
| TOP | 136 | 477 | 243 | 48 | 1 | 4 | 4 | Test outcome positive |
| TP | 65 | 165 | 91 | 10 | 0 | 1 | 2 | True positive/hit |
| TPR | 0.38235 | 0.67347 | 0.50838 | 0.12048 | 0.0 | 0.01075 | 0.03448 | Sensitivity, recall, hit rate, or true positive rate |
| Y | 0.28679 | 0.2064 | 0.3013 | 0.0747 | -0.00121 | 0.00709 | 0.03214 | Youden index |
| dInd | 0.625 | 0.56989 | 0.53346 | 0.88071 | 1.0 | 0.98925 | 0.96552 | Distance index |
| sInd | 0.55806 | 0.59703 | 0.62279 | 0.37724 | 0.29289 | 0.30049 | 0.31727 | Similarity index |

Generated By PyCM Version 3.2
