# Supplementary material for: TopEC: prediction of Enzyme Commission classes by 3D graph neural networks and localized 3D protein descriptor
Source: Nat Commun. 2025 Mar 20;16:2737. doi: 10.1038/s41467-025-57324-5 (PMC11923149; doi:10.1038/s41467-025-57324-5)
Supplement: Supplementary file 3 — Supplementary Data 1 [file 41467_2025_57324_MOESM3_ESM.zip › Data_S1/table1/mainclass/EnzyNet/full_struc/TopEnzyme_FOLD.html]

PyCM Report


# PyCM Report

## Dataset Type :

- Multi-Class Classification
- Imbalanced

Note 1 : Recommended statistics for this type of classification highlighted in aqua

Note 2 : The recommender system assumes that the input is the result of classification over the whole data rather than just a part of it.
If the confusion matrix is the result of test data classification, the recommendation is not valid.

## Confusion Matrix :

|  |  |  |  |  |  |  |  |  |  |  |  |  |  |  |  |  |  |  |  |  |  |  |  |  |  |  |  |  |  |  |  |  |  |  |  |  |  |  |  |  |  |  |  |  |  |  |  |  |  |  |  |  |  |  |  |  |  |  |  |  |  |  |  |  |  |
| --- | --- | --- | --- | --- | --- | --- | --- | --- | --- | --- | --- | --- | --- | --- | --- | --- | --- | --- | --- | --- | --- | --- | --- | --- | --- | --- | --- | --- | --- | --- | --- | --- | --- | --- | --- | --- | --- | --- | --- | --- | --- | --- | --- | --- | --- | --- | --- | --- | --- | --- | --- | --- | --- | --- | --- | --- | --- | --- | --- | --- | --- | --- | --- | --- | --- |
| Actual | Predict  |  |  |  |  |  |  |  |  | | --- | --- | --- | --- | --- | --- | --- | --- | |  | 0 | 1 | 2 | 3 | 4 | 5 | 6 | | 0 | 60 | 52 | 46 | 10 | 0 | 2 | 0 | | 1 | 39 | 140 | 55 | 6 | 2 | 3 | 0 | | 2 | 14 | 68 | 83 | 8 | 2 | 4 | 0 | | 3 | 16 | 36 | 13 | 14 | 3 | 0 | 1 | | 4 | 13 | 33 | 12 | 20 | 1 | 6 | 0 | | 5 | 10 | 41 | 21 | 7 | 1 | 11 | 2 | | 6 | 4 | 24 | 6 | 2 | 0 | 0 | 2 | |

## Overall Statistics :

|  |  |
| --- | --- |
| 95% CI | (0.31702,0.37951) |
| ACC Macro | 0.81379 |
| ARI | 0.04719 |
| AUNP | 0.58168 |
| AUNU | 0.56448 |
| Bangdiwala B | 0.17455 |
| Bennett S | 0.23964 |
| CBA | 0.20162 |
| CSI | -0.43226 |
| Chi-Squared | 225.4113 |
| Chi-Squared DF | 36 |
| Conditional Entropy | 1.84993 |
| Cramer V | 0.20511 |
| Cross Entropy | 3.01616 |
| F1 Macro | 0.24173 |
| F1 Micro | 0.34826 |
| FNR Macro | 0.75151 |
| FNR Micro | 0.65174 |
| FPR Macro | 0.11952 |
| FPR Micro | 0.10862 |
| Gwet AC1 | 0.25233 |
| Hamming Loss | 0.65174 |
| Joint Entropy | 4.45741 |
| KL Divergence | 0.40868 |
| Kappa | 0.16606 |
| Kappa 95% CI | (0.12608,0.20605) |
| Kappa No Prevalence | -0.30347 |
| Kappa Standard Error | 0.0204 |
| Kappa Unbiased | 0.15341 |
| Krippendorff Alpha | 0.15389 |
| Lambda A | 0.1142 |
| Lambda B | 0.04609 |
| Mutual Information | 0.15562 |
| NIR | 0.27436 |
| Overall ACC | 0.34826 |
| Overall CEN | 0.62475 |
| Overall J | (1.02045,0.14578) |
| Overall MCC | 0.17172 |
| Overall MCEN | 0.69907 |
| Overall RACC | 0.21848 |
| Overall RACCU | 0.23016 |
| P-Value | 0.0 |
| PPV Macro | 0.31925 |
| PPV Micro | 0.34826 |
| Pearson C | 0.44894 |
| Phi-Squared | 0.25242 |
| RCI | 0.05968 |
| RR | 127.57143 |
| Reference Entropy | 2.60748 |
| Response Entropy | 2.00555 |
| SOA1(Landis & Koch) | Slight |
| SOA2(Fleiss) | Poor |
| SOA3(Altman) | Poor |
| SOA4(Cicchetti) | Poor |
| SOA5(Cramer) | Moderate |
| SOA6(Matthews) | Negligible |
| Scott PI | 0.15341 |
| Standard Error | 0.01594 |
| TNR Macro | 0.88048 |
| TNR Micro | 0.89138 |
| TPR Macro | 0.24849 |
| TPR Micro | 0.34826 |
| Zero-one Loss | 582 |

## Class Statistics :

|  |  |  |  |  |  |  |  |  |
| --- | --- | --- | --- | --- | --- | --- | --- | --- |
| Class | 0 | 1 | 2 | 3 | 4 | 5 | 6 | Description |
| ACC | 0.76932 | 0.59798 | 0.72116 | 0.86338 | 0.89698 | 0.89138 | 0.95633 | Accuracy |
| AGF | 0.55358 | 0.61611 | 0.60488 | 0.40176 | 0.11486 | 0.35649 | 0.24813 | Adjusted F-score |
| AGM | 0.69372 | 0.59726 | 0.68451 | 0.6527 | 0.52697 | 0.64337 | 0.60441 | Adjusted geometric mean |
| AM | -14 | 149 | 57 | -16 | -76 | -67 | -33 | Difference between automatic and manual classification |
| AUC | 0.61008 | 0.58973 | 0.6247 | 0.55162 | 0.50093 | 0.54976 | 0.52456 | Area under the ROC curve |
| AUCI | Fair | Poor | Fair | Poor | Poor | Poor | Poor | AUC value interpretation |
| AUPR | 0.36878 | 0.46338 | 0.40769 | 0.18881 | 0.06144 | 0.27068 | 0.22632 | Area under the PR curve |
| BCD | 0.00784 | 0.08343 | 0.03191 | 0.00896 | 0.04255 | 0.03751 | 0.01848 | Bray-Curtis dissimilarity |
| BM | 0.22016 | 0.17945 | 0.2494 | 0.10324 | 0.00186 | 0.09953 | 0.04912 | Informedness or bookmaker informedness |
| CEN | 0.61723 | 0.61409 | 0.60047 | 0.74035 | 0.69655 | 0.63703 | 0.48053 | Confusion entropy |
| DOR | 3.5625 | 2.06824 | 3.17014 | 2.898 | 1.19048 | 7.02033 | 15.77778 | Diagnostic odds ratio |
| DP | 0.3042 | 0.174 | 0.27626 | 0.25477 | 0.04175 | 0.46662 | 0.66052 | Discriminant power |
| DPI | Poor | Poor | Poor | Poor | Poor | Poor | Poor | Discriminant power interpretation |
| ERR | 0.23068 | 0.40202 | 0.27884 | 0.13662 | 0.10302 | 0.10862 | 0.04367 | Error rate |
| F0.5 | 0.37783 | 0.3844 | 0.36955 | 0.19943 | 0.04132 | 0.27919 | 0.17241 | F0.5 score |
| F1 | 0.3681 | 0.43818 | 0.4 | 0.18667 | 0.02128 | 0.18487 | 0.09302 | F1 score - harmonic mean of precision and sensitivity |
| F2 | 0.35885 | 0.50946 | 0.43592 | 0.17544 | 0.01433 | 0.13819 | 0.06369 | F2 score |
| FDR | 0.61538 | 0.64467 | 0.64831 | 0.79104 | 0.88889 | 0.57692 | 0.6 | False discovery rate |
| FN | 110 | 105 | 96 | 69 | 84 | 82 | 36 | False negative/miss/type 2 error |
| FNR | 0.64706 | 0.42857 | 0.53631 | 0.83133 | 0.98824 | 0.88172 | 0.94737 | Miss rate or false negative rate |
| FOR | 0.14925 | 0.21042 | 0.14612 | 0.08354 | 0.09502 | 0.09458 | 0.04054 | False omission rate |
| FP | 96 | 254 | 153 | 53 | 8 | 15 | 3 | False positive/type 1 error/false alarm |
| FPR | 0.13278 | 0.39198 | 0.21429 | 0.06543 | 0.0099 | 0.01875 | 0.00351 | Fall-out or false positive rate |
| G | 0.36844 | 0.45061 | 0.40383 | 0.18774 | 0.03616 | 0.2237 | 0.1451 | G-measure geometric mean of precision and sensitivity |
| GI | 0.22016 | 0.17945 | 0.2494 | 0.10324 | 0.00186 | 0.09953 | 0.04912 | Gini index |
| GM | 0.55324 | 0.58944 | 0.60359 | 0.39704 | 0.10793 | 0.34068 | 0.22901 | G-mean geometric mean of specificity and sensitivity |
| IBA | 0.14867 | 0.33473 | 0.247 | 0.0369 | 0.00025 | 0.0159 | 0.00294 | Index of balanced accuracy |
| ICSI | -0.26244 | -0.07324 | -0.18462 | -0.62237 | -0.87712 | -0.45864 | -0.54737 | Individual classification success index |
| IS | 1.01461 | 0.37311 | 0.8111 | 1.16874 | 0.2232 | 2.02235 | 3.23266 | Information score |
| J | 0.22556 | 0.28056 | 0.25 | 0.10294 | 0.01075 | 0.10185 | 0.04878 | Jaccard index |
| LS | 2.02036 | 1.29514 | 1.75455 | 2.24816 | 1.16732 | 4.06245 | 9.4 | Lift score |
| MCC | 0.22763 | 0.16126 | 0.22643 | 0.11379 | 0.00548 | 0.18082 | 0.13288 | Matthews correlation coefficient |
| MCCI | Negligible | Negligible | Negligible | Negligible | Negligible | Negligible | Negligible | Matthews correlation coefficient interpretation |
| MCEN | 0.69306 | 0.71477 | 0.68324 | 0.78119 | 0.69978 | 0.66686 | 0.48574 | Modified confusion entropy |
| MK | 0.23536 | 0.14491 | 0.20558 | 0.12542 | 0.01609 | 0.3285 | 0.35946 | Markedness |
| N | 723 | 648 | 714 | 810 | 808 | 800 | 855 | Condition negative |
| NLR | 0.74613 | 0.70486 | 0.68258 | 0.88953 | 0.99812 | 0.89857 | 0.9507 | Negative likelihood ratio |
| NLRI | Negligible | Negligible | Negligible | Negligible | Negligible | Negligible | Negligible | Negative likelihood ratio interpretation |
| NPV | 0.85075 | 0.78958 | 0.85388 | 0.91646 | 0.90498 | 0.90542 | 0.95946 | Negative predictive value |
| OC | 0.38462 | 0.57143 | 0.46369 | 0.20896 | 0.11111 | 0.42308 | 0.4 | Overlap coefficient |
| OOC | 0.36844 | 0.45061 | 0.40383 | 0.18774 | 0.03616 | 0.2237 | 0.1451 | Otsuka-Ochiai coefficient |
| OP | 0.34783 | 0.56696 | 0.46342 | 0.16916 | -0.07954 | 0.10652 | 0.05666 | Optimized precision |
| P | 170 | 245 | 179 | 83 | 85 | 93 | 38 | Condition positive or support |
| PLR | 2.65809 | 1.45782 | 2.16387 | 2.57786 | 1.18824 | 6.30824 | 15.0 | Positive likelihood ratio |
| PLRI | Poor | Poor | Poor | Poor | Poor | Fair | Good | Positive likelihood ratio interpretation |
| POP | 893 | 893 | 893 | 893 | 893 | 893 | 893 | Population |
| PPV | 0.38462 | 0.35533 | 0.35169 | 0.20896 | 0.11111 | 0.42308 | 0.4 | Precision or positive predictive value |
| PRE | 0.19037 | 0.27436 | 0.20045 | 0.09295 | 0.09518 | 0.10414 | 0.04255 | Prevalence |
| Q | 0.56164 | 0.34816 | 0.5204 | 0.48692 | 0.08696 | 0.75063 | 0.88079 | Yule Q - coefficient of colligation |
| QI | Moderate | Weak | Moderate | Weak | Negligible | Strong | Strong | Yule Q interpretation |
| RACC | 0.03326 | 0.12105 | 0.05297 | 0.00697 | 0.00096 | 0.00303 | 0.00024 | Random accuracy |
| RACCU | 0.03332 | 0.12801 | 0.05399 | 0.00705 | 0.00277 | 0.00444 | 0.00058 | Random accuracy unbiased |
| TN | 627 | 394 | 561 | 757 | 800 | 785 | 852 | True negative/correct rejection |
| TNR | 0.86722 | 0.60802 | 0.78571 | 0.93457 | 0.9901 | 0.98125 | 0.99649 | Specificity or true negative rate |
| TON | 737 | 499 | 657 | 826 | 884 | 867 | 888 | Test outcome negative |
| TOP | 156 | 394 | 236 | 67 | 9 | 26 | 5 | Test outcome positive |
| TP | 60 | 140 | 83 | 14 | 1 | 11 | 2 | True positive/hit |
| TPR | 0.35294 | 0.57143 | 0.46369 | 0.16867 | 0.01176 | 0.11828 | 0.05263 | Sensitivity, recall, hit rate, or true positive rate |
| Y | 0.22016 | 0.17945 | 0.2494 | 0.10324 | 0.00186 | 0.09953 | 0.04912 | Youden index |
| dInd | 0.66054 | 0.58079 | 0.57754 | 0.8339 | 0.98828 | 0.88192 | 0.94737 | Distance index |
| sInd | 0.53293 | 0.58932 | 0.59162 | 0.41035 | 0.30118 | 0.37639 | 0.3301 | Similarity index |

Generated By PyCM Version 3.2
