# Supplementary material for: TopEC: prediction of Enzyme Commission classes by 3D graph neural networks and localized 3D protein descriptor
Source: Nat Commun. 2025 Mar 20;16:2737. doi: 10.1038/s41467-025-57324-5 (PMC11923149; doi:10.1038/s41467-025-57324-5)
Supplement: Supplementary file 3 — Supplementary Data 1 [file 41467_2025_57324_MOESM3_ESM.zip › Data_S1/table1/mainclass/EnzyNet/full_struc/Combined_TEMP_wflips.html]

PyCM Report


# PyCM Report

## Dataset Type :

- Multi-Class Classification
- Imbalanced

Note 1 : Recommended statistics for this type of classification highlighted in aqua

Note 2 : The recommender system assumes that the input is the result of classification over the whole data rather than just a part of it.
If the confusion matrix is the result of test data classification, the recommendation is not valid.

## Confusion Matrix :

|  |  |  |  |  |  |  |  |  |  |  |  |  |  |  |  |  |  |  |  |  |  |  |  |  |  |  |  |  |  |  |  |  |  |  |  |  |  |  |  |  |  |  |  |  |  |  |  |  |  |  |  |  |  |  |  |  |  |  |  |  |  |  |  |  |  |
| --- | --- | --- | --- | --- | --- | --- | --- | --- | --- | --- | --- | --- | --- | --- | --- | --- | --- | --- | --- | --- | --- | --- | --- | --- | --- | --- | --- | --- | --- | --- | --- | --- | --- | --- | --- | --- | --- | --- | --- | --- | --- | --- | --- | --- | --- | --- | --- | --- | --- | --- | --- | --- | --- | --- | --- | --- | --- | --- | --- | --- | --- | --- | --- | --- | --- |
| Actual | Predict  |  |  |  |  |  |  |  |  | | --- | --- | --- | --- | --- | --- | --- | --- | |  | 0 | 1 | 2 | 3 | 4 | 5 | 6 | | 0 | 407 | 113 | 112 | 7 | 0 | 0 | 0 | | 1 | 75 | 676 | 224 | 7 | 0 | 0 | 4 | | 2 | 118 | 101 | 647 | 0 | 1 | 2 | 15 | | 3 | 35 | 31 | 64 | 84 | 1 | 0 | 0 | | 4 | 7 | 32 | 21 | 1 | 48 | 0 | 0 | | 5 | 22 | 24 | 21 | 0 | 0 | 22 | 0 | | 6 | 31 | 65 | 63 | 2 | 1 | 2 | 4 | |

## Overall Statistics :

|  |  |
| --- | --- |
| 95% CI | (0.59381,0.62819) |
| ACC Macro | 0.88886 |
| ARI | 0.25657 |
| AUNP | 0.73243 |
| AUNU | 0.68724 |
| Bangdiwala B | 0.41655 |
| Bennett S | 0.54617 |
| CBA | 0.41401 |
| CSI | 0.10649 |
| Chi-Squared | 4424.74941 |
| Chi-Squared DF | 36 |
| Conditional Entropy | 1.37326 |
| Cramer V | 0.48853 |
| Cross Entropy | 2.48795 |
| F1 Macro | 0.49551 |
| F1 Micro | 0.611 |
| FNR Macro | 0.54907 |
| FNR Micro | 0.389 |
| FPR Macro | 0.07645 |
| FPR Micro | 0.06483 |
| Gwet AC1 | 0.5569 |
| Hamming Loss | 0.389 |
| Joint Entropy | 3.6994 |
| KL Divergence | 0.16181 |
| Kappa | 0.47127 |
| Kappa 95% CI | (0.44791,0.49464) |
| Kappa No Prevalence | 0.22201 |
| Kappa Standard Error | 0.01192 |
| Kappa Unbiased | 0.46902 |
| Krippendorff Alpha | 0.4691 |
| Lambda A | 0.43394 |
| Lambda B | 0.41228 |
| Mutual Information | 0.54007 |
| NIR | 0.31909 |
| Overall ACC | 0.611 |
| Overall CEN | 0.42598 |
| Overall J | (2.45344,0.35049) |
| Overall MCC | 0.47584 |
| Overall MCEN | 0.5351 |
| Overall RACC | 0.26428 |
| Overall RACCU | 0.2674 |
| P-Value | None |
| PPV Macro | 0.65556 |
| PPV Micro | 0.611 |
| Pearson C | 0.76734 |
| Phi-Squared | 1.43196 |
| RCI | 0.23218 |
| RR | 441.42857 |
| Reference Entropy | 2.32613 |
| Response Entropy | 1.91333 |
| SOA1(Landis & Koch) | Moderate |
| SOA2(Fleiss) | Intermediate to Good |
| SOA3(Altman) | Moderate |
| SOA4(Cicchetti) | Fair |
| SOA5(Cramer) | Relatively Strong |
| SOA6(Matthews) | Weak |
| Scott PI | 0.46902 |
| Standard Error | 0.00877 |
| TNR Macro | 0.92355 |
| TNR Micro | 0.93517 |
| TPR Macro | 0.45093 |
| TPR Micro | 0.611 |
| Zero-one Loss | 1202 |

## Class Statistics :

|  |  |  |  |  |  |  |  |  |
| --- | --- | --- | --- | --- | --- | --- | --- | --- |
| Class | 0 | 1 | 2 | 3 | 4 | 5 | 6 | Description |
| ACC | 0.83172 | 0.78123 | 0.75987 | 0.9521 | 0.97929 | 0.97702 | 0.94078 | Accuracy |
| AGF | 0.75013 | 0.75641 | 0.76771 | 0.64892 | 0.69626 | 0.53181 | 0.16583 | Adjusted F-score |
| AGM | 0.80846 | 0.78232 | 0.7595 | 0.80196 | 0.82812 | 0.74409 | 0.56192 | Adjusted geometric mean |
| AM | 56 | 56 | 268 | -114 | -58 | -63 | -145 | Difference between automatic and manual classification |
| AUC | 0.75971 | 0.75582 | 0.75149 | 0.69239 | 0.71968 | 0.62293 | 0.50865 | Area under the ROC curve |
| AUCI | Good | Good | Good | Fair | Good | Fair | Poor | AUC value interpretation |
| AUPR | 0.61127 | 0.66718 | 0.64677 | 0.61119 | 0.69077 | 0.54667 | 0.09886 | Area under the PR curve |
| BCD | 0.00906 | 0.00906 | 0.04337 | 0.01845 | 0.00939 | 0.01019 | 0.02346 | Bray-Curtis dissimilarity |
| BM | 0.51943 | 0.51164 | 0.50298 | 0.38478 | 0.43936 | 0.24586 | 0.01731 | Informedness or bookmaker informedness |
| CEN | 0.44235 | 0.40226 | 0.43214 | 0.41537 | 0.34295 | 0.4406 | 0.57609 | Confusion entropy |
| DOR | 13.1756 | 10.35509 | 9.19536 | 107.80063 | 781.11475 | 246.02239 | 3.72657 | Diagnostic odds ratio |
| DP | 0.61736 | 0.55968 | 0.53124 | 1.12064 | 1.59484 | 1.31821 | 0.31498 | Discriminant power |
| DPI | Poor | Poor | Poor | Limited | Limited | Limited | Poor | Discriminant power interpretation |
| ERR | 0.16828 | 0.21877 | 0.24013 | 0.0479 | 0.02071 | 0.02298 | 0.05922 | Error rate |
| F0.5 | 0.5952 | 0.6558 | 0.58904 | 0.67851 | 0.76677 | 0.56995 | 0.07692 | F0.5 score |
| F1 | 0.61019 | 0.66667 | 0.63556 | 0.53165 | 0.6 | 0.38261 | 0.04188 | F1 score - harmonic mean of precision and sensitivity |
| F2 | 0.62596 | 0.6779 | 0.69006 | 0.43704 | 0.49281 | 0.28796 | 0.02878 | F2 score |
| FDR | 0.41439 | 0.35125 | 0.43837 | 0.16832 | 0.05882 | 0.15385 | 0.82609 | False discovery rate |
| FN | 232 | 310 | 237 | 131 | 61 | 67 | 164 | False negative/miss/type 2 error |
| FNR | 0.36307 | 0.3144 | 0.2681 | 0.6093 | 0.55963 | 0.75281 | 0.97619 | Miss rate or false negative rate |
| FOR | 0.09687 | 0.15137 | 0.12229 | 0.04383 | 0.02007 | 0.02187 | 0.05347 | False omission rate |
| FP | 288 | 366 | 505 | 17 | 3 | 4 | 19 | False positive/type 1 error/false alarm |
| FPR | 0.1175 | 0.17395 | 0.22892 | 0.00591 | 0.00101 | 0.00133 | 0.0065 | Fall-out or false positive rate |
| G | 0.61073 | 0.66692 | 0.64114 | 0.57003 | 0.64379 | 0.45734 | 0.06435 | G-measure geometric mean of precision and sensitivity |
| GI | 0.51943 | 0.51164 | 0.50298 | 0.38478 | 0.43936 | 0.24586 | 0.01731 | Gini index |
| GM | 0.74973 | 0.75255 | 0.75123 | 0.62321 | 0.66327 | 0.49685 | 0.1538 | G-mean geometric mean of specificity and sensitivity |
| IBA | 0.42406 | 0.4868 | 0.54224 | 0.15404 | 0.19417 | 0.06135 | 0.00072 | Index of balanced accuracy |
| ICSI | 0.22254 | 0.33435 | 0.29353 | 0.22238 | 0.38154 | 0.09334 | -0.80228 | Individual classification success index |
| IS | 1.50173 | 1.02369 | 0.97319 | 3.5793 | 4.73774 | 4.87665 | 1.67751 | Information score |
| J | 0.43905 | 0.5 | 0.4658 | 0.36207 | 0.42857 | 0.23656 | 0.02139 | Jaccard index |
| LS | 2.83183 | 2.03311 | 1.96317 | 11.95303 | 26.68106 | 29.3777 | 3.19876 | Lift score |
| MCC | 0.50385 | 0.50446 | 0.47008 | 0.55059 | 0.63616 | 0.45018 | 0.04566 | Matthews correlation coefficient |
| MCCI | Moderate | Moderate | Weak | Moderate | Moderate | Weak | Negligible | Matthews correlation coefficient interpretation |
| MCEN | 0.5544 | 0.5218 | 0.55122 | 0.48643 | 0.40791 | 0.47959 | 0.58008 | Modified confusion entropy |
| MK | 0.48874 | 0.49739 | 0.43934 | 0.78786 | 0.9211 | 0.82429 | 0.12044 | Markedness |
| N | 2451 | 2104 | 2206 | 2875 | 2981 | 3001 | 2922 | Condition negative |
| NLR | 0.41141 | 0.38061 | 0.34769 | 0.61293 | 0.5602 | 0.75381 | 0.98258 | Negative likelihood ratio |
| NLRI | Poor | Poor | Poor | Negligible | Negligible | Negligible | Negligible | Negative likelihood ratio interpretation |
| NPV | 0.90313 | 0.84863 | 0.87771 | 0.95617 | 0.97993 | 0.97813 | 0.94653 | Negative predictive value |
| OC | 0.63693 | 0.6856 | 0.7319 | 0.83168 | 0.94118 | 0.84615 | 0.17391 | Overlap coefficient |
| OOC | 0.61073 | 0.66692 | 0.64114 | 0.57003 | 0.64379 | 0.45734 | 0.06435 | Otsuka-Ochiai coefficient |
| OP | 0.6701 | 0.68832 | 0.7338 | 0.51638 | 0.59118 | 0.37384 | -0.01241 | Optimized precision |
| P | 639 | 986 | 884 | 215 | 109 | 89 | 168 | Condition positive or support |
| PLR | 5.42056 | 3.94125 | 3.19717 | 66.07387 | 437.57798 | 185.45506 | 3.66165 | Positive likelihood ratio |
| PLRI | Fair | Poor | Poor | Good | Good | Good | Poor | Positive likelihood ratio interpretation |
| POP | 3090 | 3090 | 3090 | 3090 | 3090 | 3090 | 3090 | Population |
| PPV | 0.58561 | 0.64875 | 0.56163 | 0.83168 | 0.94118 | 0.84615 | 0.17391 | Precision or positive predictive value |
| PRE | 0.2068 | 0.31909 | 0.28608 | 0.06958 | 0.03528 | 0.0288 | 0.05437 | Prevalence |
| Q | 0.85891 | 0.82387 | 0.80383 | 0.98162 | 0.99744 | 0.9919 | 0.57686 | Yule Q - coefficient of colligation |
| QI | Strong | Strong | Strong | Strong | Strong | Strong | Moderate | Yule Q interpretation |
| RACC | 0.04651 | 0.1076 | 0.10666 | 0.00227 | 0.00058 | 0.00024 | 0.0004 | Random accuracy |
| RACCU | 0.04659 | 0.10769 | 0.10854 | 0.00261 | 0.00067 | 0.00035 | 0.00096 | Random accuracy unbiased |
| TN | 2163 | 1738 | 1701 | 2858 | 2978 | 2997 | 2903 | True negative/correct rejection |
| TNR | 0.8825 | 0.82605 | 0.77108 | 0.99409 | 0.99899 | 0.99867 | 0.9935 | Specificity or true negative rate |
| TON | 2395 | 2048 | 1938 | 2989 | 3039 | 3064 | 3067 | Test outcome negative |
| TOP | 695 | 1042 | 1152 | 101 | 51 | 26 | 23 | Test outcome positive |
| TP | 407 | 676 | 647 | 84 | 48 | 22 | 4 | True positive/hit |
| TPR | 0.63693 | 0.6856 | 0.7319 | 0.3907 | 0.44037 | 0.24719 | 0.02381 | Sensitivity, recall, hit rate, or true positive rate |
| Y | 0.51943 | 0.51164 | 0.50298 | 0.38478 | 0.43936 | 0.24586 | 0.01731 | Youden index |
| dInd | 0.38161 | 0.35932 | 0.35254 | 0.60933 | 0.55963 | 0.75281 | 0.97621 | Distance index |
| sInd | 0.73016 | 0.74592 | 0.75072 | 0.56914 | 0.60428 | 0.46768 | 0.30971 | Similarity index |

Generated By PyCM Version 3.2
