# Supplementary material for: TopEC: prediction of Enzyme Commission classes by 3D graph neural networks and localized 3D protein descriptor
Source: Nat Commun. 2025 Mar 20;16:2737. doi: 10.1038/s41467-025-57324-5 (PMC11923149; doi:10.1038/s41467-025-57324-5)
Supplement: Supplementary file 3 — Supplementary Data 1 [file 41467_2025_57324_MOESM3_ESM.zip › Data_S1/table1/mainclass/EnzyNet/full_struc/Combined_TEMP_flips.html]

PyCM Report


# PyCM Report

## Dataset Type :

- Multi-Class Classification
- Imbalanced

Note 1 : Recommended statistics for this type of classification highlighted in aqua

Note 2 : The recommender system assumes that the input is the result of classification over the whole data rather than just a part of it.
If the confusion matrix is the result of test data classification, the recommendation is not valid.

## Confusion Matrix :

|  |  |  |  |  |  |  |  |  |  |  |  |  |  |  |  |  |  |  |  |  |  |  |  |  |  |  |  |  |  |  |  |  |  |  |  |  |  |  |  |  |  |  |  |  |  |  |  |  |  |  |  |  |  |  |  |  |  |  |  |  |  |  |  |  |  |
| --- | --- | --- | --- | --- | --- | --- | --- | --- | --- | --- | --- | --- | --- | --- | --- | --- | --- | --- | --- | --- | --- | --- | --- | --- | --- | --- | --- | --- | --- | --- | --- | --- | --- | --- | --- | --- | --- | --- | --- | --- | --- | --- | --- | --- | --- | --- | --- | --- | --- | --- | --- | --- | --- | --- | --- | --- | --- | --- | --- | --- | --- | --- | --- | --- | --- |
| Actual | Predict  |  |  |  |  |  |  |  |  | | --- | --- | --- | --- | --- | --- | --- | --- | |  | 0 | 1 | 2 | 3 | 4 | 5 | 6 | | 0 | 393 | 111 | 128 | 7 | 0 | 0 | 0 | | 1 | 72 | 681 | 223 | 6 | 0 | 0 | 4 | | 2 | 108 | 111 | 650 | 0 | 0 | 0 | 15 | | 3 | 35 | 31 | 49 | 81 | 1 | 0 | 0 | | 4 | 12 | 30 | 22 | 0 | 45 | 0 | 0 | | 5 | 17 | 29 | 23 | 0 | 0 | 20 | 0 | | 6 | 39 | 64 | 60 | 0 | 0 | 1 | 4 | |

## Overall Statistics :

|  |  |
| --- | --- |
| 95% CI | (0.59278,0.62727) |
| ACC Macro | 0.88858 |
| ARI | 0.25287 |
| AUNP | 0.73045 |
| AUNU | 0.68389 |
| Bangdiwala B | 0.41582 |
| Bennett S | 0.54503 |
| CBA | 0.40871 |
| CSI | 0.12403 |
| Chi-Squared | 4431.37297 |
| Chi-Squared DF | 36 |
| Conditional Entropy | 1.35786 |
| Cramer V | 0.49032 |
| Cross Entropy | 2.48665 |
| F1 Macro | 0.49212 |
| F1 Micro | 0.61003 |
| FNR Macro | 0.55521 |
| FNR Micro | 0.38997 |
| FPR Macro | 0.07702 |
| FPR Micro | 0.065 |
| Gwet AC1 | 0.55606 |
| Hamming Loss | 0.38997 |
| Joint Entropy | 3.67475 |
| KL Divergence | 0.16976 |
| Kappa | 0.46761 |
| Kappa 95% CI | (0.44407,0.49116) |
| Kappa No Prevalence | 0.22005 |
| Kappa Standard Error | 0.01201 |
| Kappa Unbiased | 0.46529 |
| Krippendorff Alpha | 0.46538 |
| Lambda A | 0.43097 |
| Lambda B | 0.41106 |
| Mutual Information | 0.52969 |
| NIR | 0.32096 |
| Overall ACC | 0.61003 |
| Overall CEN | 0.42377 |
| Overall J | (2.43263,0.34752) |
| Overall MCC | 0.47228 |
| Overall MCEN | 0.53154 |
| Overall RACC | 0.2675 |
| Overall RACCU | 0.27068 |
| P-Value | None |
| PPV Macro | 0.67924 |
| PPV Micro | 0.61003 |
| Pearson C | 0.76849 |
| Phi-Squared | 1.4425 |
| RCI | 0.22862 |
| RR | 438.85714 |
| Reference Entropy | 2.31689 |
| Response Entropy | 1.88755 |
| SOA1(Landis & Koch) | Moderate |
| SOA2(Fleiss) | Intermediate to Good |
| SOA3(Altman) | Moderate |
| SOA4(Cicchetti) | Fair |
| SOA5(Cramer) | Relatively Strong |
| SOA6(Matthews) | Weak |
| Scott PI | 0.46529 |
| Standard Error | 0.0088 |
| TNR Macro | 0.92298 |
| TNR Micro | 0.935 |
| TPR Macro | 0.44479 |
| TPR Micro | 0.61003 |
| Zero-one Loss | 1198 |

## Class Statistics :

|  |  |  |  |  |  |  |  |  |
| --- | --- | --- | --- | --- | --- | --- | --- | --- |
| Class | 0 | 1 | 2 | 3 | 4 | 5 | 6 | Description |
| ACC | 0.8278 | 0.77832 | 0.75944 | 0.95801 | 0.97884 | 0.97721 | 0.94043 | Accuracy |
| AGF | 0.73748 | 0.75747 | 0.76911 | 0.66661 | 0.67738 | 0.51031 | 0.1658 | Adjusted F-score |
| AGM | 0.80195 | 0.77967 | 0.75918 | 0.81173 | 0.81782 | 0.73295 | 0.56183 | Adjusted geometric mean |
| AM | 37 | 71 | 271 | -103 | -63 | -68 | -145 | Difference between automatic and manual classification |
| AUC | 0.74935 | 0.75521 | 0.75224 | 0.70332 | 0.70625 | 0.61219 | 0.50863 | Area under the ROC curve |
| AUCI | Good | Good | Good | Good | Good | Fair | Poor | AUC value interpretation |
| AUPR | 0.59819 | 0.66747 | 0.64903 | 0.63643 | 0.69555 | 0.58855 | 0.09886 | Area under the PR curve |
| BCD | 0.00602 | 0.01156 | 0.04411 | 0.01676 | 0.01025 | 0.01107 | 0.0236 | Bray-Curtis dissimilarity |
| BM | 0.49871 | 0.51042 | 0.50449 | 0.40665 | 0.41251 | 0.22438 | 0.01727 | Informedness or bookmaker informedness |
| CEN | 0.45376 | 0.4024 | 0.4261 | 0.39537 | 0.33224 | 0.40646 | 0.54843 | Confusion entropy |
| DOR | 12.13695 | 10.15443 | 9.25743 | 153.72812 | 2082.65625 | 864.34783 | 3.70347 | Diagnostic odds ratio |
| DP | 0.5977 | 0.555 | 0.53285 | 1.20562 | 1.82965 | 1.61908 | 0.31349 | Discriminant power |
| DPI | Poor | Poor | Poor | Limited | Limited | Limited | Poor | Discriminant power interpretation |
| ERR | 0.1722 | 0.22168 | 0.24056 | 0.04199 | 0.02116 | 0.02279 | 0.05957 | Error rate |
| F0.5 | 0.5878 | 0.65305 | 0.59048 | 0.70681 | 0.76792 | 0.57803 | 0.07692 | F0.5 score |
| F1 | 0.59772 | 0.66667 | 0.63757 | 0.5567 | 0.58065 | 0.36364 | 0.04188 | F1 score - harmonic mean of precision and sensitivity |
| F2 | 0.60798 | 0.68086 | 0.69282 | 0.45918 | 0.4668 | 0.26525 | 0.02878 | F2 score |
| FDR | 0.41864 | 0.35572 | 0.43723 | 0.1383 | 0.02174 | 0.04762 | 0.82609 | False discovery rate |
| FN | 246 | 305 | 234 | 116 | 64 | 69 | 164 | False negative/miss/type 2 error |
| FNR | 0.38498 | 0.30933 | 0.26471 | 0.58883 | 0.58716 | 0.77528 | 0.97619 | Miss rate or false negative rate |
| FOR | 0.10267 | 0.15136 | 0.12207 | 0.03895 | 0.02115 | 0.02262 | 0.05379 | False omission rate |
| FP | 283 | 376 | 505 | 13 | 1 | 1 | 19 | False positive/type 1 error/false alarm |
| FPR | 0.11632 | 0.18025 | 0.2308 | 0.00452 | 0.00034 | 0.00034 | 0.00654 | Fall-out or false positive rate |
| G | 0.59796 | 0.66707 | 0.64327 | 0.59523 | 0.63551 | 0.46262 | 0.06435 | G-measure geometric mean of precision and sensitivity |
| GI | 0.49871 | 0.51042 | 0.50449 | 0.40665 | 0.41251 | 0.22438 | 0.01727 | Gini index |
| GM | 0.73721 | 0.75245 | 0.75205 | 0.63977 | 0.64242 | 0.47397 | 0.1538 | G-mean geometric mean of specificity and sensitivity |
| IBA | 0.39747 | 0.49309 | 0.54641 | 0.17015 | 0.17052 | 0.05056 | 0.00072 | Index of balanced accuracy |
| ICSI | 0.19638 | 0.33495 | 0.29806 | 0.27287 | 0.3911 | 0.1771 | -0.80228 | Individual classification success index |
| IS | 1.4828 | 1.00527 | 0.96768 | 3.74817 | 4.78507 | 5.03884 | 1.66908 | Information score |
| J | 0.42625 | 0.5 | 0.46796 | 0.38571 | 0.40909 | 0.22222 | 0.02139 | Jaccard index |
| LS | 2.7949 | 2.00732 | 1.95569 | 13.4373 | 27.5708 | 32.87319 | 3.18012 | Lift score |
| MCC | 0.4886 | 0.50159 | 0.47152 | 0.57842 | 0.62834 | 0.45675 | 0.04554 | Matthews correlation coefficient |
| MCCI | Weak | Moderate | Weak | Moderate | Moderate | Weak | Negligible | Matthews correlation coefficient interpretation |
| MCEN | 0.5652 | 0.52202 | 0.54331 | 0.46723 | 0.3866 | 0.43397 | 0.55182 | Modified confusion entropy |
| MK | 0.47869 | 0.49291 | 0.4407 | 0.82275 | 0.95711 | 0.92977 | 0.12012 | Markedness |
| N | 2433 | 2086 | 2188 | 2875 | 2963 | 2983 | 2904 | Condition negative |
| NLR | 0.43565 | 0.37735 | 0.34413 | 0.59151 | 0.58735 | 0.77554 | 0.98262 | Negative likelihood ratio |
| NLRI | Poor | Poor | Poor | Negligible | Negligible | Negligible | Negligible | Negative likelihood ratio interpretation |
| NPV | 0.89733 | 0.84864 | 0.87793 | 0.96105 | 0.97885 | 0.97738 | 0.94621 | Negative predictive value |
| OC | 0.61502 | 0.69067 | 0.73529 | 0.8617 | 0.97826 | 0.95238 | 0.17391 | Overlap coefficient |
| OOC | 0.59796 | 0.66707 | 0.64327 | 0.59523 | 0.63551 | 0.46262 | 0.06435 | Otsuka-Ochiai coefficient |
| OP | 0.64854 | 0.69286 | 0.73691 | 0.54261 | 0.5634 | 0.34429 | -0.01276 | Optimized precision |
| P | 639 | 986 | 884 | 197 | 109 | 89 | 168 | Condition positive or support |
| PLR | 5.28746 | 3.83175 | 3.18579 | 90.93128 | 1223.25688 | 670.33708 | 3.6391 | Positive likelihood ratio |
| PLRI | Fair | Poor | Poor | Good | Good | Good | Poor | Positive likelihood ratio interpretation |
| POP | 3072 | 3072 | 3072 | 3072 | 3072 | 3072 | 3072 | Population |
| PPV | 0.58136 | 0.64428 | 0.56277 | 0.8617 | 0.97826 | 0.95238 | 0.17391 | Precision or positive predictive value |
| PRE | 0.20801 | 0.32096 | 0.28776 | 0.06413 | 0.03548 | 0.02897 | 0.05469 | Prevalence |
| Q | 0.84776 | 0.8207 | 0.80502 | 0.98707 | 0.99904 | 0.99769 | 0.57478 | Yule Q - coefficient of colligation |
| QI | Strong | Strong | Strong | Strong | Strong | Strong | Moderate | Yule Q interpretation |
| RACC | 0.04577 | 0.11044 | 0.10819 | 0.00196 | 0.00053 | 0.0002 | 0.00041 | Random accuracy |
| RACCU | 0.04581 | 0.11057 | 0.11014 | 0.00224 | 0.00064 | 0.00032 | 0.00097 | Random accuracy unbiased |
| TN | 2150 | 1710 | 1683 | 2862 | 2962 | 2982 | 2885 | True negative/correct rejection |
| TNR | 0.88368 | 0.81975 | 0.7692 | 0.99548 | 0.99966 | 0.99966 | 0.99346 | Specificity or true negative rate |
| TON | 2396 | 2015 | 1917 | 2978 | 3026 | 3051 | 3049 | Test outcome negative |
| TOP | 676 | 1057 | 1155 | 94 | 46 | 21 | 23 | Test outcome positive |
| TP | 393 | 681 | 650 | 81 | 45 | 20 | 4 | True positive/hit |
| TPR | 0.61502 | 0.69067 | 0.73529 | 0.41117 | 0.41284 | 0.22472 | 0.02381 | Sensitivity, recall, hit rate, or true positive rate |
| Y | 0.49871 | 0.51042 | 0.50449 | 0.40665 | 0.41251 | 0.22438 | 0.01727 | Youden index |
| dInd | 0.40216 | 0.35802 | 0.3512 | 0.58885 | 0.58716 | 0.77528 | 0.97621 | Distance index |
| sInd | 0.71563 | 0.74684 | 0.75167 | 0.58362 | 0.58482 | 0.45179 | 0.30971 | Similarity index |

Generated By PyCM Version 3.2
