# Supplementary material for: TopEC: prediction of Enzyme Commission classes by 3D graph neural networks and localized 3D protein descriptor
Source: Nat Commun. 2025 Mar 20;16:2737. doi: 10.1038/s41467-025-57324-5 (PMC11923149; doi:10.1038/s41467-025-57324-5)
Supplement: Supplementary file 3 — Supplementary Data 1 [file 41467_2025_57324_MOESM3_ESM.zip › Data_S1/table1/mainclass/EnzyNet/full_struc/Combined_TEMP.html]

PyCM Report


# PyCM Report

## Dataset Type :

- Multi-Class Classification
- Imbalanced

Note 1 : Recommended statistics for this type of classification highlighted in aqua

Note 2 : The recommender system assumes that the input is the result of classification over the whole data rather than just a part of it.
If the confusion matrix is the result of test data classification, the recommendation is not valid.

## Confusion Matrix :

|  |  |  |  |  |  |  |  |  |  |  |  |  |  |  |  |  |  |  |  |  |  |  |  |  |  |  |  |  |  |  |  |  |  |  |  |  |  |  |  |  |  |  |  |  |  |  |  |  |  |  |  |  |  |  |  |  |  |  |  |  |  |  |  |  |  |
| --- | --- | --- | --- | --- | --- | --- | --- | --- | --- | --- | --- | --- | --- | --- | --- | --- | --- | --- | --- | --- | --- | --- | --- | --- | --- | --- | --- | --- | --- | --- | --- | --- | --- | --- | --- | --- | --- | --- | --- | --- | --- | --- | --- | --- | --- | --- | --- | --- | --- | --- | --- | --- | --- | --- | --- | --- | --- | --- | --- | --- | --- | --- | --- | --- | --- |
| Actual | Predict  |  |  |  |  |  |  |  |  | | --- | --- | --- | --- | --- | --- | --- | --- | |  | 0 | 1 | 2 | 3 | 4 | 5 | 6 | | 0 | 402 | 110 | 110 | 13 | 3 | 1 | 0 | | 1 | 92 | 664 | 183 | 26 | 5 | 12 | 4 | | 2 | 127 | 106 | 619 | 8 | 2 | 6 | 16 | | 3 | 42 | 26 | 41 | 85 | 2 | 1 | 0 | | 4 | 14 | 29 | 19 | 1 | 46 | 0 | 0 | | 5 | 16 | 26 | 20 | 0 | 2 | 25 | 0 | | 6 | 36 | 59 | 51 | 5 | 2 | 5 | 10 | |

## Overall Statistics :

|  |  |
| --- | --- |
| 95% CI | (0.58523,0.61984) |
| ACC Macro | 0.88644 |
| ARI | 0.25372 |
| AUNP | 0.73102 |
| AUNU | 0.68991 |
| Bangdiwala B | 0.40859 |
| Bennett S | 0.5363 |
| CBA | 0.42712 |
| CSI | 0.02626 |
| Chi-Squared | 3639.70454 |
| Chi-Squared DF | 36 |
| Conditional Entropy | 1.53031 |
| Cramer V | 0.44437 |
| Cross Entropy | 2.41482 |
| F1 Macro | 0.48548 |
| F1 Micro | 0.60254 |
| FNR Macro | 0.54333 |
| FNR Micro | 0.39746 |
| FPR Macro | 0.07685 |
| FPR Micro | 0.06624 |
| Gwet AC1 | 0.54661 |
| Hamming Loss | 0.39746 |
| Joint Entropy | 3.8472 |
| KL Divergence | 0.09793 |
| Kappa | 0.46417 |
| Kappa 95% CI | (0.44084,0.4875) |
| Kappa No Prevalence | 0.20508 |
| Kappa Standard Error | 0.0119 |
| Kappa Unbiased | 0.463 |
| Krippendorff Alpha | 0.46309 |
| Lambda A | 0.41755 |
| Lambda B | 0.42287 |
| Mutual Information | 0.49604 |
| NIR | 0.32096 |
| Overall ACC | 0.60254 |
| Overall CEN | 0.45562 |
| Overall J | (2.36466,0.33781) |
| Overall MCC | 0.46642 |
| Overall MCEN | 0.57374 |
| Overall RACC | 0.25823 |
| Overall RACCU | 0.25984 |
| P-Value | None |
| PPV Macro | 0.56959 |
| PPV Micro | 0.60254 |
| Pearson C | 0.7364 |
| Phi-Squared | 1.1848 |
| RCI | 0.2141 |
| RR | 438.85714 |
| Reference Entropy | 2.31689 |
| Response Entropy | 2.02635 |
| SOA1(Landis & Koch) | Moderate |
| SOA2(Fleiss) | Intermediate to Good |
| SOA3(Altman) | Moderate |
| SOA4(Cicchetti) | Fair |
| SOA5(Cramer) | Relatively Strong |
| SOA6(Matthews) | Weak |
| Scott PI | 0.463 |
| Standard Error | 0.00883 |
| TNR Macro | 0.92315 |
| TNR Micro | 0.93376 |
| TPR Macro | 0.45667 |
| TPR Micro | 0.60254 |
| Zero-one Loss | 1221 |

## Class Statistics :

|  |  |  |  |  |  |  |  |  |
| --- | --- | --- | --- | --- | --- | --- | --- | --- |
| Class | 0 | 1 | 2 | 3 | 4 | 5 | 6 | Description |
| ACC | 0.81641 | 0.7793 | 0.77572 | 0.94629 | 0.97428 | 0.97103 | 0.94206 | Accuracy |
| AGF | 0.73877 | 0.74967 | 0.76064 | 0.66575 | 0.6735 | 0.54967 | 0.26104 | Adjusted F-score |
| AGM | 0.79436 | 0.78049 | 0.77418 | 0.8107 | 0.81811 | 0.75629 | 0.60758 | Adjusted geometric mean |
| AM | 90 | 34 | 159 | -59 | -47 | -39 | -138 | Difference between automatic and manual classification |
| AUC | 0.74735 | 0.75138 | 0.75322 | 0.70652 | 0.70831 | 0.63626 | 0.52632 | Area under the ROC curve |
| AUCI | Good | Good | Good | Good | Good | Fair | Poor | AUC value interpretation |
| AUPR | 0.59027 | 0.6622 | 0.64685 | 0.52371 | 0.58198 | 0.39045 | 0.19643 | Area under the PR curve |
| BCD | 0.01465 | 0.00553 | 0.02588 | 0.0096 | 0.00765 | 0.00635 | 0.02246 | Bray-Curtis dissimilarity |
| BM | 0.49471 | 0.50277 | 0.50644 | 0.41304 | 0.41662 | 0.27252 | 0.05264 | Informedness or bookmaker informedness |
| CEN | 0.47169 | 0.4291 | 0.43641 | 0.50605 | 0.46776 | 0.57955 | 0.61751 | Confusion entropy |
| DOR | 10.92417 | 10.02094 | 9.71801 | 40.40937 | 134.48611 | 46.21875 | 9.12658 | Diagnostic odds ratio |
| DP | 0.57249 | 0.55183 | 0.54448 | 0.8857 | 1.1736 | 0.91786 | 0.52945 | Discriminant power |
| DPI | Poor | Poor | Poor | Poor | Limited | Poor | Poor | Discriminant power interpretation |
| ERR | 0.18359 | 0.2207 | 0.22428 | 0.05371 | 0.02572 | 0.02897 | 0.05794 | Error rate |
| F0.5 | 0.5654 | 0.65535 | 0.61214 | 0.56742 | 0.64426 | 0.43253 | 0.17361 | F0.5 score |
| F1 | 0.58772 | 0.66201 | 0.64245 | 0.50746 | 0.53801 | 0.35971 | 0.10101 | F1 score - harmonic mean of precision and sensitivity |
| F2 | 0.61187 | 0.66882 | 0.67591 | 0.45896 | 0.46185 | 0.30788 | 0.07123 | F2 score |
| FDR | 0.44856 | 0.34902 | 0.40652 | 0.38406 | 0.25806 | 0.5 | 0.66667 | False discovery rate |
| FN | 237 | 322 | 265 | 112 | 63 | 64 | 158 | False negative/miss/type 2 error |
| FNR | 0.37089 | 0.32657 | 0.29977 | 0.56853 | 0.57798 | 0.7191 | 0.94048 | Miss rate or false negative rate |
| FOR | 0.10115 | 0.15692 | 0.13061 | 0.03817 | 0.02093 | 0.02118 | 0.05194 | False omission rate |
| FP | 327 | 356 | 424 | 53 | 16 | 25 | 20 | False positive/type 1 error/false alarm |
| FPR | 0.1344 | 0.17066 | 0.19378 | 0.01843 | 0.0054 | 0.00838 | 0.00689 | Fall-out or false positive rate |
| G | 0.589 | 0.66211 | 0.64465 | 0.51552 | 0.55956 | 0.37477 | 0.14086 | G-measure geometric mean of precision and sensitivity |
| GI | 0.49471 | 0.50277 | 0.50644 | 0.41304 | 0.41662 | 0.27252 | 0.05264 | Gini index |
| GM | 0.73794 | 0.74733 | 0.75135 | 0.65078 | 0.64787 | 0.52777 | 0.24313 | G-mean geometric mean of specificity and sensitivity |
| IBA | 0.41577 | 0.47142 | 0.5047 | 0.19054 | 0.1794 | 0.08058 | 0.00393 | Index of balanced accuracy |
| ICSI | 0.18055 | 0.32441 | 0.29371 | 0.04741 | 0.16395 | -0.2191 | -0.60714 | Individual classification success index |
| IS | 1.40657 | 1.0202 | 1.04433 | 3.26378 | 4.38614 | 4.10923 | 2.60768 | Information score |
| J | 0.41615 | 0.49478 | 0.47324 | 0.34 | 0.368 | 0.2193 | 0.05319 | Jaccard index |
| LS | 2.65106 | 2.02821 | 2.06241 | 9.60494 | 20.91033 | 17.25843 | 6.09524 | Lift score |
| MCC | 0.47197 | 0.49839 | 0.48417 | 0.48851 | 0.54807 | 0.36123 | 0.1217 | Matthews correlation coefficient |
| MCCI | Weak | Weak | Weak | Weak | Moderate | Weak | Negligible | Matthews correlation coefficient interpretation |
| MCEN | 0.58623 | 0.55969 | 0.5608 | 0.60037 | 0.5602 | 0.64435 | 0.6306 | Modified confusion entropy |
| MK | 0.45029 | 0.49406 | 0.46287 | 0.57777 | 0.72101 | 0.47882 | 0.28139 | Markedness |
| N | 2433 | 2086 | 2188 | 2875 | 2963 | 2983 | 2904 | Condition negative |
| NLR | 0.42848 | 0.39377 | 0.37183 | 0.57921 | 0.58112 | 0.72518 | 0.947 | Negative likelihood ratio |
| NLRI | Poor | Poor | Poor | Negligible | Negligible | Negligible | Negligible | Negative likelihood ratio interpretation |
| NPV | 0.89885 | 0.84308 | 0.86939 | 0.96183 | 0.97907 | 0.97882 | 0.94806 | Negative predictive value |
| OC | 0.62911 | 0.67343 | 0.70023 | 0.61594 | 0.74194 | 0.5 | 0.33333 | Overlap coefficient |
| OOC | 0.589 | 0.66211 | 0.64465 | 0.51552 | 0.55956 | 0.37477 | 0.14086 | Otsuka-Ochiai coefficient |
| OP | 0.65819 | 0.67555 | 0.70536 | 0.55699 | 0.57009 | 0.41251 | 0.05515 | Optimized precision |
| P | 639 | 986 | 884 | 197 | 109 | 89 | 168 | Condition positive or support |
| PLR | 4.68079 | 3.94599 | 3.61343 | 23.40533 | 78.15252 | 33.51685 | 8.64286 | Positive likelihood ratio |
| PLRI | Poor | Poor | Poor | Good | Good | Good | Fair | Positive likelihood ratio interpretation |
| POP | 3072 | 3072 | 3072 | 3072 | 3072 | 3072 | 3072 | Population |
| PPV | 0.55144 | 0.65098 | 0.59348 | 0.61594 | 0.74194 | 0.5 | 0.33333 | Precision or positive predictive value |
| PRE | 0.20801 | 0.32096 | 0.28776 | 0.06413 | 0.03548 | 0.02897 | 0.05469 | Prevalence |
| Q | 0.83227 | 0.81853 | 0.8134 | 0.9517 | 0.98524 | 0.95764 | 0.8025 | Yule Q - coefficient of colligation |
| QI | Strong | Strong | Strong | Strong | Strong | Strong | Strong | Yule Q interpretation |
| RACC | 0.04936 | 0.10657 | 0.0977 | 0.00288 | 0.00072 | 0.00047 | 0.00053 | Random accuracy |
| RACCU | 0.04958 | 0.1066 | 0.09837 | 0.00297 | 0.00077 | 0.00051 | 0.00104 | Random accuracy unbiased |
| TN | 2106 | 1730 | 1764 | 2822 | 2947 | 2958 | 2884 | True negative/correct rejection |
| TNR | 0.8656 | 0.82934 | 0.80622 | 0.98157 | 0.9946 | 0.99162 | 0.99311 | Specificity or true negative rate |
| TON | 2343 | 2052 | 2029 | 2934 | 3010 | 3022 | 3042 | Test outcome negative |
| TOP | 729 | 1020 | 1043 | 138 | 62 | 50 | 30 | Test outcome positive |
| TP | 402 | 664 | 619 | 85 | 46 | 25 | 10 | True positive/hit |
| TPR | 0.62911 | 0.67343 | 0.70023 | 0.43147 | 0.42202 | 0.2809 | 0.05952 | Sensitivity, recall, hit rate, or true positive rate |
| Y | 0.49471 | 0.50277 | 0.50644 | 0.41304 | 0.41662 | 0.27252 | 0.05264 | Youden index |
| dInd | 0.39449 | 0.36848 | 0.35695 | 0.56883 | 0.57801 | 0.71915 | 0.9405 | Distance index |
| sInd | 0.72105 | 0.73945 | 0.74759 | 0.59778 | 0.59129 | 0.49148 | 0.33497 | Similarity index |

Generated By PyCM Version 3.2
