# Supplementary material for: TopEC: prediction of Enzyme Commission classes by 3D graph neural networks and localized 3D protein descriptor
Source: Nat Commun. 2025 Mar 20;16:2737. doi: 10.1038/s41467-025-57324-5 (PMC11923149; doi:10.1038/s41467-025-57324-5)
Supplement: Supplementary file 3 — Supplementary Data 1 [file 41467_2025_57324_MOESM3_ESM.zip › Data_S1/table1/mainclass/EnzyNet/full_struc/Combined_FOLD_wflips.html]

PyCM Report


# PyCM Report

## Dataset Type :

- Multi-Class Classification
- Imbalanced

Note 1 : Recommended statistics for this type of classification highlighted in aqua

Note 2 : The recommender system assumes that the input is the result of classification over the whole data rather than just a part of it.
If the confusion matrix is the result of test data classification, the recommendation is not valid.

## Confusion Matrix :

|  |  |  |  |  |  |  |  |  |  |  |  |  |  |  |  |  |  |  |  |  |  |  |  |  |  |  |  |  |  |  |  |  |  |  |  |  |  |  |  |  |  |  |  |  |  |  |  |  |  |  |  |  |  |  |  |  |  |  |  |  |  |  |  |  |  |
| --- | --- | --- | --- | --- | --- | --- | --- | --- | --- | --- | --- | --- | --- | --- | --- | --- | --- | --- | --- | --- | --- | --- | --- | --- | --- | --- | --- | --- | --- | --- | --- | --- | --- | --- | --- | --- | --- | --- | --- | --- | --- | --- | --- | --- | --- | --- | --- | --- | --- | --- | --- | --- | --- | --- | --- | --- | --- | --- | --- | --- | --- | --- | --- | --- | --- |
| Actual | Predict  |  |  |  |  |  |  |  |  | | --- | --- | --- | --- | --- | --- | --- | --- | |  | 0 | 1 | 2 | 3 | 4 | 5 | 6 | | 0 | 312 | 108 | 157 | 0 | 0 | 0 | 0 | | 1 | 54 | 830 | 168 | 0 | 0 | 0 | 0 | | 2 | 47 | 193 | 341 | 0 | 0 | 1 | 0 | | 3 | 60 | 89 | 63 | 1 | 1 | 1 | 0 | | 4 | 69 | 117 | 62 | 6 | 2 | 0 | 0 | | 5 | 9 | 80 | 48 | 1 | 0 | 3 | 0 | | 6 | 4 | 42 | 9 | 0 | 0 | 0 | 0 | |

## Overall Statistics :

|  |  |
| --- | --- |
| 95% CI | (0.49912,0.53563) |
| ACC Macro | 0.86211 |
| ARI | 0.18161 |
| AUNP | 0.66268 |
| AUNU | 0.59105 |
| Bangdiwala B | 0.38376 |
| Bennett S | 0.43694 |
| CBA | 0.22078 |
| CSI | None |
| Chi-Squared | None |
| Chi-Squared DF | 36 |
| Conditional Entropy | 1.24797 |
| Cramer V | None |
| Cross Entropy | 3.15546 |
| F1 Macro | 0.25068 |
| F1 Micro | 0.51737 |
| FNR Macro | 0.72152 |
| FNR Micro | 0.48263 |
| FPR Macro | 0.09638 |
| FPR Micro | 0.08044 |
| Gwet AC1 | 0.45311 |
| Hamming Loss | 0.48263 |
| Joint Entropy | 3.62223 |
| KL Divergence | None |
| Kappa | 0.326 |
| Kappa 95% CI | (0.3005,0.35149) |
| Kappa No Prevalence | 0.03475 |
| Kappa Standard Error | 0.01301 |
| Kappa Unbiased | 0.31546 |
| Krippendorff Alpha | 0.31558 |
| Lambda A | 0.24206 |
| Lambda B | 0.24806 |
| Mutual Information | 0.27609 |
| NIR | 0.36553 |
| Overall ACC | 0.51737 |
| Overall CEN | 0.45674 |
| Overall J | (1.22064,0.17438) |
| Overall MCC | 0.33834 |
| Overall MCEN | 0.54869 |
| Overall RACC | 0.28394 |
| Overall RACCU | 0.29496 |
| P-Value | None |
| PPV Macro | None |
| PPV Micro | 0.51737 |
| Pearson C | None |
| Phi-Squared | None |
| RCI | 0.11628 |
| RR | 411.14286 |
| Reference Entropy | 2.37426 |
| Response Entropy | 1.52406 |
| SOA1(Landis & Koch) | Fair |
| SOA2(Fleiss) | Poor |
| SOA3(Altman) | Fair |
| SOA4(Cicchetti) | Poor |
| SOA5(Cramer) | None |
| SOA6(Matthews) | Weak |
| Scott PI | 0.31546 |
| Standard Error | 0.00931 |
| TNR Macro | 0.90362 |
| TNR Micro | 0.91956 |
| TPR Macro | 0.27848 |
| TPR Micro | 0.51737 |
| Zero-one Loss | 1389 |

## Class Statistics :

|  |  |  |  |  |  |  |  |  |
| --- | --- | --- | --- | --- | --- | --- | --- | --- |
| Class | 0 | 1 | 2 | 3 | 4 | 5 | 6 | Description |
| ACC | 0.82349 | 0.70431 | 0.7401 | 0.92321 | 0.9114 | 0.95136 | 0.98089 | Accuracy |
| AGF | 0.69544 | 0.76435 | 0.67899 | 0.07355 | 0.09506 | 0.15917 | 0.0 | Adjusted F-score |
| AGM | 0.78383 | 0.69446 | 0.7216 | 0.51471 | 0.52279 | 0.56182 | 0 | Adjusted geometric mean |
| AM | -22 | 407 | 266 | -207 | -253 | -136 | -55 | Difference between automatic and manual classification |
| AUC | 0.71756 | 0.72225 | 0.68255 | 0.50101 | 0.50372 | 0.51027 | 0.5 | Area under the ROC curve |
| AUCI | Good | Good | Fair | Poor | Poor | Poor | Poor | AUC value interpretation |
| AUPR | 0.55145 | 0.67893 | 0.49402 | 0.06483 | 0.33724 | 0.31064 | None | Area under the PR curve |
| BCD | 0.00382 | 0.07071 | 0.04621 | 0.03596 | 0.04395 | 0.02363 | 0.00956 | Bray-Curtis dissimilarity |
| BM | 0.43512 | 0.4445 | 0.36509 | 0.00202 | 0.00743 | 0.02055 | 0.0 | Informedness or bookmaker informedness |
| CEN | 0.46682 | 0.41672 | 0.52365 | 0.50181 | 0.46774 | 0.39016 | 0.27878 | Confusion entropy |
| DOR | 9.97121 | 7.1149 | 4.99275 | 1.77303 | 20.6378 | 29.72826 | None | Diagnostic odds ratio |
| DP | 0.55064 | 0.46983 | 0.38501 | 0.13712 | 0.72481 | 0.8122 | None | Discriminant power |
| DPI | Poor | Poor | Poor | Poor | Poor | Poor | None | Discriminant power interpretation |
| ERR | 0.17651 | 0.29569 | 0.2599 | 0.07679 | 0.0886 | 0.04864 | 0.01911 | Error rate |
| F0.5 | 0.55774 | 0.6025 | 0.42904 | 0.02024 | 0.03731 | 0.09317 | 0.0 | F0.5 score |
| F1 | 0.55124 | 0.66109 | 0.47692 | 0.00897 | 0.01544 | 0.0411 | 0.0 | F1 score - harmonic mean of precision and sensitivity |
| F2 | 0.54488 | 0.73231 | 0.53684 | 0.00576 | 0.00974 | 0.02636 | 0.0 | F2 score |
| FDR | 0.43784 | 0.43112 | 0.59788 | 0.875 | 0.33333 | 0.4 | None | False discovery rate |
| FN | 265 | 222 | 241 | 214 | 254 | 138 | 55 | False negative/miss/type 2 error |
| FNR | 0.45927 | 0.21103 | 0.41409 | 0.99535 | 0.99219 | 0.97872 | 1.0 | Miss rate or false negative rate |
| FOR | 0.11408 | 0.15645 | 0.11872 | 0.07456 | 0.08835 | 0.04803 | 0.01911 | False omission rate |
| FP | 243 | 629 | 507 | 7 | 1 | 2 | 0 | False positive/type 1 error/false alarm |
| FPR | 0.10561 | 0.34447 | 0.22082 | 0.00263 | 0.00038 | 0.00073 | 0.0 | Fall-out or false positive rate |
| G | 0.55134 | 0.66995 | 0.48539 | 0.02411 | 0.07217 | 0.11299 | None | G-measure geometric mean of precision and sensitivity |
| GI | 0.43512 | 0.4445 | 0.36509 | 0.00202 | 0.00743 | 0.02055 | 0.0 | Gini index |
| GM | 0.69543 | 0.71916 | 0.67567 | 0.06811 | 0.08837 | 0.14581 | 0.0 | G-mean geometric mean of specificity and sensitivity |
| IBA | 0.31258 | 0.58621 | 0.3683 | 3e-05 | 6e-05 | 0.00047 | 0.0 | Index of balanced accuracy |
| ICSI | 0.10289 | 0.35786 | -0.01197 | -0.87035 | -0.32552 | -0.37872 | None | Individual classification success index |
| IS | 1.48748 | 0.63814 | 0.99168 | 0.74266 | 2.90589 | 3.61433 | None | Information score |
| J | 0.38049 | 0.49375 | 0.31313 | 0.0045 | 0.00778 | 0.02098 | 0.0 | Jaccard index |
| LS | 2.80399 | 1.55632 | 1.9885 | 1.67326 | 7.49479 | 12.24681 | None | Lift score |
| MCC | 0.44156 | 0.42817 | 0.32166 | 0.0101 | 0.06556 | 0.10649 | None | Matthews correlation coefficient |
| MCCI | Weak | Weak | Weak | Negligible | Negligible | Negligible | None | Matthews correlation coefficient interpretation |
| MCEN | 0.56405 | 0.54073 | 0.61232 | 0.50227 | 0.46828 | 0.39017 | 0.27878 | Modified confusion entropy |
| MK | 0.44809 | 0.41243 | 0.2834 | 0.05044 | 0.57832 | 0.55197 | None | Markedness |
| N | 2301 | 1826 | 2296 | 2663 | 2622 | 2737 | 2823 | Condition negative |
| NLR | 0.5135 | 0.32192 | 0.53144 | 0.99797 | 0.99257 | 0.97944 | 1.0 | Negative likelihood ratio |
| NLRI | Negligible | Poor | Negligible | Negligible | Negligible | Negligible | Negligible | Negative likelihood ratio interpretation |
| NPV | 0.88592 | 0.84355 | 0.88128 | 0.92544 | 0.91165 | 0.95197 | 0.98089 | Negative predictive value |
| OC | 0.56216 | 0.78897 | 0.58591 | 0.125 | 0.66667 | 0.6 | None | Overlap coefficient |
| OOC | 0.55134 | 0.66995 | 0.48539 | 0.02411 | 0.07217 | 0.11299 | None | Otsuka-Ochiai coefficient |
| OP | 0.57705 | 0.61193 | 0.59852 | -0.06751 | -0.07309 | -0.00695 | -0.01911 | Optimized precision |
| P | 577 | 1052 | 582 | 215 | 256 | 141 | 55 | Condition positive or support |
| PLR | 5.12023 | 2.29041 | 2.65335 | 1.76944 | 20.48437 | 29.11702 | None | Positive likelihood ratio |
| PLRI | Fair | Poor | Poor | Poor | Good | Good | None | Positive likelihood ratio interpretation |
| POP | 2878 | 2878 | 2878 | 2878 | 2878 | 2878 | 2878 | Population |
| PPV | 0.56216 | 0.56888 | 0.40212 | 0.125 | 0.66667 | 0.6 | None | Precision or positive predictive value |
| PRE | 0.20049 | 0.36553 | 0.20222 | 0.0747 | 0.08895 | 0.04899 | 0.01911 | Prevalence |
| Q | 0.8177 | 0.75354 | 0.66626 | 0.27877 | 0.90757 | 0.93491 | None | Yule Q - coefficient of colligation |
| QI | Strong | Strong | Moderate | Weak | Strong | Strong | None | Yule Q interpretation |
| RACC | 0.03866 | 0.18531 | 0.05959 | 0.00021 | 9e-05 | 9e-05 | 0.0 | Random accuracy |
| RACCU | 0.03868 | 0.19031 | 0.06172 | 0.0015 | 0.00202 | 0.00064 | 9e-05 | Random accuracy unbiased |
| TN | 2058 | 1197 | 1789 | 2656 | 2621 | 2735 | 2823 | True negative/correct rejection |
| TNR | 0.89439 | 0.65553 | 0.77918 | 0.99737 | 0.99962 | 0.99927 | 1.0 | Specificity or true negative rate |
| TON | 2323 | 1419 | 2030 | 2870 | 2875 | 2873 | 2878 | Test outcome negative |
| TOP | 555 | 1459 | 848 | 8 | 3 | 5 | 0 | Test outcome positive |
| TP | 312 | 830 | 341 | 1 | 2 | 3 | 0 | True positive/hit |
| TPR | 0.54073 | 0.78897 | 0.58591 | 0.00465 | 0.00781 | 0.02128 | 0.0 | Sensitivity, recall, hit rate, or true positive rate |
| Y | 0.43512 | 0.4445 | 0.36509 | 0.00202 | 0.00743 | 0.02055 | 0.0 | Youden index |
| dInd | 0.47126 | 0.40397 | 0.46929 | 0.99535 | 0.99219 | 0.97872 | 1.0 | Distance index |
| sInd | 0.66677 | 0.71435 | 0.66816 | 0.29618 | 0.29842 | 0.30794 | 0.29289 | Similarity index |

Generated By PyCM Version 3.2
