# Supplementary material for: TopEC: prediction of Enzyme Commission classes by 3D graph neural networks and localized 3D protein descriptor
Source: Nat Commun. 2025 Mar 20;16:2737. doi: 10.1038/s41467-025-57324-5 (PMC11923149; doi:10.1038/s41467-025-57324-5)
Supplement: Supplementary file 3 — Supplementary Data 1 [file 41467_2025_57324_MOESM3_ESM.zip › Data_S1/table1/mainclass/EnzyNet/full_struc/Combined_FOLD_flips.html]

PyCM Report


# PyCM Report

## Dataset Type :

- Multi-Class Classification
- Imbalanced

Note 1 : Recommended statistics for this type of classification highlighted in aqua

Note 2 : The recommender system assumes that the input is the result of classification over the whole data rather than just a part of it.
If the confusion matrix is the result of test data classification, the recommendation is not valid.

## Confusion Matrix :

|  |  |  |  |  |  |  |  |  |  |  |  |  |  |  |  |  |  |  |  |  |  |  |  |  |  |  |  |  |  |  |  |  |  |  |  |  |  |  |  |  |  |  |  |  |  |  |  |  |  |  |  |  |  |  |  |  |  |  |  |  |  |  |  |  |  |
| --- | --- | --- | --- | --- | --- | --- | --- | --- | --- | --- | --- | --- | --- | --- | --- | --- | --- | --- | --- | --- | --- | --- | --- | --- | --- | --- | --- | --- | --- | --- | --- | --- | --- | --- | --- | --- | --- | --- | --- | --- | --- | --- | --- | --- | --- | --- | --- | --- | --- | --- | --- | --- | --- | --- | --- | --- | --- | --- | --- | --- | --- | --- | --- | --- | --- |
| Actual | Predict  |  |  |  |  |  |  |  |  | | --- | --- | --- | --- | --- | --- | --- | --- | |  | 0 | 1 | 2 | 3 | 4 | 5 | 6 | | 0 | 305 | 121 | 151 | 0 | 0 | 0 | 0 | | 1 | 47 | 831 | 173 | 1 | 0 | 0 | 0 | | 2 | 51 | 193 | 338 | 0 | 0 | 0 | 0 | | 3 | 58 | 86 | 69 | 1 | 0 | 1 | 0 | | 4 | 66 | 122 | 60 | 5 | 2 | 1 | 0 | | 5 | 7 | 83 | 49 | 1 | 0 | 1 | 0 | | 6 | 6 | 42 | 7 | 1 | 0 | 0 | 0 | |

## Overall Statistics :

|  |  |
| --- | --- |
| 95% CI | (0.49511,0.53163) |
| ACC Macro | 0.86096 |
| ARI | 0.17542 |
| AUNP | 0.65923 |
| AUNU | 0.58838 |
| Bangdiwala B | 0.38008 |
| Bennett S | 0.43227 |
| CBA | 0.21564 |
| CSI | None |
| Chi-Squared | None |
| Chi-Squared DF | 36 |
| Conditional Entropy | 1.24507 |
| Cramer V | None |
| Cross Entropy | 3.23172 |
| F1 Macro | 0.24491 |
| F1 Micro | 0.51337 |
| FNR Macro | 0.72588 |
| FNR Micro | 0.48663 |
| FPR Macro | 0.09736 |
| FPR Micro | 0.0811 |
| Gwet AC1 | 0.44874 |
| Hamming Loss | 0.48663 |
| Joint Entropy | 3.62049 |
| KL Divergence | None |
| Kappa | 0.3194 |
| Kappa 95% CI | (0.29387,0.34494) |
| Kappa No Prevalence | 0.02675 |
| Kappa Standard Error | 0.01303 |
| Kappa Unbiased | 0.30825 |
| Krippendorff Alpha | 0.30837 |
| Lambda A | 0.23536 |
| Lambda B | 0.23483 |
| Mutual Information | 0.26456 |
| NIR | 0.3654 |
| Overall ACC | 0.51337 |
| Overall CEN | 0.45856 |
| Overall J | (1.19382,0.17055) |
| Overall MCC | 0.33212 |
| Overall MCEN | 0.54973 |
| Overall RACC | 0.285 |
| Overall RACCU | 0.29653 |
| P-Value | None |
| PPV Macro | None |
| PPV Micro | 0.51337 |
| Pearson C | None |
| Phi-Squared | None |
| RCI | 0.11137 |
| RR | 411.28571 |
| Reference Entropy | 2.37542 |
| Response Entropy | 1.50963 |
| SOA1(Landis & Koch) | Fair |
| SOA2(Fleiss) | Poor |
| SOA3(Altman) | Fair |
| SOA4(Cicchetti) | Poor |
| SOA5(Cramer) | None |
| SOA6(Matthews) | Weak |
| Scott PI | 0.30825 |
| Standard Error | 0.00932 |
| TNR Macro | 0.90264 |
| TNR Micro | 0.9189 |
| TPR Macro | 0.27412 |
| TPR Micro | 0.51337 |
| Zero-one Loss | 1401 |

## Class Statistics :

|  |  |  |  |  |  |  |  |  |
| --- | --- | --- | --- | --- | --- | --- | --- | --- |
| Class | 0 | 1 | 2 | 3 | 4 | 5 | 6 | Description |
| ACC | 0.8239 | 0.69851 | 0.73845 | 0.92289 | 0.91177 | 0.95068 | 0.98055 | Accuracy |
| AGF | 0.68898 | 0.7617 | 0.67562 | 0.0735 | 0.09511 | 0.09203 | 0.0 | Adjusted F-score |
| AGM | 0.78179 | 0.68772 | 0.71942 | 0.51453 | 0.52299 | 0.53024 | 0 | Adjusted geometric mean |
| AM | -37 | 426 | 265 | -206 | -254 | -138 | -56 | Difference between automatic and manual classification |
| AUC | 0.71326 | 0.7179 | 0.67958 | 0.50082 | 0.50391 | 0.50318 | 0.5 | Area under the ROC curve |
| AUCI | Good | Good | Fair | Poor | Poor | Poor | Poor | AUC value interpretation |
| AUPR | 0.54671 | 0.67609 | 0.48991 | 0.05788 | 0.50391 | 0.17021 | None | Area under the PR curve |
| BCD | 0.00643 | 0.07398 | 0.04602 | 0.03578 | 0.04411 | 0.02397 | 0.00973 | Bray-Curtis dissimilarity |
| BM | 0.42651 | 0.43579 | 0.35916 | 0.00165 | 0.00781 | 0.00636 | 0.0 | Informedness or bookmaker informedness |
| CEN | 0.46928 | 0.41997 | 0.52461 | 0.50772 | 0.45879 | 0.37624 | 0.31667 | Confusion entropy |
| DOR | 9.86288 | 6.85783 | 4.86605 | 1.5514 | None | 9.77143 | None | Diagnostic odds ratio |
| DP | 0.54802 | 0.46101 | 0.37886 | 0.10515 | None | 0.54579 | None | Discriminant power |
| DPI | Poor | Poor | Poor | Poor | None | Poor | None | Discriminant power interpretation |
| ERR | 0.1761 | 0.30149 | 0.26155 | 0.07711 | 0.08823 | 0.04932 | 0.01945 | Error rate |
| F0.5 | 0.55718 | 0.59664 | 0.42569 | 0.01992 | 0.03788 | 0.03268 | 0.0 | F0.5 score |
| F1 | 0.54611 | 0.65692 | 0.47306 | 0.00893 | 0.0155 | 0.01389 | 0.0 | F1 score - harmonic mean of precision and sensitivity |
| F2 | 0.53546 | 0.73074 | 0.53228 | 0.00575 | 0.00975 | 0.00882 | 0.0 | F2 score |
| FDR | 0.43519 | 0.43775 | 0.60094 | 0.88889 | 0.0 | 0.66667 | None | False discovery rate |
| FN | 272 | 221 | 244 | 214 | 254 | 140 | 56 | False negative/miss/type 2 error |
| FNR | 0.4714 | 0.21008 | 0.41924 | 0.99535 | 0.99219 | 0.99291 | 1.0 | Miss rate or false negative rate |
| FOR | 0.11629 | 0.15774 | 0.12008 | 0.07456 | 0.08829 | 0.04868 | 0.01945 | False omission rate |
| FP | 235 | 647 | 509 | 8 | 0 | 2 | 0 | False positive/type 1 error/false alarm |
| FPR | 0.10209 | 0.35413 | 0.22159 | 0.003 | 0.0 | 0.00073 | 0.0 | Fall-out or false positive rate |
| G | 0.54641 | 0.66643 | 0.48141 | 0.02273 | 0.08839 | 0.04862 | None | G-measure geometric mean of precision and sensitivity |
| GI | 0.42651 | 0.43579 | 0.35916 | 0.00165 | 0.00781 | 0.00636 | 0.0 | Gini index |
| GM | 0.68894 | 0.71427 | 0.67236 | 0.0681 | 0.08839 | 0.08418 | 0.0 | G-mean geometric mean of specificity and sensitivity |
| IBA | 0.29934 | 0.58368 | 0.36271 | 4e-05 | 6e-05 | 6e-05 | 0.0 | Index of balanced accuracy |
| ICSI | 0.09341 | 0.35217 | -0.02019 | -0.88424 | 0.00781 | -0.65957 | None | Individual classification success index |
| IS | 1.49477 | 0.62171 | 0.98114 | 0.57323 | 3.49135 | 2.76684 | None | Information score |
| J | 0.37562 | 0.48911 | 0.30981 | 0.00448 | 0.00781 | 0.00699 | 0.0 | Jaccard index |
| LS | 2.8182 | 1.53869 | 1.97402 | 1.48786 | 11.24609 | 6.80615 | None | Lift score |
| MCC | 0.43738 | 0.41986 | 0.31654 | 0.00776 | 0.0844 | 0.04255 | None | Matthews correlation coefficient |
| MCCI | Weak | Weak | Weak | Negligible | Negligible | Negligible | None | Matthews correlation coefficient interpretation |
| MCEN | 0.56542 | 0.54351 | 0.61218 | 0.5082 | 0.45927 | 0.37609 | 0.31667 | Modified confusion entropy |
| MK | 0.44853 | 0.4045 | 0.27898 | 0.03655 | 0.91171 | 0.28465 | None | Markedness |
| N | 2302 | 1827 | 2297 | 2664 | 2623 | 2738 | 2823 | Condition negative |
| NLR | 0.525 | 0.32526 | 0.53859 | 0.99835 | 0.99219 | 0.99363 | 1.0 | Negative likelihood ratio |
| NLRI | Negligible | Poor | Negligible | Negligible | Negligible | Negligible | Negligible | Negative likelihood ratio interpretation |
| NPV | 0.88371 | 0.84226 | 0.87992 | 0.92544 | 0.91171 | 0.95132 | 0.98055 | Negative predictive value |
| OC | 0.56481 | 0.78992 | 0.58076 | 0.11111 | 1.0 | 0.33333 | None | Overlap coefficient |
| OOC | 0.54641 | 0.66643 | 0.48141 | 0.02273 | 0.08839 | 0.04862 | None | Otsuka-Ochiai coefficient |
| OP | 0.565 | 0.59817 | 0.59303 | -0.06782 | -0.07272 | -0.03523 | -0.01945 | Optimized precision |
| P | 577 | 1052 | 582 | 215 | 256 | 141 | 56 | Condition positive or support |
| PLR | 5.17799 | 2.23059 | 2.62082 | 1.54884 | None | 9.70922 | None | Positive likelihood ratio |
| PLRI | Fair | Poor | Poor | Poor | None | Fair | None | Positive likelihood ratio interpretation |
| POP | 2879 | 2879 | 2879 | 2879 | 2879 | 2879 | 2879 | Population |
| PPV | 0.56481 | 0.56225 | 0.39906 | 0.11111 | 1.0 | 0.33333 | None | Precision or positive predictive value |
| PRE | 0.20042 | 0.3654 | 0.20215 | 0.07468 | 0.08892 | 0.04898 | 0.01945 | Prevalence |
| Q | 0.81589 | 0.74548 | 0.65906 | 0.21612 | None | 0.81432 | None | Yule Q - coefficient of colligation |
| QI | Strong | Moderate | Moderate | Negligible | None | Strong | None | Yule Q interpretation |
| RACC | 0.03759 | 0.18759 | 0.05947 | 0.00023 | 6e-05 | 5e-05 | 0.0 | Random accuracy |
| RACCU | 0.03763 | 0.19306 | 0.06159 | 0.00151 | 0.00201 | 0.00063 | 9e-05 | Random accuracy unbiased |
| TN | 2067 | 1180 | 1788 | 2656 | 2623 | 2736 | 2823 | True negative/correct rejection |
| TNR | 0.89791 | 0.64587 | 0.77841 | 0.997 | 1.0 | 0.99927 | 1.0 | Specificity or true negative rate |
| TON | 2339 | 1401 | 2032 | 2870 | 2877 | 2876 | 2879 | Test outcome negative |
| TOP | 540 | 1478 | 847 | 9 | 2 | 3 | 0 | Test outcome positive |
| TP | 305 | 831 | 338 | 1 | 2 | 1 | 0 | True positive/hit |
| TPR | 0.5286 | 0.78992 | 0.58076 | 0.00465 | 0.00781 | 0.00709 | 0.0 | Sensitivity, recall, hit rate, or true positive rate |
| Y | 0.42651 | 0.43579 | 0.35916 | 0.00165 | 0.00781 | 0.00636 | 0.0 | Youden index |
| dInd | 0.48233 | 0.41175 | 0.4742 | 0.99535 | 0.99219 | 0.99291 | 1.0 | Distance index |
| sInd | 0.65894 | 0.70885 | 0.66469 | 0.29618 | 0.29842 | 0.29791 | 0.29289 | Similarity index |

Generated By PyCM Version 3.2
