# Supplementary material for: TopEC: prediction of Enzyme Commission classes by 3D graph neural networks and localized 3D protein descriptor
Source: Nat Commun. 2025 Mar 20;16:2737. doi: 10.1038/s41467-025-57324-5 (PMC11923149; doi:10.1038/s41467-025-57324-5)
Supplement: Supplementary file 3 — Supplementary Data 1 [file 41467_2025_57324_MOESM3_ESM.zip › Data_S1/table1/mainclass/EnzyNet/full_struc/Combined_FOLD.html]

PyCM Report


# PyCM Report

## Dataset Type :

- Multi-Class Classification
- Imbalanced

Note 1 : Recommended statistics for this type of classification highlighted in aqua

Note 2 : The recommender system assumes that the input is the result of classification over the whole data rather than just a part of it.
If the confusion matrix is the result of test data classification, the recommendation is not valid.

## Confusion Matrix :

|  |  |  |  |  |  |  |  |  |  |  |  |  |  |  |  |  |  |  |  |  |  |  |  |  |  |  |  |  |  |  |  |  |  |  |  |  |  |  |  |  |  |  |  |  |  |  |  |  |  |  |  |  |  |  |  |  |  |  |  |  |  |  |  |  |  |
| --- | --- | --- | --- | --- | --- | --- | --- | --- | --- | --- | --- | --- | --- | --- | --- | --- | --- | --- | --- | --- | --- | --- | --- | --- | --- | --- | --- | --- | --- | --- | --- | --- | --- | --- | --- | --- | --- | --- | --- | --- | --- | --- | --- | --- | --- | --- | --- | --- | --- | --- | --- | --- | --- | --- | --- | --- | --- | --- | --- | --- | --- | --- | --- | --- | --- |
| Actual | Predict  |  |  |  |  |  |  |  |  | | --- | --- | --- | --- | --- | --- | --- | --- | |  | 0 | 1 | 2 | 3 | 4 | 5 | 6 | | 0 | 311 | 116 | 137 | 10 | 0 | 3 | 0 | | 1 | 86 | 764 | 187 | 11 | 1 | 3 | 4 | | 2 | 68 | 180 | 311 | 9 | 2 | 12 | 15 | | 3 | 52 | 80 | 62 | 12 | 2 | 7 | 0 | | 4 | 72 | 85 | 75 | 14 | 7 | 3 | 0 | | 5 | 25 | 56 | 50 | 3 | 1 | 6 | 0 | | 6 | 6 | 34 | 14 | 1 | 0 | 0 | 0 | |

## Overall Statistics :

|  |  |
| --- | --- |
| 95% CI | (0.46885,0.50526) |
| ACC Macro | 0.85344 |
| ARI | 0.15994 |
| AUNP | 0.65112 |
| AUNU | 0.58653 |
| Bangdiwala B | 0.34282 |
| Bennett S | 0.40156 |
| CBA | 0.22576 |
| CSI | -0.38876 |
| Chi-Squared | 1015.17254 |
| Chi-Squared DF | 36 |
| Conditional Entropy | 1.55492 |
| Cramer V | 0.24167 |
| Cross Entropy | 2.78204 |
| F1 Macro | 0.258 |
| F1 Micro | 0.48706 |
| FNR Macro | 0.72727 |
| FNR Micro | 0.51294 |
| FPR Macro | 0.09968 |
| FPR Micro | 0.08549 |
| Gwet AC1 | 0.41675 |
| Hamming Loss | 0.51294 |
| Joint Entropy | 3.92749 |
| KL Divergence | 0.40947 |
| Kappa | 0.29716 |
| Kappa 95% CI | (0.27222,0.3221) |
| Kappa No Prevalence | -0.02589 |
| Kappa Standard Error | 0.01272 |
| Kappa Unbiased | 0.29074 |
| Krippendorff Alpha | 0.29086 |
| Lambda A | 0.20532 |
| Lambda B | 0.20607 |
| Mutual Information | 0.22942 |
| NIR | 0.36452 |
| Overall ACC | 0.48706 |
| Overall CEN | 0.52066 |
| Overall J | (1.21147,0.17307) |
| Overall MCC | 0.30345 |
| Overall MCEN | 0.6219 |
| Overall RACC | 0.27018 |
| Overall RACCU | 0.27679 |
| P-Value | None |
| PPV Macro | 0.33851 |
| PPV Micro | 0.48706 |
| Pearson C | 0.5094 |
| Phi-Squared | 0.35042 |
| RCI | 0.0967 |
| RR | 413.85714 |
| Reference Entropy | 2.37257 |
| Response Entropy | 1.78434 |
| SOA1(Landis & Koch) | Fair |
| SOA2(Fleiss) | Poor |
| SOA3(Altman) | Fair |
| SOA4(Cicchetti) | Poor |
| SOA5(Cramer) | Moderate |
| SOA6(Matthews) | Weak |
| Scott PI | 0.29074 |
| Standard Error | 0.00929 |
| TNR Macro | 0.90032 |
| TNR Micro | 0.91451 |
| TPR Macro | 0.27273 |
| TPR Micro | 0.48706 |
| Zero-one Loss | 1486 |

## Class Statistics :

|  |  |  |  |  |  |  |  |  |
| --- | --- | --- | --- | --- | --- | --- | --- | --- |
| Class | 0 | 1 | 2 | 3 | 4 | 5 | 6 | Description |
| ACC | 0.80152 | 0.70901 | 0.72006 | 0.91336 | 0.91198 | 0.94373 | 0.97446 | Accuracy |
| AGF | 0.68357 | 0.73792 | 0.63716 | 0.24743 | 0.1771 | 0.21946 | 0.0 | Adjusted F-score |
| AGM | 0.76503 | 0.70761 | 0.69499 | 0.5937 | 0.56221 | 0.58775 | 0 | Adjusted geometric mean |
| AM | 43 | 259 | 239 | -155 | -243 | -107 | -36 | Difference between automatic and manual classification |
| AUC | 0.7029 | 0.7121 | 0.64634 | 0.51896 | 0.51254 | 0.5162 | 0.49666 | Area under the ROC curve |
| AUCI | Good | Good | Fair | Poor | Poor | Poor | Poor | AUC value interpretation |
| AUPR | 0.5203 | 0.65224 | 0.44647 | 0.12791 | 0.2829 | 0.10951 | 0.0 | Area under the PR curve |
| BCD | 0.00742 | 0.0447 | 0.04125 | 0.02675 | 0.04194 | 0.01847 | 0.00621 | Bray-Curtis dissimilarity |
| BM | 0.40581 | 0.42419 | 0.29268 | 0.03792 | 0.02507 | 0.03239 | -0.00669 | Informedness or bookmaker informedness |
| CEN | 0.52076 | 0.44042 | 0.59429 | 0.69287 | 0.55993 | 0.6525 | 0.56961 | Confusion entropy |
| DOR | 7.60908 | 6.1256 | 3.67649 | 3.24384 | 12.34605 | 4.33016 | 0.0 | Diagnostic odds ratio |
| DP | 0.4859 | 0.43398 | 0.31174 | 0.28176 | 0.60179 | 0.35092 | None | Discriminant power |
| DPI | Poor | Poor | Poor | Poor | Poor | Poor | None | Discriminant power interpretation |
| ERR | 0.19848 | 0.29099 | 0.27994 | 0.08664 | 0.08802 | 0.05627 | 0.02554 | Error rate |
| F0.5 | 0.50867 | 0.60481 | 0.39457 | 0.13187 | 0.11364 | 0.1083 | 0.0 | F0.5 score |
| F1 | 0.51963 | 0.64445 | 0.43405 | 0.08727 | 0.05204 | 0.06857 | 0.0 | F1 score - harmonic mean of precision and sensitivity |
| F2 | 0.53108 | 0.68966 | 0.48232 | 0.06522 | 0.03375 | 0.05017 | 0.0 | F2 score |
| FDR | 0.49839 | 0.41901 | 0.62799 | 0.8 | 0.46154 | 0.82353 | 1.0 | False discovery rate |
| FN | 266 | 292 | 286 | 203 | 249 | 135 | 55 | False negative/miss/type 2 error |
| FNR | 0.46101 | 0.27652 | 0.47906 | 0.94419 | 0.97266 | 0.95745 | 1.0 | Miss rate or false negative rate |
| FOR | 0.11682 | 0.18458 | 0.13877 | 0.07155 | 0.08634 | 0.04715 | 0.01911 | False omission rate |
| FP | 309 | 551 | 525 | 48 | 6 | 28 | 19 | False positive/type 1 error/false alarm |
| FPR | 0.13319 | 0.29929 | 0.22826 | 0.0179 | 0.00227 | 0.01016 | 0.00669 | Fall-out or false positive rate |
| G | 0.51997 | 0.64833 | 0.44022 | 0.10565 | 0.12134 | 0.08666 | 0.0 | G-measure geometric mean of precision and sensitivity |
| GI | 0.40581 | 0.42419 | 0.29268 | 0.03792 | 0.02507 | 0.03239 | -0.00669 | Gini index |
| GM | 0.68352 | 0.712 | 0.63406 | 0.23413 | 0.16517 | 0.20523 | 0.0 | G-mean geometric mean of specificity and sensitivity |
| IBA | 0.31405 | 0.5185 | 0.3012 | 0.00404 | 0.00081 | 0.00222 | 0.0 | Index of balanced accuracy |
| ICSI | 0.04061 | 0.30447 | -0.10705 | -0.74419 | -0.43419 | -0.78098 | -1.0 | Individual classification success index |
| IS | 1.33256 | 0.67253 | 0.85217 | 1.43022 | 2.60726 | 1.85829 | None | Information score |
| J | 0.35102 | 0.47542 | 0.27718 | 0.04563 | 0.02672 | 0.0355 | 0.0 | Jaccard index |
| LS | 2.5185 | 1.59387 | 1.80521 | 2.69488 | 6.09345 | 3.62578 | 0.0 | Lift score |
| MCC | 0.39516 | 0.41007 | 0.26127 | 0.06979 | 0.10647 | 0.06472 | -0.0113 | Matthews correlation coefficient |
| MCCI | Weak | Weak | Negligible | Negligible | Negligible | Negligible | Negligible | Matthews correlation coefficient interpretation |
| MCEN | 0.62498 | 0.5677 | 0.68786 | 0.70735 | 0.56456 | 0.66212 | 0.56961 | Modified confusion entropy |
| MK | 0.38479 | 0.39641 | 0.23324 | 0.12845 | 0.45212 | 0.12932 | -0.01911 | Markedness |
| N | 2320 | 1841 | 2300 | 2682 | 2641 | 2756 | 2842 | Condition negative |
| NLR | 0.53184 | 0.39462 | 0.62076 | 0.96139 | 0.97487 | 0.96727 | 1.00673 | Negative likelihood ratio |
| NLRI | Negligible | Poor | Negligible | Negligible | Negligible | Negligible | Negligible | Negative likelihood ratio interpretation |
| NPV | 0.88318 | 0.81542 | 0.86123 | 0.92845 | 0.91366 | 0.95285 | 0.98089 | Negative predictive value |
| OC | 0.53899 | 0.72348 | 0.52094 | 0.2 | 0.53846 | 0.17647 | 0.0 | Overlap coefficient |
| OOC | 0.51997 | 0.64833 | 0.44022 | 0.10565 | 0.12134 | 0.08666 | 0.0 | Otsuka-Ochiai coefficient |
| OP | 0.56833 | 0.69302 | 0.52604 | 0.02091 | -0.03467 | 0.02617 | -0.02554 | Optimized precision |
| P | 577 | 1056 | 597 | 215 | 256 | 141 | 55 | Condition positive or support |
| PLR | 4.04682 | 2.41731 | 2.2822 | 3.1186 | 12.03581 | 4.18845 | 0.0 | Positive likelihood ratio |
| PLRI | Poor | Poor | Poor | Poor | Good | Poor | Negligible | Positive likelihood ratio interpretation |
| POP | 2897 | 2897 | 2897 | 2897 | 2897 | 2897 | 2897 | Population |
| PPV | 0.50161 | 0.58099 | 0.37201 | 0.2 | 0.53846 | 0.17647 | 0.0 | Precision or positive predictive value |
| PRE | 0.19917 | 0.36452 | 0.20608 | 0.07421 | 0.08837 | 0.04867 | 0.01899 | Prevalence |
| Q | 0.76769 | 0.71932 | 0.57233 | 0.52873 | 0.85014 | 0.62478 | -1.0 | Yule Q - coefficient of colligation |
| QI | Strong | Moderate | Moderate | Moderate | Strong | Moderate | Negligible | Yule Q interpretation |
| RACC | 0.04263 | 0.16546 | 0.05947 | 0.00154 | 0.0004 | 0.00057 | 0.00012 | Random accuracy |
| RACCU | 0.04268 | 0.16746 | 0.06117 | 0.00225 | 0.00216 | 0.00091 | 0.00016 | Random accuracy unbiased |
| TN | 2011 | 1290 | 1775 | 2634 | 2635 | 2728 | 2823 | True negative/correct rejection |
| TNR | 0.86681 | 0.70071 | 0.77174 | 0.9821 | 0.99773 | 0.98984 | 0.99331 | Specificity or true negative rate |
| TON | 2277 | 1582 | 2061 | 2837 | 2884 | 2863 | 2878 | Test outcome negative |
| TOP | 620 | 1315 | 836 | 60 | 13 | 34 | 19 | Test outcome positive |
| TP | 311 | 764 | 311 | 12 | 7 | 6 | 0 | True positive/hit |
| TPR | 0.53899 | 0.72348 | 0.52094 | 0.05581 | 0.02734 | 0.04255 | 0.0 | Sensitivity, recall, hit rate, or true positive rate |
| Y | 0.40581 | 0.42419 | 0.29268 | 0.03792 | 0.02507 | 0.03239 | -0.00669 | Youden index |
| dInd | 0.47986 | 0.40748 | 0.53066 | 0.94436 | 0.97266 | 0.9575 | 1.00002 | Distance index |
| sInd | 0.66069 | 0.71187 | 0.62476 | 0.33224 | 0.31223 | 0.32294 | 0.29288 | Similarity index |

Generated By PyCM Version 3.2
