# Supplementary material for: TopEC: prediction of Enzyme Commission classes by 3D graph neural networks and localized 3D protein descriptor
Source: Nat Commun. 2025 Mar 20;16:2737. doi: 10.1038/s41467-025-57324-5 (PMC11923149; doi:10.1038/s41467-025-57324-5)
Supplement: Supplementary file 3 — Supplementary Data 1 [file 41467_2025_57324_MOESM3_ESM.zip › Data_S1/table1/mainclass/EnzyNet/full_struc/BindingMOAD_TEMP_wflips.html]

PDB\_TEMP\_enzynet\_wflips


# PyCM Report

## Dataset Type :

- Multi-Class Classification
- Imbalanced

Note 1 : Recommended statistics for this type of classification highlighted in aqua

Note 2 : The recommender system assumes that the input is the result of classification over the whole data rather than just a part of it.
If the confusion matrix is the result of test data classification, the recommendation is not valid.

## Confusion Matrix :

|  |  |  |  |  |  |  |  |  |  |  |  |  |  |  |  |  |  |  |  |  |  |  |  |  |  |  |  |  |  |  |  |  |  |  |  |  |  |  |  |  |  |  |  |  |  |  |  |  |  |  |  |  |  |  |  |  |  |  |  |  |  |  |  |  |  |
| --- | --- | --- | --- | --- | --- | --- | --- | --- | --- | --- | --- | --- | --- | --- | --- | --- | --- | --- | --- | --- | --- | --- | --- | --- | --- | --- | --- | --- | --- | --- | --- | --- | --- | --- | --- | --- | --- | --- | --- | --- | --- | --- | --- | --- | --- | --- | --- | --- | --- | --- | --- | --- | --- | --- | --- | --- | --- | --- | --- | --- | --- | --- | --- | --- | --- |
| Actual | Predict  |  |  |  |  |  |  |  |  | | --- | --- | --- | --- | --- | --- | --- | --- | |  | 0 | 1 | 2 | 3 | 4 | 5 | 6 | | 0 | 306 | 61 | 49 | 5 | 2 | 0 | 0 | | 1 | 48 | 593 | 101 | 4 | 3 | 1 | 4 | | 2 | 74 | 83 | 492 | 5 | 0 | 1 | 15 | | 3 | 17 | 10 | 9 | 88 | 1 | 0 | 0 | | 4 | 4 | 12 | 15 | 0 | 46 | 0 | 0 | | 5 | 8 | 17 | 13 | 0 | 0 | 20 | 0 | | 6 | 14 | 34 | 16 | 1 | 1 | 0 | 10 | |

## Overall Statistics :

|  |  |
| --- | --- |
| 95% CI | (0.69333,0.73131) |
| ACC Macro | 0.91781 |
| ARI | 0.39813 |
| AUNP | 0.79983 |
| AUNU | 0.75869 |
| Bangdiwala B | 0.53833 |
| Bennett S | 0.66438 |
| CBA | 0.5525 |
| CSI | 0.29827 |
| Chi-Squared | 4779.7606 |
| Chi-Squared DF | 36 |
| Conditional Entropy | 1.22798 |
| Cramer V | 0.60409 |
| Cross Entropy | 2.26427 |
| F1 Macro | 0.61912 |
| F1 Micro | 0.71232 |
| FNR Macro | 0.42543 |
| FNR Micro | 0.28768 |
| FPR Macro | 0.05719 |
| FPR Micro | 0.04795 |
| Gwet AC1 | 0.67263 |
| Hamming Loss | 0.28768 |
| Joint Entropy | 3.45367 |
| KL Divergence | 0.03858 |
| Kappa | 0.60486 |
| Kappa 95% CI | (0.57877,0.63094) |
| Kappa No Prevalence | 0.42464 |
| Kappa Standard Error | 0.01331 |
| Kappa Unbiased | 0.60455 |
| Krippendorff Alpha | 0.60464 |
| Lambda A | 0.56403 |
| Lambda B | 0.56009 |
| Mutual Information | 0.79357 |
| NIR | 0.3454 |
| Overall ACC | 0.71232 |
| Overall CEN | 0.35489 |
| Overall J | (3.30948,0.47278) |
| Overall MCC | 0.60592 |
| Overall MCEN | 0.47525 |
| Overall RACC | 0.27197 |
| Overall RACCU | 0.27253 |
| P-Value | None |
| PPV Macro | 0.7237 |
| PPV Micro | 0.71232 |
| Pearson C | 0.82854 |
| Phi-Squared | 2.18954 |
| RCI | 0.35655 |
| RR | 311.85714 |
| Reference Entropy | 2.22569 |
| Response Entropy | 2.02155 |
| SOA1(Landis & Koch) | Substantial |
| SOA2(Fleiss) | Intermediate to Good |
| SOA3(Altman) | Good |
| SOA4(Cicchetti) | Good |
| SOA5(Cramer) | Strong |
| SOA6(Matthews) | Moderate |
| Scott PI | 0.60455 |
| Standard Error | 0.00969 |
| TNR Macro | 0.94281 |
| TNR Micro | 0.95205 |
| TPR Macro | 0.57457 |
| TPR Micro | 0.71232 |
| Zero-one Loss | 628 |

## Class Statistics :

|  |  |  |  |  |  |  |  |  |
| --- | --- | --- | --- | --- | --- | --- | --- | --- |
| Class | 0 | 1 | 2 | 3 | 4 | 5 | 6 | Description |
| ACC | 0.87082 | 0.82684 | 0.82547 | 0.97618 | 0.98259 | 0.98168 | 0.96106 | Accuracy |
| AGF | 0.80953 | 0.82374 | 0.79972 | 0.84748 | 0.79326 | 0.62295 | 0.38234 | Adjusted F-score |
| AGM | 0.85279 | 0.82916 | 0.8254 | 0.91204 | 0.88213 | 0.79023 | 0.67046 | Adjusted geometric mean |
| AM | 48 | 56 | 25 | -22 | -24 | -36 | -47 | Difference between automatic and manual classification |
| AUC | 0.81483 | 0.81731 | 0.80008 | 0.84836 | 0.79704 | 0.67194 | 0.56128 | Area under the ROC curve |
| AUCI | Very Good | Very Good | Very Good | Very Good | Good | Fair | Poor | AUC value interpretation |
| AUPR | 0.68654 | 0.75929 | 0.72112 | 0.77918 | 0.73266 | 0.62696 | 0.2382 | Area under the PR curve |
| BCD | 0.01099 | 0.01283 | 0.00573 | 0.00504 | 0.0055 | 0.00825 | 0.01077 | Bray-Curtis dissimilarity |
| BM | 0.62965 | 0.63462 | 0.60016 | 0.69671 | 0.59408 | 0.34389 | 0.12256 | Informedness or bookmaker informedness |
| CEN | 0.38437 | 0.32818 | 0.36014 | 0.29956 | 0.32287 | 0.38803 | 0.56806 | Confusion entropy |
| DOR | 25.28205 | 20.57177 | 17.83694 | 323.93514 | 444.94931 | 558.68421 | 16.65072 | Diagnostic odds ratio |
| DP | 0.77341 | 0.72404 | 0.68989 | 1.38409 | 1.46009 | 1.51459 | 0.67341 | Discriminant power |
| DPI | Poor | Poor | Poor | Limited | Limited | Limited | Poor | Discriminant power interpretation |
| ERR | 0.12918 | 0.17316 | 0.17453 | 0.02382 | 0.01741 | 0.01832 | 0.03894 | Error rate |
| F0.5 | 0.6632 | 0.74236 | 0.71304 | 0.81937 | 0.79585 | 0.68493 | 0.26042 | F0.5 score |
| F1 | 0.68456 | 0.75831 | 0.72088 | 0.77193 | 0.70769 | 0.5 | 0.19048 | F1 score - harmonic mean of precision and sensitivity |
| F2 | 0.70735 | 0.77496 | 0.72889 | 0.72968 | 0.63712 | 0.3937 | 0.15015 | F2 score |
| FDR | 0.35032 | 0.2679 | 0.29209 | 0.14563 | 0.13208 | 0.09091 | 0.65517 | False discovery rate |
| FN | 117 | 161 | 178 | 37 | 31 | 38 | 66 | False negative/miss/type 2 error |
| FNR | 0.2766 | 0.21353 | 0.26567 | 0.296 | 0.4026 | 0.65517 | 0.86842 | Miss rate or false negative rate |
| FOR | 0.06834 | 0.11726 | 0.11962 | 0.01779 | 0.01455 | 0.01758 | 0.03064 | False omission rate |
| FP | 165 | 217 | 203 | 15 | 7 | 2 | 19 | False positive/type 1 error/false alarm |
| FPR | 0.09375 | 0.15185 | 0.13417 | 0.00729 | 0.00332 | 0.00094 | 0.00902 | Fall-out or false positive rate |
| G | 0.68555 | 0.7588 | 0.721 | 0.77555 | 0.72007 | 0.55989 | 0.21301 | G-measure geometric mean of precision and sensitivity |
| GI | 0.62965 | 0.63462 | 0.60016 | 0.69671 | 0.59408 | 0.34389 | 0.12256 | Gini index |
| GM | 0.80968 | 0.81673 | 0.79737 | 0.83598 | 0.77163 | 0.58694 | 0.3611 | G-mean geometric mean of specificity and sensitivity |
| IBA | 0.53571 | 0.6259 | 0.55219 | 0.4971 | 0.35768 | 0.11912 | 0.01833 | Index of balanced accuracy |
| ICSI | 0.37309 | 0.51857 | 0.44224 | 0.55837 | 0.46533 | 0.25392 | -0.52359 | Individual classification success index |
| IS | 1.74539 | 1.08379 | 1.20572 | 3.89924 | 4.62095 | 5.09661 | 3.30812 | Information score |
| J | 0.52041 | 0.61071 | 0.56357 | 0.62857 | 0.54762 | 0.33333 | 0.10526 | Jaccard index |
| LS | 3.35285 | 2.11959 | 2.30653 | 14.9207 | 24.60622 | 34.2163 | 9.90472 | Lift score |
| MCC | 0.60502 | 0.62465 | 0.59419 | 0.76345 | 0.71202 | 0.55369 | 0.19623 | Matthews correlation coefficient |
| MCCI | Moderate | Moderate | Moderate | Strong | Strong | Moderate | Negligible | Matthews correlation coefficient interpretation |
| MCEN | 0.50354 | 0.45392 | 0.4846 | 0.41496 | 0.42018 | 0.44019 | 0.59182 | Modified confusion entropy |
| MK | 0.58134 | 0.61484 | 0.58829 | 0.83658 | 0.85337 | 0.89151 | 0.31419 | Markedness |
| N | 1760 | 1429 | 1513 | 2058 | 2106 | 2125 | 2107 | Condition negative |
| NLR | 0.30521 | 0.25176 | 0.30684 | 0.29817 | 0.40394 | 0.65579 | 0.87632 | Negative likelihood ratio |
| NLRI | Poor | Poor | Poor | Poor | Poor | Negligible | Negligible | Negative likelihood ratio interpretation |
| NPV | 0.93166 | 0.88274 | 0.88038 | 0.98221 | 0.98545 | 0.98242 | 0.96936 | Negative predictive value |
| OC | 0.7234 | 0.78647 | 0.73433 | 0.85437 | 0.86792 | 0.90909 | 0.34483 | Overlap coefficient |
| OOC | 0.68555 | 0.7588 | 0.721 | 0.77555 | 0.72007 | 0.55989 | 0.21301 | Otsuka-Ochiai coefficient |
| OP | 0.75862 | 0.78911 | 0.74329 | 0.80602 | 0.73212 | 0.49486 | 0.19549 | Optimized precision |
| P | 423 | 754 | 670 | 125 | 77 | 58 | 76 | Condition positive or support |
| PLR | 7.71631 | 5.17912 | 5.4731 | 96.5888 | 179.73284 | 366.37931 | 14.59141 | Positive likelihood ratio |
| PLRI | Fair | Fair | Fair | Good | Good | Good | Good | Positive likelihood ratio interpretation |
| POP | 2183 | 2183 | 2183 | 2183 | 2183 | 2183 | 2183 | Population |
| PPV | 0.64968 | 0.7321 | 0.70791 | 0.85437 | 0.86792 | 0.90909 | 0.34483 | Precision or positive predictive value |
| PRE | 0.19377 | 0.3454 | 0.30692 | 0.05726 | 0.03527 | 0.02657 | 0.03481 | Prevalence |
| Q | 0.9239 | 0.90729 | 0.89383 | 0.99384 | 0.99552 | 0.99643 | 0.88669 | Yule Q - coefficient of colligation |
| QI | Strong | Strong | Strong | Strong | Strong | Strong | Strong | Yule Q interpretation |
| RACC | 0.04181 | 0.12816 | 0.09771 | 0.0027 | 0.00086 | 0.00027 | 0.00046 | Random accuracy |
| RACCU | 0.04193 | 0.12832 | 0.09775 | 0.00273 | 0.00089 | 0.00034 | 0.00058 | Random accuracy unbiased |
| TN | 1595 | 1212 | 1310 | 2043 | 2099 | 2123 | 2088 | True negative/correct rejection |
| TNR | 0.90625 | 0.84815 | 0.86583 | 0.99271 | 0.99668 | 0.99906 | 0.99098 | Specificity or true negative rate |
| TON | 1712 | 1373 | 1488 | 2080 | 2130 | 2161 | 2154 | Test outcome negative |
| TOP | 471 | 810 | 695 | 103 | 53 | 22 | 29 | Test outcome positive |
| TP | 306 | 593 | 492 | 88 | 46 | 20 | 10 | True positive/hit |
| TPR | 0.7234 | 0.78647 | 0.73433 | 0.704 | 0.5974 | 0.34483 | 0.13158 | Sensitivity, recall, hit rate, or true positive rate |
| Y | 0.62965 | 0.63462 | 0.60016 | 0.69671 | 0.59408 | 0.34389 | 0.12256 | Youden index |
| dInd | 0.29205 | 0.26202 | 0.29763 | 0.29609 | 0.40261 | 0.65517 | 0.86847 | Distance index |
| sInd | 0.79349 | 0.81472 | 0.78954 | 0.79063 | 0.71531 | 0.53672 | 0.3859 | Similarity index |

Generated By PyCM Version 3.1
