# Supplementary material for: TopEC: prediction of Enzyme Commission classes by 3D graph neural networks and localized 3D protein descriptor
Source: Nat Commun. 2025 Mar 20;16:2737. doi: 10.1038/s41467-025-57324-5 (PMC11923149; doi:10.1038/s41467-025-57324-5)
Supplement: Supplementary file 3 — Supplementary Data 1 [file 41467_2025_57324_MOESM3_ESM.zip › Data_S1/table1/mainclass/EnzyNet/full_struc/BindingMOAD_TEMP_flips.html]

PDB\_TEMP\_enzynet\_flips


# PyCM Report

## Dataset Type :

- Multi-Class Classification
- Imbalanced

Note 1 : Recommended statistics for this type of classification highlighted in aqua

Note 2 : The recommender system assumes that the input is the result of classification over the whole data rather than just a part of it.
If the confusion matrix is the result of test data classification, the recommendation is not valid.

## Confusion Matrix :

|  |  |  |  |  |  |  |  |  |  |  |  |  |  |  |  |  |  |  |  |  |  |  |  |  |  |  |  |  |  |  |  |  |  |  |  |  |  |  |  |  |  |  |  |  |  |  |  |  |  |  |  |  |  |  |  |  |  |  |  |  |  |  |  |  |  |
| --- | --- | --- | --- | --- | --- | --- | --- | --- | --- | --- | --- | --- | --- | --- | --- | --- | --- | --- | --- | --- | --- | --- | --- | --- | --- | --- | --- | --- | --- | --- | --- | --- | --- | --- | --- | --- | --- | --- | --- | --- | --- | --- | --- | --- | --- | --- | --- | --- | --- | --- | --- | --- | --- | --- | --- | --- | --- | --- | --- | --- | --- | --- | --- | --- | --- |
| Actual | Predict  |  |  |  |  |  |  |  |  | | --- | --- | --- | --- | --- | --- | --- | --- | |  | 0 | 1 | 2 | 3 | 4 | 5 | 6 | | 0 | 299 | 68 | 50 | 4 | 2 | 0 | 0 | | 1 | 56 | 592 | 96 | 4 | 2 | 0 | 4 | | 2 | 79 | 68 | 501 | 7 | 0 | 0 | 15 | | 3 | 21 | 8 | 10 | 86 | 0 | 0 | 0 | | 4 | 8 | 12 | 15 | 0 | 42 | 0 | 0 | | 5 | 12 | 20 | 8 | 0 | 0 | 18 | 0 | | 6 | 14 | 33 | 23 | 1 | 0 | 0 | 5 | |

## Overall Statistics :

|  |  |
| --- | --- |
| 95% CI | (0.68773,0.72592) |
| ACC Macro | 0.91624 |
| ARI | 0.39605 |
| AUNP | 0.79679 |
| AUNU | 0.74592 |
| Bangdiwala B | 0.53835 |
| Bennett S | 0.65796 |
| CBA | 0.52468 |
| CSI | 0.26815 |
| Chi-Squared | 4595.82868 |
| Chi-Squared DF | 36 |
| Conditional Entropy | 1.21907 |
| Cramer V | 0.59235 |
| Cross Entropy | 2.2795 |
| F1 Macro | 0.59446 |
| F1 Micro | 0.70683 |
| FNR Macro | 0.45009 |
| FNR Micro | 0.29317 |
| FPR Macro | 0.05806 |
| FPR Micro | 0.04886 |
| Gwet AC1 | 0.66645 |
| Hamming Loss | 0.29317 |
| Joint Entropy | 3.44476 |
| KL Divergence | 0.05381 |
| Kappa | 0.59673 |
| Kappa 95% CI | (0.57047,0.623) |
| Kappa No Prevalence | 0.41365 |
| Kappa Standard Error | 0.0134 |
| Kappa Unbiased | 0.59634 |
| Krippendorff Alpha | 0.59644 |
| Lambda A | 0.55913 |
| Lambda B | 0.55861 |
| Mutual Information | 0.77406 |
| NIR | 0.3454 |
| Overall ACC | 0.70683 |
| Overall CEN | 0.35708 |
| Overall J | (3.16848,0.45264) |
| Overall MCC | 0.59806 |
| Overall MCEN | 0.47687 |
| Overall RACC | 0.273 |
| Overall RACCU | 0.2737 |
| P-Value | None |
| PPV Macro | 0.71824 |
| PPV Micro | 0.70683 |
| Pearson C | 0.82339 |
| Phi-Squared | 2.10528 |
| RCI | 0.34778 |
| RR | 311.85714 |
| Reference Entropy | 2.22569 |
| Response Entropy | 1.99312 |
| SOA1(Landis & Koch) | Moderate |
| SOA2(Fleiss) | Intermediate to Good |
| SOA3(Altman) | Moderate |
| SOA4(Cicchetti) | Good |
| SOA5(Cramer) | Relatively Strong |
| SOA6(Matthews) | Moderate |
| Scott PI | 0.59634 |
| Standard Error | 0.00974 |
| TNR Macro | 0.94194 |
| TNR Micro | 0.95114 |
| TPR Macro | 0.54991 |
| TPR Micro | 0.70683 |
| Zero-one Loss | 640 |

## Class Statistics :

|  |  |  |  |  |  |  |  |  |
| --- | --- | --- | --- | --- | --- | --- | --- | --- |
| Class | 0 | 1 | 2 | 3 | 4 | 5 | 6 | Description |
| ACC | 0.85616 | 0.83005 | 0.83005 | 0.97481 | 0.98213 | 0.98168 | 0.95877 | Accuracy |
| AGF | 0.79396 | 0.82459 | 0.80809 | 0.83812 | 0.76499 | 0.59553 | 0.27216 | Adjusted F-score |
| AGM | 0.8378 | 0.83258 | 0.83014 | 0.90678 | 0.86564 | 0.77556 | 0.61664 | Adjusted geometric mean |
| AM | 66 | 47 | 33 | -23 | -31 | -40 | -52 | Difference between automatic and manual classification |
| AUC | 0.79945 | 0.81944 | 0.80713 | 0.84011 | 0.77178 | 0.65517 | 0.52839 | Area under the ROC curve |
| AUCI | Good | Very Good | Very Good | Very Good | Good | Fair | Poor | AUC value interpretation |
| AUPR | 0.65915 | 0.76211 | 0.73021 | 0.76557 | 0.72925 | 0.65517 | 0.13706 | Area under the PR curve |
| BCD | 0.01512 | 0.01077 | 0.00756 | 0.00527 | 0.0071 | 0.00916 | 0.01191 | Bray-Curtis dissimilarity |
| BM | 0.5989 | 0.63889 | 0.61425 | 0.68023 | 0.54356 | 0.31034 | 0.05677 | Informedness or bookmaker informedness |
| CEN | 0.41157 | 0.32476 | 0.35402 | 0.30149 | 0.32007 | 0.35403 | 0.5789 | Confusion entropy |
| DOR | 19.92487 | 21.33144 | 19.23988 | 281.42949 | 630.6 | None | 7.73907 | Diagnostic odds ratio |
| DP | 0.71639 | 0.73273 | 0.70802 | 1.35041 | 1.54359 | None | 0.48996 | Discriminant power |
| DPI | Poor | Poor | Poor | Limited | Limited | None | Poor | Discriminant power interpretation |
| ERR | 0.14384 | 0.16995 | 0.16995 | 0.02519 | 0.01787 | 0.01832 | 0.04123 | Error rate |
| F0.5 | 0.62842 | 0.74785 | 0.71941 | 0.80675 | 0.8046 | 0.69231 | 0.14535 | F0.5 score |
| F1 | 0.6557 | 0.76141 | 0.72979 | 0.75771 | 0.68293 | 0.47368 | 0.1 | F1 score - harmonic mean of precision and sensitivity |
| F2 | 0.68547 | 0.77548 | 0.74047 | 0.71429 | 0.59322 | 0.36 | 0.07622 | F2 score |
| FDR | 0.38855 | 0.26092 | 0.28734 | 0.15686 | 0.08696 | 0.0 | 0.79167 | False discovery rate |
| FN | 124 | 162 | 169 | 39 | 35 | 40 | 71 | False negative/miss/type 2 error |
| FNR | 0.29314 | 0.21485 | 0.25224 | 0.312 | 0.45455 | 0.68966 | 0.93421 | Miss rate or false negative rate |
| FOR | 0.0732 | 0.11722 | 0.11419 | 0.01874 | 0.01638 | 0.01848 | 0.03289 | False omission rate |
| FP | 190 | 209 | 202 | 16 | 4 | 0 | 19 | False positive/type 1 error/false alarm |
| FPR | 0.10795 | 0.14626 | 0.13351 | 0.00777 | 0.0019 | 0.0 | 0.00902 | Fall-out or false positive rate |
| G | 0.65743 | 0.76176 | 0.73 | 0.76163 | 0.70571 | 0.55709 | 0.11707 | G-measure geometric mean of precision and sensitivity |
| GI | 0.5989 | 0.63889 | 0.61425 | 0.68023 | 0.54356 | 0.31034 | 0.05677 | Gini index |
| GM | 0.79407 | 0.81873 | 0.80494 | 0.82623 | 0.73785 | 0.55709 | 0.25534 | G-mean geometric mean of specificity and sensitivity |
| IBA | 0.51378 | 0.62433 | 0.571 | 0.47497 | 0.29799 | 0.09631 | 0.00488 | Index of balanced accuracy |
| ICSI | 0.31831 | 0.52422 | 0.46042 | 0.53114 | 0.4585 | 0.31034 | -0.72588 | Individual classification success index |
| IS | 1.65789 | 1.09747 | 1.21537 | 3.88015 | 4.69407 | 5.23412 | 2.58113 | Information score |
| J | 0.48777 | 0.61475 | 0.57454 | 0.60993 | 0.51852 | 0.31034 | 0.05263 | Jaccard index |
| LS | 3.15555 | 2.13979 | 2.322 | 14.72455 | 25.88538 | 37.63793 | 5.9841 | Lift score |
| MCC | 0.56777 | 0.63031 | 0.60631 | 0.74885 | 0.69813 | 0.55192 | 0.0998 | Matthews correlation coefficient |
| MCCI | Moderate | Moderate | Moderate | Strong | Moderate | Moderate | Negligible | Matthews correlation coefficient interpretation |
| MCEN | 0.53042 | 0.45011 | 0.4797 | 0.41063 | 0.40509 | 0.38889 | 0.58981 | Modified confusion entropy |
| MK | 0.53825 | 0.62185 | 0.59847 | 0.8244 | 0.89667 | 0.98152 | 0.17545 | Markedness |
| N | 1760 | 1429 | 1513 | 2058 | 2106 | 2125 | 2107 | Condition negative |
| NLR | 0.32862 | 0.25166 | 0.2911 | 0.31444 | 0.45541 | 0.68966 | 0.94271 | Negative likelihood ratio |
| NLRI | Poor | Poor | Poor | Poor | Poor | Negligible | Negligible | Negative likelihood ratio interpretation |
| NPV | 0.9268 | 0.88278 | 0.88581 | 0.98126 | 0.98362 | 0.98152 | 0.96711 | Negative predictive value |
| OC | 0.70686 | 0.78515 | 0.74776 | 0.84314 | 0.91304 | 1.0 | 0.20833 | Overlap coefficient |
| OOC | 0.65743 | 0.76176 | 0.73 | 0.76163 | 0.70571 | 0.55709 | 0.11707 | Otsuka-Ochiai coefficient |
| OP | 0.74034 | 0.78819 | 0.7565 | 0.79374 | 0.68889 | 0.45536 | 0.08328 | Optimized precision |
| P | 423 | 754 | 670 | 125 | 77 | 58 | 76 | Condition positive or support |
| PLR | 6.54772 | 5.36829 | 5.60081 | 88.494 | 287.18182 | None | 7.29571 | Positive likelihood ratio |
| PLRI | Fair | Fair | Fair | Good | Good | None | Fair | Positive likelihood ratio interpretation |
| POP | 2183 | 2183 | 2183 | 2183 | 2183 | 2183 | 2183 | Population |
| PPV | 0.61145 | 0.73908 | 0.71266 | 0.84314 | 0.91304 | 1.0 | 0.20833 | Precision or positive predictive value |
| PRE | 0.19377 | 0.3454 | 0.30692 | 0.05726 | 0.03527 | 0.02657 | 0.03481 | Prevalence |
| Q | 0.90442 | 0.91044 | 0.90119 | 0.99292 | 0.99683 | None | 0.77114 | Yule Q - coefficient of colligation |
| QI | Strong | Strong | Strong | Strong | Strong | None | Strong | Yule Q interpretation |
| RACC | 0.04341 | 0.12673 | 0.09884 | 0.00268 | 0.00074 | 0.00022 | 0.00038 | Random accuracy |
| RACCU | 0.04363 | 0.12685 | 0.09889 | 0.0027 | 0.00079 | 0.0003 | 0.00052 | Random accuracy unbiased |
| TN | 1570 | 1220 | 1311 | 2042 | 2102 | 2125 | 2088 | True negative/correct rejection |
| TNR | 0.89205 | 0.85374 | 0.86649 | 0.99223 | 0.9981 | 1.0 | 0.99098 | Specificity or true negative rate |
| TON | 1694 | 1382 | 1480 | 2081 | 2137 | 2165 | 2159 | Test outcome negative |
| TOP | 489 | 801 | 703 | 102 | 46 | 18 | 24 | Test outcome positive |
| TP | 299 | 592 | 501 | 86 | 42 | 18 | 5 | True positive/hit |
| TPR | 0.70686 | 0.78515 | 0.74776 | 0.688 | 0.54545 | 0.31034 | 0.06579 | Sensitivity, recall, hit rate, or true positive rate |
| Y | 0.5989 | 0.63889 | 0.61425 | 0.68023 | 0.54356 | 0.31034 | 0.05677 | Youden index |
| dInd | 0.31239 | 0.25991 | 0.28539 | 0.3121 | 0.45455 | 0.68966 | 0.93425 | Distance index |
| sInd | 0.77911 | 0.81622 | 0.7982 | 0.77931 | 0.67859 | 0.51234 | 0.33938 | Similarity index |

Generated By PyCM Version 3.1
