# Supplementary material for: TopEC: prediction of Enzyme Commission classes by 3D graph neural networks and localized 3D protein descriptor
Source: Nat Commun. 2025 Mar 20;16:2737. doi: 10.1038/s41467-025-57324-5 (PMC11923149; doi:10.1038/s41467-025-57324-5)
Supplement: Supplementary file 3 — Supplementary Data 1 [file 41467_2025_57324_MOESM3_ESM.zip › Data_S1/table1/mainclass/EnzyNet/full_struc/BindingMOAD_TEMP.html]

PDB\_TEMP\_enzynet\_none


# PyCM Report

## Dataset Type :

- Multi-Class Classification
- Imbalanced

Note 1 : Recommended statistics for this type of classification highlighted in aqua

Note 2 : The recommender system assumes that the input is the result of classification over the whole data rather than just a part of it.
If the confusion matrix is the result of test data classification, the recommendation is not valid.

## Confusion Matrix :

|  |  |  |  |  |  |  |  |  |  |  |  |  |  |  |  |  |  |  |  |  |  |  |  |  |  |  |  |  |  |  |  |  |  |  |  |  |  |  |  |  |  |  |  |  |  |  |  |  |  |  |  |  |  |  |  |  |  |  |  |  |  |  |  |  |  |
| --- | --- | --- | --- | --- | --- | --- | --- | --- | --- | --- | --- | --- | --- | --- | --- | --- | --- | --- | --- | --- | --- | --- | --- | --- | --- | --- | --- | --- | --- | --- | --- | --- | --- | --- | --- | --- | --- | --- | --- | --- | --- | --- | --- | --- | --- | --- | --- | --- | --- | --- | --- | --- | --- | --- | --- | --- | --- | --- | --- | --- | --- | --- | --- | --- | --- |
| Actual | Predict  |  |  |  |  |  |  |  |  | | --- | --- | --- | --- | --- | --- | --- | --- | |  | 0 | 1 | 2 | 3 | 4 | 5 | 6 | | 0 | 297 | 66 | 52 | 5 | 2 | 1 | 0 | | 1 | 64 | 569 | 92 | 18 | 4 | 2 | 5 | | 2 | 69 | 94 | 460 | 15 | 8 | 6 | 18 | | 3 | 13 | 7 | 12 | 92 | 1 | 0 | 0 | | 4 | 3 | 13 | 13 | 1 | 47 | 0 | 0 | | 5 | 6 | 15 | 16 | 0 | 0 | 20 | 1 | | 6 | 14 | 28 | 19 | 2 | 1 | 0 | 12 | |

## Overall Statistics :

|  |  |
| --- | --- |
| 95% CI | (0.66628,0.70523) |
| ACC Macro | 0.91022 |
| ARI | 0.35854 |
| AUNP | 0.78497 |
| AUNU | 0.75446 |
| Bangdiwala B | 0.50178 |
| Bennett S | 0.63338 |
| CBA | 0.5496 |
| CSI | 0.21453 |
| Chi-Squared | 4102.59729 |
| Chi-Squared DF | 36 |
| Conditional Entropy | 1.3868 |
| Cramer V | 0.55966 |
| Cross Entropy | 2.2473 |
| F1 Macro | 0.59323 |
| F1 Micro | 0.68575 |
| FNR Macro | 0.42965 |
| FNR Micro | 0.31425 |
| FPR Macro | 0.06144 |
| FPR Micro | 0.05237 |
| Gwet AC1 | 0.64194 |
| Hamming Loss | 0.31425 |
| Joint Entropy | 3.61249 |
| KL Divergence | 0.0216 |
| Kappa | 0.57218 |
| Kappa 95% CI | (0.54567,0.5987) |
| Kappa No Prevalence | 0.37151 |
| Kappa Standard Error | 0.01353 |
| Kappa Unbiased | 0.572 |
| Krippendorff Alpha | 0.5721 |
| Lambda A | 0.52414 |
| Lambda B | 0.51833 |
| Mutual Information | 0.71579 |
| NIR | 0.3454 |
| Overall ACC | 0.68575 |
| Overall CEN | 0.38914 |
| Overall J | (3.0885,0.44121) |
| Overall MCC | 0.57272 |
| Overall MCEN | 0.5142 |
| Overall RACC | 0.26546 |
| Overall RACCU | 0.26578 |
| P-Value | None |
| PPV Macro | 0.64418 |
| PPV Micro | 0.68575 |
| Pearson C | 0.8079 |
| Phi-Squared | 1.87934 |
| RCI | 0.3216 |
| RR | 311.85714 |
| Reference Entropy | 2.22569 |
| Response Entropy | 2.10258 |
| SOA1(Landis & Koch) | Moderate |
| SOA2(Fleiss) | Intermediate to Good |
| SOA3(Altman) | Moderate |
| SOA4(Cicchetti) | Fair |
| SOA5(Cramer) | Relatively Strong |
| SOA6(Matthews) | Moderate |
| Scott PI | 0.572 |
| Standard Error | 0.00994 |
| TNR Macro | 0.93856 |
| TNR Micro | 0.94763 |
| TPR Macro | 0.57035 |
| TPR Micro | 0.68575 |
| Zero-one Loss | 686 |

## Class Statistics :

|  |  |  |  |  |  |  |  |  |
| --- | --- | --- | --- | --- | --- | --- | --- | --- |
| Class | 0 | 1 | 2 | 3 | 4 | 5 | 6 | Description |
| ACC | 0.86486 | 0.8131 | 0.81035 | 0.9661 | 0.97893 | 0.97847 | 0.95969 | Accuracy |
| AGF | 0.79653 | 0.80264 | 0.77018 | 0.84525 | 0.79075 | 0.61433 | 0.41455 | Adjusted F-score |
| AGM | 0.84458 | 0.81621 | 0.80938 | 0.91277 | 0.88343 | 0.78811 | 0.68659 | Adjusted geometric mean |
| AM | 43 | 38 | -6 | 8 | -14 | -29 | -40 | Difference between automatic and manual classification |
| AUC | 0.80305 | 0.79929 | 0.77587 | 0.85804 | 0.8014 | 0.6703 | 0.57325 | Area under the ROC curve |
| AUCI | Very Good | Good | Good | Very Good | Very Good | Fair | Poor | AUC value interpretation |
| AUPR | 0.66973 | 0.73654 | 0.68967 | 0.71386 | 0.67821 | 0.51724 | 0.24561 | Area under the PR curve |
| BCD | 0.00985 | 0.0087 | 0.00137 | 0.00183 | 0.00321 | 0.00664 | 0.00916 | Bray-Curtis dissimilarity |
| BM | 0.6061 | 0.59859 | 0.55174 | 0.71608 | 0.60279 | 0.34059 | 0.1465 | Informedness or bookmaker informedness |
| CEN | 0.39338 | 0.35465 | 0.40874 | 0.36196 | 0.3845 | 0.47194 | 0.60212 | Confusion entropy |
| DOR | 22.19062 | 16.63347 | 14.05556 | 137.15004 | 204.64583 | 123.74269 | 16.27344 | Diagnostic odds ratio |
| DP | 0.74218 | 0.67316 | 0.63284 | 1.1783 | 1.27412 | 1.15367 | 0.66792 | Discriminant power |
| DPI | Poor | Poor | Poor | Limited | Limited | Limited | Poor | Discriminant power interpretation |
| ERR | 0.13514 | 0.1869 | 0.18965 | 0.0339 | 0.02107 | 0.02153 | 0.04031 | Error rate |
| F0.5 | 0.64932 | 0.7254 | 0.69152 | 0.70015 | 0.71429 | 0.57471 | 0.27273 | F0.5 score |
| F1 | 0.66817 | 0.73609 | 0.68966 | 0.71318 | 0.67143 | 0.45977 | 0.21429 | F1 score - harmonic mean of precision and sensitivity |
| F2 | 0.68814 | 0.74711 | 0.6878 | 0.7267 | 0.63342 | 0.38314 | 0.17647 | F2 score |
| FDR | 0.36266 | 0.28157 | 0.30723 | 0.30827 | 0.25397 | 0.31034 | 0.66667 | False discovery rate |
| FN | 126 | 185 | 210 | 33 | 30 | 38 | 64 | False negative/miss/type 2 error |
| FNR | 0.29787 | 0.24536 | 0.31343 | 0.264 | 0.38961 | 0.65517 | 0.84211 | Miss rate or false negative rate |
| FOR | 0.07338 | 0.133 | 0.13825 | 0.0161 | 0.01415 | 0.01764 | 0.02981 | False omission rate |
| FP | 169 | 223 | 204 | 41 | 16 | 9 | 24 | False positive/type 1 error/false alarm |
| FPR | 0.09602 | 0.15605 | 0.13483 | 0.01992 | 0.0076 | 0.00424 | 0.01139 | Fall-out or false positive rate |
| G | 0.66895 | 0.73632 | 0.68966 | 0.71352 | 0.67481 | 0.48766 | 0.22942 | G-measure geometric mean of precision and sensitivity |
| GI | 0.6061 | 0.59859 | 0.55174 | 0.71608 | 0.60279 | 0.34059 | 0.1465 | Gini index |
| GM | 0.79669 | 0.79805 | 0.77071 | 0.84932 | 0.7783 | 0.58598 | 0.39509 | G-mean geometric mean of specificity and sensitivity |
| IBA | 0.50659 | 0.58 | 0.48791 | 0.54527 | 0.37435 | 0.11986 | 0.02642 | Index of balanced accuracy |
| ICSI | 0.33947 | 0.47308 | 0.37934 | 0.42773 | 0.35642 | 0.03448 | -0.50877 | Individual classification success index |
| IS | 1.71772 | 1.0566 | 1.17453 | 3.59459 | 4.40262 | 4.69806 | 3.25921 | Information score |
| J | 0.50169 | 0.5824 | 0.52632 | 0.55422 | 0.50538 | 0.29851 | 0.12 | Jaccard index |
| LS | 3.28915 | 2.08003 | 2.25719 | 12.08036 | 21.15048 | 25.95719 | 9.57456 | Lift score |
| MCC | 0.58465 | 0.59198 | 0.55313 | 0.69556 | 0.66421 | 0.47842 | 0.21087 | Matthews correlation coefficient |
| MCCI | Moderate | Moderate | Moderate | Moderate | Moderate | Weak | Negligible | Matthews correlation coefficient interpretation |
| MCEN | 0.5092 | 0.48408 | 0.54326 | 0.48345 | 0.4974 | 0.53908 | 0.63424 | Modified confusion entropy |
| MK | 0.56396 | 0.58544 | 0.55452 | 0.67563 | 0.73188 | 0.67201 | 0.30352 | Markedness |
| N | 1760 | 1429 | 1513 | 2058 | 2106 | 2125 | 2107 | Condition negative |
| NLR | 0.32951 | 0.29073 | 0.36228 | 0.26937 | 0.39259 | 0.65796 | 0.85181 | Negative likelihood ratio |
| NLRI | Poor | Poor | Poor | Poor | Poor | Negligible | Negligible | Negative likelihood ratio interpretation |
| NPV | 0.92662 | 0.867 | 0.86175 | 0.9839 | 0.98585 | 0.98236 | 0.97019 | Negative predictive value |
| OC | 0.70213 | 0.75464 | 0.69277 | 0.736 | 0.74603 | 0.68966 | 0.33333 | Overlap coefficient |
| OOC | 0.66895 | 0.73632 | 0.68966 | 0.71352 | 0.67481 | 0.48766 | 0.22942 | Otsuka-Ochiai coefficient |
| OP | 0.73919 | 0.75724 | 0.69525 | 0.82387 | 0.74059 | 0.49291 | 0.23513 | Optimized precision |
| P | 423 | 754 | 670 | 125 | 77 | 58 | 76 | Condition positive or support |
| PLR | 7.3121 | 4.8358 | 5.09204 | 36.94361 | 80.34253 | 81.41762 | 13.86184 | Positive likelihood ratio |
| PLRI | Fair | Poor | Fair | Good | Good | Good | Good | Positive likelihood ratio interpretation |
| POP | 2183 | 2183 | 2183 | 2183 | 2183 | 2183 | 2183 | Population |
| PPV | 0.63734 | 0.71843 | 0.69277 | 0.69173 | 0.74603 | 0.68966 | 0.33333 | Precision or positive predictive value |
| PRE | 0.19377 | 0.3454 | 0.30692 | 0.05726 | 0.03527 | 0.02657 | 0.03481 | Prevalence |
| Q | 0.91376 | 0.88658 | 0.86716 | 0.98552 | 0.99027 | 0.98397 | 0.88422 | Yule Q - coefficient of colligation |
| QI | Strong | Strong | Strong | Strong | Strong | Strong | Strong | Yule Q interpretation |
| RACC | 0.04136 | 0.12531 | 0.09335 | 0.00349 | 0.00102 | 0.00035 | 0.00057 | Random accuracy |
| RACCU | 0.04146 | 0.12539 | 0.09336 | 0.00349 | 0.00103 | 0.0004 | 0.00066 | Random accuracy unbiased |
| TN | 1591 | 1206 | 1309 | 2017 | 2090 | 2116 | 2083 | True negative/correct rejection |
| TNR | 0.90398 | 0.84395 | 0.86517 | 0.98008 | 0.9924 | 0.99576 | 0.98861 | Specificity or true negative rate |
| TON | 1717 | 1391 | 1519 | 2050 | 2120 | 2154 | 2147 | Test outcome negative |
| TOP | 466 | 792 | 664 | 133 | 63 | 29 | 36 | Test outcome positive |
| TP | 297 | 569 | 460 | 92 | 47 | 20 | 12 | True positive/hit |
| TPR | 0.70213 | 0.75464 | 0.68657 | 0.736 | 0.61039 | 0.34483 | 0.15789 | Sensitivity, recall, hit rate, or true positive rate |
| Y | 0.6061 | 0.59859 | 0.55174 | 0.71608 | 0.60279 | 0.34059 | 0.1465 | Youden index |
| dInd | 0.31297 | 0.29078 | 0.3412 | 0.26475 | 0.38968 | 0.65519 | 0.84218 | Distance index |
| sInd | 0.7787 | 0.79439 | 0.75873 | 0.81279 | 0.72445 | 0.53671 | 0.40449 | Similarity index |

Generated By PyCM Version 3.1
