# Supplementary material for: TopEC: prediction of Enzyme Commission classes by 3D graph neural networks and localized 3D protein descriptor
Source: Nat Commun. 2025 Mar 20;16:2737. doi: 10.1038/s41467-025-57324-5 (PMC11923149; doi:10.1038/s41467-025-57324-5)
Supplement: Supplementary file 3 — Supplementary Data 1 [file 41467_2025_57324_MOESM3_ESM.zip › Data_S1/table1/mainclass/EnzyNet/full_struc/BindingMOAD_FOLD_wflips.html]

PDB\_FOLD\_enzynet\_wflips


# PyCM Report

## Dataset Type :

- Multi-Class Classification
- Imbalanced

Note 1 : Recommended statistics for this type of classification highlighted in aqua

Note 2 : The recommender system assumes that the input is the result of classification over the whole data rather than just a part of it.
If the confusion matrix is the result of test data classification, the recommendation is not valid.

## Confusion Matrix :

|  |  |  |  |  |  |  |  |  |  |  |  |  |  |  |  |  |  |  |  |  |  |  |  |  |  |  |  |  |  |  |  |  |  |  |  |  |  |  |  |  |  |  |  |  |  |  |  |  |  |  |  |  |  |  |  |  |  |  |  |  |  |  |  |  |  |
| --- | --- | --- | --- | --- | --- | --- | --- | --- | --- | --- | --- | --- | --- | --- | --- | --- | --- | --- | --- | --- | --- | --- | --- | --- | --- | --- | --- | --- | --- | --- | --- | --- | --- | --- | --- | --- | --- | --- | --- | --- | --- | --- | --- | --- | --- | --- | --- | --- | --- | --- | --- | --- | --- | --- | --- | --- | --- | --- | --- | --- | --- | --- | --- | --- | --- |
| Actual | Predict  |  |  |  |  |  |  |  |  | | --- | --- | --- | --- | --- | --- | --- | --- | |  | 0 | 1 | 2 | 3 | 4 | 5 | 6 | | 0 | 236 | 83 | 86 | 1 | 1 | 0 | 0 | | 1 | 28 | 672 | 103 | 2 | 1 | 1 | 0 | | 2 | 73 | 210 | 115 | 1 | 1 | 3 | 0 | | 3 | 44 | 62 | 26 | 0 | 0 | 0 | 0 | | 4 | 69 | 47 | 42 | 0 | 13 | 0 | 0 | | 5 | 7 | 23 | 17 | 0 | 0 | 1 | 0 | | 6 | 1 | 14 | 2 | 0 | 0 | 0 | 0 | |

## Overall Statistics :

|  |  |
| --- | --- |
| 95% CI | (0.50044,0.54439) |
| ACC Macro | 0.86355 |
| ARI | 0.217 |
| AUNP | 0.65315 |
| AUNU | 0.57865 |
| Bangdiwala B | 0.41853 |
| Bennett S | 0.44282 |
| CBA | 0.21462 |
| CSI | None |
| Chi-Squared | None |
| Chi-Squared DF | 36 |
| Conditional Entropy | 1.2416 |
| Cramer V | None |
| Cross Entropy | 2.65345 |
| F1 Macro | 0.24469 |
| F1 Micro | 0.52242 |
| FNR Macro | 0.7436 |
| FNR Micro | 0.47758 |
| FPR Macro | 0.0991 |
| FPR Micro | 0.0796 |
| Gwet AC1 | 0.46183 |
| Hamming Loss | 0.47758 |
| Joint Entropy | 3.45866 |
| KL Divergence | None |
| Kappa | 0.30205 |
| Kappa 95% CI | (0.26994,0.33417) |
| Kappa No Prevalence | 0.04484 |
| Kappa Standard Error | 0.01638 |
| Kappa Unbiased | 0.29303 |
| Krippendorff Alpha | 0.2932 |
| Lambda A | 0.19864 |
| Lambda B | 0.20023 |
| Mutual Information | 0.27273 |
| NIR | 0.40655 |
| Overall ACC | 0.52242 |
| Overall CEN | 0.4442 |
| Overall J | (1.17783,0.16826) |
| Overall MCC | 0.3118 |
| Overall MCEN | 0.53627 |
| Overall RACC | 0.31573 |
| Overall RACCU | 0.32447 |
| P-Value | None |
| PPV Macro | None |
| PPV Micro | 0.52242 |
| Pearson C | None |
| Phi-Squared | None |
| RCI | 0.12301 |
| RR | 283.57143 |
| Reference Entropy | 2.21706 |
| Response Entropy | 1.51433 |
| SOA1(Landis & Koch) | Fair |
| SOA2(Fleiss) | Poor |
| SOA3(Altman) | Fair |
| SOA4(Cicchetti) | Poor |
| SOA5(Cramer) | None |
| SOA6(Matthews) | Weak |
| Scott PI | 0.29303 |
| Standard Error | 0.01121 |
| TNR Macro | 0.9009 |
| TNR Micro | 0.9204 |
| TPR Macro | 0.2564 |
| TPR Micro | 0.52242 |
| Zero-one Loss | 948 |

## Class Statistics :

|  |  |  |  |  |  |  |  |  |
| --- | --- | --- | --- | --- | --- | --- | --- | --- |
| Class | 0 | 1 | 2 | 3 | 4 | 5 | 6 | Description |
| ACC | 0.80202 | 0.71083 | 0.71587 | 0.93149 | 0.91889 | 0.97431 | 0.99144 | Accuracy |
| AGF | 0.7064 | 0.78242 | 0.48534 | 0.0 | 0.29457 | 0.15775 | 0.0 | Adjusted F-score |
| AGM | 0.77384 | 0.68722 | 0.63623 | 0 | 0.62065 | 0.56584 | 0 | Adjusted geometric mean |
| AM | 51 | 304 | -12 | -128 | -155 | -43 | -17 | Difference between automatic and manual classification |
| AUC | 0.71958 | 0.73002 | 0.55545 | 0.49892 | 0.53718 | 0.50938 | 0.5 | Area under the ROC curve |
| AUCI | Good | Good | Poor | Poor | Poor | Poor | Poor | AUC value interpretation |
| AUPR | 0.54757 | 0.71879 | 0.28974 | 0.0 | 0.44426 | 0.11042 | None | Area under the PR curve |
| BCD | 0.01285 | 0.07657 | 0.00302 | 0.03224 | 0.03904 | 0.01083 | 0.00428 | Bray-Curtis dissimilarity |
| BM | 0.43917 | 0.46005 | 0.1109 | -0.00216 | 0.07437 | 0.01877 | 0.0 | Informedness or bookmaker informedness |
| CEN | 0.47879 | 0.36325 | 0.59543 | 0.47237 | 0.45648 | 0.49572 | 0.23274 | Confusion entropy |
| DOR | 8.4299 | 8.37945 | 1.88947 | 0.0 | 49.66878 | 10.28191 | None | Diagnostic odds ratio |
| DP | 0.51043 | 0.509 | 0.15235 | None | 0.9351 | 0.55799 | None | Discriminant power |
| DPI | Poor | Poor | Poor | None | Poor | Poor | None | Discriminant power interpretation |
| ERR | 0.19798 | 0.28917 | 0.28413 | 0.06851 | 0.08111 | 0.02569 | 0.00856 | Error rate |
| F0.5 | 0.52702 | 0.63988 | 0.29232 | 0.0 | 0.2766 | 0.07353 | 0.0 | F0.5 score |
| F1 | 0.54566 | 0.70073 | 0.28967 | 0.0 | 0.13904 | 0.03774 | 0.0 | F1 score - harmonic mean of precision and sensitivity |
| F2 | 0.56568 | 0.77437 | 0.28707 | 0.0 | 0.09286 | 0.02538 | 0.0 | F2 score |
| FDR | 0.48472 | 0.39514 | 0.70588 | 1.0 | 0.1875 | 0.8 | None | False discovery rate |
| FN | 171 | 135 | 288 | 132 | 158 | 47 | 17 | False negative/miss/type 2 error |
| FNR | 0.42015 | 0.16729 | 0.71464 | 1.0 | 0.92398 | 0.97917 | 1.0 | Miss rate or false negative rate |
| FOR | 0.11198 | 0.15446 | 0.18068 | 0.06663 | 0.08024 | 0.02374 | 0.00856 | False omission rate |
| FP | 222 | 439 | 276 | 4 | 3 | 4 | 0 | False positive/type 1 error/false alarm |
| FPR | 0.14068 | 0.37267 | 0.17446 | 0.00216 | 0.00165 | 0.00207 | 0.0 | Fall-out or false positive rate |
| G | 0.54662 | 0.7097 | 0.28971 | 0.0 | 0.24853 | 0.06455 | None | G-measure geometric mean of precision and sensitivity |
| GI | 0.43917 | 0.46005 | 0.1109 | -0.00216 | 0.07437 | 0.01877 | 0.0 | Gini index |
| GM | 0.70589 | 0.72277 | 0.48536 | 0.0 | 0.2755 | 0.14419 | 0.0 | G-mean geometric mean of specificity and sensitivity |
| IBA | 0.35903 | 0.62968 | 0.10832 | 0.0 | 0.0059 | 0.00048 | 0.0 | Index of balanced accuracy |
| ICSI | 0.09514 | 0.43757 | -0.42052 | -1.0 | -0.11148 | -0.77917 | None | Individual classification success index |
| IS | 1.32948 | 0.57317 | 0.53475 | None | 3.23751 | 3.04803 | None | Information score |
| J | 0.3752 | 0.53933 | 0.16937 | 0.0 | 0.07471 | 0.01923 | 0.0 | Jaccard index |
| LS | 2.51312 | 1.48779 | 1.44869 | 0.0 | 9.43165 | 8.27083 | None | Lift score |
| MCC | 0.42085 | 0.4552 | 0.11216 | -0.01199 | 0.23336 | 0.05752 | None | Matthews correlation coefficient |
| MCCI | Weak | Weak | Negligible | Negligible | Negligible | Negligible | None | Matthews correlation coefficient interpretation |
| MCEN | 0.57832 | 0.47919 | 0.64397 | 0.47237 | 0.46376 | 0.49774 | 0.23274 | Modified confusion entropy |
| MK | 0.4033 | 0.4504 | 0.11344 | -0.06663 | 0.73226 | 0.17626 | None | Markedness |
| N | 1578 | 1178 | 1582 | 1853 | 1814 | 1937 | 1968 | Condition negative |
| NLR | 0.48893 | 0.26666 | 0.86567 | 1.00216 | 0.92551 | 0.98119 | 1.0 | Negative likelihood ratio |
| NLRI | Poor | Poor | Negligible | Negligible | Negligible | Negligible | Negligible | Negative likelihood ratio interpretation |
| NPV | 0.88802 | 0.84554 | 0.81932 | 0.93337 | 0.91976 | 0.97626 | 0.99144 | Negative predictive value |
| OC | 0.57985 | 0.83271 | 0.29412 | 0.0 | 0.8125 | 0.2 | None | Overlap coefficient |
| OOC | 0.54662 | 0.7097 | 0.28971 | 0.0 | 0.24853 | 0.06455 | None | Otsuka-Ochiai coefficient |
| OP | 0.60783 | 0.57017 | 0.22962 | -0.06851 | 0.06041 | 0.01521 | -0.00856 | Optimized precision |
| P | 407 | 807 | 403 | 132 | 171 | 48 | 17 | Condition positive or support |
| PLR | 4.12165 | 2.23448 | 1.63565 | 0.0 | 45.96881 | 10.08854 | None | Positive likelihood ratio |
| PLRI | Poor | Poor | Poor | Negligible | Good | Good | None | Positive likelihood ratio interpretation |
| POP | 1985 | 1985 | 1985 | 1985 | 1985 | 1985 | 1985 | Population |
| PPV | 0.51528 | 0.60486 | 0.29412 | 0.0 | 0.8125 | 0.2 | None | Precision or positive predictive value |
| PRE | 0.20504 | 0.40655 | 0.20302 | 0.0665 | 0.08615 | 0.02418 | 0.00856 | Prevalence |
| Q | 0.78791 | 0.78677 | 0.30783 | -1.0 | 0.96053 | 0.82273 | None | Yule Q - coefficient of colligation |
| QI | Strong | Strong | Weak | Negligible | Strong | Strong | None | Yule Q interpretation |
| RACC | 0.04731 | 0.22754 | 0.03999 | 0.00013 | 0.00069 | 6e-05 | 0.0 | Random accuracy |
| RACCU | 0.04747 | 0.23341 | 0.04 | 0.00117 | 0.00222 | 0.00018 | 2e-05 | Random accuracy unbiased |
| TN | 1356 | 739 | 1306 | 1849 | 1811 | 1933 | 1968 | True negative/correct rejection |
| TNR | 0.85932 | 0.62733 | 0.82554 | 0.99784 | 0.99835 | 0.99793 | 1.0 | Specificity or true negative rate |
| TON | 1527 | 874 | 1594 | 1981 | 1969 | 1980 | 1985 | Test outcome negative |
| TOP | 458 | 1111 | 391 | 4 | 16 | 5 | 0 | Test outcome positive |
| TP | 236 | 672 | 115 | 0 | 13 | 1 | 0 | True positive/hit |
| TPR | 0.57985 | 0.83271 | 0.28536 | 0.0 | 0.07602 | 0.02083 | 0.0 | Sensitivity, recall, hit rate, or true positive rate |
| Y | 0.43917 | 0.46005 | 0.1109 | -0.00216 | 0.07437 | 0.01877 | 0.0 | Youden index |
| dInd | 0.44308 | 0.40849 | 0.73563 | 1.0 | 0.92398 | 0.97917 | 1.0 | Distance index |
| sInd | 0.6867 | 0.71115 | 0.47983 | 0.29289 | 0.34665 | 0.30762 | 0.29289 | Similarity index |

Generated By PyCM Version 3.1
