# Supplementary material for: TopEC: prediction of Enzyme Commission classes by 3D graph neural networks and localized 3D protein descriptor
Source: Nat Commun. 2025 Mar 20;16:2737. doi: 10.1038/s41467-025-57324-5 (PMC11923149; doi:10.1038/s41467-025-57324-5)
Supplement: Supplementary file 3 — Supplementary Data 1 [file 41467_2025_57324_MOESM3_ESM.zip › Data_S1/table1/mainclass/EnzyNet/full_struc/BindingMOAD_FOLD_flips.html]

PDB\_FOLD\_enzynet\_flips


# PyCM Report

## Dataset Type :

- Multi-Class Classification
- Imbalanced

Note 1 : Recommended statistics for this type of classification highlighted in aqua

Note 2 : The recommender system assumes that the input is the result of classification over the whole data rather than just a part of it.
If the confusion matrix is the result of test data classification, the recommendation is not valid.

## Confusion Matrix :

|  |  |  |  |  |  |  |  |  |  |  |  |  |  |  |  |  |  |  |  |  |  |  |  |  |  |  |  |  |  |  |  |  |  |  |  |  |  |  |  |  |  |  |  |  |  |  |  |  |  |  |  |  |  |  |  |  |  |  |  |  |  |  |  |  |  |
| --- | --- | --- | --- | --- | --- | --- | --- | --- | --- | --- | --- | --- | --- | --- | --- | --- | --- | --- | --- | --- | --- | --- | --- | --- | --- | --- | --- | --- | --- | --- | --- | --- | --- | --- | --- | --- | --- | --- | --- | --- | --- | --- | --- | --- | --- | --- | --- | --- | --- | --- | --- | --- | --- | --- | --- | --- | --- | --- | --- | --- | --- | --- | --- | --- | --- |
| Actual | Predict  |  |  |  |  |  |  |  |  | | --- | --- | --- | --- | --- | --- | --- | --- | |  | 0 | 1 | 2 | 3 | 4 | 5 | 6 | | 0 | 230 | 75 | 96 | 5 | 1 | 0 | 0 | | 1 | 30 | 673 | 101 | 2 | 0 | 1 | 0 | | 2 | 72 | 216 | 112 | 1 | 1 | 1 | 0 | | 3 | 50 | 54 | 28 | 0 | 0 | 0 | 0 | | 4 | 72 | 44 | 41 | 1 | 13 | 0 | 0 | | 5 | 7 | 24 | 16 | 0 | 0 | 1 | 0 | | 6 | 1 | 14 | 2 | 0 | 0 | 0 | 0 | |

## Overall Statistics :

|  |  |
| --- | --- |
| 95% CI | (0.49641,0.54037) |
| ACC Macro | 0.8624 |
| ARI | 0.22328 |
| AUNP | 0.65199 |
| AUNU | 0.57699 |
| Bangdiwala B | 0.41844 |
| Bennett S | 0.43812 |
| CBA | 0.21206 |
| CSI | None |
| Chi-Squared | None |
| Chi-Squared DF | 36 |
| Conditional Entropy | 1.24667 |
| Cramer V | None |
| Cross Entropy | 2.60104 |
| F1 Macro | 0.24207 |
| F1 Micro | 0.51839 |
| FNR Macro | 0.74659 |
| FNR Micro | 0.48161 |
| FPR Macro | 0.09943 |
| FPR Micro | 0.08027 |
| Gwet AC1 | 0.45711 |
| Hamming Loss | 0.48161 |
| Joint Entropy | 3.46372 |
| KL Divergence | None |
| Kappa | 0.29742 |
| Kappa 95% CI | (0.26536,0.32949) |
| Kappa No Prevalence | 0.03678 |
| Kappa Standard Error | 0.01636 |
| Kappa Unbiased | 0.28882 |
| Krippendorff Alpha | 0.289 |
| Lambda A | 0.1927 |
| Lambda B | 0.20678 |
| Mutual Information | 0.28144 |
| NIR | 0.40655 |
| Overall ACC | 0.51839 |
| Overall CEN | 0.44588 |
| Overall J | (1.16349,0.16621) |
| Overall MCC | 0.30648 |
| Overall MCEN | 0.53766 |
| Overall RACC | 0.3145 |
| Overall RACCU | 0.3228 |
| P-Value | None |
| PPV Macro | None |
| PPV Micro | 0.51839 |
| Pearson C | None |
| Phi-Squared | None |
| RCI | 0.12694 |
| RR | 283.57143 |
| Reference Entropy | 2.21706 |
| Response Entropy | 1.5281 |
| SOA1(Landis & Koch) | Fair |
| SOA2(Fleiss) | Poor |
| SOA3(Altman) | Fair |
| SOA4(Cicchetti) | Poor |
| SOA5(Cramer) | None |
| SOA6(Matthews) | Weak |
| Scott PI | 0.28882 |
| Standard Error | 0.01121 |
| TNR Macro | 0.90057 |
| TNR Micro | 0.91973 |
| TPR Macro | 0.25341 |
| TPR Micro | 0.51839 |
| Zero-one Loss | 956 |

## Class Statistics :

|  |  |  |  |  |  |  |  |  |
| --- | --- | --- | --- | --- | --- | --- | --- | --- |
| Class | 0 | 1 | 2 | 3 | 4 | 5 | 6 | Description |
| ACC | 0.79395 | 0.71738 | 0.71033 | 0.92897 | 0.9194 | 0.97531 | 0.99144 | Accuracy |
| AGF | 0.69484 | 0.78664 | 0.47751 | 0.0 | 0.2948 | 0.15858 | 0.0 | Adjusted F-score |
| AGM | 0.76457 | 0.69503 | 0.62963 | 0 | 0.62096 | 0.56639 | 0 | Adjusted geometric mean |
| AM | 55 | 293 | -7 | -123 | -156 | -45 | -17 | Difference between automatic and manual classification |
| AUC | 0.70904 | 0.73574 | 0.5492 | 0.49757 | 0.53746 | 0.5099 | 0.5 | Area under the ROC curve |
| AUCI | Good | Good | Poor | Poor | Poor | Poor | Poor | AUC value interpretation |
| AUPR | 0.53147 | 0.72289 | 0.28037 | 0.0 | 0.47135 | 0.17708 | None | Area under the PR curve |
| BCD | 0.01385 | 0.0738 | 0.00176 | 0.03098 | 0.03929 | 0.01134 | 0.00428 | Bray-Curtis dissimilarity |
| BM | 0.41809 | 0.47147 | 0.0984 | -0.00486 | 0.07492 | 0.0198 | 0.0 | Informedness or bookmaker informedness |
| CEN | 0.49584 | 0.35618 | 0.59349 | 0.52525 | 0.45314 | 0.46085 | 0.23274 | Confusion entropy |
| DOR | 7.53896 | 8.83329 | 1.75906 | 0.0 | 74.5443 | 20.58511 | None | Diagnostic odds ratio |
| DP | 0.48369 | 0.52162 | 0.13523 | None | 1.03232 | 0.7242 | None | Discriminant power |
| DPI | Poor | Poor | Poor | None | Limited | Poor | None | Discriminant power interpretation |
| ERR | 0.20605 | 0.28262 | 0.28967 | 0.07103 | 0.0806 | 0.02469 | 0.00856 | Error rate |
| F0.5 | 0.50998 | 0.64625 | 0.28183 | 0.0 | 0.28139 | 0.08333 | 0.0 | F0.5 score |
| F1 | 0.52934 | 0.70582 | 0.28035 | 0.0 | 0.13978 | 0.03922 | 0.0 | F1 score - harmonic mean of precision and sensitivity |
| F2 | 0.55024 | 0.7775 | 0.27888 | 0.0 | 0.09299 | 0.02564 | 0.0 | F2 score |
| FDR | 0.50216 | 0.38818 | 0.71717 | 1.0 | 0.13333 | 0.66667 | None | False discovery rate |
| FN | 177 | 134 | 291 | 132 | 158 | 47 | 17 | False negative/miss/type 2 error |
| FNR | 0.43489 | 0.16605 | 0.72208 | 1.0 | 0.92398 | 0.97917 | 1.0 | Miss rate or false negative rate |
| FOR | 0.11622 | 0.15141 | 0.18313 | 0.0668 | 0.0802 | 0.02371 | 0.00856 | False omission rate |
| FP | 232 | 427 | 284 | 9 | 2 | 2 | 0 | False positive/type 1 error/false alarm |
| FPR | 0.14702 | 0.36248 | 0.17952 | 0.00486 | 0.0011 | 0.00103 | 0.0 | Fall-out or false positive rate |
| G | 0.53041 | 0.7143 | 0.28036 | 0.0 | 0.25668 | 0.08333 | None | G-measure geometric mean of precision and sensitivity |
| GI | 0.41809 | 0.47147 | 0.0984 | -0.00486 | 0.07492 | 0.0198 | 0.0 | Gini index |
| GM | 0.69428 | 0.72915 | 0.47752 | 0.0 | 0.27557 | 0.14426 | 0.0 | G-mean geometric mean of specificity and sensitivity |
| IBA | 0.34327 | 0.6361 | 0.10431 | 0.0 | 0.00586 | 0.00046 | 0.0 | Index of balanced accuracy |
| ICSI | 0.06295 | 0.44577 | -0.43926 | -1.0 | -0.05731 | -0.64583 | None | Individual classification success index |
| IS | 1.27978 | 0.58967 | 0.47829 | None | 3.33062 | 3.785 | None | Information score |
| J | 0.35994 | 0.54538 | 0.16303 | 0.0 | 0.07514 | 0.02 | 0.0 | Jaccard index |
| LS | 2.42802 | 1.50491 | 1.39309 | 0.0 | 10.06043 | 13.78472 | None | Lift score |
| MCC | 0.39944 | 0.46591 | 0.09904 | -0.01801 | 0.24274 | 0.0783 | None | Matthews correlation coefficient |
| MCCI | Weak | Weak | Negligible | Negligible | Negligible | Negligible | None | Matthews correlation coefficient interpretation |
| MCEN | 0.59512 | 0.4708 | 0.63938 | 0.52525 | 0.46023 | 0.46226 | 0.23274 | Modified confusion entropy |
| MK | 0.38162 | 0.46041 | 0.09969 | -0.0668 | 0.78646 | 0.30962 | None | Markedness |
| N | 1578 | 1178 | 1582 | 1853 | 1814 | 1937 | 1968 | Condition negative |
| NLR | 0.50985 | 0.26046 | 0.88008 | 1.00488 | 0.925 | 0.98018 | 1.0 | Negative likelihood ratio |
| NLRI | Negligible | Poor | Negligible | Negligible | Negligible | Negligible | Negligible | Negative likelihood ratio interpretation |
| NPV | 0.88378 | 0.84859 | 0.81687 | 0.9332 | 0.9198 | 0.97629 | 0.99144 | Negative predictive value |
| OC | 0.56511 | 0.83395 | 0.28283 | 0.0 | 0.86667 | 0.33333 | None | Overlap coefficient |
| OOC | 0.53041 | 0.7143 | 0.28036 | 0.0 | 0.25668 | 0.08333 | None | Otsuka-Ochiai coefficient |
| OP | 0.59096 | 0.58389 | 0.21637 | -0.07103 | 0.06084 | 0.01617 | -0.00856 | Optimized precision |
| P | 407 | 807 | 403 | 132 | 171 | 48 | 17 | Condition positive or support |
| PLR | 3.84373 | 2.30069 | 1.54811 | 0.0 | 68.95322 | 20.17708 | None | Positive likelihood ratio |
| PLRI | Poor | Poor | Poor | Negligible | Good | Good | None | Positive likelihood ratio interpretation |
| POP | 1985 | 1985 | 1985 | 1985 | 1985 | 1985 | 1985 | Population |
| PPV | 0.49784 | 0.61182 | 0.28283 | 0.0 | 0.86667 | 0.33333 | None | Precision or positive predictive value |
| PRE | 0.20504 | 0.40655 | 0.20302 | 0.0665 | 0.08615 | 0.02418 | 0.00856 | Prevalence |
| Q | 0.76578 | 0.79661 | 0.27512 | -1.0 | 0.97353 | 0.90734 | None | Yule Q - coefficient of colligation |
| QI | Strong | Strong | Weak | Negligible | Strong | Strong | None | Yule Q interpretation |
| RACC | 0.04772 | 0.22529 | 0.0405 | 0.0003 | 0.00065 | 4e-05 | 0.0 | Random accuracy |
| RACCU | 0.04791 | 0.23074 | 0.04051 | 0.00126 | 0.0022 | 0.00017 | 2e-05 | Random accuracy unbiased |
| TN | 1346 | 751 | 1298 | 1844 | 1812 | 1935 | 1968 | True negative/correct rejection |
| TNR | 0.85298 | 0.63752 | 0.82048 | 0.99514 | 0.9989 | 0.99897 | 1.0 | Specificity or true negative rate |
| TON | 1523 | 885 | 1589 | 1976 | 1970 | 1982 | 1985 | Test outcome negative |
| TOP | 462 | 1100 | 396 | 9 | 15 | 3 | 0 | Test outcome positive |
| TP | 230 | 673 | 112 | 0 | 13 | 1 | 0 | True positive/hit |
| TPR | 0.56511 | 0.83395 | 0.27792 | 0.0 | 0.07602 | 0.02083 | 0.0 | Sensitivity, recall, hit rate, or true positive rate |
| Y | 0.41809 | 0.47147 | 0.0984 | -0.00486 | 0.07492 | 0.0198 | 0.0 | Youden index |
| dInd | 0.45907 | 0.3987 | 0.74407 | 1.00001 | 0.92398 | 0.97917 | 1.0 | Distance index |
| sInd | 0.67539 | 0.71808 | 0.47387 | 0.29288 | 0.34665 | 0.30762 | 0.29289 | Similarity index |

Generated By PyCM Version 3.1
