# Supplementary material for: TopEC: prediction of Enzyme Commission classes by 3D graph neural networks and localized 3D protein descriptor
Source: Nat Commun. 2025 Mar 20;16:2737. doi: 10.1038/s41467-025-57324-5 (PMC11923149; doi:10.1038/s41467-025-57324-5)
Supplement: Supplementary file 3 — Supplementary Data 1 [file 41467_2025_57324_MOESM3_ESM.zip › Data_S1/table1/mainclass/EnzyNet/full_struc/BindingMOAD_FOLD.html]

PDB\_FOLD\_enzynet


# PyCM Report

## Dataset Type :

- Multi-Class Classification
- Imbalanced

Note 1 : Recommended statistics for this type of classification highlighted in aqua

Note 2 : The recommender system assumes that the input is the result of classification over the whole data rather than just a part of it.
If the confusion matrix is the result of test data classification, the recommendation is not valid.

## Confusion Matrix :

|  |  |  |  |  |  |  |  |  |  |  |  |  |  |  |  |  |  |  |  |  |  |  |  |  |  |  |  |  |  |  |  |  |  |  |  |  |  |  |  |  |  |  |  |  |  |  |  |  |  |  |  |  |  |  |  |  |  |  |  |  |  |  |  |  |  |
| --- | --- | --- | --- | --- | --- | --- | --- | --- | --- | --- | --- | --- | --- | --- | --- | --- | --- | --- | --- | --- | --- | --- | --- | --- | --- | --- | --- | --- | --- | --- | --- | --- | --- | --- | --- | --- | --- | --- | --- | --- | --- | --- | --- | --- | --- | --- | --- | --- | --- | --- | --- | --- | --- | --- | --- | --- | --- | --- | --- | --- | --- | --- | --- | --- | --- |
| Actual | Predict  |  |  |  |  |  |  |  |  | | --- | --- | --- | --- | --- | --- | --- | --- | |  | 0 | 1 | 2 | 3 | 4 | 5 | 6 | | 0 | 226 | 96 | 76 | 4 | 4 | 0 | 1 | | 1 | 39 | 649 | 104 | 6 | 7 | 0 | 2 | | 2 | 98 | 167 | 119 | 4 | 4 | 10 | 1 | | 3 | 34 | 63 | 31 | 3 | 1 | 0 | 0 | | 4 | 57 | 41 | 48 | 4 | 15 | 1 | 5 | | 5 | 6 | 19 | 22 | 0 | 0 | 1 | 0 | | 6 | 1 | 11 | 5 | 0 | 0 | 0 | 0 | |

## Overall Statistics :

|  |  |
| --- | --- |
| 95% CI | (0.48834,0.53232) |
| ACC Macro | 0.86009 |
| ARI | 0.20908 |
| AUNP | 0.65225 |
| AUNU | 0.5779 |
| Bangdiwala B | 0.40429 |
| Bennett S | 0.42872 |
| CBA | 0.2194 |
| CSI | -0.44276 |
| Chi-Squared | 723.7295 |
| Chi-Squared DF | 36 |
| Conditional Entropy | 1.43569 |
| Cramer V | 0.24651 |
| Cross Entropy | 2.47142 |
| F1 Macro | 0.24812 |
| F1 Micro | 0.51033 |
| FNR Macro | 0.74485 |
| FNR Micro | 0.48967 |
| FPR Macro | 0.09936 |
| FPR Micro | 0.08161 |
| Gwet AC1 | 0.44685 |
| Hamming Loss | 0.48967 |
| Joint Entropy | 3.65275 |
| KL Divergence | 0.25436 |
| Kappa | 0.29492 |
| Kappa 95% CI | (0.26326,0.32659) |
| Kappa No Prevalence | 0.02065 |
| Kappa Standard Error | 0.01616 |
| Kappa Unbiased | 0.28886 |
| Krippendorff Alpha | 0.28904 |
| Lambda A | 0.1893 |
| Lambda B | 0.15868 |
| Mutual Information | 0.25138 |
| NIR | 0.40655 |
| Overall ACC | 0.51033 |
| Overall CEN | 0.4833 |
| Overall J | (1.18094,0.16871) |
| Overall MCC | 0.30106 |
| Overall MCEN | 0.58454 |
| Overall RACC | 0.30551 |
| Overall RACCU | 0.31143 |
| P-Value | None |
| PPV Macro | 0.30208 |
| PPV Micro | 0.51033 |
| Pearson C | 0.5169 |
| Phi-Squared | 0.3646 |
| RCI | 0.11339 |
| RR | 283.57143 |
| Reference Entropy | 2.21706 |
| Response Entropy | 1.68707 |
| SOA1(Landis & Koch) | Fair |
| SOA2(Fleiss) | Poor |
| SOA3(Altman) | Fair |
| SOA4(Cicchetti) | Poor |
| SOA5(Cramer) | Moderate |
| SOA6(Matthews) | Weak |
| Scott PI | 0.28886 |
| Standard Error | 0.01122 |
| TNR Macro | 0.90064 |
| TNR Micro | 0.91839 |
| TPR Macro | 0.25515 |
| TPR Micro | 0.51033 |
| Zero-one Loss | 972 |

## Class Statistics :

|  |  |  |  |  |  |  |  |  |
| --- | --- | --- | --- | --- | --- | --- | --- | --- |
| Class | 0 | 1 | 2 | 3 | 4 | 5 | 6 | Description |
| ACC | 0.79043 | 0.7204 | 0.71285 | 0.92594 | 0.91335 | 0.97078 | 0.9869 | Accuracy |
| AGF | 0.68799 | 0.77518 | 0.49184 | 0.16068 | 0.31293 | 0.15496 | 0.0 | Adjusted F-score |
| AGM | 0.75992 | 0.70516 | 0.63703 | 0.5557 | 0.62735 | 0.56392 | 0 | Adjusted geometric mean |
| AM | 54 | 239 | 2 | -111 | -140 | -36 | -8 | Difference between automatic and manual classification |
| AUC | 0.70318 | 0.7336 | 0.55725 | 0.50651 | 0.53945 | 0.50758 | 0.49771 | Area under the ROC curve |
| AUCI | Good | Good | Poor | Poor | Poor | Poor | Poor | AUC value interpretation |
| AUPR | 0.52276 | 0.71234 | 0.29456 | 0.08279 | 0.2858 | 0.05208 | 0.0 | Area under the PR curve |
| BCD | 0.0136 | 0.0602 | 0.0005 | 0.02796 | 0.03526 | 0.00907 | 0.00202 | Bray-Curtis dissimilarity |
| BM | 0.40636 | 0.4672 | 0.1145 | 0.01301 | 0.0789 | 0.01515 | -0.00457 | Informedness or bookmaker informedness |
| CEN | 0.50212 | 0.37702 | 0.64612 | 0.59107 | 0.61 | 0.53488 | 0.63233 | Confusion entropy |
| DOR | 7.13572 | 8.08068 | 1.89875 | 2.3708 | 10.80529 | 3.72534 | 0.0 | Diagnostic odds ratio |
| DP | 0.47053 | 0.5003 | 0.15353 | 0.20669 | 0.56987 | 0.3149 | None | Discriminant power |
| DPI | Poor | Poor | Poor | Poor | Poor | Poor | None | Discriminant power interpretation |
| ERR | 0.20957 | 0.2796 | 0.28715 | 0.07406 | 0.08665 | 0.02922 | 0.0131 | Error rate |
| F0.5 | 0.502 | 0.65017 | 0.29412 | 0.06944 | 0.25424 | 0.05208 | 0.0 | F0.5 score |
| F1 | 0.52074 | 0.70049 | 0.29455 | 0.03922 | 0.14851 | 0.03333 | 0.0 | F1 score - harmonic mean of precision and sensitivity |
| F2 | 0.54093 | 0.75924 | 0.29499 | 0.02732 | 0.1049 | 0.02451 | 0.0 | F2 score |
| FDR | 0.50976 | 0.37954 | 0.70617 | 0.85714 | 0.51613 | 0.91667 | 1.0 | False discovery rate |
| FN | 181 | 158 | 284 | 129 | 156 | 47 | 17 | False negative/miss/type 2 error |
| FNR | 0.44472 | 0.19579 | 0.70471 | 0.97727 | 0.91228 | 0.97917 | 1.0 | Miss rate or false negative rate |
| FOR | 0.11877 | 0.16826 | 0.17975 | 0.06568 | 0.07984 | 0.02382 | 0.0086 | False omission rate |
| FP | 235 | 397 | 286 | 18 | 16 | 11 | 9 | False positive/type 1 error/false alarm |
| FPR | 0.14892 | 0.33701 | 0.18078 | 0.00971 | 0.00882 | 0.00568 | 0.00457 | Fall-out or false positive rate |
| G | 0.52175 | 0.70639 | 0.29456 | 0.05698 | 0.20602 | 0.04167 | 0.0 | G-measure geometric mean of precision and sensitivity |
| GI | 0.40636 | 0.4672 | 0.1145 | 0.01301 | 0.0789 | 0.01515 | -0.00457 | Gini index |
| GM | 0.68745 | 0.73019 | 0.49184 | 0.15002 | 0.29487 | 0.14393 | 0.0 | G-mean geometric mean of specificity and sensitivity |
| IBA | 0.3328 | 0.60848 | 0.11516 | 0.00073 | 0.00839 | 0.00055 | 0.0 | Index of balanced accuracy |
| ICSI | 0.04552 | 0.42467 | -0.41089 | -0.83442 | -0.42841 | -0.89583 | -1.0 | Individual classification success index |
| IS | 1.25759 | 0.60991 | 0.53333 | 1.10317 | 2.48977 | 1.785 | None | Information score |
| J | 0.35202 | 0.53904 | 0.17271 | 0.02 | 0.08021 | 0.01695 | 0.0 | Jaccard index |
| LS | 2.39097 | 1.52616 | 1.44726 | 2.14827 | 5.61686 | 3.44618 | 0.0 | Lift score |
| MCC | 0.38852 | 0.45964 | 0.11429 | 0.03169 | 0.17854 | 0.03003 | -0.00627 | Matthews correlation coefficient |
| MCCI | Weak | Weak | Negligible | Negligible | Negligible | Negligible | Negligible | Matthews correlation coefficient interpretation |
| MCEN | 0.60023 | 0.50027 | 0.70467 | 0.59509 | 0.63037 | 0.5373 | 0.63233 | Modified confusion entropy |
| MK | 0.37147 | 0.45219 | 0.11408 | 0.07717 | 0.40403 | 0.05951 | -0.0086 | Markedness |
| N | 1578 | 1178 | 1582 | 1853 | 1814 | 1937 | 1968 | Condition negative |
| NLR | 0.52253 | 0.29531 | 0.86023 | 0.98686 | 0.9204 | 0.98476 | 1.00459 | Negative likelihood ratio |
| NLRI | Negligible | Poor | Negligible | Negligible | Negligible | Negligible | Negligible | Negative likelihood ratio interpretation |
| NPV | 0.88123 | 0.83174 | 0.82025 | 0.93432 | 0.92016 | 0.97618 | 0.9914 | Negative predictive value |
| OC | 0.55528 | 0.80421 | 0.29529 | 0.14286 | 0.48387 | 0.08333 | 0.0 | Overlap coefficient |
| OOC | 0.52175 | 0.70639 | 0.29456 | 0.05698 | 0.20602 | 0.04167 | 0.0 | Otsuka-Ochiai coefficient |
| OP | 0.5801 | 0.62415 | 0.24274 | -0.02918 | 0.07596 | 0.01183 | -0.0131 | Optimized precision |
| P | 407 | 807 | 403 | 132 | 171 | 48 | 17 | Condition positive or support |
| PLR | 3.72866 | 2.3863 | 1.63336 | 2.33965 | 9.94518 | 3.66856 | 0.0 | Positive likelihood ratio |
| PLRI | Poor | Poor | Poor | Poor | Fair | Poor | Negligible | Positive likelihood ratio interpretation |
| POP | 1985 | 1985 | 1985 | 1985 | 1985 | 1985 | 1985 | Population |
| PPV | 0.49024 | 0.62046 | 0.29383 | 0.14286 | 0.48387 | 0.08333 | 0.0 | Precision or positive predictive value |
| PRE | 0.20504 | 0.40655 | 0.20302 | 0.0665 | 0.08615 | 0.02418 | 0.00856 | Prevalence |
| Q | 0.75417 | 0.77975 | 0.31005 | 0.40667 | 0.83058 | 0.57675 | -1.0 | Yule Q - coefficient of colligation |
| QI | Strong | Strong | Weak | Weak | Strong | Moderate | Negligible | Yule Q interpretation |
| RACC | 0.04762 | 0.21423 | 0.04142 | 0.0007 | 0.00135 | 0.00015 | 4e-05 | Random accuracy |
| RACCU | 0.0478 | 0.21786 | 0.04142 | 0.00149 | 0.00259 | 0.00023 | 4e-05 | Random accuracy unbiased |
| TN | 1343 | 781 | 1296 | 1835 | 1798 | 1926 | 1959 | True negative/correct rejection |
| TNR | 0.85108 | 0.66299 | 0.81922 | 0.99029 | 0.99118 | 0.99432 | 0.99543 | Specificity or true negative rate |
| TON | 1524 | 939 | 1580 | 1964 | 1954 | 1973 | 1976 | Test outcome negative |
| TOP | 461 | 1046 | 405 | 21 | 31 | 12 | 9 | Test outcome positive |
| TP | 226 | 649 | 119 | 3 | 15 | 1 | 0 | True positive/hit |
| TPR | 0.55528 | 0.80421 | 0.29529 | 0.02273 | 0.08772 | 0.02083 | 0.0 | Sensitivity, recall, hit rate, or true positive rate |
| Y | 0.40636 | 0.4672 | 0.1145 | 0.01301 | 0.0789 | 0.01515 | -0.00457 | Youden index |
| dInd | 0.46899 | 0.38976 | 0.72753 | 0.97732 | 0.91232 | 0.97918 | 1.00001 | Distance index |
| sInd | 0.66837 | 0.7244 | 0.48556 | 0.30893 | 0.35489 | 0.30761 | 0.29289 | Similarity index |

Generated By PyCM Version 3.1
