# Supplementary material for: TopEC: prediction of Enzyme Commission classes by 3D graph neural networks and localized 3D protein descriptor
Source: Nat Commun. 2025 Mar 20;16:2737. doi: 10.1038/s41467-025-57324-5 (PMC11923149; doi:10.1038/s41467-025-57324-5)
Supplement: Supplementary file 5 — Reporting Summary [file 41467_2025_57324_MOESM5_ESM.pdf]

nature portfolio

Corresponding author(s): Holger Gohlke

Last updated by author(s): 27. Jan 2025

Reporting Summary

Nature Portfolio wishes to improve the reproducibility of the work that we publish. This form provides structure for consistency and transparency in reporting. For further information on Nature Portfolio policies, see our Editorial Policies and the Editorial Policy Checklist.

Statistics

For all statistical analyses, confirm that the following items are present in the figure legend, table legend, main text, or Methods section.

n/a	Confirmed	
☐	☒	The exact sample size (*n*) for each experimental group/condition, given as a discrete number and unit of measurement
☐	☒	A statement on whether measurements were taken from distinct samples or whether the same sample was measured repeatedly
☐	☒	The statistical test(s) used AND whether they are one- or two-sided<br>*Only common tests should be described solely by name; describe more complex techniques in the Methods section.*
☐	☒	A description of all covariates tested
☐	☒	A description of any assumptions or corrections, such as tests of normality and adjustment for multiple comparisons
☐	☒	A full description of the statistical parameters including central tendency (e.g. means) or other basic estimates (e.g. regression coefficient) AND variation (e.g. standard deviation) or associated estimates of uncertainty (e.g. confidence intervals)
☐	☒	For null hypothesis testing, the test statistic (e.g. *F*, *t*, *r*) with confidence intervals, effect sizes, degrees of freedom and *P* value noted<br>*Give P values as exact values whenever suitable.*
☒	☐	For Bayesian analysis, information on the choice of priors and Markov chain Monte Carlo settings
☐	☒	For hierarchical and complex designs, identification of the appropriate level for tests and full reporting of outcomes
☒	☐	Estimates of effect sizes (e.g. Cohen's *d*, Pearson's *r*), indicating how they were calculated

Our web collection on statistics for biologists contains articles on many of the points above.

Software and code

Policy information about availability of computer code

Data collection	We utilized python environments to control the version for each software package. The main packages are python3.9 and pytorch2.1 using CUDA12.1 with Nvidia A100(40Gb) GPU. The version details for all dependencies are shown in the requirements.txt.
Data analysis	We utilized python environments to control the version for each software package. The main packages are python3.9 and pytorch2.1 using CUDA12.1 with Nvidia A100(40Gb) GPU. The version details for all dependencies are shown in the requirements.txt.

For manuscripts utilizing custom algorithms or software that are central to the research but not yet described in published literature, software must be made available to editors and reviewers. We strongly encourage code deposition in a community repository (e.g. GitHub). See the Nature Portfolio guidelines for submitting code & software for further information.

Data

Policy information about availability of data

All manuscripts must include a data availability statement. This statement should provide the following information, where applicable:

- Accession codes, unique identifiers, or web links for publicly available datasets
- A description of any restrictions on data availability
- For clinical datasets or third party data, please ensure that the statement adheres to our policy

The raw PDB files, a hierarchical data format (HDF5) of the structures, source data for this paper, and the trained networks used in this study are available in researchdata.hhu.de under accession code https://doi.org/10.25838/d5p-66.

Source Data are available with this paper as a Source Data file.

Research involving human participants, their data, or biological material

Policy information about studies with human participants or human data. See also policy information about sex, gender (identity/presentation), and sexual orientation and race, ethnicity and racism.

Reporting on sex and gender	n/a
Reporting on race, ethnicity, or other socially relevant groupings	n/a
Population characteristics	n/a
Recruitment	n/a
Ethics oversight	n/a

Note that full information on the approval of the study protocol must also be provided in the manuscript.

Field-specific reporting

Please select the one below that is the best fit for your research. If you are not sure, read the appropriate sections before making your selection.

- [x] Life sciences
- [ ] Behavioural & social sciences
- [ ] Ecological, evolutionary & environmental sciences

For a reference copy of the document with all sections, see nature.com/documents/nr-reporting-summary-flat.pdf

Life sciences study design

All studies must disclose on these points even when the disclosure is negative.

Sample size	Multiple datasets. Sample size is described per dataset in the "Datasets used" section of the paper. The complete combinatorial dataset is 257,165 samples from experimental and computationally generated protein structures.
Data exclusions	Any non-standard amino acid residues are encoded as a unknown residue in the neural network. We removed all HETATM records from the experimental PDBs. We also removed all DNA and RNA atoms.
Replication	We use PyTorch to model the neural networks. Completely reproducible results are not guaranteed across PyTorch releases, individual commits, or different platforms. We however do set a seed to control the random number generators across the software.
Randomization	Samples are divided over train,ing validation and test sets. We utilize two splits described in the paper. The temporal split is divided by publication date for the protein structure. For the fold split ,we determine the sequence identity between samples using MMSeqs. For each enzyme classification we order the resulting clusters by size. We pick the largest possible clusters to populate the test set until we reach the desired split threshold and repeat this for the validation set. In this case 10% of the data are used for the test set, 10% for the validation set, and the rest is added to the training set.
Blinding	A full blind study is not possible. To prevent data leakage, we tested on benchmark datasets, removing any structures with more than 30% sequence identity to the training database. Furthermore, we can only test enzyme classifications inside the training dataspace.

Reporting for specific materials, systems and methods

We require information from authors about some types of materials, experimental systems and methods used in many studies. Here, indicate whether each material, system or method listed is relevant to your study. If you are not sure if a list item applies to your research, read the appropriate section before selecting a response.

Materials & experimental systems

n/a	Involved in the study	
[x]	[ ]	Antibodies
[x]	[ ]	Eukaryotic cell lines
[x]	[ ]	Palaeontology and archaeology
[x]	[ ]	Animals and other organisms
[x]	[ ]	Clinical data
[x]	[ ]	Dual use research of concern
[x]	[ ]	Plants

Methods

n/a	Involved in the study	
[x]	[ ]	ChIP-seq
[x]	[ ]	Flow cytometry
[x]	[ ]	MRI-based neuroimaging

Plants

Seed stocks	n/a
Novel plant genotypes	n/a
Authentication	n/a
